# Vicinal difunctionalization of carbon–carbon double bond for the platform synthesis of trifluoroalkyl amines

**DOI:** 10.1038/s41467-020-19748-z

**Published:** 2020-11-23

**Authors:** Ferenc Béke, Ádám Mészáros, Ágnes Tóth, Bence Béla Botlik, Zoltán Novák

**Affiliations:** ELTE “Lendület” Catalysis and Organic Synthesis Research Group, Institute of Chemistry, Eötvös Loránd University, Pázmány Péter stny. 1/A, 1117 Budapest, Hungary

**Keywords:** Diversity-oriented synthesis, Reaction mechanisms, Synthetic chemistry methodology

## Abstract

Regioselective vicinal diamination of carbon–carbon double bonds with two different amines is a synthetic challenge under transition metal-free conditions, especially for the synthesis of trifluoromethylated amines. However, the synthesis of ethylene diamines and fluorinated amine compounds is demanded, especially in the pharmaceutical sector. Herein, we demonstrate that the controllable double nucleophilic functionalization of an activated alkene synthon, originated from a trifluoropropenyliodonium salt with two distinct nucleophiles, enables the selective synthesis of trifluoromethylated ethylene amines and diamines on broad scale with high efficiency under mild reaction conditions. Considering the chemical nature of the reactants, our synthetic approach brings forth an efficient methodology and provides versatile access to highly fluorinated amines.

## Abstract (web summary)

Metal-free olefin diamination may display challenges, especially when targeting fluorinated amines. Here, the authors report a double nucleophilic functionalization of an activated alkene originated from a trifluoropropenyliodonium salt with two nucleophiles for the selective synthesis of trifluoromethylated ethylene amines and diamines.

## Introduction

**Fig. 1:**
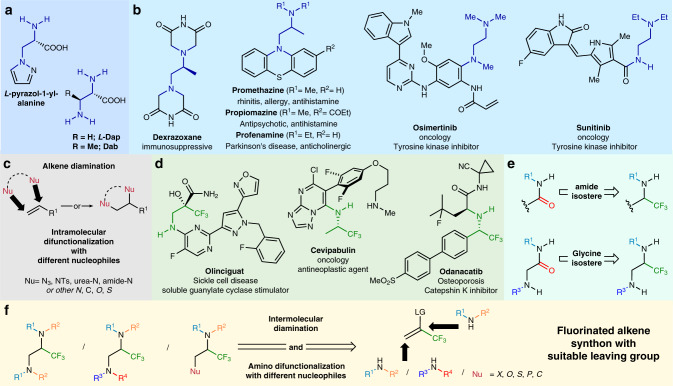
General overview of ethylene diamines and trifluoromethyl amines. **a** Vicinal diamine backbones in natural products. **b** Pharmaceutical molecules with vicinal diamine motifs. **c** Alkene diamination and diazidation methodologies. **d** Fluoroalkylamine containing drug candidates. **e** Bioisosteric relations of fluoroalkylamine moiety. **f** This work: The intermolecular nucleophilic difunctionalization of a fluorinated alkene synthon provides a high degree of structural diversity of α-trifluoromethyl diamines and substituted trifluoropropylamines in one preparative step.

The vicinal diamine backbone is a prevalent motif in natural products, chelating agents, chiral ligands, and pharmaceuticals^1,2^. Nature presents this structural motif in the form of non-proteinogenic amino acids which constitute the skeleton of numerous peptide antibiotics^3^. (Fig. 1a) Along with natural occurrence, the diamine moiety can be found in approved drugs, such as Promethazine, Osimertinib, and Sunitinib which are placed among the most marketed pharmaceuticals^4^. (Fig. 1b) Concurrently, many drug candidates that are investigated in the treatment of chronic pain and metastatic melanoma share the *vic*-diamine skeleton. Considering the importance of this moiety, the development of methods for the construction of vicinal diamines is an important area of research in organic synthesis^1,2^. Among the various synthetic possibilities of accessing the target diamine molecules, conceptually, the most straightforward way is the direct introduction of nitrogen atoms across a carbon–carbon double bond (Fig. 1c)^1,5^. Indeed, a diverse set of methodologies have been developed to introduce nitrogen atoms attached the same substituents onto carbon–carbon double bond, i.e., diazidation and homodiamination reactions in metal-assisted^6,7^, metal-catalyzed^8–13^, organoselenium-catalyzed^14^, hypervalent iodine-assisted and -catalyzed^15–19^ photochemical^20–23^ and electrochemical^24,25^ fashions.

In general, the diamination reaction of alkenes requires radical or oxidative conditions, which permit the introduction of *N*-atoms in form of azide, sulfonamide, urea, and amide functionalities, while easily oxidizable alkyl- and aryl-amines are introduced by transition metal-assisted and -catalyzed methods limited to few examples^1,26–28^. Regarding the abundant *N*-atom sources, nucleophilic difunctionalization of alkenes with simple amines without the use of transition metals would be a cornerstone to improve structural diversity of diamine synthesis. This vicinal amination of the carbon–carbon double bond under metal free conditions has been presented with bifunctional nucleophiles in an intramolecular manner to afford *N*,*N*- and *N*,*O*-heterocyclic systems using alkenyl-sulfonium salts with two adjacent electrophilic centers^29–32^. However, the intermolecular difunctionalization of alkenes has not been performed by the utilization of two separate external nitrogen nucleophiles via metal-free vicinal heterodiamination. Moreover, the major challenge of the present approach is the selective heterodiamination with two different nitrogen nucleophiles due to the control of reactivity of the nucleophiles and intermediates.

The presence of trifluoromethyl group could fine tune the physical, biological, and chemical properties of organic molecules in medicinal chemistry development^33–35^. Therefore, the incorporation of this structure is an emerging field of synthetic methodology^36–40^. The presence of fluoroalkylamine group in several drug candidates, such as Olinciguat, Cevipabulin, and Odanacatib (Fig. 1d) is essential for high potency, and the bioisosteric relationships to amide and amino acid moieties have been recognized in several instances (Fig. 1e)^41–44^. In this respect, the syntheses of homo- and heterofunctionalized, α-trifluoromethyl vicinal diamines^45–52^ and other substituted trifluoropropylamines^52–55^ are demanding.

In this work, we aim to design a versatile selective intermolecular fluorous alkene difunctionalization strategy with the utilization of different nucleophiles to afford valuable functionalized trifluoromethylamines and diamines.

## Results

### Optimization and mechanistic studies

**Fig. 2:**
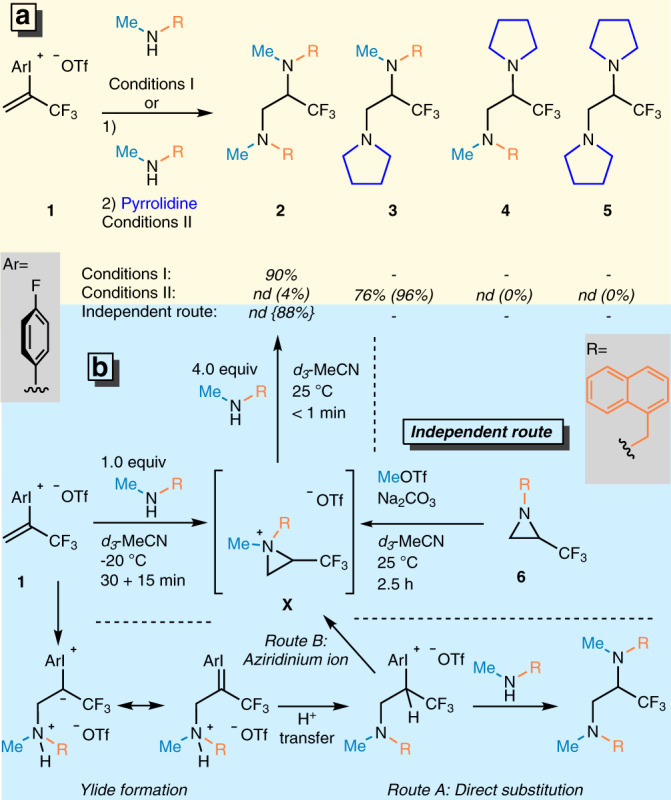
Experimental mechanistic studies. **a** Optimized conditions of homodiamination and heterodiamination procedures and mechanistic studies. Conditions I: **1** (0.20 mmol) added in one portion, *c* = 0.1 M, MeCN, 25 °C, 1 h. Conditions II: (1) **1** (0.15 mmol), MeNHR (0.15 mmol, 1.0 equiv.) added dropwise with syringe pump in 30 min, *c* = 0.05 M, MeCN, −20 °C, 15 min; (2) pyrrolidine (0.375 mmol, 2.5 equiv.) added in one portion, 25 °C, 16 h. Yields are isolated yields; numbers given in parentheses () are GC-MS yields of products, relative to 4-F-iodobenzene. **b** Study of reaction intermediates and resolving their structures by independent route. Number given in {} represents the ^19^F-NMR yield of **2**.

For the realization of our goals on selective alkene difunctionalization to obtain trifluoropropyl amines, the presence of an efficient leaving group (LG) was necessary (Fig. 1f). A hypervalent iodonium moiety^56–63^ could be an appropriate choice for LG, due to its super leaving group ability. Moreover, in alkenyliodonium salts^64–68^ the carbon–carbon bond is activated to accept nucleophiles. A perfect candidate of this reagent class for the realization of our goals is trifluoroalkenyl-iodonium salt **1** as a stable potential synthon^69^ allowing for the vicinal functionalization with two nucleophilic species. We expected that the secondary amine nucleophile would attach to the *β*-carbon due to the excellent Michael-acceptor character of the terminal sp^2^ carbon center and form a stabilized iodonium ylide intermediate first. After a proton transfer step, the iodonium part attached to the sp^3^ carbon center could act as a super leaving group and make the carbon atom more electrophilic to accept a second nucleophile. Theoretically, this step could occur intermolecularly through direct substitution by the second nucleophile (Fig. 2b, Route A) or through aziridinium ion formation (Fig. 2b, Route B) and subsequent ring opening. This is an important mechanistic question of our study.

The first attempts of the construction of the trifluoromethylated ethylene diamine backbone via diamination were performed with *N*-methyl-naphthylmethylamine as the nucleophile in CH_2_Cl_2_ at 25 °C in the presence of Na_2_CO_3_ base (Supplementary Table 1). We found that the desired homofunctionalized diamine was formed even in the presence of 1 equivalent of the amine nucleophile, albeit in 12% isolated yield. The study of different molar ratios of amine and inorganic base in various solvents led to conditions I (Fig. 2a) which ensures the isolation of the target compound **2** in 90% yield after 1 h reaction time. To determine the operative mechanistic path, the reaction was monitored with ^19^F-NMR spectroscopy and complete conversion was observed within 15 s (Supplementary Fig. 1) in the presence of an excess amount (4 equiv.) of amine with only the ^19^F signal of the diaminated product observed in the spectra. In contrast, utilizing one equivalent of amine led to the formation of a diastereomeric mixture of the corresponding aziridinium ion intermediates^70^ (Fig. 2b and Supplementary Figs. 8–10). To prove the presence of this intermediate, we synthesized the putative aziridinium ion in situ through an independent route from aziridine **6** and MeOTf in *d*_3_-MeCN at 25 °C (Fig. 2b). Gratifyingly, we observed the complete consumption of aziridine **6** in 2.5 h and the appearance of a new signal in the ^19^F-NMR spectra at −63.65 ppm, which we assign to aziridinium intermediate **X** (Supplementary Fig. 10). This peak was identical with the major intermediate’s signal observed in the diamination NMR experiment (Supplementary Fig. 9), and this observation supports the presence of an aziridinium ion as the key intermediate of this diamination process. We postulate that a minor intermediate also observed is the other diastereomer which cannot be formed as easily in the *N*-alkylation route due to the stereoselective attack of the methyl triflate governed by the steric repulsion of trifluoromethyl group.

In the second step of the mechanistic study, the aziridinium intermediate **X** generated from aziridine **6** was reacted with 4 equivalents of *N*-methyl-naphthylmethylamine (Fig. 2b), and immediate formation of the corresponding diamine **2** through ring opening was observed in the ^19^F-NMR spectrum (Supplementary Fig. 10). This result is identical to the outcome of the reaction between the trifluoropropenyliodonium salt **1** and amine and confirms the intermediacy of aziridinium ion in the transformation.

With these experiments our key question on the mechanism was answered and the aziridinium path was confirmed. However, we aimed to expand the synthetic potential of the aziridine ring formation. The major synthetic challenge of this methodology development is the control of selectivity in the ring opening step. To overcome this challenge, we performed further optimization to develop a method for selective heterodifunctionalization reactions which afford the desired trifluoropropylamines.

In general, ring opening of substituted aziridinium ions by nucleophiles leads to mixture of constitutional isomers, which is governed by the nature of substituents of the heterocycle^70^. Among these substituents, the trifluoromethyl group attached to the aziridine carbon lowers the activation energy of the ring opening considerably at both *C-2* and *C-3* position, but in general ring opening is favored at the *C-3* position both thermodynamically and kinetically^71^. Taking the advantage of the electronic influence on the ring opening could enable the selective heterodifunctionalizations of the trifluoromethyl alkenes via aziridinium intermediates. However, the implementation of this approach is challenging and requires precise control of intermediate generation. Thus, the aziridinium ion formation from the iodonium salt should be quantitative with complete consumption of 1.00 equivalent of amine. To reach this goal we optimized the reaction conditions considering addition time, concentration, and temperature (Supplementary Table 2) and found the most efficient reaction parameters culminated in conditions II. After the in situ formation of the aziridinium ion, pyrrolidine was added and the heterofunctionalized diamine **3** was isolated with high chemoselectivity and complete regioselectivity (Fig. 2a, Conditions II) in 76% chemical yield.

### Substrate scope

**Fig. 3:**
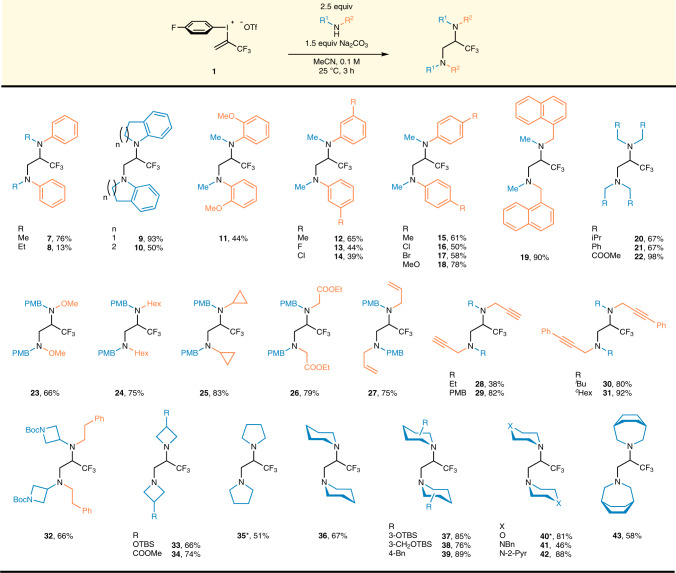
Scope of the homofunctionalized diamine synthesis. **1** (0.2–0.3 mmol), R^1^R^2^NH (2.5 equiv.), Na_2_CO_3_ (1.5 equiv.), MeCN, *c* = 0.1 M, 25 °C, 3 h. *Isolated as the corresponding bis(ammonium) trifluoroacetate salt (2 moles of trifluoroacetate ion per mole product).

After the optimization studies, the scope of this homodiamination reaction was explored under the optimized conditions I. (Fig. 3) First of all, aromatic amines were evaluated regarding substituent effects on the *N*-atom. The simplest, *N*-Me-aniline afforded diamine (**7**) in 76% yield, but the more sterically demanding *N*-Et-aniline performed poorly and provided diamine **8** only in 13% yield. To prove the steric sensitivity of the amine nucleophile, the cyclic analog of *N*-Et-aniline, indoline was tested in the homodiamination reaction, and the corresponding diamine **9** was obtained in excellent 93% yield (reactivity of these amines was compared with ^19^F-NMR, for further details see Supplementary Figs. 2–5). Increasing the steric bulk of the cyclic nucleophile, we used six-membered tetrahydroquinoline and the corresponding diamine **10** has formed with considerably lower yield. The steric and electronic effects of substituents attached to phenyl ring of the aniline were investigated in case of *N*-Me-aniline derivatives. Although, the presence of methoxy group in *ortho*-position allowed the diamination and product **11** was isolated in 44% yield (Fig. 3), other tested groups in the *ortho*-position had a deleterious effect on the transformation (Supplementary Fig. 6). Methyl, halogen (F, Cl, Br), and methoxy functionalities are well tolerated in *meta*- and *para*-positions of the phenyl ring and the corresponding diamines (**12**–**18**) were isolated in the 39–78% yield range. Additionally, the importance of the nucleophilic character of the nucleophile was demonstrated with various anilines having electron withdrawing groups such as COOMe, CF_3_, NO_2_. In these cases, we were not able to obtain the desired diamines (Supplementary Fig. 6).

After the study of aniline derivatives, we used different aliphatic secondary amines. Naphthalene substituted dimethylamine afforded diamine **19** in an excellent 90% yield. Increasing the steric bulk of the amine, reactions with diisobutylamine and dibenzylamine led to 67% yields of products (**20**, **21**). In contrast to the aromatic systems, the presence of an electron withdrawing ester group on the alkyl chain was well tolerated and iminodiacetic acid ester provided diamine **22** in almost quantitative yield. The *para*-methoxybenzyl (PMB) protected amines could be applied generally (**23**–**27**) and their substituent effects were studied. Regarding the electronic effects, a hydroxylamine derived nucleophile with high electron density is accepted and resulted in the isolation of the appropriate product (**23**) in 66% yield. The presence of a simple alkyl chain, such as *n*-hexyl, in the amine afforded product **24** in 75% yield. The highly strained cyclopropyl group could also be utilized and product **25** was obtained in 83% yield. The presence of an electron withdrawing ester functionality on the alkyl chain had no negative influence on the transformation and diamine **26** was isolated in 79% yield. The reactivity study revealed that amines with unsaturated hydrocarbon chains are also suitable for the diamination, with allyl derivative **27** isolated in 75% yield and the terminal and internal alkyne derivatives (**28**–**31**) could also be prepared in **38**–**92**% yields. Diamine **32** represents the feasibility of a *t*-butoxycarbonyl protected amine and the strained azetidinyl ring motif.

Next, saturated cyclic amines were examined from four- to seven-membered rings. In this series we could prepare the substituted azetidine derivatives (**33**–**34**) in 66 and 74% yields, respectively. Unsubstituted pyrrolidine and piperidine proved to be excellent reaction partners and products **35** and **36** were isolated in 51 and 67% yield, respectively, which suggest that ring size has little impact on the diamination. Both substituents on the piperidine ring and heteroatom replacements within the ring were also evaluated. The sterically demanding benzyl, *t*-butyldimethylsilyloxy and the homolog *t*-butyldimethyl-silyloxymethylene substituents at *C-4* and *C-3* positions are tolerated and products **37**–**39** were isolated in 76–89% yield. As expected, the ether functional group is tolerated as we obtained the morpholine derived diamine **40** in 81% yield. Both aliphatic tertiary and the 2-pyridyl substituted aza- replacements were compatible with the reaction conditions and diamines **41** and **42** were obtained in 46 and 88% yield, respectively. Additionally, the bridged 3-azabicyclo[3.2.2]nonane provided the corresponding diamine **43** in 58% yield.

**Fig. 4:**
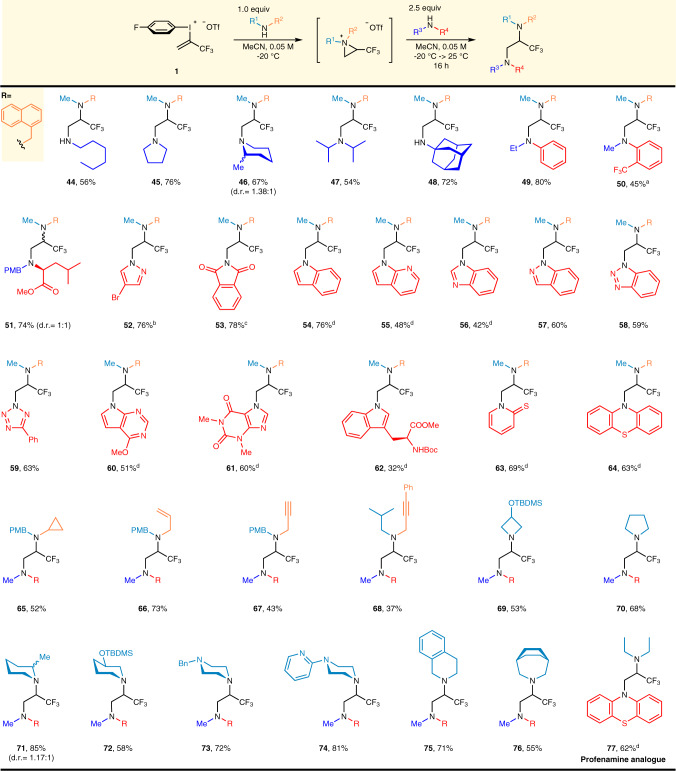
Scope of heterodiamination. 1st stage: **1** (0.2–0.3 mmol) added at 0.3 mmol h^−1^ rate, R^1^R^2^NH (1.0 equiv.), MeCN, *c* = 0.05 M, −20 °C, 0.16 h mmol^−1^; 2nd stage: R^3^R^4^NH or Nu (2.5 equiv.), −20 → 25 °C, 16 h. ^a^Additional base: 2.5 equiv. 2,6-di-*tert*-butylpyridine. ^b^Additional base: 2.5 equiv. NaH. ^c^Potassium phthalimide was used. ^d^2nd stage: 2.6 equiv. R^3^R^4^NH, 2.5 equiv. LiHMDS, 2.5 equiv. HMPA, THF, −78 → 25 °C, 16 h.

To expand the applicability of the heterodiamination method under the optimized conditions II, we evaluated various amines at the first and second stages of the reaction. (Fig. 4) As a starting point we used *N*-methyl-naphthylmethylamine at the first stage of the reaction sequence to form the aziridinium intermediate **X** in situ. Ring opening of intermediate **X** is allowed by primary amine (**44**) at the second stage of the reaction sequence. Subsequently, we used saturated cyclic amines and found these amines can be utilized efficiently and provided the corresponding diamines (**45**, **46**) in 76 and 67% yield, respectively. Further increasing the steric demands of the amine at the second stage by introducing two *i*-propyl or one 1-adamantyl groups resulted also the formation of heterodiaminated products **47** and **48** in 54 and 72% yield, respectively. Among aromatic amines, *N*-Et-aniline provided diamine **49** in good yield while the more sterically demanding and considerably less nucleophilic 2-trifluoromethyl-*N*-methylaniline was found to be reactive enough to afford **50** in 45% yield in the presence of di-*t*-butylpyridine. Still, the more complex structure of methyl *N*-PMB-l-leucinate enables this transformation and the diastereomeric mixture of diamine derivative **51** was obtained in 74% yield without any difficulties. As a heterocyclic amine representative, 4-Br-pyrazole was evaluated in the ring opening step, after its deprotonation, to obtain heterocyclic diamine **52** in 76% yield. Reactivity of imide functionality was also evaluated, thus potassium phthalimide was reacted with aziridinium intermediate and afforded the corresponding product (**53**) in 78% yield. To further expand the substrate scope, we performed the heterodiamination reaction with various *N*-heterocyclic scaffolds such as indole, azaindole, benzimidazole, indazole, benzotriazole, tetrazole, deazapurine, theophylline, tryptophane, 2-mercaptopyridine, phenothiazine and isolated the corresponding heterodiaminated products (**54**–**64**) in 32–76% yield range.

Next, we examined the applicability of various amines at the first stage of the reaction while *N*-methyl-naphthylmethylamine was utilized at the second stage of the reaction sequence to gain complementary structures. Likewise to the homodiamination procedure, we started the study with the evaluation of aromatic amines, but unfortunately we could only observe the formation of the corresponding homofunctionalized diamines in the case of *N-Me-* and *N-Et*-aniline and the cyclic analog, indoline (Supplementary Fig. 11). We, therefore, turned our attention to aliphatic amines, and the reactivity of various PMB-protected amines was studied. In this series, the cyclopropyl substituent is tolerated and derivative **65** was obtained in 52% yield.

The presence of an allyl group on the amine had little impact on the efficiency as diamine **66** was isolated in 73% yield. Triple bonds are accepted in form of both terminal and internal alkynes, albeit, the corresponding diamines (**67**, **68**) were obtained in 43 and 37% yield, respectively. It is worth noting the higher reactivity of the corresponding aziridinium intermediates in the case of propargylamines as the homodiaminated products were also formed and their separation was difficult, leading to relatively lower yields. Application of saturated cyclic amines as reactants in first stage of the reaction could lead to the formation of azaspiro-ammonium ions, which might possess different reactivity toward nucleophiles than previous monocyclic intermediates. Indeed, saturated cyclic amines from four- to seven-membered ring-size react in the same manner as the acyclic secondary amines. From this series, the four-membered, TBDMS protected azetidinol derivative **69** could be obtained in 53% yield while the TBDMS protective group remained intact. The simplest five-membered pyrrolidine gave diamine **70** with higher efficiency (68%). Among six-membered piperidines, 2-Me-piperidine afforded product **71** in 85% yield, despite the steric encumbrance. On the other hand, the performance of the TBDMS protected 3-piperidinol (**72**) was comparable to the TBDMS protected azetidinol derivative **69**. *N*-benzyl- (**73**) and *N*-2-pyridyl-piperazine (**74**) derivatives were obtained in 72% and 81% yields, respectively, in spite of the presence of additional basic *N*-atoms. Benzannulated and bridged bicyclic amines are also applicable, tetrahydroisoquinoline and the seven-membered 3-azabicyclo[3.2.2]nonane afforded diamines (**75**, **76**) in 71% and 55% yields, respectively. Finally, we have demonstrated that the trifluoromethyl analog of Profenamine (**77**) could be assembled by the developed heterodiamination procedure.

**Fig. 5:**
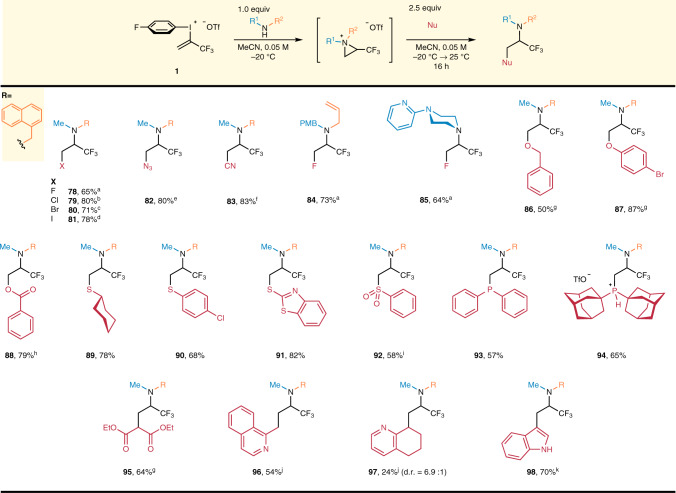
Scope of trifluoropropylamines. 1st stage: **1** (0.2–0.3 mmol) added at 0.3 mmol h^−1^ rate, R^1^R^2^NH (1.0 equiv.), MeCN, *c* = 0.05 M, −20 °C, 0.16 h mmol^−1^; 2nd stage: Nu (2.5 equiv.), −20 °C → 25 °C, 16 h. ^a^Nu = CsF, ^b^Nu = tetrabutylammonium chloride, ^c^Nu = tetrabutylammonium bromide, ^d^Nu = NaI, ^e^Nu = NaN_3_, ^f^Nu = KCN, ^g^NuH was deprotonated previously by 1.0 equiv. NaH in THF, ^h^Nu = sodium benzoate, ^i^Nu = sodium benzenesulfinate, ^j^1st stage: **1** (0.2–0.3 mmol) added at 0.3 mmol h^−1^ rate, R^1^R^2^NH (1.0 equiv.), THF, *c* = 0.05 M, −55 °C, 0.16 h mmol^−1^; 2nd stage: 1.1 equiv. NuH, 1.15 equiv. *n*-BuLi in THF at −78 → 25 °C, 16 h. ^k^1st stage: **1** (0.2–0.3 mmol) added at 0.3 mmol h^−1^ rate, R^1^R^2^NH (1.0 equiv.), MeCN, *c* = 0.05 M, −20 °C, 0.16 h mmol^−1^; 2nd stage: 1.2 equiv. indole, 1.2 equiv. Et_2_Zn, PhMe, 25 °C, 16 h.

After the extensive study of heterodiamination, we took the opportunity to expand the scope of heterofunctionalization under conditions II in the synthesis of substituted trifluoropropylamines (Fig. 5). Among the halogens, we first used the least nucleophilic fluoride ion, and successfully isolated the appropriate tetrafluoropropylamine derivative **78** in 65% yield. Other halides and pseudohalides were utilized in the second reaction step and the corresponding products (**79**–**83**) were isolated in 71–83% yield range. Interestingly, the 1,1,1,3-tetrafluoropropyl moiety previously had limited options in the literature for its preparation^50,72^. Therefore, *N*-allyl-PMB-amine and *N*-pyrid-2-yl-piperazine were utilized to demonstrate the feasibility of conditions II in the synthesis of various tetrafluoropropylamines (**84**, **85**). We also examined the applicability of different chalcogen nucleophiles. From this series, deprotonated benzylalcohol gave derivative **86** in 50% yield while 4-bromophenol and sodium benzoate afforded products **87** and **88** in 87 and 79% yield, respectively. Sulfur nucleophiles could be utilized without any additional base and cycloalkyl (**89**), aryl (**90**), and heteroaryl (**91**) substituted thioethers were obtained. On the other hand, applying sodium phenylsulfinate, the sulfone **92** could be accessed directly. Expanding the variety of heteroatoms at β-position, we performed the reaction with two different phosphines and prepared aminophosphanes **93**, **94** having potential bidentate *N*-*P* ligand structure. Additionally, to prove the applicability of *C*-nucleophiles, deprotonated diethyl malonate was reacted with the aziridinium intermediate, and the corresponding malonate derivative **95** was obtained in 64% yield. Finally, methylisoquinoline and tetrahydroquinoline were deprotonated with *n*-BuLi and converted to the corresponding trifluoroalkylamines (**96**, **97**) in the 24–54% yield range, while deprotonation of indole with Et_2_Zn gave rise to the selective alkylation at the *C-3* position of the heteroaromatic ring to provide **98** in 70% yield.

## Discussion

In summary, we have developed an efficient intermolecular three-component diamination strategy which enables the selective 1,2-difunctionalization of C–C double bonds with two different nucleophilic agents in the absence of transition metal catalysts. Mild reaction conditions allowed the utilization of various functional groups and substitution patterns on amine nitrogen atom, providing diverse set of homo- and heterofunctionalized diamines from trifluoropropyl synthon **1**. Additionally, we demonstrated the applicability of various nucleophiles such as halides, oxygen, sulfur, phosphorous and carbon nucleophiles for the synthesis of β-substituted trifluoropropylamines. The developed methodology offers a versatile synthetic tool to build high structural diversity on trifluoropropyl backbone from readily available amines and other simple nucleophiles.

## Methods

### General procedure for the synthesis of homofunctionalized diamines under Conditions I

An 8 mL vial was charged with stirring bar, appropriate amine (2.5 equiv.) and acetonitrile (1 mL/0.1 mmol). To the vigorously stirred mixture, sodium carbonate (1.5 equiv.) was added, then (4-fluorophenyl)(3,3,3-trifluoroprop-1-en-2-yl)iodonium trifluoromethanesulfonate (**1**) (1 equiv.) was added portionwise. The reaction mixture was stirred for 3 h, diluted with dichloromethane and concentrated onto Celite® under reduced pressure. Residue was purified by flash column chromatography (hexanes:ethyl acetate = 100:0 → 50:50).

### General procedure for the synthesis of heterofunctionalized diamines under Conditions II

A 30 mL screwed cap vial was charged with rare-earth magnetic stirring bar, **1** (1 equiv.) and acetonitrile (1 mL/0.1 mmol), then the vial was sealed with Teflon septa and screwed cap. The stirred reaction mixture was cooled to −20 °C (bath temp = −23 °C), then solution of first amine (1 equiv., 0.1 M in acetonitrile) was added dropwise (0.3 mmol h^−1^) by syringe pump. Subsequently, the reaction mixture was stirred for 10 min/0.1 mmol, then the second amine or other nucleophile (2.5 equiv.) was added in one portion. The mixture was allowed to warm to room temperature over 16 h, then was concentrated onto Celite under reduced pressure. The obtained residue was purified by flash column chromatography. Gradient elution was performed by using either hexanes:ethyl acetate or hexanes:diisopropyl ether eluent system, according to TLC elution experiments.

## Supplementary information

Supplementary Information

## Data Availability

The authors declare that the main data supporting the findings of this study are available within the article and its Supplementary Information file. Extra data are available from the corresponding author upon request.
