# Supplementary material for: Vicinal difunctionalization of carbon–carbon double bond for the platform synthesis of trifluoroalkyl amines
Source: Nat Commun. 2020 Nov 23;11:5924. doi: 10.1038/s41467-020-19748-z (PMC7683743; doi:10.1038/s41467-020-19748-z)
Supplement: Supplementary file 1 — Supplementary Information [file 41467_2020_19748_MOESM1_ESM.pdf]

Vicinal difunctionalization of carbon–carbon double bond for the platform synthesis of trifluoroalkyl amines

Béke, et al.

Supplementary Information

for

Vicinal difunctionalization of carbon–carbon double bond for the platform synthesis of trifluoroalkyl amines

by

Ferenc Béke, Ádám Mészáros, Ágnes Tóth, Bence Béla Botlik, Zoltán Novák

ELTE Catalysis and Organic Synthesis Research Group,
Institute of Chemistry, Eötvös University,
Pázmány Péter stny. 1/a H-1117 Budapest, Hungary
E-mail: novakz@elte.hu
Web: zng.elte.hu

Table of contents

General conditions **12**

Preparation of starting materials **13**

*Synthesis of compound **1*** 13

General procedure for the synthesis of *p*-methoxybenzyl protected amines[2] 14

N-(4-methoxybenzyl)prop-2-en-1-amine[2] (**A1**) 14

Methyl (4-methoxybenzyl)-*L*-leucinate[3] (**A2**) 15

Ethyl (4-methoxybenzyl)-*L*-phenylalaninate[4] (**A3**) 15

N-(4-methoxybenzyl)adamantan-1-amine[5] (**A4**) 16

N-(4-methoxybenzyl)cyclopropanamine[6] (**A5**) 16

(*R*)-*N*-(4-methoxybenzyl)-1-phenylethan-1-amine[7] (**A6**) 17

Ethyl (4-methoxybenzyl)glycinate[8] (**A7**) 17

N-(4-methoxybenzyl)hexan-1-amine[9] (**A8**) 17

N-(4-methoxybenzyl)heptan-2-amine (**A9**) 18

N-(4-methoxybenzyl)octan-1-amine[10] (**A10**) 18

N-(4-methoxybenzyl)aniline[11] (**A11**) 19

N-(4-methoxybenzyl)-3-nitroaniline[12] (**A12**) 19

3-phenylprop-2-yn-1-ol[13] 20

3-Phenylprop-2-yn-1-yl 4-methylbenzenesulfonate[14] 20

General procedure for the synthesis of propargyl amines 21

N-isobutyl-3-phenylprop-2-yn-1-amine[15] (**A13**) 21

N-(3-phenylprop-2-yn-1-yl)cyclohexanamine[16] (**A14**) 21

N-hexyl-1-(phenylethynyl)cyclohexan-1-amine[17] (**A15**) 22

N-(4-methoxybenzyl)prop-2-yn-1-amine[18] (**A16**) 22

tert-butyl 3-(phenethylamino)azetidine-1-carboxylate (**A17**) 23

3-((*tert*-butyldimethylsilyl)oxy)azetidine (**A18**) 23

3-(methoxycarbonyl)azetidin-1-ium trifluoromethanesulfonate (**A19**) 24

General procedure for the synthesis of piperidine derivatives 24

3-((*tert*-butyldimethylsilyl)oxy)piperidine (**A20**) 25

3-(((*tert*-butyldimethylsilyl)oxy)methyl)piperidine (**A21**) 25

*Synthesis of compound **6*** 26

Supplementary Table 1: Optimization of reaction conditions of homodiamination **27**

Reaction monitoring by ^{19}F-NMR **28**

Supplementary Figure 1: Reaction scheme and monitoring of homodiamination reaction with of *N*-methyl-1-(naphthalen-1-yl)methanamine....28
Supplementary Figure 2: Reaction monitoring of homodiamination reaction with *N*-methylaniline by ^{19}F-NMR-spectroscopy....29
Supplementary Figure 3: Reaction monitoring of homodiamination reaction with *N*-ethylaniline by ^{19}F-NMR-spectroscopy....30
Supplementary Figure 4: GC-MS TIC of homodiamination reaction with *N*-ethylaniline....31
Supplementary Figure 5: Reaction monitoring of homodiamination reaction with indoline by ^{19}F-NMR-spectroscopy....32
General procedure for the synthesis of homofunctionalized diamines 33
*Synthesis of compound **7***....33
*Synthesis of compound **8***....33
*Synthesis of compound **9***....34
*Synthesis of compound **10***....34
*Synthesis of compound **11***....35
*Synthesis of compound **12***....35
*Synthesis of compound **13***....36
*Synthesis of compound **14***....37
*Synthesis of compound **15***....37
*Synthesis of compound **16***....38
*Synthesis of compound **17***....38
*Synthesis of compound **18***....39
*Synthesis of compound **19***....39
*Synthesis of compound **20***....40
*Synthesis of compound **21***....40
*Synthesis of compound **22***....41
*Synthesis of compound **23***....41
*Synthesis of compound **24***....42
*Synthesis of compound **25***....42
*Synthesis of compound **26***....43
*Synthesis of compound **27***....43
*Synthesis of compound **28***....44
*Synthesis of compound **29***....44
*Synthesis of compound **30***....45

*Synthesis of compound **31***......46
*Synthesis of compound **32***......46
*Synthesis of compound **33***......47
*Synthesis of compound **34***......47
*Synthesis of compound **35***......48
*Synthesis of compound **36***......48
*Synthesis of compound **37***......49
*Synthesis of compound **38***......49
*Synthesis of compound **39***......50
*Synthesis of compound **40***......50
*Synthesis of compound **41***......51
*Synthesis of compound **42***......51
*Synthesis of compound **43***......52
Supplementary Figure 6: Unsuccessful substrates under conditions of homodiamination procedure......52
Optimization of reaction conditions of heterodiamination......53
Supplementary Figure 7: Reaction scheme of heterodiamination procedure......53
Supplementary Table 2: Optimization of reaction conditions of heterodiamination......53
Study of reaction mechanism by ^{19}F-NMR spectroscopy......55
Supplementary Figure 8: Reaction scheme and monitoring of homodiamination reaction by ^{19}F-NMR-spectroscopy, in the presence of one equivalent amine......55
Supplementary Figure 9: Reaction scheme of the first stage of reaction conditions II and study by ^{19}F-NMR spectroscopy......56
Supplementary Figure 10: Generation of intermediate **X** from trifluoromethyl aziridine **6** and its reaction with excess amine......57
Synthesis of heterofunctionalized diamines......58
General procedure A......58
General procedure B......58
General procedure C......58
General procedure D......59
*Synthesis of compound **44***......59
*Synthesis of compound **45***......60
*Synthesis of compound **46***......60
*Synthesis of compound **47***......61

Synthesis of compound ***48***........61
Synthesis of compound ***49***........62
Synthesis of compound ***50***........63
Synthesis of compound ***51***........63
Synthesis of compound ***52***........64
Synthesis of compound ***53***........65
Synthesis of compound ***54***........65
Synthesis of compound ***55***........66
Synthesis of compound ***56***........67
Synthesis of compound ***57***........67
Synthesis of compound ***58***........68
Synthesis of compound ***59***........69
Synthesis of compound ***60***........69
Synthesis of compound ***61***........70
Synthesis of compound ***62***........71
Synthesis of compound ***63***........71
Synthesis of compound ***64***........72
Synthesis of compound ***65***........73
Synthesis of compound ***66***........73
Synthesis of compound ***67***........74
Synthesis of compound ***68***........75
Synthesis of compound ***69***........75
Synthesis of compound ***70***........76
Synthesis of compound ***71***........76
Synthesis of compound ***72***........77
Synthesis of compound ***73***........78
Synthesis of compound ***74***........78
Synthesis of compound ***75***........79
Synthesis of compound ***76***........80
Synthesis of compound ***77***........80
Synthesis of compound ***78***........81
Synthesis of compound ***79***........81

*Synthesis of compound **80***......82
*Synthesis of compound **81***......82
*Synthesis of compound **82***......83
*Synthesis of compound **83***......83
*Synthesis of compound **84***......84
*Synthesis of compound **85***......84
*Synthesis of compound **86***......85
*Synthesis of compound **87***......86
*Synthesis of compound **88***......86
*Synthesis of compound **89***......87
*Synthesis of compound **90***......87
*Synthesis of compound **91***......88
*Synthesis of compound **92***......89
*Synthesis of compound **93***......89
*Synthesis of compound **94***......90
*Synthesis of compound **95***......90
*Synthesis of compound **96***......91
*Synthesis of compound **97***......92
*Synthesis of compound **98***......92
Supplementary Figure 11: Unsuccessful substrates under conditions of aziridinium ion intermediate generation......93
Supplementary Figure 12: Ethylene diamine and trifluoroethylamine moieties in natural products and drugs......94
NMR & IR spectra......95
Supplementary Figure 13: *^{1}H-NMR for compound **1***......96
Supplementary Figure 17: *^{1}H-NMR for compound **A1***......100
Supplementary Figure 19: *^{1}H-NMR for compound **A2***......102
Supplementary Figure 21: *^{1}H-NMR for compound **A3***......104
Supplementary Figure 23: *^{1}H-NMR for compound **A4***......106
Supplementary Figure 25: *^{1}H-NMR for compound **A5***......108
Supplementary Figure 27: *^{1}H-NMR for compound **A6***......110
Supplementary Figure 29: *^{1}H-NMR for compound **A7***......112
Supplementary Figure 31: *^{1}H-NMR for compound **A8***......114

Supplementary Figure 33: *^{1}H-NMR for compound* ***A9*** 116
Supplementary Figure 35: *^{1}H-NMR for compound* ***A10*** 118
Supplementary Figure 37: *^{1}H-NMR for compound* ***A11*** 120
Supplementary Figure 39: *^{1}H-NMR for compound* ***A12*** 122
Supplementary Figure 41: *^{1}H-NMR for compound* ***3-phenylprop-2-yn-1-ol*** 124
Supplementary Figure 43: *^{1}H-NMR for compound* ***3-phenylprop-2-yn-1-yl 4-methylbenzenesulfonate*** 126
Supplementary Figure 45: *^{1}H-NMR for compound* ***A13*** 128
Supplementary Figure 47: *^{1}H-NMR for compound* ***A14*** 130
Supplementary Figure 49: *^{1}H-NMR for compound* ***A15*** 132
Supplementary Figure 51: *^{1}H-NMR for compound* ***A16*** 134
Supplementary Figure 53: *^{1}H-NMR for compound* ***A17*** 136
Supplementary Figure 56: *^{1}H-NMR for compound* ***A18*** 139
Supplementary Figure 58: *^{1}H-NMR for compound* ***A19*** 141
Supplementary Figure 61: *^{1}H-NMR for compound* ***A20*** 144
Supplementary Figure 63: *^{1}H-NMR for compound* ***A21*** 146
Supplementary Figure 65: *^{1}H-NMR for compound* ***6*** 148
Supplementary Figure 68: *^{1}H-NMR for compound* ***7*** 151
Supplementary Figure 72: *^{1}H-NMR for compound* ***8*** 155
Supplementary Figure 76: *^{1}H-NMR for compound* ***9*** 159
Supplementary Figure 80: *^{1}H-NMR for compound* ***10*** 163
Supplementary Figure 84: *^{1}H-NMR for compound* ***11*** 167
Supplementary Figure 88: *^{1}H-NMR for compound* ***12*** 171
Supplementary Figure 92: *^{1}H-NMR for compound* ***13*** 175
Supplementary Figure 96: *^{1}H-NMR for compound* ***14*** 179
Supplementary Figure 100: *^{1}H-NMR for compound* ***15*** 183
Supplementary Figure 104: *^{1}H-NMR for compound* ***16*** 187
Supplementary Figure 108: *^{1}H-NMR for compound* ***17*** 191
Supplementary Figure 112: *^{1}H-NMR for compound* ***18*** 195
Supplementary Figure 116: *^{1}H-NMR for compound* ***19*** 199
Supplementary Figure 120: *^{1}H-NMR for compound* ***20*** 203
Supplementary Figure 124: *^{1}H-NMR for compound* ***21*** 207
Supplementary Figure 128: *^{1}H-NMR for compound* ***22*** 211

Supplementary Figure 132: *^{1}H-NMR for compound **23***......215
Supplementary Figure 136: *^{1}H-NMR for compound **24***......219
Supplementary Figure 140: *^{1}H-NMR for compound **25***......223
Supplementary Figure 144: *^{1}H-NMR for compound **26***......227
Supplementary Figure 148: *^{1}H-NMR for compound **27***......231
Supplementary Figure 152: *^{1}H-NMR for compound **28***......235
Supplementary Figure 156: *^{1}H-NMR for compound **29***......239
Supplementary Figure 160: *^{1}H-NMR for compound **30***......243
Supplementary Figure 164: *^{1}H-NMR for compound **31***......247
Supplementary Figure 168: *^{1}H-NMR for compound **32***......251
Supplementary Figure 172: *^{1}H-NMR for compound **33***......255
Supplementary Figure 176: *^{1}H-NMR for compound **34***......259
Supplementary Figure 180: *^{1}H-NMR for compound **35***......263
Supplementary Figure 184: *^{1}H-NMR for compound **36***......267
Supplementary Figure 188: *^{1}H-NMR for compound **37***......271
Supplementary Figure 192: *^{1}H-NMR for compound **38***......275
Supplementary Figure 196: *^{1}H-NMR for compound **39***......279
Supplementary Figure 200: *^{1}H-NMR for compound **40***......283
Supplementary Figure 204: *^{1}H-NMR for compound **41***......287
Supplementary Figure 208: *^{1}H-NMR for compound **42***......291
Supplementary Figure 212: *^{1}H-NMR for compound **43***......295
Supplementary Figure 216: *^{1}H-NMR for compound **44***......299
Supplementary Figure 220: *^{1}H-NMR for compound **45***......303
Supplementary Figure 227: *^{1}H-NMR for compound **46***......311
Supplementary Figure 231: *^{1}H-NMR for compound **47***......315
Supplementary Figure 235: *^{1}H-NMR for compound **48***......319
Supplementary Figure 239: *^{1}H-NMR for compound **49***......323
Supplementary Figure 243: *^{1}H-NMR for compound **50***......327
Supplementary Figure 247: *^{1}H-NMR for compound **51***......331
Supplementary Figure 251: *^{1}H-NMR for compound **52***......335
Supplementary Figure 255: *^{1}H-NMR for compound **53***......339
Supplementary Figure 259: *^{1}H-NMR for compound **54***......343

Supplementary Figure 266: ^{1}*H-NMR for compound* ***55*** 350
Supplementary Figure 270: ^{1}*H-NMR for compound* ***56*** 354
Supplementary Figure 274: ^{1}*H-NMR for compound* ***57*** 358
Supplementary Figure 278: ^{1}*H-NMR for compound* ***58*** 362
Supplementary Figure 282: ^{1}*H-NMR for compound* ***59*** 366
Supplementary Figure 290: ^{1}*H-NMR for compound* ***60*** 374
Supplementary Figure 297: ^{1}*H-NMR for compound* ***61*** 381
Supplementary Figure 305: ^{1}*H-NMR for compound* ***62*** 389
Supplementary Figure 312: ^{1}*H-NMR for compound* ***63*** 396
Supplementary Figure 316: ^{1}*H-NMR for compound* ***64*** 400
Supplementary Figure 320: ^{1}*H-NMR for compound* ***65*** 404
Supplementary Figure 324: ^{1}*H-NMR for compound* ***66*** 408
Supplementary Figure 328: ^{1}*H-NMR for compound* ***67*** 412
Supplementary Figure 332: ^{1}*H-NMR for compound* ***68*** 416
Supplementary Figure 336: ^{1}*H-NMR for compound* ***69*** 420
Supplementary Figure 340: ^{1}*H-NMR for compound* ***70*** 424
Supplementary Figure 348: ^{1}*H-NMR for compound* ***71*** 432
Supplementary Figure 352: ^{1}*H-NMR for compound* ***72*** 436
Supplementary Figure 356: ^{1}*H-NMR for compound* ***73*** 440
Supplementary Figure 360: ^{1}*H-NMR for compound* ***74*** 444
Supplementary Figure 364: ^{1}*H-NMR for compound* ***75*** 448
Supplementary Figure 368: ^{1}*H-NMR for compound* ***76*** 452
Supplementary Figure 372: ^{1}*H-NMR for compound* ***77*** 456
Supplementary Figure 376: ^{1}*H-NMR for compound* ***78*** 460
Supplementary Figure 380: ^{1}*H-NMR for compound* ***79*** 464
Supplementary Figure 384: ^{1}*H-NMR for compound* ***80*** 468
Supplementary Figure 388: ^{1}*H-NMR for compound* ***81*** 472
Supplementary Figure 392: ^{1}*H-NMR for compound* ***82*** 476
Supplementary Figure 396: ^{1}*H-NMR for compound* ***83*** 480
Supplementary Figure 400: ^{1}*H-NMR for compound* ***84*** 484
Supplementary Figure 404: ^{1}*H-NMR for compound* ***85*** 488
Supplementary Figure 408: ^{1}*H-NMR for compound* ***86*** 492

Supplementary Figure 412: *^{1}H-NMR for compound* ***87*** 496
Supplementary Figure 416: *^{1}H-NMR for compound* ***88*** 500
Supplementary Figure 420: *^{1}H-NMR for compound* ***89*** 504
Supplementary Figure 424: *^{1}H-NMR for compound* ***90*** 508
Supplementary Figure 428: *^{1}H-NMR for compound* ***91*** 512
Supplementary Figure 432: *^{1}H-NMR for compound* ***92*** 516
Supplementary Figure 436: *^{1}H-NMR for compound* ***93*** 520
Supplementary Figure 441: *^{1}H-NMR for compound* ***94*** 525
Supplementary Figure 446: *^{1}H-NMR for compound* ***95*** 530
Supplementary Figure 450: *^{1}H-NMR for compound* ***96*** 534
Supplementary Figure 457: *^{1}H-NMR for compound* ***97*** 541
Supplementary Figure 465: *^{1}H-NMR for compound* ***98*** 549
Supplementary References 557

General conditions

Analytical thin-layer chromatography (TLC) was performed on Merck DC pre coated TLC plates with 0.25 mm Kieselgel 60 F_{254}. Visualization was performed by fluorescence quenching under 254 nm irradiation, and staining with *p*-anisaldehyde or potassium-permanganate stains. Purification of the crude products was performed by medium pressure liquid chromatography (instrument: Biotage® Isolera™ Prime, stationary phase: Teledyne ISCO RediSep Rf® Normal phase silica, 24 g silica / cartridge, 20-40 μm particle size, 60 Å avarage pore size, mobile phase: according to TLC elution experiment, 15 mL/min). The ^{1}H-, ^{13}C-, ^{19}F- and ^{31}P-NMR spectra were recorded on a Bruker Avance-250 MHz or a Varian Inova 300 MHz or a Varian Inova 500 MHz spectrometer in $CDCl_3$, CD_2Cl_2, acetonitrile-d_3, methanol-d_4 or acetone-d_6. Chemical shifts are expressed in parts per million (δ) and referenced to residual protiated solvent peaks as internal standards for ^{1}H and ^{13}C nuclei, while ^{19}F and ^{31}P chemical shifts are referenced to $CFCl_3$ and H_3PO_4 (40 w% aqueous solution) external standards, respectively. Coupling constants (J) are reported in Hertz (Hz) and splitting patterns are designated as s (singlet), bs (broad singlet), d (doublet), t (triplet), q (quartet), p (pentet), hept (heptet), dd (doublet of doublets), dt (doublet of triplets), td (triplet of doublets), tt (triplet of triplets), dq (doublet of quartets), qd (quartet of doublets), pd (pentet of doublets), ddt (doublet of doublet of triplets), ddq (doublet of doublet of quartets), dqd (doublet of quartet of doublets), tdq (triplet of doublet of quartets), dqdd (doublet of quartet of doublet of doublets), and m (multiplet). IR spectra were obtained on a Bruker IFS55 spectrometer on a single-reflection diamond ATR unit.

Conversions determined on an Agilent 6890N Gas Chromatograph (30 m x 0.25 mm column with 0.25 μm HP-5MS coating, He carrier gas) and low resolution mass spectrometry was obtained on an Agilent 5973 Mass Spectrometer (Ion source: EI+, 70eV, 230 °C, interface 300 °C). *4-Fluoro-iodobenzene* was used as internal standard, which formed upon decomposition of (4-fluorophenyl)(3,3,3-trifluoroprop-1-en-2-yl)iodonium trifluoromethanesulfonate.

All melting points were measured on Büchi 510 apparatus and are uncorrected.

High-resolution mass spectra were acquired on an Agilent 6230 time-of-flight mass spectrometer equipped with a Jet Stream electrospray ion source in positive ion mode. Injections of 0.1-0.3 μl were directed to the mass spectrometer at a flow rate 0.5 ml/min (70% acetonitrile-water mixture, 0.1 % formic acid), using an Agilent 1260 Infinity HPLC system. Jet Stream parameters: drying

gas (N_2) flow and temperature: 10.0 l/min and 325 °C, respectively; nebulizer gas (N2) pressure: 10 psi; capillary voltage: 4000V; sheath gas flow and temperature: 325°C and 7.5 l/min; TOFMS parameters: fragmentor voltage: 120 V; skimmer potential: 120 V; OCT 1 RF Vpp: 750 V. Full-scan mass spectra were acquired over the m/z range 100-2500 at an acquisition rate of 250 ms/spectrum and processed by Agilent MassHunter B.03.01 software.

Starting materials were obtained from commercial suppliers and were used without further purification. Liquid state amines (diisobutylamine, pyrrolidine, piperidine, 2-methyl-piperidine, 3-methyl-piperidine, 4-benzyl-piperidine, *N*-methyl-1-(naphthalen-1-yl)methanamine and *N*-methylaniline) were dried over and distilled from CaH_2, under argon atmosphere or high vacuum. Solid state amines (3-hydroxy-piperidine, 3-hydroxymethylene-piperidine, 3-azabicyclo[3.2.2]nonane and adamant-1-ylamine) were sublimed under high vacuum. 4-Methoxybenzaldehyde was distilled under high vacuum. Dichloromethane was HPLC grade, while acetonitrile (contained <200 ppm water) and methanol were reagent grade, and used without further purification, tetrahydrofuran was distilled from benzophenone ketyl-sodium prior usage. Toluene was dried over activated 4Å molecular sieves and stored under argon.

Preparation of starting materials

*Synthesis of compound **1***

(4-fluorophenyl)(3,3,3-trifluoroprop-1-en-2-yl)iodonium trifluoromethanesulfonate[1]

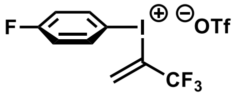

A 40 mL screwed cap vial with a stirring bar was evacuated and refilled with argon 3 times. Trifluoroacetic anhydride (10 mL, 70.5 mmol, 7.2 equiv) and trifluoroacetic acid (77 µL, 1 mmol, 10 mol%) was added via syringe. The mixture was cooled to 0 °C, then hydrogen peroxide (980 µL, 50 wt% in water, 17 mmol, 1.7 equiv) was added dropwise within 2 minutes. 2-Iodo-3,3,3-trifluoropropene (1080 µL, 10 mmol) was added dropwise via syringe. The off-white suspension was kept between 0 °C and 4 °C for 16 hours. After that, the mixture was cooled to -20°C, then dichloromethane (10 mL) was added slowly. Fluorobenzene (1.45 mL, 15.5 mmol, 1.55 equiv) was added dropwise to the suspension, followed by the addition of trifluoromethanesulfonic acid (880 µL, 10 mmol, 1 equiv) to form an emerald green solution. Consider that the temperature is not allowed to reach more than -20 °C throughout the addition of triflic acid! *δ*

The reaction mixture was kept between 0 °C and 4 °C for 6 hours. After that, all volatiles were removed under reduced pressure at 0 °C, protected from light. The brownish green oil was shaken

with cold diethyl ether (20 mL, -20 °C), getting white precipitate. The suspension was kept at -20°C for 12 hours, then the white precipitate was filtered and washed with cold ether 3 times. Title compound was obtained as a white solid (4.37 g, 9.76 mmol, 97.6% yield).

M.p.= 144-147 °C. **^{1}H NMR** (250 MHz, Acetonitrile-d_3) δ 8.20 (dd, J = 7.8, 4.2 Hz, 2H), 7.51 – 7.22 (m, 4H). **^{19}F NMR** (235 MHz, Acetonitrile-d_3) δ -64.53 (3F), -79.40 (3F), -105.44 (1F). **^{13}C NMR** (63 MHz, Acetonitrile-d_3) δ 166.4 (d, J = 254.6 Hz), 145.1 (q, J = 4.7 Hz), 139.9 (d, J = 9.5 Hz), 121.9 (q, J = 321.0 Hz), 121.2 (q, J = 273.8 Hz), 111.8 (q, J = 39.8 Hz), 108.3 (d, J = 3.2 Hz). **IR** (ATR, cm^{-1}) 3094, 3063, 3026, 2973, 2929, 2873, 1598, 1502, 1449, 1353, 1299, 1262, 1232, 1188, 1161, 1141, 1103, 1055, 1038, 973, 913, 888, 851, 795, 744, 692, 615, 579, 522, 468, 413.

General procedure for the synthesis of *p*-methoxybenzyl protected amines[2]

A 40 mL screwed cap vial was chraged with stirring bar, 4 Å molecular sieves (1.5 g, beads), *p*-methoxybenzaldehyde (1.22 mL, 10 mmol) and dichloromethane (10 mL) at room temperature. Amine (12 mmol, 1.2 equiv) or amine hydrochloride (12 mmol, 1.2 equiv) and triethylamine (1.67 mL, 12 mmol, 1.2 equiv) were added and the reaction mixture was monitorized by GC-MS. After complete conversion of *p*-methoxybenzaldehyde (5 min – 24 h) reaction mixture was filtered through a pad of Celite, filter cake was washed with diethylether. Filtrates were combined and evaporated to dryness under reduced pressure. Residue was dissolved in methanol (20 mL) and cooled to 0 °C, then sodium borohydride (454 mg, 12 mmol, 1.2 equiv) was added portionwise over 5 minutes to the stirred mixture. Reaction was allowed to warm room temperature over 16 hours, then concentrated onto Celite *in vacuo*. The obtained residue was purified by flash column chromatograpy (Hexanes:EtOAc = from 100:0 to 50:50).

N-(4-methoxybenzyl)prop-2-en-1-amine[2] (**A1**)

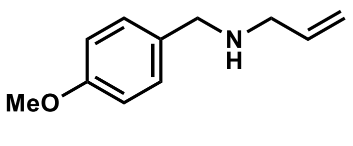

Amine **A1** was synthesized according to general procedure from *p*-methoxybenzaldehyde (1.22 mL, 10 mmol), allylamine (900 uL, 12 mmol, 1.2 equiv) and sodium borohydride (454 mg, 12 mmol, 1.2 equiv) to afford the title compound as a pale yellow liquid (1.46 g, 8.24 mmol, 82% yield).

R$_f$= 0.30 (hexanes:ethyl acetate= 7:3). **LRMS** (EI, 70 eV): m/z (%): 121(100), 176(27), 122(18), 78(15), 56(15), 77(14), 135(12), 134(11), 177(10), 148(9), 91(9). **^{1}H NMR** (250 MHz, $CDCl_3$) δ 7.15 (d, J = 8.5 Hz, 2H), 6.77 (d, J = 8.6 Hz, 2H), 5.84 (ddt, J = 6.9, 5.9 Hz, 1H), 5.11 (dd, J = 17.2, 1.7 Hz, 1H), 5.02 (dd, J = 10.3, 1.2 Hz, 1H), 3.69 (s, 3H), 3.63 (s, 2H), 3.17 (bs, 2H), 1.52 (bs, 1H). **^{13}C NMR** (63 MHz, $CDCl_3$) δ 158.6, 136.8, 132.3, 129.3, 115.9, 113.7, 55.1, 52.6, 51.6.

IR (ATR, cm^{-1}) 3073, 3002, 2971, 2932, 2911, 2834, 1738, 1611, 1510, 1455, 1442, 1418, 1364, 1300, 1241, 1173, 1103, 1034, 994, 916, 811, 771, 702, 637, 561, 516.

Methyl (4-methoxybenzyl)-*L*-leucinate[3] (**A2**)

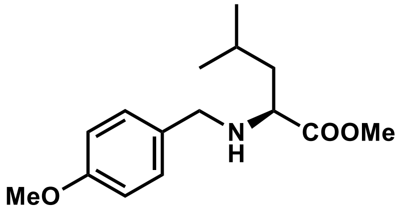

Amine **A2** was synthesized according to general procedure from *p*-methoxybenzaldehyde (1.22 mL, 10 mmol), methyl *L*-leucinate hydrochloride (2.18 g, 12 mmol, 1.2 equiv), triethylamine (1.67 mL, 12 mmol, 1.2 equiv) and sodium borohydride (454 mg, 12 mmol, 1.2 equiv) to afford the title compound as a pale yellow oil (2.28 g, 8.61 mmol, 86% yield).

R_f= 0.38 (hexanes:ethyl acetate= 10:1). **LRMS** (EI, 70 eV): m/z (%): 207(15), 206(100), 137(7), 136(75), 122(62), 121(100), 106(5), 91(18), 90(8), 89(8), 78(26), 77(26), 65(6). **^{1}H NMR** (250 MHz, $CDCl_3$) δ 7.2 (d, J = 8.6 Hz, 2H), 6.8 (d, J = 8.7 Hz, 2H), 3.7 (s, 3H), 3.6 (s, 3H), 3.5 (d, J = 12.7 Hz, 1H), 3.2 (t, J = 7.2 Hz, 1H), 1.8 – 1.6 (m, 2H), 1.4 (dd, J = 7.5, 6.5 Hz, 2H), 0.8 (d, J = 6.6 Hz, 3H), 0.8 (d, J = 6.6 Hz, 3H). **^{13}C NMR** (63 MHz, $CDCl_3$) δ 176.5, 158.7, 132.0, 129.5, 113.7, 59.1, 55.2, 51.6, 42.8, 24.9, 22.8, 22.2. **IR** (ATR, cm^{-1}) 3063, 2997, 2953, 2870, 2836, 1733, 1612, 1511, 1463, 1367, 1300, 1244, 1195, 1170, 1150, 1106, 1035, 991, 824, 777, 577, 513.

Ethyl (4-methoxybenzyl)-*L*-phenylalaninate[4] (**A3**)

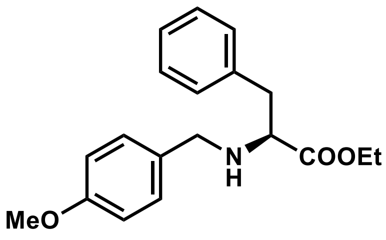

Amine **A3** was synthesized according to general procedure from *p*-methoxybenzaldehyde (1.22 mL, 10 mmol), *L*-phenylalanine ethyl ester hydrochloride (2.76 g, 12 mmol, 1.2 equiv), triethylamine (1.67 mL, 12 mmol, 1.2 equiv) and sodium borohydride (454 mg, 12 mmol, 1.2 equiv) to afford the title compound as a pale yellow oil (2.90 g, 9.27 mmol, 93% yield).

R_f= 0.28 (hexanes:ethyl acetate= 10:1). **LRMS** (EI, 70 eV): m/z (%): 121(100), 222(41), 122(25), 91(23), 77(14), 78(14), 240(12), 65(6), 136(6), 223(6), 92(5). **^{1}H NMR** (250 MHz, Chloroform-*d*) δ 7.25 – 7.08 (m, 7H), 6.78 (d, J = 8.7 Hz, 2H), 4.06 (q, J = 7.1 Hz, 2H), 3.72 (d, J = 12.7 Hz, 1H), 3.71 (s, 3H), 3.55 (d, J = 12.9 Hz, 1H), 3.49 (t, J = 7.0 Hz, 1H), 2.92 (d, J = 7.0 Hz, 2H), 1.89 (s, 1H), 1.11 (t, J = 7.1 Hz, 3H). **^{13}C NMR** (63 MHz, $CDCl_3$) δ 174.4, 158.6, 137.3, 131.6, 129.2, 129.2, 128.2, 126.5, 113.6, 61.8, 60.4, 55.0, 51.3, 39.7, 14.1. **IR** (ATR, cm^{-1}) 3063, 3029, 2979, 2934, 2834, 1727, 1611, 1585, 1510, 1455, 1371, 1300, 1244, 1173, 1129, 1031, 811, 744, 699, 564, 513.

N-(4-methoxybenzyl)adamantan-1-amine[5] (**A4**)

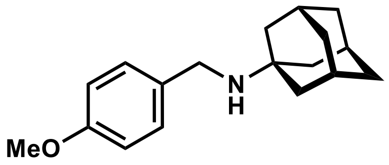

Amine **A3** was synthesized according to general procedure from *p*-methoxybenzaldehyde (1.22 mL, 10 mmol), adamantan-1-amine (900 µL, 12 mmol, 1.2 equiv) and sodium borohydride (454 mg, 12 mmol, 1.2 equiv) to afford the title compound as a colorless oil (1.71 g, 6.29 mmol, 63% yield).

R_f= 0.30 (hexanes:ethyl acetate= 7:3). **M.p.=** 73-76 °C. **LRMS** (EI, 70 eV): m/z (%): 272(8), 271(43), 270(7), 215(12), 214(64), 177(17), 136(5), 135(22), 122(21), 121(100), 107(11), 106(30), 93(11), 91(14), 79(12), 78(12), 77(18), 67(7), 65(5), 55(5). **^{1}H NMR** (250 MHz, $CDCl_3$) δ 7.25 (d, *J* = 8.3 Hz, 2H), 6.85 (d, *J* = 8.7 Hz, 2H), 3.79 (d, *J* = 0.9 Hz, 3H), 3.70 (s, 2H), 2.14 – 2.04 (m, 3H), 1.76 – 1.56 (m, 12H), 1.16 (s, 1H). **^{13}C NMR** (63 MHz, $CDCl_3$) δ 158.5, 133.9, 129.5, 113.9, 77.7, 77.2, 76.7, 55.3, 50.9, 44.6, 43.0, 36.9, 29.8. **IR** (ATR, cm^{-1}) 3292, 2997, 2894, 2846, 1611, 1585, 1510, 1466, 1455, 1367, 1360, 1341, 1300, 1239, 1209, 1177, 1140, 1099, 1075, 1031, 1014, 973, 852, 831, 808, 787, 773, 760, 717, 706, 611, 523.

N-(4-methoxybenzyl)cyclopropanamine[6] (**A5**)

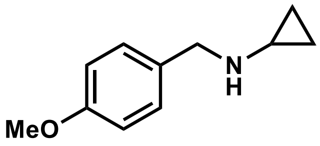

Amine **A5** was synthesized according to general procedure from *p*-methoxybenzaldehyde (1.22 mL, 10 mmol), cyclopropylamine (840 µL, 12 mmol, 1.2 equiv) and sodium borohydride (454 mg, 12 mmol, 1.2 equiv) to afford the title compound as a pale yellow oil (1.41 g, 7.94 mmol, 79% yield).

R_f= 0.39 (hexanes:ethyl acetate= 10:1). **LRMS** (EI, 70 eV): m/z (%): 177(23), 176(14), 162(24), 135(5), 134(6), 122(27), 121(100), 91(15), 89(7), 78(23), 77(22), 65(6), 51(8). **^{1}H NMR** (250 MHz, $CDCl_3$) δ 7.14 (d, *J* = 8.6 Hz, 2H), 6.77 (d, *J* = 8.6 Hz, 2H), 3.68 (s, 2H), 3.67 (s, 3H), 2.05 (tt, *J* = 6.3, 3.8 Hz, 1H), 1.80 (s, 1H), 0.45 – 0.23 (m, 4H). **^{13}C NMR** (63 MHz, $CDCl_3$) δ 158.3, 132.6, 129.1, 113.5, 54.9, 52.9, 29.8, 6.2. **IR** (ATR, cm^{-1}) 3316, 3085, 3005, 2935, 2834, 1655, 1611, 1585, 1510, 1463, 1441, 1371, 1343, 1300, 1242, 1174, 1109, 1034, 1014, 926, 848, 817, 742, 700, 637, 581, 563, 519, 448, 417.

(*R***)**-*N*-(4-methoxybenzyl)-1-phenylethan-1-amine[7] (**A6**)

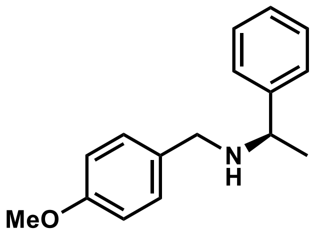

Amine **A6** was synthesized according to general procedure from *p*-methoxybenzaldehyde (1.22 mL, 10 mmol), (R)-(+)-α-methylbenzylamine (1.56 mL, 12 mmol, 1.2 equiv) and sodium borohydride (454 mg, 12 mmol, 1.2 equiv) to afford the title compound as a pale yellow oil (2.26 g, 9.35 mmol, 93% yield).

R_f= 0.31 (hexanes:ethyl acetate= 10:1).

LRMS (EI, 70 eV): m/z (%): 121(100), 226(56), 105(24), 77(22), 136(19), 122(19), 78(14), 91(11), 227(9), 79(9), 104(6), 51(6), 241(6). **^{1}H NMR** (250 MHz, $CDCl_3$) *δ* 7.4 – 7.1 (m, 7H), 6.9 (d, *J* = 8.9 Hz, 2H), 3.9 – 3.7 (m, 4H), 3.7 – 3.5 (m, 2H), 1.6 (s, 1H), 1.4 (d, *J* = 6.6 Hz, 3H). **^{13}C NMR** (63 MHz, $CDCl_3$) *δ* 158.6, 145.7, 132.8, 129.3, 128.5, 126.9, 126.7, 113.8, 57.4, 55.2, 51.0, 24.5. **IR** (ATR, cm^{-1}) 3061, 3026, 2999, 2959, 2931, 2833, 1738, 1611, 1584, 1510, 1492, 1451, 1368, 1300, 1242, 1173, 1113, 1075, 1034, 988, 820, 757, 699, 600, 570, 540, 520.

Ethyl (4-methoxybenzyl)glycinate[8] (**A7**)

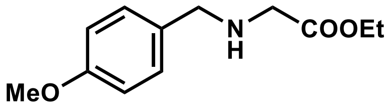

Amine **A7** was synthesized according to general procedure from *p*-methoxybenzaldehyde (2.43 mL, 20 mmol), ethyl glycinate hydrochloride (3.35 g, 24 mmol, 1.2 equiv), triethylamine (3.35 mL, 24 mmol, 1.2 equiv), sodium borohydride (908 mg, 24 mmol, 1.2 equiv) and applied ethanol instead of methanol in the second step to afford the title compound as a pale yellow oil (3.07 g, 13.8 mmol, 69% yield).

R_f= 0.45 (hexanes:ethyl acetate= 7:3). **LRMS** (EI, 70 eV): m/z (%): 223(6), 150(36), 148(11), 137(16), 136(100), 122(50), 121(100), 107(5), 106(8), 91(21), 90(8), 89(12), 79(6), 78(40), 77(30), 75(7), 65(7), 63(6), 52(8), 51(9). **^{1}H NMR** (250 MHz, $CDCl_3$) *δ* 7.20 (d, *J* = 8.5 Hz, 2H), 6.82 (d, *J* = 8.5 Hz, 2H), 4.14 (q, *J* = 7.1 Hz, 2H), 3.74 (s, 3H), 3.69 (s, 2H), 3.34 (s, 2H), 2.18 (s, 1H), 1.22 (t, *J* = 7.1 Hz, 3H). **^{13}C NMR** (63 MHz, $CDCl_3$) *δ* 158.8, 131.5, 129.4, 113.8, 60.6, 55.1, 52.6, 49.9, 14.1. **IR** (ATR, cm^{-1}) 2971, 2936, 2910, 2836, 1734, 1611, 1511, 1462, 1419, 1373, 1300, 1242, 1177, 1137, 1109, 1028, 812, 760, 571, 518.

N-(4-methoxybenzyl)hexan-1-amine[9] (**A8**)

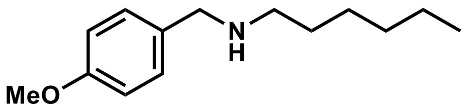

Amine **A8** was synthesized according to general procedure from *p*-methoxybenzaldehyde (1.22 mL, 10 mmol), *n*-hexylamine (1.59 mL, 12 mmol, 1.2 equiv) and sodium

borohydride (454 mg, 12 mmol, 1.2 equiv) to afford the title compound as a pale yellow oil (1.40 g, 6.33 mmol, 63% yield).

R$_f$= 0.30 (hexanes:ethyl acetate= 10:1). **LRMS** (EI, 70 eV): m/z (%): 221(1), 150(9), 136(4), 122(9), 121(100), 91(2), 78(3), 77(3). **^{1}H NMR** (250 MHz, $CDCl_3$) δ 7.14 (d, J = 8.5 Hz, 2H), 6.76 (d, J = 8.6 Hz, 2H), 3.69 (s, 3H), 3.63 (s, 2H), 2.52 (t, J = 7.2 Hz, 2H), 1.60 (s, 1H), 1.40 (q, J = 7.0, 6.4 Hz, 2H), 1.29 – 1.07 (m, 6H), 0.79 (t, J = 6.4 Hz, 3H). **^{13}C NMR** (63 MHz, $CDCl_3$) δ 158.6, 132.7, 129.3, 113.8, 55.2, 53.5, 49.4, 31.8, 30.1, 27.1, 22.7, 14.1. **IR** (ATR, cm^{-1}) 3064, 3029, 2999, 2953, 2925, 2854, 2834, 1738, 1611, 1585, 1510, 1456, 1377, 1300, 1244, 1173, 1105, 1036, 818, 751, 726, 699, 637, 574, 513, 418, 397.

N-(4-methoxybenzyl)heptan-2-amine (**A9**)

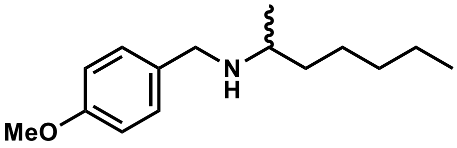

Amine **A9** was synthesized according to general procedure from *p*-methoxybenzaldehyde (1.22 mL, 10 mmol), 2-aminoheptane (1.81 mL, 12 mmol, 1.2 equiv) and sodium borohydride (454 mg, 12 mmol, 1.2 equiv) to afford the title compound as a pale yellow oil (2.34 g, 9.93 mmol, 99% yield).

R$_f$= 0.36 (hexanes:ethyl acetate= 10:1). **LRMS** (EI, 70 eV): m/z (%): 204(1), 165(2), 164(18), 164(3), 162(2), 136(3), 135(2), 134(1), 122(11), 121(100), 91(3), 90(1), 89(1), 82(1), 78(4), 77(4), 57(1). **^{1}H NMR** (250 MHz, $CDCl_3$) δ 7.16 (d, J = 8.5 Hz, 2H), 6.77 (d, J = 8.6 Hz, 2H), 3.70 (s, 3H), 3.61 (q, J = 12.8 Hz, 2H), 2.56 (dq, J = 12.2, 7.4, 6.5 Hz, 1H), 1.39 (s, 2H), 1.33 – 1.10 (m, 7H), 0.99 (d, J = 6.3 Hz, 3H), 0.81 (t, J = 6.6 Hz, 3H). **^{13}C NMR** (63 MHz, $CDCl_3$) δ 158.6, 133.0, 129.3, 113.8, 55.2, 52.5, 50.8, 37.1, 32.1, 25.7, 22.7, 20.3, 14.1. **IR** (ATR, cm^{-1}) 3063, 2996, 2955, 2925, 2856, 1738, 1612, 1585, 1510, 1463, 1374, 1300, 1244, 1171, 1106, 1036, 822, 808, 750, 727, 696, 574, 515.

N-(4-methoxybenzyl)octan-1-amine[10] (**A10**)

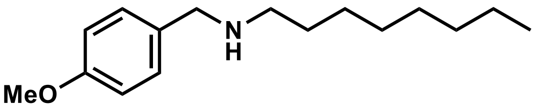

Amine **A10** was synthesized according to general procedure from *p*-methoxybenzaldehyde (1.22 mL, 10 mmol), 2-aminoheptane (1.81 mL, 12 mmol, 1.2 equiv) and sodium borohydride (454 mg, 12 mmol, 1.2 equiv) to afford the title compound as a pale yellow oil (2.34 g, 9.93 mmol, 99% yield).

R$_f$= 0.39 (hexanes:ethyl acetate= 10:1). **LRMS** (EI, 70 eV): m/z (%): 249(3), 248(4), 150(33), 137(11), 136(12), 128(7), 122(33), 121(100), 106(3), 91(7), 78(9), 77(9), 75(3). **^{1}H NMR** (250 MHz, $CDCl_3$) δ 7.14 (d, J = 8.5 Hz, 2H), 6.76 (d, J = 8.5 Hz, 2H), 3.68 (s, 3H), 3.62 (s, 2H), 2.51 (t, J = 7.2 Hz, 2H), 1.67 (s, 1H), 1.49 – 1.32 (m, 2H), 1.18 (s, 10H), 0.79 (t, J = 6.1 Hz, 3H). **^{13}C NMR** (63 MHz, $CDCl_3$) δ 158.6, 132.6, 129.3, 113.7, 55.2, 53.5, 49.4, 31.9, 30.1, 29.6, 29.3, 27.4, 22.7, 14.1. **IR** (ATR, cm^{-1}) 3063, 2953, 2922, 2853, 1738, 1612, 1585, 1510, 1456, 1365, 1300, 1244, 1173, 1106, 1036, 820, 754, 723, 700, 573, 515.

N-(4-methoxybenzyl)aniline[11] (**A11**)

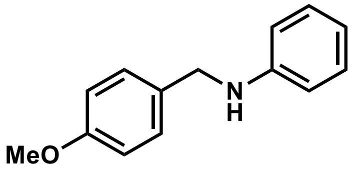

Amine **A11** was synthesized according to general procedure from *p*-methoxybenzaldehyde (1.22 mL, 10 mmol), aniline (1.09 mL, 12 mmol, 1.2 equiv) and sodium borohydride (454 mg, 12 mmol, 1.2 equiv) to afford the title compound as an off-white solid (1.34 g, 6.29 mmol, 63% yield).

R$_f$= 0.31 (hexanes:ethyl acetate= 10:1). **M. p.=** 54-58 °C. **LRMS** (EI, 70 eV): m/z (%): 212(12), 211(89), 210(100), 196(4), 195(5), 168(4), 167(13), 166(2), 139(2), 104(3), 92(2), 91(2), 78(4), 77(31), 76(3), 65(5), 64(2), 63(3), 51(10), 50(3). **^{1}H NMR** (250 MHz, $CDCl_3$) δ 7.29 (d, J = 8.5 Hz, 2H), 7.19 (t, J = 7.9 Hz, 2H), 6.89 (d, J = 8.6 Hz, 2H), 6.74 (t, J = 7.3 Hz, 1H), 6.64 (d, J = 7.7 Hz, 2H), 4.24 (s, 2H), 3.93 (s, 1H), 3.79 (s, 3H). **^{13}C NMR** (63 MHz, $CDCl_3$) δ 158.9, 148.2, 131.4, 129.3, 128.9, 117.6, 114.1, 113.0, 55.3, 47.8. **IR** (ATR, cm^{-1}) 3075, 3047, 3020, 3002, 2961, 2934, 2907, 2897, 2856, 2836, 1599, 1582, 1511, 1499, 1470, 1460, 1438, 1424, 1300, 1246, 1171, 1153, 1110, 1095, 1068, 1032, 872, 835, 818, 746, 692, 544, 523, 513.

N-(4-methoxybenzyl)-3-nitroaniline[12] (**A12**)

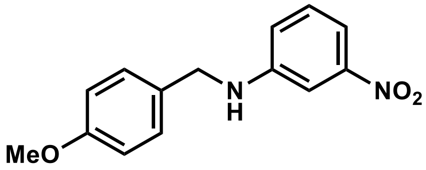

Amine **A12** was synthesized according to general procedure from *p*-methoxybenzaldehyde (1.22 mL, 10 mmol), 3-nitroaniline (1.09 mL, 12 mmol, 1.2 equiv) and sodium borohydride (454 mg, 12 mmol, 1.2 equiv) to afford the title compound as an orange solid (1.34 g, 6.29 mmol, 63% yield).

R$_f$= 0.59 (hexanes:ethyl acetate= 7:3). **M. p.=** 98-102 °C. **LRMS** (EI, 70 eV): m/z (%): 257(15), 256(100), 255(50), 210(7), 209(21), 195(10), 168(6), 167(20), 166(11), 140(7), 139(9), 77(9), 76(10), 51(7). **^{1}H NMR** (250 MHz, $CDCl_3$) δ 7.51 – 7.39 (m, 1H), 7.35 (t, J = 2.1 Hz, 1H), 7.27 – 7.11 (m, 3H), 6.83 (dd, J = 6.4, 2.1 Hz, 3H), 4.44 (d, J = 26.7 Hz, 1H), 4.22 (s, 2H), 3.73 (s, 3H).

^{13}C NMR (63 MHz, $CDCl_3$) δ 159.2, 149.4, 148.8, 130.0, 129.8, 128.9, 118.9, 114.3, 112.1, 106.6, 55.3, 47.6. **IR** (ATR, cm^{-1}) 3406, 3098, 3075, 3033, 3012, 2952, 2928, 2900, 2853, 2836, 1611, 1582, 1536, 1509, 1341, 1275, 1241, 1170, 1097, 1031, 844, 812, 787, 733, 669, 506, 468.

3-phenylprop-2-yn-1-ol[13]

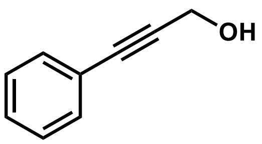

A 250 mL round bottom flask was charged with stirring bar, $Pd(PPh_3)_2Cl_2$ (140 mg, 0.2 mmol, 1 mol%) and CuI (76.2 mg, 0.04 mmol, 2 mol%), then the sealed flask was purged with argon for 5 minutes. Triethylamine (80 mL, purged with argon) followed by iodobenzene (2.28 mL, 20 mmol) was added under argon atmosphere and stirred for 5 minutes at room temperature. Propargyl alcohol (1.31 mL, 22 mmol, 1.1 equiv) was added dropwise over 5 minutes, then the mixture was stirred for 16 hours at room temperature. Reaction mixture was filtered through a pad of Celite, solids were washed with EtOAc and the combined filtrates were concentrated *in vacuo*. Solid residue was purified by flash column chromatography (eluent: hexanes:ethyl acetate= 7:3) to afford the title compound as a colorless liquid (2.64 g, 20 mmol, 99% yield).

R_f= 0.41 (hexanes:ethyl acetate= 7:3). **LRMS** (EI, 70 eV): m/z (%): 132(58), 131(100), 115(25), 104(28), 103(53), 102(14), 89(7), 78(28), 77(38), 76(7), 75(9), 74(10), 63(13), 62(7), 51(17). **^{1}H NMR** (250 MHz, $CDCl_3$) δ 7.38 (dd, J = 6.8, 2.8 Hz, 2H), 7.23 (dd, J = 5.1, 1.8 Hz, 3H), 4.44 (s, 2H), 2.77 (s, 1H). **^{13}C NMR** (63 MHz, $CDCl_3$) δ 131.7, 128.5, 128.3, 122.6, 87.4, 85.6, 51.4.

3-Phenylprop-2-yn-1-yl 4-methylbenzenesulfonate[14]

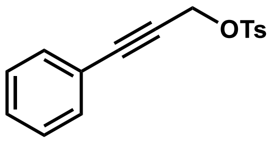

A 50 mL round bottom flask was charged with stirring bar, 3-phenylprop-2-yn-1-ol (2.78 g, 21 mmol), tosyl chloride (4.80 g, 25.2 mmol, 1.2 equiv) and diethyl ether (33 mL), then cooled to 0 °C. Pulverized potassium hydroxide (6.6 g, 118 mmol, 5.6 equiv) was added in one portion to the vigorously stirred mixture and stirring was continued for 1 hour at the same temperature. Reaction mixture was quenched by pouring cold water, extracted with diethyl ether. Combined organic layers were washed with brine, dried over Na_2SO_4 and filtered. The filtrate was concentrated *in vacuo* and the residue was purified bz flash column chromatography (eluent: hexanes:ethyl acetate= 7:3). The title compound was obtained as a white solid (2.67 g, 9.32 mmol, 44% yield).

R_f= 0.55 (hexanes:ethyl acetate= 7:3). **LRMS** (EI, 70 eV): m/z (%): 192(7), 139(41), 131(41), 116(7), 115(100), 114(55), 105(14), 103(10), 103(8), 102(7), 92(11), 91(12), 91(12), 89(15), 77(8), 77(7), 65(18). **^{1}H NMR** (250 MHz, $CDCl_3$) δ 7.77 (d, J = 8.4 Hz, 2H), 7.31 – 7.09 (m, 7H), 4.87

(s, 2H), 2.30 (s, 3H). ^{13}**C NMR** (63 MHz, $CDCl_3$) δ 145.1, 133.5, 131.8, 129.9, 129.1, 128.3, 128.2, 121.5, 89.0, 80.7, 58.7, 21.6.

General procedure for the synthesis of propargyl amines

A 20 mL vial was charged with stirring bar, appropriate amine (12 mmol, 4 equiv), potassium carbonate (419 mg, 3 mmol, 1 equiv) and acetonitrile (9 mL). 3-Phenylprop-2-yn-1-yl 4-methylbenzenesulfonate (859 mg, 3 mmol) was added in one portion, then the mixture was heated to 70°C and stirred for 16 hours. Reaction mixture was concentrated onto Celite and the residue was purified by flash column chromatogarphy (hexanex:EtOAc = 100:0 -> 50:50).

N-isobutyl-3-phenylprop-2-yn-1-amine[15] (**A13**)

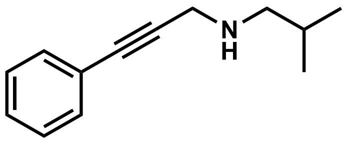

Amine **A13** was synthesized according to general procedure from isobutylamine (1.19 mL, 12 mmol, 4 equiv), potassium carbonate (419 mg, 3 mmol, 1 equiv) and 3-phenylprop-2-yn-1-yl 4-methylbenzenesulfonate (859 mg, 3 mmol) to afford the title compound as a brown liquid (425 mg, 2.27 mmol, 76% yield).

R$_f$= 0.52 (hexanes:ethyl acetate= 7:3). **LRMS** (EI, 70 eV): m/z (%): 115(100), 144(78), 116(15), 89(10), 145(9), 63(6), 65(4), 114(4), 130(4), 187(4), 77(3). ^{1}**H NMR** (250 MHz, Methylene Chloride-*d*$_2$) δ 7.3 – 7.2 (m, 2H), 7.2 – 7.1 (m, 3H), 3.4 (s, 2H), 2.4 (d, *J* = 6.7 Hz, 2H), 1.6 (dp, *J* = 13.3, 6.7 Hz, 1H), 1.2 (s, 1H), 0.8 (s, 3H), 0.8 (s, 3H). ^{13}**C NMR** (63 MHz, Methylene Chloride-*d*$_2$) δ 133.9, 130.6, 130.2, 125.9, 90.8, 85.2, 59.4, 41.5, 30.8, 22.9. **IR** (ATR, cm^{-1}) 3057, 3023, 2953, 2870, 2815, 1792, 1738, 1598, 1489, 1466, 1442, 1365, 1330, 1252, 1229, 1217, 1112, 1069, 1028, 950, 913, 753, 689, 525.

N-(3-phenylprop-2-yn-1-yl)cyclohexanamine[16] (**A14**)

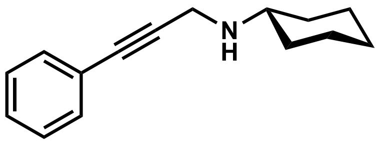

Amine **A14** was synthesized according to general procedure from cyclohexylamine (1.37 mL, 12 mmol, 4 equiv), potassium carbonate (419 mg, 3 mmol, 1 equiv) and 3-phenylprop-2-yn-1-yl 4-methylbenzenesulfonate (859 mg, 3 mmol) to afford the title compound as a brown liquid (269 mg, 1.26 mmol, 42% yield).

R$_f$= 0.24 (hexanes:ethyl acetate= 7:3). **LRMS** (EI, 70 eV): m/z (%): 115(100), 170(34), 156(33), 157(23), 130(13), 80(12), 116(11), 89(10), 213(9), 55(8), 128(6), 212(5), 85(5). ^{1}**H NMR** (250 MHz, Methylene Chloride-*d*$_2$) δ 7.4 (dq, *J* = 8.3, 3.4, 2.6 Hz, 2H), 7.4 – 7.3 (m, 3H), 3.7 (s, 2H), 2.7 (tt, *J* = 10.0, 3.8 Hz, 1H), 2.0 – 1.6 (m, 5H), 1.5 – 1.0 (m, 6H). ^{13}**C NMR** (63 MHz, Methylene

Chloride-d_2) δ 133.8, 130.6, 130.2, 125.9, 91.0, 84.9, 57.6, 38.3, 35.5, 28.6, 27.2. **IR** (ATR, cm^{-1}) 3080, 3057, 3020, 2924, 2851, 1738, 1489, 1443, 1368, 1326, 1253, 1229, 1217, 1119, 1069, 1028, 951, 913, 889, 753, 689, 596, 540, 525, 509, 459, 424.

N-hexyl-1-(phenylethynyl)cyclohexan-1-amine[17] (**A15**)

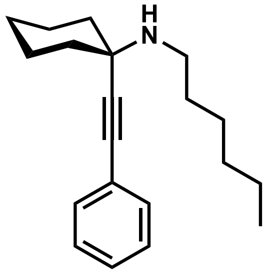

A 10 mL microwave instrument vial was charged with stirring bar, phenylacetylene (336 uL, 3 mmol, 1 equiv), cyclohexanone (311 uL, 3 mmol, 1 equiv), hexylamine (396 uL, 3 mmol, 1 equiv) and copper(I) iodide (114 mg, 0.6 mmol, 20 mol%), then sealed with Teflon septa. Vial was evacuated and backfilled with argon (repeated three times), then placed in the microwave cavity and irradiated at ceiling temperature 100°C for 25 minutes. After completion of reaction, the mixture was cooled to room temperature, diluted with methanol and concentrated onto Celite at reduced pressure. Residue was purified by flash column chromatography (eluent: hexanes:ethyl acetate= 7:3) to afford the title compound as a brown liquid (531 mg, 1.87 mmol, 62% yield).

R_f= 0.55 (hexanes:ethyl acetate= 7:3). **LRMS** (EI, 70 eV): m/z (%): 240(100), 268(26), 212(23), 241(19), 115(17), 283(14), 156(14), 128(14), 141(14), 127(12), 183(9), 198(8), 155(8), 254(8), 282(8). **^{1}H NMR** (250 MHz, $CDCl_3$) δ 7.42 – 7.30 (m, 2H), 7.26 – 7.15 (m, 3H), 2.73 (t, J = 6.8 Hz, 2H), 1.87 (d, J = 11.9 Hz, 2H), 1.70 – 1.51 (m, 5H), 1.52 – 0.92 (m, 12H), 0.92 – 0.70 (m, 3H). **^{13}C NMR** (63 MHz, $CDCl_3$) δ 131.6, 128.2, 127.7, 123.8, 93.6, 84.5, 55.1, 43.3, 38.3, 31.8, 30.7, 27.2, 26.0, 23.1, 22.7, 14.1. **IR** (ATR, cm^{-1}) 3080, 3056, 3020, 2927, 2854, 1738, 1489, 1443, 1375, 1365, 1290, 1265, 1228, 1217, 1158, 1120, 1069, 951, 909, 753, 689, 658, 637, 559, 536, 516, 491.

N-(4-methoxybenzyl)prop-2-yn-1-amine[18] (**A16**)

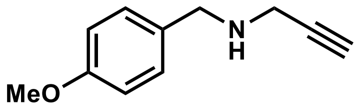

A 250 mL round bottom flask was charged with stirring bar, *p*-methoxybenzaldehyde (1.84 mL, 15.1 mmol) and methanol (100 mL), followed by propargylamine (1.16 mL, 18.2 mmol, 1.2 equiv). The mixture was stirred for 4 hours, then cooled to 0°C and sodium borohydride (1.00 g, 26.5 mmol, 1.75 equiv) was added portionwise. Reaction mixture was allowed to warm room temperature over 16 hours, then concentrated onto Celite under reduced pressure. The obtained residue was purified by flash column chromatography (eluent: hexanes:ethyl acetate= 7:3) to afford the title compound as a colorless liquid (2.01 g, 11.5 mmol, 76% yield).

R$_f$= 0.41 (hexanes:ethyl acetate= 7:3). **LRMS** (EI, 70 eV): m/z (%):121(100), 174(68), 146(45), 134(26), 122(24), 77(22), 78(21), 144(15), 135(14), 91(14), 175(12), 68(12). **^{1}H NMR** (250 MHz, $CDCl_3$) δ 7.18 (d, J = 8.4 Hz, 2H), 6.78 (d, J = 8.5 Hz, 2H), 3.73 (s, 2H), 3.70 (s, 3H), 3.32 (d, J = 2.3 Hz, 2H), 2.19 (t, J = 2.0 Hz, 1H), 1.43 (s, 1H).**^{13}C NMR** (63 MHz, $CDCl_3$) δ 158.8, 131.5, 129.6, 113.8, 82.2, 71.5, 55.2, 51.6, 37.2. **IR** (ATR, cm^{-1}) 3288, 3030, 3002, 2934, 2912, 2834, 1738, 1611, 1585, 1510, 1455, 1364, 1300, 1241, 1174, 1102, 1032, 811, 776, 759, 702, 635, 580, 560, 515.

tert-butyl 3-(phenethylamino)azetidine-1-carboxylate (**A17**)

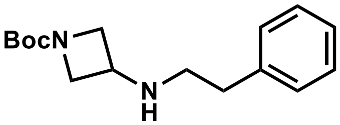

A 100 mL round bottom flask was charged with stirring bar, *tert*-butyl 3-oxoazetidine-1-carboxylate (873 mg, 5.0 mmol) and dichloromethane (50 mL). To the stirred mixture, acetic acid (28.6 µL, 0.5 mmol, 10 mol%), followed by phenylethylamine (882 µL, 7.0 mmol, 1.4 equiv) was added at room temperature and stirred for 3 hours. Then the reaction mixture was cooled to 0°C and sodium triacetoxyborohydride (4.45 g, 21 mmol, 4.2 equiv) was added portionwise. The mixture was allowed to warm room temperature over 16 hours, then volatiles were evaporated under reduced pressure. The obtained residue was purified by flash column choramtography (eluent: hexanes:ethyl acetate= 7:3) to afford the title compound as a yellow syrup (539 mg, 1.95 mmol, 39% yield).

R$_f$= 0.31 (hexanes:ethyl acetate= 7:3). **LRMS** (EI, 70 eV): m/z (%): 203(3), 148(3), 147(28), 129(2), 129(2), 105(12), 104(23), 103(2), 91(4), 79(2), 77(3), 57(18), 56(100), 55(4). **^{1}H NMR** (250 MHz, Methanol-d_4) δ 7.23 (ddt, J = 14.8, 10.4, 7.0 Hz, 5H), 4.04 (dd, J = 8.8, 7.0 Hz, 2H), 3.78 – 3.47 (m, 3H), 2.74 (d, J = 2.9 Hz, 4H), 1.43 (s, 9H). **^{13}C NMR** (63 MHz, Methanol-d_4) δ 158.1, 140.9, 129.6, 127.3, 80.8, 49.5, 48.6, 37.0, 28.7. **IR** (ATR, cm^{-1}) 3307, 3061, 3026, 2973, 2877, 2863, 1692, 1604, 1476, 1455, 1399, 1364, 1292, 1252, 1156, 1126, 1109, 1029, 936, 861, 771, 747, 699, 564, 499, 464. **HRMS** m/z $[M+H]^+$ calculated for $C_{16}H_{25}N_2O_2^+$: 277.1916, found: 277.1910.

3-((*tert*-butyldimethylsilyl)oxy)azetidine (**A18**)

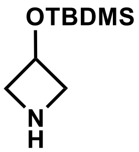

A 40 mL vial was charged with stirring bar and 3-hydroxyazetidin-1-ium chloride (657 mg, 6.0 mmol), vial was sealed with Teflon septa and screw cap, then evacuated and backfilled with argon (three cycles). Solution of imidazole (1.23 g, 18 mmol, 3 equiv) and dimethylformamide (23 µL, 0.3 mmol, 5 mol%) in dicholormethane (10

mL) was added under argon atmosphere and stirred at room temperature. Solution of *tert*-butyldimethylsilyl chloride (1.03 g, 6.6 mmol, 1.1 equiv) in dichloromethane (10 mL) was added dropwise under argon atmosphere. Reaction mixture was stirred for 3 hours, then concentrated under reduced pressure. Residue was suspended in diisopropyl ether and filtered through a pad of Celite. Solids were washed with diisopropyl ether two times and combined filtrates concentrated under reduced pressure to afford the title compound as a colorless (689 mg, 3.68 mmol, 61% yield).

R$_f$= 0.45 (hexanes:ethyl acetate= 7:3). **LRMS** (EI, 70 eV): m/z (%): 186(1), 174(5), 173(20), 172(100), 98(16), 97(6), 96(28), 89(17), 84(10), 82(15), 75(17), 74(4), 73(13), 70(7), 59(11), 57(4). **^{1}H NMR** (250 MHz, Chloroform-*d*) δ 4.45 (s, 1H), 3.89 – 2.80 (m, 4H), 2.53 (bs, 1H), 0.83 (s, 9H), -0.01 (s, 6H). **^{13}C NMR** (63 MHz, Chloroform-*d*) δ 71.4, 58.1, 25.9, 18.1, -4.9. **IR** (ATR, cm^{-1}) 2952, 2929, 2856, 1738, 1472, 1463, 1371, 1252, 1229, 1217, 1202, 1158, 1068, 1005, 879, 834, 774, 736, 671, 398.

3-(methoxycarbonyl)azetidin-1-ium trifluoromethanesulfonate (**A19**)

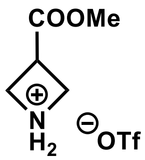

A 30 mL vial was charged with stirring bar, 1-(tert-butoxycarbonyl)azetidine-3-carboxylic acid (402 mg, 2.0 mmol), methanol (10 mL) and trifluoromethanesulfonic acid (186 µL, 2.1 mmol, 1.05 equiv) then vial was sealed with Teflon septa and screw cap. Reaction mixture was stirred for 16 hours at room temperature, then volatiles were evaporated under reduced pressure. Residues were dissolved in dry diethylether and volatiles were evaporated under reduced pressure (repeated three times), then the residue was dried under high vacuum. Title compound was obtained as a hygroscopic, colorless solid (522 mg, 1.97 mmol, 98% yield).

R$_f$= 0.36 (toluene:ethyl acetate:acetic acid= 7:2:1). **LRMS** (EI, 70 eV): m/z (%): 57(100), 55(20), 56(20), 87(18), 142(13), 160(9), 55(5), 58(5), 59(4), 115(4). **^{1}H NMR** (250 MHz, Acetonitrile-*d*$_3$) δ 7.32 (bs, 2H), 4.34 – 4.18 (m, 1H), 3.70 (dd, J = 16.6, 8.7 Hz, 1H), 3.70 (s, 3H). **^{19}F NMR** (235 MHz, Acetonitrile-*d*$_3$) δ -79.28. **^{13}C NMR** (63 MHz, Acetonitrile-*d*$_3$) δ 172.1, 121.5 (q, J = 318.7 Hz), 53.3, 50.0, 35.4. **IR** (ATR, cm^{-1}) 3238, 3085, 3046, 2969, 2895, 1736, 1700, 1441, 1384, 1365, 1320, 1265, 1242, 1226, 1207, 1158, 1080, 1065, 1029, 922, 878, 852, 838, 773, 753, 734, 637, 573, 513.

General procedure for the synthesis of piperidine derivatives

A 40 mL vial was charged with stirring bar, appropiate alcohol (10.0 mmol), imidazole (1.02 g, 15.0 mmol), dimethylformamide (77 µL, 1 mmol, 10 mol%) and dichloromethane (20 mL). To the

stirred reaction mixture, *tert*-butyldimethylsilyl chloride (1.71 g, 11 mmol, 1.1 equiv) was added at room temperature and stirring was continued for 16 hours. Reaction mixture was diluted with diethylether (120 mL) and washed with water (3× 50 mL) and brine (1× 20 mL). Organic phase was separated, dried over anh. $MgSO_4$, filtered and the filtrate was concentrated in vacuo.

3-((*tert*-butyldimethylsilyl)oxy)piperidine (**A20**)

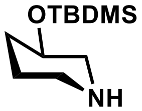

Compound **A20** was prepared according to general procedure from piperidine-3-ol (1.01 g, 10 mmol) to afford a pale yellow oil (1.99 g, 9.26 mmol, 93% yield).

R_f= 0.52 (hexanes:ethyl acetate= 7:3). **LRMS** (EI, 70 eV): m/z (%): 158(100), 84(44), 82(32), 75(26), 73(21), 159(14), 59(12), 56(11), 57(9), 55(9), 156(8), 101(7), 58(4), 200(3). **^{1}H NMR** (250 MHz, $CDCl_3$) δ 3.52 (dt, J = 7.3, 3.7 Hz, 1H), 2.83 (d, J = 12.2 Hz, 1H), 2.75 – 2.62 (m, 1H), 2.60 – 2.34 (m, 2H), 2.20 (bs, 1H), 1.81 – 1.54 (m, 2H), 1.33 (tt, J = 12.0, 7.5 Hz, 2H), 0.88 – 0.68 (m, 9H), 0.01 – -0.13 (m, 6H). **^{13}C NMR** (63 MHz, $CDCl_3$) δ 67.9, 54.0, 46.1, 33.8, 25.8, 24.4, 18.1, -4.7, -4.8. **IR** (ATR, cm^{-1}) 2929, 2911, 2894, 2856, 2809, 1738, 1472, 1462, 1439, 1363, 1251, 1229, 1217, 1149, 1093, 1066, 1046, 1029, 1005, 879, 851, 832, 804, 771, 668.

3-(((*tert*-butyldimethylsilyl)oxy)methyl)piperidine (**A21**)

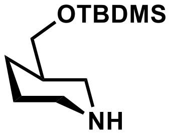

Compound **A21** was prepared according to general procedure from piperidin-3-ylmethanol (1.15 g, 10 mmol) to afford a pale yellow oil (1.47 g, 6.4 mmol, 64% yield).

R_f= 0.23 (hexanes:ethyl acetate= 10:1). **LRMS** (EI, 70 eV): m/z (%): 172(100), 96(36), 75(26), 89(24), 73(22), 82(22), 59(21), 98(19), 173(19), 84(15), 70(12), 57(8), 214(6), 229(3). **^{1}H NMR** (250 MHz, $CDCl_3$) δ 3.47 – 3.21 (m, 2H), 3.06 (d, J = 11.5 Hz, 1H), 2.93 (d, J = 12.1 Hz, 1H), 2.46 (td, J = 11.8, 2.7 Hz, 1H), 2.34 – 2.13 (m, 1H), 1.91 (s, 1H), 1.76 – 1.48 (m, 3H), 1.48 – 1.24 (m, 1H), 1.01 (qd, J = 11.9, 3.9 Hz, 1H), 0.82 (s, 9H), -0.03 (d, J = 3.7 Hz, 6H). **^{13}C NMR** (63 MHz, $CDCl_3$) δ 66.6, 50.2, 47.2, 40.0, 27.8, 26.3, 26.0, 18.3, -5.4, -5.4. **IR** (ATR, cm^{-1}) 2928, 2854, 2802, 2734, 1738, 1470, 1442, 1385, 1361, 1252, 1217, 1078, 1005, 939, 832, 773, 665, 567.

Synthesis of compound ***6***

1-(naphthalen-1-ylmethyl)-2-(trifluoromethyl)aziridine

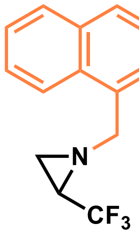

To a stirred suspension of sodium carbonate (63.6 mg, 0.60 mmol), 1-naphthylmethylamine (64.2 mg, 0.4 mmol) and dichloromethane (4 ml) 4-fluorophenyl)(3,3,3-trifluoroprop-1-en-2-yl)iodonium trifluoromethanesulfonate (196 mg, 0.42 mmol) was added in one portion. Reaction was monitorized by TLC and after completion the mixture was concentrated onto Celite and purified by flash chromatogarpy (eluent: hexanes:ethyl acetate= 10:1). Title compound was obtained as a colorless syrup (91.6 mg, 0.365 mmol, 91% yield).

R_f= 0.33 (hexanes:ethyl acetate= 10:1). **LRMS** (EI, 70 eV): m/z (%): 141(100), 115(27), 251(17), 142(14), 139(10), 127(6), 128(5), 154(3), 126(3), 63(3), 140(3), 60(3), 252(3), 116(3). **^{1}H NMR** 1H NMR (250 MHz, Acetonitrile-d3) δ 8.29 – 8.07 (m, 1H), 8.03 – 7.76 (m, 2H), 7.67 – 7.32 (m, 4H), 3.97 (s, 2H), 2.49 (pd, J = 5.6, 3.0 Hz, 1H), 2.12 (d, J = 3.1 Hz, 1H), 1.87 (d, J = 6.4 Hz, 1H). **^{19}F NMR** (235 MHz, Acetonitrile-*d*3) δ -71.67. **^{13}C NMR** (63 MHz, Acetonitrile-*d*3) δ 135.1, 134.6, 132.5, 129.4, 129.0, 127.0, 127.0, 126.8, 126.4, 125.7 (d, *J* = 271.3 Hz), 125.0, 37.9 (q, *J* = 39.0 Hz), 30.8 (q, *J* = 1.9 Hz). **IR** (ATR, cm^{-1}) 3041, 2988, 2971, 2901, 1738, 1598, 1510, 1475, 1429, 1283, 1232, 1131, 1072, 1058, 1028, 957, 865, 835, 793, 771, 734, 655, 537, 408.

Optimization of reaction conditions of homodiamination

A 4 mL screwed cap vial was charged with stirring bar, sodium carbonate (0-3.0 equiv), solvent (2 mL) and *N*-methyl-1-(naphthalen-1-yl)methanamine (1.0-3.0 equiv). To the vigorously stirred mixture, (4-fluorophenyl)(3,3,3-trifluoroprop-1-en-2-yl)iodonium trifluoromethanesulfonate (93.2 mg, 0.20 mmol) was added portionwise within one minute. Reaction mixture was stirred for the indicated time, diluted with the corresponding solvent and concentrated onto Celite under reduced pressure. Residue was purified by flash column chromatography.

Supplementary Table 1: Optimization of reaction conditions of homodiamination

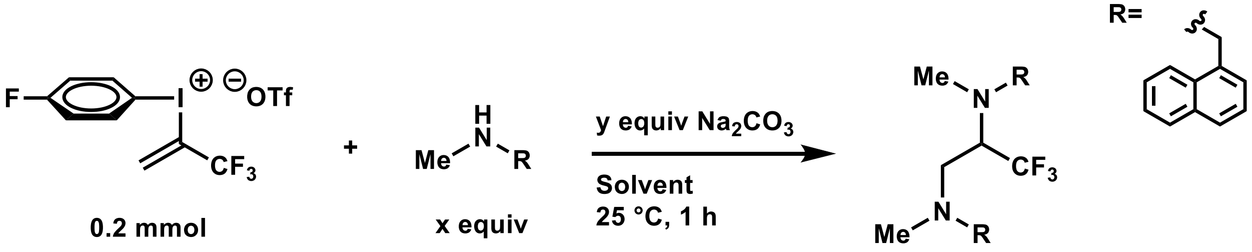

#	MeNHR [equiv]	Na_2CO_3 [equiv]	Solvent	Isolated yield [%]
1	1.0	0	CH_2Cl_2	12
2	2.0	0	CH_2Cl_2	15
3	3.0	0	CH_2Cl_2	81
4	2.5	1.5	MeCN	90
5	2.0	2.0	MeCN	84
6	2.0	2.0	CH_2Cl_2	82
7	3.0	2.0	CH_2Cl_2	89
8	2.0	2.0	PhMe	62
9	3.0	2.0	MeCN	82
10	2.0	3.0	MeCN	83
11	2.5	3.0	MeCN	92

Reaction monitoring by ^{19}F-NMR

A 5 mm O.D. NMR tube was charged with (4-fluorophenyl)(3,3,3-trifluoroprop-1-en-2-yl)iodonium trifluoromethanesulfonate (23.3 mg, 0.05 mmol) acetonitrile-d_3 (500 μL) and sealed with a hollow cap. An LDPE tube was filled with five layers of substances in a following sequence: acetonitrile (100 μL), air (100 μL), appropriate secondary amine (0.20 mmol, 4 equiv), air (100 μL) and acetonitrile (100 μL), then was placed in the NMR tube. These substances were added in one portion to the reaction mixture at *t*= 0 second. Recording of ^{19}F-NMR spectra (proton decoupled, $No._{dummy\ scan}$= 0, $No._{scan}$= 1) was started at *t*= −60 seconds and 15 seconds time intervals were applied between experiments.

Supplementary Figure 1: Reaction scheme and monitoring of homodiamination reaction with of *N*-methyl-1-(naphthalen-1-yl)methanamine

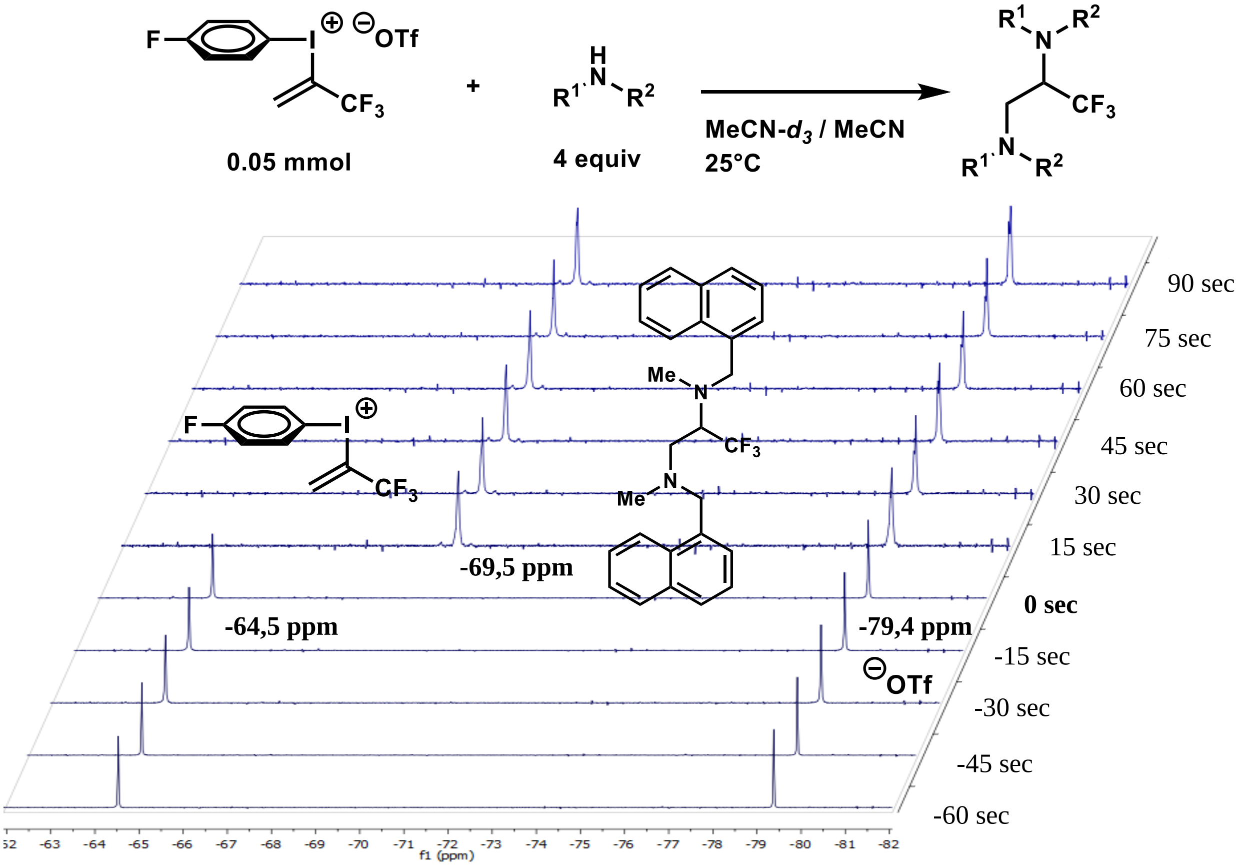

Supplementary Figure 2: Reaction monitoring of homodiamination reaction with *N*-methylaniline by ^{19}F-NMR-spectroscopy

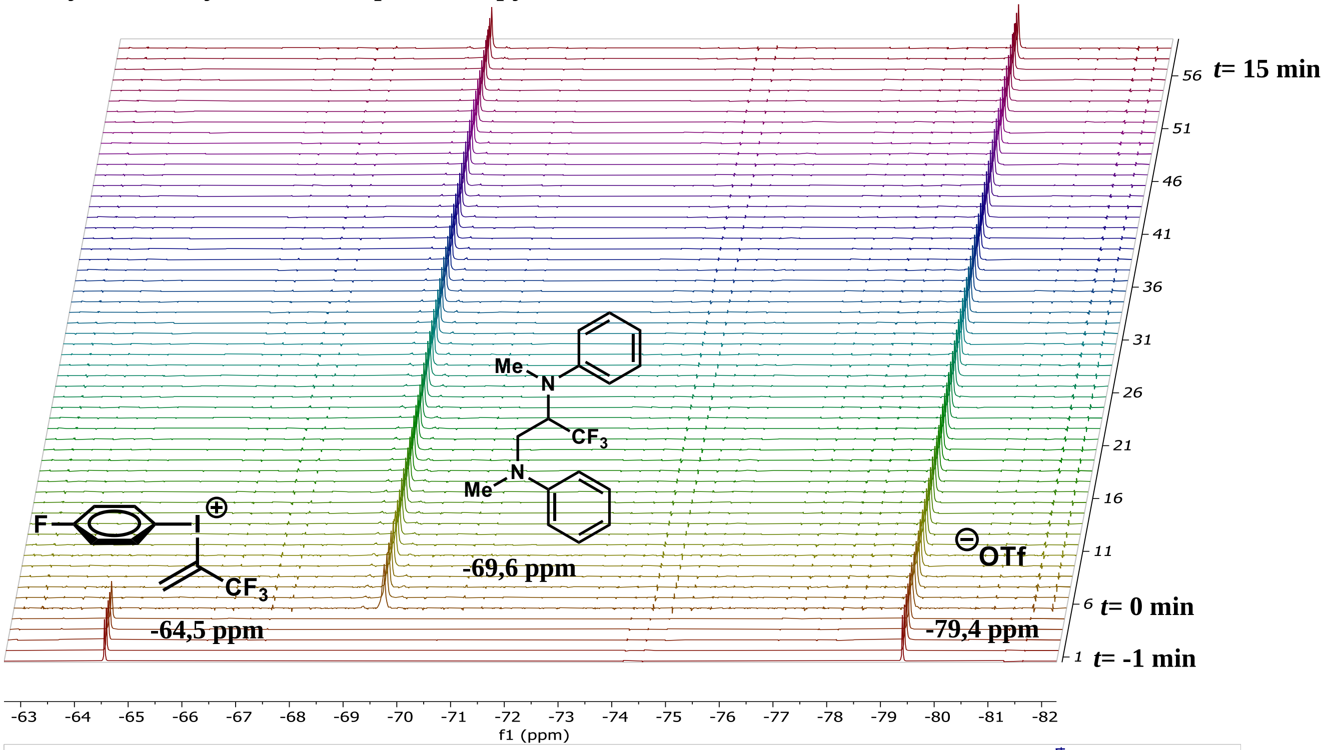

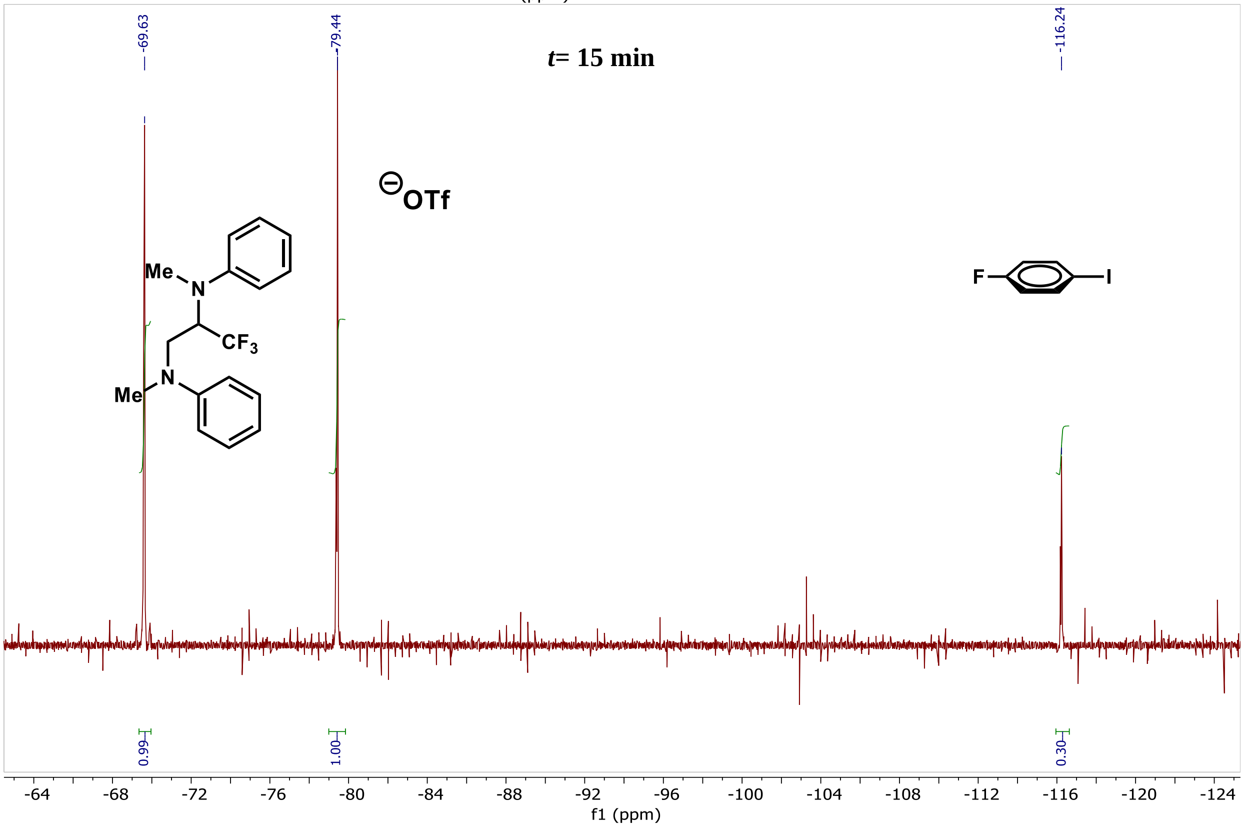

Supplementary Figure 3: Reaction monitoring of homodiamination reaction with *N*-ethylaniline by ^{19}F-NMR-spectroscopy

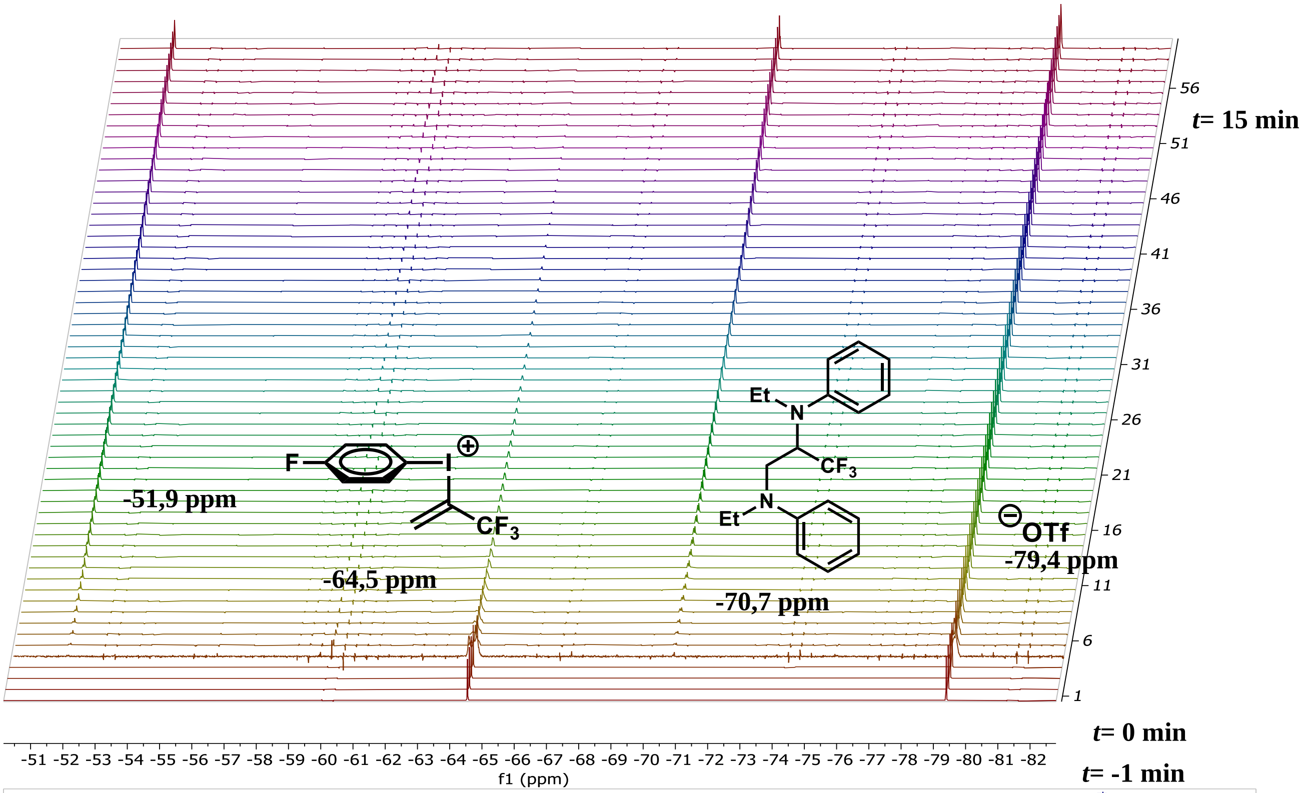

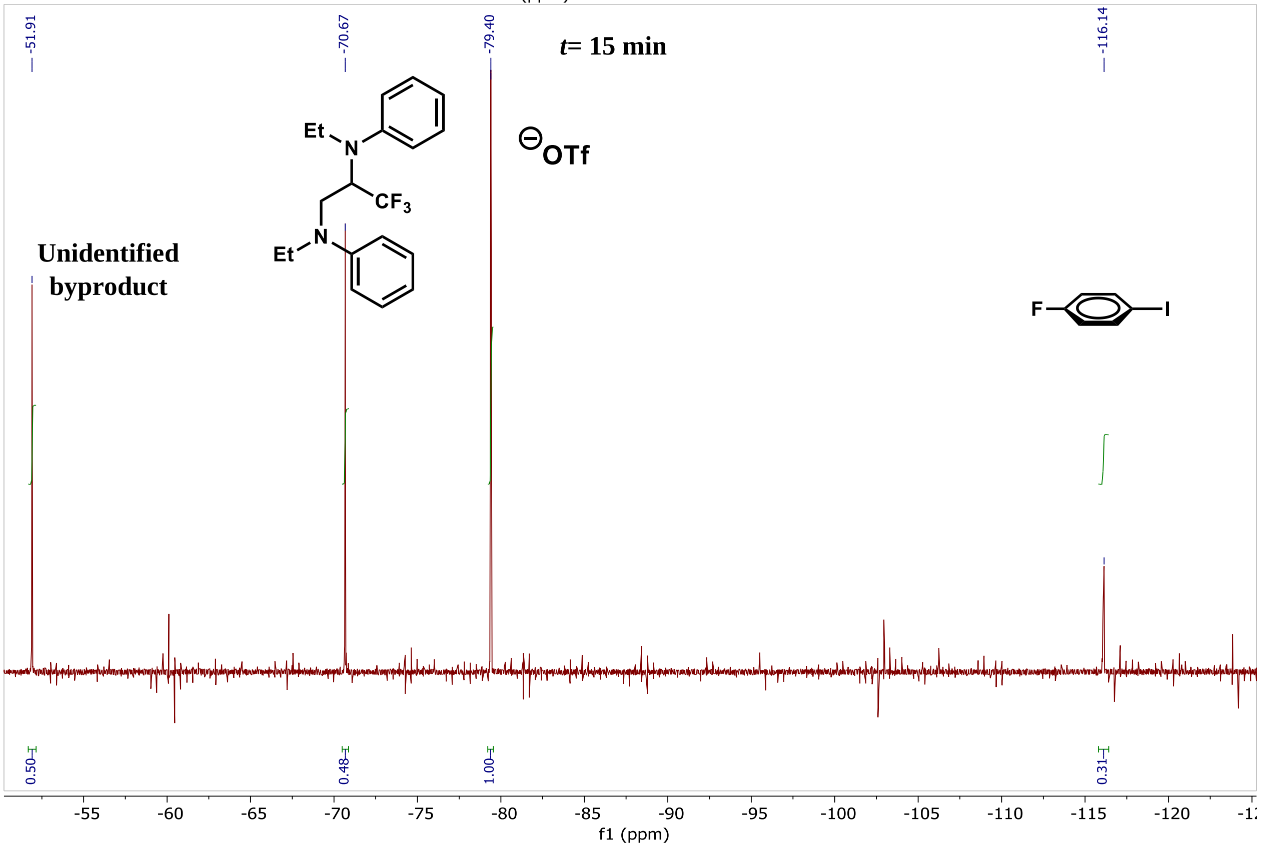

Supplementary Figure 4: GC-MS TIC of homodiamination reaction with *N*-ethylaniline

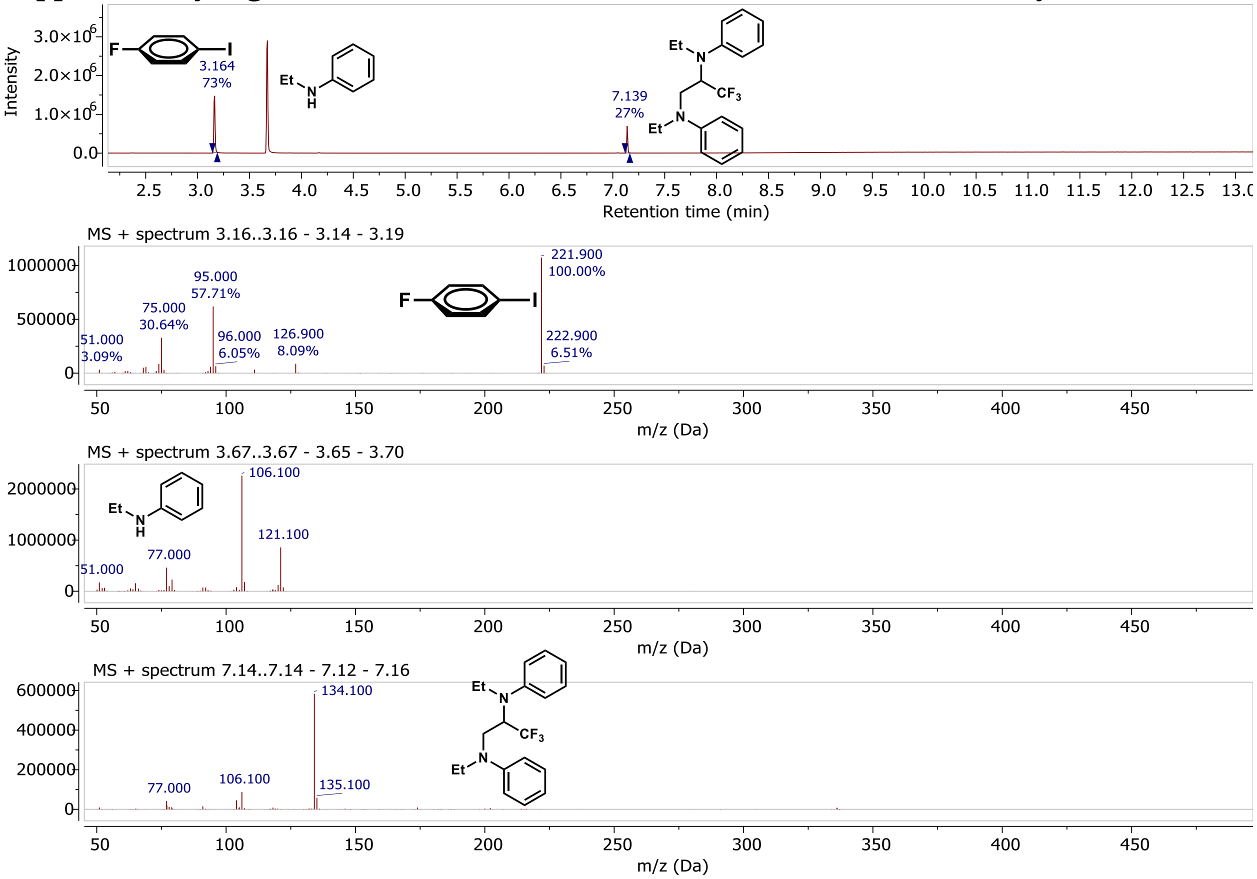

Supplementary Figure 5: Reaction monitoring of homodiamination reaction with indoline by ^{19}F-NMR-spectroscopy

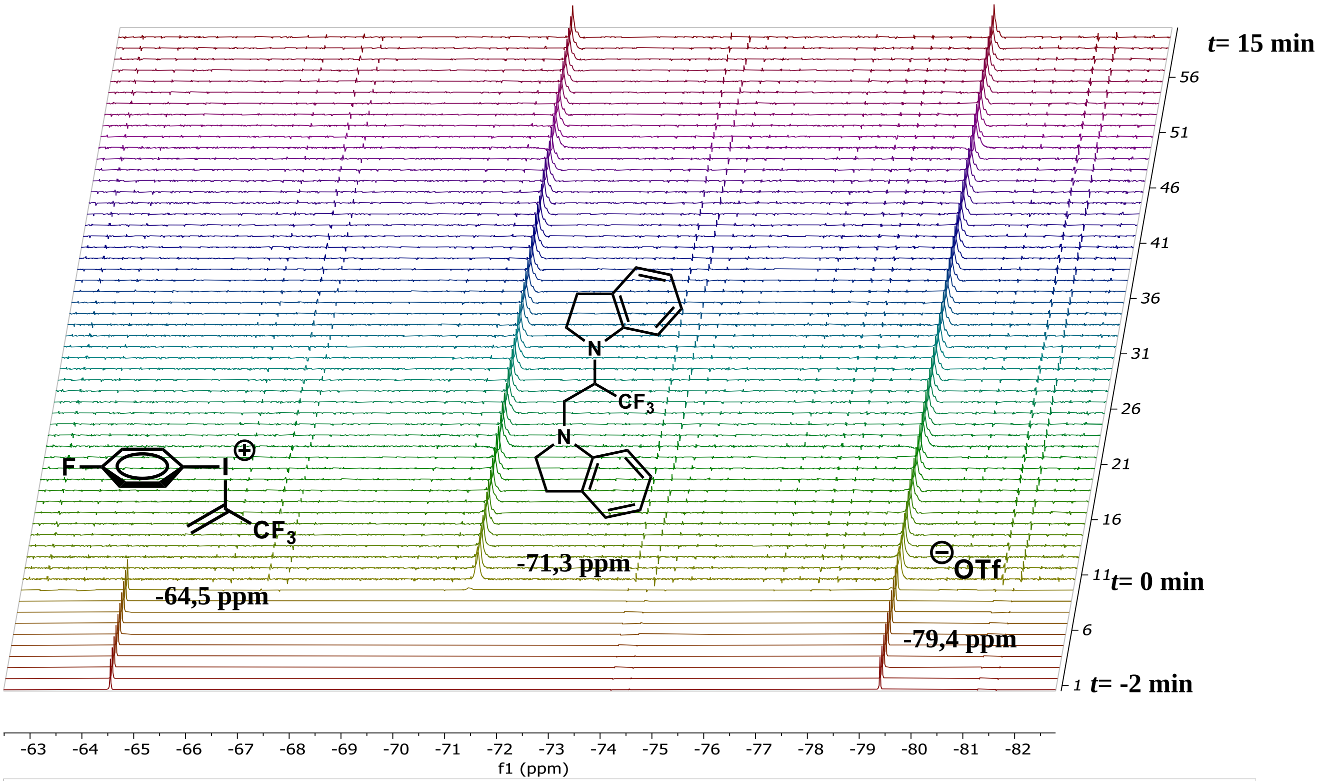

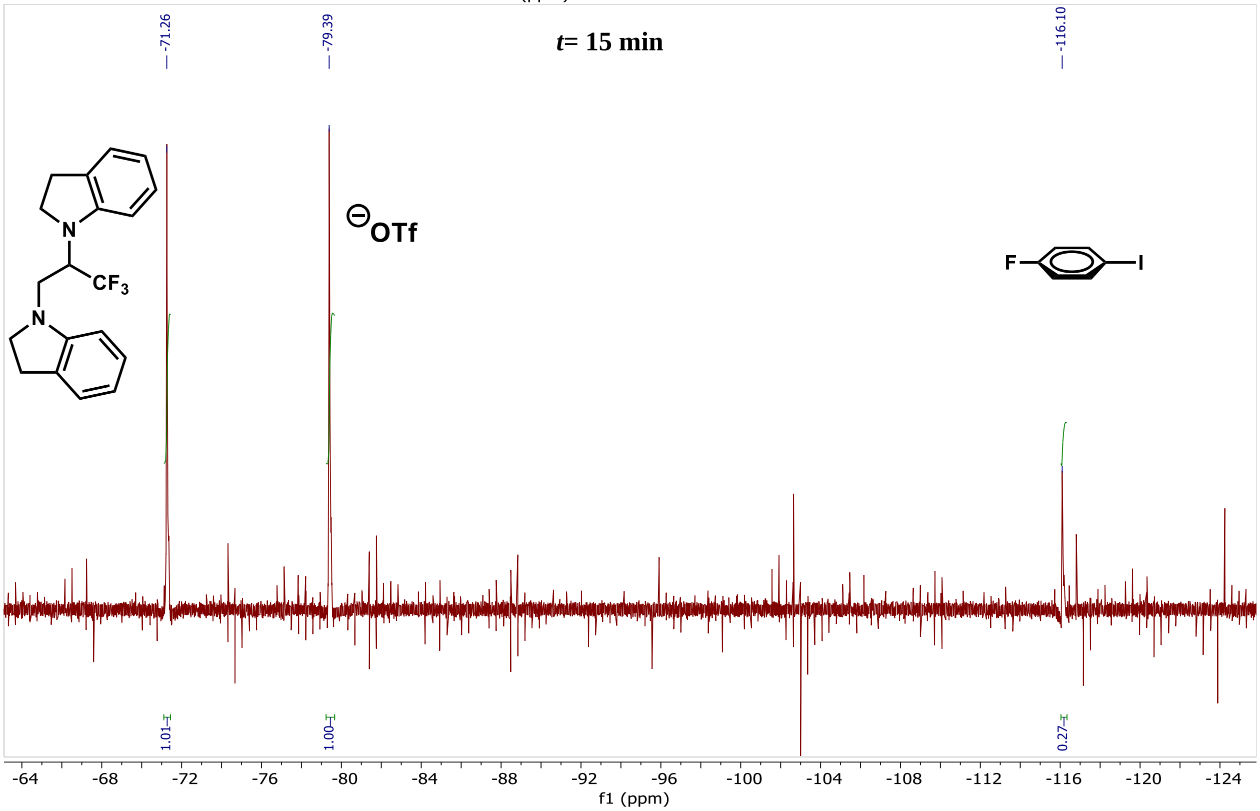

General procedure for the synthesis of homofunctionalized diamines

Vial was charged with stirring bar, appropiate amine (2.5 equiv) and acetonitrile (1 mL / 0.1 mmol). To the vigorously stirred mixture, sodium carbonate (1.5 equiv) was added, then (4-fluorophenyl)(3,3,3-trifluoroprop-1-en-2-yl)iodonium trifluoromethanesulfonate **(1)** (1 equiv) was added portionwise. Reaction mixture was stirred for 3 hours, diluted with dichloromethane and concentrated onto Celite under reduced pressure. Residue was purified by flash column chromatography (hexanes:ethyl acetate = 100:0 → 50:50).

*Synthesis of compound **7***

3,3,3-trifluoro-N^1,N^2-dimethyl-N^1,N^2-diphenylpropane-1,2-diamine

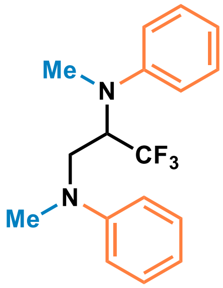

Compound **7** was prepared according to general procedure from *N*-methylaniline (54 µL, 0.5 mmol, 2.5 equiv), sodium carbonate (31.8 mg, 0.3 mmol, 1.5 equiv) and **1** (92.3 mg, 0.2 mmol, 1 equiv) to afford a pale yellow oil (47.1 mg, 0.153 mmol, 76%). **R$_f$**= 0.53 (hexanes:ethyl acetate= 10:1). **LRMS** (EI, 70 eV): m/z (%): 308(5), 188(3), 132(2), 121(9), 120(100), 119(2), 118(3), 105(6), 104(9), 91(4), 79(2), 78(2), 77(14), 51(3). **^{1}H NMR** (250 MHz, $CDCl_3$) δ 7.2 (t, *J* = 8.6, 7.5 Hz, 2H), 7.1 (t, *J* = 8.6, 7.4 Hz, 2H), 6.8 – 6.6 (m, 6H), 4.5 (pd, *J* = 8.4, 4.0 Hz, 1H), 3.9 (dd, *J* = 15.4, 4.0 Hz, 1H), 3.7 (dd, *J* = 15.4, 8.5 Hz, 1H), 2.9 (s, 6H). **^{19}F NMR** (235 MHz, $CDCl_3$) δ -70.01. **^{13}C NMR** (63 MHz, $CDCl_3$) δ 149.9, 148.2, 129.7, 129.3, 126.0 (q, *J* = 288.8 Hz), 119.0, 117.5, 114.5, 112.6, 59.2 (q, *J* = 26.3 Hz), 49.4 (d, *J* = 0.9 Hz), 39.5, 32.3 (d, *J* = 1.6 Hz). **IR** (ATR, cm^{-1}) 3095, 3063, 3029, 2910, 2827, 1598, 1503, 1451, 1363, 1314, 1272, 1239, 1207, 1166, 1141, 1117, 1095, 1034, 1000, 963, 895, 858, 746, 691, 516. **HRMS** m/z $[M+H]^+$ calculated for $C_{17}H_{20}N_2F_3^+$: 309.1579, found: 309.1579.

*Synthesis of compound **8***

N^1,N^2-diethyl-3,3,3-trifluoro-N^1,N^2-diphenylpropane-1,2-diamine

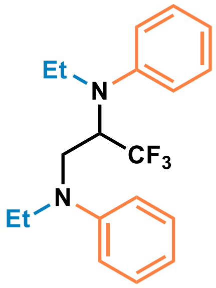

Compound **8** was prepared according to general procedure from *N*-ethylaniline (96 µL, 0.75 mmol, 2.5 equiv), sodium carbonate (47.7 mg, 0.45 mmol, 1.5 equiv) and **1** (140 mg, 0.3 mmol, 1 equiv) to afford a pale yellow oil (13.3 mg, 0.0395 mmol, 13% yield). **R$_f$**= 0.55 (hexanes:ethyl acetate= 10:1). **LRMS** (EI, 70 eV): m/z (%): 336(1), 214(1), 202(1), 174(1), 135(10), 134(100), 132(1), 118(1), 107(1), 106(9), 106(6), 105(2), 105(1), 104(7), 91(3), 79(3), 78(1), 77(11), 65(1), 51(2). **^{1}H NMR** (250 MHz, $CDCl_3$) δ 7.22 – 7.02 (m, 4H), 6.78 – 6.54 (m, 6H), 4.36 (td, *J* = 7.9, 4.6 Hz, 1H), 3.77 – 3.53 (m, 2H), 3.42 – 3.16 (m, 4H), 1.08 (t, *J* = 6.9 Hz, 3H), 1.01

(t, J = 7.1 Hz, 3H). **^{19}F NMR** (235 MHz, Chloroform-d) δ -69.86. **^{13}C NMR** (63 MHz, Chloroform-d) δ 147.9, 129.7, 129.2, 126.4 (q, J = 288.3 Hz), 119.4, 117.7, 116.6, 113.6, 60.6 (q, J = 25.5 Hz), 48.6, 46.5, 39.1, 13.1, 12.1. **IR** (ATR, cm^{-1}) 3094, 3063, 3026, 2973, 2929, 2873, 1598, 1502, 1449, 1353, 1299, 1262, 1232, 1188, 1161, 1141, 1103, 1055, 1038, 973, 913, 888, 851, 795, 744, 692, 615, 579, 522, 468, 413. **HRMS** m/z [M+H]$^+$ calculated for $C_{19}H_{24}N_2F_3^+$: 337.1892, found: 337.1893.

*Synthesis of compound **9***

1,1'-(3,3,3-trifluoropropane-1,2-diyl)diindoline

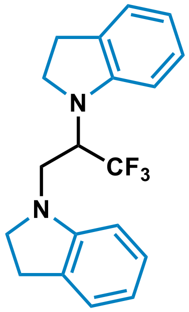

Compound **9** was prepared according to general procedure from indoline (57 µL, 0.5 mmol, 2.5 equiv), sodium carbonate (31.8 mg, 0.3 mmol, 1.5 equiv) and **1** (93.2 mg, 0.2 mmol, 1 equiv) to afford a brown oil (62.1 mg, 0.187 mmol, 93% yield). **R_f**= 0.55 (hexanes:ethyl acetate= 10:1). **LRMS** (EI, 70 eV): m/z (%): 332(17), 200(8), 133(15), 133(8), 132(100), 132(49), 131(8), 130(20), 130(9), 118(6), 117(18), 117(10), 115(7), 103(6), 91(7), 89(6), 77(10), 77(5). **^{1}H NMR** (250 MHz, Methanol-d_4) δ 7.15 – 6.92 (m, 4H), 6.74 – 6.41 (m, 4H), 4.57 (ddt, J = 16.7, 8.5, 4.2 Hz, 1H), 3.78 (dd, J = 14.0, 9.5 Hz, 2H), 3.61 (q, J = 8.7 Hz, 1H), 3.48 – 3.32 (m, 2H), 3.26 (q, J = 8.9 Hz, 1H), 2.99 (t, J = 8.7 Hz, 2H), 2.89 – 2.65 (m, 2H). **^{19}F NMR** (235 MHz, Methanol-d_4) δ -72.00. **^{13}C NMR** (63 MHz, Methanol-d_4) δ 152.8, 151.7, 131.0, 129.8, 128.3, 128.2, 127.5 (q, J = 287.1 Hz), 125.6, 125.5, 119.2, 118.9, 107.5, 106.9, 56.6 (q, J = 27.0 Hz), 54.5, 48.1, 46.3 (q, J = 1.9 Hz), 29.3, 29.1. **IR** (ATR, cm^{-1}) 3048, 3029, 2952, 2929, 2850, 1605, 1487, 1475, 1460, 1263, 1242, 1192, 1157, 1146, 1120, 1090, 1054, 1025, 991, 868, 739, 713, 700, 630, 577, 417. **HRMS** m/z [M+H]$^+$ calculated for $C_{19}H_{20}N_2F_3^+$: 333.1579, found: 333.1579.

*Synthesis of compound **10***

1,1'-(3,3,3-trifluoropropane-1,2-diyl)bis(1,2,3,4-tetrahydroquinoline)

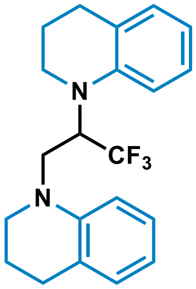

Compound **10** was prepared according to general procedure from 1,2,3,4-tetrahydroquinoline (64 µL, 0.5 mmol, 2.5 equiv), sodium carbonate (31.8 mg, 0.3 mmol, 1.5 equiv) and **1** (93.2 mg, 0.2 mmol, 1 equiv) to afford a brown oil (36.1 mg, 0.10 mmol, 50% yield). **R_f**= 0.55 (hexanes:ethyl acetate= 10:1). **LRMS** (EI, 70 eV): m/z (%): 360(3), 147(11), 146(100), 144(4), 131(7), 130(11), 118(8), 117(9), 115(3), 91(11), 77(3). **^{1}H NMR** (250 MHz, Chloroform-d) δ 7.14 (t, J = 7.8 Hz, 1H), 7.07 – 6.90 (m, 3H), 6.76 – 6.57 (m, 3H), 6.52 (d, J = 8.3 Hz, 1H), 4.76 (tt, J = 12.9, 6.3 Hz, 1H), 3.88 (dd, J = 15.3, 4.4 Hz, 1H), 3.71 (dd, J = 15.3, 7.6 Hz,

1H), 3.39 (dt, J = 16.3, 5.5 Hz, 4H), 2.84 (h, J = 9.8 Hz, 2H), 2.72 (t, J = 6.4 Hz, 2H), 2.14 – 1.74 (m, 4H). **^{19}F NMR** (235 MHz, Chloroform-*d*) δ -69.3. **^{13}C NMR** (63 MHz, Chloroform-*d*) δ 144.8, 144.2, 129.9, 129.6, 127.5, 127.2, 124.8 (q, J = 289.1 Hz), 123.7, 123.2, 117.7, 116.6, 112.0, 110.0, 56.3 (q, J = 25.2 Hz), 50.8, 48.3, 43.6, 28.2, 28.1, 22.2, 22.1. **IR** (ATR, cm^{-1}) 3067, 3022, 2932, 2890, 2846, 1713, 1602, 1575, 1496, 1458, 1348, 1302, 1275, 1235, 1218, 1188, 1157, 1129, 1107, 1061, 742, 689, 529. **HRMS** m/z [M+H]$^+$ calculated for $C_{21}H_{24}N_2F_3^+$: 361.1892, found: 361.1906.

*Synthesis of compound **11***

3,3,3-trifluoro-*N*^{1},*N*^{2}-bis(2-methoxyphenyl)-*N*^{1},*N*^{2}-dimethylpropane-1,2-diamine

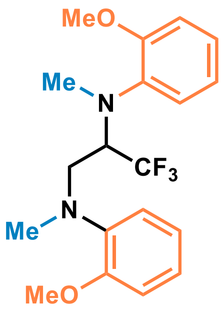

Compound **11** was prepared according to general procedure from 2-methoxy-*N*-methylaniline (70.7 mg, 0.5 mmol, 2.5 equiv), sodium carbonate (31.8 mg, 0.3 mmol, 1.5 equiv) and **1** (93.2 mg, 0.2 mmol, 1 equiv) to afford a brown oil (32.2 mg, 0.087 mmol, 44% yield). **R$_f$**= 0.28 (hexanes:ethyl acetate= 10:1). **LRMS** (EI, 70 eV): m/z (%): 368(1), 218(1), 151(10), 150(100), 148(1), 136(2), 135(20), 134(28), 121(2), 120(5), 107(1), 106(1), 104(1), 93(2), 92(2), 91(2), 79(2), 78(2), 77(5), 65(3), 51(1). **^{1}H NMR** (250 MHz, Chloroform-*d*) δ 7.04 – 6.72 (m, 8H), 4.60 (h, J = 8.5 Hz, 1H), 3.81 (s, 3H), 3.72 (s, 3H), 3.66 (s, 1H), 3.63 (s, 1H), 2.87 (s, 6H). **^{19}F NMR** (235 MHz, Chloroform-*d*) δ -68.26. **^{13}C NMR** (63 MHz, Chloroform-*d*) δ 152.7, 151.5, 140.5, 129.3 (q, J = 290.6 Hz), 122.5, 122.1, 121.1, 121.0, 120.4, 111.8, 111.7, 60.1 (q, J = 25.3 Hz), 55.4 (d, J = 2.4 Hz), 51.1, 41.1, 34.0 (d, J = 1.1 Hz). **IR** (ATR, cm^{-1}) 3063, 2997, 2956, 2904, 2836, 1594, 1500, 1456, 1438, 1235, 1160, 1110, 1090, 1055, 1027, 991, 961, 909, 855, 733, 689, 591, 530, 488, 451. **HRMS** m/z [M+H]$^+$ calculated for $C_{19}H_{24}N_2O_2F_3^+$: 369.179, found: 369.1789.

*Synthesis of compound **12***

3,3,3-trifluoro-*N*^{1},*N*^{2}-dimethyl-*N*^{1},*N*^{2}-di-m-tolylpropane-1,2-diamine

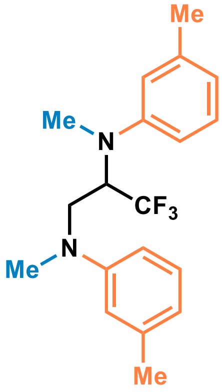

Compound **12** was prepared according to general procedure from *N*-methyl-3-toluidine (77.3 mg, 0.625 mmol, 2.5 equiv), sodium carbonate (39.7 mg, 0.375 mmol, 1.5 equiv) and **1** (117 mg, 0.25 mmol, 1 equiv) to afford a colorless oil (54.6 mg, 0.162 mmol, 65% yield). **R$_f$**= 0.65 (hexanes:ethyl acetate= 10:1). **LRMS** (EI, 70 eV): m/z (%): 337(2), 336(11), 202(3), 146(2), 135(17), 134(100), 133(3), 132(2), 120(2), 119(11), 118(12), 105(3), 92(3), 91(19), 90(2), 89(2), 77(3), 65(6). **^{1}H NMR** (250 MHz, Chloroform-*d*) δ 7.14 – 7.01 (m, 1H), 6.96 (t, J = 7.8 Hz, 1H), 6.56 – 6.49 (m, 2H), 6.47 – 6.36 (m, 3H), 6.31 – 6.27 (m, 1H), 4.47 (pd, J = 8.4, 4.1 Hz, 1H), 3.82 (dd, J = 15.4, 4.1 Hz, 1H), 3.59 (dd, J = 15.4,

8.4 Hz, 1H), 2.83 (s, 6H), 2.20 (s, 3H), 2.10 (s, 3H). **^{19}F NMR** (235 MHz, Chloroform-*d*) δ -69.99. **^{13}C NMR** (63 MHz, Chloroform-*d*) δ 150.0, 148.1, 139.4, 139.0, 129.5, 129.1, 126.0 (q, J = 288.8 Hz), 119.9, 118.4, 115.4, 113.5, 111.6, 109.9, 58.9 (q, J = 26.2 Hz), 49.4, 39.8, 32.3 (q, J = 1.6 Hz), 22.0, 21.8. **IR** (ATR, cm^{-1}) 3046, 2945, 2918, 2826, 1602, 1582, 1496, 1449, 1360, 1302, 1273, 1245, 1181, 1163, 1139, 1119, 1099, 1046, 1017, 995, 968, 909, 839, 766, 757, 732, 691, 576, 478, 441. **HRMS** m/z [M+H]$^+$ calculated for $C_{19}H_{24}N_2F_3^+$: 337.1892, found: 337.1897.

*Synthesis of compound **13***

3,3,3-trifluoro- N^1,N^2-bis(3-fluorophenyl)- N^1,N^2-dimethylpropane-1,2-diamine

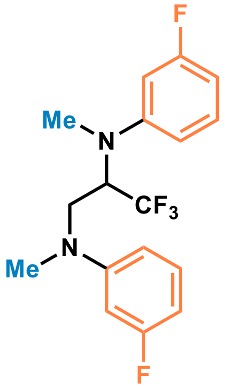

Compound **13** was prepared according to general procedure from 3-fluoro-*N*-methylaniline (58 µL, 0.5 mmol, 2.5 equiv), sodium carbonate (31.8 mg, 0.3 mmol, 1.5 equiv) and **1** (93.2 mg, 0.2 mmol, 1 equiv) to afford a pale yellow oil (30.2 mg, 0.088 mmol, 44% yield). **R$_f$**= 0.56 (hexanes:ethyl acetate= 10:1). **LRMS** (EI, 70 eV): m/z (%): 344(2), 206(3), 150(2), 139(9), 138(100), 136(3), 123(5), 122(10), 110(2), 109(5), 96(3), 95(11), 75(3). **^{1}H NMR** (250 MHz, Chloroform-*d*) δ 7.21 (dd, J = 8.2, 7.1 Hz, 1H), 7.09 (dd, J = 8.2, 7.6 Hz, 1H), 6.56 – 6.27 (m, 6H), 4.51 (pd, J = 8.3, 4.0 Hz, 1H), 3.90 (dd, J = 15.5, 3.9 Hz, 1H), 3.71 (dd, J = 15.5, 8.6 Hz, 1H), 2.93 (s, 3H), 2.92 (s, 3H). **^{19}F NMR** (235 MHz, Chloroform-*d*) δ -70.20 (s, 3F), -111.62 (s, 1F), -111.91 (s, 1F). **^{13}C NMR** (63 MHz, Chloroform-*d*) δ 164.5 (d, J = 243.1 Hz), 164.0 (d, J = 243.6 Hz), 151.5 (d, J = 10.2 Hz), 149.8 (d, J = 10.5 Hz), 130.8 (d, J = 10.3 Hz), 130.4 (d, J = 10.1 Hz), 125.7 (q, J = 288.2 Hz), 109.8 (d, J = 2.5 Hz), 107.9 (d, J = 2.4 Hz), 105.7 (d, J = 21.4 Hz), 104.1 (d, J = 21.6 Hz), 101.6 (d, J = 26.0 Hz), 99.7 (d, J = 26.2 Hz), 58.9 (q, J = 26.9 Hz), 49.2, 39.6, 32.5 (q, J = 1.7 Hz). **IR** (ATR, cm^{-1}) 3078, 3043, 2917, 2830, 1616, 1580, 1497, 1452, 1297, 1272, 1234, 1157, 1130, 1117, 1097, 1076, 1048, 1014, 1004, 978, 903, 822, 754, 681, 496, 451. **HRMS** m/z [M+H]$^+$ calculated for $C_{17}H_{18}N_2F_5^+$: 345.139, found: 345.1396.

*Synthesis of compound **14***

N[1],*N*[2]-bis(3-chlorophenyl)-3,3,3-trifluoro- *N*[1],*N*[2]-dimethylpropane-1,2-diamine

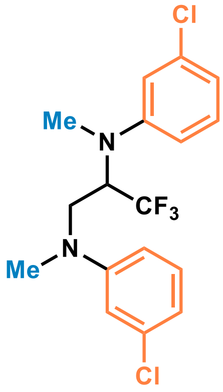

Compound **14** was prepared according to general procedure from 3-chloro-*N*-methylaniline (63 µL, 0.50 mmol, 2.5 equiv), sodium carbonate (31.8 mg, 0.30 mmol, 1.5 equiv) and **1** (93.2 mg, 0.20 mmol, 1 equiv) to afford a pale yellow oil (29.7 mg, 0.079 mmol, 39% yield). **R**$_f$= 0.50 (hexanes:ethyl acetate= 10:1). **LRMS** (EI, 70 eV): m/z (%): 376(1), 222(2), 157(3), 156(32), 155(9), 154(100), 141(2), 140(3), 139(4), 138(7), 125(2), 119(4), 118(6), 111(4), 111(4), 91(2), 77(3), 76(2), 75(4). ^{1}**H NMR** (250 MHz, Chloroform-*d*) *δ* 7.18 (t, *J* = 8.1 Hz, 1H), 7.07 (t, *J* = 8.1 Hz, 1H), 6.77 (dt, *J* = 7.9, 2.2 Hz, 2H), 6.70 – 6.52 (m, 3H), 6.48 (dd, *J* = 8.4, 2.6 Hz, 1H), 4.47 (qd, *J* = 8.3, 4.0 Hz, 1H), 3.88 (dd, *J* = 15.5, 4.0 Hz, 1H), 3.71 (dd, *J* = 15.5, 8.6 Hz, 1H), 2.91 (s, 6H). ^{19}**F NMR** (235 MHz, Chloroform-*d*) *δ* -70.13. ^{13}**C NMR** (63 MHz, Chloroform-*d*) *δ* 150.8, 148.9, 135.7, 135.3, 130.7, 130.3, 125.6 (q, *J* = 288.2 Hz), 119.2, 117.9, 114.5, 112.9, 112.5, 110.8, 58.8 (q, *J* = 26.9 Hz), 49.4, 39.8, 32.5. **IR** (ATR, cm^{-1}) 3077, 2951, 2912, 2829, 1592, 1563, 1489, 1435, 1364, 1293, 1270, 1239, 1212, 1167, 1147, 1097, 1046, 1002, 987, 971, 913, 832, 814, 759, 716, 681, 571, 491, 441, 410. **HRMS** m/z $[M+H]^+$ calculated for $C_{17}H_{18}N_2F_3Cl_2^+$: 377.0799, found: 377.0804.

*Synthesis of compound **15***

3,3,3-trifluoro- *N*[1],*N*[2]-dimethyl- *N*[1],*N*[2]-di-p-tolylpropane-1,2-diamine

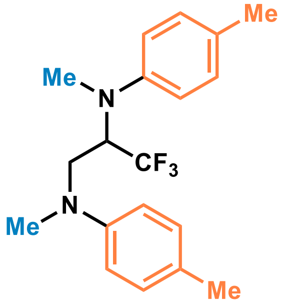

Compound **15** was prepared according to general procedure from *N*-methyl-4-toluidine (75.7 mg, 0.625 mmol, 2.5 equiv), sodium carbonate (39.7 mg, 0.375 mmol, 1.5 equiv) and **1** (117 mg, 0.25 mmol, 1 equiv) to afford a colorless oil (51.2 mg, 0.152 mmol, 61% yield). **R**$_f$= 0.55 (hexanes:ethyl acetate= 10:1). **LRMS** (EI, 70 eV): m/z (%): 336(3), 202(2), 135(10), 134(100), 119(7), 118(8), 105(2), 91(10), 77(2), 65(3). ^{1}**H NMR** (250 MHz, Chloroform-*d*) *δ* 7.13 (d, *J* = 8.5 Hz, 2H), 7.03 (d, *J* = 8.5 Hz, 2H), 6.69 (d, *J* = 8.5 Hz, 2H), 6.62 (d, *J* = 8.6 Hz, 2H), 4.54 (pd, *J* = 8.4, 4.0 Hz, 1H), 3.91 (dd, *J* = 15.3, 4.0 Hz, 1H), 3.72 (dd, *J* = 15.3, 8.6 Hz, 1H), 3.04 – 2.86 (m, 6H), 2.34 (s, 3H), 2.28 (s, 3H). ^{19}**F NMR** (235 MHz, Chloroform-*d*) *δ* -69.96. ^{13}**C NMR** (63 MHz, Chloroform-*d*) *δ* 147.8, 146.1, 130.2, 129.8, 128.2, 126.7, 126.1 (q, *J* = 289.4 Hz), 114.8, 112.9, 59.5 (q, *J* = 25.9 Hz), 49.6, 39.7, 32.3 (q, *J* = 1.4 Hz), 20.4. **IR** (ATR, cm^{-1}) 3012, 2919, 2866, 2824, 1618, 1517, 1363, 1310, 1270, 1238, 1205,

1164, 1140, 1116, 1095, 1046, 998, 964, 909, 800, 732, 678, 518. **HRMS** m/z $[M+H]^+$ calculated for $C_{19}H_{24}N_2F_3^+$: 337.1892, found: 337.1889.

*Synthesis of compound **16***
N^1,N^2-bis(4-chlorophenyl)-3,3,3-trifluoro- N^1,N^2-dimethylpropane-1,2-diamine

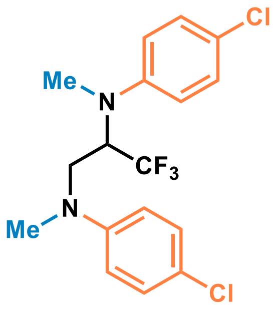

Compound **16** was prepared according to general procedure from 4-chloro-*N*-methylaniline (94 μL, 0.75 mmol, 2.5 equiv), sodium carbonate (47.7 mg, 0.45 mmol, 1.5 equiv) and **1** (140 mg, 0.3 mmol, 1 equiv) to afford a pale yellow oil (56.4 mg, 0.15 mmol, 50% yield). $\mathbf{R_f}$= 0.53 (hexanes:ethyl acetate= 10:1). **LRMS** (EI, 70 eV): m/z (%): 378(2), 376(2), 222(2), 157(3), 156(32), 155(9), 154(100), 141(2), 140(2), 140(2), 139(6), 138(9), 125(2), 119(5), 118(5), 113(2), 111(6), 77(2), 75(2). **^{1}H NMR** (250 MHz, Chloroform-*d*) δ 7.27 – 7.17 (m, 2H), 7.17 – 7.07 (m, 2H), 6.61 (d, *J* = 9.1 Hz, 2H), 6.55 (d, *J* = 9.1 Hz, 2H), 4.44 (ddq, *J* = 12.1, 8.3, 4.2 Hz, 1H), 3.88 (dd, *J* = 15.5, 3.8 Hz, 1H), 3.70 (dd, *J* = 15.5, 8.7 Hz, 1H), 2.91 (s, 6H). **^{19}F NMR** (235 MHz, Chloroform-*d*) δ -70.13. **^{13}C NMR** (63 MHz, Chloroform-*d*) δ 148.4, 146.6, 129.5, 129.2, 125.7 (q, *J* = 288.4 Hz), 124.1, 122.7, 115.6, 113.8, 59.1 (q, *J* = 26.6 Hz), 49.3, 39.7, 32.4 (q, *J* = 1.4 Hz). **IR** (ATR, cm^{-1}) 3078, 3047, 2990, 2945, 2904, 2829, 1597, 1496, 1364, 1309, 1269, 1238, 1207, 1166, 1141, 1117, 1095, 1046, 997, 964, 807, 763, 710, 621, 510, 411. **HRMS** m/z $[M+H]^+$ calculated for $C_{17}H_{18}N_2F_3Cl_2^+$: 377.0799, found: 377.0797.

*Synthesis of compound **17***
N^1,N^2-bis(4-bromophenyl)-3,3,3-trifluoro- N^1,N^2-dimethylpropane-1,2-diamine

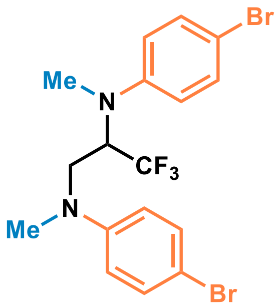

Compound **17** was prepared according to general procedure from 3-bromo-*N*-methylaniline (95.9 mg, 0.5 mmol, 2.5 equiv), sodium carbonate (31.8 mg, 0.3 mmol, 1.5 equiv) and **1** (93.2 mg, 0.2 mmol, 1 equiv) to afford a pale yellow oil (53.7 mg, 0.115 mmol, 58% yield). $\mathbf{R_f}$= 0.43 (hexanes:ethyl acetate= 10:1). **LRMS** (EI, 70 eV): m/z (%): 468(1), 466(2), 464(1), 201(9), 200(96), 199(10), 198(100), 185(6), 184(9), 183(6), 182(8), 157(6), 155(6), 119(14), 118(16), 105(3), 104(5), 91(3), 77(4), 76(5). **^{1}H NMR** (250 MHz, Chloroform-*d*) δ ^{1}H NMR (250 MHz, Chloroform-*d*) δ 7.25 (d, *J* = 8.8 Hz, 2H), 7.16 (d, *J* = 8.7 Hz, 2H), 6.46 (d, *J* = 8.9 Hz, 2H), 6.40 (d, *J* = 8.9 Hz, 2H), 4.34 (td, *J* = 8.2, 3.7 Hz, 1H), 3.78 (dd, *J* = 15.4, 3.7 Hz, 1H), 3.60 (dd, *J* = 15.5, 8.7 Hz, 1H), 2.80 (s, 6H). **^{19}F NMR** (235 MHz, Chloroform-*d*) δ -70.16. **^{13}C NMR** (63 MHz, Chloroform-*d*) δ 148.8, 146.8, 132.4, 132.1, 123.3 (q, *J* = 289.3, 288.6 Hz), 116.0, 114.2, 111.4, 109.9, 58.9 (q, *J* = 26.7 Hz), 49.3, 39.7, 32.4.

IR (ATR, cm^{-1}) 3078, 3044, 2989, 2946, 2902, 2829, 1866, 1741, 1589, 1493, 1373, 1312, 1269, 1238, 1205, 1167, 1141, 1117, 1096, 1046, 994, 963, 906, 861, 804, 756, 730, 705, 688, 648, 638, 628, 596, 566, 540, 510, 481, 461. **HRMS** m/z $[M+H]^+$ calculated for $C_{17}H_{18}N_2Br_2F_3^+$: 464.9789, found: 464.9785.

*Synthesis of compound **18***

3,3,3-trifluoro- N^1,N^2-bis(4-methoxyphenyl)- N^1,N^2-dimethylpropane-1,2-diamine

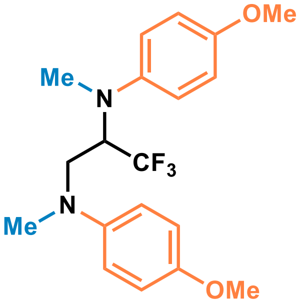

Compound **18** was prepared according to general procedure from 4-methoxy-*N*-methylaniline (70 mg, 0.75 mmol, 2.5 equiv), sodium carbonate (31.8 mg, 0.45 mmol, 1.5 equiv) and **1** (93.2 mg, 0.2 mmol, 1 equiv) to afford a pale yellow oil (57.5 mg, 0.156 mmol, 78% yield). $\mathbf{R_f}$= 0.60 (hexanes:ethyl acetate= 7:3). **LRMS** (EI, 70 eV): m/z (%): 368(8), 218(3), 151(14), 150(100), 136(3), 135(13), 134(5), 121(3), 120(9), 92(3), 77(3). **^{1}H NMR** (250 MHz, Chloroform-*d*) δ 6.89 (d, J = 8.8 Hz, 2H), 6.78 (d, J = 9.1 Hz, 2H), 6.73 (d, J = 8.4 Hz, 2H), 6.65 (d, J = 8.9 Hz, 2H), 4.39 (td, J = 8.2, 3.7 Hz, 1H), 3.80 (s, 3H), 3.78 – 3.60 (m, 5H), 2.91 (bs, 6H). **^{19}F NMR** (235 MHz, Chloroform-*d*) δ -69.95. **^{13}C NMR** (63 MHz, Chloroform-*d*) δ 153.1, 152.5, 144.5, 142.9, 126.2 (q, J = 289.4 Hz), 116.6, 115.2, 114.9, 114.6, 60.5 (q, J = 25.7 Hz), 55.8, 55.7, 50.2, 40.2, 32.6 (q, J = 1.1 Hz). **IR** (ATR, cm^{-1}) 3044, 2995, 2935, 2905, 2833, 1509, 1463, 1272, 1234, 1163, 1141, 1116, 1095, 1035, 998, 964, 909, 814, 730, 693, 676, 525. **HRMS** m/z $[M+H]^+$ calculated for $C_{19}H_{24}N_2O_2F_3^+$: 369.1790, found: 369.1790.

*Synthesis of compound **19***

3,3,3-trifluoro-N^1,N^2-dimethyl-N^1,N^2-bis(naphthalen-1-ylmethyl)propane-1,2-diamine

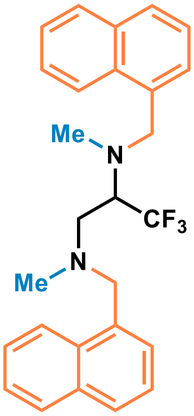

Compound **19** was prepared according to general procedure from *N*-methyl-1-(naphthalen-1-yl)methanamine (85.6 mg, 0.5 mmol, 2.5 equiv), sodium carbonate (31.8 mg, 0.3 mmol, 1.5 equiv) and **1** (93.2 mg, 0.2 mmol, 1 equiv) to afford a colorless oil (78.8 mg, 0.180 mmol, 90% yield). $\mathbf{R_f}$= 0.55 (hexanes:ethyl acetate= 10:1). **LRMS** (EI, 70 eV): m/z (%): 185(7), 184(51), 182(1), 170(1), 168(1), 167(1), 143(1), 142(13), 141(100), 139(3), 127(1), 116(1), 115(14), 89(1). **^{1}H NMR** (250 MHz, Chloroform-*d*) δ 8.04 – 7.88 (m, 1H), 7.79 (d, J = 7.7 Hz, 1H), 7.54 – 7.29 (m, 4H), 7.18 – 6.85 (m, 8H), 3.81 (s, 2H), 3.64 (d, J = 13.0 Hz, 1H), 3.41 (d, J = 12.9 Hz, 1H), 3.20 – 2.98 (m, 1H), 2.76 – 2.55 (m, 1H), 2.25 (dd, J = 13.6, 3.1 Hz, 1H), 1.85 (s, 3H), 1.79 (s, 3H). **^{19}F NMR** (235 MHz, Chloroform-*d*) δ -67.95. **^{13}C NMR** (63 MHz,

Chloroform-*d*) δ 134.5, 134.0, 134.0, 132.6, 132.4, 128.5, 128.4, 128.2, 128.1, 127.6, 127.4 (q, *J* = 290.4 Hz), 127.2, 125.8, 125.8, 125.7, 125.3, 125.2, 125.1, 124.7, 61.7 (q, *J* = 24.3 Hz), 61.5, 58.0, 53.6, 42.5, 36.5. **IR** (ATR, cm^{-1}) 3047, 2946, 2849, 2807, 1597, 1509, 1462, 1367, 1310, 1253, 1158, 1100, 1051, 1008, 907, 790, 773, 730, 703, 649, 520, 454, 414. **HRMS** m/z $[M+H]^+$ calculated for $C_{27}H_{28}N_2F_3^+$: 437.2205, found: 437.2198.

Synthesis of compound ***20***

3,3,3-trifluoro- N^1,N^1,N^2,N^2-tetraisobutylpropane-1,2-diamine

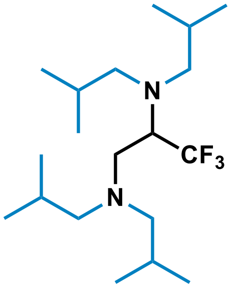

Compound **20** was prepared according to general procedure from diisobutylamine (130 μL, 0.75 mmol, 2.5 equiv), sodium carbonate (47.7 mg, 0.45 mmol, 1.5 equiv) and **1** (140 mg, 0.3 mmol, 1 equiv) to afford a pale yellow oil (70.8 mg, 0.201 mmol, 67% yield). $\mathbf{R_f}$= 0.75 (hexanes:ethyl acetate= 10:1). **LRMS** (EI, 70 eV): m/z (%): 309(1), 209(2), 190(2), 143(11), 142(100), 140(2), 126(1), 124(1), 100(4), 98(2), 86(7), 84(2), 57(10), 56(1), 55(1). ^{1}**H NMR** (250 MHz, Chloroform-*d*) δ 3.16 (qt, *J* = 8.9, 5.2 Hz, 1H), 2.74 – 2.48 (m, 2H), 2.46 – 2.21 (m, 4H), 2.06 (h, *J* = 7.9, 6.9 Hz, 4H), 1.69 (tp, *J* = 13.3, 6.6 Hz, 4H), 1.06 – 0.68 (m, 24H). ^{19}**F NMR** (235 MHz, Chloroform-*d*) δ -68.12. ^{13}**C NMR** (63 MHz, Chloroform-*d*) δ 127.7 (q, *J* = 289.1 Hz), 64.6, 60.8, 60.8 (q, *J* = 23.8 Hz), 53.9, 27.3, 26.5, 21.2, 21.1, 20.8, 20.7. **IR** (ATR, cm^{-1}) 2953, 2907, 2870, 2824, 2805, 2749, 2722, 1469, 1388, 1365, 1278, 1248, 1170, 1153, 1117, 1082, 1035, 990, 959, 927, 849, 824, 710, 620, 598, 484. **HRMS** m/z $[M+H]^+$ calculated for $C_{17}H_{40}N_2F_3^+$: 353.3144, found: 353.3148.

Synthesis of compound ***21***

N^1,N^1,N^2,N^2-tetrabenzyl-3,3,3-trifluoropropane-1,2-diamine

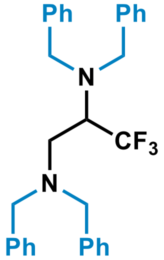

Compound **21** was prepared according to general procedure from dibenzylamine (100 μL, 0.5 mmol, 2.5 equiv), sodium carbonate (31.8 mg, 0.3 mmol, 1.5 equiv) and **1** (93.2 mg, 0.2 mmol, 1 equiv) to afford a white solid (65.2 mg, 0.133 mmol, 67% yield). $\mathbf{R_f}$= 0.63 (hexanes:ethyl acetate= 10:1). **M.p.=** 102-105 °C. **LRMS** (EI, 70 eV): m/z (%): 91(100), 210(79), 211(13), 92(8), 65(8), 181(4), 118(2), 89(2), 212(1), 208(1), 73(1), 109(1), 63(1), 207(1), 397(1), 51(1), 182(1), 165(1), 77(1). ^{1}**H NMR** (250 MHz, Chloroform-*d*) δ 7.76 – 6.93 (m, 20H), 3.87 – 3.62 (m, 6H), 3.54 (tt, *J* = 12.8, 6.4 Hz, 1H), 3.35 (d, *J* = 13.5 Hz, 2H), 3.03 (dd, *J* = 13.6, 7.2 Hz, 1H), 2.74 (dd, *J* = 13.6, 4.2 Hz, 1H). ^{19}**F NMR** (235 MHz, Chloroform-*d*) δ -67.32. ^{13}**C NMR** (63 MHz, Chloroform-*d*) δ 139.2, 138.7, 129.3, 129.1, 128.4, 128.4, 127.6 (d, *J* = 290.1 Hz), 127.3, 127.2, 58.7, 57.5 (q, *J* = 24.5 Hz), 54.5, 51.6.

IR (ATR, cm^{-1}) 3083, 3063, 3026, 3005, 2968, 2939, 2841, 2810, 2722, 1738, 1599, 1493, 1452, 1370, 1280, 1262, 1239, 1207, 1163, 1134, 1109, 1096, 1069, 1045, 1028, 973, 959, 913, 746, 734, 695, 588, 547, 516, 482, 471, 403. **HRMS** m/z $[M+H]^+$ calculated for $C_{31}H_{32}N_2F_3^+$: 489.2518, found: 489.2516.

Synthesis of compound ***22***

Tetramethyl 2,2',2'',2'''-((3,3,3-trifluoropropane-1,2-diyl)bis(azanetriyl))tetraacetate

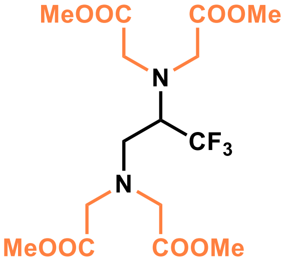

Compound **22** was prepared according to general procedure from dimethyl 2,2'-azanediyldiacetate (121 mg, 0.75 mmol, 2.5 equiv), sodium carbonate (47.7 mg, 0.45 mmol, 1.5 equiv) and **1** (140 mg, 0.3 mmol, 1 equiv) to afford a colorless liquid (122 mg, 0.293 mmol, 98% yield). $\mathbf{R_f}$= 0.30 (hexanes:ethyl acetate= 7:3). **LRMS** (EI, 70 eV): m/z (%): 416(1), 358(3), 357(20), 256(3), 256(3), 225(4), 198(3), 196(4), 175(8), 174(100), 147(2), 146(17), 146(14), 116(13), 74(2), 56(2). **^{1}H NMR** (250 MHz, Chloroform-*d*) δ 3.64 (s, 21H), 3.11 (dd, J = 14.3, 8.1 Hz, 1H), 2.97 (dd, J = 14.3, 4.2 Hz, 1H). **^{19}F NMR** (235 MHz, Chloroform-*d*) δ -70.72. **^{13}C NMR** (63 MHz, Chloroform-*d*) δ 171.8, 171.5, 126.5 (q, J = 290.2, 289.6, 289.1 Hz), 63.1 (q, J = 25.3 Hz), 55.1, 52.6, 51.7, 51.5, 50.7. **IR** (ATR, cm^{-1}) 3063, 2968, 2885, 2863, 2812, 1721, 1601, 1510, 1451, 1395, 1314, 1270, 1245, 1160, 1106, 1069, 1054, 1027, 862, 790, 773, 734, 706, 686, 584, 544, 518, 503, 452, 415. **HRMS** m/z $[M+H]^+$ calculated for $C_{15}H_{24}N_2O_8F_3^+$: 417.1485, found: 417.1489.

Synthesis of compound ***23***

N,*N*'-(3,3,3-trifluoropropane-1,2-diyl)bis(*N*-(4-methoxybenzyl)-*O*-methylhydroxylamine)

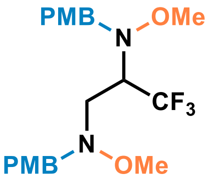

Compound **23** was prepared according to general procedure from *N*-(4-methoxybenzyl)-*O*-methylhydroxylamine (125 mg, 0.75 mmol, 2.5 equiv), sodium carbonate (47.7 mg, 0.45 mmol, 1.5 equiv) and **1** (140 mg, 0.3 mmol, 1 equiv) to afford a colorless liquid (84.9 mg, 0.198 mmol, 66% yield). $\mathbf{R_f}$= 0.44 (hexanes:ethyl acetate= 10:1). **LRMS** (EI, 70 eV): m/z (%): 398(1), 397(6), 365(1), 207(1), 180(3), 122(9), 121(100), 91(3), 78(3), 77(3), 73(2). **^{1}H NMR** (250 MHz, Chloroform-*d*) δ 7.20 (d, J = 8.6 Hz, 2H), 7.17 (d, J = 8.6 Hz, 2H), 6.77 (d, J = 8.5 Hz, 2H), 6.74 (d, J = 8.6 Hz, 2H), 3.98 (d, J = 12.8 Hz, 1H), 3.82 (d, J = 12.8 Hz, 1H), 3.76 – 3.62 (m, 8H), 3.31 (ddt, J = 16.1, 11.2, 5.7 Hz, 1H), 3.20 (s, 7H), 2.81 (dd, J = 14.1, 4.9 Hz, 1H). **^{19}F NMR** (235 MHz, Chloroform-*d*) δ -71.11. **^{13}C NMR** (63 MHz, Chloroform-*d*) δ 159.2 (d, J = 3.4 Hz), 130.9, 129.1, 129.0, 125.8 (q, J = 283.0 Hz), 113.8, 62.3, 61.8 (q, J = 26.5 Hz), 61.1, 61.0, 59.0, 55.3, 51.9. **IR** (ATR, cm^{-1}) 3104, 3061,

2996, 2938, 2901, 2837, 2815, 1612, 1585, 1511, 1463, 1442, 1350, 1302, 1245, 1170, 1144, 1109, 1034, 960, 910, 822, 783, 763, 733, 638, 601, 567, 520, 464, 425. **HRMS** m/z $[M+H]^+$ calculated for $C_{21}H_{28}N_2O_4F_3^+$: 429.2001, found: 429.2007.

*Synthesis of compound **24***

3,3,3-trifluoro- N^1,N^2-dihexyl- N^1,N^2-bis(4-methoxybenzyl)propane-1,2-diamine

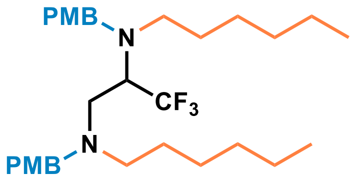

Compound **24** was prepared according to general procedure from *N*-(4-methoxybenzyl)hexan-1-amine (111 mg, 0.5 mmol, 2.5 equiv), sodium carbonate (31.8 mg, 0.3 mmol, 1.5 equiv) and **1** (93.2 mg, 0.2 mmol, 1 equiv) to afford a colorless oil (80.1 mg, 0.149 mmol, 75% yield). **R$_f$**= 0.53 (hexanes:ethyl acetate= 10:1). **LRMS** (EI, 70 eV): m/z (%): 236(1), 235(8), 234(46), 232(1), 220(1), 123(1), 122(11), 121(100), 112(1), 91(2), 90(1), 78(2), 77(2). **^{1}H NMR** (250 MHz, Chloroform-*d*) δ 7.2 – 7.1 (m, 4H), 6.8 (d, *J* = 3.5 Hz, 2H), 6.8 (d, *J* = 3.5 Hz, 2H), 3.7 (s, 3H), 3.7 (s, 3H), 3.7 – 3.5 (m, 2H), 3.5 (d, *J* = 13.5 Hz, 1H), 3.4 – 3.2 (m, 2H), 2.7 (dd, *J* = 13.7, 7.0 Hz, 1H), 2.6 (dd, *J* = 9.2, 5.6 Hz, 1H), 2.6 – 2.4 (m, 2H), 2.3 (qt, *J* = 13.0, 7.0 Hz, 2H), 1.5 – 1.3 (m, 4H), 1.4 – 1.0 (m, 12H), 0.9 – 0.8 (m, 6H). **^{19}F NMR** (235 MHz, Chloroform-*d*) δ -68.1. **^{13}C NMR** (63 MHz, Chloroform-*d*) δ 158.8, 158.7, 132.0, 131.5, 130.2, 130.0, 127.6 (q, *J* = 290.6 Hz), 113.6, 113.6, 58.9 (q, *J* = 23.8 Hz), 58.4, 55.3, 54.7, 54.1, 51.7, 50.5, 31.9, 31.9, 28.9, 27.2, 26.8, 22.8, 14.2. **IR** (ATR, cm^{-1}) 3061, 3031, 2997, 2955, 2929, 2857, 2836, 1737, 1612, 1510, 1465, 1374, 1300, 1244, 1170, 1153, 1100, 1036, 980, 909, 820, 760, 733, 700, 571, 516. **HRMS** m/z $[M+H]^+$ calculated for $C_{31}H_{48}N_2O_2F_3^+$: 537.3668, found: 537.3671.

*Synthesis of compound **25***

N^1,N^2-dicyclopropyl-3,3,3-trifluoro-N^1,N^2-bis(4-methoxybenzyl)propane-1,2-diamine

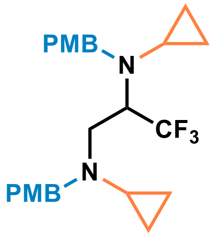

Compound **25** was prepared according to general procedure from *N*-(4-methoxybenzyl)cyclopropanamine (66.5 mg, 0.375 mmol, 2.5 equiv), sodium carbonate (23.8 mg, 0.225 mmol, 1.5 equiv) and **1** (69.9 mg, 0.15 mmol, 1 equiv) to afford a pale yellow oil (56.1 mg, 0.125 mmol, 83% yield). **R$_f$**= 0.45 (hexanes:ethyl acetate= 10:1). **LRMS** (EI, 70 eV): m/z (%): 447(1), 327(1), 191(2), 190(16), 190(8), 176(2), 122(10), 121(100), 91(3), 78(4), 77(4). **^{1}H NMR** (250 MHz, Chloroform-*d*) δ 7.26 – 7.14 (m, 4H), 6.96 – 6.81 (m, 4H), 3.93 – 3.57 (m, 9H), 3.76 (d, *J* = 13.6 Hz, 1H), 3.63 (d, *J* = 13.6 Hz, 1H), 3.03 (dd, *J* = 13.7, 7.1 Hz, 1H), 2.92 (dd, *J* = 13.6, 5.4 Hz, 1H), 2.39 – 2.24 (m, 1H), 2.26 (s, 0H), 1.81 (p, *J* = 5.5 Hz, 1H), 0.57 – 0.20 (m, 8H). **^{19}F NMR** (235 MHz, Chloroform-*d*) δ -67.9. **^{13}C NMR** (63 MHz, Chloroform-*d*) δ 158.85, 158.76, 132.18, 130.81,

130.27, 130.02, 127.43 (q, J = 289.1 Hz), 113.50, 113.43, 60.77 (q, J = 24.2 Hz), 58.68, 55.65, 55.24, 52.27, 37.23, 35.06, 8.46, 7.45, 7.26, 7.15. **IR** (ATR, cm^{-1}) 3090, 3074, 3006, 2952, 2936, 2910, 2836, 1738, 1611, 1585, 1510, 1458, 1442, 1350, 1300, 1242, 1173, 1144, 1106, 1035, 1021, 907, 821, 761, 733, 598, 516. **HRMS** m/z $[M+H]^+$ calculated for $C_{25}H_{32}N_2O_2F_3^+$: 449.2416, found: 449.2406.

*Synthesis of compound **26***

2,2'-((3,3,3-trifluoropropane-1,2-diyl)bis((4-methoxybenzyl)azanediyl))diacetate

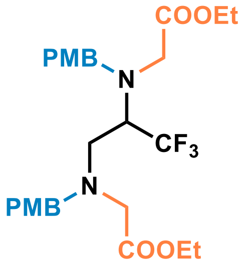

Compound **26** was prepared according to general procedure from ethyl (4-methoxybenzyl)glycinate (167 mg, 0.75 mmol, 2.5 equiv), sodium carbonate (47.7 mg, 0.45 mmol, 1.5 equiv) and **1** (140 mg, 0.3 mmol, 1 equiv) to afford a colorless liquid (128.0 mg, 0.237 mmol, 79% yield). $\mathbf{R_f}$= 0.22 (hexanes:ethyl acetate= 10:1). **LRMS** (EI, 70 eV): m/z (%): 376(2), 222(1), 157(3), 156(32), 155(10), 154(100), 140(3), 139(4), 138(6), 125(2), 119(4), 118(6), 113(2), 111(6), 91(2), 77(3), 75(3). ^{1}**H NMR** (250 MHz, Chloroform-*d*) δ 7.32 (d, J = 8.6 Hz, 2H), 7.15 (d, J = 8.6 Hz, 2H), 6.84 (d, J = 6.4 Hz, 2H), 6.81 (d, J = 6.2 Hz, 2H), 4.22 – 4.03 (m, 4H), 3.92 (d, J = 13.3 Hz, 1H), 3.84 – 3.62 (m, 9H), 3.68 – 3.24 (m, 5H), 3.11 (dd, J = 14.2, 7.9 Hz, 1H), 2.95 (dd, J = 14.0, 4.7 Hz, 1H), 1.32 – 1.17 (m, 6H). ^{19}**F NMR** (235 MHz, Chloroform-*d*) δ -68.56. ^{13}**C NMR** (63 MHz, Chloroform-*d*) δ 171.4, 159.1, 159.0, 130.5, 130.5, 130.3, 130.1, 127.2 (q, J = 290.7 Hz), 113.7, 113.7, 60.6, 60.2, 59.4 (q, J = 24.2 Hz), 57.8, 55.3, 55.2, 53.6, 51.6, 50.4, 14.3, 14.2. **IR** (ATR, cm^{-1}) 3101, 3073, 3061, 3034, 2980, 2958, 2938, 2908, 2837, 1733, 1612, 1585, 1510, 1463, 1443, 1371, 1302, 1244, 1171, 1144, 1097, 1031, 916, 832, 812, 761, 732, 698, 571, 518. **HRMS** m/z $[M+H]^+$ calculated for $C_{27}H_{36}N_2O_6F_3^+$: 541.2525, found: 541.2537.

*Synthesis of compound **27***

N^1,N^2-diallyl-3,3,3-trifluoro- N^1,N^2-bis(4-methoxybenzyl)propane-1,2-diamine

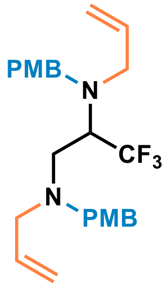

Compound **27** was prepared according to general procedure from *N*-(4-methoxybenzyl)prop-2-en-1-amine **A1** (88.6 mg, 0.5 mmol, 2.5 equiv), sodium carbonate (31.8 mg, 0.3 mmol, 1.5 equiv) and **1** (93.2 mg, 0.2 mmol, 1 equiv) to afford a pale yellow oil (66.9 mg, 0.149 mmol, 75% yield). $\mathbf{R_f}$= 0.47 (hexanes:ethyl acetate= 10:1). **LRMS** (EI, 70 eV): m/z (%): 91(100), 210(79), 211(13), 92(8), 65(8), 181(4), 118(2), 89(2), 212(1), 208(1), 73(1), 109(1), 63(1), 207(1), 397(1), 51(1), 182(1), 165(1), 77(1). ^{1}**H NMR** (250 MHz, Chloroform-*d*) δ 7.25 – 7.08 (m, 4H), 6.87 – 6.70 (m, 4H), 5.93 – 5.59 (m, 2H), 5.23 – 4.98 (m, 4H), 3.75 (s, 3H), 3.74 (s, 3H), 3.72 – 3.63 (m,

2H), 3.58 (d, *J* = 17.8 Hz, 2H), 3.44 (td, *J* = 8.5, 4.3 Hz, 1H), 3.32 (d, *J* = 13.2 Hz, 1H), 3.19 (dd, *J* = 18.9, 6.3 Hz, 1H), 3.06 (dd, *J* = 14.7, 6.0 Hz, 1H), 2.88 (dd, *J* = 6.9, 5.0 Hz, 1H), 2.80 (dd, *J* = 11.4, 5.9 Hz, 1H), 2.57 (dd, *J* = 13.7, 4.3 Hz, 1H). **^{19}F NMR** (235 MHz, Chloroform-*d*) δ -68.3. **^{13}C NMR** (63 MHz, Chloroform-*d*) δ 158.9, 137.0, 135.5, 131.6, 131.0, 130.3, 130.0, 127.5 (q, *J* = 289.8 Hz), 117.9, 117.5, 113.8, 113.7, 58.0, 57.7 (q, *J* = 24.0 Hz), 56.8, 55.3, 53.7, 53.4, 50.5. **IR** (ATR, cm^{-1}) 3075, 3002, 2953, 2936, 2910, 2836, 1738, 1612, 1510, 1463, 1442, 1368, 1300, 1242, 1170, 1149, 1093, 1035, 993, 920, 879, 808, 760, 703, 564, 515. **HRMS** m/z [M+H]$^+$ calculated for $C_{25}H_{32}N_2O_2F_3^+$: 449.2416, found: 449.2421.

*Synthesis of compound **28***

N^1,*N*^{2}-diethyl-3,3,3-trifluoro- *N*^{1},*N*^{2}-di(prop-2-yn-1-yl)propane-1,2-diamine

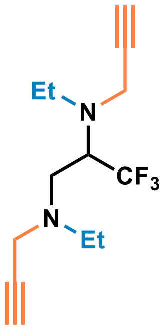

Compound **28** was prepared according to general procedure from *N*-ethylprop-2-yn-1-aminium chloride (89.7 mg, 0.75 mmol, 2.5 equiv), sodium carbonate (127 mg, 0.75 mmol, 4.0 equiv) and **1** (140 mg, 0.3 mmol, 1 equiv) to afford a colorless liquid (29.4 mg, 0.113 mmol, 38% yield). **R$_f$**= 0.34 (hexanes:ethyl acetate= 10:1). **LRMS** (EI, 70 eV): m/z (%): 221(2), 221(1), 164(1), 110(1), 108(1), 97(4), 97(3), 96(100), 69(1), 68(3), 68(3), 66(2), 56(6). **^{1}H NMR** (250 MHz, Chloroform-*d*) δ 3.72 – 3.40 (m, 5H), 2.97 – 2.77 (m, 2H), 2.71 (dd, *J* = 13.9, 4.6 Hz, 2H), 2.61 (q, *J* = 7.2 Hz, 2H), 2.23 (t, *J* = 2.3 Hz, 1H), 2.18 (t, *J* = 2.2 Hz, 1H), 1.10 (t, *J* = 7.0 Hz, 3H), 1.07 (t, *J* = 7.1 Hz, 3H). **^{19}F NMR** (235 MHz, Chloroform-*d*) δ -69.41. **^{13}C NMR** (63 MHz, Chloroform-*d*) δ 127.0 (q, *J* = 289.2 Hz), 80.8, 78.6, 73.1, 72.4, 60.3 (q, *J* = 24.7 Hz), 49.6 (d, *J* = 1.6 Hz), 48.0, 44.3, 41.9, 40.1, 13.9, 12.9 (q, *J* = 1.5 Hz). **IR** (ATR, cm^{-1}) 3308, 2973, 2938, 2849, 1737, 1459, 1435, 1382, 1326, 1296, 1249, 1156, 1097, 1063, 1044, 981, 936, 900, 856, 798, 700, 627, 498. **HRMS** m/z [M+H]$^+$ calculated for $C_{13}H_{20}N_2F_3^+$: 261.1579, found: 261.1580.

*Synthesis of compound **29***

3,3,3-trifluoro-*N*^{1},*N*^{2}-bis(4-methoxybenzyl)-*N*^{1},*N*^{2}-di(prop-2-yn-1-yl)propane-1,2-diamine

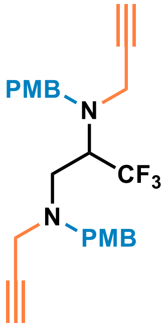

Compound **29** was prepared according to general procedure from *N*-(4-methoxybenzyl)prop-2-yn-1-amine (131 mg, 0.75 mmol, 2.5 equiv), sodium carbonate (47.7 mg, 0.45 mmol, 1.5 equiv) and **1** (140 mg, 0.3 mmol, 1 equiv) to afford a colorless liquid (110.2 mg, 0.248 mmol, 82% yield). **R$_f$**= 0.37 (hexanes:ethyl acetate= 10:1). **LRMS** (EI, 70 eV): m/z (%): 365(1), 323(2), 283(1), 189(3), 188(20), 122(9), 121(100), 91(2), 78(3), 77(2), 77(1). **^{1}H NMR** (250 MHz, Chloroform-*d*) δ 7.28 – 7.08 (m, 4H), 6.75 (d, *J* = 8.3 Hz, 4H), 3.84 (d, *J* = 13.6 Hz, 1H), 3.76 –

3.43 (m, 10H), 3.37 (dd, *J* = 5.8, 2.3 Hz, 2H), 3.26 (s, 2H), 2.91 (dd, *J* = 13.4, 8.9 Hz, 1H), 2.76 (dd, *J* = 13.6, 4.5 Hz, 1H), 2.22 – 2.09 (m, 2H). **^{19}F NMR** (235 MHz, Chloroform-*d*) δ -68.86. **^{13}C NMR** (63 MHz, Chloroform-*d*) δ 159.1, 130.5, 130.2, 130.0, 127.1 (q, *J* = 288.9 Hz), 113.9 (d, *J* = 4.6 Hz), 80.6, 78.2, 73.7, 72.8, 58.7 (q, *J* = 24.8 Hz), 57.6, 55.3, 53.3, 49.5, 41.3, 39.9. **IR** (ATR, cm^{-1}) 3292, 3064, 3034, 3000, 2955, 2935, 2908, 2836, 2725, 1612, 1585, 1510, 1463, 1442, 1367, 1302, 1244, 1171, 1146, 1095, 1034, 981, 903, 875, 831, 811, 761, 699, 632, 515, 428. **HRMS** m/z [M+H]$^+$ calculated for $C_{25}H_{28}N_2O_2F_3^+$: 445.2103, found: 445.2104.

Synthesis of compound ***30***

3,3,3-trifluoro-N^1,N^2-diisobutyl-N^1,N^2-bis(3-phenylprop-2-yn-1-yl)propane-1,2-diamine

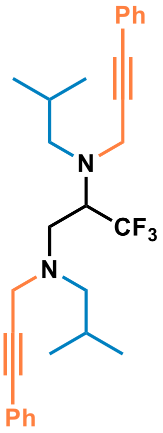

Compound **30** was prepared according to general procedure from *N*-isobutyl-3-phenylprop-2-yn-1-amine (154.8 mg, 0.80 mmol, 2.5 equiv), sodium carbonate (50.9 mg, 0.48 mmol, 1.5 equiv) and **1** (149.1 mg, 0.32 mmol, 1 equiv) to afford a colorless oil (119.4 mg, 0.255 mmol, 80% yield). **R_f**= 0.68 (hexanes:diisopropyl ether= 10:1). **^{1}H NMR** (250 MHz, Chloroform-*d*) δ 7.46 – 7.33 (m, 4H), 7.31 – 7.16 (m, 6H), 3.80 (d, *J* = 17.3 Hz, 1H), 3.75 – 3.55 (m, 4H), 2.96 (dd, *J* = 13.8, 7.5 Hz, 1H), 2.83 (dd, *J* = 13.7, 4.9 Hz, 1H), 2.66 (dd, *J* = 12.9, 7.1 Hz, 1H), 2.51 (dd, *J* = 12.9, 7.6 Hz, 1H), 2.34 (td, *J* = 12.3, 11.1, 7.3 Hz, 2H), 1.80 (dhept, *J* = 20.1, 6.7 Hz, 2H), 0.98 – 0.85 (m, 12H). **^{19}F NMR** (235 MHz, Chloroform-*d*) δ -68.95. **^{13}C NMR** (63 MHz, Chloroform-*d*) δ 131.9, 131.7, 128.4, 128.1, 127.2 (q, *J* = 289.1 Hz), 123.5, 123.4, 86.6, 85.7, 84.5, 84.4, 62.8, 61.4 (q, *J* = 24.7 Hz), 59.2, 51.4, 42.9, 41.4, 26.7, 26.2, 20.9, 20.7, 20.5. **IR** (ATR, cm^{-1}) 3081, 3060, 3034, 3022, 2955, 2928, 2905, 2870, 2824, 2746, 2725, 1598, 1489, 1468, 1442, 1385, 1365, 1327, 1251, 1156, 1103, 1044, 1029, 946, 913, 868, 754, 689, 600, 526, 509, 628, 596, 566, 540, 510, 481, 461. **HRMS** m/z [M+H]$^+$ calculated for $C_{29}H_{36}N_2F_3^+$: 469.2831, found: 469.2831.

*Synthesis of compound **31***

N[1],*N*[2]-dicyclohexyl-3,3,3-trifluoro-*N*[1],*N*[2]-bis(3-phenylprop-2-yn-1-yl)propane-1,2-diamine

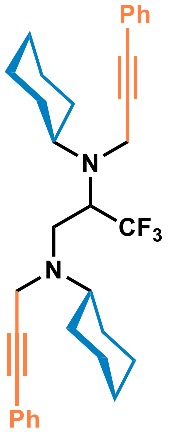

Compound **31** was prepared according to general procedure from *N*-(3-phenylprop-2-yn-1-yl)cyclohexanamine (160 mg, 0.75 mmol, 2.5 equiv), sodium carbonate (47.7 mg, 0.45 mmol, 1.5 equiv) and **1** (140 mg, 0.3 mmol, 1 equiv) to afford a colorless liquid (143.3 mg, 0.275 mmol, 92% yield). **R$_f$**= 0.63 (hexanes:diisopropyl ether= 10:1). **^{1}H NMR** (250 MHz, Chloroform-*d*) δ 7.41 – 7.24 (m, 4H), 7.23 – 7.03 (m, 6H), 3.76 (s, 2H), 3.73 – 3.56 (m, 3H), 2.96 (d, *J* = 6.0 Hz, 2H), 2.84 – 2.67 (m, 1H), 2.50 (t, *J* = 9.7, 7.8 Hz, 1H), 1.86 (q, *J* = 11.2, 10.7 Hz, 4H), 1.77 – 1.60 (m, 4H), 1.50 (d, *J* = 11.3 Hz, 2H), 1.43 – 0.87 (m, 10H). **^{19}F NMR** (235 MHz, Chloroform-*d*) δ -69.58 **^{13}C NMR** (63 MHz, Chloroform-*d*) δ 131.7, 128.3, 128.3, 128.0, 127.5 (q, *J* = 290.4 Hz), 123.7, 123.6, 88.2, 87.1, 84.9, 83.6, 62.3, 59.4 (q, *J* = 24.5 Hz), 58.3, 47.0, 40.0, 36.6, 32.6, 31.3, 30.5, 30.2, 26.3, 26.1, 26.1, 26.0. **IR** (ATR, cm^{-1}) 3081, 3057, 3033, 3020, 2927, 2853, 2667, 1598, 1489, 1443, 1382, 1346, 1252, 1154, 1107, 1071, 1028, 1001, 947, 913, 892, 873, 846, 753, 689, 604, 526, 510. **HRMS** m/z [M+H]$^+$ calculated for $C_{33}H_{40}N_2F_3^+$: 521.3144, found: 521.3142.

*Synthesis of compound **32***

Di-*tert*-butyl 3,3'-((3,3,3-trifluoropropane-1,2-diyl)bis(phenethylazanediyl))bis(azetidine-1-carboxylate)

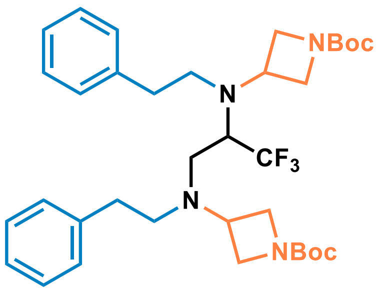

Compound **32** was prepared according to general procedure from *tert*-butyl 3-(phenethylamino)azetidine-1-carboxylate (138 mg, 0.5 mmol, 2.5 equiv), sodium carbonate (31.8 mg, 0.3 mmol, 1.5 equiv) and **1** (93.2 mg, 0.2 mmol, 1 equiv) to afford a pale yellow oil (85.9 mg, 0.133 mmol, 66% yield). **R$_f$**= 0.48 (hexanes:ethyl acetate= 7:3). **LRMS** (EI, 70 eV): m/z (%): 362(24), 361(100), 271(5), 269(10), 257(5), 189(10), 165(12), 163(15), 160(5), 134(6), 106(8), 105(89), 103(6), 91(12), 79(11), 77(9), 56(6). **^{1}H NMR** (250 MHz, Chloroform-*d*) δ 7.3 – 7.0 (m, 10H), 4.0 – 3.8 (m, 6H), 3.7 – 3.5 (m, 4H), 3.3 – 3.2 (m, 1H), 3.2 – 2.9 (m, 2H), 2.8 – 2.5 (m, 8H), 1.4 (s, 9H), 1.4 (s, 9H). **^{19}F NMR** (235 MHz, Chloroform-*d*) δ -69.5. **^{13}C NMR** (63 MHz, Chloroform-*d*) δ 156.3, 156.1, 139.5, 139.2, 128.8, 128.7, 128.6, 128.5, 126.8 (q, *J* = 290.4 Hz), 126.5, 126.4, 79.7, 79.6, 60.7 (q, *J* = 24.0 Hz), 55.1, 53.7, 51.8, 49.4, 47.9, 47.0, 36.4, 33.2, 28.4, 28.4. **IR** (ATR, cm^{-1}) 3087, 3064, 3027, 2976, 2965, 2934, 2884, 2247, 1690, 1477, 1455, 1391, 1365, 1295, 1253, 1157, 1117, 1031,

907, 859, 773, 727, 698, 647, 569, 503. **HRMS** m/z $[M+H]^+$ calculated for $C_{35}H_{50}N_4O_4F_3^+$: 647.3784, found: 647.3795.

Synthesis of compound ***33***

1,1'-(3,3,3-trifluoropropane-1,2-diyl)bis(3-((*tert*-butyldimethylsilyl)oxy)azetidine)

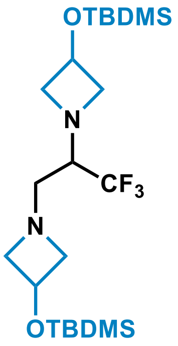

Compound **33** was prepared according to general procedure from 3-((*tert*-butyldimethylsilyl)oxy)azetidine (141 mg, 0.75 mmol, 2.5 equiv), sodium carbonate (47.7 mg, 0.45 mmol, 1.5 equiv) and **1** (140 mg, 0.3 mmol, 1 equiv) to afford a colorless oil (92.8 mg, 0.198 mmol, 66% yield). $\mathbf{R_f}$= 0.33 (hexanes:ethyl acetate= 10:1). **LRMS** (EI, 70 eV): m/z (%): 453(2), 453(1), 411(3), 202(9), 201(17), 201(14), 200(100), 200(82), 158(2), 144(2), 128(2), 115(2), 103(6), 102(4), 101(37), 77(2), 75(5), 74(2), 73(16), 60(2), 59(11), 57(1). **^{1}H NMR** (250 MHz, Chloroform-*d*) δ 4.4 (dp, *J* = 12.4, 6.2 Hz, 2H), 3.7 (dt, *J* = 12.0, 4.9 Hz, 4H), 3.1 (t, *J* = 6.6 Hz, 1H), 3.0 (t, *J* = 6.8 Hz, 1H), 2.7 (td, *J* = 6.4, 3.1 Hz, 2H), 2.7 – 2.3 (m, 3H), 0.8 (s, 18H), -0.0 (s, 12H). **^{19}F NMR** (235 MHz, Chloroform-*d*) δ -72.1. **^{13}C NMR** (63 MHz, Chloroform-*d*) δ 125.9 (q, *J* = 282.8 Hz), 65.5, 65.5 (q, *J* = 25.7 Hz), 65.0, 62.7, 62.1, 57.0 (d, *J* = 2.0 Hz), 25.9, 18.1, -4.9. **IR** (ATR, cm^{-1}) 2952, 2931, 2888, 2857, 2836, 2800, 2773, 2744, 2712, 1473, 1377, 1253, 1208, 1184, 1161, 1127, 988, 939, 881, 834, 800, 774, 698, 671, 580, 513. **HRMS** m/z $[M+H]^+$ calculated for $C_{21}H_{44}N_2O_2F_3Si_2^+$: 469.2893, found: 469.2892.

Synthesis of compound ***34***

Dimethyl 1,1'-(3,3,3-trifluoropropane-1,2-diyl)bis(azetidine-3-carboxylate)

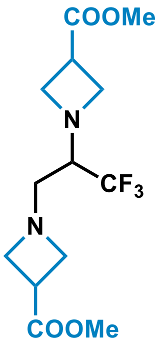

Compound **34** was prepared according to the general procedure 3-(methoxycarbonyl)azetidin-1-ium trifluoromethanesulfonate (137 mg, 0.5 mmol, 2.5 equiv), sodium carbonate (84.8 mg, 0.8 mmol, 4.0 equiv) and **1** (93.2 mg, 0.2 mmol, 1 equiv) to afford a pale yellow oil (47.9 mg, 0.148 mmol, 74% yield). $\mathbf{R_f}$= 0.28 (hexanes:ethyl acetate= 7:3). **LRMS** (EI, 70 eV): m/z (%): 293(5), 239(3), 196(2), 129(7), 128(100), 110(2), 69(1), 68(5), 59(4), 55(11). **^{1}H NMR** (250 MHz, Chloroform-*d*) δ 3.76 – 3.59 (m, 8H), 3.63 – 3.49 (m, 3H), 3.47 (t, *J* = 7.3 Hz, 1H), 3.41 – 3.16 (m, 4H), 2.80 – 2.59 (m, 1H), 2.62 – 2.47 (m, 2H). **^{19}F NMR** (235 MHz, Chloroform-*d*) δ -72.00. **^{13}C NMR** (63 MHz, Chloroform-*d*) δ 173.3, 173.1, 125.6 (q, *J* = 282.9 Hz), 64.5 (q, *J* = 25.8 Hz), 57.7, 56.9, 56.0 (q, *J* = 2.1 Hz), 55.3, 52.0, 34.5, 33.7. **IR** (ATR, cm^{-1}) 2955, 2846, 2771, 1733, 1480, 1438, 1367, 1306, 1259, 1201, 1153, 1123, 1048, 960, 913, 865, 733, 679, 584, 505. **HRMS** m/z $[M+H]^+$ calculated for $C_{13}H_{20}N_2O_4F_3^+$: 325.1375, found: 325.1380.

*Synthesis of compound **35***

1,1'-(3,3,3-trifluoropropane-1,2-diyl)bis(pyrrolidin-1-ium) 2,2,2-trifluoroacetate

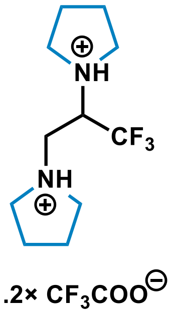

Compound **35** was prepared according to the general procedure from pyrrolidine (63 µL, 0.75 mmol, 2.5 equiv), sodium carbonate (47.7 mg, 0.45 mmol, 1.5 equiv) and **1** (140 mg, 0.3 mmol, 1 equiv) and isolated as the corresponding bisamminium trifluoroacetate salt, which is a pale yellow syrup (71.5 mg, 0.154 mmol, 51% yield). **R**$_f$= 0.25 (hexanes:ethyl acetate= 7:3). **LRMS** (EI, 70 eV): m/z (%): 84(100), 55(9), 85(6), 110(2), 54(2), 56(2), 96(2), 69(1), 83(1), 60(1), 82(1), 70(1), 152(1). **^{1}H NMR** (250 MHz, Methylenechloride-*d*$_2$) δ 11.26 (bs, 2H), 4.10 – 3.10 (m, 6H), 2.92 (dq, J = 38.7, 7.0 Hz, 4H), 2.23 – 1.97 (m, 4H), 1.79 (d, J = 6.4 Hz, 4H). **^{19}F NMR** (235 MHz, Methylenechloride-*d*$_2$) δ -66.21 (s, 3F), -74.39 (s, 6F). **^{13}C NMR** (63 MHz, Methylenechloride-*d*$_2$) δ 126.1 (q, J = 294.0 Hz), 114.3 (q, J = 291.1 Hz), 58.8 (q, J = 26.7 Hz), 56.0, 52.5 (q, J = 2.4 Hz), 48.9, 24.4, 23.5. **IR** (ATR, cm^{-1}) 2971, 2946, 2927, 2856, 1740, 1672, 1458, 1365, 1139, 1113, 876, 835, 795, 720, 703, 597, 518, 428. **HRMS** m/z [M+H]$^+$ calculated for $C_{11}H_{20}N_2F_3^+$: 237.1579, found: 237.1582.

*Synthesis of compound **36***

1,1'-(3,3,3-trifluoropropane-1,2-diyl)dipiperidine

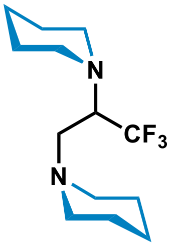

Compound **36** was prepared according to general procedure from piperidine (103 µL, 0.75 mmol, 2.5 equiv), sodium carbonate (47.7 mg, 0.45 mmol, 1.5 equiv) and **1** (140 mg, 0.3 mmol, 1 equiv) to afford a colorless oil (52.9 mg, 0.200 mmol, 67% yield). **R**$_f$= 0.33 (hexanes:ethyl acetate= 10:1). **LRMS** (EI, 70 eV): m/z (%): 293(5), 239(3), 196(2), 129(7), 128(100), 110(2), 69(1), 68(5), 59(4), 55(11). **^{1}H NMR** (250 MHz, Chloroform-*d*) δ 3.16 (ddq, J = 12.5, 9.0, 4.5, 3.6 Hz, 1H), 2.90 – 2.57 (m, 5H), 2.56 – 2.32 (m, 5H), 1.67 – 1.22 (m, 12H). **^{19}F NMR** (235 MHz, Chloroform-*d*) δ -68.79. **^{13}C NMR** (63 MHz, Chloroform-*d*) δ 127.2 (q, J = 290.6 Hz), 64.3 (q, J = 23.8 Hz), 55.1 (q, J = 1.7 Hz), 54.8, 51.0, 27.1, 26.3, 24.8, 24.5. **IR** (ATR, cm^{-1}) 2932, 2854, 2823, 2813, 2782, 1469, 1442, 1381, 1356, 1319, 1309, 1292, 1253, 1201, 1151, 1134, 1099, 1078, 1065, 1041, 1018, 995, 963, 906, 868, 844, 784, 764, 710, 699, 630, 593, 457. **HRMS** m/z [M+H]$^+$ calculated for $C_{13}H_{24}N_2F_3^+$: 265.1892, found: 265.1892.

Synthesis of compound ***37***

1,1'-(3,3,3-trifluoropropane-1,2-diyl)bis(3-((*tert*-butyldimethylsilyl)oxy)piperidine)

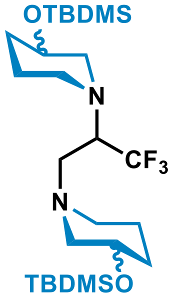

Compound **37** was prepared according to general procedure from 3-((*tert*-butyldimethylsilyl)oxy)piperidine (162 mg, 0.75 mmol, 2.5 equiv), sodium carbonate (47.7 mg, 0.45 mmol, 1.5 equiv) and **1** (140 mg, 0.3 mmol, 1 equiv) to afford a colorless oil (134 mg, 0.255 mmol, 85% yield). **R_f**= 0.65 (hexanes:ethyl acetate= 10:1). **LRMS** (EI, 70 eV): m/z (%): 467(1), 310(1), 231(1), 230(6), 229(23), 228(100), 226(1), 190(1), 170(1), 142(1), 128(1), 115(1), 112(1), 106(2), 101(1), 96(4), 89(1), 77(1), 75(2), 74(1), 73(11), 59(1). **^{1}H NMR** (250 MHz, Chloroform-*d*) 3.77 – 3.49 (m, 2H), 3.35 – 3.10 (m, 1H), 3.06 – 2.25 (m, 8H), 2.13 – 1.76 (m, 4H), 1.74 – 1.34 (m, 4H), 1.32 – 1.07 (m, 2H), 0.88 (s, 18H), 0.19 – -0.08 (m, 12H). **^{19}F NMR** (235 MHz, Chloroform-*d*) δ -68.97, -69.44, -69.48, -69.52. **^{13}C NMR** (63 MHz, Chloroform-*d*) δ 129.3, 129.3, 129.2, 124.7, 124.7, 124.6, 69.1, 69.0, 68.9, 68.7, 68.5, 68.5, 64.5, 64.1, 64.1, 63.8, 63.7, 63.7, 63.4, 63.3, 63.0, 62.9, 62.7, 62.1, 62.1, 61.6, 61.3, 58.9, 58.7, 57.6, 56.5, 54.3, 54.1, 54.0, 53.8, 53.7, 53.4, 53.4, 51.3, 50.3, 49.5, 49.4, 34.5, 34.4, 34.1, 34.1, 26.0, 26.0, 18.3, 18.3, 18.3, -4.5, -4.5. **IR** (ATR, cm^{-1}) 2931, 2888, 2857, 2826, 2802, 2710, 1472, 1388, 1361, 1320, 1253, 1164, 1093, 1039, 987, 939, 832, 773, 702, 668, 466, 394. **HRMS** m/z $[M+H]^+$ calculated for $C_{25}H_{52}N_2O_2F_3Si_2^+$: 525.3519, found: 525.3508.

Synthesis of compound ***38***

1,1'-(3,3,3-trifluoropropane-1,2-diyl)bis(3-(((*tert*-butyldimethylsilyl)oxy)methyl)piperidine)

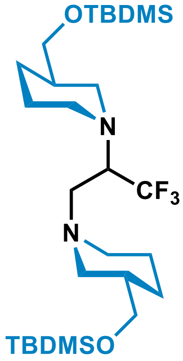

Compound **38** was prepared according to general procedure from 3-(((*tert*-butyldimethylsilyl)oxy)methyl)piperidine (172 mg, 0.75 mmol, 2.5 equiv), sodium carbonate (47.7 mg, 0.45 mmol, 1.5 equiv) and **1** (140 mg, 0.3 mmol, 1 equiv) to afford a colorless oil (126 mg, 0.228 mmol, 76% yield). **R_f**= 0.55 (hexanes:ethyl acetate= 10:1). **LRMS** (EI, 70 eV): m/z (%):**^{1}H NMR** (250 MHz, Chloroform-*d*) 3.54 – 3.30 (m, 4H), 3.21 (p, J = 8.4 Hz, 1H), 3.08 – 2.36 (m, 8H), 2.06 (t, J = 10.2 Hz, 1H), 1.95 – 1.35 (m, 9H), 0.89 (s, 20H), 0.03 (s, 12H). **^{19}F NMR** (235 MHz, Chloroform-*d*) δ -68.87, -68.96, -69.00, -69.12. **^{13}C NMR** (63 MHz, Chloroform-*d*) δ 127.2 (q, J = 290.5 Hz), 64.6 (q, J = 23.7, 23.2 Hz), 64.0 (q, J = 24.0 Hz), 57.8, 57.7, 57.4, 55.0, 54.9, 54.8, 54.8, 54.6, 54.5, 52.8, 51.9, 51.8, 50.3, 50.1, 39.9, 39.8, 39.3, 39.3, 39.1, 39.1, 27.5, 27.2, 27.1, 26.1, 26.0, 25.9, 25.7, 25.2, 25.2, 25.1, 18.5, 18.4, -5.2, -5.3. **IR** (ATR, cm^{-1}) 2951, 2929, 2904, 2856, 2803, 2788, 2746, 2714, 1470, 1388, 1361, 1253, 1174, 1156, 1097, 1005, 939, 909, 834,

773, 734, 666, 397. **HRMS** m/z $[M+H]^+$ calculated for $C_{27}H_{56}N_2O_2F_3Si_2^+$: 553.3832, found: 553.3842.

Synthesis of compound ***39***

1,1'-(3,3,3-trifluoropropane-1,2-diyl)bis(4-benzylpiperidine)

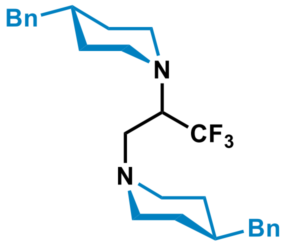

Compound **39** was prepared according to general procedure from 4-benzylpiperidine (88 μL, 0.5 mmol, 2.5 equiv), sodium carbonate (31.8 mg, 0.3 mmol, 1.5 equiv) and **1** (93.2 mg, 0.2 mmol, 1 equiv) to afford a pale yellow oil (79.1 mg, 0.178 mmol, 89% yield). $\mathbf{R_f}$= 0.23 (hexanes:ethyl acetate= 10:1). **LRMS** (EI, 70 eV): m/z (%): 256(1), 190(1), 189(15), 188(100), 186(1), 117(2), 96(2), 92(1), 91(14), 82(1), 70(2), 68(1), 67(1), 55(2). **^{1}H NMR** (250 MHz, Chloroform-*d*) δ 7.3 – 7.0 (m, 10H), 4.0 – 3.8 (m, 6H), 3.7 – 3.5 (m, 4H), 3.3 – 3.2 (m, 1H), 3.2 – 2.9 (m, 2H), 2.8 – 2.5 (m, 8H), 1.4 (s, 9H), 1.4 (s, 9H). **^{19}F NMR** (235 MHz, Chloroform-*d*) δ -68.79. **^{13}C NMR** (63 MHz, Chloroform-*d*) δ 140.8, 140.7, 129.2, 128.3, 128.3, 126.2 (q, J = 291.0 Hz), 126.0, 125.9, 63.7 (q, J = 24.2 Hz), 54.4, 54.1, 51.3, 48.8, 43.3, 43.2, 38.2, 37.8, 33.3, 33.1, 32.2, 32.1. **IR** (ATR, cm^{-1}) 3084, 3063, 3027, 2917, 2846, 2832, 2785, 2751, 2681, 1494, 1468, 1453, 1391, 1347, 1306, 1259, 1158, 1140, 1099, 1056, 1031, 973, 910, 862, 744, 698, 647, 613, 591, 574, 509, 444. **HRMS** m/z $[M+H]^+$ calculated for $C_{27}H_{36}N_2F_3^+$: 445.2831, found: 445.2832.

Synthesis of compound ***40***

4,4'-(3,3,3-trifluoropropane-1,2-diyl)bis(morpholin-4-ium) trifluoroacetate

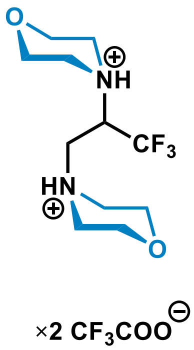

Compound **40** was prepared according to general procedure from morpholine (66 μL, 0.75 mmol, 2.5 equiv), sodium carbonate (47.7 mg, 0.45 mmol, 1.5 equiv) and **1** (140 mg, 0.3 mmol, 1 equiv) and isolated as the corresponding bisamminium trifluoroacetate salt, which is a colorless syrup (120 mg, 0.242 mmol, 81% yield). $\mathbf{R_f}$= 0.45 (ethyl acetate). **LRMS** (EI, 70 eV): m/z (%): 100(100), 56(18), 101(9), 70(6), 55(5), 54(4), 124(3), 72(3), 74(2), 57(2), 98(2), 123(2), 168(2). **^{1}H NMR** (250 MHz, Methylenechloride-d_2) δ 9.53 (s, 2H), 3.98 (s, 4H), 3.91 – 3.75 (m, 2H), 3.73 – 3.52 (m, 4H), 3.51 – 3.07 (m, 4H), 3.07 – 2.87 (m, 2H), 2.80 – 2.57 (m, 2H). **^{19}F NMR** (235 MHz, Methylenechloride-d_2) -65.33 (s, 3F), -74.44 (s, 6F). **^{13}C NMR** (63 MHz, Methylenechloride-d_2) 161.3 (q, J = 38.1 Hz), 125.7 (q, J = 293.0 Hz), 114.0 (q, J = 289.8, 286.9 Hz), 67.5, 64.1, 62.0 (q, J = 26.2 Hz), 53.3 (q, J = 2.8 Hz), 49.8. **IR** (ATR, cm^{-1}) 3458, 3305, 3020, 2968, 2929, 2863, 2708, 2576, 2473, 1777, 1709, 1672, 1458, 1367, 1304, 1248, 1158,

1139, 1110, 1051, 1018, 984, 950, 923, 909, 869, 854, 835, 797, 753, 720, 706, 639, 518, 482, 447, 428, 387. **HRMS** m/z $[M+H]^+$ calculated for $C_{11}H_{20}N_2O_2F_3^+$: 269.1477, found: 269.1482.

*Synthesis of compound **41***

4,4'-(3,3,3-trifluoropropane-1,2-diyl)bis(1-benzylpiperazine)

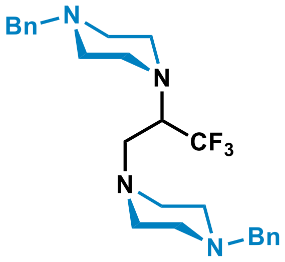

Compound **41** was prepared according to general procedure from morpholine (132 mg, 0.75 mmol, 2.5 equiv), sodium carbonate (47.7 mg, 0.45 mmol, 1.5 equiv) and **1** (140 mg, 0.3 mmol, 1 equiv) to afford a colorless syrup (61.1 mg, 0.137 mmol, 46% yield). $\mathbf{R_f}$= 0.30 (hexanes:ethyl acetate= 7:3). **LRMS** (EI, 70 eV): m/z (%): 447(1), 446(5), 190(14), 189(100), 187(2), 146(5), 99(2), 98(3), 97(5), 92(4), 91(45), 83(2), 70(8), 65(2), 56(3). ^{1}**H NMR** (250 MHz, Chloroform-*d*) 7.38 – 7.05 (m, 10H), 3.42 (d, *J* = 6.4 Hz, 4H), 3.13 (td, *J* = 8.7, 3.3 Hz, 1H), 2.90 – 2.18 (m, 18H). ^{19}**F NMR** (235 MHz, Chloroform-*d*) δ -69.19. ^{13}**C NMR** (63 MHz, Chloroform-*d*) δ 138.1, 138.0, 129.3, 129.3, 128.3, 128.3, 127.2, 127.2, 126.8 (q, *J* = 289.9 Hz), 63.4 (q, *J* = 24.4 Hz), 63.1, 63.1, 54.0, 53.4, 53.2, 49.5. **IR** (ATR, cm^{-1}) 3108, 3085, 3063, 3029, 3003, 2935, 2877, 2809, 2768, 2695, 2681, 1494, 1455, 1367, 1350, 1314, 1262, 1150, 1130, 1105, 1073, 1029, 1010, 909, 864, 825, 732, 696, 642, 605, 580, 455, 407. **HRMS** m/z $[M+H]^+$ calculated for $C_{25}H_{34}N_4F_3^+$: 447.2736, found: 447.2746.

*Synthesis of compound **42***

4,4'-(3,3,3-trifluoropropane-1,2-diyl)bis(1-(pyridin-2-yl)piperazine)

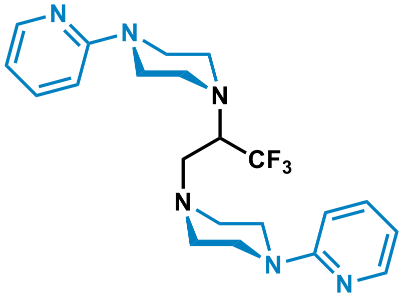

Compound **42** was prepared according to general procedure from 1-(pyridin-2-yl)piperazine (57.1 µL, 0.375 mmol, 2.5 equiv), sodium carbonate (23.8 mg, 0.225 mmol, 1.5 equiv) and **1** (70 mg, 0.15 mmol, 1 equiv) to afford a pale yellow oil (55.2 mg, 0.131 mmol, 88% yield). $\mathbf{R_f}$= 0.28 (hexanes:ethyl acetate= 7:3). **LRMS** (EI, 70 eV): m/z (%): 420(3), 177(12), 176(100), 147(21), 133(5), 121(28), 119(7), 107(11), 95(5), 82(5), 79(9), 78(11), 56(6). ^{1}**H NMR** (250 MHz, Chloroform-*d*) 8.17 (dd, *J* = 4.9, 1.9 Hz, 2H), 7.53 – 7.36 (m, 2H), 6.69 – 6.53 (m, 4H), 3.63 – 3.25 (m, 9H), 3.09 – 2.74 (m, 5H), 2.76 – 2.47 (m, 5H). ^{19}**F NMR** (235 MHz, Chloroform-*d*) δ -69.33. ^{13}**C NMR** (63 MHz, Chloroform-*d*) δ 159.6 (d, *J* = 7.8 Hz), 148.0 (d, *J* = 1.6 Hz), 137.6, 126.6 (q, *J* = 289.1 Hz), 107.2 (d, *J* = 4.3 Hz), 63.4 (q, *J* = 24.8 Hz), 54.0 (q, *J* = 2.0 Hz), 53.3, 49.6, 46.3, 45.4. **IR** (ATR, cm^{-1}) 3097, 3050, 3005, 2936, 2888, 2836, 2756, 2690, 1592, 1563, 1479, 1455,

1435, 1380, 1337, 1312, 1241, 1154, 1106, 1024, 1007, 980, 937, 907, 872, 771, 727, 702, 645, 622, 523, 458, 407. **HRMS** m/z $[M+H]^+$ calculated for $C_{21}H_{28}N_6F_3^+$: 421.2328, found: 421.2337.

Synthesis of compound ***43***

3,3'-(3,3,3-trifluoropropane-1,2-diyl)bis(3-azabicyclo[3.2.2]nonane)

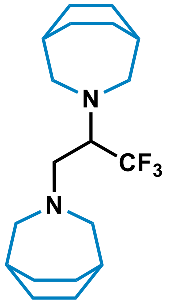

Compound **43** was prepared according to general procedure from 3-azabicyclo[3.2.2]nonane (125 mg, 0.4 mmol, 2.5 equiv), sodium carbonate (63.6 mg, 0.6 mmol, 1.5 equiv) and **1** (186 mg, 0.4 mmol, 1 equiv) to afford a white solid (80.5 mg, 0.234 mmol, 58% yield). **R$_f$**= 0.75 (hexanes:diisopropyl ether= 10:1). **M.p.=** 69-72 °C. **LRMS** (EI, 70 eV): m/z (%): 186(1), 139(10), 138(100), 112(2), 106(1), 95(1), 95(1), 93(1), 91(1), 81(1), 81(1), 79(3), 77(2), 73(1), 68(1), 67(4), 67(3), 58(14), 55(4). **^{1}H NMR** (250 MHz, Chloroform-*d*) 3.30 (dd, *J* = 8.6, 5.5 Hz, 1H), 2.94 (qd, *J* = 11.4, 3.8 Hz, 7H), 2.62 (dddd, *J* = 35.2, 23.9, 13.4, 6.3 Hz, 11H), 1.90 (s, 5H), 1.71 (dd, *J* = 25.8, 5.3 Hz, 14H), 1.62 – 1.54 (m, 17H). **^{19}F NMR** (235 MHz, Chloroform-*d*) δ -69.56. **^{13}C NMR** (63 MHz, Chloroform-*d*) δ 127.6 (q, *J* = 291.2, 290.7 Hz), 63.8 (q, *J* = 23.9 Hz), 63.3, 59.4, 55.3 (q, *J* = 1.5 Hz), 31.6, 30.7, 25.9, 25.8, 25.7, 25.7. **IR** (ATR, cm^{-1}) 2925, 2900, 2857, 2812, 2793, 2758, 2715, 2677, 1451, 1402, 1387, 1364, 1323, 1292, 1263, 1200, 1177, 1150, 1136, 1122, 1095, 1042, 1011, 983, 957, 920, 875, 864, 852, 839, 805, 757, 713, 661, 590, 519, 484, 475. **HRMS** m/z $[M+H]^+$ calculated for $C_{19}H_{32}N_2F_3^+$: 345.2525, found: 345.2525.

Supplementary Figure 6: Unsuccessful substrates under conditions of homodiamination procedure

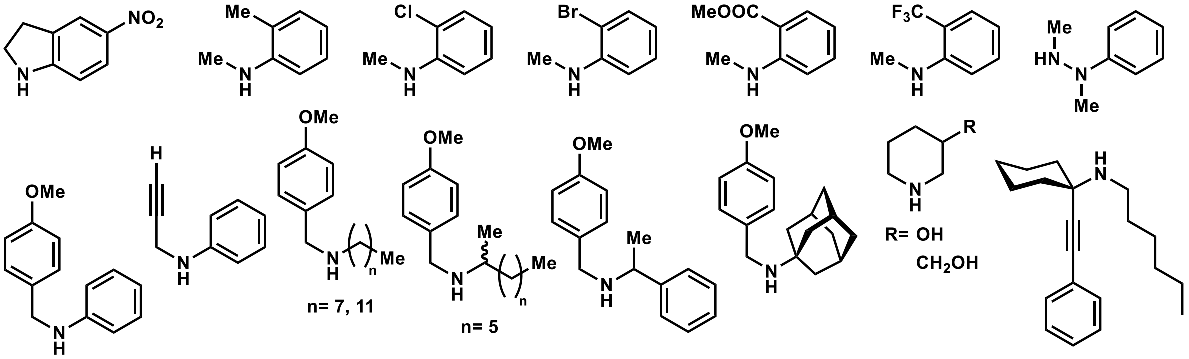

Optimization of the reaction conditions for heterodiamination

An 8 mL test tube was charged with stirring bar, **1** (23.3 mg, 0.05 mmol), solvent (250 or 500 μL/0.05 mmol) and sodium carbonate (0-7.9 mg, 0-0.075 mmol, 0-1.5 equiv) according to **Supplementary Table 2**. To a stirred mixture, *N*-methyl-1-(naphthalen-1-yl)methanamine (1.0 equiv, neat or in corresponding solvent at 0.1-0.2 M concentration) was added via syringe in one portion or by syringe pump over the indicated time (t_1) and at temperature (T_1). After completion of addition, reaction mixture was stirred for indicated time (t_2) at the same temperature (T_1). Pyrrolidine (1.0-2.5 equiv) was added in one portion and the temperature was maintained at or allowed to reach T_2. Aliquots were taken after the indicated time (t_3) and analysed by GC-MS.

Supplementary Figure 7: Reaction scheme of heterodiamination procedure

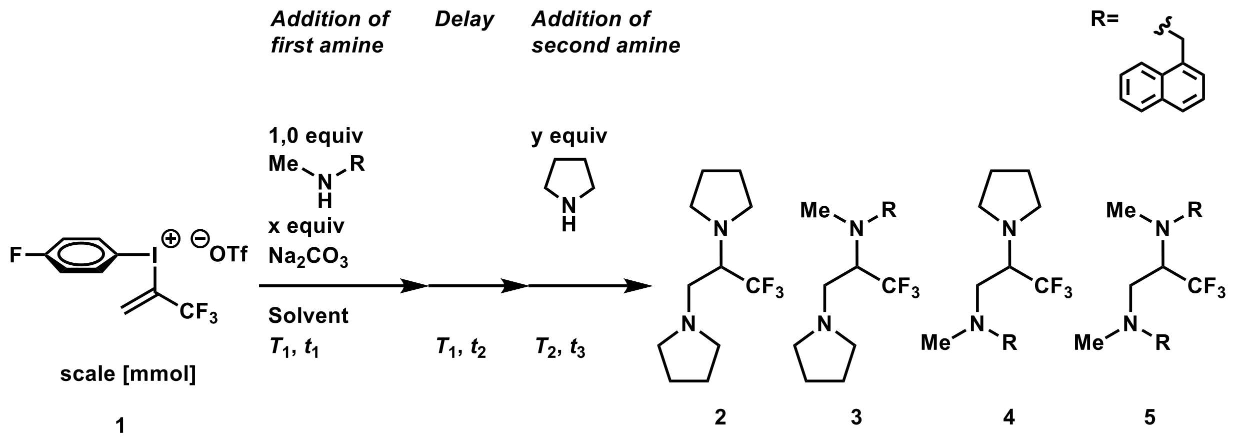

Supplementary Table 2: Optimization of reaction conditions for heterodiamination

#	Scale [mmol]	Na_2CO_3 [x equiv]	Pyrrolidine [y equiv]	c_{final} [mol/L]	Solvent	T_1 [°C]	T_2 [°C]	Medium of amine	Mode of addition	t_1 addition of amine [min]	t_2 delay [min]	t_3 [h; min]	GC-MS conversions [%] *(isolated yield)* 2	3	4	5
1	0.05	1.5	1.5	0.1	CH_2Cl_2	-78	25	neat	one portion	0	30	*16 h*	10	**57**	0	33
2	0.05	0.0	1.5	0.1	CH_2Cl_2	-78	25	neat	one portion	0	30	*16 h*	11	**64**	0	25
3	0.05	0.0	1.5	0.1	CH_2Cl_2	-78	25	CH_2Cl_2 sol.	one portion	0	30	*16 h*	15	**49**	0	36
4	0.05	0.0	1.5	0.1	CH_2Cl_2	-78	25	CH_2Cl_2 sol.	one portion	0	30	*16 h*	15	**58**	0	26
5	0.05	1.5	1.5	0.1	MeCN	25	25	neat	one portion	0	30	60	0	**51**	0	49
6	0.05	1.5	1.0	0.1	MeCN	25	25	neat	one portion	0	30	60	0	**53**	0	47

7	0.05	1.5	1.5	0.1	CH_2Cl_2	25	25	CH_2Cl_2 sol.	syringe pump	5	0	30	68	**7**	0	21
8	0.05	1.5	1.5	0.1	MeCN	25	25	MeCN sol.	syringe pump	5	0	30	20	**45**	0	35
9	0.05	0.0	2.5	0.1	CH_2Cl_2	25	25	CH_2Cl_2 sol.	one portion	0	5	30	53	**15**	0	39
10	0.05	0.0	2.5	0.1	MeCN	25	25	MeCN sol.	one portion	0	5	30	16	**75**	0	9
11	0.05	0.0	2.5	0.05	MeCN	25	25	MeCN sol.	one portion	0	10	30	16	**75**	0	9
12	0.05	0.0	2.5	0.05	MeCN	25	25	MeCN sol.	one portion	0	15	30	13	**79**	0	8
13	0.05	0.0	2.5	0.05	MeCN	25	25	MeCN sol.	syringe pump	10	5	30	0	**35**	0	65
14	0.05	0.0	2.5	0.05	MeCN	25	25	MeCN sol.	one portion	0	30	30	10	**81**	0	9
15	0.05	0.0	2.5	0.05	MeCN	0	0	MeCN sol.	one portion	0	30	30	15	**77**	0	8
16	0.05	0.0	2.5	0.05	MeCN	-20	-20	MeCN sol.	one portion	0	30	30	4	**93**	0	2
17	0.05	0.0	2.5	0.05	MeCN	-20	-20	MeCN sol.	syringe pump	30	15	30	3	**92**	0	5
18	0.15	0.0	2.5	0.05	MeCN	-20	-20	MeCN sol.	one portion	0	30	30	11	**82** ***(59)***	0	7
19	0.15	0.0	2.5	0.05	MeCN	-20	-20	MeCN sol.	syringe pump	30	15	30	0	**96** ***(76)***	0	4

Study of reaction mechanism by ^{19}F-NMR spectroscopy

A 5 mm O.D. NMR tube was charged with (4-fluorophenyl)(3,3,3-trifluoroprop-1-en-2-yl)iodonium trifluoromethanesulfonate (23.3 mg, 0.05 mmol) acetonitrile-d3 (500 µL) and sealed with a hollow cap. An LDPE tube was filled with five layers of substances in a following sequence: acetonitrile (100 µL), air (100 µL), solution of *N*-methyl-1-(naphthalen-1-yl)methanamine (8.56 mg, 0.05 mmol, 1.00 equiv in 100 µL MeCN), air (100 µL) and acetonitrile (100 µL), then was placed in the NMR tube. These substances were added in one portion to the reaction mixture at t= 0 second. Recording of ^{19}F-NMR spectra (proton decoupled, No. dummy scan= 0, No. scan= 1) was started at t= −60 seconds and 15 seconds time intervals were applied between experiments.

Supplementary Figure 8: Reaction scheme and monitoring of homodiamination reaction by ^{19}F-NMR-spectroscopy, in the presence of one equivalent amine

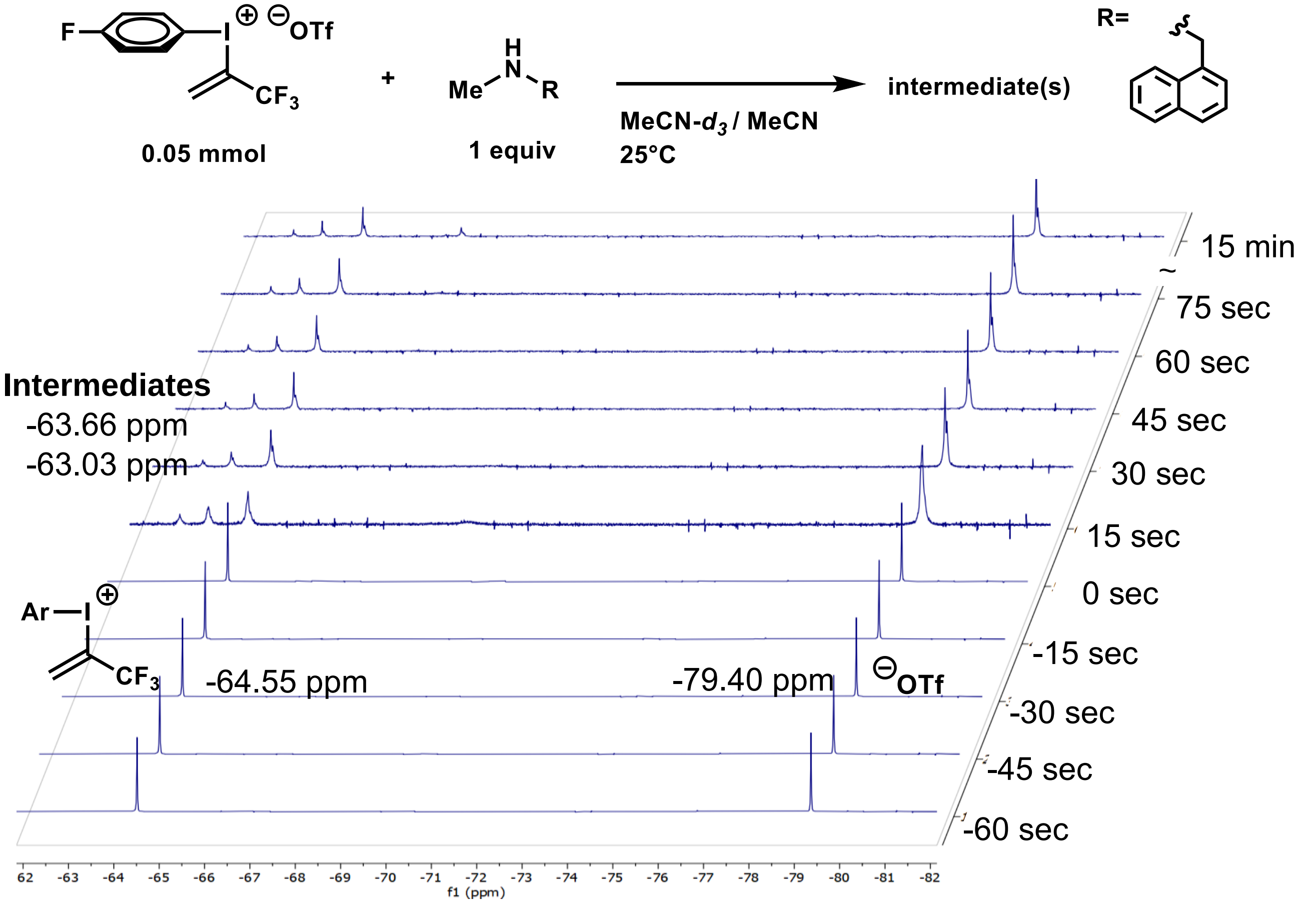

Study of the first stage of reaction conditions II by ^{19}F-NMR spectroscopy

An 8 mL test tube was charged with stirring bar, (4-fluorophenyl)(3,3,3-trifluoroprop-1-en-2-yl)iodonium trifluoromethanesulfonate (23.3 mg, 0.05 mmol), acetonitrile-*d*3 (500 µL) and cooled to -20°C. To the stirred mixture, solution of *N*-methyl-1-(naphthalen-1-yl)methanamine (8.56 mg, 0.05 mmol, 1.00 equiv, in 500 µL acetonitrile-*d*3) was added via syringe over 30 minutes. After completion of addition, reaction mixture was stirred for 15 minutes at the same temperature, then the complete reaction mixture was transferred to a 0.5 mm O.D. NMR tube at room temperature. ^{19}F-NMR spectra was recorded within 10 minutes.

Supplementary Figure 9: Reaction scheme of the first stage of reaction conditions II and study by ^{19}F-NMR spectroscopy

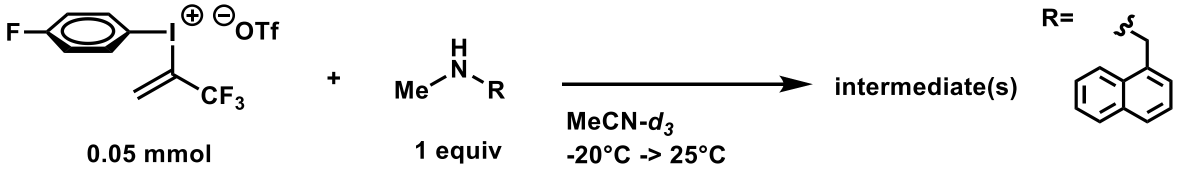

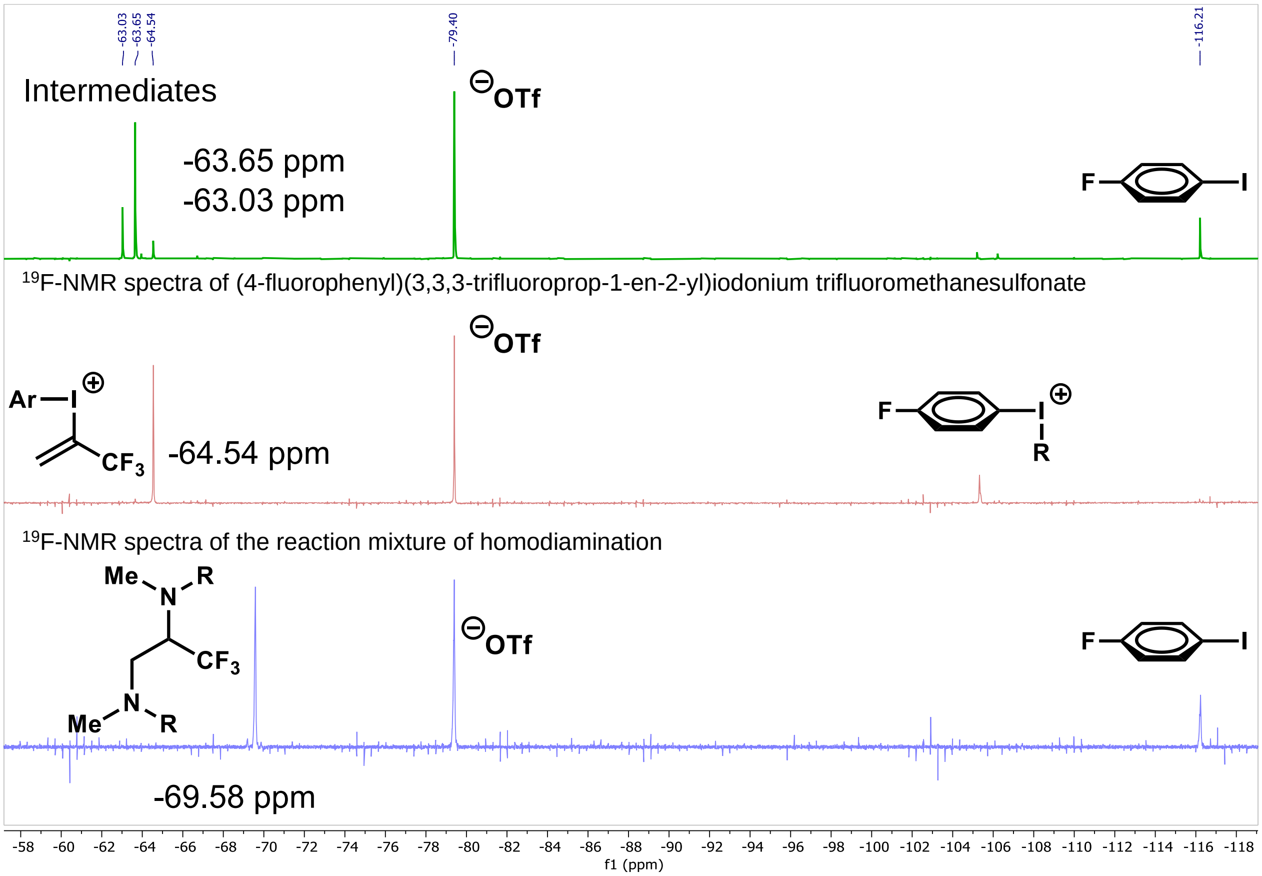

*Generation of intermediate **X** by independent route*

A 0.5 mm O.D. NMR tube was charged with 1-(naphthalen-1-ylmethyl)-2-(trifluoromethyl)aziridine (**6**) (18 mg, 0.72 mmol), sodium carbonate (38 mg, 0.358 mmol, 5 equiv) and acetonitrile-*d*3 (500 µL). The mixture was treated with methyl triflate (20 µL, 0.179 mmol, 2.5 equiv) at room temperature and recording of ^{19}F-NMR spectra was started immediately. After 2.5 hours, complete consumption of 1-(naphthalen-1-ylmethyl)-2-(trifluoromethyl)aziridine was observed.

Supplementary Figure 10: Generation of intermediate **X** from trifluoromethyl aziridine **6** and its reaction with excess amine

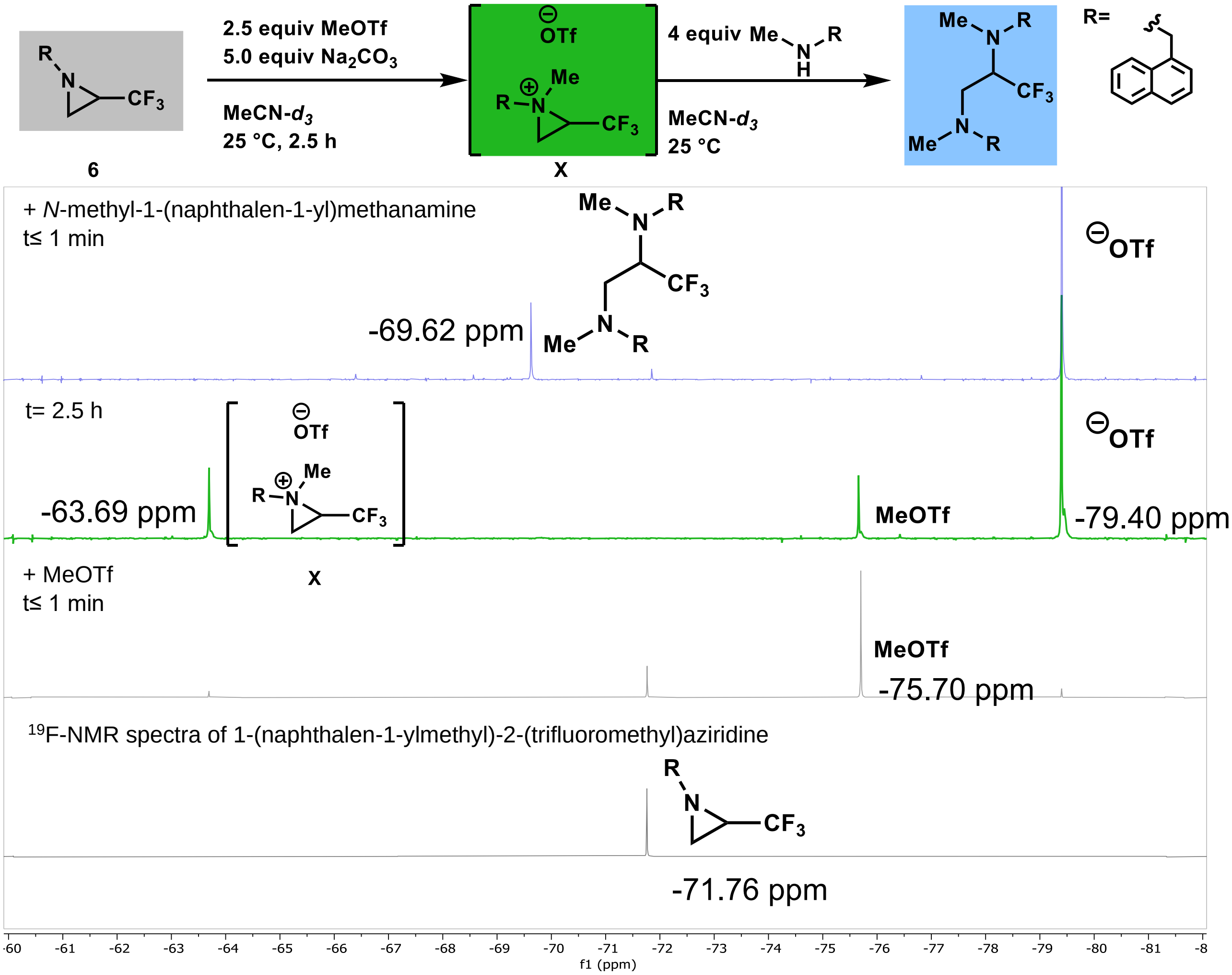

Addition of *N*-methyl-1-(naphthalen-1-yl)methanamine (49 mg, 0.286 mmol, 4.0 equiv) led to the formation of corresponding diamine. Also, this result was confirmed by GC-MS spectra of the mixture.

Synthesis of heterofunctionalized diamines

General procedure A

A 30 mL screwed cap vial was charged with rare-earth magnetic stirring bar, **1** (1 equiv) and acetonitrile (10 mL/mmol), then the vial was sealed with Teflon septa and screwed cap. The stirred reaction mixture was cooled to -20 °C (bath temp= -23 °C) and solution of first amine (1 equiv, 0.1 M in acetonitrile) was added dropwise (0.3 mmol/h) by syringe pump, then the stirring was continued for 10 mins/0.1 mmol to obtain the solution of aziridinium intermediate. After that second amine or other nucleophile (2.5 equiv) was added in one portion and the mixture was allowed to warm room temperature over 16 hours. Reaction mixture was concentrated onto Celite under reduced pressure and the obtained residue was purified by flash column chromatography. Gradient elution was performed by using either hexanes:ethyl acetate or hexanes:diisopropyl ether or dichloromethane:isopropanol eluent system, according to TLC eluents.

General procedure B

Aziridinium intermediate solution was prepared according to ***general procedure A***. A separate, oven dried vial was charged with stirring bar, appropriate heterocycle (2.6 equiv) and sealed with Teflon septa and screw cap. Vial was evacuated and backfilled with argon (repeated three times), then THF (3.2 mL/mmol heterocycle) was added under argon atmosphere. The stirred mixture was cooled to -78°C and solution of LiHMDS (2.5 equiv, 1 M in THF) was added dropwise. After 1 hour, HMPA (1 equiv) was added in one portion and stirring was continued at -78°C for 10 minutes. To the solution of deprotonated heterocycle, solution of aziridinium intermediate was added at -78°C, then allowed to warm room temperature over 16 hours. Purification was carried out according to ***general procedure A***.

General procedure C

Aziridinium intermediate solution was prepared according to ***general procedure A***. A separate, oven dried vial was charged with stirring bar, appropriate nucleophile (2.5 equiv) and sealed with Teflon septa and screw cap. Vial was evacuated and backfilled with argon (repeated three times), then THF (3.2 mL/mmol nucleophile) was added under argon atmosphere. To the stirred mixture, NaH (2.5 equiv, 60 w% in oil) was added in one portion under argon atmosphere at room temperature and stirring was continued for 1 hour. After that, solution of aziridinium intermediate was added and the mixture was stirred at room temperature for 16 hours. Purification was carried out according to ***general procedure A***.

General procedure D

A 30 mL screwed cap vial was charged with rare-earth magnetic stirring bar, **1** (1 equiv) and THF (10 mL/mmol), then the vial was sealed with Teflon septa and screwed cap. The stirred reaction mixture was cooled to -55 °C (bath temp= -58 °C) and solution of *N*-methyl-1-(naphthalen-1-yl)methanamine (1 equiv, 0.1 M in THF) was added dropwise (0.3 mmol/h) by syringe pump, then the stirring was continued for 10 mins/0.1 mmol to obtain the solution of aziridinium intermediate. A separate, oven dried vial was charged with stirring bar, appropriate heterocycle (1.1 equiv) and sealed with Teflon septa and screw cap. Vial was evacuated and backfilled with argon (repeated three times), then THF (3.2 mL/mmol heterocycle) was added under argon atmosphere. The stirred mixture was cooled to -78°C and solution of ^{n}BuLi (1.15 equiv, 1.6 M in hexanes) was added dropwise. After 1 hour, solution of aziridinium intermediate was added at -78°C, then allowed to warm room temperature over 16 hours. Purification was carried out according to ***general procedure A***.

*Synthesis of compound **44***

3,3,3-trifluoro-N^1-hexyl-N^2-methyl-N^2-(naphthalen-1-ylmethyl)propane-1,2-diamine

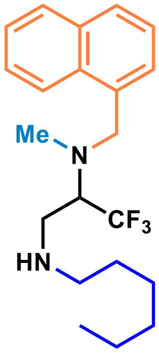

Compound **44** was prepared according to ***general procedure A*** from **1** (93.2 mg, 0.2 mmol), *N*-methyl-1-(naphthalen-1-yl)methanamine (34.2 mg, 0.2 mmol, 1 equiv) and hexylamine (66 µL, 0.5 mmol, 2.5 equiv) to afford a pale yellow oil (41.3 mg, 0.113 mmol, 56% yield). $\mathbf{R_f}$= 0.48 (hexanes:ethyl acetate= 7:3). **LRMS** (EI, 70 eV): m/z (%): 366(1), 253(3), 252(2), 234(8), 233(49), 232(2), 212(4), 202(4), 197(4), 194(4), 190(2), 182(2), 170(2), 168(2), 142(13), 141(100), 140(2), 139(6), 116(2), 115(25), 114(63), 57(2), 56(2), 55(2). **^{1}H NMR** (250 MHz, Chloroform-*d*) δ 8.19 – 8.06 (m, 1H), 7.94 – 7.69 (m, 2H), 7.61 – 7.44 (m, 2H), 7.50 – 7.35 (m, 2H), 4.36 (d, *J* = 13.0 Hz, 1H), 4.27 (d, *J* = 13.2 Hz, 1H), 3.47 (dd, *J* = 8.6, 3.5 Hz, 1H), 2.87 (dd, *J* = 12.7, 10.6 Hz, 1H), 2.64 (dd, *J* = 12.7, 3.4 Hz, 1H), 2.49 (q, *J* = 2.0 Hz, 3H), 2.46 – 1.88 (m, 3H), 1.39 – 0.92 (m, 8H), 0.87 (t, *J* = 6.9 Hz, 3H). **^{19}F NMR** (235 MHz, Chloroform-*d*) δ -66.63. **^{13}C NMR** (63 MHz, Chloroform-*d*) δ 134.2, 134.0, 132.4, 128.9, 128.7, 127.7 (q, *J* = 292.0 Hz), 126.2, 125.9, 125.3, 124.4, 60.9 (q, *J* = 24.4 Hz), 58.4, 49.3, 45.2 (q, *J* = 2.0 Hz), 35.9, 31.8, 29.8, 27.0, 22.7, 14.2. **IR** (ATR, cm^{-1}) 3047, 2955, 2928, 2856, 2840, 2826, 1510, 1463, 1375, 1255, 1157, 1103, 1052, 1019, 907, 862, 790, 774, 730, 706, 644, 605, 543, 519, 503, 420. **HRMS** m/z $[M+H]^+$ calculated for $C_{21}H_{30}N_2F_3^+$: 367.2361, found: 367.2359.

*Synthesis of compound **45***

1,1,1-trifluoro-*N*-methyl-*N*-(naphthalen-1-ylmethyl)-3-(pyrrolidin-1-yl)propan-2-amine

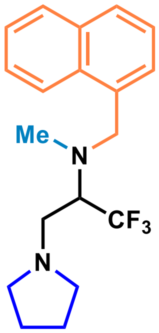

Compound **45** was prepared according to ***general procedure A*** from **1** (140 mg, 0.3 mmol), *N*-methyl-1-(naphthalen-1-yl)methanamine (51.4 mg, 0.3 mmol, 1 equiv) and pyrrolidine (63 µL, 0.75 mmol, 2.5 equiv) to afford an off white solid (76.9 mg, 0.229 mmol, 76% yield). **R_f**= 0.60 (hexanes:ethyl acetate= 7:3). **M.p.=** 45-47 °C. **LRMS** (EI, 70 eV): m/z (%): 336(1), 168(1), 142(4), 141(30), 140(1), 139(4), 116(1), 115(14), 85(8), 84(100), 82(1), 70(1), 56(2), 55(5). **^{1}H NMR** (300 MHz, Chloroform-*d*) δ 8.33 (d, *J* = 7.2 Hz, 1H), 7.86 (dd, *J* = 7.0, 2.3 Hz, 1H), 7.80 (d, *J* = 8.2 Hz, 1H), 7.57 – 7.40 (m, 4H), 4.40 (d, *J* = 13.4 Hz, 1H), 4.31 (d, *J* = 13.4 Hz, 1H), 3.50 (pd, *J* = 8.8, 3.9 Hz, 1H), 3.02 (dd, *J* = 13.1, 9.3 Hz, 1H), 2.65 (dd, *J* = 13.1, 3.9 Hz, 1H), 2.56 – 2.39 (m, 7H), 1.80 – 1.66 (m, 4H). **^{19}F NMR** (235 MHz, Chloroform-*d*) δ -67.8. **^{13}C NMR** (75 MHz, Chloroform-*d*) δ 134.5, 134.0, 132.5, 128.5, 128.2, 127.6, 127.4 (q, *J* = 291.7 Hz), 125.8, 125.8, 125.3, 124.9, 62.7 (q, *J* = 24.1 Hz), 58.3, 54.4, 52.3, 36.8, 23.6. **IR** (ATR, cm^{-1}) 3091, 3046, 2955, 2911, 2874, 2816, 2781, 2698, 1597, 1509, 1460, 1391, 1354, 1312, 1252, 1202, 1170, 1149, 1119, 1097, 1071, 1051, 1031, 946, 883, 845, 791, 774, 709, 622, 559, 542, 520, 454, 414. **HRMS** m/z $[M+H]^+$ calculated for $C_{19}H_{24}N_2F_3^+$: 337.1892, found: 337.1895.

*Synthesis of compound **46***

1,1,1-trifluoro-*N*-methyl-3-(2-methylpiperidin-1-yl)-*N*-(naphthalen-1-ylmethyl)propan-2-amine

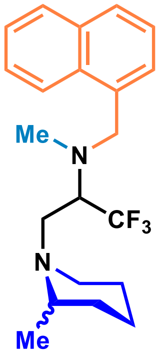

Compound **46** was prepared according to ***general procedure A*** from **1** (140 mg, 0.3 mmol), *N*-methyl-1-(naphthalen-1-yl)methanamine (51.4 mg, 0.3 mmol, 1 equiv) and 2-methylpiperidine (88 µL, 0.75 mmol, 2.5 equiv) to afford a pale yellow oil (73.5 mg, 0.201 mmol, 67% yield). **R_f**= 0.33 (hexanes:ethyl acetate= 10:1). **LRMS** (EI, 70 eV): m/z (%): 142(2), 141(18), 139(2), 116(1), 115(5), 115(1), 113(8), 112(100), 110(2), 96(1), 84(1), 83(1), 70(1), 69(1), 56(1), 55(4). **^{1}H NMR** (250 MHz, Chloroform-*d*) δ 8.32 (dd, J = 20.1, 7.6 Hz, 1H), 7.85 (dd, J = 16.0, 8.2 Hz, 2H), 7.63 – 7.36 (m, 4H), 4.51 – 4.23 (m, 2H), 3.47 (dq, J = 12.1, 4.9, 3.5 Hz, 1H), 3.13 (dd, J = 13.8, 9.4 Hz, 0H), 2.76 (dtd, J = 38.9, 14.3, 5.9 Hz, 3H), 2.55 – 2.45 (m, 3H), 2.41 – 1.99 (m, 2H), 1.57 (d, J = 11.6 Hz, 2H), 1.51 – 1.40 (m, 2H), 1.34 – 1.13 (m, 2H), 1.02 (t, J = 5.9 Hz, 3H). **^{19}F NMR** (235 MHz, Chloroform-*d*) δ -67.46. **^{13}C NMR** (63 MHz, Chloroform-*d*) δ 134.6, 134.5, 134.0, 132.6, 128.5, 128.5, 128.2, 127.7 (q, *J* = 291.6, 291.1 Hz), 127.5, 125.9, 125.7, 125.3, 125.2, 125.2, 124.9, 62.2 (q, *J* = 23.4 Hz), 61.0 (q, *J* = 23.4 Hz), 58.7, 58.4, 56.5, 56.2, 52.8, 52.2, 51.5,

50.2, 36.8, 36.6, 34.8, 34.3, 26.1, 23.8, 23.0, 19.0, 17.8. **IR** (ATR, cm^{-1}) 3047, 2932, 2856, 2796, 1598, 1510, 1448, 1374, 1334, 1307, 1251, 1150, 1102, 1054, 1025, 998, 970, 907, 856, 791, 774, 730, 703, 649, 598, 455, 417. **HRMS** m/z $[M+H]^+$ calculated for $C_{21}H_{28}N_2F_3^+$: 365.2205, found: 365.2202.

*Synthesis of compound **47***

3,3,3-trifluoro-N^1,N^1-diisopropyl-N^2-methyl-N^2-(naphthalen-1-ylmethyl)propane-1,2-diamine

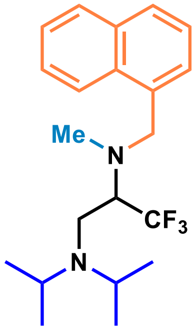

Compound **47** was prepared according to ***general procedure A*** with **1** (140 mg, 0.3 mmol), *N*-methyl-1-(naphthalen-1-yl)methanamine (51.4 mg, 0.3 mmol, 1 equiv) and diisopropylamine (105 µL, 0.75 mmol, 2.5 equiv) to afford a pale yellow oil (59.3 mg, 0.162 mmol, 54% yield). **R_f**= 0.65 (hexanes:ethyl acetate= 10:1). **M.p.=** 43-46 °C. **LRMS** (EI, 70 eV): m/z (%): 209(1), 170(1), 168(1), 167(1), 153(1), 152(1), 142(4), 141(35), 140(1), 139(3), 138(1), 116(1), 115(21), 114(100), 112(1), 89(1), 84(1), 73(1), 72(13), 72(5), 71(1), 70(1), 70(1), 56(2). **^{1}H NMR** (250 MHz, Chloroform-*d*) δ 8.32 (d, J = 8.6 Hz, 1H), 7.97 – 7.76 (m, 2H), 7.64 – 7.38 (m, 4H), 4.51 – 4.27 (m, 2H), 3.48 – 3.24 (m, 1H), 2.94 (hept, J = 6.6 Hz, 2H), 2.78 (d, J = 5.8 Hz, 2H), 2.50 (s, 3H), 0.96 (s, 6H), 0.93 (s, 6H). **^{19}F NMR** (235 MHz, Chloroform-*d*) δ -66.27. **^{13}C NMR** (63 MHz, Chloroform-*d*) δ 134.7, 134.0, 132.6, 130.5, 128.5, 128.1, 127.4, 125.8, 125.7, 125.3, 125.1, 63.6 (q, J = 22.0 Hz), 59.1, 48.0, 42.7, 36.4, 20.9, 20.6. **IR** (ATR, cm^{-1}) 3094, 3050, 2965, 2939, 2927, 2883, 2856, 2817, 2800, 2732, 1598, 1510, 1459, 1390, 1377, 1361, 1309, 1306, 1290, 1249, 1209, 1160, 1127, 1096, 1052, 1042, 1018, 966, 941, 888, 855, 837, 811, 793, 773, 695, 603, 564, 532, 519, 505, 414. **HRMS** m/z $[M+H]^+$ calculated for $C_{21}H_{30}N_2F_3^+$: 367.2361, found: 367.2355.

*Synthesis of compound **48***

N^1-(adamantan-1-yl)-3,3,3-trifluoro-N^2-methyl-N^2-(naphthalen-1-ylmethyl)propane-1,2-diamine

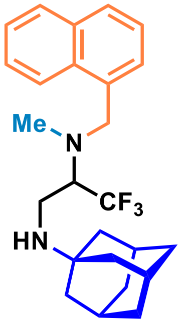

Compound **48** was prepared according to ***general procedure A*** with **1** (140 mg, 0.3 mmol), *N*-methyl-1-(naphthalen-1-yl)methanamine (51.4 mg, 0.3 mmol, 1 equiv) and 1-adamantylamine (47.6 mg, 0.315 mmol, 1.0 equiv) to afford a pale yellow oil (90.1 mg, 0.216 mmol, 72% yield). **R_f**= 0.60 (hexanes:ethyl acetate= 7:3). **LRMS** (EI, 70 eV): m/z (%): 417(1), 416(1), 415(1), 355(1), 287(2), 233(9), 233(5), 165(11), 164(82), 141(39), 141(14), 136(12), 135(100), 115(7), 93(7), 79(12). **^{1}H NMR** (250 MHz, Chloroform-*d*) δ 8.15 (d, J = 8.2 Hz, 1H), 7.94 – 7.72 (m, 2H), 7.64 – 7.34 (m, 4H), 4.32 (s, 2H), 3.59 (bs, J = 9.2 Hz, 0H), 3.49 (ddd, J = 11.2, 8.4, 3.3

Hz, 2H), 2.90 (t, J = 11.4 Hz, 1H), 2.71 (dd, J = 11.9, 3.5 Hz, 1H), 2.45 (s, 3H), 2.02 – 1.89 (m, 3H), 1.66 – 1.41 (m, 6H), 1.34 (d, J = 17.1 Hz, 6H). **^{19}F NMR** (235 MHz, Chloroform-*d*) δ -66.63. **^{13}C NMR** (63 MHz, Chloroform-*d*) δ 134.3, 133.8, 132.4, 128.9, 128.7, 128.2, 127.1 (q, J = 292.4 Hz), 126.5, 125.9, 125.3, 124.6, 62.6 (q, J = 24.8 Hz), 58.6, 51.1, 41.8, 36.4, 36.1 – 35.6 (m), 35.1, 29.4. **IR** (ATR, cm^{-1}) 3047, 2904, 2849, 2819, 1510, 1482, 1452, 1368, 1357, 1289, 1256, 1236, 1197, 1151, 1119, 1103, 1079, 1052, 1027, 907, 852, 791, 774, 729, 702, 638, 563, 539, 518, 478, 441, 418. **HRMS** m/z $[M+H]^+$ calculated for $C_{25}H_{32}N_2F_3^+$: 417.2518, found: 417.2518.

*Synthesis of compound **49***

N[1]-ethyl-3,3,3-trifluoro-*N*[2]-methyl-*N*[2]-(naphthalen-1-ylmethyl)-*N*[1]-phenylpropane-1,2-diamine

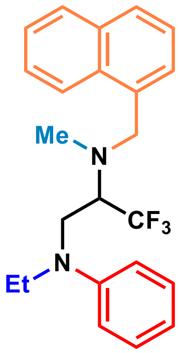

Compound **49** was prepared according to ***general procedure A*** with **1** (140 mg, 0.3 mmol), *N*-methyl-1-(naphthalen-1-yl)methanamine (51.4 mg, 0.3 mmol, 1 equiv) and *N*-ethylaniline (96 µL, 0.75 mmol, 2.5 equiv) to afford a pale yellow oil (92.3 mg, 0.239 mmol, 80% yield). **R_f**= 0.70 (hexanes:ethyl acetate= 10:1). **LRMS** (EI, 70 eV): m/z (%): 387(1), 386(6), 142(3), 141(24), 139(3), 135(16), 134(100), 115(11), 107(2), 106(24), 105(2), 104(4), 91(2), 79(4), 78(2), 77(8). **^{1}H NMR** (250 MHz, Chloroform-*d*) δ 8.13 (d, J = 8.2 Hz, 1H), 7.91 (d, J = 7.9 Hz, 1H), 7.82 (dd, J = 6.0, 3.3 Hz, 1H), 7.62 – 7.34 (m, 4H), 7.27 – 7.11 (m, 2H), 6.75 (t, J = 7.2 Hz, 1H), 6.57 (d, J = 8.3 Hz, 2H), 4.35 (s, 2H), 3.82 – 3.67 (m, 1H), 3.68 (dd, J = 15.7, 4.2 Hz, 1H), 3.52 (dd, J = 15.3, 8.0 Hz, 1H), 3.48 – 3.11 (m, 2H), 2.60 (s, 3H), 1.07 (t, J = 7.0 Hz, 3H). **^{19}F NMR** (235 MHz, Chloroform-*d*) δ -66.22. **^{13}C NMR** (63 MHz, Chloroform-*d*) δ 146.8, 134.0, 133.9, 132.4, 129.4, 128.5 (d, J = 1.3 Hz), 127.8, 127.7 (q, J = 293.6 Hz), 126.0, 125.7, 125.1, 124.9, 116.2, 112.1, 60.5 (q, J = 22.9 Hz), 59.6, 47.7, 46.0, 35.9, 11.5. **IR** (ATR, cm^{-1}) 3095, 3060, 3043, 2971, 2932, 2897, 2861, 2810, 1598, 1504, 1463, 1374, 1353, 1242, 1211, 1192, 1153, 1126, 1100, 1061, 1039, 1022, 973, 907, 854, 790, 774, 744, 691, 649, 574, 547, 506, 445, 431, 417. **HRMS** m/z $[M+H]^+$ calculated for $C_{23}H_{26}N_2F_3^+$: 387.2048, found: 387.2049.

Synthesis of compound ***50***

3,3,3-trifluoro-*N*[1],*N*[2]-dimethyl-*N*[2]-(naphthalen-1-ylmethyl)-*N*[1]-(2-(trifluoromethyl)phenyl)propane-1,2-diamine

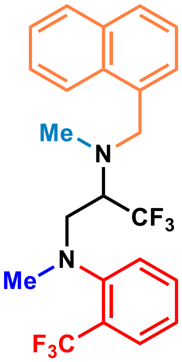

Compound **50** was prepared according to ***general procedure A*** with **1** (93.2 mg, 0.2 mmol), *N*-methyl-1-(naphthalen-1-yl)methanamine (34.2 mg, 0.2 mmol, 1 equiv), 2-trifluoromethyl-*N*-methylaniline (91.2 mg, 0.5 mmol, 2.5 equiv) and 2,6-di-*tert*-butylpyridine (116 µL, 0.5 mmol, 2.5 equiv) to afford a colorless oil (39.3 mg, 0.089 mmol, 45% yield). $\mathbf{R_f}$= 0.65 (hexanes:ethyl acetate= 10:1). **LRMS** (EI, 70 eV): m/z (%): 189(10), 188(100), 173(2), 172(3), 168(1), 159(1), 154(1), 145(2), 142(3), 141(20), 140(1), 139(3), 128(1), 127(4), 126(1), 118(1), 116(1), 115(8), 109(1), 91(2), 77(1). ^{1}**H NMR** (250 MHz, Chloroform-*d*) 8.14 (d, *J* = 7.2 Hz, 1H), 7.84 – 7.68 (m, 2H), 7.56 (d, *J* = 7.8 Hz, 1H), 7.52 – 7.28 (m, 5H), 7.14 (dd, *J* = 14.2, 7.3 Hz, 2H), 4.27 (s, 2H), 3.51 (ddq, *J* = 12.7, 8.4, 4.3 Hz, 1H), 3.29 (dd, *J* = 13.6, 8.1 Hz, 1H), 3.16 (dd, *J* = 13.6, 4.2 Hz, 1H), 2.54 (s, 3H), 2.34 (s, 3H). ^{19}**F NMR** (235 MHz, Chloroform-*d*) δ -59.64 (s, 3F), -67.66 (s, 3F). ^{13}**C NMR** (63 MHz, Chloroform-*d*) δ 153.1, 134.0, 133.0, 132.4, 129.6, 128.6, 128.3, 127.7 (d, *J* = 5.5 Hz), 127.5, 127.5, 127.1, 126.5 (d, *J* = 20.7 Hz), 126.1, 125.8, 125.4, 124.7, 124.7, 124.6, 122.0, 62.7 (q, *J* = 24.0 Hz), 58.2, 53.8, 45.5, 36.4. **IR** (ATR, cm^{-1}) 3067, 3048, 2959, 2901, 2861, 2810, 1602, 1584, 1510, 1496, 1453, 1431, 1392, 1314, 1252, 1105, 1052, 1035, 980, 934, 854, 791, 763, 734, 703, 647, 600, 564, 417. **HRMS** m/z [M+H]$^+$ calculated for $C_{21}H_{20}N_2F_6^+$: 441.1760, found: 441.1775.

Synthesis of compound ***51***

N-(4-methoxybenzyl)-*N*-(3,3,3-trifluoro-2-(methyl(naphthalen-1-ylmethyl)amino)propyl)-*L*-leucinate

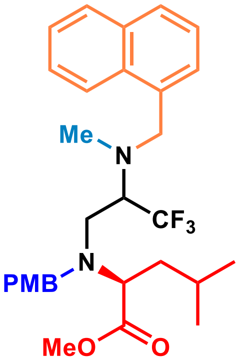

Compound **51** was prepared according to ***general procedure A*** with **1** (140 mg, 0.3 mmol), *N*-methyl-1-(naphthalen-1-yl)methanamine (51.4 mg, 0.3 mmol, 1 equiv) and methyl (4-methoxybenzyl)-*L*-leucinate (199 mg, 0.75 mmol, 2.5 equiv) to afford a pale yellow oil (118 mg, 0.223 mmol, 74% yield). $\mathbf{R_f}$= 0.35 (hexanes:ethyl acetate= 10:1). **LRMS** (EI, 70 eV): m/z (%): 471(2), 279(9), 278(48), 142(3), 141(25), 122(11), 121(100), 115(4), 91(2), 77(2). ^{1}**H NMR** (250 MHz, Chloroform-*d*) 8.32 – 8.08 (m, 1H), 7.99 – 7.72 (m, 2H), 7.64 – 7.36 (m, 4H), 7.08 (d, *J* = 8.1 Hz, 1H), 7.04 (d, *J* = 8.3 Hz, 1H), 6.79 (d, *J* = 8.4 Hz, 1H), 6.74 (d, *J* = 8.4 Hz, 1H), 4.44 – 4.19 (m, 2H), 3.86 – 3.61 (m, 7H), 3.55 – 3.27 (m, 3H), 3.23 – 3.06 (m, 1H), 3.03 – 2.68 (m, 1H), 2.54 – 2.33 (m, 3H), 1.75 – 1.41 (m, 3H), 0.91 – 0.60 (m, 6H). ^{19}**F**

NMR (235 MHz, Chloroform-*d*) δ -66.1, -66.6 (d. r.= 50:50). ^{13}**C NMR** (63 MHz, Chloroform-*d*) δ 173.8, 173.7, 158.8, 134.3, 134.1, 134.0, 132.4, 132.3, 131.0, 130.8, 130.3, 130.2, 128.6, 128.6, 128.2, 128.1, 127.3, 126.0, 125.9, 125.7, 125.3, 124.6, 124.3, 113.6, 63.8, 63.5, 63.3, 62.9, 62.5, 60.6, 60.5, 58.2, 58.1, 56.0, 55.8, 55.2, 55.2, 51.1, 51.1, 48.5, 48.1, 39.3, 38.6, 36.9, 36.3, 30.9, 24.7, 24.6, 22.8, 22.7, 22.3, 22.2. **IR** (ATR, cm^{-1}) 3039, 2996, 2955, 2929, 2908, 2868, 2837, 1731, 1612, 1585, 1510, 1463, 1367, 1302, 1245, 1156, 1103, 1054, 1035, 984, 909, 824, 791, 774, 732, 706, 519, 417. **HRMS** m/z $[M+H]^+$ calculated for $C_{30}H_{38}N_2O_3F_3^+$: 531.2835, found: 531.2838.

Synthesis of compound ***52***

3-(4-bromo-1*H*-pyrazol-1-yl)-1,1,1-trifluoro-*N*-methyl-*N*-(naphthalen-1-ylmethyl)propan-2-amine

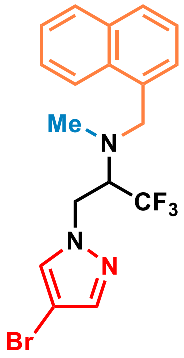

Compound **52** was prepared according to ***general procedure A*** with **1** (140 mg, 0.3 mmol), *N*-methyl-1-(naphthalen-1-yl)methanamine (51.4 mg, 0.3 mmol, 1 equiv) and 4-bromopyrazole (112 mg, 0.75 mmol, 2.5 equiv) and sodium hydride (30 mg, 60 w% in oil, 0.75 mmol, 2.5 equiv) to afford a white solid (94.2 mg, 0.229 mmol, 76% yield). $\mathbf{R_f}$= 0.35 (hexanes:ethyl acetate= 10:1). **M.p.=** 131-133 °C. **LRMS** (EI, 70 eV): m/z (%): 270(1), 252(5), 234(2), 196(2), 171(14), 170(100), 168(4), 142(23), 141(97), 141(87), 139(10), 115(31). ^{1}**H NMR** (250 MHz, Chloroform-*d*) 7.75 – 7.59 (m, 2H), 7.44 (d, J = 8.2 Hz, 1H), 7.39 – 7.16 (m, 4H), 7.11 (d, J = 7.1 Hz, 1H), 7.03 (s, 1H), 4.24 – 3.98 (m, 4H), 3.80 (ddp, J = 12.9, 8.6, 4.6 Hz, 1H), 2.53 – 2.17 (m, 3H). ^{19}**F NMR** (235 MHz, Chloroform-*d*) δ -67.00. ^{13}**C NMR** (63 MHz, Chloroform-*d*) δ 140.4, 133.9, 133.1, 132.1, 130.6, 128.7, 128.4, 127.7, 126.6 (q, J = 292.7 Hz), 126.3, 125.8, 125.1, 123.9, 93.2, 63.1 (q, J = 25.0 Hz), 58.7, 48.7 (q, J = 2.2 Hz), 35.2. **IR** (ATR, cm^{-1}) 3138, 3094, 3066, 3033, 2997, 2963, 2929, 2880, 2861, 2806, 1507, 1473, 1449, 1382, 1295, 1252, 1224, 1192, 1164, 1156, 1106, 1068, 1019, 950, 866, 846, 791, 734, 720, 692, 645, 610, 580, 448, 420. **HRMS** m/z $[M+H]^+$ calculated for $C_{18}H_{18}N_3F_3Br^+$: 412.0636, found: 412.0640.

*Synthesis of compound **53***

2-(3,3,3-trifluoro-2-(methyl(naphthalen-1-ylmethyl)amino)propyl)isoindoline-1,3-dione

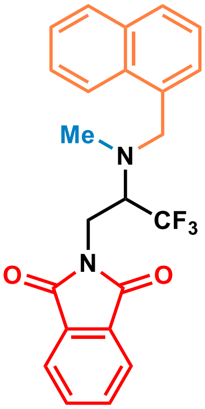

Compound **53** was prepared according to ***general procedure A*** with **1** (140 mg, 0.3 mmol), *N*-methyl-1-(naphthalen-1-yl)methanamine (51.4 mg, 0.3 mmol, 1 equiv) and potassium phthalimide (139 mg, 0.75 mmol, 2.5 equiv) to afford a white solid (97.0 mg, 0.235 mmol, 78% yield). **R$_f$**= 0.70 (hexanes:ethyl acetate= 7:3). **M.p.=** 112-114 °C. **LRMS** (EI, 70 eV): m/z (%): 412(1), 271(3), 252(9), 171(3), 170(21), 160(4), 160(2), 154(2), 142(13), 141(100), 139(4), 115(14), 104(2), 77(3), 76(2). **^{1}H NMR** (250 MHz, Chloroform-*d*) 7.59 – 7.32 (m, 7H), 7.22 – 7.06 (m, 2H), 6.76 (t, *J* = 7.5 Hz, 1H), 6.52 (t, *J* = 7.6 Hz, 1H), 4.17 – 3.85 (m, 3H), 3.60 (dqd, *J* = 11.4, 8.2, 3.3 Hz, 1H), 3.33 (dd, *J* = 13.9, 3.4 Hz, 1H), 2.57 (s, 3H). **^{19}F NMR** (235 MHz, Chloroform-*d*) δ -66.58. **^{13}C NMR** (63 MHz, Chloroform-*d*) δ 167.3, 133.7, 133.5, 133.2, 131.9, 131.8, 128.1, 126.8 (q, *J* = 293.3 Hz), 123.7, 123.1, 58.4, 57.6 (q, *J* = 24.5 Hz), 36.1, 33.9 (q, *J* = 2.6 Hz). **IR** (ATR, cm^{-1}) 3048, 3005, 2951, 2895, 2860, 2826, 2746, 1771, 1707, 1616, 1598, 1510, 1466, 1428, 1405, 1377, 1334, 1252, 1226, 1192, 1161, 1103, 1055, 1018, 970, 953, 875, 801, 786, 770, 726, 713, 702, 614, 529, 509, 449, 432. **HRMS** m/z [M+H]$^+$ calculated for $C_{23}H_{20}N_2O_2F_3^+$: 413.1477, found: 413.1469.

*Synthesis of compound **54***

1,1,1-trifluoro-3-(1*H*-indol-1-yl)-*N*-methyl-*N*-(naphthalen-1-ylmethyl)propan-2-amine

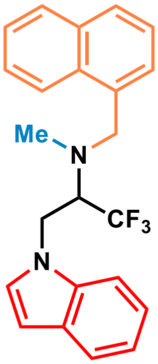

Compound **54** was prepared according to ***general procedure B*** from **1** (140 mg, 0.3 mmol), *N*-methyl-1-(naphthalen-1-yl)methanamine (51.4 mg, 0.3 mmol, 1 equiv), indole (91.4 mg, 0.78 mmol, 2.6 equiv), solution of LiHMDS (750 µL, 1 M in THF, 0.75 mmol, 2.5 equiv) and HMPA (132 µL, 0.75 mmol, 2.5 equiv) to afford a pale yellow oil (86.7 mg, 0.227 mmol, 76% yield). **R$_f$**= 0.60 (hexanes:ethyl acetate= 1:1). **LRMS** (EI, 70 eV): m/z (%): 383(3), 382(12), 252(11), 142(12), 141(100), 139(4), 131(4), 130(30), 116(3), 115(14), 103(5), 89(3), 77(5). **^{1}H NMR** (300 MHz, Chloroform-*d*) 7.89 (d, *J* = 8.2 Hz, 1H), 7.81 (d, *J* = 8.3 Hz, 1H), 7.80 – 7.74 (m, 1H), 7.72 (d, *J* = 7.9 Hz, 1H), 7.52 (t, *J* = 7.5 Hz, 1H), 7.37 – 7.29 (m, 2H), 7.25 – 7.20 (m, 2H), 7.18 (d, *J* = 7.2 Hz, 1H), 7.14 – 7.05 (m, 1H), 7.01 (d, *J* = 3.2 Hz, 1H), 6.53 (d, *J* = 3.2 Hz, 1H), 4.49 (dd, *J* = 15.0, 4.5 Hz, 1H), 4.38 (dd, *J* = 15.0, 8.6 Hz, 1H), 4.29 (d, *J* = 13.5 Hz, 1H), 4.23 (d, *J* = 13.3 Hz, 1H), 3.85 (pd, *J* = 8.4, 4.5 Hz, 1H), 2.68 (s, 3H). **^{19}F NMR** (282 MHz, Chloroform-*d*) δ -66.82 (d, *J* = 8.4 Hz). **^{13}C NMR** (75 MHz, Chloroform-*d*) δ 135.8, 133.8, 133.1, 132.1, 129.0, 128.9, 128.7, 128.4,

128.4, 127.6, 126.1, 125.7, 125.1, 124.1, 121.9, 121.3, 119.7, 108.9, 101.8, 62.4 (q, *J* = 24.4 Hz), 58.8, 43.5 (q, *J* = 2.4 Hz), 35.7. **IR** (ATR, cm^{-1}) 3050, 2983, 2944, 2860, 2810, 1511, 1479, 1463, 1377, 1314, 1248, 1174, 1158, 1105, 1069, 1052, 1014, 964, 907, 864, 791, 776, 763, 730, 696, 648, 584, 424. **HRMS** m/z $[M+H]^+$ calculated for $C_{23}H_{22}N_2F_3^+$: 383.1730, found: 383.1734.

*Synthesis of compound **55***

1,1,1-trifluoro-N-methyl-N-(naphthalen-1-ylmethyl)-3-(1H-pyrrolo[2,3-b]pyridin-1-yl)propan-2-amine

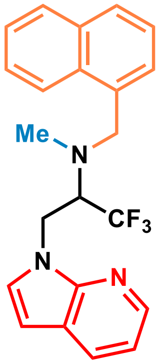

Compound **55** was prepared according to ***general procedure B*** from **1** (140 mg, 0.3 mmol), *N*-methyl-1-(naphthalen-1-yl)methanamine (51.4 mg, 0.3 mmol, 1 equiv), 7-azaindole (92.1 mg, 0.78 mmol, 2.6 equiv), solution of LiHMDS (750 µL, 1 M in THF, 0.75 mmol, 2.5 equiv) and HMPA (132 µL, 0.75 mmol, 2.5 equiv) to afford a pale yellow solid (55.7 mg, 0.145 mmol, 48% yield). $\mathbf{R_f}$= 0.54 (hexanes:ethyl acetate= 1:1). **M.p.=** 75-78 °C. **LRMS** (EI, 70 eV): m/z (%): 383(4), 265(3), 242(11), 222(11), 170(29), 142(12), 141(100), 139(6), 131(23), 119(11), 118(24), 115(20), 104(4), 104(3), 78(3), 77(4). **^{1}H NMR** (300 MHz, Chloroform-*d*) 8.27 (d, *J* = 4.7 Hz, 1H), 7.94 – 7.86 (m, 1H), 7.76 (d, *J* = 8.3 Hz, 1H), 7.69 (d, *J* = 8.3 Hz, 1H), 7.48 (d, *J* = 8.6 Hz, 1H), 7.38 (t, *J* = 7.6 Hz, 1H), 7.31 – 7.22 (m, 1H), 7.17 – 7.03 (m, 3H), 6.96 (d, *J* = 3.5 Hz, 1H), 6.33 (d, *J* = 3.5 Hz, 1H), 4.58 (d, *J* = 7.0 Hz, 2H), 4.22 (d, *J* = 13.5 Hz, 1H), 4.19 – 4.07 (m, 2H), 2.61 (s, 3H). **^{19}F NMR** (282 MHz, Chloroform-*d*) δ -66.97 (d, *J* = 8.0 Hz). **^{13}C NMR** (75 MHz, Chloroform-*d*) δ 147.3, 142.6, 133.7, 133.4, 132.1, 129.0, 128.6, 128.3, 128.2, 127.4, 127.0 (q, *J* = 293.4 Hz), 125.8, 125.5, 125.0, 123.9, 120.9, 115.9, 100.1, 62.4 (q, *J* = 24.3 Hz), 58.5, 41.2, 35.7. **IR** (ATR, cm^{-1}) 3053, 2988, 2952, 2884, 2864, 2812, 2249, 1595, 1572, 1510, 1432, 1310, 1249, 1208, 1175, 1157, 1110, 1073, 1054, 1017, 906, 866, 793, 773, 726, 699, 648, 597, 479, 415. **HRMS** m/z $[M+H]^+$ calculated for $C_{22}H_{21}N_3F_3^+$: 384.1682, found: 384.1686.

Synthesis of compound ***56***

3-(1*H*-benzo[d]imidazol-1-yl)-1,1,1-trifluoro-*N*-methyl-*N*-(naphthalen-1-ylmethyl)propan-2-amine

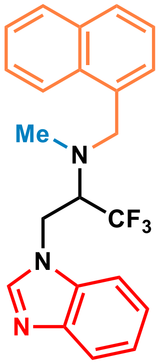

Compound **56** was prepared according to ***general procedure B*** from **1** (140 mg, 0.3 mmol), *N*-methyl-1-(naphthalen-1-yl)methanamine (51.4 mg, 0.3 mmol, 1 equiv), benzimidazole (92.1 mg, 0.78 mmol, 2.6 equiv), solution of LiHMDS (750 µL, 1 M in THF, 0.75 mmol, 2.5 equiv) and HMPA (132 µL, 0.75 mmol, 2.5 equiv) to afford an off-white solid (54.1 mg, 0.141 mmol, 47% yield). $\mathbf{R_f}$= 0.25 (hexanes:ethyl acetate= 1:1). **M.p.=** 73-76 °C. **LRMS** (EI, 70 eV): m/z (%): 383(3), 253(3), 252(16), 221(1), 142(13), 141(100), 139(3), 131(9), 115(12), 77(5) ^{1}**H NMR** (250 MHz, Chloroform-*d*) 7.68 (dt, *J* = 16.3, 7.0 Hz, 4H), 7.46 (d, *J* = 8.6 Hz, 1H), 7.37 (ddd, *J* = 8.2, 6.8, 1.4 Hz, 1H), 7.31 – 7.13 (m, 3H), 7.14 – 6.98 (m, 2H), 6.82 (d, *J* = 8.1 Hz, 1H), 4.26 (d, *J* = 7.7 Hz, 1H), 4.23 (d, *J* = 13.8 Hz, 1H), 4.12 (d, *J* = 13.0 Hz, 1H), 3.66 (pd, *J* = 8.3, 5.8 Hz, 1H), 2.66 – 2.56 (m, 4H). ^{19}**F NMR** (235 MHz, Chloroform-*d*) δ -66.56. ^{13}**C NMR** (75 MHz, Chloroform-*d*) δ 143.5, 143.4, 133.7, 133.1, 132.4, 131.8, 128.7, 128.4, 127.8, 126.7 (q, *J* = 293.2 Hz), 126.3, 125.9, 124.9, 123.4, 123.1, 122.3, 120.6, 108.7, 60.8 (q, *J* = 24.7 Hz), 58.5, 41.9 (d, *J* = 2.2 Hz), 35.8. **IR** (ATR, cm^{-1} 3210, 3056, 2927, 2900, 2867, 2812, 1727, 1616, 1598, 1502, 1459, 1371, 1287, 1251, 1229, 1175, 1153, 1103, 1082, 1038, 1025, 954, 865, 776, 733, 698, 588, 451, 425. **HRMS** m/z [M+H]$^{+}$ calculated for $C_{22}H_{20}N_3F_3^{+}$: 384.1682, found: 384.1685.

Synthesis of compound ***57***

1,1,1-trifluoro-N-methyl-N-(naphthalen-1-ylmethyl)-3-(1H-pyrrolo[2,3-b]pyridin-1-yl)propan-2-amine

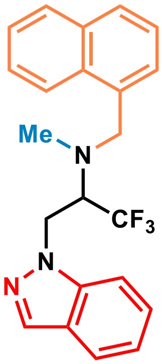

Compound **57** was prepared according to ***general procedure A*** with **1** (140 mg, 0.3 mmol), *N*-methyl-1-(naphthalen-1-yl)methanamine (51.4 mg, 0.3 mmol, 1 equiv) and indazole (89.5 mg, 0.75 mmol, 2.5 equiv) to afford a white solid (69.3 mg, 0.181 mmol, 60% yield). $\mathbf{R_f}$= 0.43 (hexanes:ethyl acetate= 1:1). **M.p.=** 130-132 °C. **LRMS** (EI, 70 eV): m/z (%): 383(1), 327(1), 265(3), 171(5), 170(64), 168(12), 142(13), 141(100), 124(5), 119(9), 118(43), 115(36), 103(5), 89(5), 77(6), 77(4). ^{1}**H NMR** (300 MHz, Chloroform-*d*) 7.78 – 7.65 (m, 4H), 7.58 (d, *J* = 8.4 Hz, 1H), 7.38 (dd, *J* = 8.8, 6.6 Hz, 1H), 7.31 – 7.23 (m, 1H), 7.22 – 7.18 (m, 2H), 7.15 (d, *J* = 8.1 Hz, 2H), 6.48 (t, *J* = 7.7 Hz, 1H), 4.69 (dd, *J* = 13.9, 3.5 Hz, 1H), 4.57 (dd, *J* = 13.9, 10.2 Hz, 1H), 4.42 – 4.26 (m, 1H), 4.22 (s, 2H), 2.51 (s, 3H). ^{19}**F NMR** (282 MHz, Chloroform-*d*) δ -66.87 (d, *J* = 8.3 Hz). ^{13}**C NMR** (75 MHz, Chloroform-*d*) δ 149.1, 133.6, 133.1, 131.9, 128.5, 128.0, 127.7, 126.4, 125.7,

125.6, 124.8, 124.7, 124.0 (q, *J* = 289.9 Hz), 123.7, 121.8, 120.5, 117.4, 64.0 (q, *J* = 25.2 Hz), 59.2, 49.6 (d, *J* = 2.7 Hz), 34.6. **IR** (ATR, cm^{-1}) 3119, 3053, 3009, 3000, 2975, 2952, 2856, 2841, 2807, 1628, 1595, 1516, 1509, 1443, 1382, 1365, 1302, 1289, 1259, 1244, 1174, 1127, 1096, 1072, 1052, 1031, 871, 829, 790. **HRMS** m/z $[M+H]^+$ calculated for $C_{22}H_{21}N_3F_3^+$: 384.1682, found: 384.1686.

Synthesis of compound ***58***

3-(1*H*-benzo[d][1,2,3]triazol-1-yl)-1,1,1-trifluoro-*N*-methyl-*N*-(naphthalen-1-ylmethyl)propan-2-amine

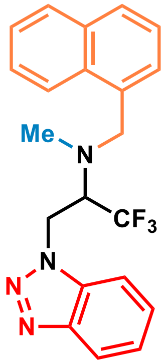

Compound **58** was prepared according to ***general procedure A*** with **1** (140 mg, 0.3 mmol), *N*-methyl-1-(naphthalen-1-yl)methanamine (51.4 mg, 0.3 mmol, 1 equiv) and 1*H*-benzotriazole (89.3 mg, 0.75 mmol, 2.5 equiv) to afford a pale yellow oil (68.5 mg, 0.178 mmol, 59% yield). $\mathbf{R_f}$= 0.30 (hexanes:ethyl acetate= 1:1). **M.p.=** 77-82 °C. **LRMS** (EI, 70 eV): m/z (%): 384(1), 252(1), 171(3), 170(29), 142(12), 141(100), 139(7), 115(12), 115(7), 77(8), 77(5), 51(3). ^{1}**H NMR** (300 MHz, Chloroform-*d*) 7.98 – 7.89 (m, 1H), 7.68 – 7.59 (m, 2H), 7.24 (ddt, *J* = 15.1, 12.7, 5.0 Hz, 5H), 7.03 (d, *J* = 6.9 Hz, 1H), 7.00 – 6.92 (m, 2H), 4.90 (dd, *J* = 14.7, 10.3 Hz, 1H), 4.66 (dd, *J* = 14.7, 3.9 Hz, 1H), 4.17 (d, *J* = 12.9 Hz, 1H), 4.11 (d, *J* = 13.3 Hz, 1H), 4.04 – 3.89 (m, 1H), 2.71 (q, *J* = 2.0 Hz, 3H). ^{19}**F NMR** (282 MHz, Chloroform-*d*) δ -66.98 (d, *J* = 8.0 Hz). ^{13}**C NMR** (75 MHz, Chloroform-*d*) δ 145.7, 133.5, 132.9, 132.4, 131.7, 128.6, 128.2, 127.7, 127.4, 126.5 (q, *J* = 292.6 Hz), 125.9, 125.6, 124.7, 123.7, 123.3, 120.1, 108.4, 60.8 (q, *J* = 25.2 Hz), 58.3, 44.3, 35.8. **IR** (ATR, cm^{-1}) 3064, 3046, 3002, 2976, 2955, 2900, 2854, 2816, 1510, 1449, 1367, 1313, 1251, 1234, 1157, 1100, 1082, 1054, 1015, 953, 790, 776, 746, 720, 698, 567, 431. **HRMS** m/z $[M+H]^+$ calculated for $C_{21}H_{19}N_4F_3^+$: 385.1635, found: 385.1636.

Synthesis of compound ***59***

1,1,1-trifluoro-*N*-methyl-*N*-(naphthalen-1-ylmethyl)-3-(5-phenyl-2*H*-tetrazol-2-yl)propan-2-amine

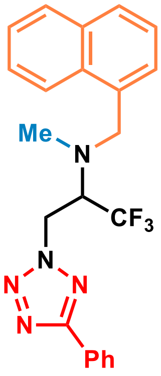

Compound **59** was prepared according to ***general procedure A*** with **1** (140 mg, 0.3 mmol), *N*-methyl-1-(naphthalen-1-yl)methanamine (51.4 mg, 0.3 mmol, 1 equiv) and 5-phenyl-1*H*-tetrazole (110 mg, 0.75 mmol, 2.5 equiv) to afford a colorless oil (77.6 mg, 0.189 mmol, 63% yield). $\mathbf{R_f}$= 0.49 (hexanes:ethyl acetate= 1:1). **LRMS** (EI, 70 eV): m/z (%): 384(5), 383(23), 382(6), 142(13), 141(100), 139(5), 131(10), 115(17), 77(11). ^{1}**H NMR** (300 MHz, Chloroform-*d*) 7.98 – 7.85 (m, 2H), 7.65 (d, *J* = 8.2 Hz, 1H), 7.48 (qd, *J* = 9.5, 8.6, 5.1 Hz, 4H), 7.30 (t, *J* = 7.6 Hz, 1H), 7.21 (d, *J* = 6.9 Hz, 1H), 7.09 (dt, *J* = 6.4, 3.4 Hz, 2H), 5.03 (dd, *J* = 14.0, 11.0 Hz, 1H), 4.59 (dd, *J* = 14.0, 3.5 Hz, 1H), 4.20 (dd, *J* = 13.2, 8.7 Hz, 2H), 4.09 (ddd, *J* = 16.1, 8.1, 4.2 Hz, 1H), 2.67 (q, *J* = 2.0 Hz, 3H). ^{19}**F NMR** (282 MHz, Chloroform-*d*) δ -66.99 (d, *J* = 7.9 Hz). ^{13}**C NMR** (75 MHz, Chloroform-*d*) δ 165.0, 133.7, 132.4, 132.0, 130.3, 128.9, 128.7, 128.2, 127.9, 127.2, 126.9, 126.2, 125.7, 124.9, 124.1 (q, *J* = 293.0 Hz), 61.1 (q, *J* = 25.9 Hz), 58.2, 48.8, 35.7. **IR** (ATR, cm^{-1}) 3685, 3661, 2988, 2971, 2901, 2847, 2253, 1530, 1510, 1466, 1451, 1375, 1312, 1255, 1171, 1140, 1109, 1073, 1052, 1044, 1025, 873, 788, 774, 732, 703, 693, 588, 447, 431, 418. **HRMS** m/z $[M+H]^+$ calculated for $C_{22}H_{21}N_4F_3^+$: 412.1744, found: 412.1749.

Synthesis of compound ***60***

1,1,1-trifluoro-3-(4-methoxy-7H-pyrrolo[2,3-d]pyrimidin-7-yl)-N-methyl-N-(naphthalen-1-ylmethyl)propan-2-amine

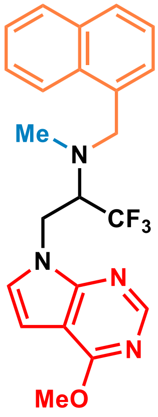

Compound **60** was prepared according to ***general procedure B*** from **1** (140 mg, 0.3 mmol), *N*-methyl-1-(naphthalen-1-yl)methanamine (51.4 mg, 0.3 mmol, 1 equiv), 4-methoxy-7H-pyrrolo[2,3-d]pyrimidine (116 mg, 0.78 mmol, 2.6 equiv), solution of LiHMDS (750 µL, 1 M in THF, 0.75 mmol, 2.5 equiv) and HMPA (132 µL, 0.75 mmol, 2.5 equiv) to afford an beige solid (63.8 mg, 0.154 mmol, 51% yield). $\mathbf{R_f}$= 0.35 (hexanes:ethyl acetate= 1:1). **M.p.=** 78-80 °C. **LRMS** (EI, 70 eV): m/z (%): 414(1), 273(4), 273(3), 253(7), 170(27), 163(3), 162(5), 162(5), 150(10), 149(24), 142(7), 142(5), 141(100), 116(3), 115(26), 93(6), 92(3), 78(3). ^{1}**H NMR** (500 MHz, Chloroform-*d*) 8.22 (s, 1H), 7.68 (d, *J* = 8.1 Hz, 1H), 7.65 (d, *J* = 8.2 Hz, 1H), 7.34 (d, *J* = 8.5 Hz, 1H), 7.31 (ddd, *J* = 8.0, 6.9, 1.2 Hz, 1H), 7.24 (dd, *J* = 8.3, 6.9 Hz, 1H), 7.10 (dd, *J* = 6.9, 1.2 Hz, 1H), 7.07 (ddd, *J* = 8.3, 6.9, 1.3 Hz, 1H), 6.64 (d, *J* = 3.5 Hz, 1H), 6.22 (dd, *J* = 3.5, 0.7 Hz, 1H), 4.37 (dd, *J* = 14.5, 10.1 Hz, 1H), 4.32 (dd, *J* = 14.4, 4.4 Hz, 1H), 4.17 (d, *J* = 12.8 Hz, 1H), 4.14

(s, 3H), 4.09 (d, J = 13.1 Hz, 1H), 4.01 – 3.90 (m, 1H), 2.63 (q, J = 2.0 Hz, 3H). **^{19}F NMR** (282 MHz, Chloroform-*d*) δ -66.78 (d, J = 8.2 Hz). **^{13}C NMR** (75 MHz, Chloroform-*d*) δ 162.6, 151.5, 150.4, 133.6, 133.0, 132.0, 128.4, 128.1, 127.8, 126.9 (q, J = 293.1 Hz), 126.1, 125.7, 125.4, 124.9, 123.8, 105.5, 98.5, 60.7 (q, J = 24.5 Hz), 58.3, 53.8, 41.4 (q, J = 1.8 Hz), 35.9. **IR** (ATR, cm^{-1}) 3119, 3036, 3002, 2952, 2897, 2856, 2819, 2253, 1595, 1561, 1509, 1475, 1414, 1388, 1357, 1323, 1265, 1248, 1164, 1124, 1103, 1066, 1052, 1012, 883, 862, 793, 777, 764, 717, 702, 639, 607, 586, 542, 442, 417. **HRMS** m/z $[M+H]^+$ calculated for $C_{22}H_{22}N_4F_3^+$: 415.1740, found: 415.1739.

*Synthesis of compound **61***
1,3-dimethyl-7-(3,3,3-trifluoro-2-(methyl(naphthalen-1-ylmethyl)amino)propyl)-3,7-dihydro-1*H*-purine-2,6-dione

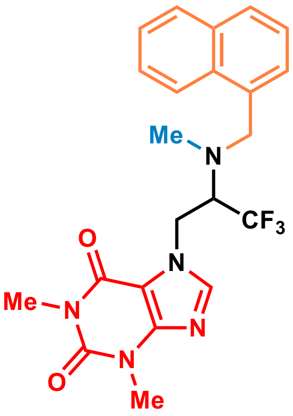

Compound **61** was prepared according to ***general procedure B*** from **1** (140 mg, 0.3 mmol), *N*-methyl-1-(naphthalen-1-yl)methanamine (51.4 mg, 0.3 mmol, 1 equiv), methyl theophylline (141 mg, 0.78 mmol, 2.6 equiv), solution of LiHMDS (750 µL, 1 M in THF, 0.75 mmol, 2.5 equiv) and HMPA (132 µL, 0.75 mmol, 2.5 equiv) to afford a white solid (81.0 mg, 0.182 mmol, 60% yield). **R_f**= 0.49 (dichloromethane:isopropanol= 1:1). **M.p.=** 215-218 °C. **LRMS** (EI, 70 eV): m/z (%): 355(1), 304(9), 193(4), 170(23), 170(9), 142(11), 141(100), 115(19), 109(4). **^{1}H NMR** (300 MHz, Chloroform-*d*) 7.66 (d, J = 7.6 Hz, 1H), 7.62 (d, J = 7.4 Hz, 1H), 7.40 (d, J = 8.4 Hz, 1H), 7.37 – 7.28 (m, 2H), 7.28 – 7.23 (m, 1H), 7.22 (s, 1H), 7.21 – 7.14 (m, 1H), 4.22 (d, J = 11.5 Hz, 2H), 4.07 (d, J = 13.1 Hz, 1H), 4.03 (s, 1H), 4.02 – 3.95 (m, 1H), 3.34 (s, 3H), 3.05 (s, 3H), 2.78 (q, J = 2.3 Hz, 3H). **^{19}F NMR** (282 MHz, Chloroform-*d*) δ -66.36 (d, J = 6.3 Hz). **^{13}C NMR** (75 MHz, Chloroform-*d*) δ 153.9, 151.0, 148.1, 141.3, 133.5, 132.4, 132.0, 129.1, 128.4, 127.9, 126.7 (d, J = 293.4 Hz), 125.8, 125.2, 125.0, 123.5, 105.9, 57.9, 57.9 (q, J = 24.8 Hz), 43.2, 36.5, 29.6, 27.6. **IR** (ATR, cm^{-1}) 3112, 3075, 2951, 2927, 2901, 2860, 2819, 2798, 1697, 1642, 1599, 1548, 1473, 1407, 1378, 1292, 1248, 1231, 1180, 1141, 1109, 1088, 1072, 1028, 970, 871, 784, 770, 759, 749, 702, 621, 590, 492, 417, 397. **HRMS** m/z $[M+H]^+$ calculated for $C_{22}H_{23}N_5O_2F_3^+$: 446.1798, found: 446.1797.

*Synthesis of compound **62***

methyl N^{α}-(*tert*-butoxycarbonyl)-1-(3,3,3-trifluoro-2-(methyl(naphthalen-1-ylmethyl)amino)propyl)-*L*-tryptophanate

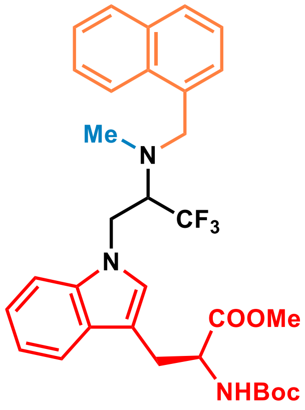

Compound **62** was prepared according to ***general procedure B*** from **1** (140 mg, 0.3 mmol), *N*-methyl-1-(naphthalen-1-yl)methanamine (51.4 mg, 0.3 mmol, 1 equiv), methyl *N*-((tert-butoxy)carbonyl)-*L*-tryptophanate (115 mg, 0.78 mmol, 2.6 equiv), solution of LiHMDS (750 µL, 1 M in THF, 0.75 mmol, 2.5 equiv) and HMPA (132 µL, 0.75 mmol, 2.5 equiv) to afford a colorless oil (56.8 mg, 0.0973 mmol, 32% yield). **R_f**= 0.29 (hexanes:ethyl acetate= 1:1). **LRMS** (EI, 70 eV): m/z (%): 489(1), 430(3), 207(5), 193(3), 171(2), 170(27), 170(10), 168(2), 168(2), 165(2), 142(8), 142(5), 141(100), 115(9), 73(2). **^{1}H NMR** (500 MHz, Chloroform-*d*) 7.78 (dt, *J* = 8.5, 1.6 Hz, 1H), 7.70 (dd, *J* = 8.4, 2.5 Hz, 1H), 7.62 (dd, *J* = 8.7, 3.9 Hz, 1H), 7.50 (td, *J* = 7.8, 7.3, 1.5 Hz, 1H), 7.41 (dddd, *J* = 8.1, 6.8, 2.3, 1.2 Hz, 1H), 7.24 – 7.15 (m, 2H), 7.13 – 7.03 (m, 3H), 6.84 (d, *J* = 8.0 Hz, 1H), 6.68 (d, *J* = 6.6 Hz, 1H), 4.98 (d, *J* = 8.3 Hz, 1H), 4.62 – 4.53 (m, 1H), 4.35 – 4.24 (m, 2H), 4.20 (d, *J* = 13.4 Hz, 1H), 4.17 – 4.13 (m, 1H), 3.70 (pt, *J* = 12.1, 7.0, 6.0 Hz, 1H), 3.62 (d, *J* = 6.1 Hz, 3H), 3.24 – 3.08 (m, 2H), 2.63 (dt, *J* = 3.6, 1.9 Hz, 3H), 1.42 (s, 9H).**^{19}F NMR** (282 MHz, Chloroform-*d*) δ -66.72 (d, *J* = 8.1 Hz). **^{13}C NMR** (75 MHz, Chloroform-*d*) δ 172.8, 155.3, 136.0, 133.9, 132.6, 132.0, 127.9, 126.8 (q, *J* = 292.2 Hz), 126.0, 125.7, 125.1, 123.9, 122.2, 119.6, 108.9, 79.9, 61.6 (d, *J* = 24.3 Hz), 58.6, 58.5, 54.3, 54.1, 52.3, 43.5, 36.2, 28.4. **IR** (ATR, cm^{-1}) 3431, 3379, 3051, 2976, 2951, 2931, 2861, 1741, 1710, 1614, 1497, 1468, 1439, 1367, 1251, 1161, 1102, 1054, 1017, 861, 793, 776, 739, 428. **HRMS** m/z $[M+H]^+$ calculated for $C_{32}H_{37}N_3O_4F_3^+$: 584.2731, found: 584.2730.

*Synthesis of compound **63***

1-(3,3,3-trifluoro-2-(methyl(naphthalen-1-ylmethyl)amino)propyl)pyridine-2(1*H*)-thione

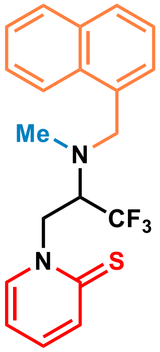

Compound **63** was prepared according to ***general procedure B*** from **1** (140 mg, 0.3 mmol), *N*-methyl-1-(naphthalen-1-yl)methanamine (51.4 mg, 0.3 mmol, 1 equiv), 2-mercaptopyridine (86.7 mg, 0.78 mmol, 2.6 equiv), solution of LiHMDS (750 µL, 1 M in THF, 0.75 mmol, 2.5 equiv) and HMPA (132 µL, 0.75 mmol, 2.5 equiv) to afford a yellow oil (78.5 mg, 0.209 mmol, 69% yield). **R_f**= 0.59 (hexanes:ethyl acetate= 1:1). **LRMS** (EI, 70 eV): m/z (%): 265(8), 235(6), 196(5), 169(6), 168(22), 142(12), 141(100), 139(6), 115(23), 112(7), 111(6), 78(6) **^{1}H NMR** (300 MHz,

Chloroform-*d*) 8.36 (dd, *J* = 5.1, 1.9 Hz, 1H), 8.26 – 8.19 (m, 1H), 7.83 (dt, *J* = 8.3, 3.0 Hz, 1H), 7.76 (d, *J* = 8.3 Hz, 1H), 7.53 – 7.46 (m, 3H), 7.45 – 7.34 (m, 2H), 7.06 (d, *J* = 8.1 Hz, 1H), 6.95 (dd, *J* = 7.4, 4.9 Hz, 1H), 4.40 (d, *J* = 13.7 Hz, 1H), 4.34 (d, *J* = 13.7 Hz, 1H), 3.84 – 3.66 (m, 2H), 3.45 (dd, *J* = 14.6, 11.3 Hz, 1H), 2.52 (d, *J* = 2.2 Hz, 3H). **^{19}F NMR** (282 MHz, Chloroform-*d*) δ -67.71 (d, *J* = 7.7 Hz). **^{13}C NMR** (75 MHz, Chloroform-*d*) δ 158.2, 149.3, 132.3, 128.4, 128.2, 127.5, 127.2 (q, *J* = 292.4 Hz), 125.8, 125.7, 125.2, 124.9, 122.2, 62.5 (q, *J* = 25.2 Hz), 58.4, 36.1, 27.2. **IR** (ATR, cm^{-1}) 3047, 2988, 2942, 2863, 2810, 2253, 1578, 1557, 1510, 1455, 1415, 1374, 1327, 1252, 1166, 1123, 1103, 1072, 1010, 985, 916, 852, 793, 781, 757, 733, 722, 689, 479, 415. **HRMS** m/z [M+H]$^+$ calculated for $C_{20}H_{20}N_2SF_3^+$: 377.1294, found: 377.1298.

*Synthesis of compound **64***

1,1,1-trifluoro-N-methyl-N-(naphthalen-1-ylmethyl)-3-(10H-phenothiazin-10-yl)propan-2-amine

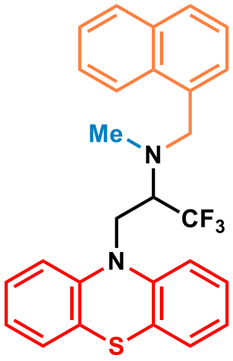

Compound **64** was prepared according to ***general procedure B*** from **1** (140 mg, 0.3 mmol), *N*-methyl-1-(naphthalen-1-yl)methanamine (51.4 mg, 0.3 mmol, 1 equiv), phenothiazine (159 mg, 0.78 mmol, 2.6 equiv), solution of LiHMDS (750 µL, 1 M in THF, 0.75 mmol, 2.5 equiv) and HMPA (132 µL, 0.75 mmol, 2.5 equiv) to afford a yellow oil (88.2 mg, 0.190 mmol, 63% yield). **R_f**= 0.63 (hexanes:ethyl acetate= 1:1). **LRMS** (EI, 70 eV): m/z (%): 465(5), 464(15), 213(14), 212(100), 212(19), 181(7), 180(49), 179(9), 141(34), 115(12). **^{1}H NMR** (300 MHz, Chloroform-*d*) 8.24 – 8.11 (m, 1H), 7.93 – 7.82 (m, 1H), 7.76 (d, *J* = 8.2 Hz, 1H), 7.53 (tt, *J* = 7.1, 3.7 Hz, 2H), 7.36 – 7.23 (m, 3H), 7.22 (t, *J* = 7.5 Hz, 1H), 7.19 (t, *J* = 7.8 Hz, 1H), 7.10 – 6.99 (m, 3H), 6.92 (s, 1H), 6.89 (s, 1H), 4.40 (dd, *J* = 12.0 Hz, 2H), 4.27 (d, *J* = 13.5 Hz, 1H), 4.24 (dd, *J* = 14.1, 3.9 Hz, 1H), 3.99 (ddq, *J* = 14.3, 8.9, 5.3, 4.6 Hz, 1H), 2.51 (s, 3H). **^{19}F NMR** (282 MHz, Chloroform-*d*) δ -69.80 (d, *J* = 8.4 Hz). **^{13}C NMR** (75 MHz, Chloroform-*d*) δ 144.8, 134.0, 133.9, 132.3, 128.4, 128.1, 127.7, 127.4, 126.7 (q, *J* = 287.8 Hz), 126.4, 125.8, 125.6, 125.2, 124.6, 123.3, 115.4, 59.0, 58.9 (q, *J* = 25.6 Hz), 43.1, 36.9. **IR** (ATR, cm^{-1}) 3063, 2952, 2884, 2864, 2807, 2252, 1592, 1571, 1456, 1337, 1279, 1251, 1231, 1207, 1158, 1099, 1049, 1028, 900, 856, 791, 774, 749, 604, 508, 445, 427. **HRMS** m/z [M+H]$^+$ calculated for $C_{27}H_{24}N_2SF_3^+$: 465.1607, found: 465.1610.

*Synthesis of compound **65***

*N*²-cyclopropyl-3,3-trifluoro-*N*²-(4-methoxybenzyl)-*N*¹-methyl-*N*¹-(naphthalen-1-ylmethyl)propane-1,2-diamine

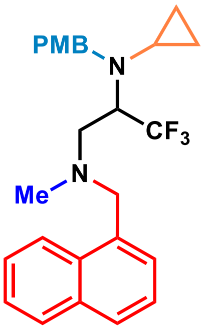

Compound **65** was prepared according to ***general procedure A*** from **1** (140 mg, 0.3 mmol), *N*-(4-methoxybenzyl)cyclopropanamine (53.2 mg, 0.3 mmol, 1 equiv) and *N*-methyl-1-(naphthalen-1-yl)methanamine (122 µL, 0.75 mmol, 2.5 equiv) to afford a white solid (68.9 mg, 0.156 mmol, 52% yield). **R**$_f$= 0.38 (hexanes:ethyl acetate= 10:1). **LRMS** (EI, 70 eV): m/z (%): 321(1), 301(4), 265(1), 212(2), 185(11), 184(78), 176(4), 142(20), 141(100), 141(66), 139(4), 122(5), 121(51), 115(13), 91(4), 78(4), 77(5). **^{1}H NMR** (250 MHz, Chloroform-*d*) 8.43 – 8.29 (m, 1H), 7.95 – 7.75 (m, 2H), 7.60 – 7.37 (m, 4H), 7.04 (d, *J* = 8.2 Hz, 2H), 6.75 (d, *J* = 8.5 Hz, 2H), 4.10 (d, *J* = 12.9 Hz, 1H), 3.86 – 3.73 (m, 4H), 3.66 (s, 2H), 3.55 (ddt, *J* = 15.6, 6.7, 4.0 Hz, 1H), 3.13 (dd, *J* = 13.2, 9.4 Hz, 1H), 2.57 (dd, *J* = 13.2, 3.8 Hz, 1H), 2.27 (s, 3H), 2.24 – 2.15 (m, 1H), 0.33 – 0.07 (m, 4H). **^{19}F NMR** (235 MHz, Chloroform-*d*) δ -69.20. **^{13}C NMR** (63 MHz, Chloroform-*d*) δ 158.6, 134.5, 134.0, 132.7, 132.2, 130.2, 128.5, 128.3, 127.7, 127.1 (q, *J* = 287.8 Hz), 125.8, 125.3, 125.2, 113.4, 61.8, 60.9 (q, *J* = 24.8 Hz), 55.4, 55.3, 54.5, 42.6, 35.0, 7.8, 7.7. **IR** (ATR, cm^{-1}) 3048, 3005, 2951, 2895, 2860, 2826, 2746, 1771, 1707, 1616, 1598, 1510, 1466, 1428, 1405, 1377, 1334, 1252, 1226, 1192, 1161, 1103, 1055, 1018, 970, 953, 875, 801, 786, 770, 726, 713, 702, 614, 529, 509, 449, 432. **HRMS** m/z [M+H]$^+$ calculated for $C_{26}H_{30}N_2OF_3^+$: 443.2310, found: 443.2313.

*Synthesis of compound **66***

*N*²-allyl-3,3,3-trifluoro-*N*²-(4-methoxybenzyl)-*N*¹-methyl-*N*¹-(naphthalen-1-ylmethyl)propane-1,2-diamine

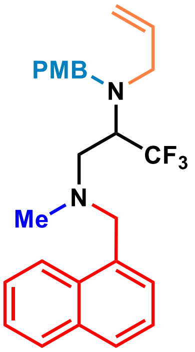

Compound **66** was prepared according to ***general procedure A*** from **1** (93.2 mg, 0.2 mmol), **A1** (35.5 mg, 0.2 mmol, 1 equiv) and *N*-methyl-1-(naphthalen-1-yl)methanamine (81.5 µL, 0.5 mmol, 2.5 equiv) to afford a colorless oil (64.6 mg, 0.146 mmol, 73% yield). **R**$_f$= 0.50 (hexanes:ethyl acetate= 10:1). **LRMS** (EI, 70 eV): m/z (%): 321(2), 185(8), 184(54), 142(12), 141(100), 139(2), 122(2), 121(24), 115(8), 78(2), 77(2). **^{1}H NMR** (250 MHz, Chloroform-*d*) 8.44 – 8.31 (m, 1H), 7.99 – 7.77 (m, 2H), 7.62 – 7.48 (m, 2H), 7.52 – 7.38 (m, 2H), 7.13 (d, *J* = 8.3 Hz, 2H), 6.81 (d, *J* = 8.5 Hz, 2H), 5.69 (ddt, *J* = 16.6, 10.1, 6.4 Hz, 1H), 5.20 – 5.02 (m, 2H), 4.12 (d, *J* = 12.9 Hz, 1H), 3.79 (s, 4H), 3.64 – 3.44 (m, 3H), 3.09 (dt, *J* = 27.5, 7.6 Hz, 3H), 2.60 (dd, *J* = 13.3, 3.8 Hz, 1H), 2.29 (s, 3H). **^{19}F NMR** (235 MHz, Chloroform-*d*) δ -69.11. **^{13}C**

NMR (63 MHz, Chloroform-*d*) 158.8, 137.0, 134.5, 134.1, 132.7, 131.6, 129.9, 128.5, 128.3, 127.8, 127.3 (q, *J* = 288.5 Hz), 125.9, 125.8, 125.3, 117.4, 113.7, 61.7, 57.3 (q, *J* = 24.6 Hz), 55.3, 54.2, 53.5, 53.4, 42.7. **IR** (ATR, cm^{-1}) 3067, 3047, 3031, 3002, 2952, 2905, 2836, 2815, 2789, 1642, 1612, 1585, 1510, 1463, 1365, 1302, 1246, 1207, 1170, 1149, 1126, 1096, 1036, 1010, 994, 909, 885, 831, 791, 776, 732, 703, 649, 603, 554, 519, 414, 432. **HRMS** m/z $[M+H]^+$ calculated for $C_{26}H_{30}N_2OF_3^+$: 443.2310, found: 443.2309.

*Synthesis of compound **67***

3,3,3-trifluoro-N^2-(4-methoxybenzyl)-N^1-methyl-N^1-(naphthalen-1-ylmethyl)-N^2-(prop-2-yn-1-yl)propane-1,2-diamine

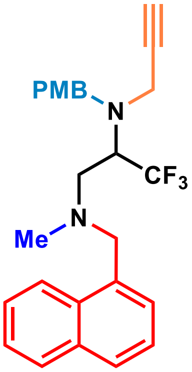

Compound **67** was prepared according to ***general procedure A*** from **1** (140 mg, 0.3 mmol), *N*-(4-methoxybenzyl)prop-2-yn-1-amine (52.6 mg, 0.3 mmol, 1 equiv) and *N*-methyl-1-(naphthalen-1-yl)methanamine (122 µL, 0.75 mmol, 2.5 equiv) to afford a white solid (57.0 mg, 0.129 mmol, 43% yield). $\mathbf{R_f}$= 0.43 (hexanes:diisopropyl ether= 10:1). **LRMS** (EI, 70 eV): m/z (%): 355(1), 299(1), 185(13), 184(88), 142(13), 142(8), 141(100), 141(68), 139(5), 121(37), 115(16), 77(4). **^{1}H NMR** (250 MHz, Chloroform-*d*) 8.40 – 8.29 (m, 1H), 7.96 – 7.78 (m, 2H), 7.58 – 7.49 (m, 1H), 7.48 (t, *J* = 7.8 Hz, 3H), 7.10 (d, *J* = 8.4 Hz, 2H), 6.80 (d, *J* = 8.5 Hz, 2H), 4.16 (d, *J* = 12.9 Hz, 1H), 3.87 (t, *J* = 10.6 Hz, 2H), 3.79 (s, 3H), 3.55 (dd, *J* = 17.8, 13.5 Hz, 2H), 3.47 (d, *J* = 17.3 Hz, 1H), 3.29 (d, *J* = 17.1 Hz, 1H), 3.11 (dd, *J* = 13.1, 9.3 Hz, 1H), 2.67 (dd, *J* = 13.3, 3.3 Hz, 1H), 2.37 (s, 3H), 2.21 (d, *J* = 9.0 Hz, 1H). **^{19}F NMR** (235 MHz, Chloroform-*d*) δ -69.40. **^{13}C NMR** (63 MHz, Chloroform-*d*) δ 158.9, 134.4, 134.0, 132.6, 130.5, 129.9, 128.6, 128.4, 127.9, 126.9 (q, *J* = 288.1, 287.6, 287.6 Hz), 126.0, 125.8, 125.3, 125.2, 113.8, 80.9, 72.6, 61.5, 58.1 (q, *J* = 25.2 Hz), 55.3, 53.8, 53.1, 42.6, 40.0. **HRMS** m/z $[M+H]^+$ calculated for $C_{26}H_{28}N_2OF_3^+$: 441.2154, found: 441.2148.

Synthesis of compound ***68***

3,3,3-trifluoro-N^2-isobutyl-N^1-methyl-N^1-(naphthalen-1-ylmethyl)-N^2-(3-phenylprop-2-yn-1-yl)propane-1,2-diamine

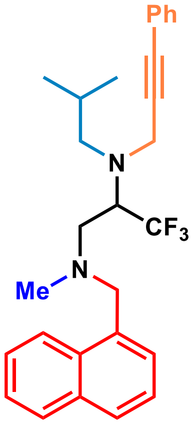

Compound **68** was prepared according to ***general procedure A*** from **1** (140 mg, 0.3 mmol), *N*-isobutyl-3-phenylprop-2-yn-1-amine (56.2 mg, 0.3 mmol, 1 equiv) and *N*-methyl-1-(naphthalen-1-yl)methanamine (122 µL, 0.75 mmol, 2.5 equiv) to afford a white solid (49.7 mg, 0.11 mmol, 37% yield). **R**$_f$= 0.60 (hexanes:diisopropyl ether= 10:1). **LRMS** (EI, 70 eV): m/z (%): 311(2), 200(2), 185(13), 184(92), 142(16), 142(6), 141(100), 141(67), 140(3), 139(4), 116(3), 116(2), 115(53), 89(2), 73(2). **^{1}H NMR** (250 MHz, Chloroform-*d*) 8.25 – 8.12 (m, 1H), 7.82 – 7.59 (m, 2H), 7.30 (ddd, *J* = 21.7, 10.0, 5.6 Hz, 6H), 7.15 (s, 3H), 3.97 (d, *J* = 13.0 Hz, 1H), 3.78 (d, *J* = 13.0 Hz, 1H), 3.70 – 3.36 (m, 3H), 2.90 (dd, *J* = 13.2, 8.2 Hz, 1H), 2.58 (dd, *J* = 13.3, 3.8 Hz, 1H), 2.24 (s, 5H), 1.59 (dp, *J* = 13.6, 7.2 Hz, 1H), 0.76 (d, *J* = 6.4 Hz, 3H), 0.67 (d, *J* = 6.4 Hz, 3H). **^{19}F NMR** (235 MHz, Chloroform-*d*) δ -69.38. **^{13}C NMR** (63 MHz, Chloroform-*d*) δ 134.5, 134.0, 132.6, 131.7, 128.5, 128.4, 128.1, 127.6, 127.0 (q, *J* = 288.4, 287.7 Hz), 125.9, 125.8, 125.3, 125.0, 123.4, 86.7, 84.4, 61.4, 60.5 (q, *J* = 25.0 Hz), 58.5, 54.4, 42.6, 41.7, 26.6, 20.6, 20.4. **IR** (ATR, cm^{-1}) 3054, 2955, 2945, 2928, 2915, 2908, 2868, 2849, 2820, 1716, 1598, 1510, 1490, 1465, 1443, 1365, 1255, 1160, 1097, 1054, 1010, 907, 899, 885, 861, 841, 793, 776, 756, 732, 691, 649, 645, 621, 605, 554, 526, 430, 415. **HRMS** m/z [M+H]$^+$ calculated for $C_{28}H_{32}N_2F_3^+$: 453.2518, found: 453.2516.

Synthesis of compound ***69***

2-(3-((*tert*-butyldimethylsilyl)oxy)azetidin-1-yl)-3,3,3-trifluoro-*N*-methyl-*N*-(naphthalen-1-ylmethyl)propan-1-amine

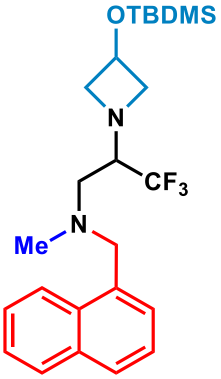

Compound **69** was prepared according to ***general procedure A*** from **1** (140 mg, 0.3 mmol), 3-((*tert*-butyldimethylsilyl)oxy)azetidine (56.2 mg, 0.3 mmol, 1 equiv) and *N*-methyl-1-(naphthalen-1-yl)methanamine (122 µL, 0.75 mmol, 2.5 equiv) to afford a white solid (71.5 mg, 0.158 mmol, 53% yield). **R**$_f$= 0.48 (hexanes:ethyl acetate= 10:1). **LRMS** (EI, 70 eV): m/z (%): 268(1), 185(10), 184(69), 168(1), 142(12), 141(100), 140(2), 115(4), 115(3), 110(2), 101(3), 101(2), 75(2), 73(5), 59(4). **^{1}H NMR** (250 MHz, Chloroform-*d*) 8.26 (d, *J* = 7.7 Hz, 1H), 7.94 – 7.72 (m, 2H), 7.61 – 7.35 (m, 4H), 4.31 (p, *J* = 6.2 Hz, 1H), 4.09 (d, *J* = 12.9 Hz, 1H), 3.76 (d, *J* = 13.0 Hz, 1H), 3.71 – 3.60 (m, 1H), 3.30 (td, *J* = 6.5, 2.6 Hz, 1H), 3.15 (t, *J* = 6.7 Hz, 1H), 3.06 – 2.89 (m, 2H), 2.65 (qd, *J* = 13.6, 4.8 Hz, 2H), 2.32 (s, 3H), 0.88 (s, 9H), -0.00

(s, 6H). **^{19}F NMR** (235 MHz, Chloroform-*d*) *δ* -72.80. **^{13}C NMR** (63 MHz, Chloroform-*d*) *δ* 134.0, 134.0, 132.6, 128.6, 128.4, 127.9, 126.0, 125.8, 125.2, 124.8, 123.8 (q, *J* = 283.1, 282.7, 282.1 Hz), 65.1, 64.9 (q, *J* = 25.2 Hz), 62.8, 62.5, 61.5, 55.7 (q, *J* = 2.2, 1.7 Hz), 43.2, 25.9, 18.1, -4.9, -5.0. **IR** (ATR, cm^{-1}) 3094, 3047, 2953, 2929, 2887, 2854, 2789, 2773, 2712, 1510, 1463, 1377, 1304, 1259, 1218, 1134, 1119, 1079, 1007, 939, 883, 835, 791, 774, 733, 698, 674, 414, 397. **HRMS** m/z [M+H]$^+$ calculated for $C_{24}H_{36}N_2OF_3Si^+$: 453.2549, found: 453.2548.

*Synthesis of compound **70***

3,3,3-trifluoro-*N*-methyl-*N*-(naphthalen-1-ylmethyl)-2-(pyrrolidin-1-yl)propan-1-amine

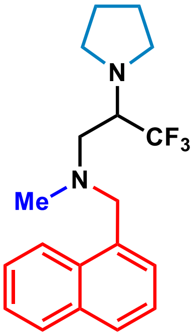

Compound **70** was prepared according to ***general procedure A*** from **1** (93.2 mg, 0.2 mmol), pyrrolidine (14.2 mg, 0.2 mmol, 1 equiv) and *N*-methyl-1-(naphthalen-1-yl)methanamine (81.5 µL, 0.5 mmol, 2.5 equiv) to afford a colorless oil (45.9 mg, 0.136 mmol, 68% yield). **R$_f$**= 0.43 (hexanes:ethyl acetate= 10:1). **M.p.= 5**1-54 °C. **LRMS** (EI, 70 eV): m/z (%): 296(1), 210(1), 185(10), 184(66), 142(13), 141(100), 140(2), 139(2), 115(9), 91(2), 75(2), 73(8). **^{1}H NMR** (250 MHz, Chloroform-*d*) 8.41 – 8.27 (m, 1H), 7.99 – 7.73 (m, 2H), 7.61 – 7.37 (m, 4H), 4.09 (d, *J* = 12.8 Hz, 1H), 3.87 (d, *J* = 12.8 Hz, 1H), 3.58 (pd, *J* = 8.7, 3.6 Hz, 1H), 2.99 (dd, *J* = 13.3, 9.0 Hz, 1H), 2.88 – 2.71 (m, 4H), 2.63 (dd, *J* = 13.3, 3.5 Hz, 1H), 2.31 (s, 3H), 1.75 – 1.54 (m, 4H). **^{19}F NMR** (235 MHz, Chloroform-*d*) *δ* - 69.81. **^{13}C NMR** (63 MHz, Chloroform-*d*) *δ* 134.6, 134.0, 132.7, 128.5, 128.2, 127.7, 124.8 (q, *J* = 289.1 Hz), 61.3, 59.6 (q, *J* = 24.7 Hz), 54.3 – 53.6 (m), 48.7, 42.7, 24.1. **IR** (ATR, cm^{-1}) 3094, 3064, 3043, 2959, 2874, 2837, 2806, 2769, 2751, 2718, 1597, 1507, 1487, 1462, 1446, 1387, 1360, 1310, 1296, 1253, 1147, 1124, 1093, 1051, 1015, 971, 878, 859, 835, 794, 774, 705, 655, 613, 553, 520, 466, 411. **HRMS** m/z [M+H]$^+$ calculated for $C_{19}H_{24}N_2F_3^+$: 337.1892, found: 337.1885.

*Synthesis of compound **71***

3,3,3-trifluoro-*N*-methyl-2-(2-methylpiperidin-1-yl)-*N*-(naphthalen-1-ylmethyl)propan-1-amine

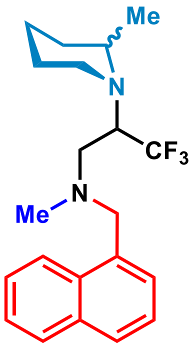

Compound **71** was prepared according to ***general procedure A*** from **1** (93.2 mg, 0.2 mmol), 2-methylpiperidine (19.8 mg, 0.2 mmol, 1 equiv) and *N*-methyl-1-(naphthalen-1-yl)methanamine (81.5 µL, 0.5 mmol, 2.5 equiv) to afford a colorless oil (61.7 mg, 0.169 mmol, 85% yield). **R$_f$**= 0.68 (hexanes:ethyl acetate= 10:1). **LRMS** (EI, 70 eV): m/z (%): 185(7), 184(47), 180(3), 142(13), 141(100), 140(2), 139(3), 115(11), 112(3), 110(2), 55(6). **^{1}H NMR** (250 MHz, Chloroform-*d*) 8.55 – 8.27 (m, 1H), 7.97 – 7.74 (m, 2H), 7.62 – 7.36 (m, 4H), 4.24 – 3.77 (m,

2H), 3.77 – 3.58 (m, 1H), 3.22 – 2.80 (m, 2H), 2.78 – 2.42 (m, 2H), 2.43 – 2.19 (m, 3H), 2.02 (td, *J* = 10.9, 4.6 Hz, 1H), 1.82 – 1.22 (m, 6H), 1.17 (d, *J* = 6.3 Hz, 1H), 1.04 (d, *J* = 6.1 Hz, 1H), 0.89 (dtd, *J* = 24.7, 12.4, 6.3 Hz, 1H). **^{19}F NMR** (235 MHz, Chloroform-*d*) δ -66.66, -70.84 (d. r.= 46:54). **^{13}C NMR** (63 MHz, Chloroform-*d*) δ 134.5, 134.0, 134.0, 132.7, 132.7, 128.5, 128.4, 128.3, 128.1, 127.8, 127.4, 126.6 (q, *J* = 284.8, 283.8, 283.5 Hz), 125.8, 125.7, 125.4, 125.2, 125.1, 61.9, 61.3, 57.5 (q, *J* = 22.8 Hz), 57.0 (q, *J* = 25.4 Hz), 55.6, 54.6, 52.4, 46.5 (q, *J* = 1.8 Hz), 46.4, 42.9, 42.7, 36.5, 36.3, 26.8, 26.5, 25.2, 24.4, 21.0, 20.3. **IR** (ATR, cm^{-1}) 3047, 2966, 2931, 2847, 2813, 1510, 1453, 1377, 1343, 1309, 1256, 1231, 1146, 1127, 1099, 1061, 1010, 967, 907, 889, 858, 829, 791, 774, 733, 698, 649, 624, 557, 520, 457, 414, 655, 613, 553, 520, 466, 411. **HRMS** m/z [M+H]$^+$ calculated for $C_{21}H_{28}N_2F_3^+$: 365.2205, found: 365.2205.

*Synthesis of compound **72***

2-(3-((*tert*-butyldimethylsilyl)oxy)piperidin-1-yl)-3,3,3-trifluoro-*N*-methyl-*N*-(naphthalen-1-ylmethyl)propan-1-amine

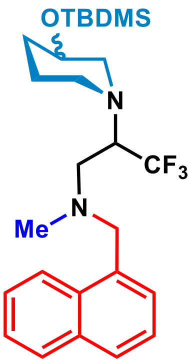

Compound **72** was prepared according to ***general procedure A*** from **1** (93.2 mg, 0.2 mmol), 3-((*tert*-butyldimethylsilyl)oxy)piperidine (43.1 mg, 0.2 mmol, 1 equiv) and *N*-methyl-1-(naphthalen-1-yl)methanamine (81.5 µL, 0.5 mmol, 2.5 equiv) to afford a colorless oil (55.6 mg, 0.116 mmol, 58% yield). **R$_f$**= 0.38 (hexanes:ethyl acetate= 10:1). **M.p.=** 64-67 °C. **LRMS** (EI, 70 eV): m/z (%): 296(1), 210(1), 185(10), 184(66), 144(1), 142(13), 141(100), 140(2), 139(2), 118(1), 115(9), 91(2), 77(1), 75(2), 73(8), 68(1), 57(1). **^{1}H NMR** (250 MHz, Methanol-*d*$_4$ & Acetone-*d*$_6$) δ 8.35 (d, *J* = 7.9 Hz, 1H), 7.92 – 7.70 (m, 2H), 7.45 (dt, *J* = 17.3, 7.4 Hz, 4H), 4.09 (d, *J* = 12.8 Hz, 1H), 3.85 (d, *J* = 12.7 Hz, 1H), 3.50 – 3.24 (m, 2H), 3.04 – 2.80 (m, 2H), 2.68 – 2.39 (m, 3H), 2.33 (s, 4H), 1.65 – 1.23 (m, 4H), 0.88 (s, 9H), 0.03 (s, 6H). **^{19}F NMR** (235 MHz, Methanol-*d*$_4$ & Acetone-*d*$_6$) δ -69.95. **^{13}C NMR** (63 MHz, Methanol-*d*$_4$ & Acetone-*d*$_6$) δ 135.4, 133.9, 129.4, 129.3, 128.9, 128.3 (q, *J* = 290.5, 289.9 Hz), 126.8, 126.3, 126.1, 69.9, 64.6 (q, *J* = 24.1 Hz), 62.1, 54.0, 47.6, 43.1, 36.9, 36.7, 26.4, 18.9, -4.4. **IR** (ATR, cm^{-1}) 3044, 2959, 2876, 2840, 2809, 2771, 2738, 2717, 1686, 1597, 1509, 1462, 1448, 1419, 1387, 1360, 1310, 1255, 1175, 1149, 1124, 1093, 1051, 1017, 971, 878, 859, 837, 794, 776, 737, 705, 655, 613, 522, 468, 466. **HRMS** m/z [M+H]$^+$ calculated for $C_{26}H_{40}N_2OF_3Si^+$: 481.2857, found: 481.2859.

*Synthesis of compound **73***

2-(4-benzylpiperazin-1-yl)-3,3,3-trifluoro-*N*-methyl-*N*-(naphthalen-1-ylmethyl)propan-1-amine

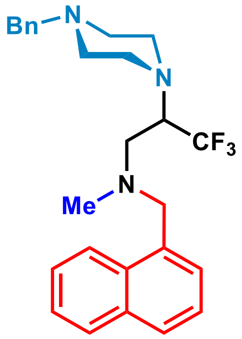

Compound **73** was prepared according to ***general procedure A*** from **1** (93.2 mg, 0.2 mmol), *N*-benzylpiperazine (35.3 mg, 0.2 mmol, 1 equiv) and *N*-methyl-1-(naphthalen-1-yl)methanamine (81.5 µL, 0.5 mmol, 2.5 equiv) to afford a colorless oil (63.9 mg, 0.145 mmol, 72% yield). **R$_f$**= 0.58 (hexanes:ethyl acetate= 7:3). **LRMS** (EI, 70 eV): m/z (%): 257(1), 185(7), 184(51), 177(2), 146(1), 142(12), 141(100), 140(1), 139(2), 115(7), 92(2), 91(24), 65(2), 56(1). **^{1}H NMR** (250 MHz, Chloroform-*d*) δ 8.48 – 8.34 (m, 1H), 7.97 – 7.77 (m, 2H), 7.61 – 7.43 (m, 3H), 7.47 – 7.22 (m, 5H), 4.12 – 3.92 (m, 2H), 3.52 (s, 2H), 3.29 (pd, *J* = 8.8, 3.3 Hz, 1H), 2.93 (dd, *J* = 13.7, 9.1 Hz, 1H), 2.85 (d, *J* = 4.7 Hz, 2H), 2.73 (q, *J* = 7.3, 5.9 Hz, 2H), 2.62 (dd, *J* = 13.4, 3.4 Hz, 1H), 2.41 (dq, *J* = 13.1, 5.7, 4.3 Hz, 4H), 2.33 (s, 3H). **^{19}F NMR** (235 MHz, Chloroform-*d*) δ -68.1. **^{13}C NMR** (63 MHz, Chloroform-*d*) δ 137.9, 134.7, 134.0, 132.6, 129.3, 128.5, 128.4, 128.2, 127.5, 127.3, 127.0 (d, *J* = 290.7 Hz), 125.9, 125.8, 125.2 (d, *J* = 1.6 Hz), 64.0 (q, *J* = 23.9 Hz), 63.1, 61.4, 53.8, 53.5 (d, *J* = 2.0 Hz), 49.2, 42.8. **IR** (ATR, cm^{-1}) 3061, 2969, 2956, 2941, 2901, 2844, 2810, 2769, 1509, 1494, 1455, 1394, 1367, 1351, 1295, 1255, 1217, 1156, 1124, 1102, 1076, 1052, 1028, 1010, 940, 907, 879, 858, 835, 793, 776, 732, 698, 638, 607, 519, 457, 414. **HRMS** m/z [M+H]$^+$ calculated for $C_{26}H_{31}N_3F_3^+$: 442.2470, found: 442.2474.

*Synthesis of compound **74***

3,3,3-trifluoro-*N*-methyl-*N*-(naphthalen-1-ylmethyl)-2-(4-(pyridin-2-yl)piperazin-1-yl)propan-1-amine

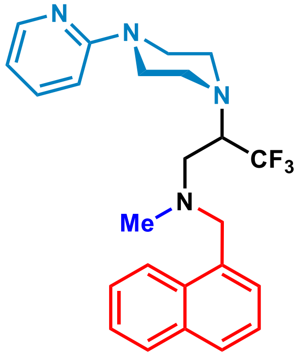

Compound **74** was prepared according to ***general procedure A*** from **1** (93.2 mg, 0.2 mmol), *N*-benzylpiperazine (35.3 mg, 0.2 mmol, 1 equiv) and *N*-methyl-1-(naphthalen-1-yl)methanamine (81.5 µL, 0.5 mmol, 2.5 equiv) to afford a colorless oil (69.7 mg, 0.163 mmol, 81% yield). **R$_f$**= 0.58 (hexanes:ethyl acetate= 7:3). **M.p.=** 92-95 °C. **LRMS** (EI, 70 eV): m/z (%): 185(7), 184(47), 142(12), 141(100), 139(2), 133(2), 121(3), 119(3), 115(8), 107(4), 79(5), 78(4). **^{1}H NMR** (250 MHz, Chloroform-*d*) δ 8.4 – 8.3 (m, 1H), 8.2 (dd, *J* = 5.0, 2.0 Hz, 1H), 7.8 (t, *J* = 9.3 Hz, 2H), 7.4 (dt, *J* = 7.8, 4.9 Hz, 5H), 6.6 (d, *J* = 7.0 Hz, 1H), 6.6 (d, *J* = 9.0 Hz, 1H), 4.1 (d, *J* = 12.9 Hz, 1H), 4.0 (d, *J* = 12.9 Hz, 1H), 3.6 – 3.2 (m, 5H), 3.0 (dd, *J* = 13.6, 9.3 Hz, 1H), 2.8 (t, *J* = 5.2 Hz, 2H), 2.8 (q, *J* = 6.7, 5.8

Hz, 2H), 2.6 (dd, *J* = 13.5, 3.5 Hz, 1H), 2.3 (s, 3H). **^{19}F NMR** (235 MHz, Chloroform-*d*) *δ* -68.3. **^{13}C NMR** (63 MHz, Chloroform-*d*) *δ* 159.4, 147.9, 137.6, 134.4, 134.0, 132.5, 128.5, 128.3, 127.6, 126.9 (q, *J* = 290.8 Hz), 125.9, 125.8, 125.2, 125.0, 113.2, 107.2, 64.1 (q, *J* = 24.1 Hz), 61.4, 53.4 (q, *J* = 2.0 Hz), 49.2, 46.1, 42.9. **IR** (ATR, cm^{-1}) 3058, 3036, 3005, 2978, 2959, 2949, 2918, 2887, 2854, 2834, 2782, 1737, 1594, 1563, 1509, 1479, 1435, 1380, 1363, 1336, 1310, 1258, 1239, 1150, 1126, 1100, 1025, 1014, 978, 951, 916, 878, 837, 771, 729, 712, 614, 550, 522, 445, 403. **HRMS** m/z [M+H]$^+$ calculated for $C_{24}H_{28}N_4F_3^+$: 429.2266, found: 429.2264.

*Synthesis of compound **75***

2-(3,4-dihydroisoquinolin-2(1*H*)-yl)-3,3,3-trifluoro-*N*-methyl-*N*-(naphthalen-1-ylmethyl)propan-1-amine

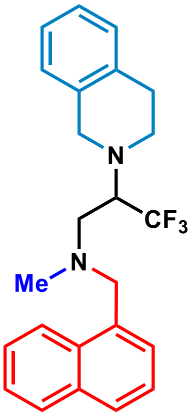

Compound **75** was prepared according to ***general procedure A*** from **1** (93.2 mg, 0.2 mmol), 1,2,3,4-tetrahydroisoquinoline (37.6 µL, 0.2 mmol, 1 equiv) and *N*-methyl-1-(naphthalen-1-yl)methanamine (81.5 µL, 0.5 mmol, 2.5 equiv) to afford a colorless oil (84.6 mg, 0.212 mmol, 71% yield). **R_f**= 0.40 (hexanes:ethyl acetate= 10:1). **LRMS** (EI, 70 eV): m/z (%): 257(1), 214(3), 185(4), 185(3), 184(47), 142(13), 141(100), 139(3), 117(5), 115(16), 105(3). **^{1}H NMR** (300 MHz, Chloroform-*d*) *δ* 8.35 (d, *J* = 8.6 Hz, 1H), 7.84 (d, *J* = 8.5 Hz, 1H), 7.80 (d, *J* = 8.5 Hz, 1H), 7.51 – 7.34 (m, 3H), 7.24 – 7.10 (m, 4H), 7.04 – 6.95 (m, 1H), 4.12 (d, *J* = 12.8 Hz, 1H), 4.11 (d, *J* = 14.5 Hz, 1H), 3.97 (d, *J* = 14.8 Hz, 1H), 3.96 (d, *J* = 12.8 Hz, 1H), 3.64 – 3.47 (m, 1H), 3.20 – 2.93 (m, 3H), 2.92 – 2.81 (m, 2H), 2.75 (dd, *J* = 13.5, 3.6 Hz, 1H), 2.34 (s, 3H). **^{19}F NMR** (282 MHz, Chloroform-*d*) *δ* -67.90 (d, *J* = 8.5 Hz). **^{13}C NMR** (75 MHz, Chloroform-*d*) *δ* 135.2, 134.6, 133.9, 132.5, 128.3, 128.2, 127.6, 127.2 (q, *J* = 291.4 Hz), 126.4, 126.0, 125.8, 125.7, 125.6, 125.1, 63.7 (q, *J* = 23.9 Hz), 61.2, 53.8, 51.6, 47.3, 42.8, 30.4. **IR** (ATR, cm^{-1}) 3047, 3023, 2921, 2819, 1597, 1509, 1499, 1465, 1453, 1392, 1253, 1147, 1100, 1051, 1038, 1018, 933, 883, 859, 829, 793, 776, 739, 716, 702, 550, 520, 431, 414. **HRMS** m/z [M+H]$^+$ calculated for $C_{24}H_{26}N_2F_3^+$: 399.2043, found: 399.2044.

*Synthesis of compound **76***

2-(3-azabicyclo[3.2.2]nonan-3-yl)-3,3,3-trifluoro-*N*-methyl-*N*-(naphthalen-1-ylmethyl)propan-1-amine

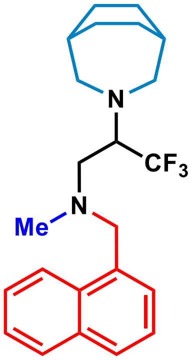

Compound **76** was prepared according to ***general procedure A*** from **1** (140 mg, 0.3 mmol), 3-azabicyclo[3.2.2]nonane (37.6 mg, 0.3 mmol, 1 equiv) and *N*-methyl-1-(naphthalen-1-yl)methanamine (122 µL, 0.75 mmol, 2.5 equiv) to afford a white solid (64.0 mg, 0.164 mmol, 55% yield). **R$_f$**= 0.70 (hexanes:ethyl acetate= 10:1). **M.p.=** 114-117 °C. **LRMS** (EI, 70 eV): m/z (%): 206(1), 185(9), 184(62), 142(13), 141(100), 139(3), 126(2), 115(9), 112(5), 67(4), 55(2). **^{1}H NMR** (250 MHz, Chloroform-*d*) 8.40 – 8.29 (m, 1H), 7.93 – 7.73 (m, 2H), 7.60 – 7.37 (m, 4H), 4.05 (d, *J* = 12.9 Hz, 1H), 3.94 (d, *J* = 12.9 Hz, 1H), 3.36 (tt, *J* = 11.3, 5.7 Hz, 1H), 2.97 (dd, *J* = 13.1, 8.7 Hz, 1H), 2.81 (qd, *J* = 11.7, 4.2 Hz, 4H), 2.59 (dd, *J* = 13.2, 3.6 Hz, 1H), 2.33 (s, 3H), 1.81 (s, 2H), 1.67 (d, *J* = 9.1 Hz, 4H), 1.63 – 1.44 (m, 4H). **^{19}F NMR** (235 MHz, Chloroform-*d*) δ -68.81. **^{13}C NMR** (63 MHz, Chloroform-*d*) δ 134.6, 134.0, 132.7, 129.8 (q, *J* = 292.0, 291.5 Hz), 128.5, 128.3, 127.7, 125.8, 125.2, 125.1, 64.0 (q, *J* = 24.2 Hz), 61.8, 59.0, 54.6, 42.3, 31.4, 25.6, 25.5. **IR** (ATR, cm^{-1}) 3064, 3037, 2996, 2921, 2857, 2827, 2802, 2765, 1509, 1458, 1449, 1402, 1382, 1358, 1344, 1300, 1256, 1173, 1151, 1134, 1102, 1089, 1051, 1011, 964, 882, 869, 859, 839, 793, 774, 708, 681, 614, 583, 552, 522, 464, 415. **HRMS** m/z [M+H]$^+$ calculated for $C_{23}H_{30}N_2F_3^+$: 391.2361, found: 391.2363.

*Synthesis of compound **77***

N,*N*-diethyl-1,1,1-trifluoro-3-(10*H*-phenothiazin-10-yl)propan-2-amine

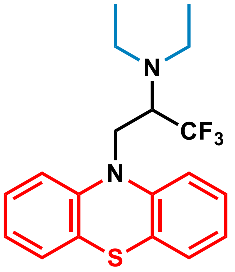

Compound **77** was prepared according to ***general procedure B*** from **1** (140 mg, 0.3 mmol), diethylamine (31 µL, 0.3 mmol, 1 equiv), phenothiazine (159 mg, 0.78 mmol, 2.6 equiv), solution of LiHMDS (750 µL, 1 M in THF, 0.75 mmol, 2.5 equiv) and HMPA (132 µL, 0.75 mmol, 2.5 equiv) to afford a white solid (68.9 mg, 0.188 mmol, 62% yield). **R$_f$**= 0.67 (hexanes:ethyl acetate= 3:1). **M.p.=** 65-69 °C **LRMS** (EI, 70 eV): m/z (%): 366(7), 293(2), 224(1), 213(15), 212(100), 198(9), 180(41), 179(7), 126(3), 98(3), 77(1), 56(1). **^{1}H NMR** (250 MHz, Methanol-*d$_4$*) δ 7.23 – 7.03 (m, 4H), 7.00 – 6.84 (m, 4H), 4.21 (dd, *J* = 14.6, 9.2 Hz, 1H), 4.12 (dd, *J* = 14.4, 4.4 Hz, 1H), 3.76 (pd, *J* = 8.8, 4.2 Hz, 1H), 2.61 (qd, *J* = 6.9, 2.8 Hz, 4H), 0.77 (t, *J* = 7.1 Hz, 6H). **^{19}F NMR** (235 MHz, Methanol-*d$_4$*) δ -71.60. **^{13}C NMR** (63 MHz, Methanol-*d$_4$*) δ 145.1, 127.6, 127.5, 127.4 (q, *J* = 289.0 Hz), 126.4, 123.0, 58.0 (q, *J* = 24.9 Hz), 44.6, 43.5 (q, *J* = 1.8

Hz), 13.8. **IR** (ATR, cm^{-1}) 3067, 2971, 2931, 2871, 2832, 1594, 1572, 1455, 1384, 1339, 1310, 1276, 1249, 1228, 1183, 1156, 1096, 1066, 1045, 1032, 929, 889, 845, 746, 729, 722, 706, 672, 603, 536, 509, 445. **HRMS** m/z $[M+H]^+$ calculated for $C_{19}H_{22}F_3N_2S^+$: 367.1450, found: 367.1452.

*Synthesis of compound **78***

1,1,1,3-tetrafluoro-*N*-methyl-*N*-(naphthalen-1-ylmethyl)propan-2-amine

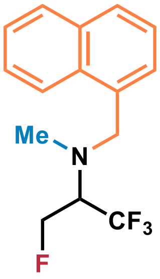

Compound **78** was prepared according to ***general procedure A*** from **1** (140 mg, 0.3 mmol), *N*-methyl-1-(naphthalen-1-yl)methanamine (51.4 mg, 0.3 mmol, 1 equiv) and cesium fluoride (114 mg, 0.75 mmol, 2.5 equiv) to afford a colorless oil (55.6 mg, 0.195 mmol, 65% yield). $\mathbf{R_f}$= 0.63 (hexanes:ethyl acetate= 10:1). **LRMS** (EI, 70 eV): m/z (%): 286(2), 285(14), 284(2), 252(2), 216(2), 168(2), 158(4), 142(17), 141(100), 140(2), 139(7), 127(2), 126(2), 116(2), 115(18), 89(2). **^{1}H NMR** (250 MHz, Chloroform-*d*) 8.31 – 8.18 (m, 1H), 7.97 – 7.79 (m, 2H), 7.64 – 7.46 (m, 3H), 7.52 – 7.39 (m, 1H), 4.92 – 4.73 (m, 1H), 4.74 – 4.54 (m, 1H), 4.35 (s, 2H), 3.61 (dqdd, *J* = 17.3, 8.6, 6.8, 4.9 Hz, 1H), 2.57 (s, 3H). **^{19}F NMR** (235 MHz, Chloroform-*d*) δ -67.89 (t, *J* = 7.7 Hz, 3F), -229.51 (tdq, *J* = 46.1, 17.8, 6.5 Hz, 1F). **^{13}C NMR** (63 MHz, Chloroform-*d*) δ 134.1, 133.6, 132.4, 128.7, 127.7, 126.1, 126.1 (qd, *J* = 289.2, 10.0 Hz), 125.9, 125.3, 124.5, 79.5 (qd, *J* = 174.7, 2.3 Hz), 62.5 (qd, *J* = 26.0, 20.8 Hz), 58.9, 37.5. **IR** (ATR, cm^{-1}) 3048, 2962, 2897, 2864, 2817, 1598, 1510, 1463, 1397, 1374, 1316, 1272, 1249, 1160, 1112, 1054, 1018, 852, 791, 774, 734, 708, 614, 586, 564, 544, 532, 519, 444, 415. **HRMS** m/z $[M+H]^+$ calculated for $C_{15}H_{16}NF_4^+$: 286.1219, found: 286.1219.

*Synthesis of compound **79***

3-chloro-1,1,1-trifluoro-*N*-methyl-*N*-(naphthalen-1-ylmethyl)propan-2-amine

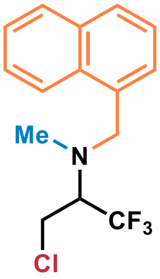

Compound **79** was prepared according to ***general procedure A*** from **1** (140 mg, 0.3 mmol), *N*-methyl-1-(naphthalen-1-yl)methanamine (51.4 mg, 0.3 mmol, 1 equiv) and tetrabutylammonium chloride (215 mg, 0.75 mmol, 2.5 equiv) to afford a colorless oil (72.3 mg, 0.239 mmol, 80% yield). $\mathbf{R_f}$= 0.68 (hexanes:ethyl acetate= 10:1). **LRMS** (EI, 70 eV): m/z (%): 303(3), 301(10), 252(4), 232(3), 142(16), 141(100), 139(7), 115(17). **^{1}H NMR** (250 MHz, Chloroform-*d*) 8.28 (dd, *J* = 7.2, 1.8 Hz, 1H), 7.92 (dt, *J* = 6.4, 2.5 Hz, 1H), 7.85 (d, *J* = 8.2 Hz, 1H), 7.58 (qd, *J* = 4.3, 1.9 Hz, 2H), 7.52 (d, *J* = 5.5 Hz, 1H), 7.46 (d, *J* = 7.2 Hz, 1H), 4.42 (dd, *J* = 13.5, 2.8 Hz, 2H), 3.91 – 3.70 (m, 2H), 3.63 (pd, *J* = 8.1, 4.6 Hz, 1H), 2.53 (s, 3H). **^{19}F NMR** (235 MHz, Chloroform-*d*) δ -68.17. **^{13}C NMR** (63 MHz, Chloroform-*d*) δ 134.0, 133.7, 132.3, 128.6, 128.5, 127.6, 126.0, 125.9, 125.4 (d,

J = 290.8 Hz), 125.3, 124.6, 65.2 (q, *J* = 25.9 Hz), 58.6, 39.2 (d, *J* = 2.0 Hz), 36.1. **IR** (ATR, cm^{-1}) 3048, 2945, 2863, 2816, 1598, 1510, 1477, 1448, 1374, 1337, 1256, 1171, 1137, 1105, 1083, 1017, 970, 940, 784, 770, 733, 688, 649, 576, 542, 519, 415. **HRMS** m/z $[M+H]^+$ calculated for $C_{15}H_{16}N^{37}ClF_3^+$: 304.0894, found: 304.0893.

Synthesis of compound ***80***
3-bromo-1,1,1-trifluoro-*N*-methyl-*N*-(naphthalen-1-ylmethyl)propan-2-amine

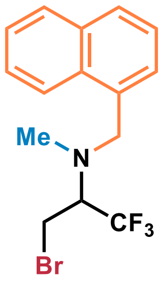

Compound **80** was prepared according to ***general procedure A*** from **1** (140 mg, 0.3 mmol), *N*-methyl-1-(naphthalen-1-yl)methanamine (51.4 mg, 0.3 mmol, 1 equiv) and tetrabutylammonium bromide (244 mg, 0.75 mmol, 2.5 equiv) to afford a colorless oil (74.1 mg, 0.214 mmol, 71% yield). $\mathbf{R_f}$= 0.75 (hexanes:ethyl acetate= 10:1). **LRMS** (EI, 70 eV): m/z (%): 347(4), 345(4), 278(4), 276(4), 252(3), 218(1), 168(2), 142(16), 141(100), 140(2), 139(8), 127(2), 126(2), 116(2), 115(17), 89(1), 63(1). **^{1}H NMR** (250 MHz, Chloroform-*d*) 8.35 – 8.21 (m, 1H), 7.97 – 7.79 (m, 2H), 7.69 – 7.30 (m, 4H), 4.42 (dd, *J* = 13.5, 3.9 Hz, 2H), 3.79 – 3.61 (m, 1H), 3.67 – 3.54 (m, 2H), 2.55 – 2.47 (m, 3H). **^{19}F NMR** (235 MHz, Chloroform-*d*) δ -67.98. **^{13}C NMR** (63 MHz, Chloroform-*d*) δ 134.0, 133.6, 132.3, 128.6, 128.5, 127.6, 126.0, 125.9, 125.4 (d, *J* = 291.7 Hz), 125.3, 124.7, 65.2 (q, *J* = 25.8 Hz), 58.3, 35.9, 25.9 (q, *J* = 1.7 Hz). **IR** (ATR, cm^{-1}) 3048, 2979, 2944, 2860, 2813, 1598, 1510, 1453, 1373, 1326, 1249, 1167, 1102, 1075, 1008, 968, 913, 858, 844, 791, 780, 771, 732, 658, 641, 573, 539, 518, 415. **HRMS** m/z $[M+H]^+$ calculated for $C_{15}H_{16}N^{81}BrF_3^+$: 348.0398, found: 348.0401.

Synthesis of compound ***81***
1,1,1-trifluoro-3-iodo-*N*-methyl-*N*-(naphthalen-1-ylmethyl)propan-2-amine

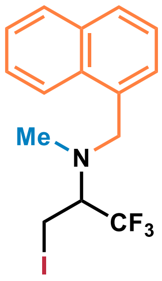

Compound **81** was prepared according to ***general procedure A*** from **1** (140 mg, 0.3 mmol), *N*-methyl-1-(naphthalen-1-yl)methanamine (51.4 mg, 0.3 mmol, 1 equiv) and sodium iodide (114 mg, 0.75 mmol, 2.5 equiv) to afford a colorless oil (91.8 mg, 0.234 mmol, 78% yield). $\mathbf{R_f}$= 0.77 (hexanes:ethyl acetate= 10:1). **LRMS** (EI, 70 eV): m/z (%): 393(5), 324(11), 252(5), 223(1), 196(1), 168(4), 142(14), 141(100), 139(8), 115(19), 98(2), 77(1). **^{1}H NMR** (250 MHz, Acetone-d_6) 8.36 – 8.25 (m, 1H), 7.98 – 7.80 (m, 2H), 7.64 (d, *J* = 7.0 Hz, 1H), 7.61 – 7.40 (m, 3H), 4.45 (s, 2H), 3.81 (td, *J* = 8.1, 6.1 Hz, 1H), 3.65 – 3.45 (m, 2H), 2.43 (t, *J* = 1.8 Hz, 3H). **^{19}F NMR** (235 MHz, Acetone-d_6) δ -68.19. **^{13}C NMR** (63 MHz, Acetone-d_6) δ 134.9, 134.8, 133.1, 129.2, 129.0, 128.1, 126.6, 126.5, 126.1 (q, *J* = 292.6 Hz), 126.0, 125.8, 66.3 (q, *J* = 25.4 Hz), 58.3 (d, *J* = 1.4 Hz),

35.6, -2.8. **IR** (ATR, cm^{-1}) 3047, 2979, 2941, 2857, 2810, 1598, 1510, 1463, 1452, 1373, 1319, 1246, 1160, 1102, 1071, 1018, 1000, 888, 855, 837, 791, 777, 732, 710, 621, 605, 570, 537, 518, 414. **HRMS** m/z [M+H]$^+$ calculated for $C_{15}H_{16}NIF_3^+$: 394.0280, found: 394.0280.

*Synthesis of compound **82***

3-azido-1,1,1-trifluoro-*N*-methyl-*N*-(naphthalen-1-ylmethyl)propan-2-amine

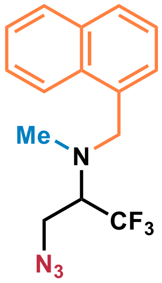

Compound **82** was prepared according to ***general procedure A*** from **1** (140 mg, 0.3 mmol), *N*-methyl-1-(naphthalen-1-yl)methanamine (51.4 mg, 0.3 mmol, 1 equiv) and sodium azide (48.8 mg, 0.75 mmol, 2.5 equiv) to afford a white crystalline solid (73.7 mg, 0.239 mmol, 80% yield). **R$_f$**= 0.65 (hexanes:ethyl acetate= 10:1). **M.p.=** 79-80 °C. **LRMS** (EI, 70 eV): m/z (%): 308(1), 280(1), 253(3), 252(17), 169(2), 168(4), 142(13), 141(100), 140(2), 139(7), 116(2), 115(17). **^{1}H NMR** (250 MHz, Chloroform-*d*) δ 8.26 (d, J = 8.0 Hz, 1H), 7.91 (dd, J = 7.5, 1.9 Hz, 1H), 7.86 (dd, J = 7.7, 1.7 Hz, 1H), 7.67 – 7.41 (m, 4H), 4.43 (d, J = 13.2 Hz, 1H), 4.35 (d, J = 13.2 Hz, 1H), 3.61 (dd, J = 12.6, 9.6 Hz, 1H), 3.56 – 3.34 (m, 1H), 3.31 (dd, J = 12.6, 3.6 Hz, 1H), 2.55 (s, 3H). **^{19}F NMR** (235 MHz, Chloroform-*d*) δ -67.45. **^{13}C NMR** (63 MHz, Chloroform-*d*) δ 134.4, 133.6, 132.7, 129.0, 128.9, 128.2, 126.4, 126.3, 126.0 (q, J = 290.4 Hz), 125.6, 124.7, 62.8 (q, J = 25.1 Hz), 59.0, 47.4 (q, J = 2.1 Hz), 36.3. **IR** (ATR, cm^{-1}) 3061, 3037, 3000, 2975, 2942, 2917, 2877, 2856, 2806, 2169, 2106, 1597, 1509, 1479, 1455, 1394, 1377, 1320, 1290, 1248, 1198, 1177, 1157, 1112, 1071, 1051, 1031, 973, 951, 915, 845, 810, 793, 776, 722, 712, 649, 583, 542, 440, 421. **HRMS** m/z [M+H]$^+$ calculated for $C_{15}H_{16}N_4F_3^+$: 309.1327, found: 309.1326.

*Synthesis of compound **83***

4,4,4-trifluoro-3-(methyl(naphthalen-1-ylmethyl)amino)butanenitrile

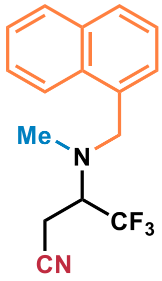

Compound **83** was prepared according to ***general procedure A*** from **1** (140 mg, 0.3 mmol), *N*-methyl-1-(naphthalen-1-yl)methanamine (51.4 mg, 0.3 mmol, 1 equiv) and potassium cyanide (48.8 mg, 0.75 mmol, 2.5 equiv) to afford an off white solid (71.9 mg, 0.248 mmol, 83% yield). **R$_f$**= 0.23 (hexanes:ethyl acetate= 10:1). **M.p.=** 50-54 °C. **LRMS** (EI, 70 eV): m/z (%): 293(5), 292(26), 291(2), 252(3), 223(1), 168(2), 142(21), 141(100), 139(8), 115(21), 54(3). **^{1}H NMR** (250 MHz, Chloroform-*d*) δ 8.3 – 8.1 (m, 1H), 8.0 – 7.8 (m, 2H), 7.7 – 7.4 (m, 4H), 4.4 (d, J = 2.9 Hz, 2H), 3.7 (dqd, J = 10.1, 7.7, 4.9 Hz, 1H), 2.8 (dd, J = 17.1, 10.1 Hz, 1H), 2.6 (dd, J = 17.1, 4.9 Hz, 1H), 2.5 (q, J = 1.8 Hz, 3H). **^{19}F NMR** (235 MHz, Chloroform-*d*) δ -69.2. **^{13}C NMR** (63 MHz, Chloroform-*d*) δ 134.0, 132.7, 132.2, 128.8, 128.6, 127.7, 126.3, 126.1, 125.9 (q, J = 290.9 Hz),

125.3, 124.4, 116.4, 59.6 (q, J = 27.3 Hz), 58.2 (d, J = 1.5 Hz), 36.0 (d, J = 1.4 Hz), 16.1 (q, J = 2.3 Hz). **IR** (ATR, cm^{-1}) 3088, 3054, 3002, 2968, 2941, 2915, 2893, 2851, 2810, 2254, 1598, 1510, 1465, 1438, 1419, 1390, 1368, 1356, 1303, 1285, 1259, 1241, 1191, 1171, 1150, 1110, 1065, 1051, 1012, 968, 946, 844, 804, 783, 774, 737, 720, 703, 608, 546, 421. **HRMS** m/z $[M+H]^+$ calculated for $C_{16}H_{14}N_2F_3^+$: 291.1109, found: 291.1110.

*Synthesis of compound **84***

N-(4-methoxybenzyl)-*N*-(1,1,1,3-tetrafluoropropan-2-yl)prop-2-en-1-amine

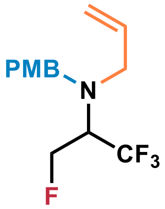

Compound **84** was prepared according to ***general procedure A*** from **1** (140 mg, 0.3 mmol), *N*-(4-methoxybenzyl)prop-2-en-1-amine (53.2 mg, 0.3 mmol, 1 equiv) and cesium fluoride (114 g, 0.75 mmol, 2.5 equiv) to afford a colorless oil (64.1 mg, 0.220 mmol, 73% yield). **R_f**= 0.60 (hexanes:ethyl acetate= 10:1). **LRMS** (EI, 70 eV): m/z (%): 291(2), 222(1), 183(1), 170(1), 148(7), 122(17), 121(100), 121(68), 110(4), 91(8), 90(3), 89(5), 78(12), 77(12), 65(3), 52(2), 51(3). **^{1}H NMR** (250 MHz, Chloroform-*d*) 7.17 (d, J = 8.5 Hz, 2H), 6.78 (d, J = 8.6 Hz, 2H), 5.70 (ddt, J = 16.5, 10.1, 6.3 Hz, 1H), 5.14 (d, J = 21.4 Hz, 1H), 5.08 (d, J = 15.4 Hz, 1H), 4.67 (dt, J = 6.5, 3.8 Hz, 1H), 4.56 – 4.42 (m, 1H), 3.74 (s, 2H), 3.71 (s, 3H), 3.68 – 3.38 (m, 1H), 3.25 (d, J = 6.3 Hz, 2H). **^{19}F NMR** (235 MHz, Chloroform-*d*) δ -68.3 (d, J = 6.6 Hz), -229.1 (q, J = 6.6 Hz). **^{13}C NMR** (63 MHz, Chloroform-*d*) 159.1, 136.0, 130.8, 129.8, 126.1 (qd, J = 288.0, 8.9 Hz), 118.2, 114.0, 80.6 (qd, J = 174.9, 1.8 Hz), 59.5 (qd, J = 26.4, 20.8 Hz), 55.4, 54.2, 54.0. **IR** (ATR, cm^{-1}) 3075, 2958, 2938, 2911, 2839, 1612, 1587, 1511, 1465, 1371, 1302, 1244, 1153, 1105, 1071, 1028, 997, 924, 831, 807, 760, 706, 635, 613, 570, 515, 485, 434. **HRMS** m/z $[M+H]^+$ calculated for $C_{14}H_{18}NOF_4^+$: 292.1325, found: 292.1319.

*Synthesis of compound **85***

1-(pyridin-2-yl)-4-(1,1,1,3-tetrafluoropropan-2-yl)piperazine

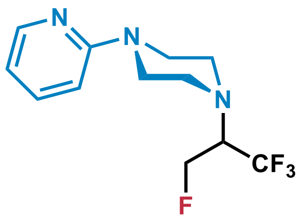

Compound **85** was prepared according to ***general procedure A*** from with **1** (140 mg, 0.3 mmol), *N*-benzylpiperazine (49.0 mg, 0.3 mmol, 1 equiv) and cesium fluoride (114 mg, 0.5 mmol, 2.5 equiv) to afford a white solid (53.2 mg, 0.192 mmol, 64% yield). **R_f**= 0.60 (hexanes:ethyl acetate= 7:3). **M.p.=** 44-47 °C. **LRMS** (EI, 70 eV): m/z (%): 277(10), 208(1), 133(13), 121(6), 120(7), 119(13), 110(6), 108(7), 107(100), 79(22), 78(17), 74(5), 56(6), 52(4), 51(6). **^{1}H NMR** (250 MHz, Chloroform-*d*) δ 8.3 – 8.0 (m, 1H), 7.5 (td, J = 7.9, 7.1, 2.0 Hz, 1H), 6.6 (dd, J = 7.7, 4.7 Hz, 2H), 4.8 (q, J = 5.6 Hz, 1H), 4.7 – 4.4 (m, 1H), 3.5 (t, J = 5.0 Hz, 4H), 3.5 – 3.3 (m,

1H), 2.9 (p, J = 6.3, 5.3 Hz, 4H). **^{19}F NMR** (235 MHz, Chloroform-*d*) δ -68.3 (d, J = 6.8 Hz, 3F), -228.8 (q, J = 6.8 Hz, 1F). **^{13}C NMR** (63 MHz, Chloroform-*d*) δ 159.43, 147.97, 137.66, 125.51 (qd, J = 288.1, 9.7 Hz), 113.54, 107.23, 80.53 (qd, J = 175.1, 2.3 Hz), 65.40 (qd, J = 26.2, 20.6 Hz), 50.08, 46.07. **IR** (ATR, cm^{-1}) 3097, 3060, 2999, 2961, 2917, 2895, 2843, 2768, 2710, 2649, 1598, 1560, 1483, 1439, 1385, 1364, 1340, 1316, 1302, 1282, 1245, 1231, 1205, 1154, 1112, 1097, 1015, 997, 980, 963, 940, 924, 851, 771, 737, 717, 702, 622, 605, 569, 520, 495, 458. **HRMS** m/z $[M+H]^+$ calculated for $C_{12}H_{16}N_3F_4^+$: 278.1280, found: 278.1281.

*Synthesis of compound **86***

3-(benzyloxy)-1,1,1-trifluoro-*N*-methyl-*N*-(naphthalen-1-ylmethyl)propan-2-amine

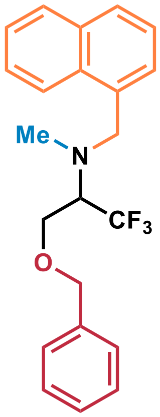

Compound **86** was prepared according to ***general procedure C*** from **1** (93.2 mg, 0.2 mmol), *N*-methyl-1-(naphthalen-1-yl)methanamine (34.2 mg, 0.2 mmol, 1 equiv), benzyl alcohol (52 µL, 0.5 mmol, 2.5 equiv) and sodium hydride (20 mg, 60 w% in oil, 0.5 mmol, 2.5 equiv) to afford a colorless oil (37.2 mg, 0.0996 mmol, 50% yield). **R_f**= 0.50 (hexanes:ethyl acetate= 10:1). **LRMS** (EI, 70 eV): m/z (%): 267(4), 252(2), 232(1), 171(7), 170(3), 156(3), 142(15), 141(100), 139(3), 126(5), 115(11), 91(13), 65(2). **^{1}H NMR** (250 MHz, Chloroform-*d*) 8.28 (dd, J = 6.4, 2.7 Hz, 1H), 7.97 – 7.75 (m, 2H), 7.62 – 7.28 (m, 9H), 4.55 (s, 2H), 4.37 (s, 2H), 3.97 – 3.84 (m, 1H), 3.79 (dd, J = 10.3, 4.3 Hz, 1H), 3.64 (ddq, J = 12.6, 8.5, 4.2 Hz, 1H), 2.51 (s, 3H). **^{19}F NMR** (235 MHz, Chloroform-*d*) δ -68.13. **^{13}C NMR** (63 MHz, Chloroform-*d*) δ 137.9, 134.3, 134.1, 132.5, 128.6, 128.3, 127.9, 127.7, 127.6, 126.7 (q, J = 289.1, 288.7 Hz), 126.0, 125.8, 125.3, 124.7, 73.5, 66.1 (q, J = 1.8 Hz), 63.2 (q, J = 25.4 Hz), 58.9, 37.3. **IR** (ATR, cm^{-1}) 3064, 3036, 2942, 2863, 2809, 1714, 1598, 1510, 1455, 1367, 1313, 1249, 1209, 1160, 1105, 1072, 1029, 907, 849, 791, 774, 730, 696, 649, 625, 587, 544, 461, 417. **HRMS** m/z $[M+H]^+$ calculated for $C_{22}H_{23}NOF_3^+$: 374.1732, found: 374.1732.

*Synthesis of compound **87***

3-(4-bromophenoxy)-1,1,1-trifluoro-*N*-methyl-*N*-(naphthalen-1-ylmethyl)propan-2-amine

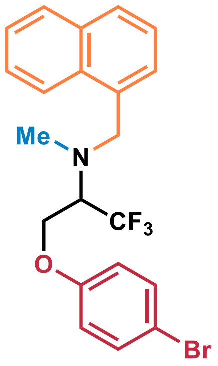

Compound **87** was prepared according to ***general procedure C*** from **1** (140 mg, 0.3 mmol), *N*-methyl-1-(naphthalen-1-yl)methanamine (51.4 mg, 0.3 mmol, 1 equiv), 4-bromo-phenol (130 mg, 0.75 mmol, 2.5 equiv) and sodium hydride (30 mg, 60 w% in oil, 0.75 mmol, 2.5 equiv) to afford a colorless oil (114 mg, 0.261 mmol, 87% yield). **R_f**= 0.68 (hexanes:ethyl acetate= 10:1). **LRMS** (EI, 70 eV): m/z (%): 440(1), 439(3), 437(2), 253(3), 252(20), 207(1), 185(1), 168(2), 155(3), 142(13), 142(6), 141(100), 141(48), 139(5), 115(10), 115(5), 63(1). **^{1}H NMR** (250 MHz, Chloroform-*d*) 8.31 (dd, *J* = 6.0, 3.4 Hz, 1H), 8.00 – 7.81 (m, 2H), 7.67 – 7.43 (m, 4H), 7.40 (d, *J* = 8.7 Hz, 2H), 6.73 (d, *J* = 8.7 Hz, 2H), 4.42 (d, *J* = 3.0 Hz, 2H), 4.54 – 4.08 (m, 2H), 3.92 – 3.66 (m, 1H), 2.61 (s, 3H). **^{19}F NMR** (235 MHz, Chloroform-*d*) δ -67.62. **^{13}C NMR** (63 MHz, Chloroform-*d*) δ 157.2, 134.1, 133.8, 132.4, 128.6, 127.8, 126.5 (q, *J* = 289.7, 289.6, 288.5 Hz), 126.0, 125.9, 125.3, 124.7, 116.4, 113.7, 64.1 (q, *J* = 1.6 Hz), 61.9 (q, *J* = 25.7 Hz), 59.0, 37.1. **IR** (ATR, cm^{-1}) 3061, 3048, 3007, 2948, 2917, 2891, 2867, 2813, 2793, 1592, 1578, 1489, 1469, 1446, 1395, 1382, 1374, 1272, 1235, 1215, 1166, 1126, 1100, 1071, 1054, 1036, 1024, 1001, 892, 821, 803, 790, 776, 739, 710, 654, 641, 625, 607, 518, 503, 476, 442, 428. **HRMS** m/z $[M+H]^+$ calculated for $C_{21}H_{20}NOBrF_3^+$: 438.0680, found: 438.0680.

*Synthesis of compound **88***

3,3,3-trifluoro-2-(methyl(naphthalen-1-ylmethyl)amino)propyl benzoate

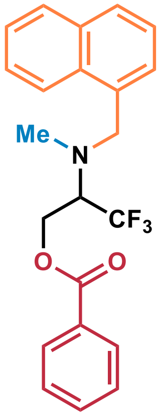

Compound **88** was prepared according to ***general procedure A*** from **1** (140 mg, 0.3 mmol), *N*-methyl-1-(naphthalen-1-yl)methanamine (51.4 mg, 0.3 mmol, 1 equiv) and sodium benzoate (108 mg, 0.75 mmol, 2.5 equiv) to afford a white solid (91.7 mg, 0.237 mmol, 79% yield). **R_f**= 0.49 (hexanes:ethyl acetate= 10:1). **M.p.=** 94-96 °C. **LRMS** (EI, 70 eV): m/z (%): 440(1), 439(3), 437(2), 253(3), 252(20), 207(1), 185(1), 168(2), 155(3), 142(13), 142(6), 141(100), 141(48), 139(5), 115(10), 115(5), 63(1). **^{1}H NMR** (250 MHz, Chloroform-*d*) 8.16 (d, *J* = 8.4 Hz, 1H), 8.00 – 7.89 (m, 2H), 7.84 (t, *J* = 8.3 Hz, 2H), 7.61 (t, *J* = 7.3 Hz, 1H), 7.54 – 7.37 (m, 5H), 7.31 (t, *J* = 7.6 Hz, 1H), 4.74 (dd, *J* = 11.7, 8.6 Hz, 1H), 4.53 (dd, *J* = 11.8, 4.5 Hz, 1H), 4.38 (s, 2H), 3.80 (ddq, *J* = 13.1, 8.5, 4.8, 4.2 Hz, 1H), 2.62 (s, 3H). **^{19}F NMR** (235 MHz, Chloroform-*d*) δ -67.16. **^{13}C NMR** (63 MHz, Chloroform-*d*) δ 166.1, 134.0, 133.6, 133.2, 132.4, 129.8, 129.7, 128.5, 128.5, 127.7, 126.1, 125.8, 125.2, 124.3, 124.3 (q, *J* = 291.0, 290.5 Hz), 61.7 (q, *J* = 25.5 Hz), 59.8 (d, *J*

= 2.1 Hz), 58.7, 36.7. **IR** (ATR, cm^{-1}) 3064, 3036, 2969, 2861, 2812, 1721, 1601, 1510, 1451, 1374, 1314, 1270, 1245, 1217, 1160, 1106, 1069, 1054, 1027, 790, 773, 706, 686, 584, 518, 503, 415. **HRMS** m/z $[M+H]^+$ calculated for $C_{22}H_{21}NO_2F_3^+$: 388.1524, found: 388.1527.

*Synthesis of compound **89***

3-(cyclohexylthio)-1,1,1-trifluoro-*N*-methyl-*N*-(naphthalen-1-ylmethyl)propan-2-amine

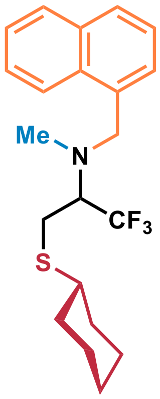

Compound **89** was prepared according to ***general procedure A*** from **1** (140 mg, 0.3 mmol), *N*-methyl-1-(naphthalen-1-yl)methanamine (51.4 mg, 0.3 mmol, 1 equiv), cyclohexanethiol (92 µL, 0.75 mmol, 2.5 equiv) to afford a colorless oil (89.3 mg, 0.234 mmol, 78% yield). **R$_f$**= 0.63 (hexanes:ethyl acetate= 10:1). **LRMS** (EI, 70 eV): m/z (%): 381(1), 267(2), 253(2), 252(11), 171(3), 170(16), 168(2), 142(16), 141(100), 139(3), 115(11), 83(2), 55(4), 55(2). **^{1}H NMR** (250 MHz, Chloroform-*d*) 8.31 (d, *J* = 7.9 Hz, 1H), 7.95 – 7.77 (m, 2H), 7.65 – 7.40 (m, 4H), 4.43 (d, *J* = 13.8 Hz, 1H), 4.36 (d, *J* = 13.7 Hz, 1H), 3.50 (ddh, *J* = 16.3, 8.2, 4.2 Hz, 1H), 2.98 (dd, *J* = 13.5, 10.3 Hz, 1H), 2.82 (dd, *J* = 13.5, 4.0 Hz, 1H), 2.50 (s, 4H), 2.00 – 1.53 (m, 5H), 1.43 – 1.12 (m, 5H). **^{19}F NMR** (235 MHz, Chloroform-*d*) δ -68.07. **^{13}C NMR** (63 MHz, Chloroform-*d*) δ 134.1, 134.0, 132.5, 128.5, 128.3, 127.6, 125.9, 125.8, 125.3, 124.9, 124.9 (q, *J* = 292.2 Hz), 63.9 (q, *J* = 24.8 Hz), 58.4, 43.9, 36.0, 33.4, 33.4, 27.0, 26.1, 26.0, 25.9. **IR** (ATR, cm^{-1}) 3047, 2928, 2853, 2832, 2810, 1598, 1510, 1448, 1373, 1327, 1252, 1163, 1102, 1080, 1001, 968, 907, 886, 856, 791, 773, 732, 689, 649, 574, 540, 519, 415. **HRMS** m/z $[M+H]^+$ calculated for $C_{21}H_{27}NF_3S^+$: 382.1816, found: 382.1810.

*Synthesis of compound **90***

3-((4-chlorophenyl)thio)-1,1,1-trifluoro-*N*-methyl-*N*-(naphthalen-1-ylmethyl)propan-2-amine

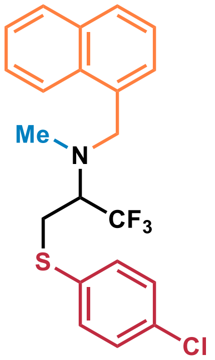

Compound **90** was prepared according to ***general procedure A*** from **1** (140 mg, 0.3 mmol), *N*-methyl-1-(naphthalen-1-yl)methanamine (51.4 mg, 0.3 mmol, 1 equiv), 4-chlorotiophenol (108 mg, 0.75 mmol, 2.5 equiv) to afford a white solid (83.0 mg, 0.203 mmol, 68% yield). **R$_f$**= 0.68 (hexanes:ethyl acetate= 10:1). **M.p.= 71**-73 °C. **LRMS** (EI, 70 eV): m/z (%): 141(100), 252(26), 142(15), 115(14), 253(4), 139(4), 143(3), 409(2), 168(2), 108(2), 170(2), 157(2). **^{1}H NMR** (250 MHz, Chloroform-*d*) δ 8.28 (d, *J* = 7.5 Hz, 1H), 8.00 – 7.80 (m, 2H), 7.68 – 7.40 (m, 5H), 7.20 (d, *J* = 8.5 Hz, 2H), 7.10 (d, *J* = 8.6 Hz, 2H), 4.39 (td, *J* = 13.5, 6.5 Hz, 2H), 3.52 (tt, *J* = 12.9, 6.4 Hz, 1H), 3.27 (dd, *J* = 13.6, 9.6 Hz, 1H), 3.17 (dd, *J* = 13.5, 7.3 Hz, 1H), 2.56 (s, 3H). **^{19}F NMR** (235 MHz, Chloroform-*d*) δ -67.35. **^{13}C NMR** (63 MHz,

Chloroform-*d*) δ 134.2, 134.0, 133.5, 132.5, 132.4, 130.8, 129.2, 128.6, 128.4, 127.7, 126.0, 125.8, 125.3, 124.8, 124.7 (q, *J* = 292.8, 292.3 Hz), 62.0 (q, *J* = 25.1 Hz), 58.2, 36.1, 31.8 (q, *J* = 1.0 Hz). **IR** (ATR, cm^{-1}) 3068, 3047, 3010, 3000, 2959, 2935, 2919, 2870, 2856, 2805, 1509, 1473, 1387, 1364, 1327, 1296, 1255, 1226, 1171, 1154, 1123, 1102, 1090, 1073, 1012, 966, 864, 852, 827, 817, 804, 787, 771, 742, 719, 716, 691, 587, 540, 496, 420. **HRMS** m/z $[M+H]^+$ calculated for $C_{21}H_{20}NF_3SCl^+$: 410.0957, found: 410.0964.

Synthesis of compound ***91***

3-(benzo[d]thiazol-2-ylthio)-1,1,1-trifluoro-*N*-methyl-*N*-(naphthalen-1-ylmethyl)propan-2-amine

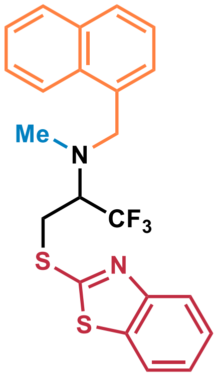

Compound **91** was prepared according to general ***general procedure A*** from **1** (140 mg, 0.3 mmol), *N*-methyl-1-(naphthalen-1-yl)methanamine (51.4 mg, 0.3 mmol, 1 equiv) and 2-mercaptobenzthiazole (125 mg, 0.75 mmol, 2.5 equiv) to afford a pale yellow solid (106 mg, 0.245 mmol, 82% yield). $\mathbf{R_f}$= 0.55 (hexanes:ethyl acetate= 10:1). **M.p.=** 82-85 °C. **LRMS** (EI, 70 eV): m/z (%): 432(2), 291(4), 284(4), 266(3), 265(17), 264(8), 196(8), 170(3), 169(9), 168(33), 167(7), 154(2), 149(4), 142(12), 141(100), 139(4), 136(4), 116(2), 115(15), 108(2). ^{1}**H NMR** (250 MHz, Chloroform-*d*) 8.27 – 8.16 (m, 1H), 7.92 – 7.68 (m, 4H), 7.63 – 7.38 (m, 4H), 7.44 – 7.22 (m, 2H), 4.42 (d, *J* = 13.2 Hz, 1H), 4.36 (d, *J* = 13.6 Hz, 1H), 4.03 – 3.80 (m, 2H), 3.64 (dd, *J* = 13.8, 11.1 Hz, 1H), 2.57 (t, *J* = 2.0 Hz, 3H). ^{19}**F NMR** (235 MHz, Chloroform-*d*) δ -67.14. ^{13}**C NMR** (63 MHz, Chloroform-*d*) δ 166.0, 153.1 (d, *J* = 292.9 Hz), 135.3, 133.9, 133.4, 132.3, 128.5, 128.4, 127.6, 127.0 (q, *J* = 292.9 Hz), 126.0, 125.9, 125.8, 125.1, 124.7, 124.3, 121.6, 121.0, 61.7 (q, *J* = 25.5 Hz), 58.2, 35.9, 30.6 (q, *J* = 1.8 Hz). **IR** (ATR, cm^{-1}) 3091, 3051, 3040, 2997, 2968, 2932, 2912, 2857, 2810, 1509, 1458, 1428, 1388, 1363, 1351, 1330, 1309, 1302, 1251, 1164, 1122, 1103, 1069, 1049, 1018, 995, 907, 864, 851, 804, 784, 773, 757, 729, 703, 691, 672, 649, 603, 586, 540, 428, 420. **HRMS** m/z $[M+H]^+$ calculated for $C_{22}H_{20}N_2F_3S_2^+$: 433.1020, found: 433.1016.

Synthesis of compound ***92***

1,1,1-trifluoro-*N*-methyl-*N*-(naphthalen-1-ylmethyl)-3-(phenylsulfonyl)propan-2-amine

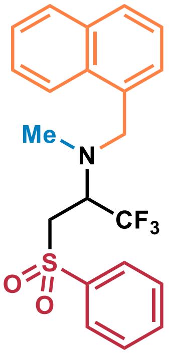

Compound **92** was prepared according to ***general procedure A*** from **1** (140 mg, 0.3 mmol), *N*-methyl-1-(naphthalen-1-yl)methanamine (51.4 mg, 0.3 mmol, 1 equiv) and sodium benzenesulfinate (126 mg, 0.75 mmol, 2.5 equiv) to afford a colorless oil (70.7 mg, 0.174 mmol, 58% yield). **R**$_f$= 0.65 (ethyl acetate). ^{1}**H NMR** (250 MHz, Chloroform-*d*) *δ* 8.28 – 8.10 (m, 1H), 7.94 – 7.77 (m, 2H), 7.73 – 7.36 (m, 9H), 4.42 – 4.18 (m, 3H), 3.90 – 3.74 (m, 1H), 3.73 – 3.42 (m, 1H), 2.56 – 2.29 (m, 3H). ^{19}**F NMR** (235 MHz, Chloroform-*d*) *δ* -67.74, -67.79. ^{13}**C NMR** (63 MHz, Chloroform-*d*) *δ* 144.1, 143.9, 134.0, 133.7, 133.6, 132.5, 132.4, 132.3, 129.2, 129.2, 128.6, 128.5, 127.6, 127.6, 126.0, 126.0, 125.9, 125.8, 125.4, 125.3, 124.6, 124.6, 123.7 (q, *J* = 290.0, 289.5 Hz), 63.3 (q, *J* = 26.5, 26.0 Hz), 62.2 (q, *J* = 26.3, 26.2, 25.8 Hz), 58.7, 36.8, 36.4. **IR** (ATR, cm^{-1}) 3058, 2952, 2888, 2863, 2815, 1714, 1690, 1598, 1510, 1477, 1462, 1445, 1371, 1312, 1297, 1249, 1157, 1109, 1052, 1034, 968, 855, 791, 776, 753, 732, 689, 611, 587, 559, 440. **HRMS** m/z [M+H]$^{+}$ calculated for $C_{21}H_{21}NO_2F_3S^{+}$: 408.1245, found: 408.1241.

Synthesis of compound ***93***

3-(diphenylphosphanyl)-1,1,1-trifluoro-*N*-methyl-*N*-(naphthalen-1-ylmethyl)propan-2-amine

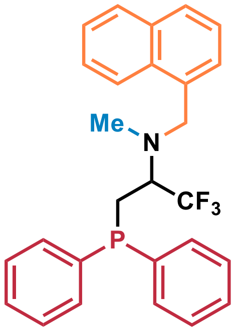

Compound **93** was prepared according to ***general procedure A*** from **1** (140 mg, 0.3 mmol), *N*-methyl-1-(naphthalen-1-yl)methanamine (51.4 mg, 0.3 mmol, 1 equiv) and diphenylphosphane (53.3 uL, 0.75 mmol, 2.5 equiv), under argon atmosphere to afford a colorless oil (77.7 mg, 0.172 mmol, 57% yield). **R**$_f$= 0.53 (hexanes:ethyl acetate= 10:1). **LRMS** (EI, 70 eV): m/z (%): 452(1), 451(5), 408(1), 382(1), 312(12), 310(6), 281(3), 264(1), 236(3), 199(9), 187(9), 186(100), 185(6), 183(21), 171(8), 170(53), 168(8), 142(7), 141(37), 141(36), 121(24), 115(21), 109(6), 108(43), 91(5). ^{1}**H NMR** (250 MHz, Chloroform-*d*) *δ* 8.22 (d, *J* = 7.9 Hz, 1H), 7.86 (dd, *J* = 23.7, 7.9 Hz, 2H), 7.65 – 7.48 (m, 3H), 7.39 (dt, *J* = 22.4, 7.9 Hz, 11H), 4.37 (q, *J* = 13.8 Hz, 2H), 3.42 (td, *J* = 14.3, 12.7, 5.3 Hz, 1H), 2.73 – 2.53 (m, 2H), 2.50 (s, 3H). ^{19}**F NMR** (235 MHz, Chloroform-*d*) *δ* -67.93 (d, *J* = 5.8 Hz). ^{31}**P NMR** (101 MHz, Chloroform-*d*) *δ* -22.55 (q, *J* = 5.8 Hz). ^{13}**C NMR** (63 MHz, Chloroform-*d*) *δ* 138.9 (d, *J* = 13.8 Hz), 137.0 (d, *J* = 15.1 Hz), 134.0, 133.4, 133.4, 133.0, 132.6, 132.3, 132.3, 129.9 (qd, *J* = 293.8, 9.5 Hz), 129.2, 128.8, 128.7, 128.6, 128.6, 128.5, 128.2, 127.5 (d, *J* = 1.6 Hz), 126.1, 125.7, 125.3, 124.5 (d, *J* = 2.3 Hz), 61.3 (qd, *J* = 25.6, 14.9 Hz), 57.8, 36.2, 26.6 (d, *J* = 14.4 Hz). **IR** (ATR, cm^{-}

[1]) 3068, 3053, 3014, 3000, 2953, 2924, 2854, 2807, 1952, 1884, 1812, 1738, 1726, 1597, 1587, 1510, 1480, 1434, 1374, 1256, 1226, 1157, 1100, 1069, 1025, 998, 907, 889, 845, 793, 780, 734, 693, 580, 540, 503, 476, 415. **HRMS** m/z $[M+H]^+$ calculated for $C_{27}H_{26}NF_3P^+$: 452.1755, found: 452.1757.

Synthesis of compound ***94***

Di(adamantan-1-yl)(3,3,3-trifluoro-2-(methyl(naphthalen-1-ylmethyl)amino)propyl)phosphonium trifluoromethanesulfonate

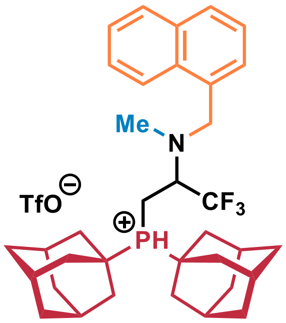

Compound **94** was prepared according to ***general procedure A*** from **1** (140 mg, 0.3 mmol), *N*-methyl-1-(naphthalen-1-yl)methanamine (51.4 mg, 0.3 mmol, 1 equiv) and di(adamantan-1-yl)phosphane (90.7 mg, 0.75 mmol, 2.5 equiv), under argon atmosphere to afford a white solid (140 mg, 0.195 mmol, 65% yield). $\mathbf{R_f}$= 0.73 (hexanes:diisopropyl ether= 10:1). **M.p.=** 210-214 °C. ^{1}**H NMR** (250 MHz, Methylene Chloride-d_2) δ 8.30 – 7.84 (m, 3H), 7.85 – 7.24 (m, 4H), 4.75 (bs, 1H), 4.64 – 4.32 (m, 1H), 4.32 – 3.89 (m, 1H), 3.37 (d, J = 26.2 Hz, 2H), 2.88 (s, 1H), 2.76 (s, 3H), 2.41 – 1.33 (m, 24H), 1.09 (s, 6H). ^{19}**F NMR** (235 MHz, Methylene Chloride-d_2) δ -65.25 (s, 3F), -76.93 (s, 3F). ^{31}**P NMR** (101 MHz, Methylene Chloride-d_2) δ 30.67. ^{13}**C NMR** (63 MHz, Methylene Chloride-d_2) δ 134.8, 128.6 (qd, J = 292.4, 14.7 Hz), 118.8 (q, J = 321.2 Hz), 65.9, 57.7, 56.2 (qd, J = 26.9, 5.2 Hz), 37.8 (d, J = 40.9 Hz), 37.4 (h, J = 17.0, 17.0, 15.9, 15.7, 14.9 Hz), 35.3 (d, J = 19.6 Hz), 27.7 (d, J = 18.8 Hz), 27.5 (d, J = 18.4 Hz), 22.9, 15.4, 11.0, 10.2. **IR** (ATR, cm^{-1}) 3067, 3050, 3040, 3007, 2912, 2856, 2817, 2413, 1745, 1716, 1452, 1374, 1347, 1304, 1269, 1249, 1221, 1166, 1140, 1107, 1046, 1027, 1002, 966, 943, 878, 844, 811, 803, 793, 781, 751, 634, 571, 516, 482, 448, 418. **HRMS** m/z $[M+H]^+$ calculated for $C_{35}H_{46}NOF_3P^+$: 568.3320, found: 568.3309.

Synthesis of compound ***95***

Diethyl 2-(3,3,3-trifluoro-2-(methyl(naphthalen-1-ylmethyl)amino)propyl)malonate

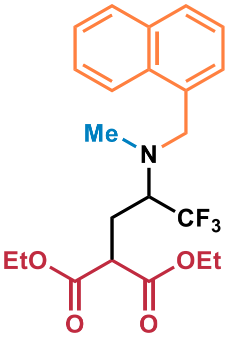

Compound **95** was prepared according to ***general procedure A*** with **1** (140 mg, 0.3 mmol), *N*-methyl-1-(naphthalen-1-yl)methanamine (51.4 mg, 0.3 mmol, 1 equiv), diethyl malonate (114 uL, 0.75 mmol, 2.5 equiv) and sodium hydride (30 mg, 60 w% in oil, 0.75 mmol, 2.5 equiv) to afford a colorless oil (82.1 mg, 0.193 mmol, 64% yield). $\mathbf{R_f}$= 0.33 (hexanes:ethyl acetate= 10:1). **LRMS** (EI, 70 eV): m/z (%): 425(1), 380(1), 357(3), 356(12), 284(2), 192(3), 173(2), 171(5), 170(34), 168(2), 142(14), 141(100), 139(4), 115(11). ^{1}**H NMR** (250

MHz, Chloroform-*d*) *δ* 8.08 (d, *J* = 8.0 Hz, 1H), 7.93 – 7.71 (m, 2H), 7.63 – 7.37 (m, 4H), 4.36 (d, *J* = 13.3 Hz, 1H), 4.24 (d, *J* = 13.5 Hz, 1H), 4.15 (q, *J* = 7.1 Hz, 2H), 4.01 (dq, *J* = 10.8, 7.1 Hz, 1H), 3.83 (dq, *J* = 10.8, 7.1 Hz, 1H), 3.52 (dd, *J* = 9.1, 5.1 Hz, 1H), 3.50 – 3.29 (m, 1H), 2.53 – 2.43 (m, 3H), 2.20 (dt, *J* = 9.9, 4.5 Hz, 2H), 1.23 (t, *J* = 7.1 Hz, 3H), 1.14 (t, *J* = 7.1 Hz, 3H). **^{19}F NMR** (235 MHz, Chloroform-*d*) *δ* -67.43. **^{13}C NMR** (63 MHz, Chloroform-*d*) *δ* 169.5, 168.6, 134.1, 133.6, 132.3, 128.7, 128.4 (q, *J* = 293.1 Hz), 128.4, 127.9, 126.2, 125.8, 125.3, 124.2, 61.6, 61.3, 59.9 (q, *J* = 25.1 Hz), 57.9, 47.8, 35.9, 25.6 (d, *J* = 1.9 Hz), 14.1, 13.9. **IR** (ATR, cm^{-1}) 3050, 2983, 2941, 2905, 2871, 2810, 1730, 1510, 1466, 1448, 1371, 1339, 1242, 1207, 1161, 1141, 1110, 1068, 1029, 849, 791, 776, 734, 705, 581, 518, 417. **HRMS** m/z $[M+H]^+$ calculated for $C_{22}H_{27}NO_4F_3^+$: 426.1892, found: 426.1895.

*Synthesis of compound **96***

1,1,1-trifluoro-4-(isoquinolin-1-yl)-*N*-methyl-*N*-(naphthalen-1-ylmethyl)butan-2-amine

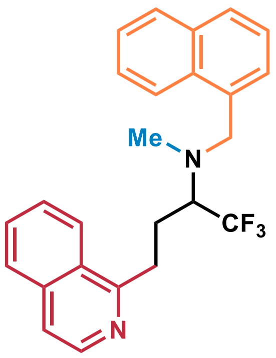

Compound **96** was prepared according to ***general procedure D*** from **1** (140 mg, 0.3 mmol), *N*-methyl-1-(naphthalen-1-yl)methanamine (51.4 mg, 0.3 mmol, 1 equiv), 1-methylisoquinoline (44 µL, 0.33 mmol, 1.1 equiv) and ^{n}BuLi solution (216 µL, 1.6 M in hexanes, 0.345 mmol, 1.15 equiv) to afford a white solid (66.3 mg, 0.162 mmol, 54% yield). **R_f**= 0.50 (hexanes:ethyl acetate= 5:1). **M.p.=** 133-135 °C. **LRMS** (EI, 70 eV): m/z (%): 355(1), 281(2), 207(2), 185(3), 185(3), 184(28), 184(12), 142(12), 141(100), 140(2), 139(3), 115(14), 73(2). **^{1}H NMR** (300 MHz, Chloroform-*d*) *δ* 8.35 (d, *J* = 8.6 Hz, 1H), 7.84 (d, *J* = 8.5 Hz, 1H), 7.80 (d, *J* = 8.5 Hz, 1H), 7.51 – 7.34 (m, 3H), 7.24 – 7.10 (m, 4H), 7.04 – 6.95 (m, 1H), 4.12 (d, *J* = 12.8 Hz, 1H), 4.11 (d, *J* = 14.5 Hz, 1H), 3.97 (d, *J* = 14.8 Hz, 1H), 3.96 (d, *J* = 12.8 Hz, 1H), 3.64 – 3.47 (m, 1H), 3.20 – 2.93 (m, 3H), 2.92 – 2.81 (m, 2H), 2.75 (dd, *J* = 13.5, 3.6 Hz, 1H), 2.34 (s, 3H). **^{19}F NMR** (282 MHz, Chloroform-*d*) *δ* -67.33 (d, *J* = 8.2 Hz). **^{13}C NMR** (75 MHz, Chloroform-*d*) *δ* 160.5, 141.5, 136.0, 134.3, 134.1, 132.5, 129.9, 128.7, 128.4, 127.9, 127.4, 127.1, 126.9, 126.2 (q, *J* = 293.4 Hz), 125.9, 125.7, 125.3, 125.0, 124.7, 119.4, 61.4 (q, *J* = 24.5 Hz), 58.3, 36.1, 30.9, 24.4. **IR** (ATR, cm^{-1}) 3050, 3006, 2986, 2959, 2931, 2854, 2805, 1624, 1588, 1565, 1506, 1392, 1373, 1304, 1252, 1245, 1156, 1124, 1105, 1063, 844, 821, 803, 783, 777, 730, 698, 571, 536, 505, 461. **HRMS** m/z $[M+H]^+$ calculated for $C_{25}H_{24}N_2F_3^+$: 409.1886, found: 409.1889.

*Synthesis of compound **97***

1,1,1-trifluoro-*N*-methyl-*N*-(naphthalen-1-ylmethyl)-3-(5,6,7,8-tetrahydroquinolin-8-yl)propan-2-amine

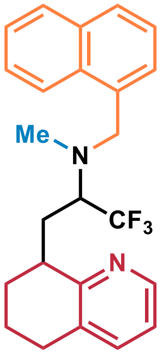

Compound **97** was prepared according to ***general procedure D*** from **1** (140 mg, 0.3 mmol), *N*-methyl-1-(naphthalen-1-yl)methanamine (51.4 mg, 0.3 mmol, 1 equiv), 5,6,7,8-tetrahydroquinoline (43 μL, 0.33 mmol, 1.1 equiv) and ^{n}BuLi solution (216 μL, 1.6 M in hexanes, 0.345 mmol, 1.15 equiv) to afford a white solid (29.2 mg, 0.0733 mmol, 24% yield). **R$_f$**= 0.23 (hexanes:ethyl acetate= 10:1). **M.p.=** 102-105 °C. **LRMS** (EI, 70 eV): m/z (%): 398(1), 378(2), 335(1), 257(17), 237(23), 228(4), 171(4), 170(28), 146(38), 146(19), 142(9), 141(61), 139(7), 134(10), 133(100), 132(59), 130(13), 118(9), 117(13), 115(26). ^{1}**H NMR** (300 MHz, Chloroform-*d*) *δ* 8.42 (d, *J* = 4.7 Hz, 1H), 8.26 – 8.15 (m, 1H), 7.93 – 7.78 (m, 2H), 7.64 – 7.38 (m, 4H), 7.36 – 7.25 (m, 1H), 7.03 (dd, *J* = 7.6, 4.8 Hz, 1H), 4.48 (d, *J* = 13.3 Hz, 1H), 4.32 (d, *J* = 13.2 Hz, 1H), 3.31 (ddt, *J* = 16.4, 11.5, 5.8 Hz, 1H), 2.92 (tt, *J* = 11.2, 4.6 Hz, 1H), 2.79 – 2.72 (m, 4H), 2.59 (q, *J* = 6.1 Hz, 2H), 1.53 – 1.33 (m, 2H), 1.25 – 1.05 (m, 1H), 1.02 – 0.76 (m, 2H). (major diastereomer). ^{19}**F NMR** (282 MHz, Chloroform-*d*) *δ* -66.30 (d, *J* = 8.3 Hz) minor diastereomer, -66.94 (d, *J* = 8.2 Hz), major diastereomer. ^{13}**C NMR** (75 MHz, Chloroform-*d*) *δ* 159.8, 146.5, 137.2, 134.4, 134.1, 132.7, 129.0, 128.6, 128.4, 128.3, 128.1, 126.6, 126.0, 125.8, 125.3, 124.9, 123.7, 121.0, 58.6, 58.2, 57.9 (d, *J* = 24.2 Hz), 36.2, 35.7, 31.1, 29.1, 26.5, 19.8, (major diastereomer). **IR** (ATR, cm^{-1}) 3066, 3044, 3007, 2972, 2941, 2912, 2897, 2857, 2803, 1571, 1509, 1442, 1425, 1388, 1371, 1258, 1239, 1150, 1129, 1110, 1086, 1065, 1017, 1005, 966, 862, 845, 807, 793, 776, 726, 695, 580, 544, 418. **HRMS** m/z [M+H]$^+$ calculated for $C_{24}H_{26}N_2F_3^+$: 399.2043, found: 399.2046.

*Synthesis of compound **98***

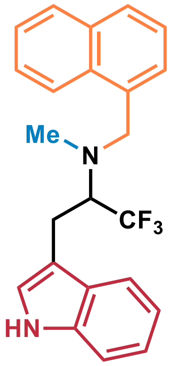

1,1,1-trifluoro-3-(1H-indol-3-yl)-N-methyl-N-(naphthalen-1-ylmethyl)propan-2-amine

Aziridinium intermediate solution was prepared according to ***general procedure A*** from **1** (140 mg, 0.3 mmol) and *N*-methyl-1-(naphthalen-1-yl)methanamine (51.4 mg, 0.3 mmol, 1 equiv) in acetonitrile. A separate, oven dried vial was charged with stirring bar, indole (42.6 mg, 0.36 mmol, 1.2 equiv) and sealed with Teflon septa and screw cap. Vial was evacuated and backfilled with argon (repeated three times), then toluene (2.5 mL) was added under argon atmosphere. To the stirred mixture, solution of Et_2Zn (1 M in toluene, 360 uL, 0.36 mmol, 1.2 equiv) was added dropwise under argon atmosphere at room temperature and stirring was continued for 1 hour. After

that, solution of aziridinium intermediate was added and the mixture was stirred at room temperature for 16 hours. Purification was carried out according to ***general procedure A*** to afford a colorless oil (79.9 mg, 0.209 mmol, 70% yield). $\mathbf{R_f}$= 0.43 (hexanes:ethyl acetate= 5:1). **M.p.=** 73-76 °C. **LRMS** (EI, 70 eV): m/z (%): 382(6), 252(11), 142(13), 141(100), 139(4), 131(5), 130(41), 115(13), 103(3), 77(4). ^{1}**H NMR** (250 MHz, Chloroform-*d*) δ 7.78 (d, J = 8.2 Hz, 1H), 7.70 (d, J = 8.0 Hz, 1H), 7.67 – 7.58 (m, 1H), 7.48 – 7.17 (m, 6H), 7.11 (d, J = 7.0 Hz, 1H), 7.05 (d, J = 7.4 Hz, 1H), 6.74 (d, J = 2.4 Hz, 1H), 4.24 (d, J = 13.6 Hz, 1H), 4.16 (d, J = 13.4 Hz, 1H), 3.80 – 3.52 (m, 1H), 3.15 (s, 1H), 3.12 (s, 1H), 2.57 – 2.43 (m, 3H). ^{19}**F NMR** (235 MHz, Chloroform-*d*) δ -67.64. ^{13}**C NMR** (75 MHz, Chloroform-*d*) δ 136.2, 134.1, 133.8, 132.2, 129.1 (d, J = 292.8 Hz), 128.2, 128.1, 127.5, 127.3, 125.4, 125.4, 125.0, 124.5, 123.1, 122.0, 119.5, 118.4, 111.8, 111.2, 63.8 (q, J = 24.0 Hz), 58.6 (d, J = 1.4 Hz), 35.6, 22.5 (q, J = 1.9 Hz). **IR** (ATR, cm^{-1}) 3421, 3278, 3054, 2939, 2857, 2833, 1704, 1619, 1597, 1509, 1456, 1354, 1336, 1251, 1164, 1137, 1102, 1065, 1011, 859, 791, 774, 739, 692, 586, 458, 423. **HRMS** m/z $[M+H]^+$ calculated for $C_{23}H_{22}N_2F_3^+$: 383.1730, found: 383.1730.

Supplementary Figure 11: Unsuccessful substrates under conditions of aziridinium ion intermediate generation

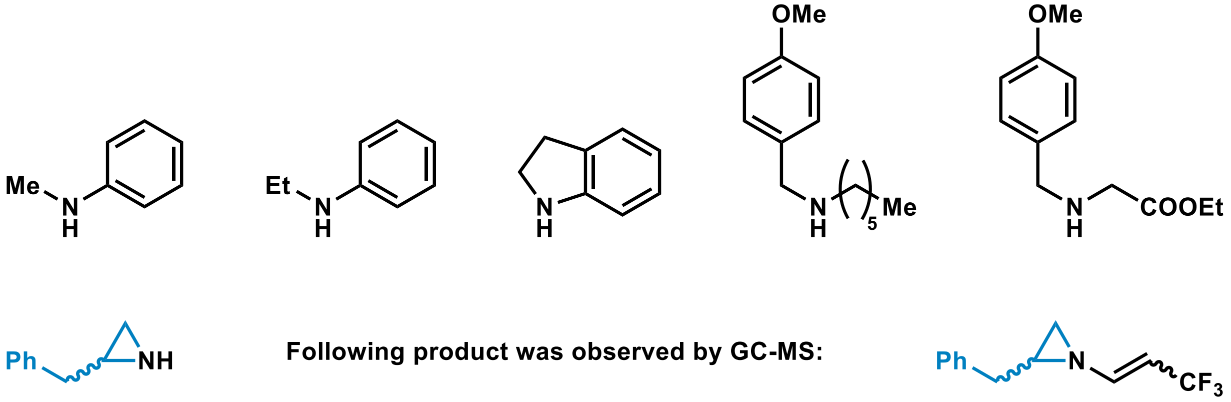

Supplementary Figure 12: Ethylene diamine and trifluoroethylamine moieties in natural products and drugs

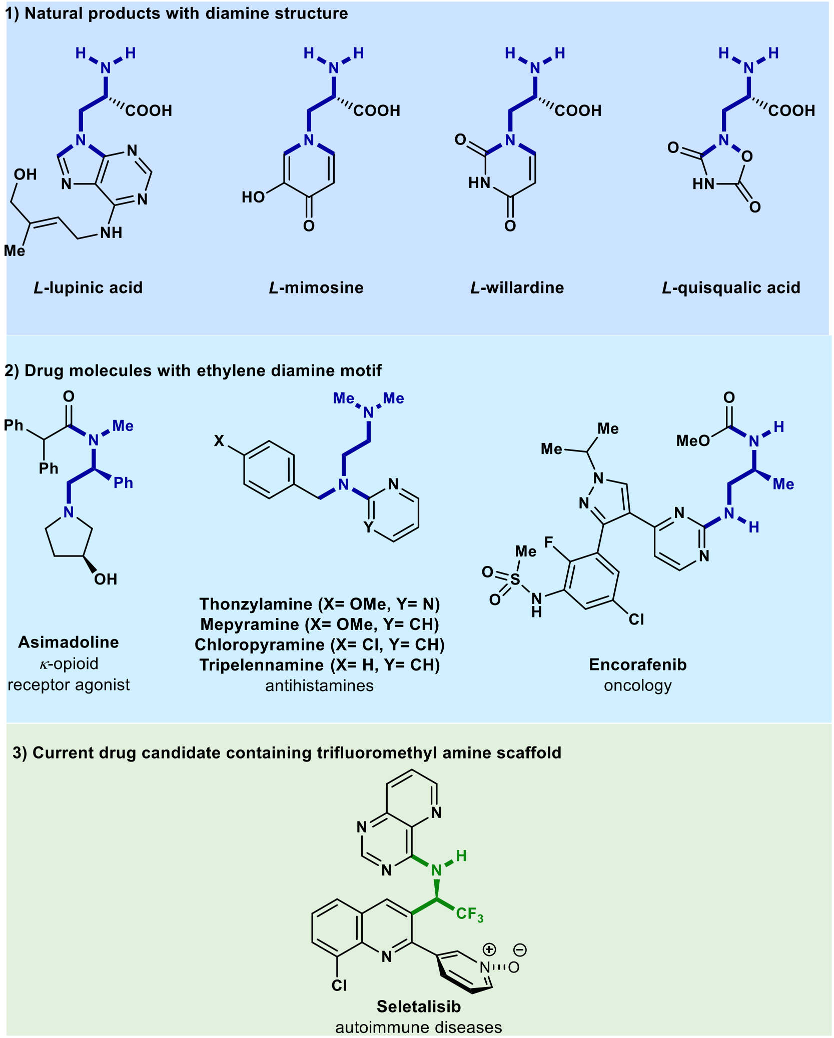

NMR & IR spectra

Supplementary Figure 13: *^{1}H-NMR for compound* ***1***

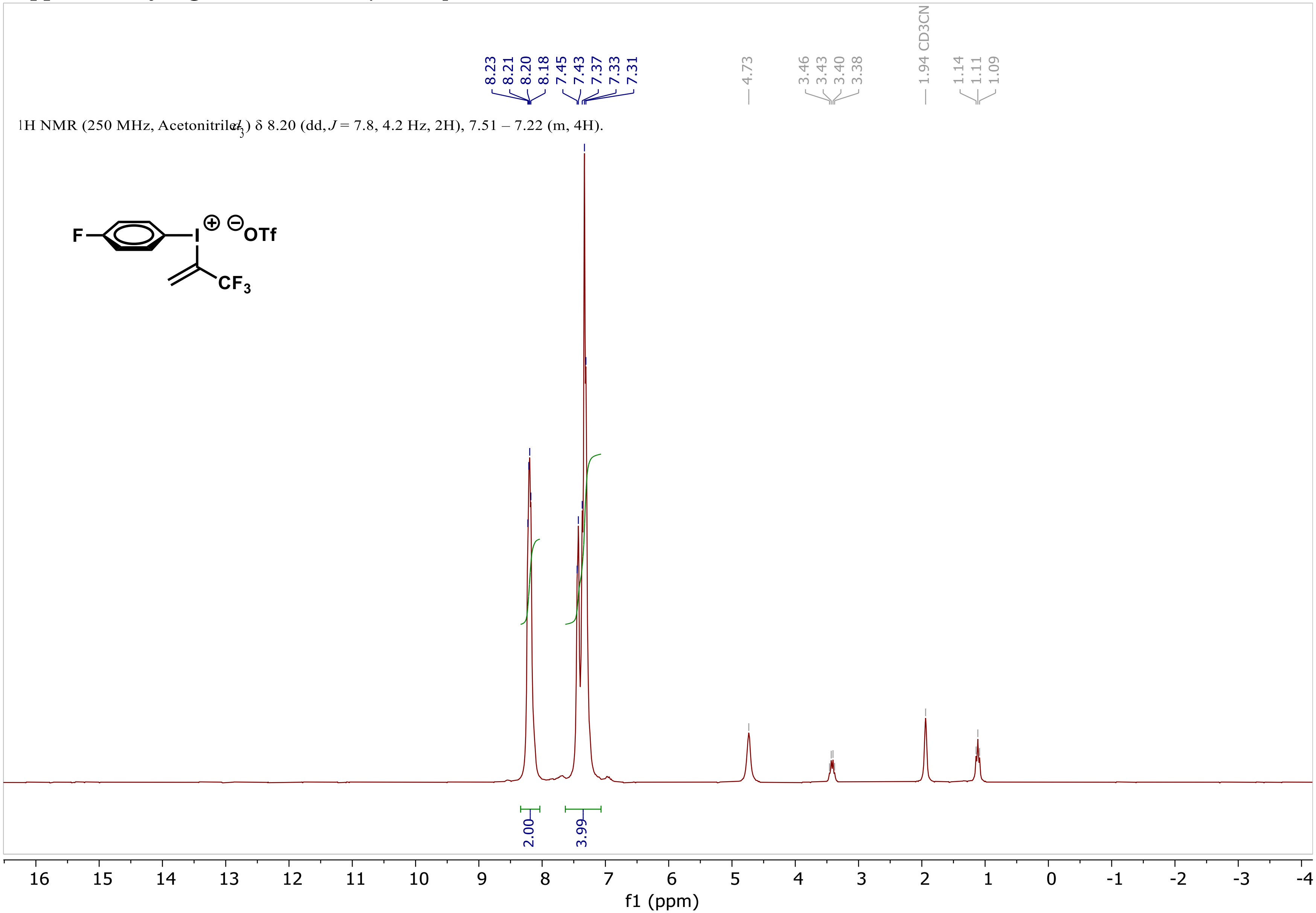

Supplementary Figure 14: *^{19}F NMR for compound* ***1***

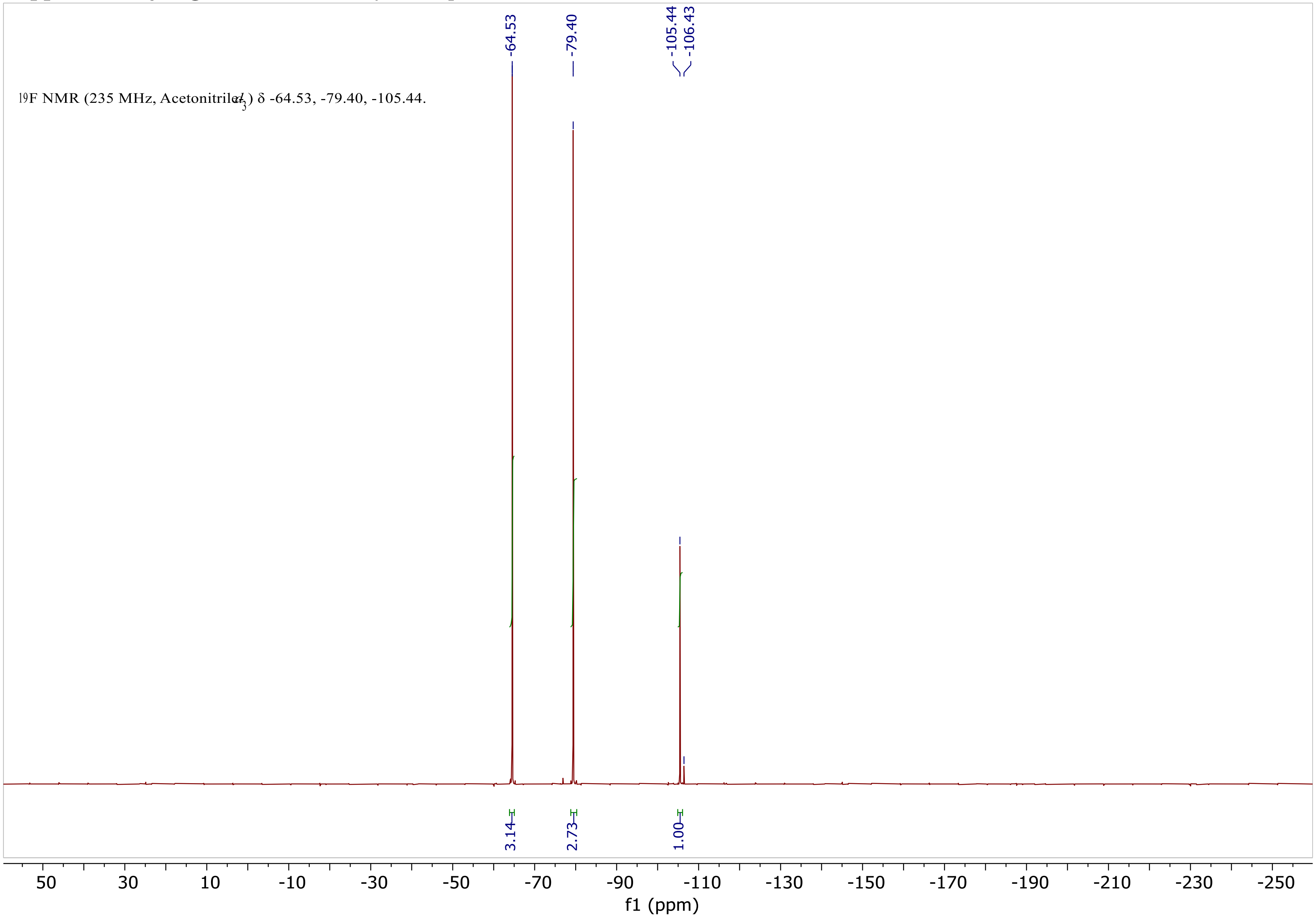

Supplementary Figure 15: *^{13}C NMR for compound* ***1***

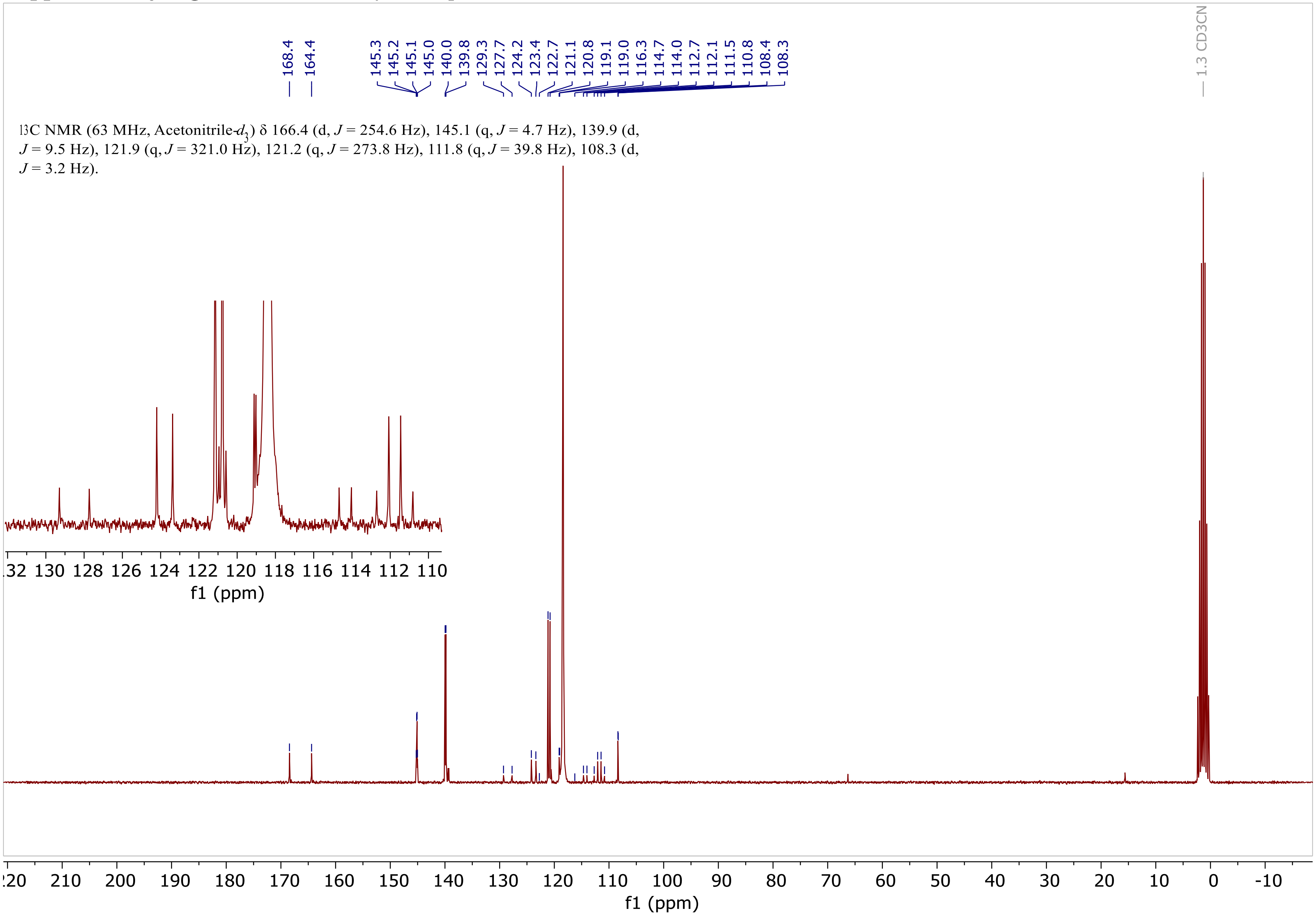

Supplementary Figure 16: *IR for compound* ***1***

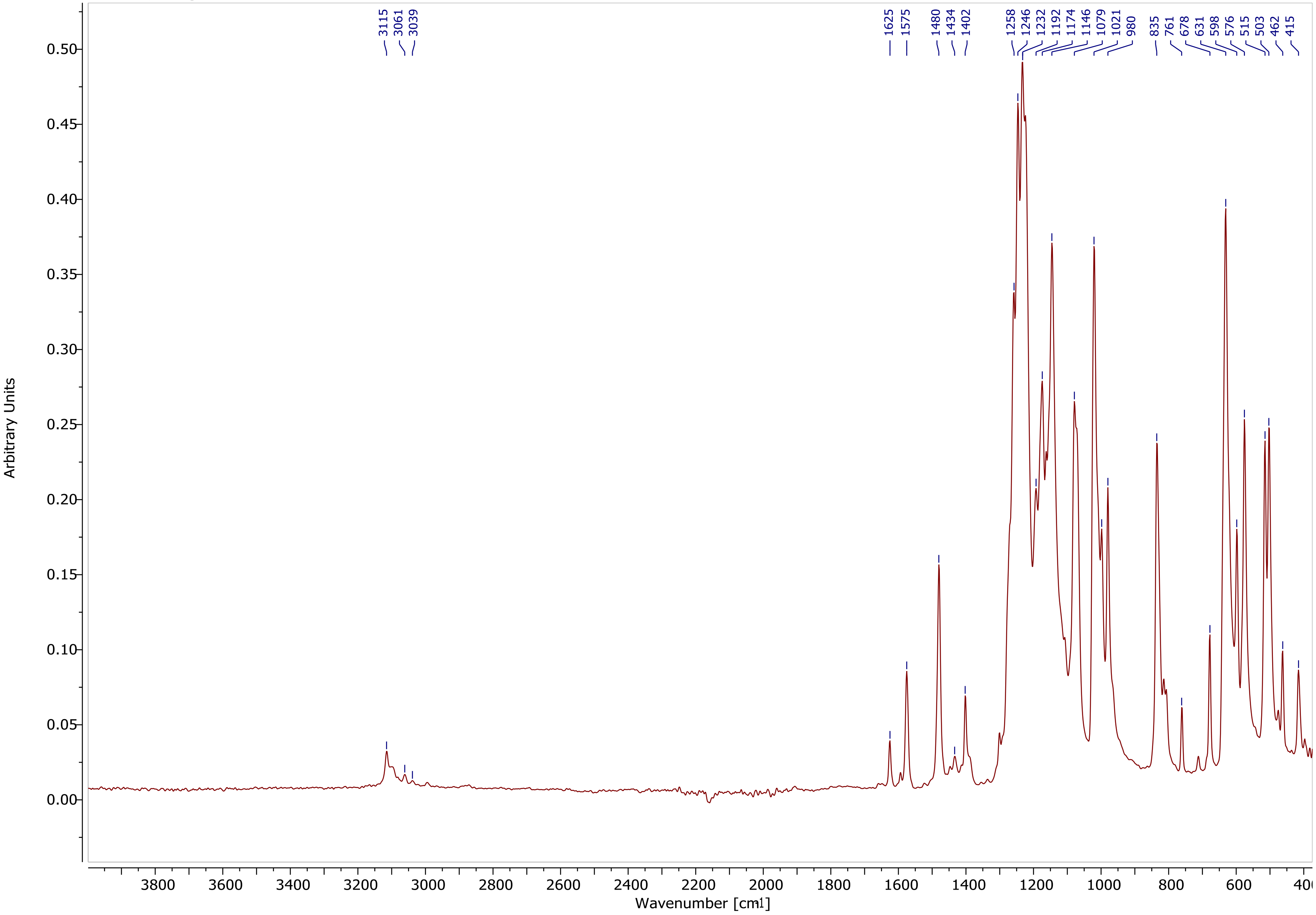

Supplementary Figure 17: *^{1}H-NMR for compound* ***A1***

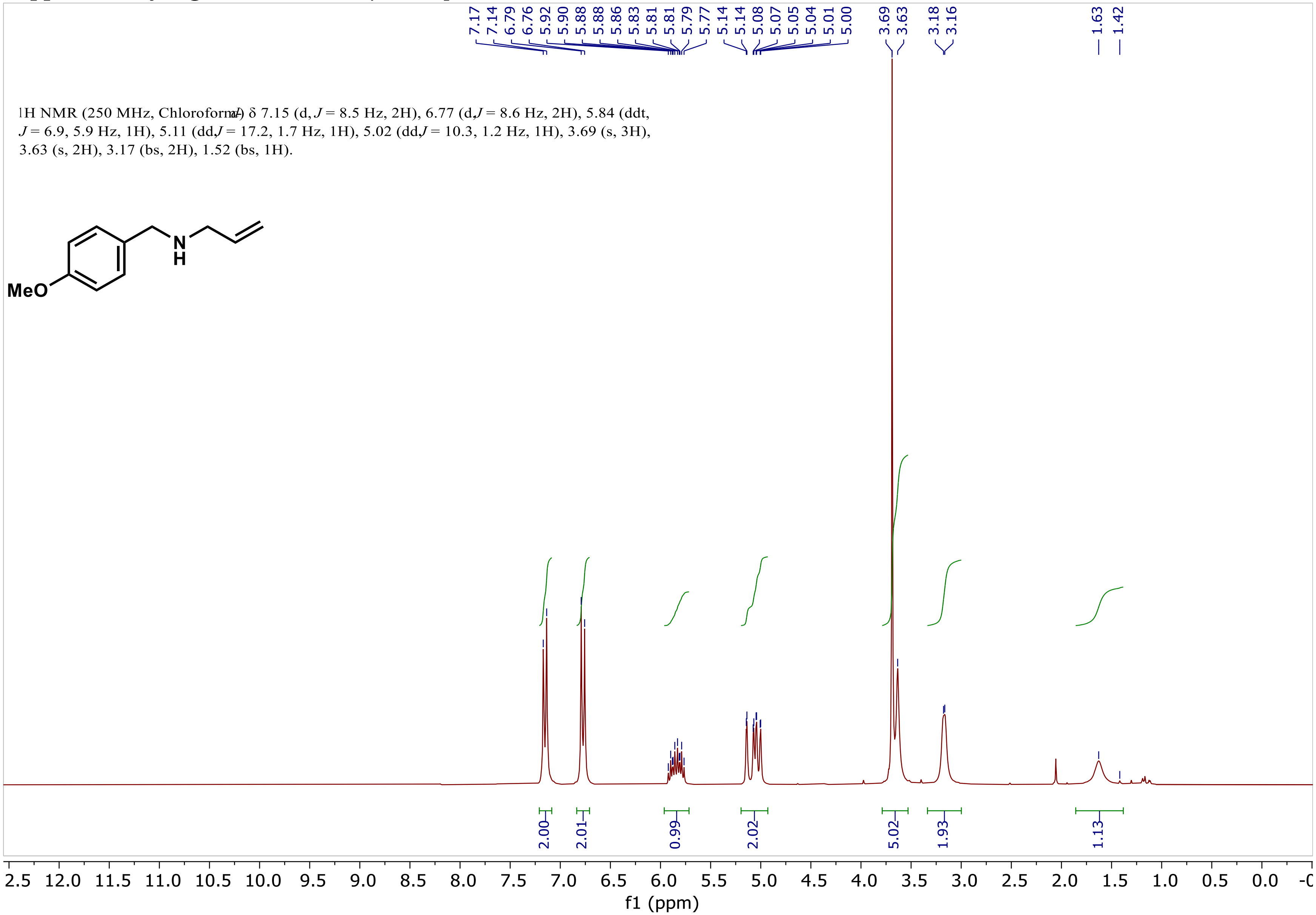

Supplementary Figure 18: ^{13}*C NMR for compound* ***A1***

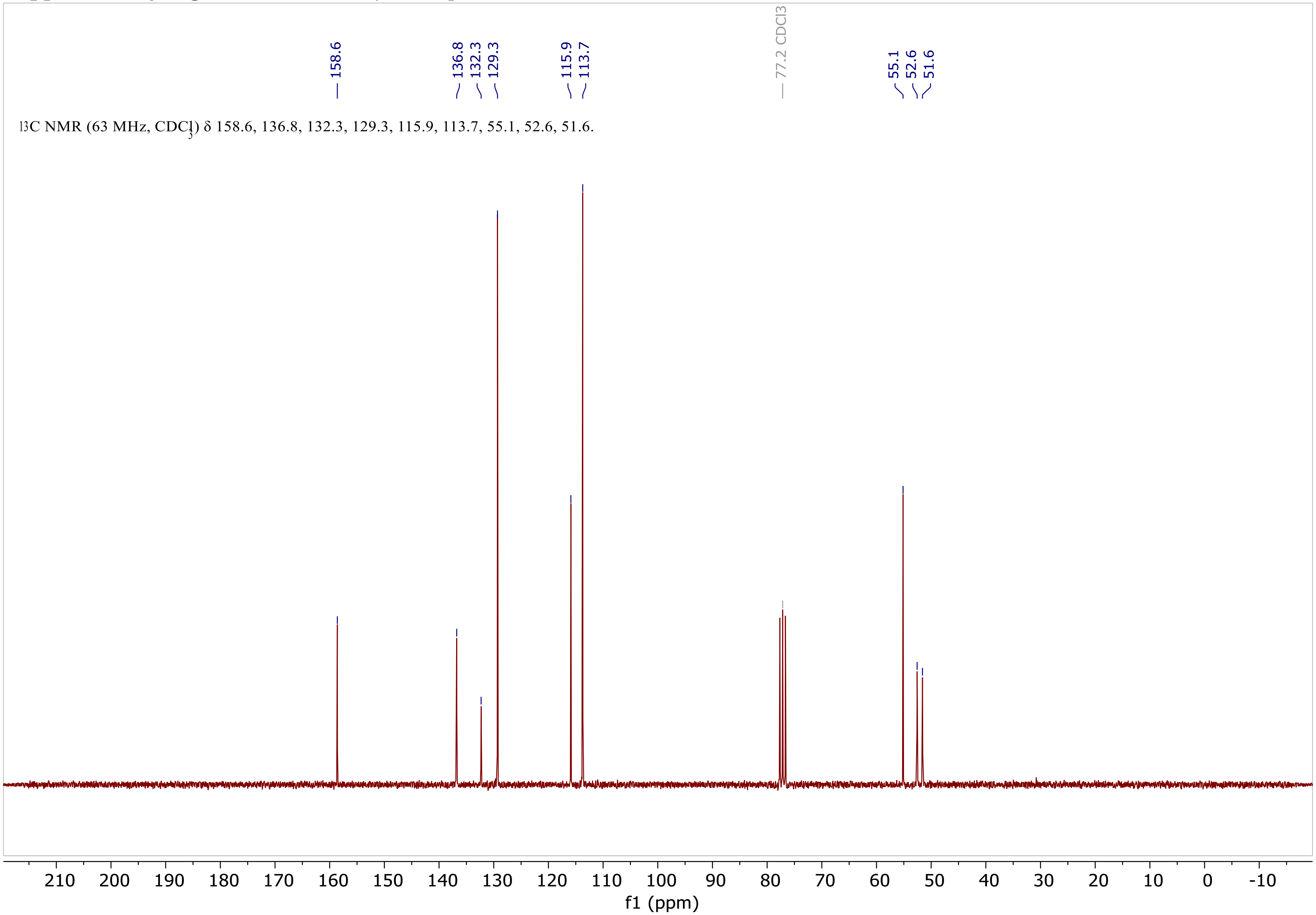

Supplementary Figure 19: *^{1}H-NMR for compound* ***A2***

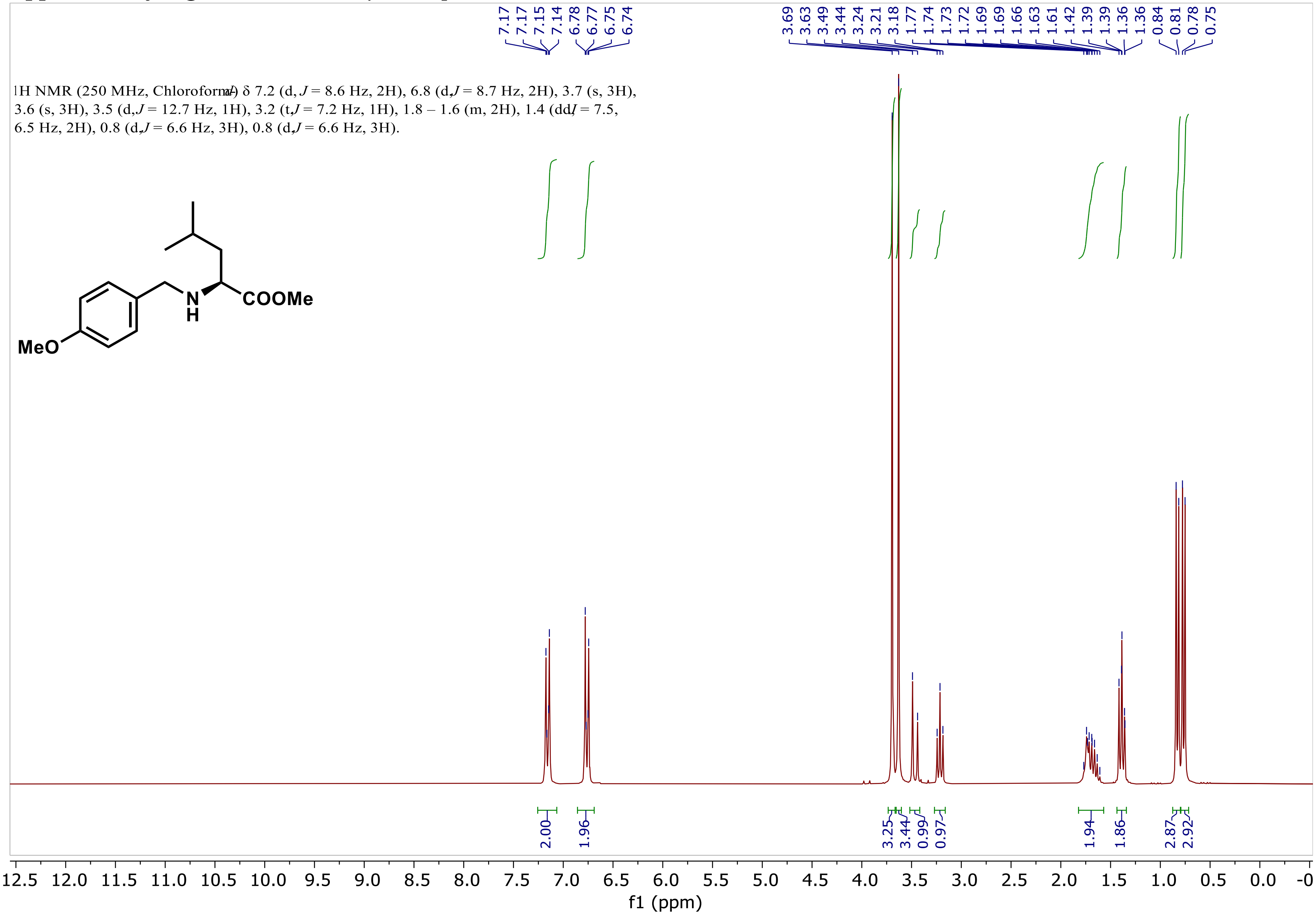

Supplementary Figure 20: *^{13}C NMR for compound* ***A2***

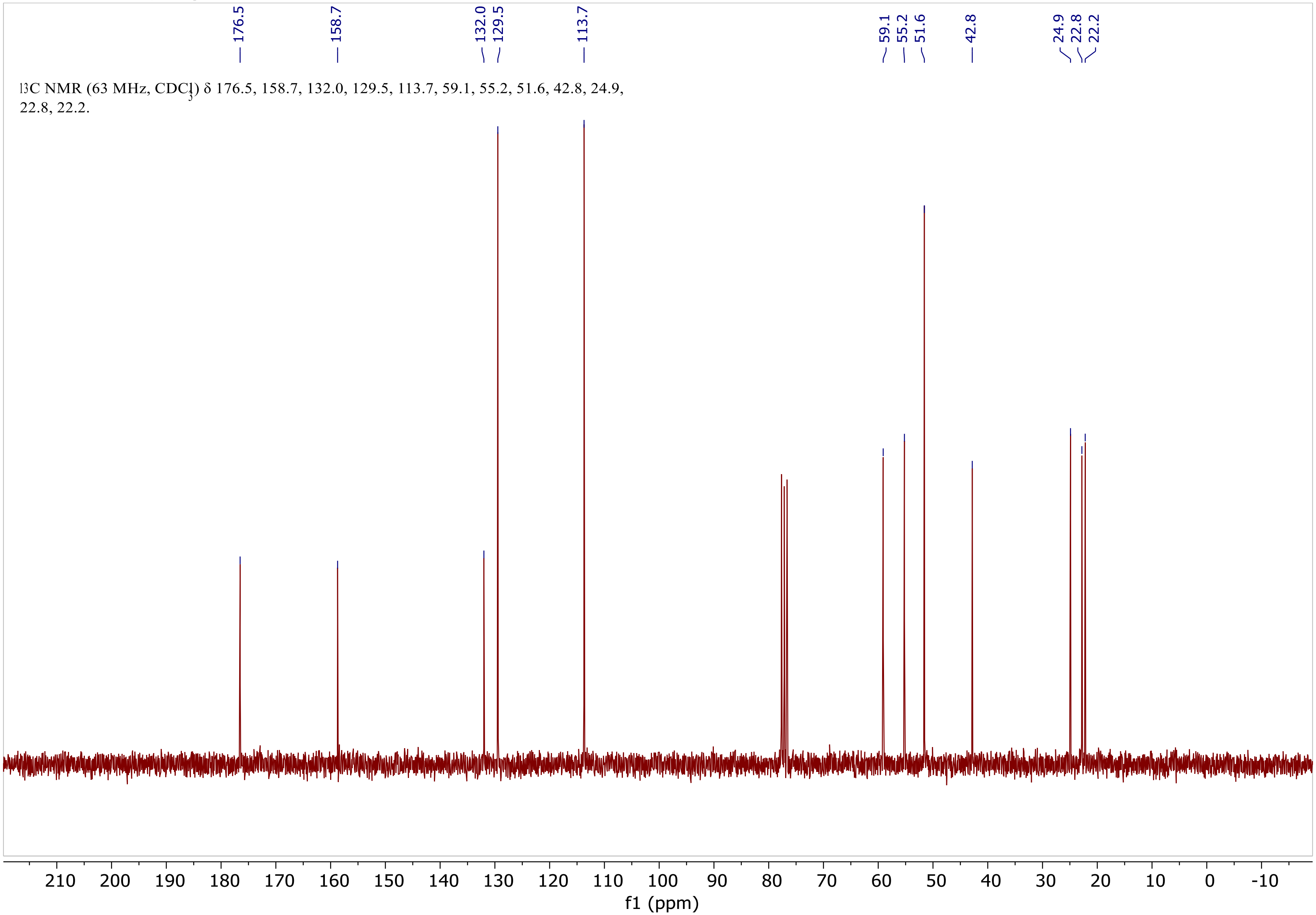

Supplementary Figure 21: *^{1}H-NMR for compound* ***A3***

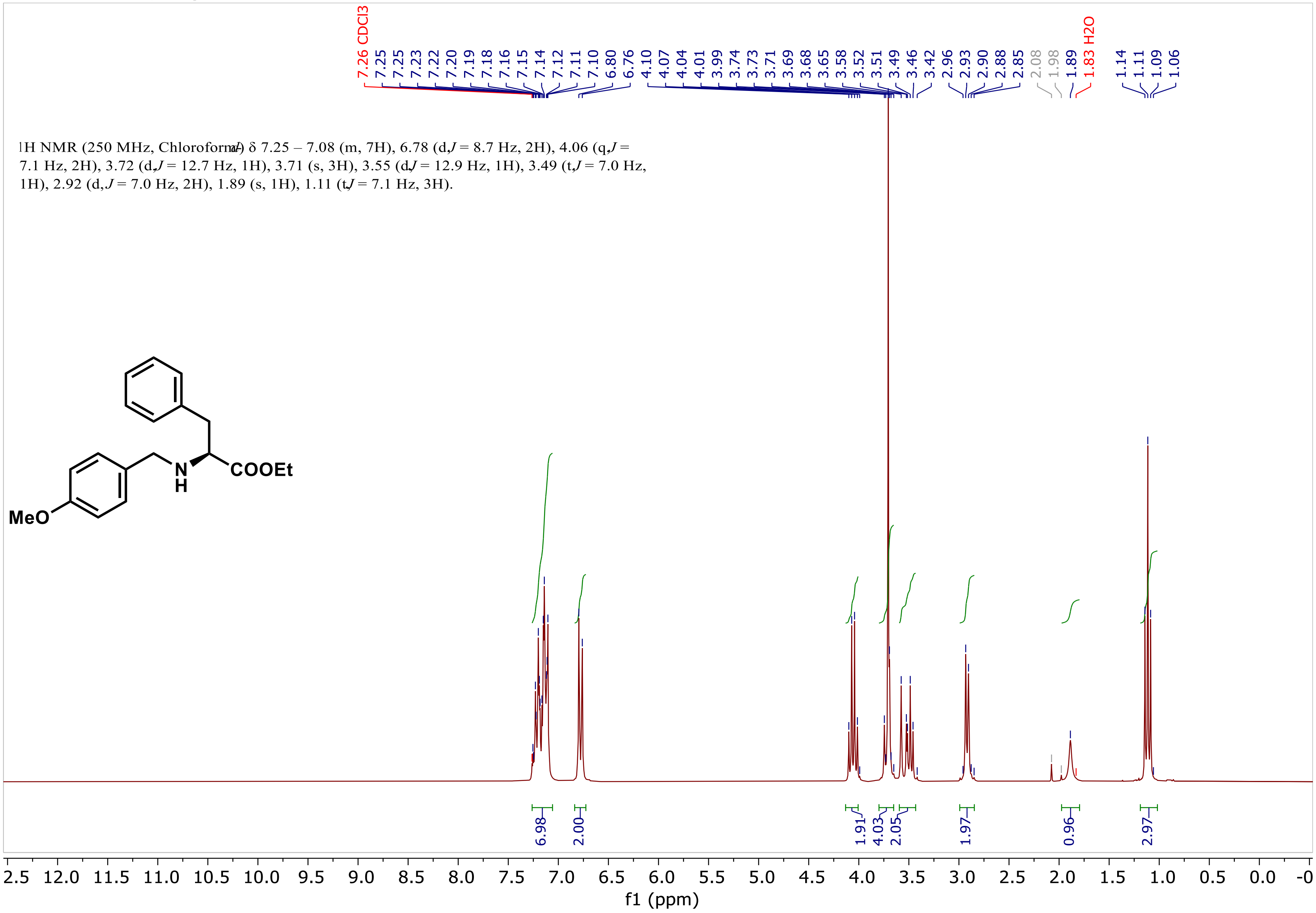

Supplementary Figure 22: *^{13}C NMR for compound* ***A3***

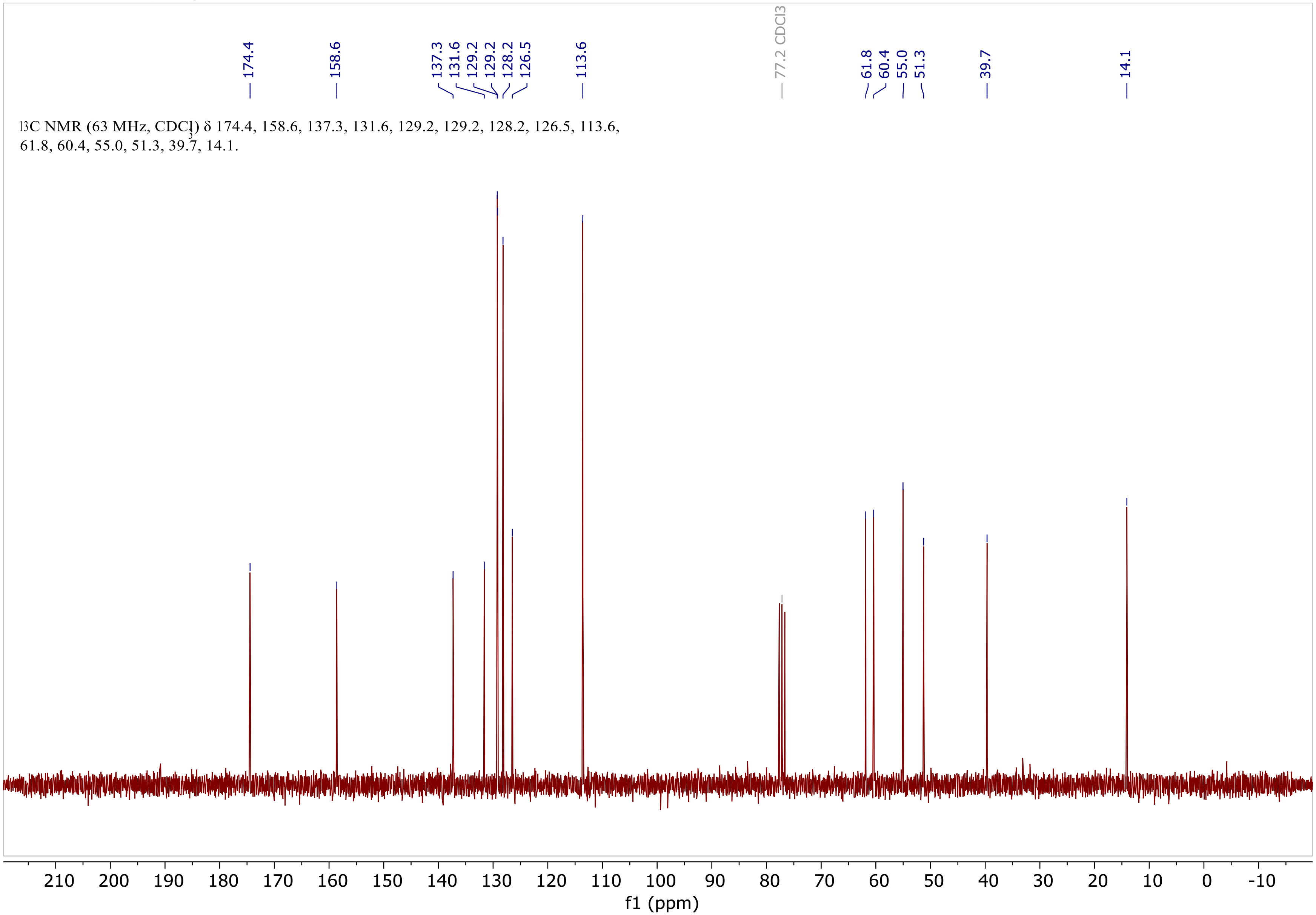

Supplementary Figure 23: *¹H-NMR for compound* ***A4***

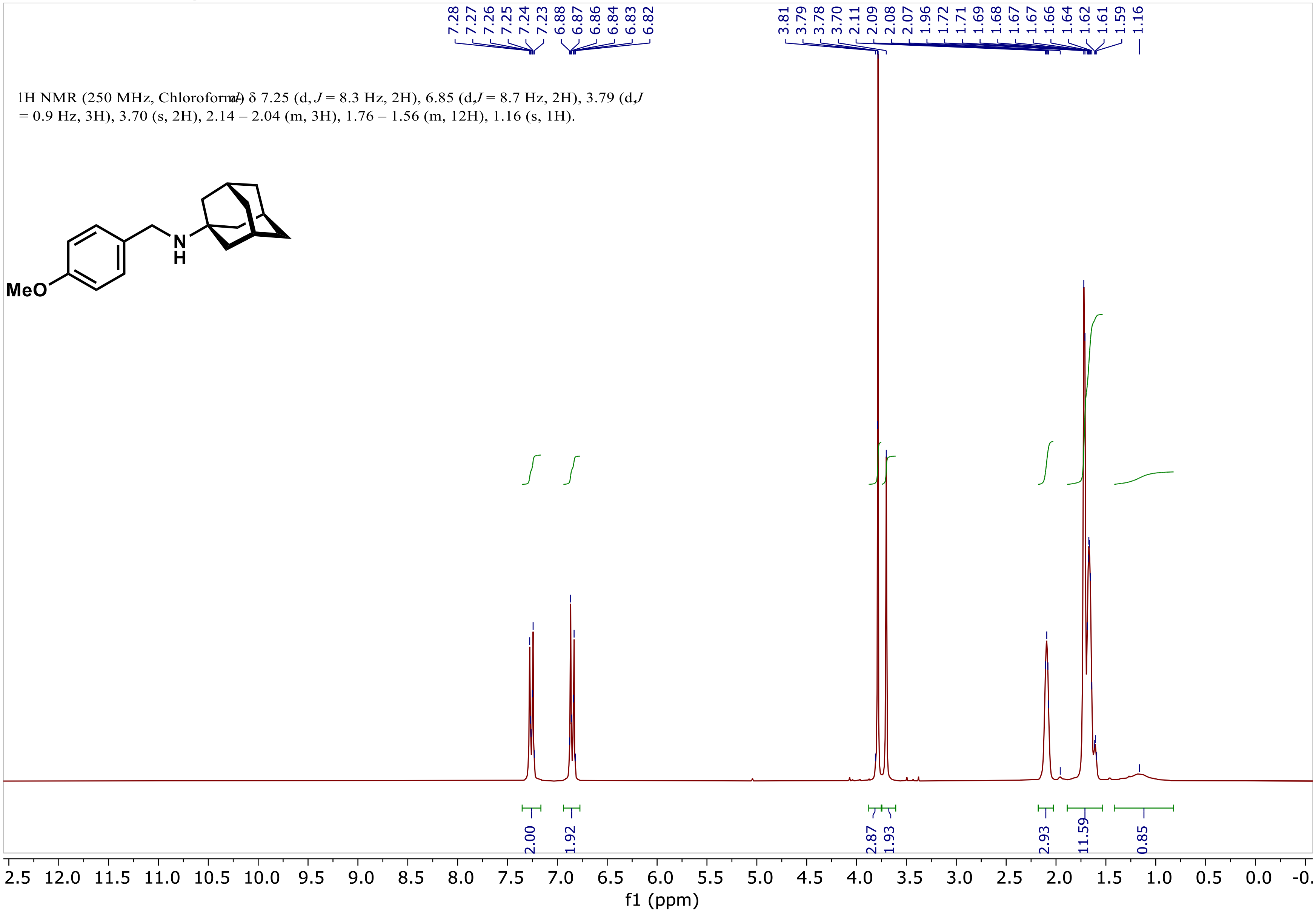

Supplementary Figure 24: *^{13}C NMR for compound* ***A4***

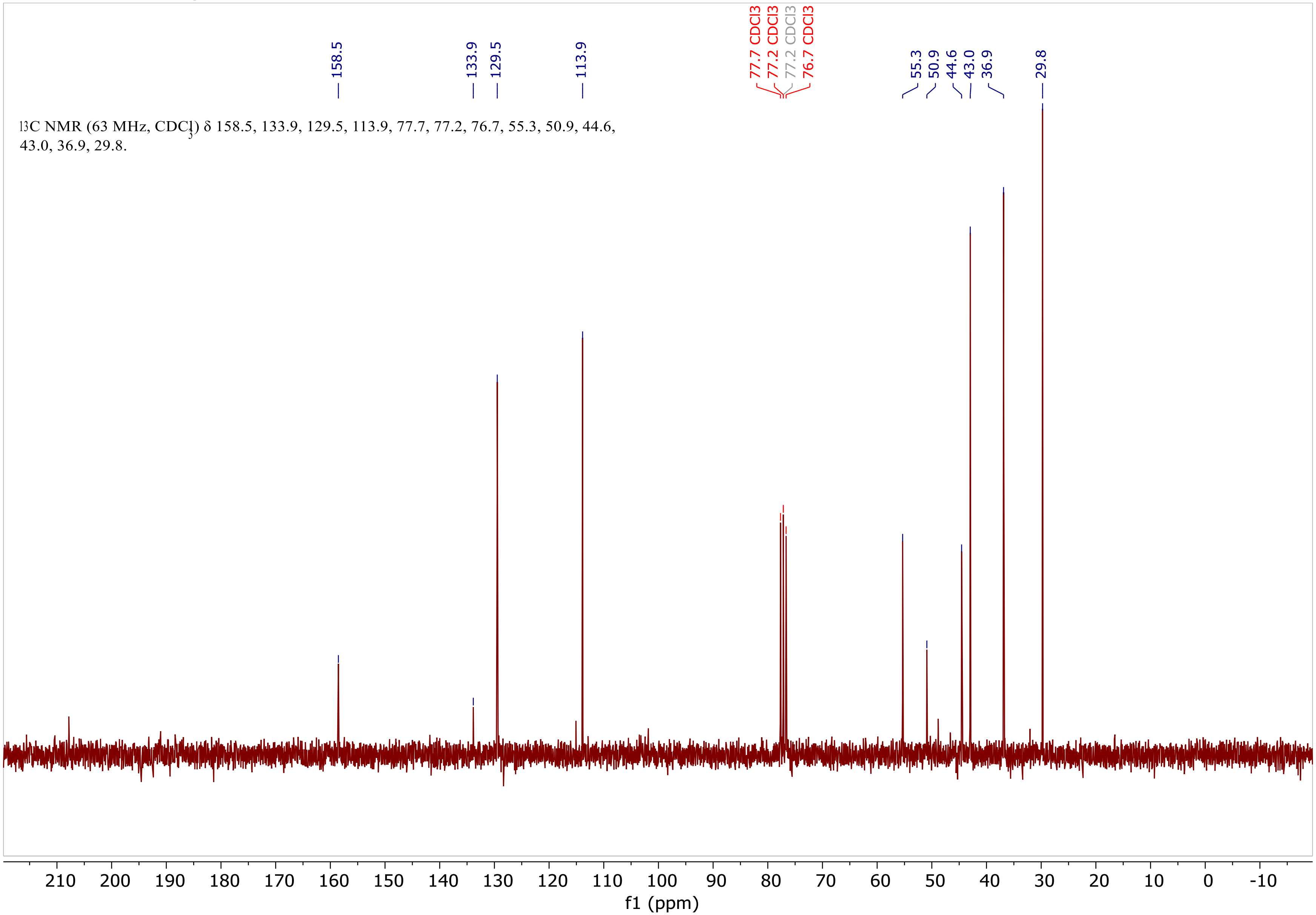

Supplementary Figure 25: *^{1}H-NMR for compound* ***A5***

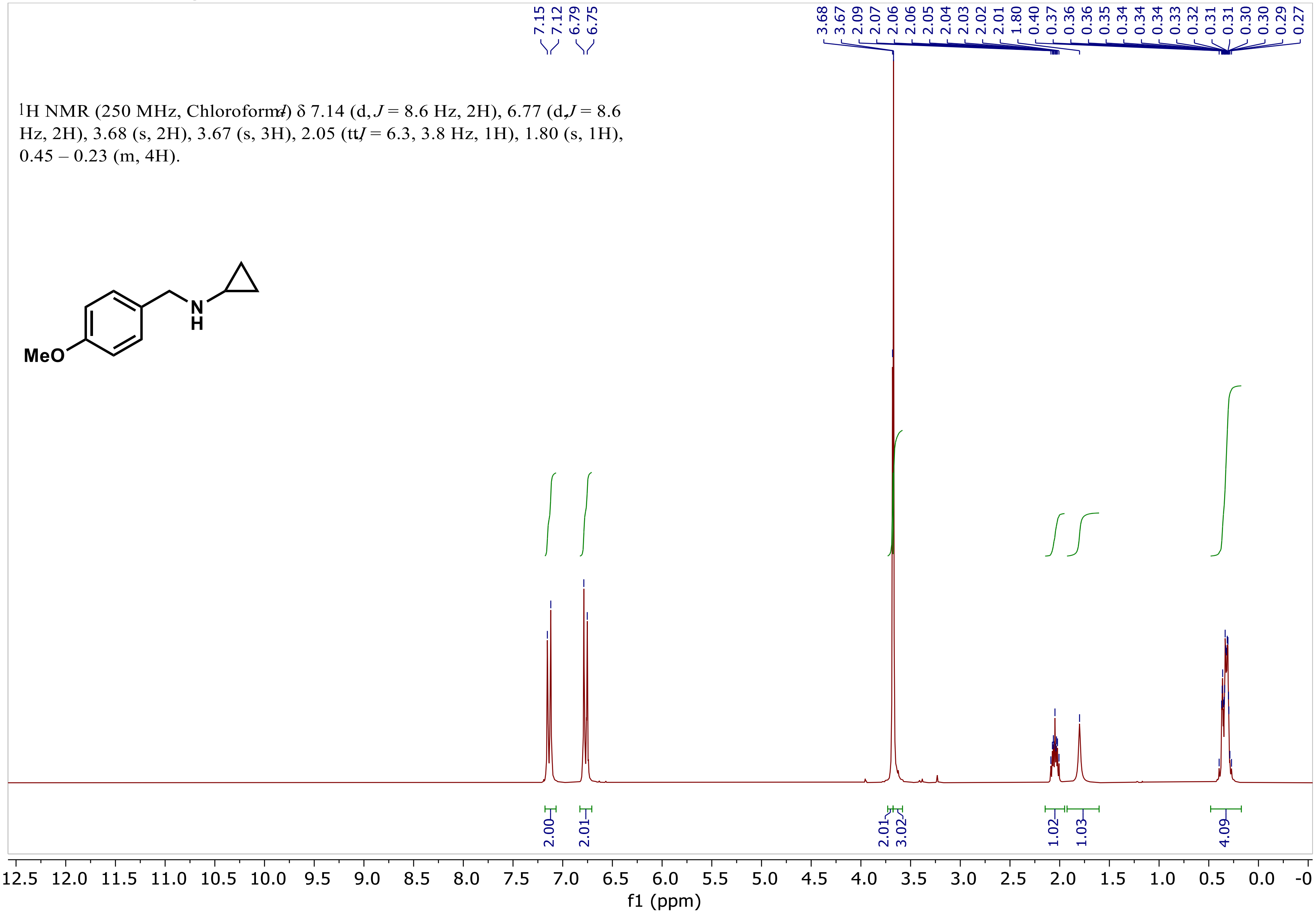

Supplementary Figure 26: ^{13}*C NMR for compound* ***A5***

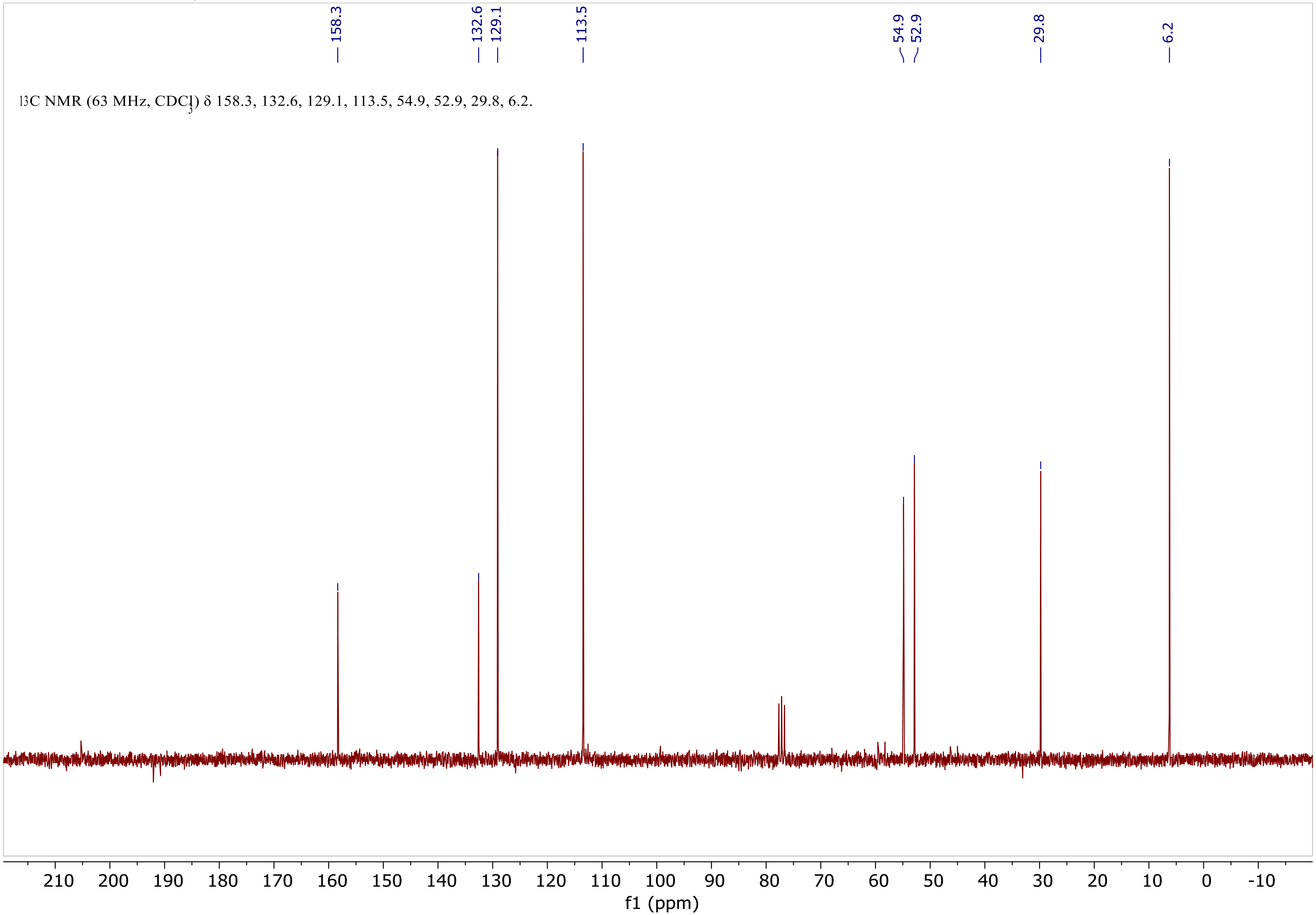

Supplementary Figure 27: *^{1}H-NMR for compound* ***A6***

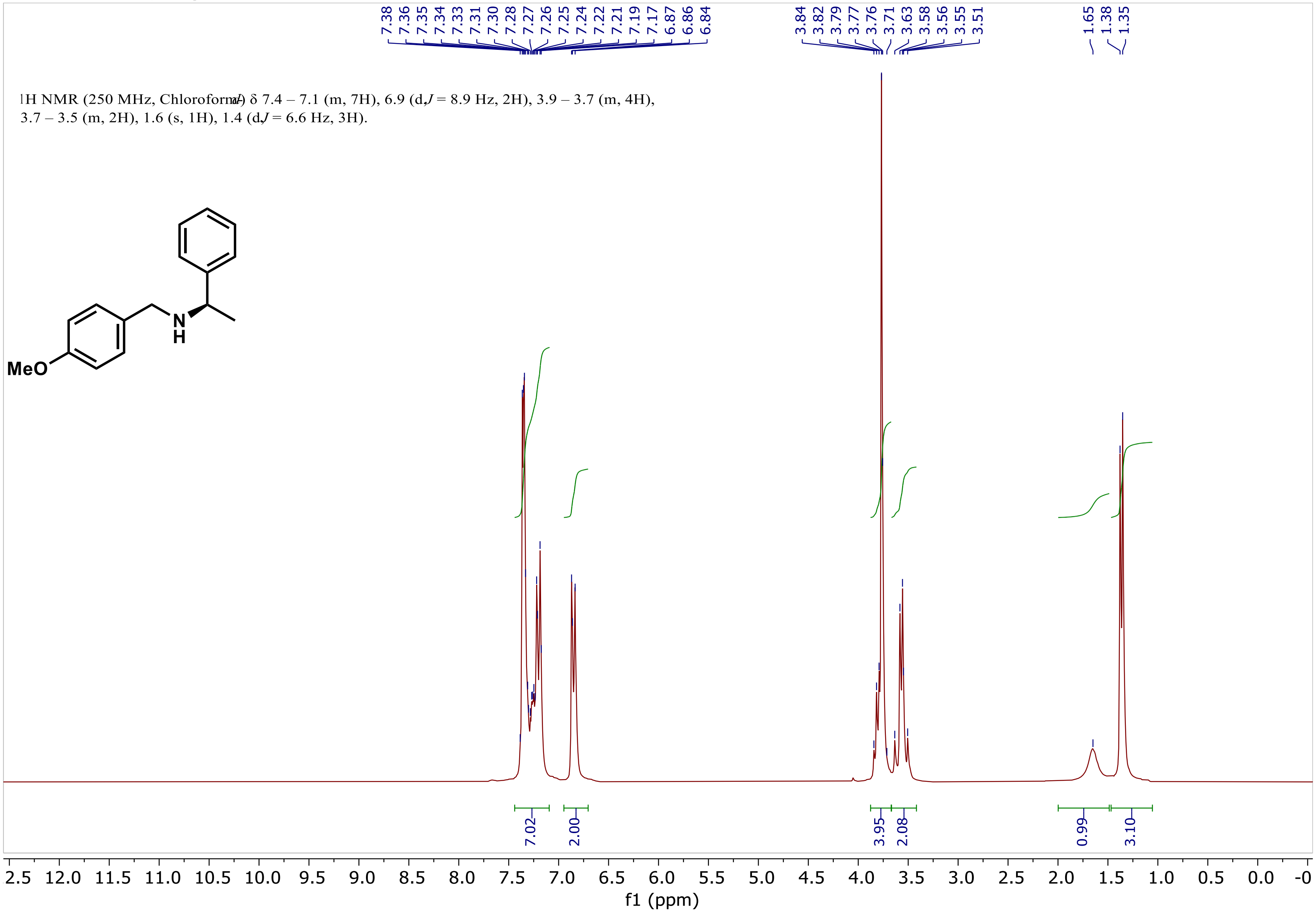

Supplementary Figure 28: *^{13}C NMR for compound* ***A6***

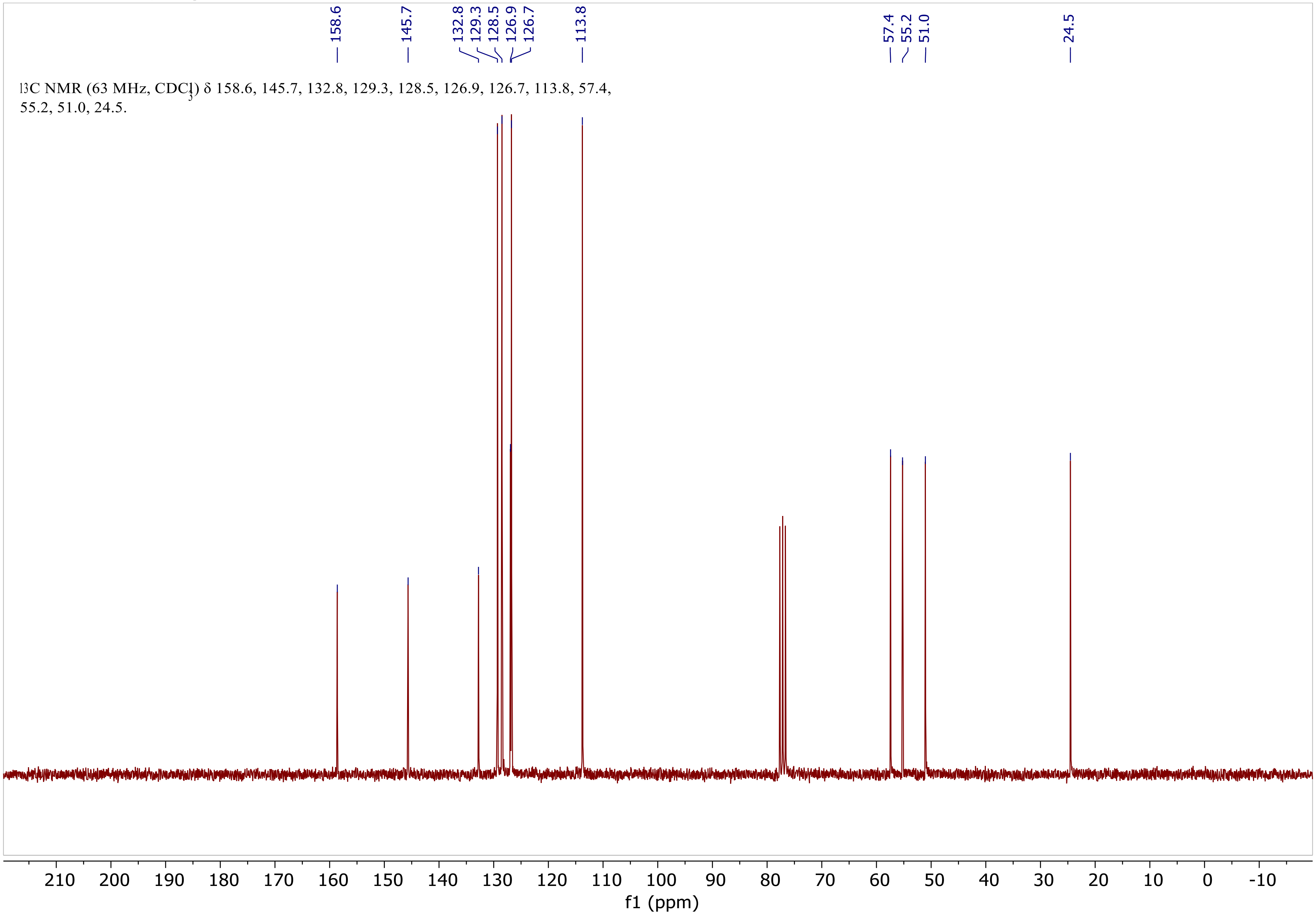

Supplementary Figure 29: *^{1}H-NMR for compound* ***A7***

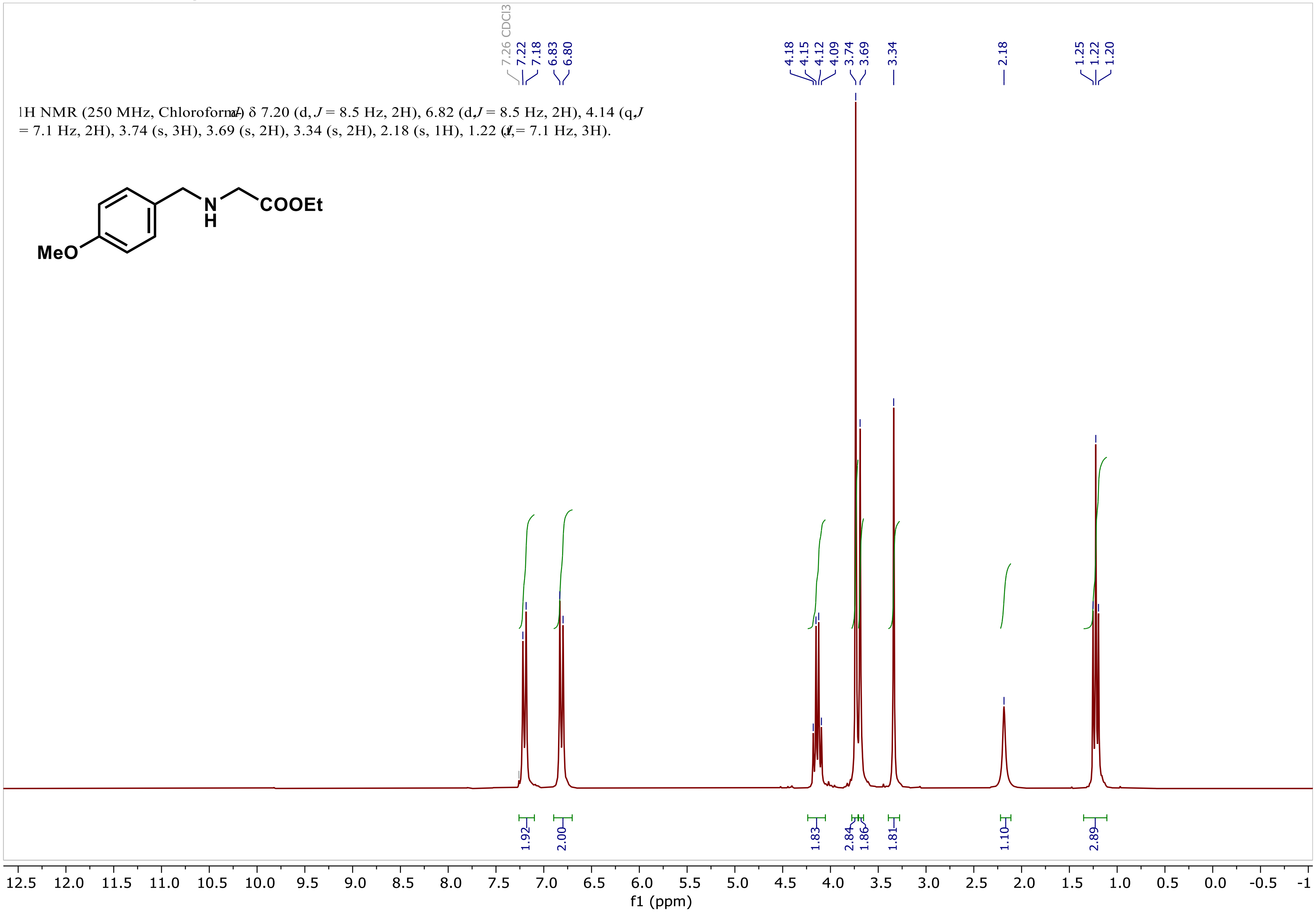

Supplementary Figure 30: *^{13}C NMR for compound* ***A7***

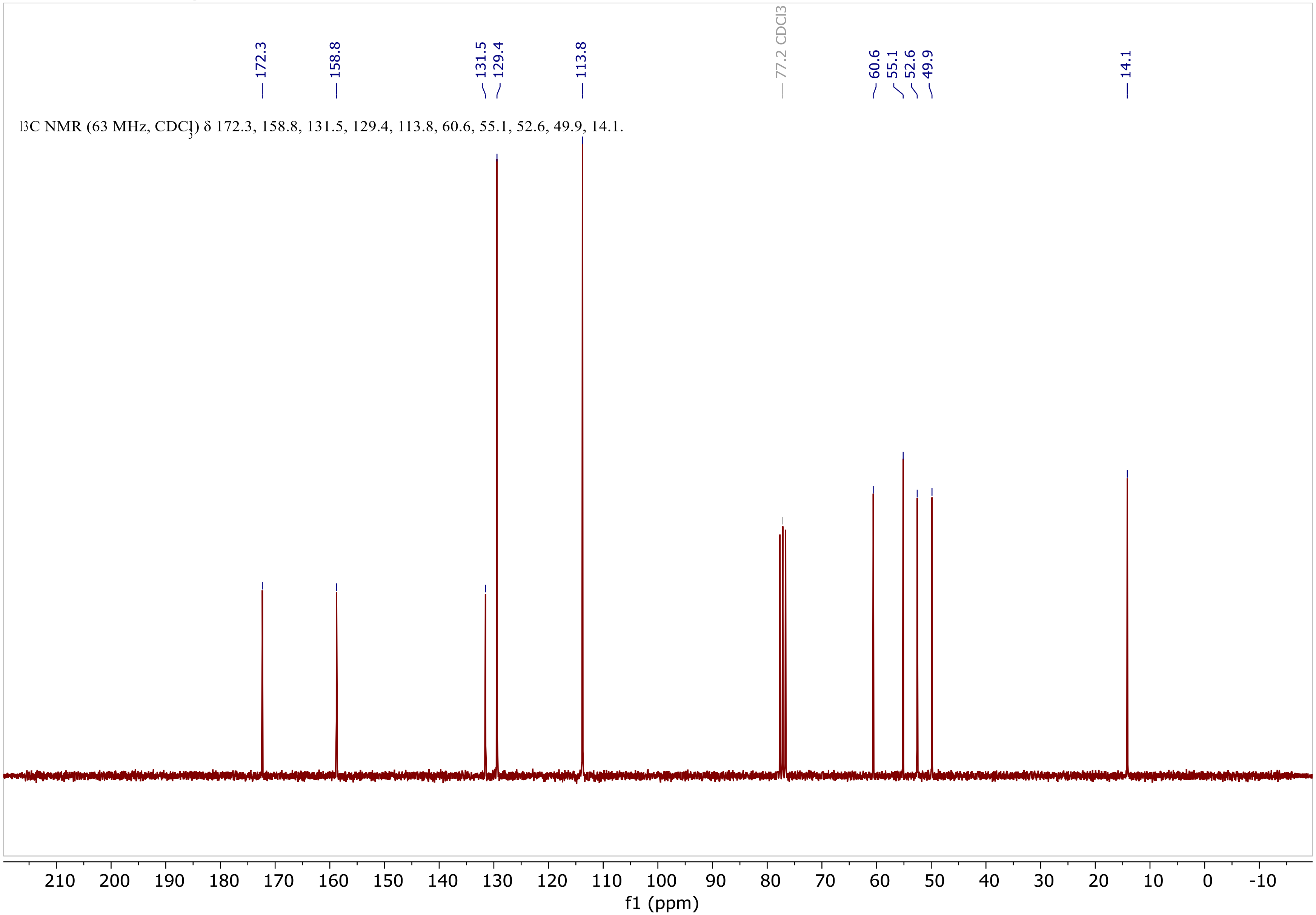

Supplementary Figure 31: *^{1}H-NMR for compound **A8***

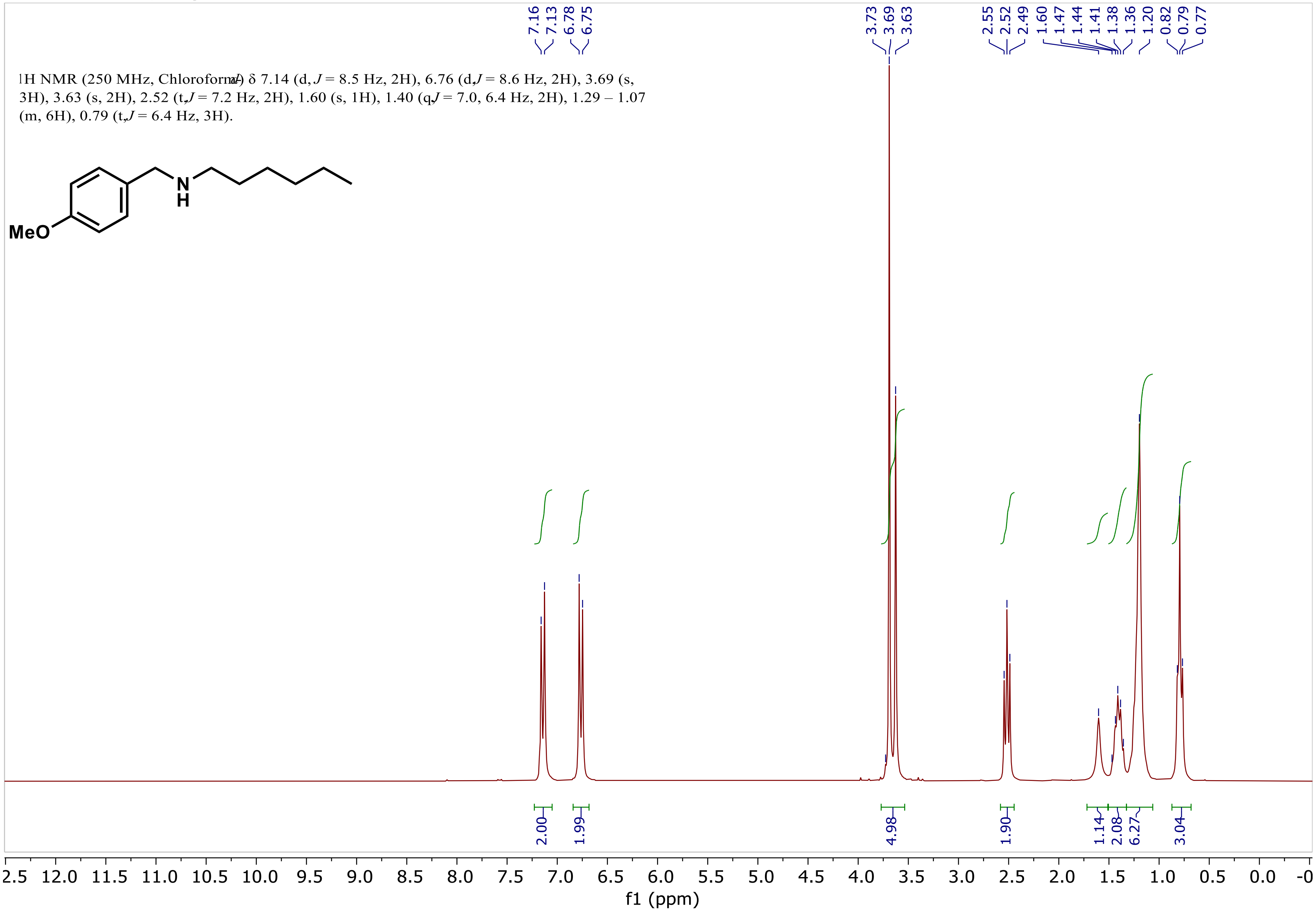

Supplementary Figure 32: *^{13}C NMR for compound* ***A8***

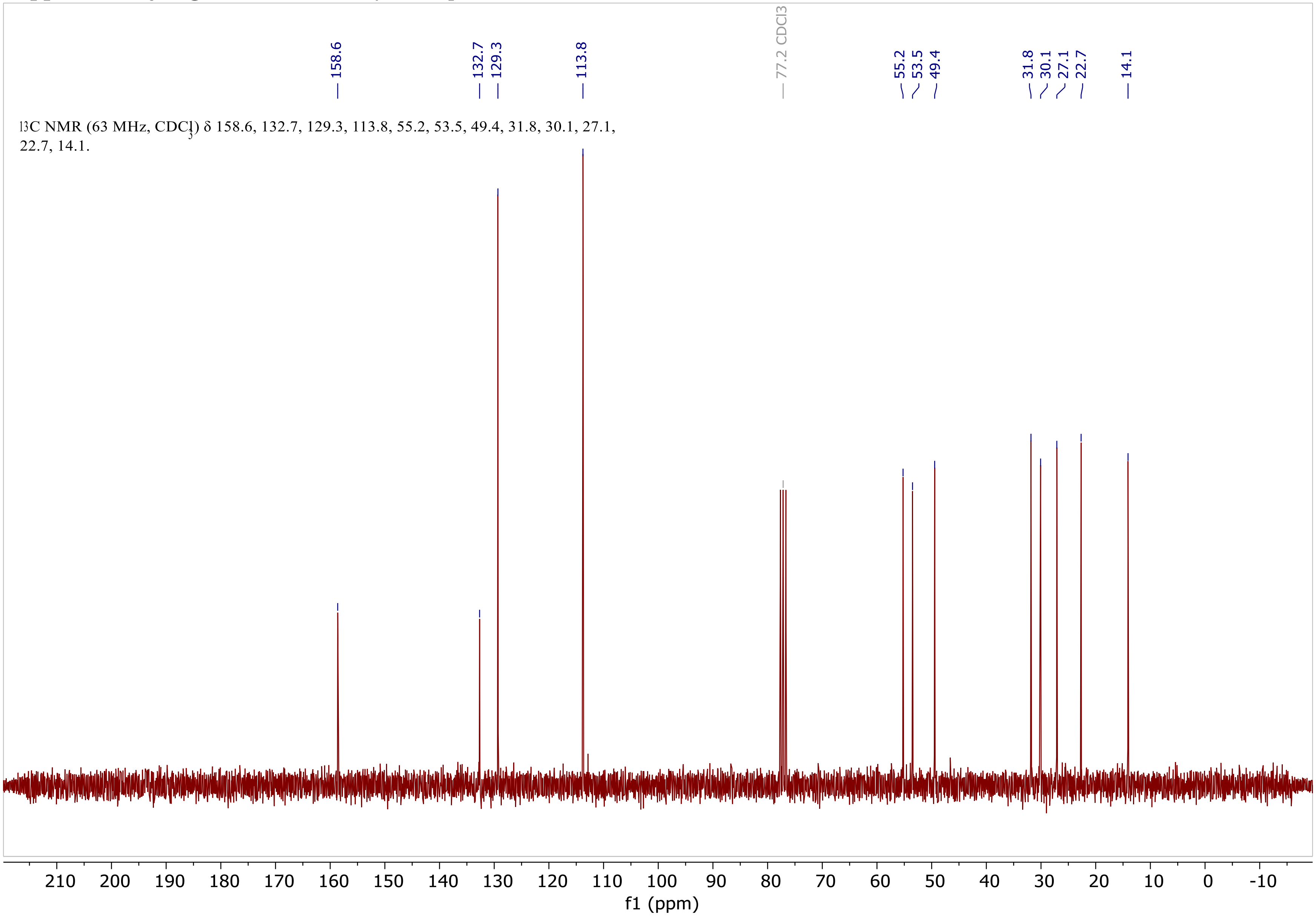

Supplementary Figure 33: ^{1}*H-NMR for compound* ***A9***

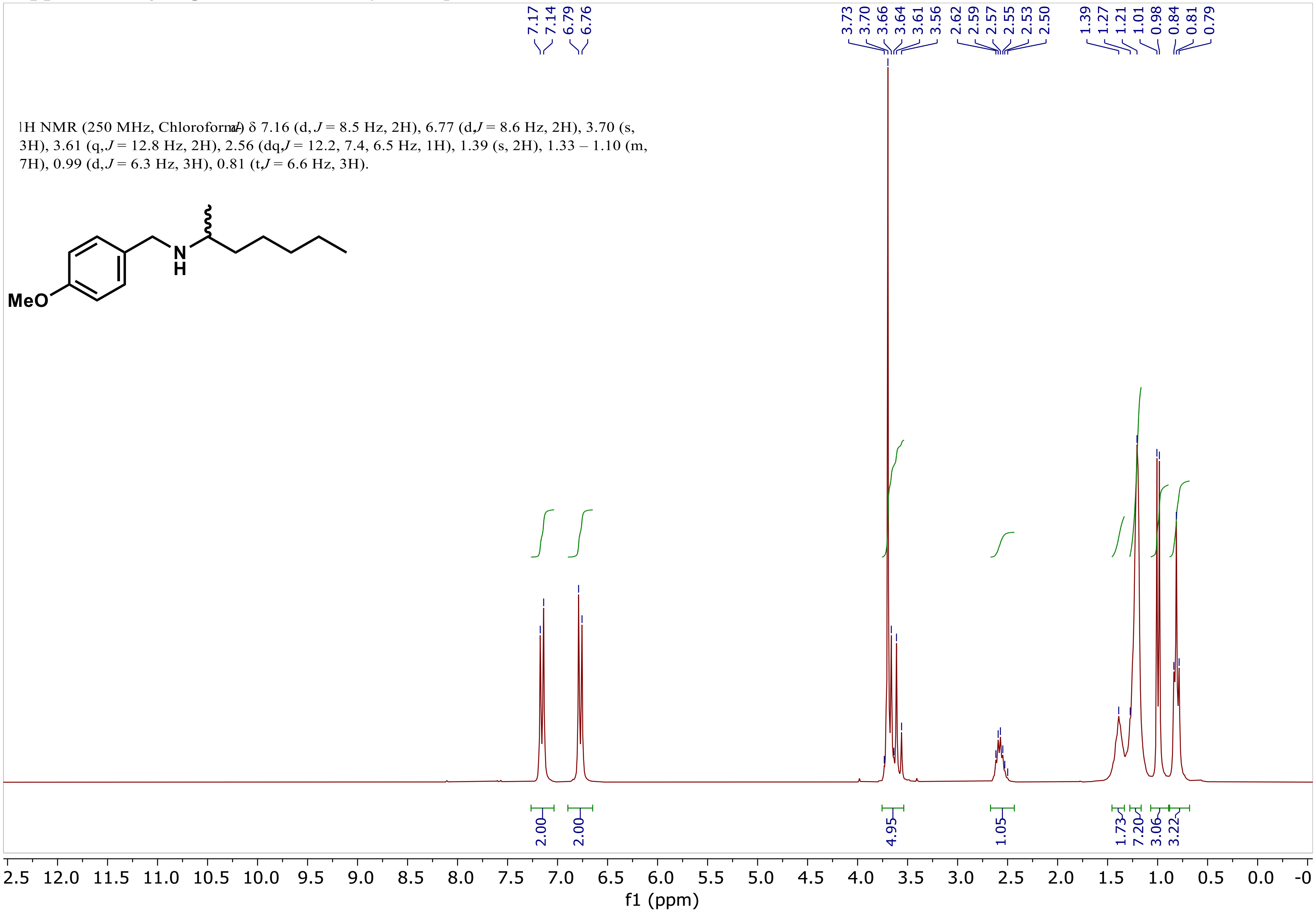

Supplementary Figure 34: *^{13}C NMR for compound* ***A9***

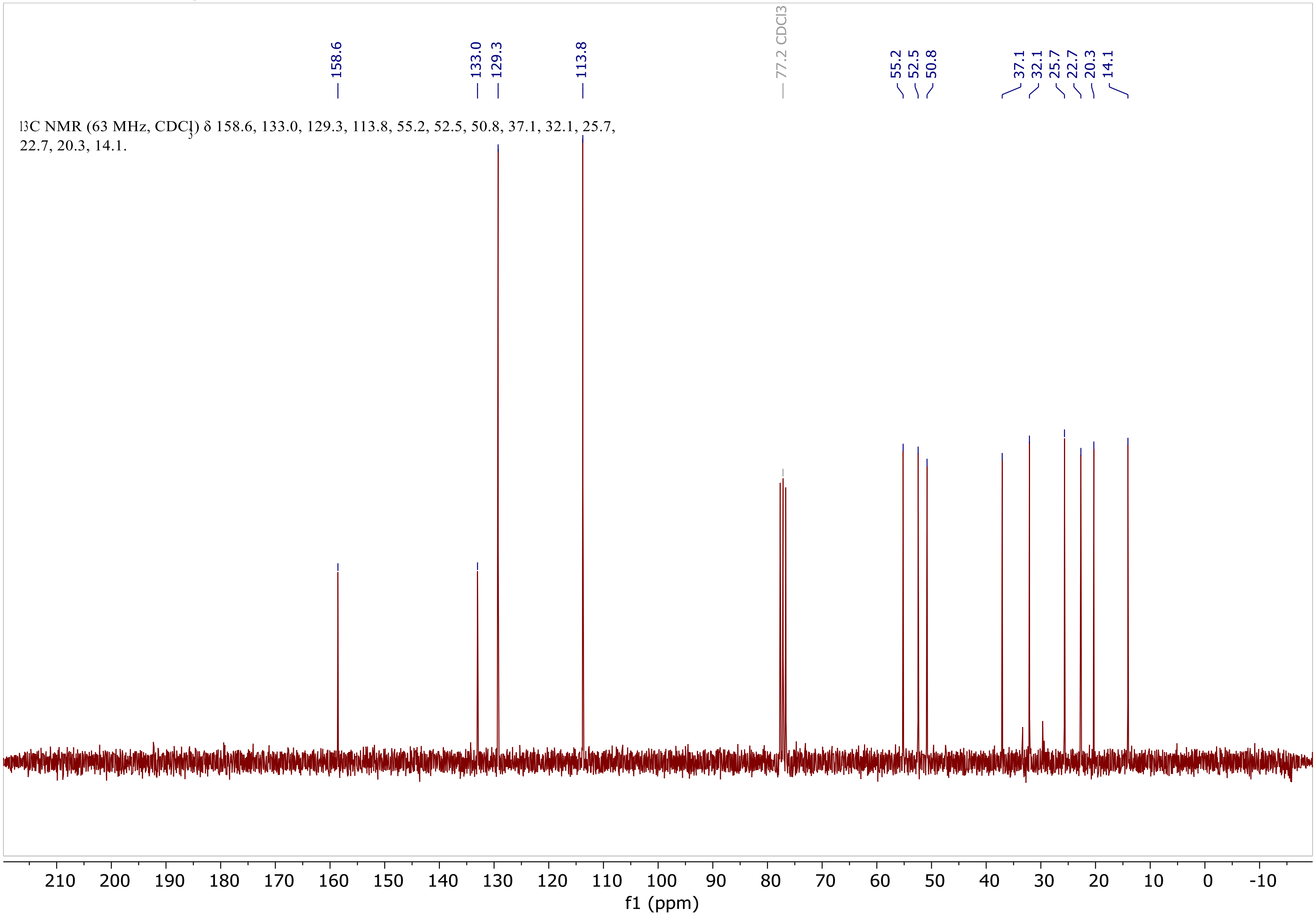

Supplementary Figure 35: *^{1}H-NMR for compound* ***A10***

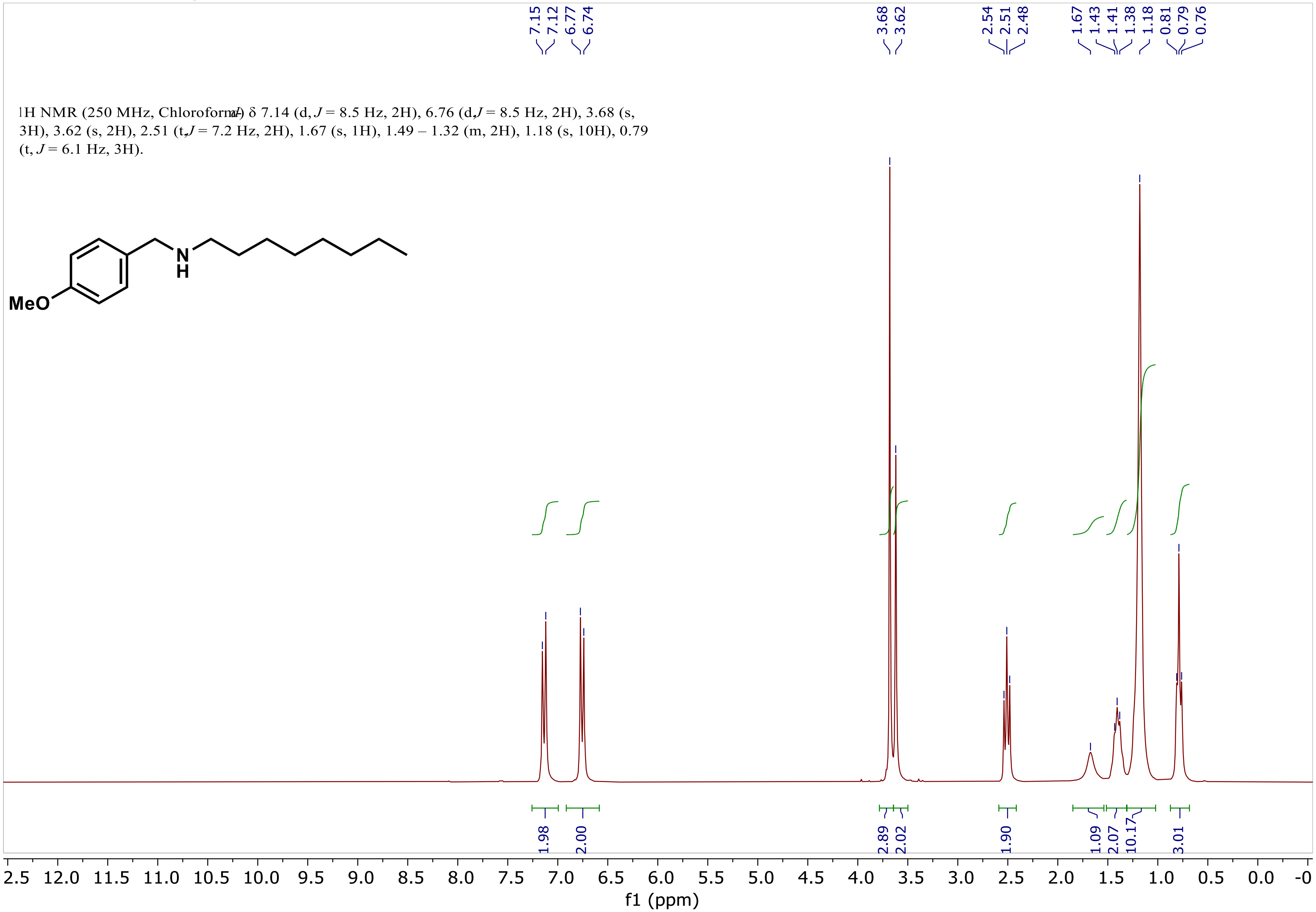

Supplementary Figure 36: *^{13}C NMR for compound* ***A10***

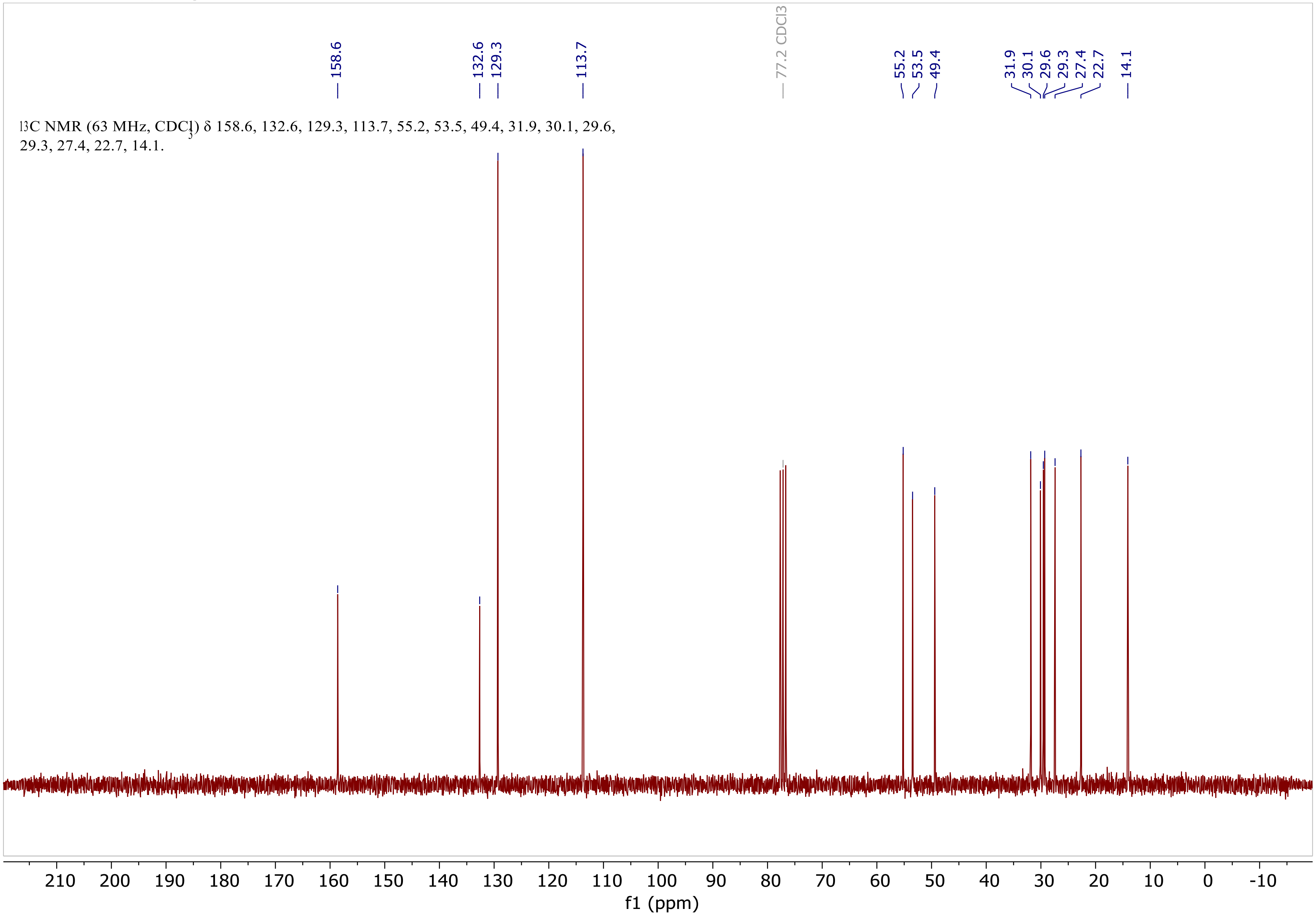

Supplementary Figure 37: *^{1}H-NMR for compound* ***A11***

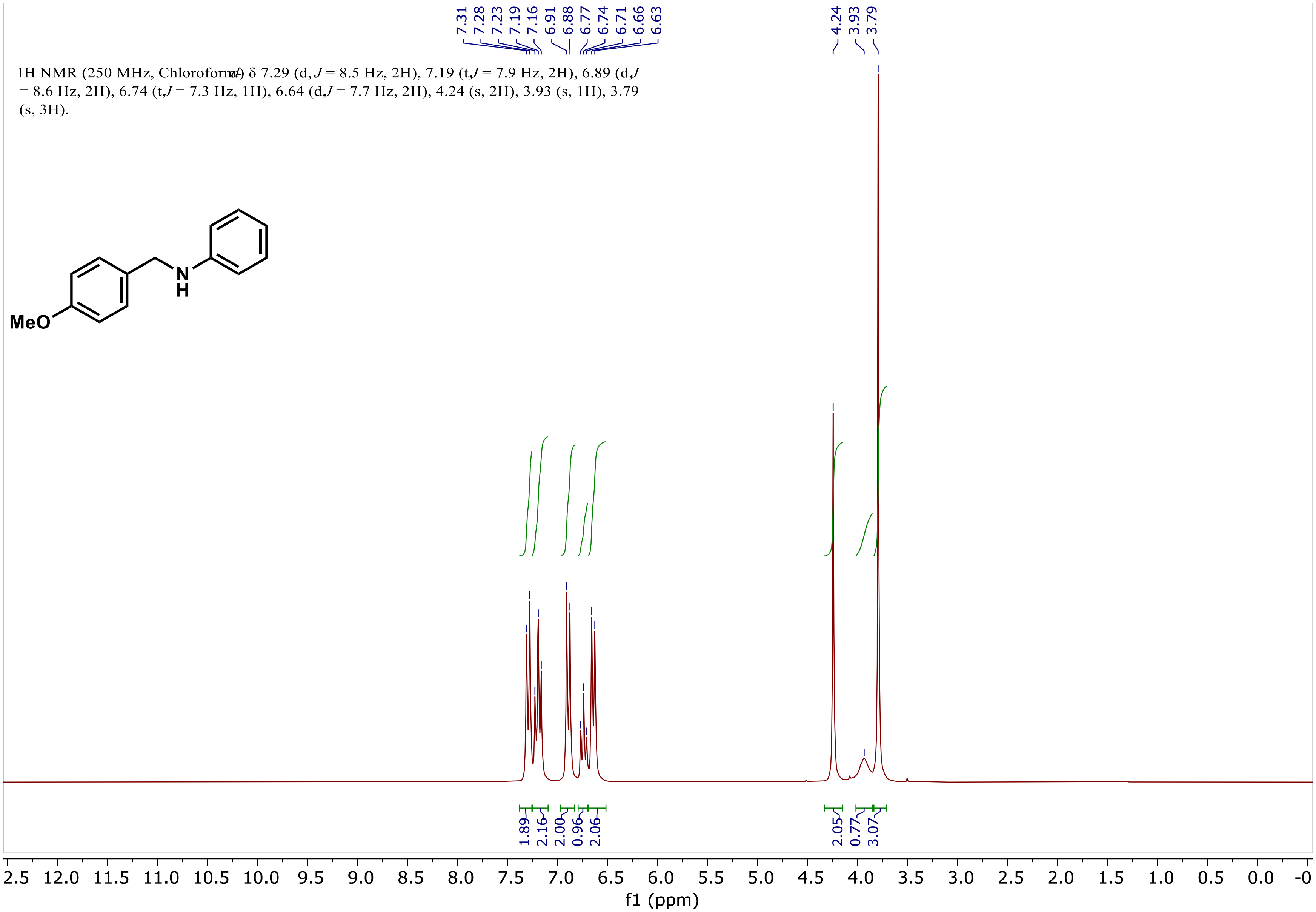

Supplementary Figure 38: *^{13}C NMR for compound **A11***

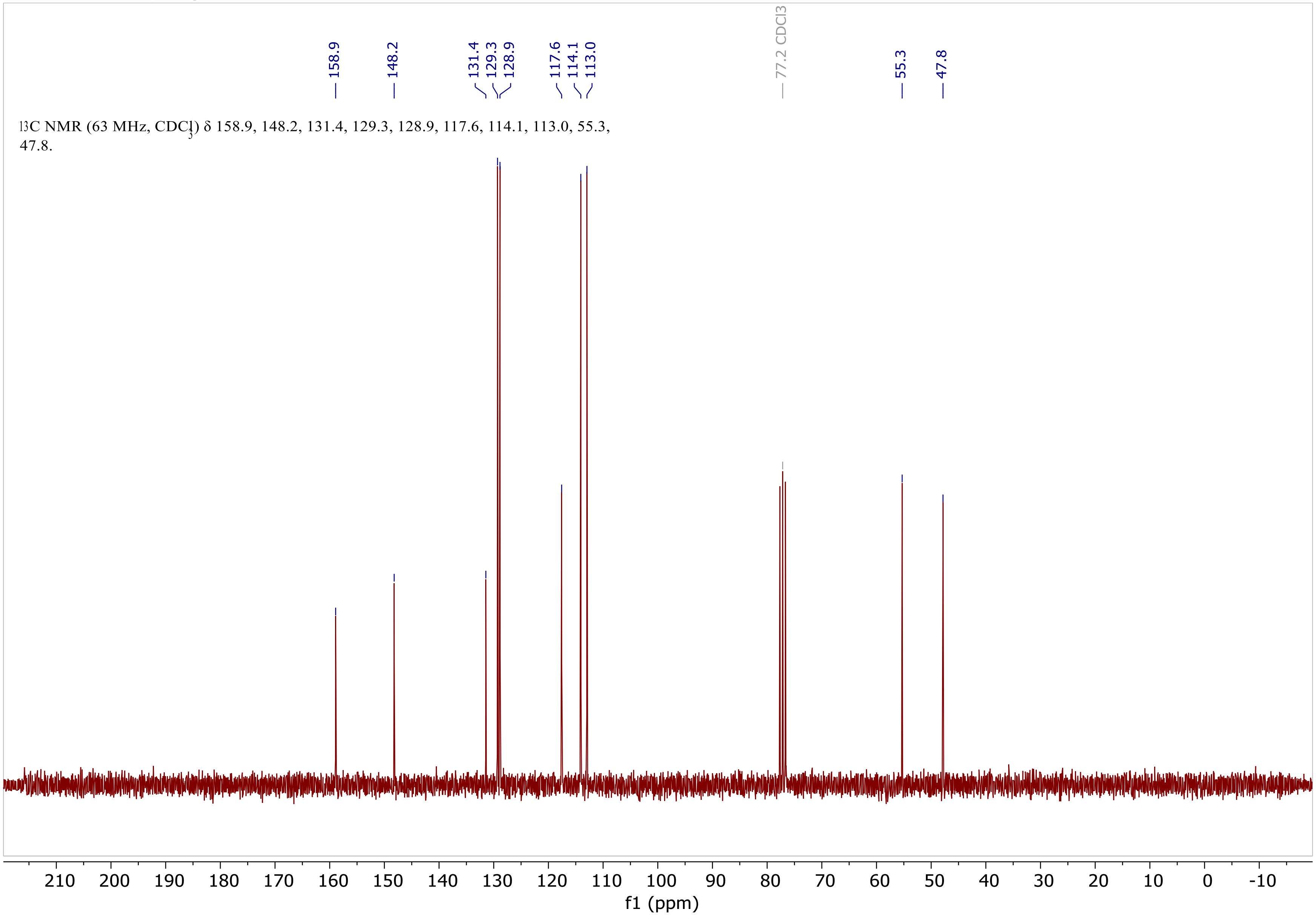

Supplementary Figure 39: *^{1}H-NMR for compound* ***A12***

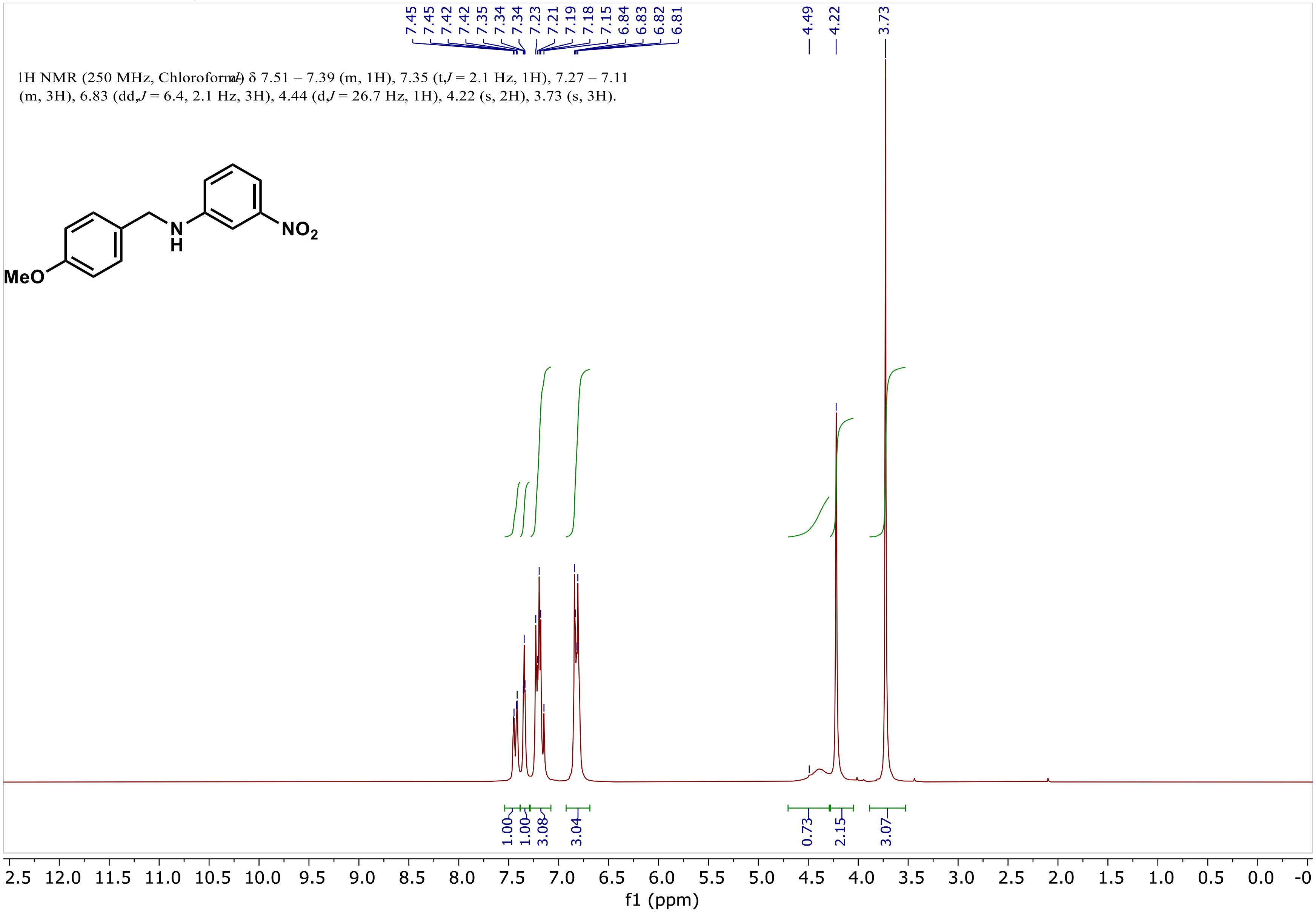

Supplementary Figure 40: *^{13}C NMR for compound* ***A12***

13C NMR (63 MHz, $CDCl_3$) δ 159.2, 149.4, 148.8, 130.0, 129.8, 128.9, 118.9, 114.3, 112.1, 106.6, 55.3, 47.6.

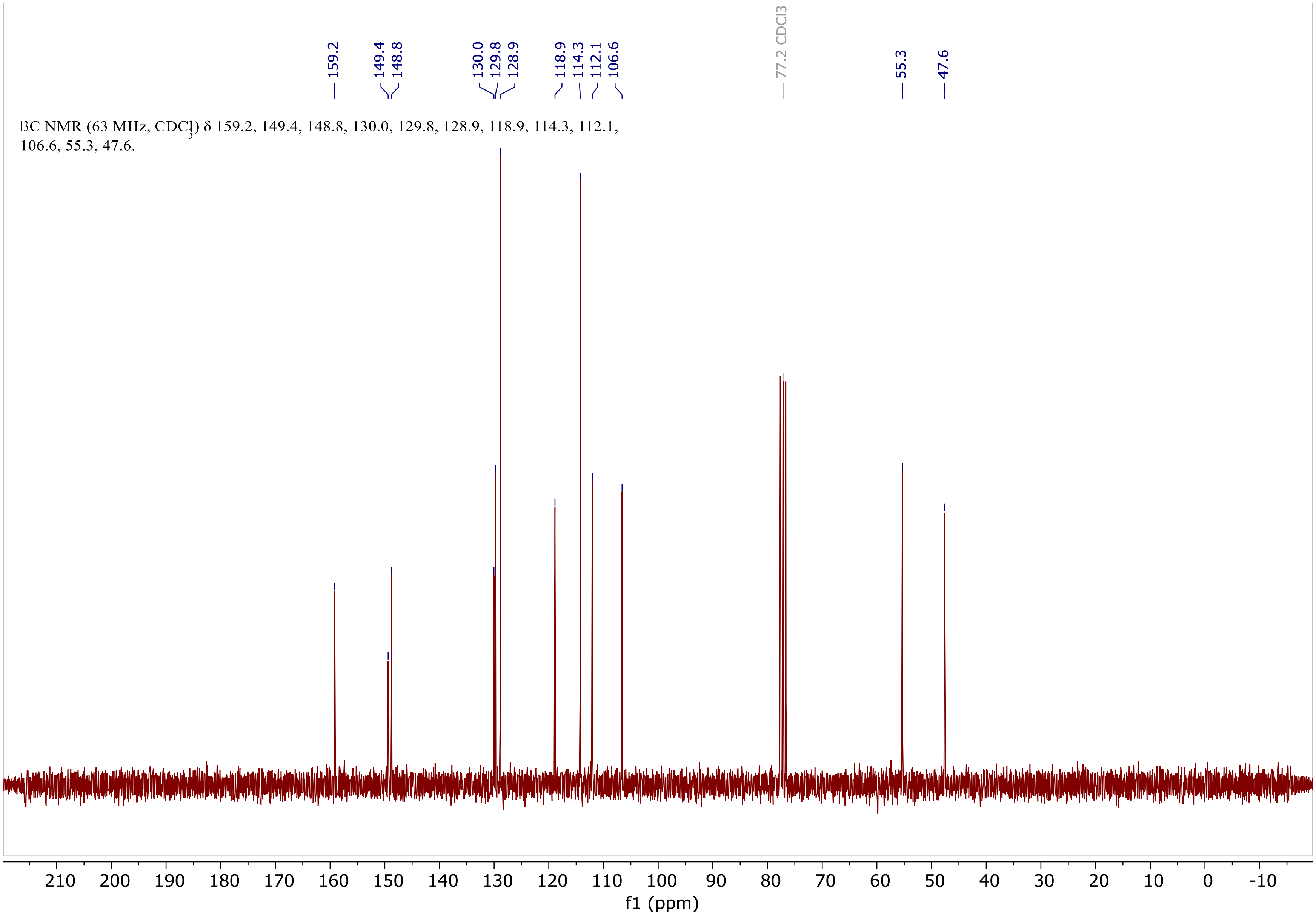

Supplementary Figure 41: *^{1}H-NMR for compound* ***3-phenylprop-2-yn-1-ol***

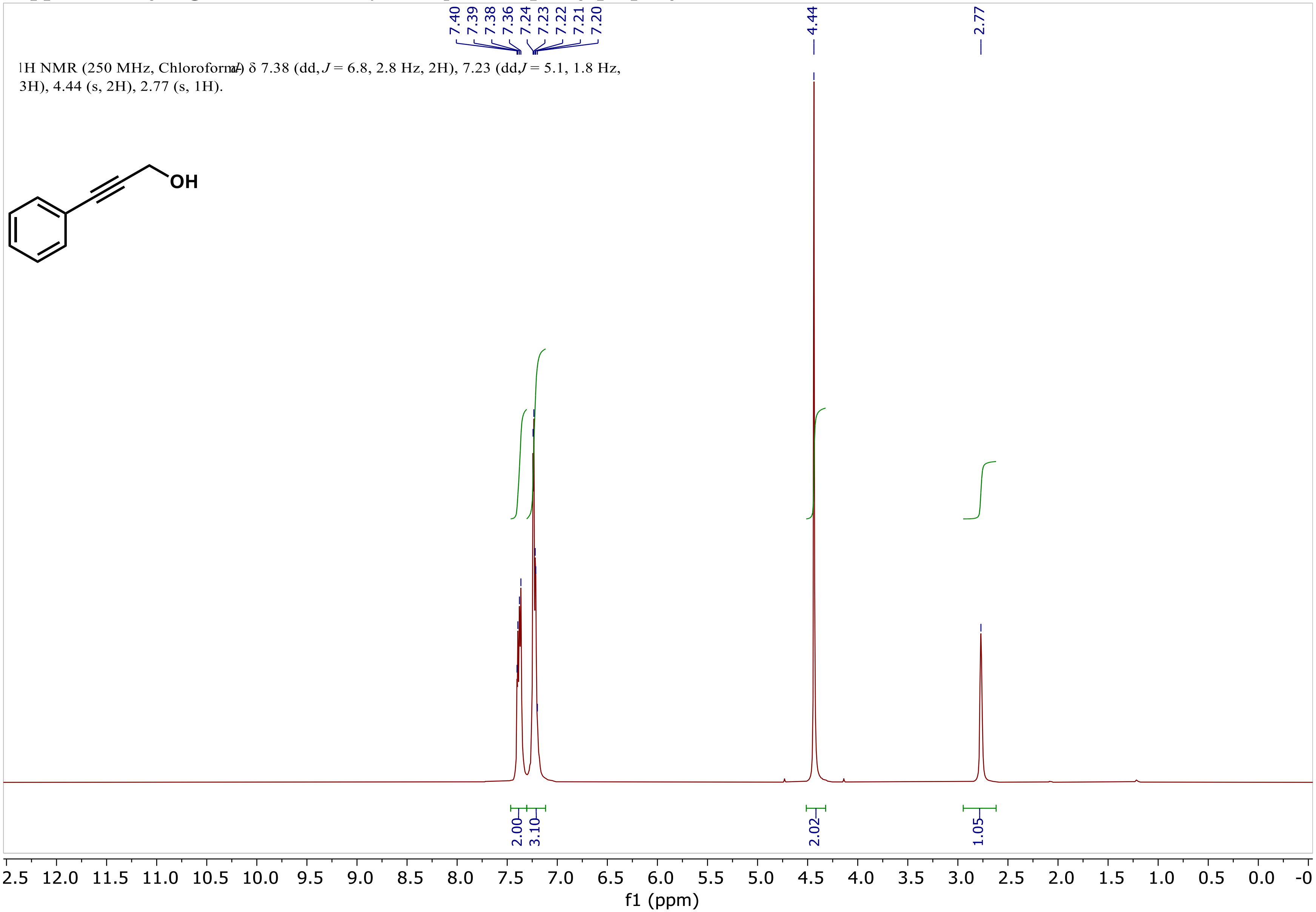

Supplementary Figure 42: ^{13}*C NMR for* ***3-phenylprop-2-yn-1-ol***

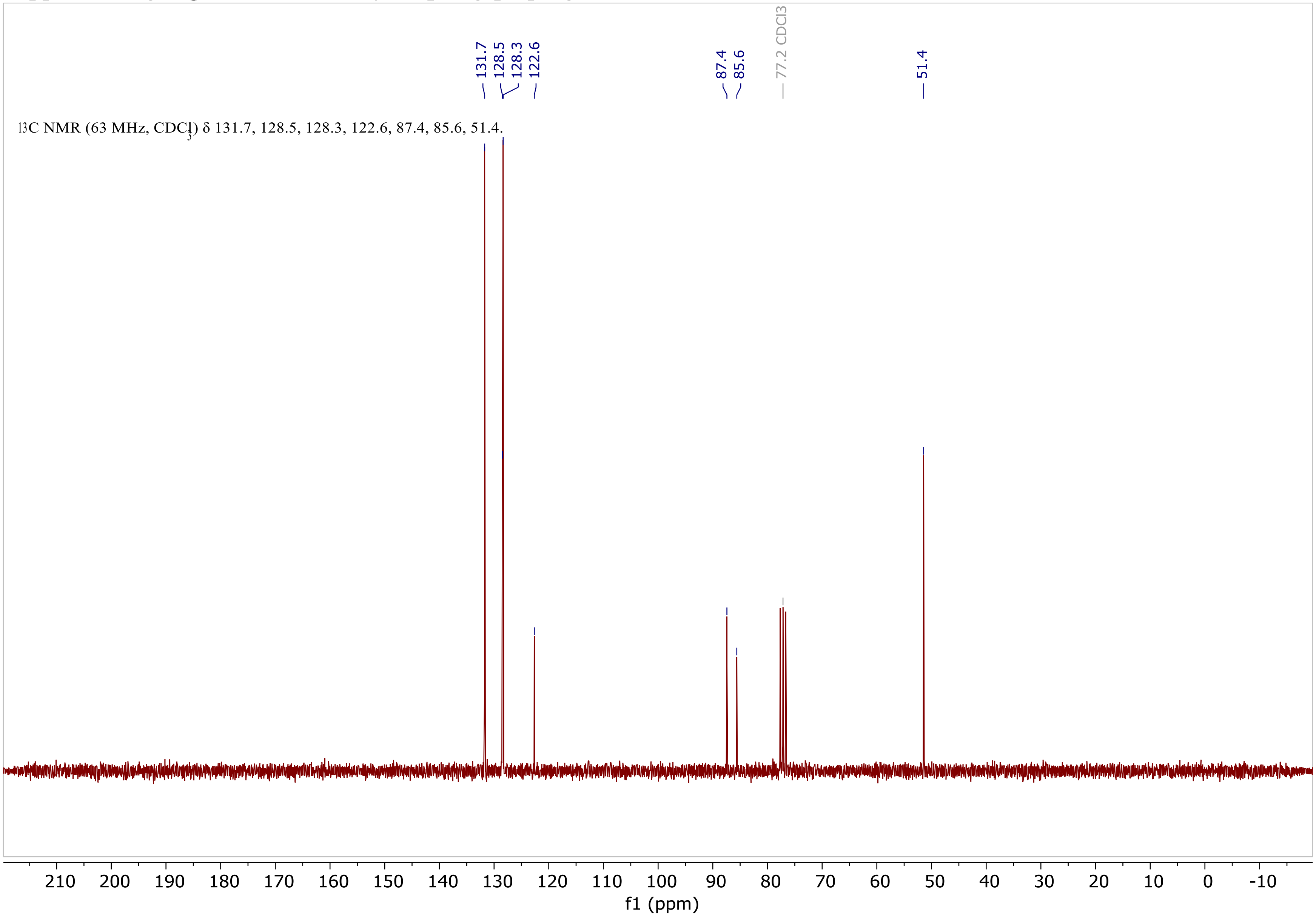

Supplementary Figure 43: ^{1}*H-NMR for compound* ***3-phenylprop-2-yn-1-yl 4-methylbenzenesulfonate***

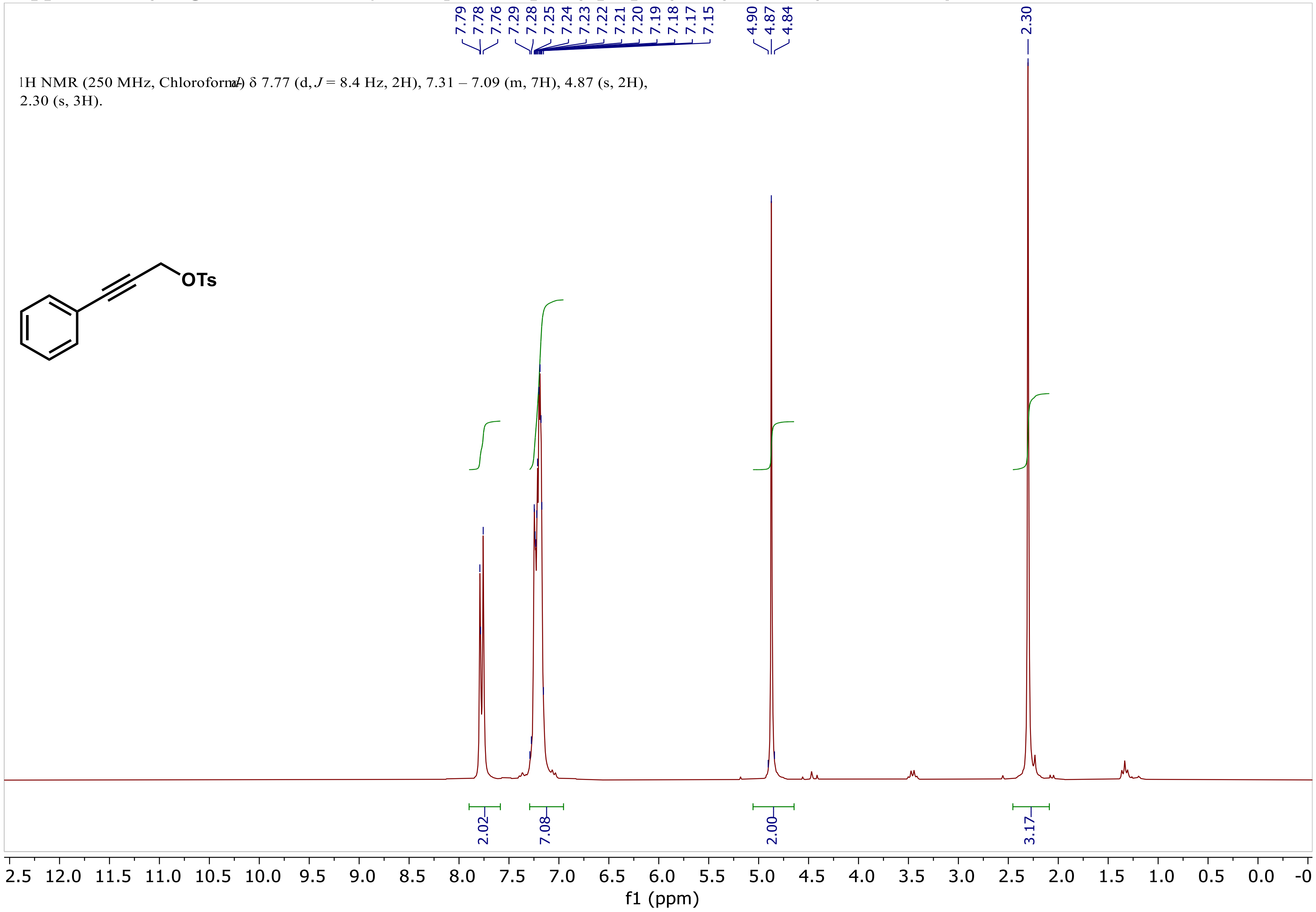

Supplementary Figure 44: *^{13}C NMR for* ***3-phenylprop-2-yn-1-yl 4-methylbenzenesulfonate***

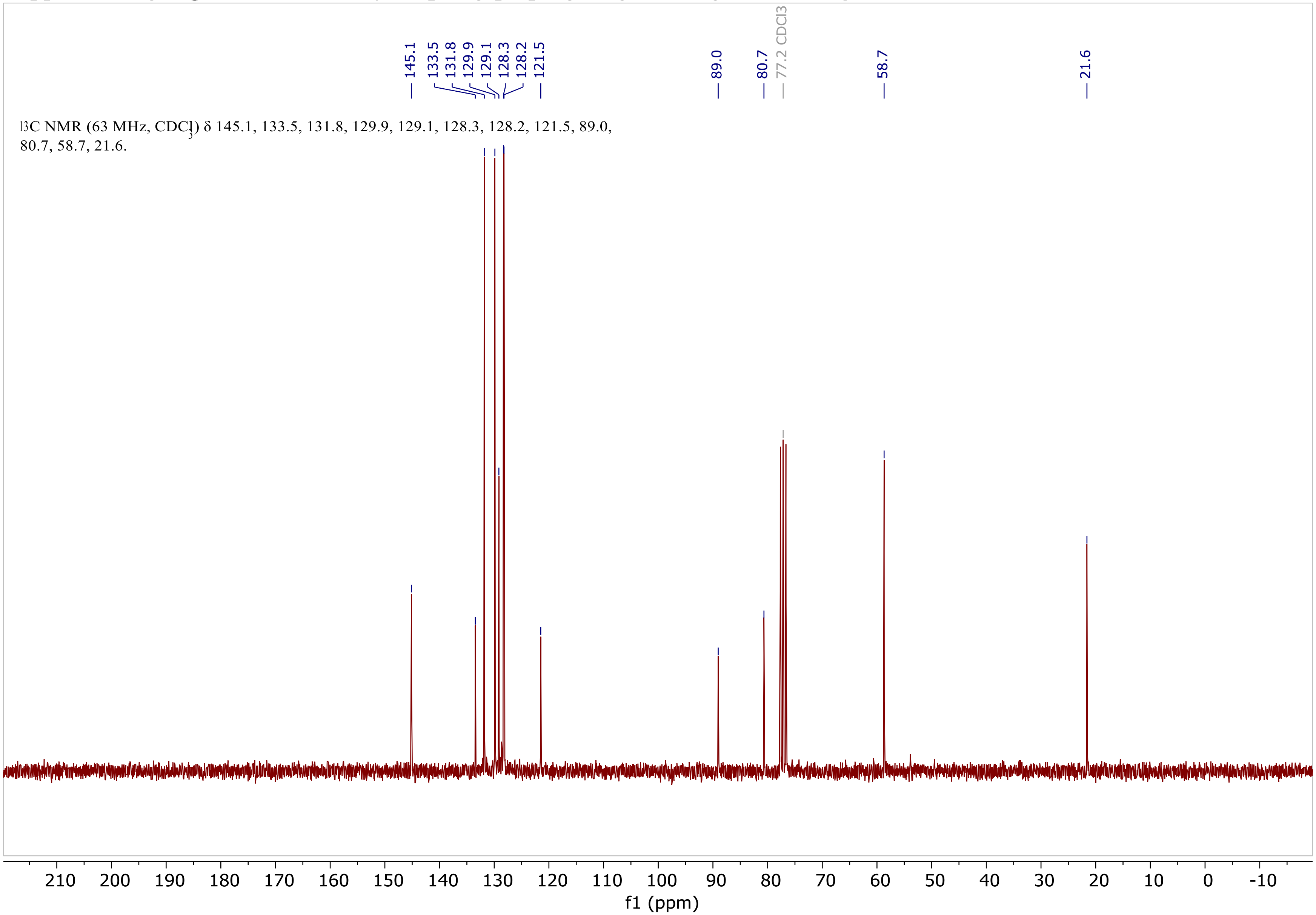

Supplementary Figure 45: *^{1}H-NMR for compound* ***A13***

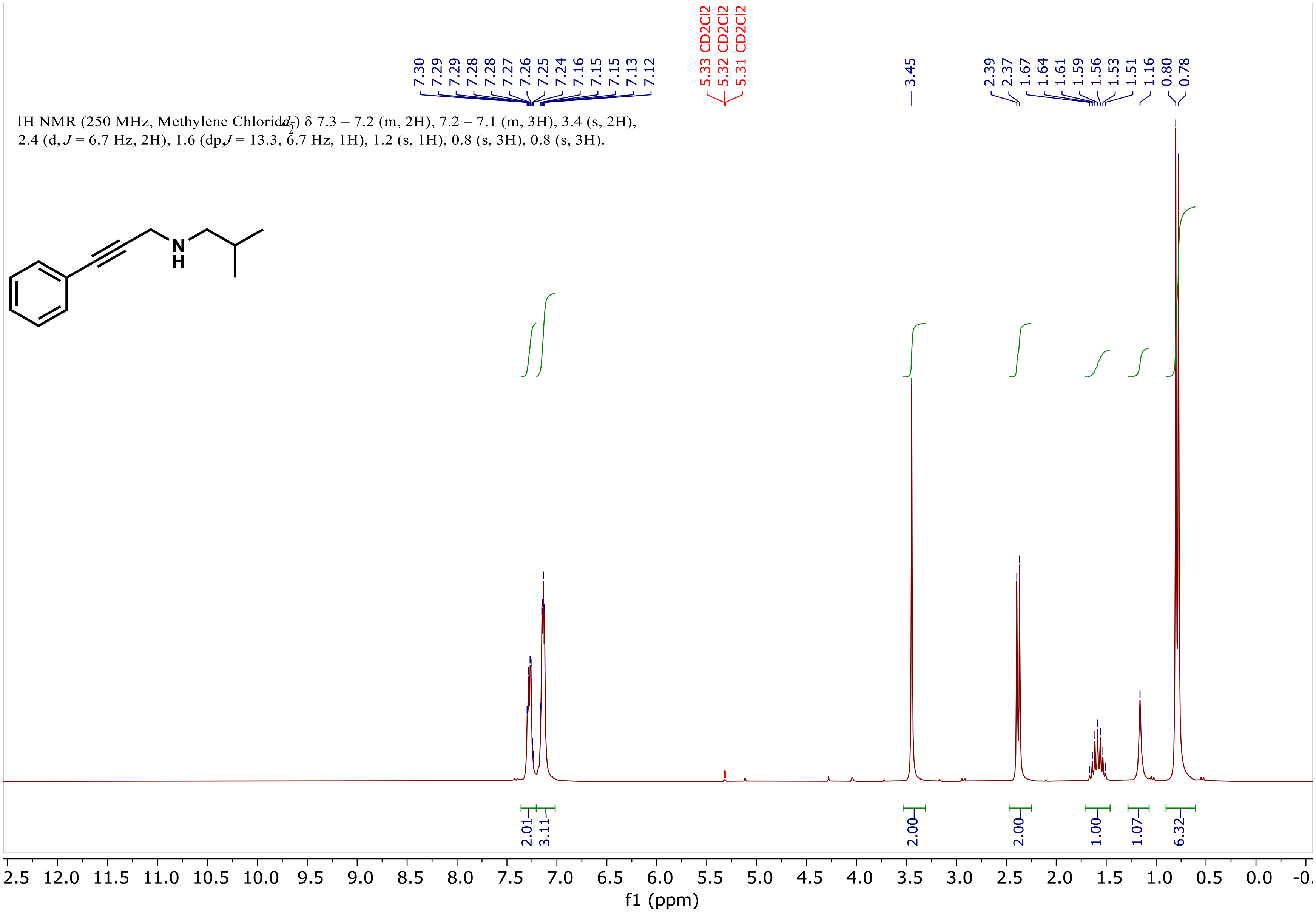

Supplementary Figure 46: *^{13}C NMR for compound* ***A13***

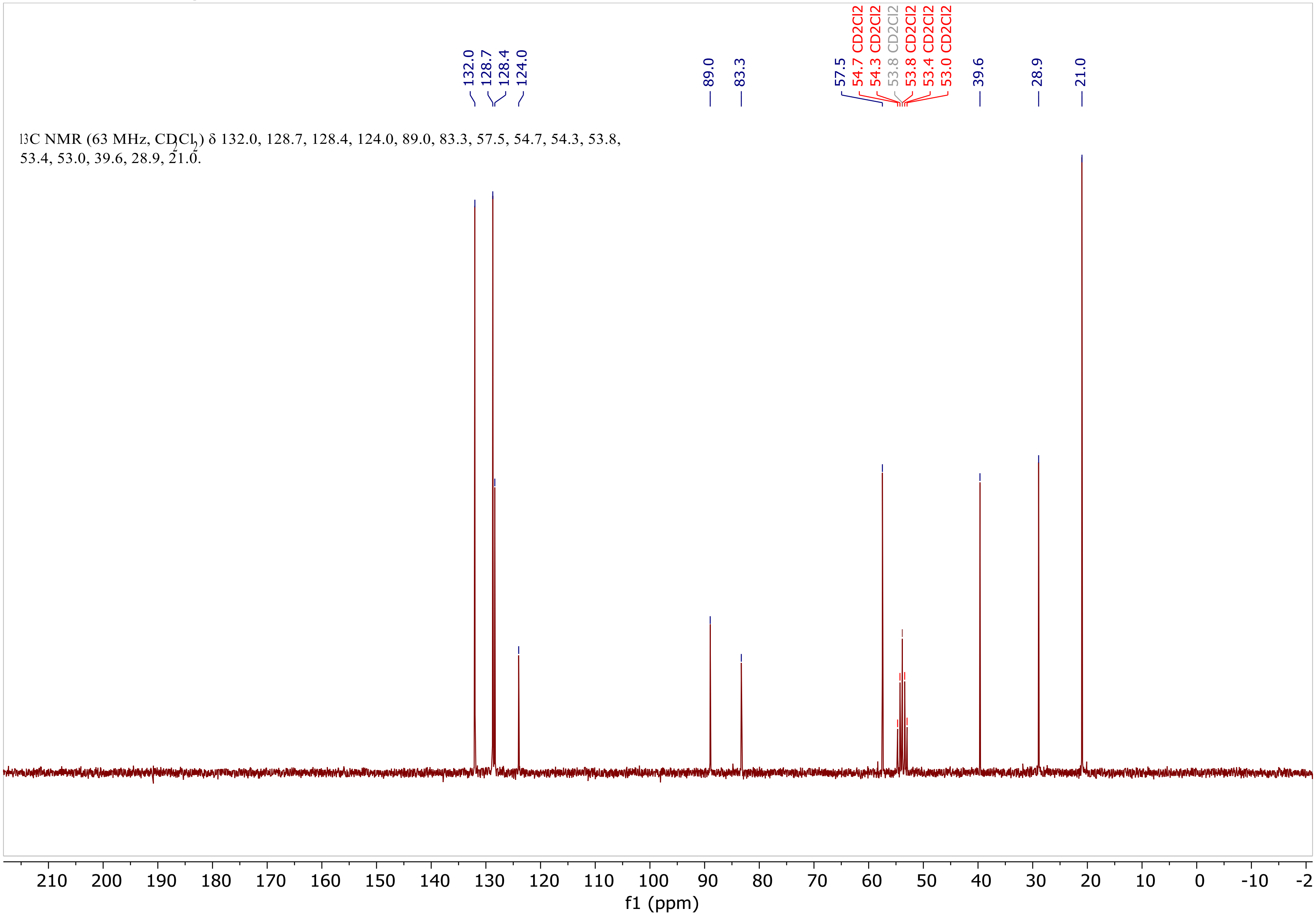

Supplementary Figure 47: *^{1}H-NMR for compound **A14***

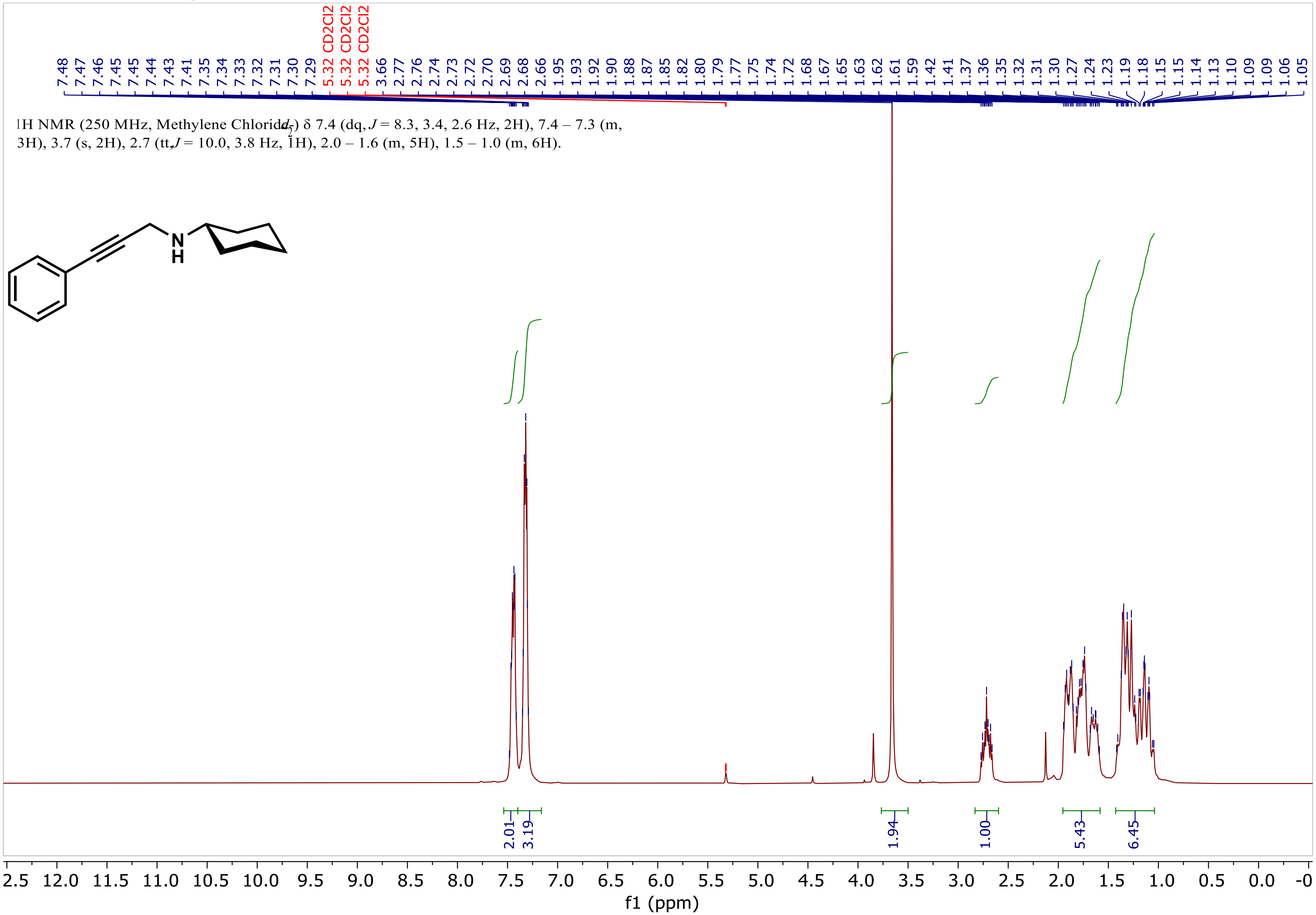

Supplementary Figure 48: *^{13}C NMR for compound* ***A14***

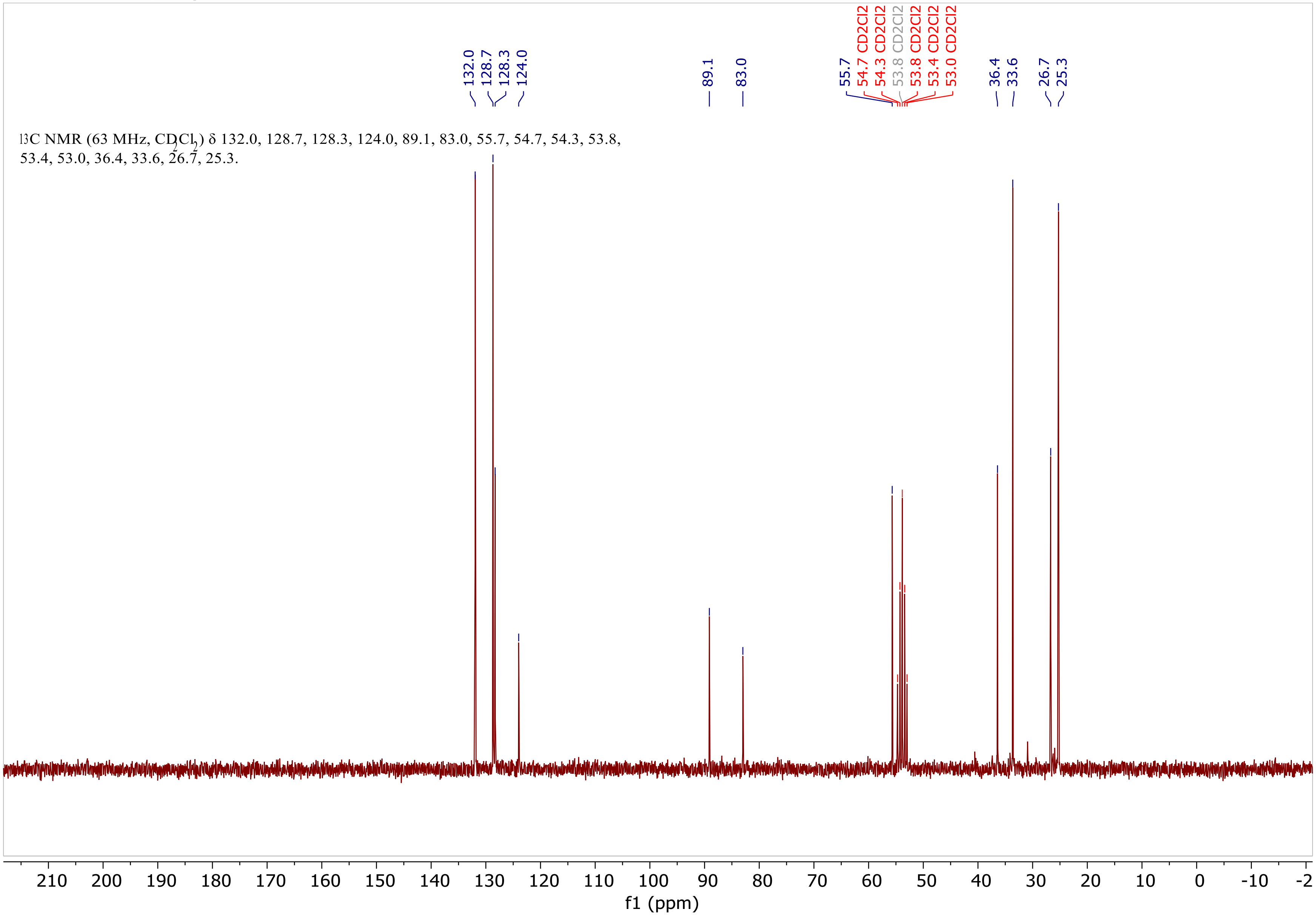

Supplementary Figure 49: *^{1}H-NMR for compound* ***A15***

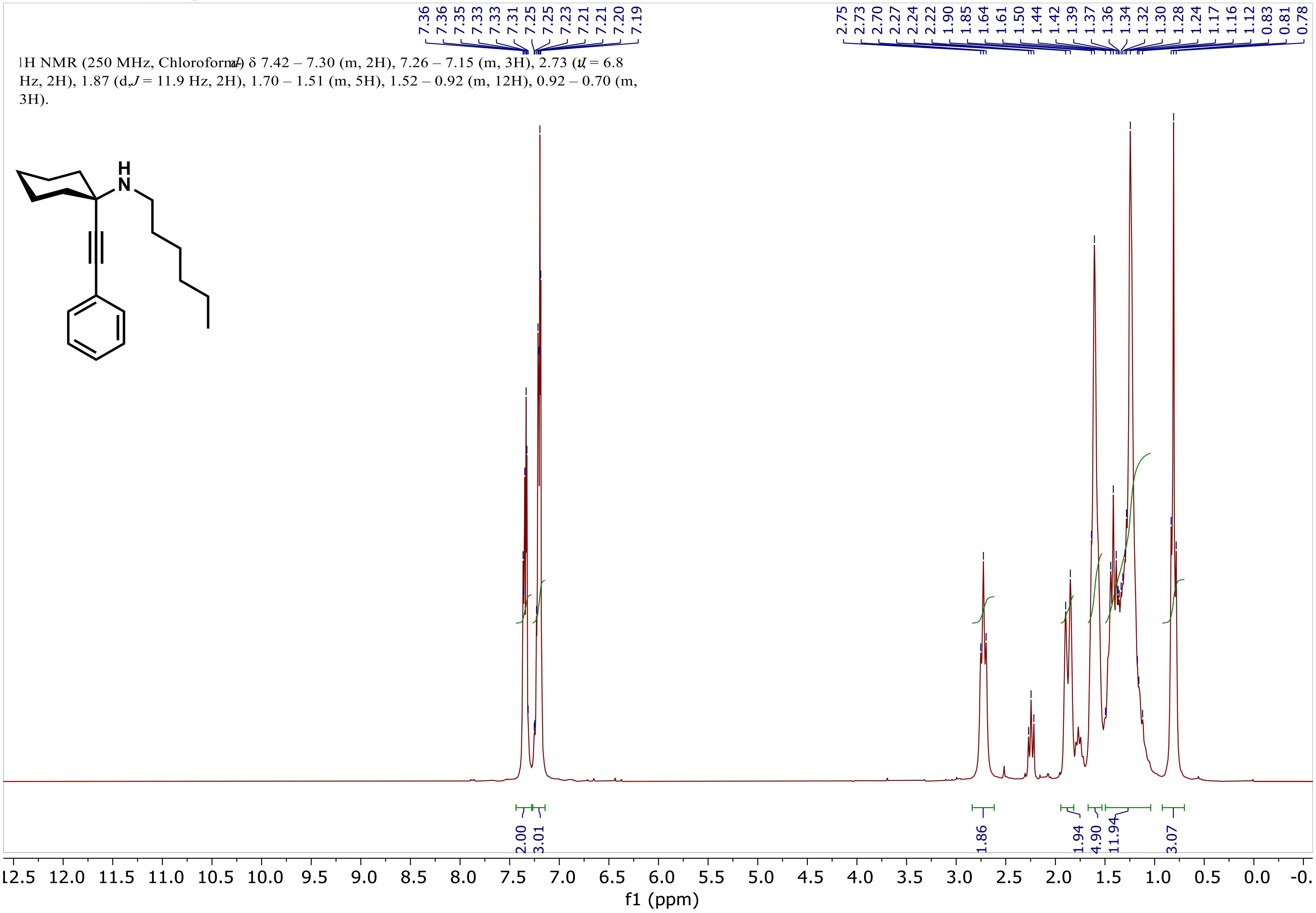

Supplementary Figure 50: ^{13}*C NMR for compound* ***A15***

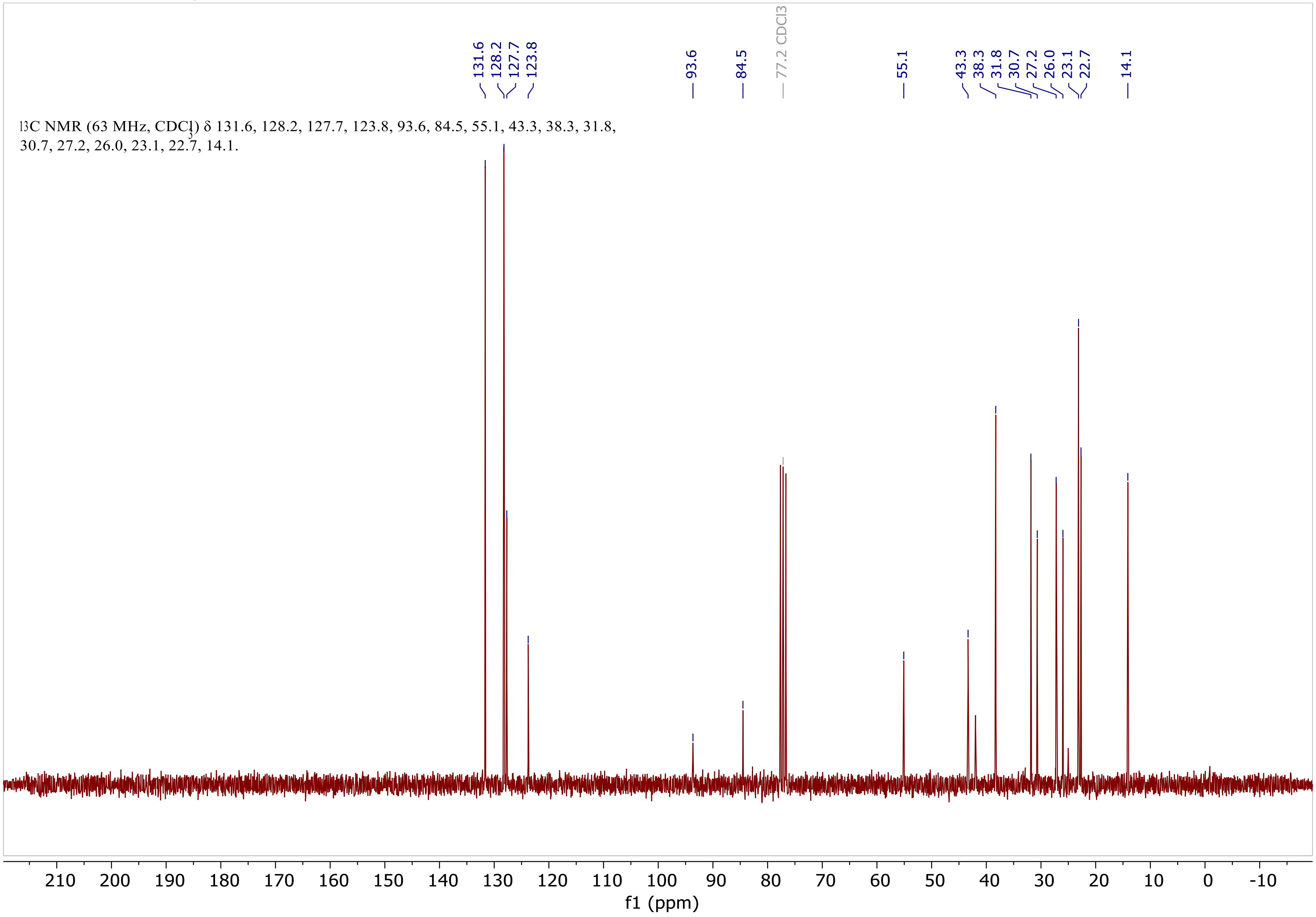

Supplementary Figure 51: *^{1}H-NMR for compound* ***A16***

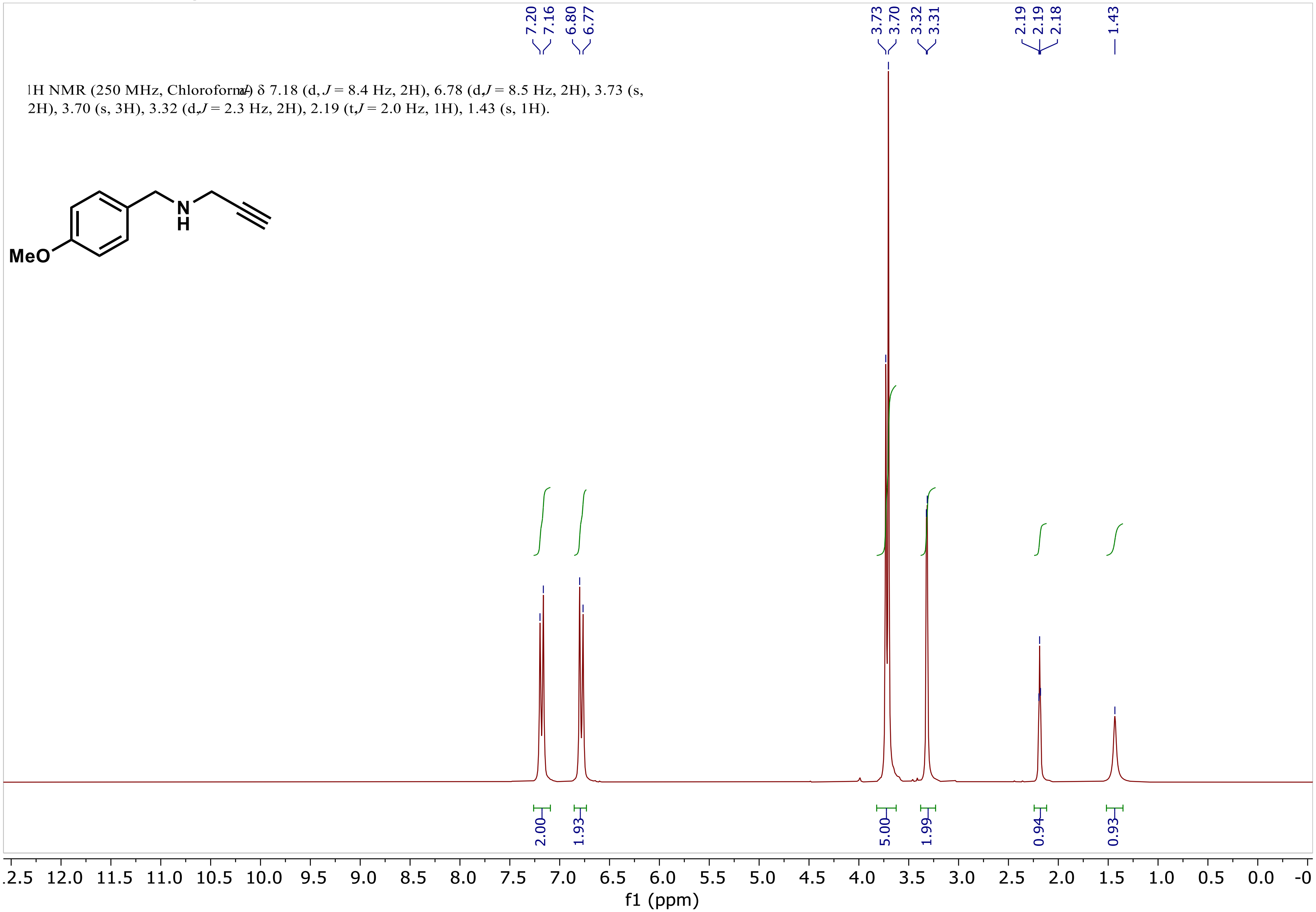

Supplementary Figure 52: ^{13}*C NMR for compound* ***A16***

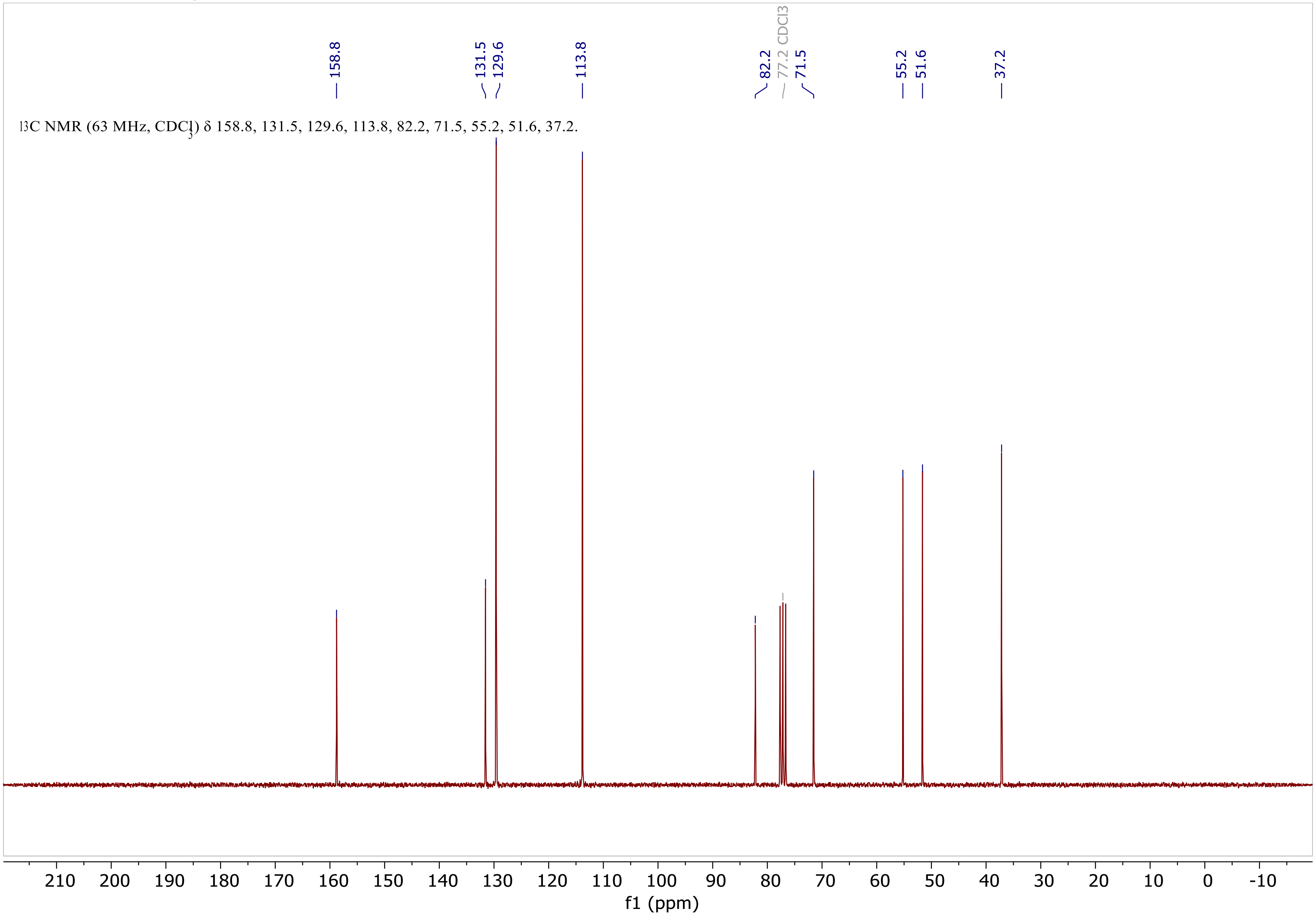

Supplementary Figure 53: *¹H-NMR for compound* ***A17***

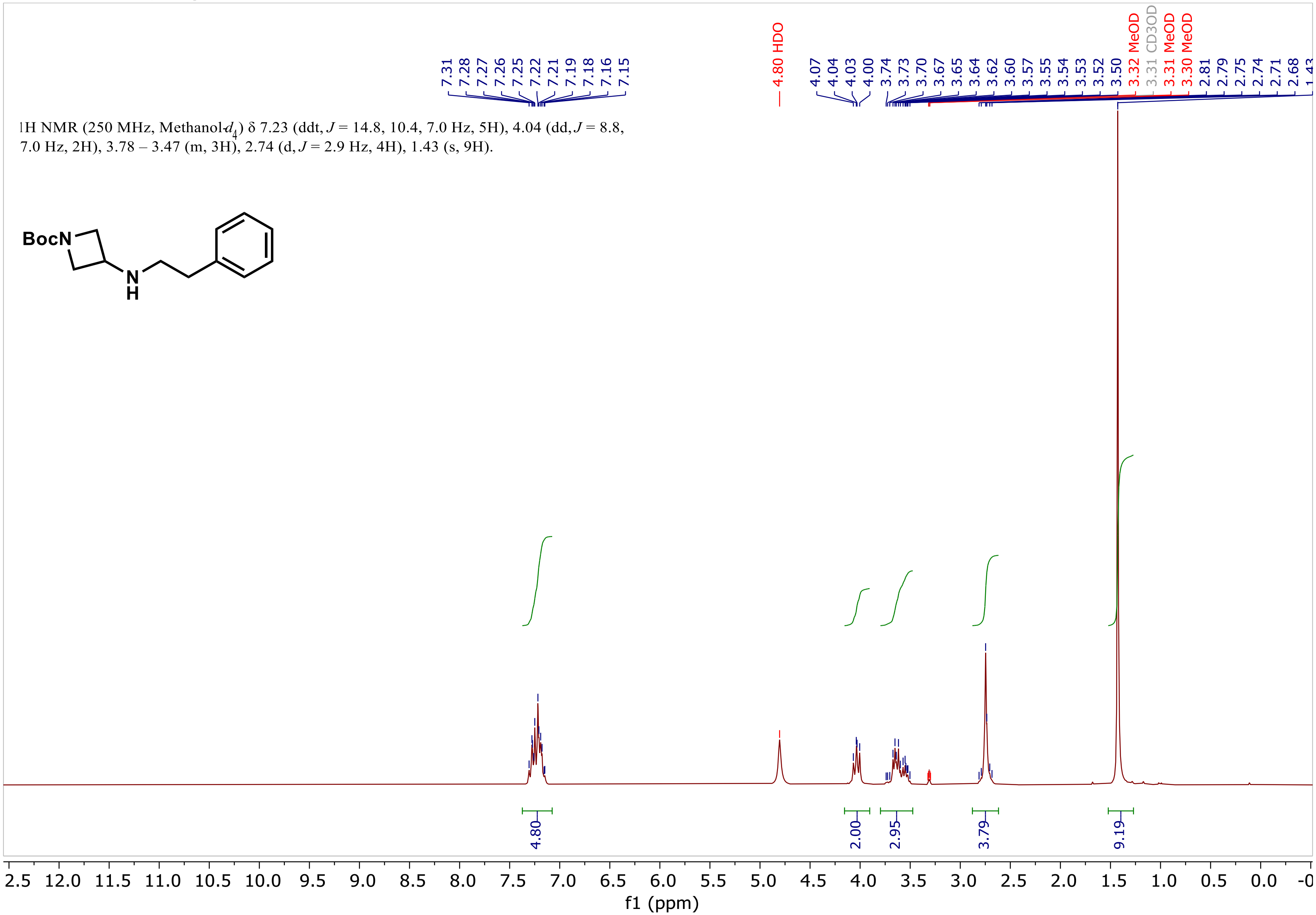

Supplementary Figure 54: ^{13}*C NMR for compound* ***A17***

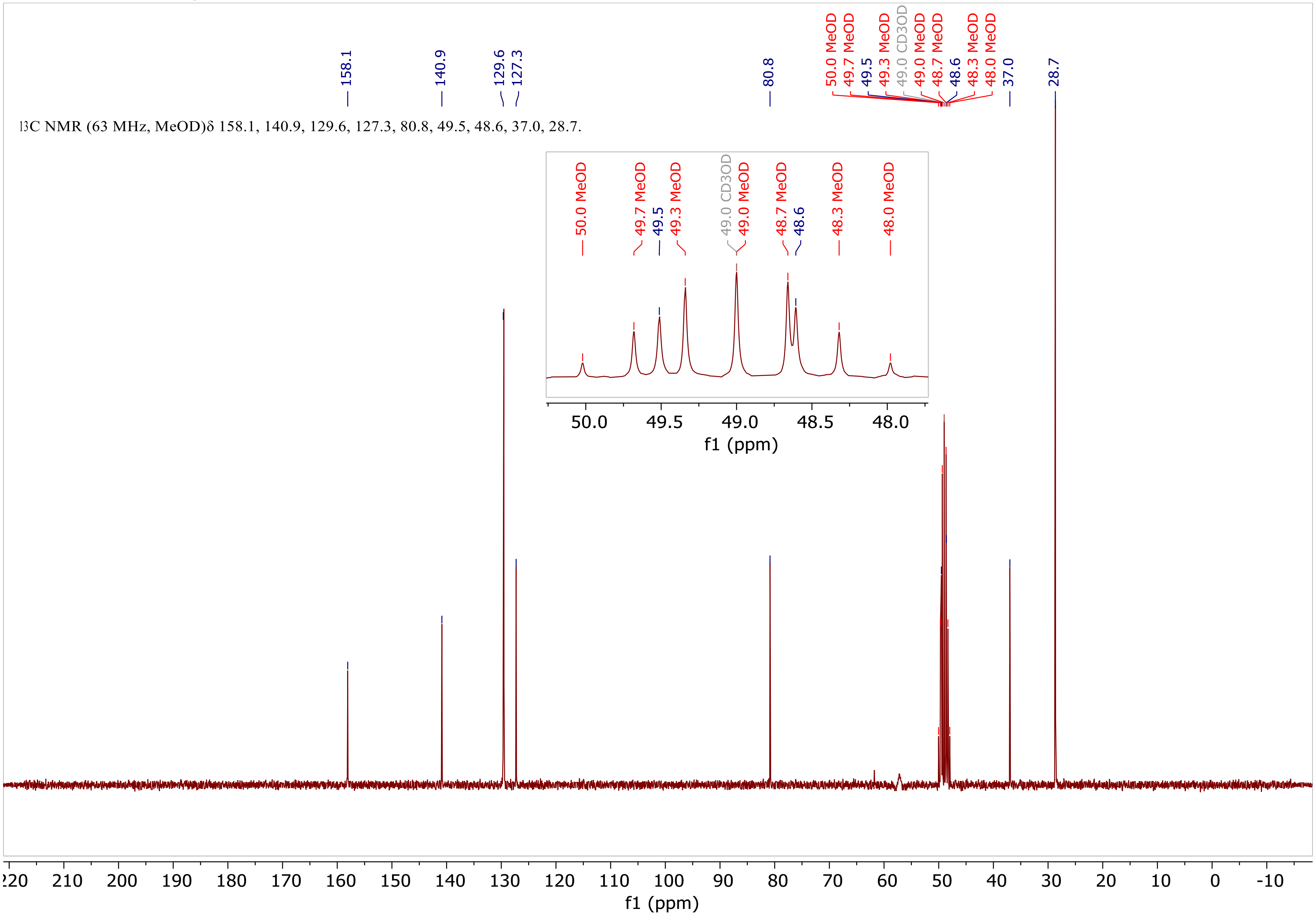

Supplementary Figure 55: *IR for compound* ***A17***

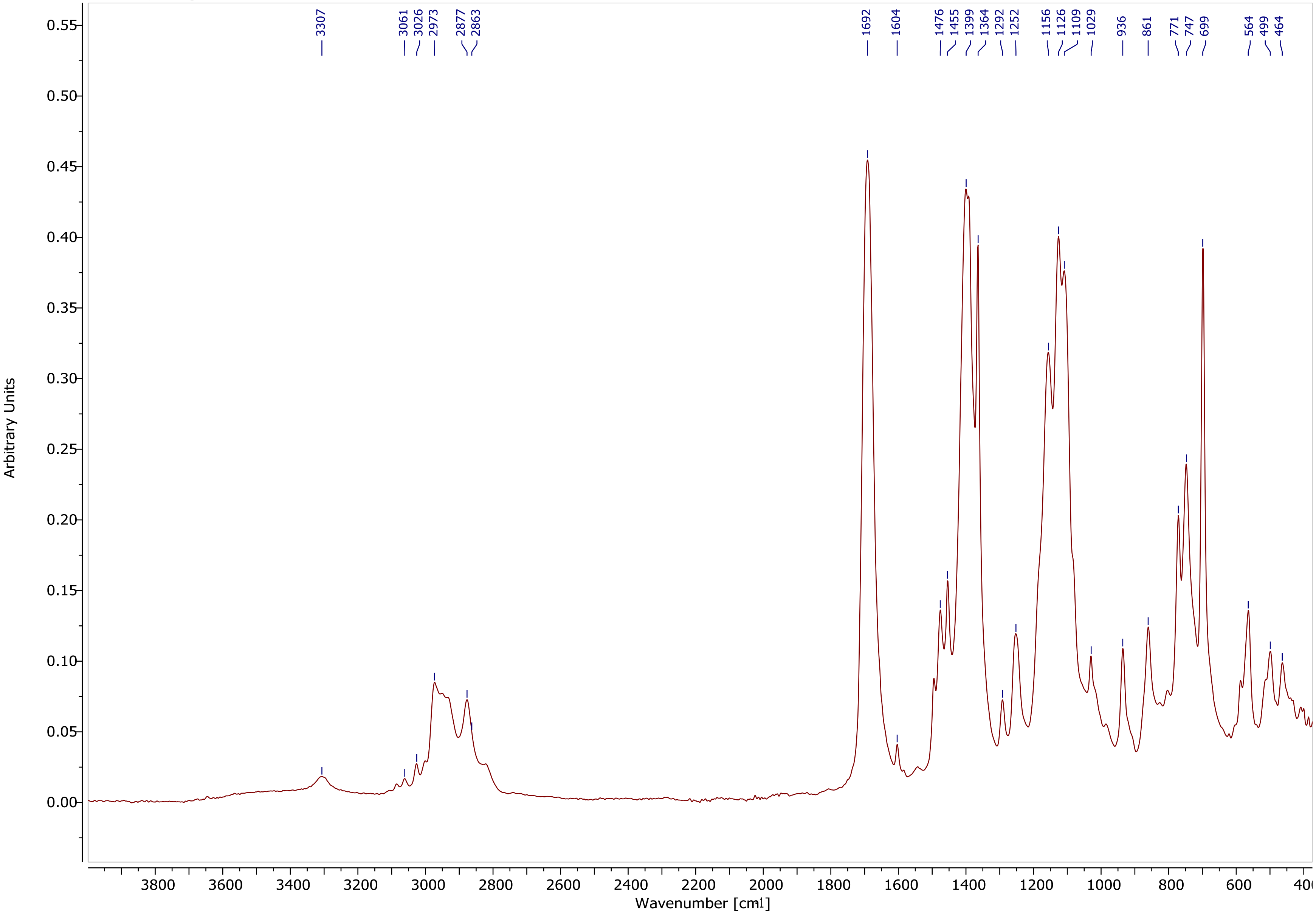

Supplementary Figure 56: *^{1}H-NMR for compound* ***A18***

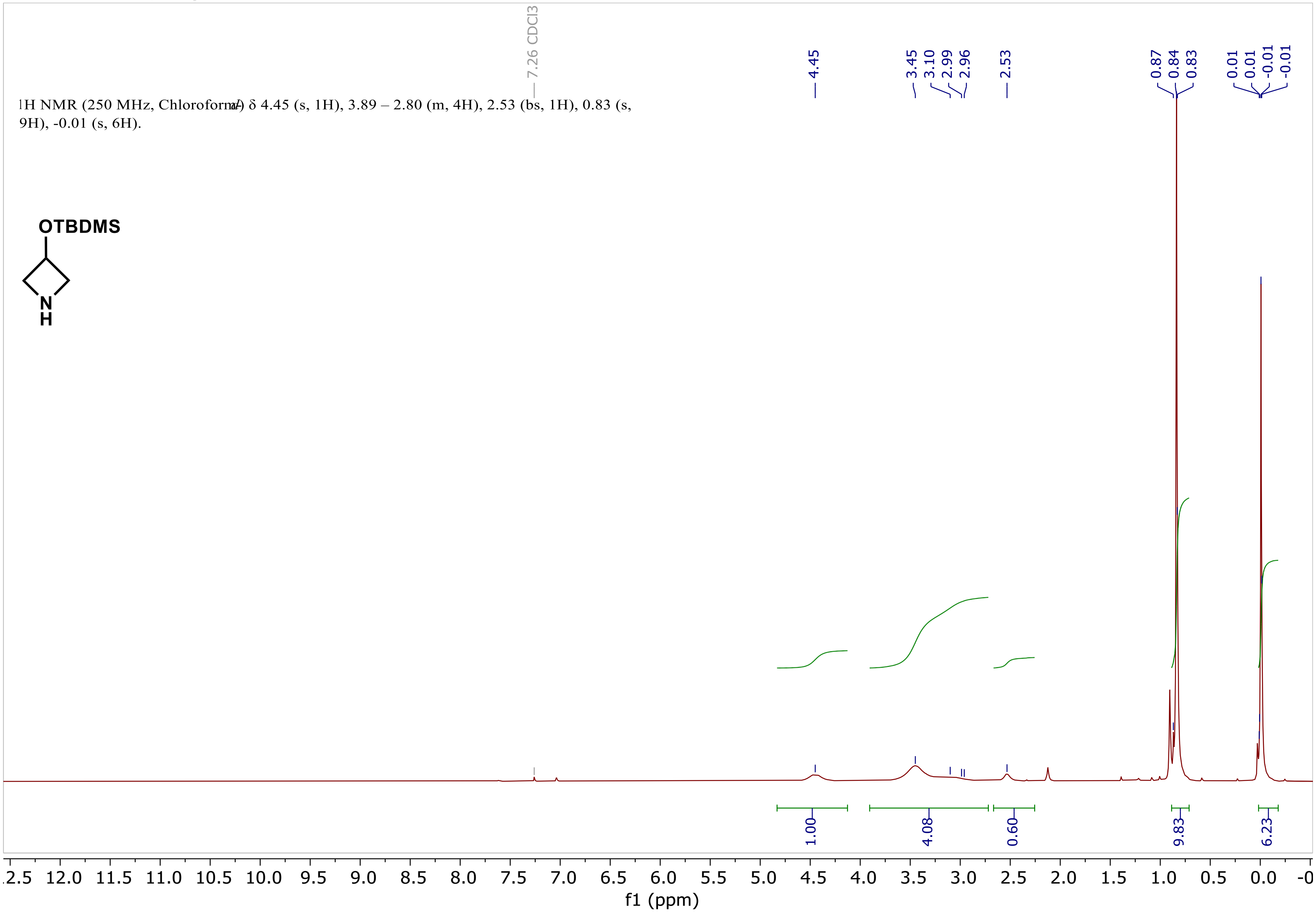

Supplementary Figure 57: *^{13}C NMR for compound* ***A18***

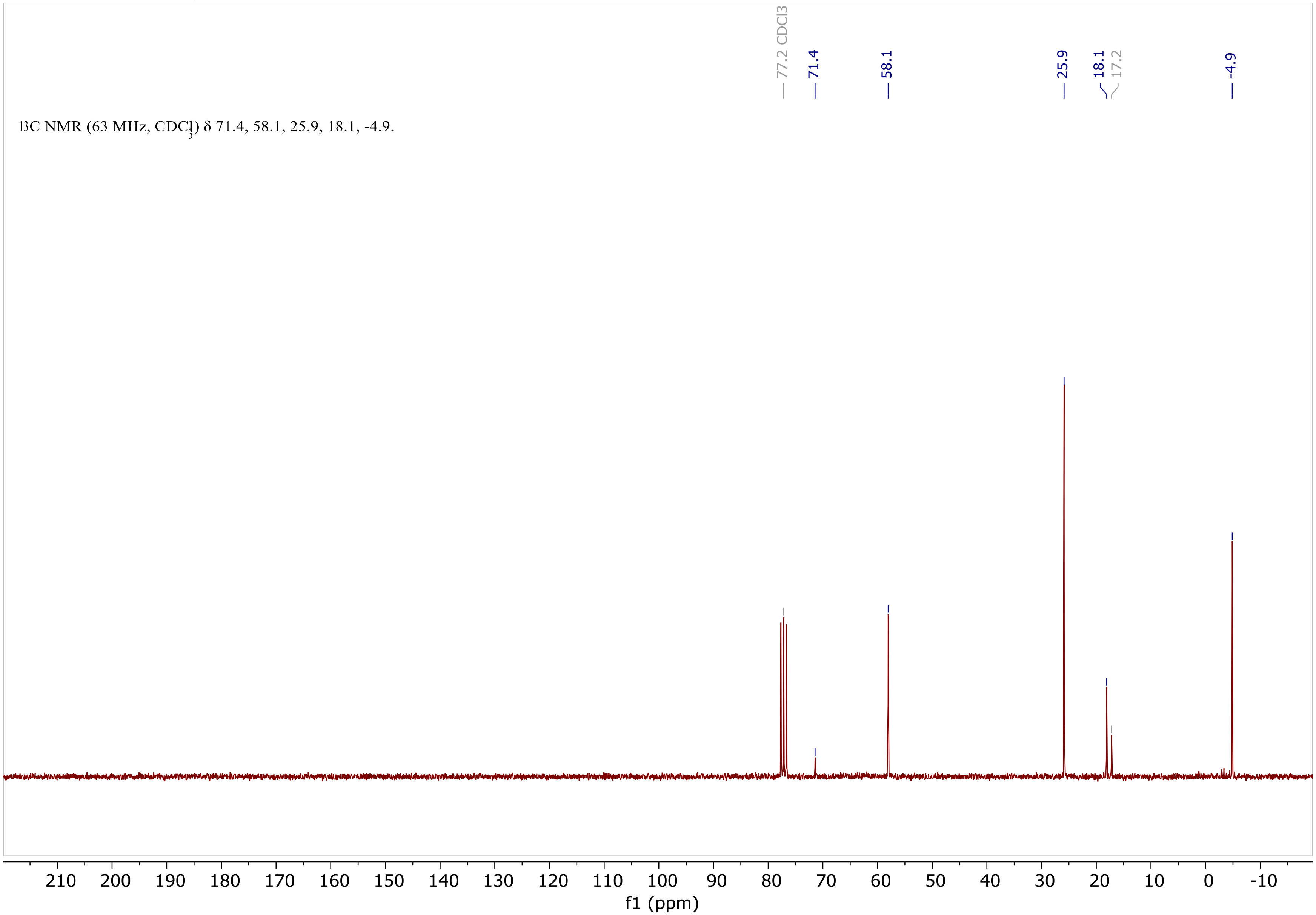

Supplementary Figure 58: *^{1}H-NMR for compound* ***A19***

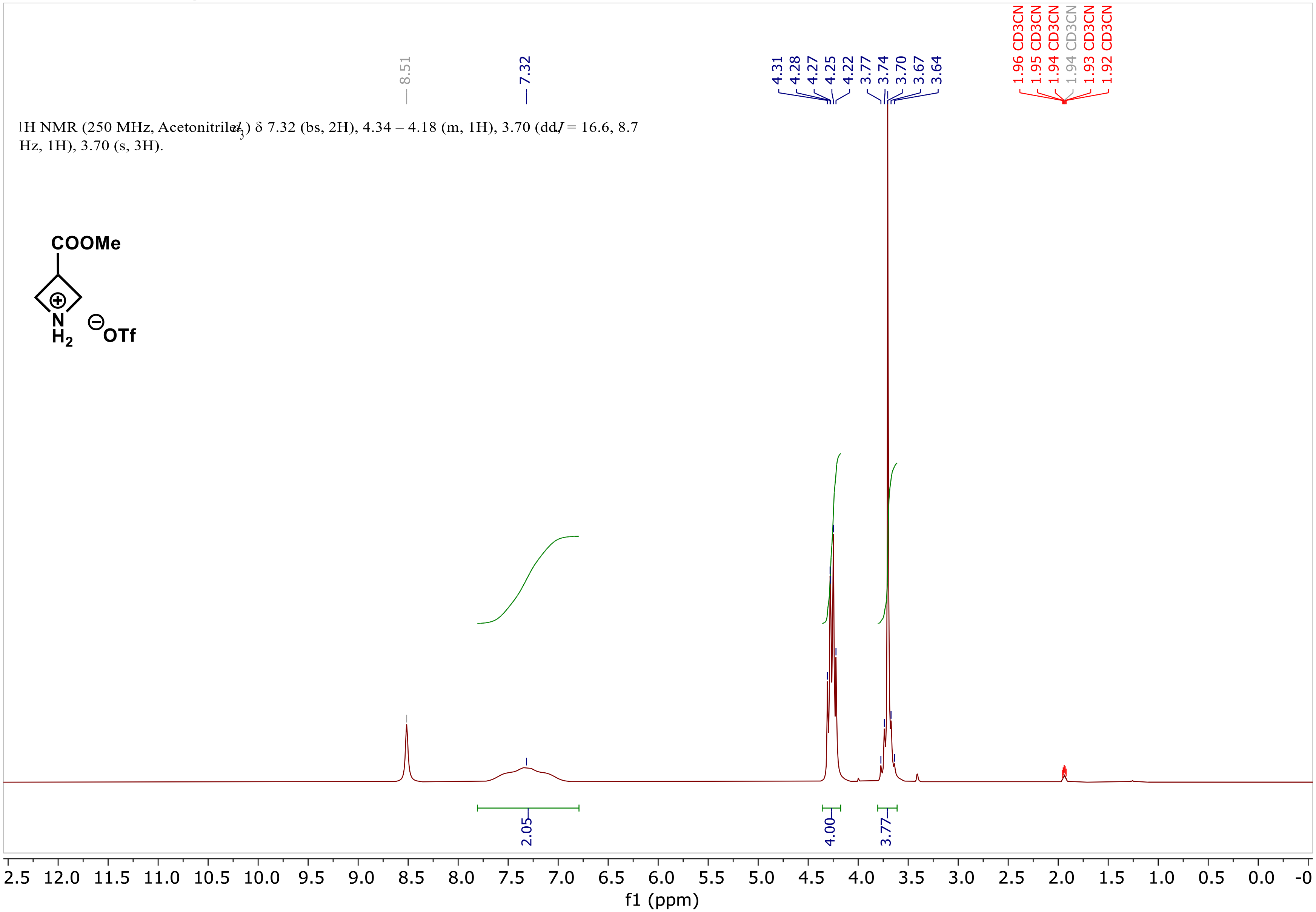

Supplementary Figure 59: *^{19}F NMR for compound **A19***

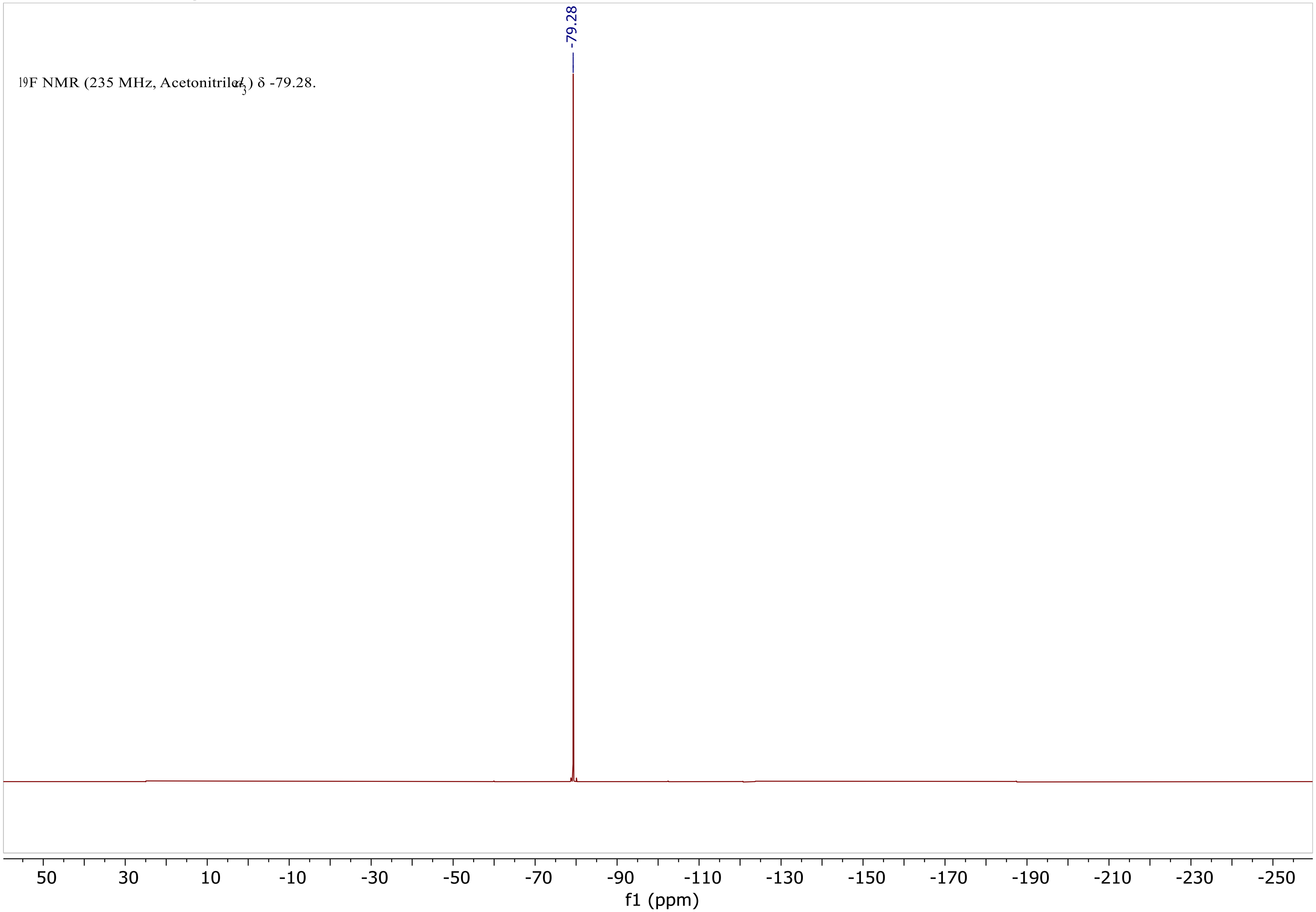

Supplementary Figure 60: *^{13}C NMR for compound* ***A19***

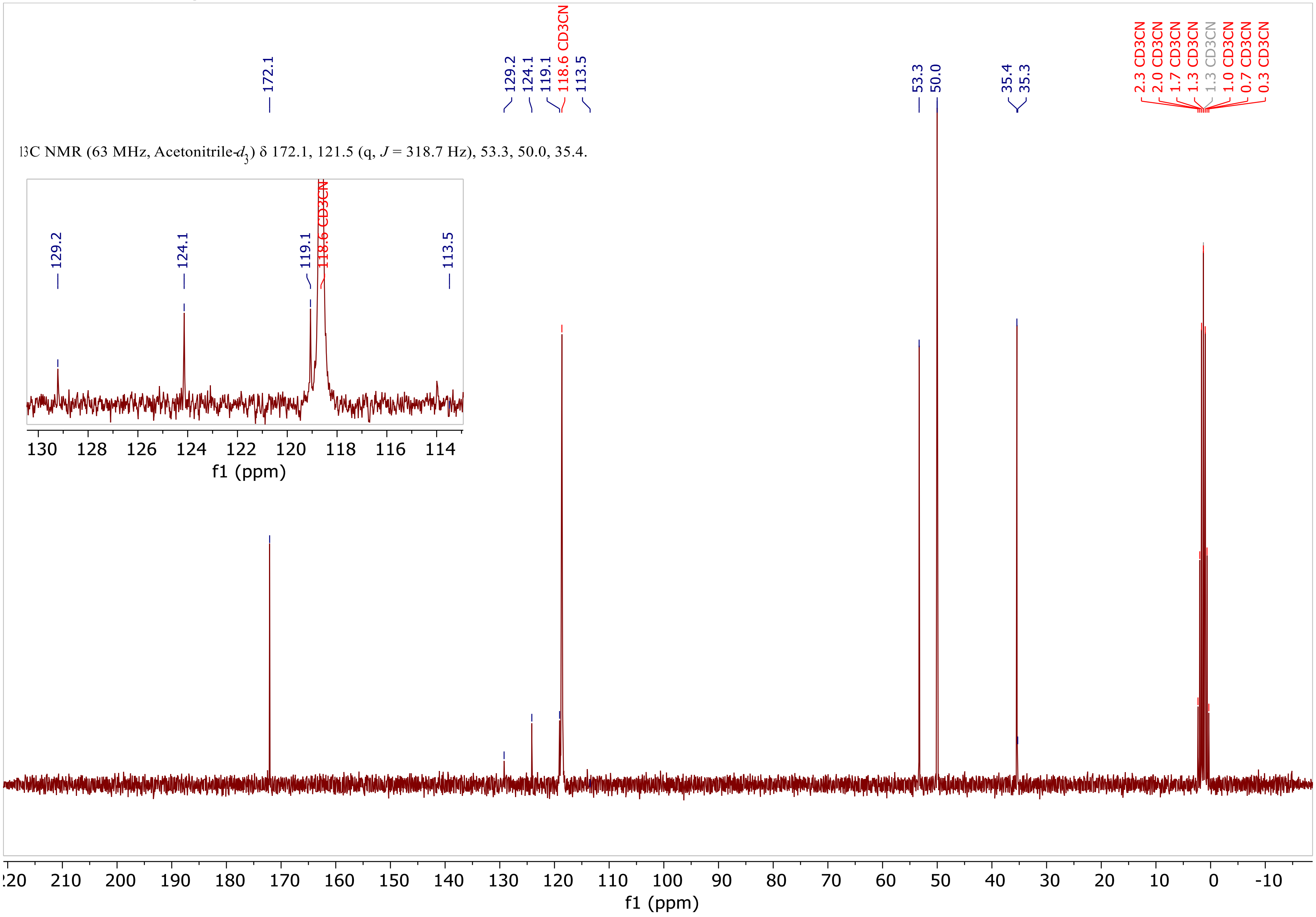

Supplementary Figure 61: *^{1}H-NMR for compound* ***A20***

1H NMR (250 MHz, Chloroform-*d*) δ 3.52 (dt, *J* = 7.3, 3.7 Hz, 1H), 2.83 (d, *J* = 12.2 Hz, 1H), 2.75 – 2.62 (m, 1H), 2.60 – 2.34 (m, 2H), 2.20 (bs, 1H), 1.81 – 1.54 (m, 2H), 1.33 (dt, *J* = 12.0, 7.5 Hz, 2H), 0.88 – 0.68 (m, 9H), 0.01 – -0.13 (m, 6H).

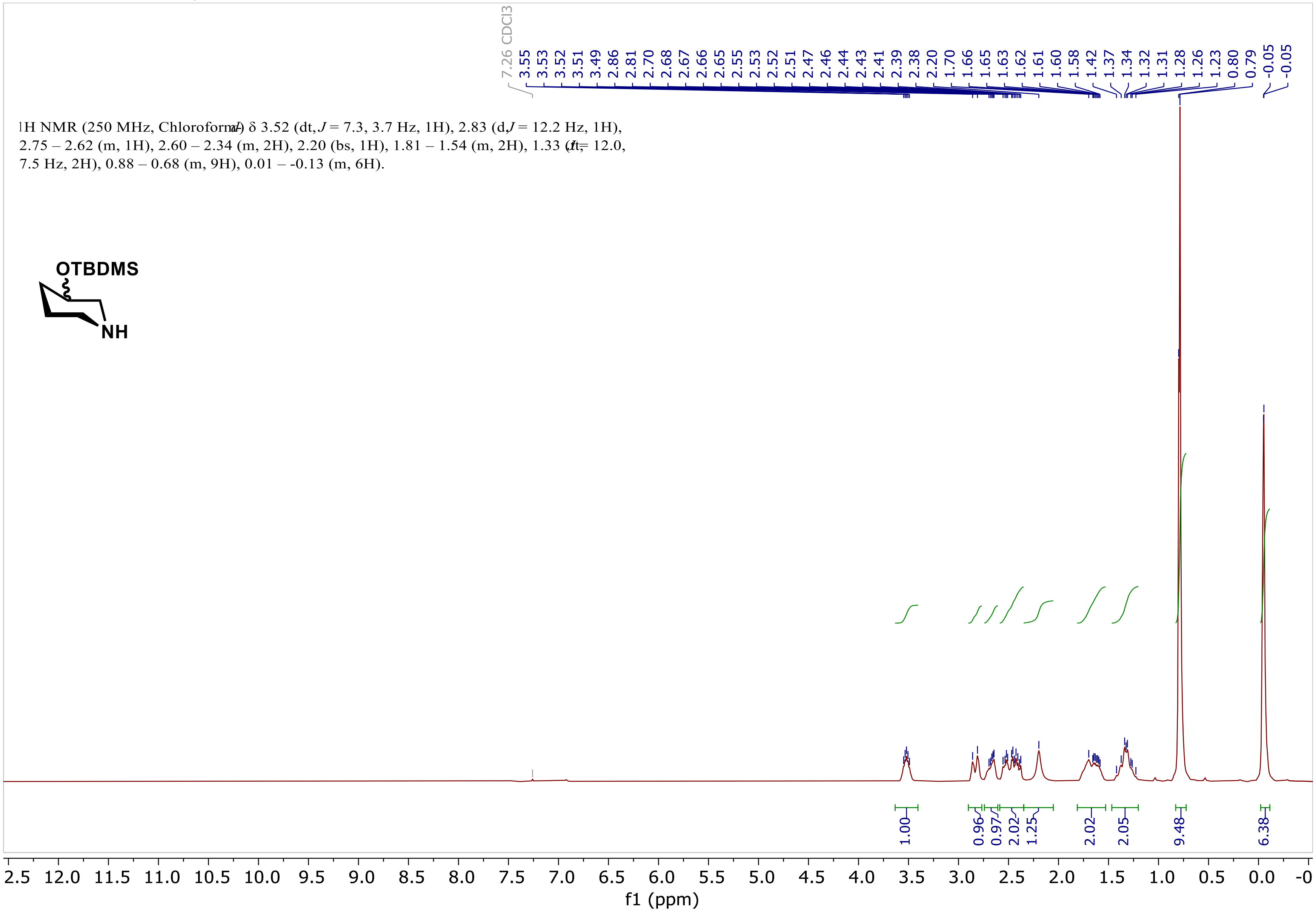

Supplementary Figure 62: *^{13}C NMR for compound* ***A20***

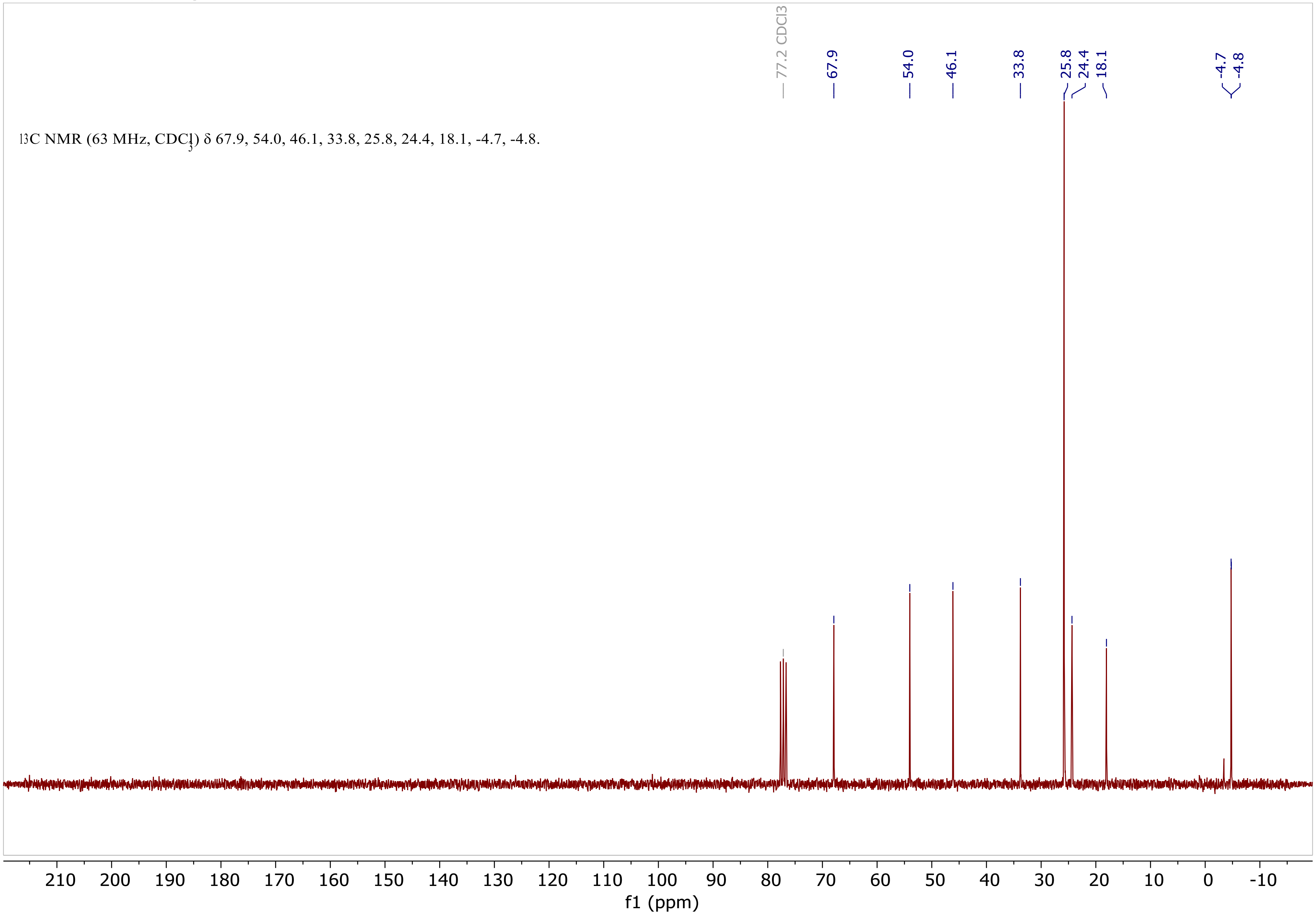

Supplementary Figure 63: *¹H-NMR for compound* ***A21***

1H NMR (250 MHz, Chloroform-*d*) δ 3.47 – 3.21 (m, 2H), 3.06 (d, *J* = 11.5 Hz, 1H), 2.93 (d, *J* = 12.1 Hz, 1H), 2.46 (td, *J* = 11.8, 2.7 Hz, 1H), 2.34 – 2.13 (m, 1H), 1.91 (s, 1H), 1.76 – 1.48 (m, 3H), 1.48 – 1.24 (m, 1H), 1.01 (qd, *J* = 11.9, 3.9 Hz, 1H), 0.82 (s, 9H), -0.03 (d, *J* = 3.7 Hz, 6H).

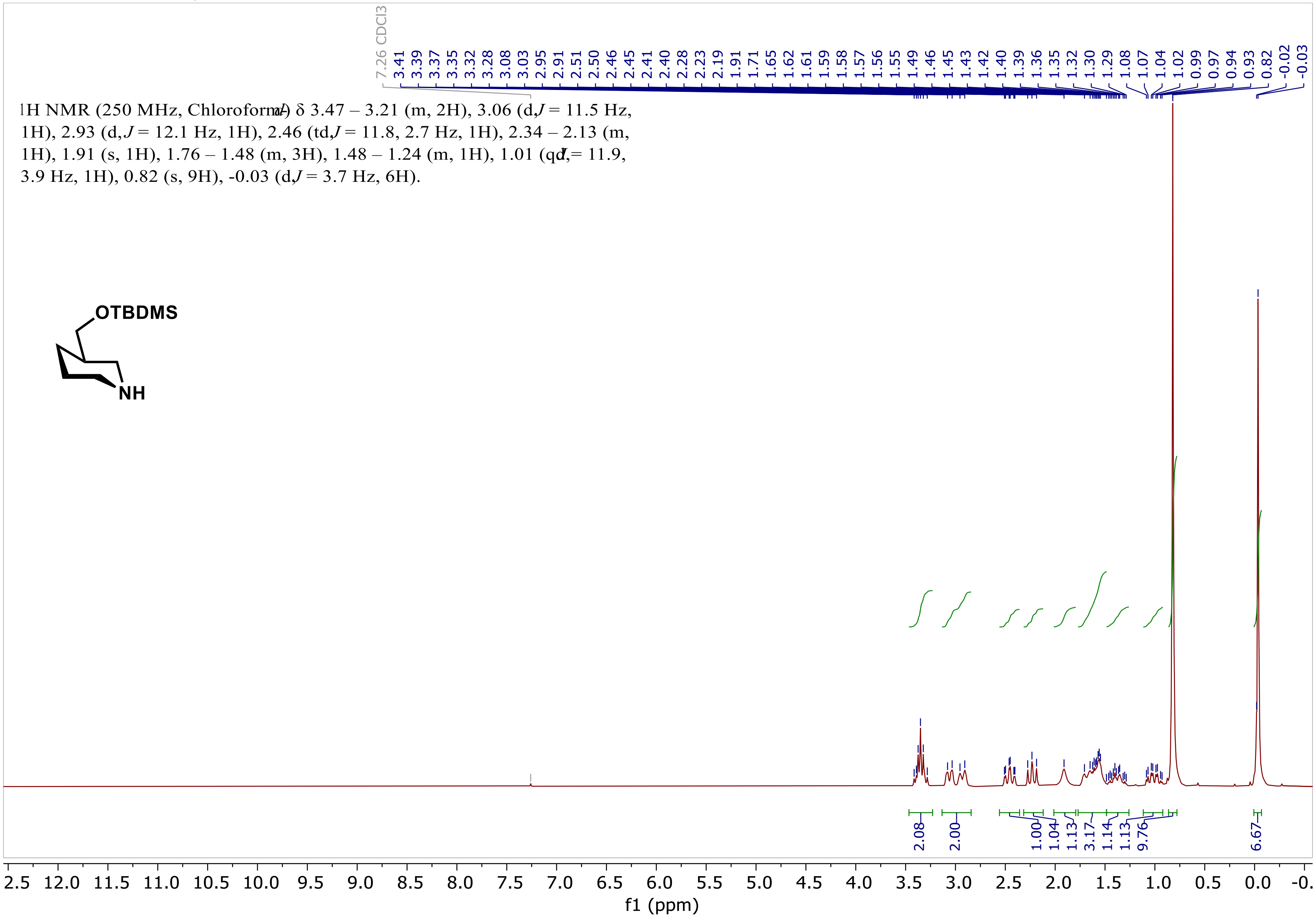

Supplementary Figure 64: *^{13}C NMR for compound* ***A21***

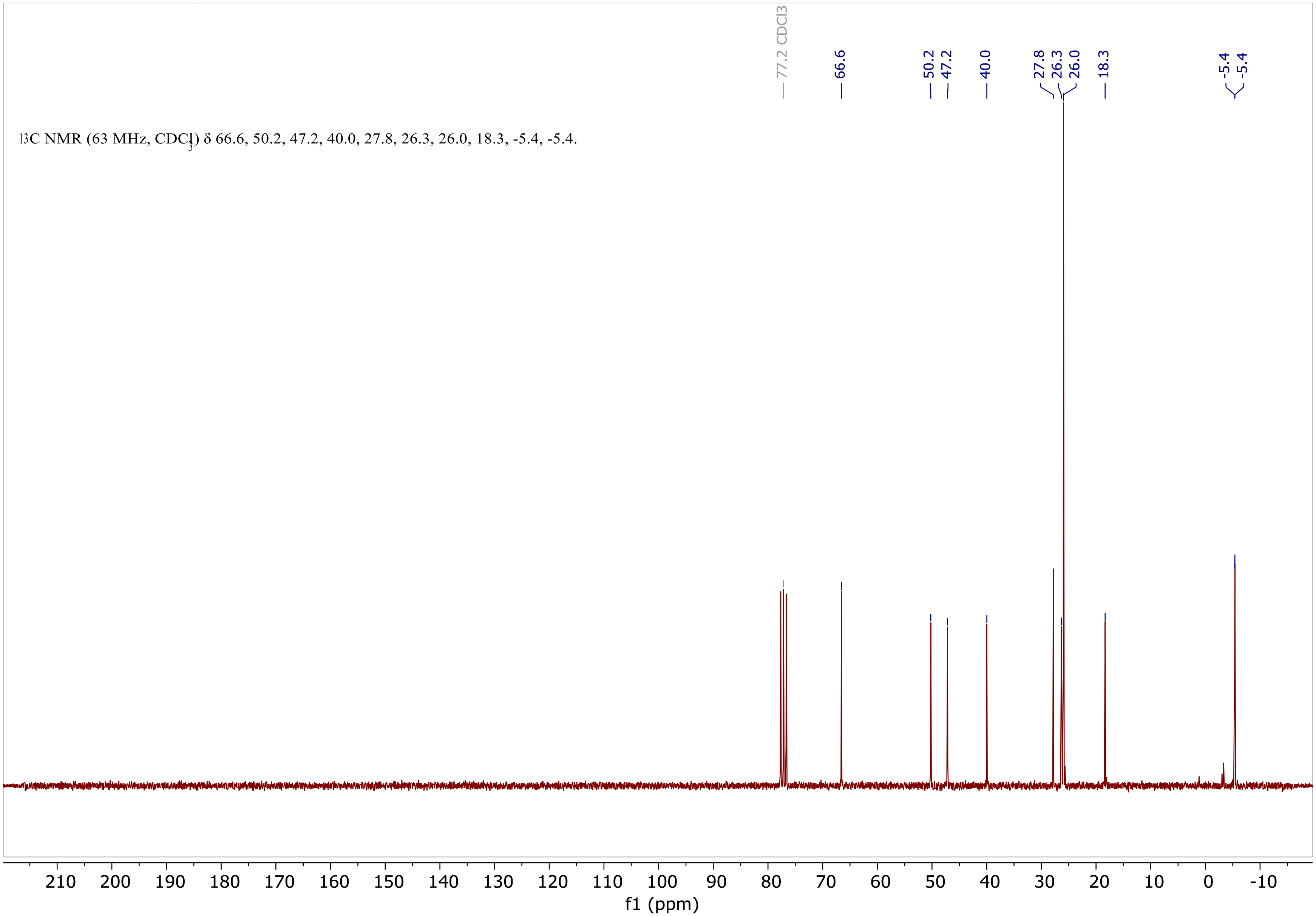

Supplementary Figure 65: *^{1}H-NMR for compound* ***6***

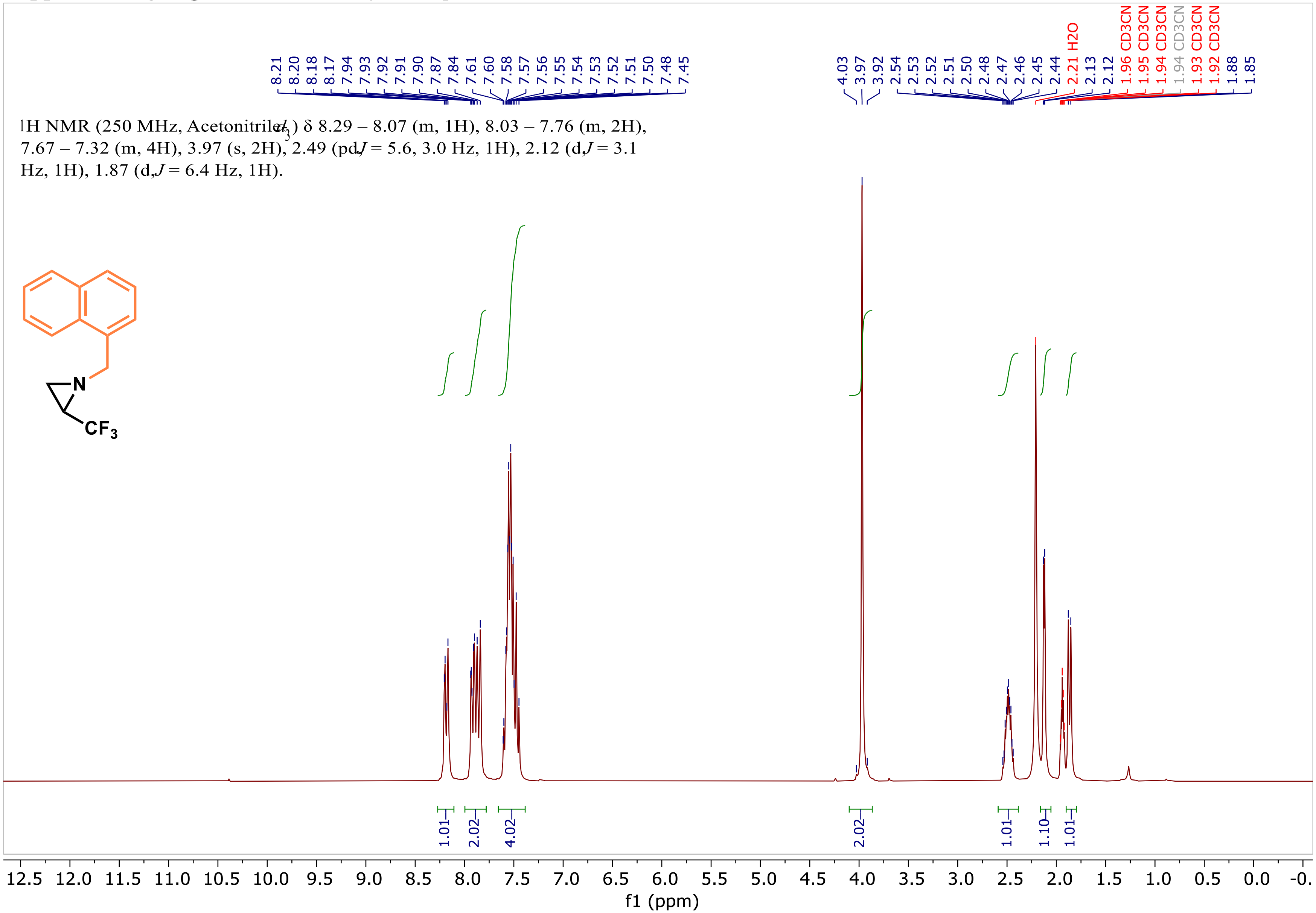

Supplementary Figure 66: *^{19}F NMR for compound* ***6***

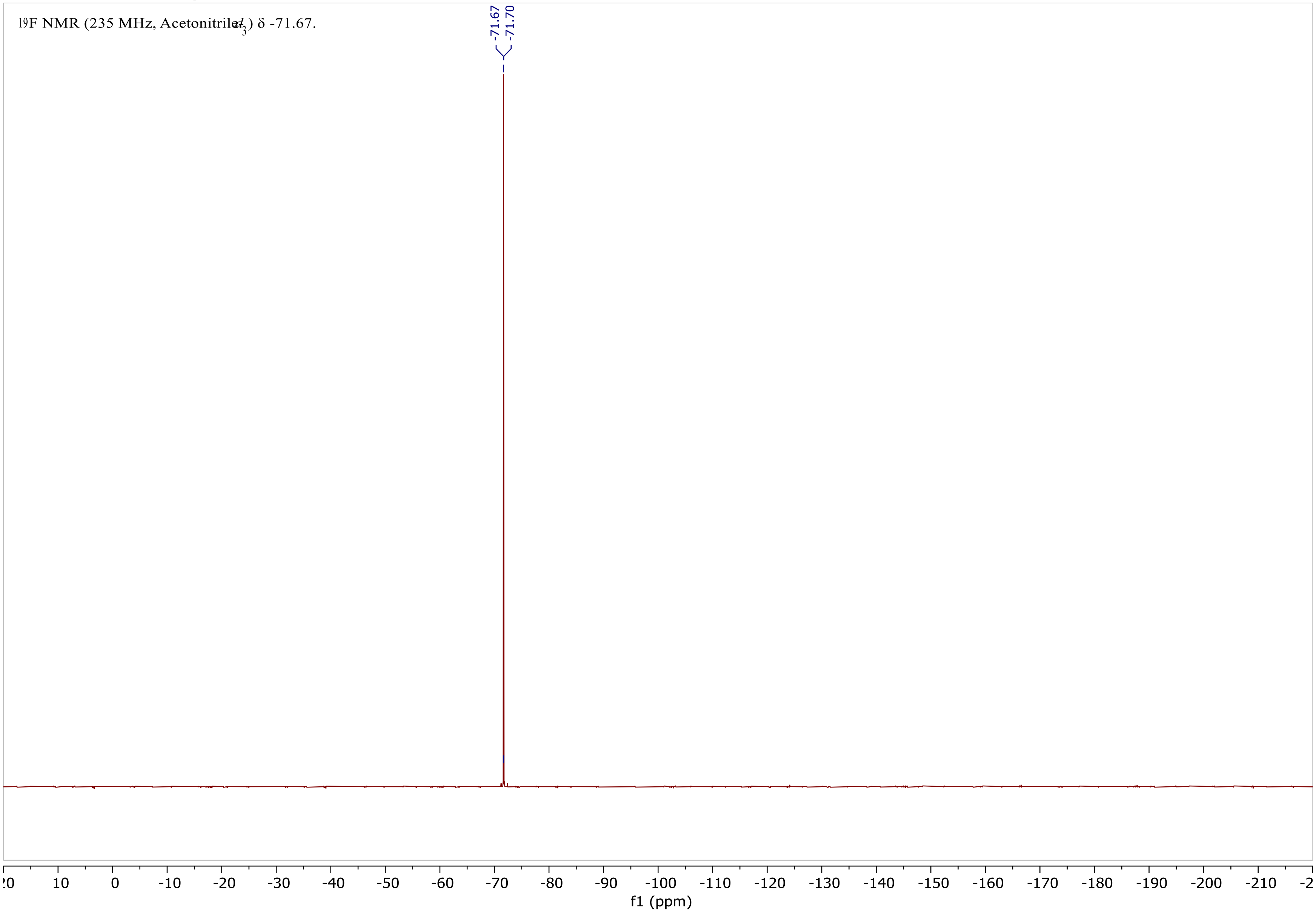

Supplementary Figure 67: ^{13}*C NMR for compound* ***6***

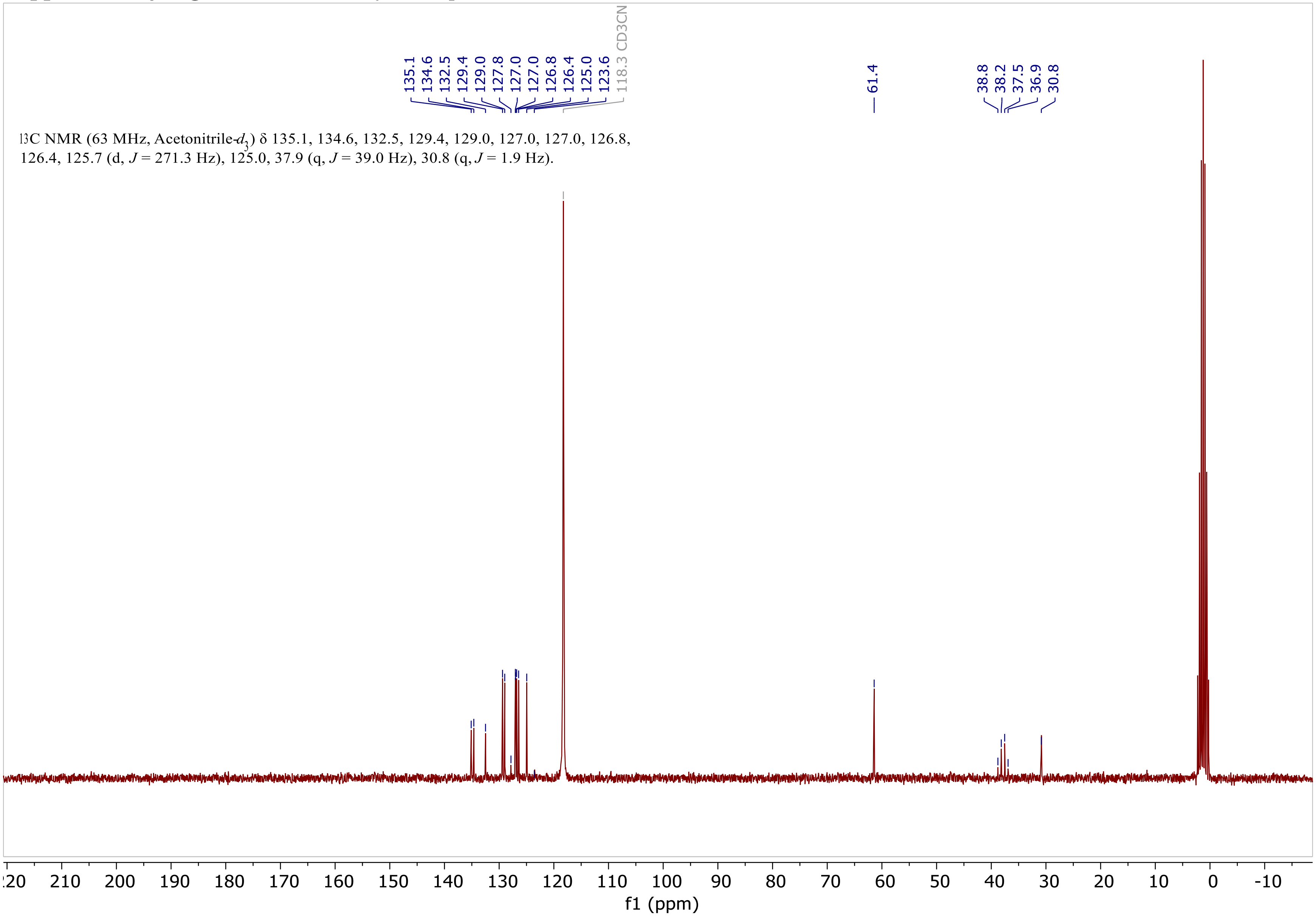

Supplementary Figure 68: *^{1}H-NMR for compound* ***7***

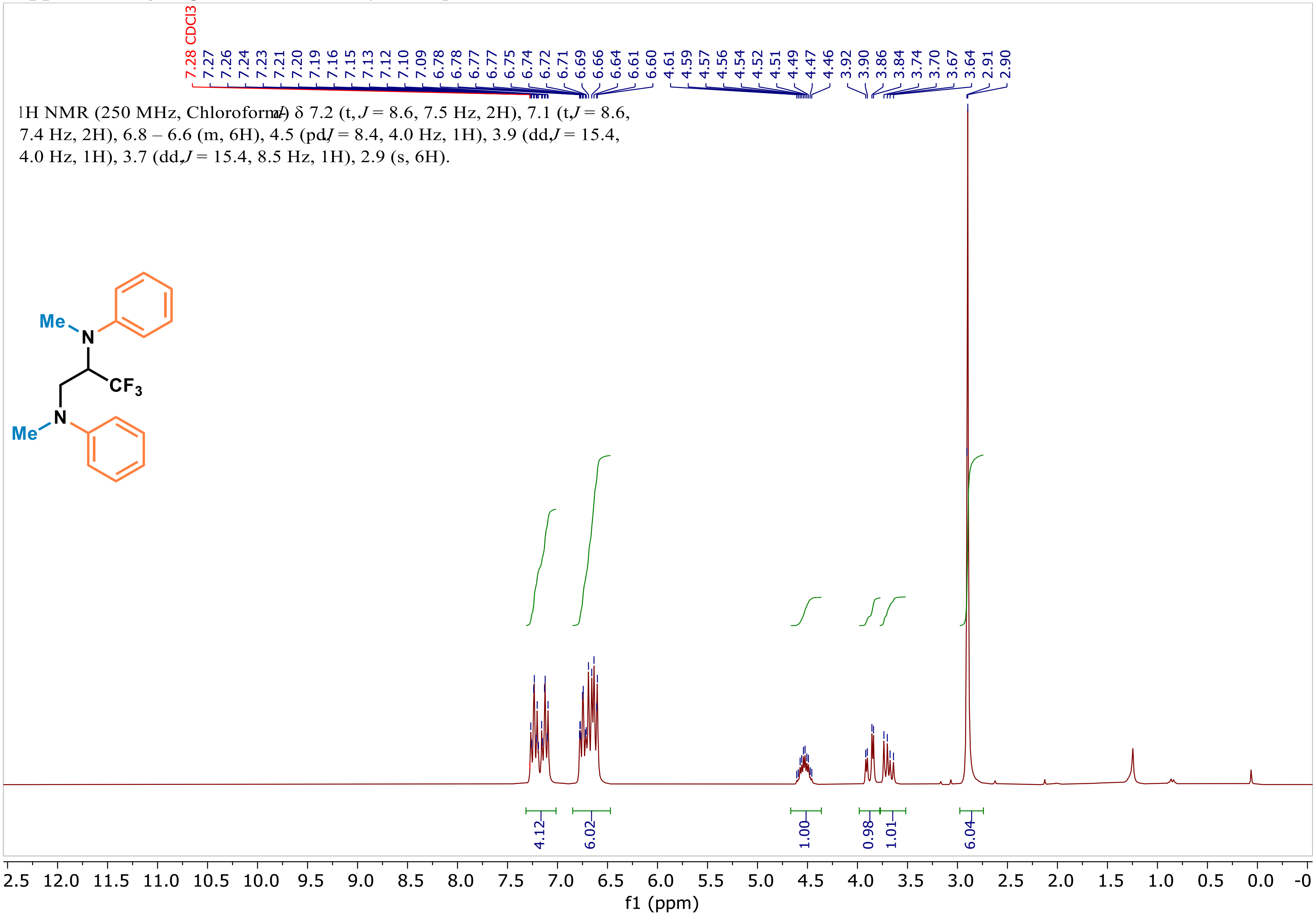

Supplementary Figure 69: *^{19}F NMR for compound* ***7***

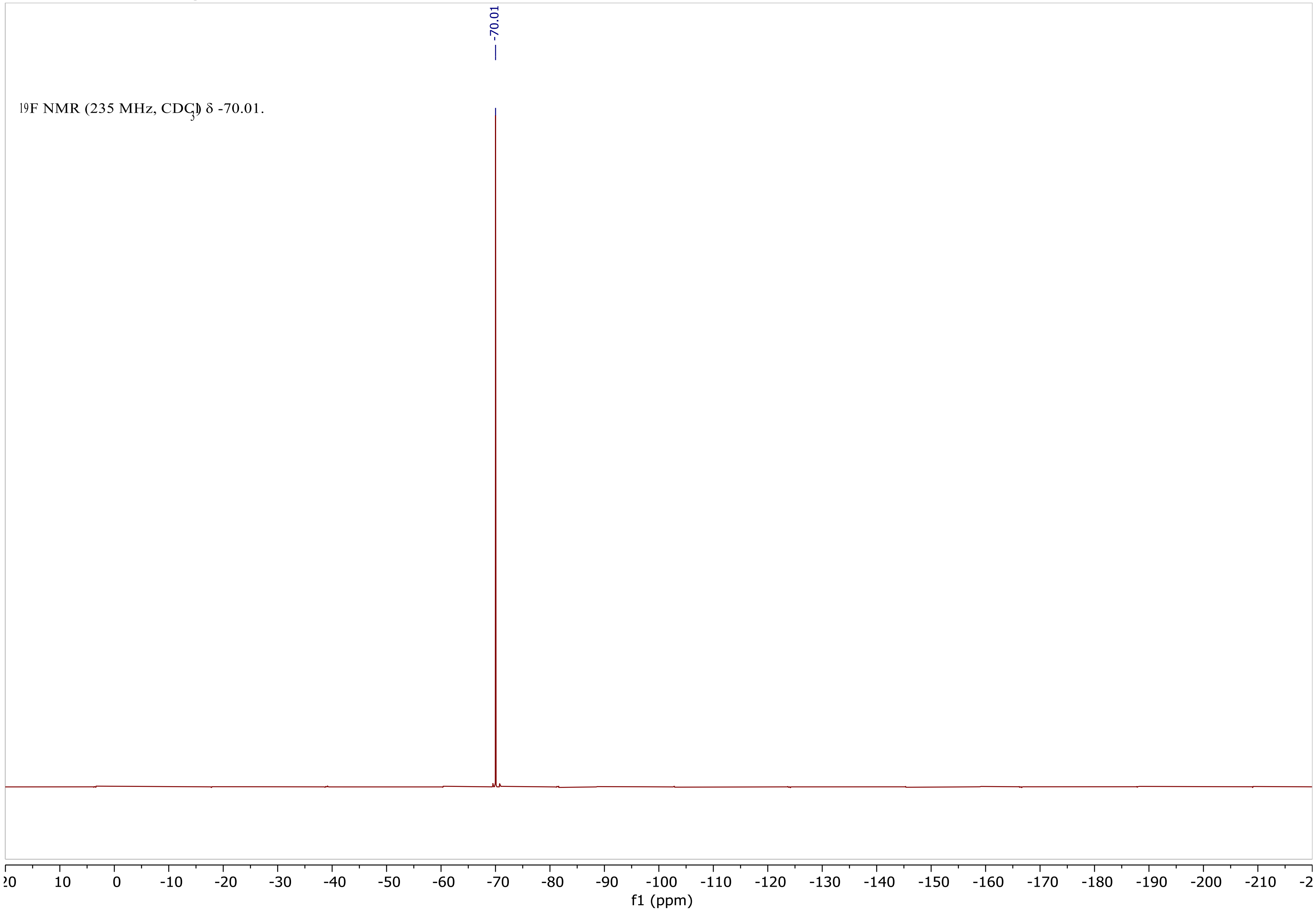

Supplementary Figure 70: *^{13}C NMR for compound **7***

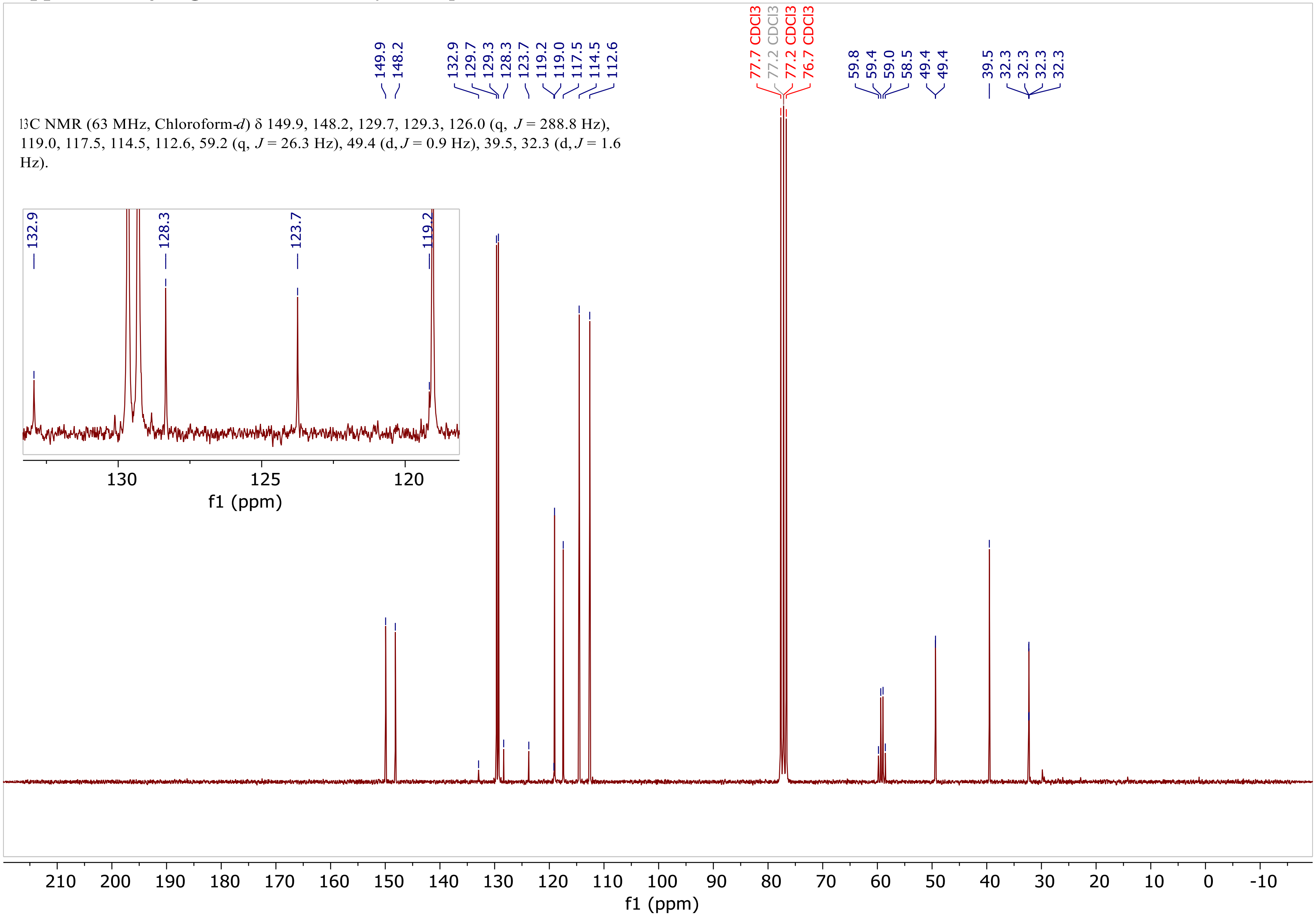

Supplementary Figure 71: *IR for compound* ***7***

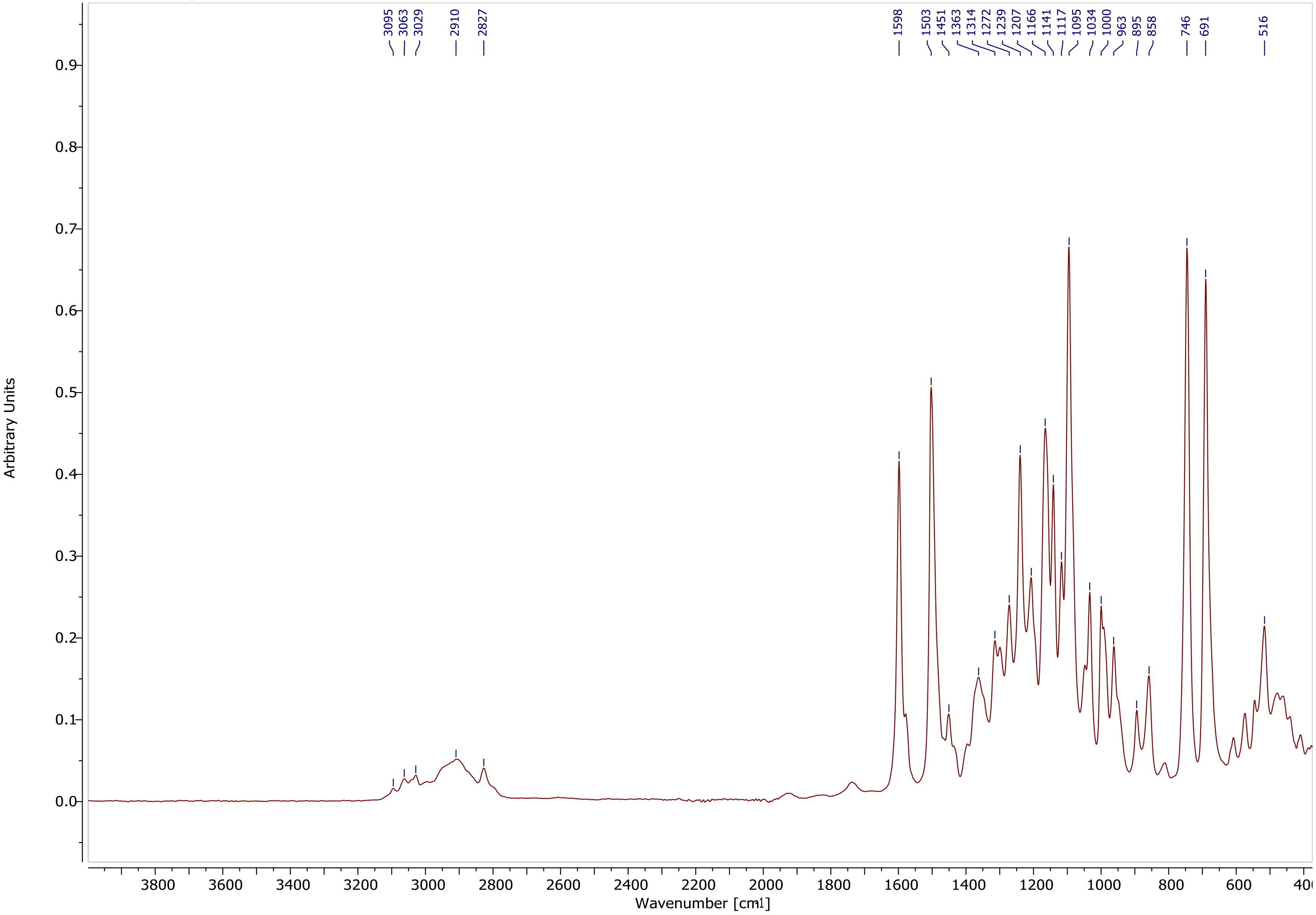

Supplementary Figure 72: *^{1}H-NMR for compound **8***

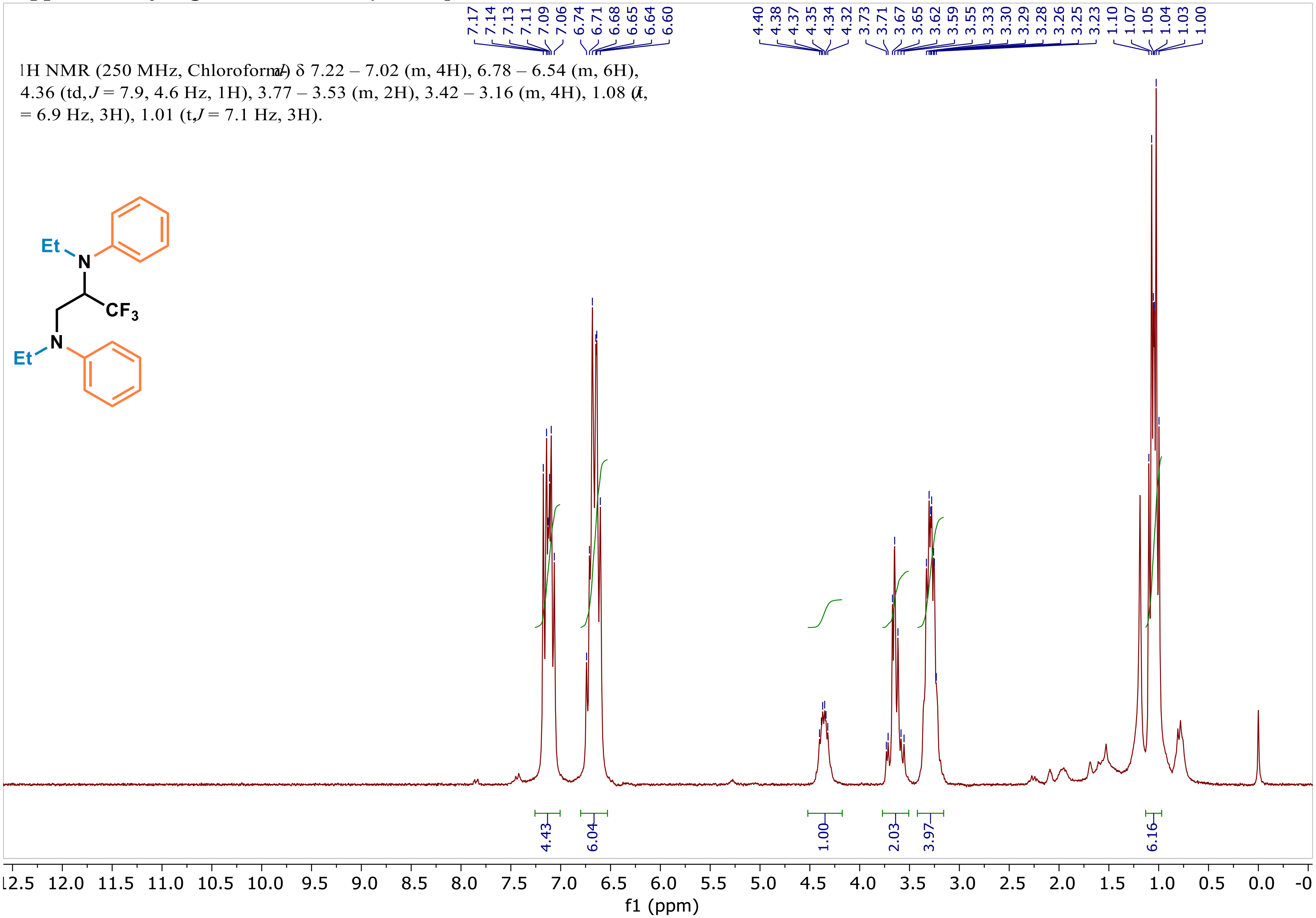

Supplementary Figure 73: *^{19}F NMR for compound* ***8***

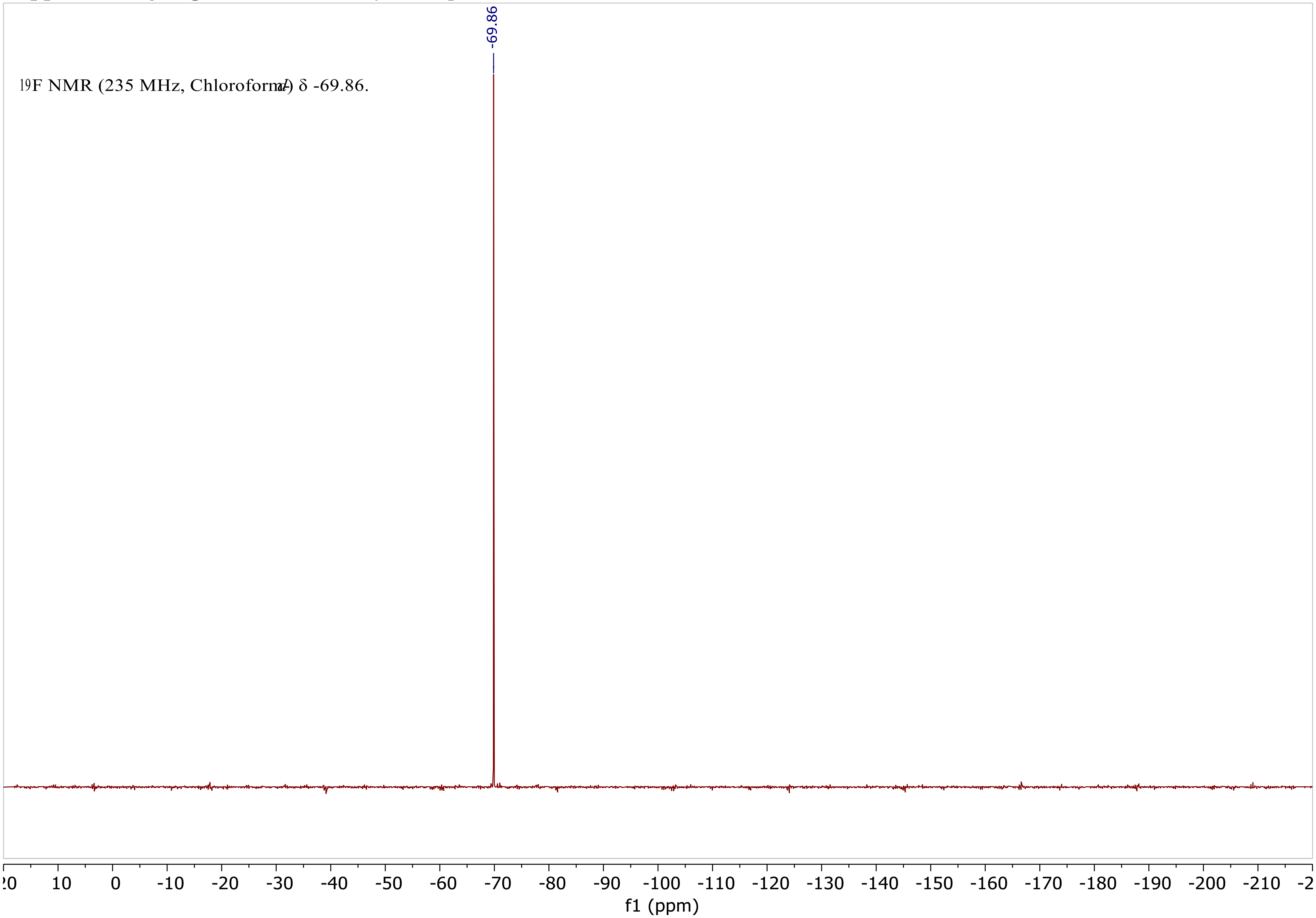

Supplementary Figure 74: *^{13}C NMR for compound* ***8***

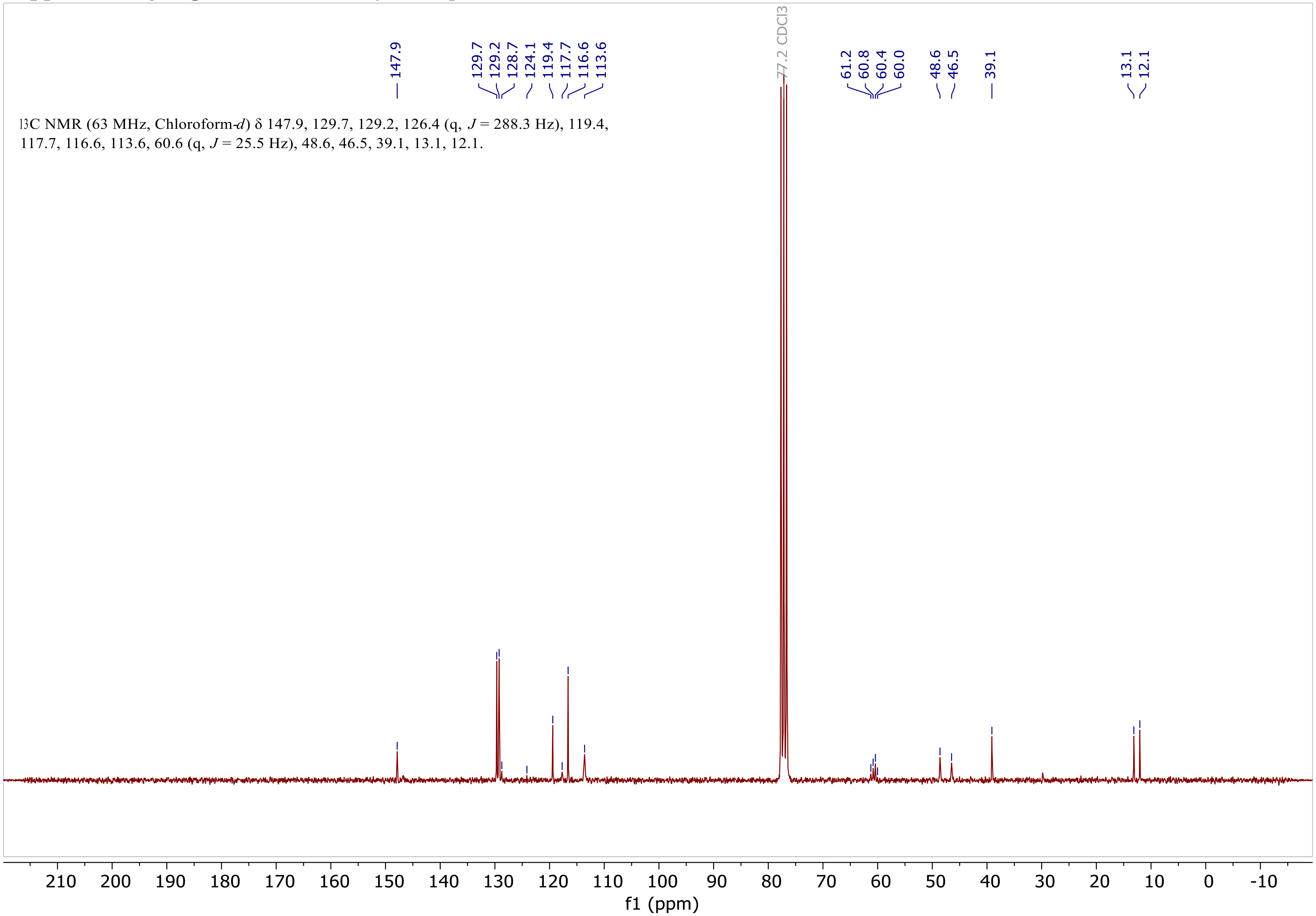

Supplementary Figure 75: *IR for compound **8***

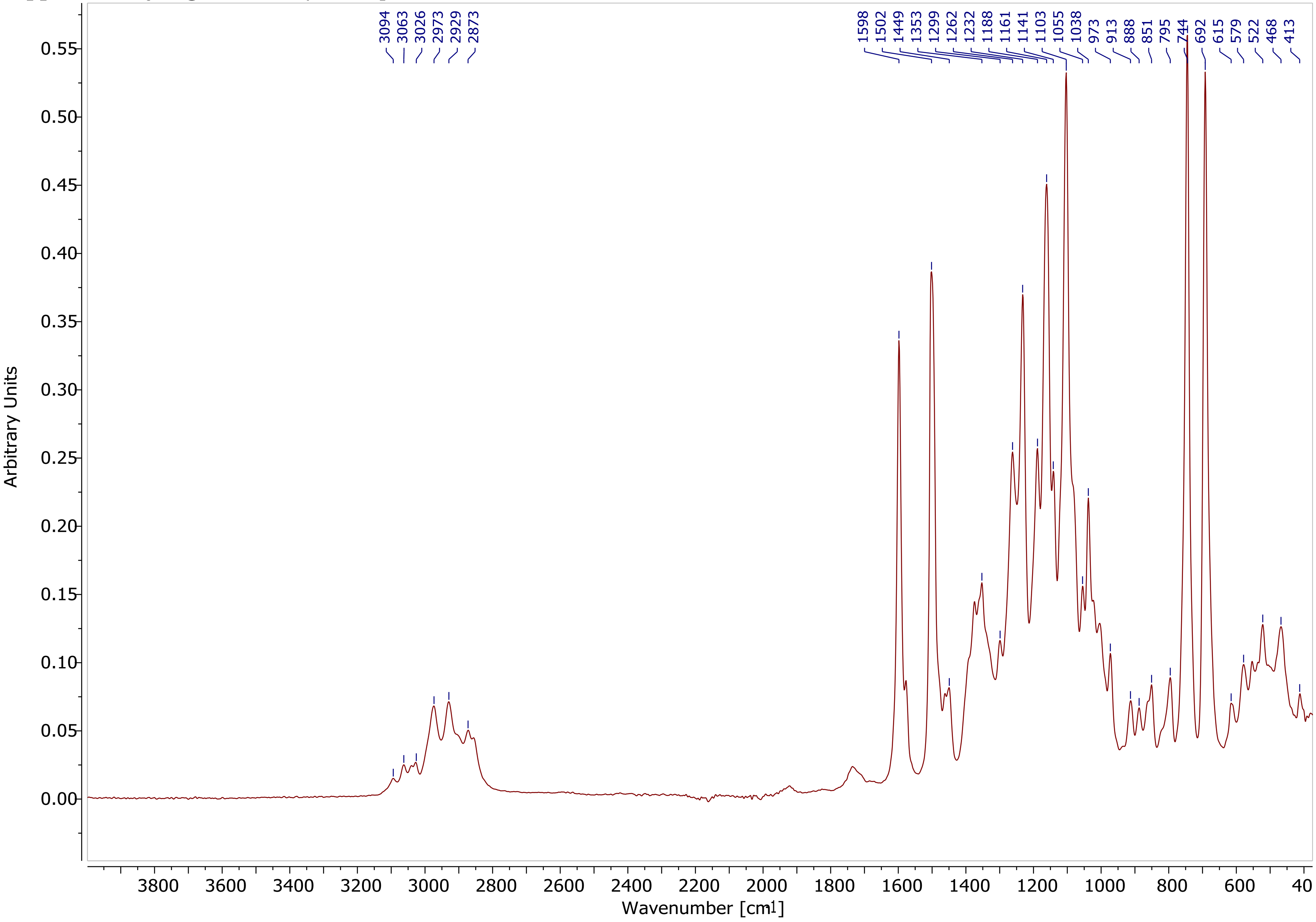

Supplementary Figure 76: *^{1}H-NMR for compound* ***9***

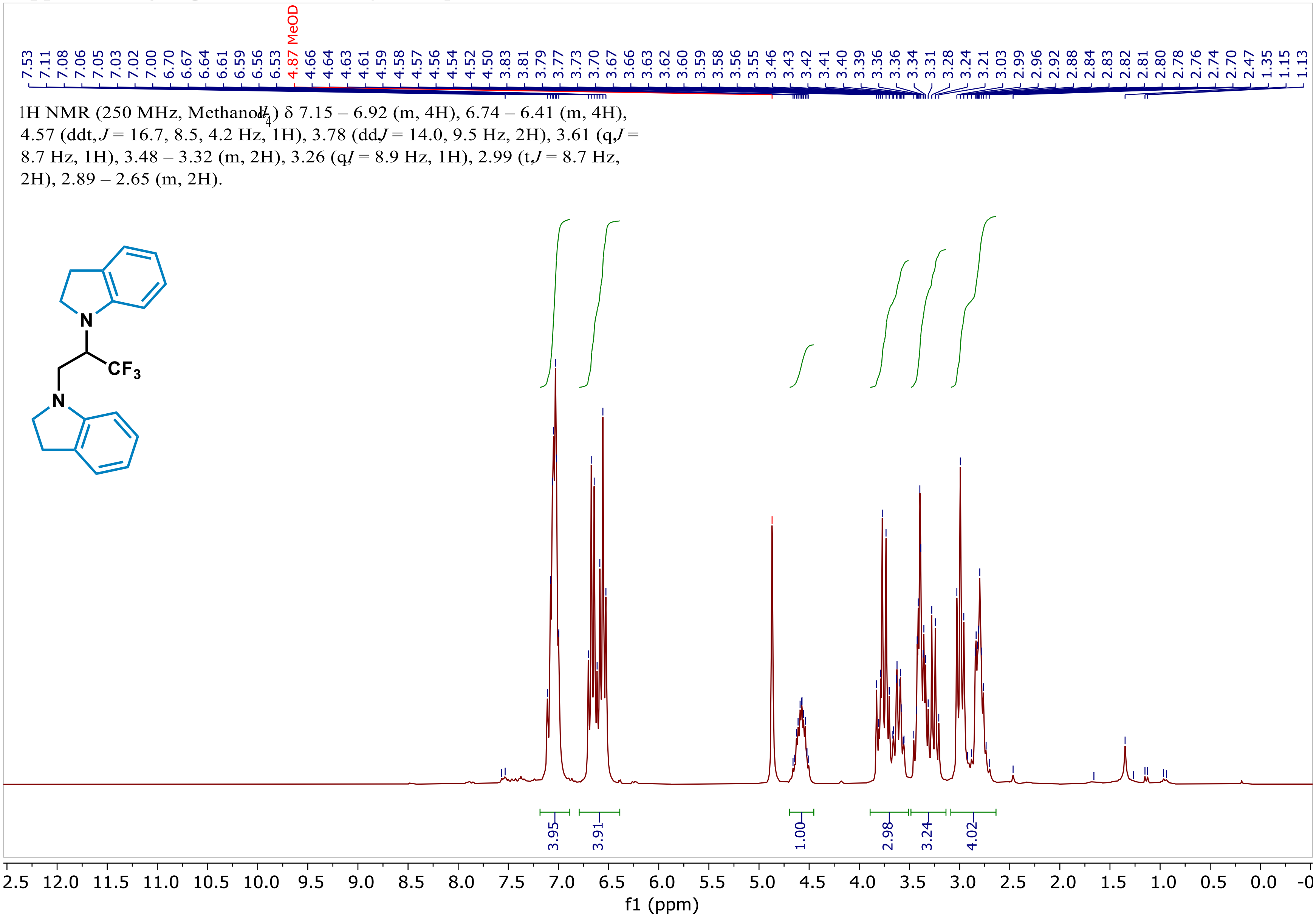

Supplementary Figure 77: *^{19}F NMR for compound* ***9***

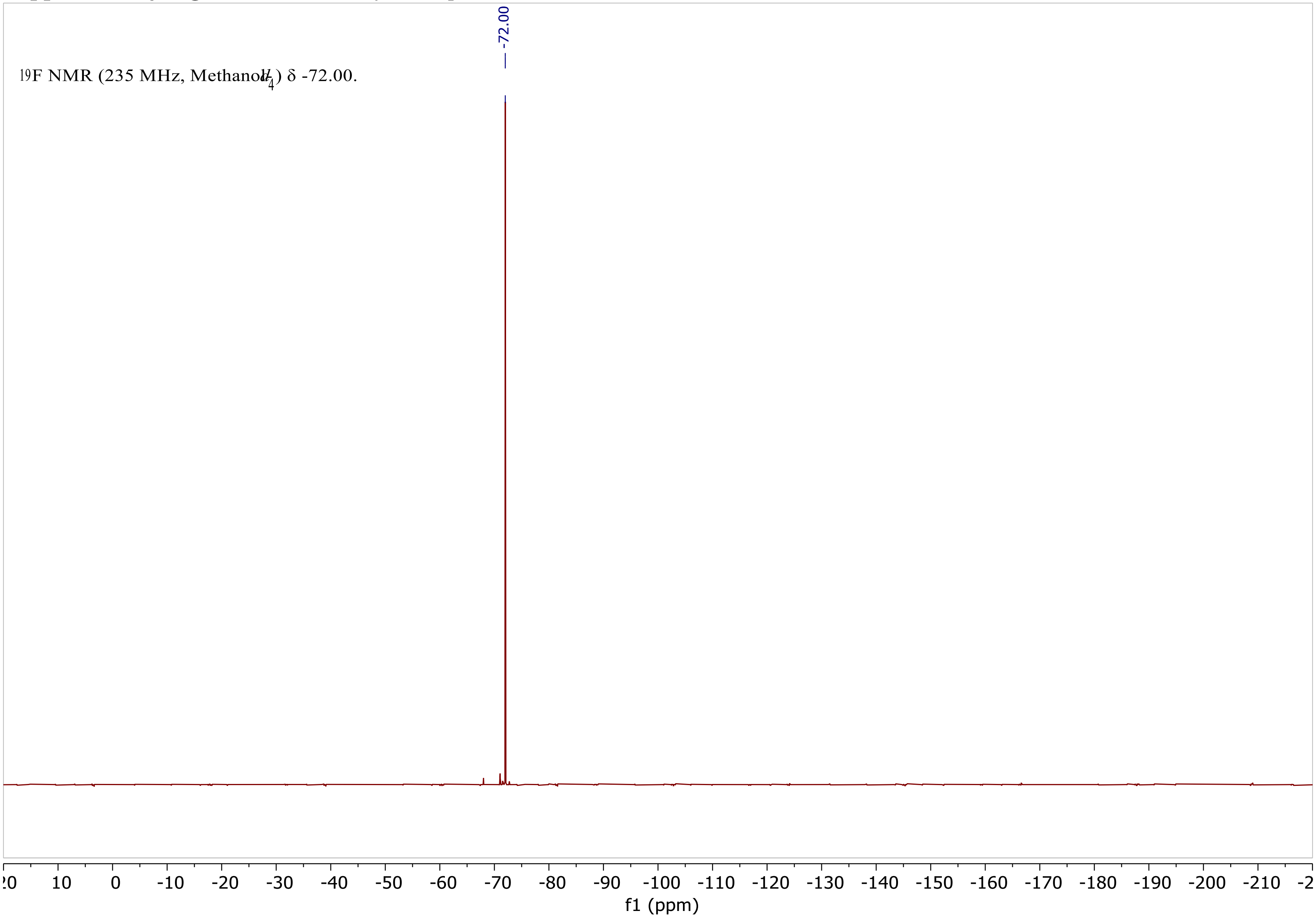

Supplementary Figure 78: *^{13}C NMR for compound **9***

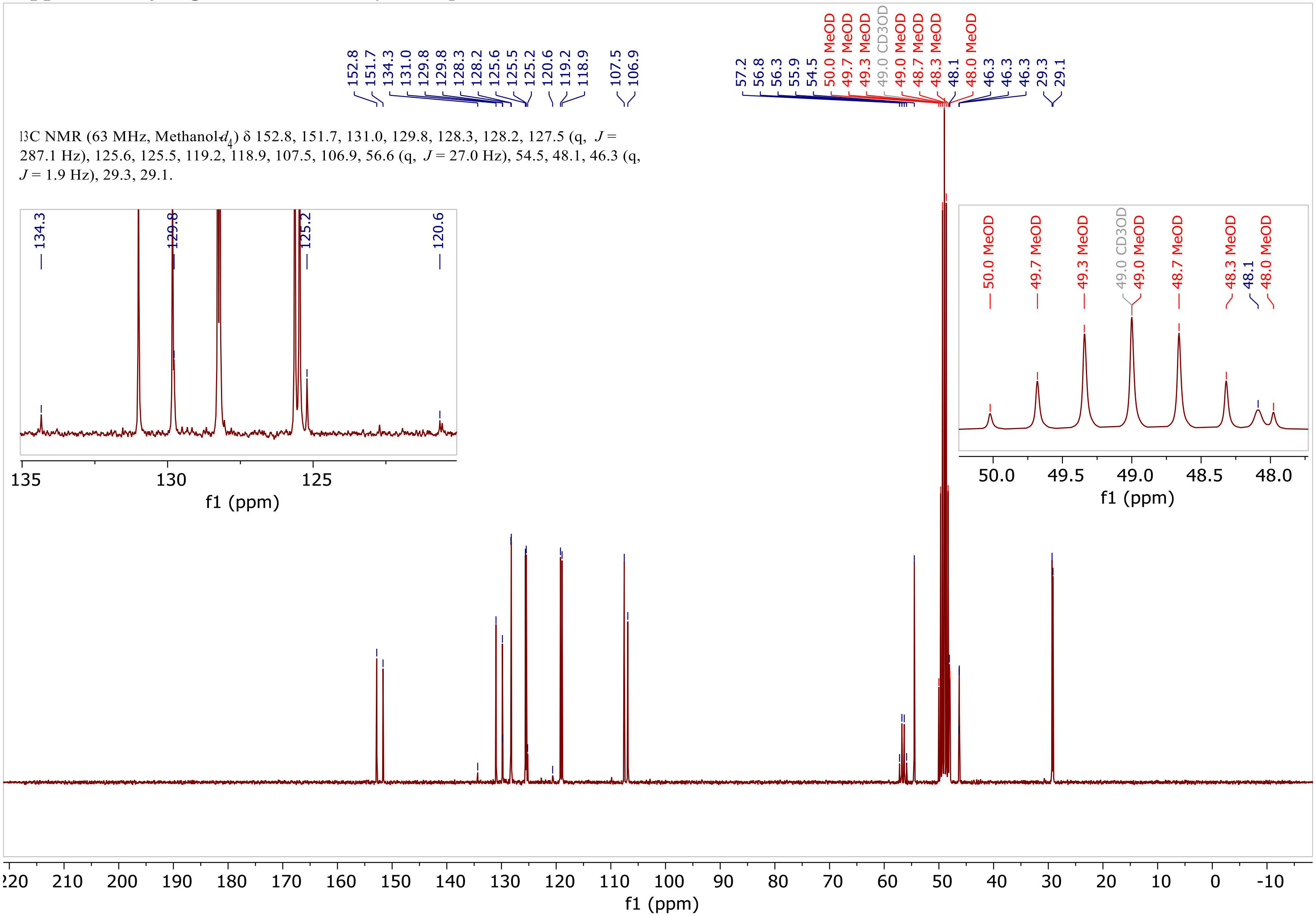

Supplementary Figure 79: *IR for compound* ***9***

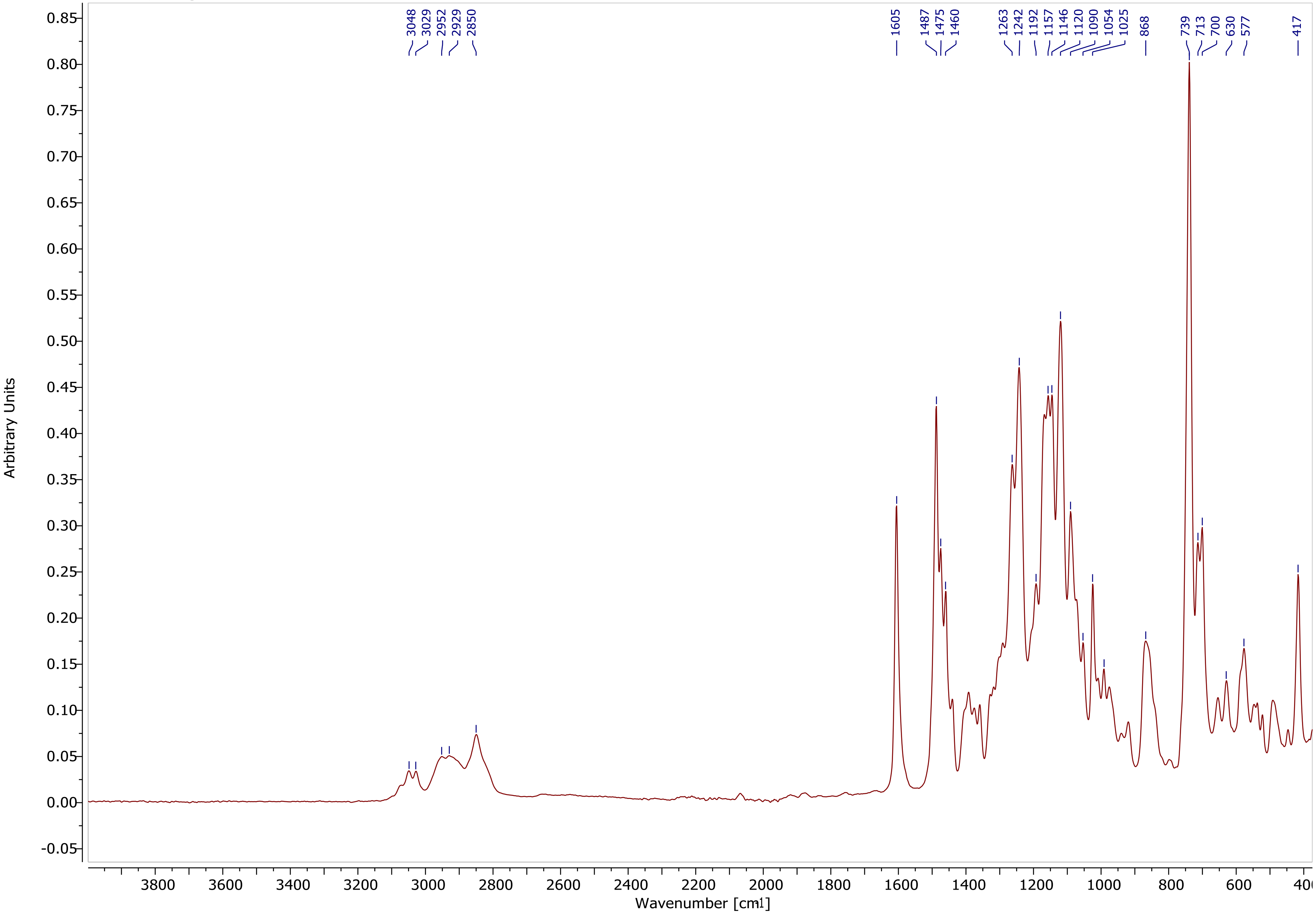

Supplementary Figure 80: *¹H-NMR for compound* ***10***

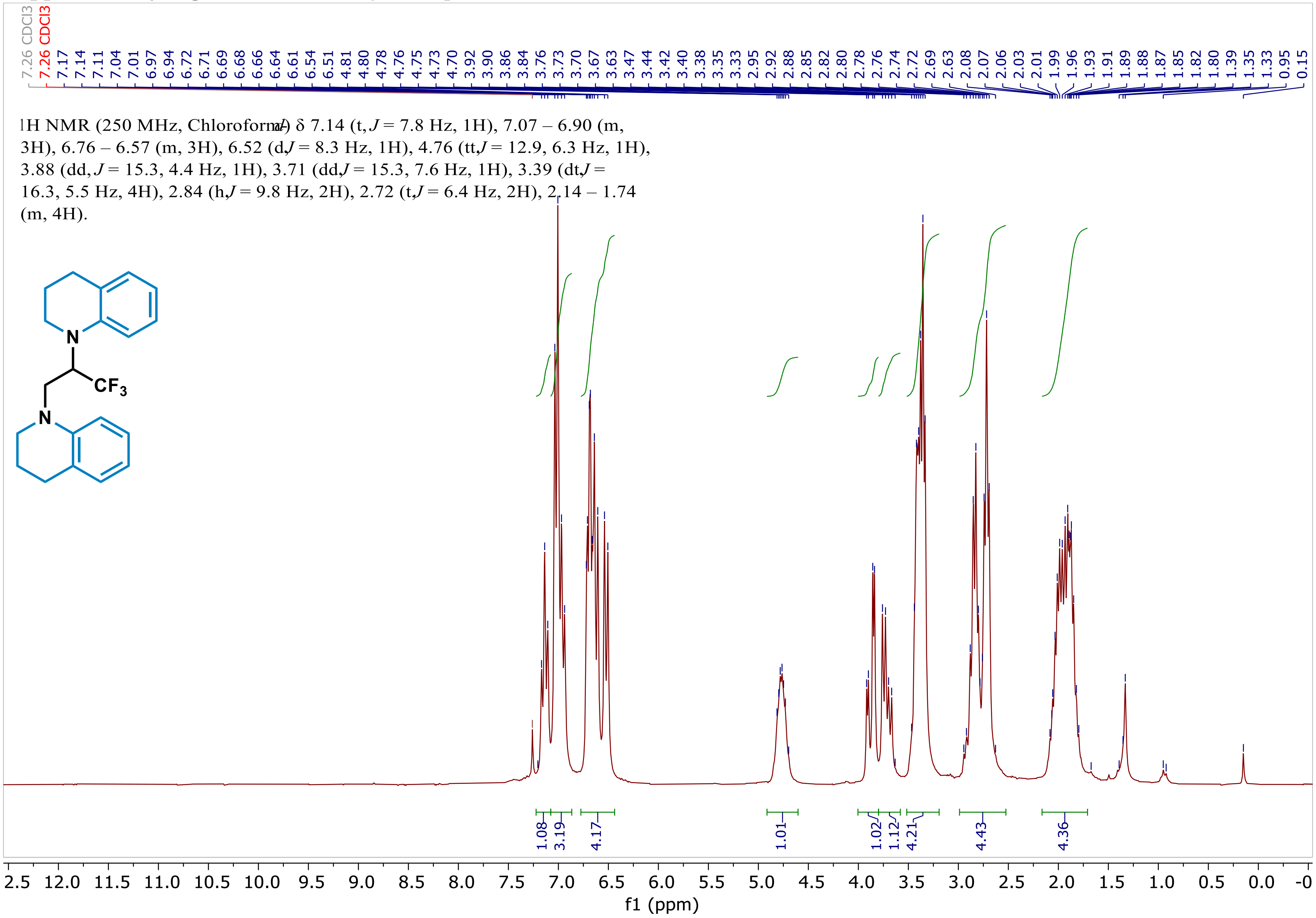

Supplementary Figure 81: *^{19}F NMR for compound* ***10***

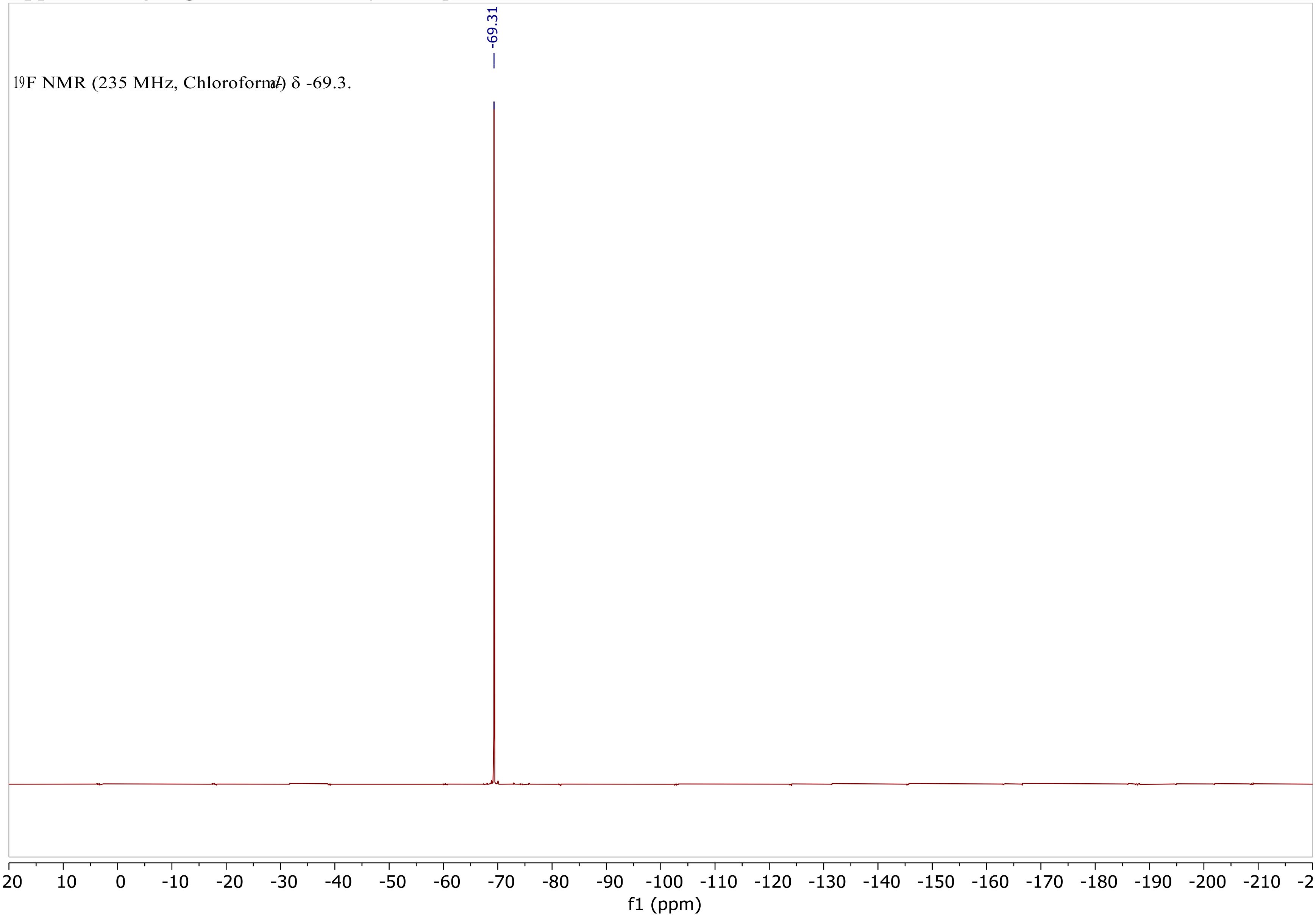

Supplementary Figure 82: *^{13}C NMR for compound* ***10***

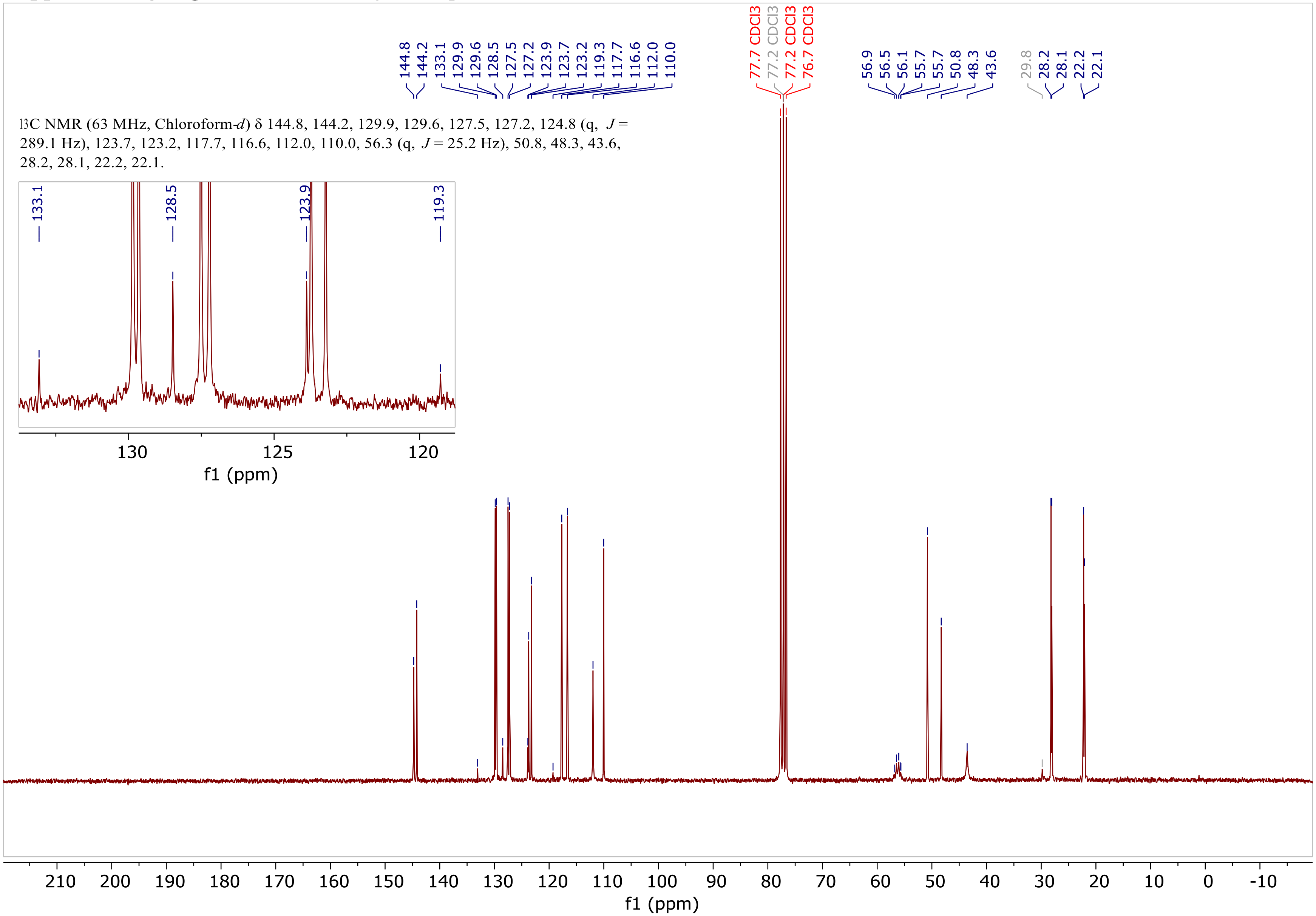

Supplementary Figure 83: *IR for compound **10***

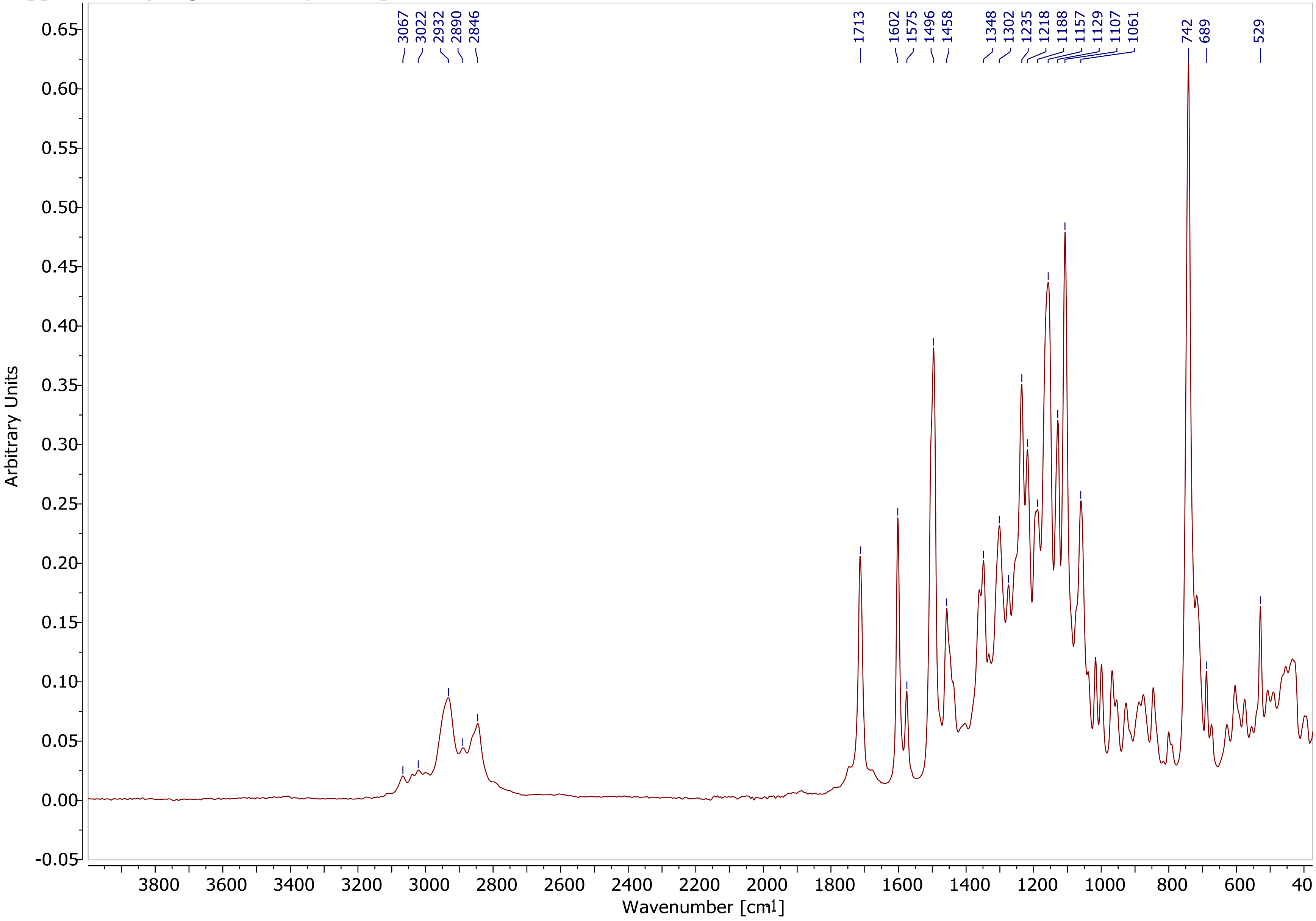

Supplementary Figure 84: *^{1}H-NMR for compound **11***

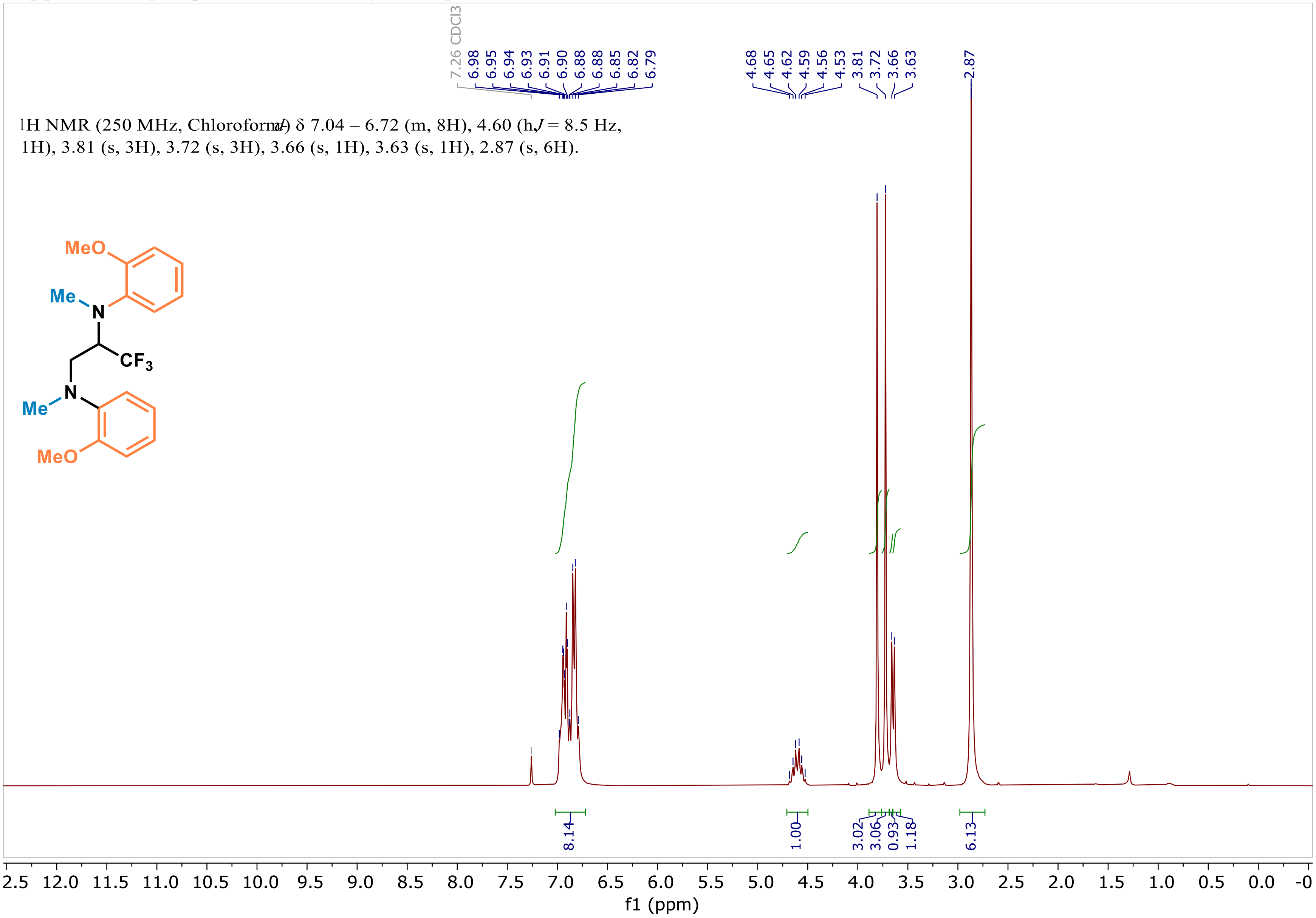

Supplementary Figure 85: ^{19}*F NMR for compound* ***11***

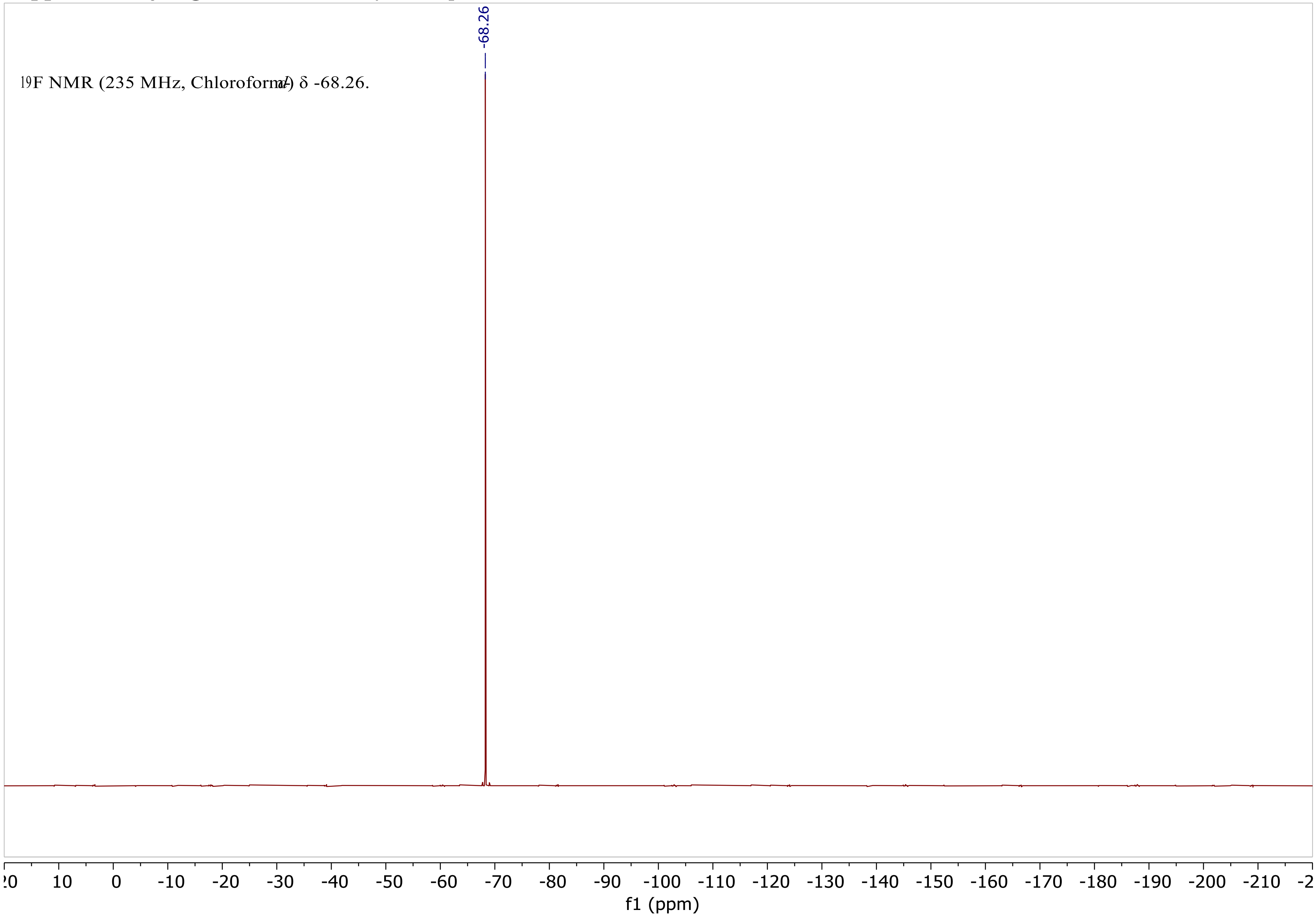

Supplementary Figure 86: *^{13}C NMR for compound* ***11***

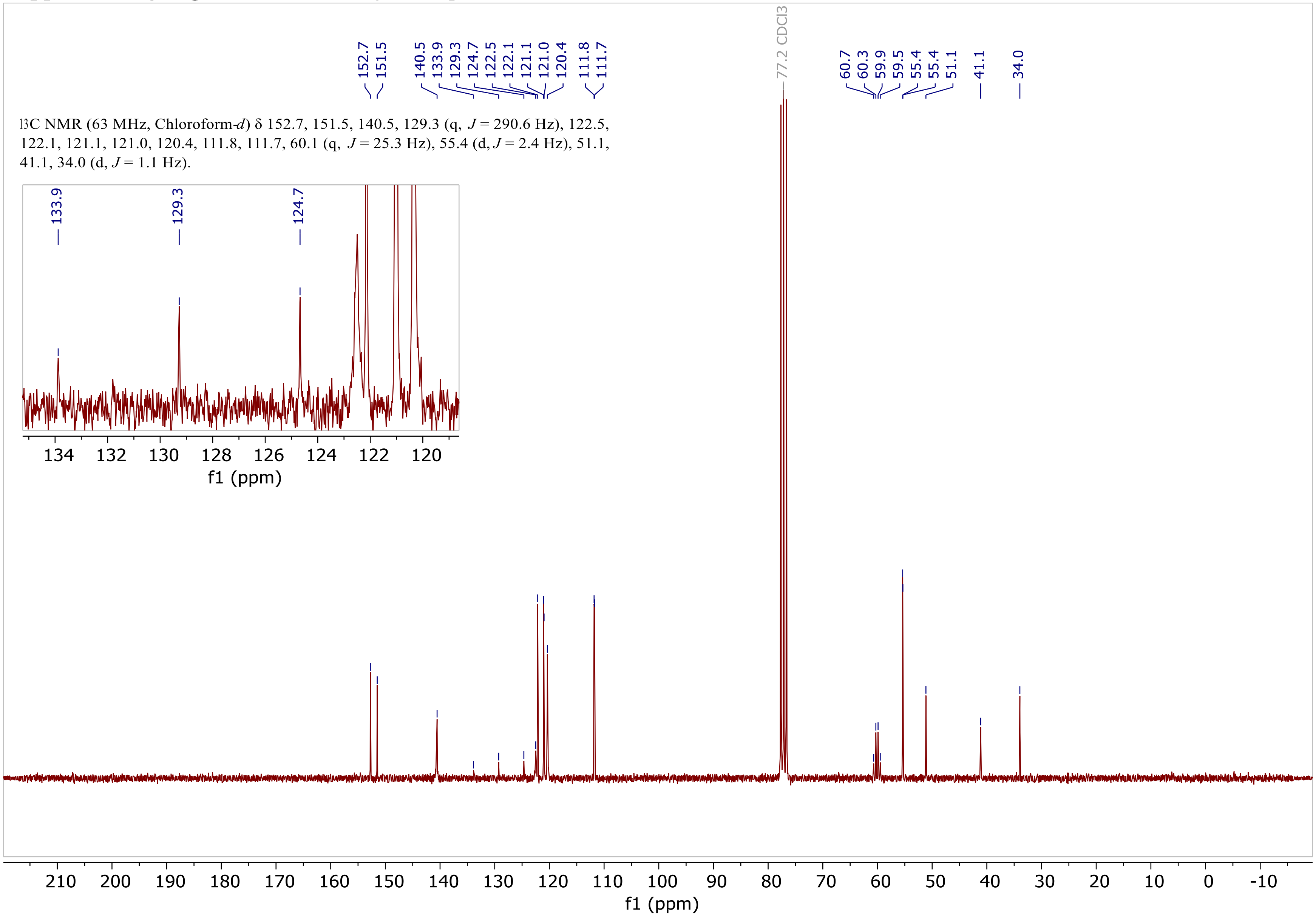

Supplementary Figure 87: *IR for compound* ***11***

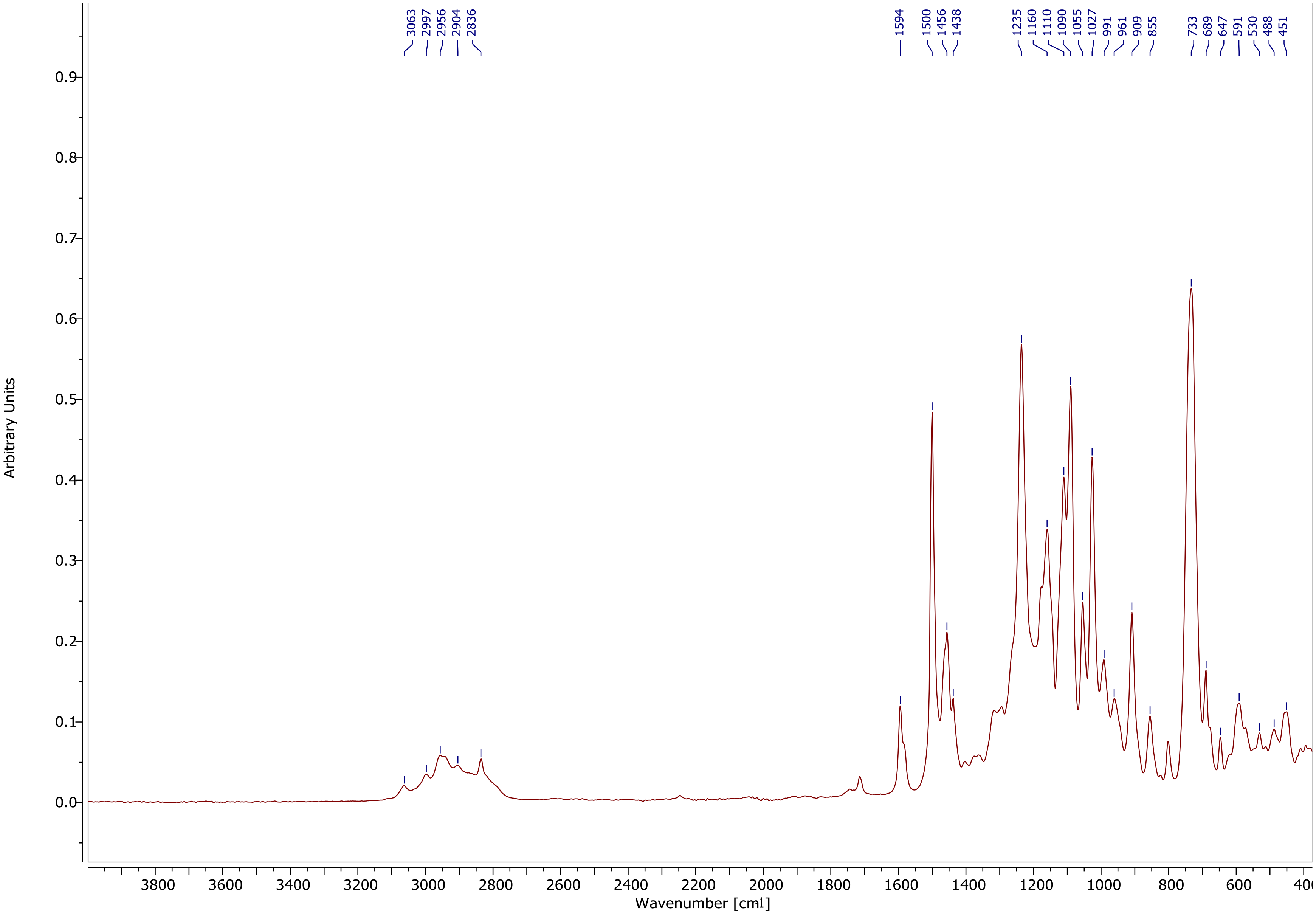

Supplementary Figure 88: *^{1}H-NMR for compound* ***12***

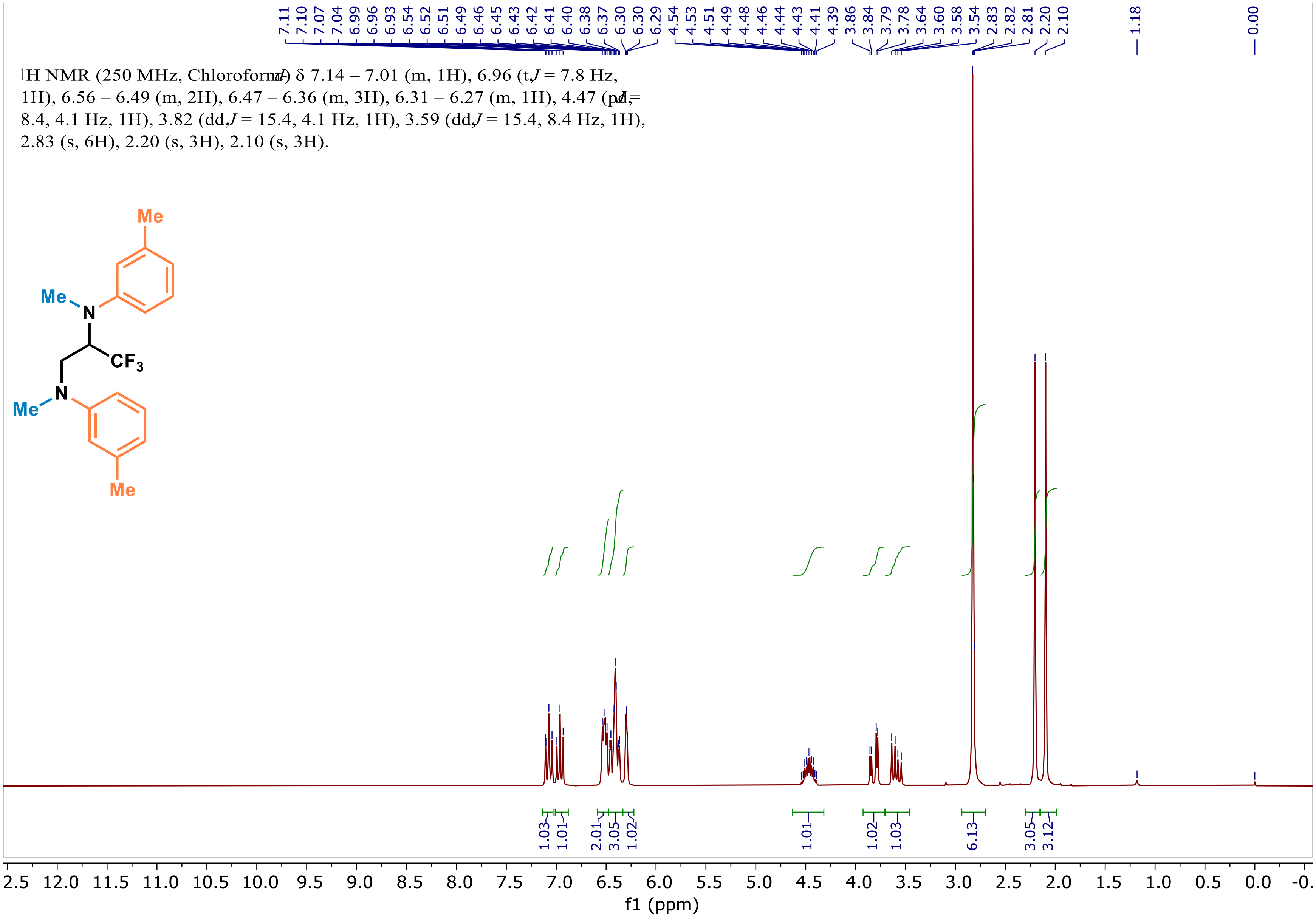

Supplementary Figure 89: *^{19}F NMR for compound* ***12***

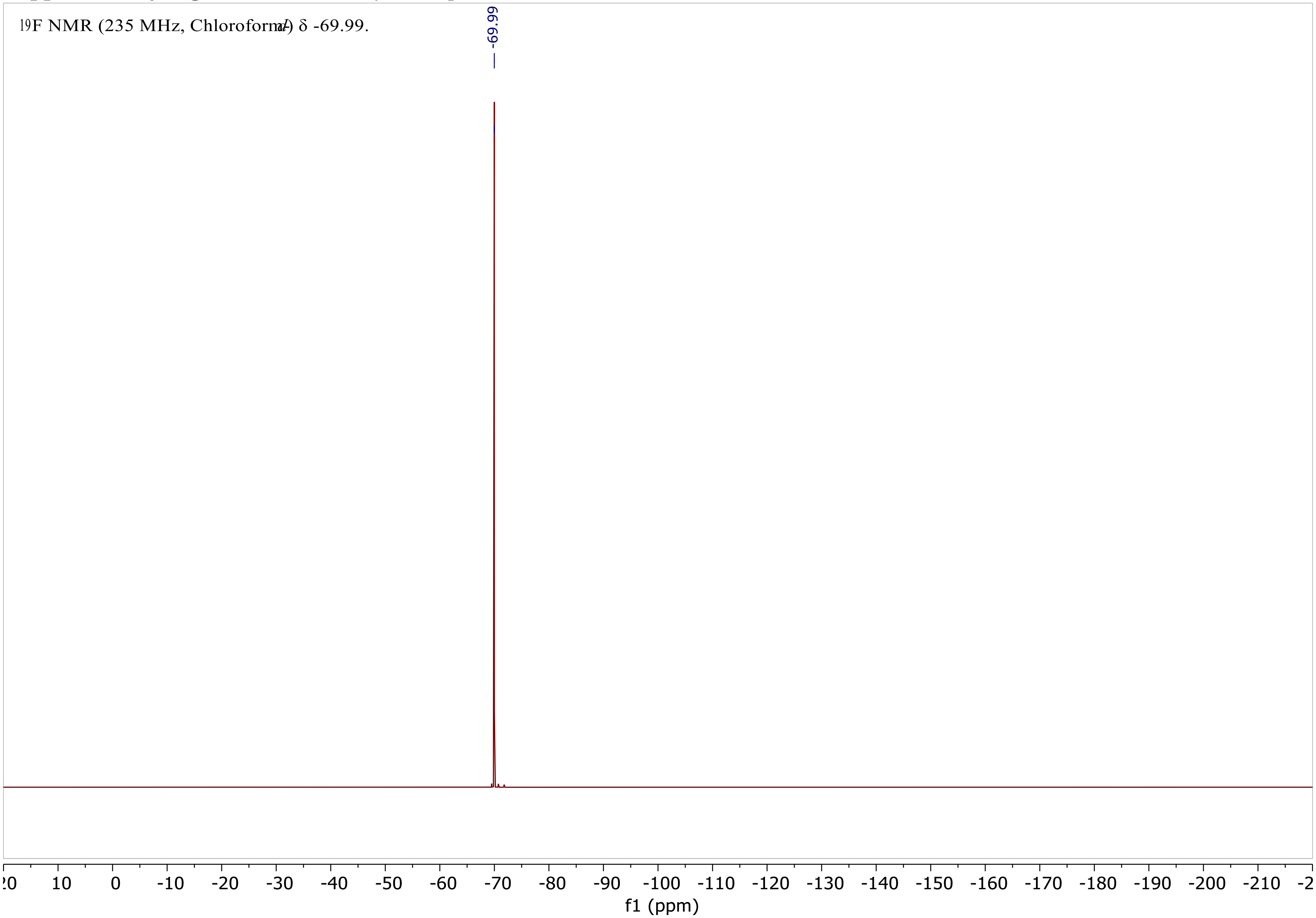

Supplementary Figure 90: *^{13}C NMR for compound* ***12***

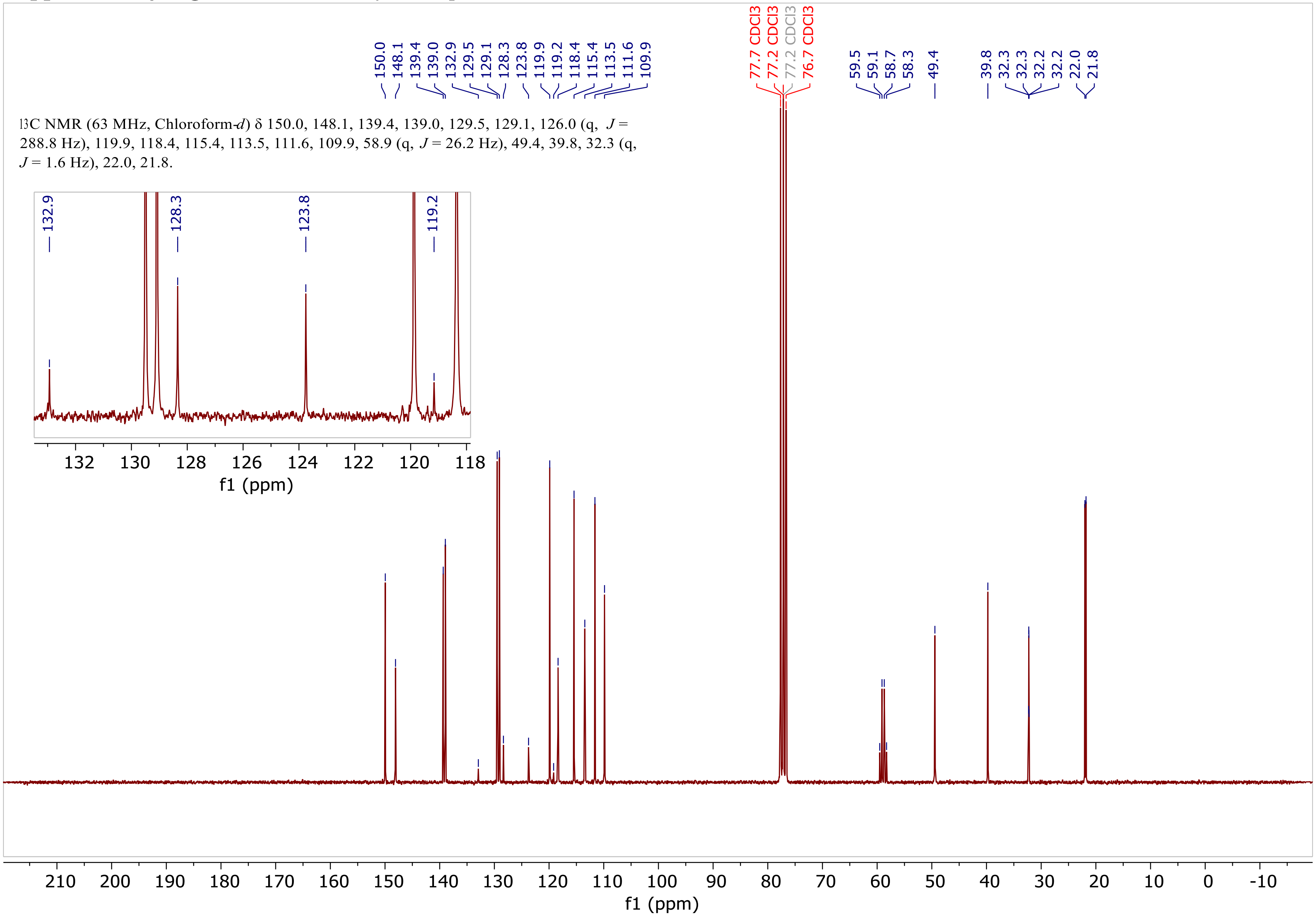

Supplementary Figure 91: *IR for compound* ***12***

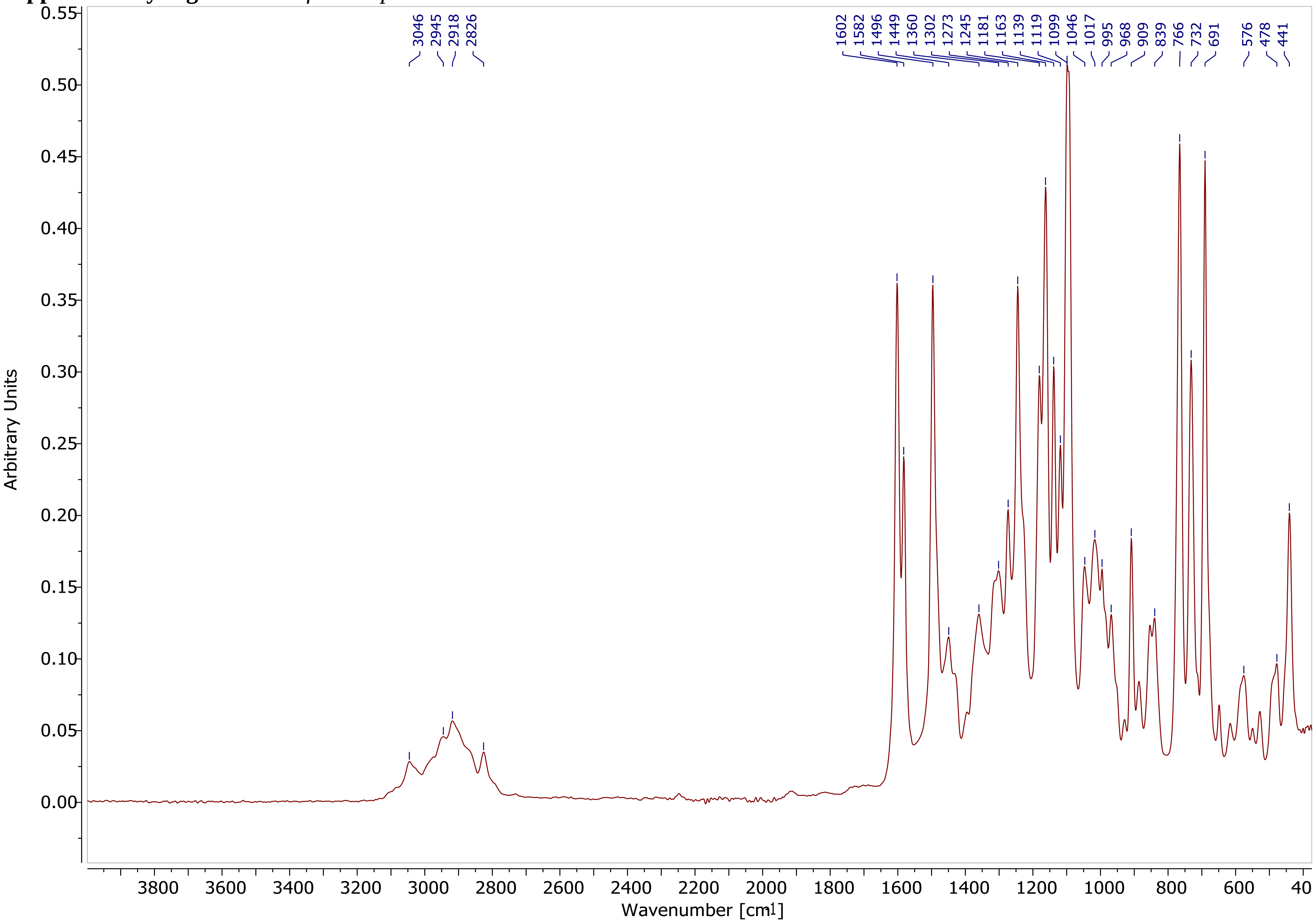

Supplementary Figure 92: *^{1}H-NMR for compound **13***

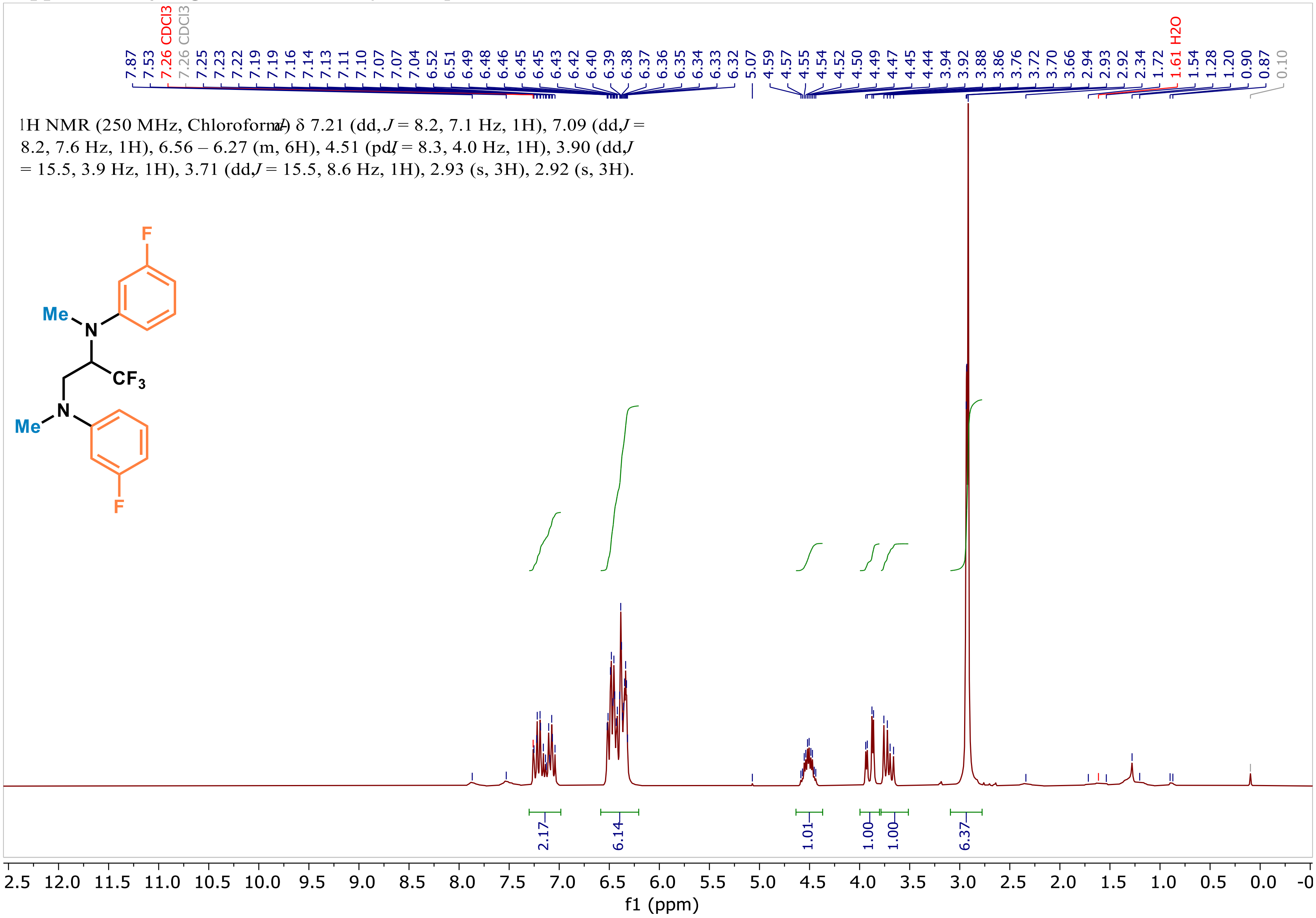

Supplementary Figure 93: *^{19}F NMR for compound* ***13***

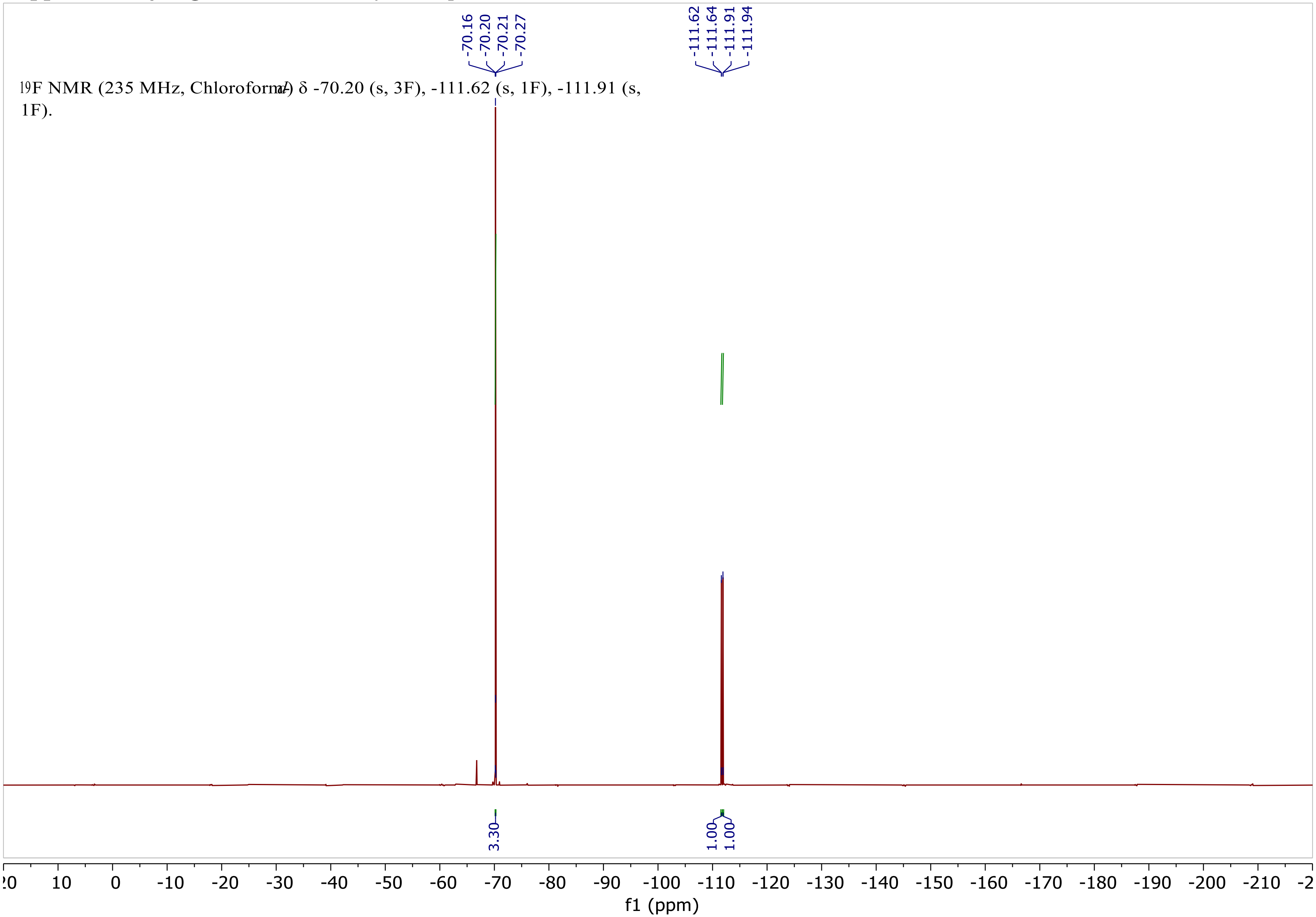

Supplementary Figure 94: *^{13}C NMR for compound* ***13***

13C NMR (63 MHz, Chloroform-*d*) δ 164.5 (d, *J* = 243.1 Hz), 164.0 (d, *J* = 243.6 Hz), 151.5 (d, *J* = 10.2 Hz), 149.8 (d, *J* = 10.5 Hz), 130.8 (d, *J* = 10.3 Hz), 130.4 (d, *J* = 10.1 Hz), 125.7 (q, *J* = 288.2 Hz), 109.8 (d, *J* = 2.5 Hz), 107.9 (d, *J* = 2.4 Hz), 105.7 (d, *J* = 21.4 Hz), 104.1 (d, *J* = 21.6 Hz), 101.6 (d, *J* = 26.0 Hz), 99.7 (d, *J* = 26.2 Hz), 58.9 (q, *J* = 26.9 Hz), 49.2, 39.6, 32.5 (q, *J* = 1.7 Hz).

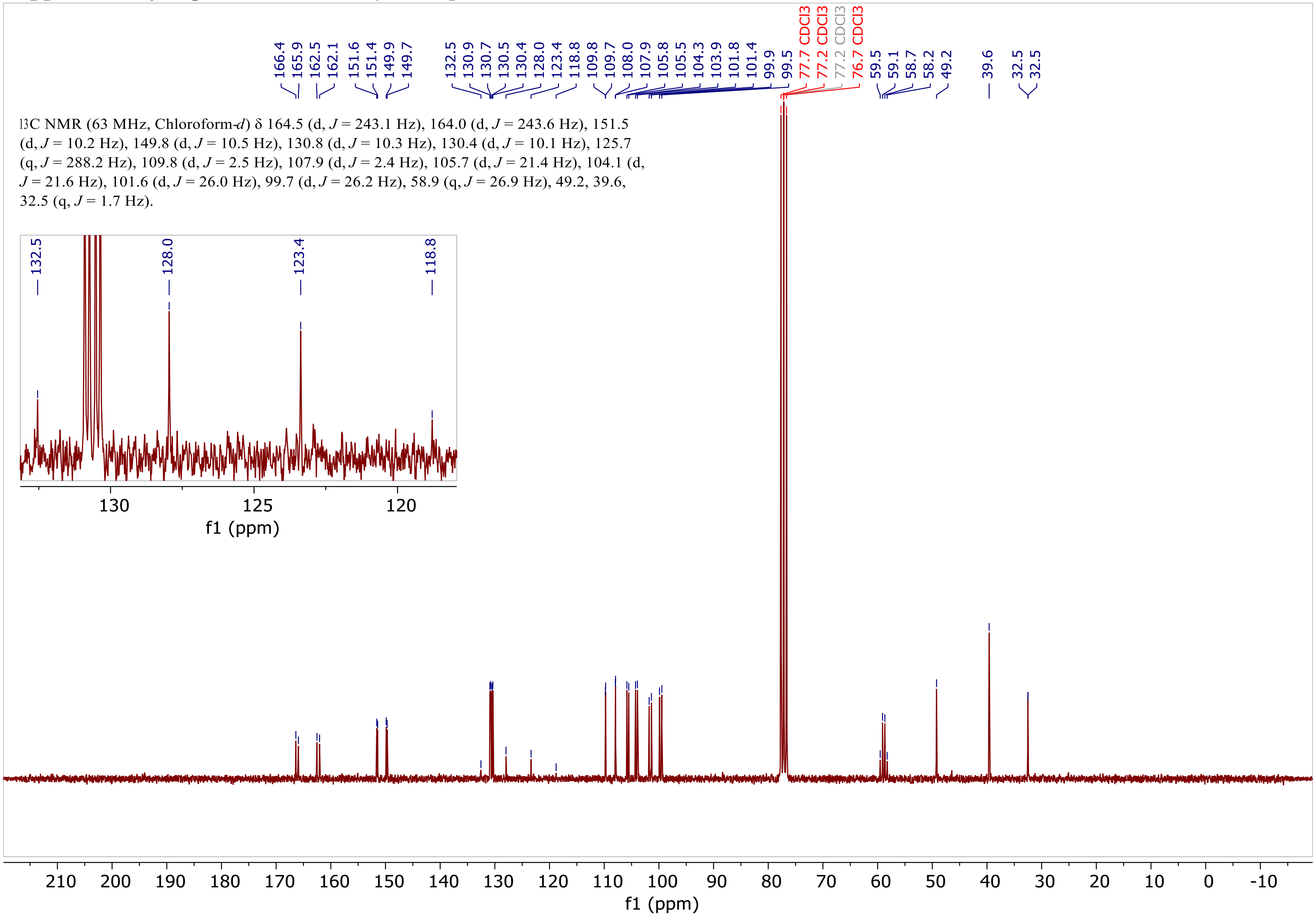

Supplementary Figure 95: *IR for compound* ***13***

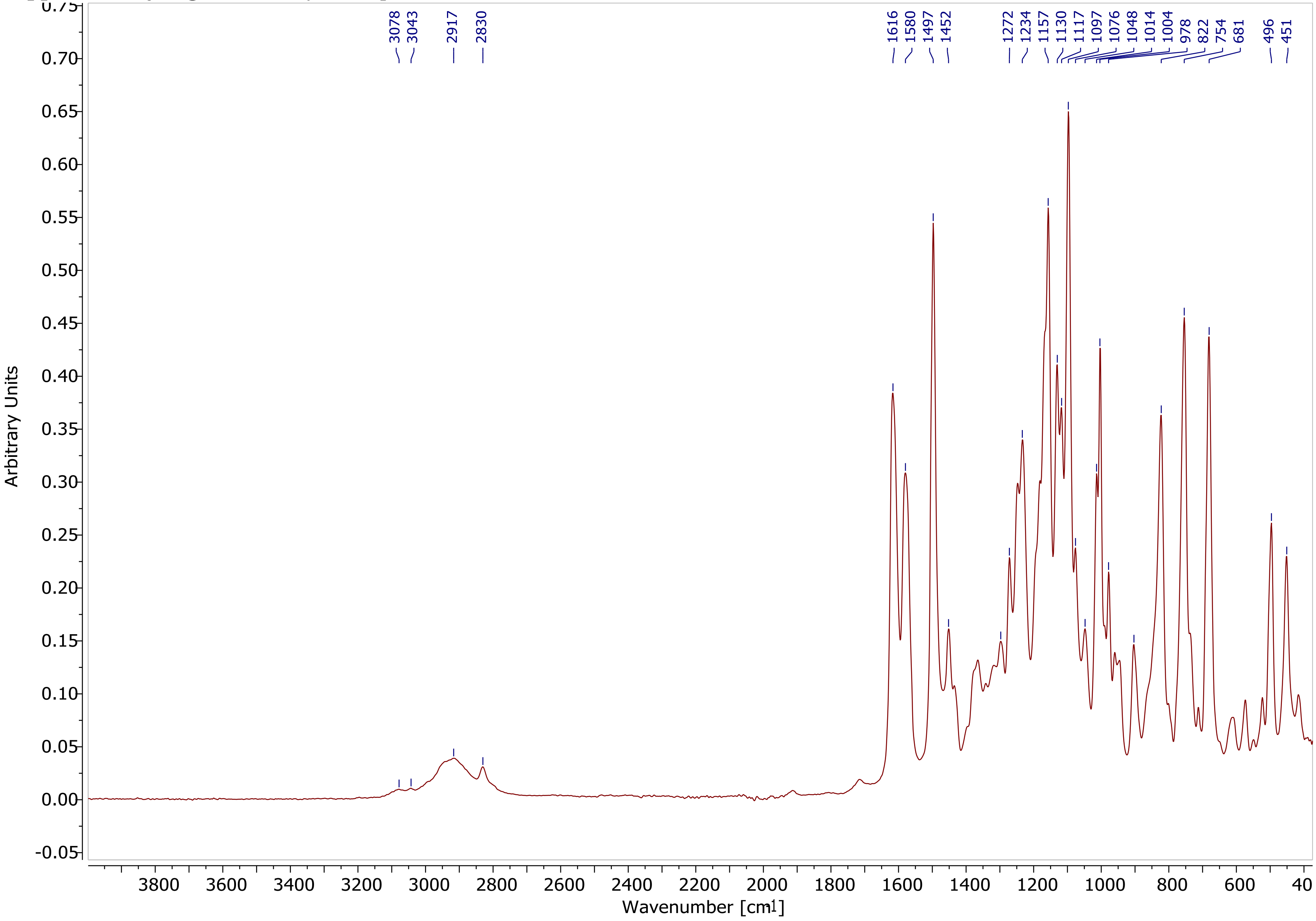

Supplementary Figure 96: *1H-NMR for compound* ***14***

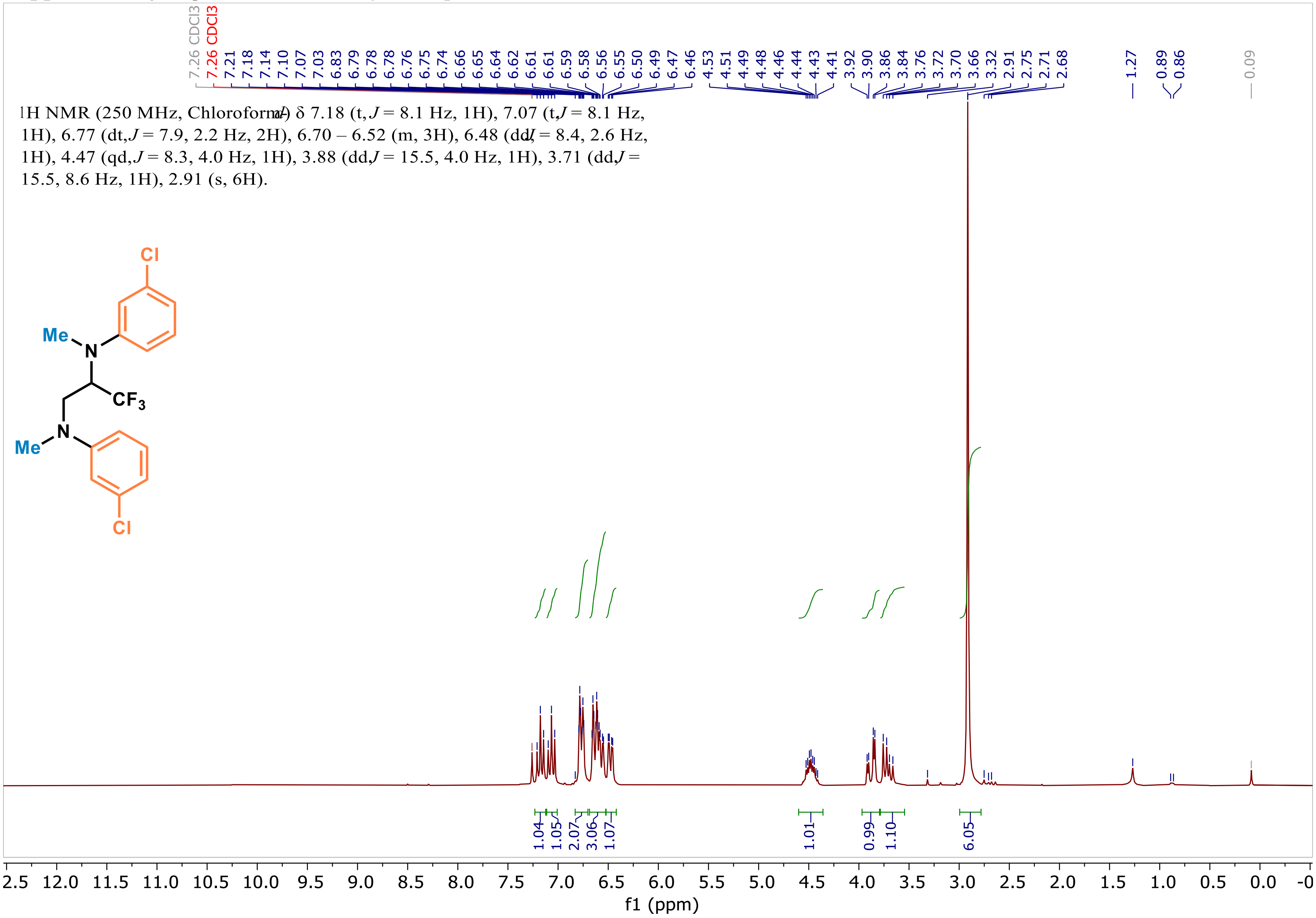

Supplementary Figure 97: ^{19}*F NMR for compound* ***14***

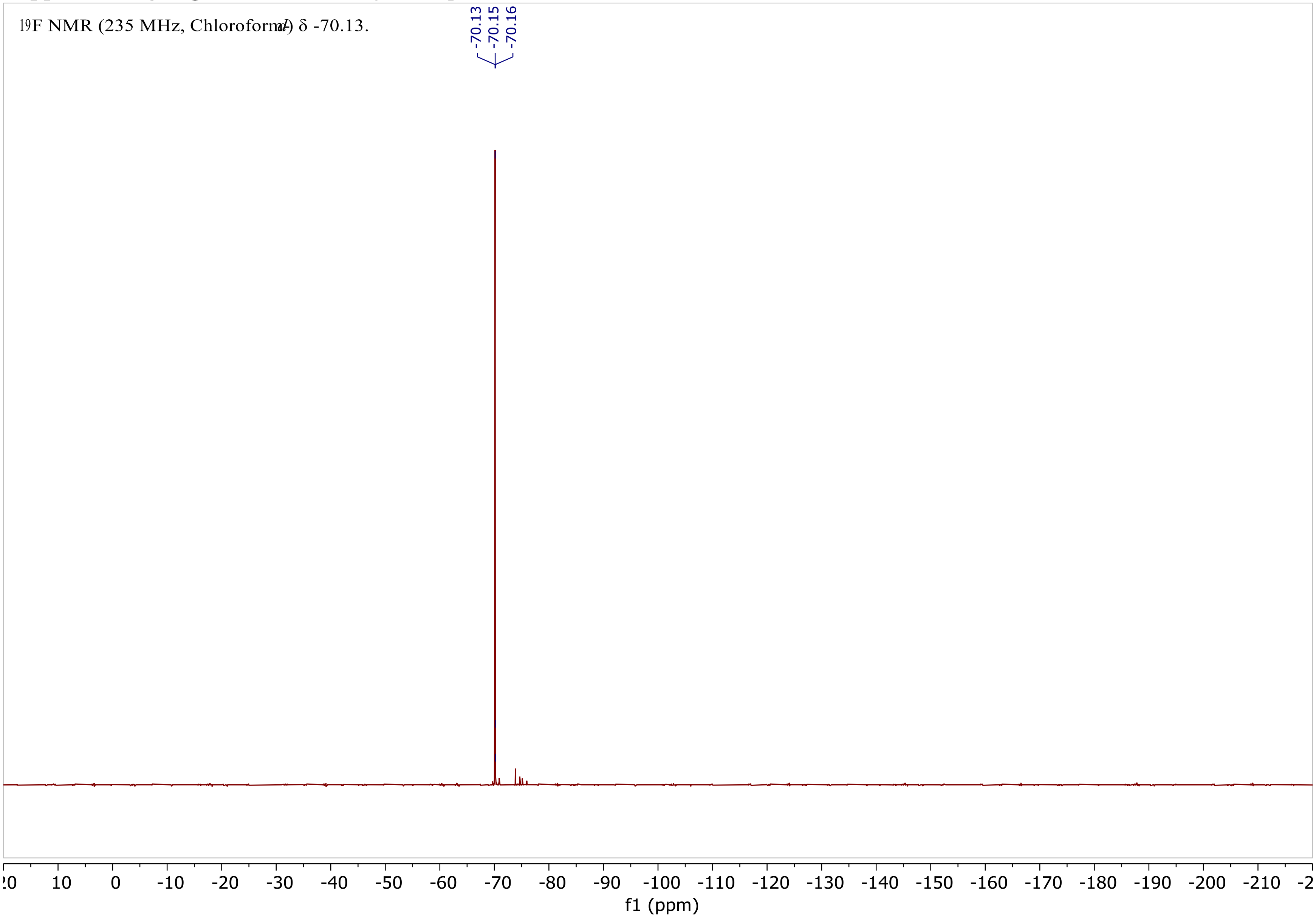

Supplementary Figure 98: *^{13}C NMR for compound* ***14***

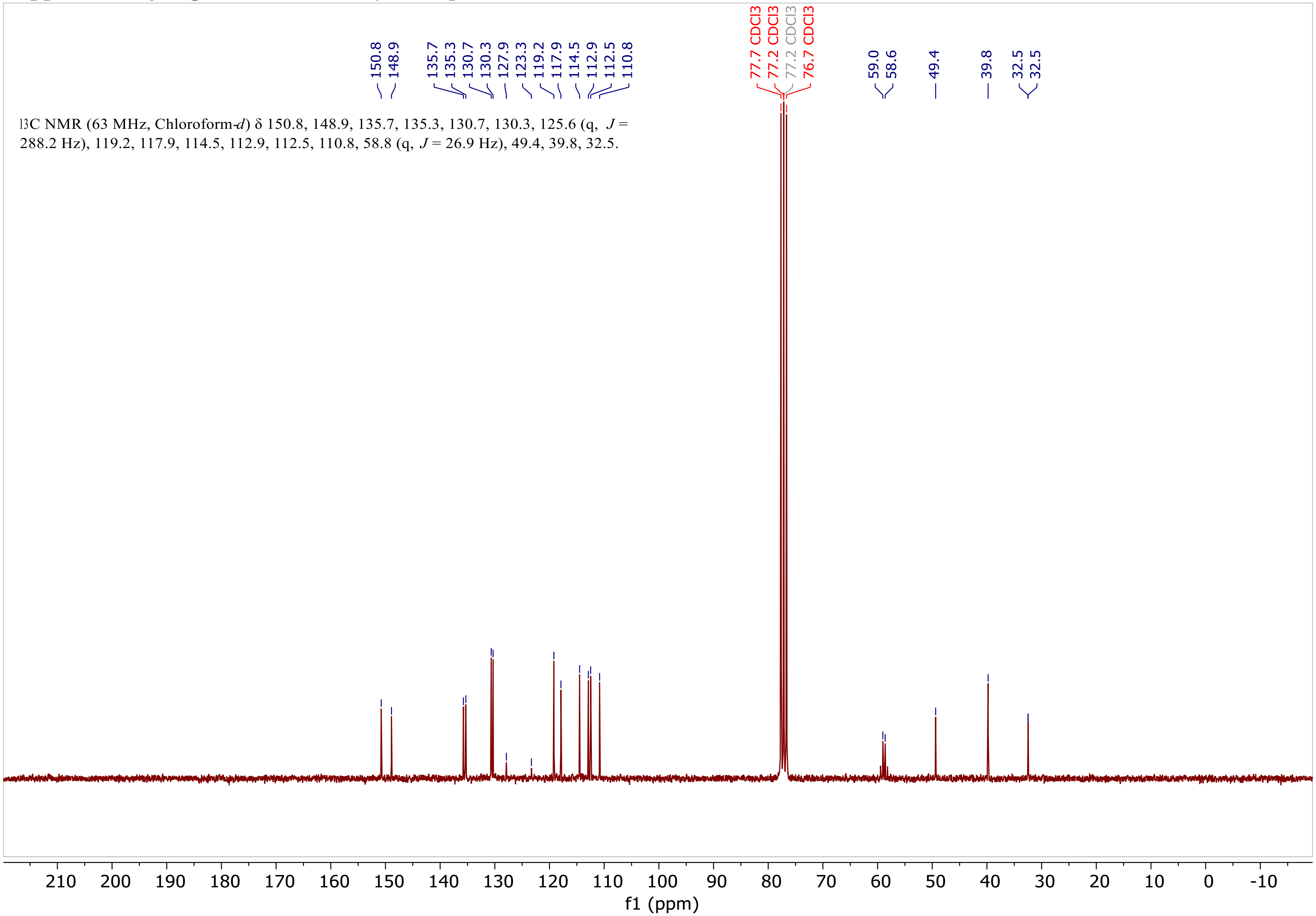

Supplementary Figure 99: *IR for compound* ***14***

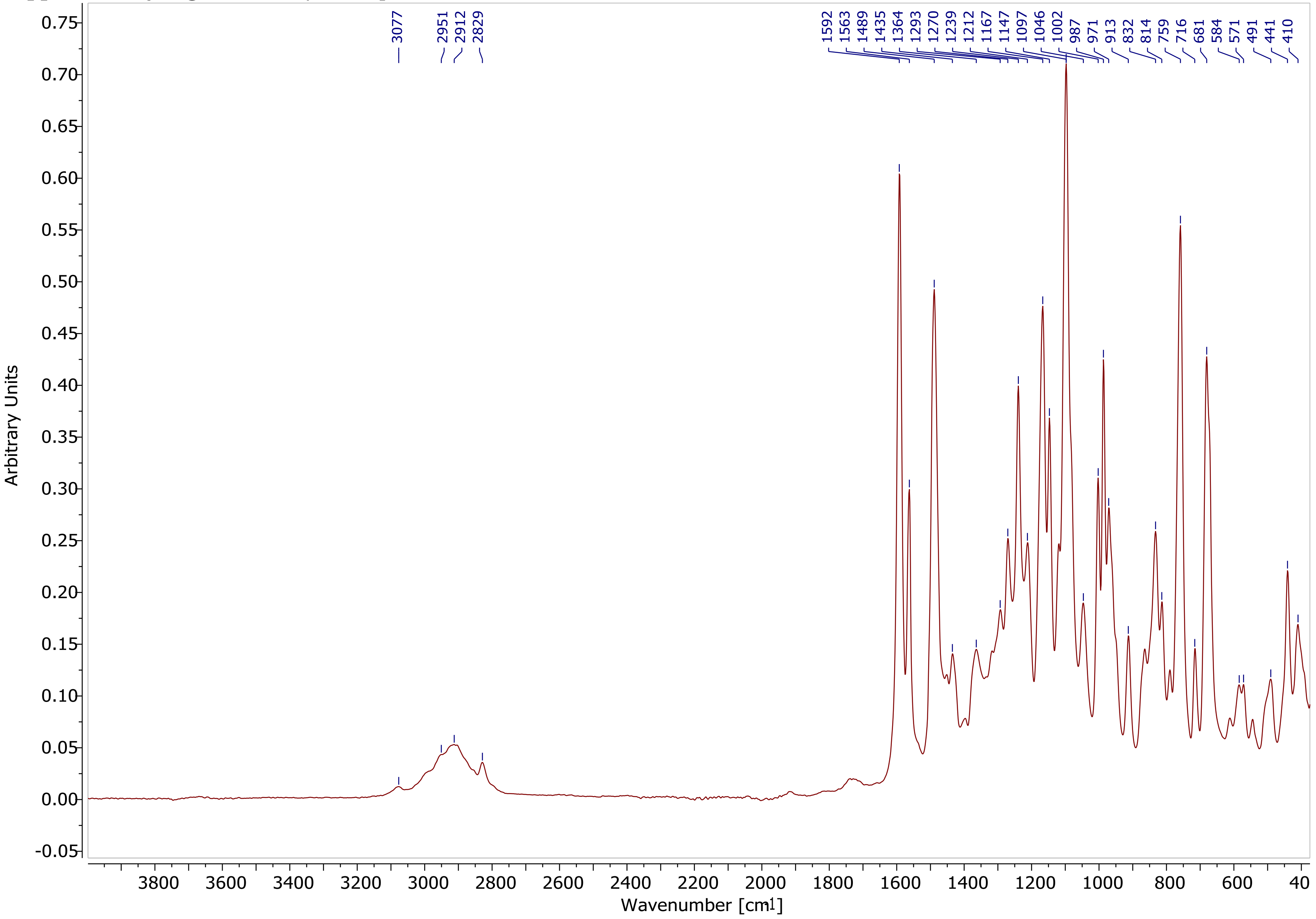

Supplementary Figure 100: *^{1}H-NMR for compound* ***15***

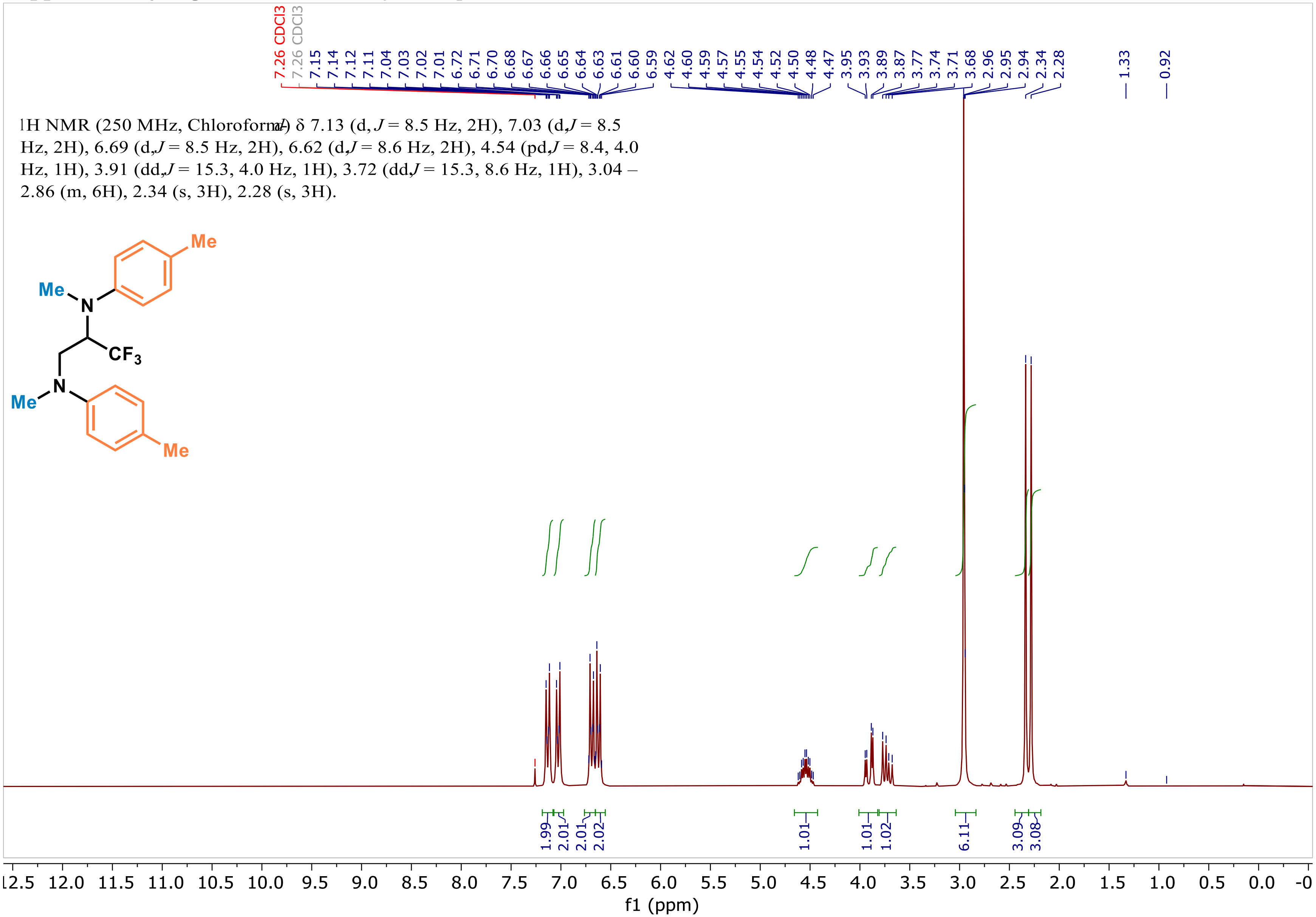

Supplementary Figure 101: *^{19}F NMR for compound **15***

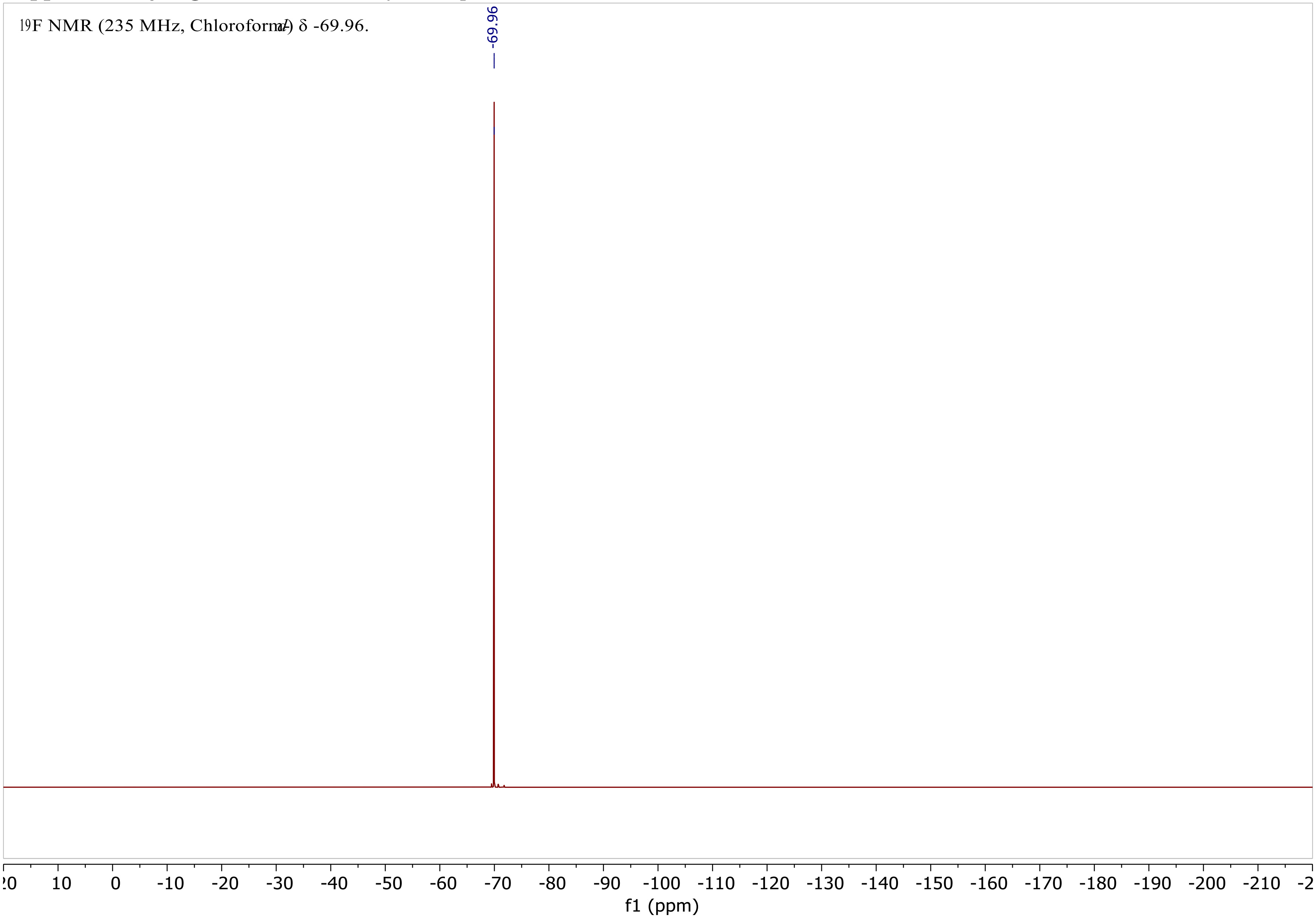

Supplementary Figure 102: ^{13}*C NMR for compound* ***15***

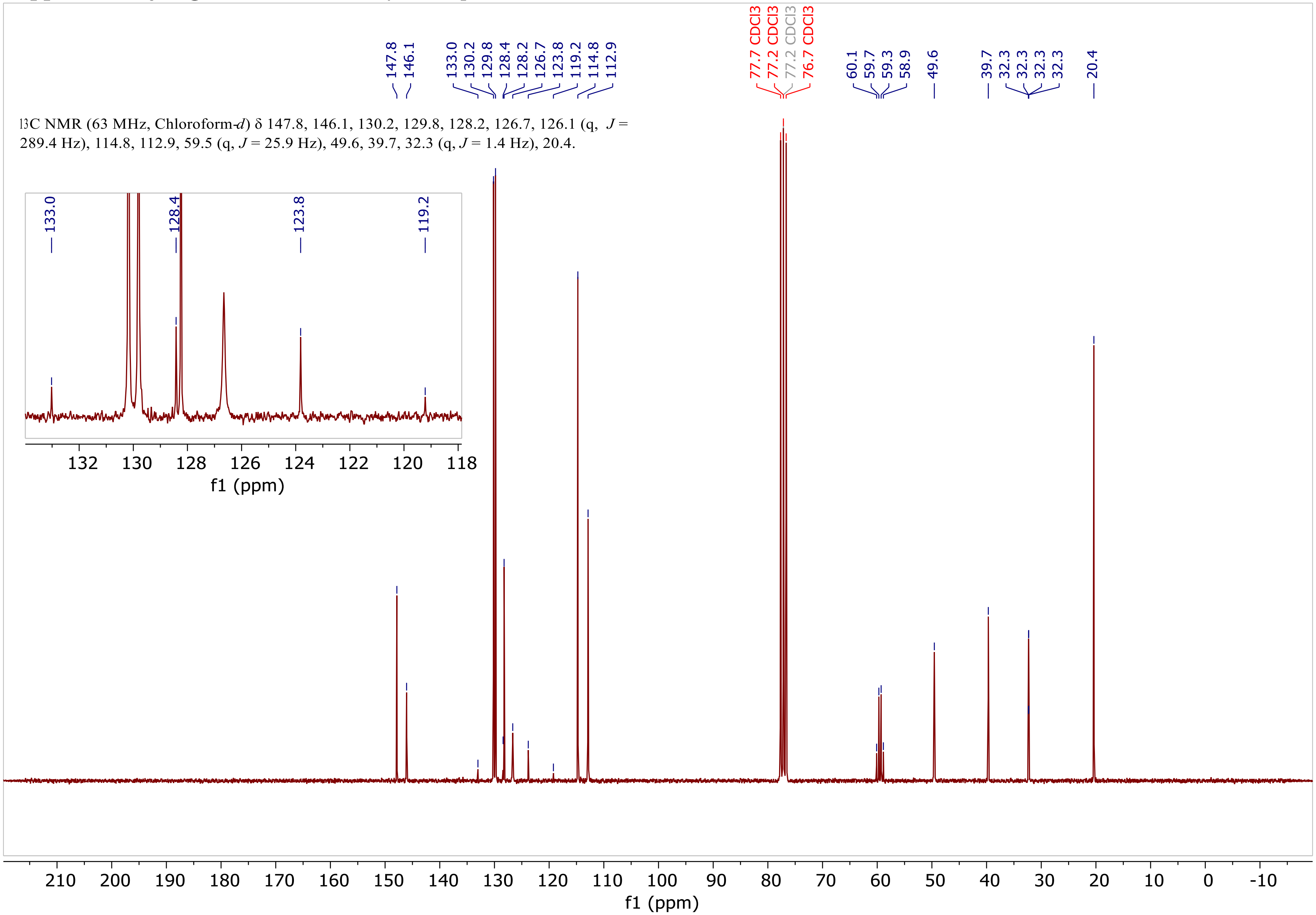

Supplementary Figure 103: *IR for compound **15***

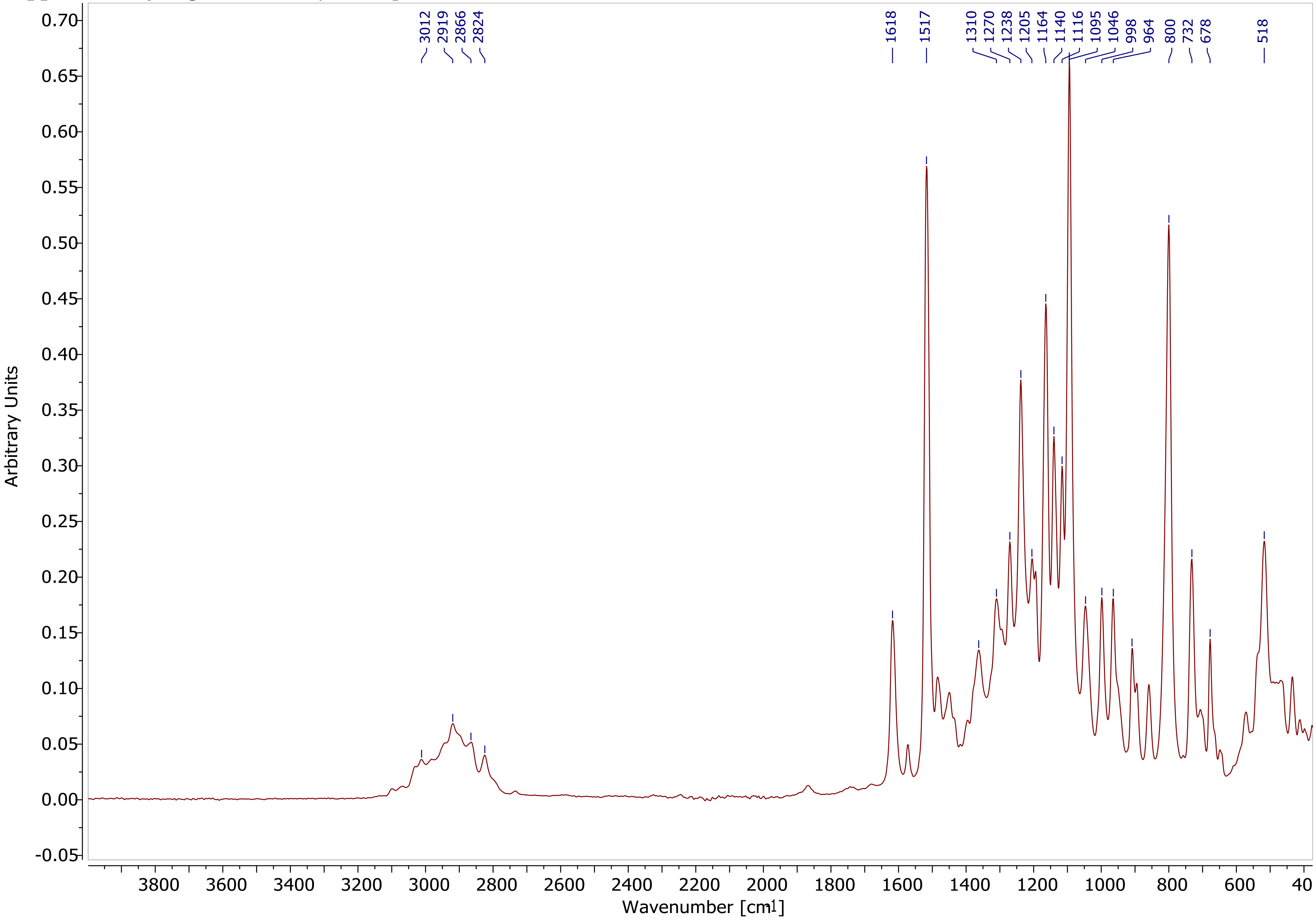

Supplementary Figure 104: *^{1}H-NMR for compound* ***16***

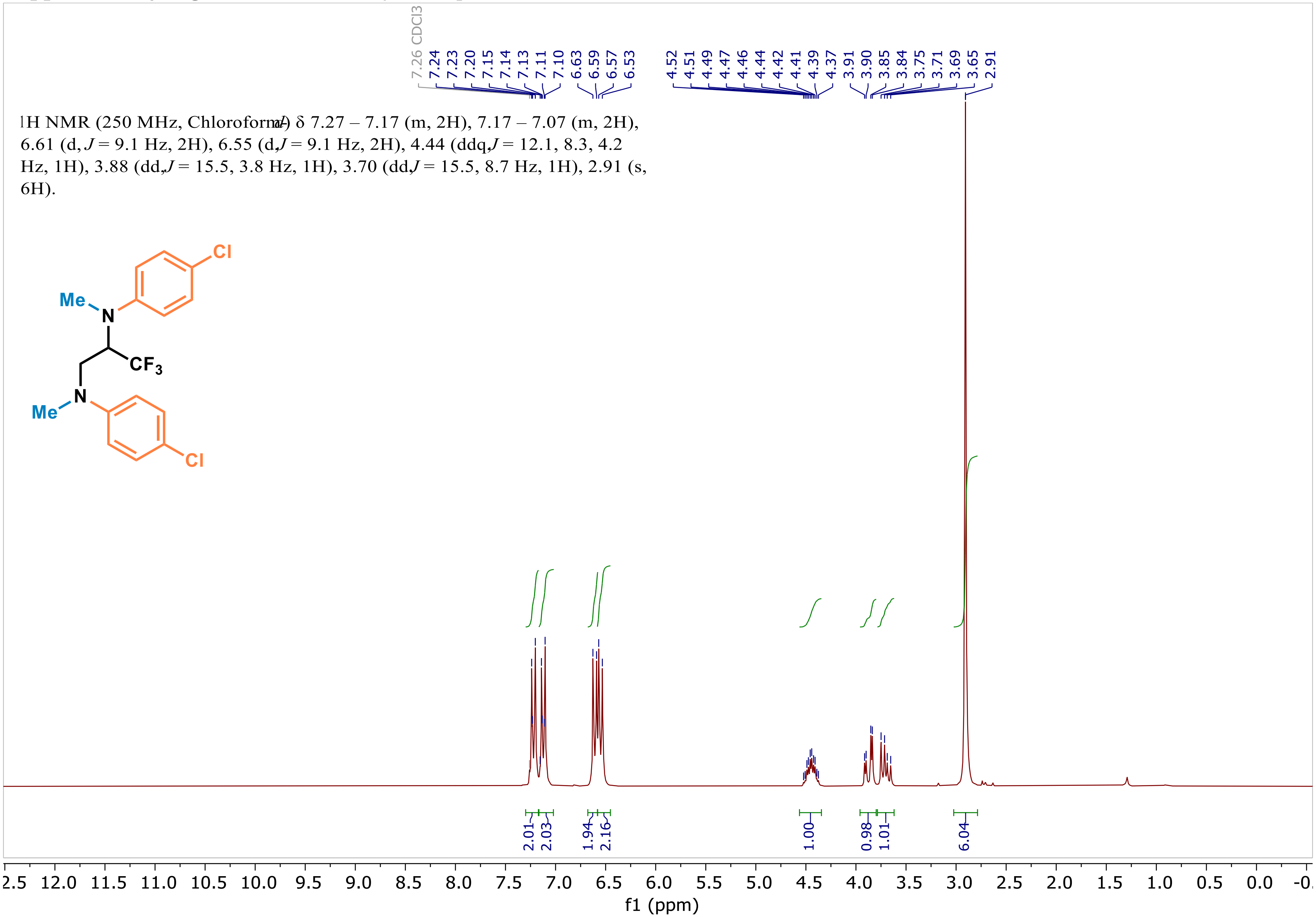

Supplementary Figure 105: *^{19}F NMR for compound* ***16***

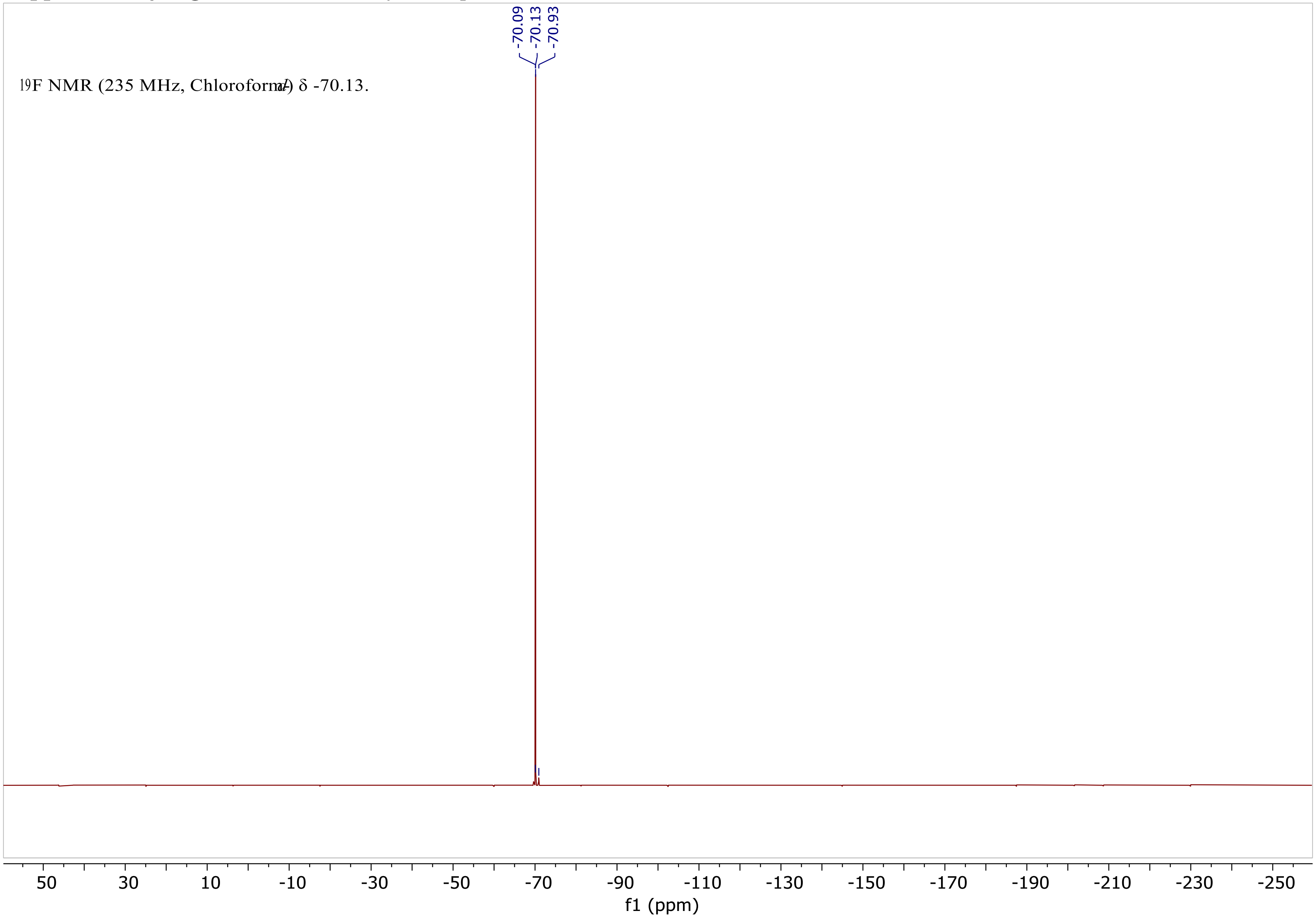

Supplementary Figure 106: *^{13}C NMR for compound* ***16***

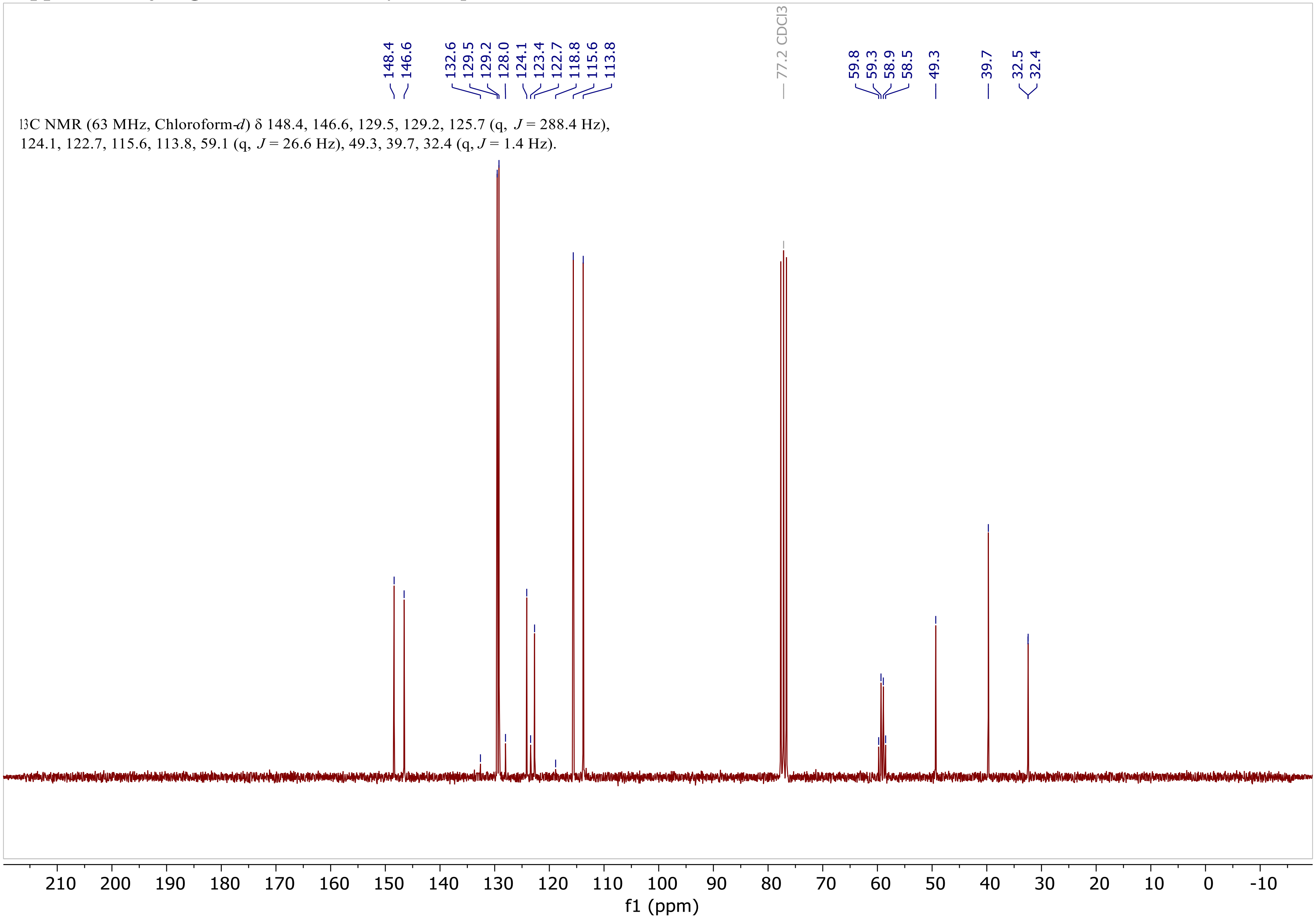

Supplementary Figure 107: *IR for compound* ***16***

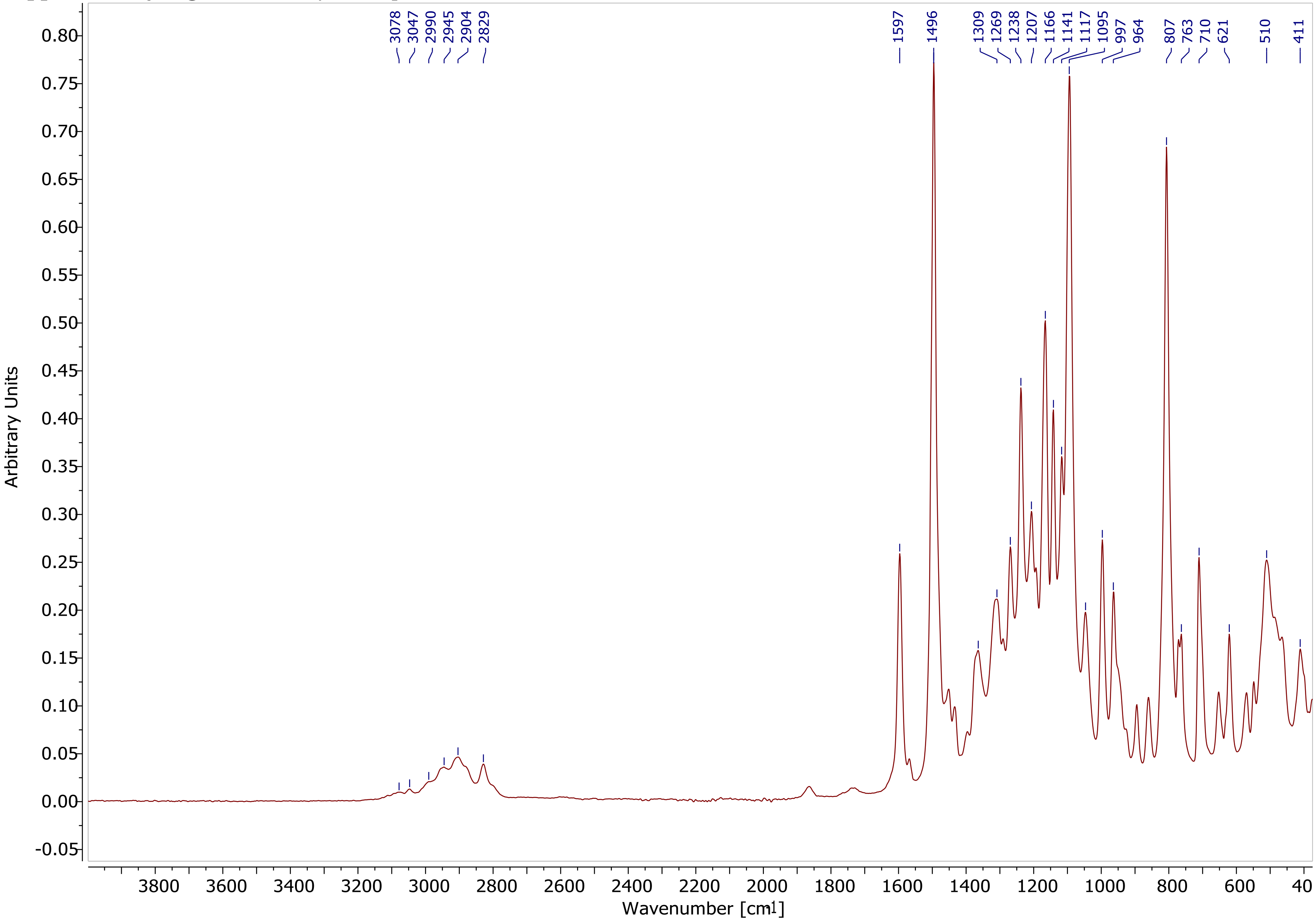

Supplementary Figure 108: *^{1}H-NMR for compound* ***17***

1H NMR (250 MHz, Chloroform-*d*) δ 7.25 (d, *J* = 8.8 Hz, 2H), 7.16 (d, *J* = 8.7 Hz, 2H), 6.46 (d, *J* = 8.9 Hz, 2H), 6.40 (d, *J* = 8.9 Hz, 2H), 4.34 (td, *J* = 8.2, 3.7 Hz, 1H), 3.78 (dd, *J* = 15.4, 3.7 Hz, 1H), 3.60 (dd, *J* = 15.5, 8.7 Hz, 1H), 2.80 (s, 6H).

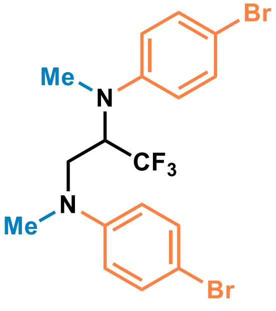

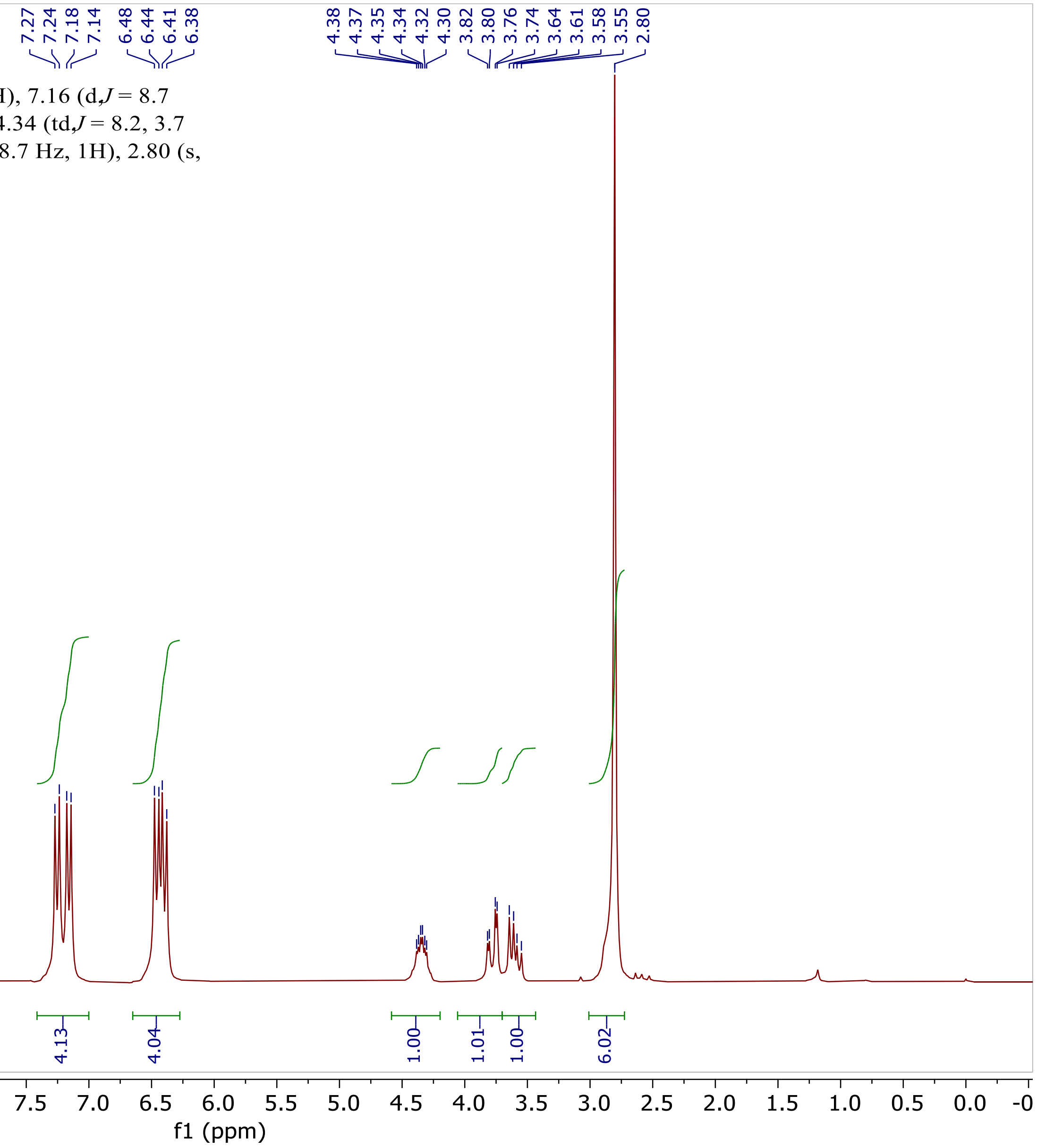

Supplementary Figure 109: *^{19}F NMR for compound **17***

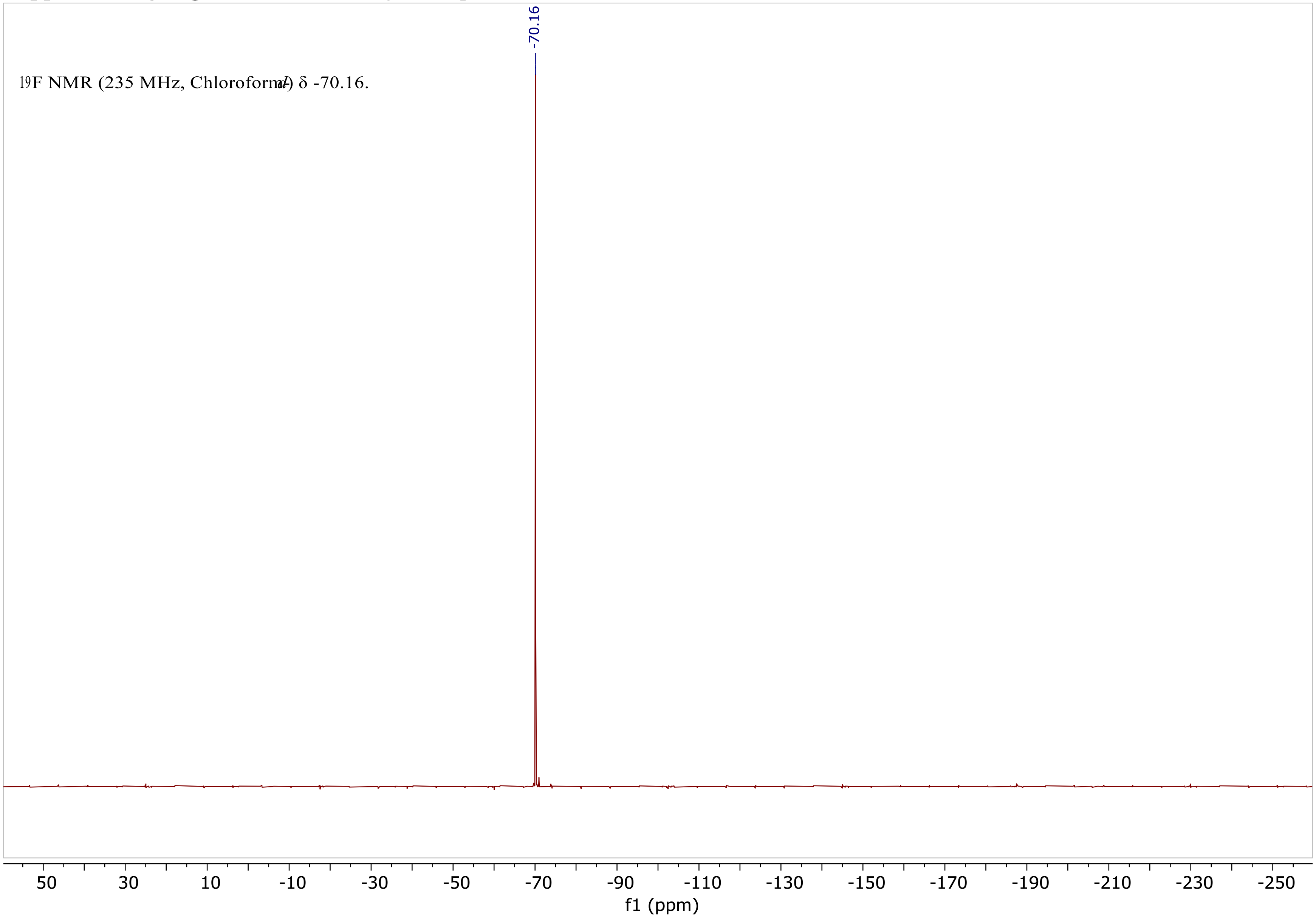

Supplementary Figure 110: *^{13}C NMR for compound **17***

13C NMR (63 MHz, Chloroform-*d*) δ 148.8, 146.8, 132.4, 132.1, 123.3 (q, *J* = 289.3, 288.6 Hz), 116.0, 114.2, 111.4, 109.9, 58.9 (q, *J* = 26.7 Hz), 49.3, 39.7, 32.4.

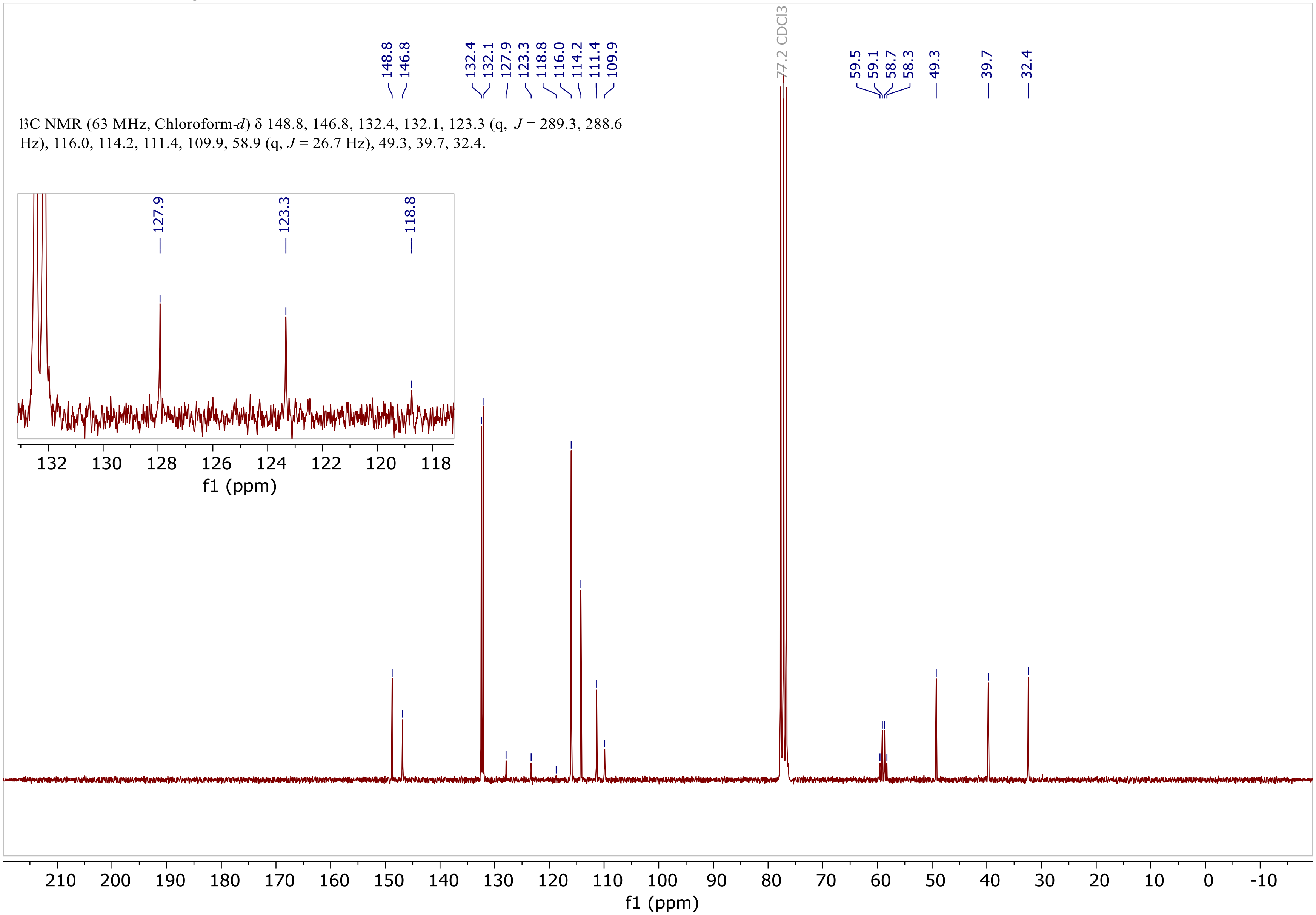

Supplementary Figure 111: *IR for compound* ***17***

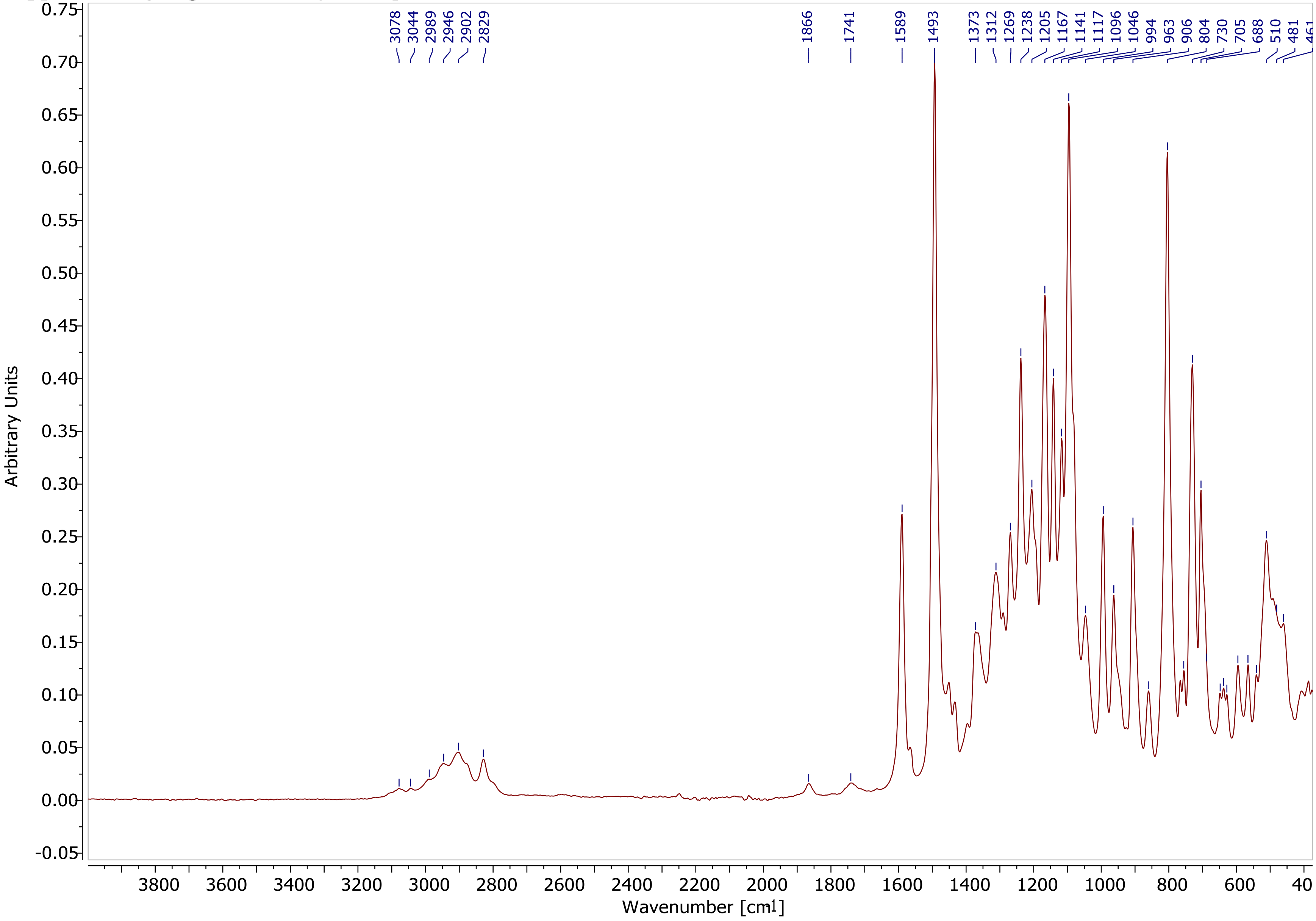

Supplementary Figure 112: *^{1}H-NMR for compound* ***18***

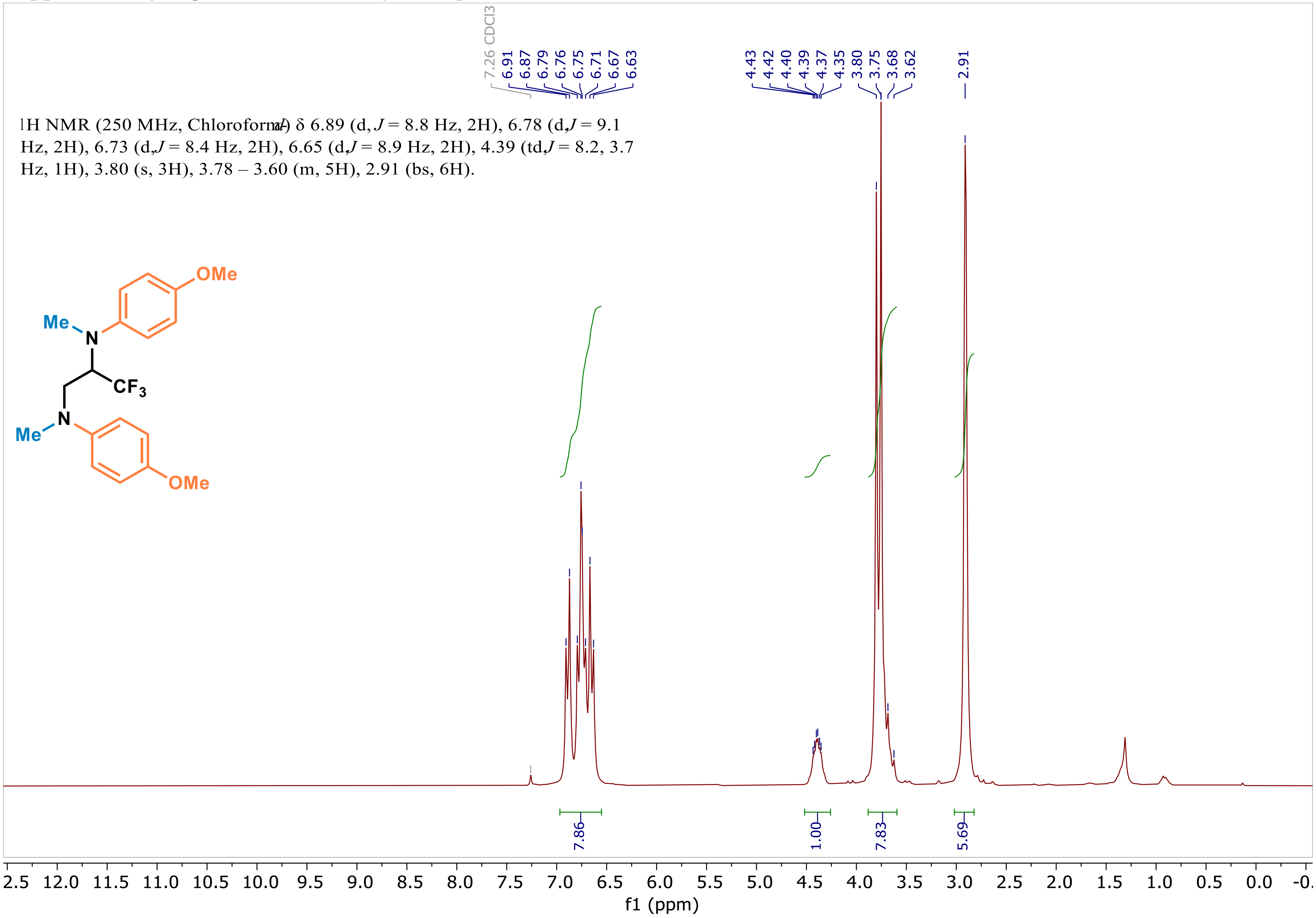

Supplementary Figure 113: *^{19}F NMR for compound* ***18***

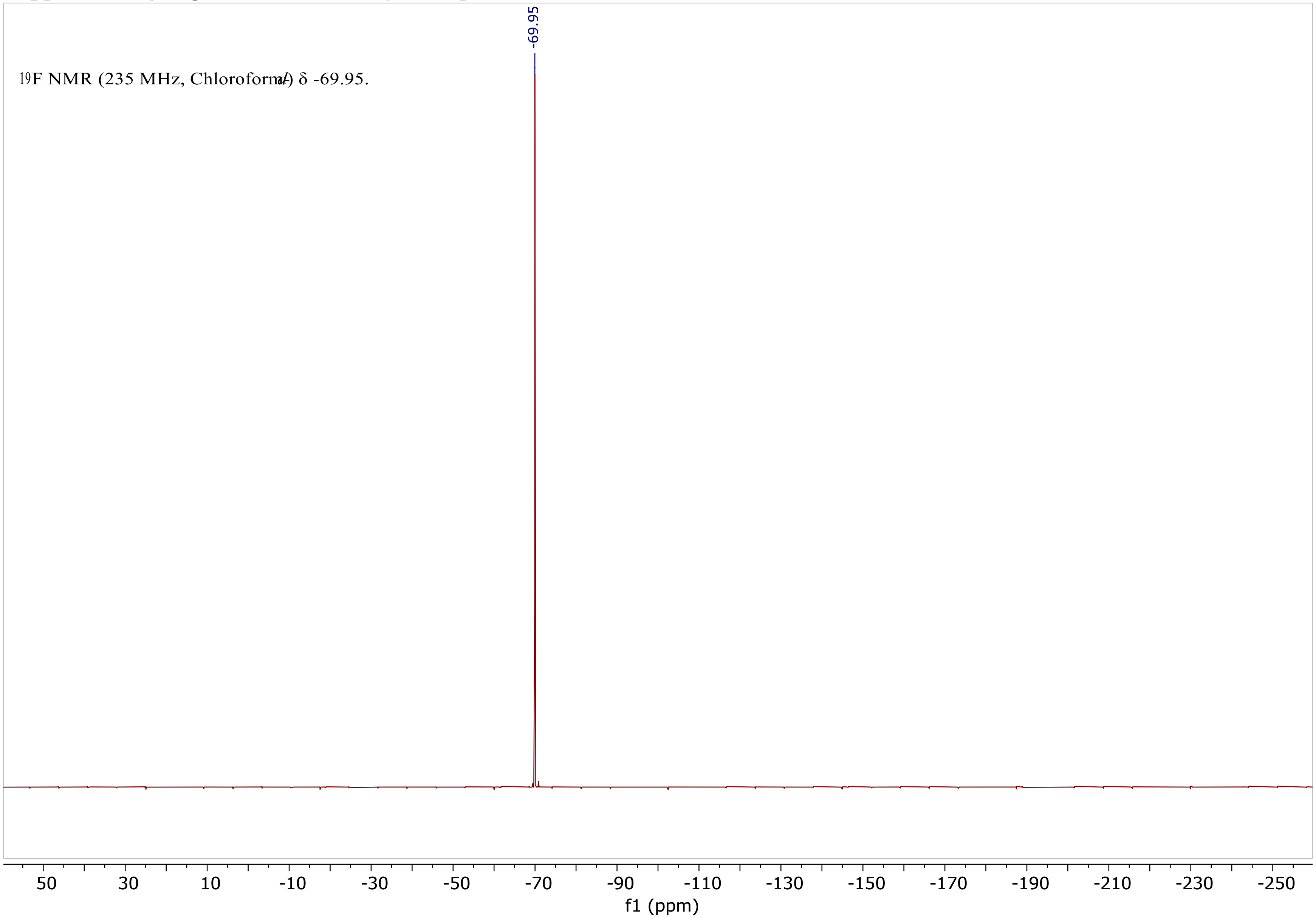

Supplementary Figure 114: *^{13}C NMR for compound* ***18***

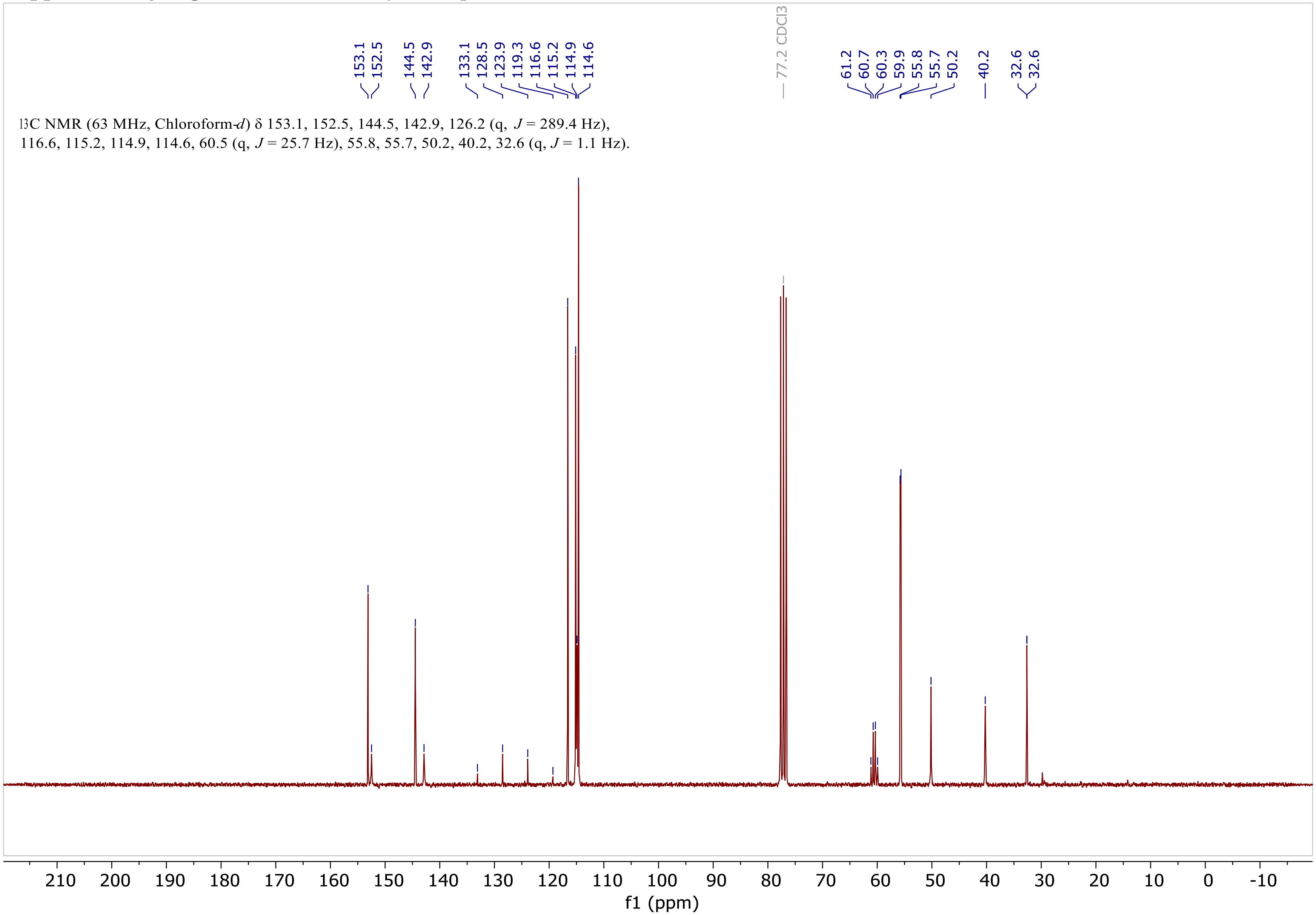

Supplementary Figure 115: *IR for compound* ***18***

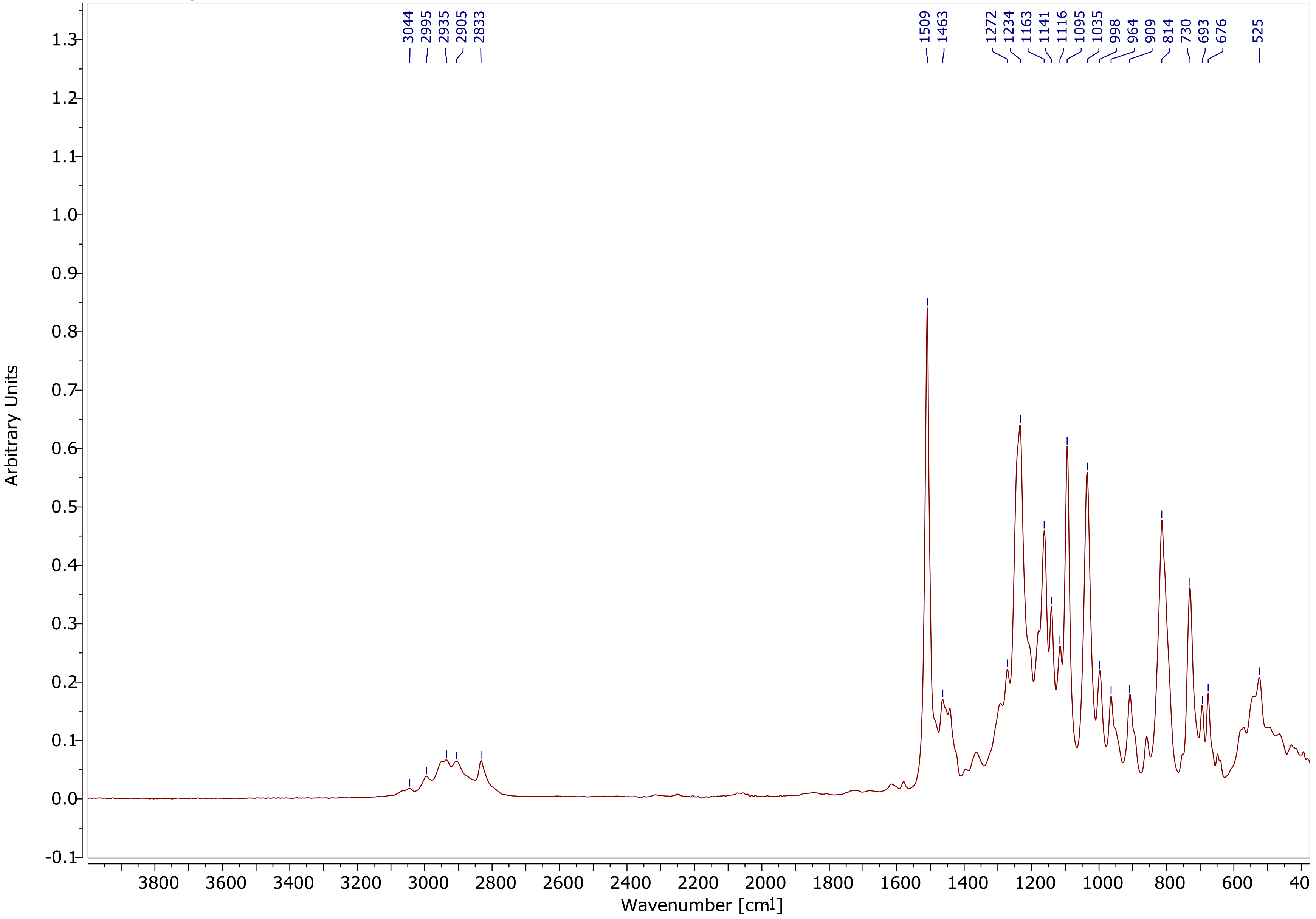

Supplementary Figure 116: *¹H-NMR for compound **19***

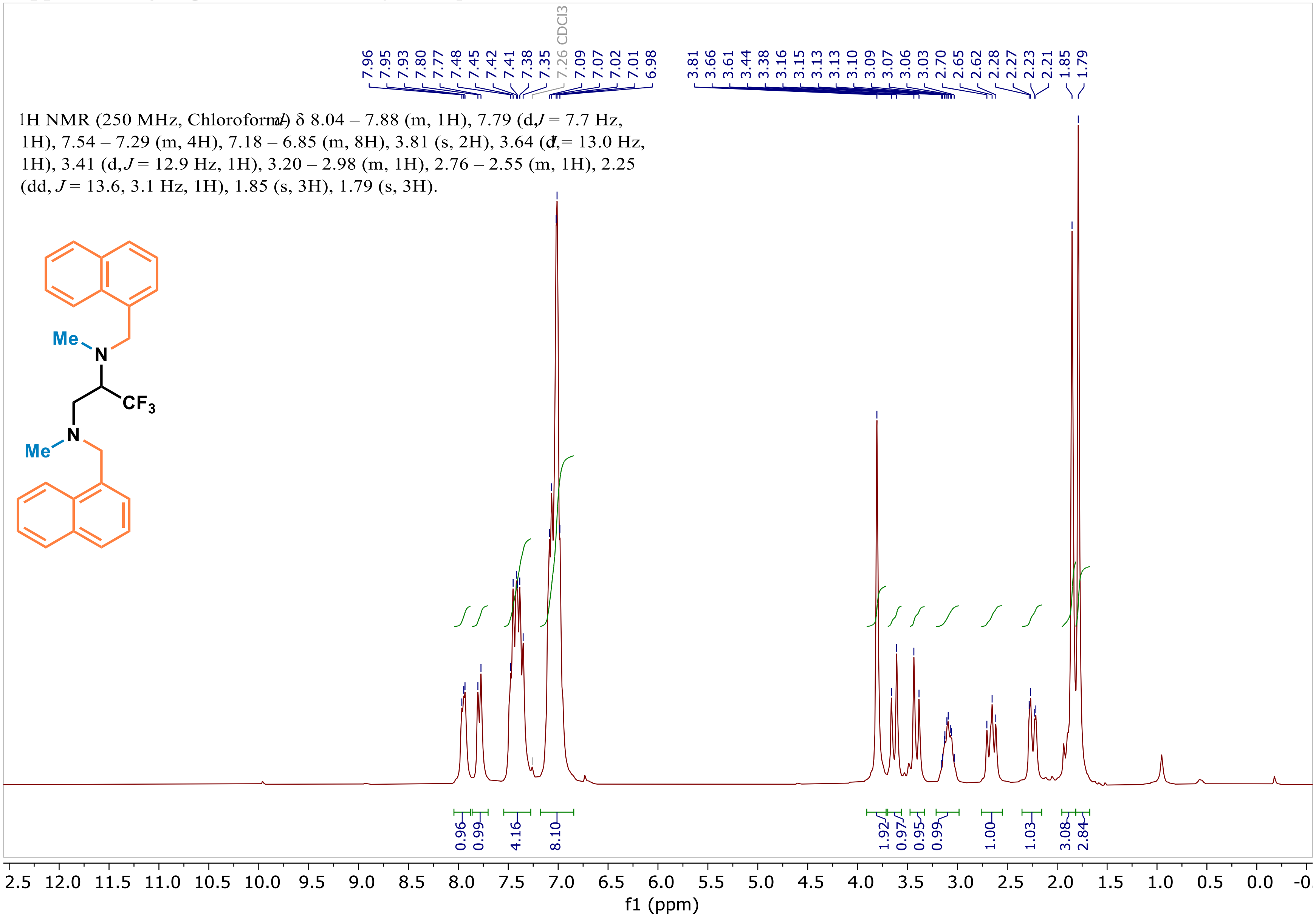

Supplementary Figure 117: *^{19}F NMR for compound* ***19***

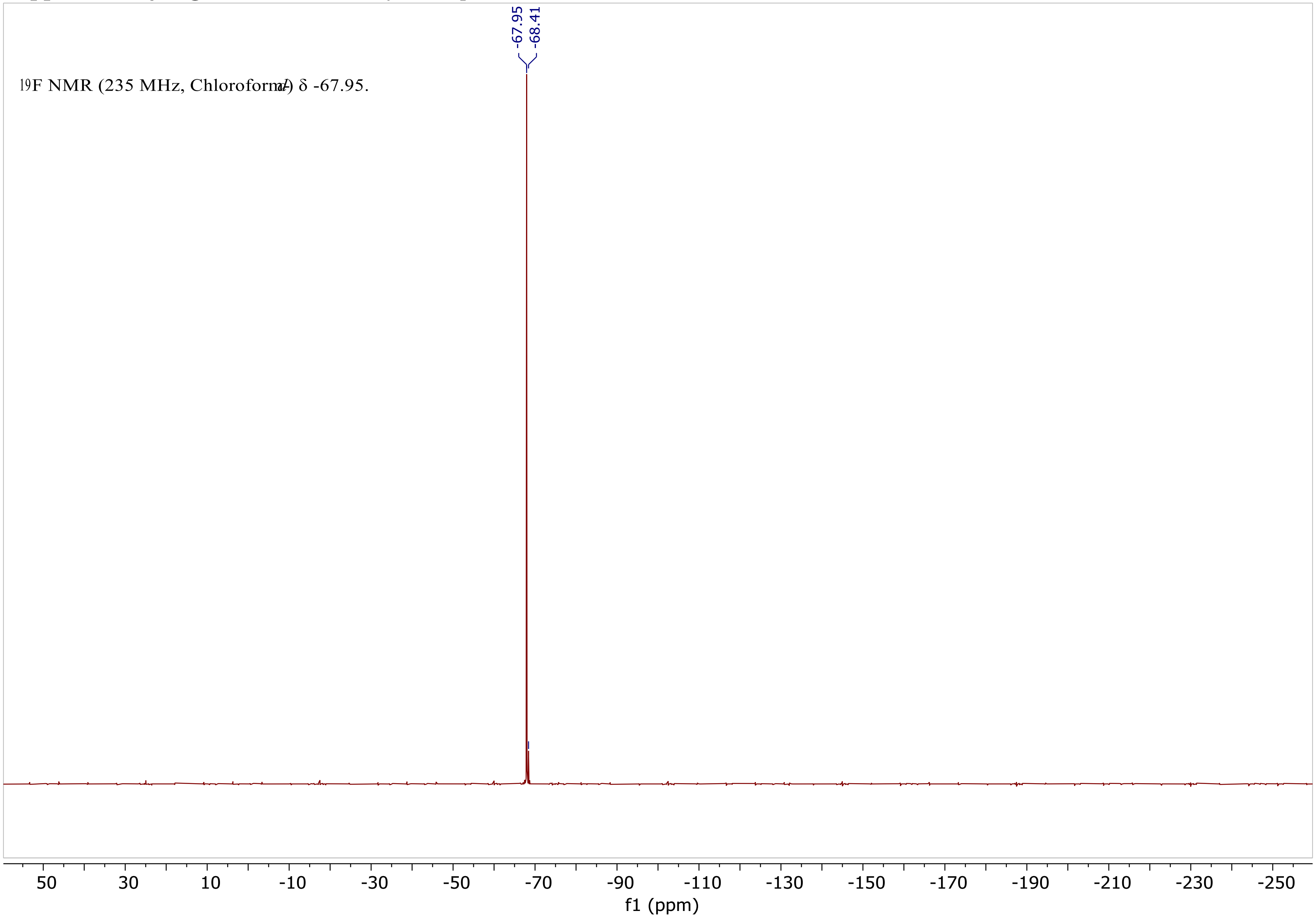

Supplementary Figure 118: *^{13}C NMR for compound* ***19***

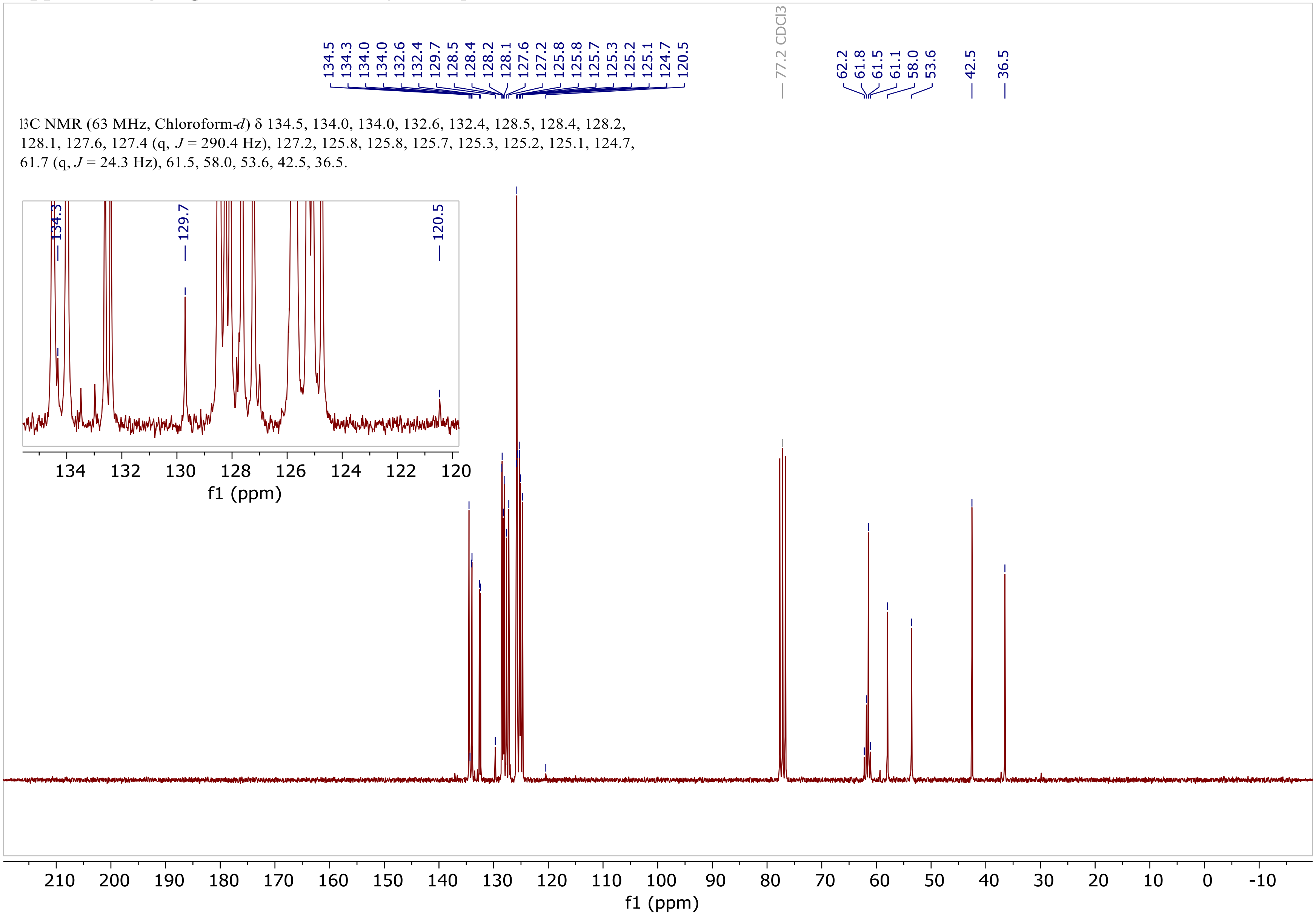

Supplementary Figure 119: *IR for compound* ***19***

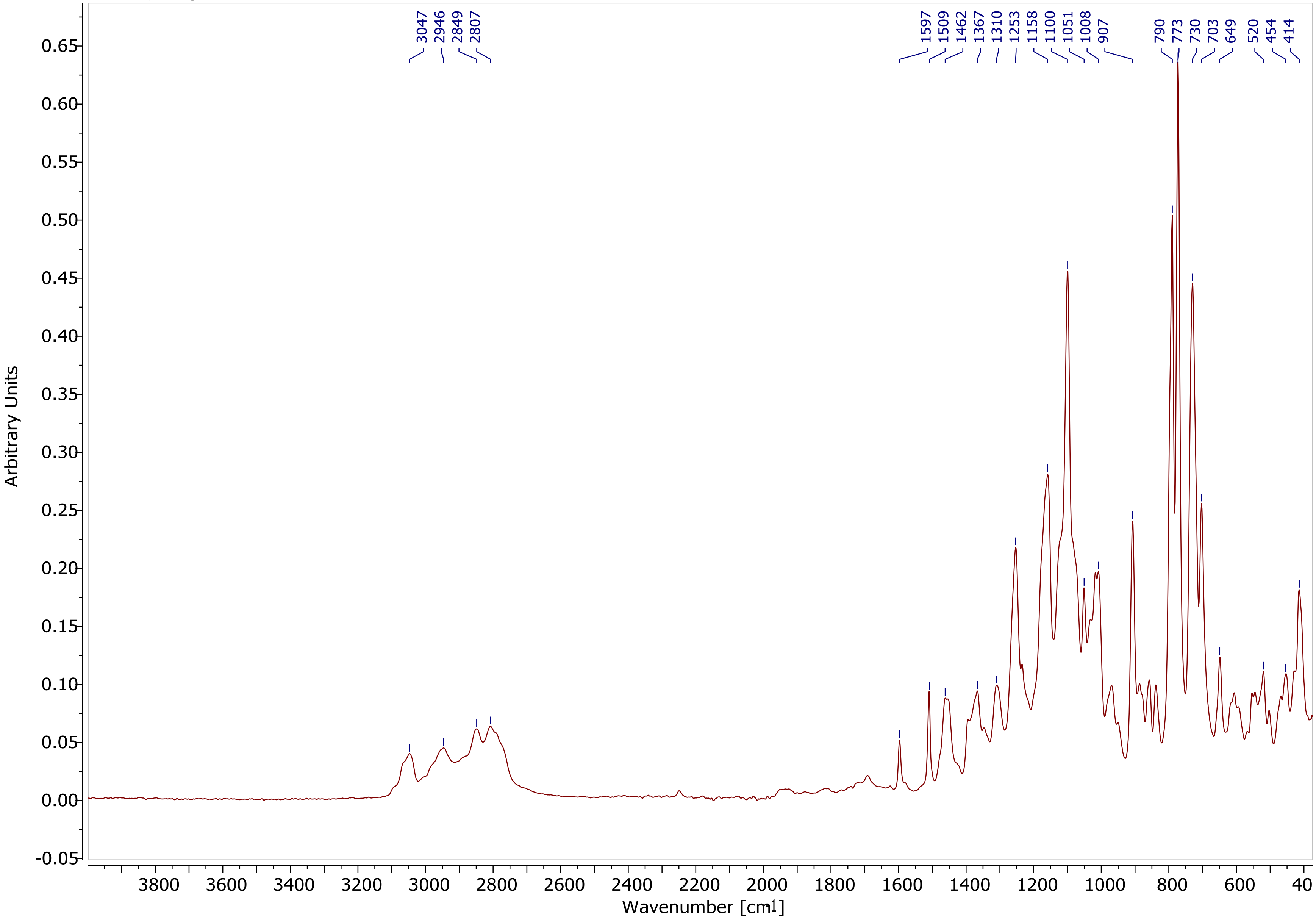

Supplementary Figure 120: *^{1}H-NMR for compound* ***20***

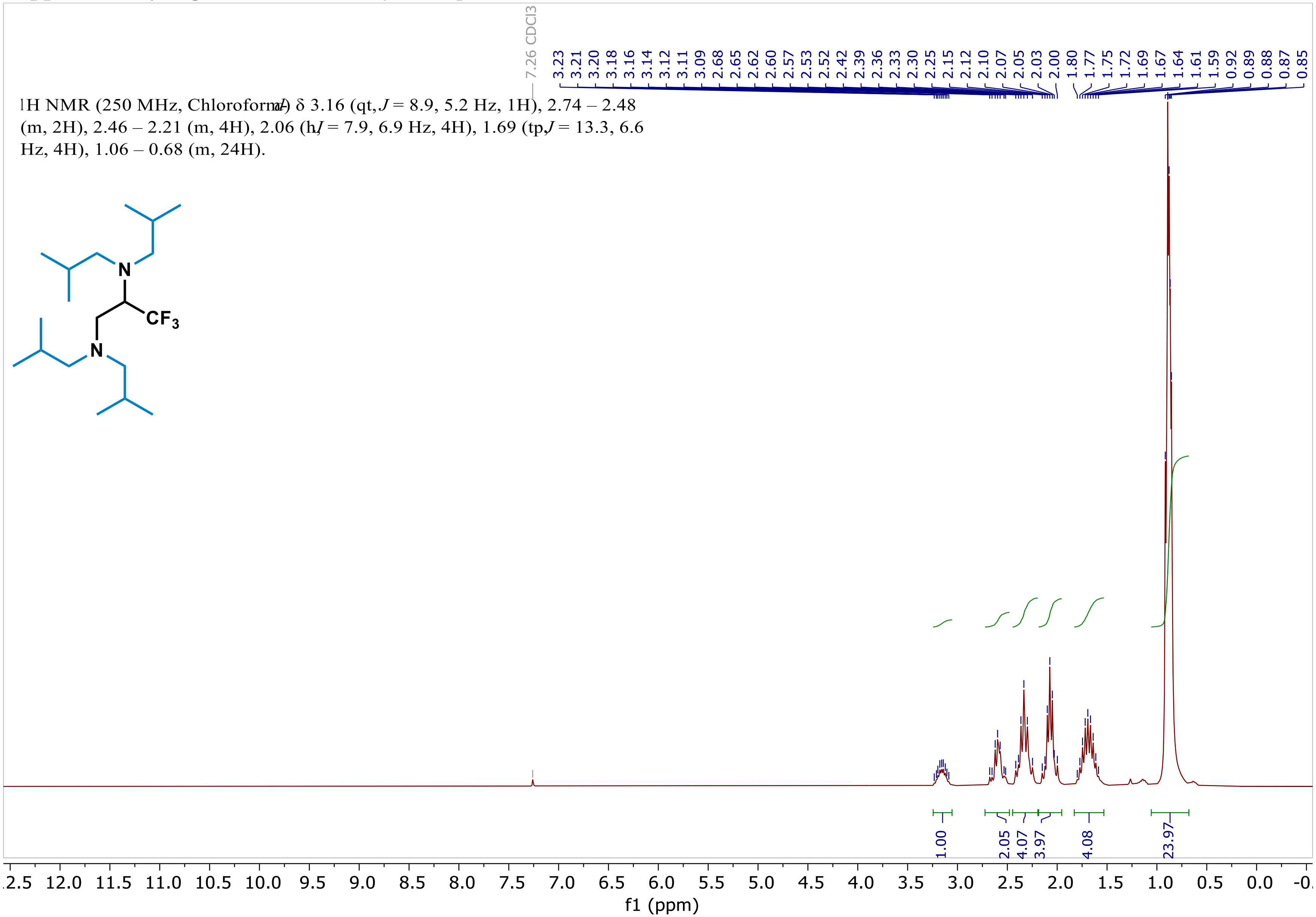

Supplementary Figure 121: *^{19}F NMR for compound* ***20***

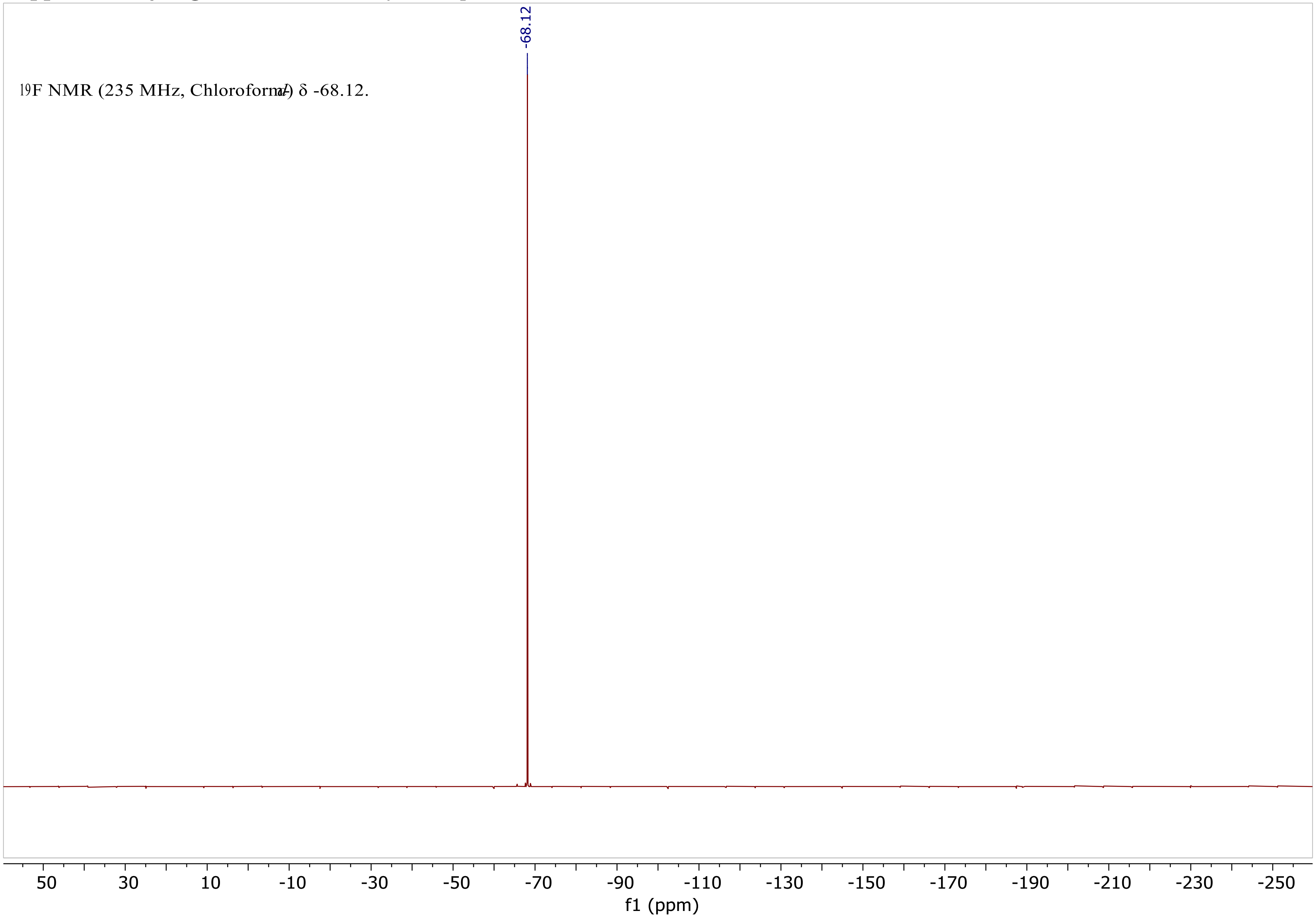

Supplementary Figure 122: *^{13}C NMR for compound* ***20***

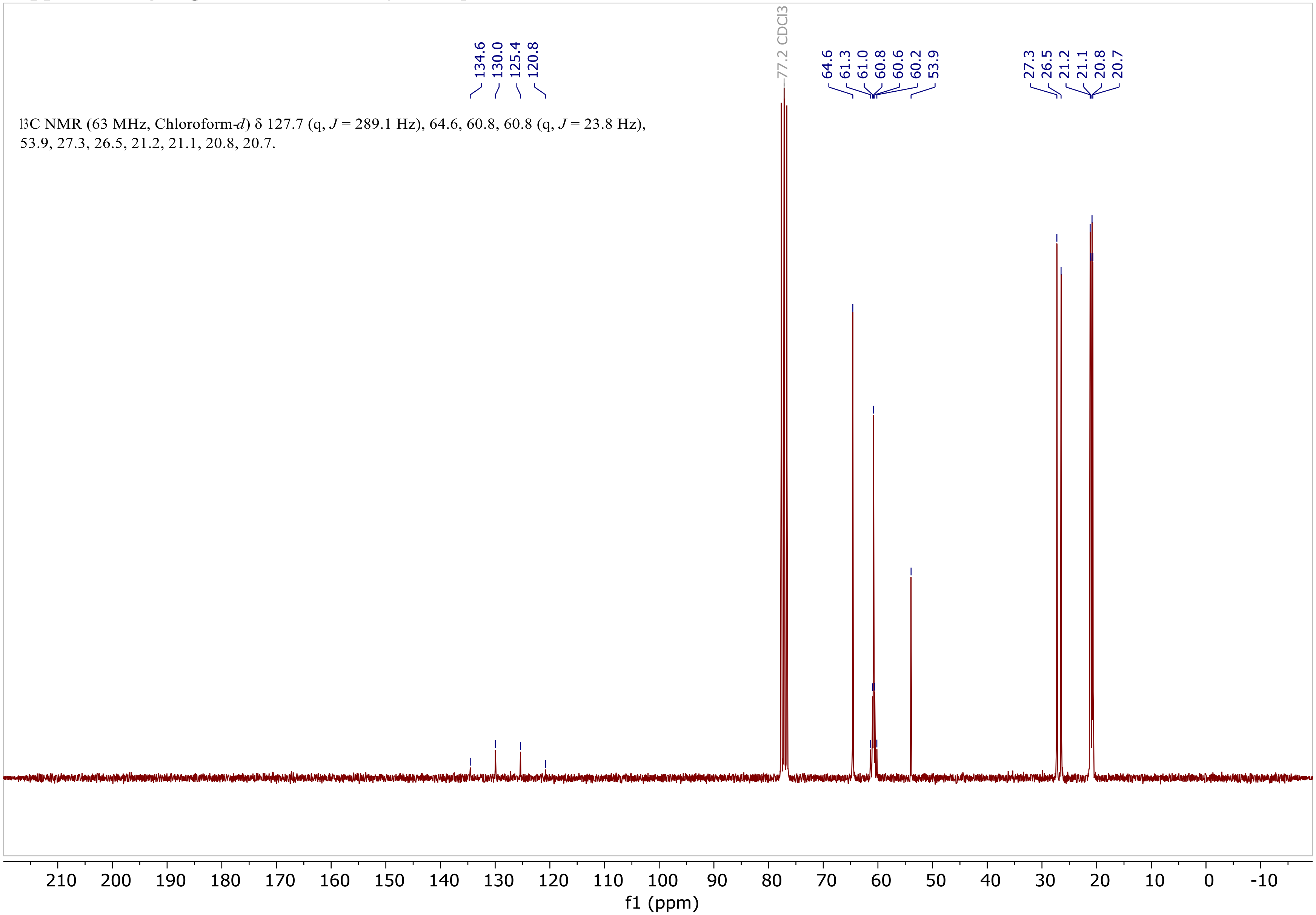

Supplementary Figure 123: *IR for compound **20***

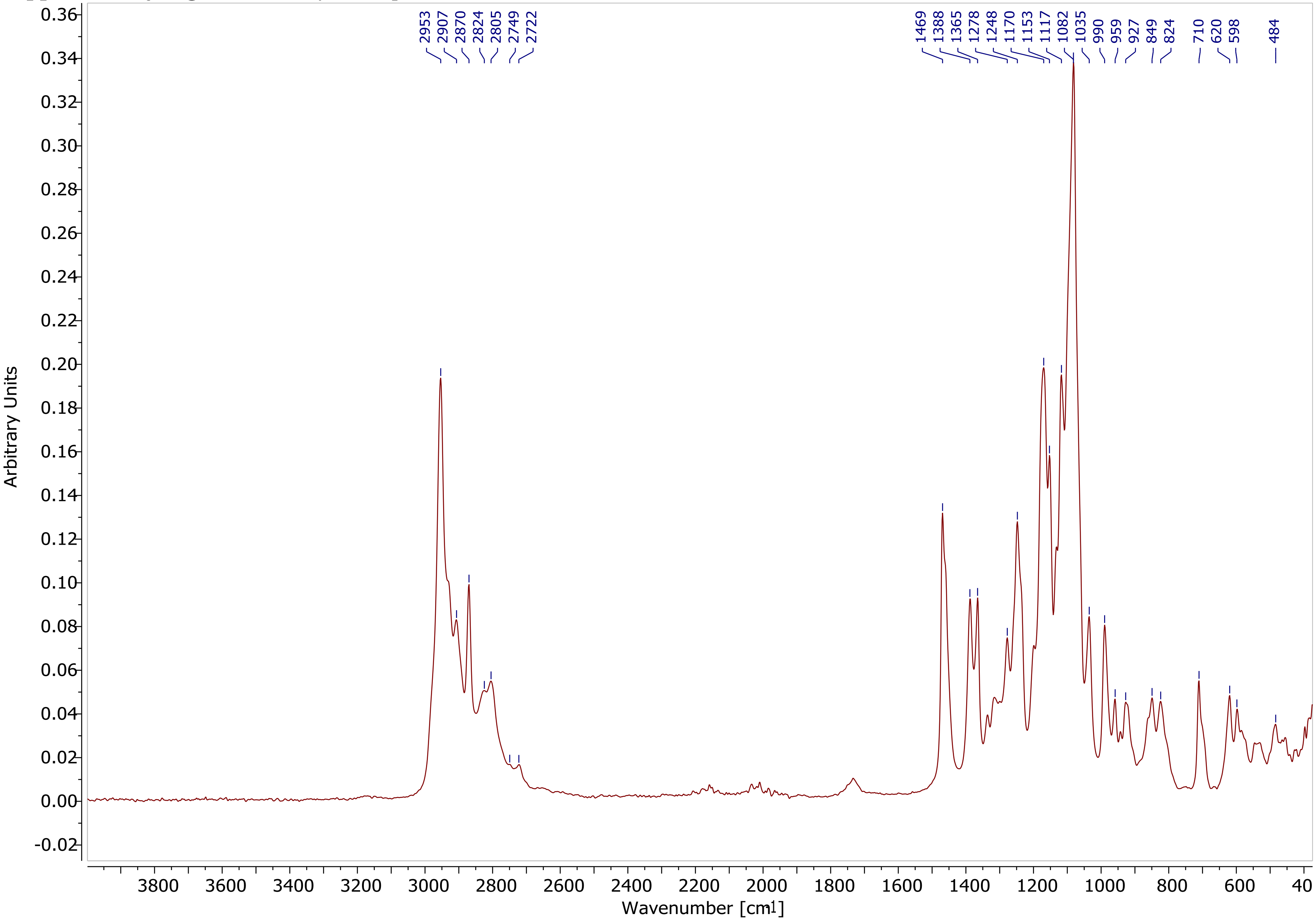

Supplementary Figure 124: *^{1}H-NMR for compound* ***21***

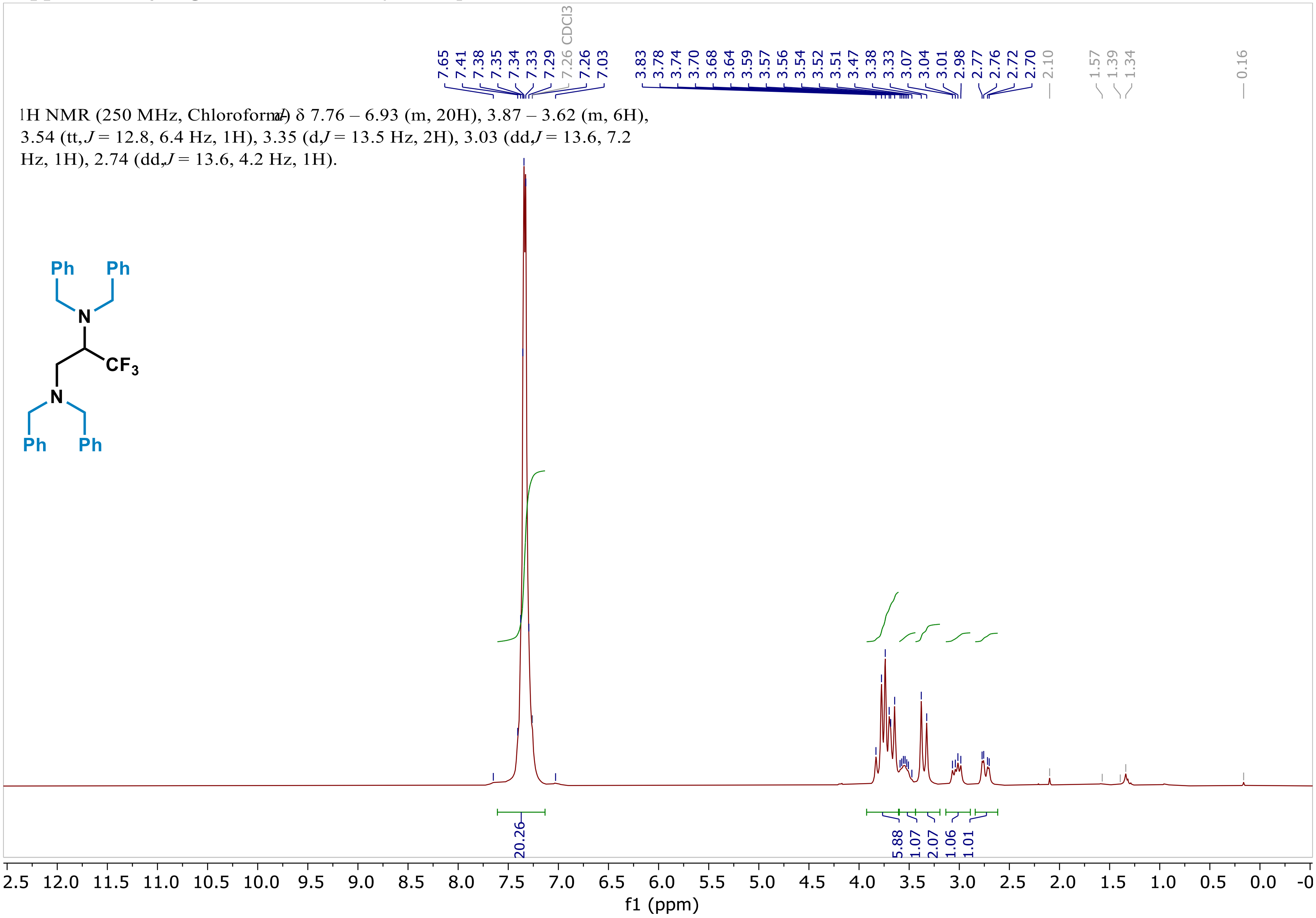

Supplementary Figure 125: *^{19}F NMR for compound* ***21***

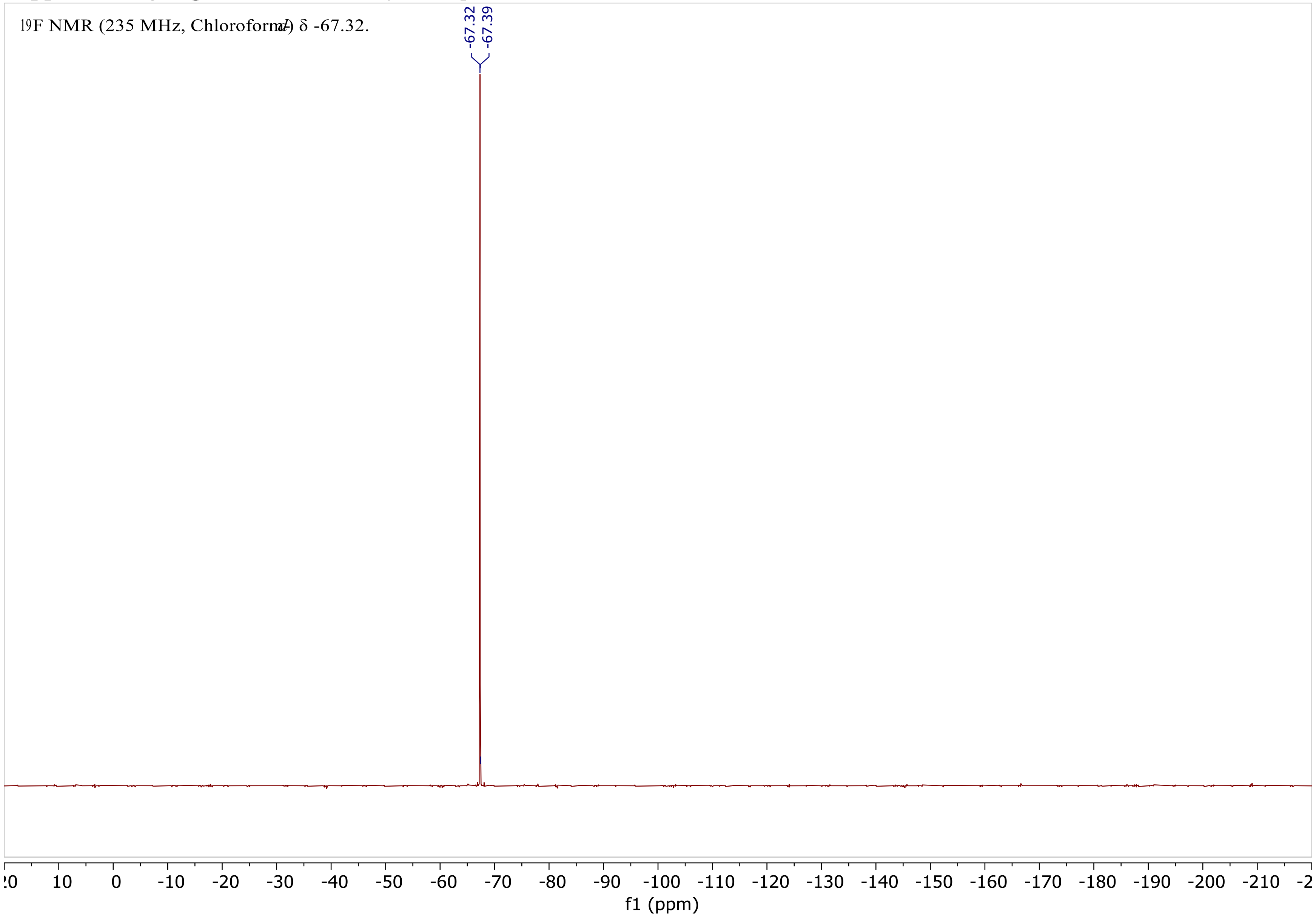

Supplementary Figure 126: *^{13}C NMR for compound **21***

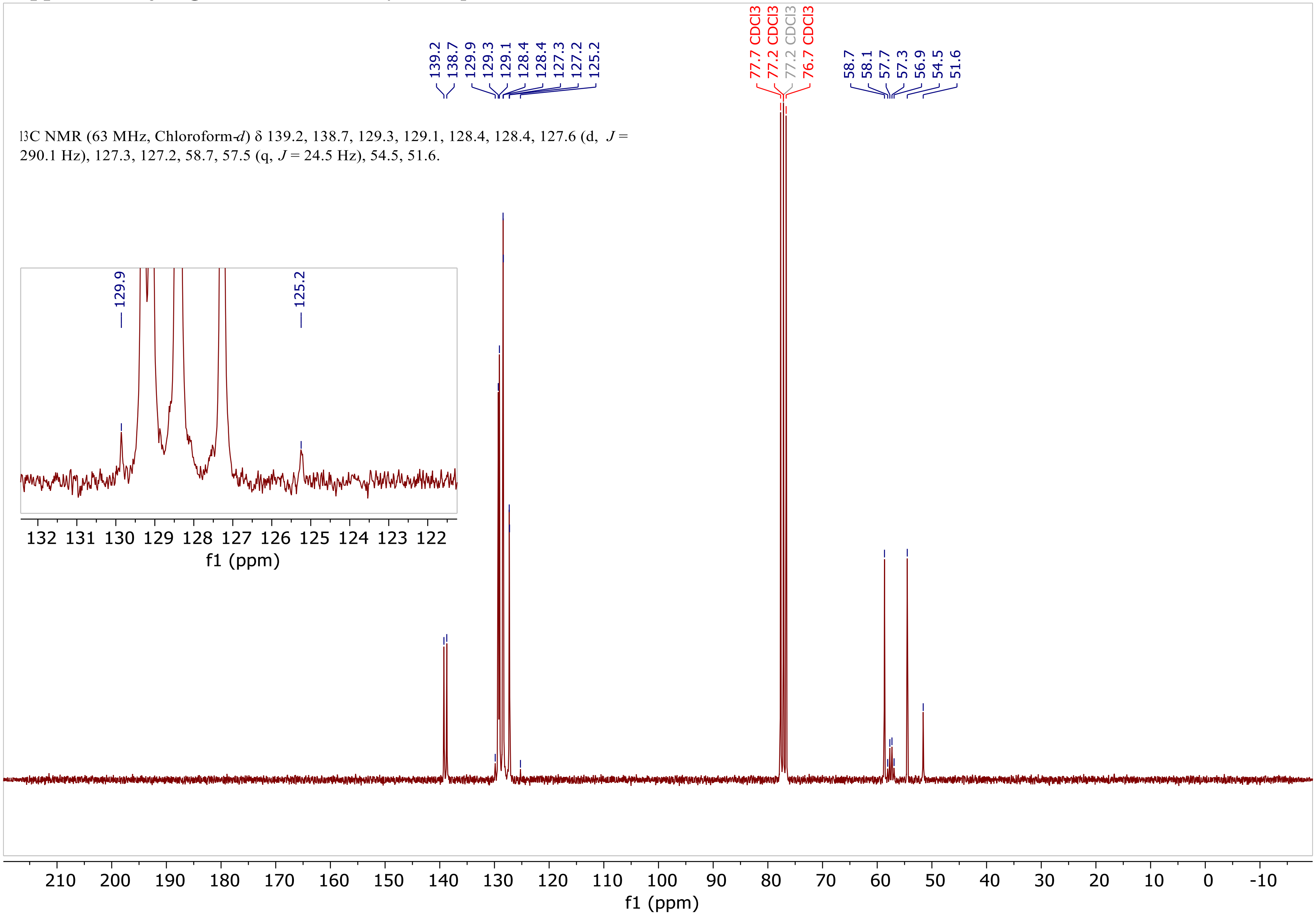

Supplementary Figure 127: *IR for compound **21***

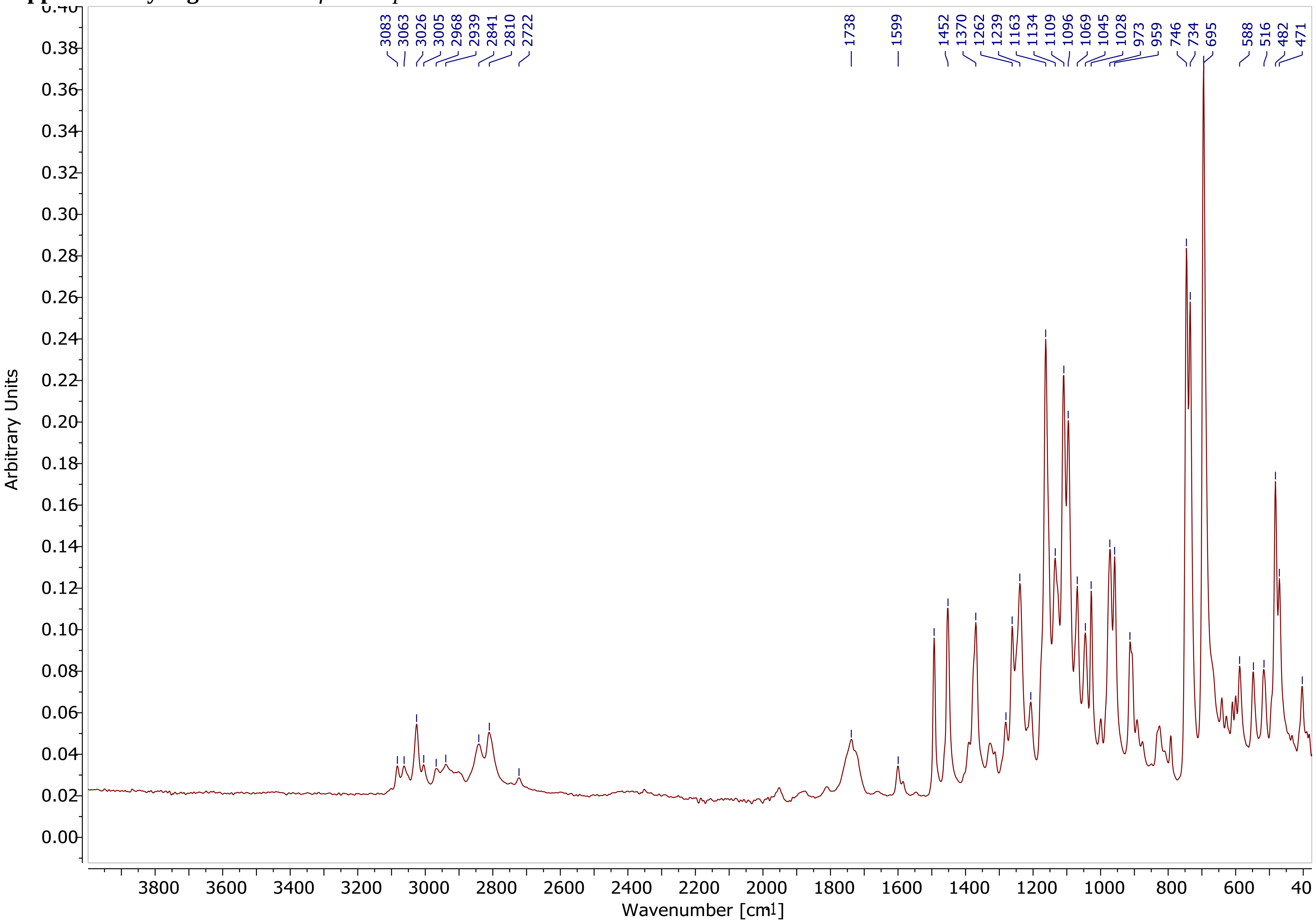

Supplementary Figure 128: *¹H-NMR for compound* ***22***

1H NMR (250 MHz, Chloroform-*d*) δ 3.64 (s, 21H), 3.11 (dd, *J* = 14.3, 8.1 Hz, 1H), 2.97 (dd, *J* = 14.3, 4.2 Hz, 1H).

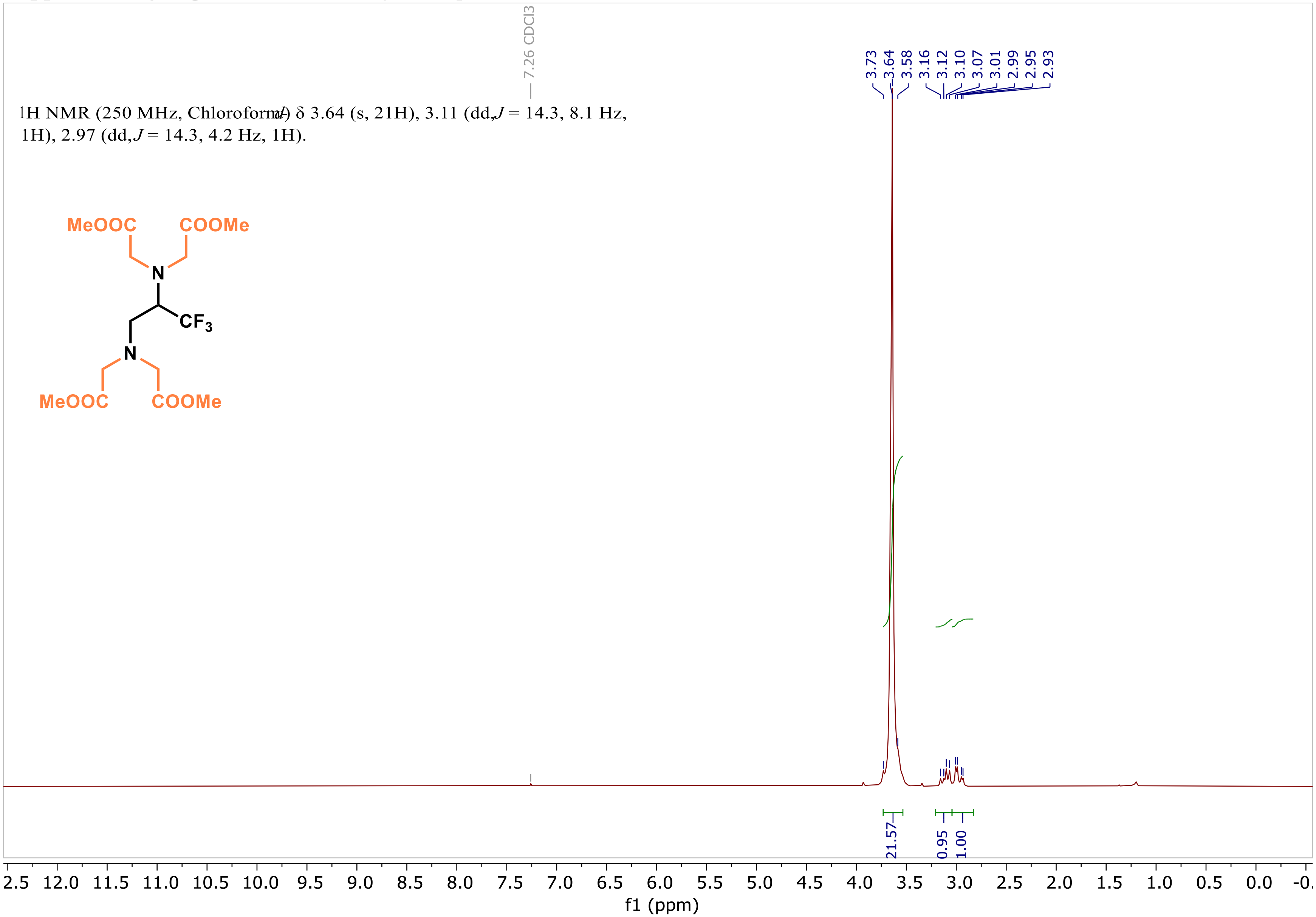

Supplementary Figure 129: *^{19}F NMR for compound **22***

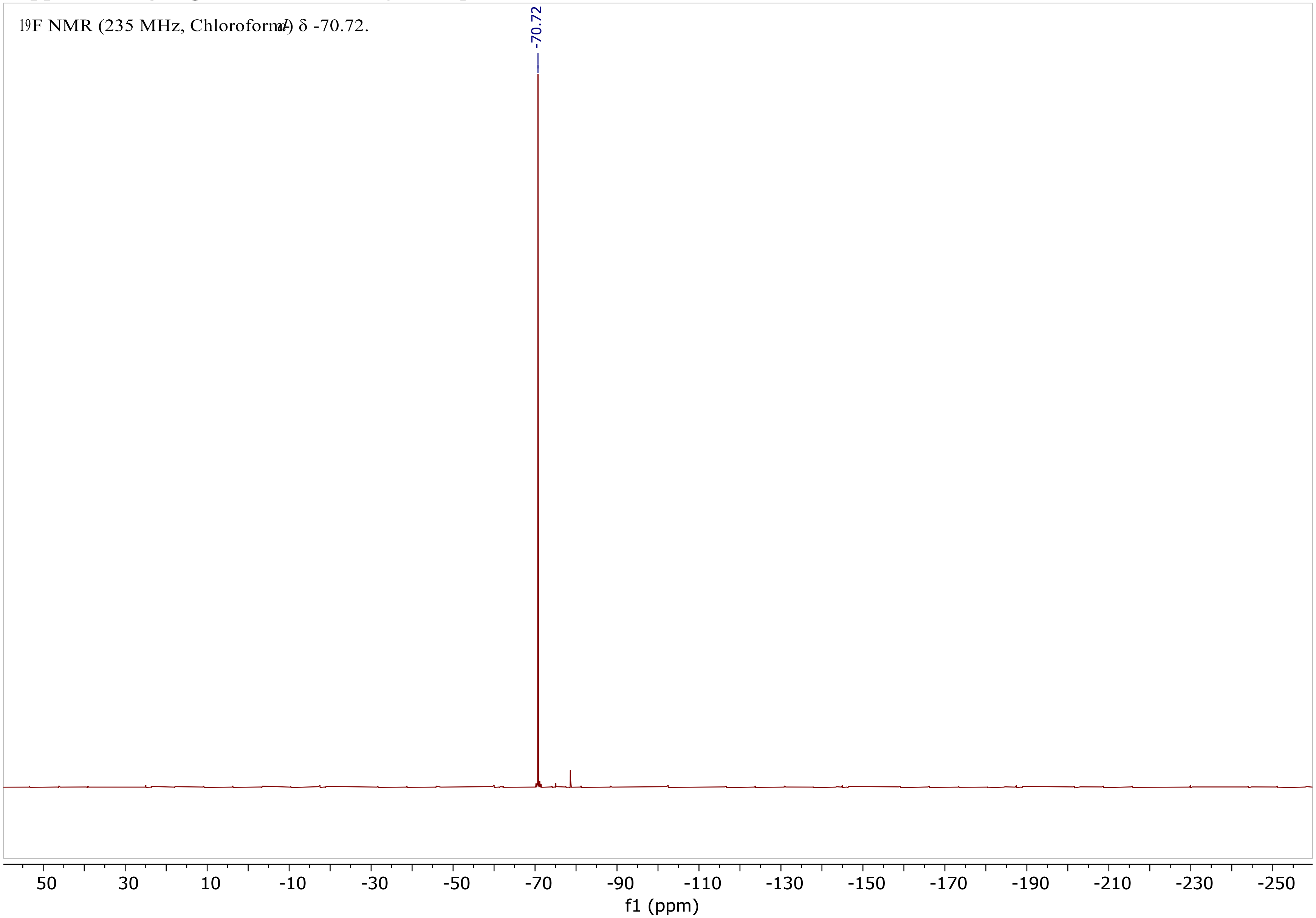

Supplementary Figure 130: *^{13}C NMR for compound* ***22***

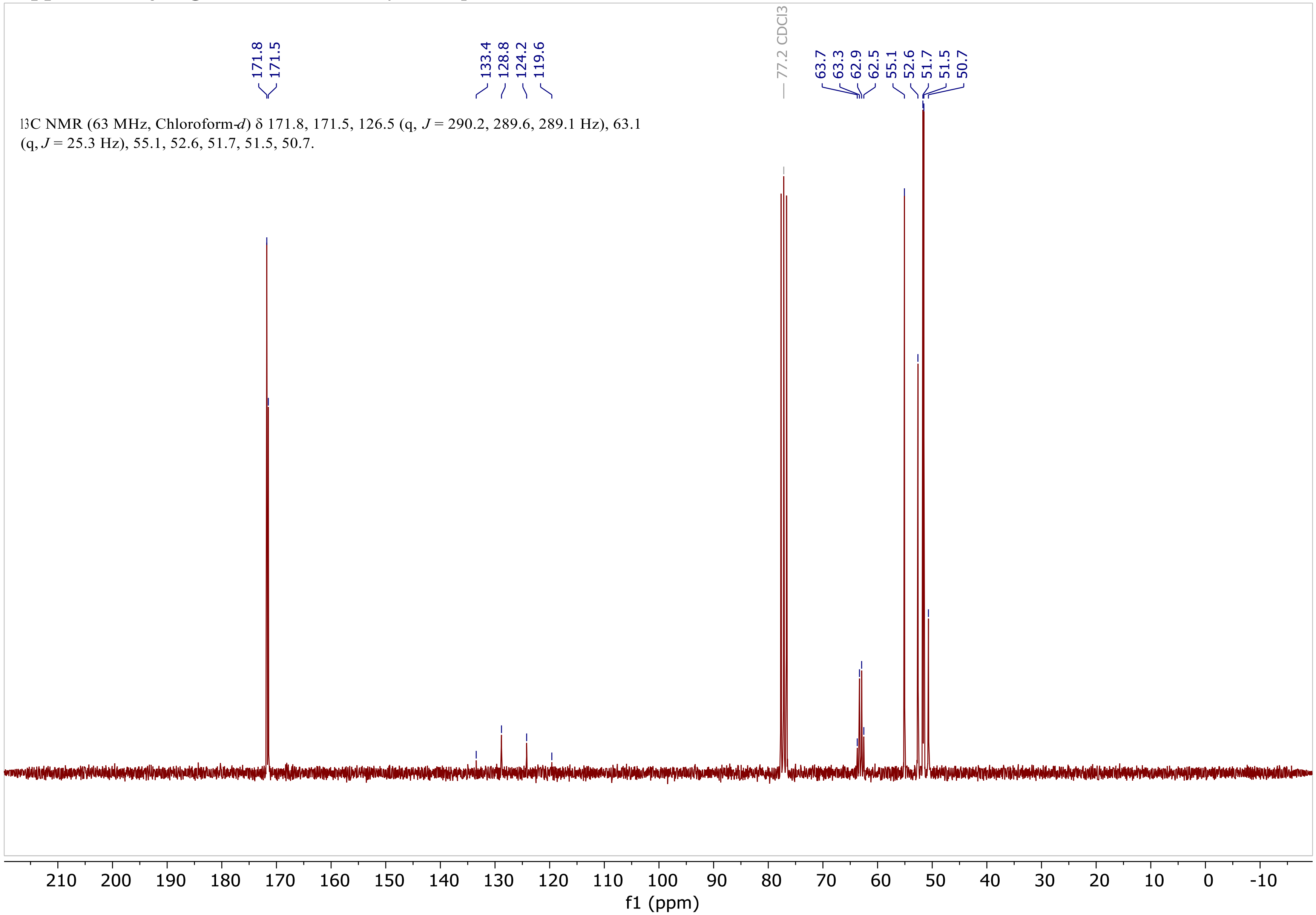

Supplementary Figure 131: *IR for compound* ***22***

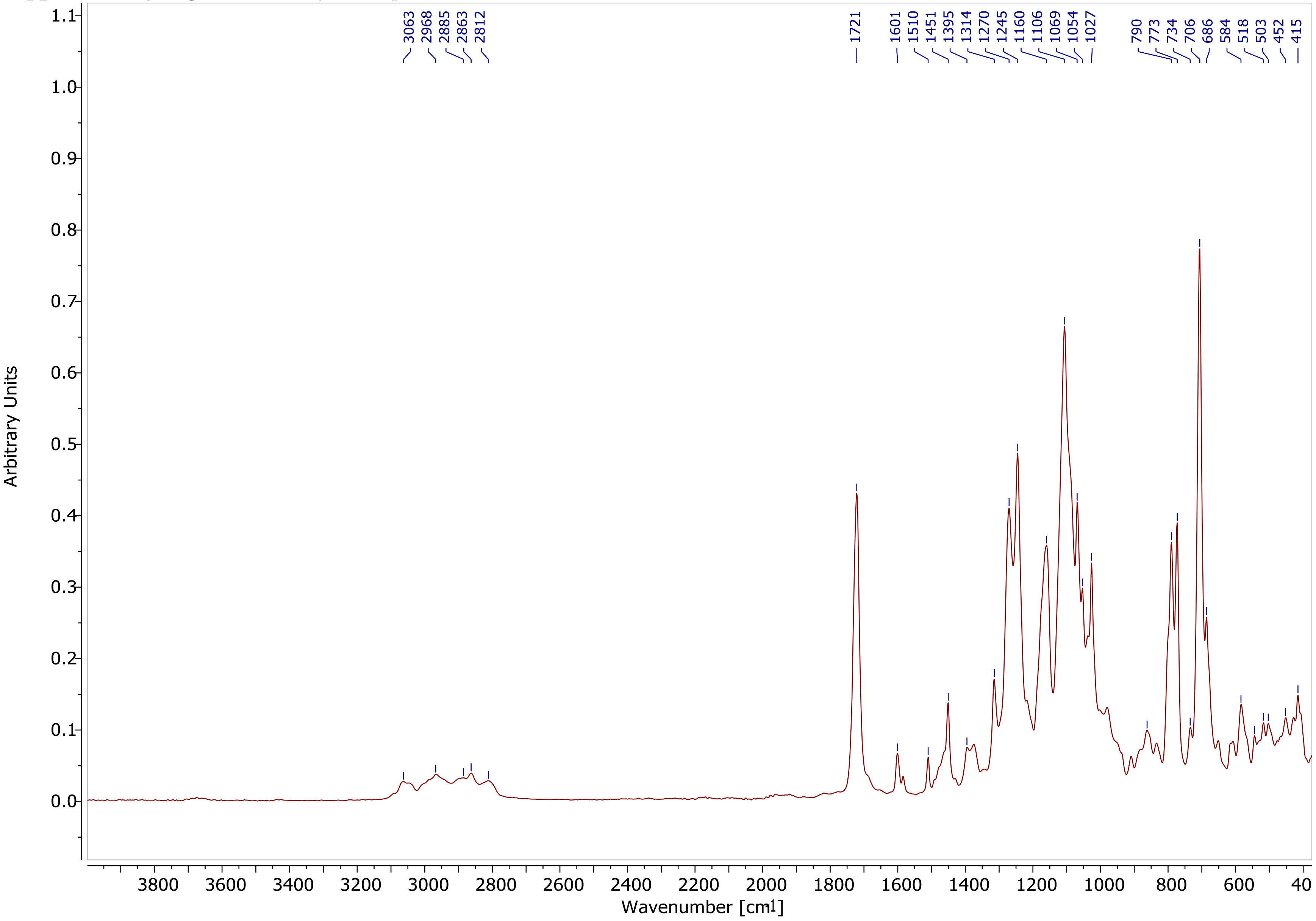

Supplementary Figure 132: *^{1}H-NMR for compound* ***23***

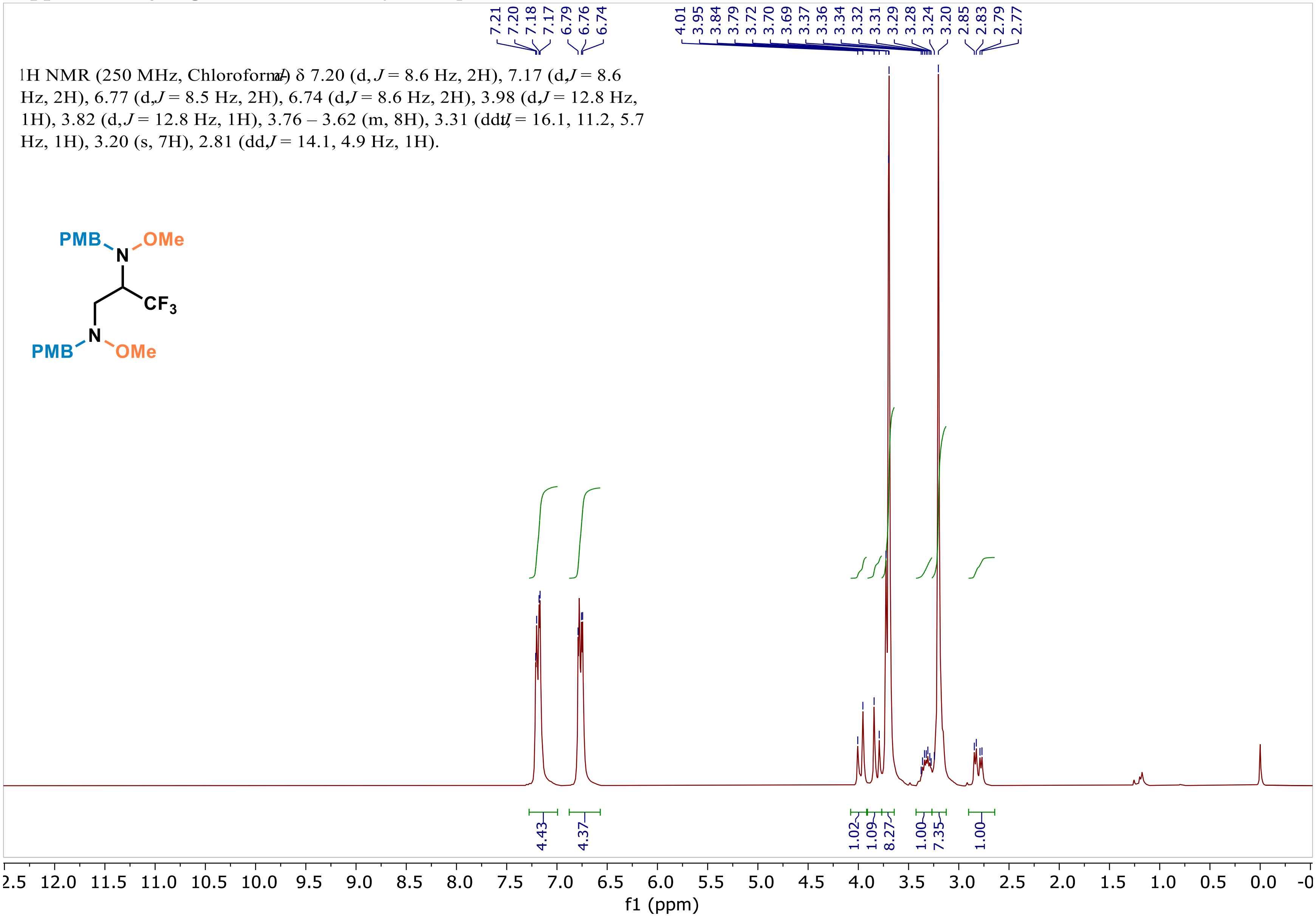

Supplementary Figure 133: *^{19}F NMR for compound* ***23***

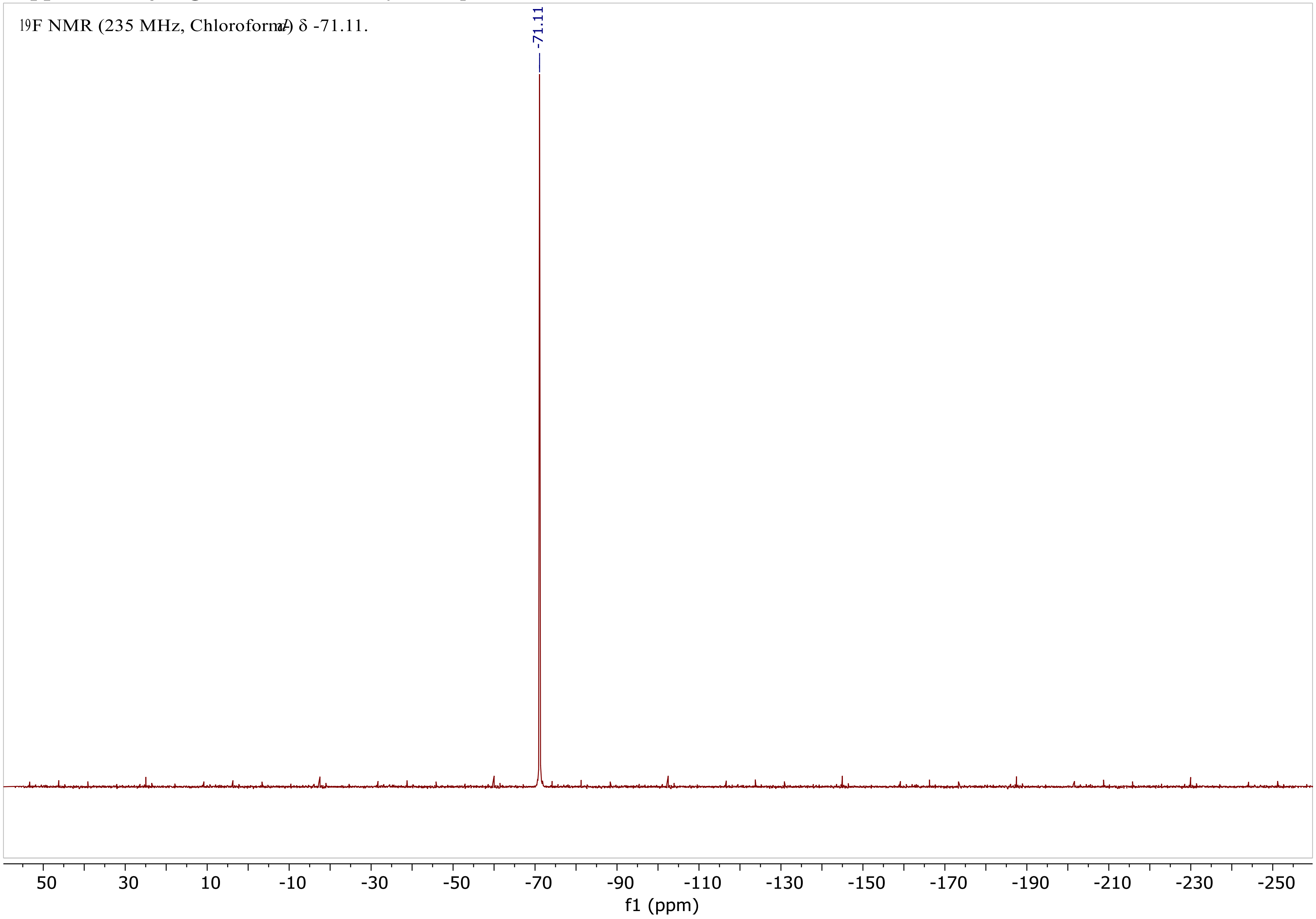

Supplementary Figure 134: *^{13}C NMR for compound **23***

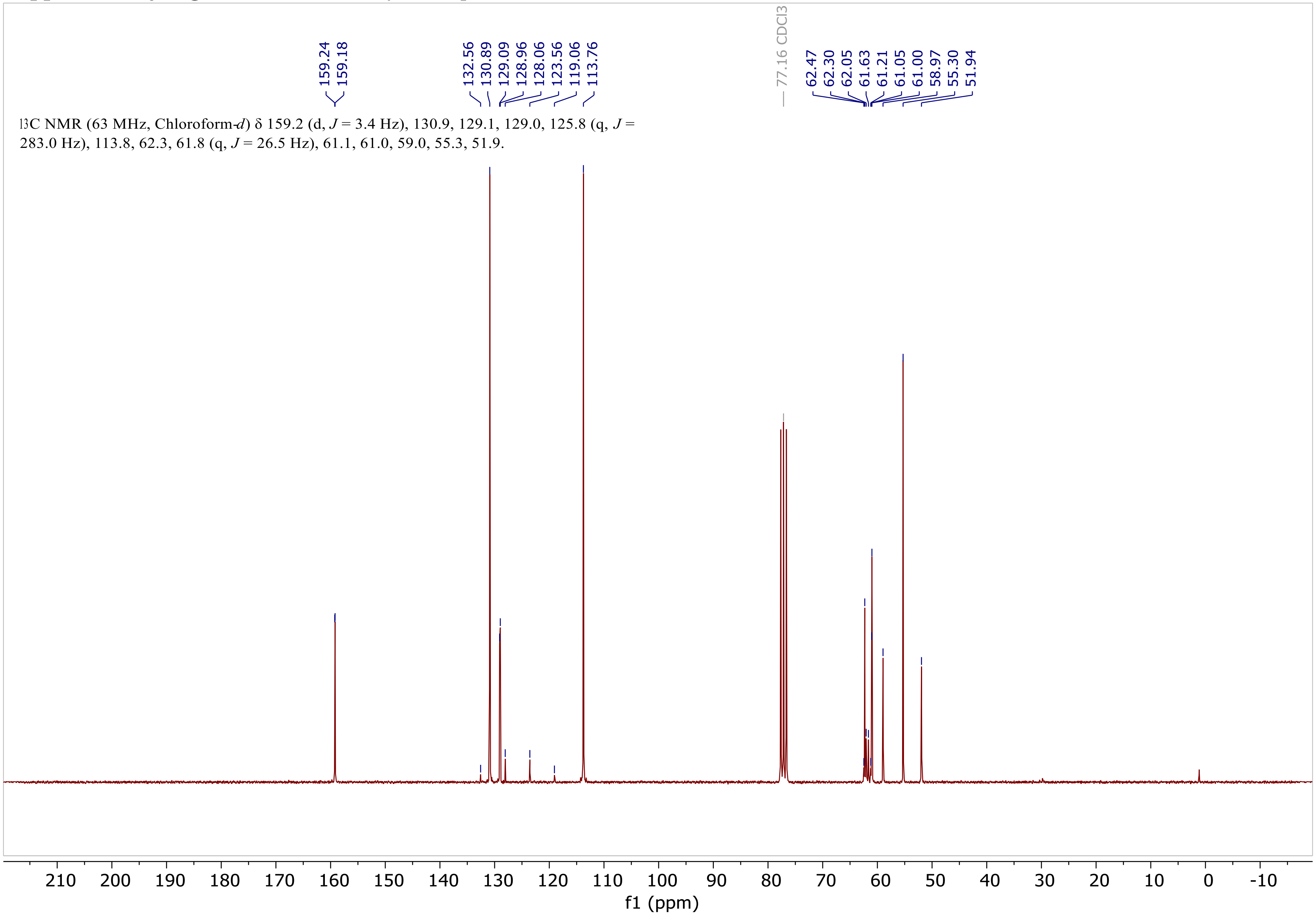

Supplementary Figure 135: *IR for compound **23***

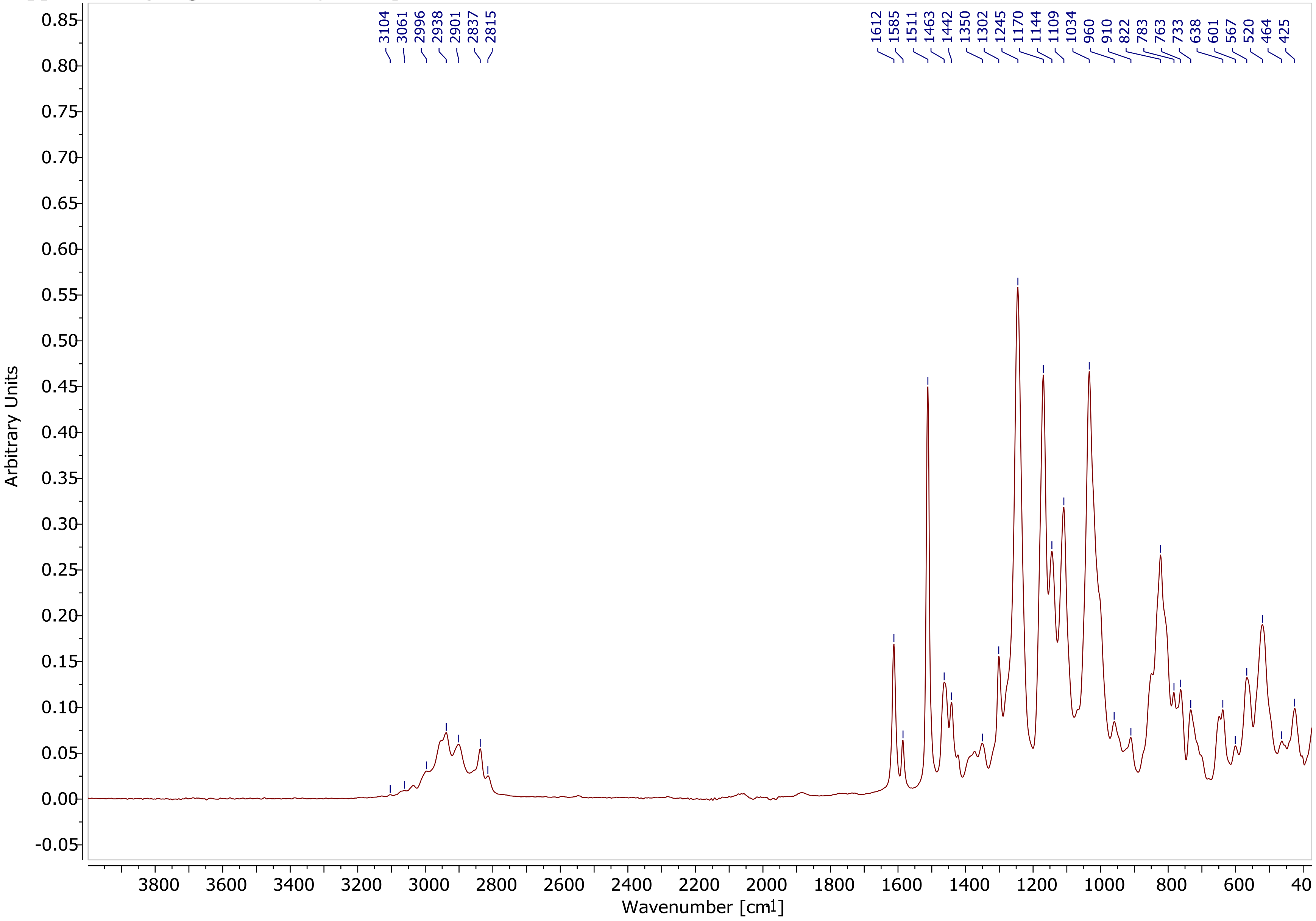

Supplementary Figure 136: *^{1}H-NMR for compound* ***24***

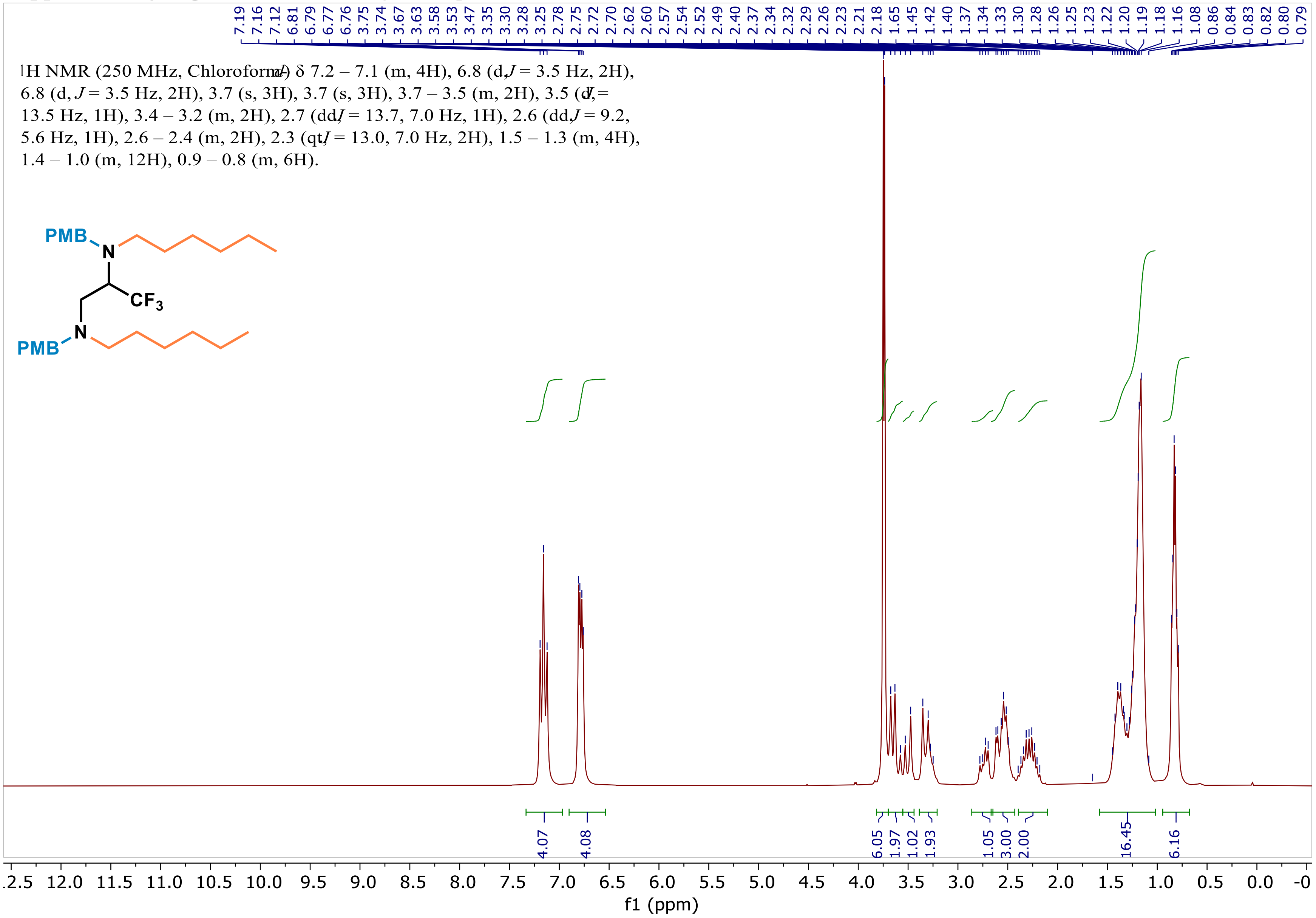

Supplementary Figure 137: *^{19}F NMR for compound* ***24***

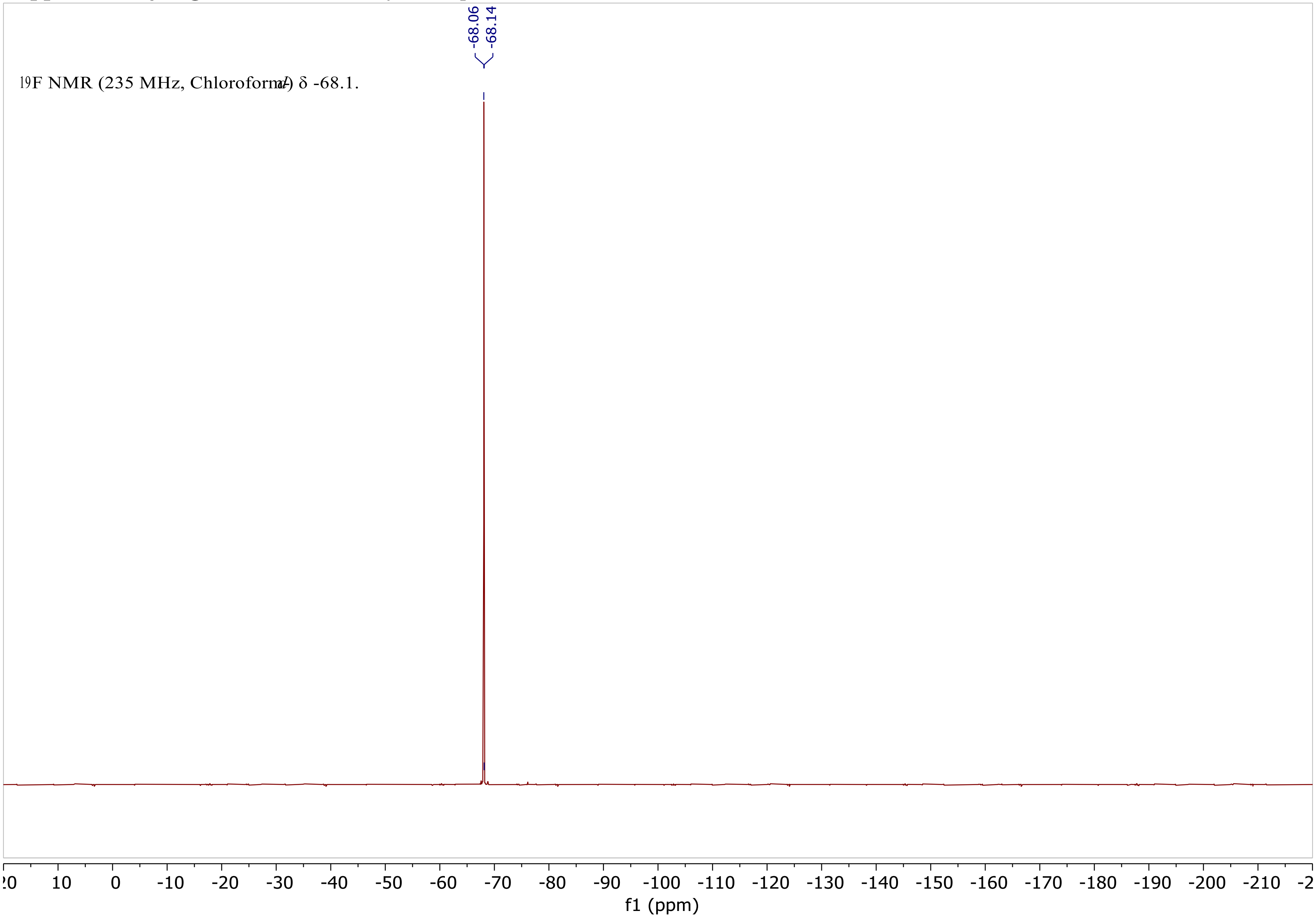

Supplementary Figure 138: *^{13}C NMR for compound* ***24***

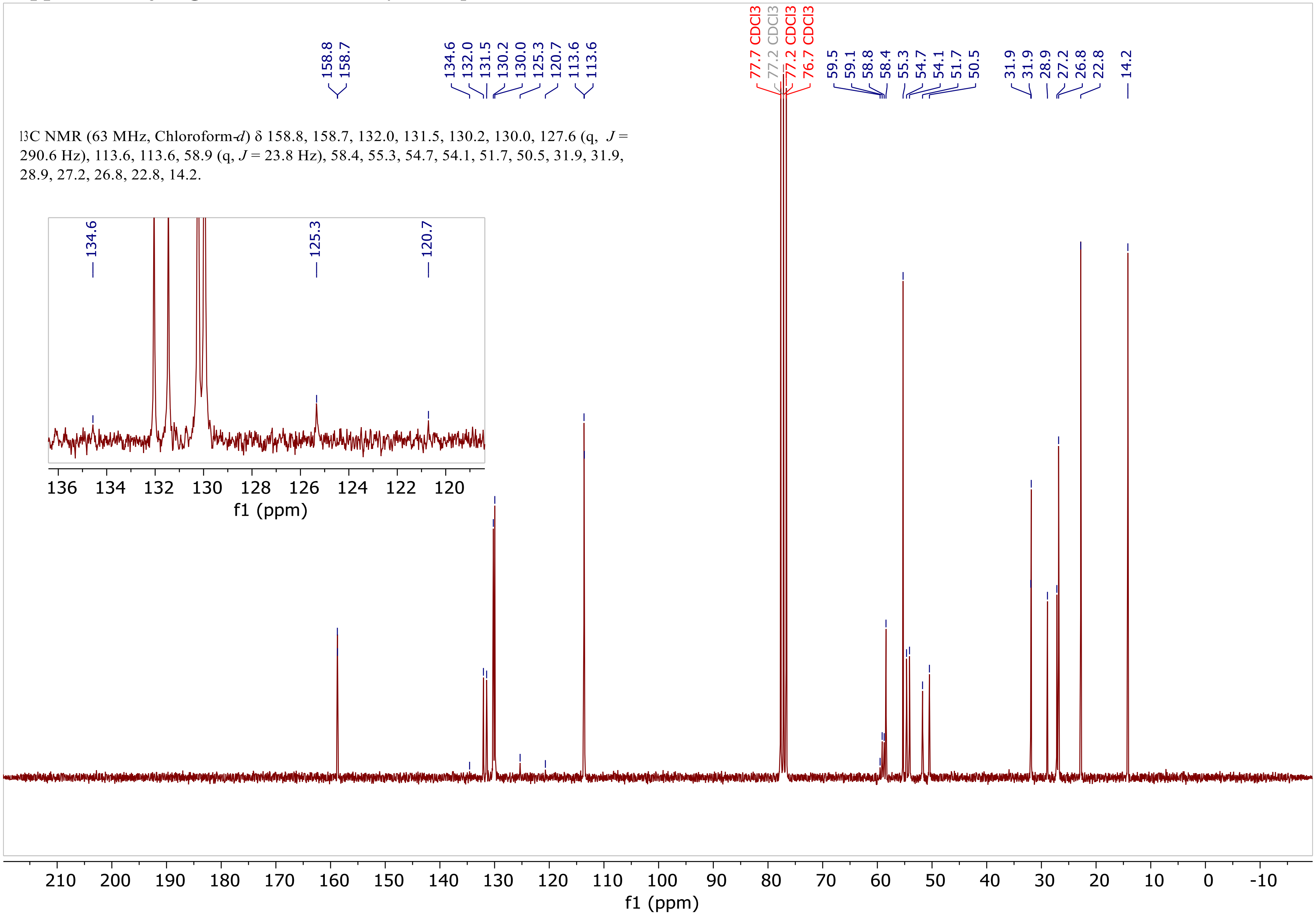

Supplementary Figure 139: *IR for compound* ***24***

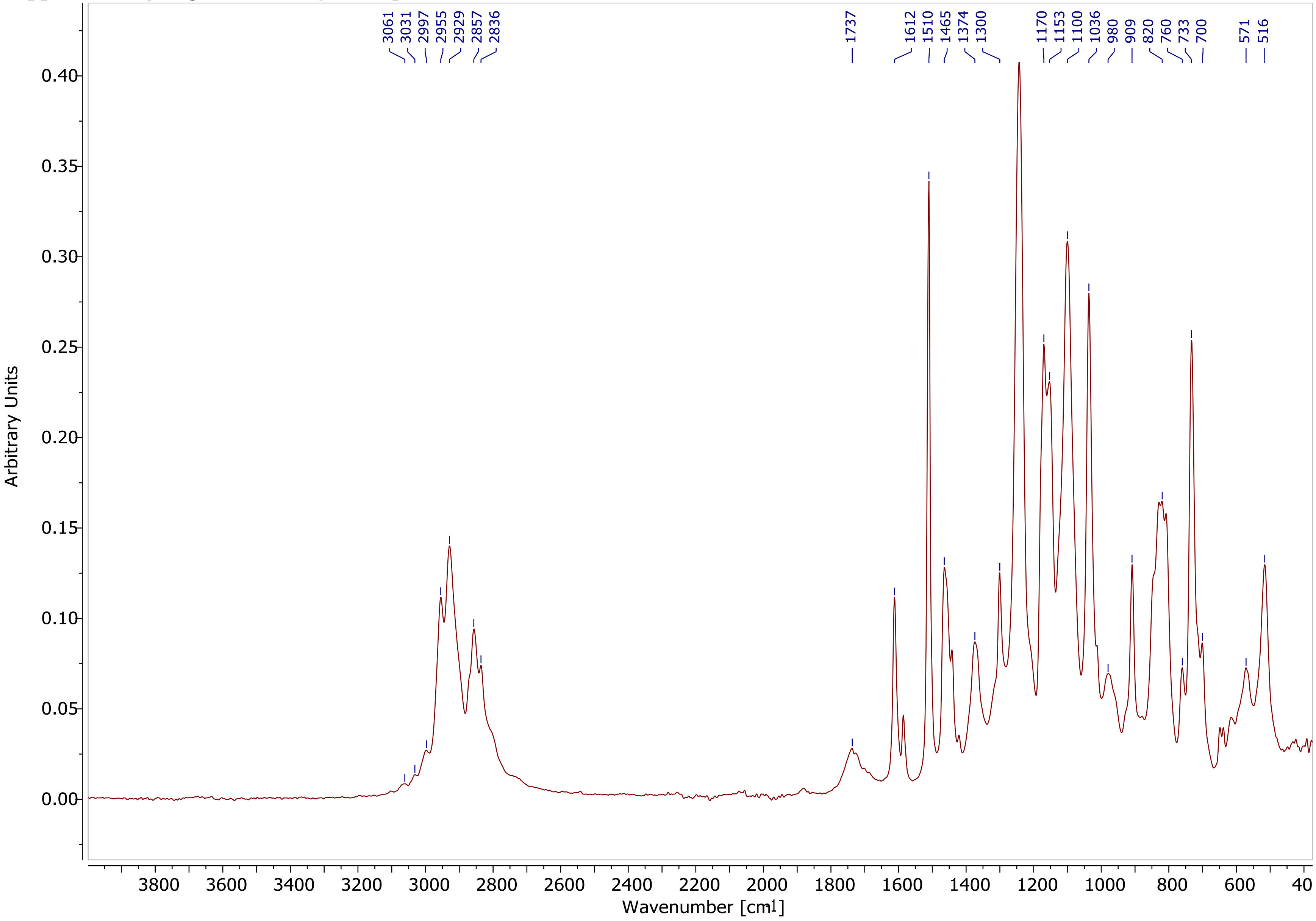

Supplementary Figure 140: *^{1}H-NMR for compound* ***25***

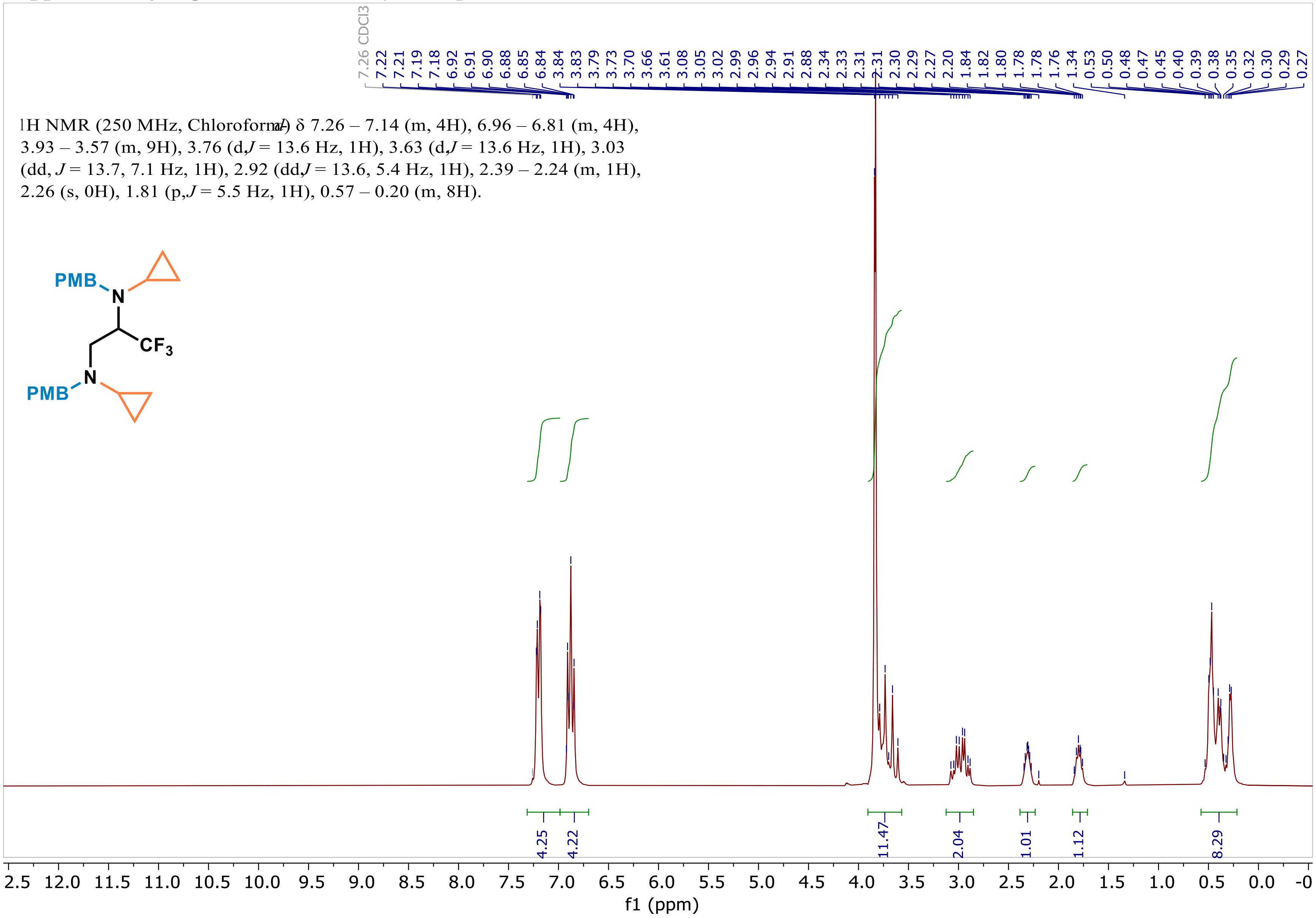

Supplementary Figure 141: *^{19}F NMR for compound* ***25***

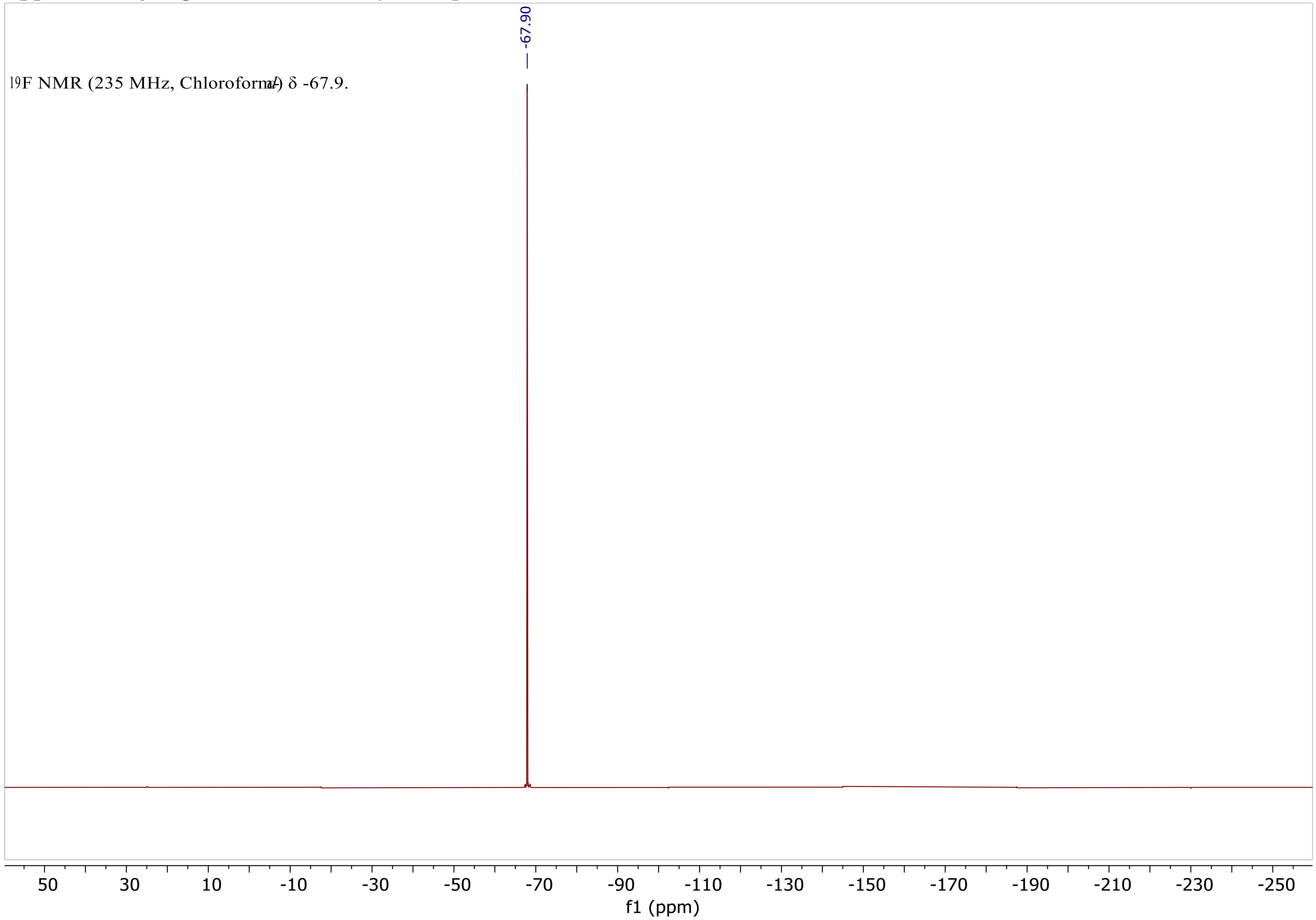

Supplementary Figure 142: *^{13}C NMR for compound* ***25***

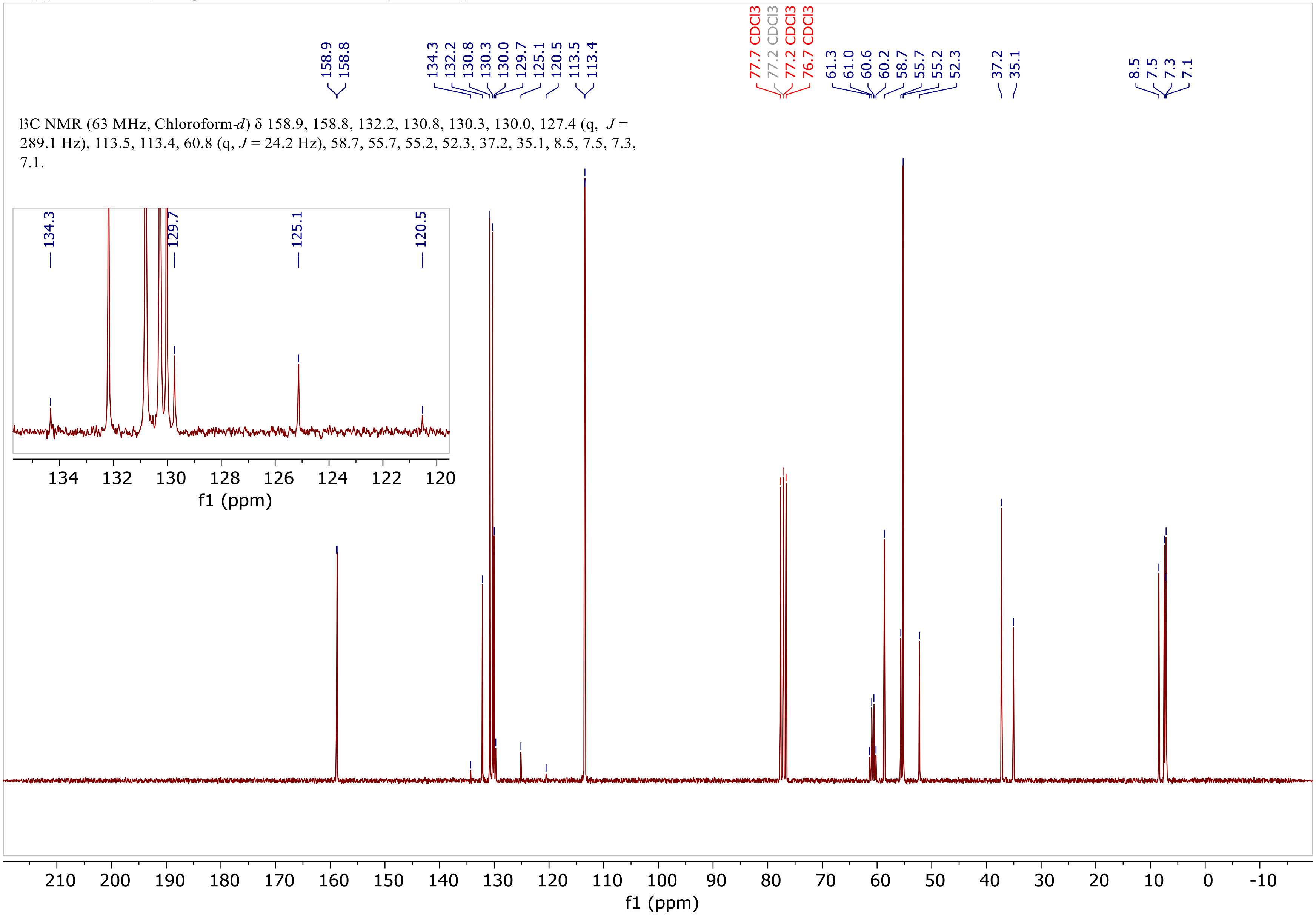

Supplementary Figure 143: *IR for compound* ***25***

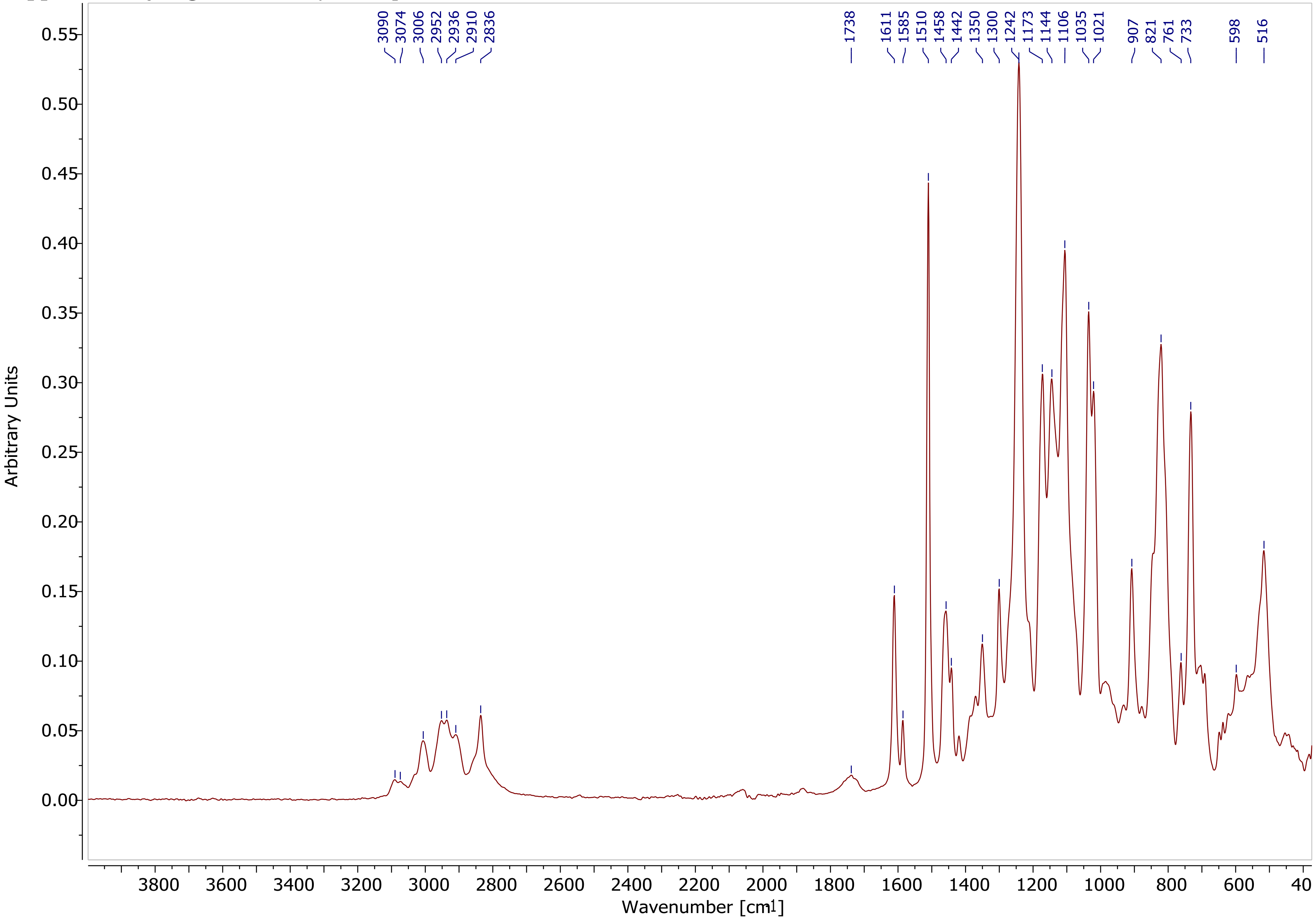

Supplementary Figure 144: *^{1}H-NMR for compound* ***26***

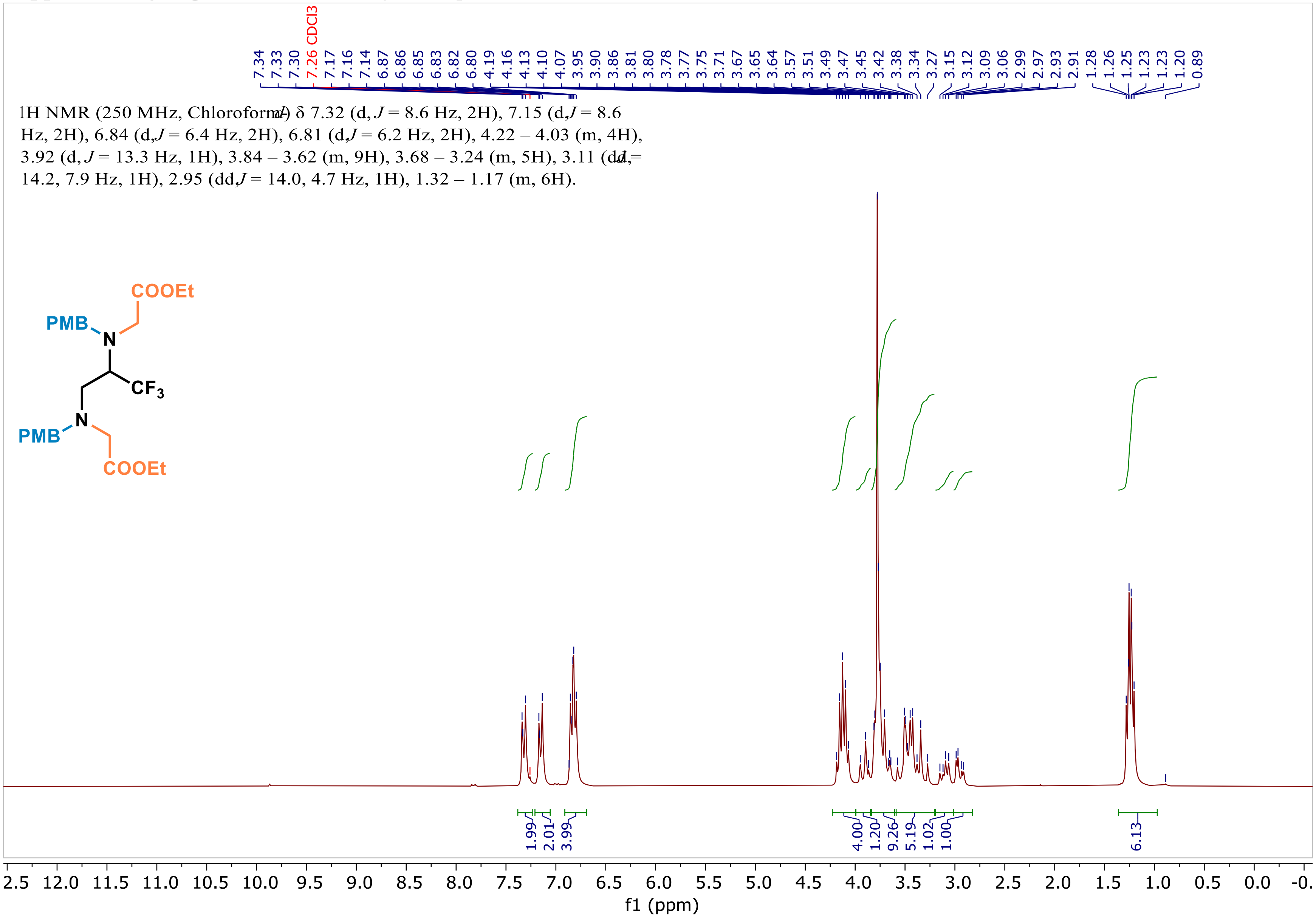

Supplementary Figure 145: *^{19}F NMR for compound* ***26***

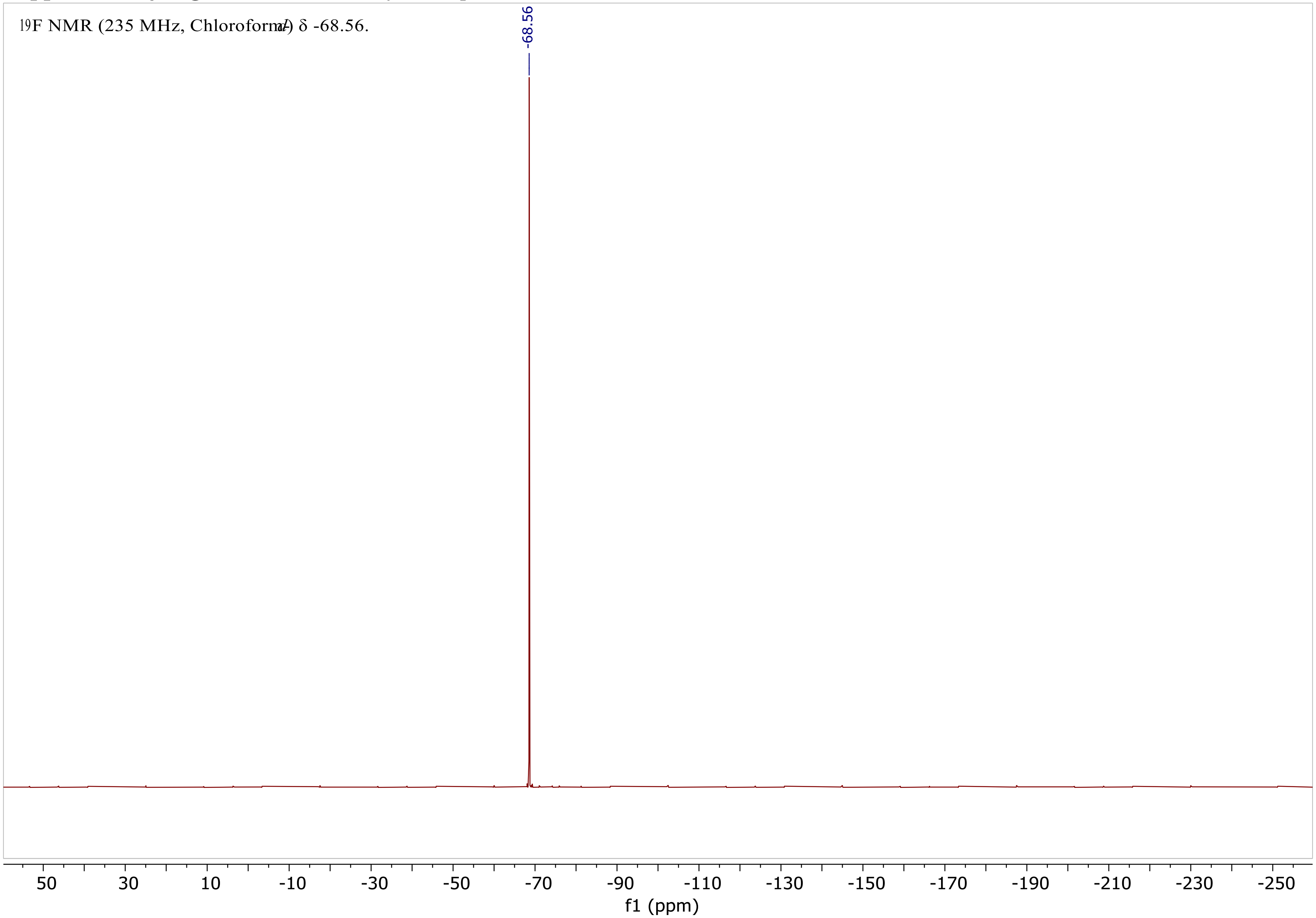

Supplementary Figure 146: *^{13}C NMR for compound* ***26***

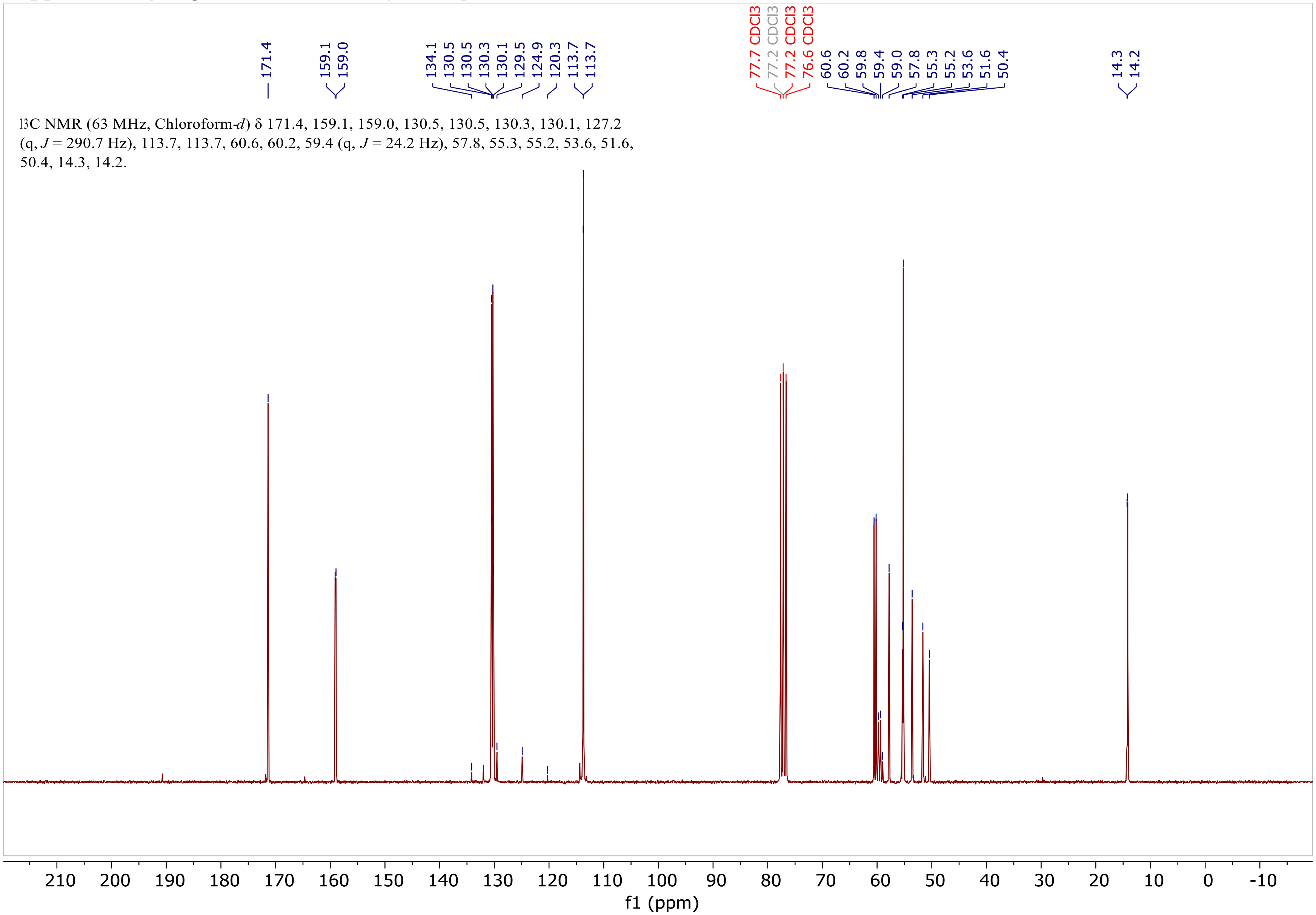

Supplementary Figure 147: *IR for compound* ***26***

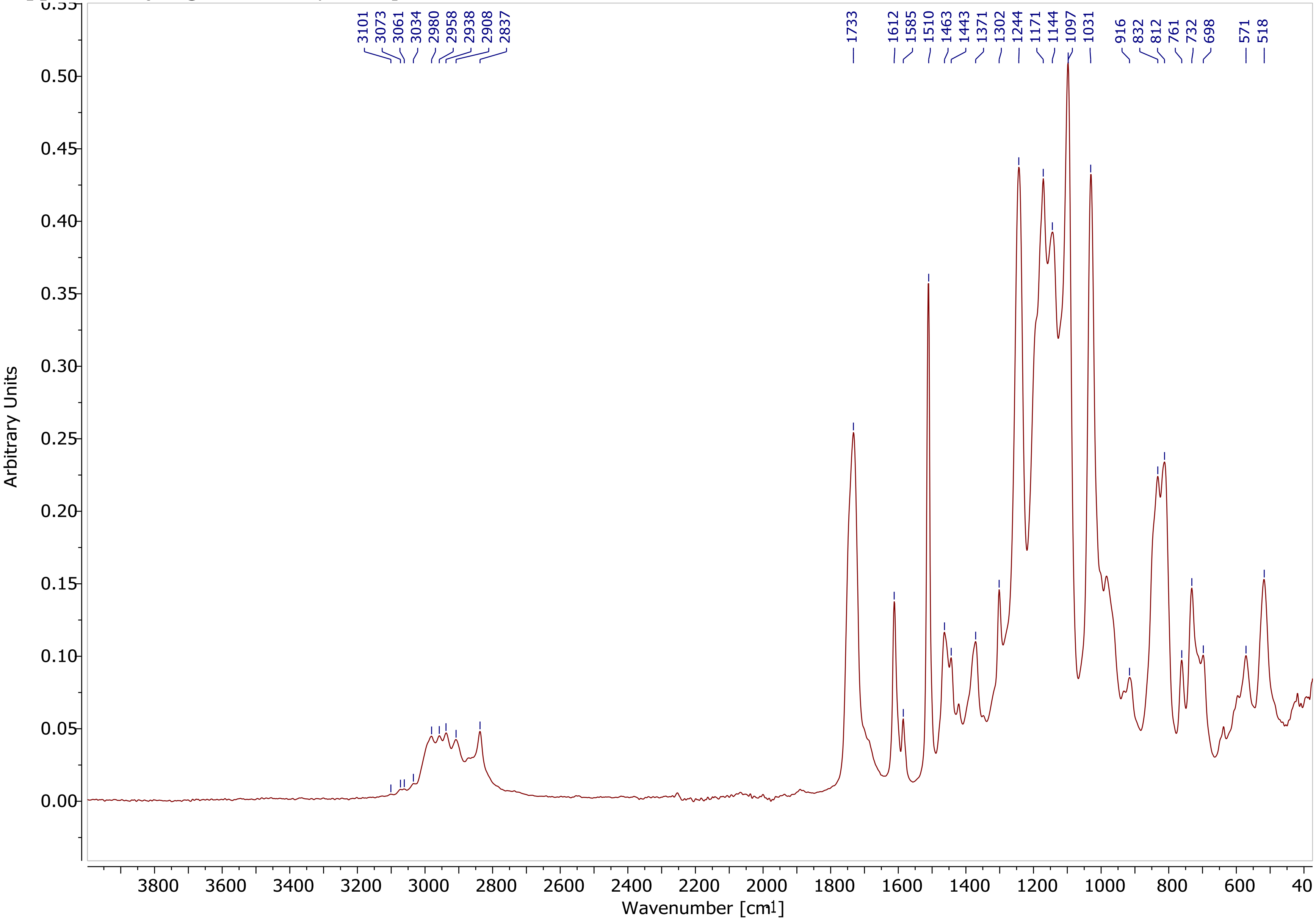

Supplementary Figure 148: *^{1}H-NMR for compound **27***

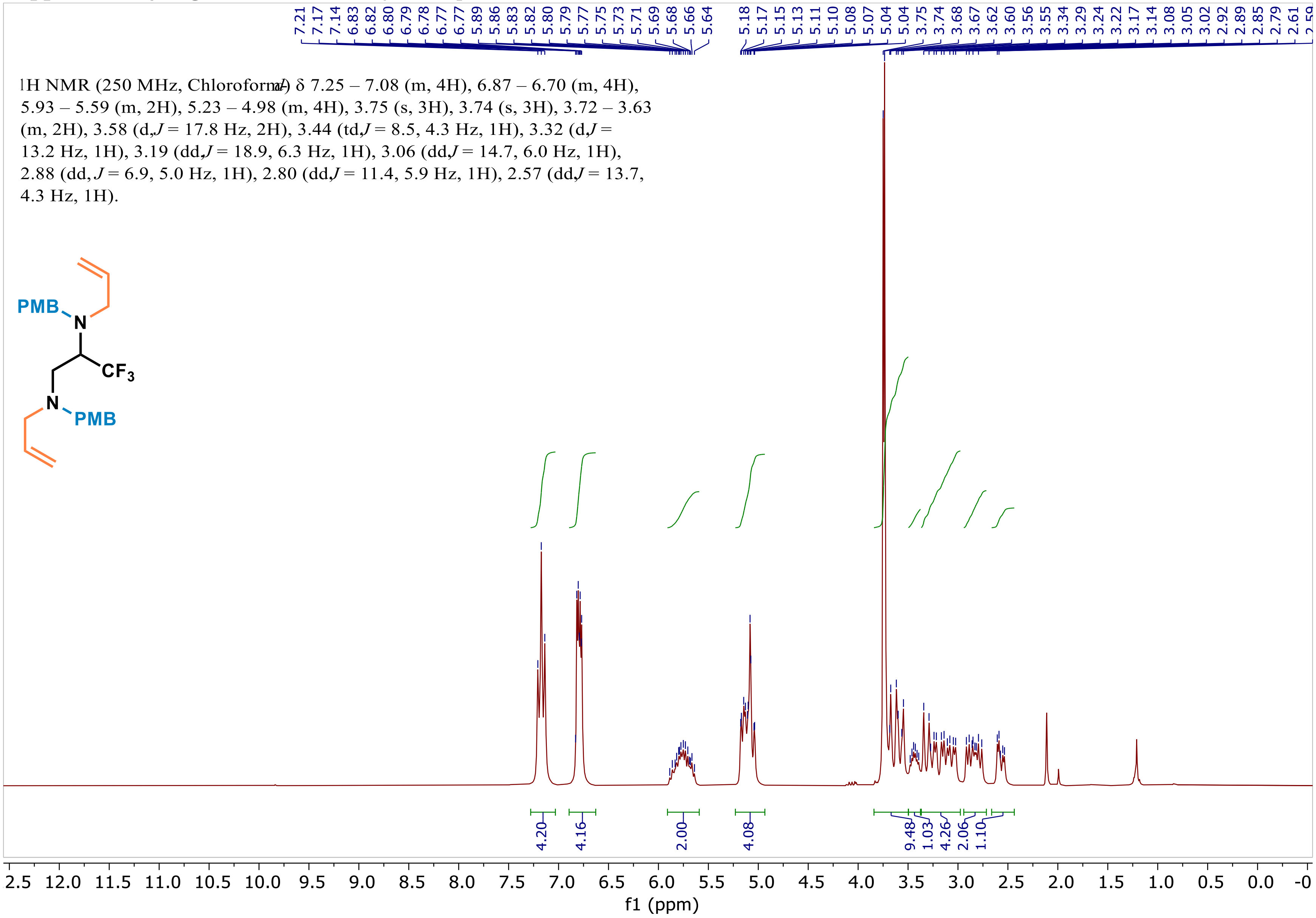

Supplementary Figure 149: *^{19}F NMR for compound* ***27***

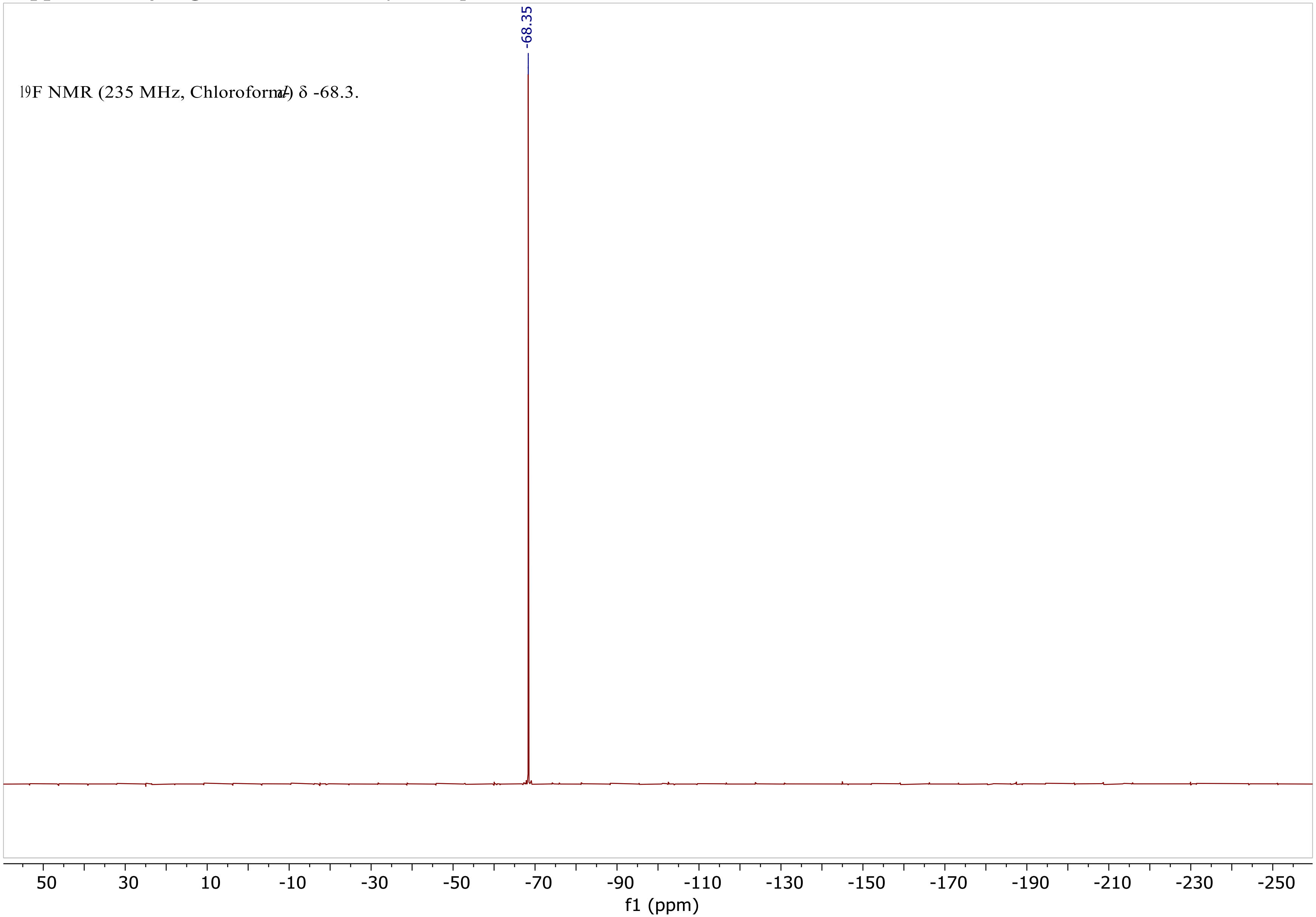

Supplementary Figure 150: *^{13}C NMR for compound* ***27***

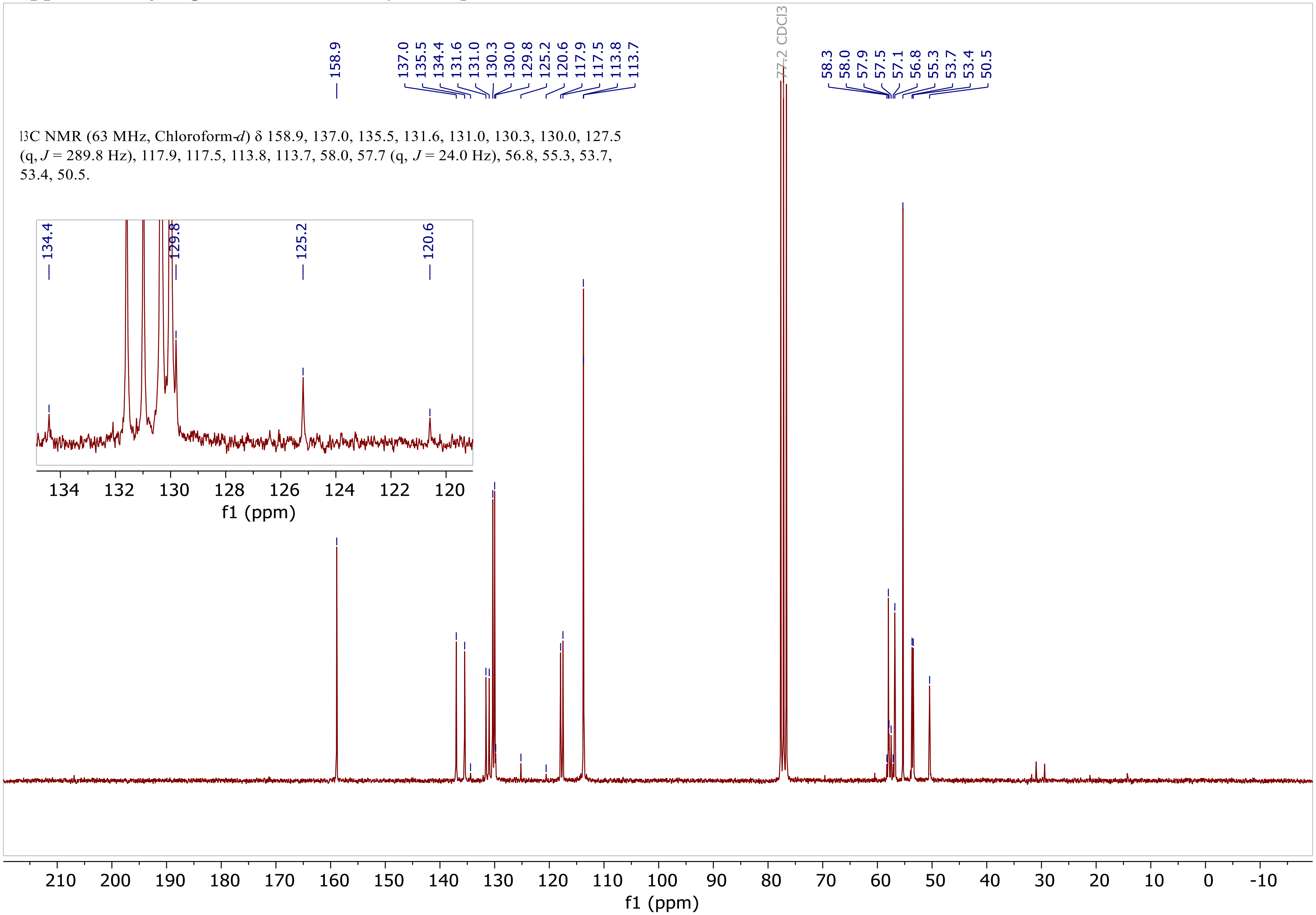

Supplementary Figure 151: *IR for compound **27***

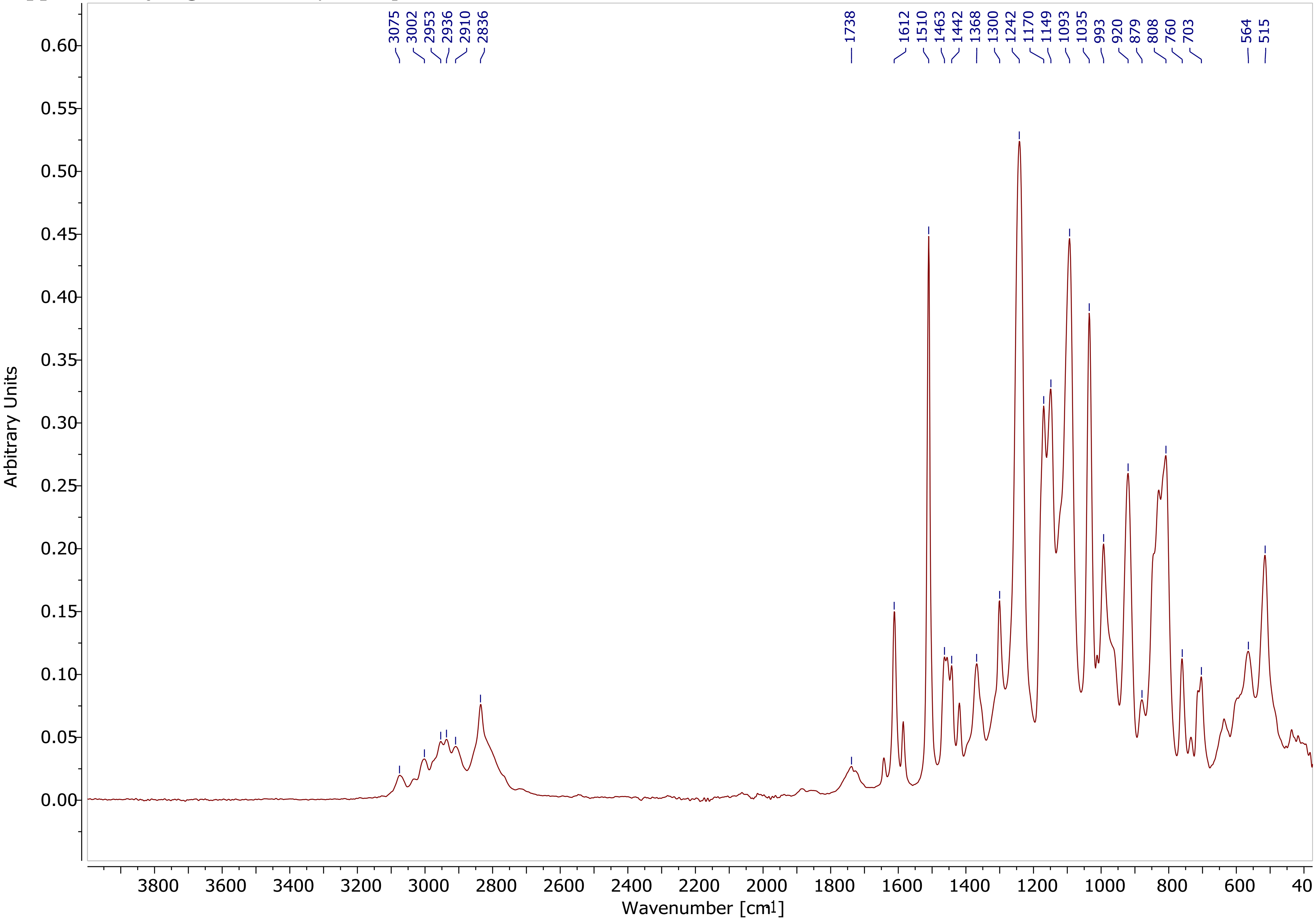

Supplementary Figure 152: *^{1}H-NMR for compound **28***

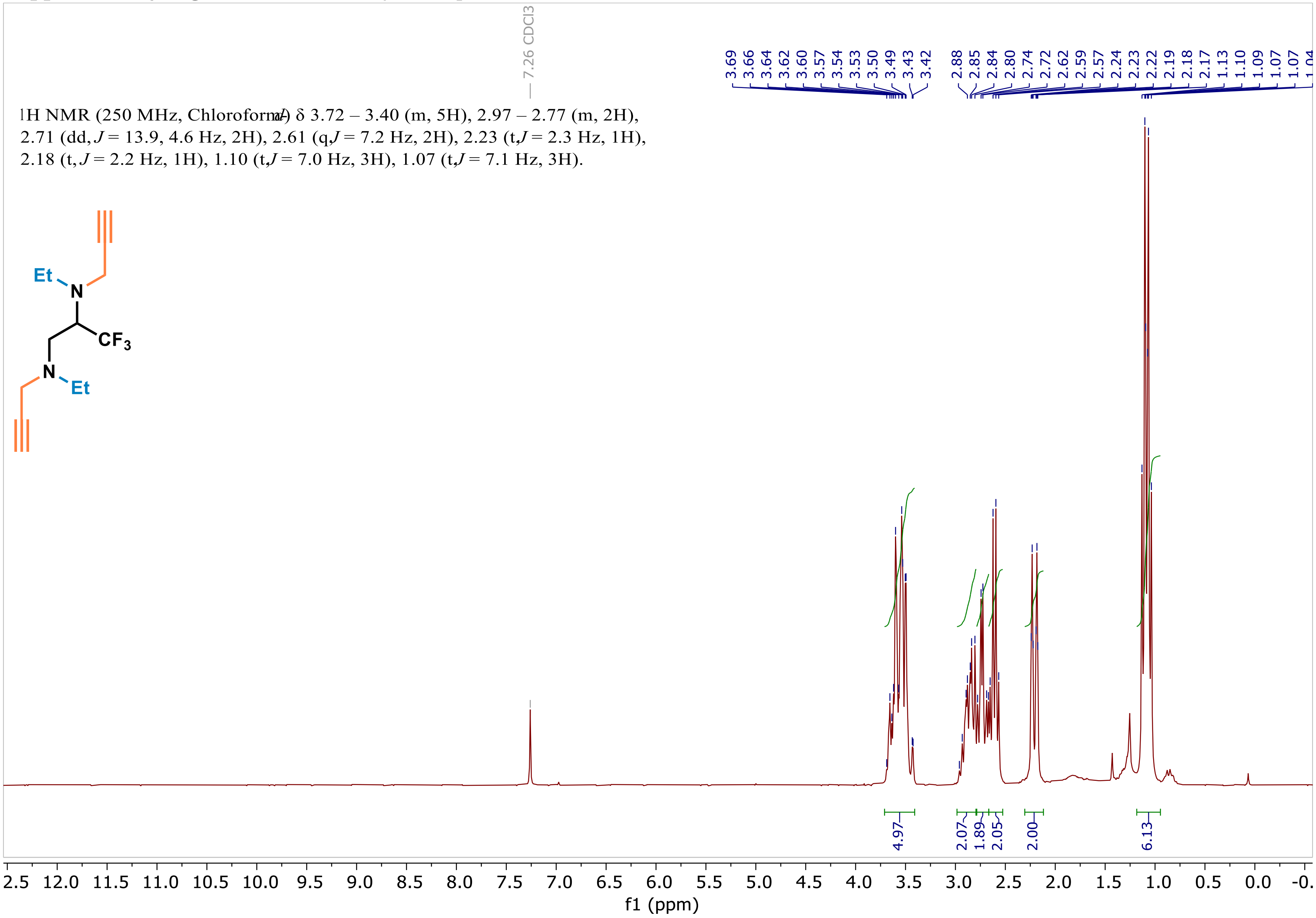

Supplementary Figure 153: *^{19}F NMR for compound* ***28***

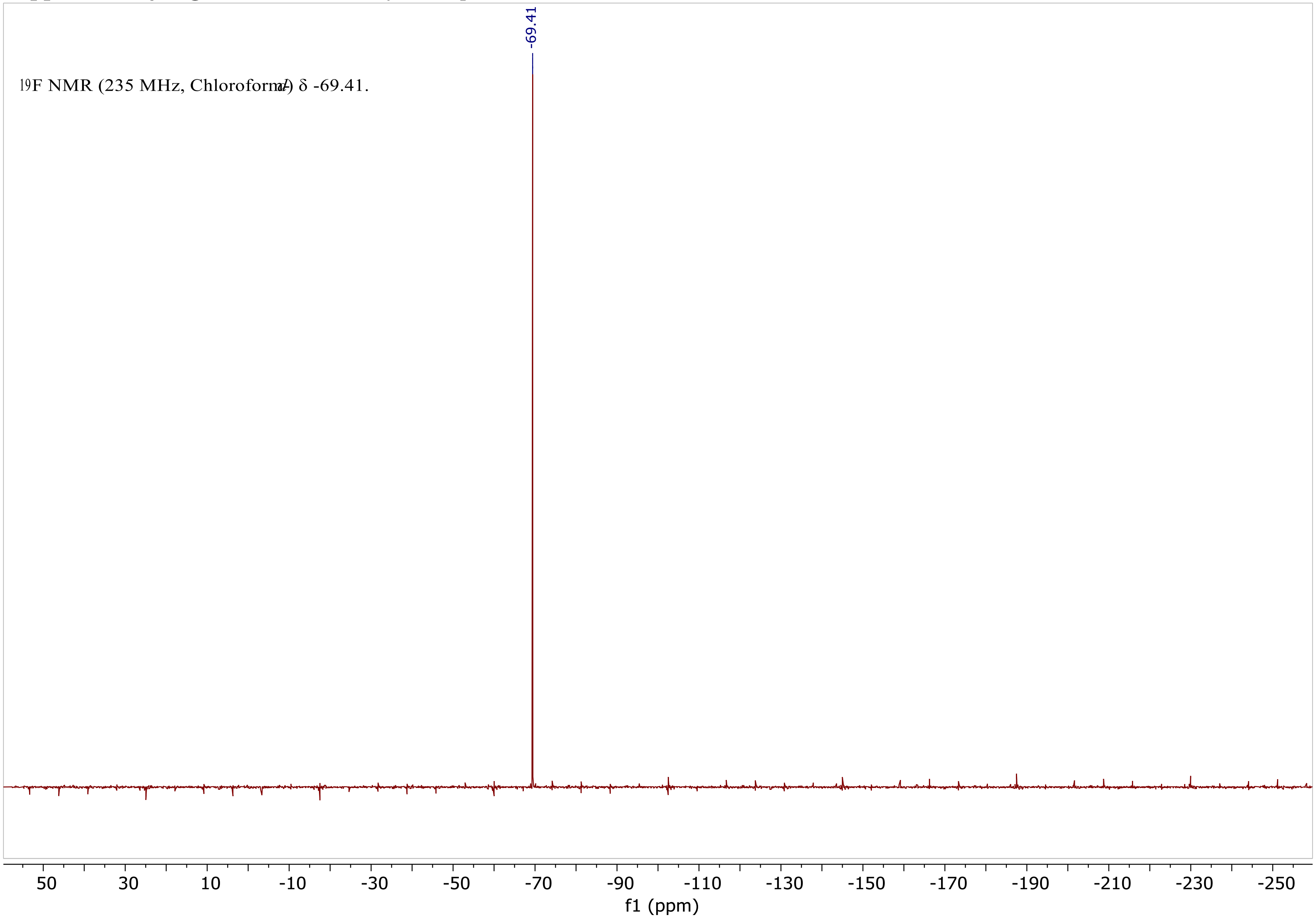

Supplementary Figure 154: *^{13}C NMR for compound* ***28***

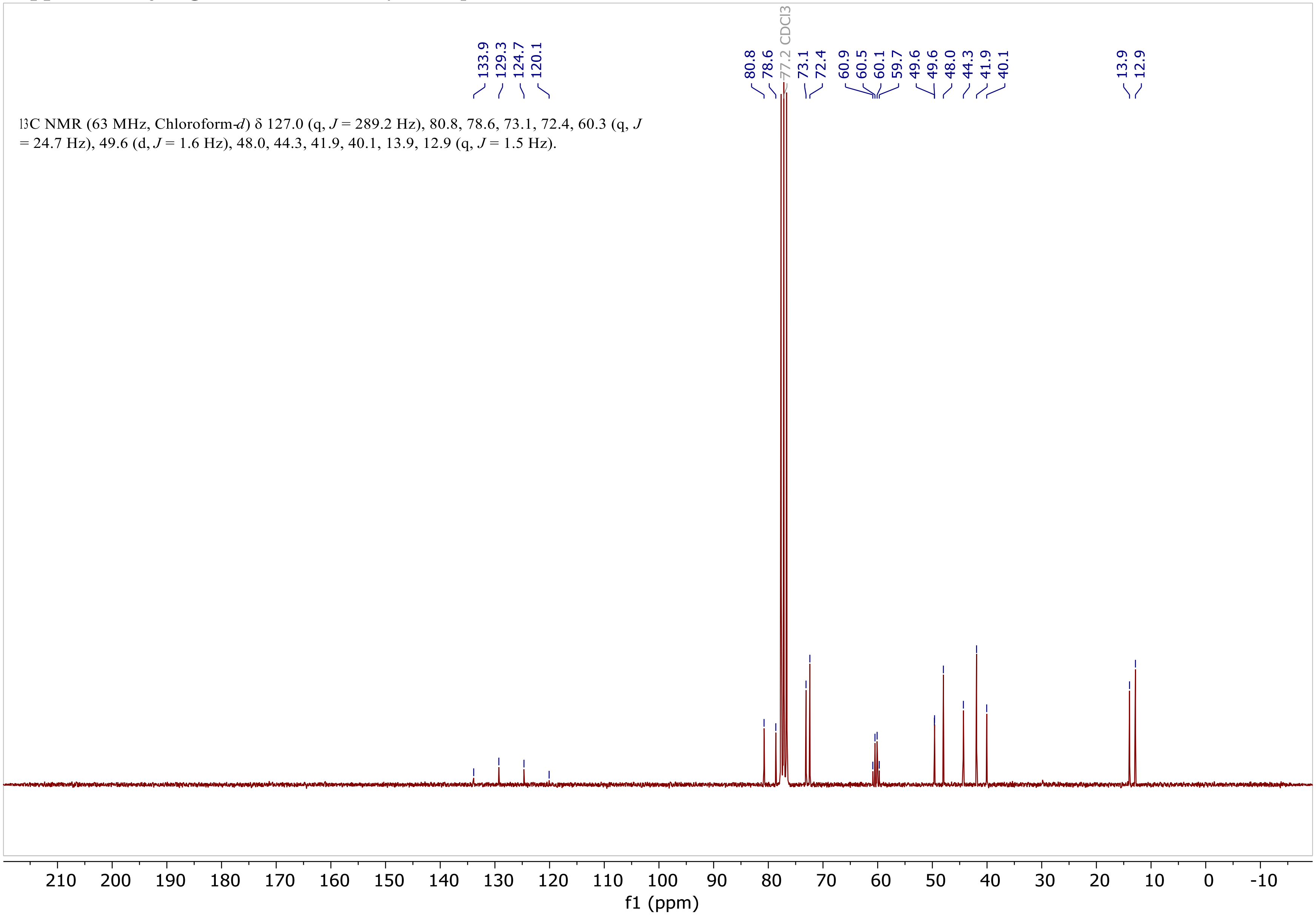

Supplementary Figure 155: *IR for compound **28***

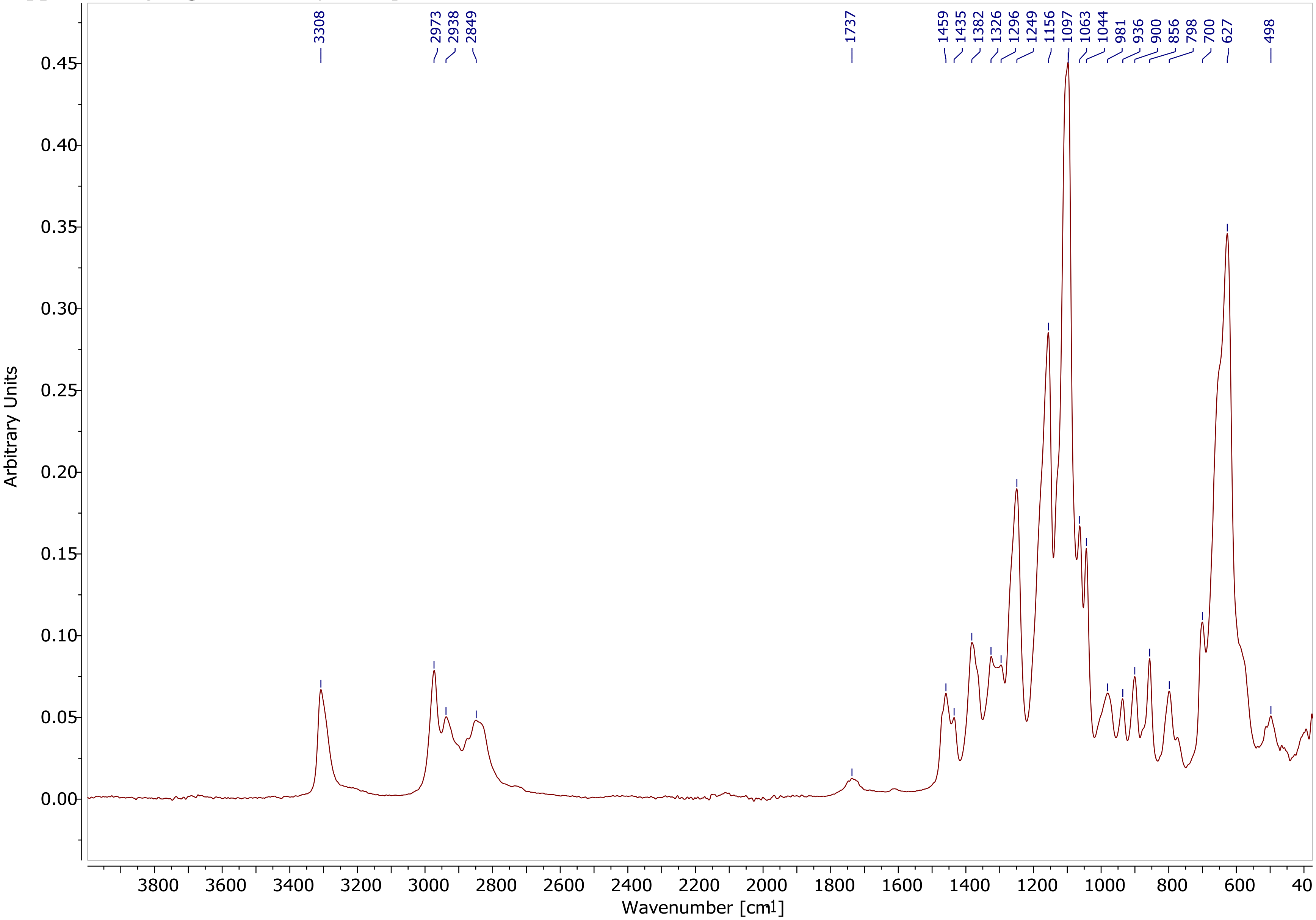

Supplementary Figure 156: *^{1}H-NMR for compound* ***29***

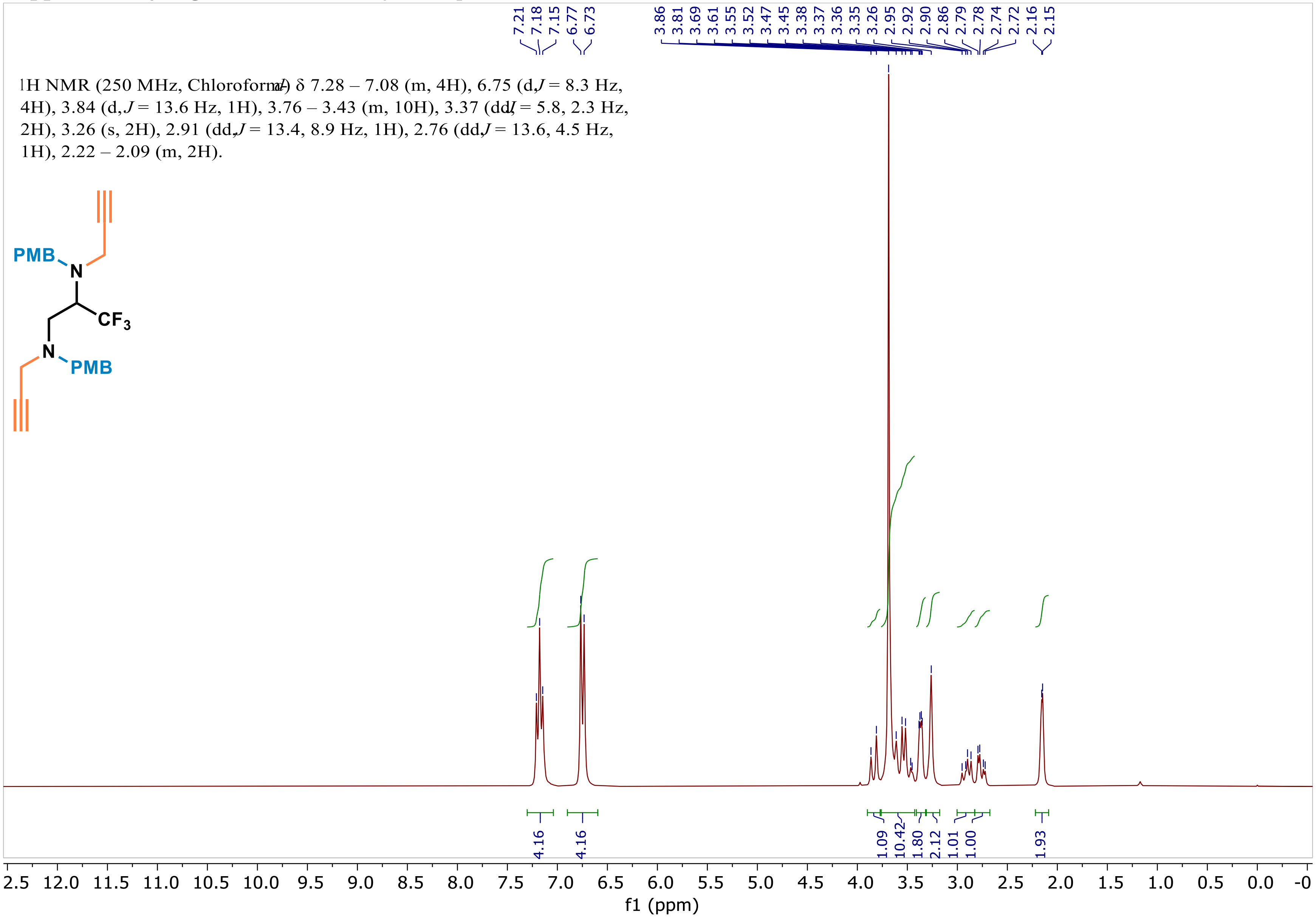

Supplementary Figure 157: *^{19}F NMR for compound* ***29***

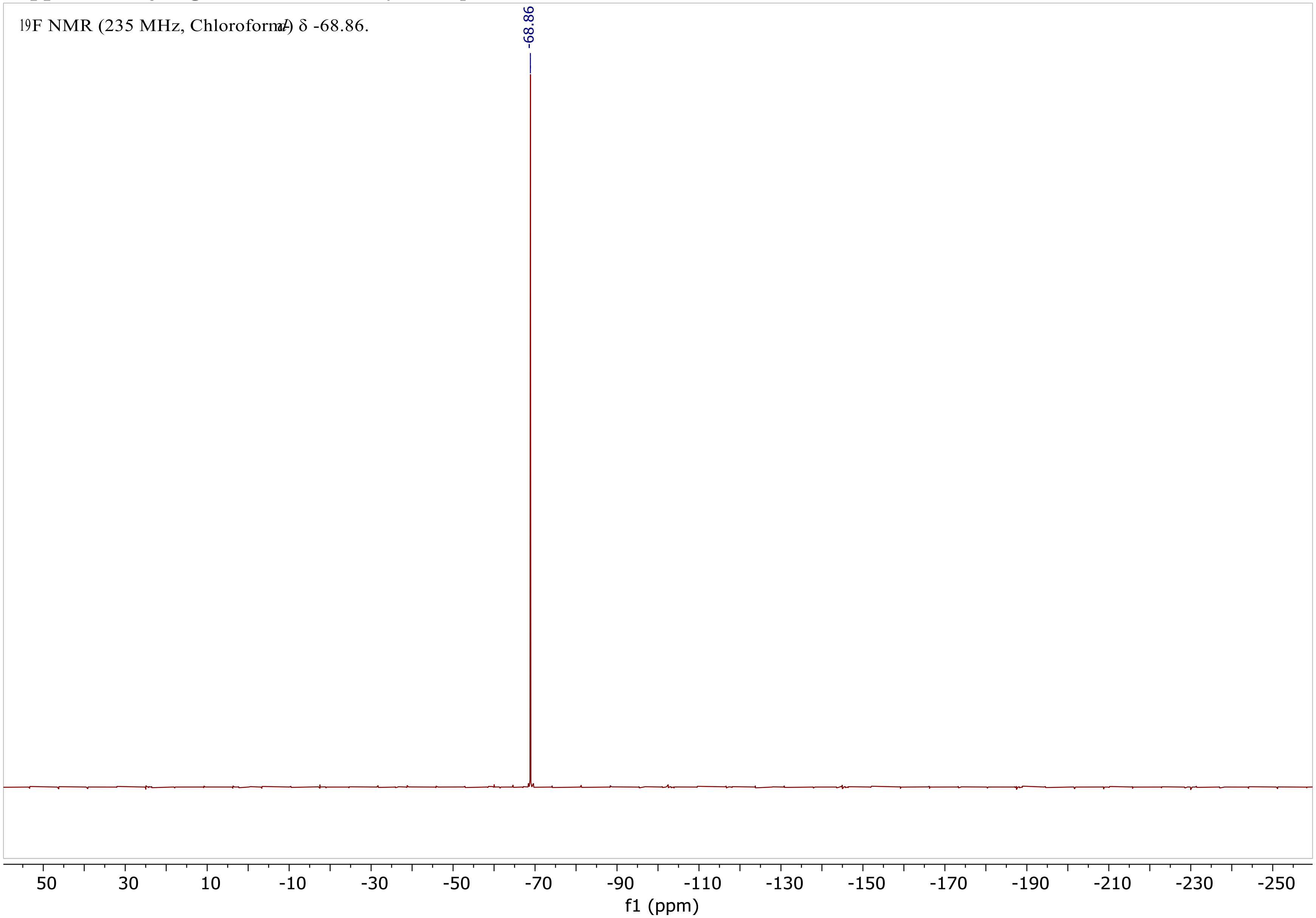

Supplementary Figure 158: *^{13}C NMR for compound* ***29***

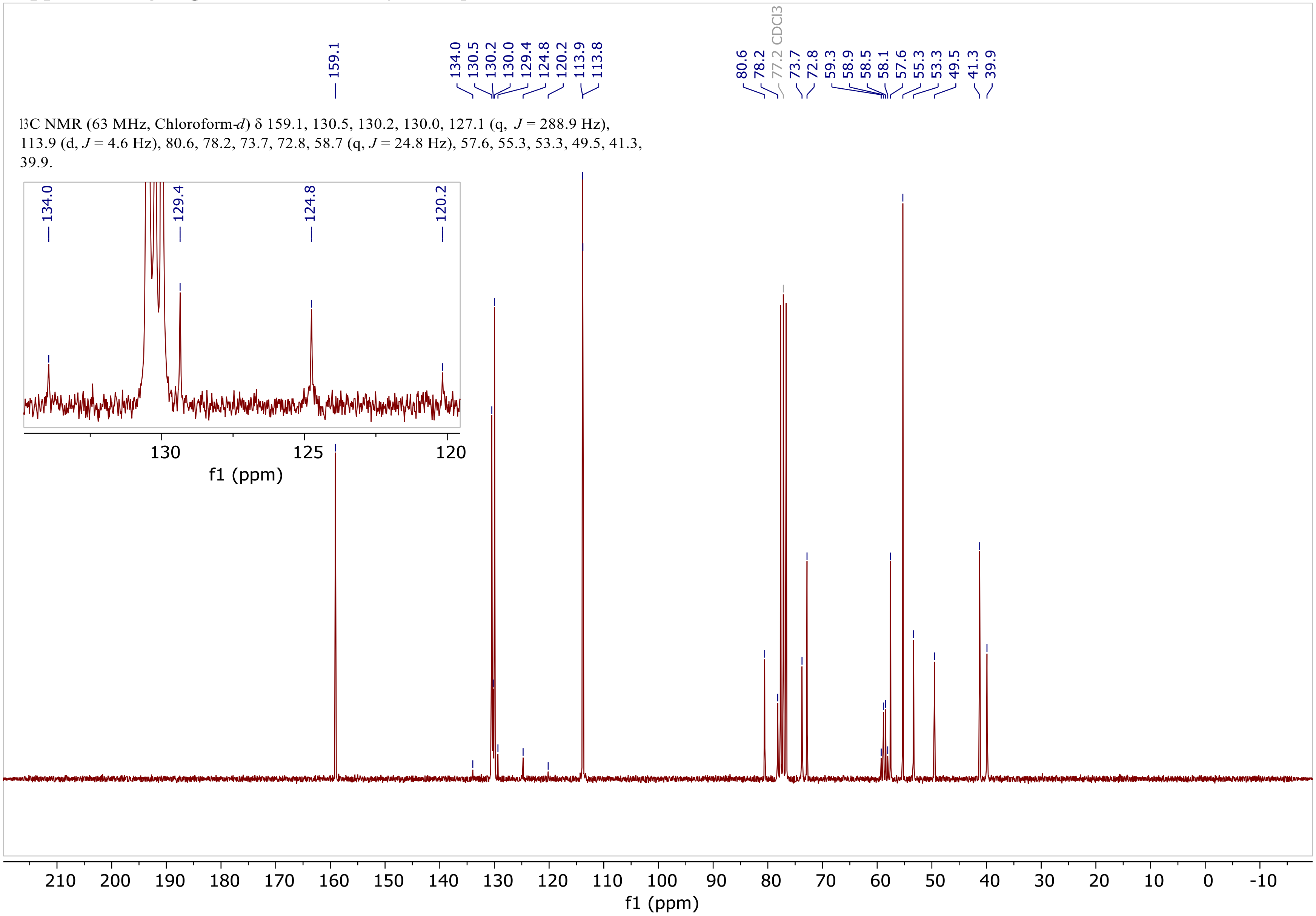

Supplementary Figure 159: *IR for compound **29***

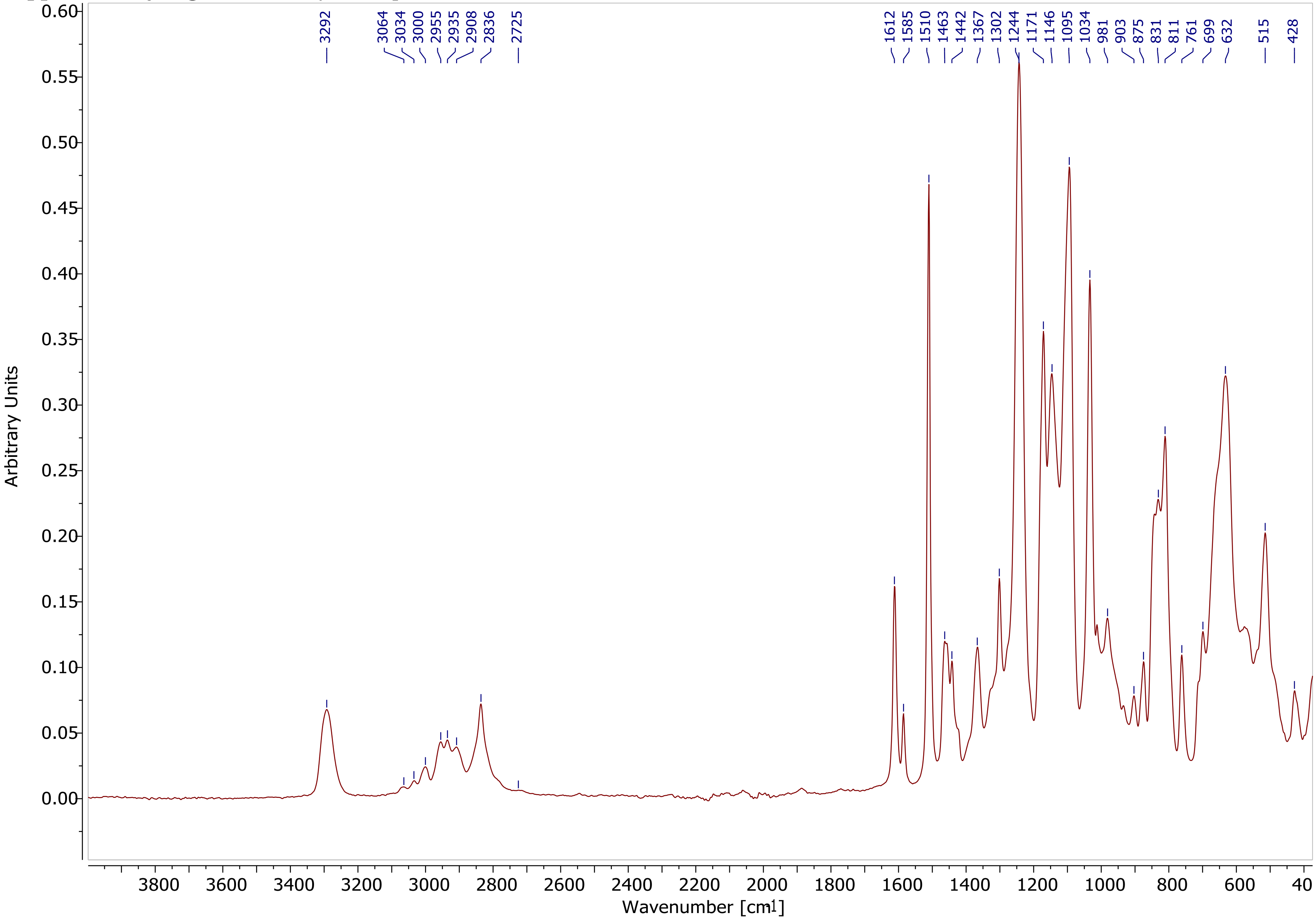

Supplementary Figure 160: ^{1}*H-NMR for compound **30***

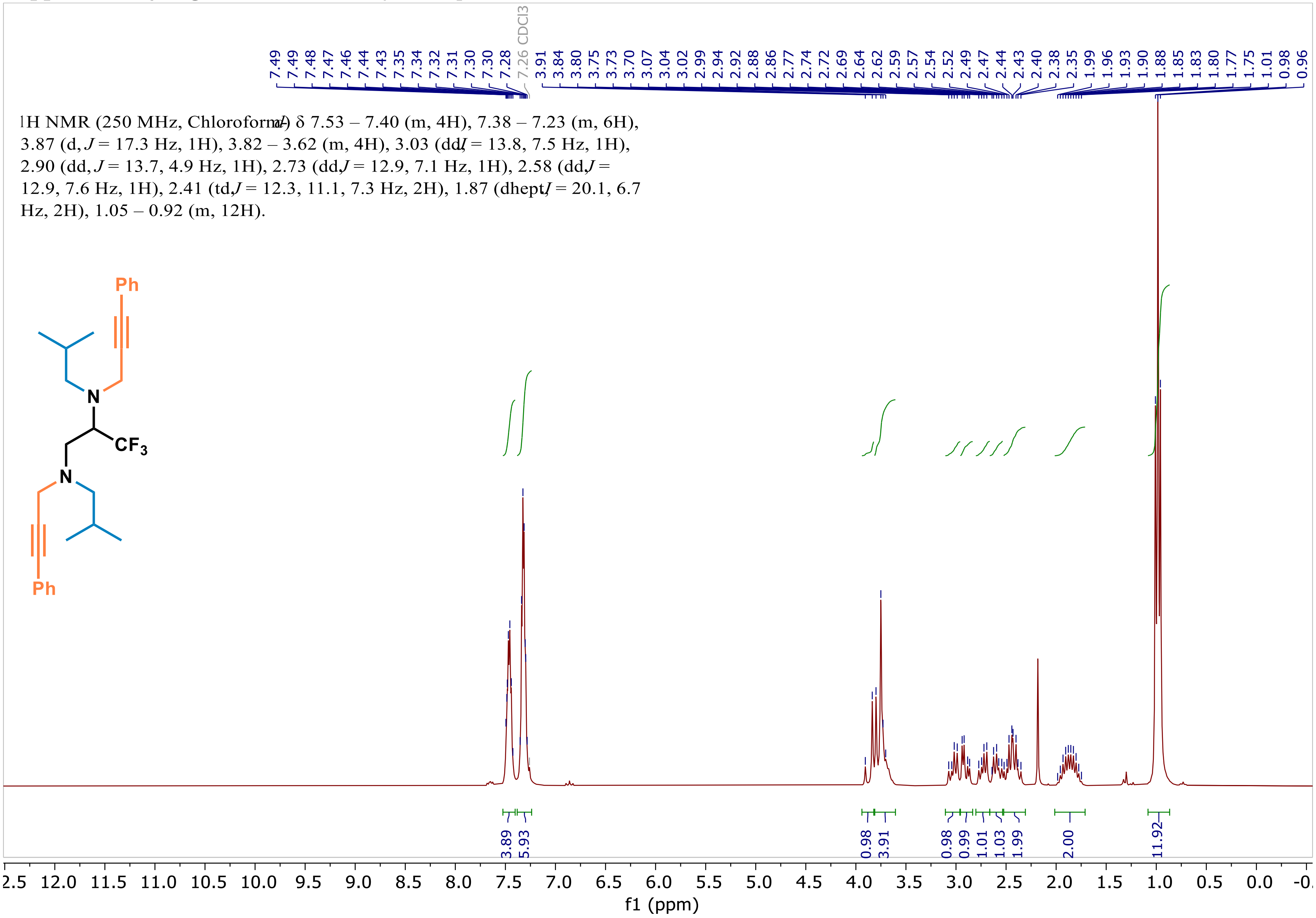

Supplementary Figure 161: *^{19}F NMR for compound* ***30***

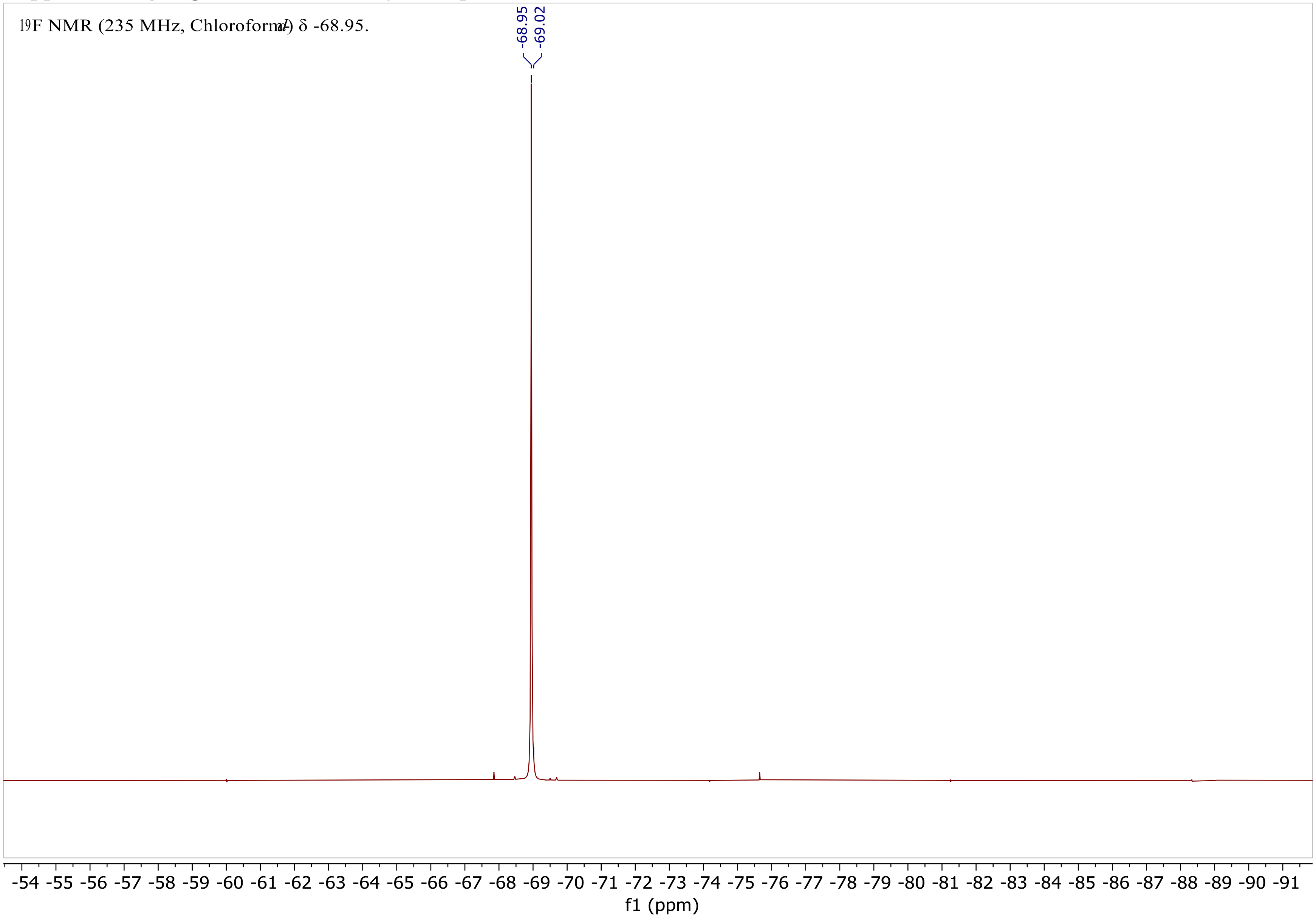

Supplementary Figure 162: *^{13}C NMR for compound* ***30***

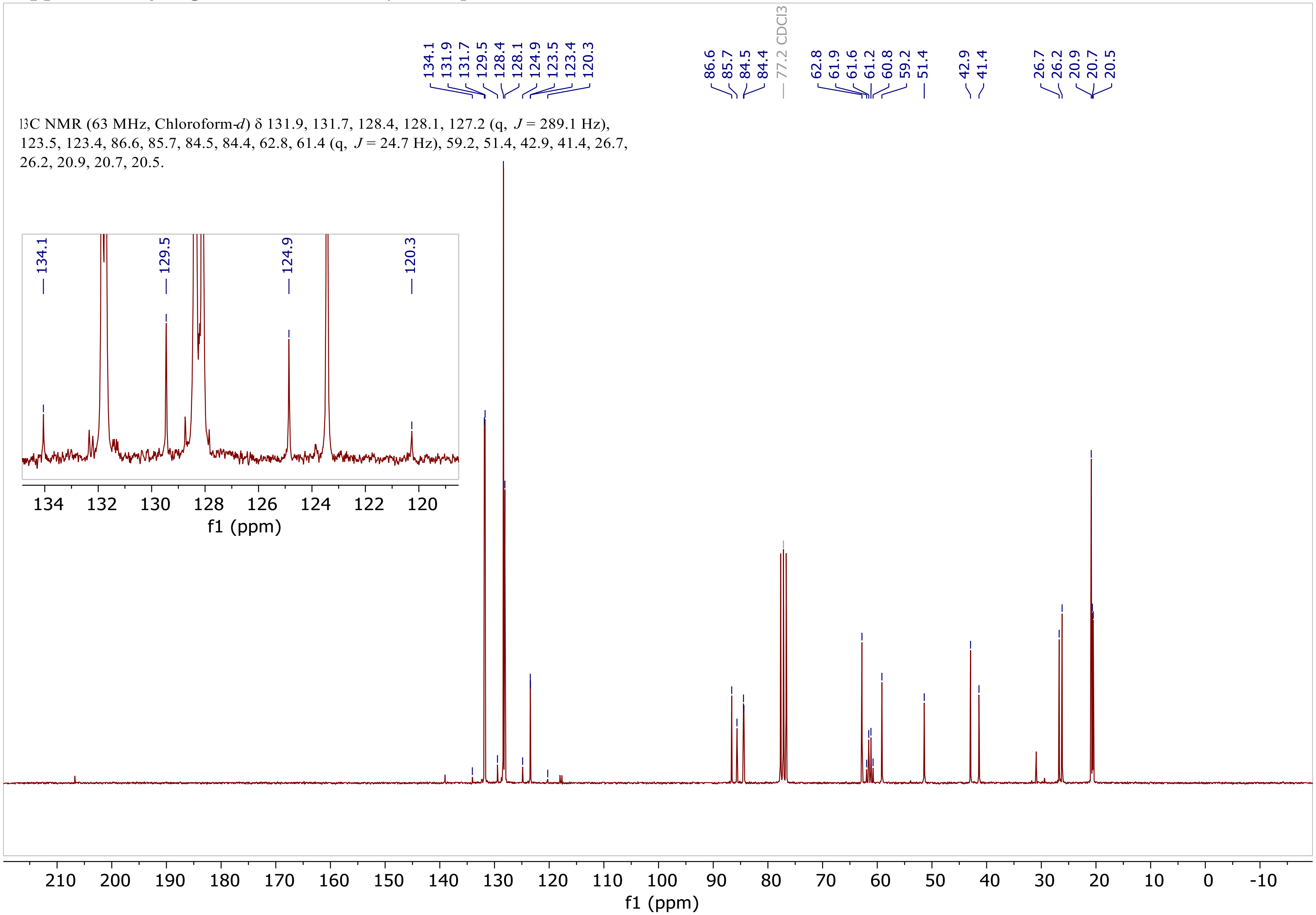

Supplementary Figure 163: *IR for compound **30***

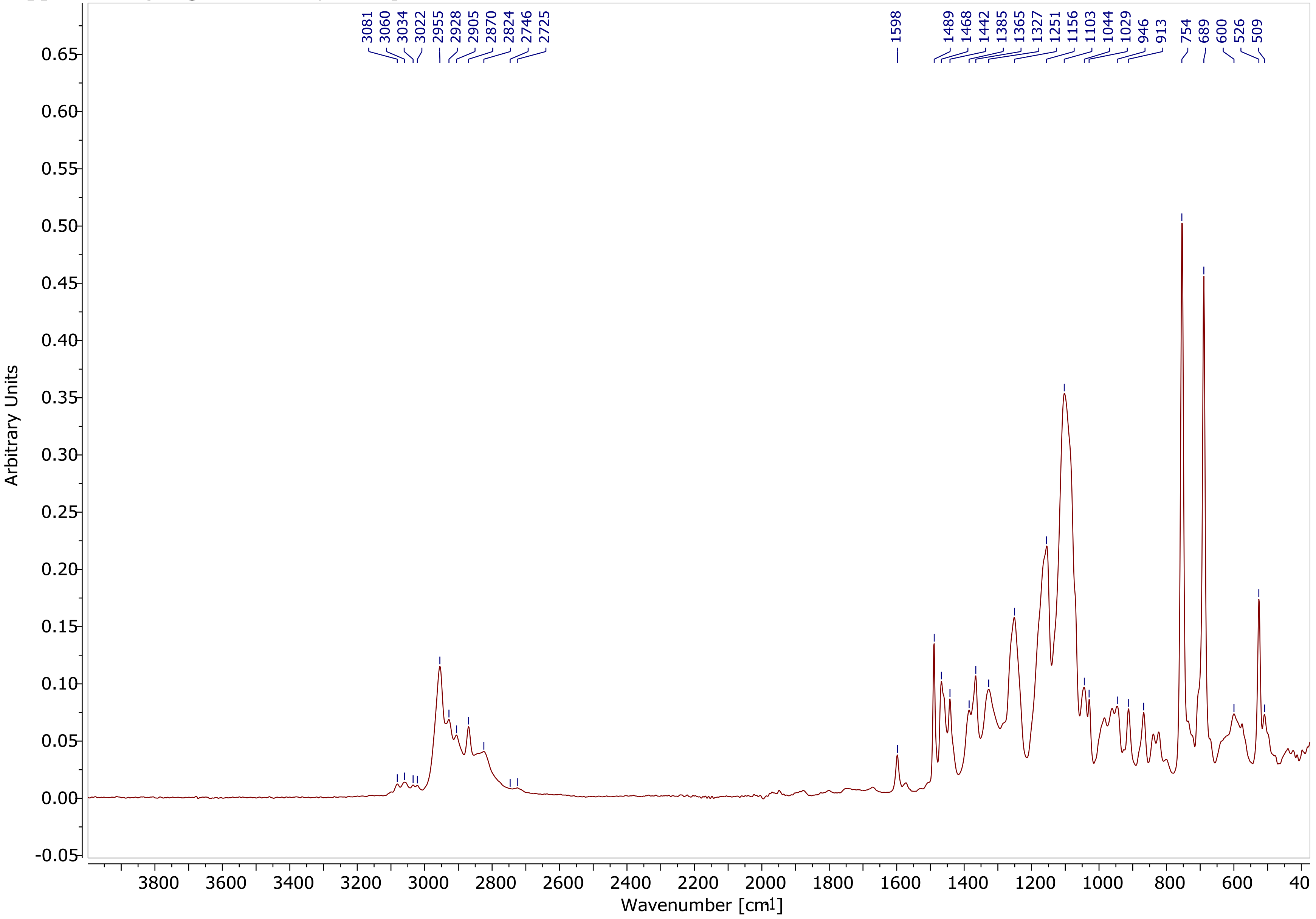

Supplementary Figure 164: *^{1}H-NMR for compound* ***31***

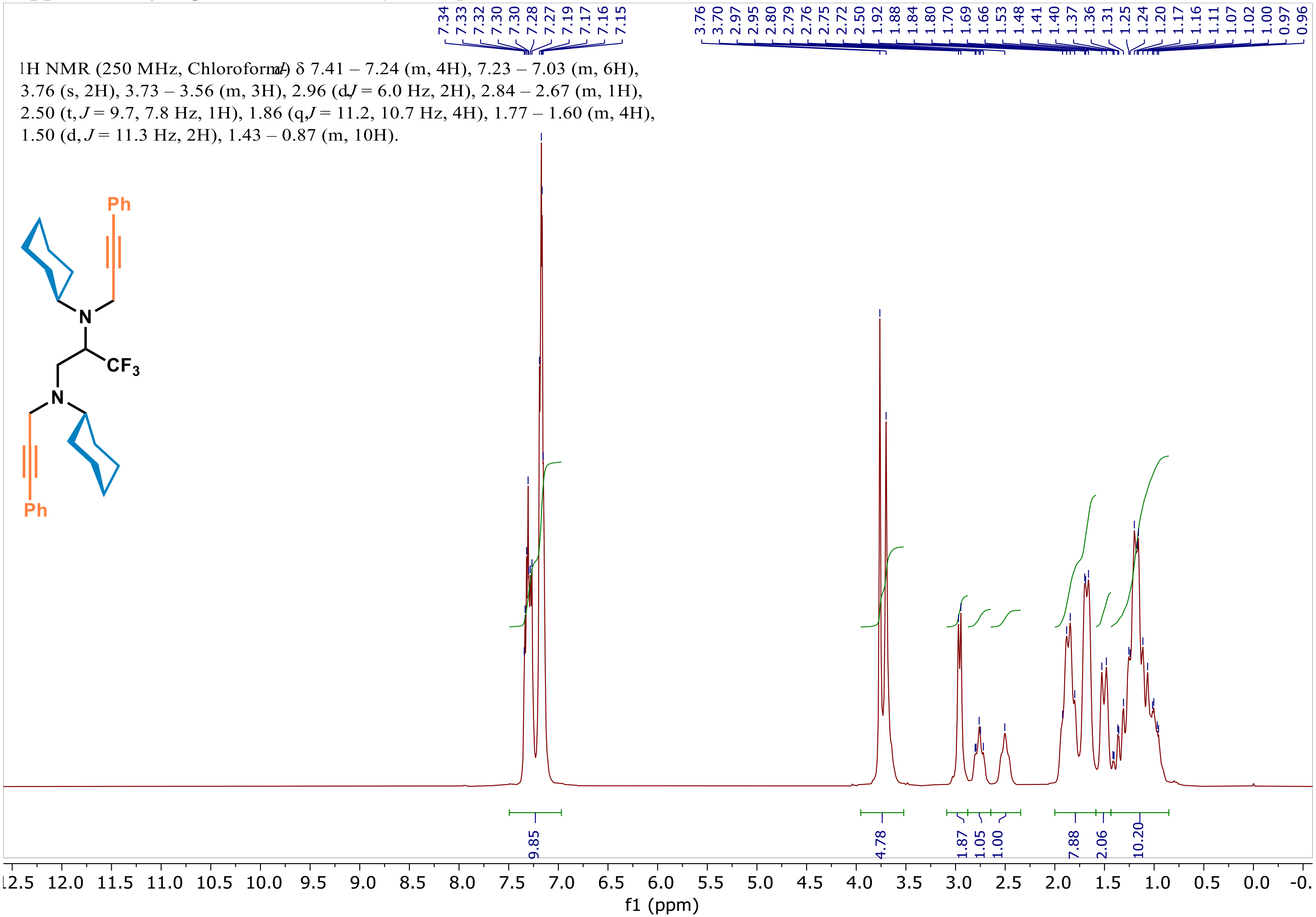

Supplementary Figure 165: *^{19}F NMR for compound* ***31***

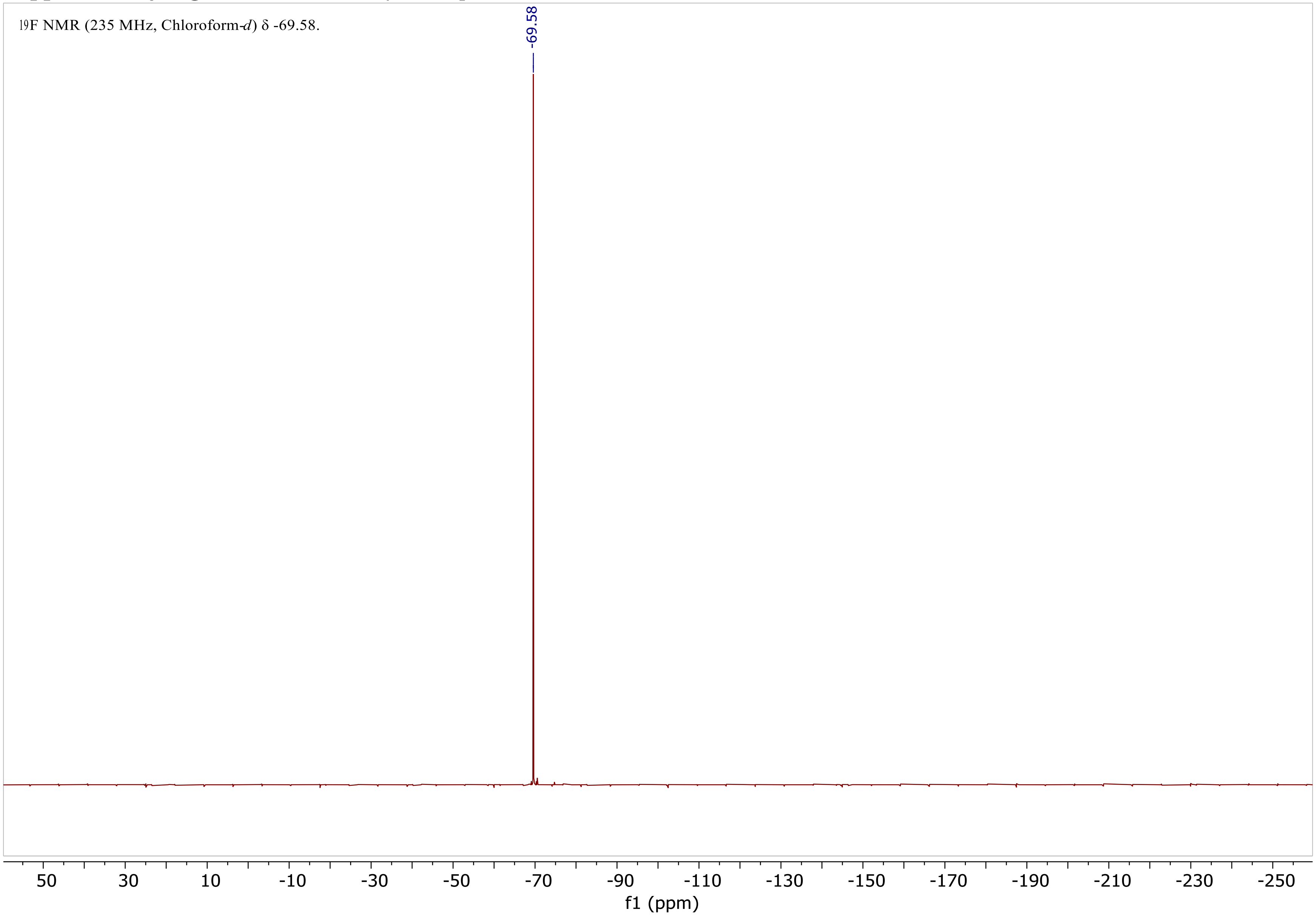

Supplementary Figure 166: *^{13}C NMR for compound* ***31***

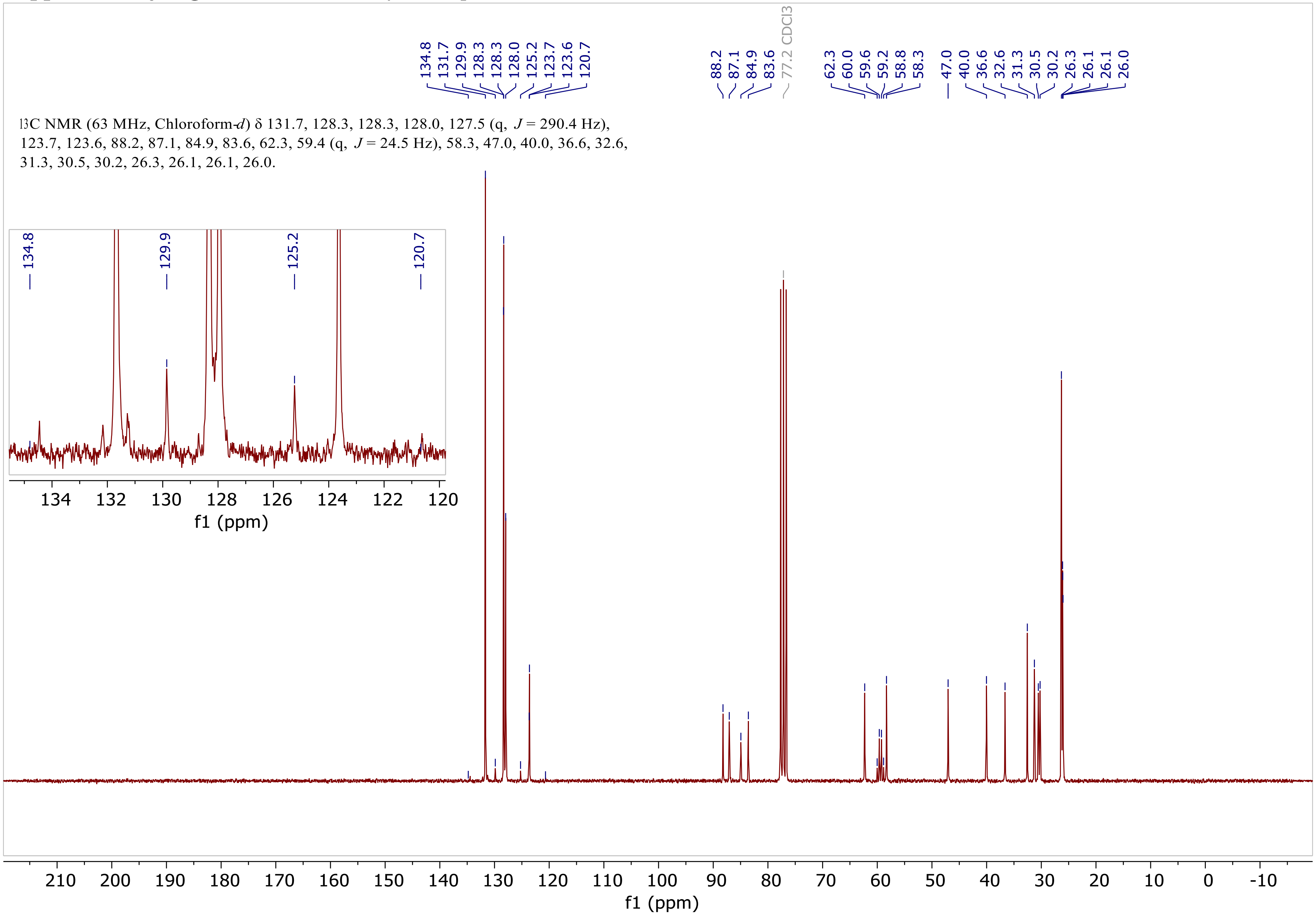

Supplementary Figure 167: *IR for compound **31***

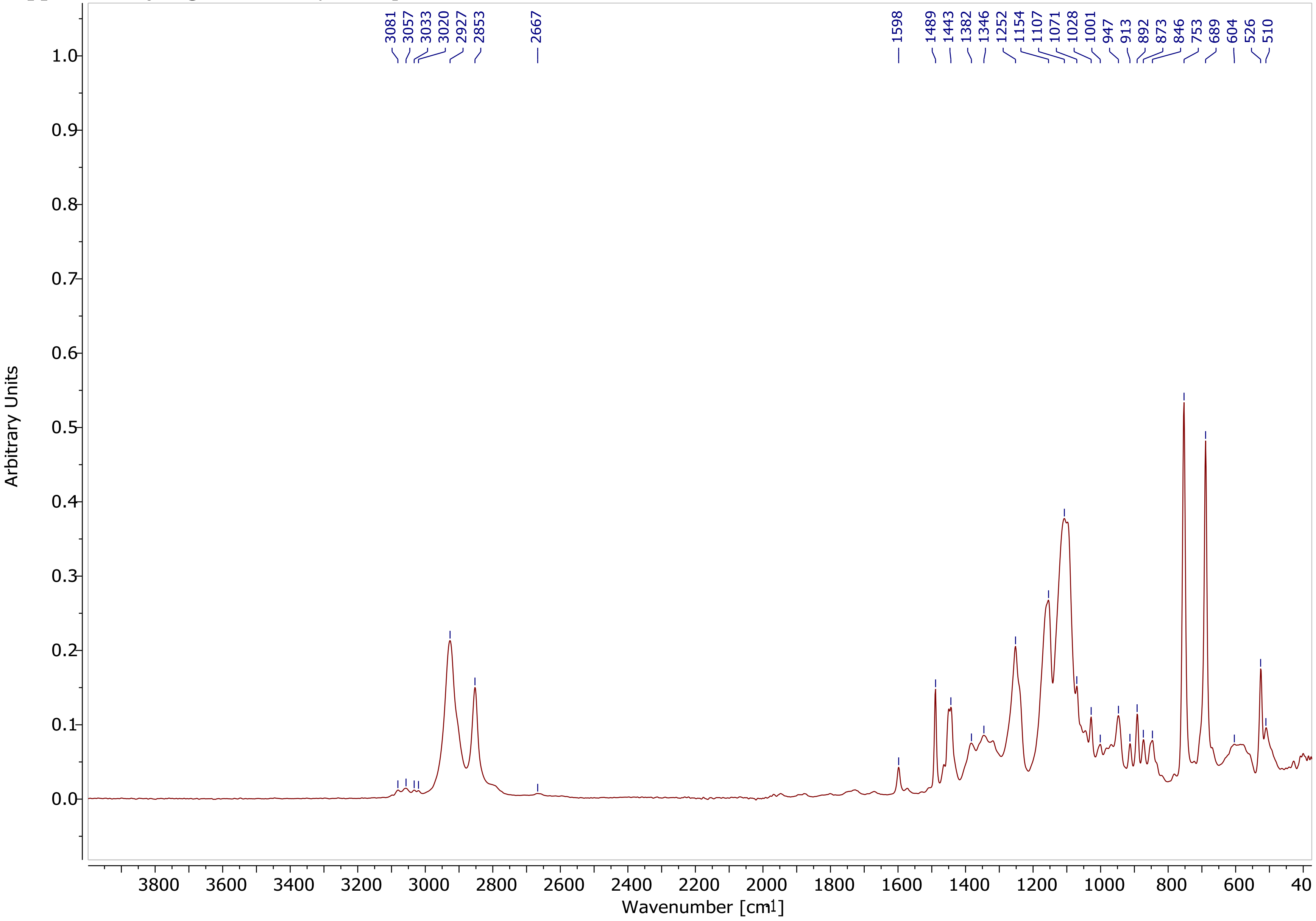

Supplementary Figure 168: *¹H-NMR for compound **32***

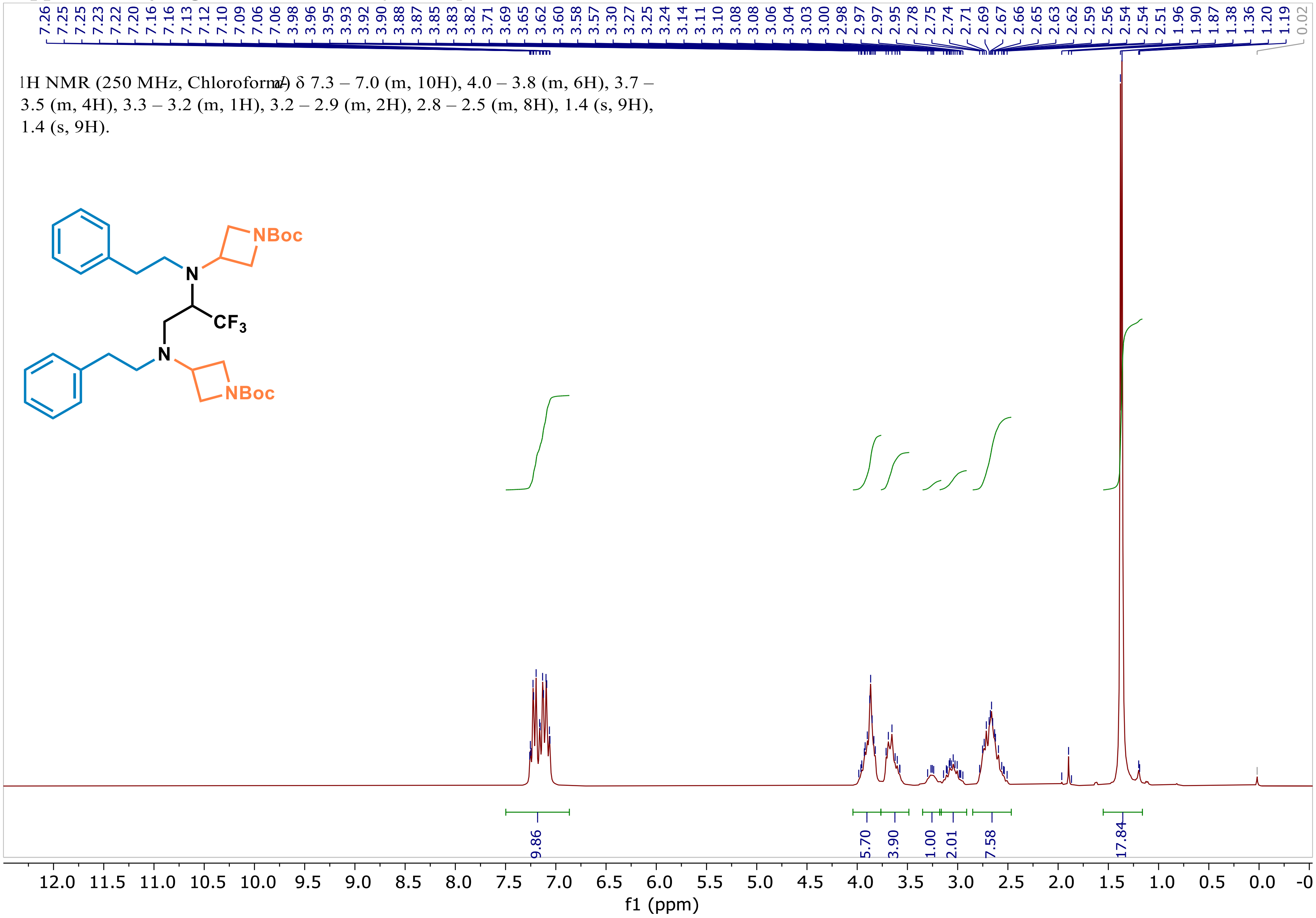

Supplementary Figure 169: *^{19}F NMR for compound* ***32***

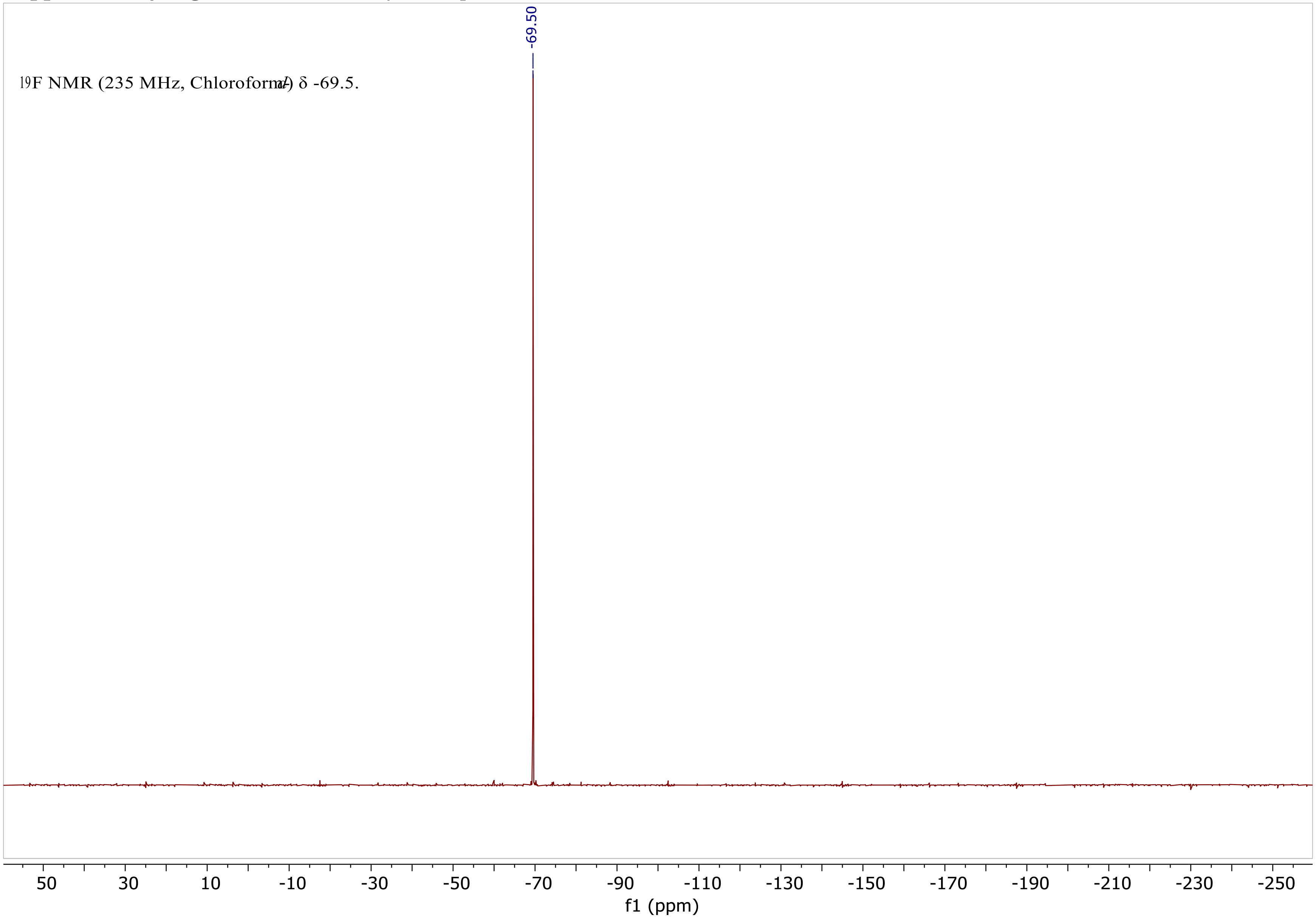

Supplementary Figure 170: *^{13}C NMR for compound* ***32***

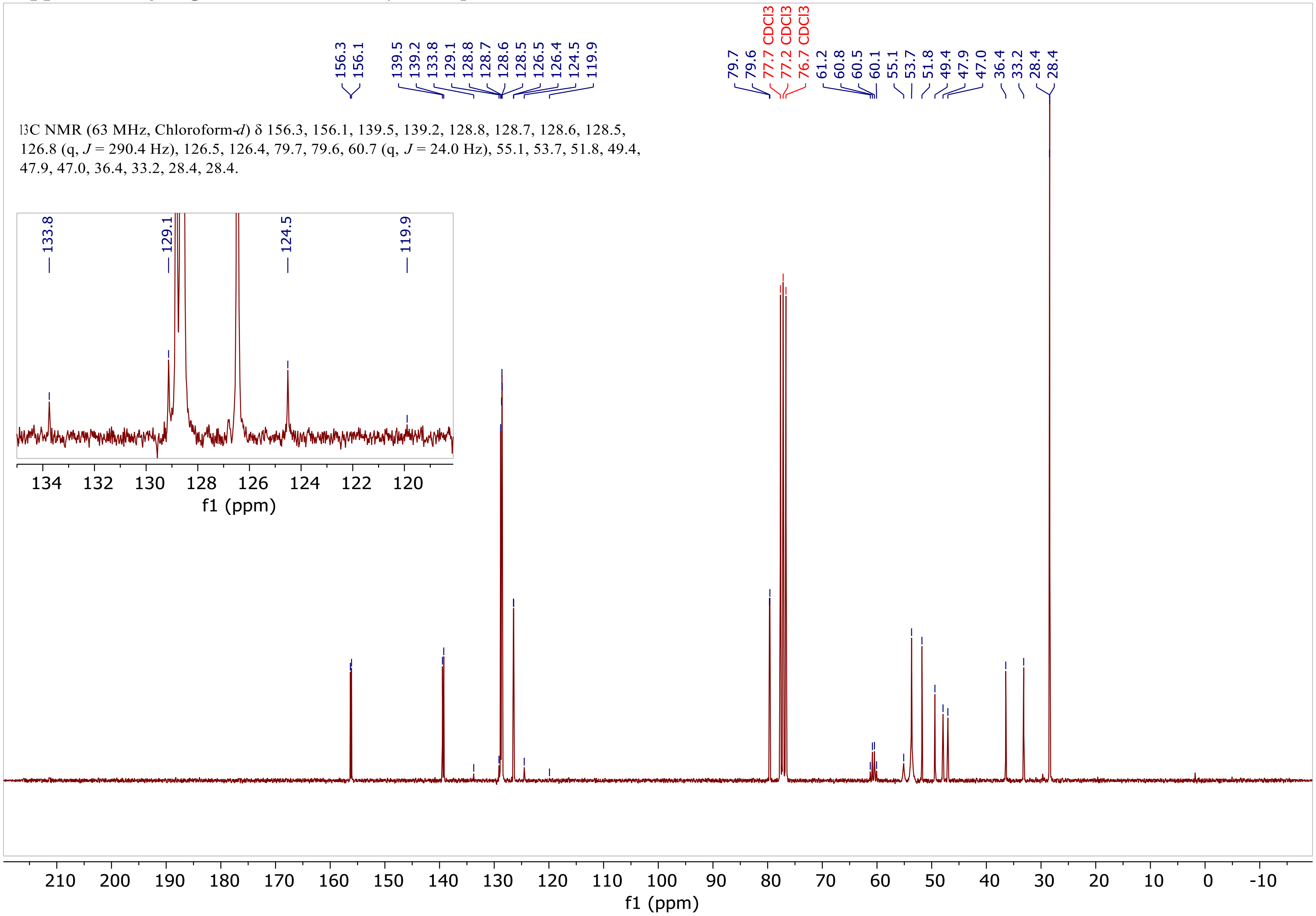

Supplementary Figure 171: *IR for compound* ***32***

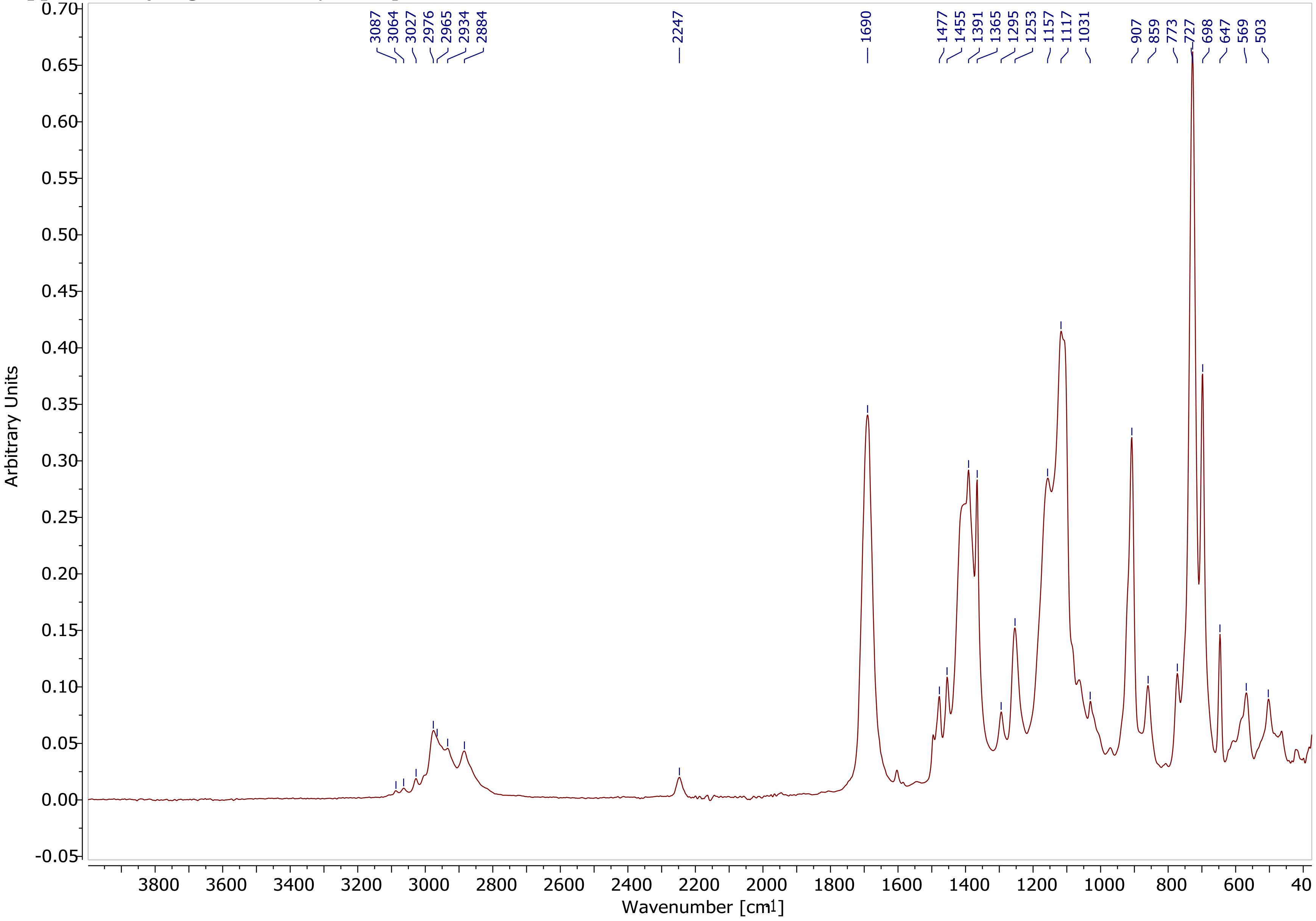

Supplementary Figure 172: *^{1}H-NMR for compound **33***

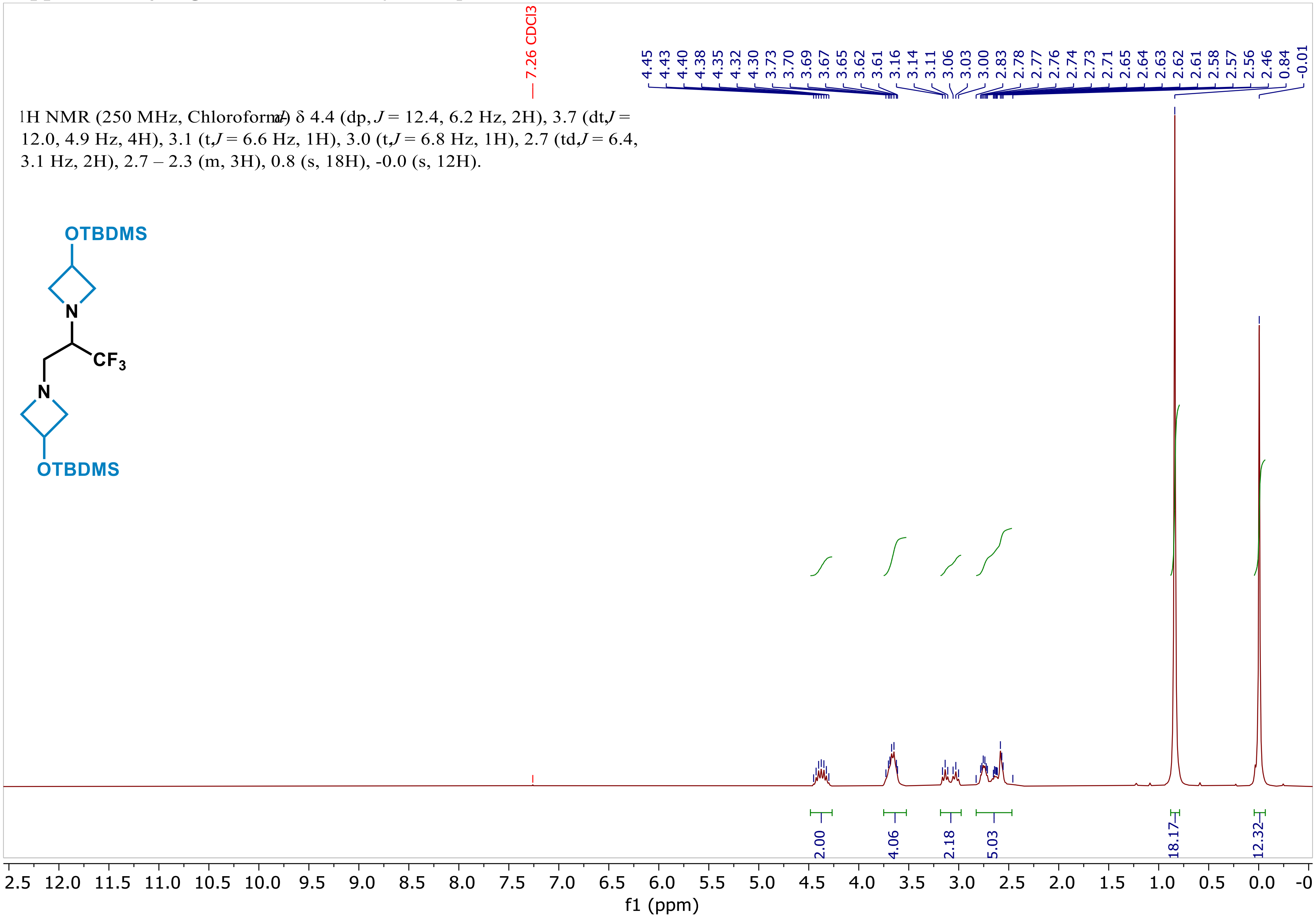

Supplementary Figure 173: *^{19}F NMR for compound **33***

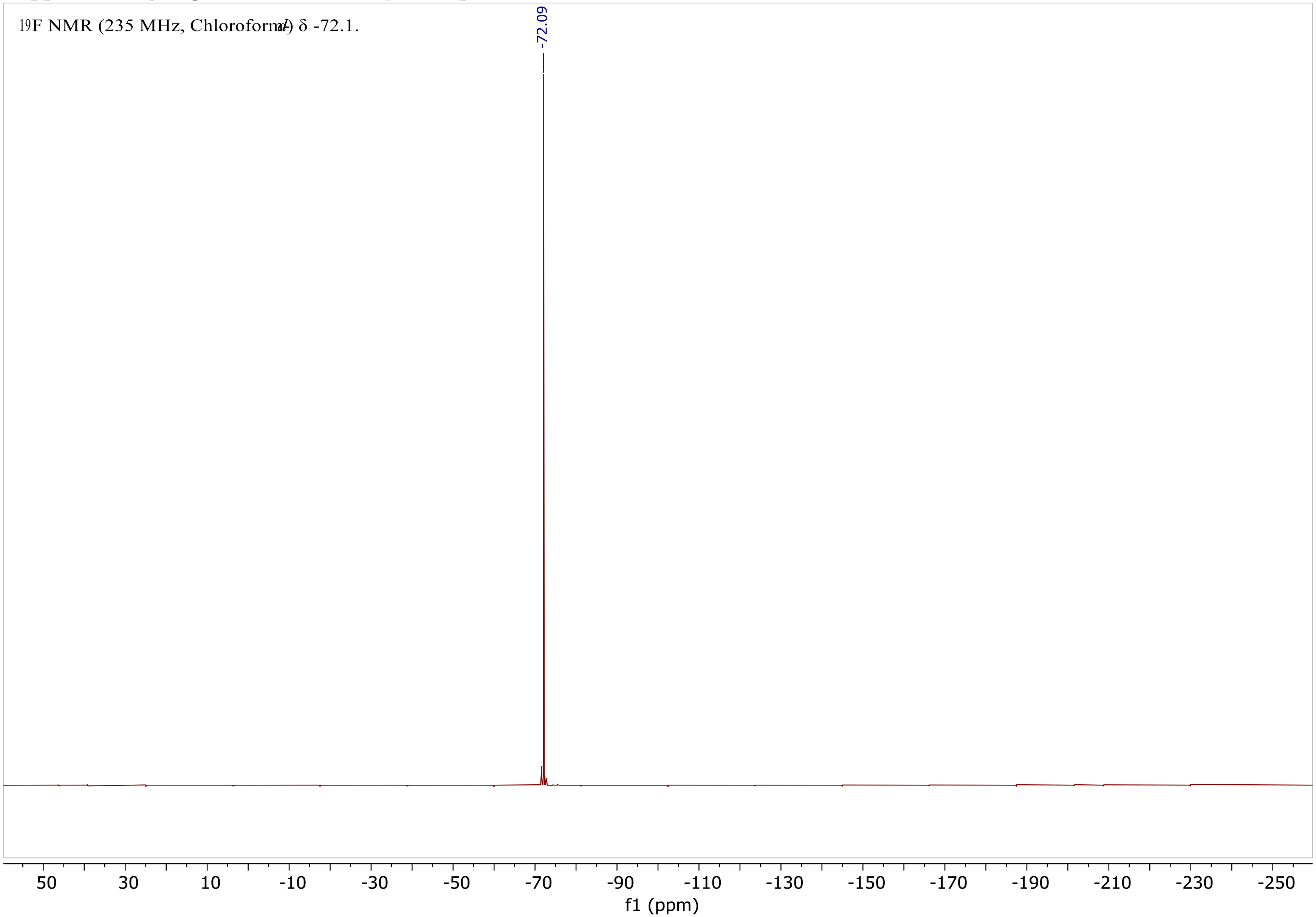

Supplementary Figure 174: *^{13}C NMR for compound **33***

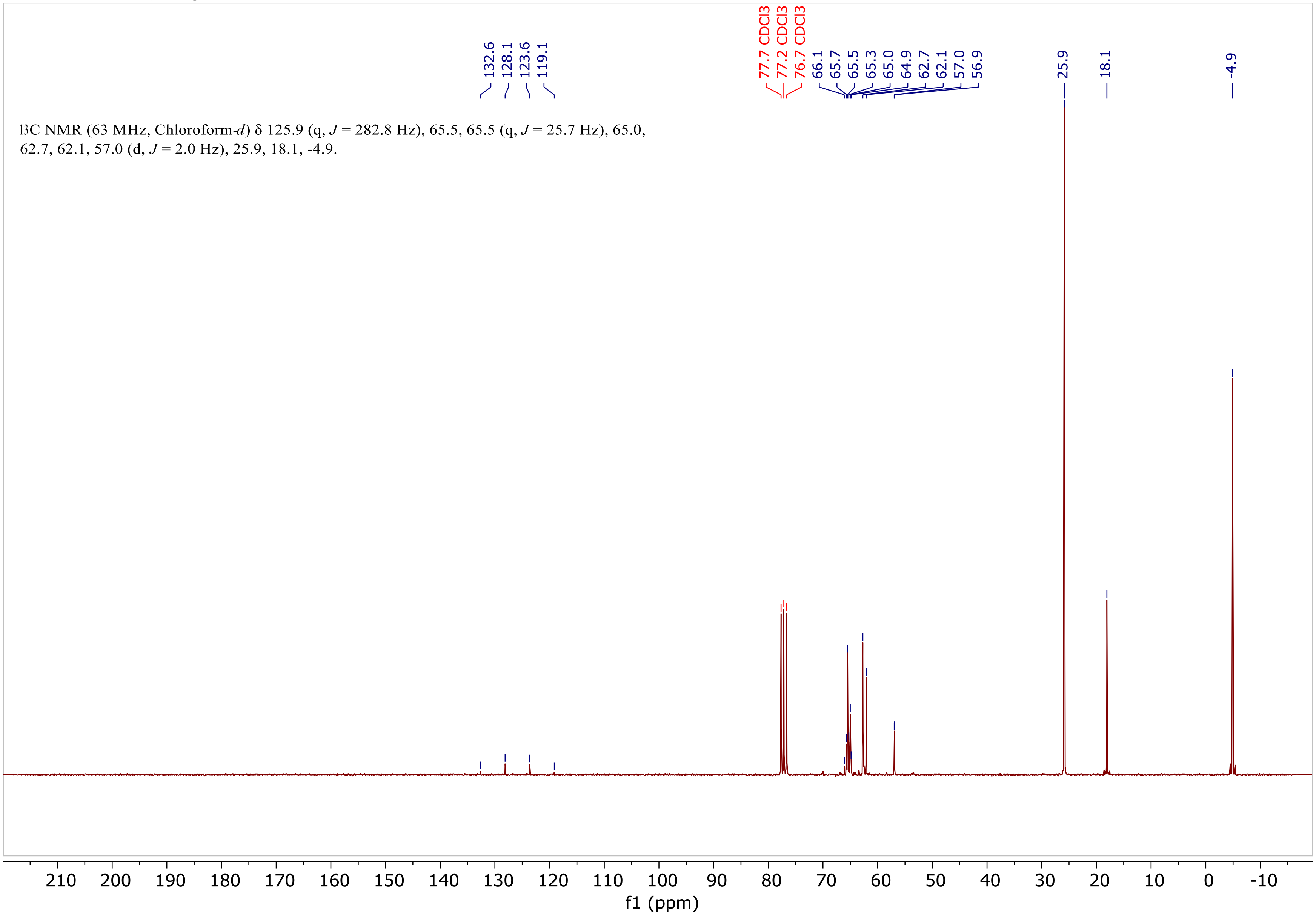

Supplementary Figure 175: *IR for compound **33***

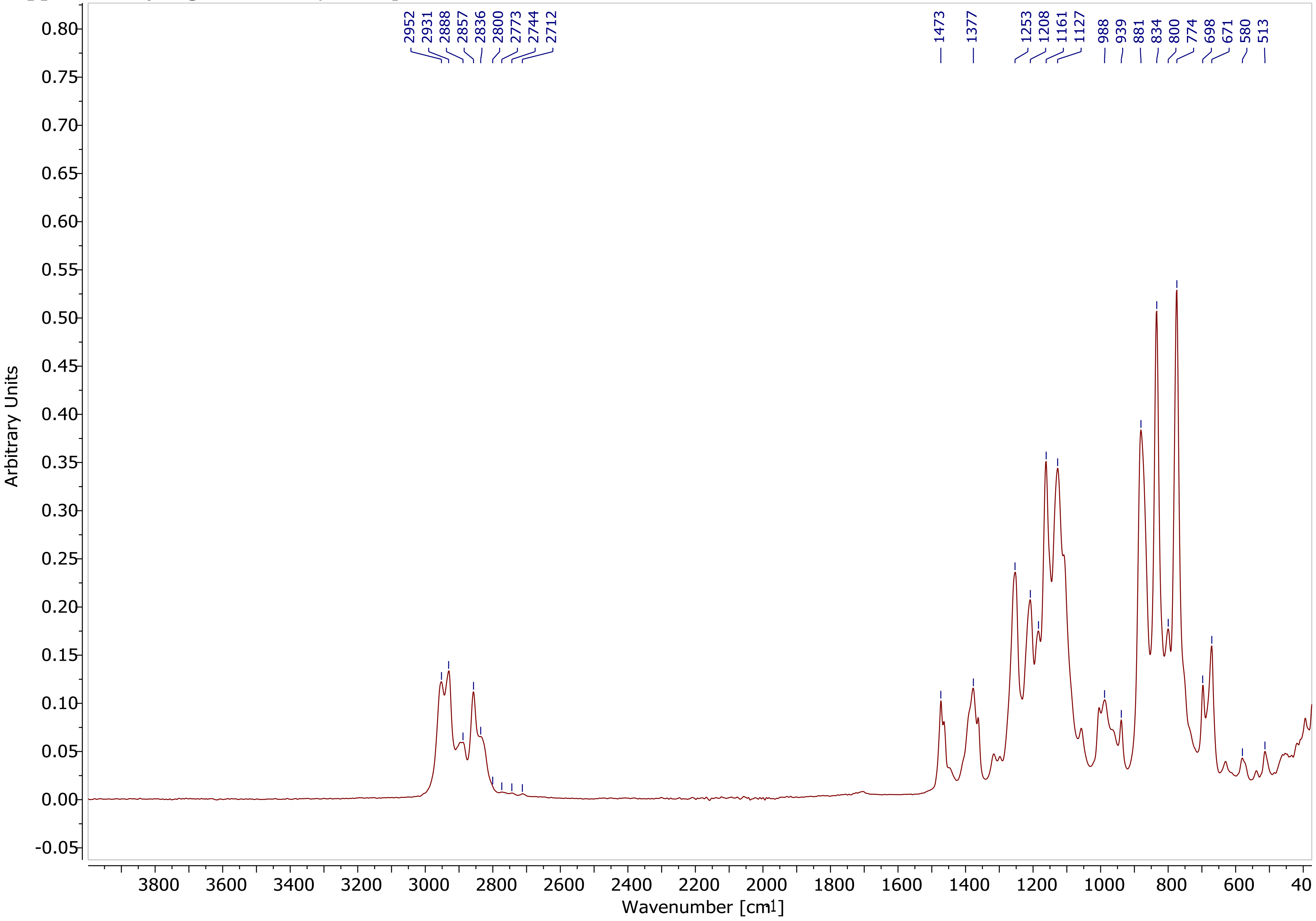

Supplementary Figure 176: *^{1}H-NMR for compound **34***

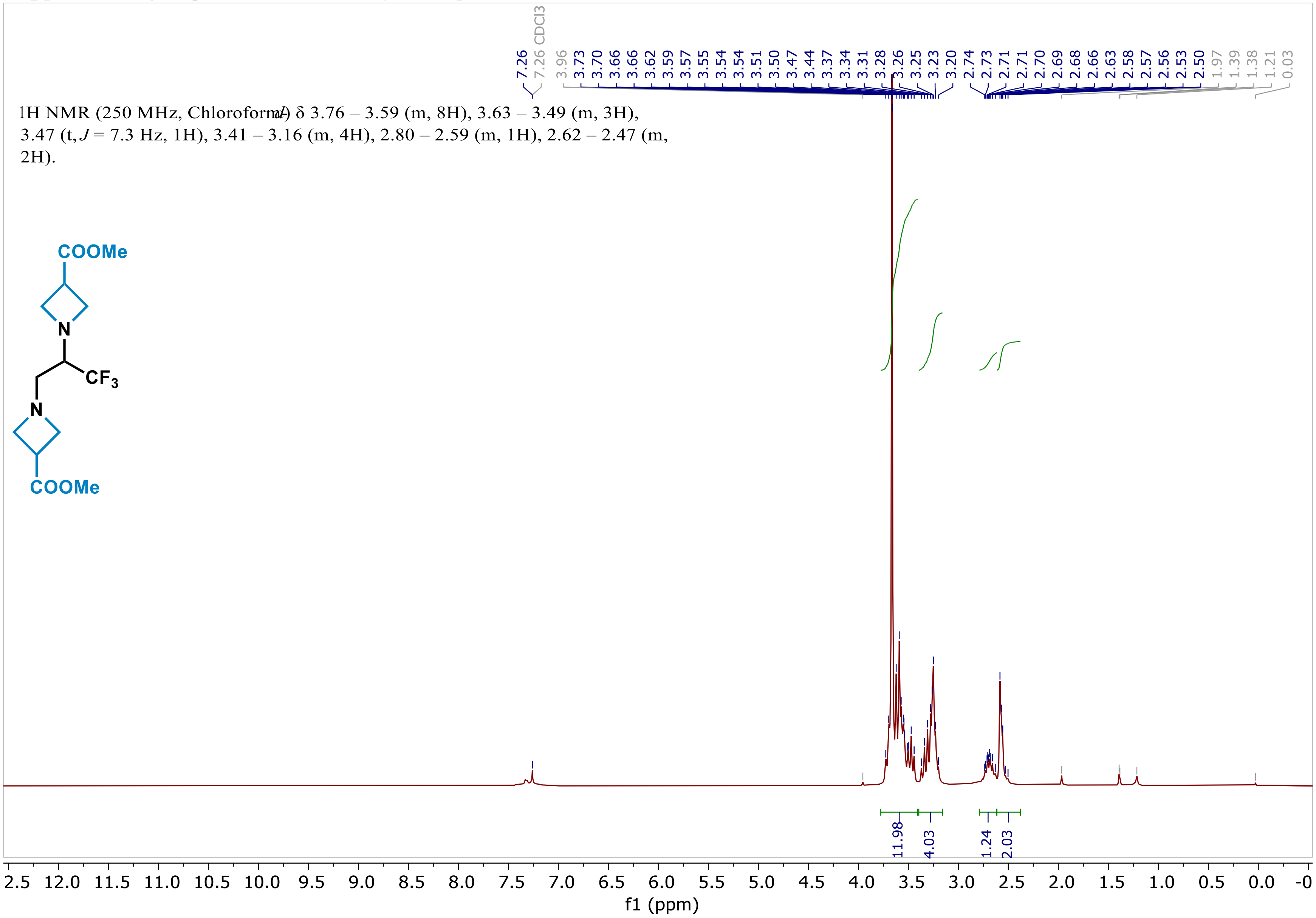

Supplementary Figure 177: *^{19}F NMR for compound **34***

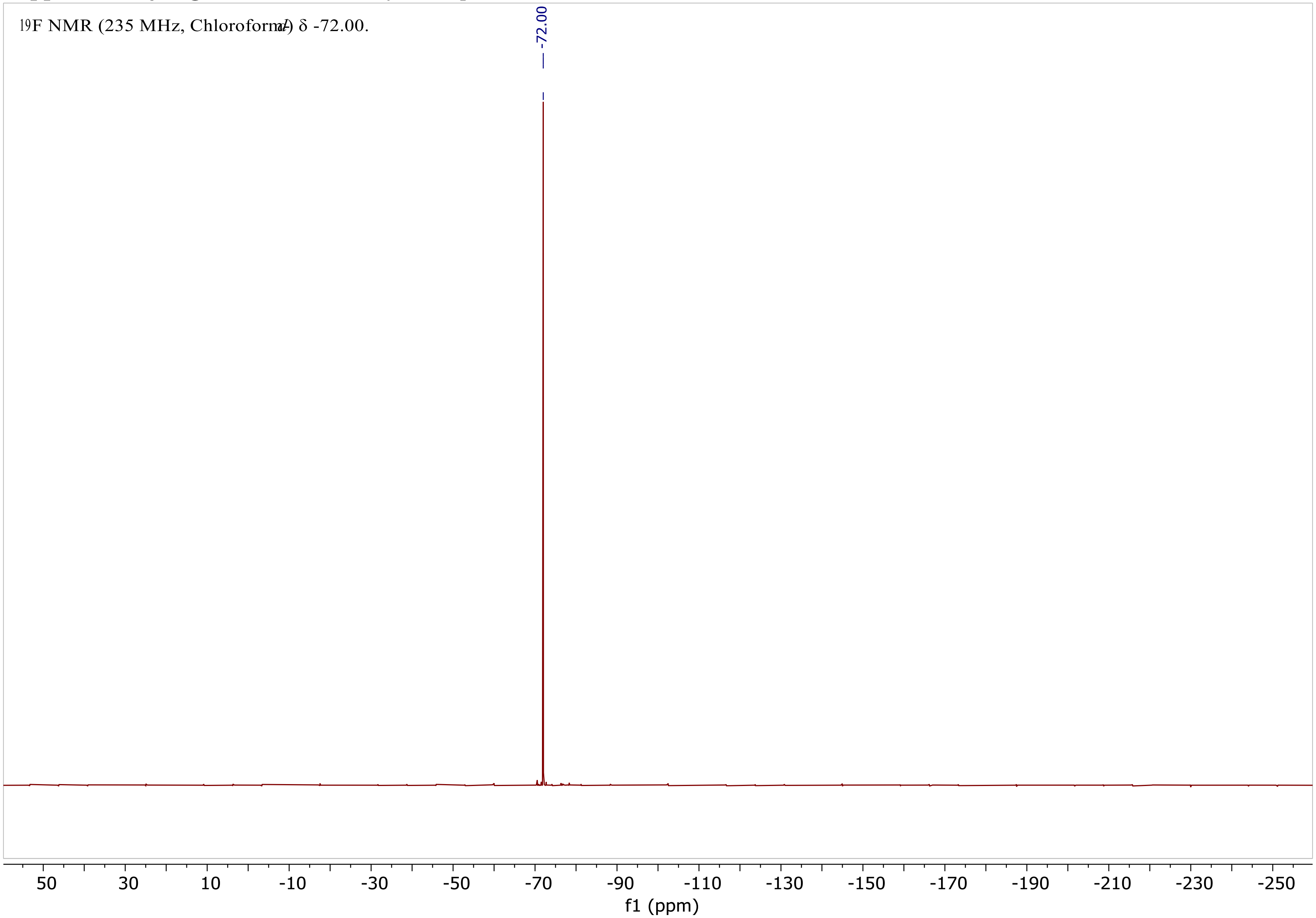

Supplementary Figure 178: *^{13}C NMR for compound* ***34***

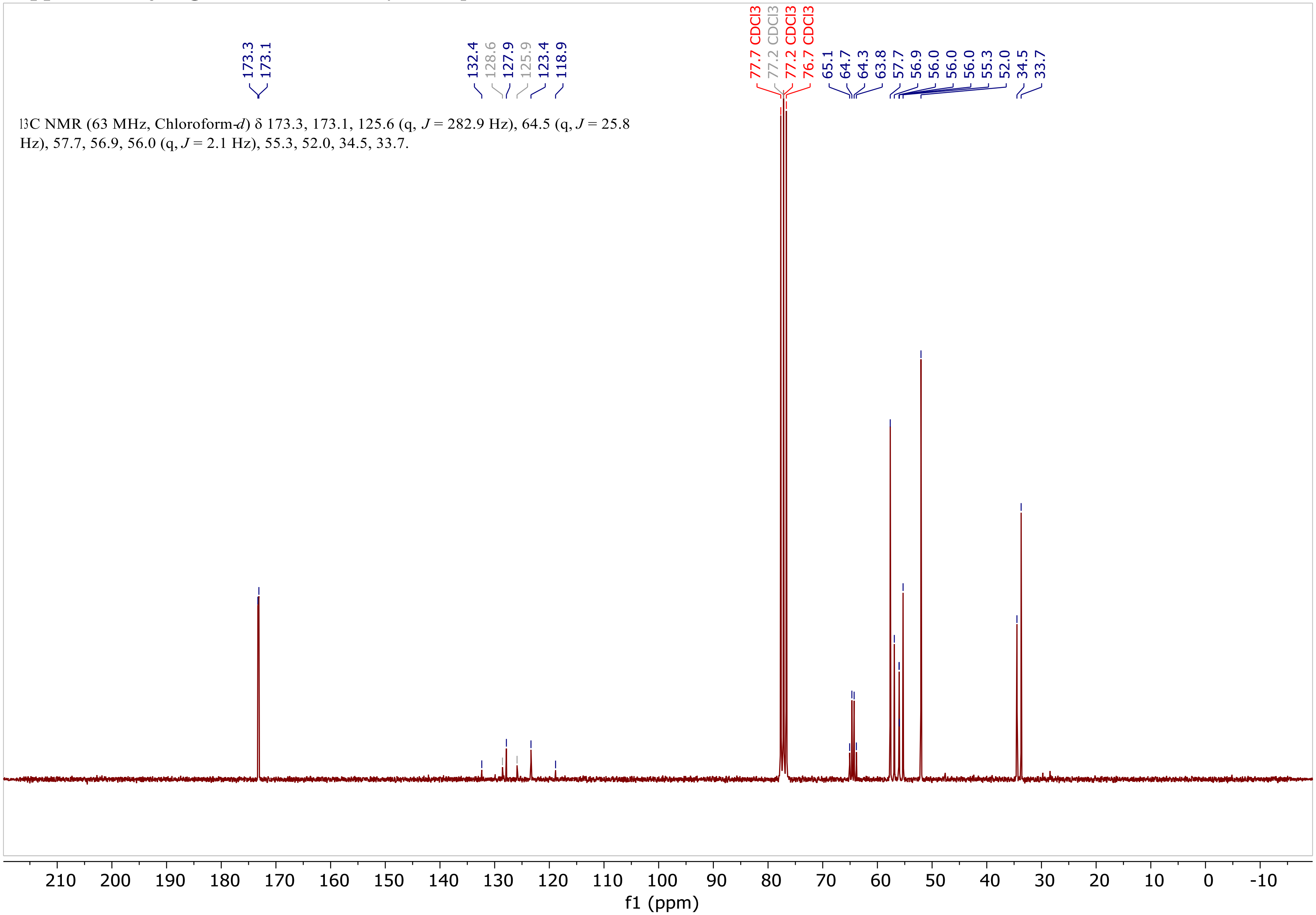

Supplementary Figure 179: *IR for compound **34***

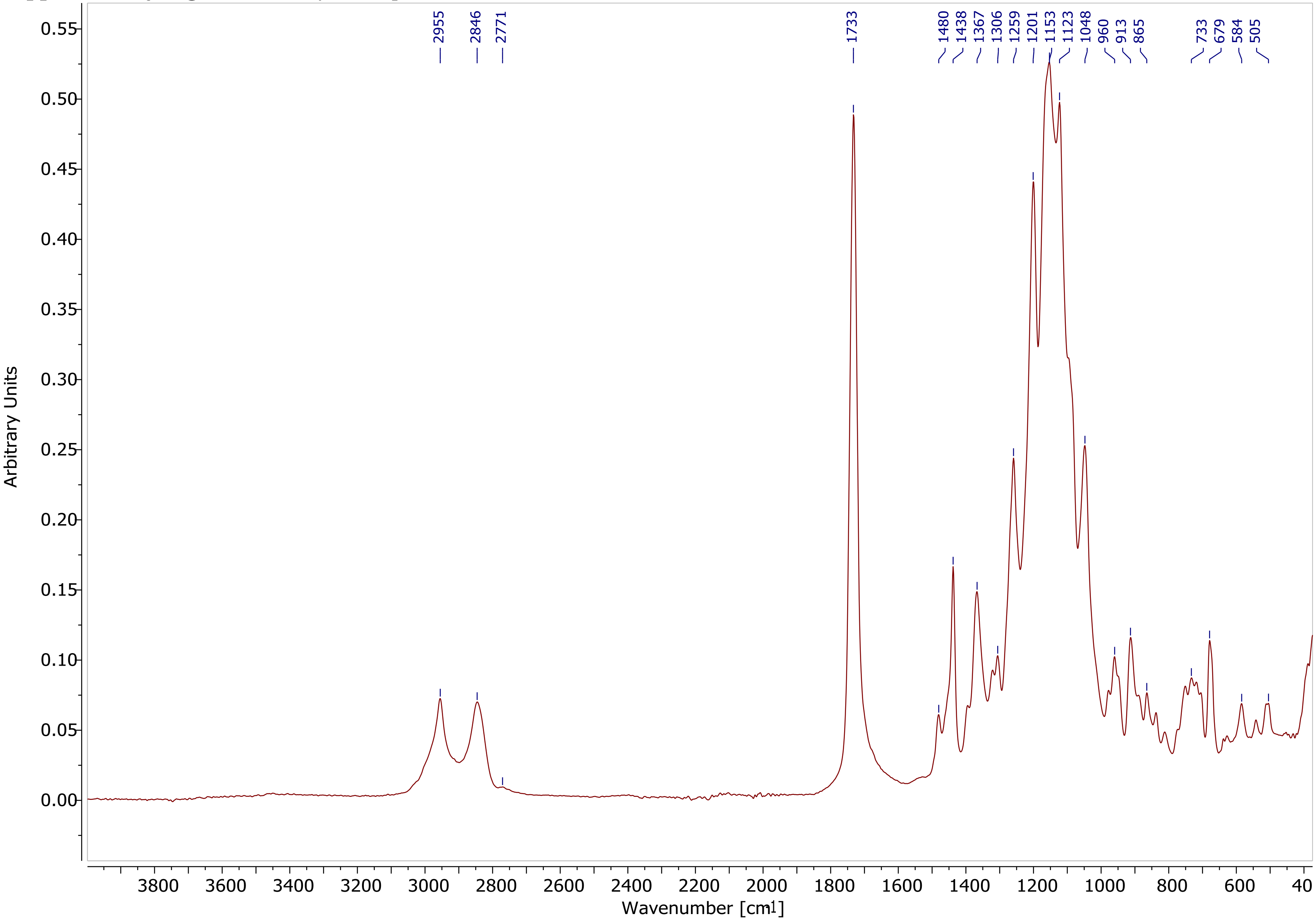

Supplementary Figure 180: *¹H-NMR for compound* ***35***

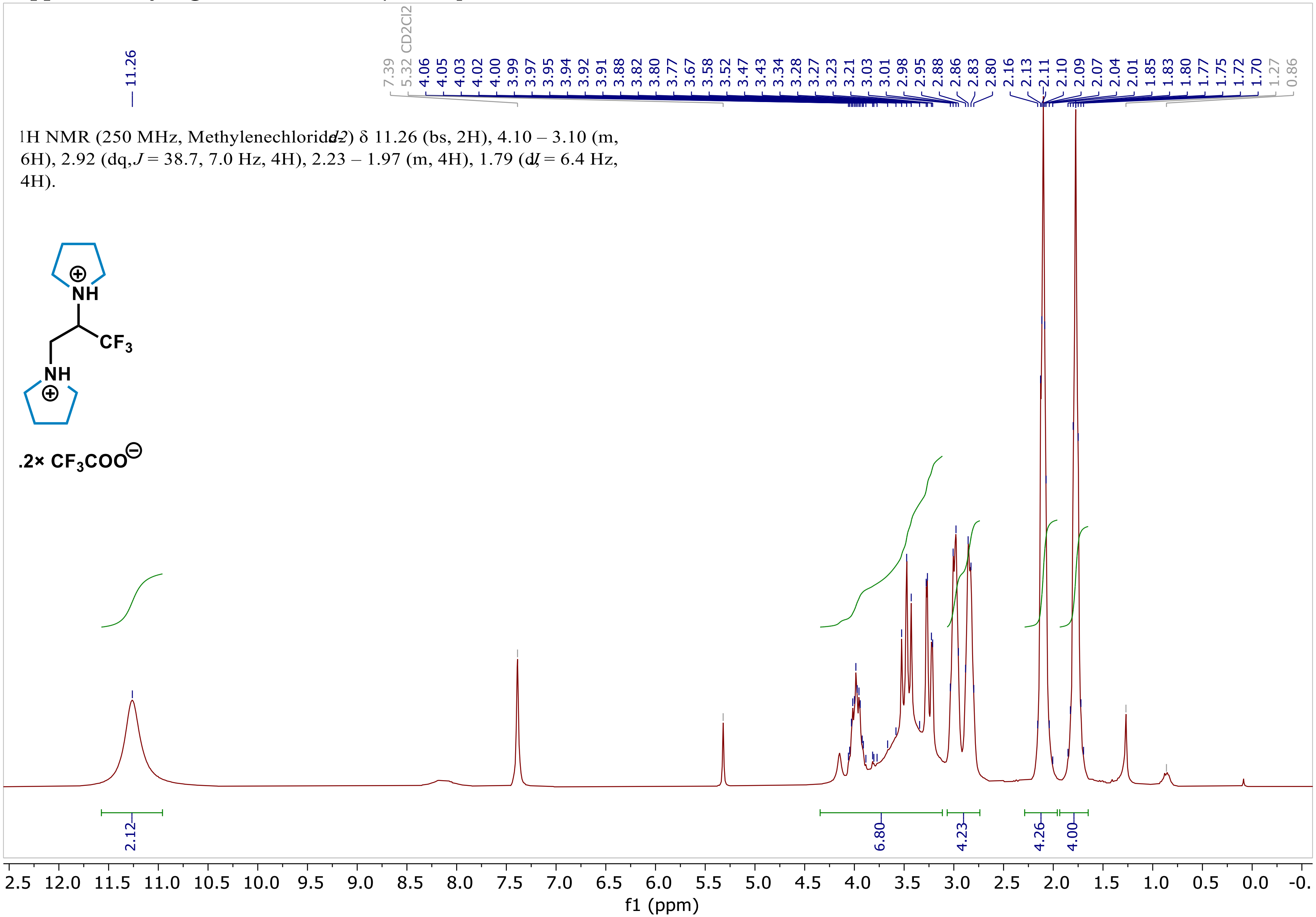

Supplementary Figure 181: ^{19}*F NMR for compound* ***35***

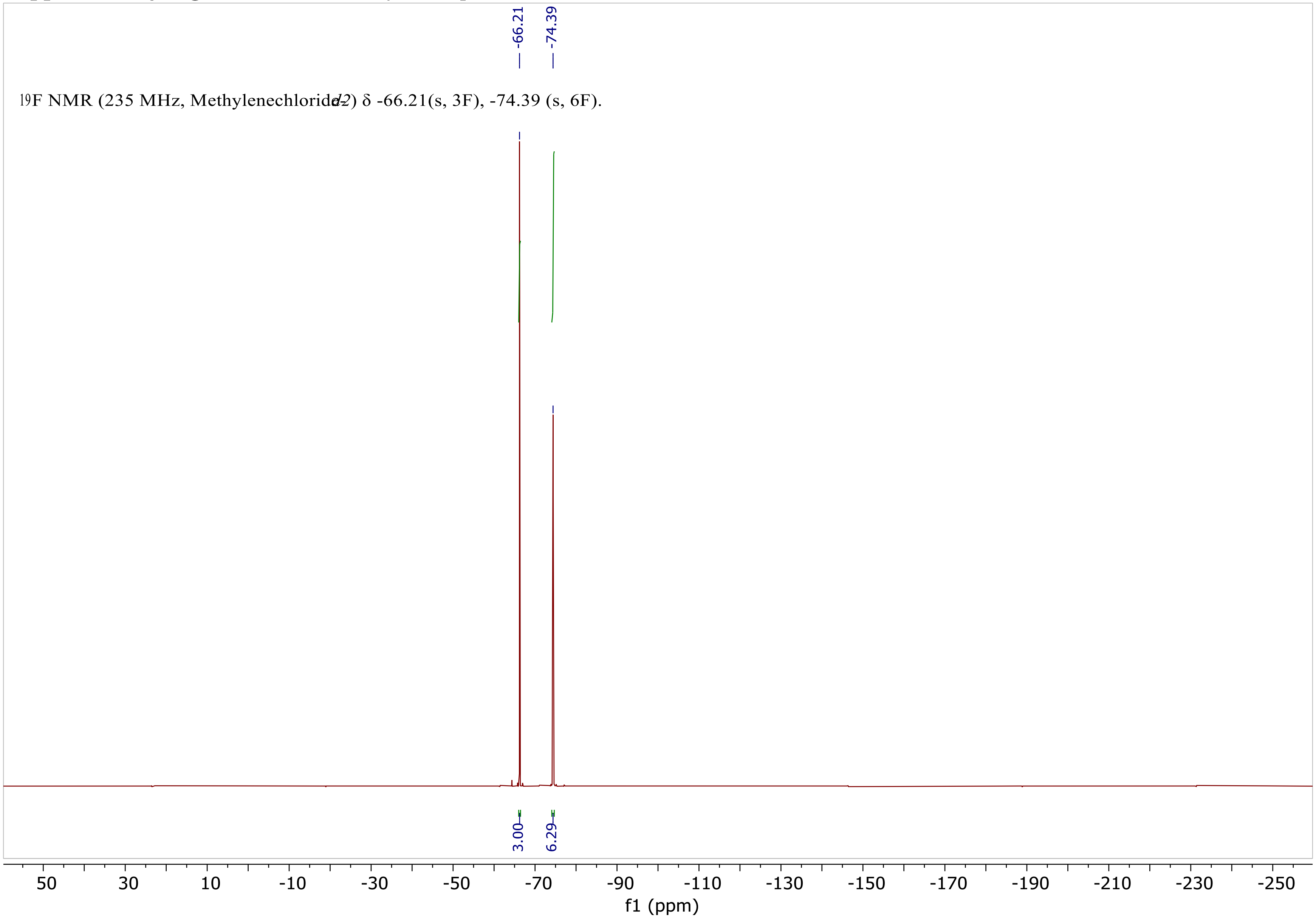

Supplementary Figure 182: *13C NMR for compound* ***35***

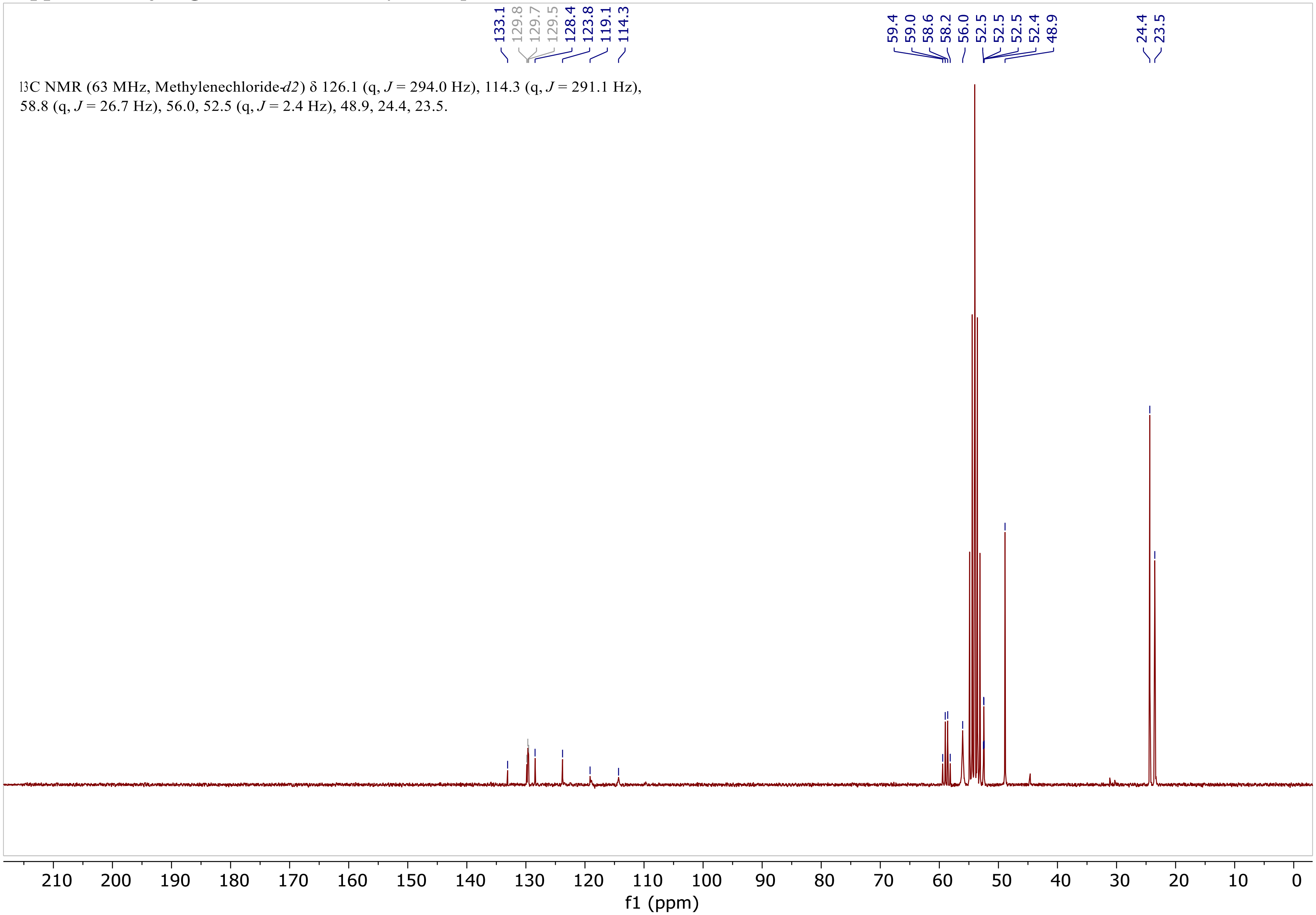

Supplementary Figure 183: *IR for compound **35***

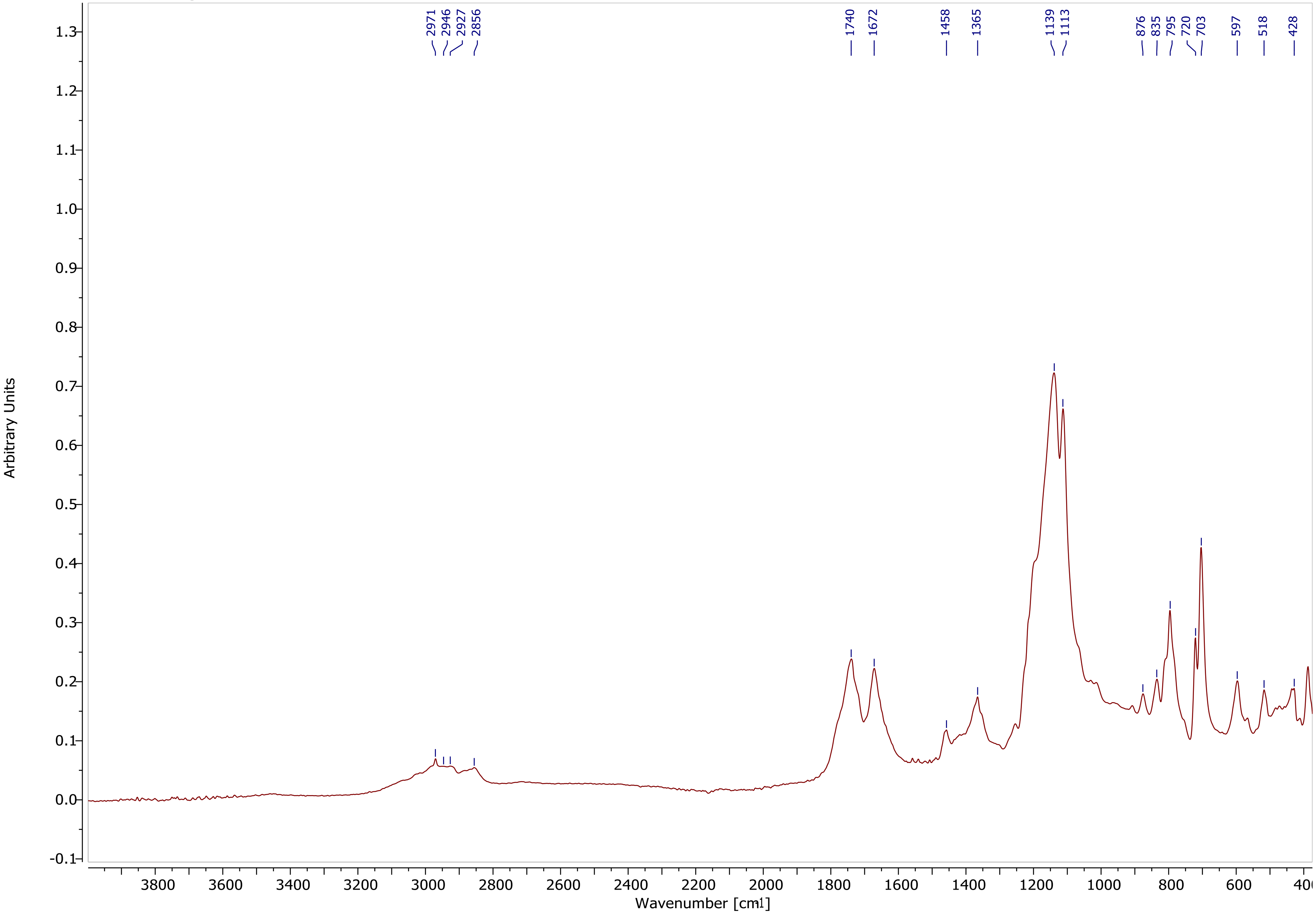

Supplementary Figure 184: *^{1}H-NMR for compound* ***36***

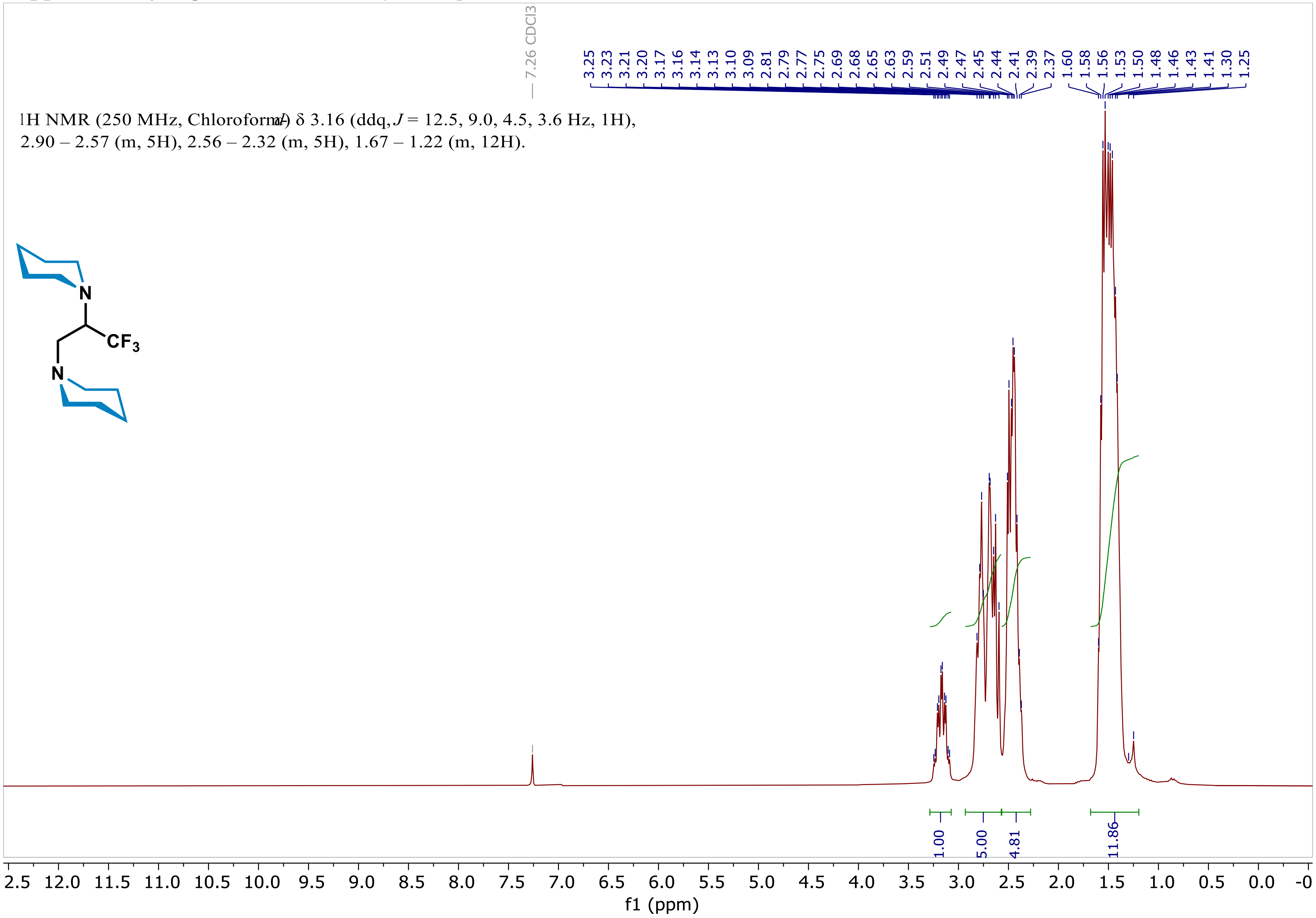

Supplementary Figure 185: *^{19}F NMR for compound* ***36***

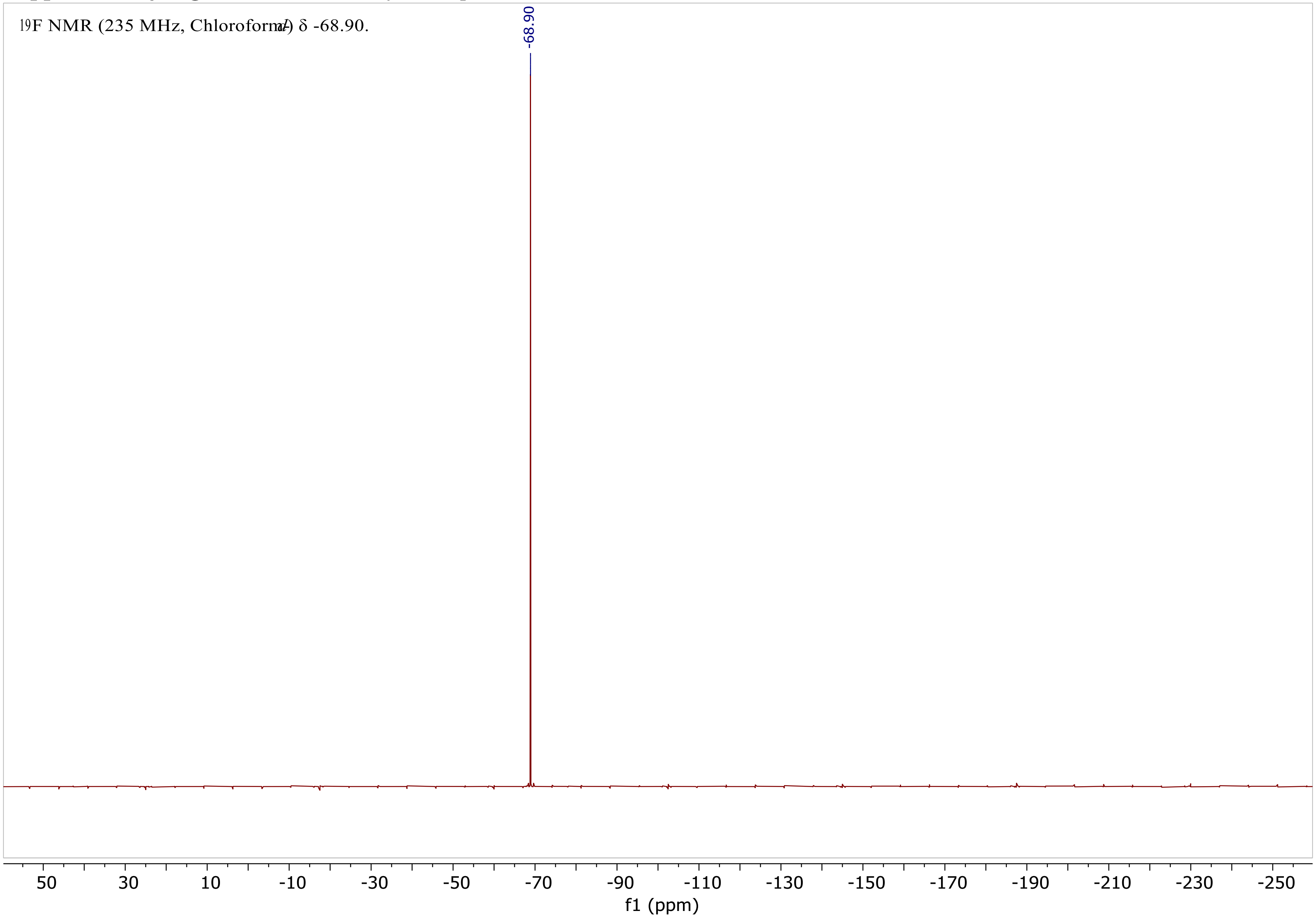

Supplementary Figure 186: *^{13}C NMR for compound* ***36***

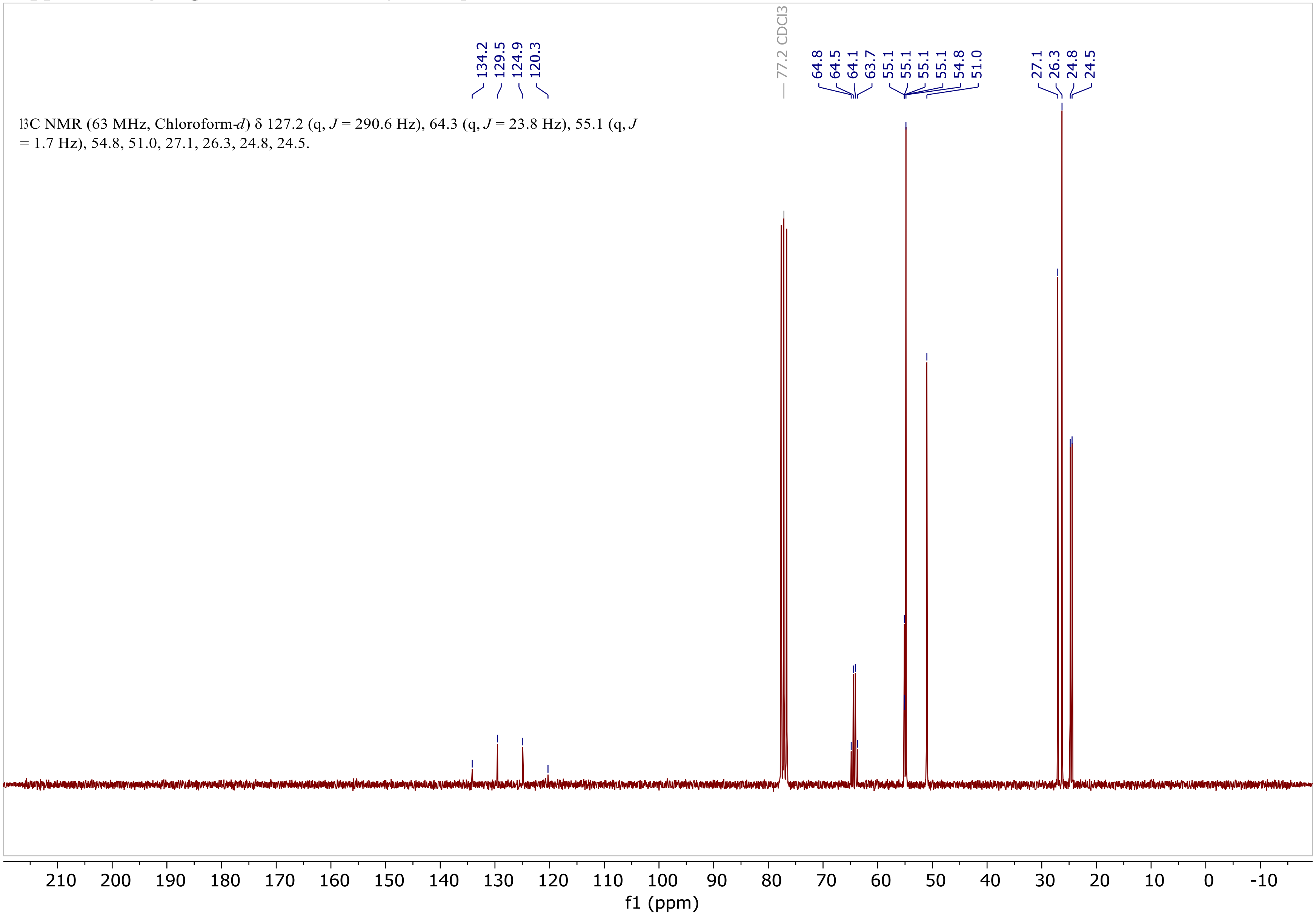

Supplementary Figure 187: *IR for compound* ***36***

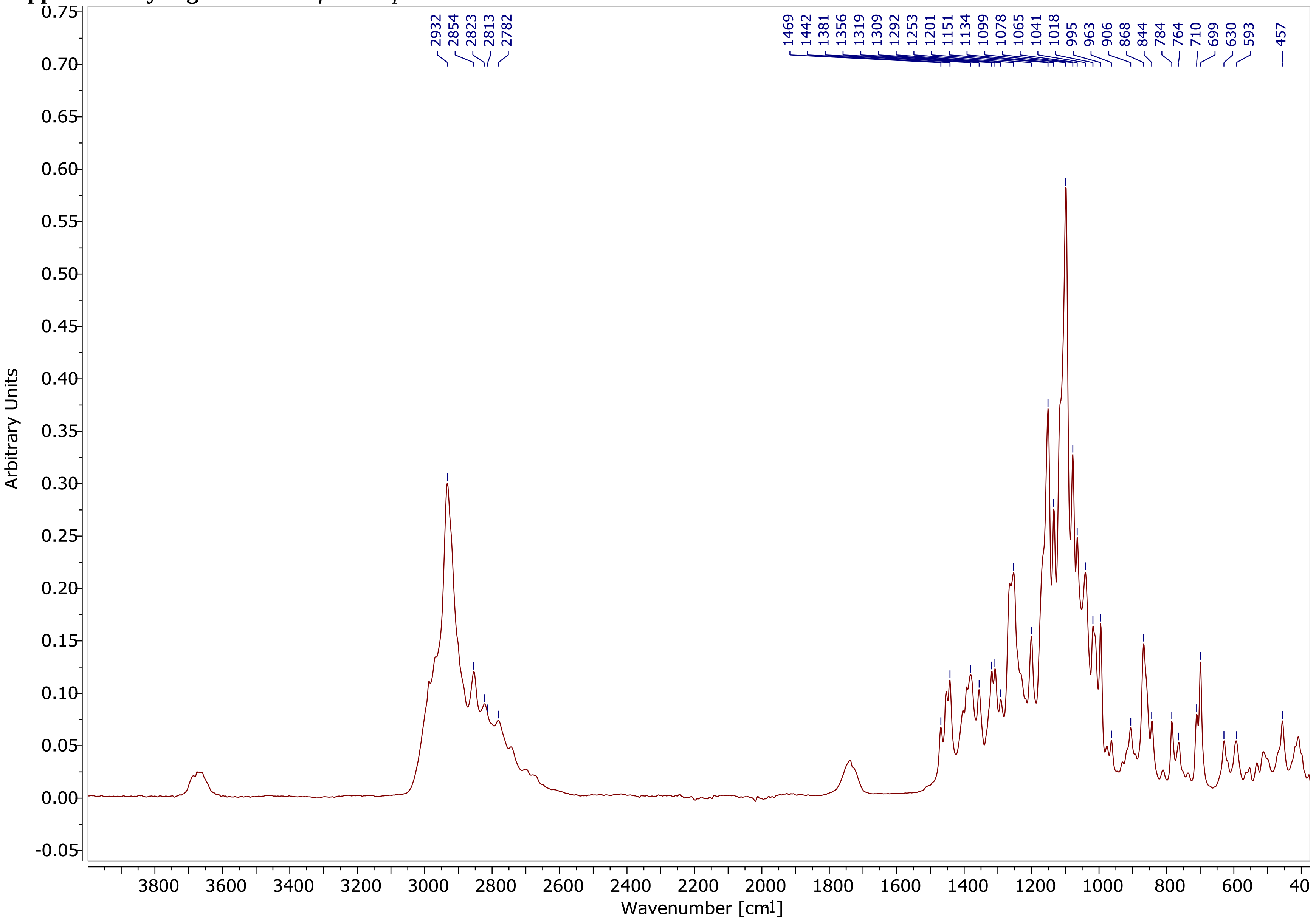

Supplementary Figure 188: *^{1}H-NMR for compound* ***37***

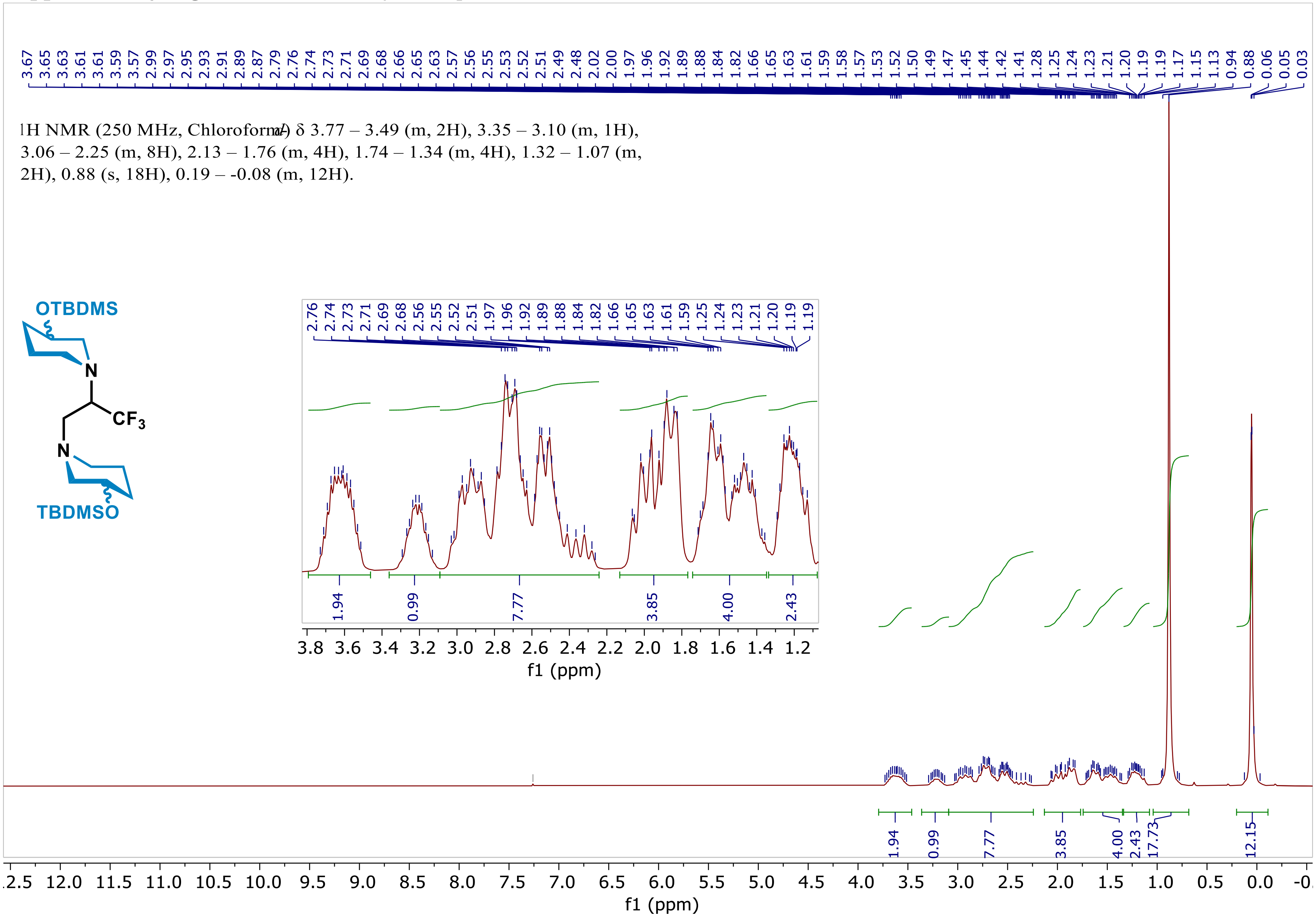

Supplementary Figure 189: *^{19}F NMR for compound* ***37***

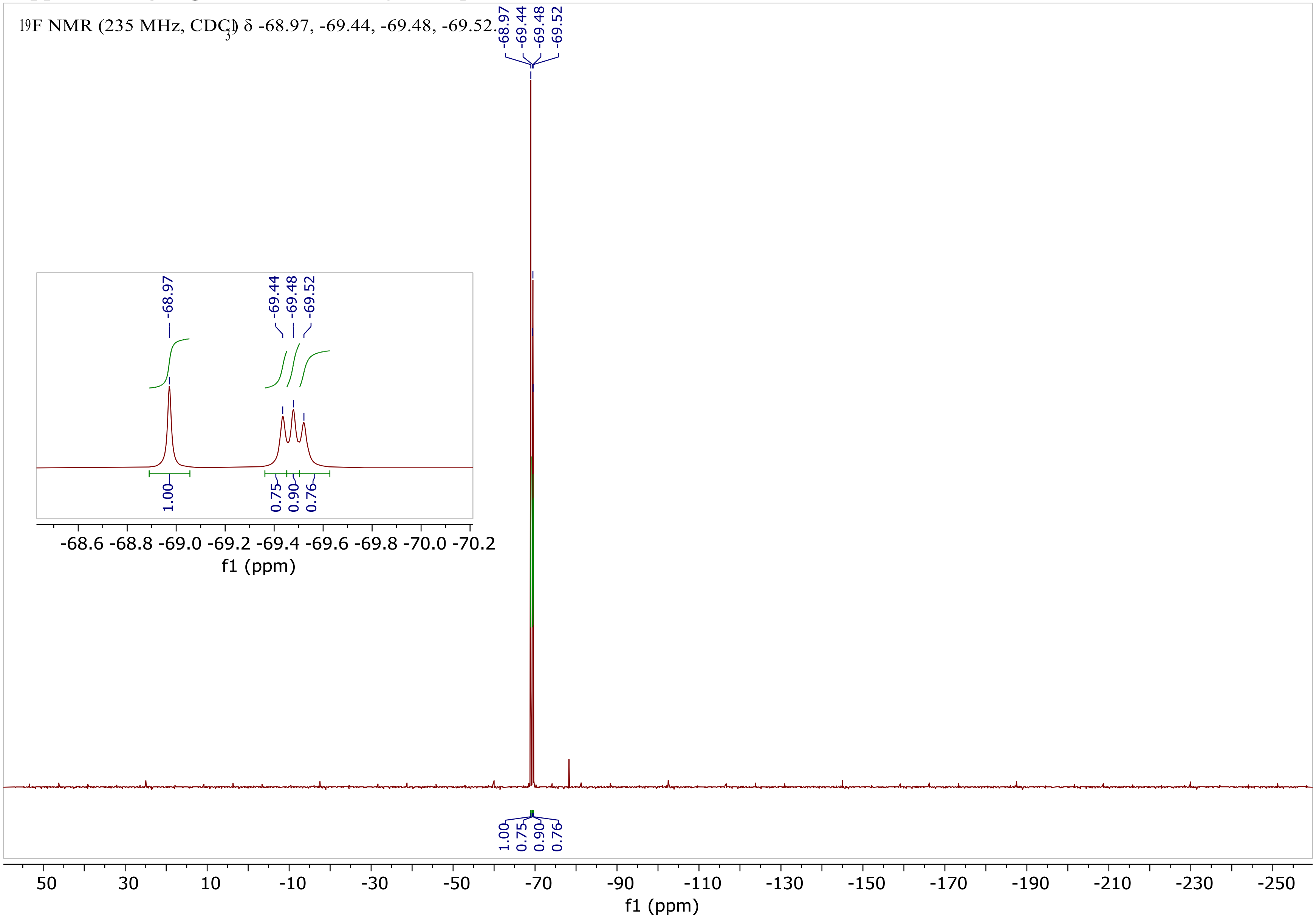

Supplementary Figure 190: *^{13}C NMR for compound* ***37***

^{13}C NMR (63 MHz, $CDCl_3$) δ 129.3, 129.3, 129.2, 124.7, 124.7, 124.6, 69.1, 69.0, 68.9, 68.7, 68.5, 68.5, 64.5, 64.1, 64.1, 63.8, 63.7, 63.7, 63.4, 63.3, 63.0, 62.9, 62.7, 62.1, 62.1, 61.6, 61.3, 58.9, 58.7, 57.6, 56.5, 54.3, 54.1, 54.0, 53.8, 53.7, 53.4, 53.4, 51.3, 50.3, 49.5, 49.4, 34.5, 34.4, 34.1, 34.1, 26.0, 26.0, 18.3, 18.3, 18.3, -4.5, -4.5.

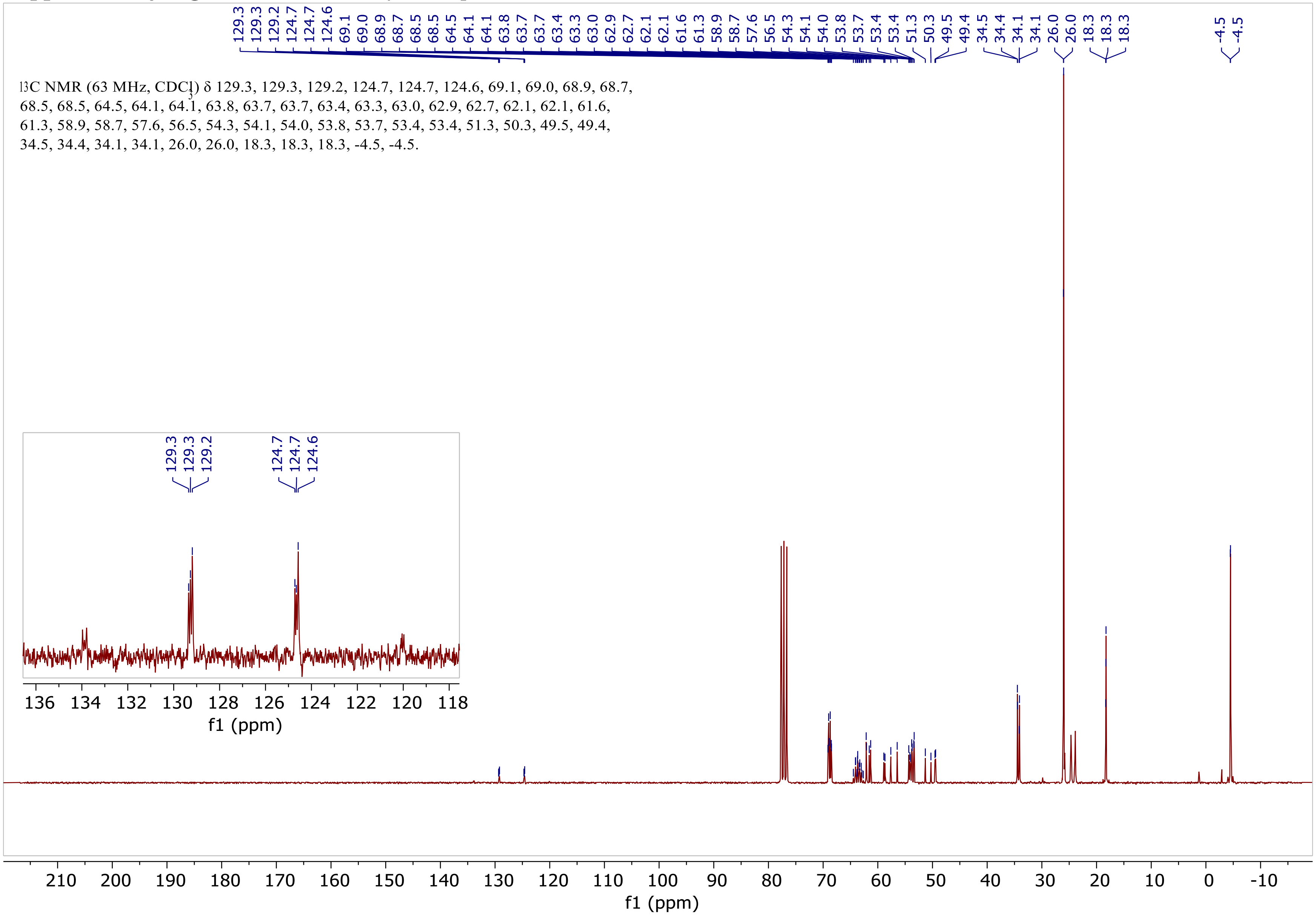

Supplementary Figure 191: *IR for compound* ***37***

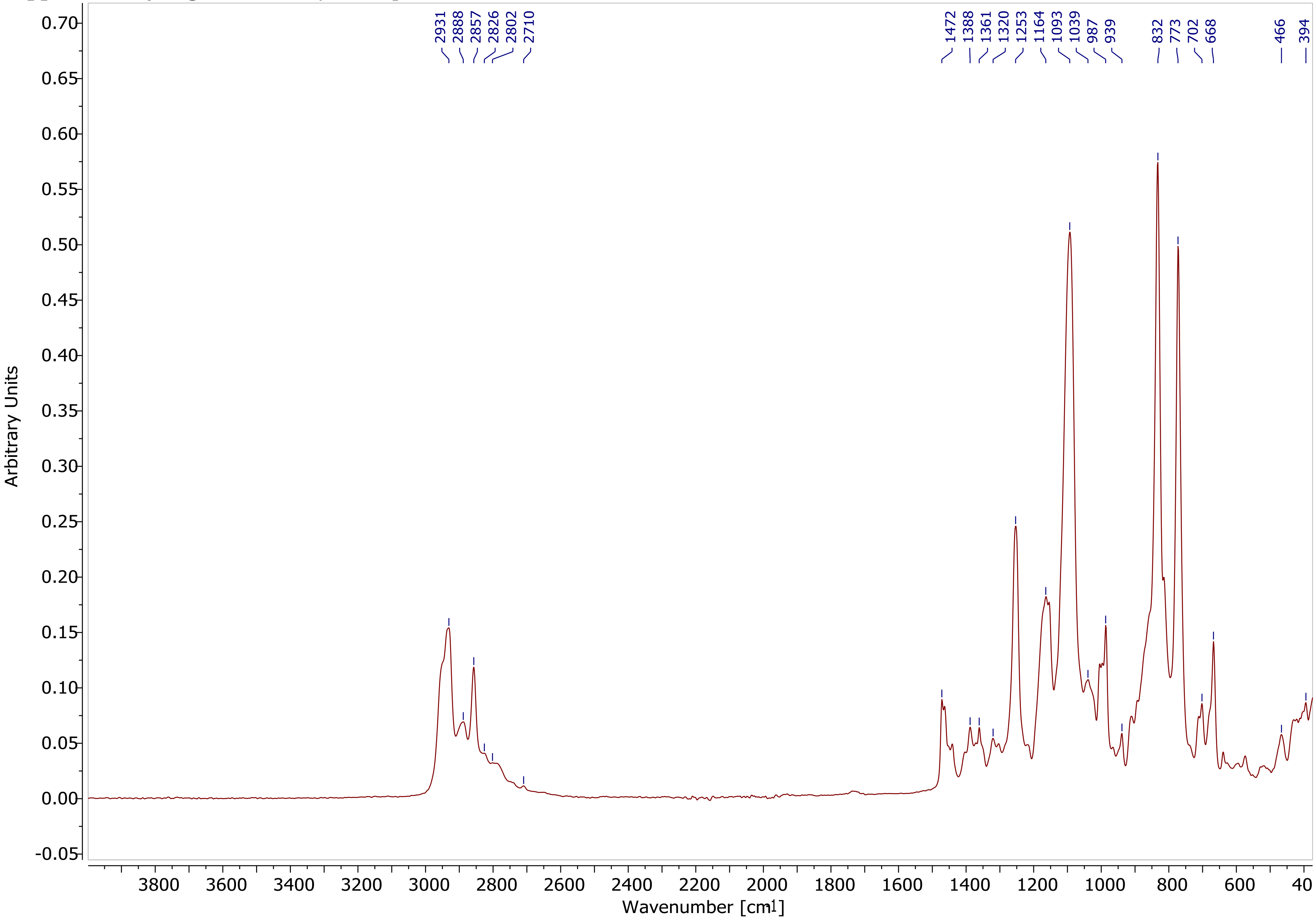

Supplementary Figure 192: *^{1}H-NMR for compound* ***38***

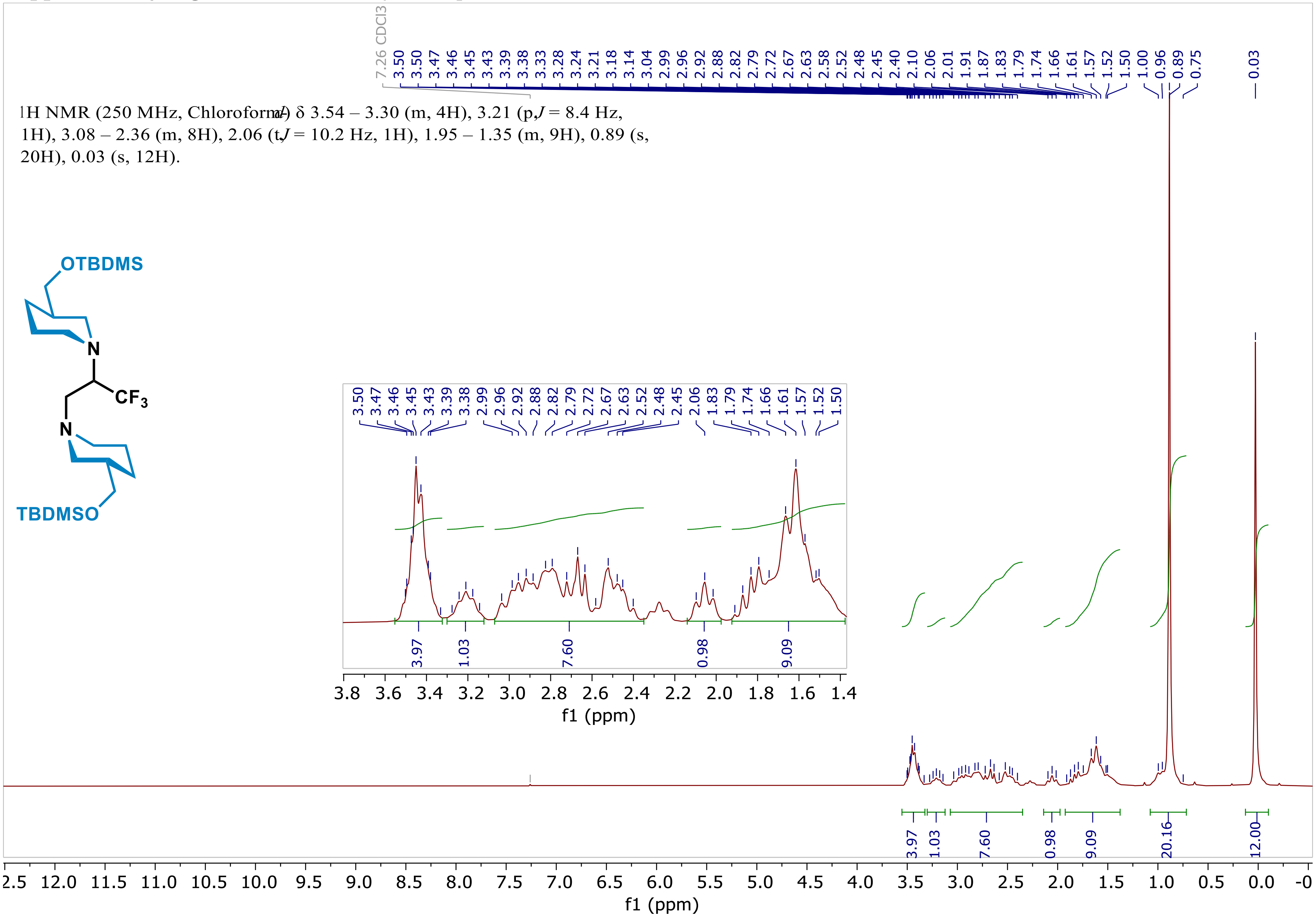

Supplementary Figure 193: *^{19}F NMR for compound* ***38***

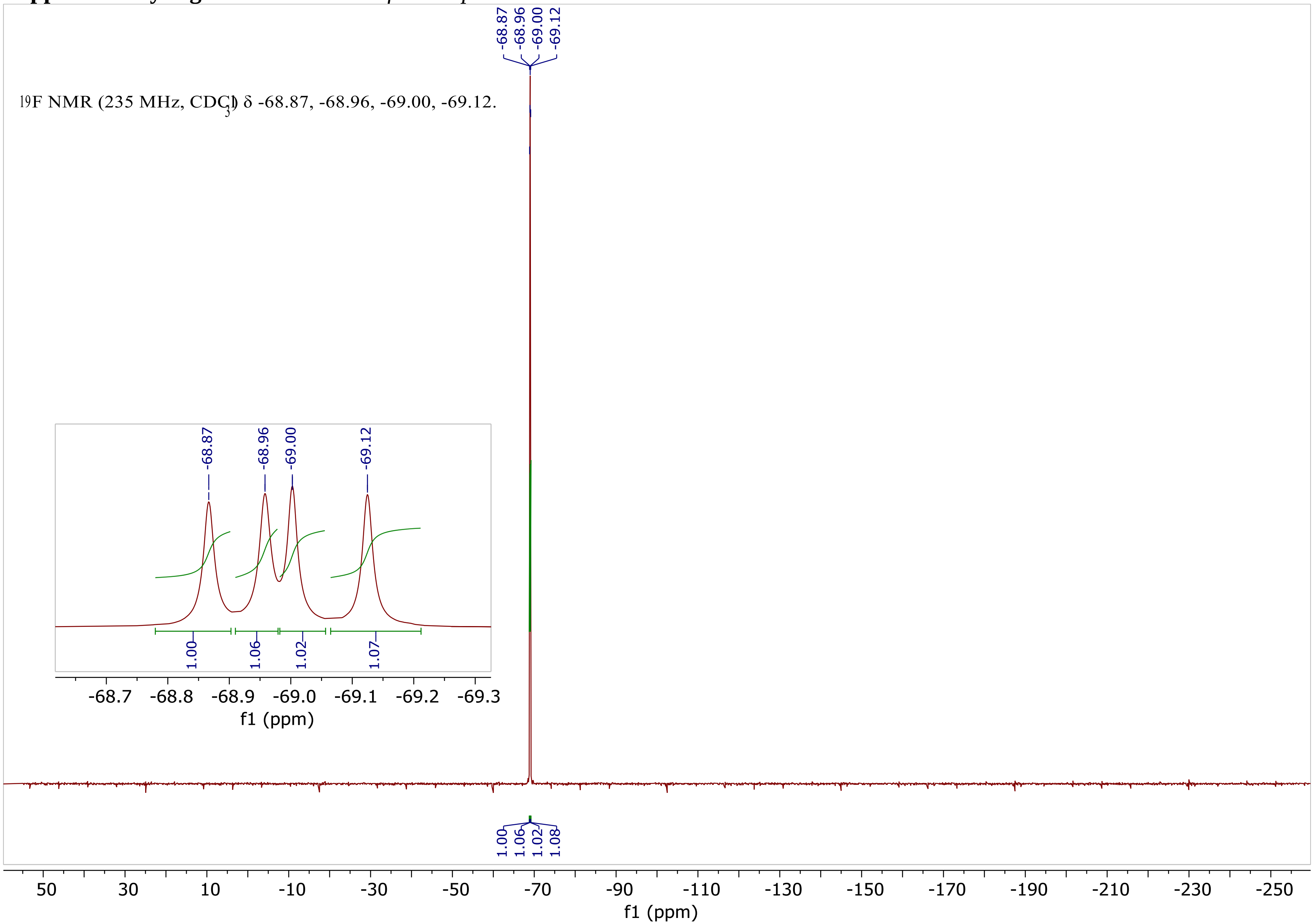

Supplementary Figure 194: ^{13}*C NMR for compound* ***38***

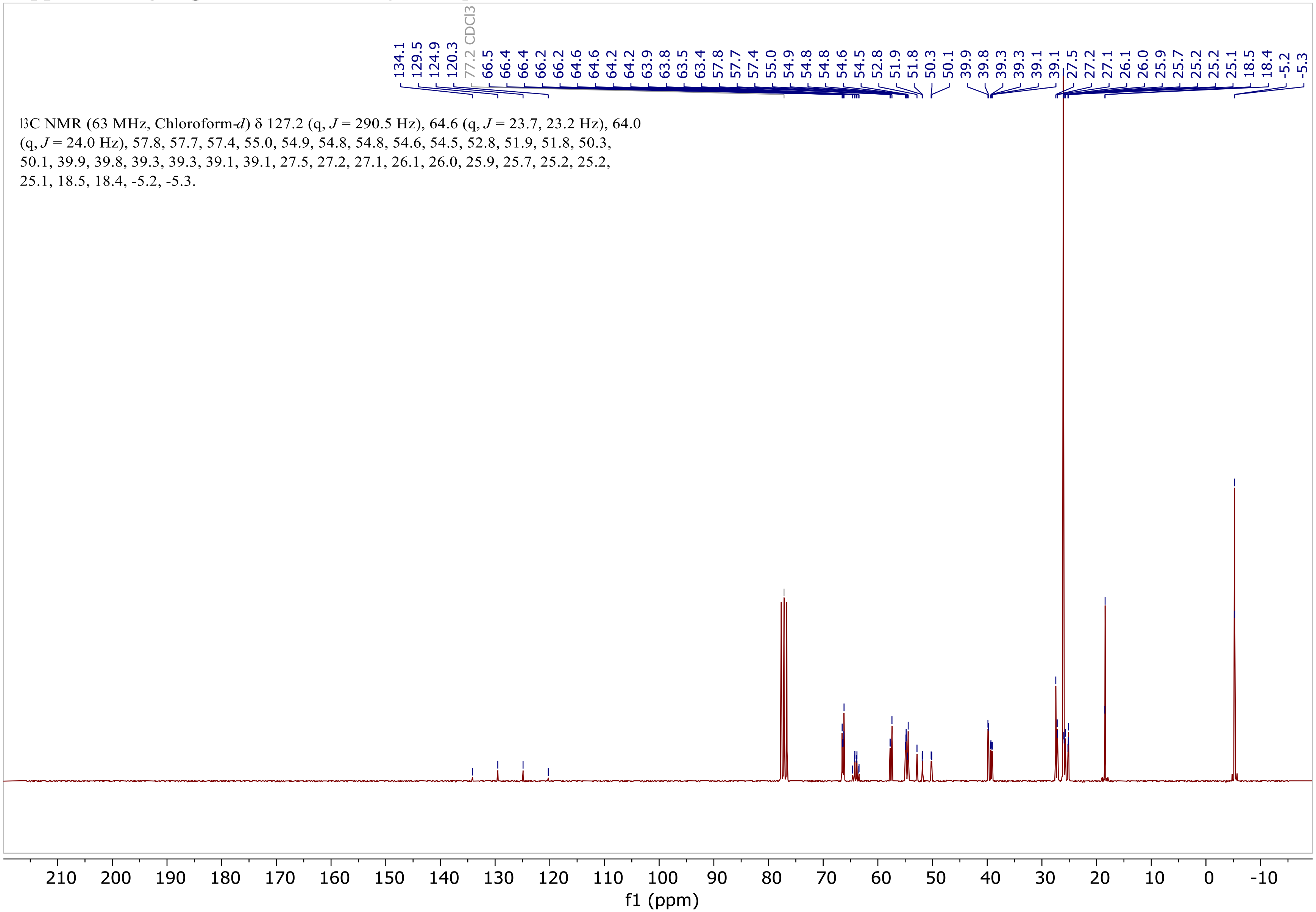

Supplementary Figure 195: *IR for compound* ***38***

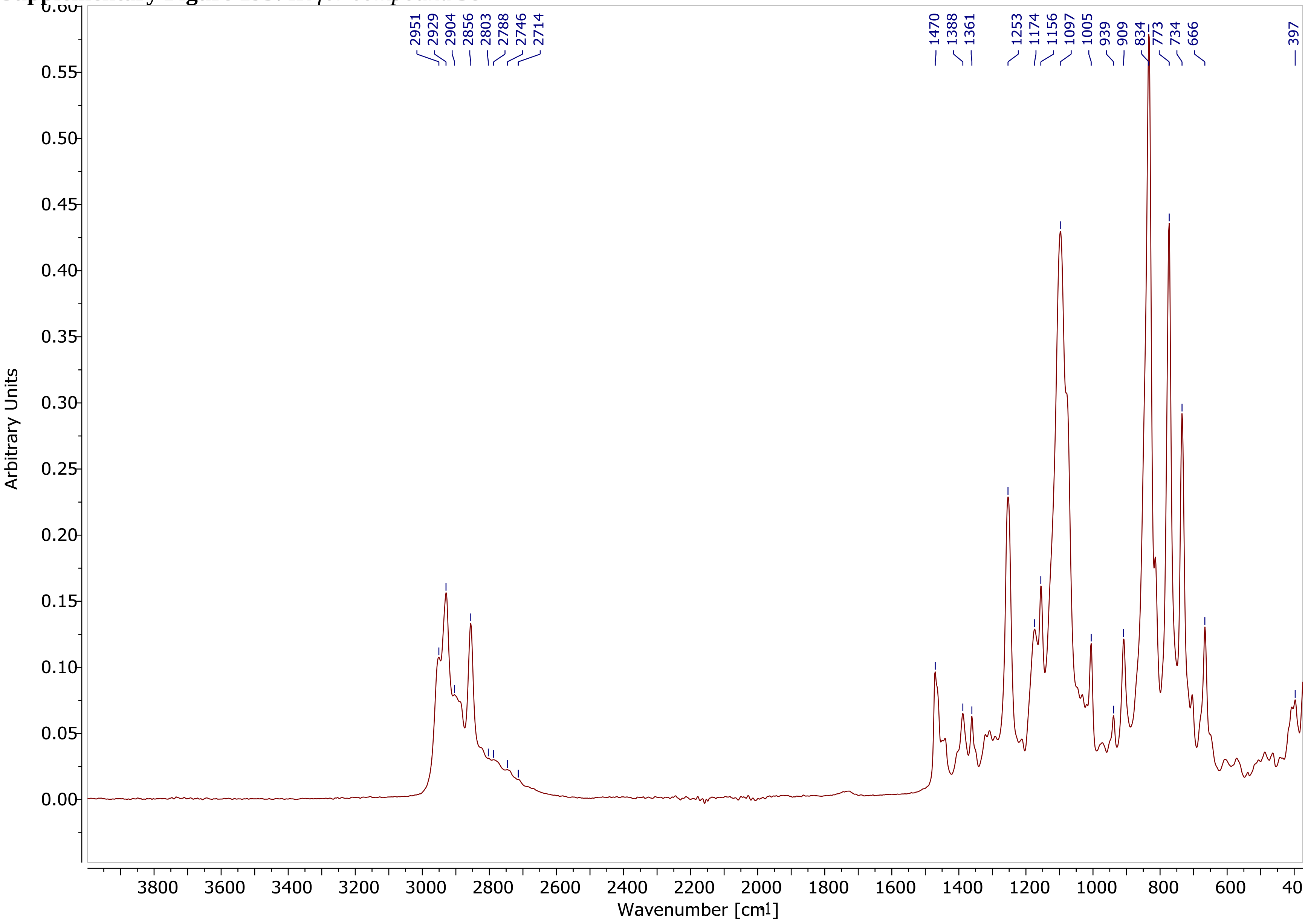

Supplementary Figure 196: *^{1}H-NMR for compound **39***

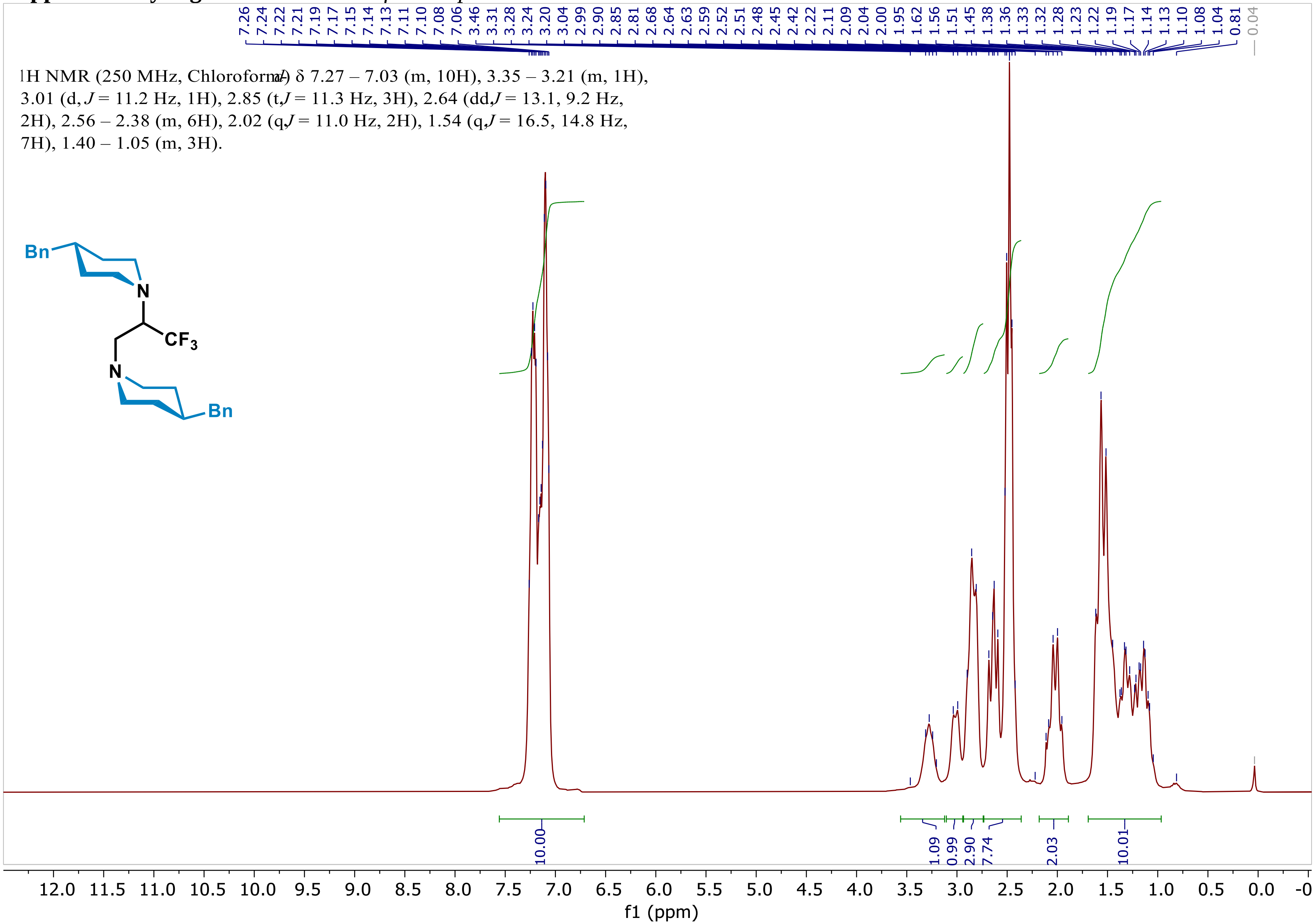

Supplementary Figure 197: ^{19}*F NMR for compound* ***39***

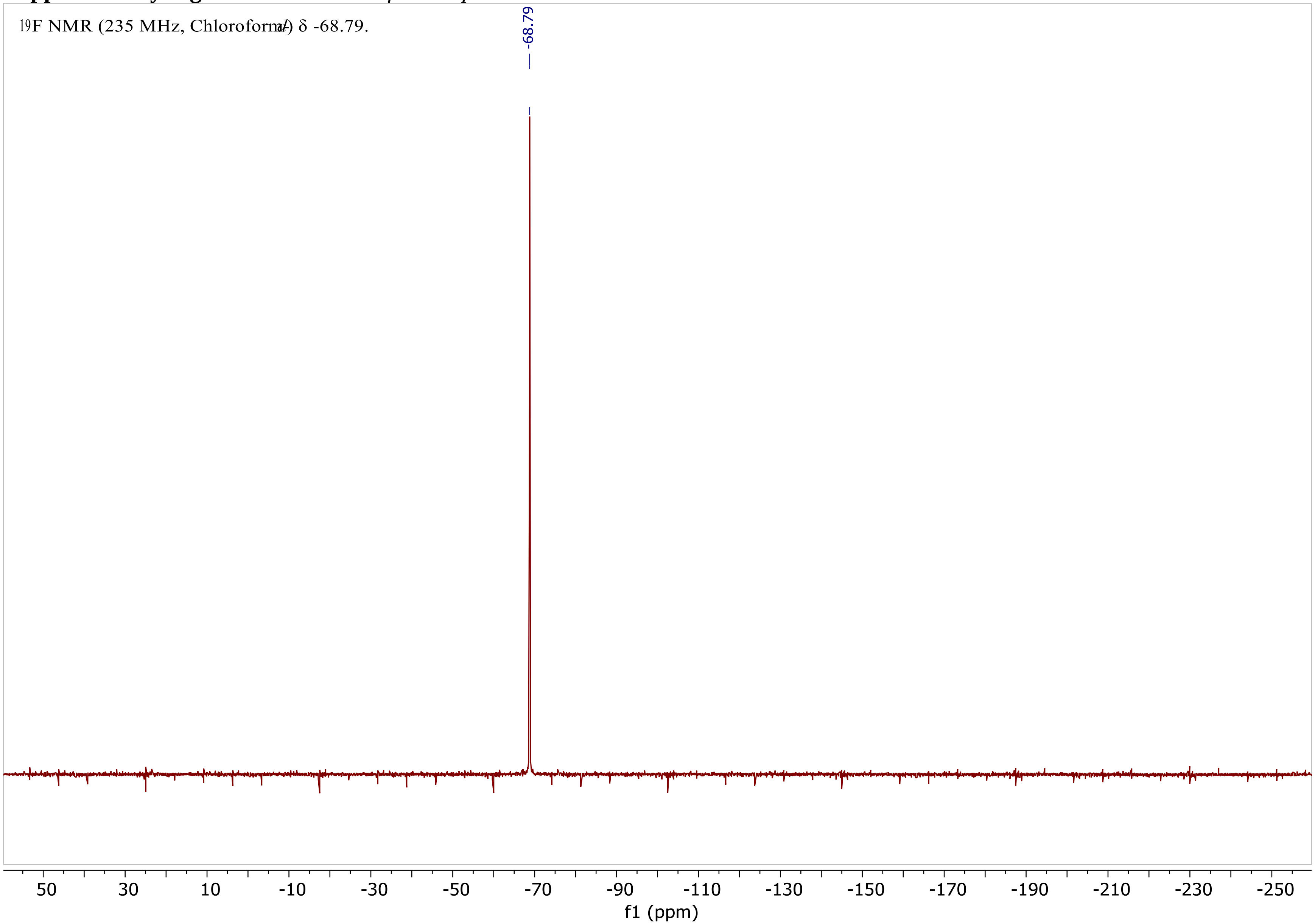

Supplementary Figure 198: *^{13}C NMR for compound **39***

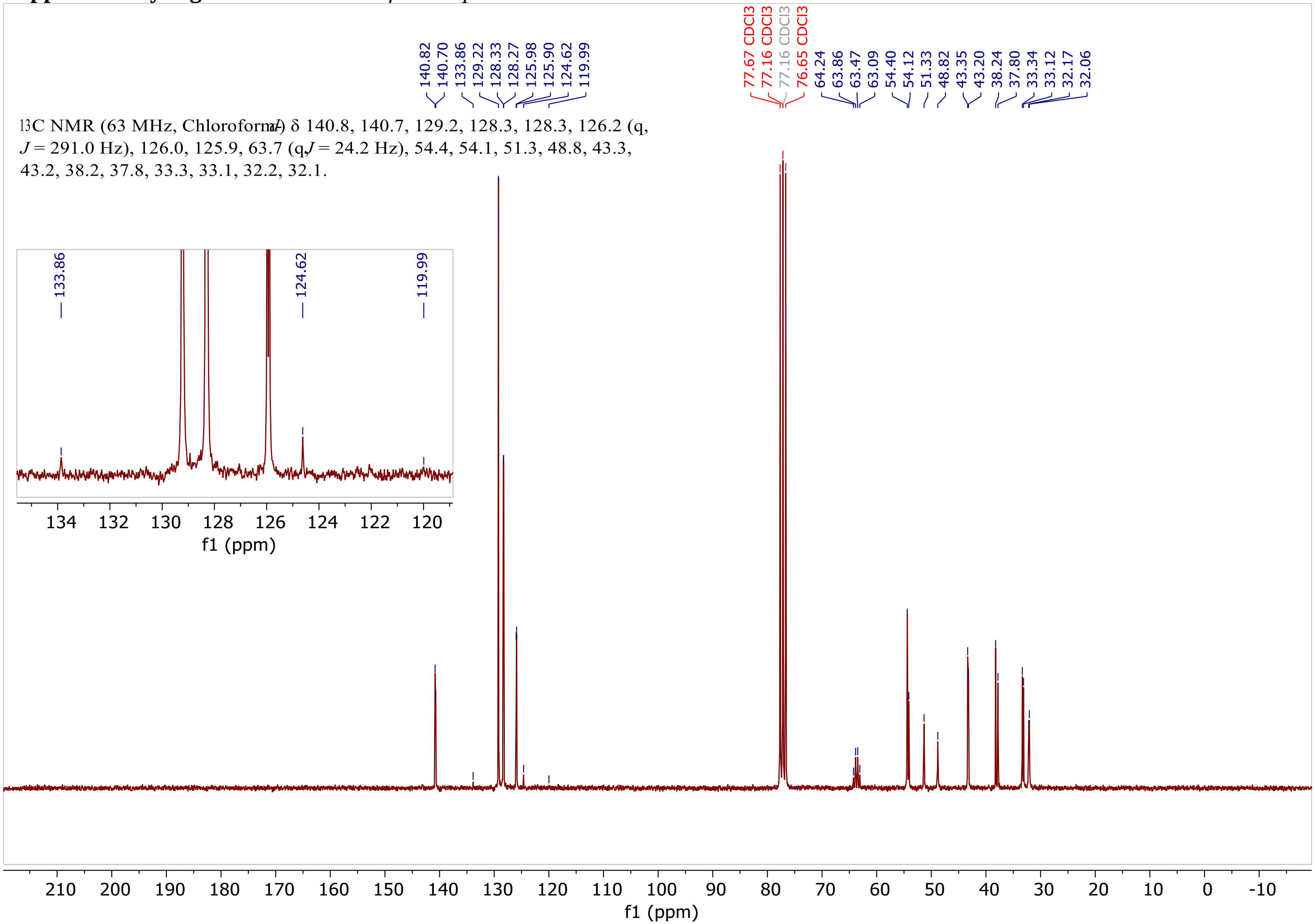

Supplementary Figure 199: *IR for compound **39***

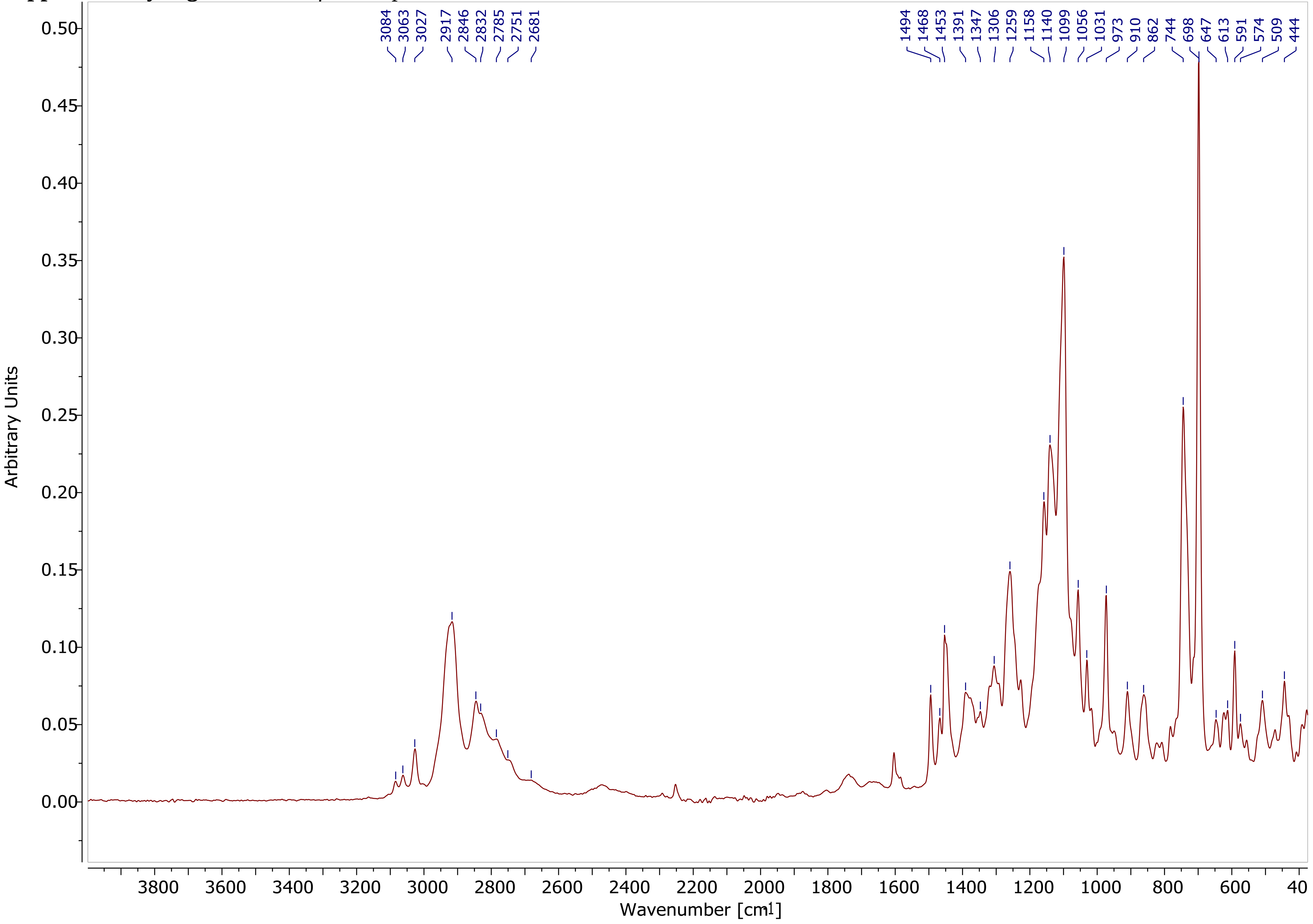

Supplementary Figure 200: *^{1}H-NMR for compound **40***

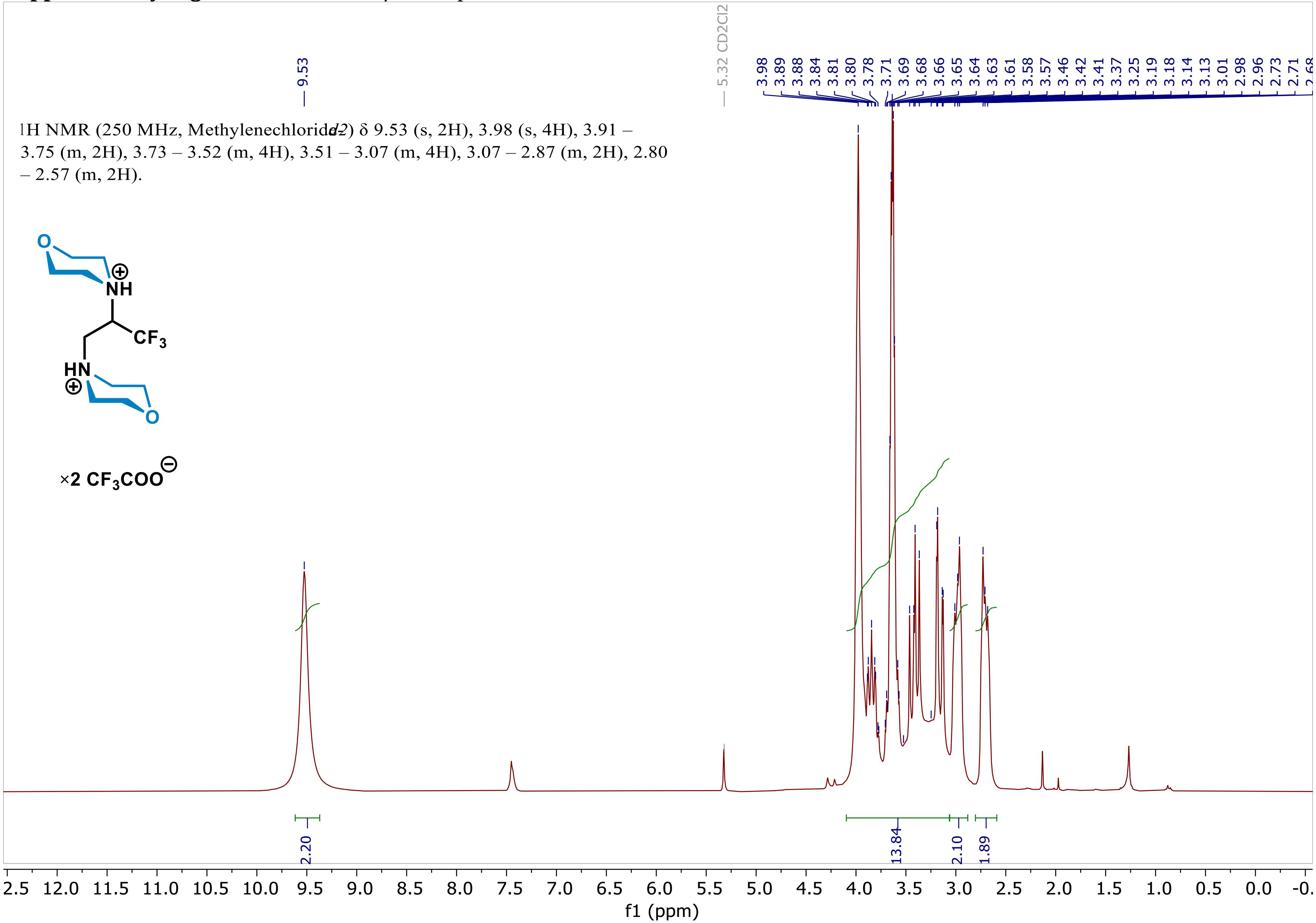

Supplementary Figure 201: *^{19}F NMR for compound* ***40***

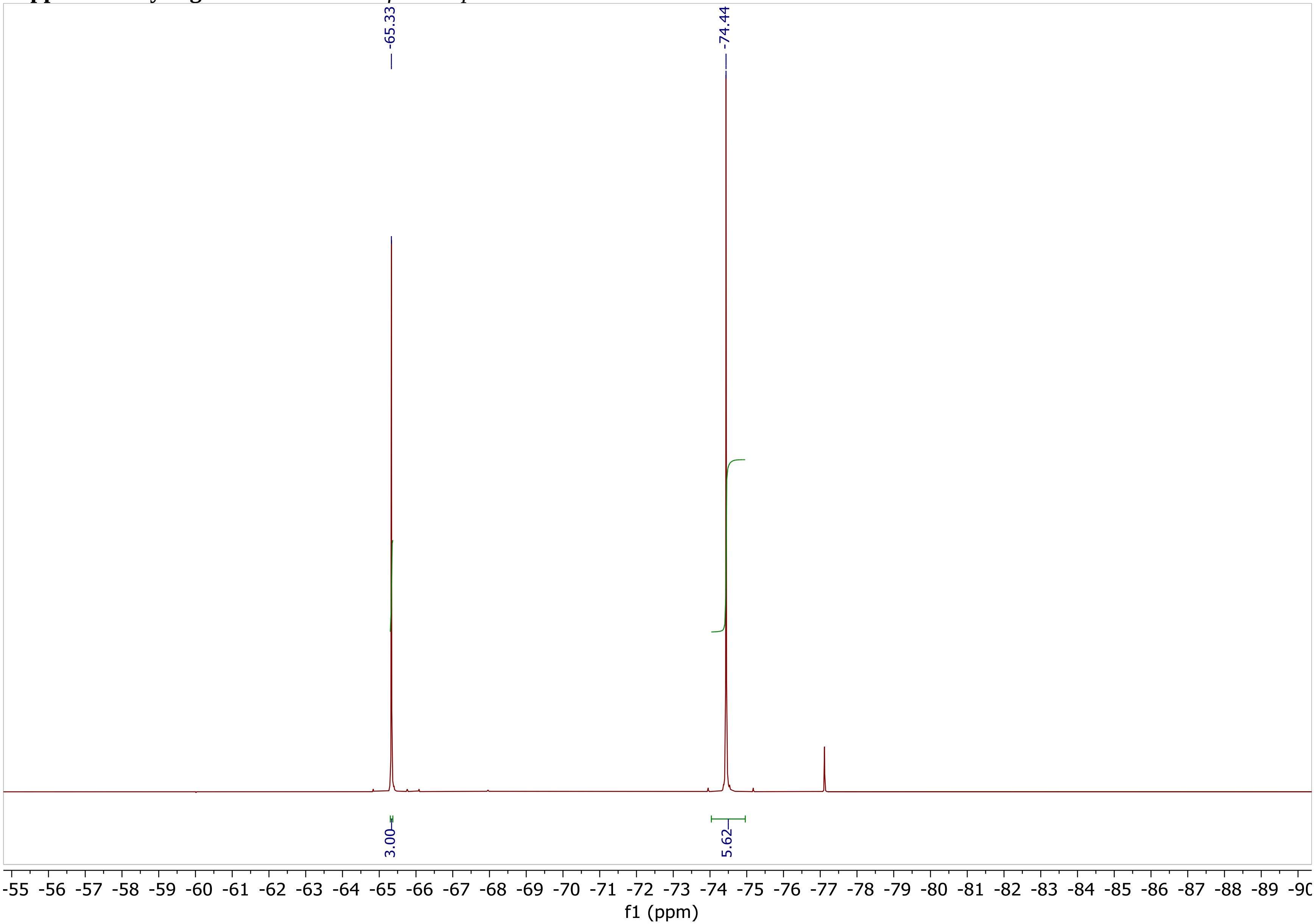

Supplementary Figure 202: *^{13}C NMR for compound* ***40***

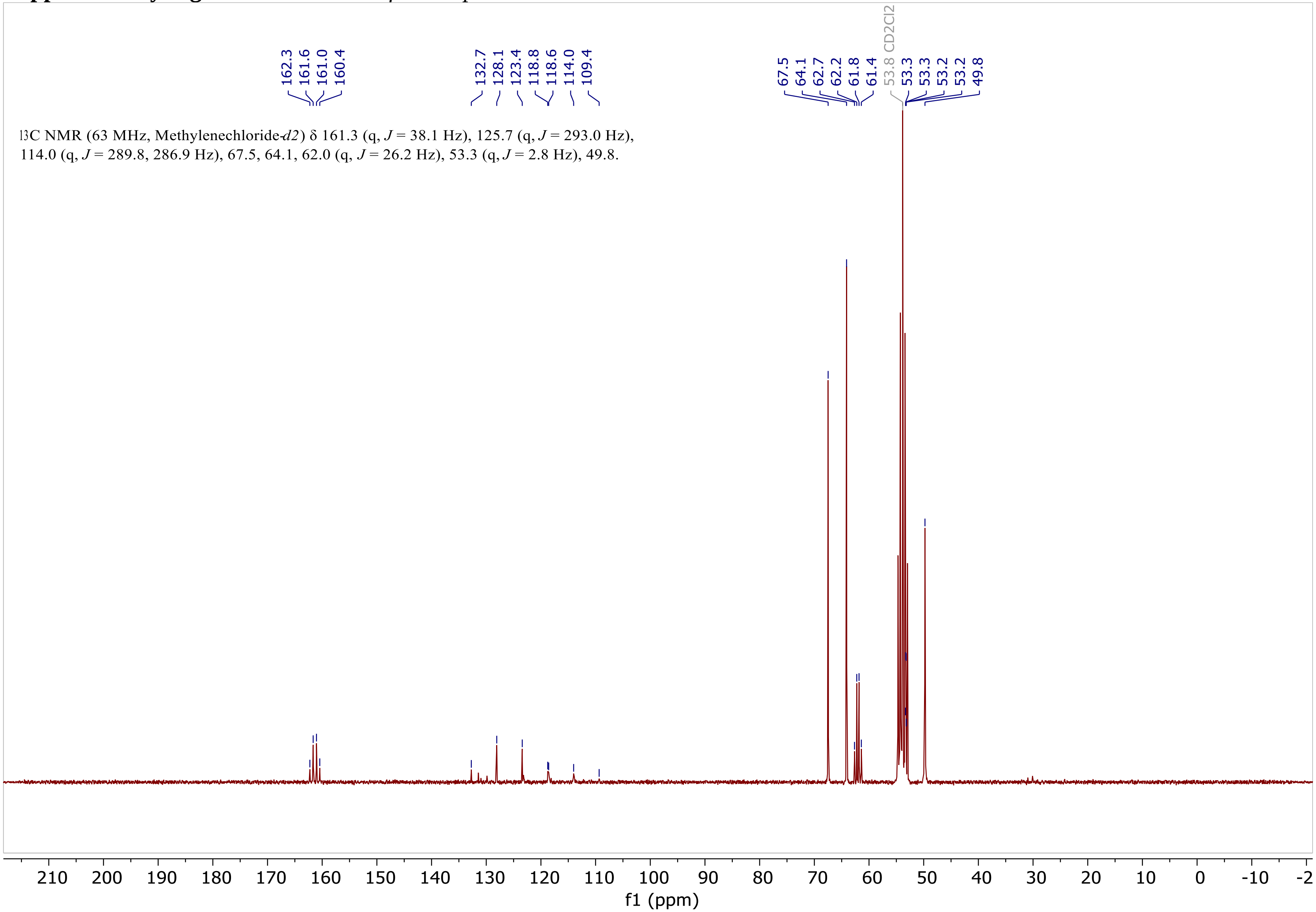

Supplementary Figure 203: *IR for compound **40***

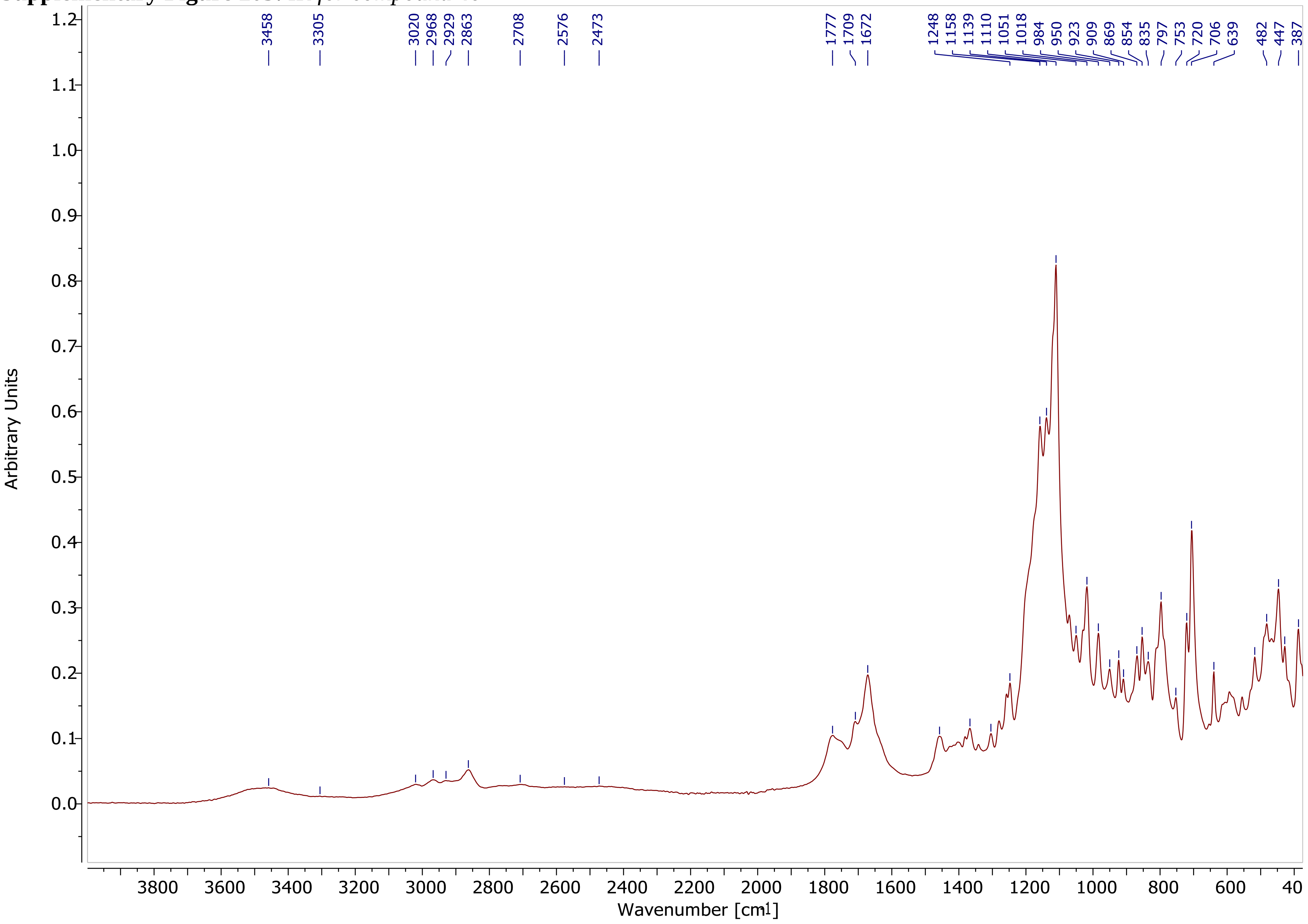

Supplementary Figure 204: *^{1}H-NMR for compound* ***41***

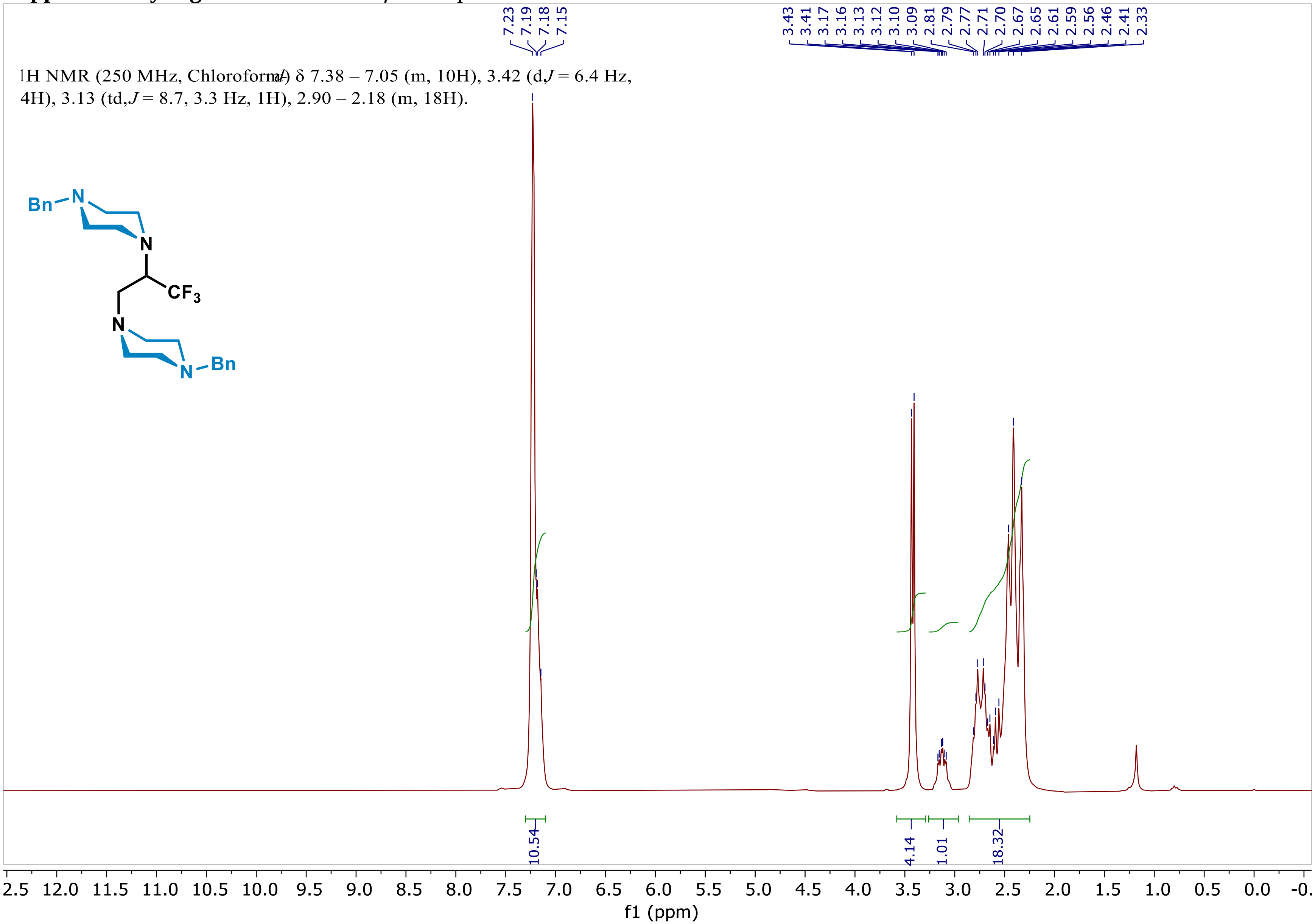

Supplementary Figure 205: *^{19}F NMR for compound **41***

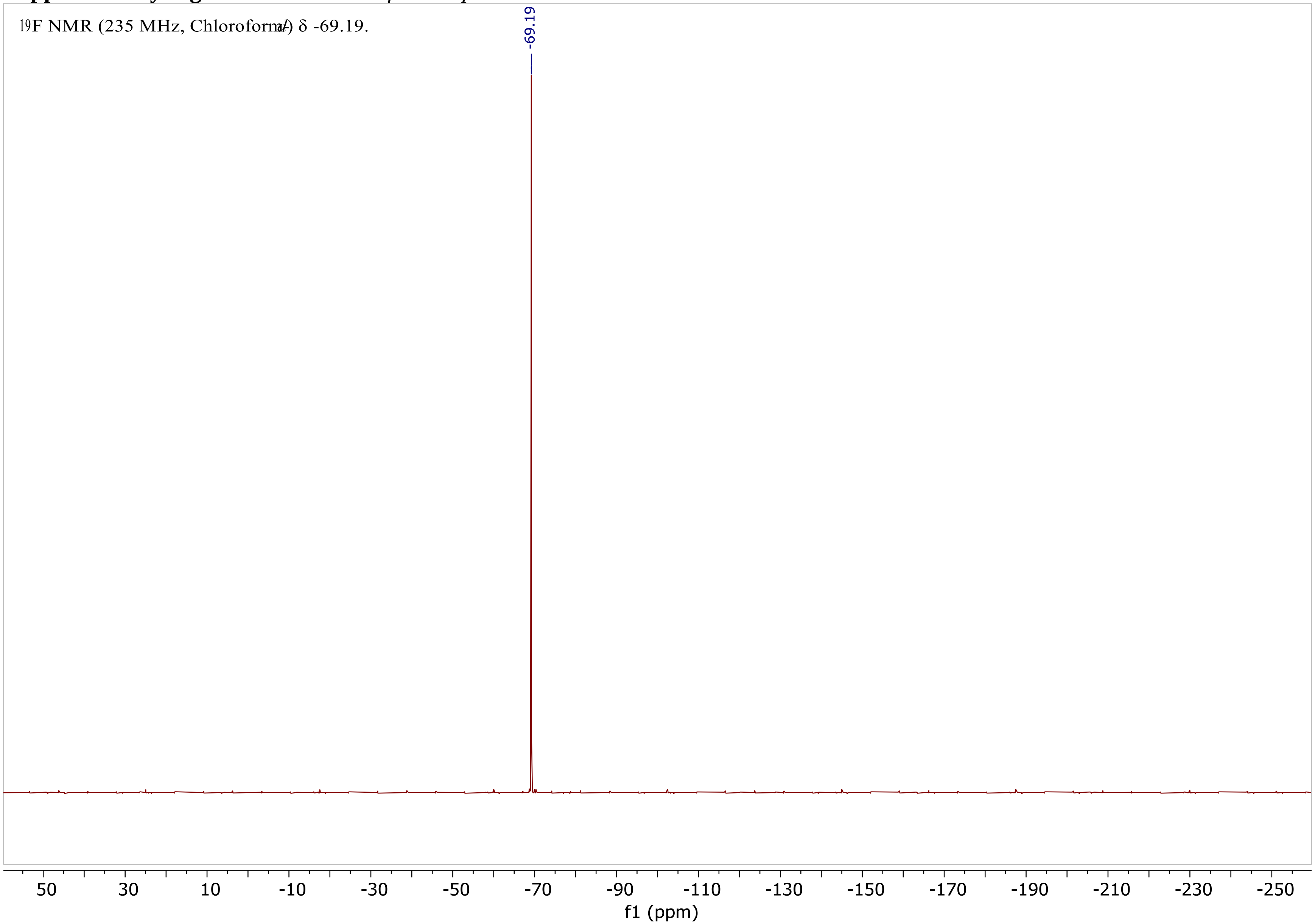

Supplementary Figure 206: *^{13}C NMR for compound **41***

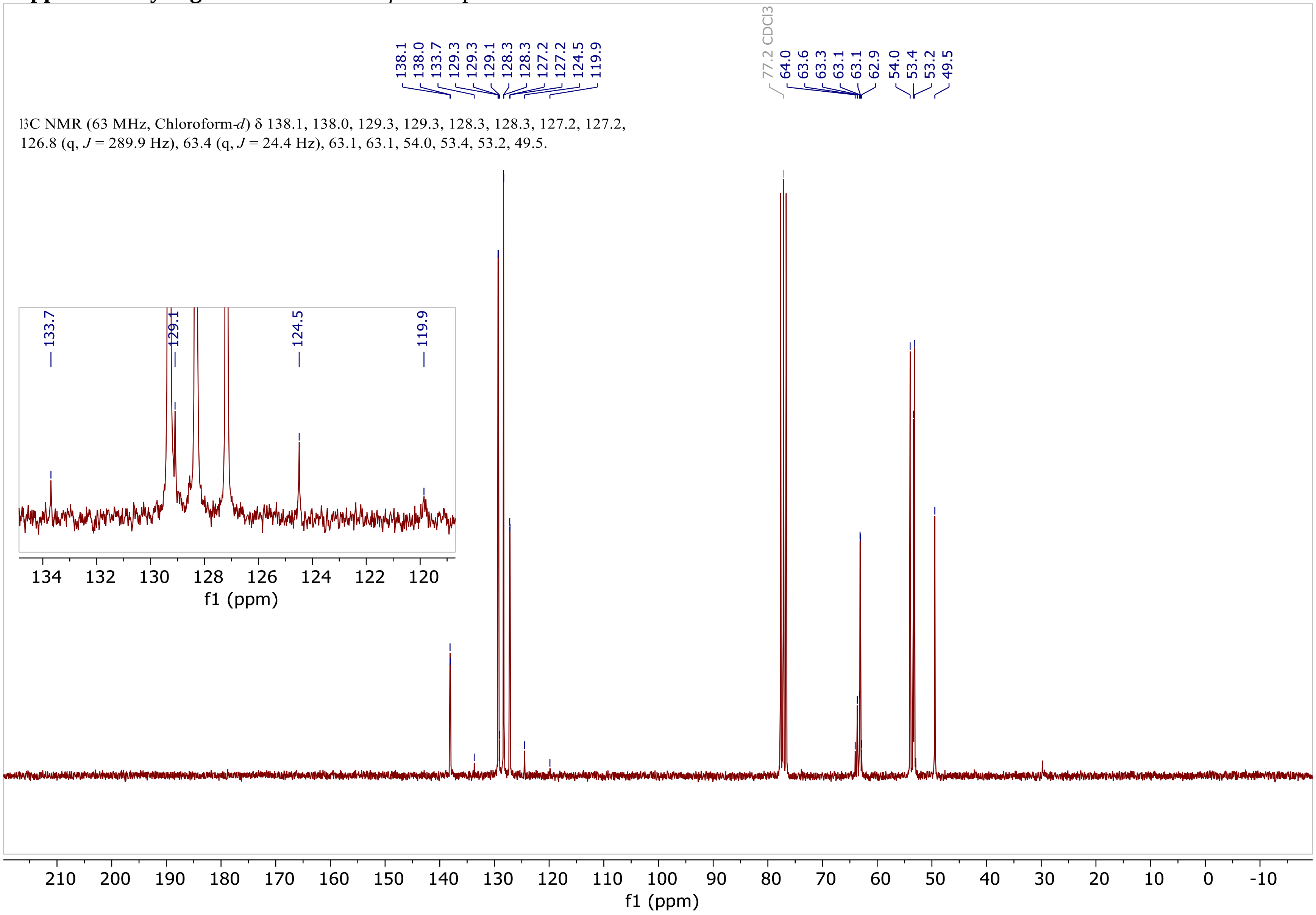

Supplementary Figure 207: *IR for compound* ***41***

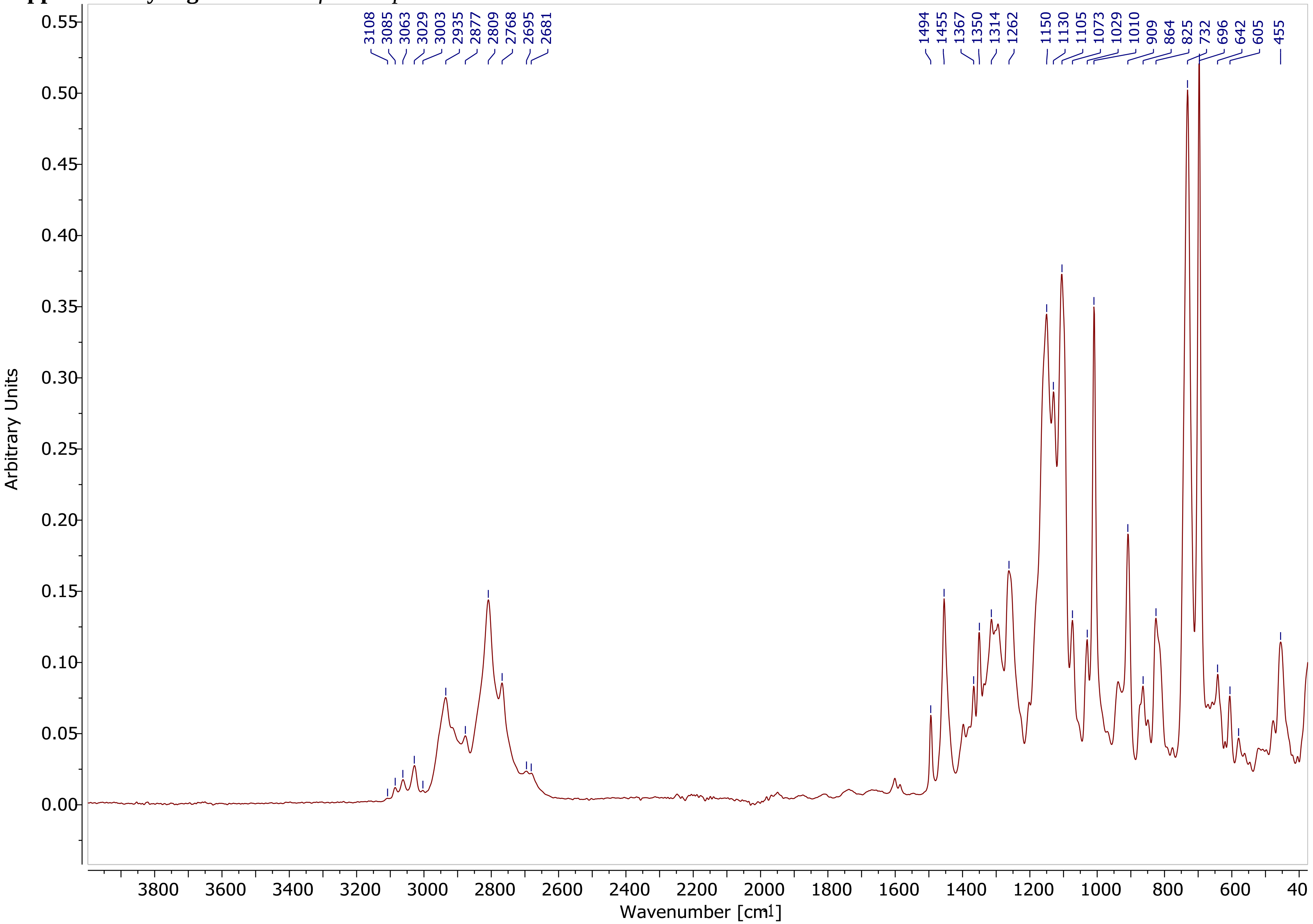

Supplementary Figure 208: *¹H-NMR for compound* ***42***

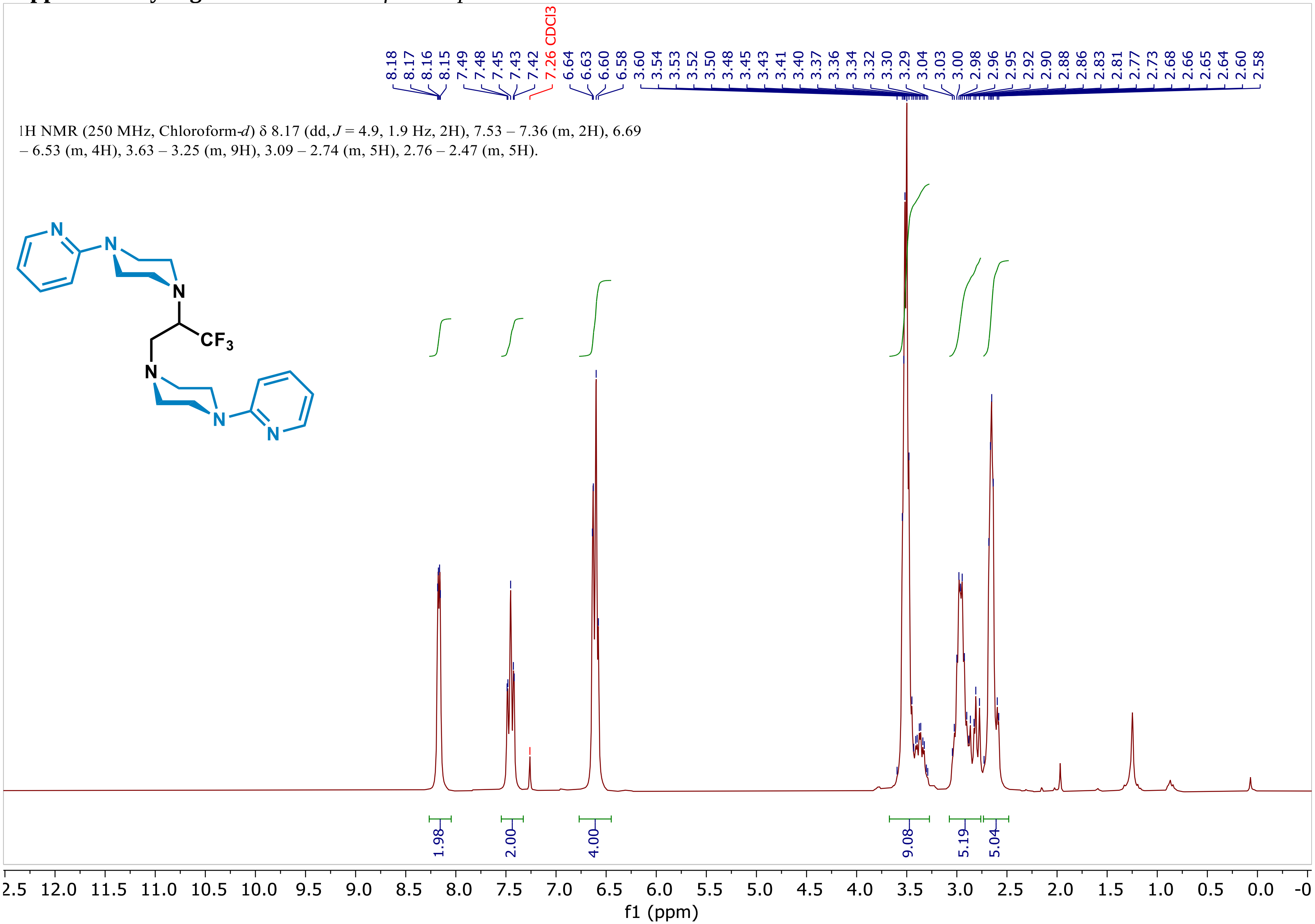

Supplementary Figure 209: *^{19}F NMR for compound* ***42***

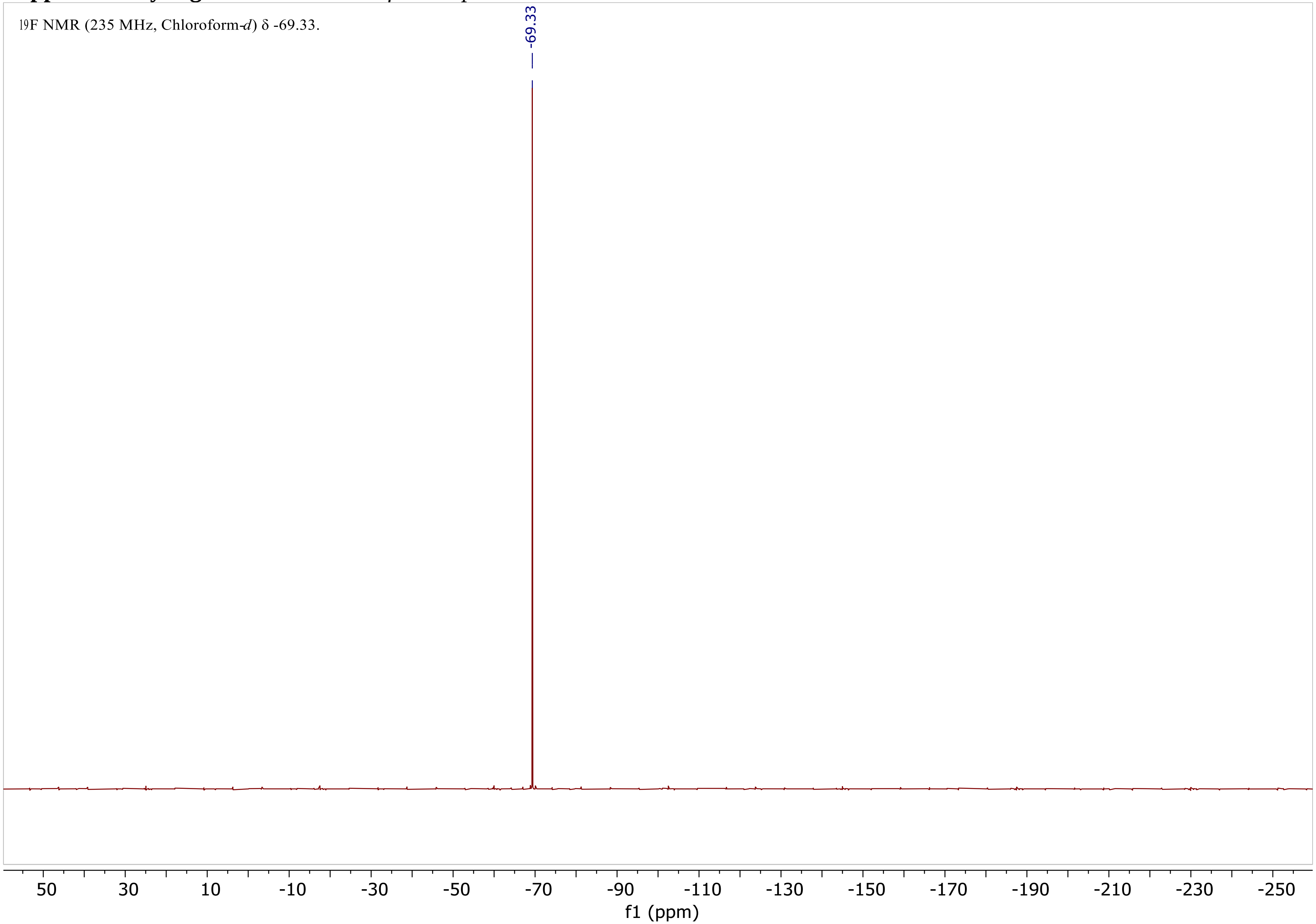

Supplementary Figure 210: *^{13}C NMR for compound* ***42***

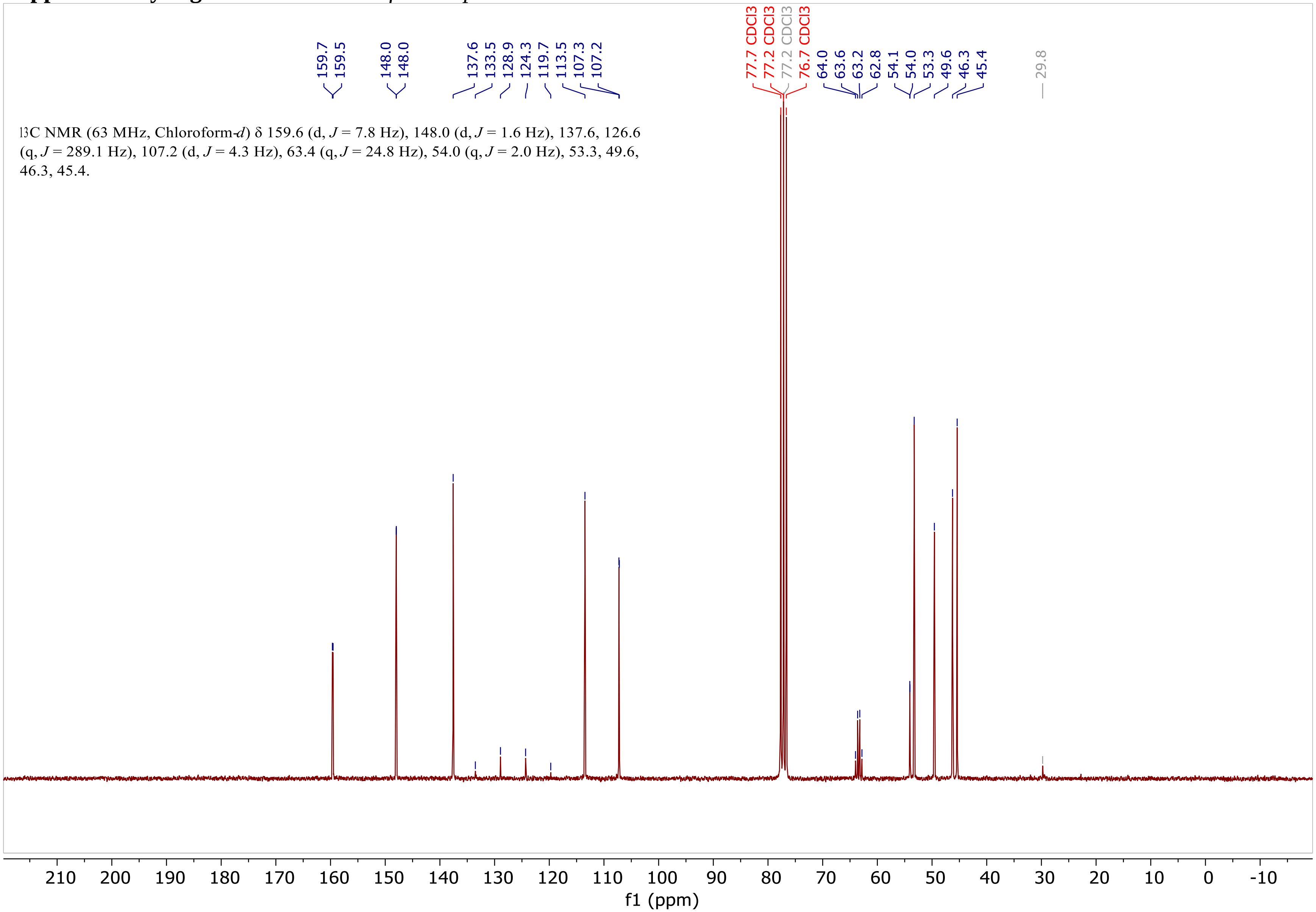

Supplementary Figure 211: *IR for compound* ***42***

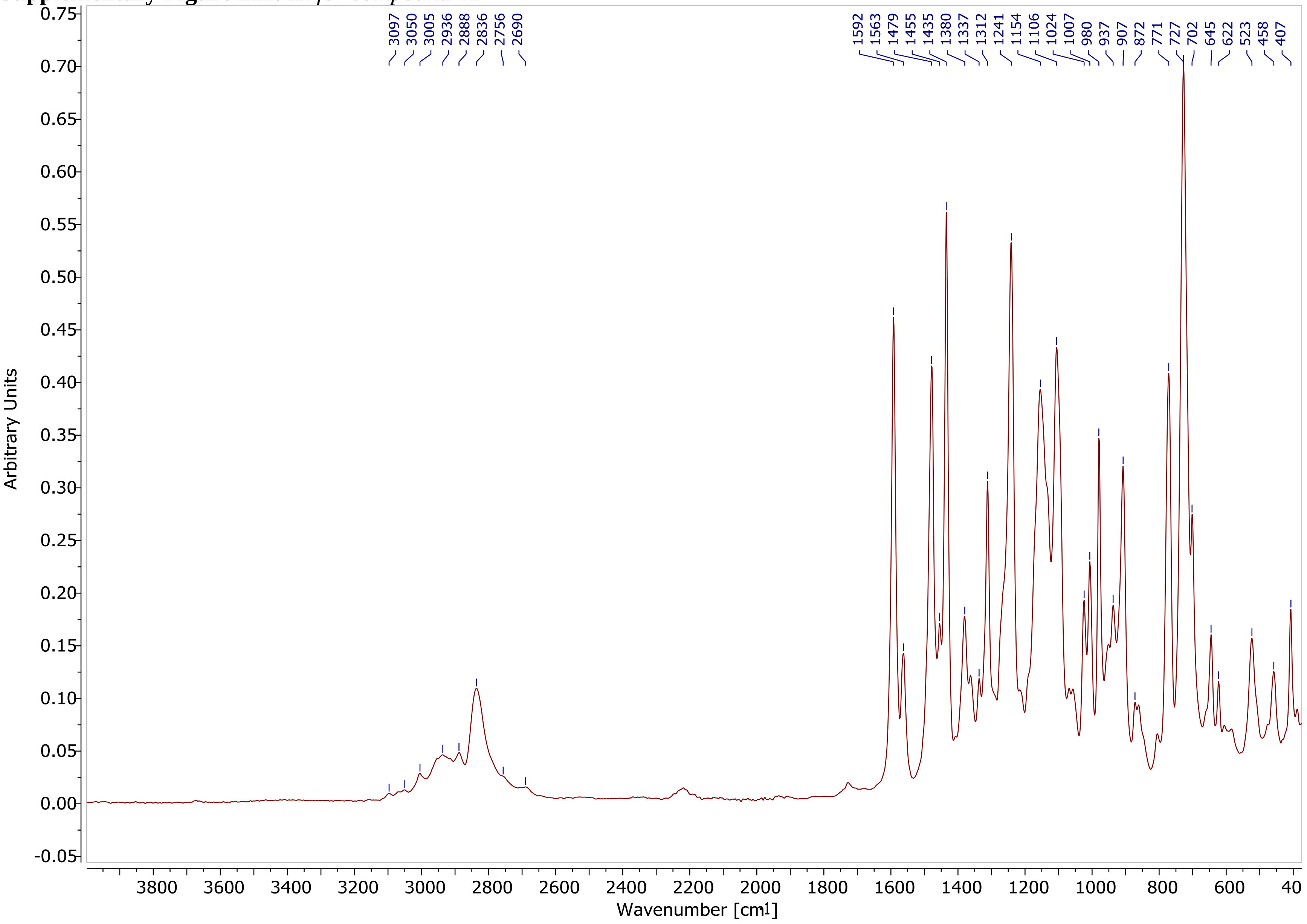

Supplementary Figure 212: *^{1}H-NMR for compound* ***43***

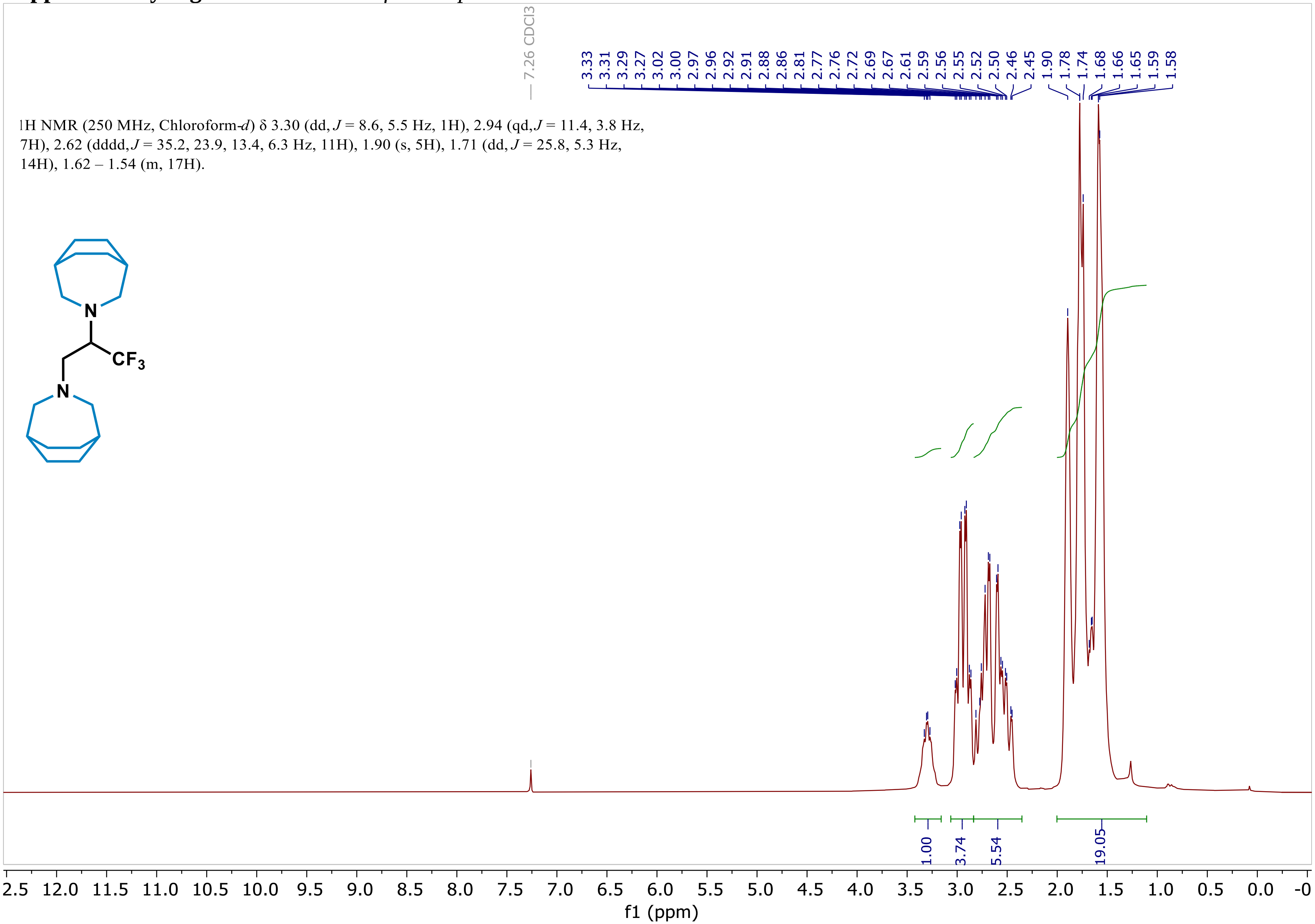

Supplementary Figure 213: *^{19}F NMR for compound* ***43***

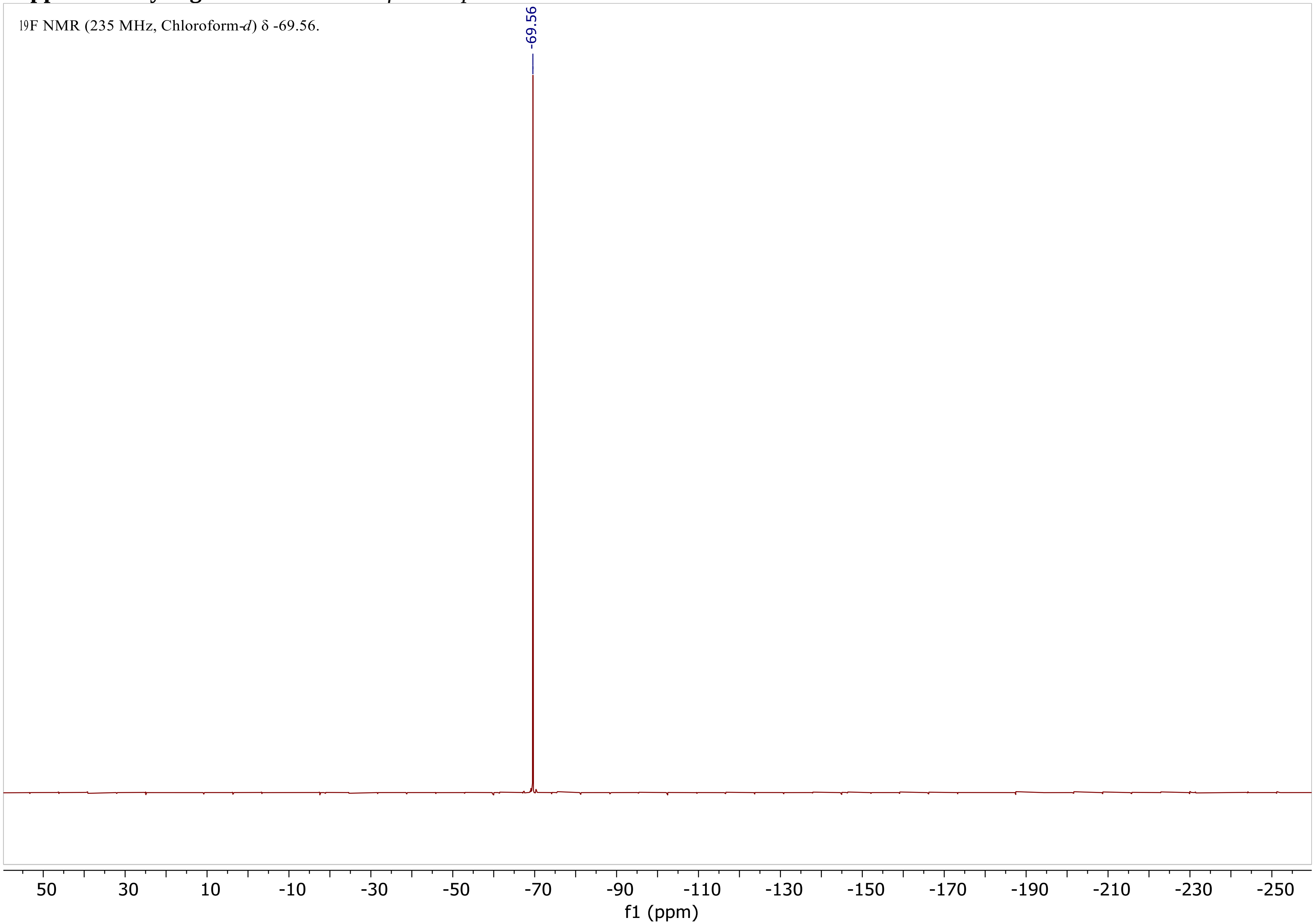

Supplementary Figure 214: *^{13}C NMR for compound* ***43***

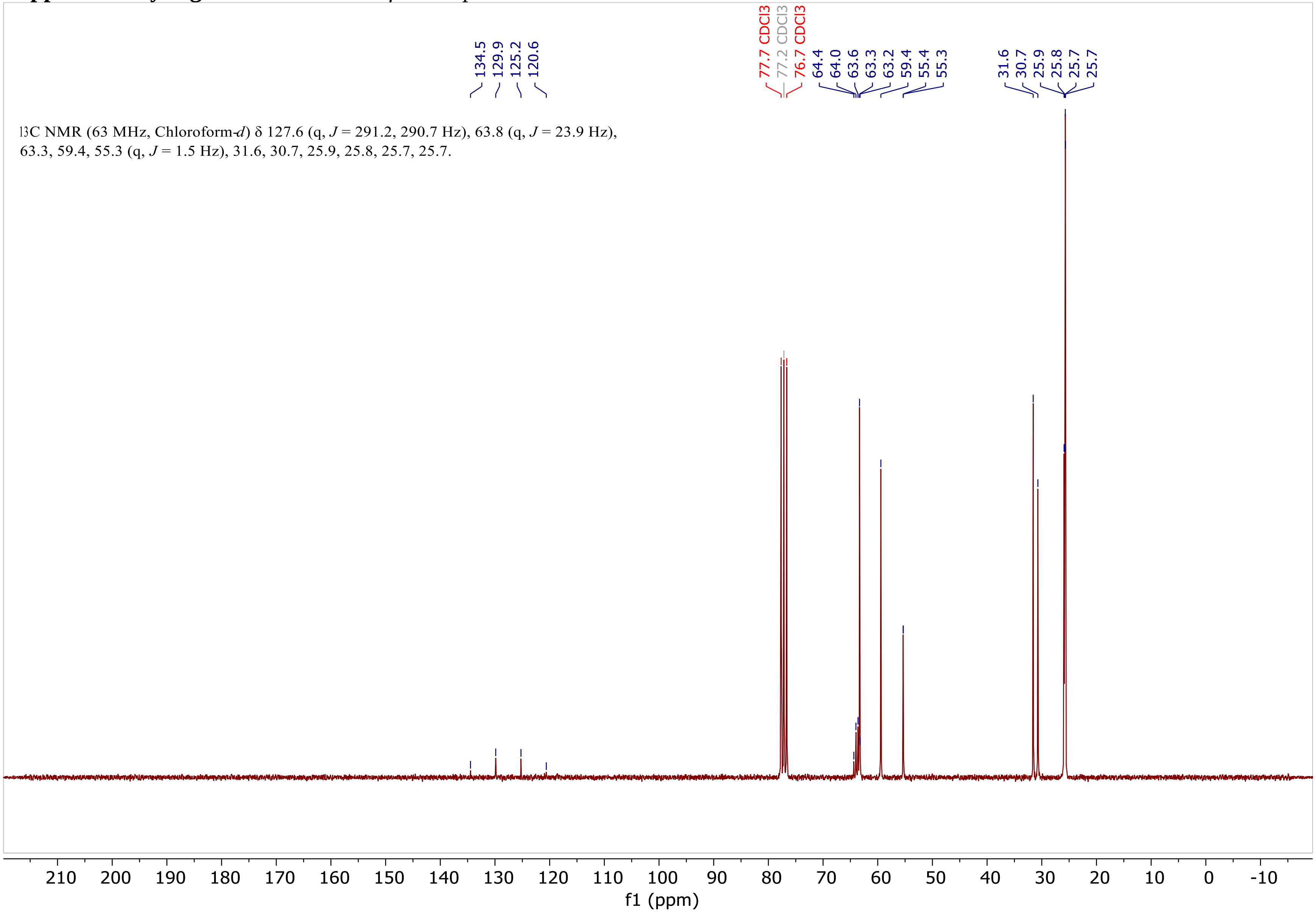

Supplementary Figure 215: *IR for compound* ***43***

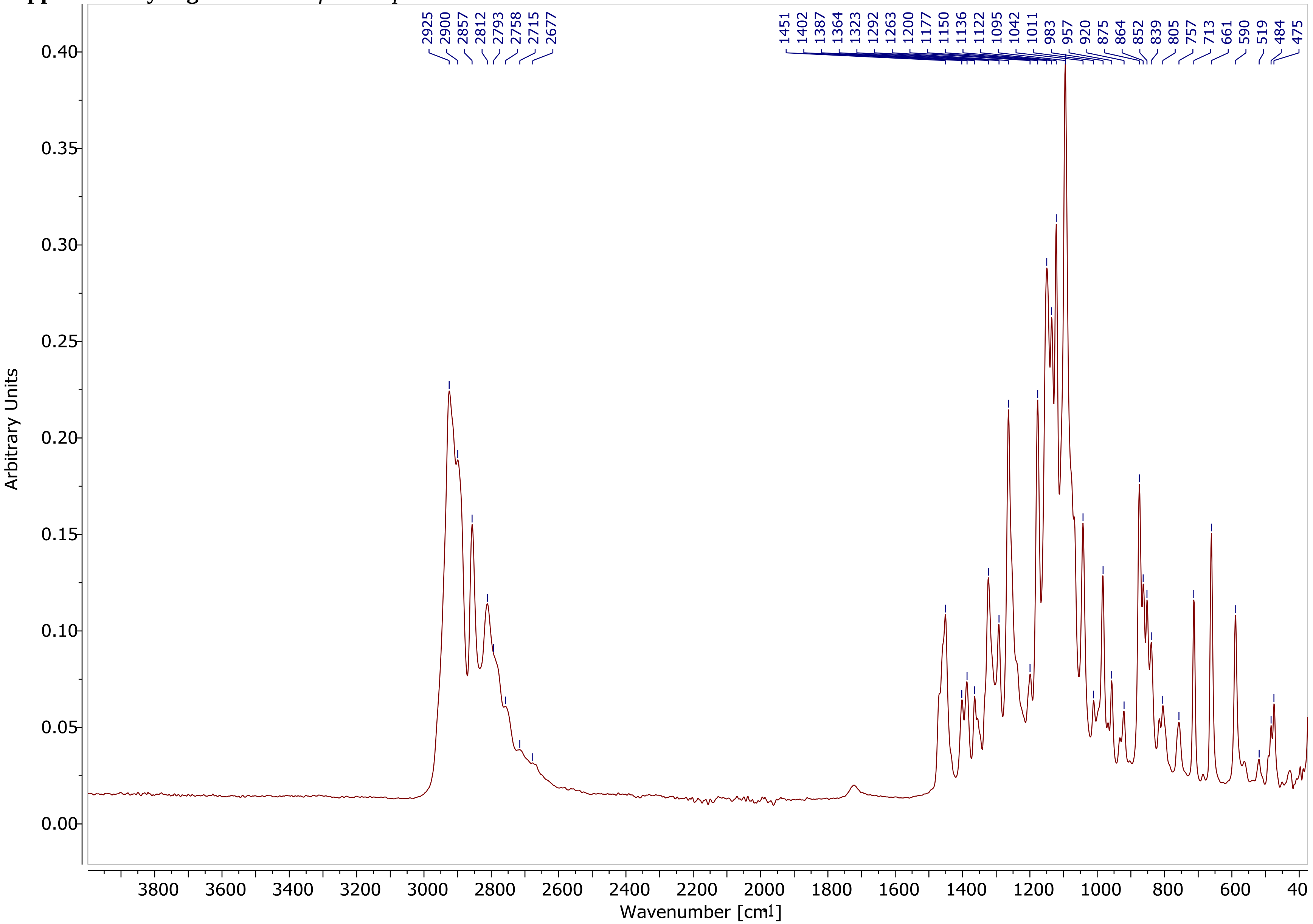

Supplementary Figure 216: *^{1}H-NMR for compound* ***44***

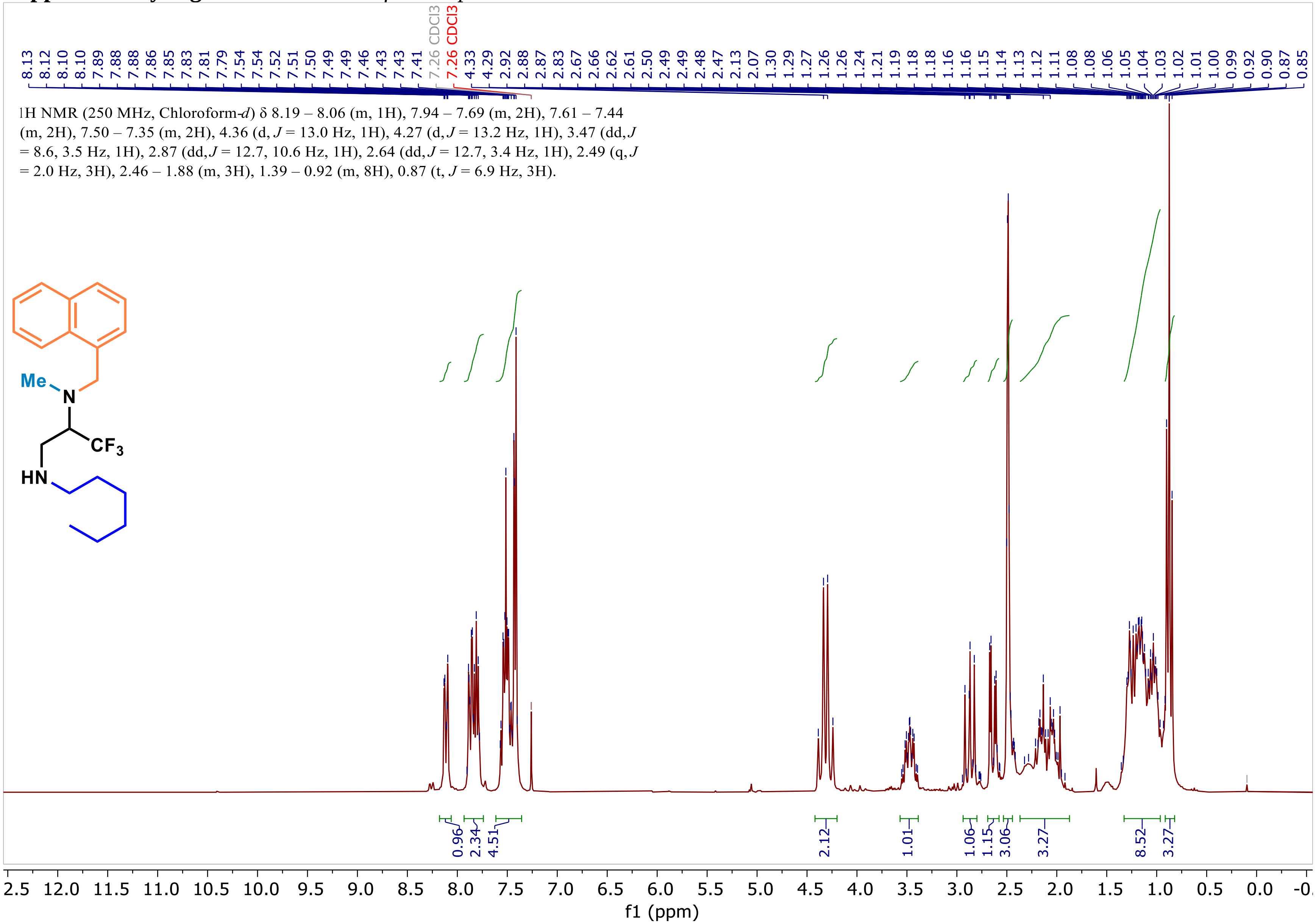

Supplementary Figure 217: *^{19}F NMR for compound* ***44***

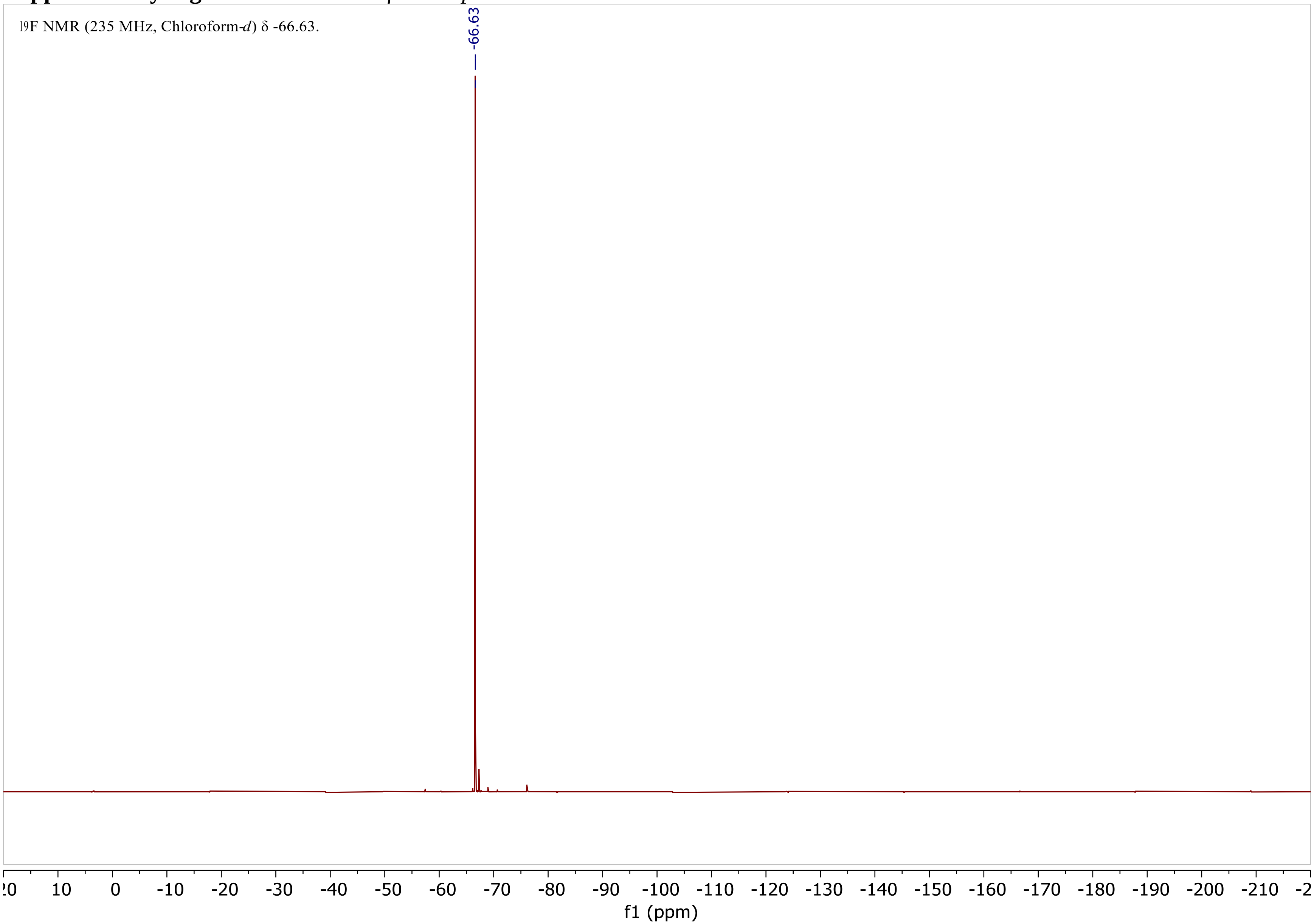

Supplementary Figure 218: *^{13}C NMR for compound* ***44***

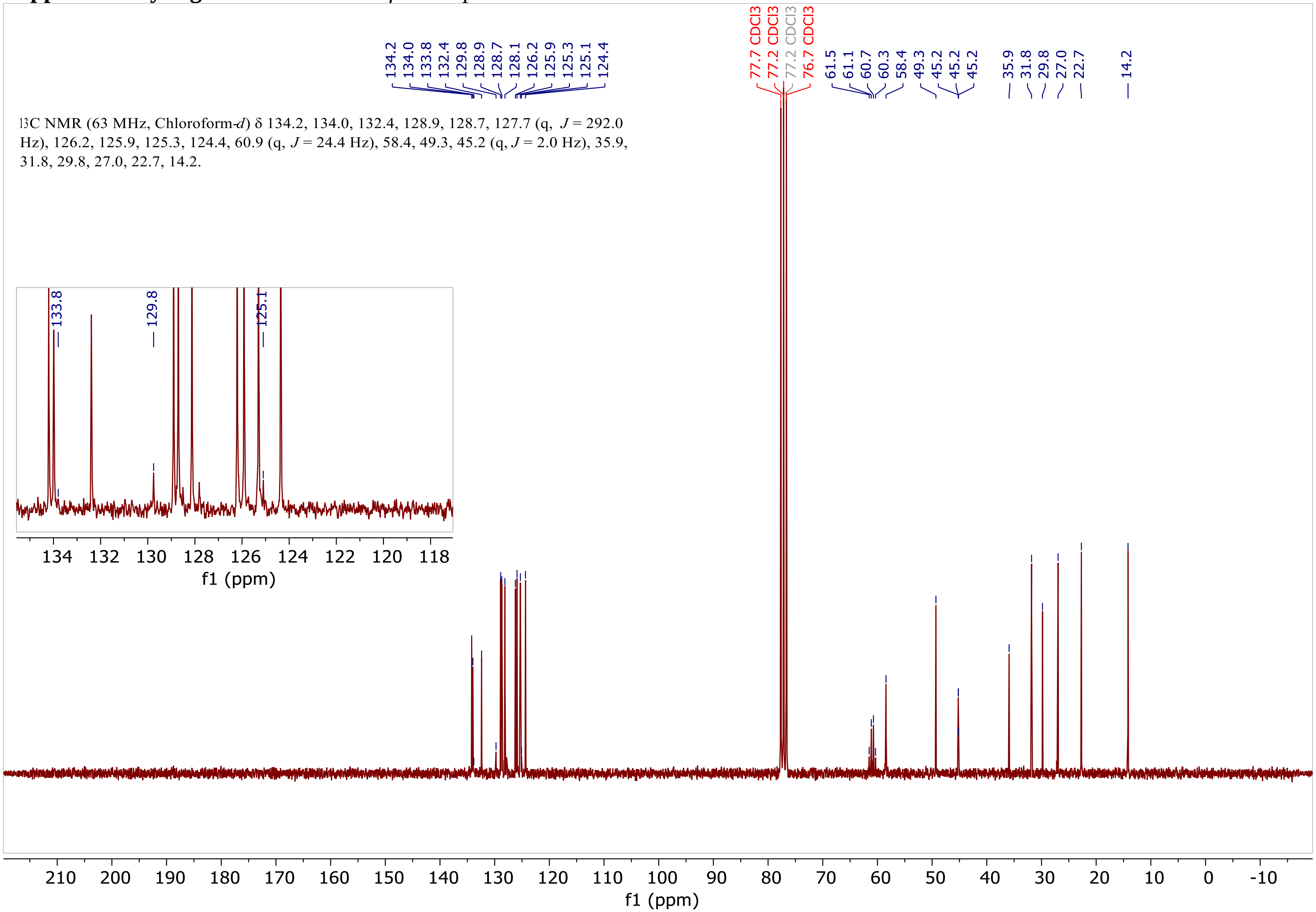

Supplementary Figure 219: *IR for compound **44***

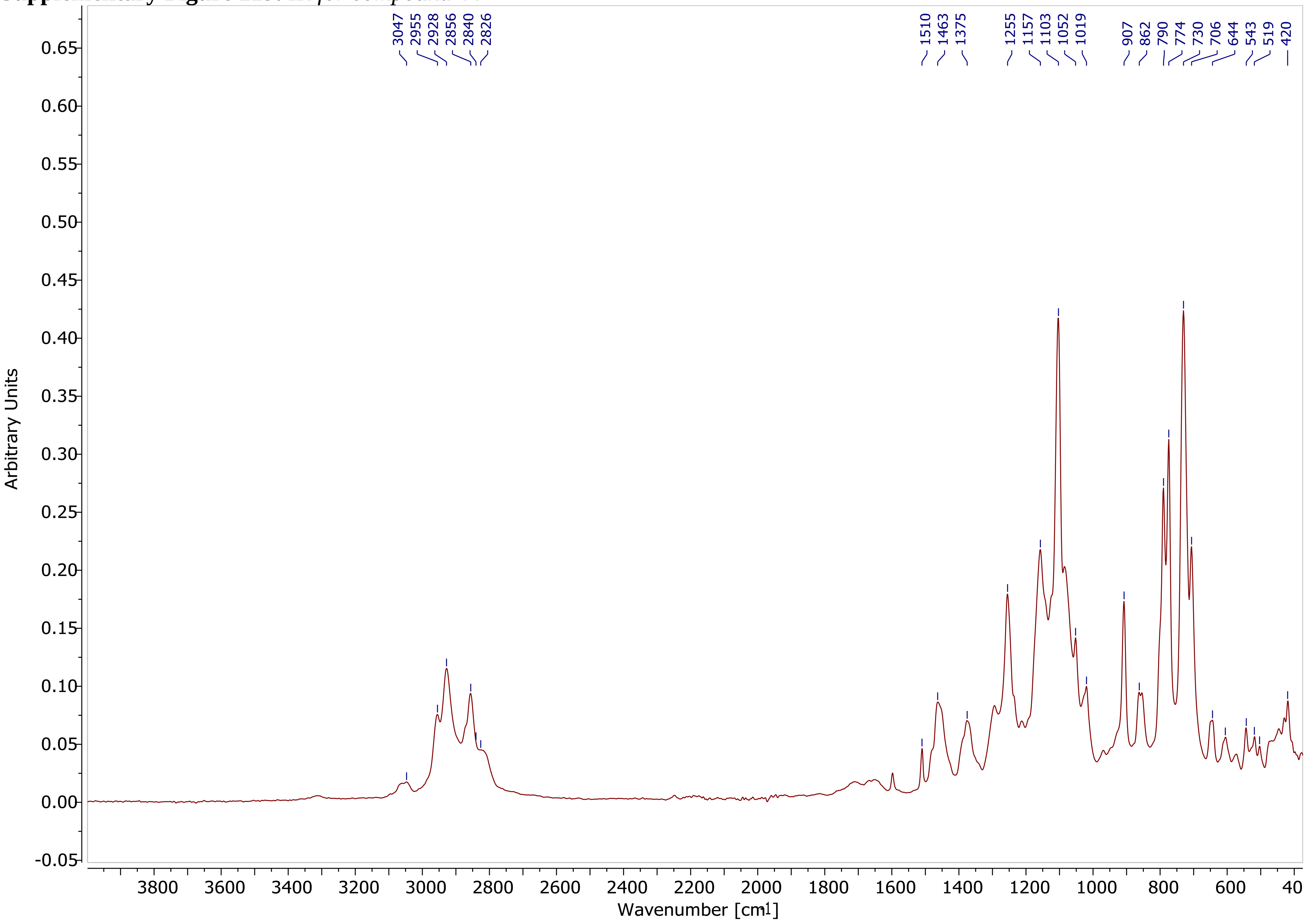

Supplementary Figure 220: *¹H-NMR for compound **45***

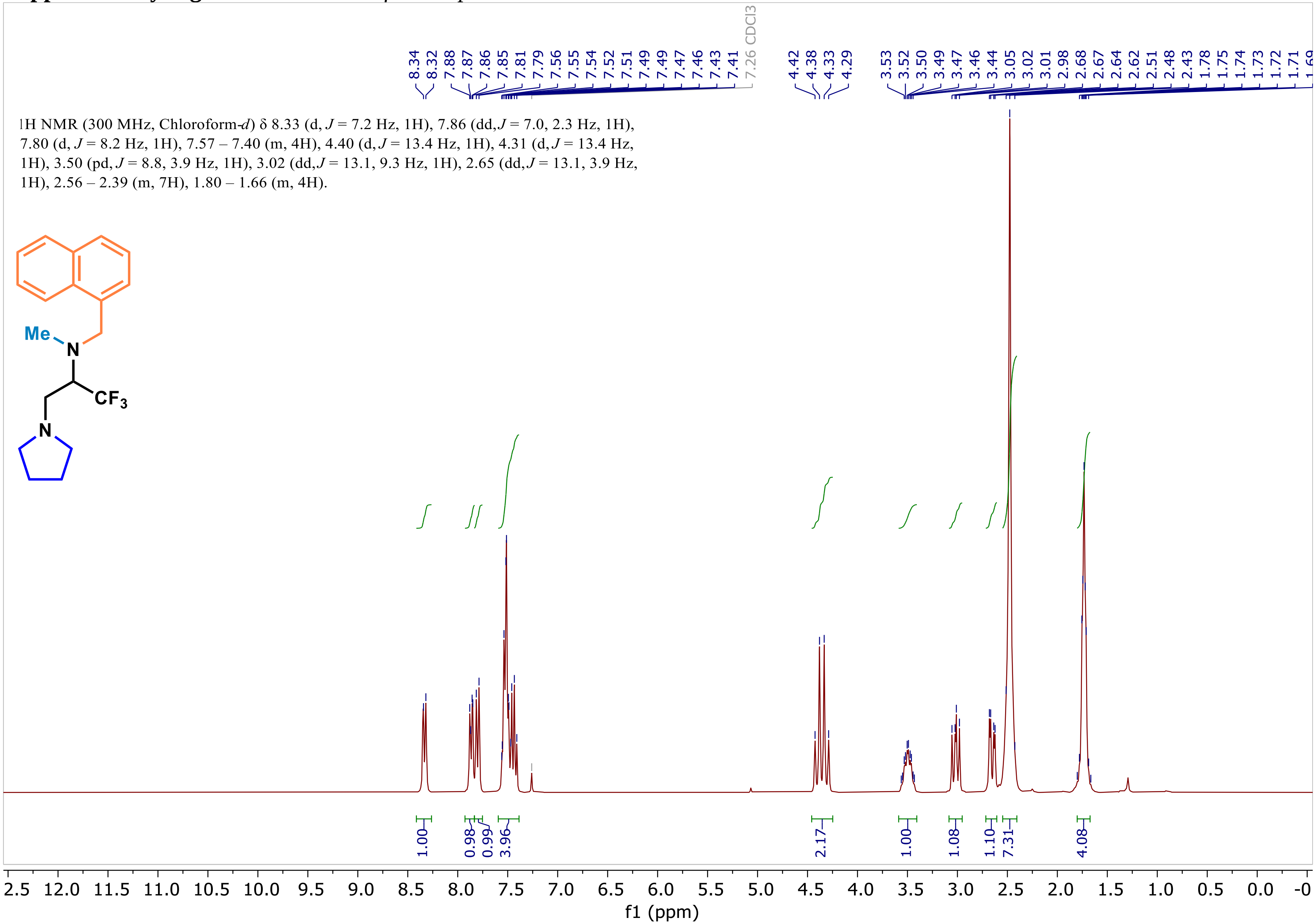

Supplementary Figure 221: *COSY NMR for compound* ***45***

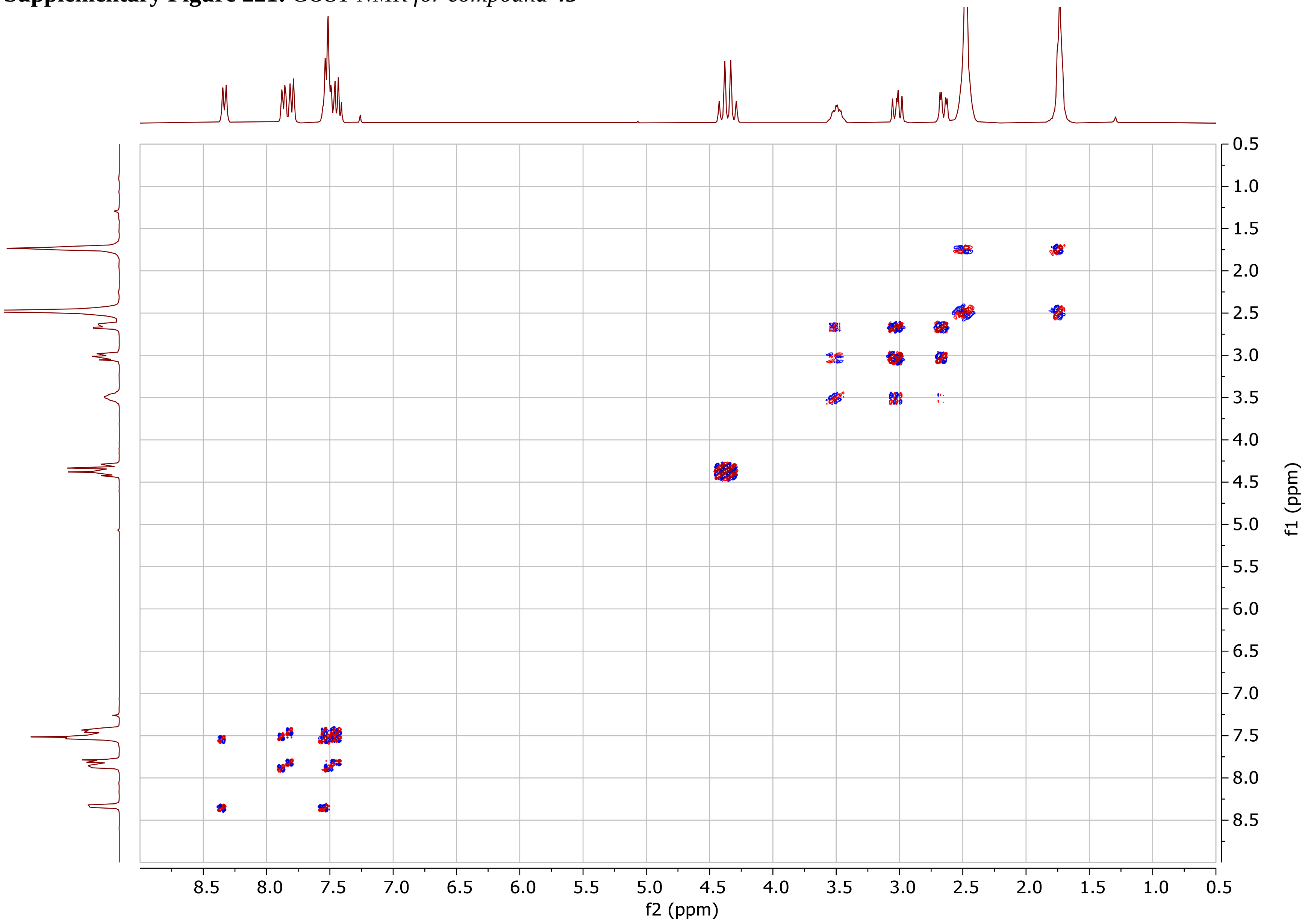

Supplementary Figure 222: *^{19}F NMR for compound* ***45***

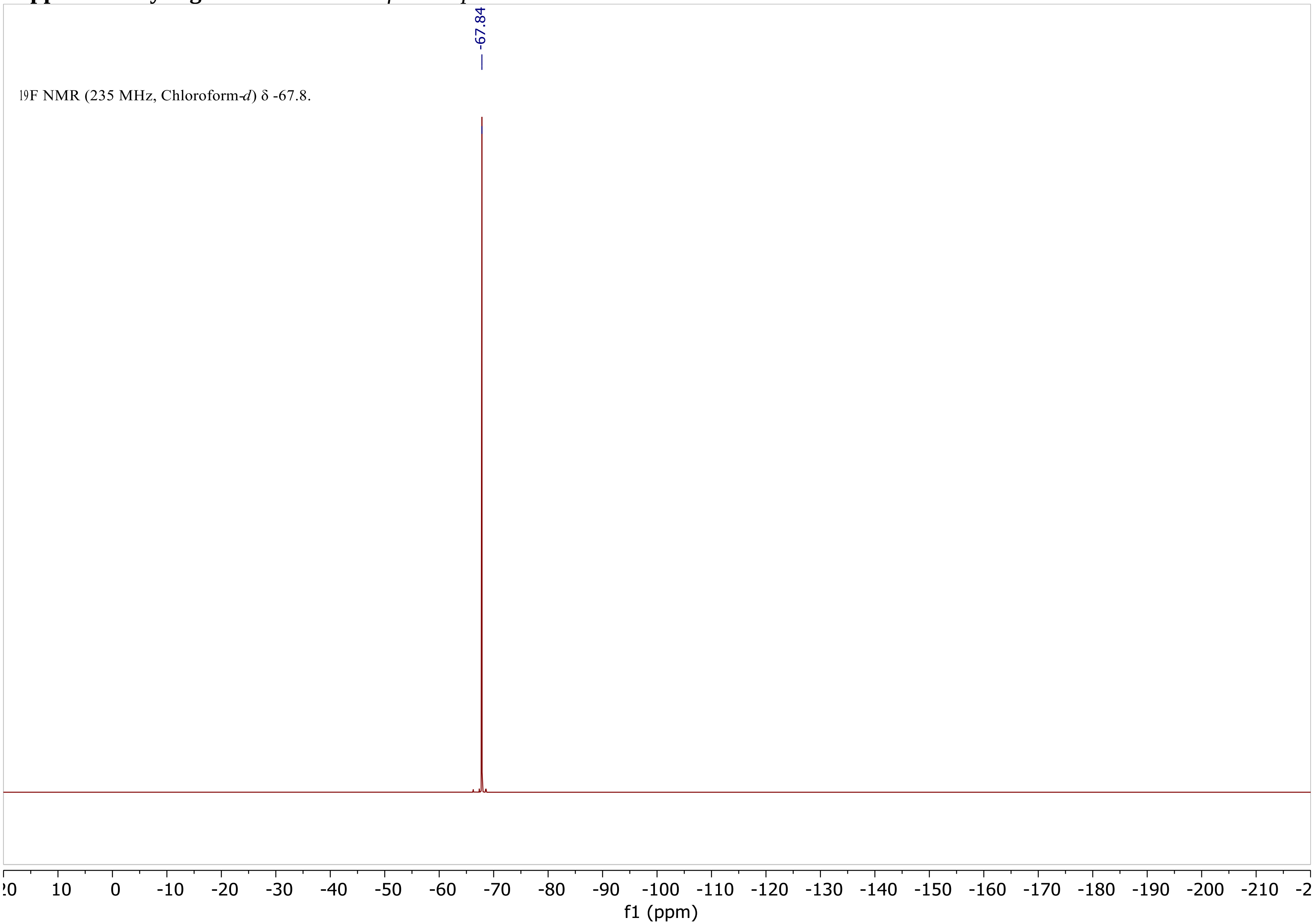

Supplementary Figure 223: ^{13}*C NMR for compound* ***45***

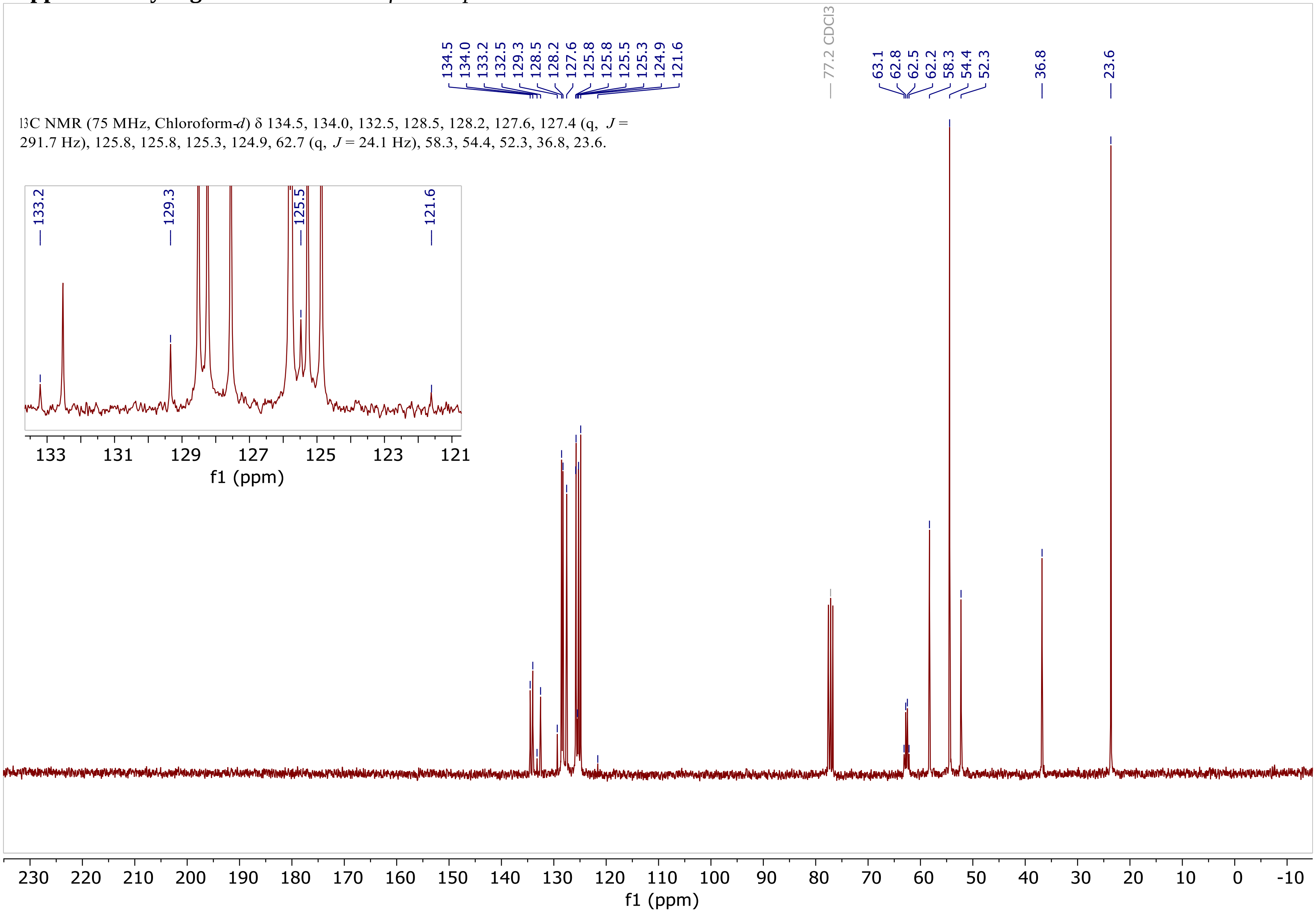

Supplementary Figure 224: *HSQC NMR for compound* ***45***

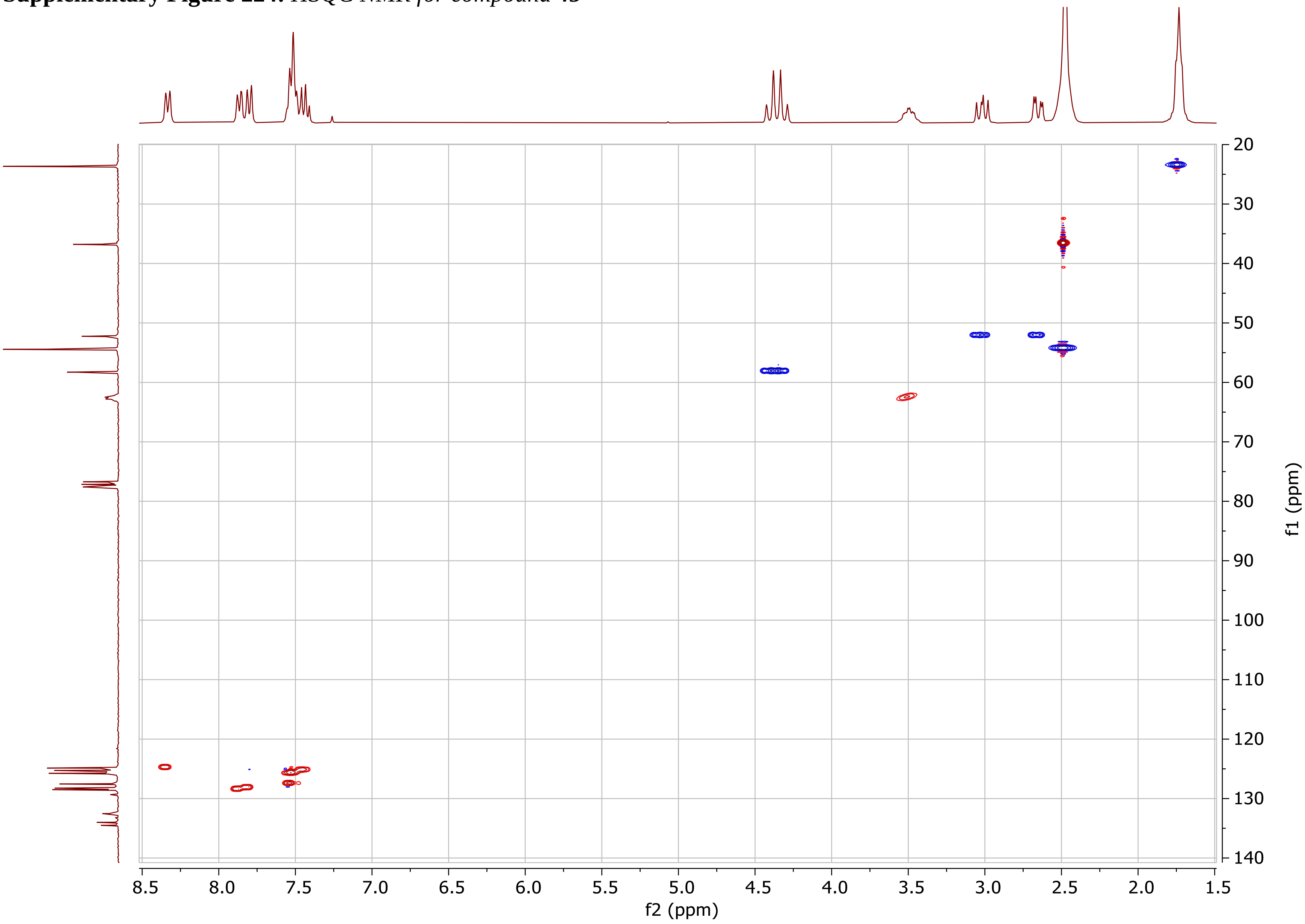

Supplementary Figure 225: *HMBC NMR for compound* ***45***

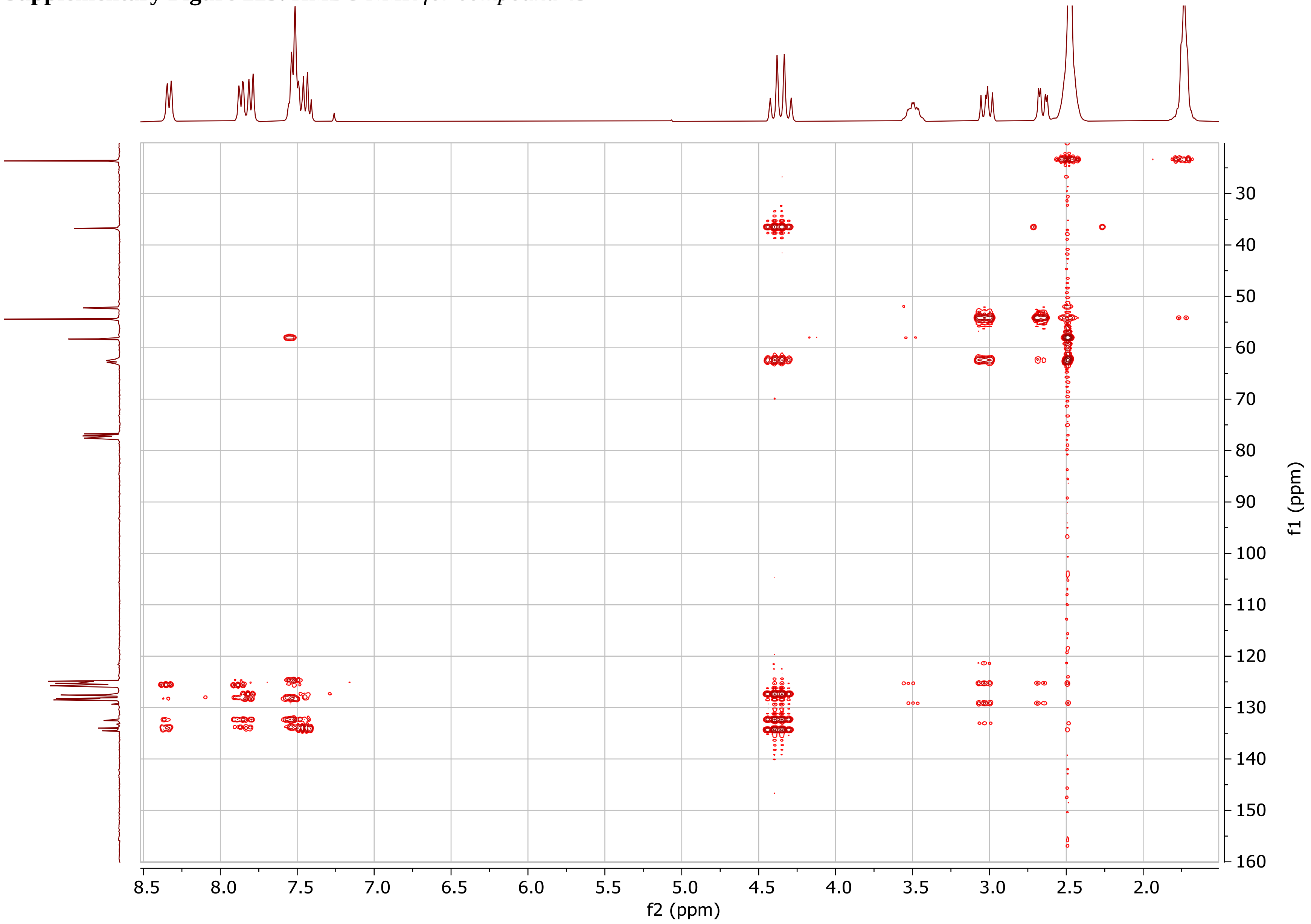

*GC-MS TIC for compound **45***

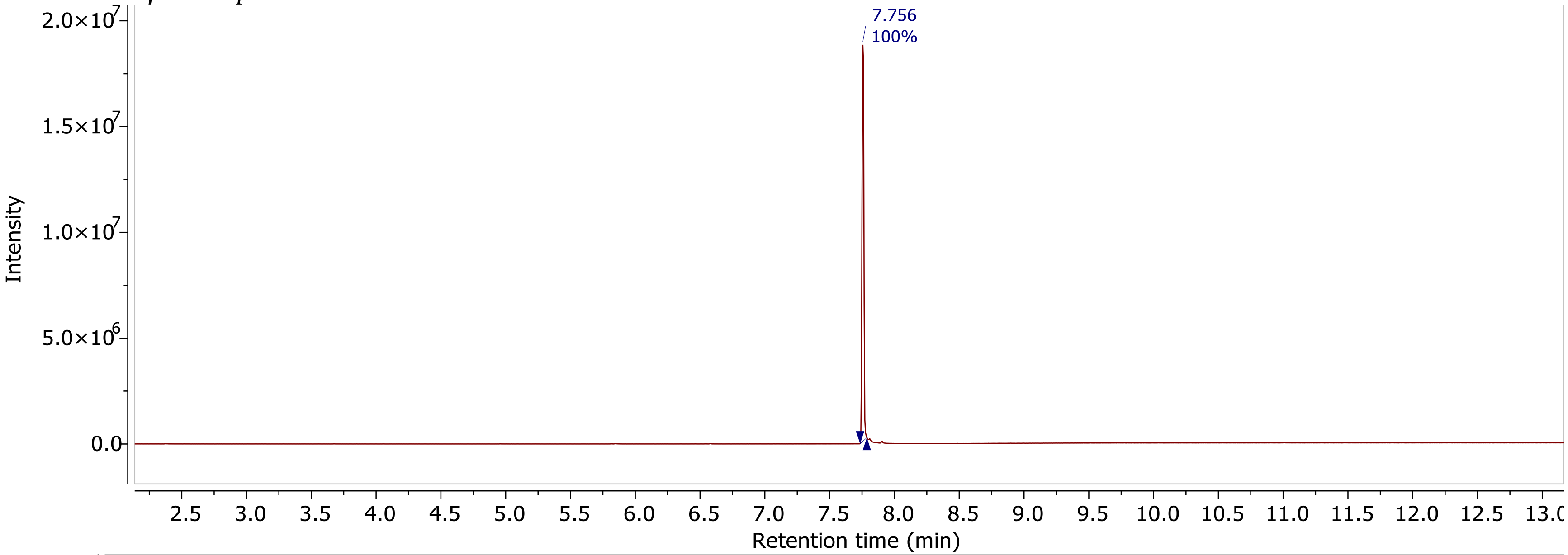

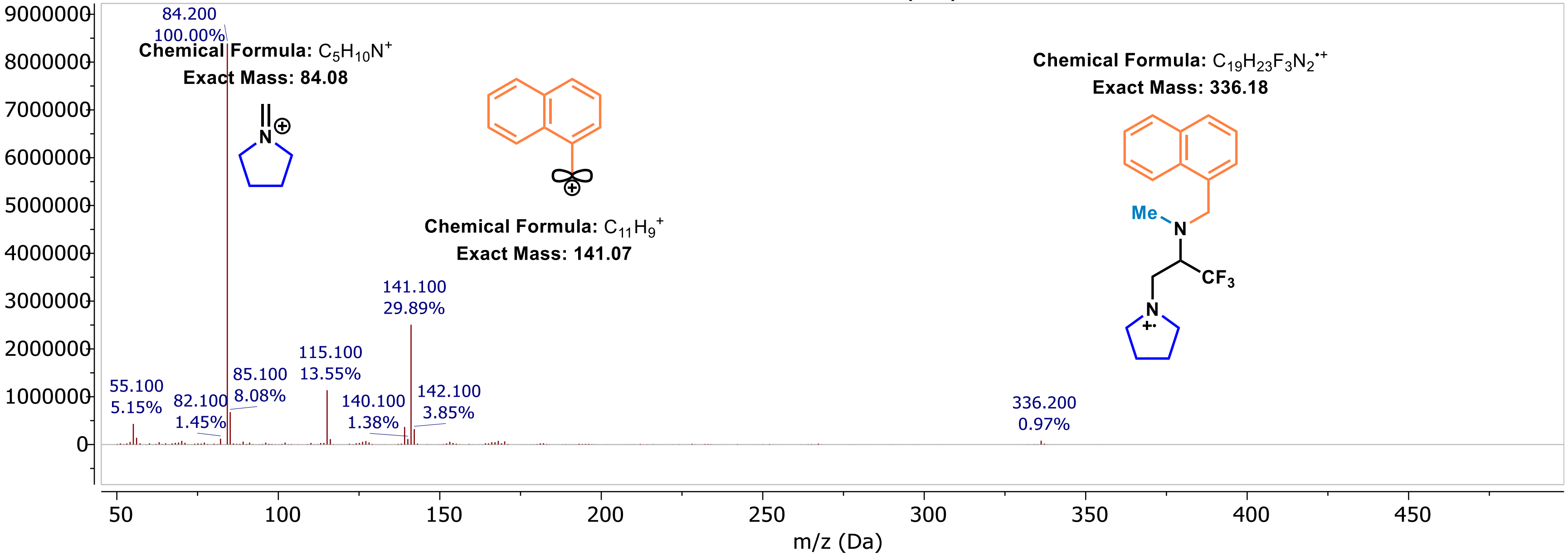

Supplementary Figure 226: *IR for compound **45***

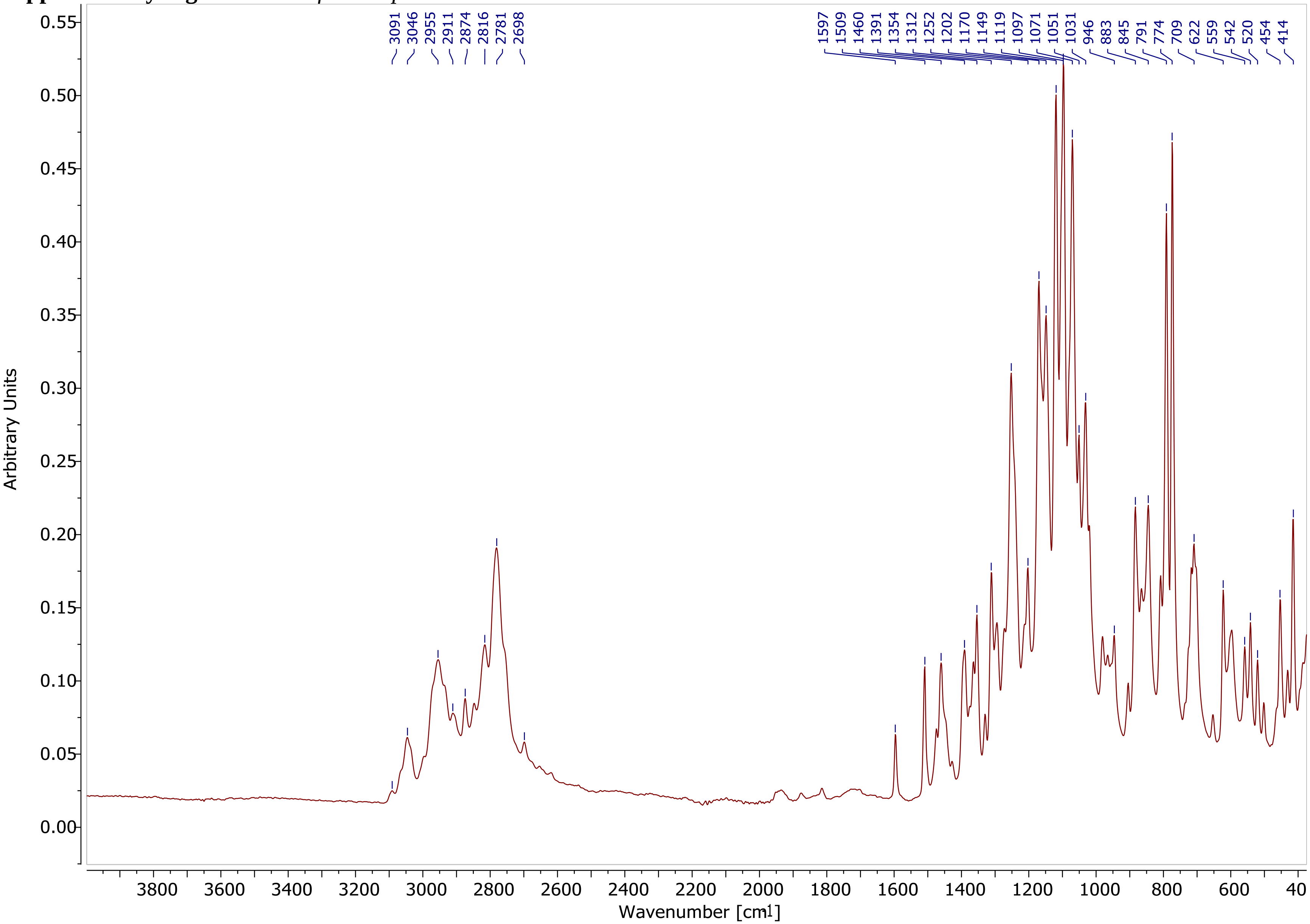

Supplementary Figure 227: *^{1}H-NMR for compound* ***46***

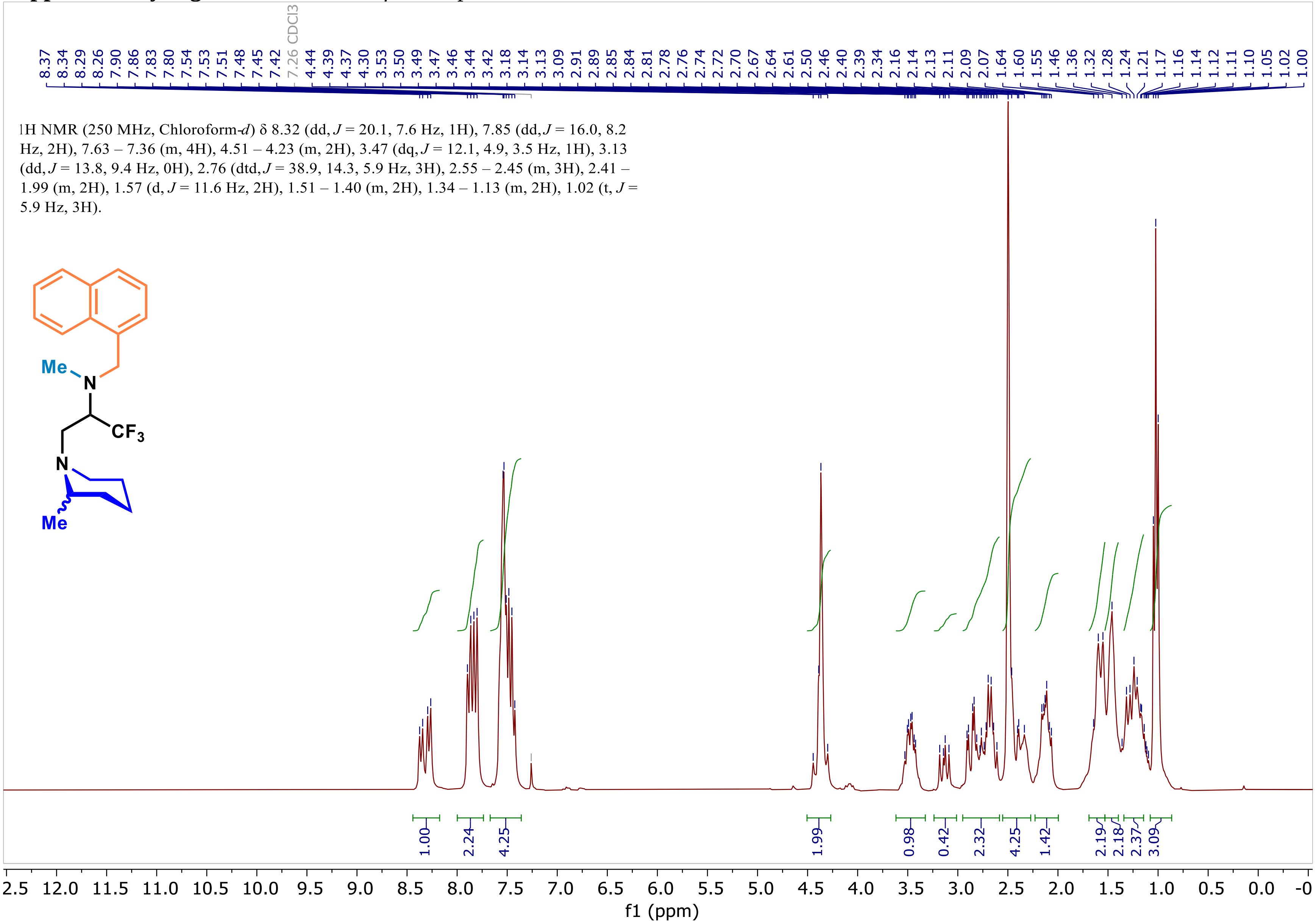

Supplementary Figure 228: *^{19}F NMR for compound* ***46***

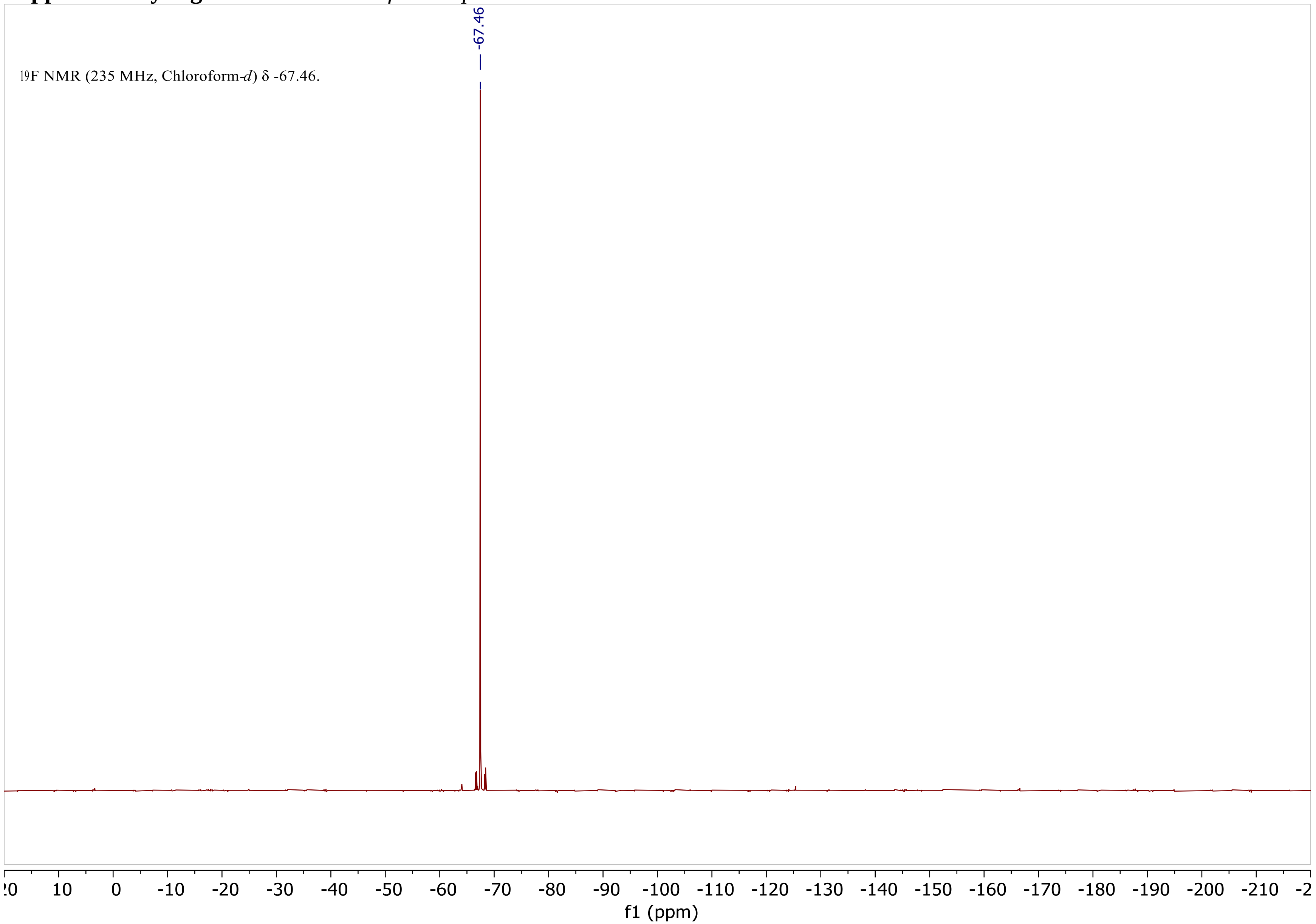

Supplementary Figure 229: *^{13}C NMR for compound* ***46***

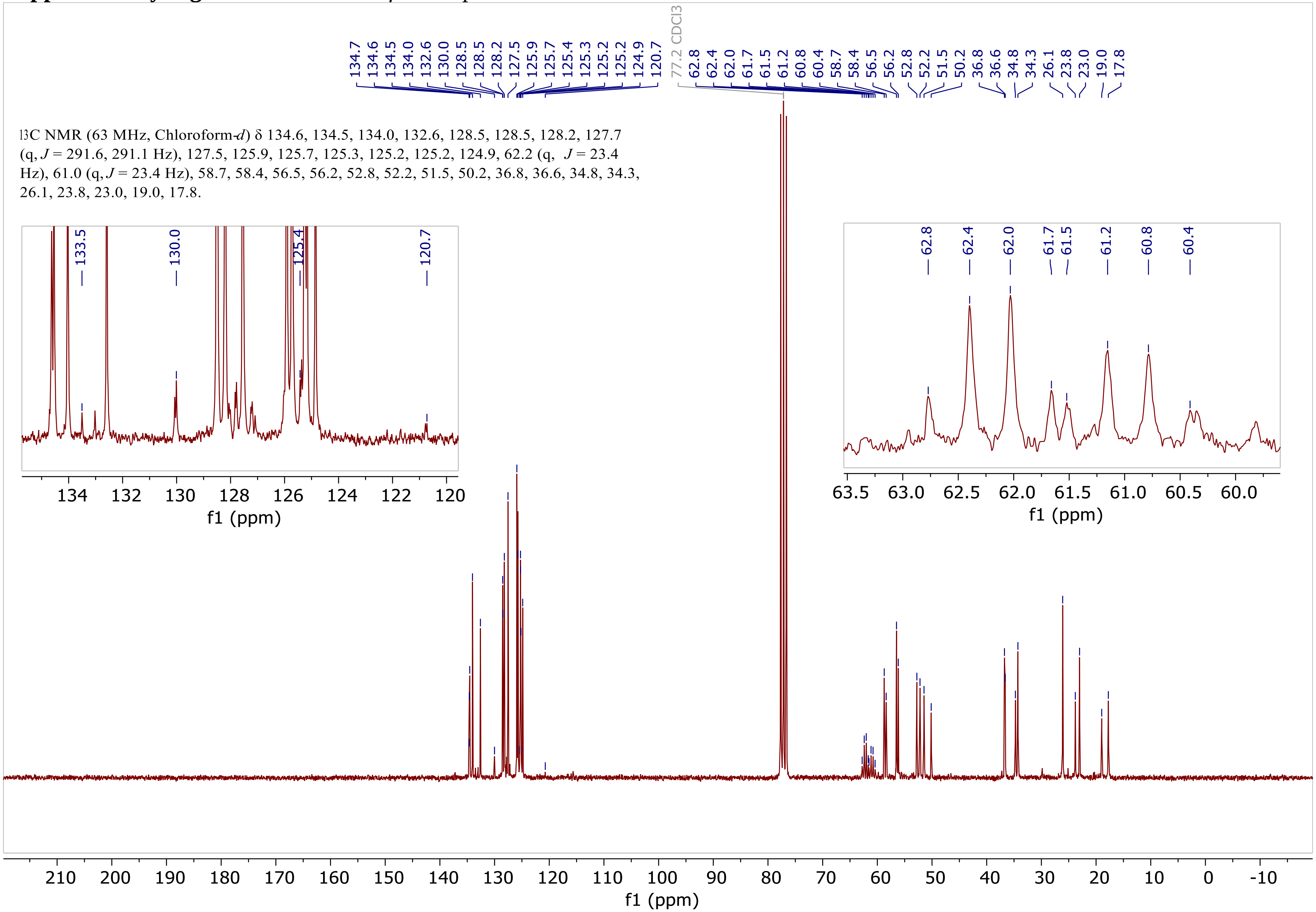

Supplementary Figure 230: *IR for compound **46***

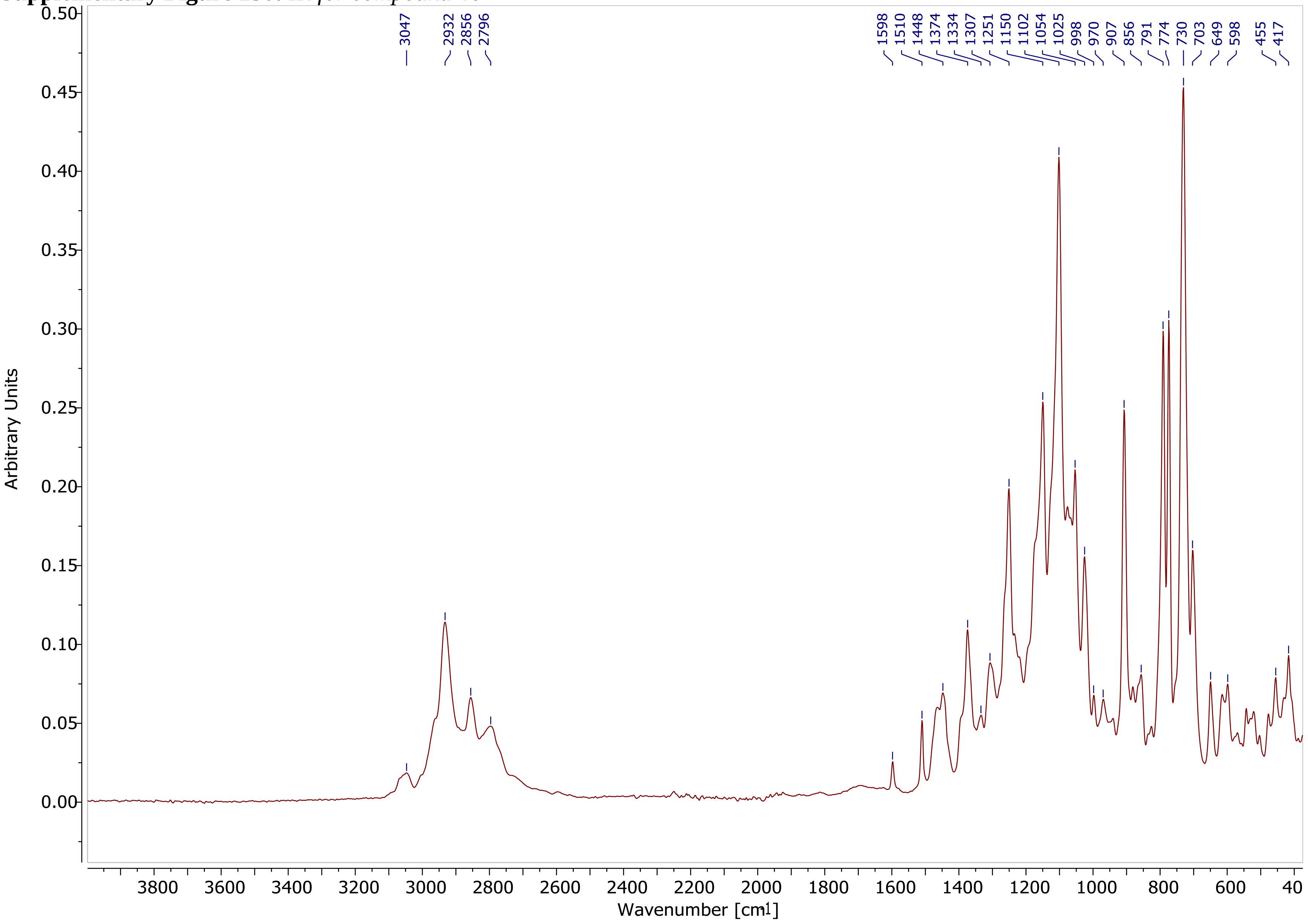

Supplementary Figure 231: *¹H-NMR for compound* ***47***

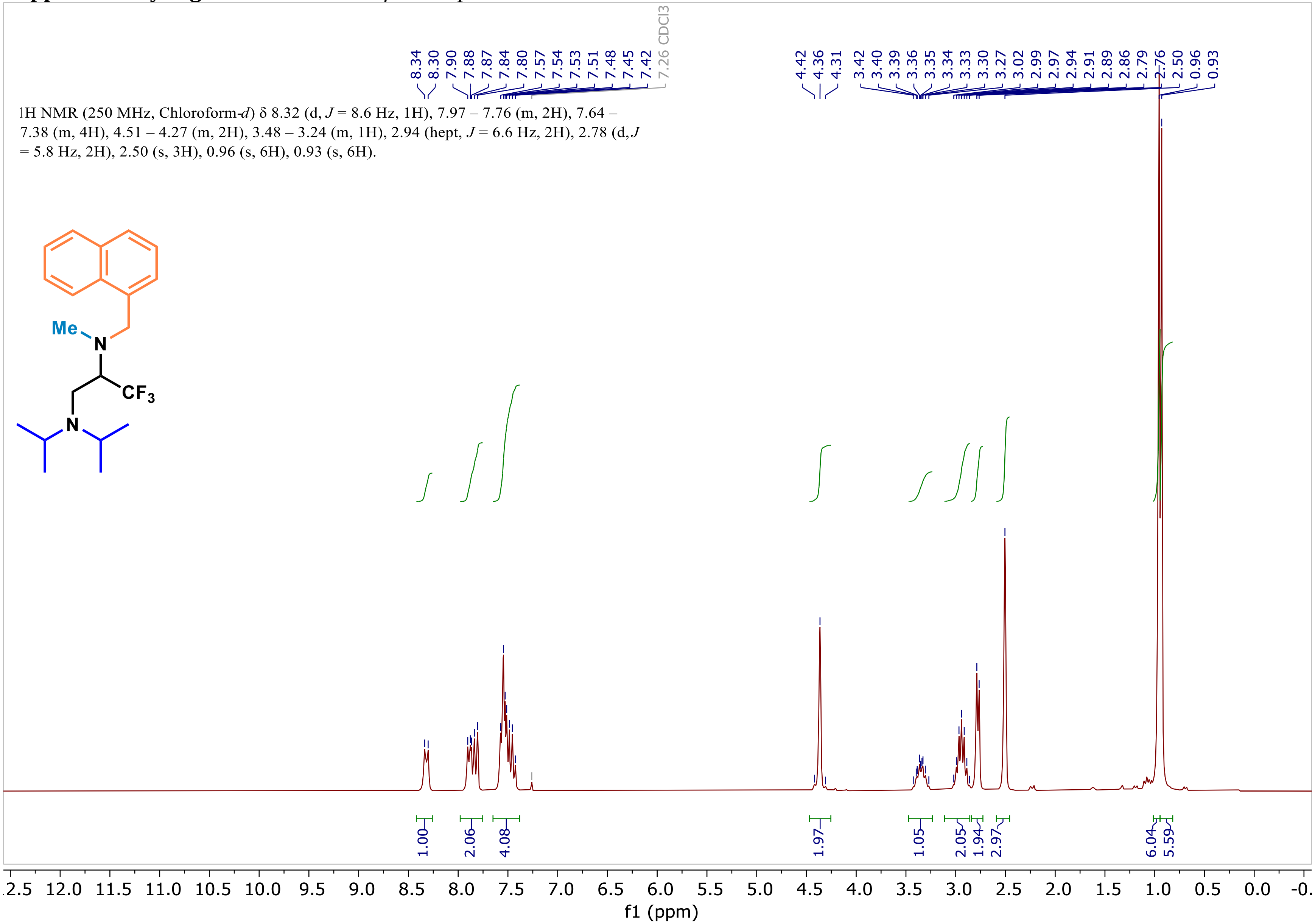

Supplementary Figure 232: *^{19}F NMR for compound* ***47***

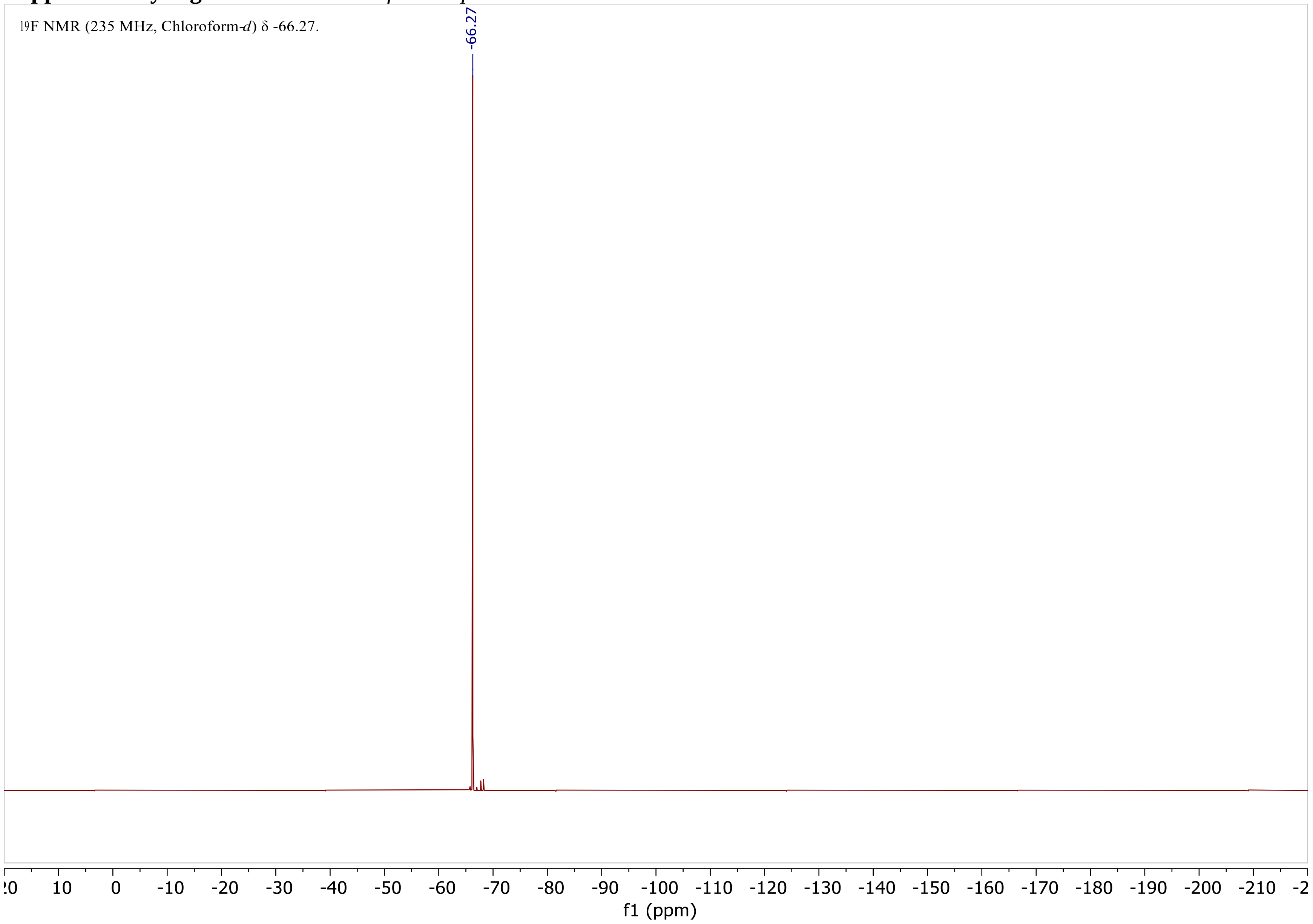

Supplementary Figure 233: *^{13}C NMR for compound* ***47***

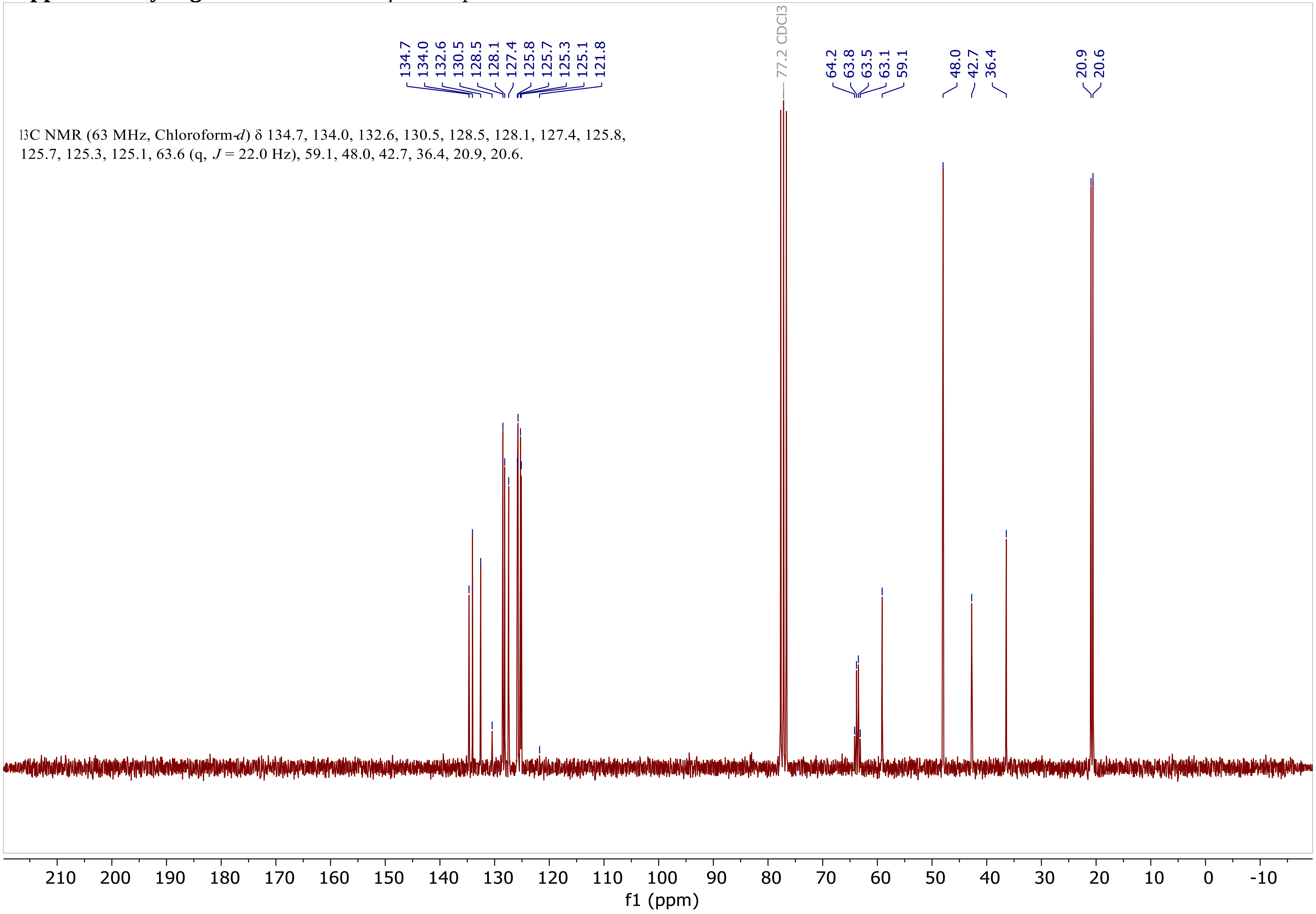

Supplementary Figure 234: *IR for compound* ***47***

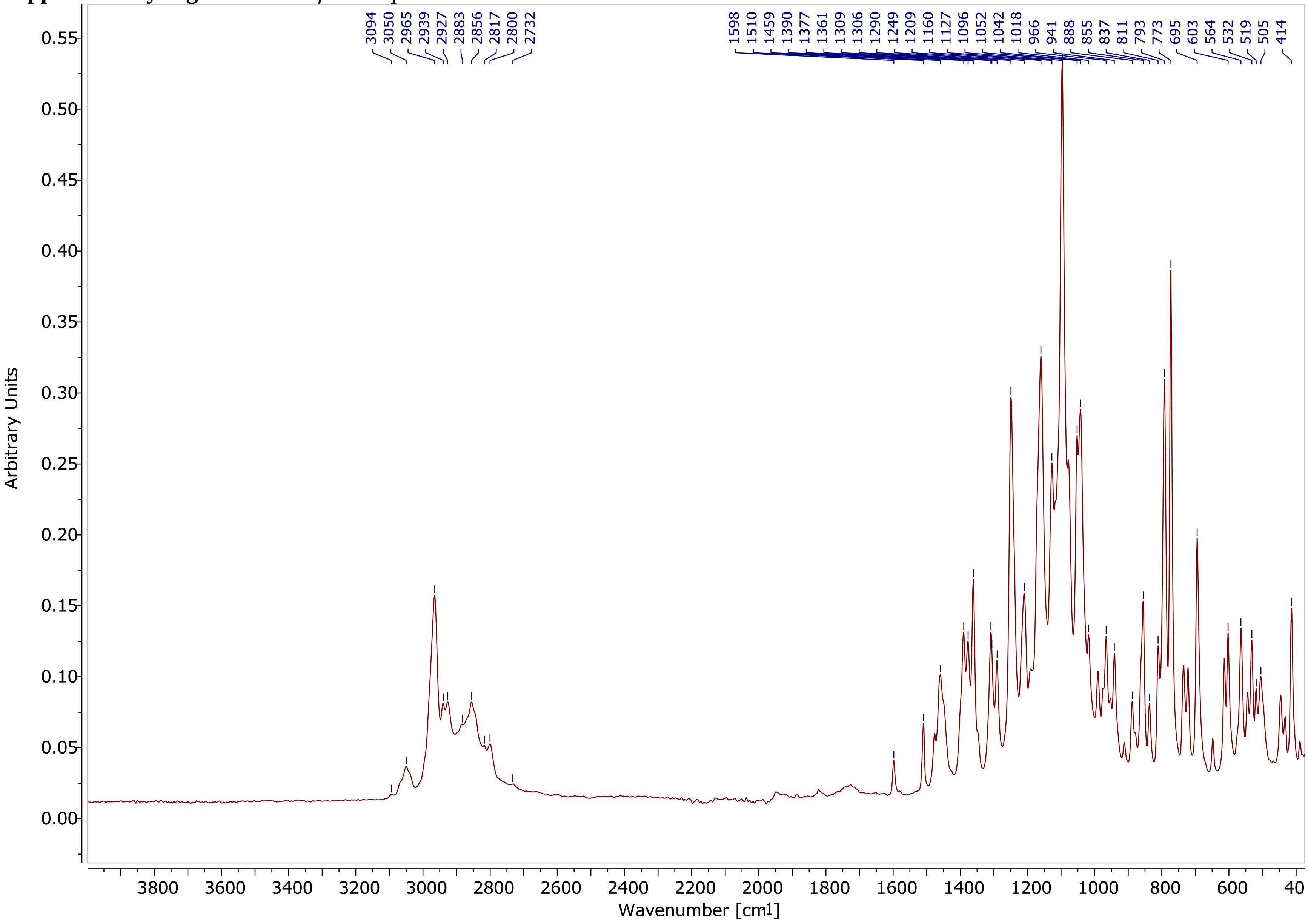

Supplementary Figure 235: *^{1}H-NMR for compound* ***48***

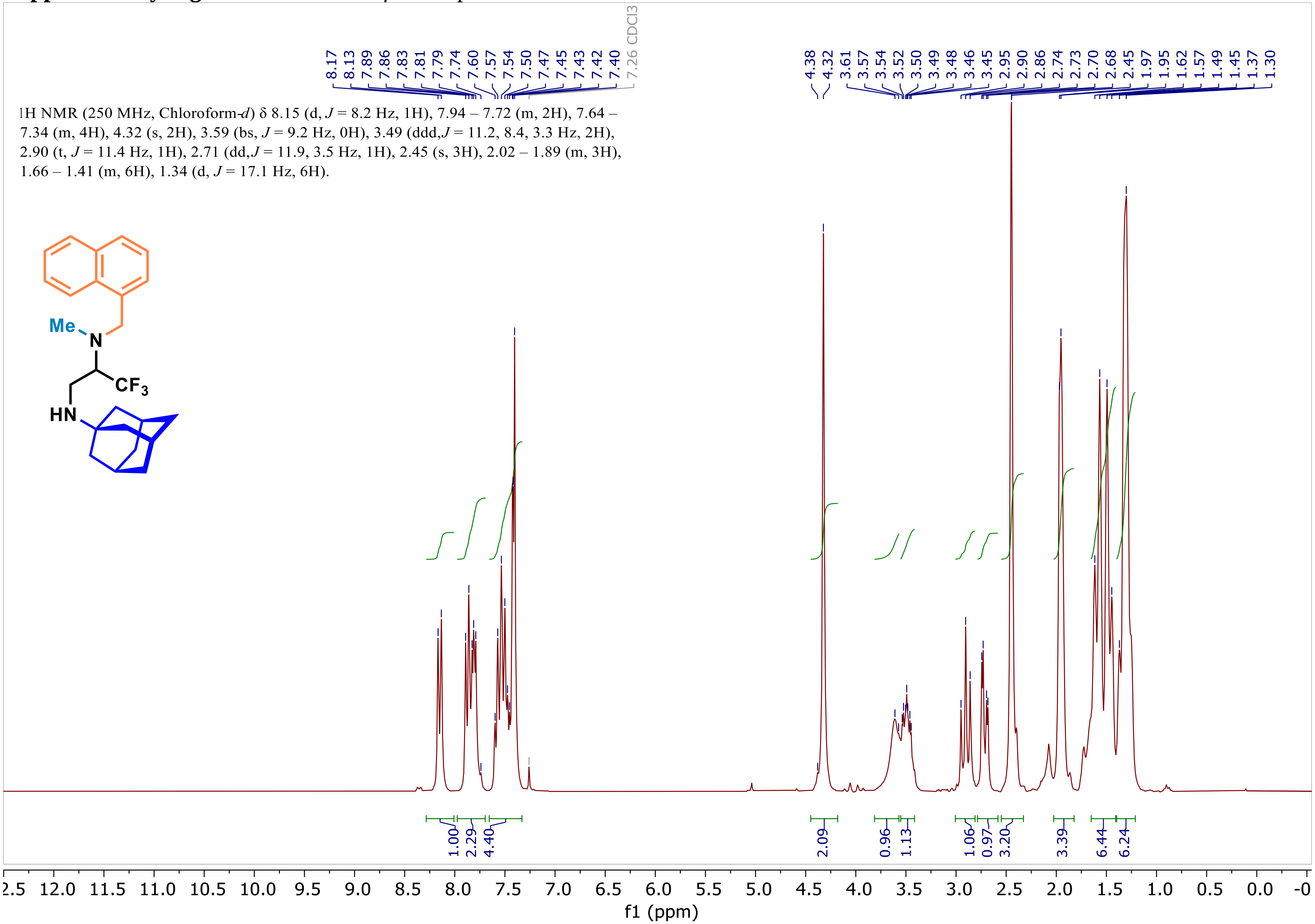

Supplementary Figure 236: *^{19}F NMR for compound* ***48***

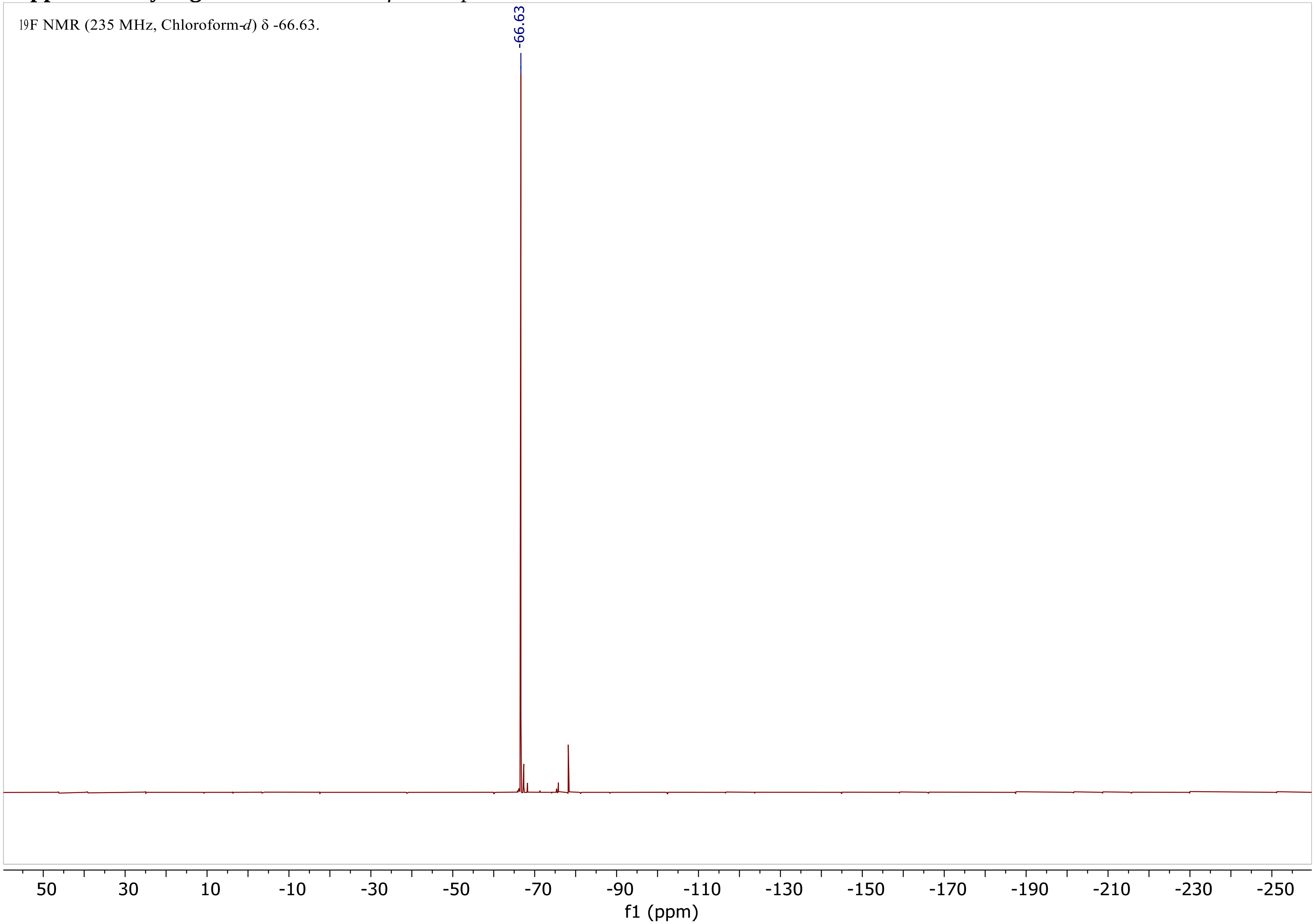

Supplementary Figure 237: *^{13}C NMR for compound* ***48***

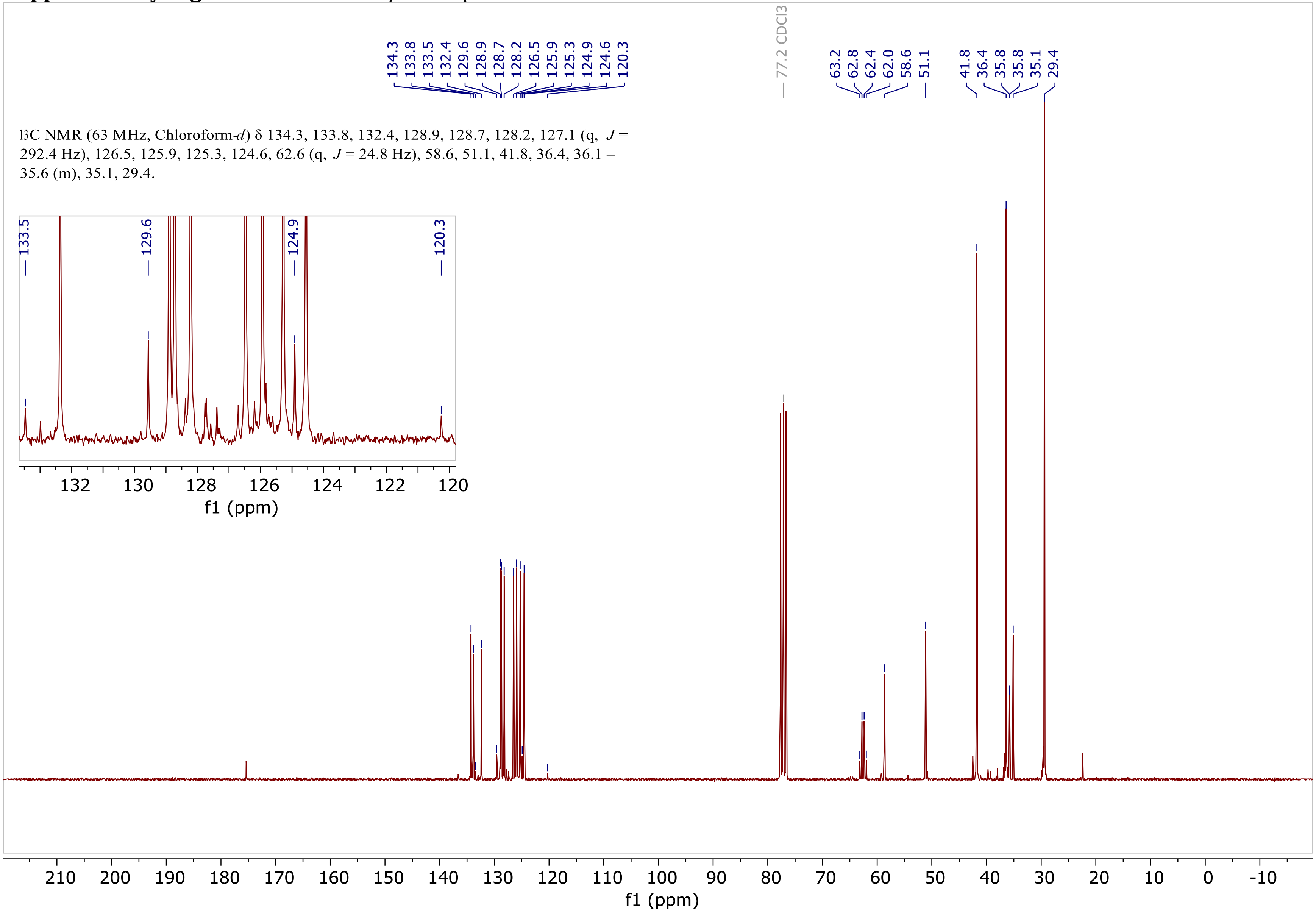

Supplementary Figure 238: *IR for compound* ***48***

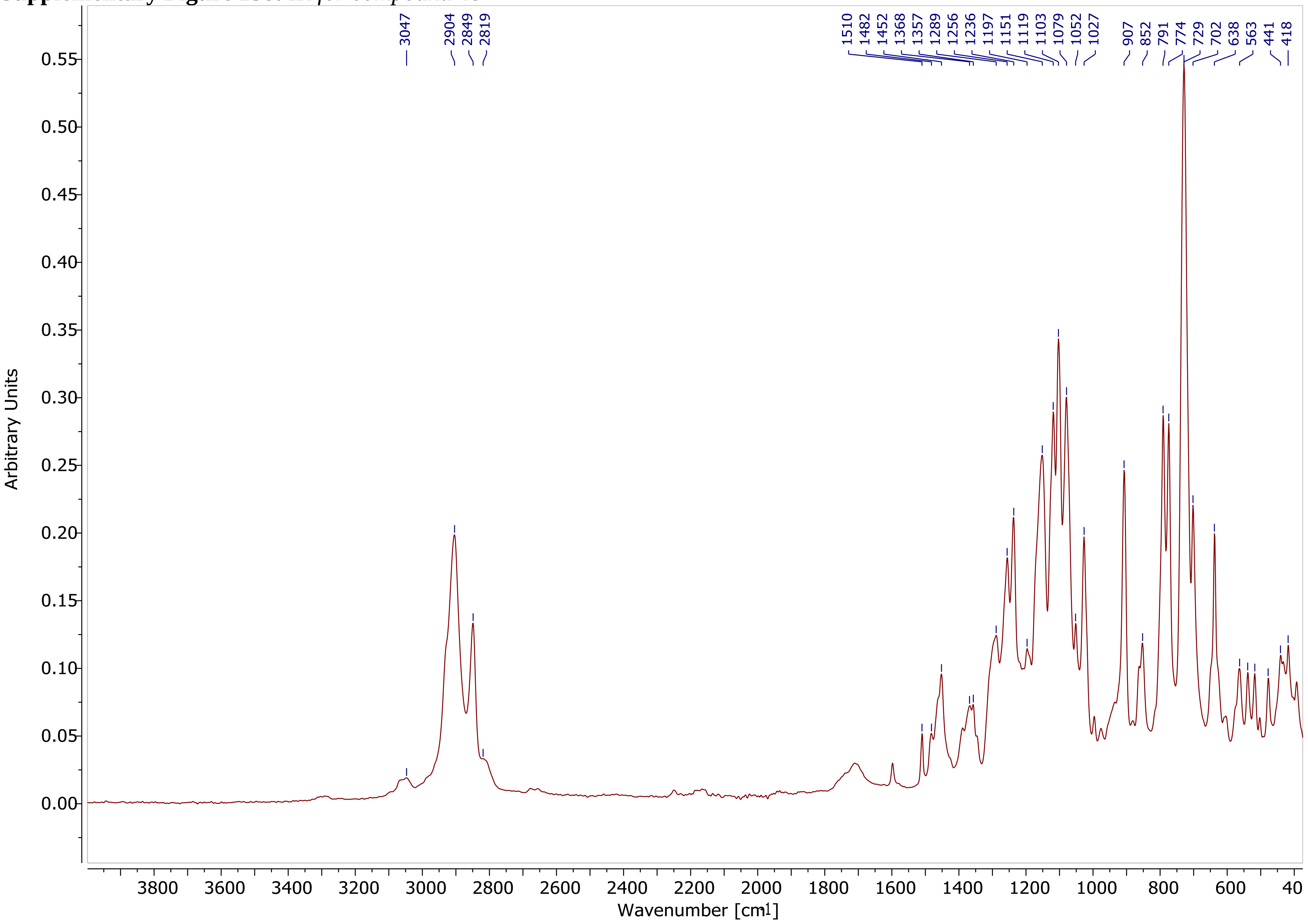

Supplementary Figure 239: *^{1}H-NMR for compound* ***49***

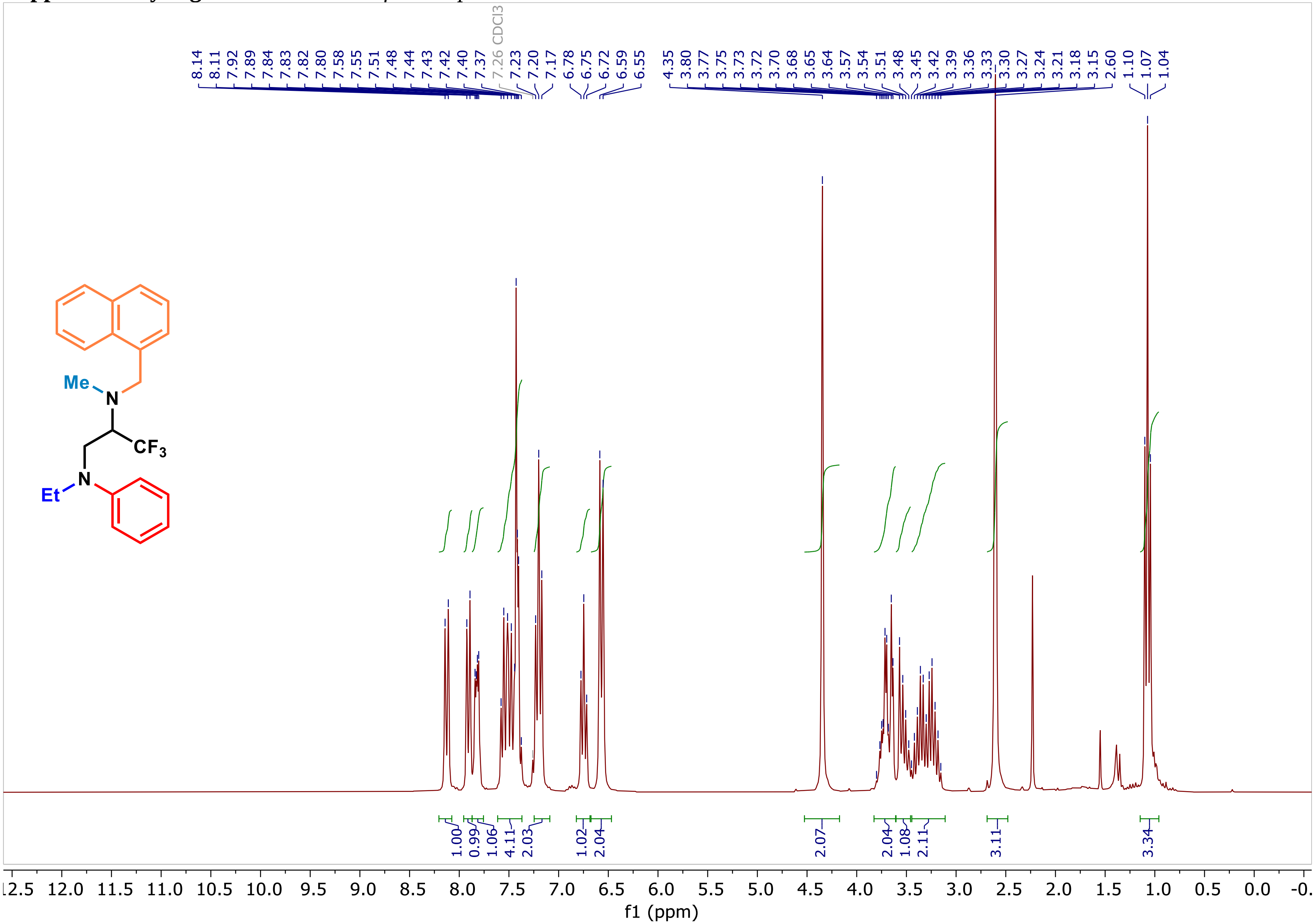

Supplementary Figure 240: *^{19}F NMR for compound* ***49***

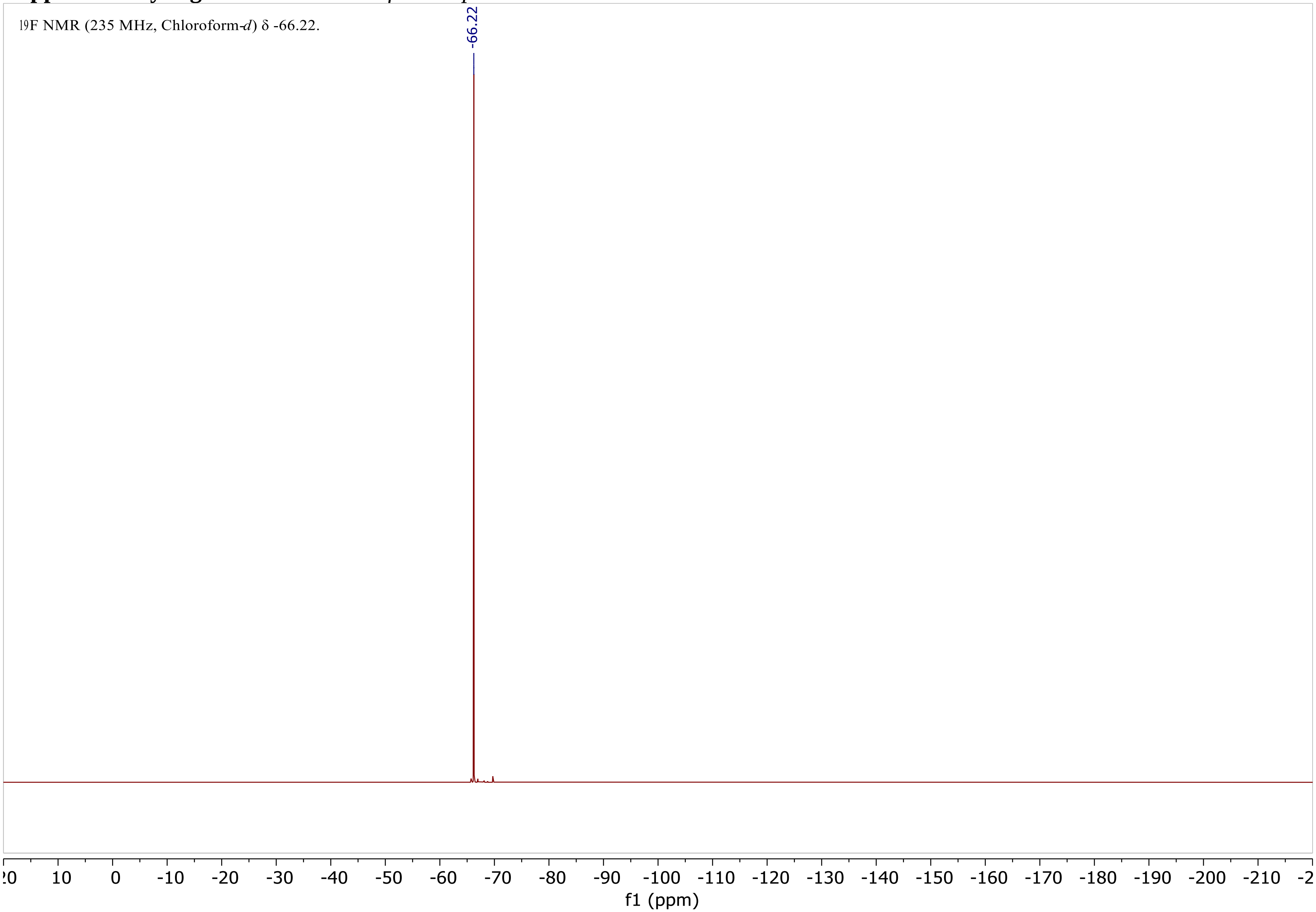

Supplementary Figure 241: *^{13}C NMR for compound* ***49***

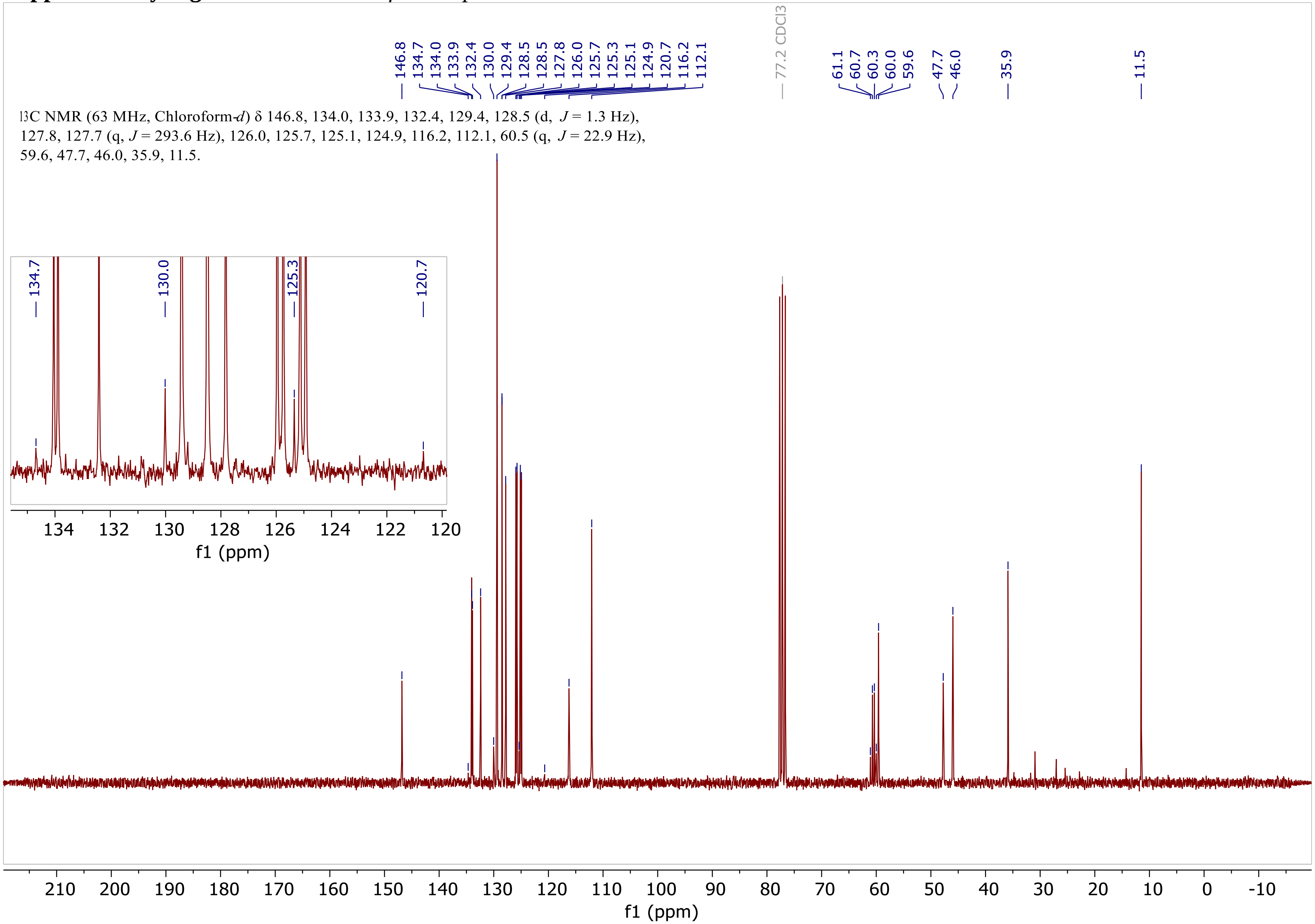

Supplementary Figure 242: *IR for compound **49***

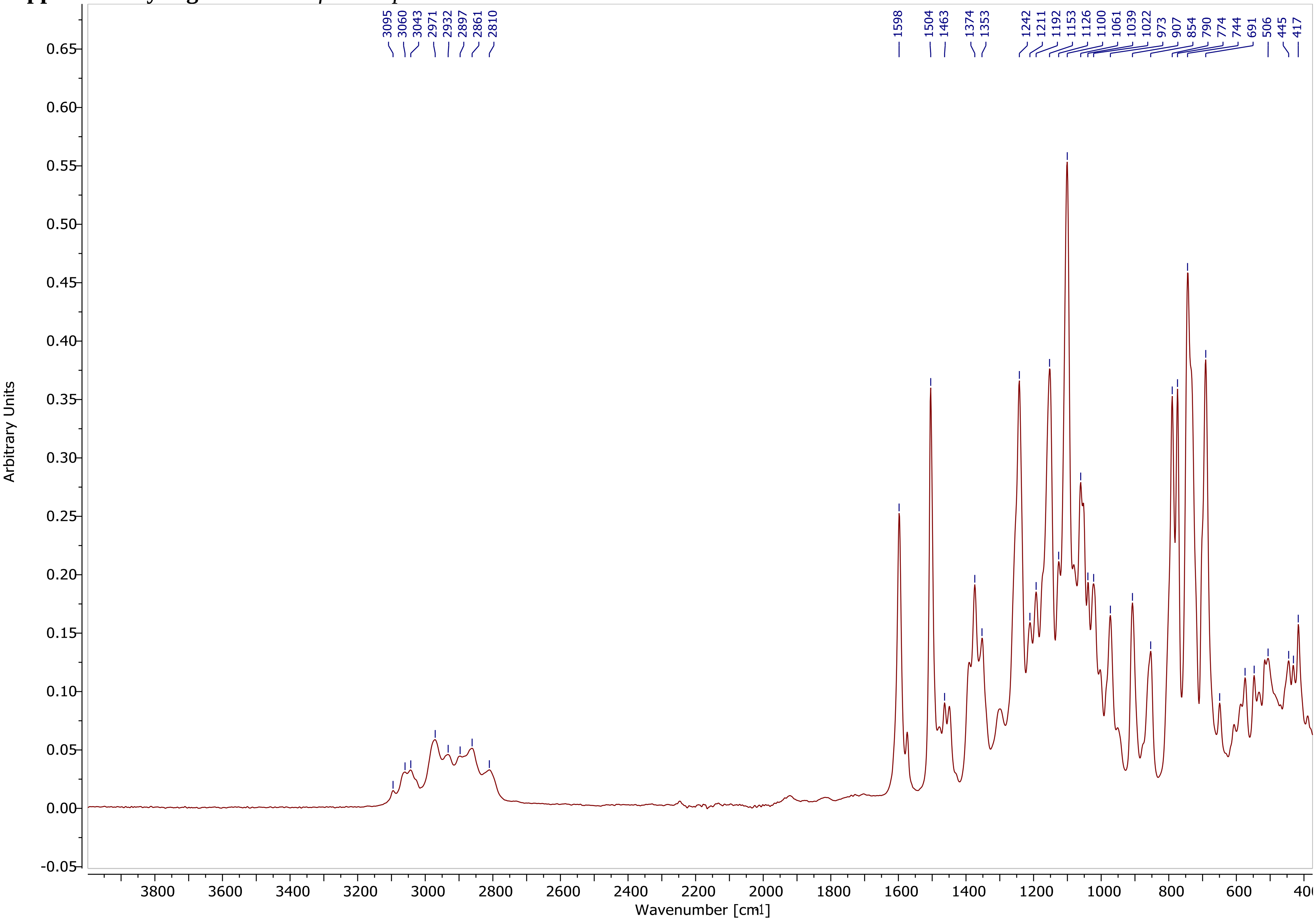

Supplementary Figure 243: *^{1}H-NMR for compound* ***50***

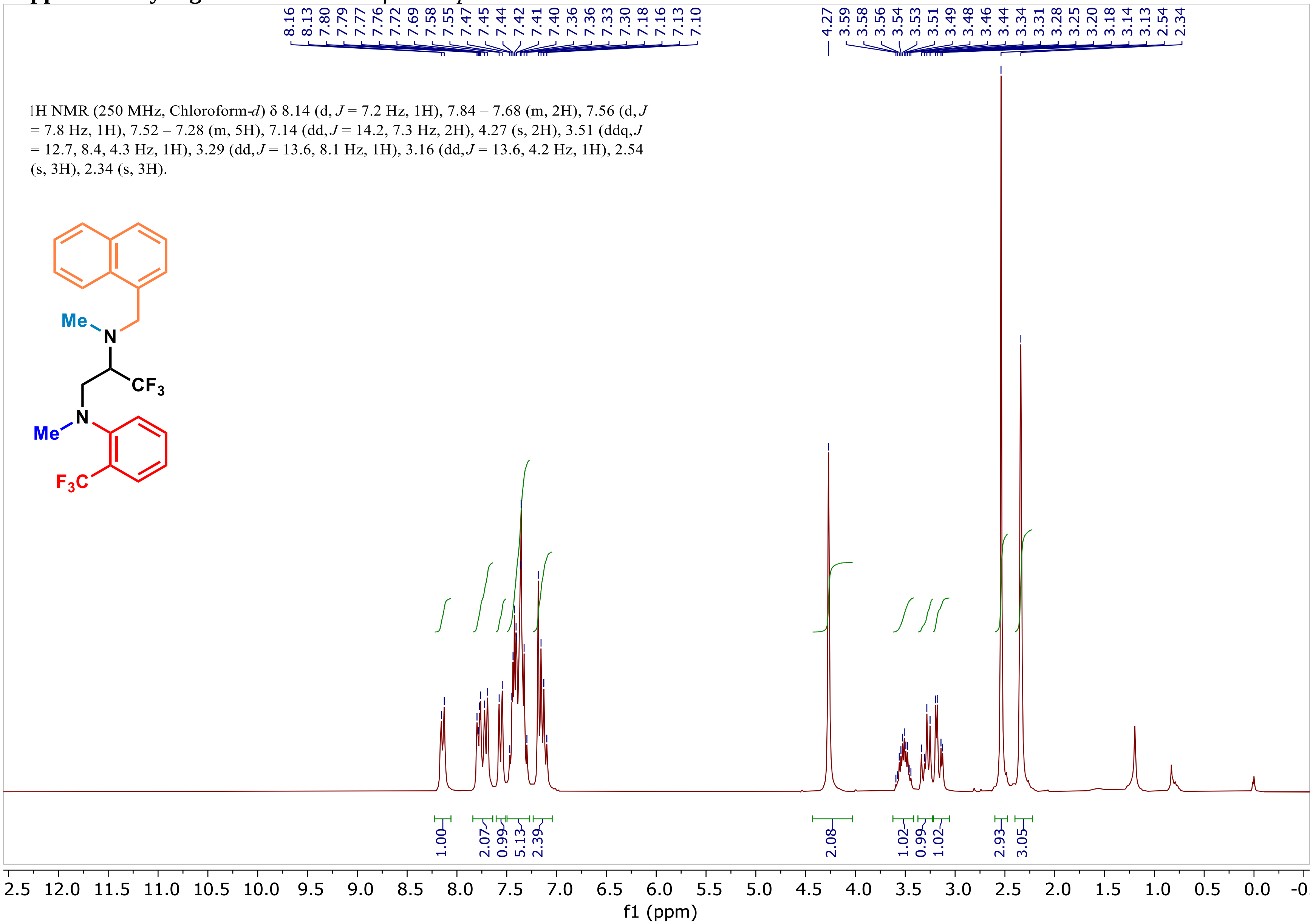

Supplementary Figure 244: *^{19}F NMR for compound* ***50***

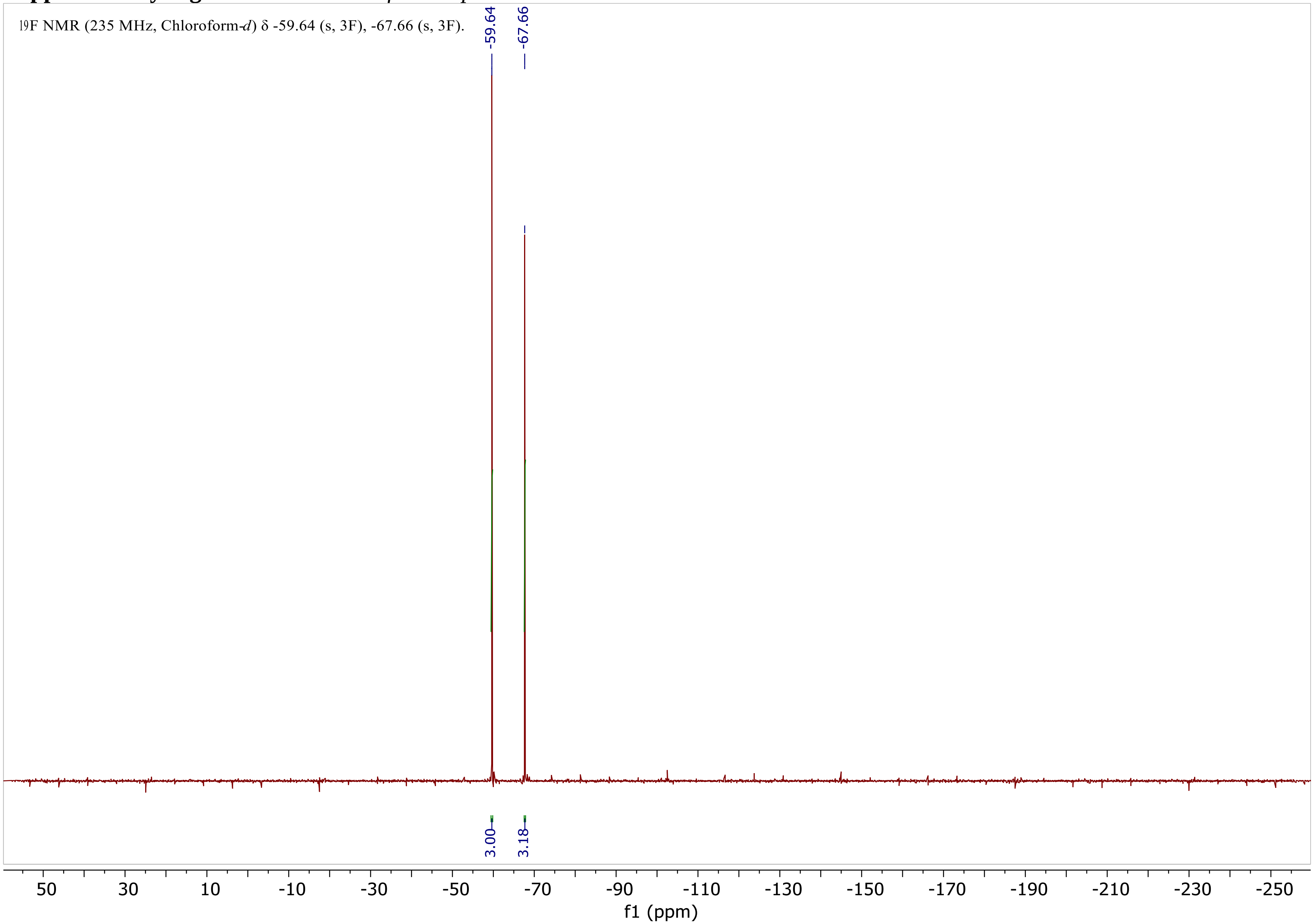

Supplementary Figure 245: ^{13}*C NMR for compound* ***50***

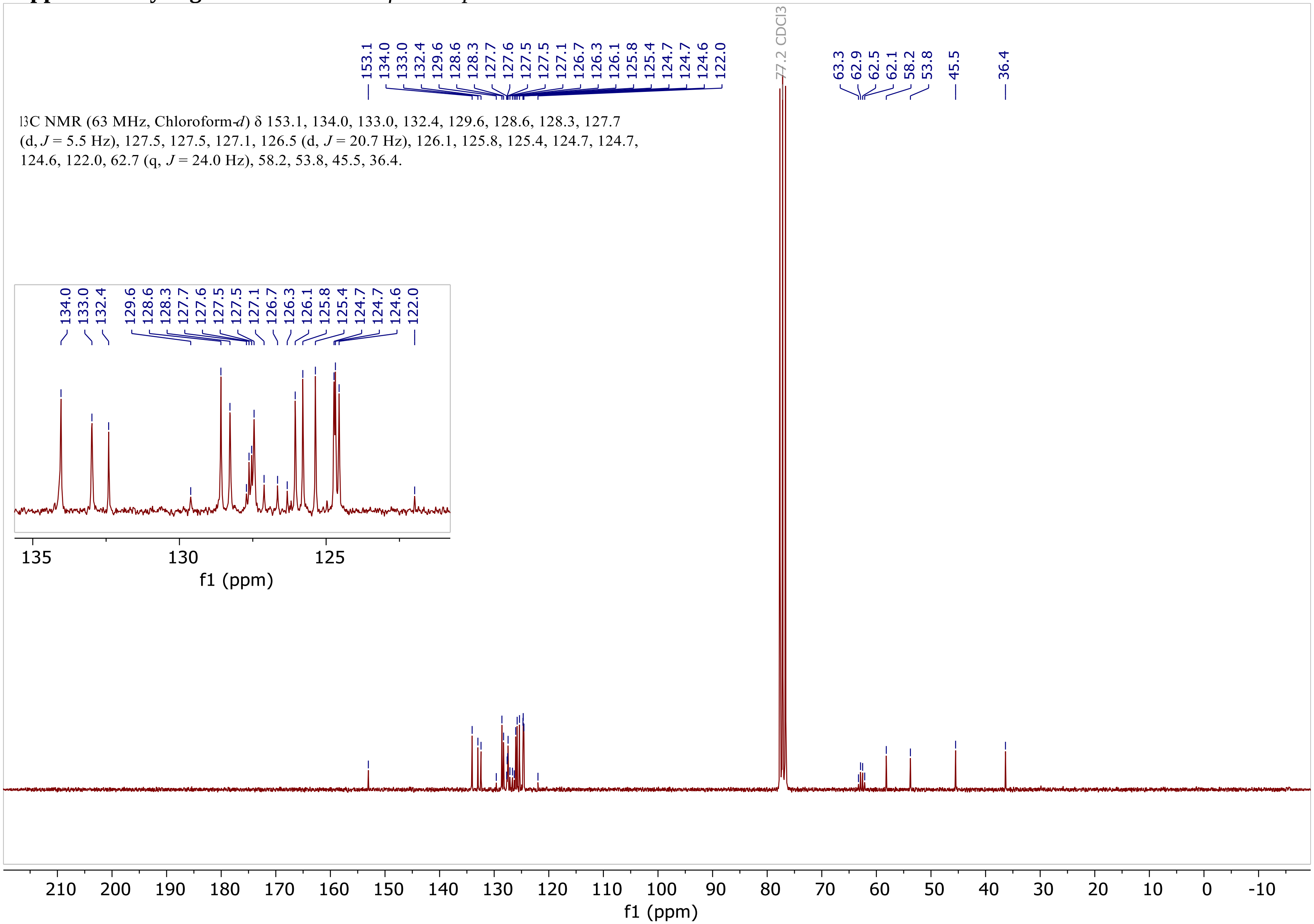

Supplementary Figure 246: *IR for compound **50***

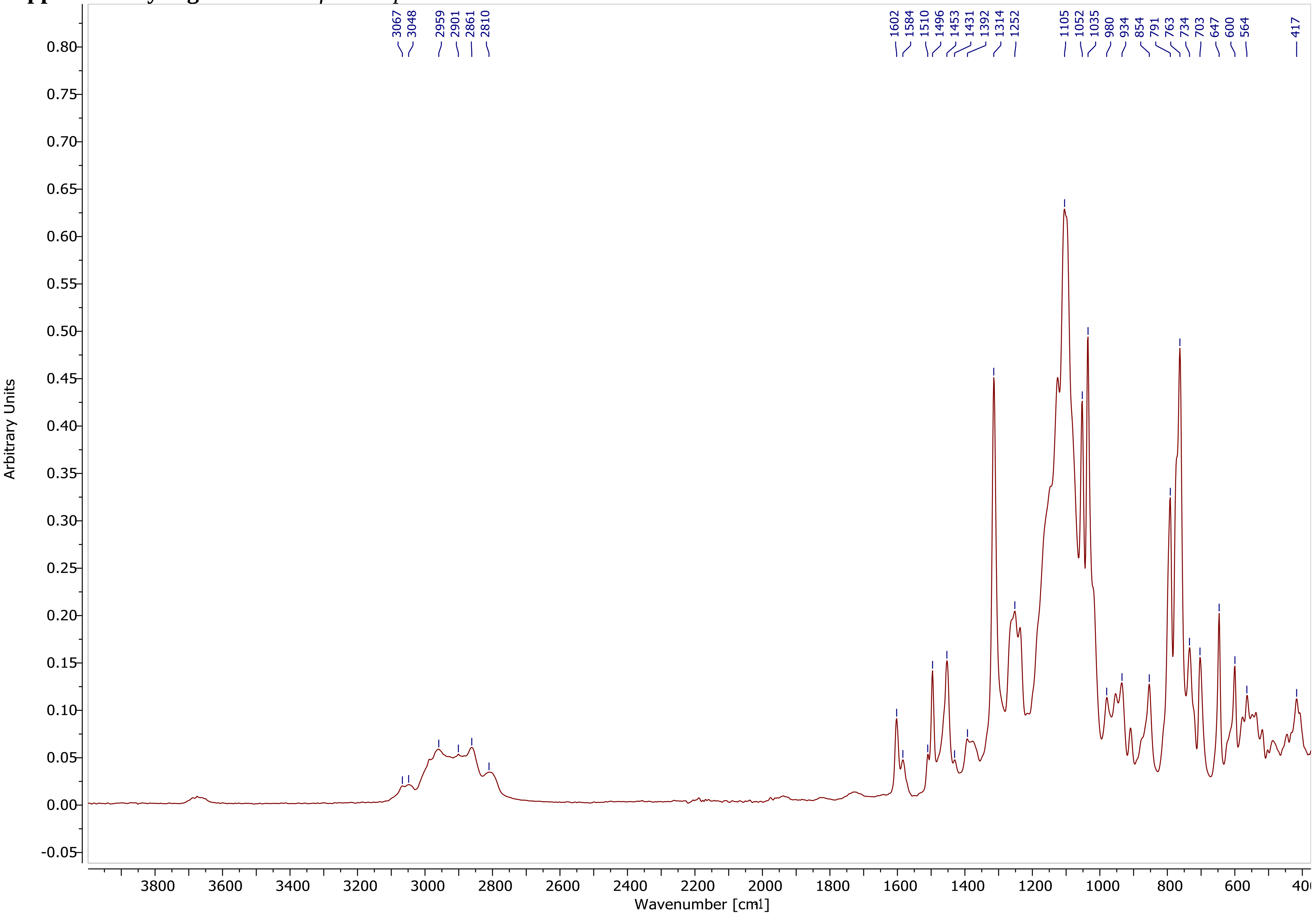

Supplementary Figure 247: *^{1}H-NMR for compound* ***51***

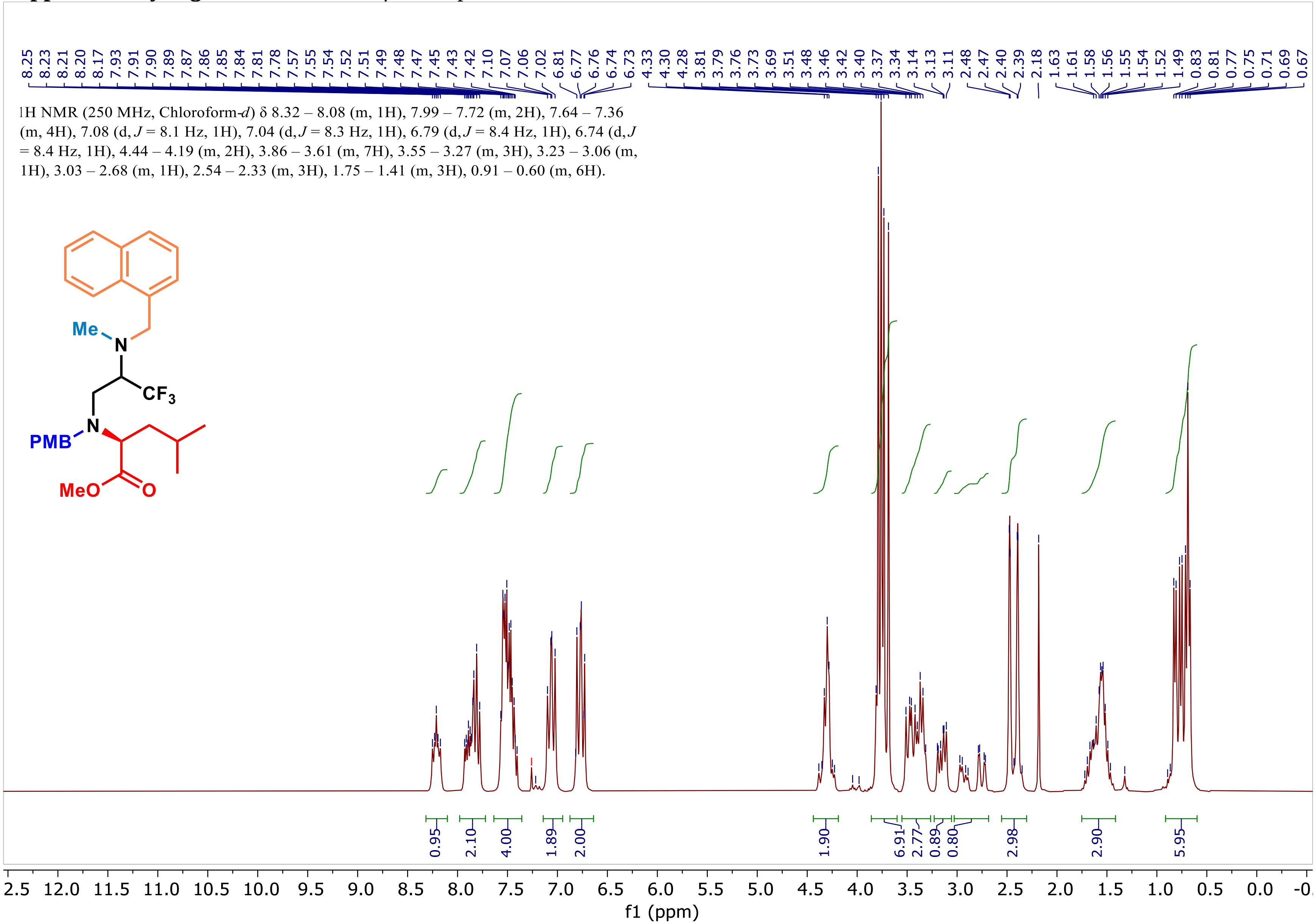

Supplementary Figure 248: *^{19}F NMR for compound **51***

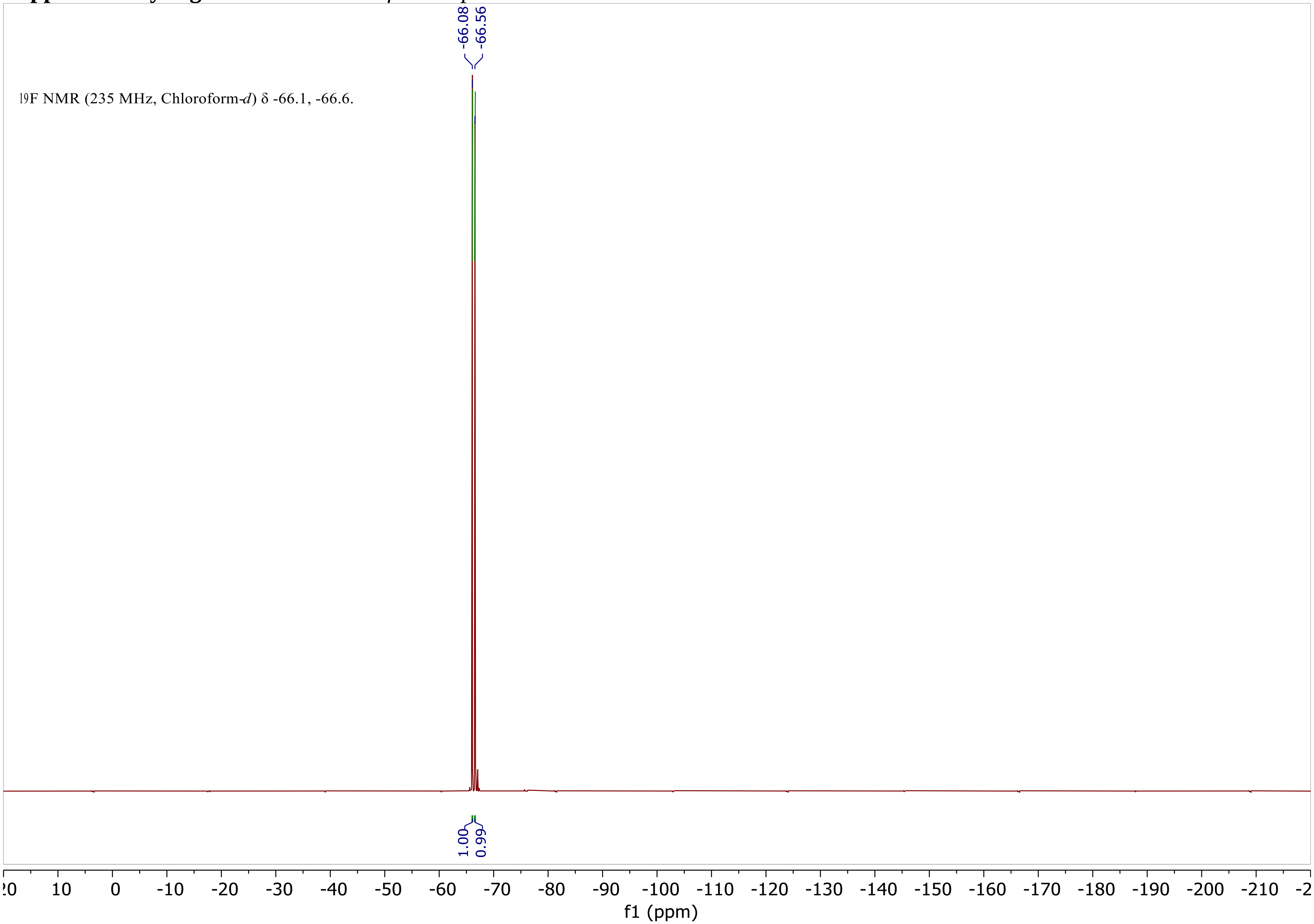

Supplementary Figure 249: *^{13}C NMR for compound **51***

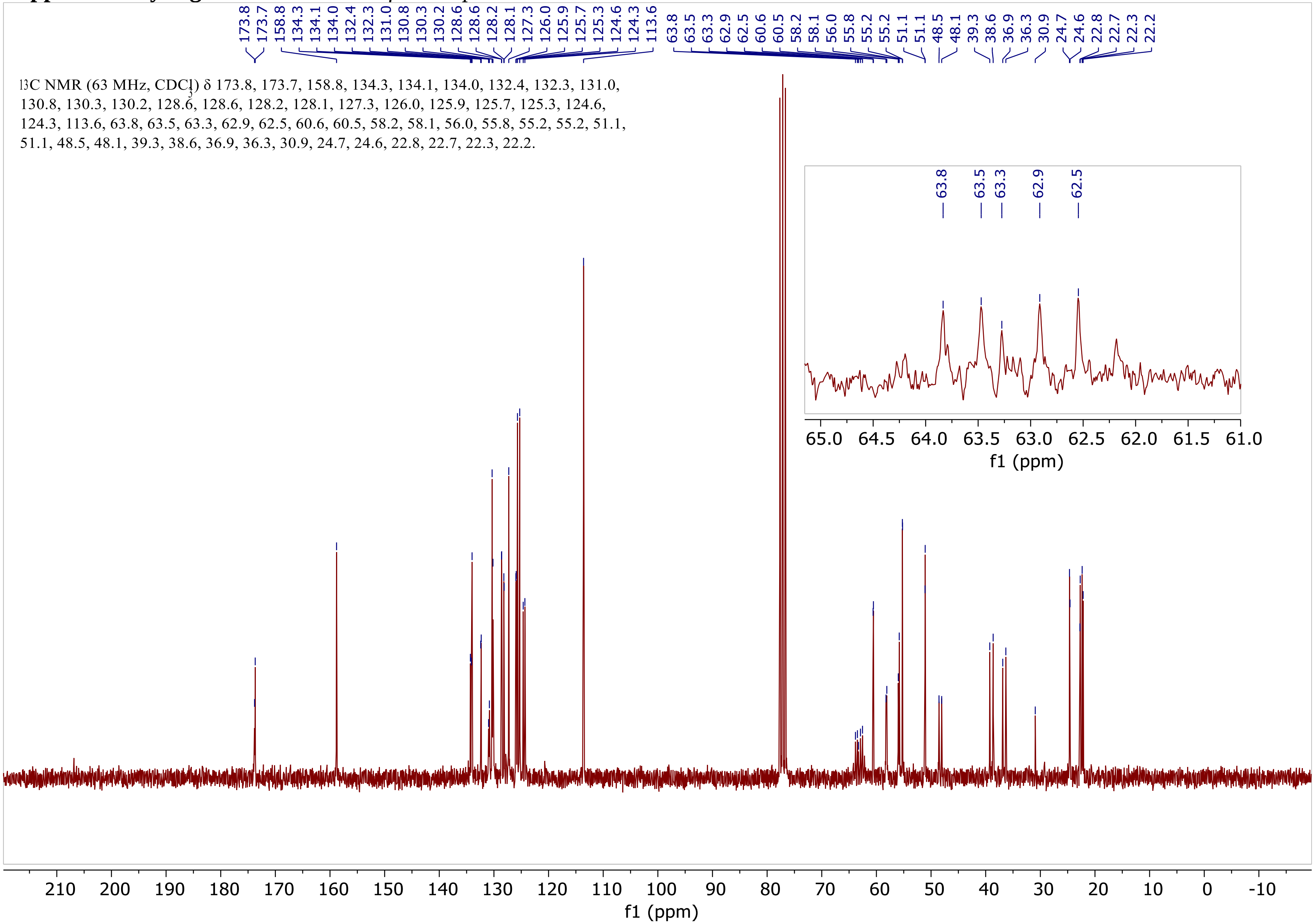

Supplementary Figure 250: *IR for compound* ***51***

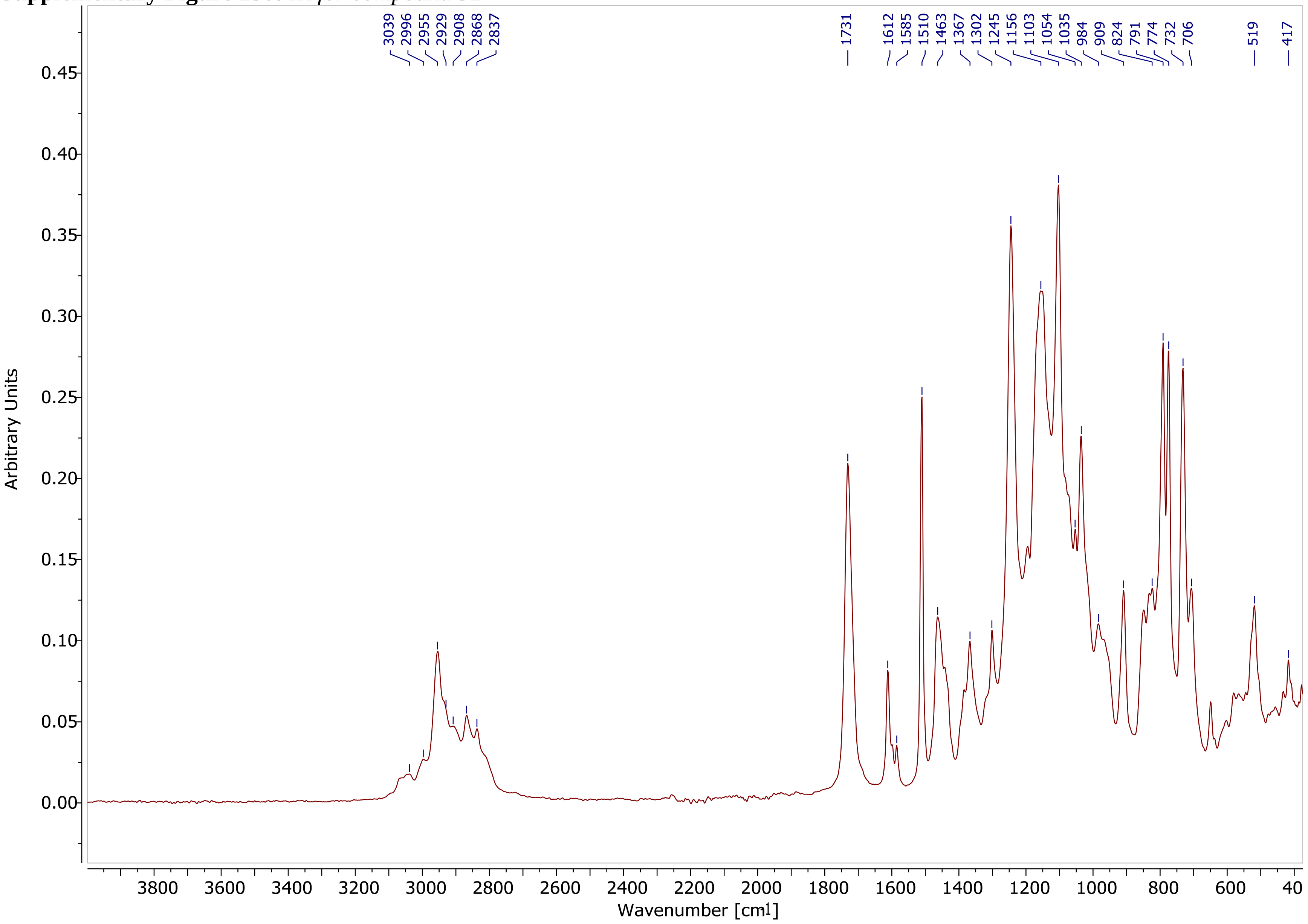

Supplementary Figure 251: *^{1}H-NMR for compound* ***52***

1H NMR (250 MHz, Chloroform-*d*) δ 7.75 – 7.59 (m, 2H), 7.44 (d, *J* = 8.2 Hz, 1H), 7.39 – 7.16 (m, 4H), 7.11 (d, *J* = 7.1 Hz, 1H), 7.03 (s, 1H), 4.24 – 3.98 (m, 4H), 3.80 (ddp, *J* = 12.9, 8.6, 4.6 Hz, 1H), 2.53 – 2.17 (m, 3H).

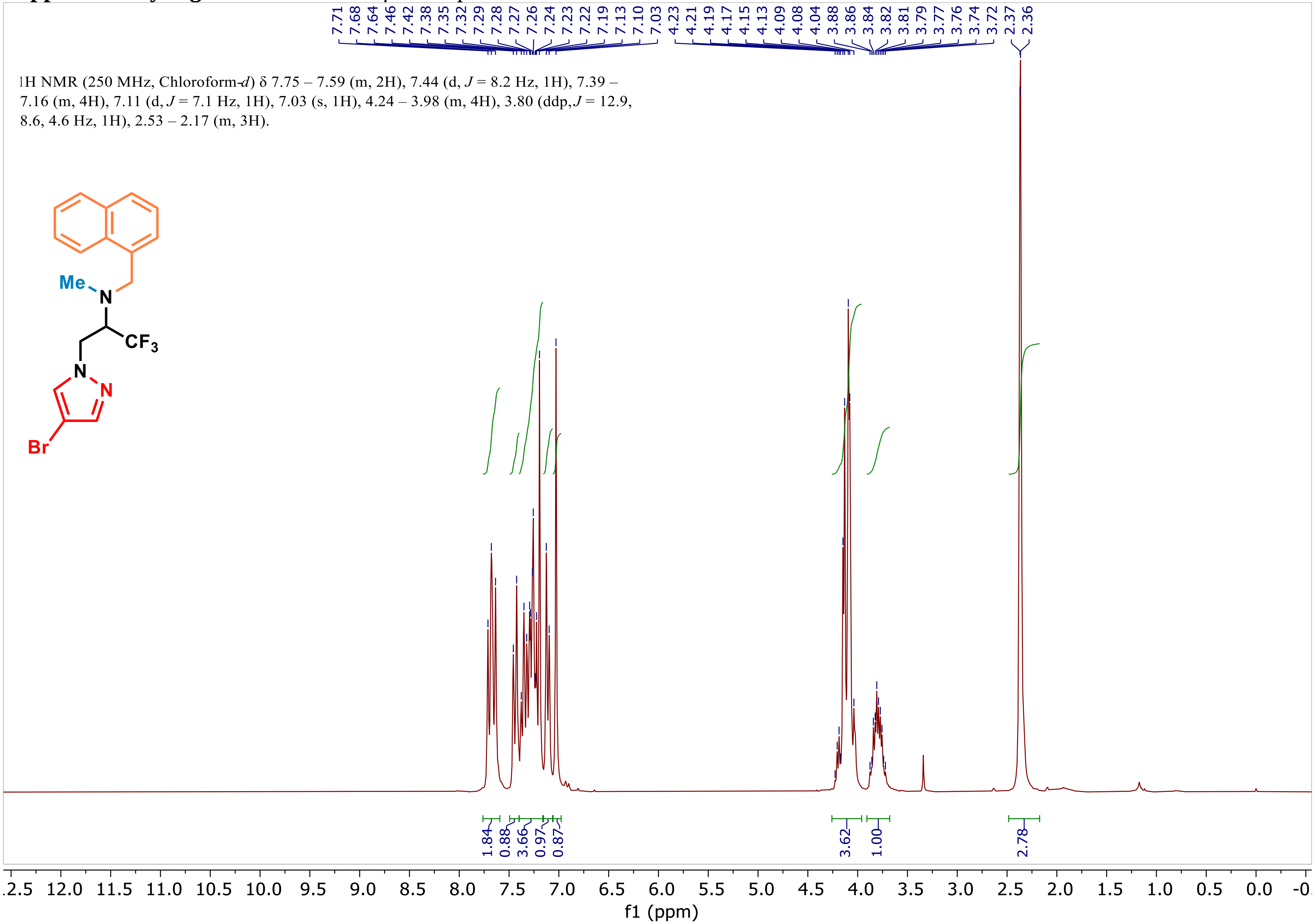

Supplementary Figure 252: *^{19}F NMR for compound* ***52***

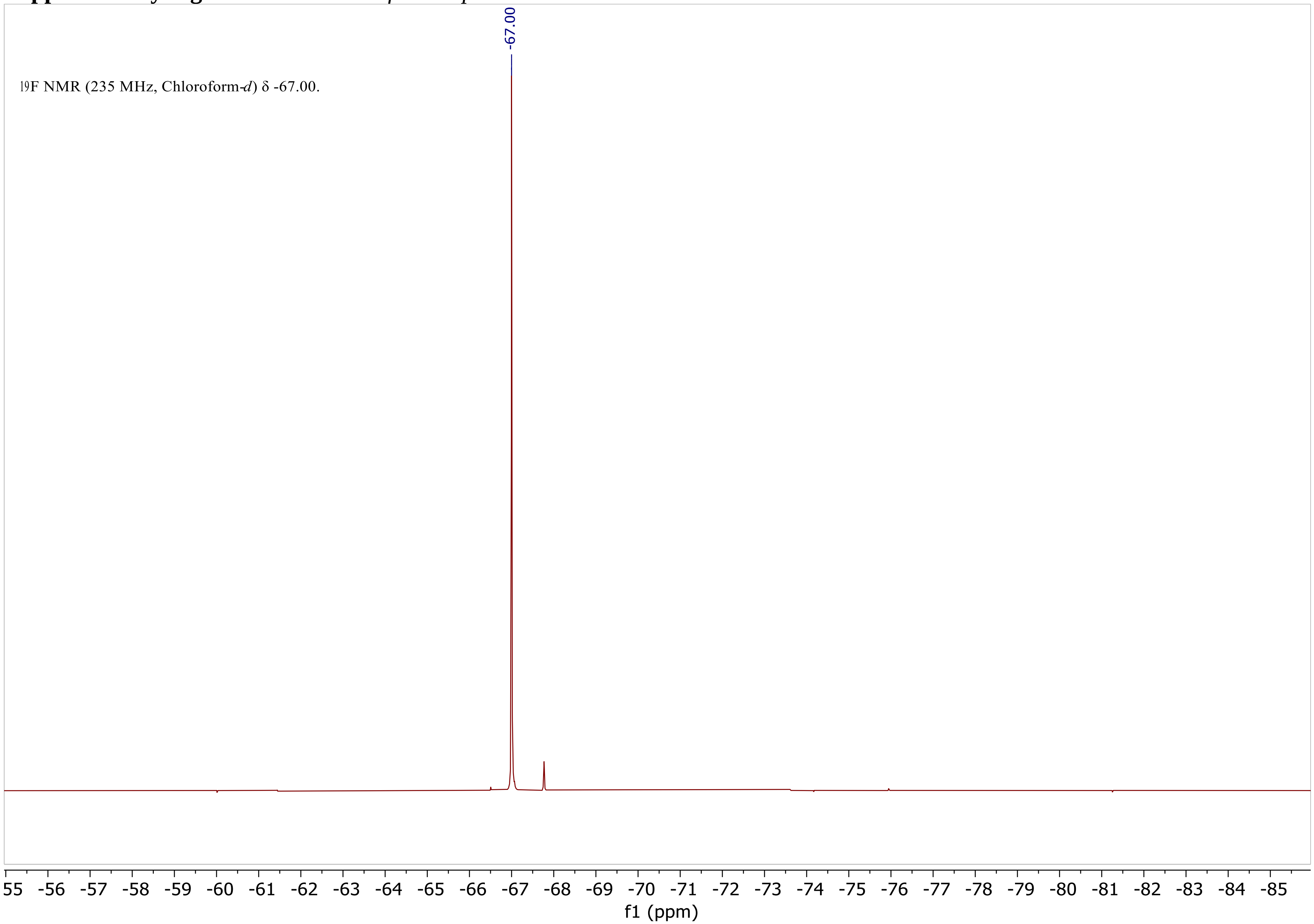

Supplementary Figure 253: *^{13}C NMR for compound* ***52***

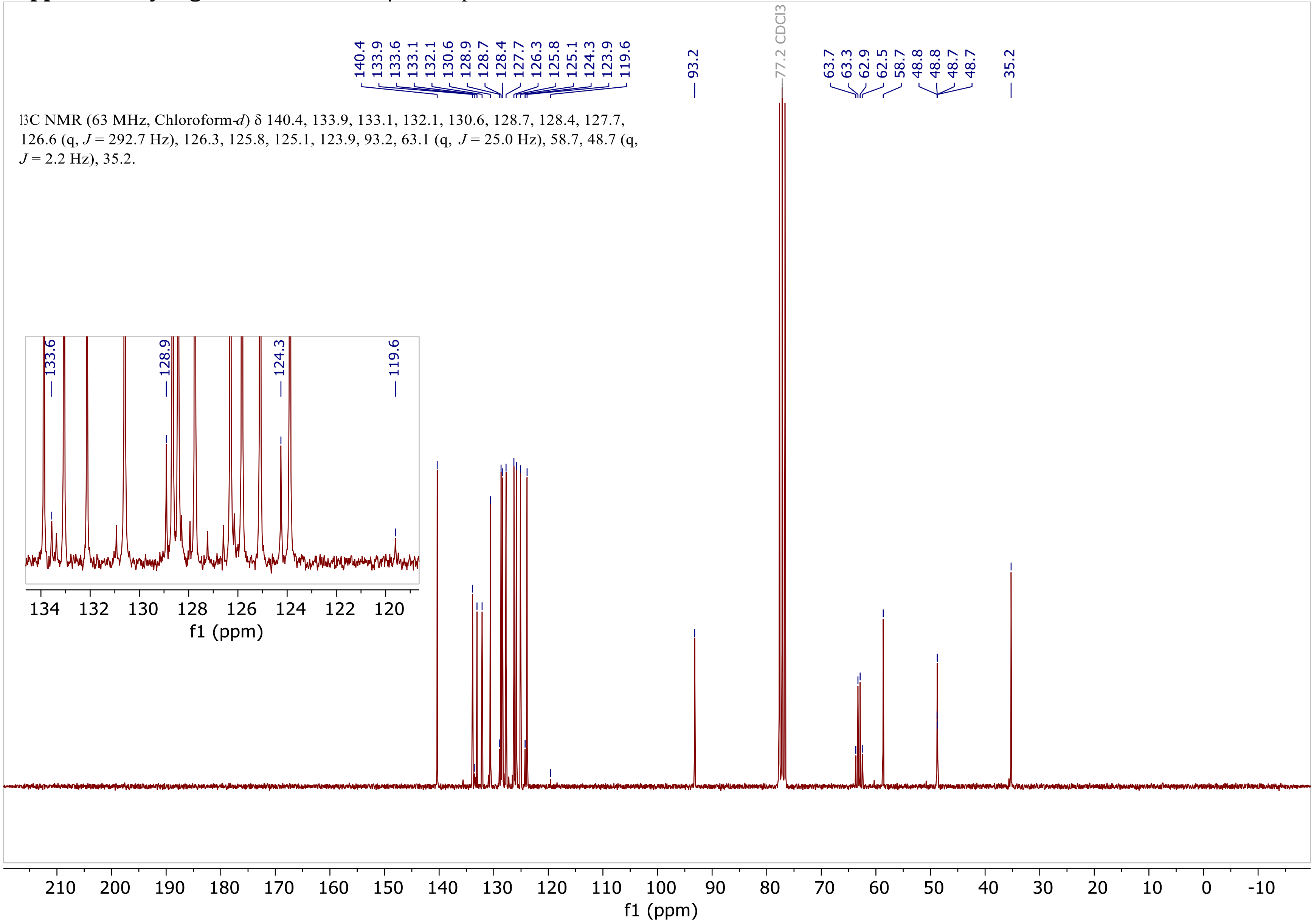

^{13}C NMR (63 MHz, Chloroform-*d*) δ 140.4, 133.9, 133.1, 132.1, 130.6, 128.7, 128.4, 127.7, 126.6 (q, *J* = 292.7 Hz), 126.3, 125.8, 125.1, 123.9, 93.2, 63.1 (q, *J* = 25.0 Hz), 58.7, 48.7 (q, *J* = 2.2 Hz), 35.2.

Supplementary Figure 254: *IR for compound **52***

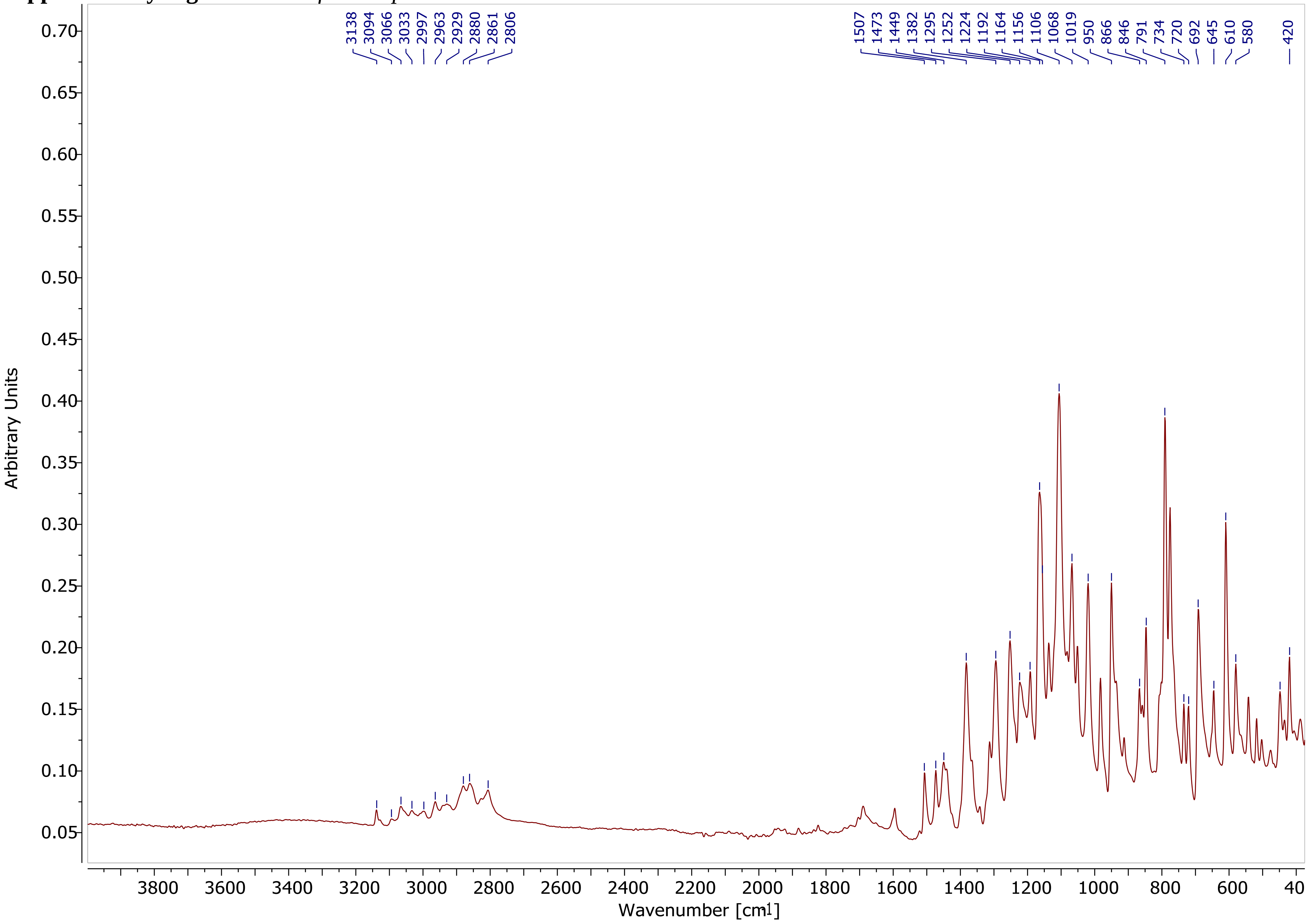

Supplementary Figure 255: *^{1}H-NMR for compound* ***53***

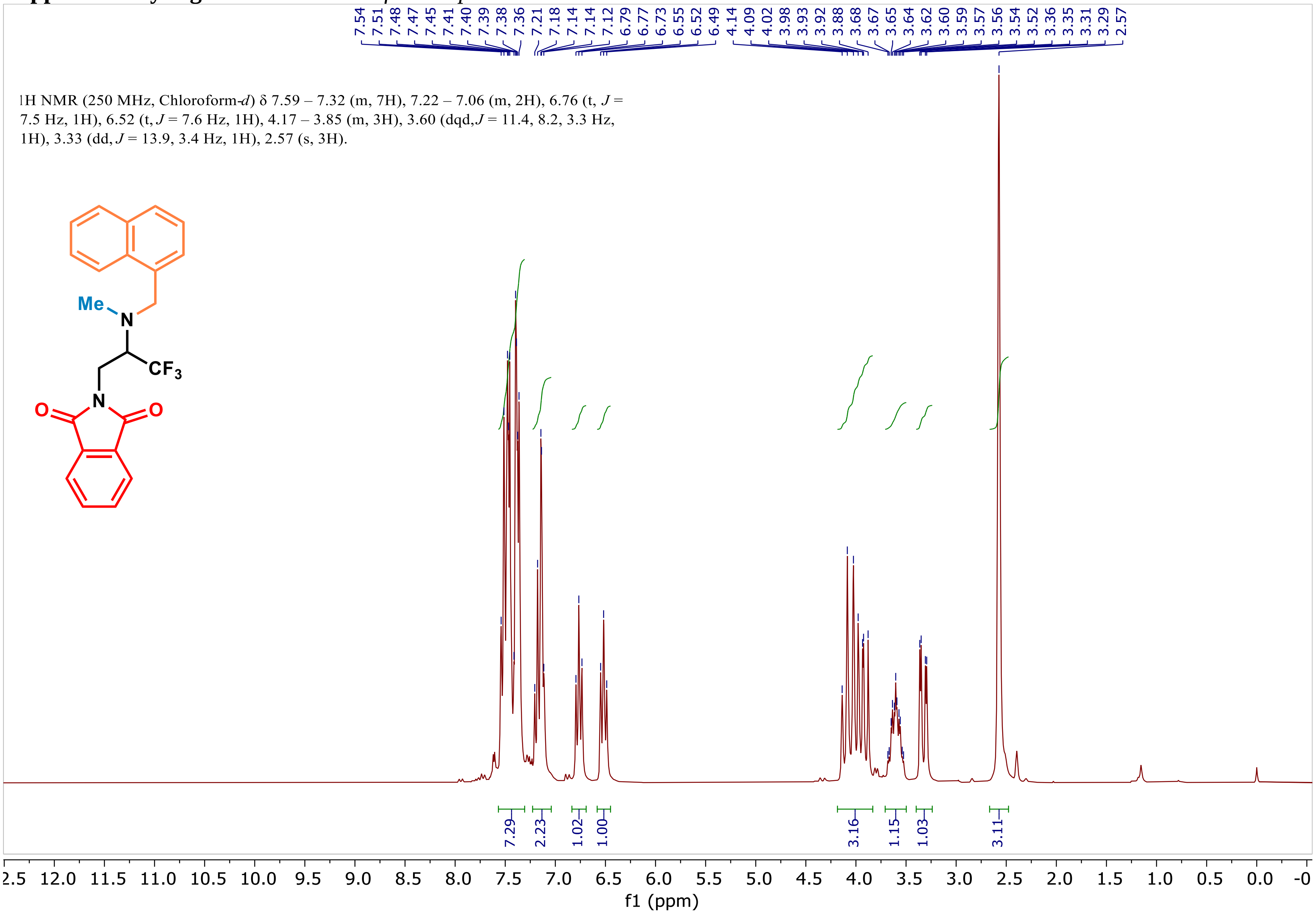

Supplementary Figure 256: *^{19}F NMR for compound* ***53***

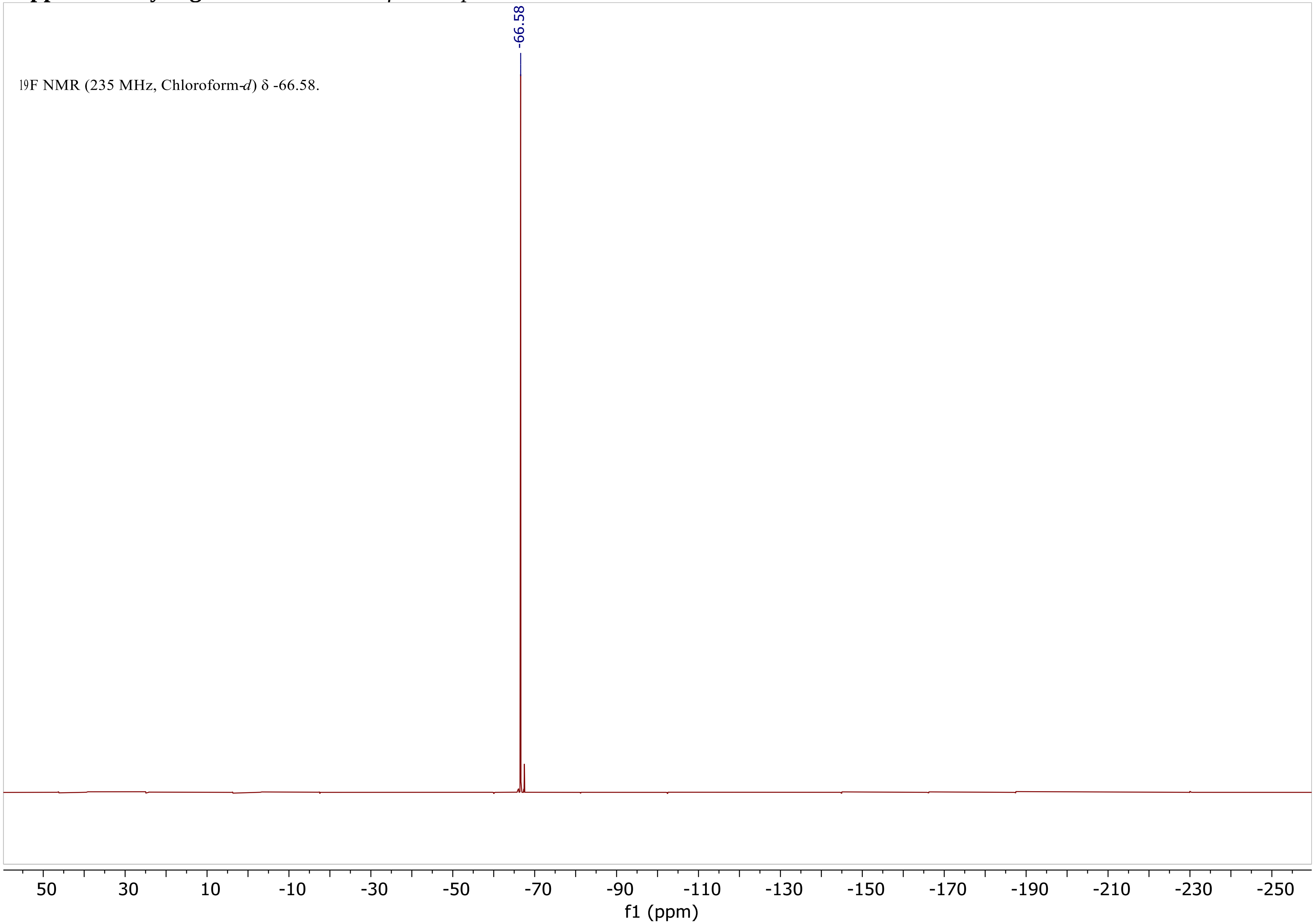

Supplementary Figure 257: ^{13}*C NMR for compound* ***53***

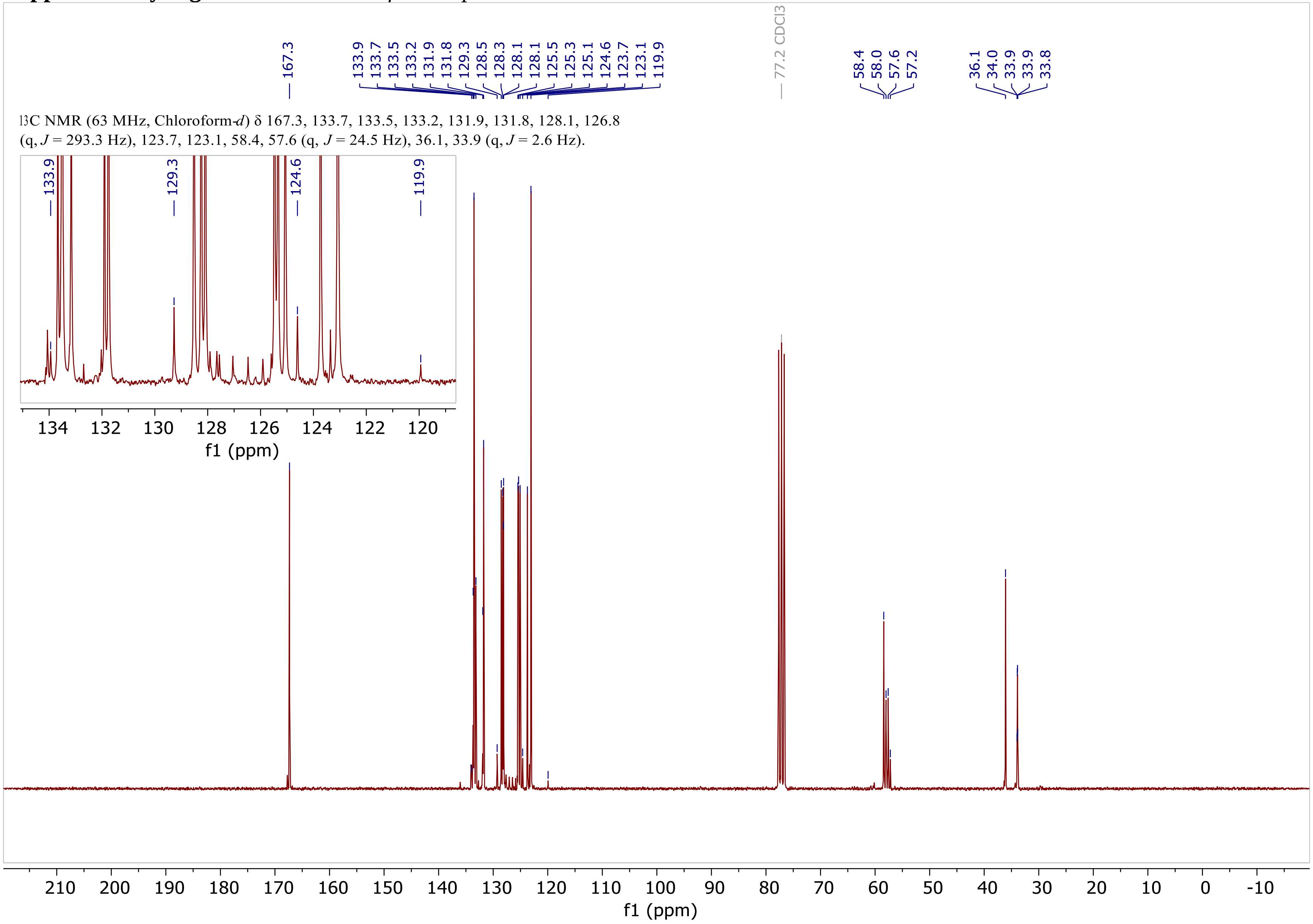

Supplementary Figure 258: *IR for compound* ***53***

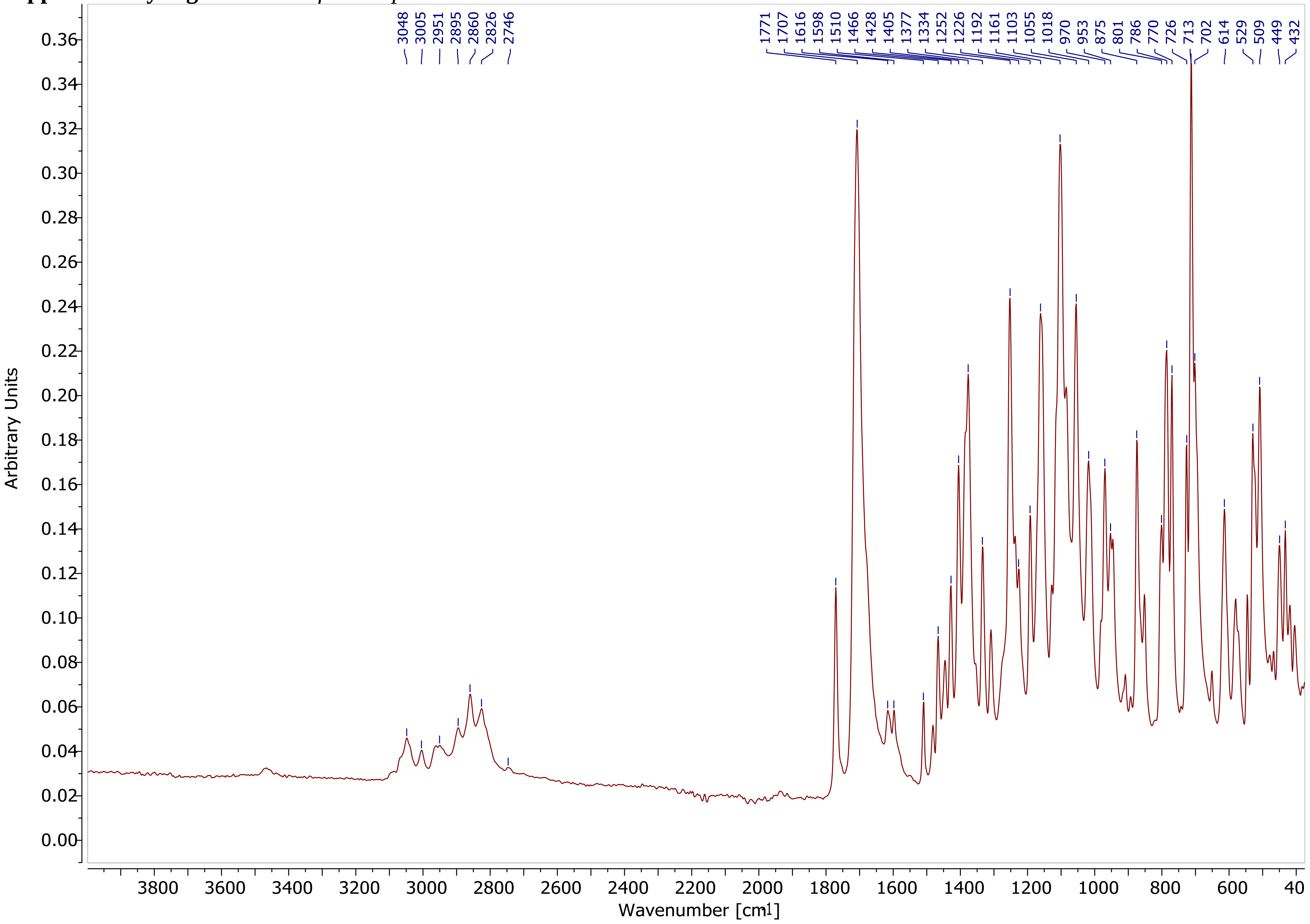

Supplementary Figure 259: *^{1}H-NMR for compound* ***54***

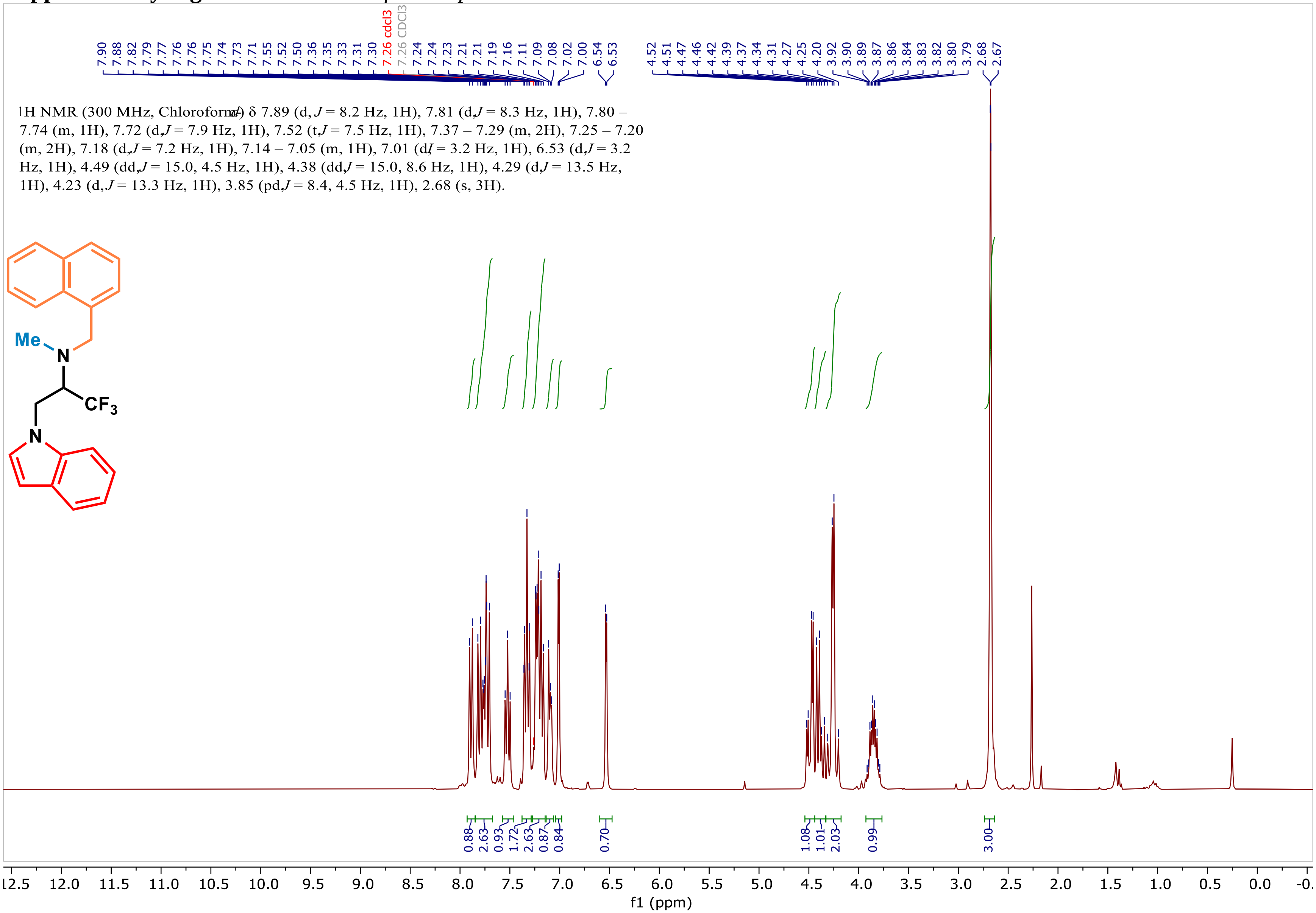

Supplementary Figure 260: *COSY NMR for compound **54***

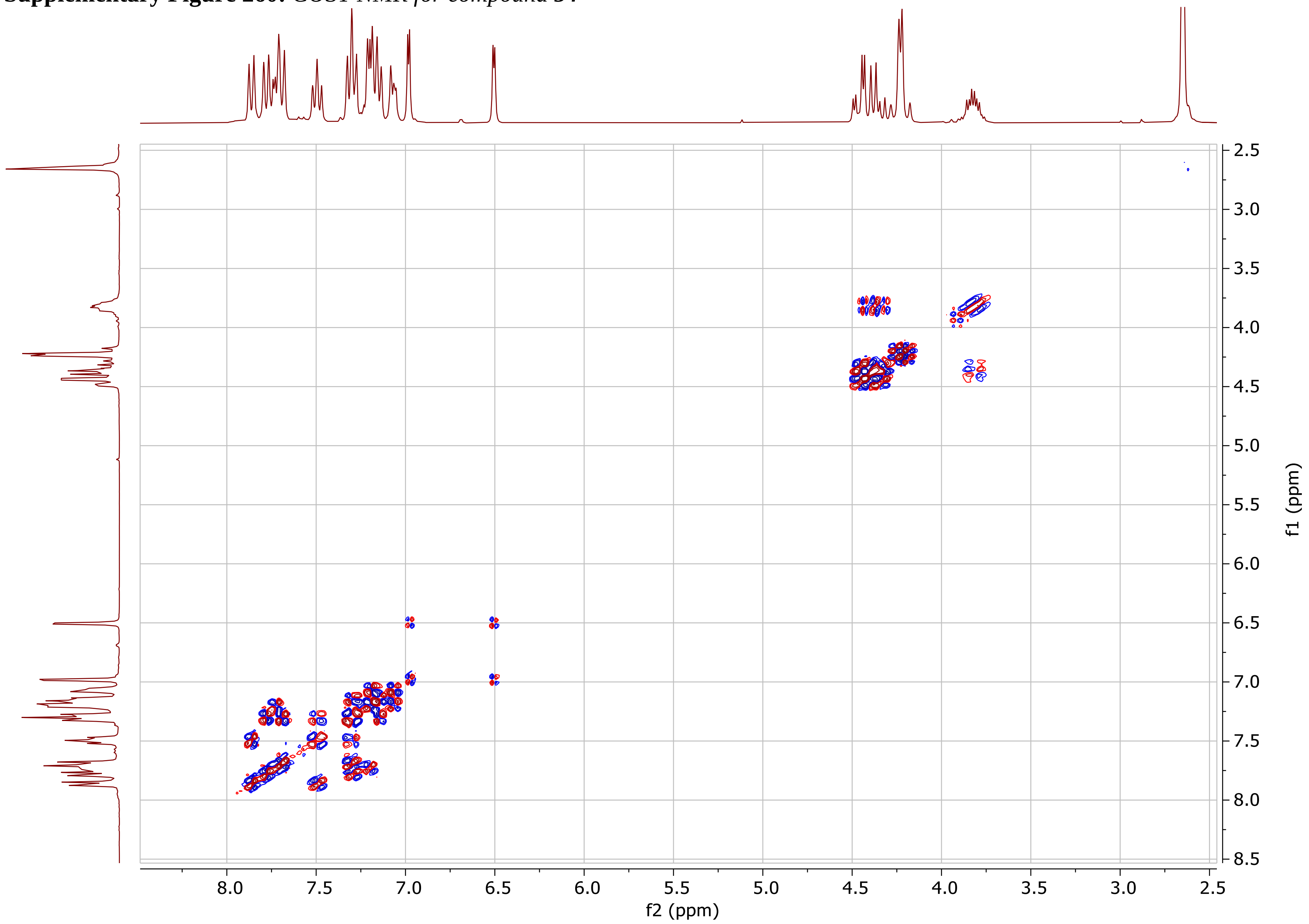

Supplementary Figure 261: *^{19}F NMR for compound* ***54***

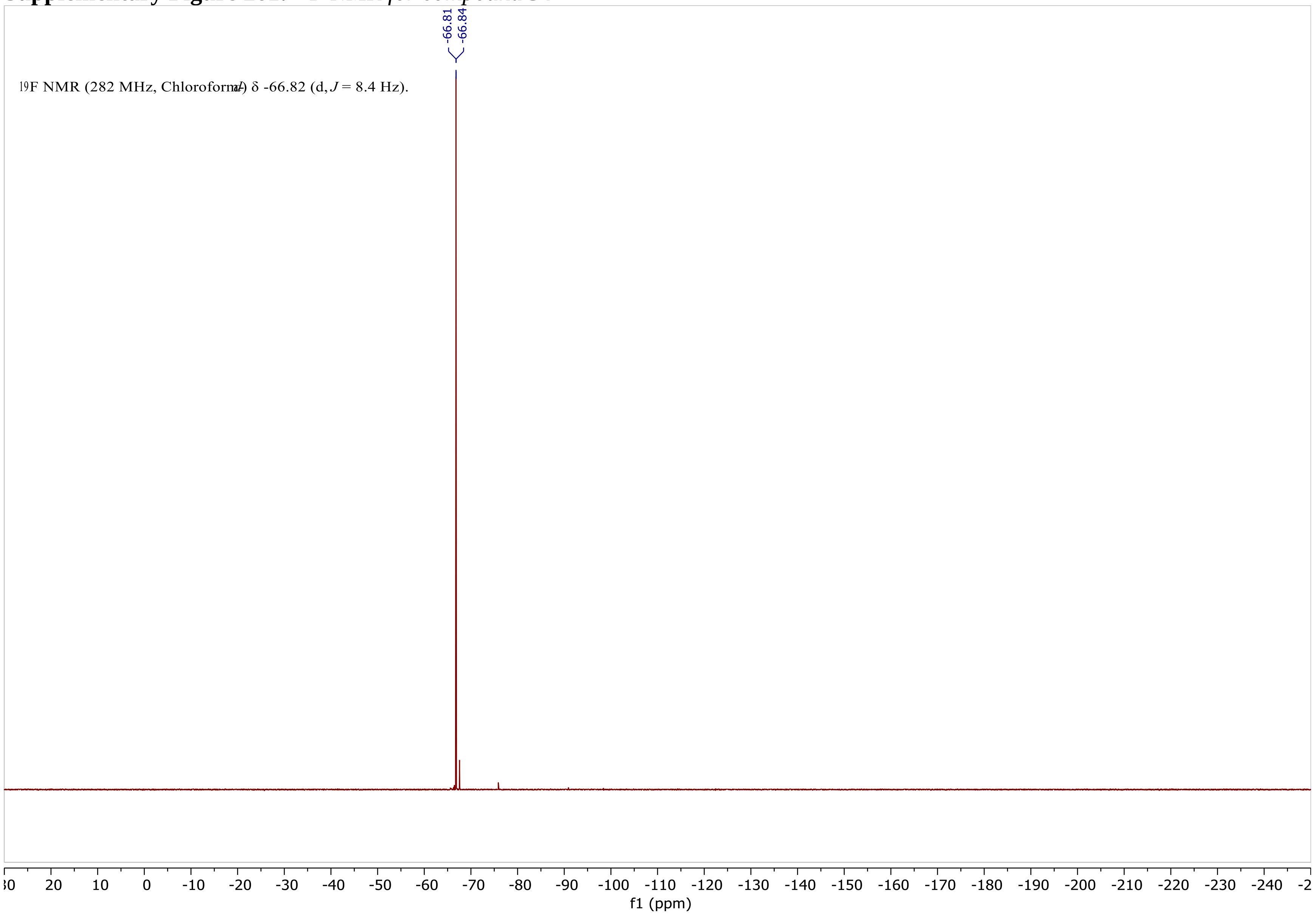

Supplementary Figure 262: *^{13}C NMR for compound* ***54***

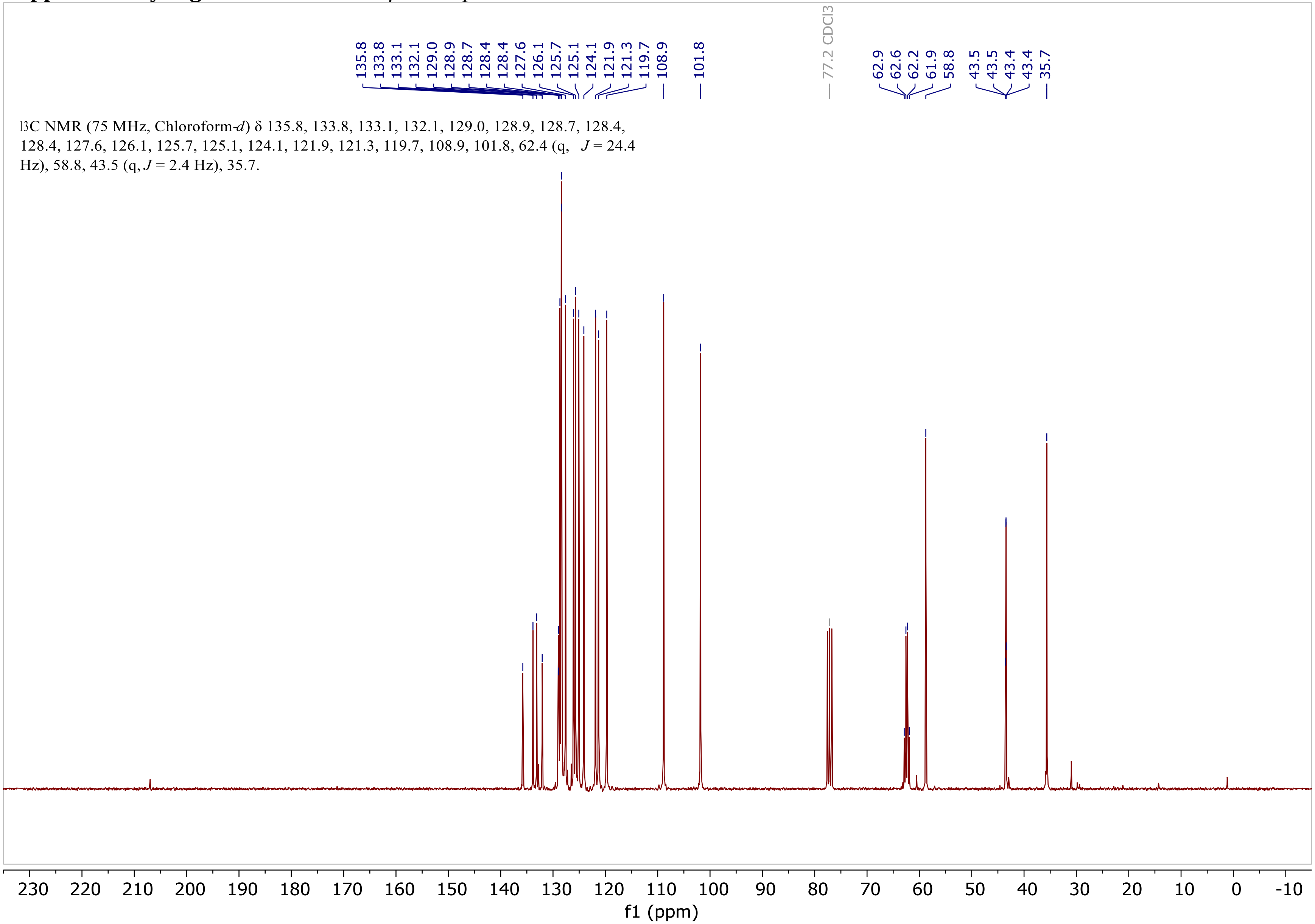

Supplementary Figure 263: *HSQC NMR for compound* ***54***

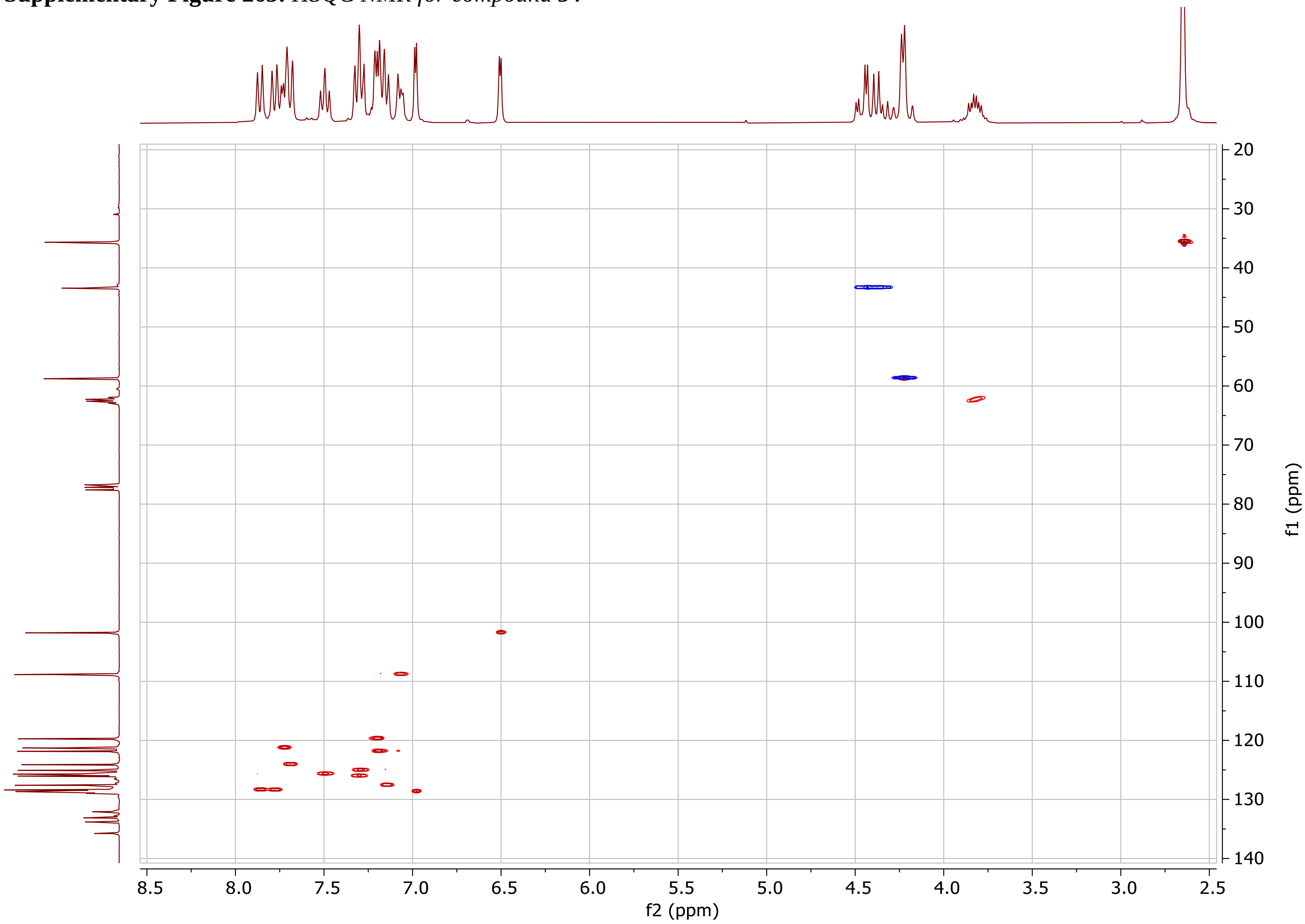

Supplementary Figure 264: *HMBC NMR for compound **54***

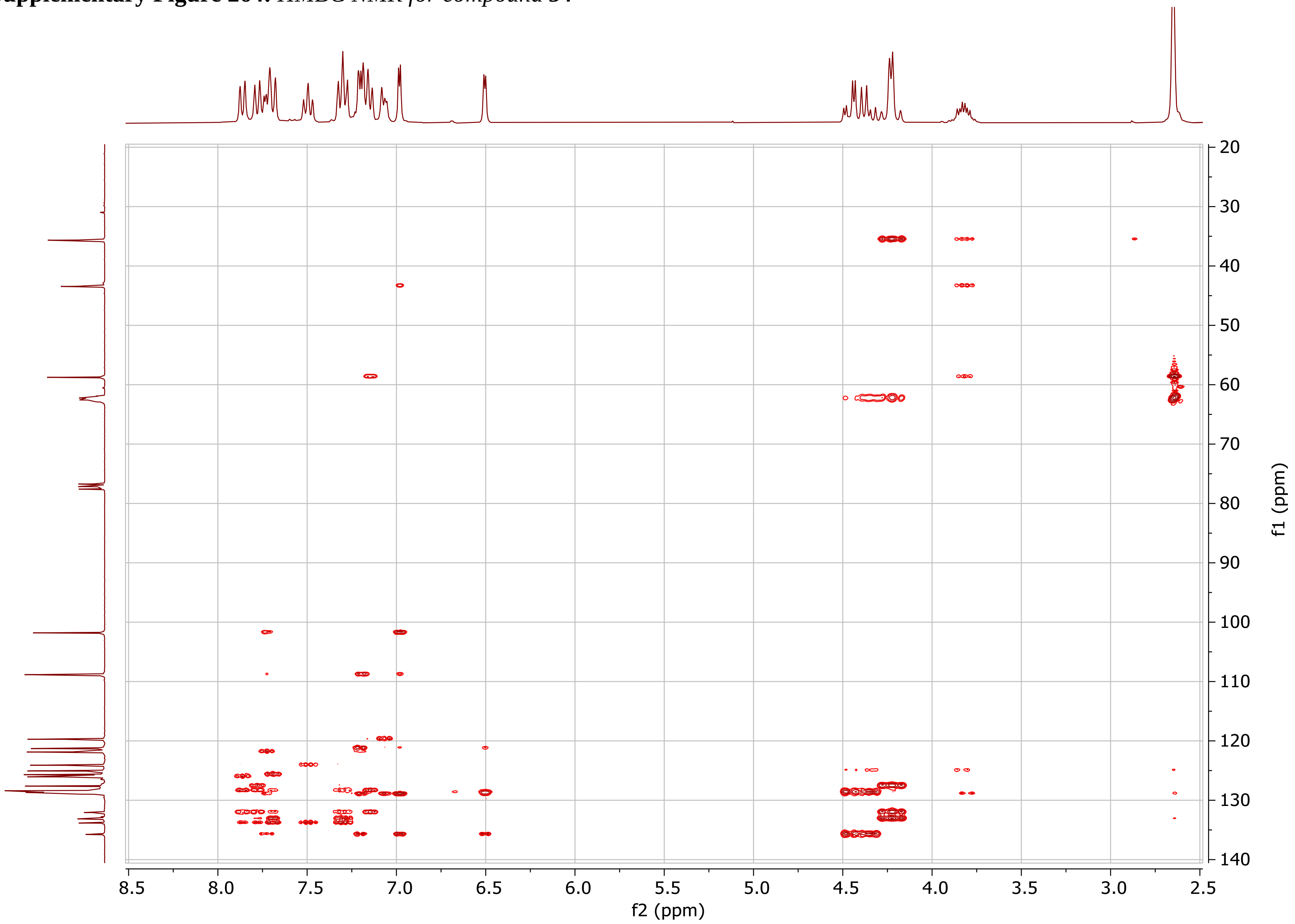

Supplementary Figure 265: *IR for compound **54***

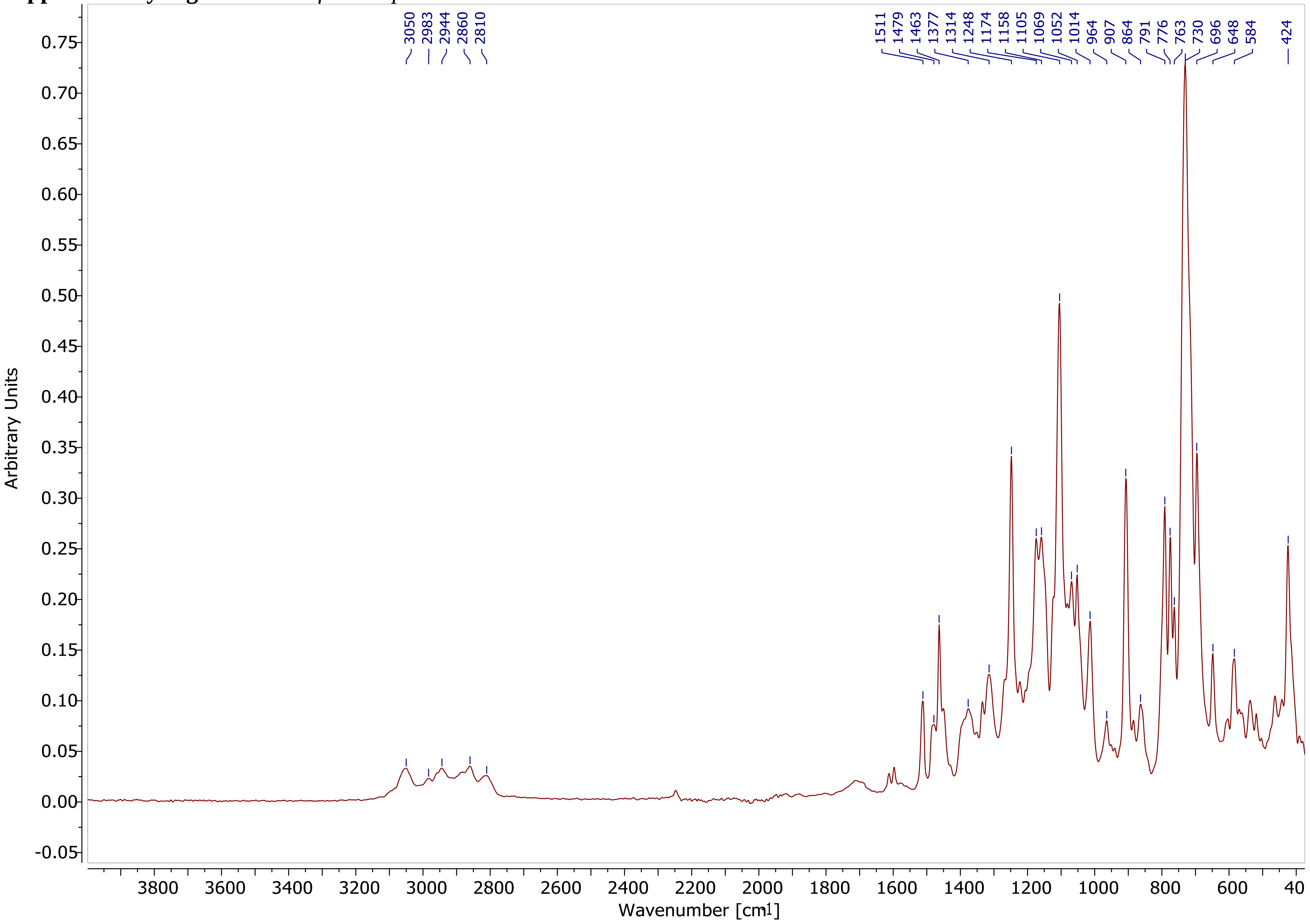

Supplementary Figure 266: *^{1}H-NMR for compound* ***55***

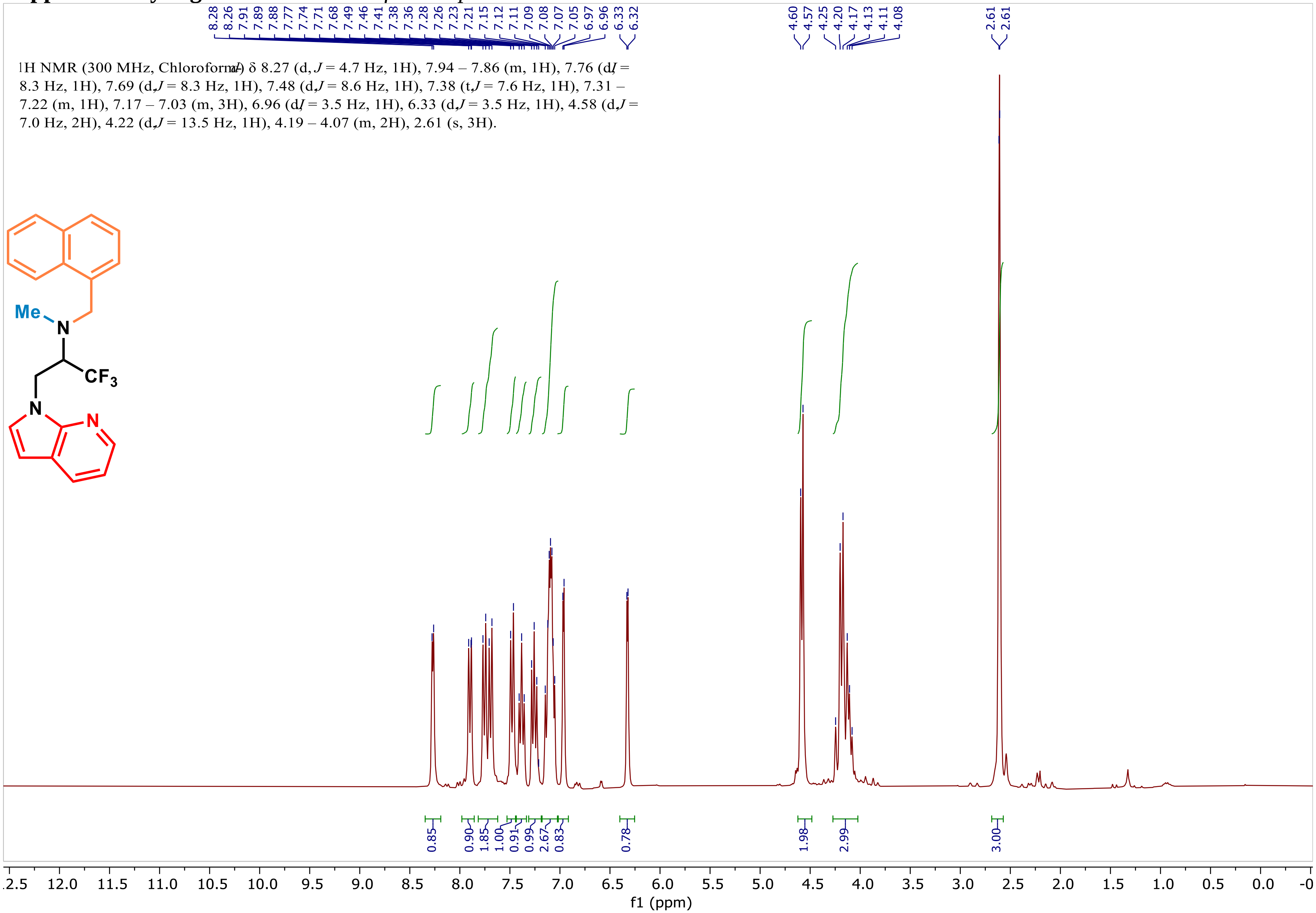

Supplementary Figure 267: *^{19}F NMR for compound* ***55***

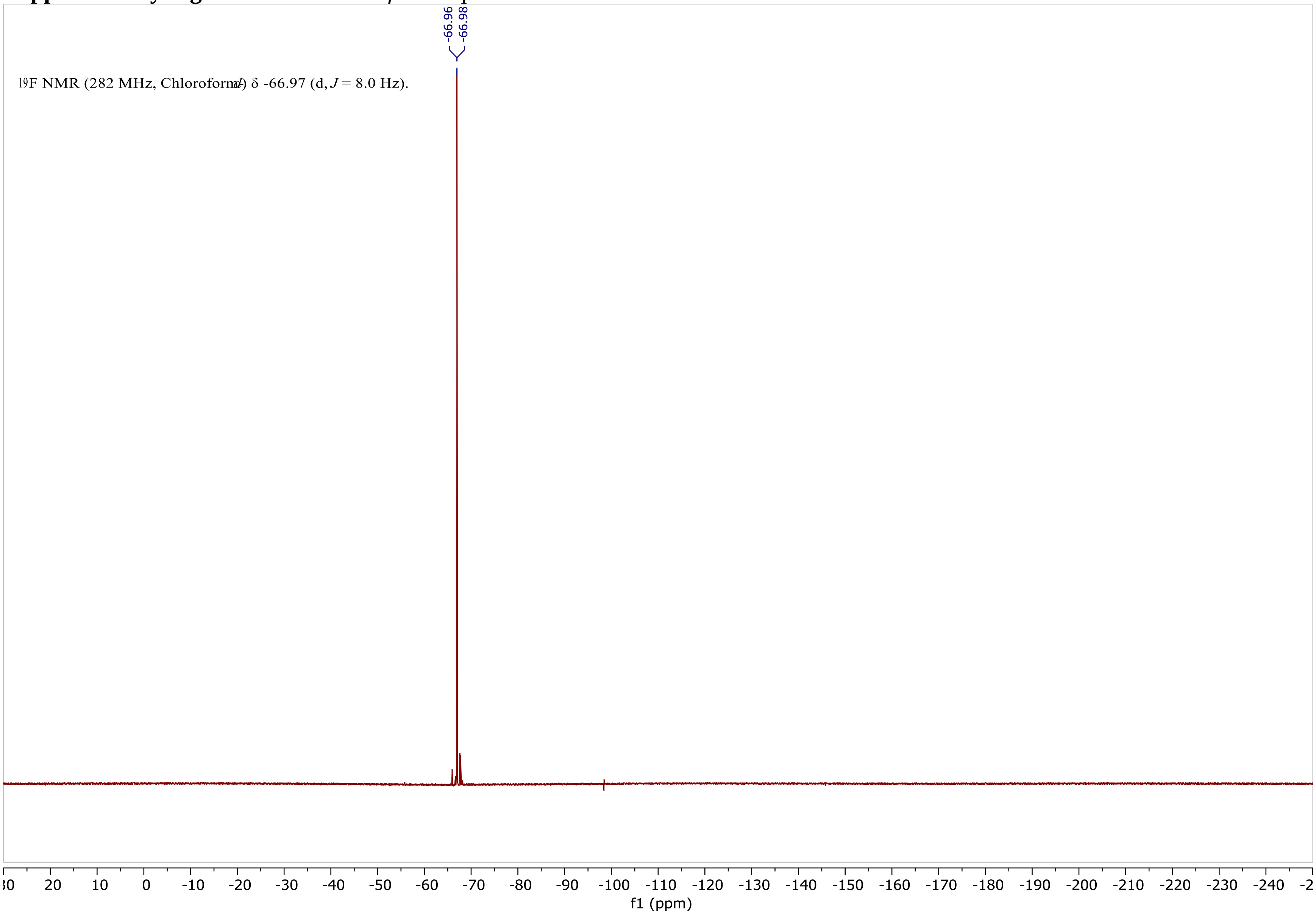

Supplementary Figure 268: *^{13}C NMR for compound* ***55***

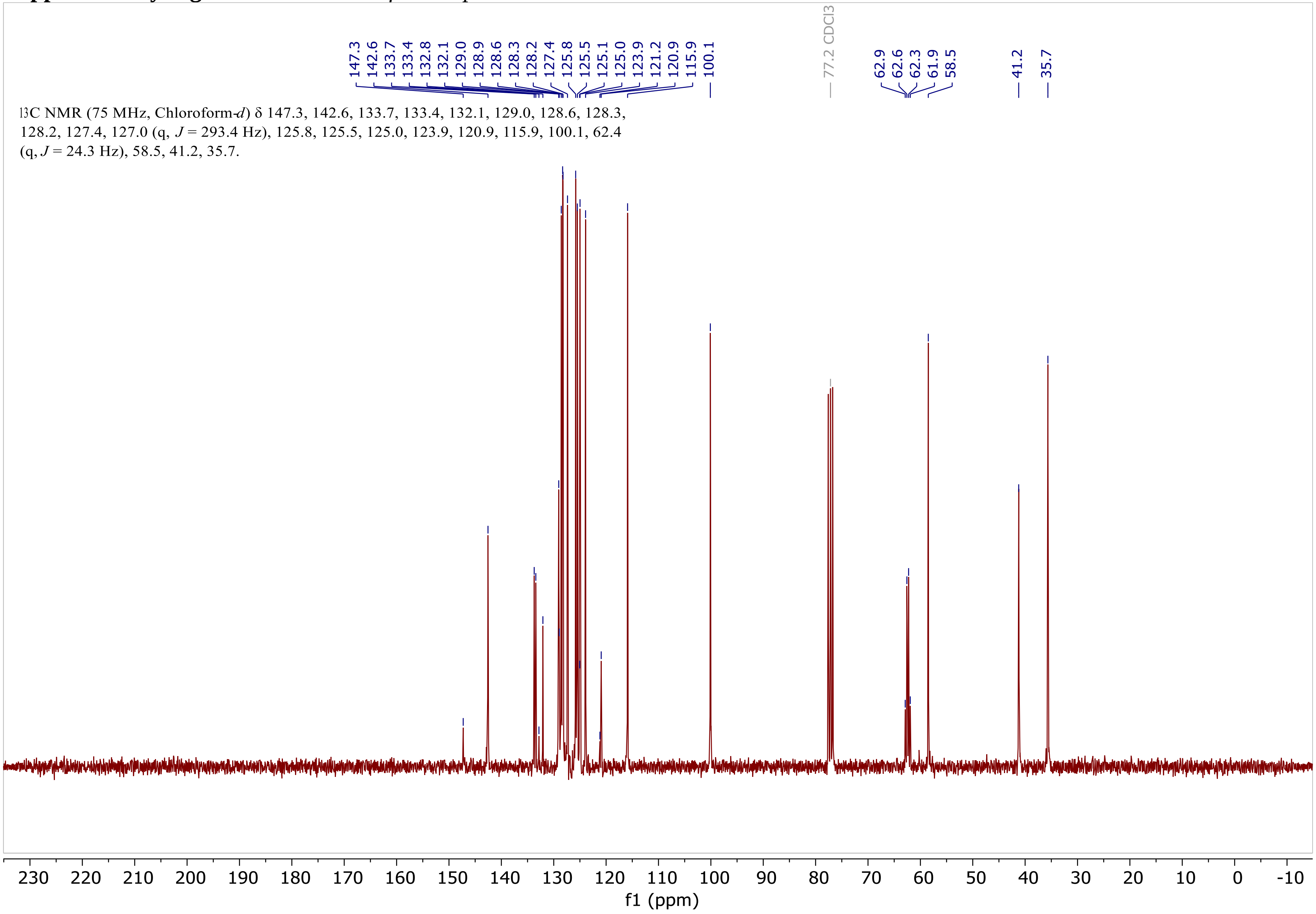

Supplementary Figure 269: *IR for compound* ***55***

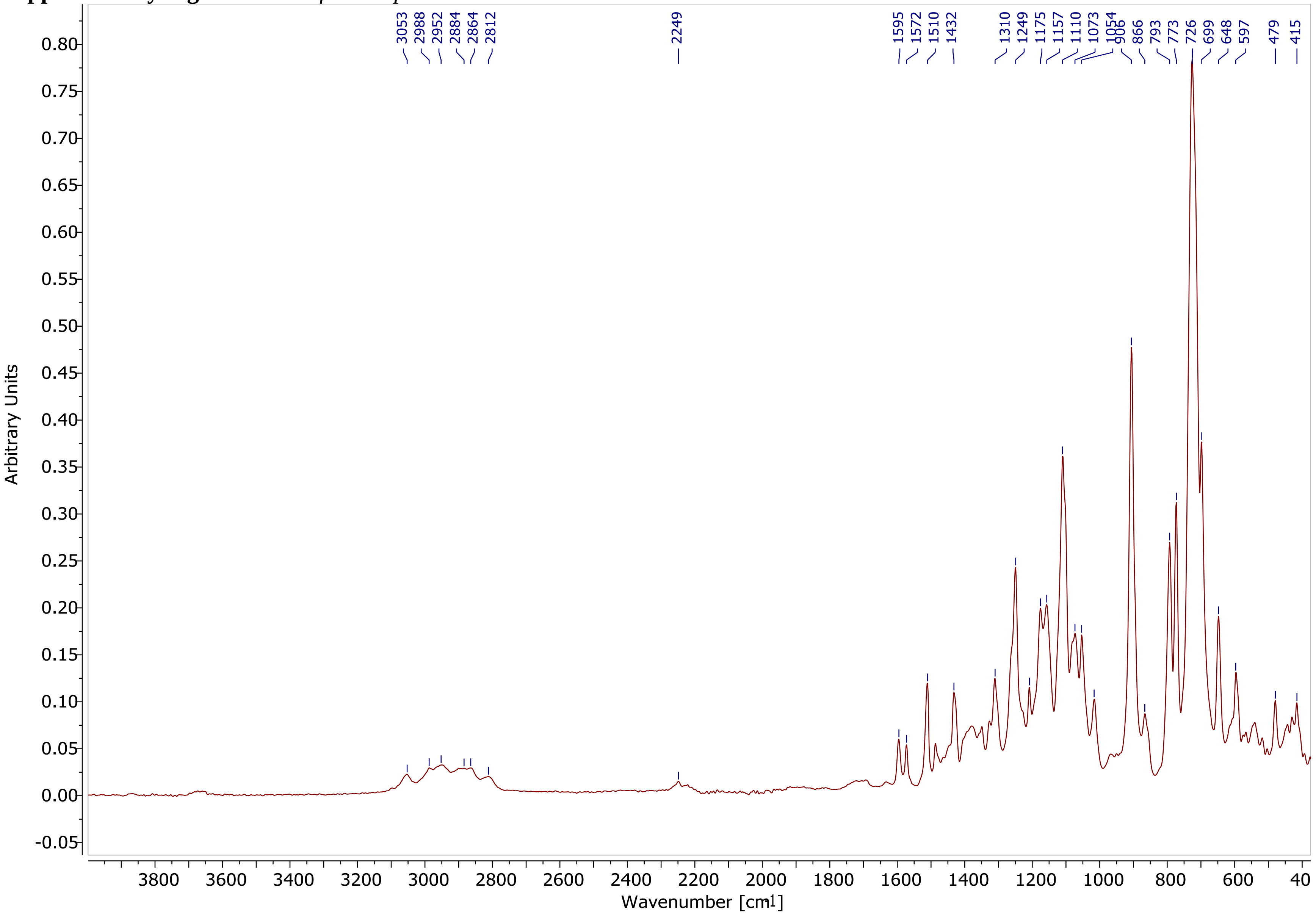

Supplementary Figure 270: *^{1}H-NMR for compound* ***56***

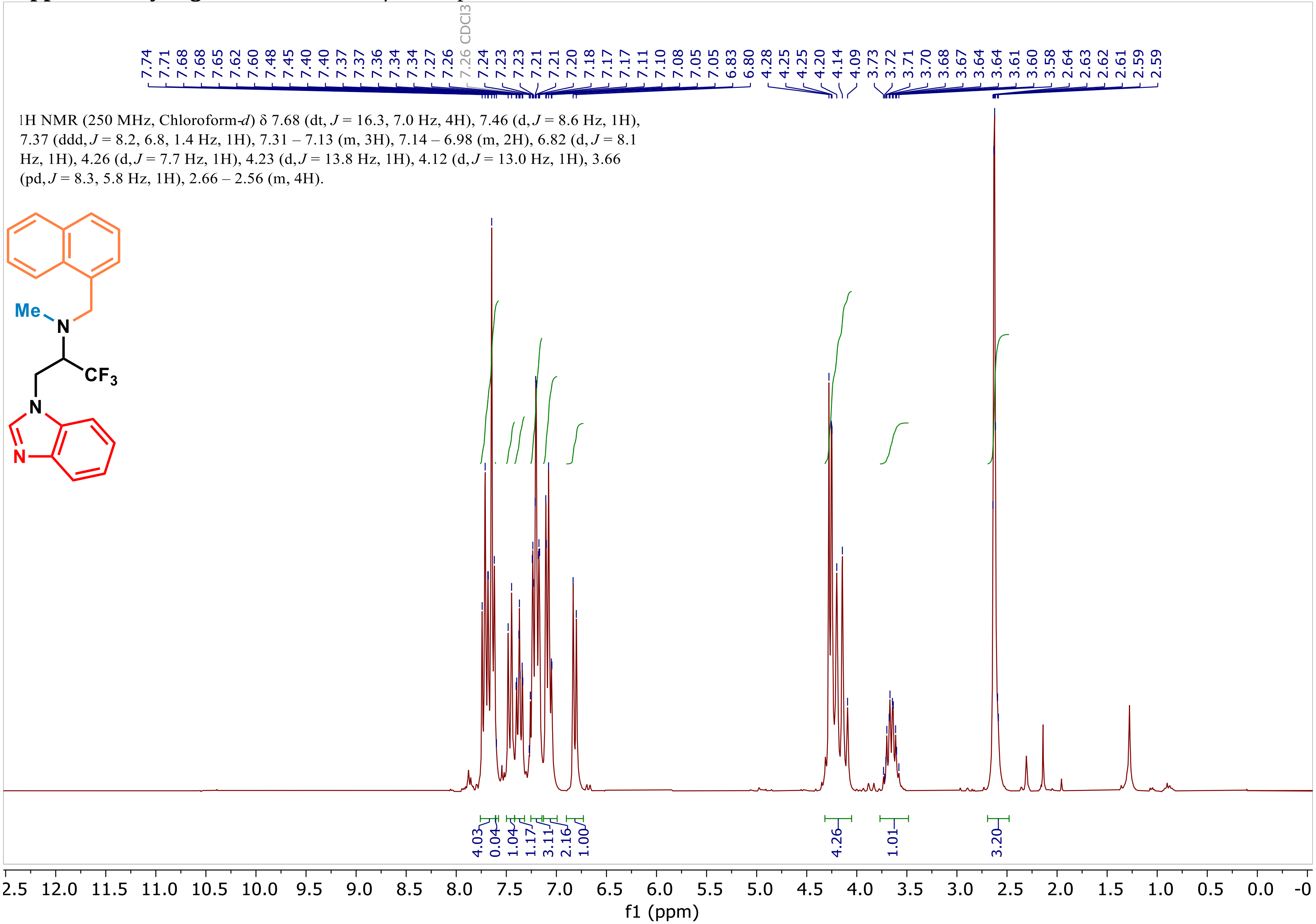

Supplementary Figure 271: *^{19}F NMR for compound* ***56***

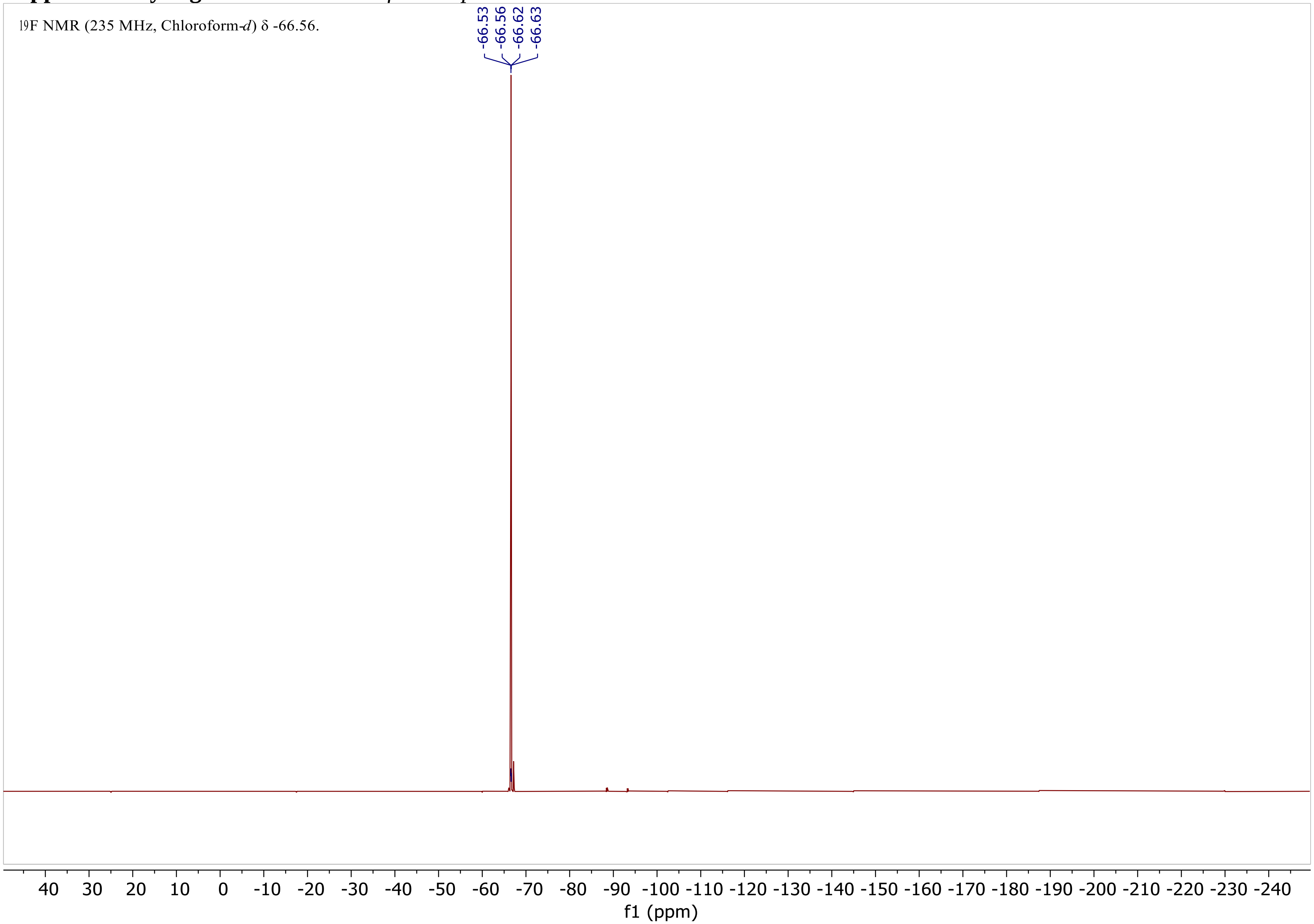

Supplementary Figure 272: *^{13}C NMR for compound 5*6

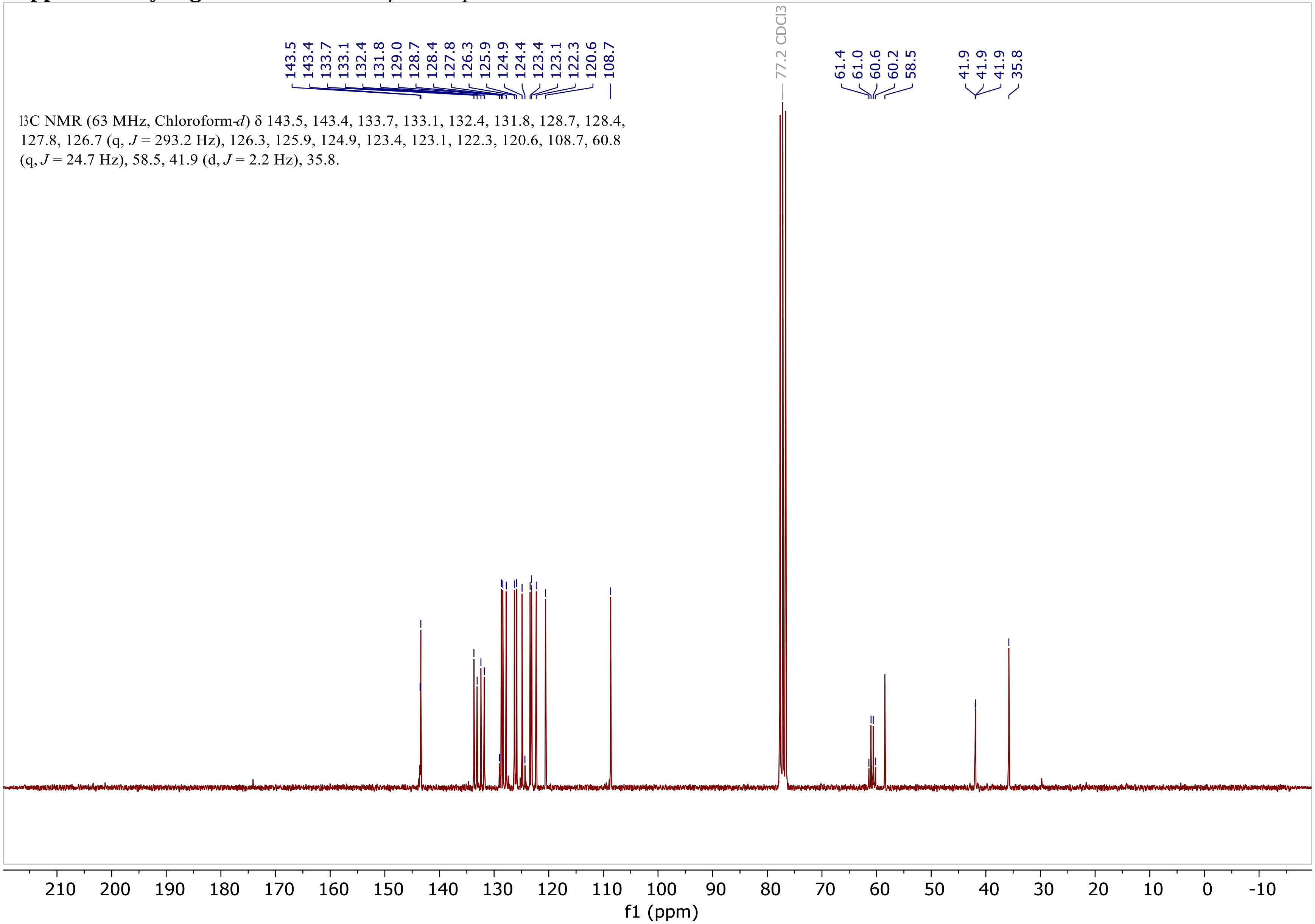

Supplementary Figure 273: *IR for compound **56***

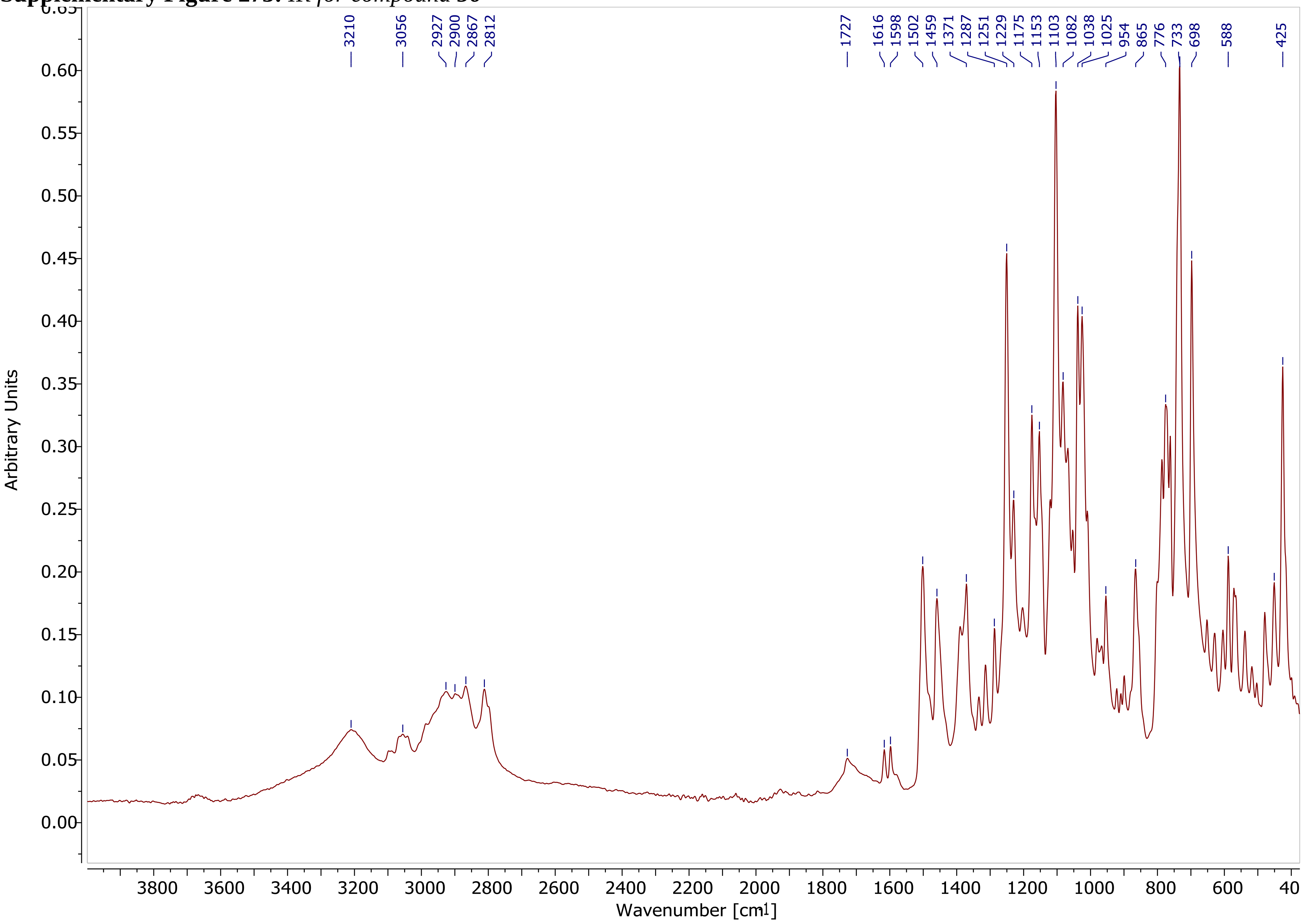

Supplementary Figure 274: *^{1}H-NMR for compound* ***57***

1H NMR (300 MHz, Chloroform-*d*) δ 7.78 – 7.65 (m, 4H), 7.58 (d, *J* = 8.4 Hz, 1H), 7.38 (dd, *J* = 8.8, 6.6 Hz, 1H), 7.31 – 7.23 (m, 1H), 7.22 – 7.18 (m, 2H), 7.15 (d, *J* = 8.1 Hz, 2H), 6.48 (t, *J* = 7.7 Hz, 1H), 4.69 (dd, *J* = 13.9, 3.5 Hz, 1H), 4.57 (dd, *J* = 13.9, 10.2 Hz, 1H), 4.42 – 4.26 (m, 1H), 4.22 (s, 2H), 2.51 (s, 3H).

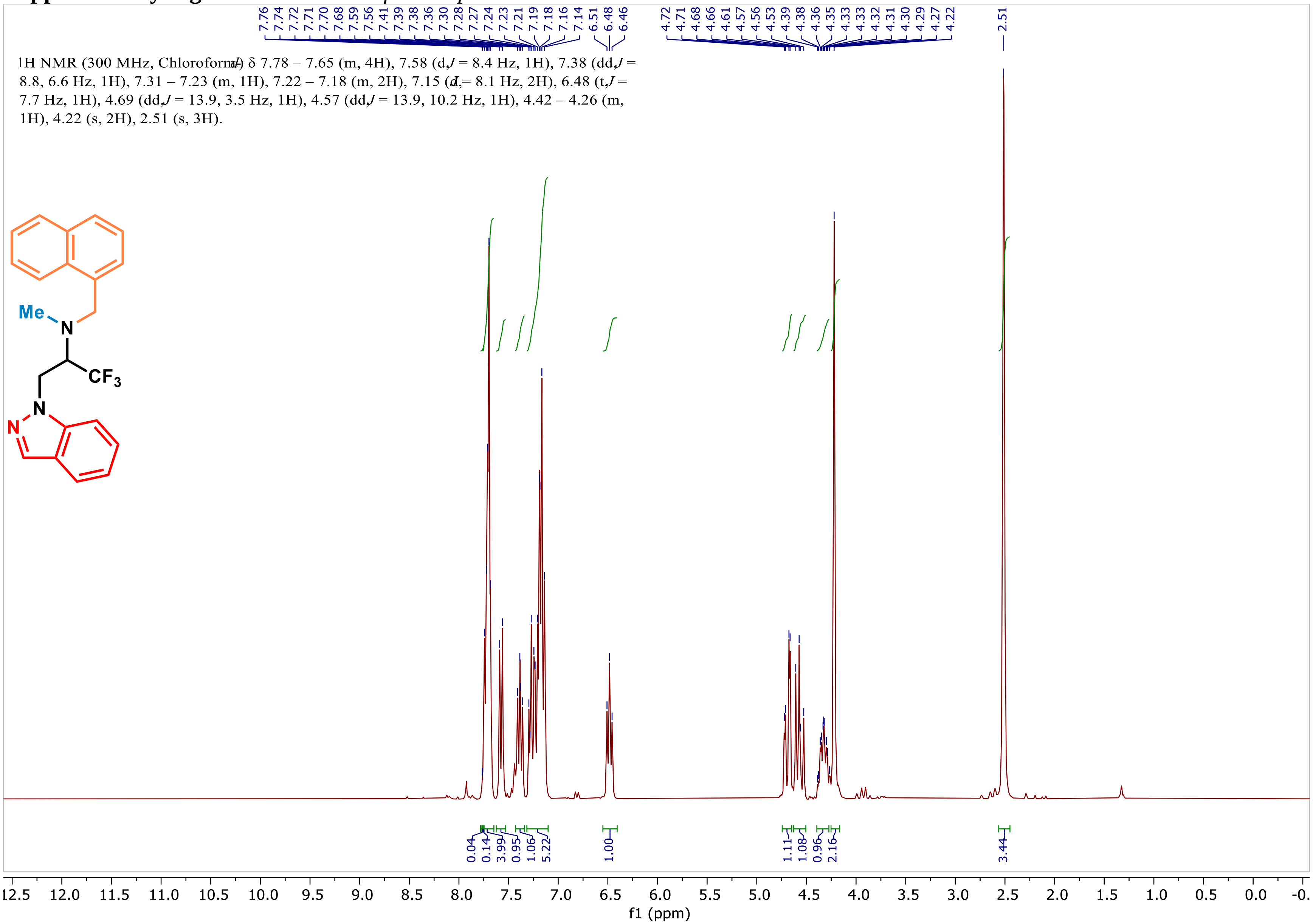

Supplementary Figure 275: *^{19}F NMR for compound* ***57***

^{19}F NMR (282 MHz, Chloroform-*d*) δ -66.87 (d, *J* = 8.3 Hz).

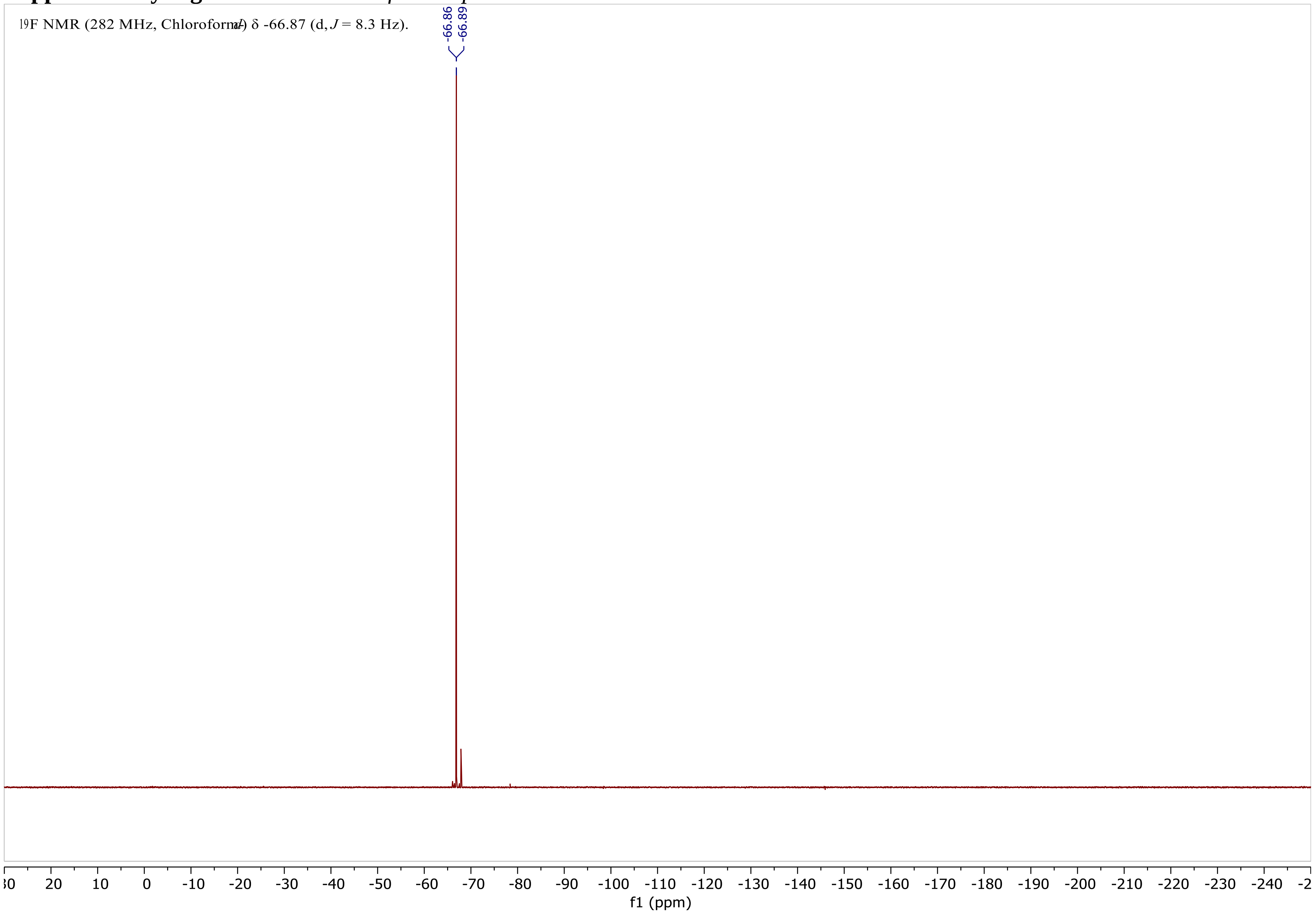

Supplementary Figure 276: *^{13}C NMR for compound* ***57***

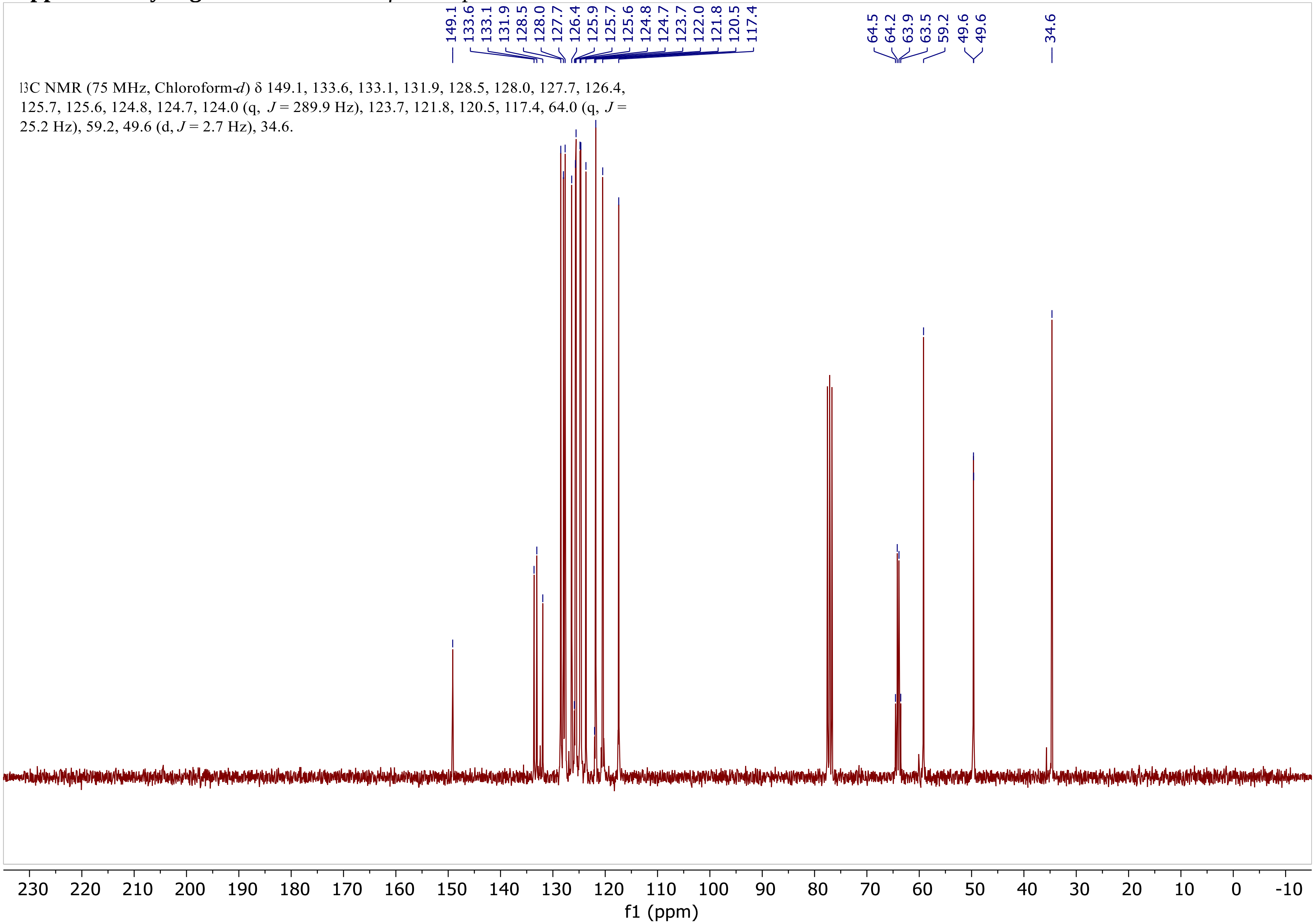

Supplementary Figure 277: *IR for compound* ***57***

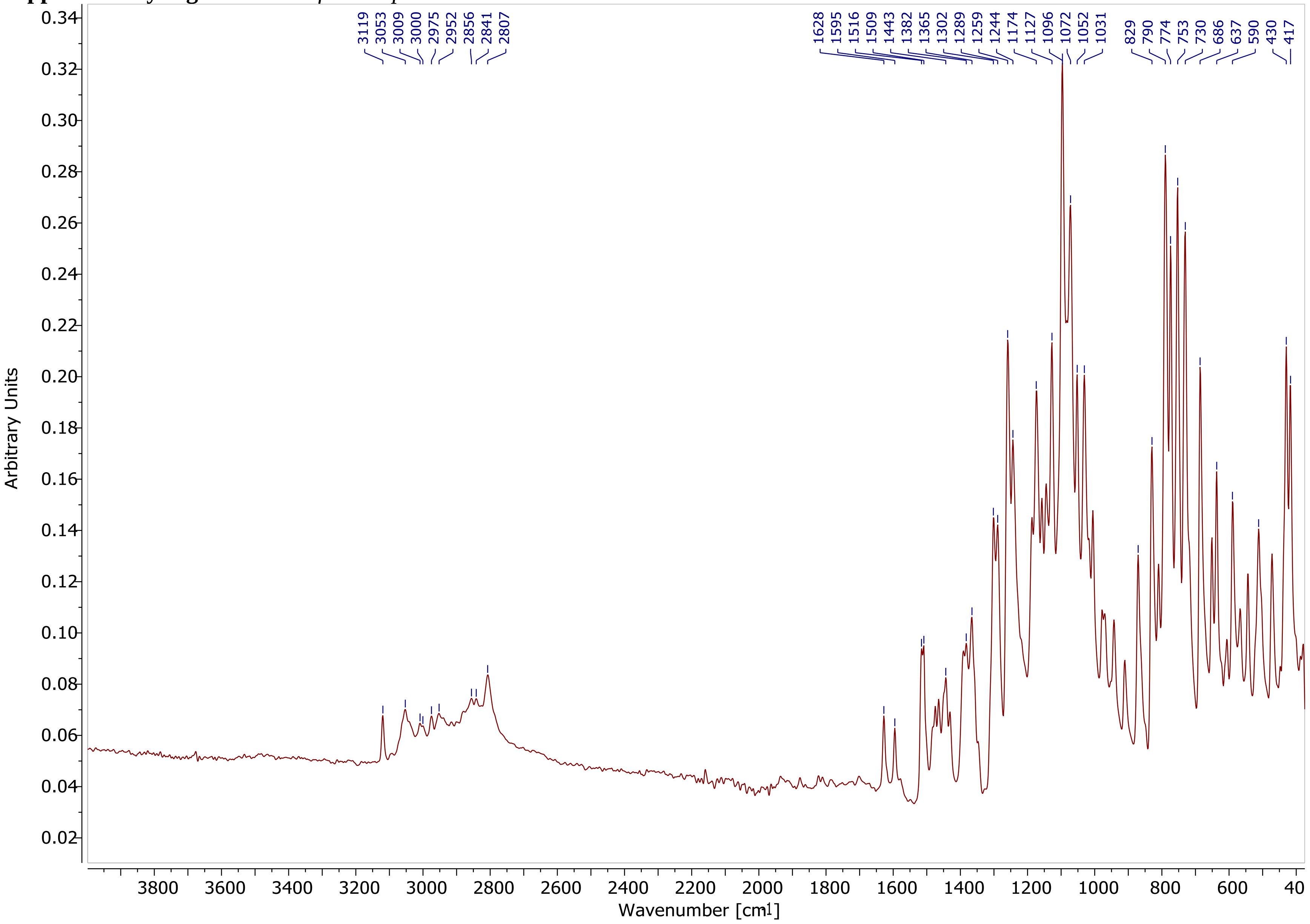

Supplementary Figure 278: *^{1}H-NMR for compound* ***58***

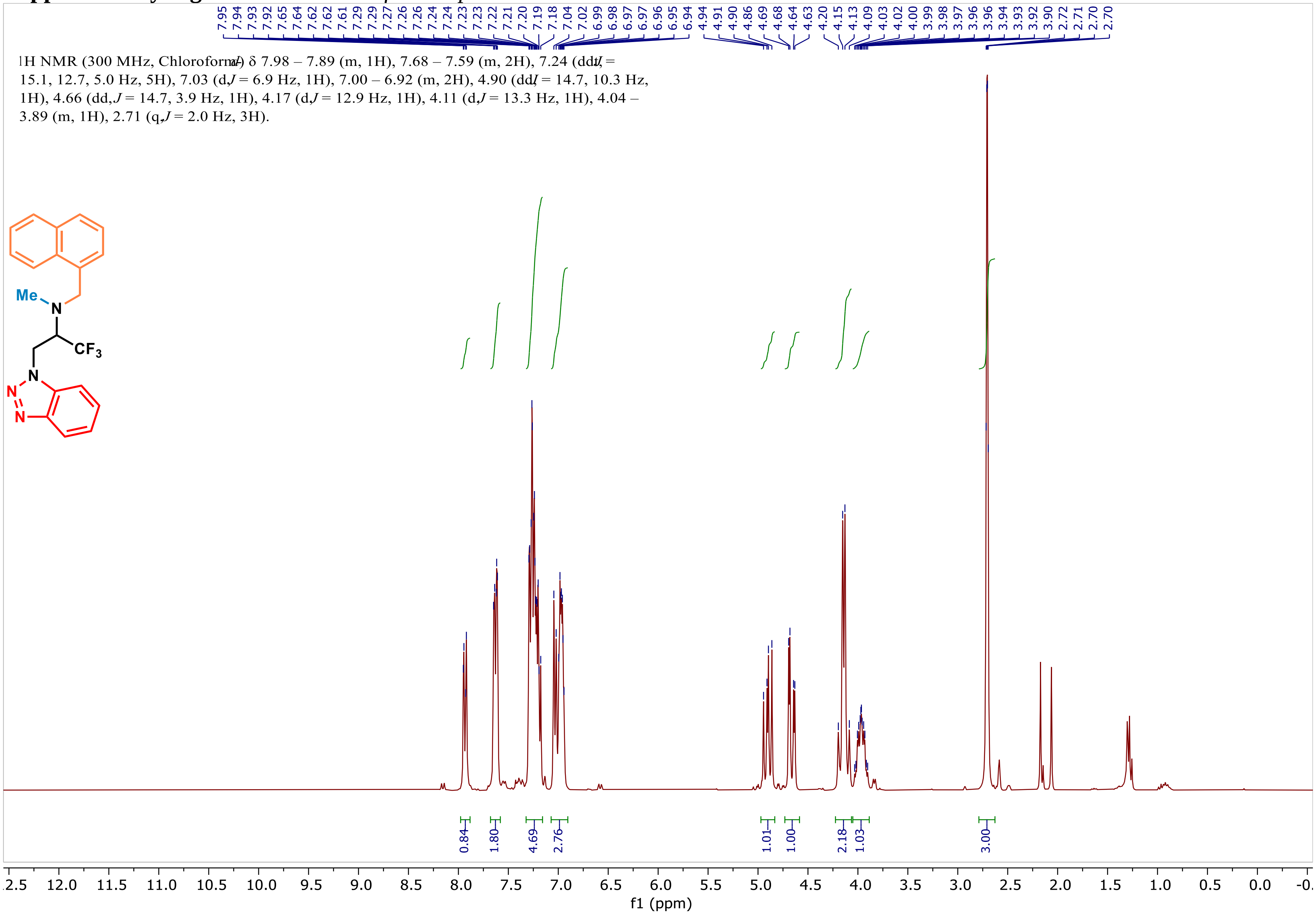

Supplementary Figure 279: *^{19}F NMR for compound **58***

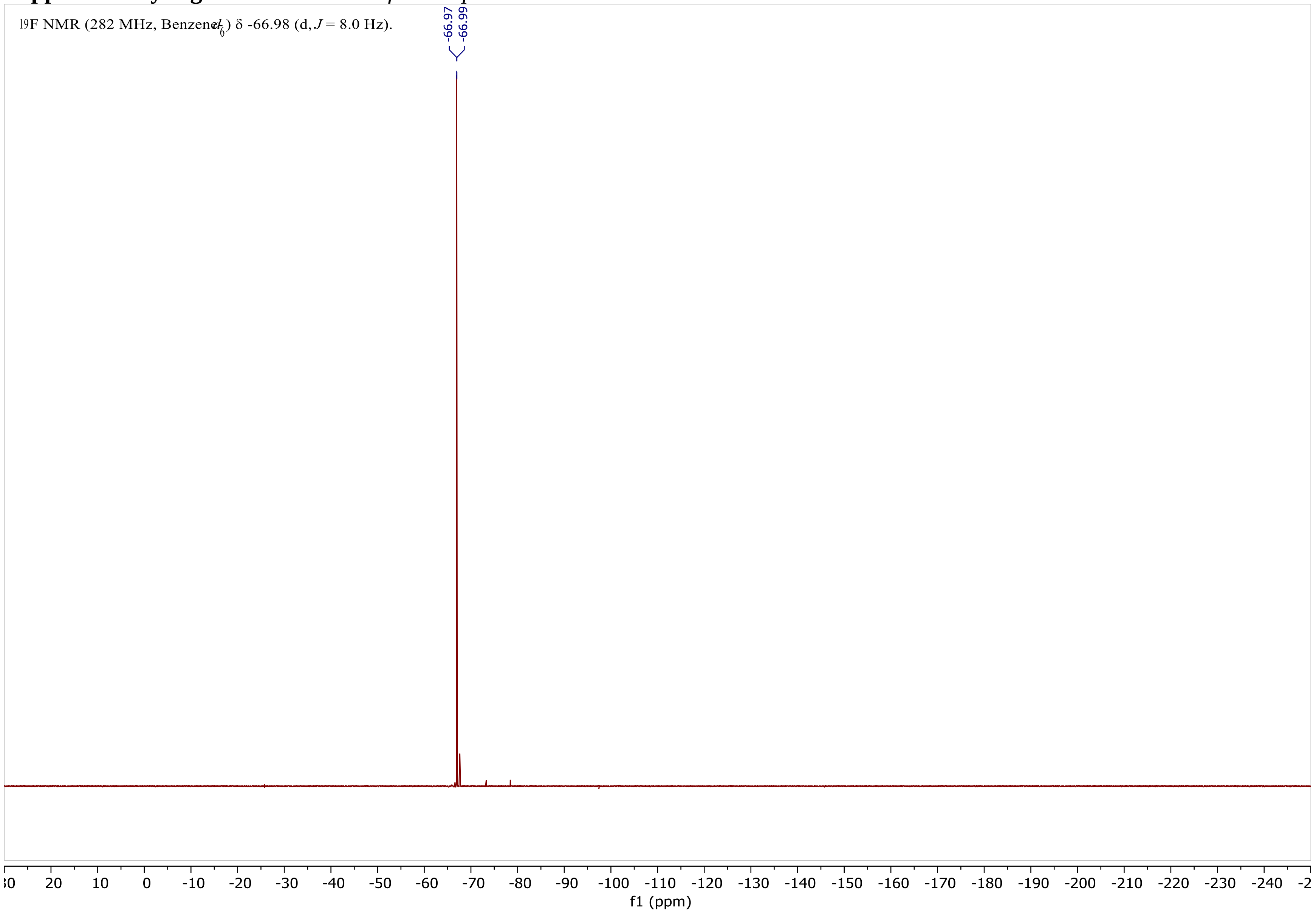

Supplementary Figure 280: *^{13}C NMR for compound **58***

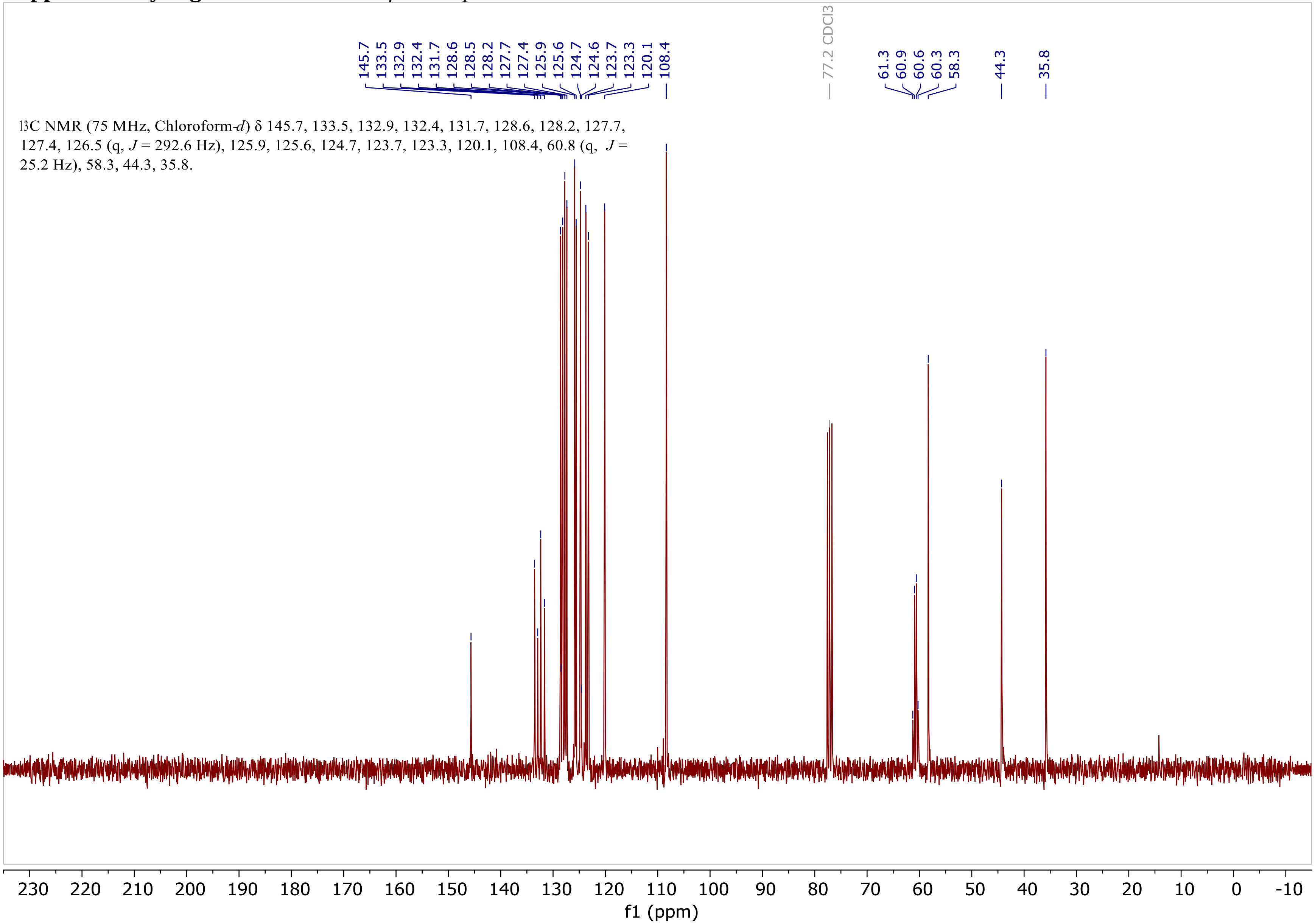

Supplementary Figure 281: *IR for compound **58***

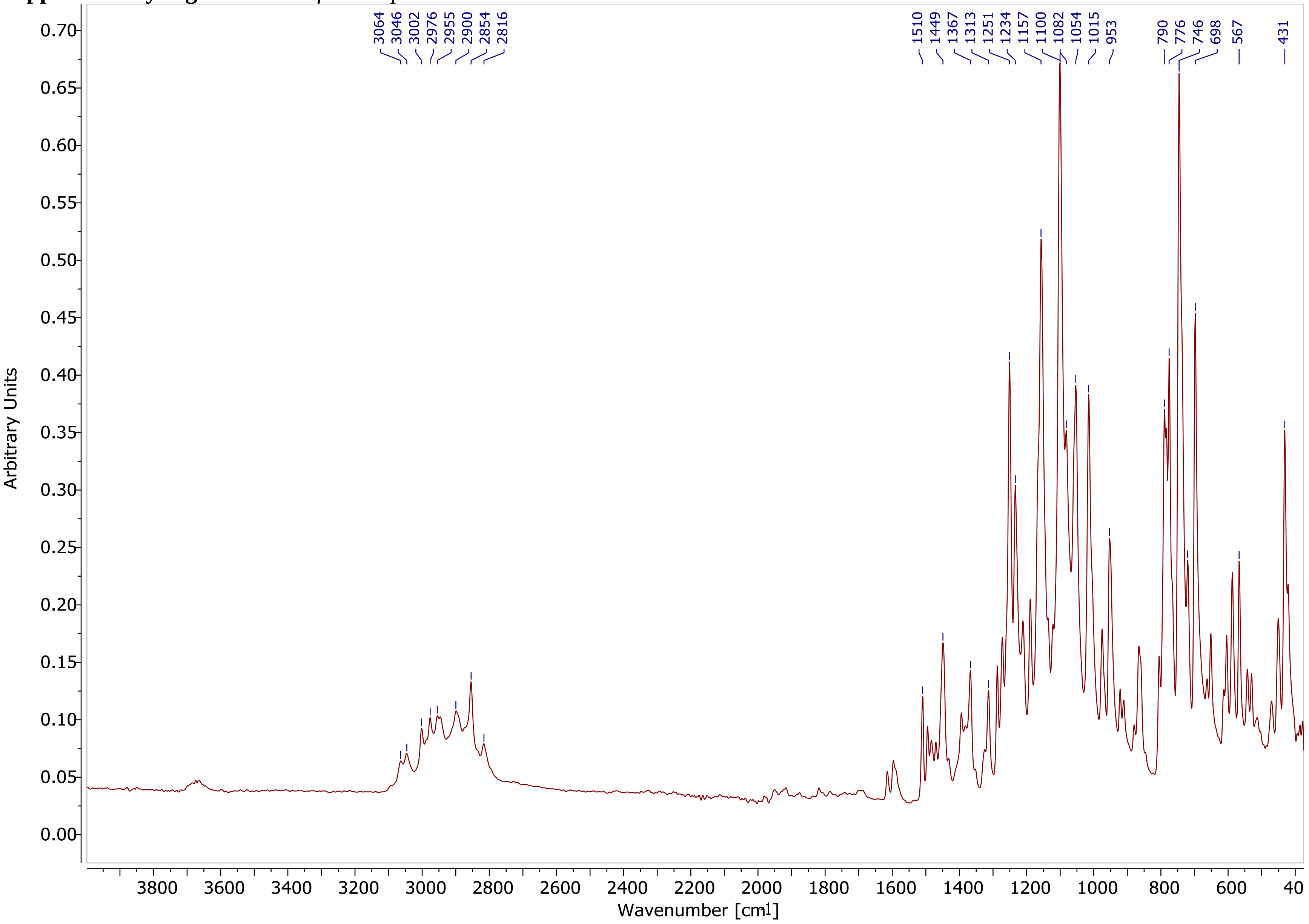

Supplementary Figure 282: *^{1}H-NMR for compound* ***59***

1H NMR (300 MHz, Chloroform-*d*) δ 7.98 – 7.85 (m, 2H), 7.65 (d, *J* = 8.2 Hz, 1H), 7.48 (qd, *J* = 9.5, 8.6, 5.1 Hz, 4H), 7.30 (t, *J* = 7.6 Hz, 1H), 7.21 (d, *J* = 6.9 Hz, 1H), 7.09 (dt, *J* = 6.4, 3.4 Hz, 2H), 5.03 (dd, *J* = 14.0, 11.0 Hz, 1H), 4.59 (dd, *J* = 14.0, 3.5 Hz, 1H), 4.20 (dd, *J* = 13.2, 8.7 Hz, 2H), 4.09 (ddd, *J* = 16.1, 8.1, 4.2 Hz, 1H), 2.67 (q, *J* = 2.0 Hz, 3H).

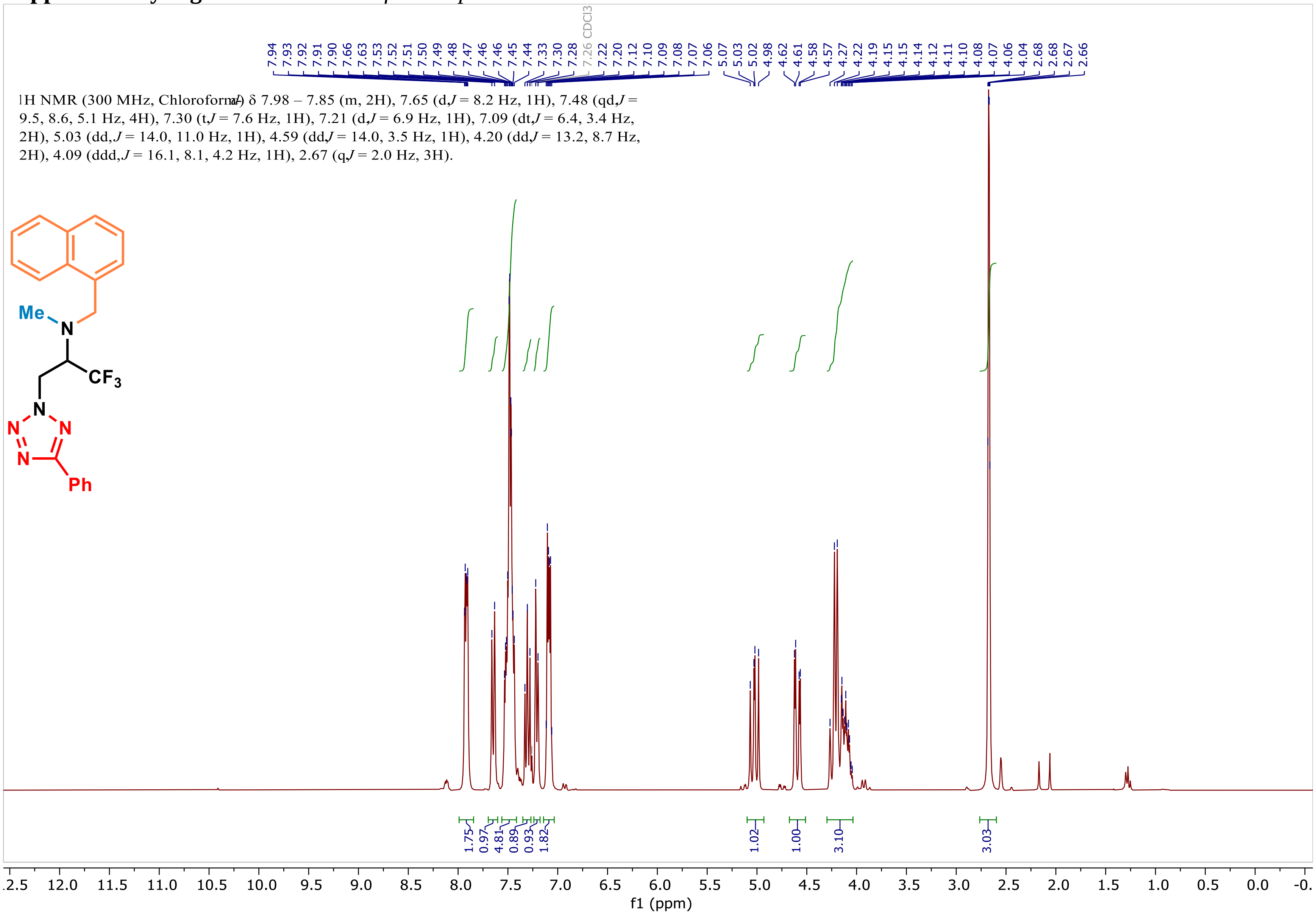

Supplementary Figure 283: *COSY NMR for compound* ***59***

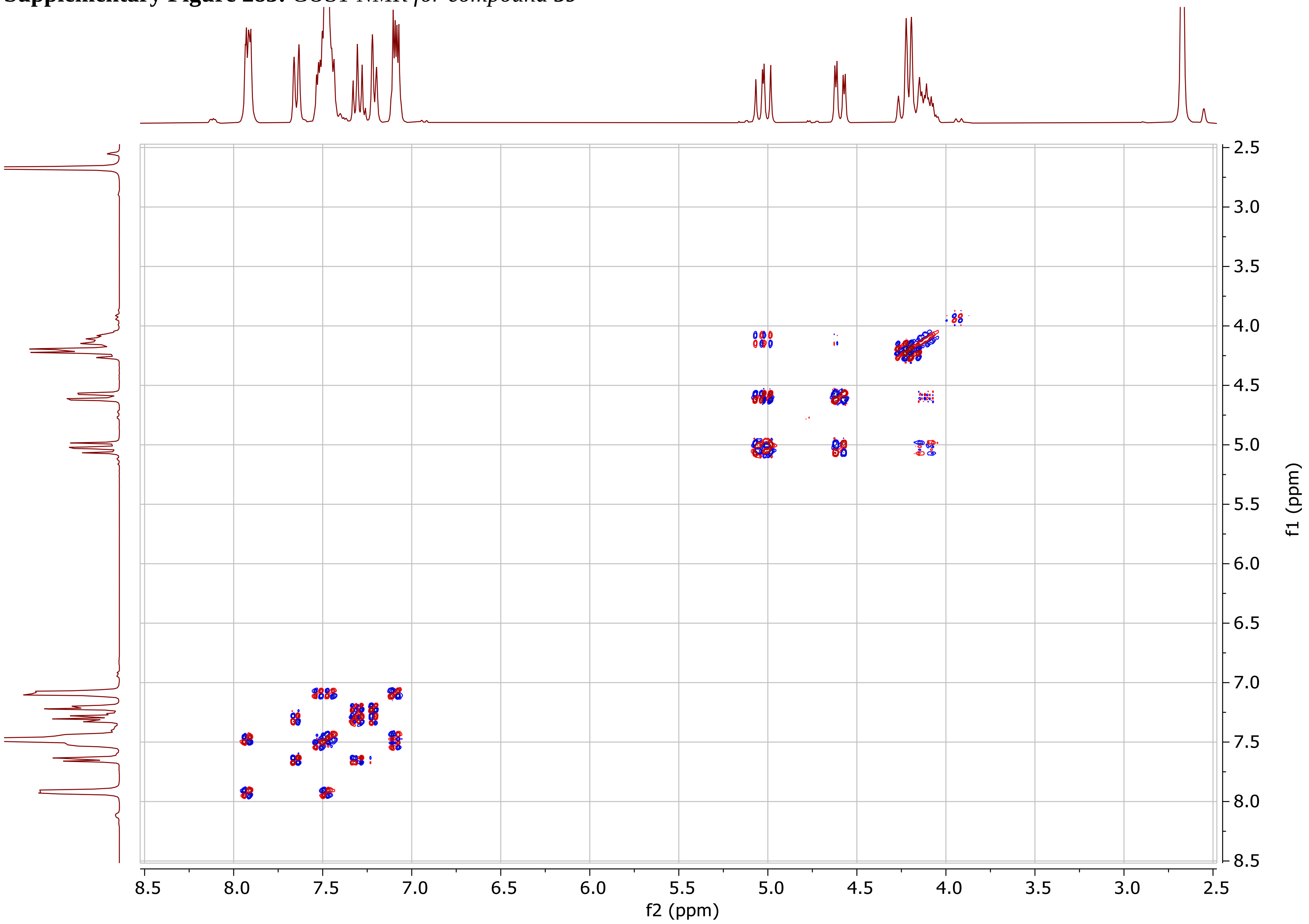

Supplementary Figure 284: *^{19}F NMR for compound* ***59***

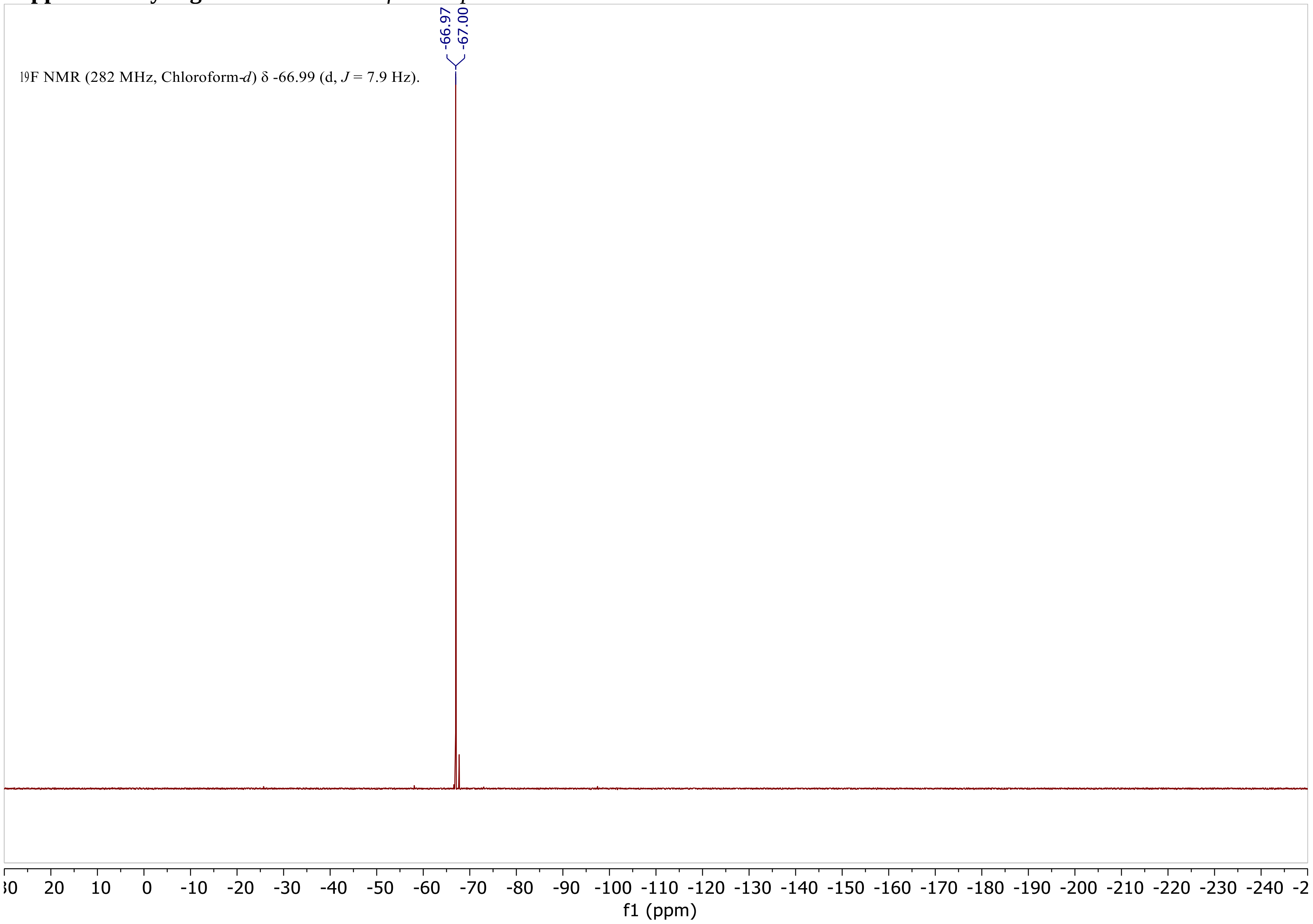

Supplementary Figure 285: *^{13}C NMR for compound* ***59***

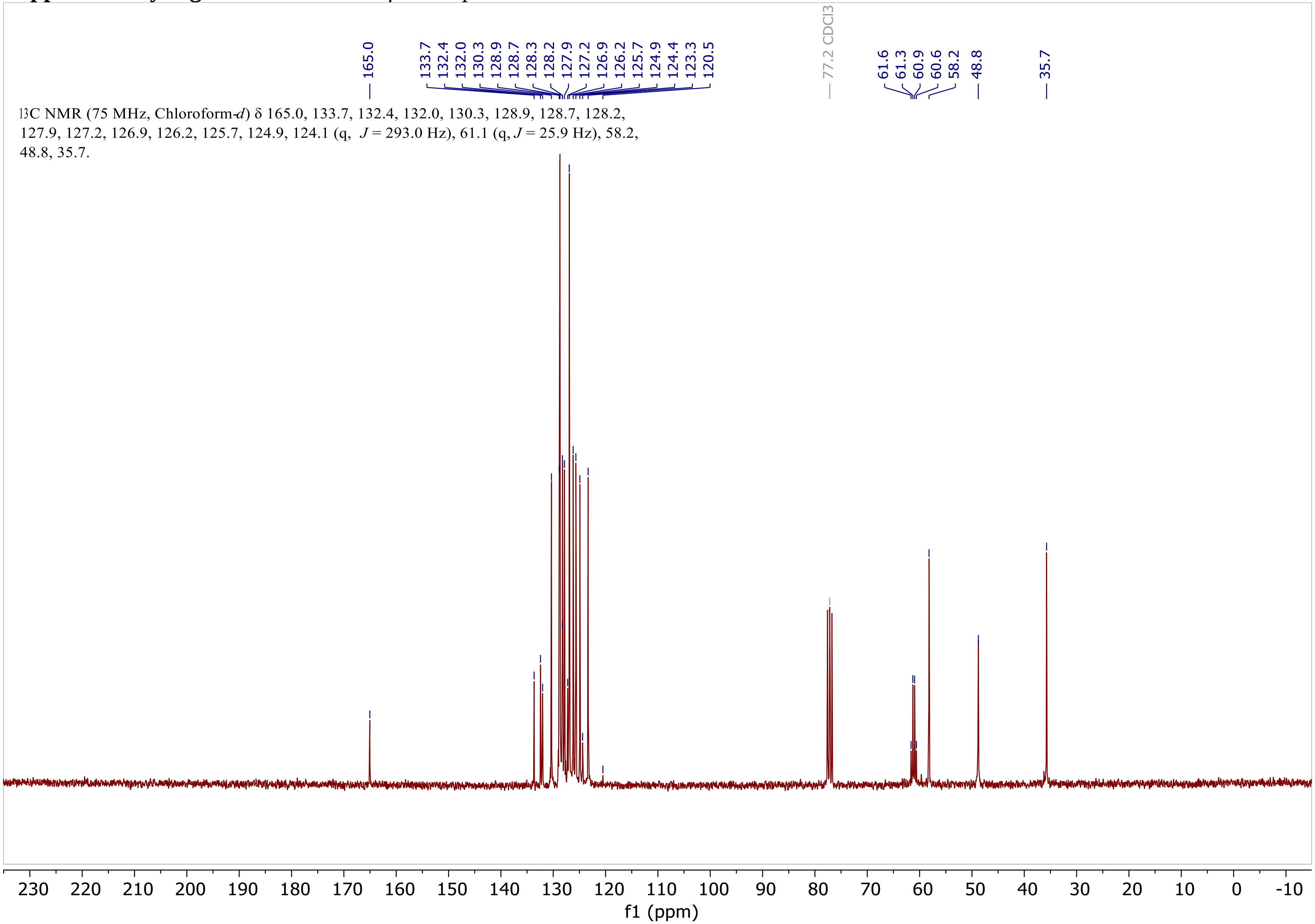

Supplementary Figure 286: *HSQC NMR for compound* ***59***

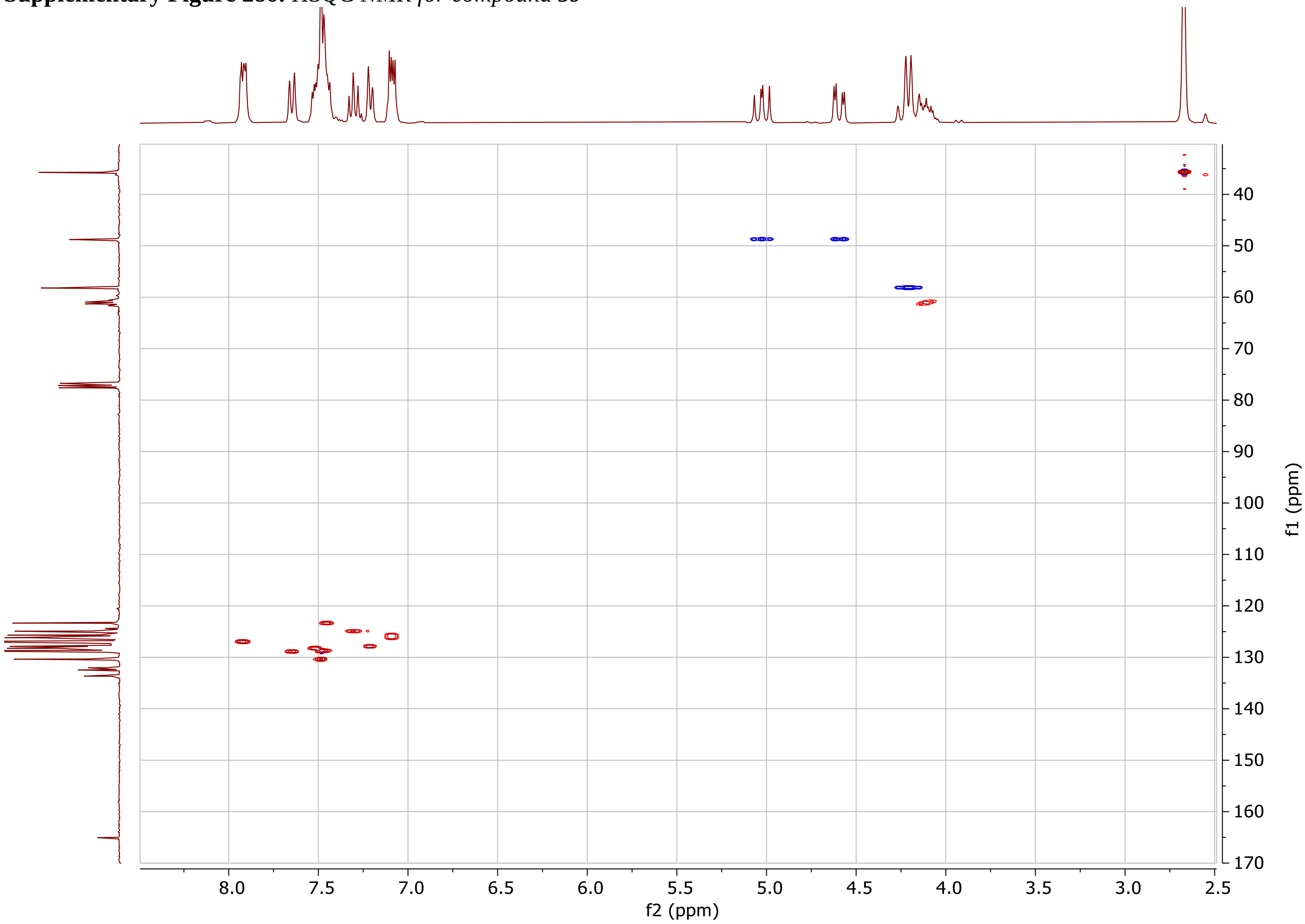

Supplementary Figure 287: *HMBC NMR for compound* ***59***

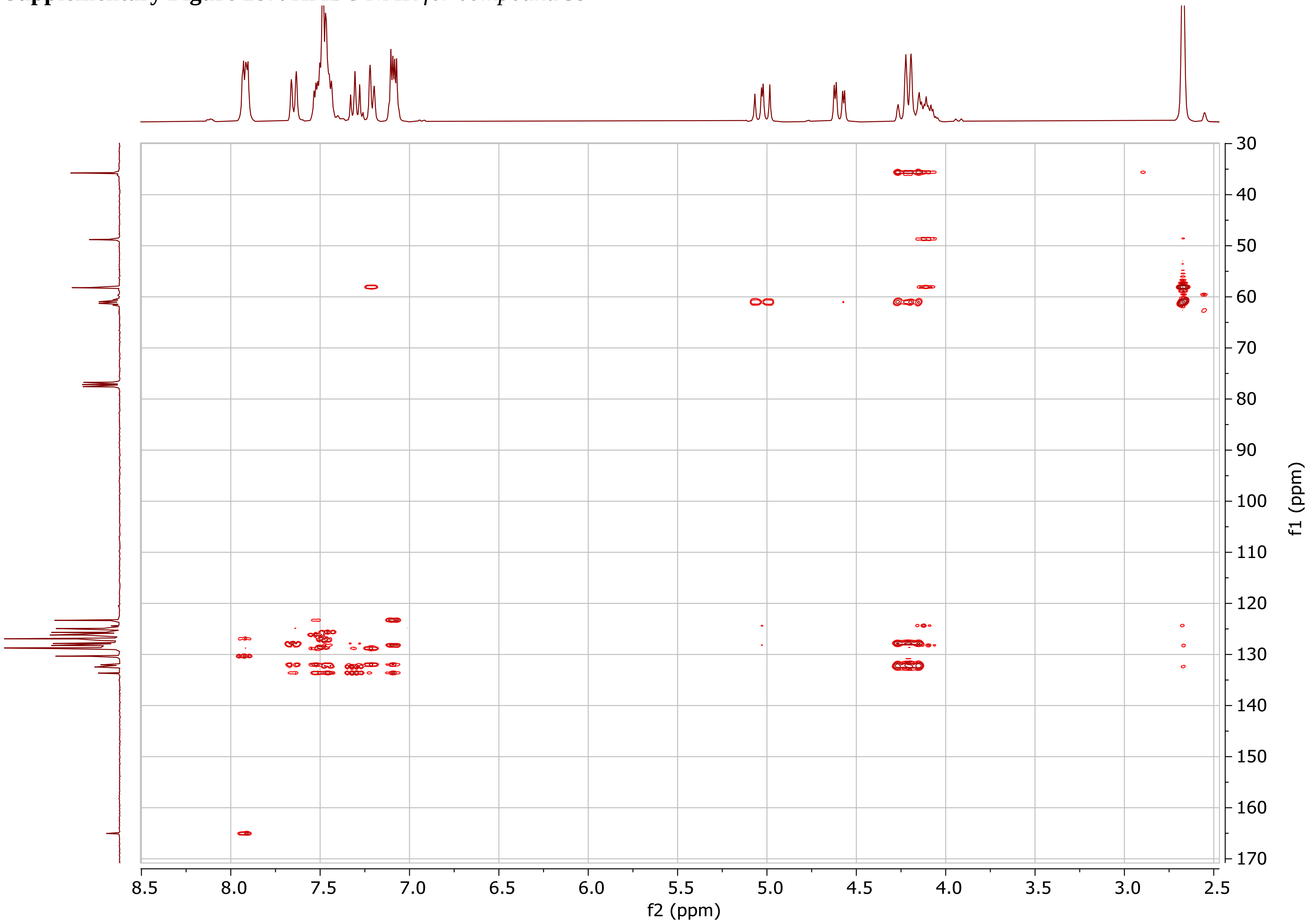

Supplementary Figure 288: *NOESY NMR for compound* ***59***

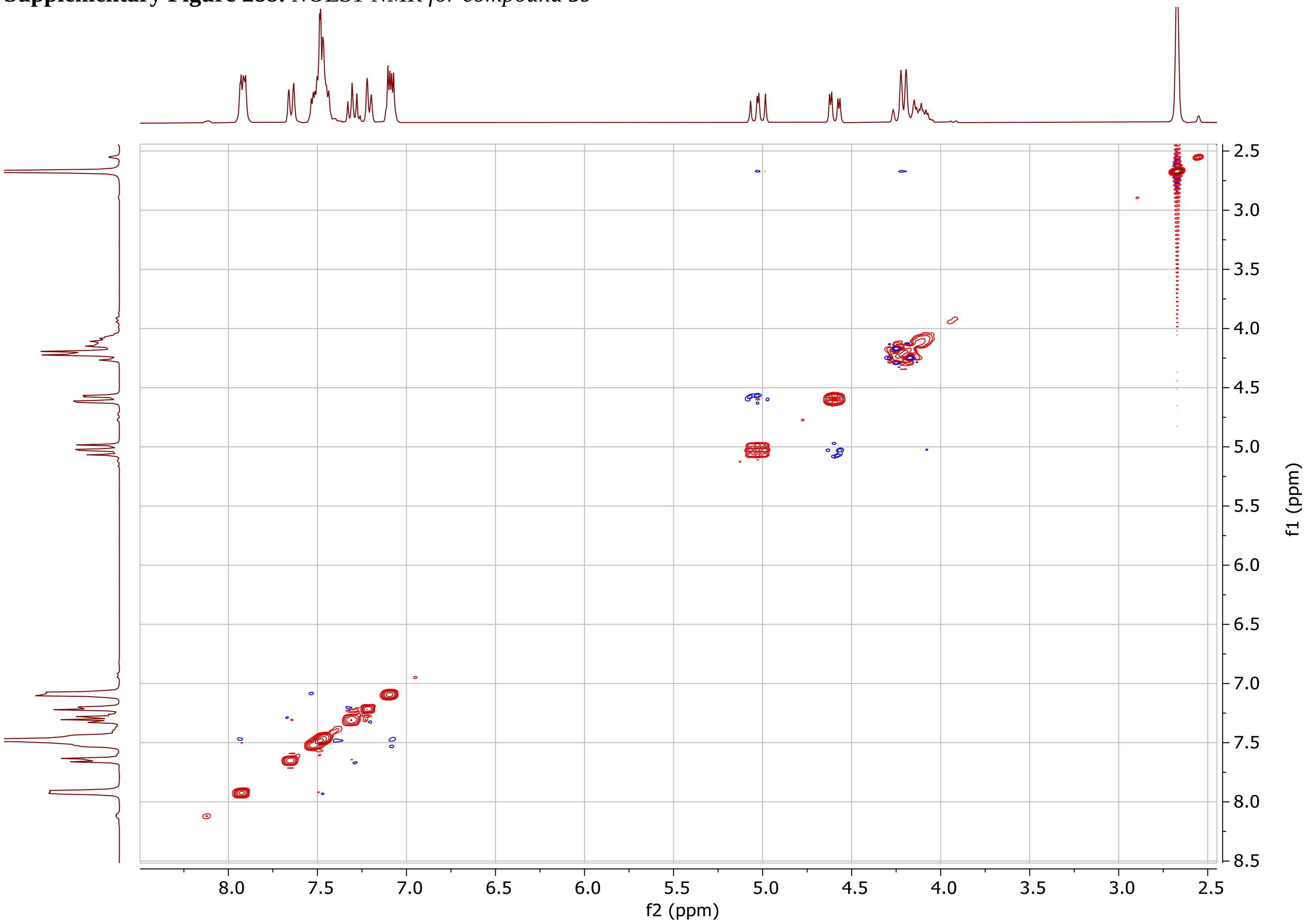

Supplementary Figure 289: *NOESY NMR for compound* ***59***

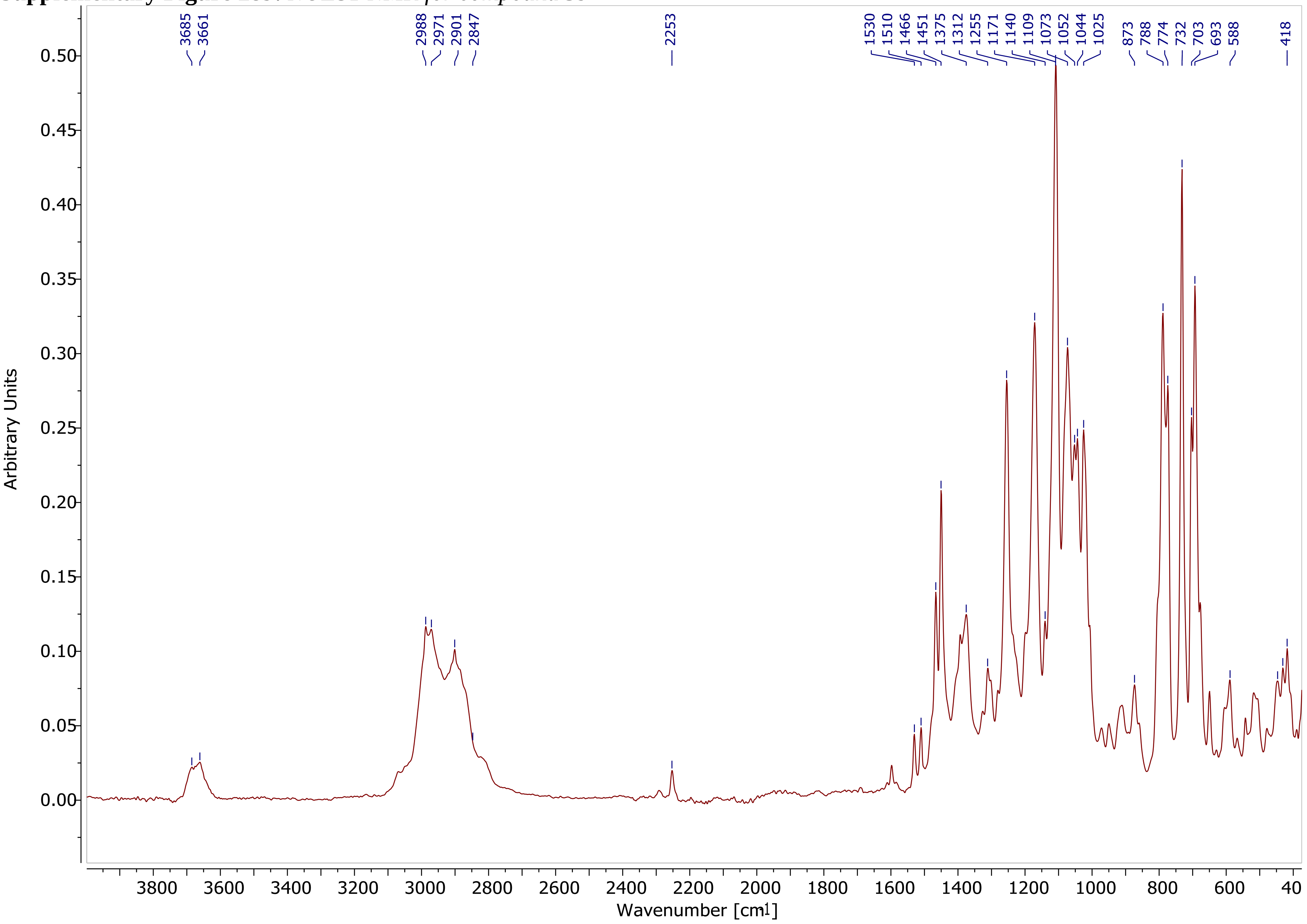

Supplementary Figure 290: *^{1}H-NMR for compound* ***60***

1H NMR (500 MHz, Chloroform-*d*) δ 8.22 (s, 1H), 7.68 (d, *J* = 8.1 Hz, 1H), 7.65 (d, *J* = 8.2 Hz, 1H), 7.34 (d, *J* = 8.5 Hz, 1H), 7.31 (ddd, *J* = 8.0, 6.9, 1.2 Hz, 1H), 7.24 (dd, *J* = 8.3, 6.9 Hz, 1H), 7.10 (dd, *J* = 6.9, 1.2 Hz, 1H), 7.07 (ddd, *J* = 8.3, 6.9, 1.3 Hz, 1H), 6.64 (d, *J* = 3.5 Hz, 1H), 6.22 (dd, *J* = 3.5, 0.7 Hz, 1H), 4.37 (dd, *J* = 14.5, 10.1 Hz, 1H), 4.32 (dd, *J* = 14.4, 4.4 Hz, 1H), 4.17 (d, *J* = 12.8 Hz, 1H), 4.14 (s, 3H), 4.09 (d, *J* = 13.1 Hz, 1H), 4.01 – 3.90 (m, 1H), 2.63 (q, *J* = 2.0 Hz, 3H).

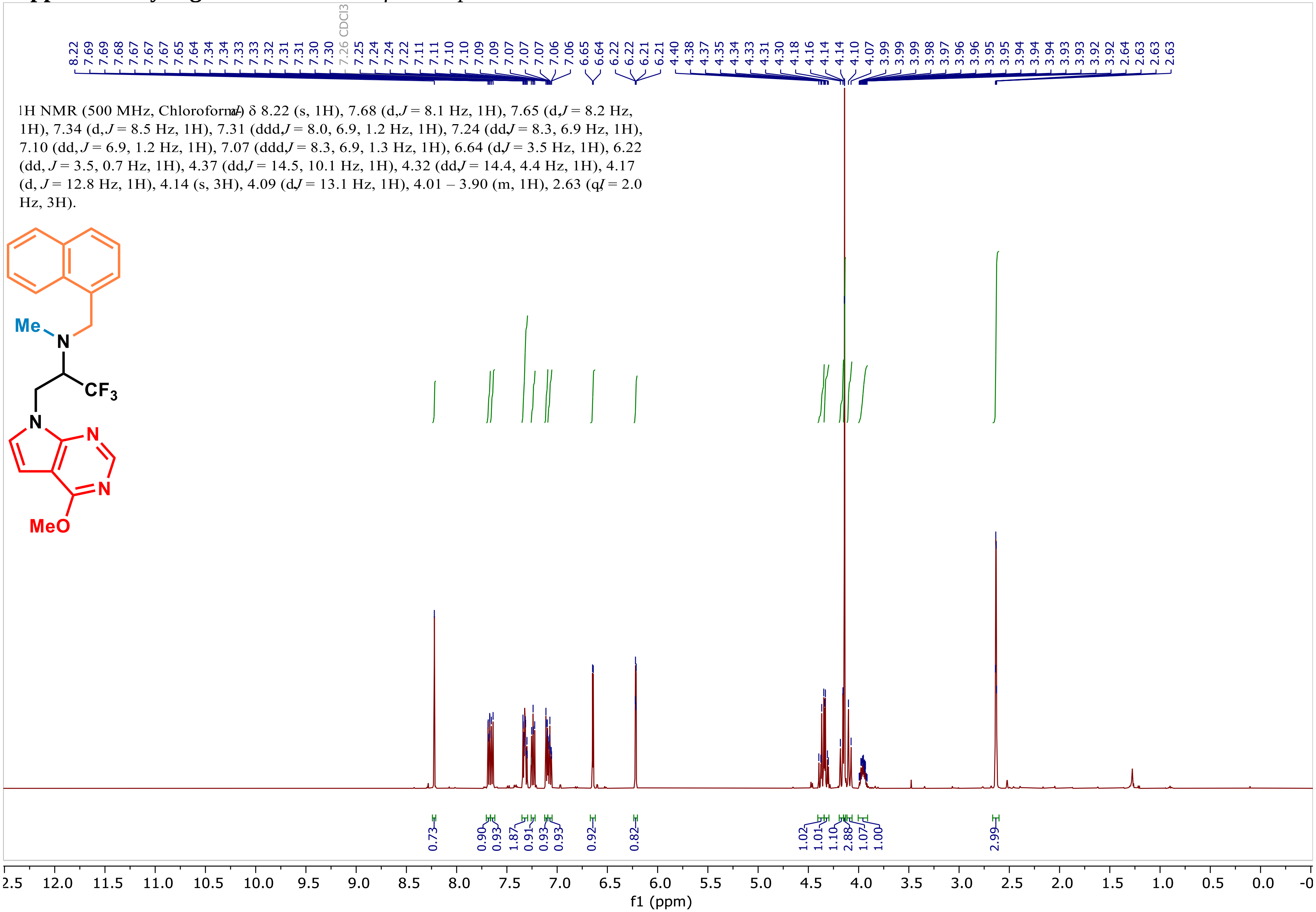

Supplementary Figure 291: *COSY NMR for compound* ***60***

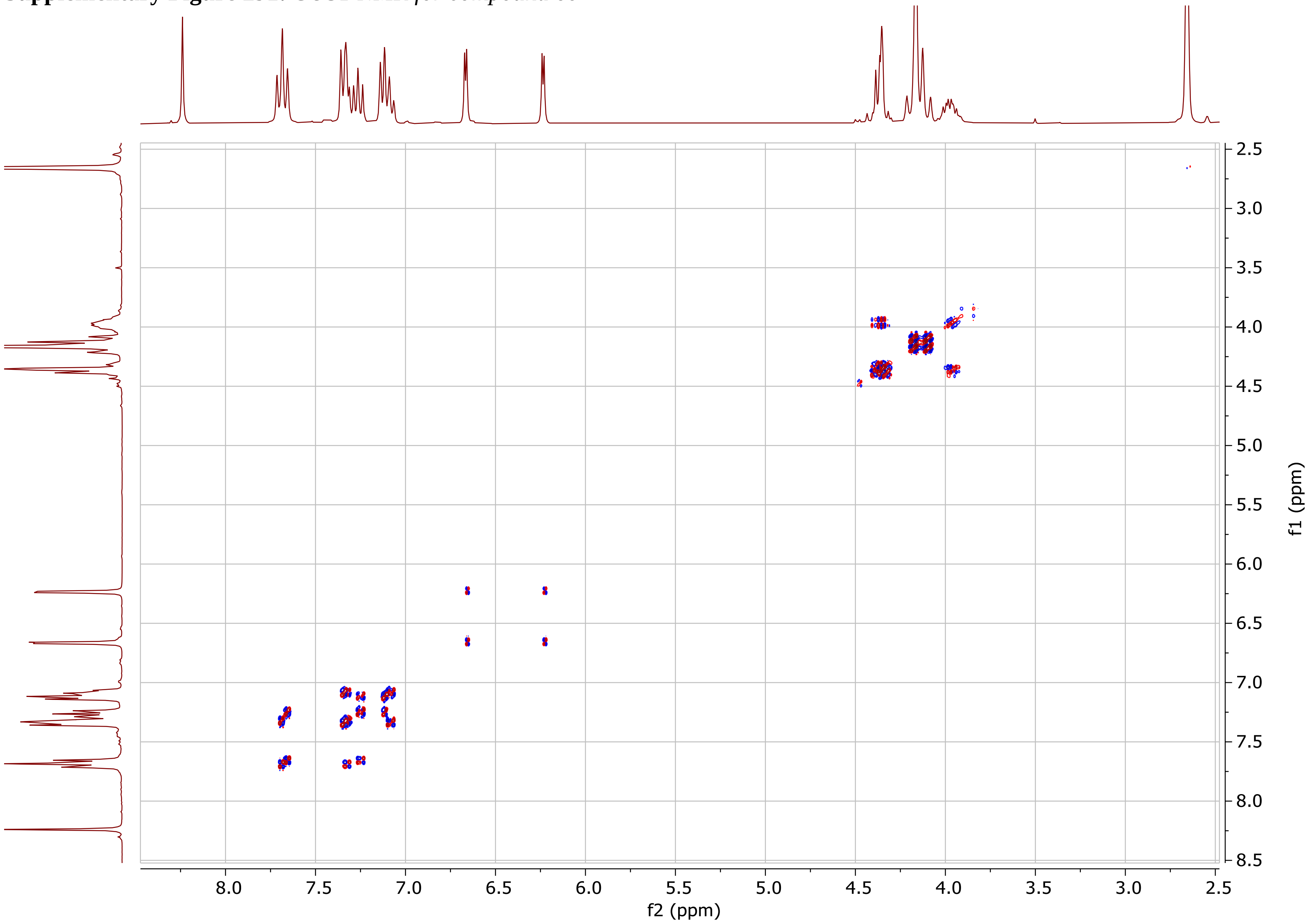

Supplementary Figure 292: *^{19}F NMR for compound* ***60***

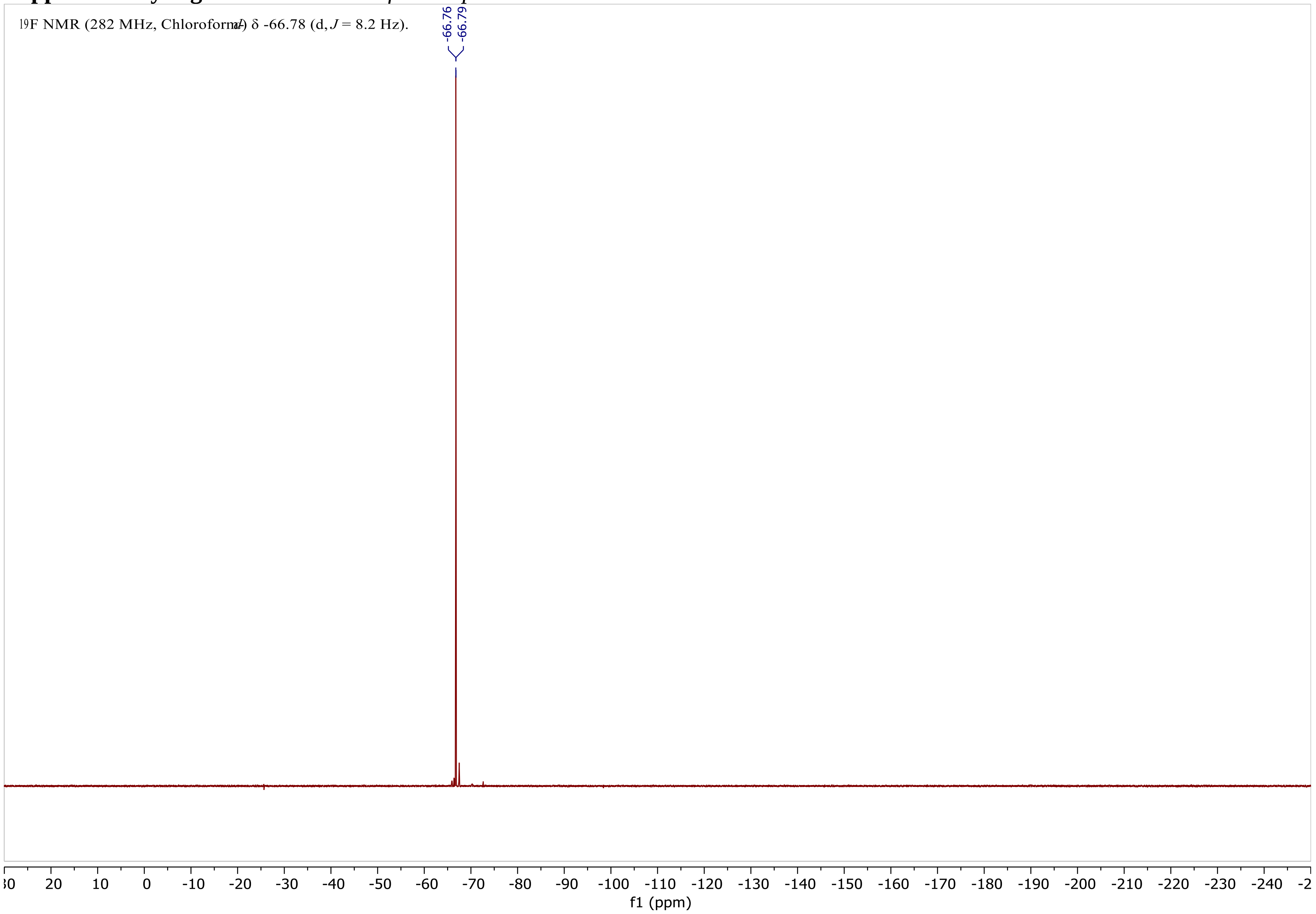

Supplementary Figure 293: ^{13}*C NMR for compound* ***60***

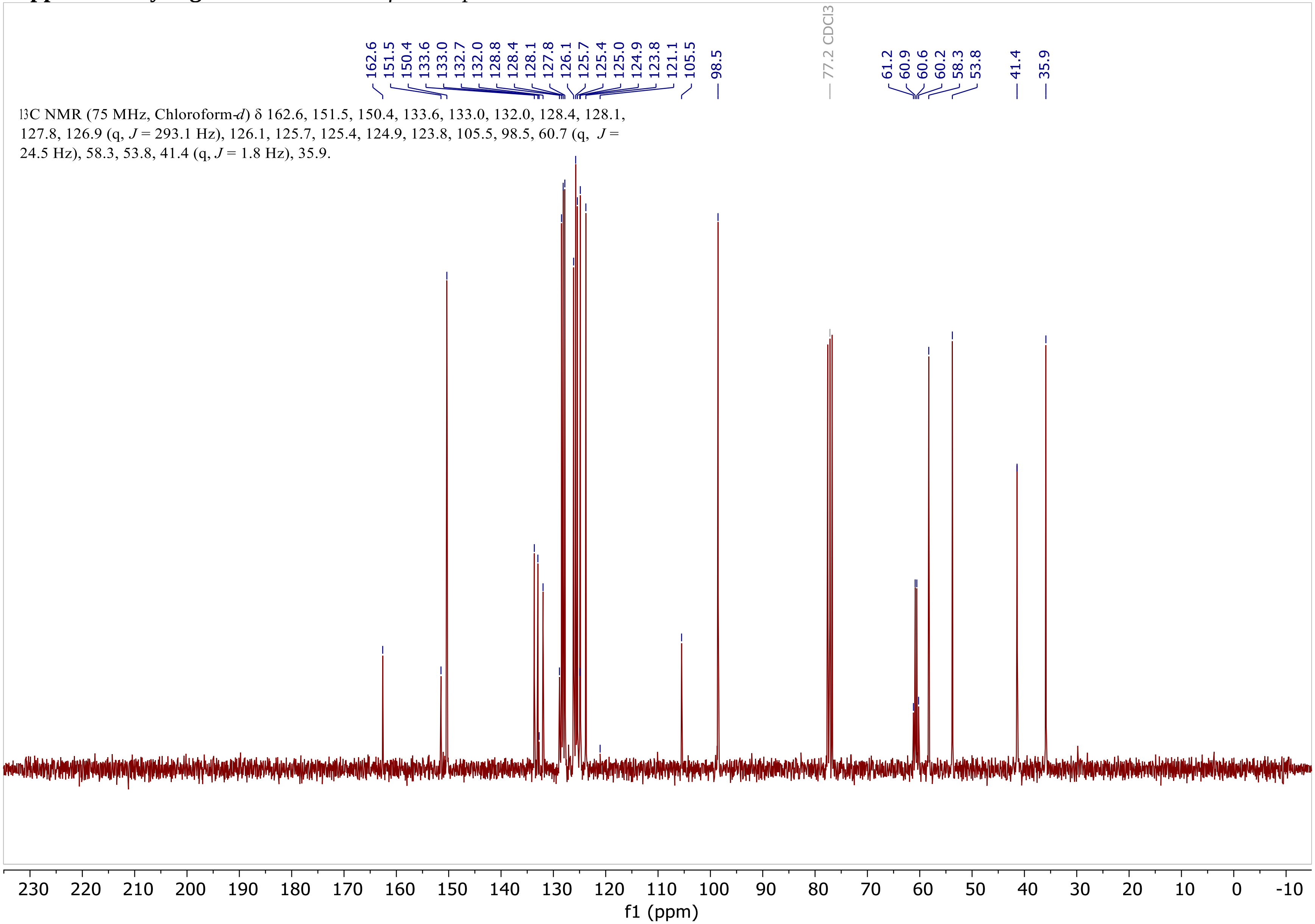

Supplementary Figure 294: *HSQC NMR for compound* ***60***

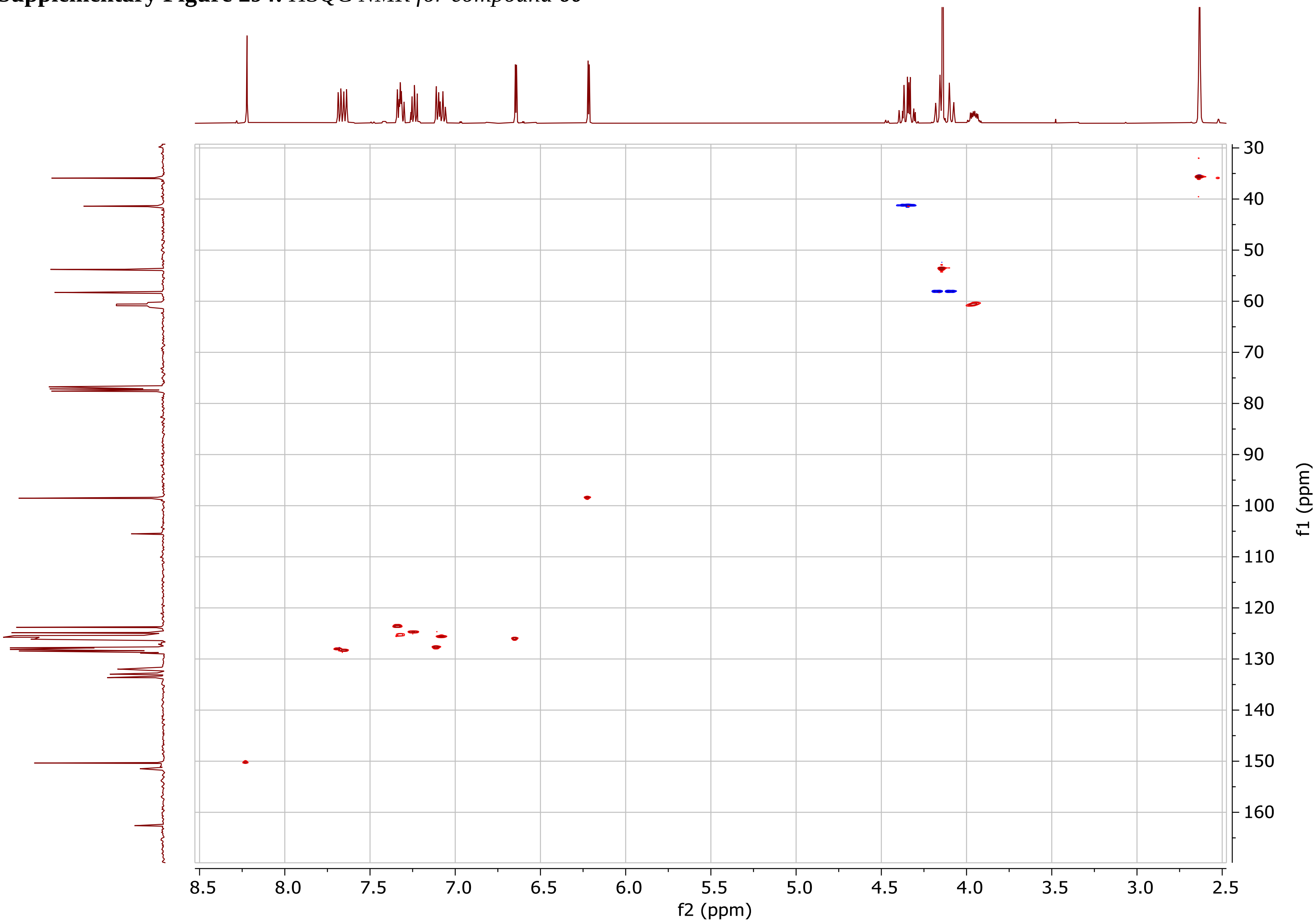

Supplementary Figure 295: *HMBC NMR for compound* ***60***

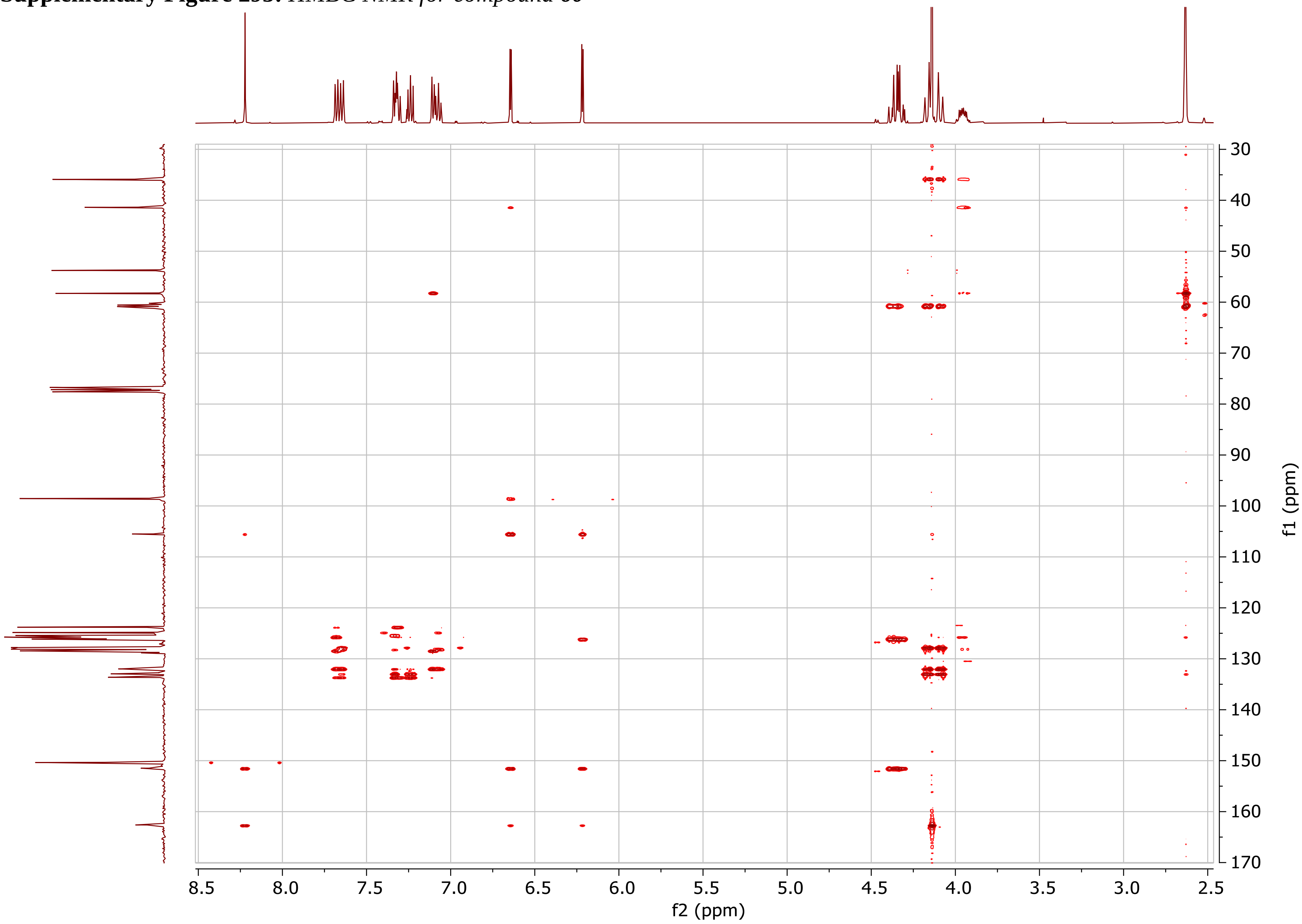

Supplementary Figure 296: *IR for compound* ***60***

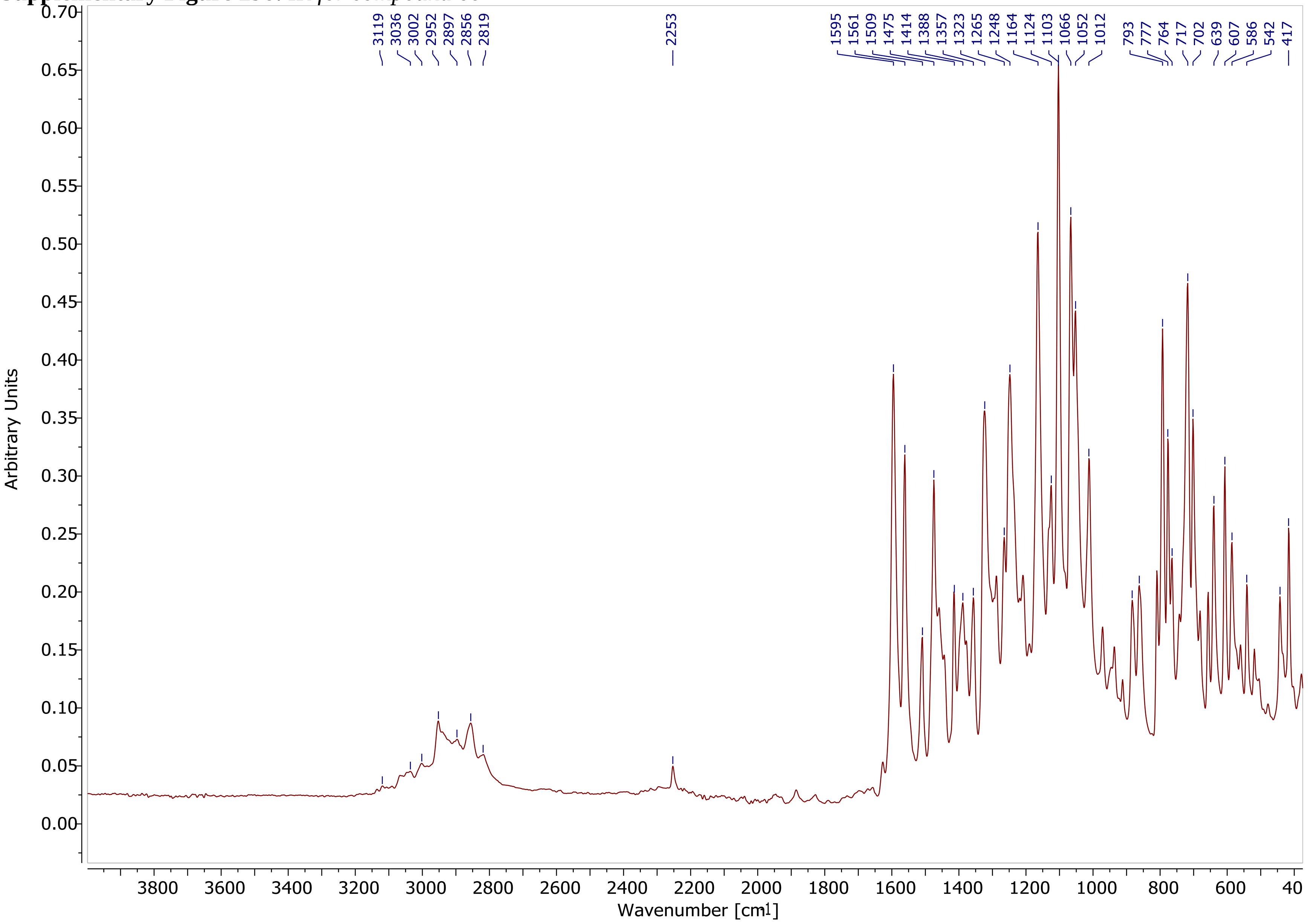

Supplementary Figure 297: *^{1}H-NMR for compound **61***

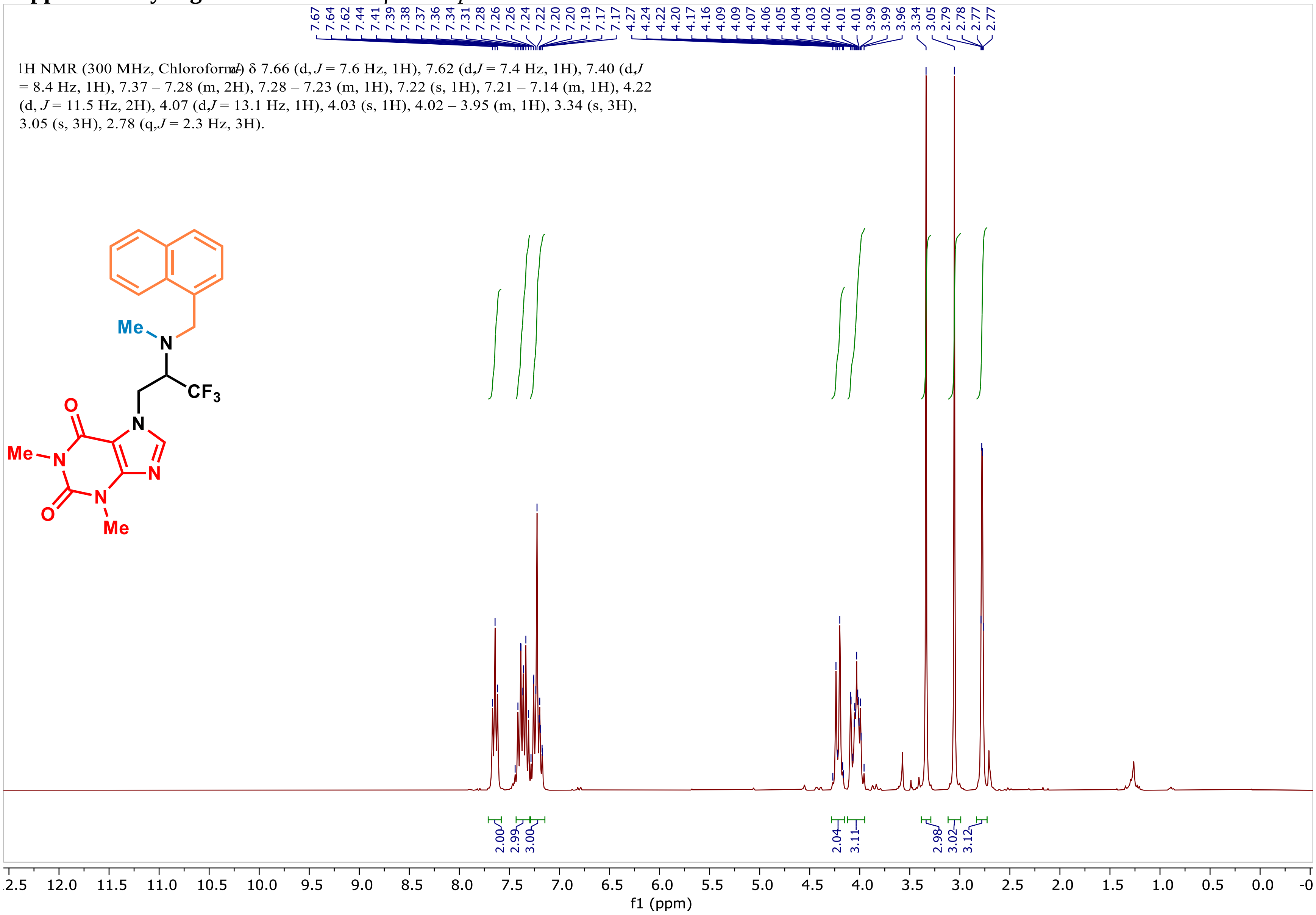

Supplementary Figure 298: *COSY NMR for compound **61***

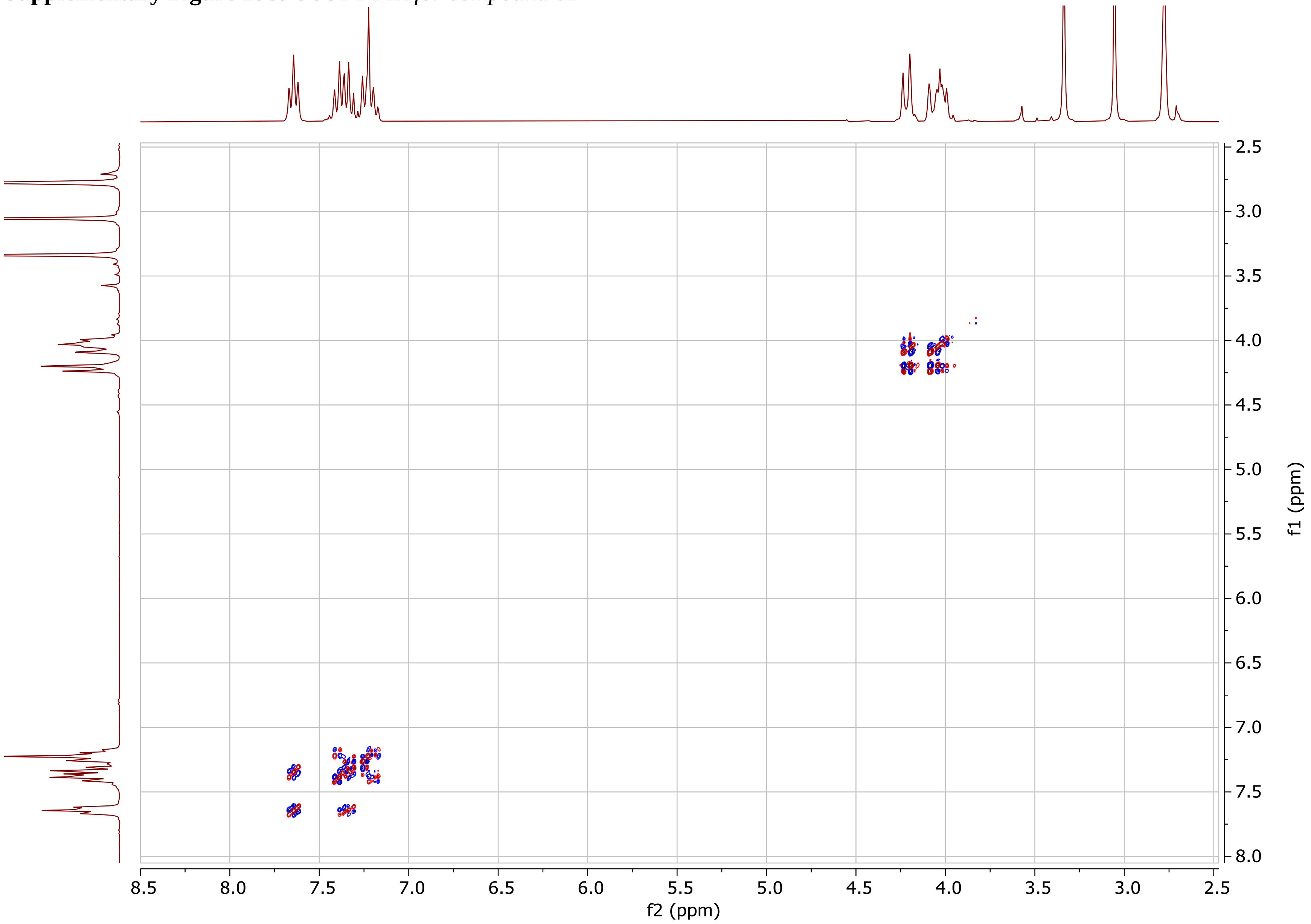

Supplementary Figure 299: *^{19}F NMR for compound **61***

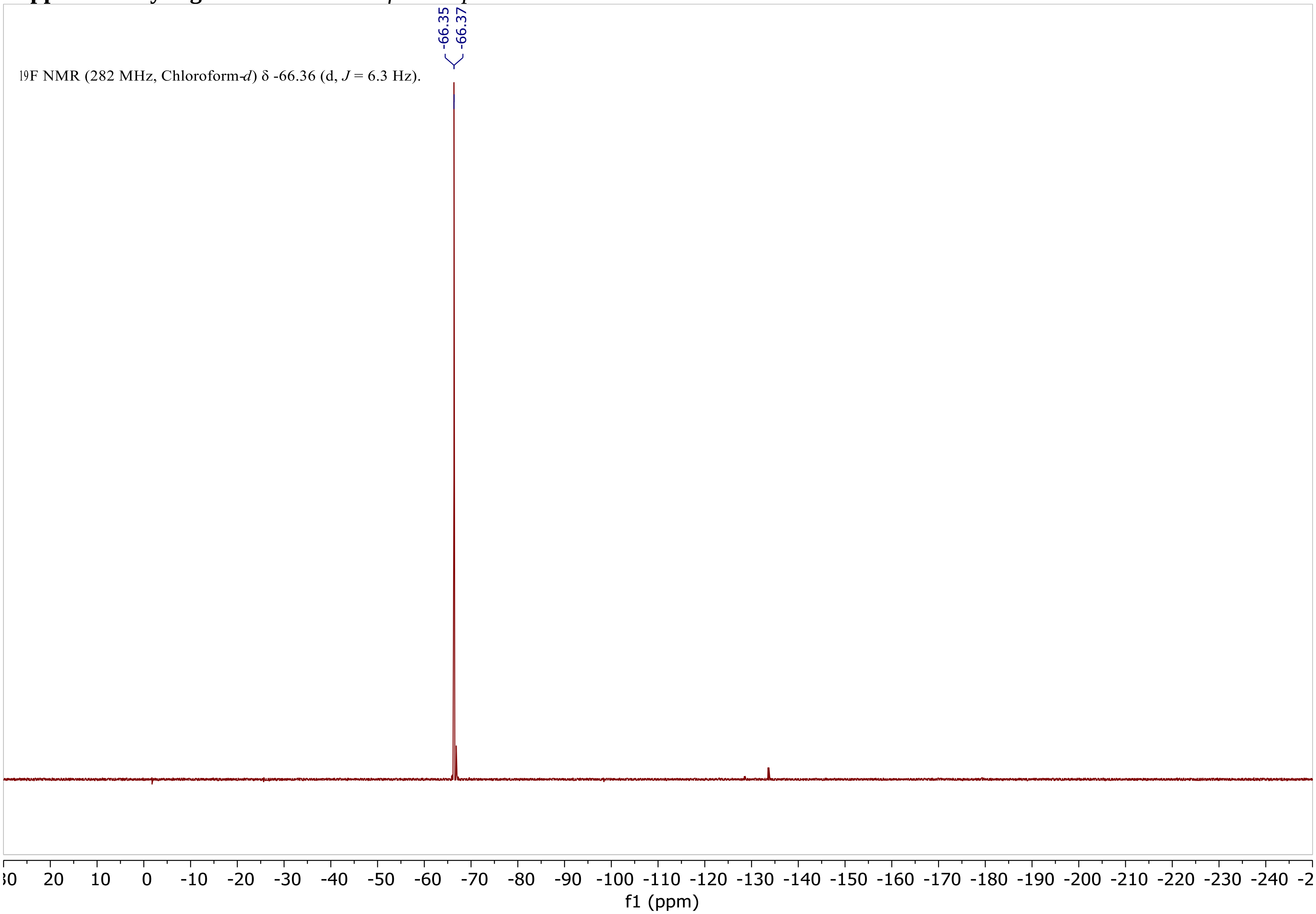

Supplementary Figure 300: *^{13}C NMR for compound* ***61***

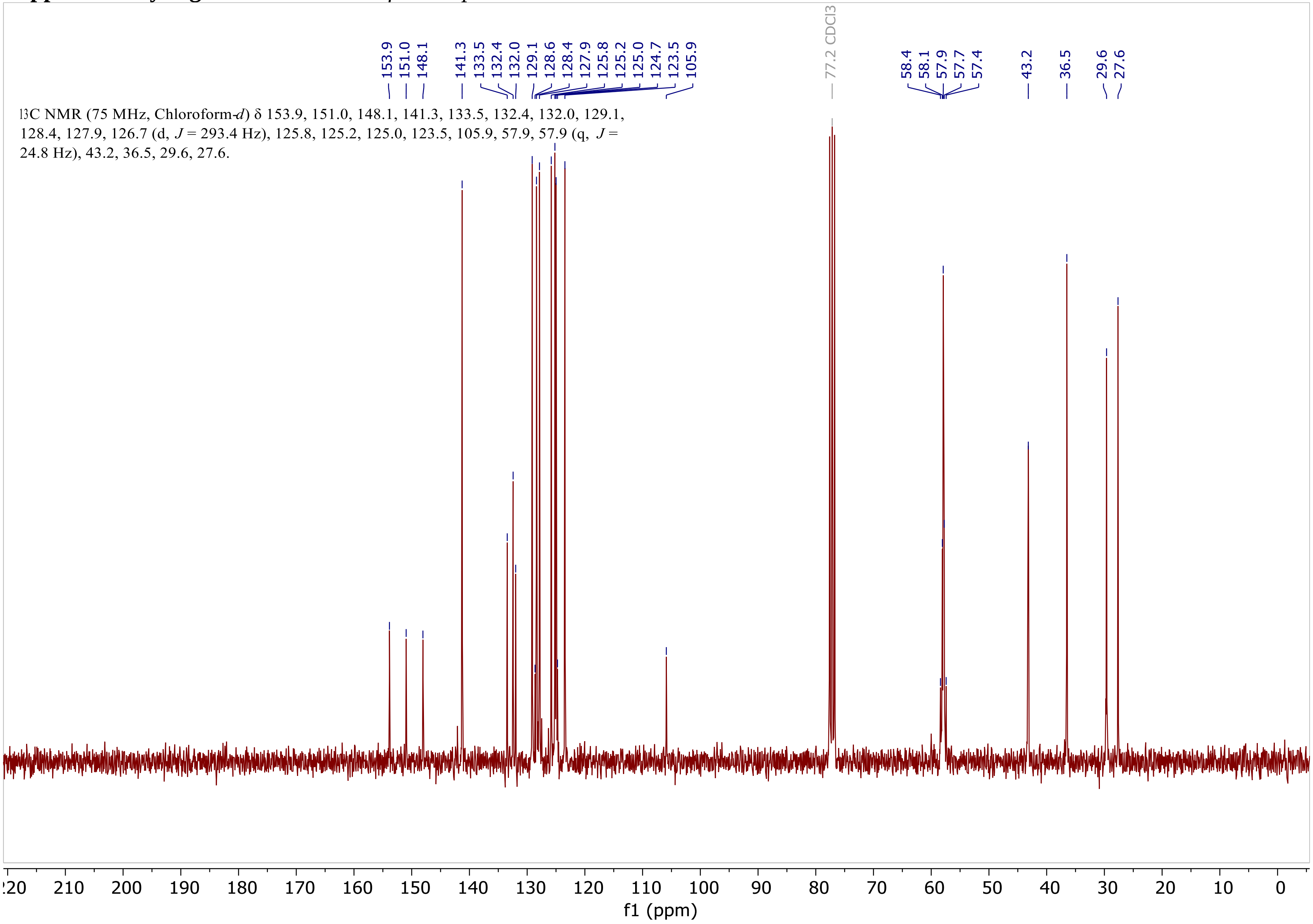

Supplementary Figure 301: *HSQC NMR for compound* ***61***

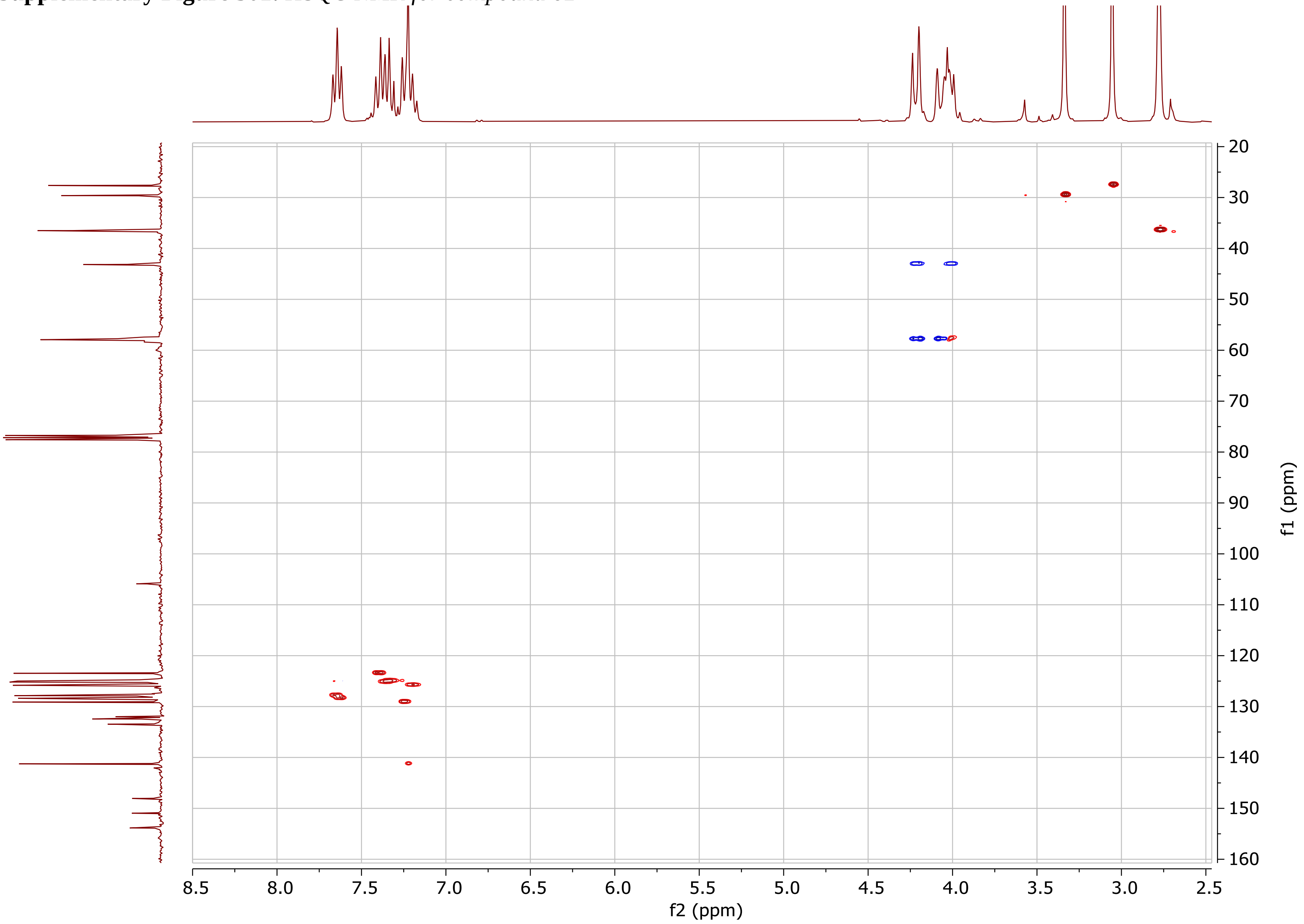

Supplementary Figure 302: *HMBC NMR for compound **61***

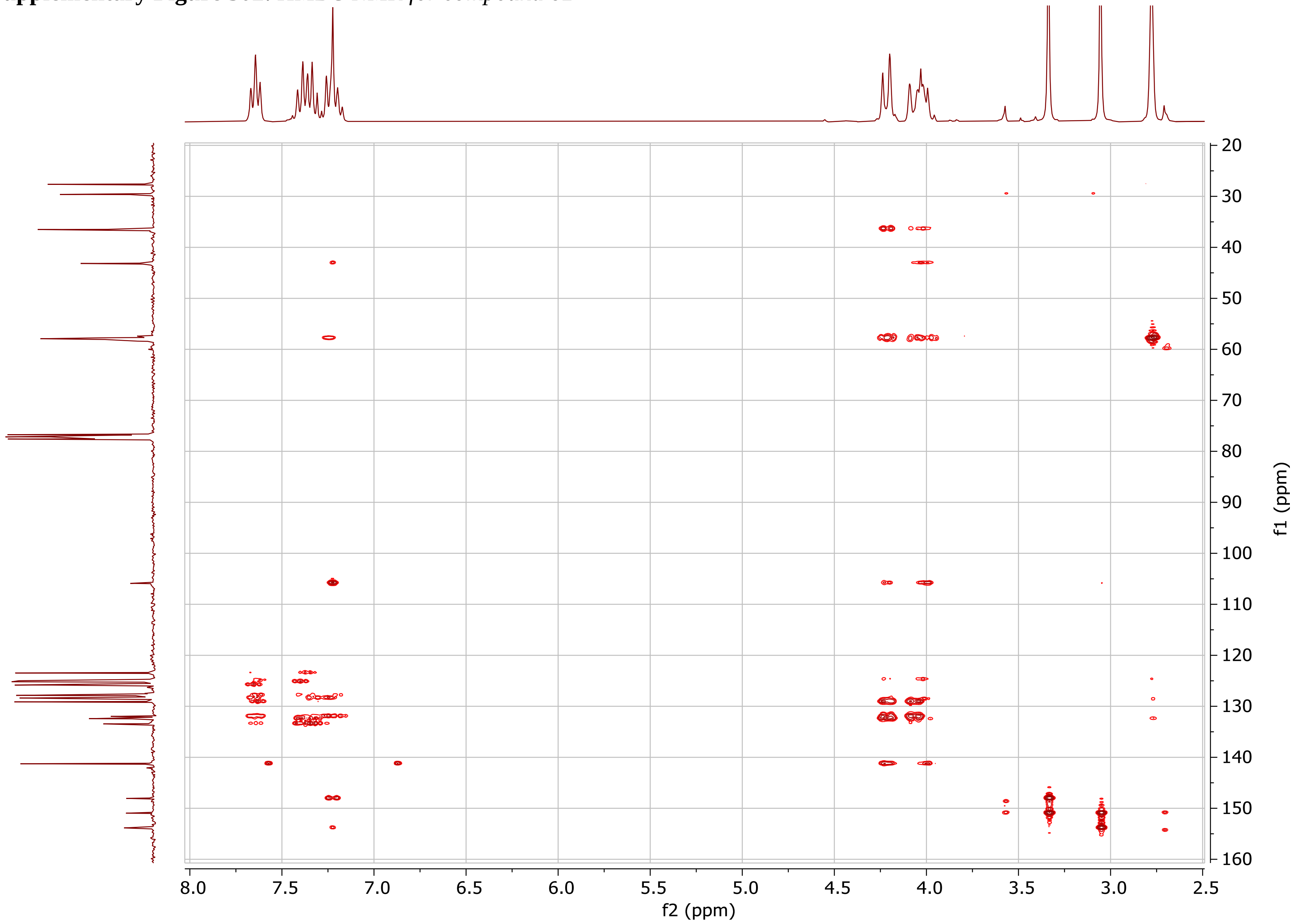

Supplementary Figure 303: *NOESY NMR for compound* ***61***

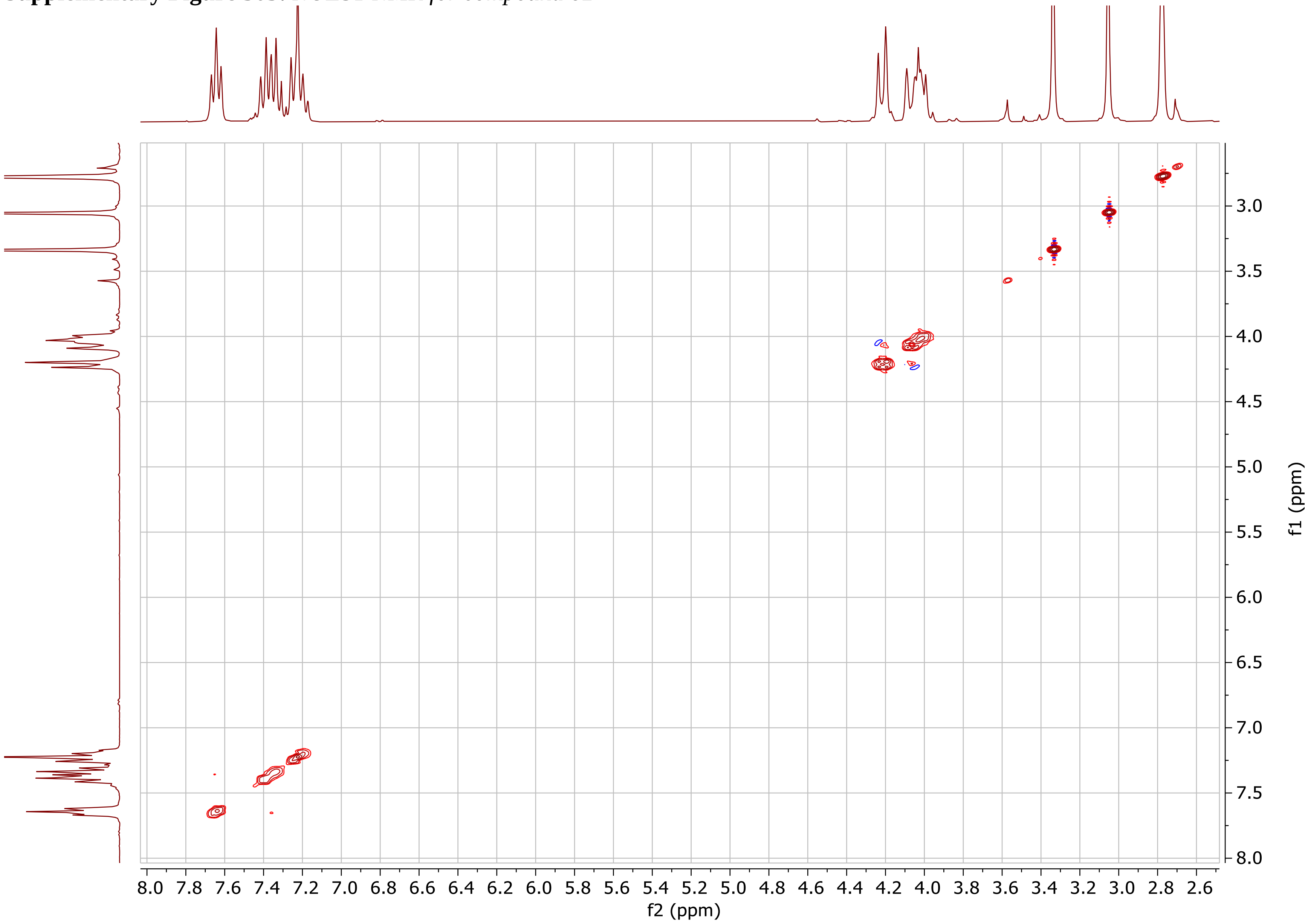

Supplementary Figure 304: *IR for compound **61***

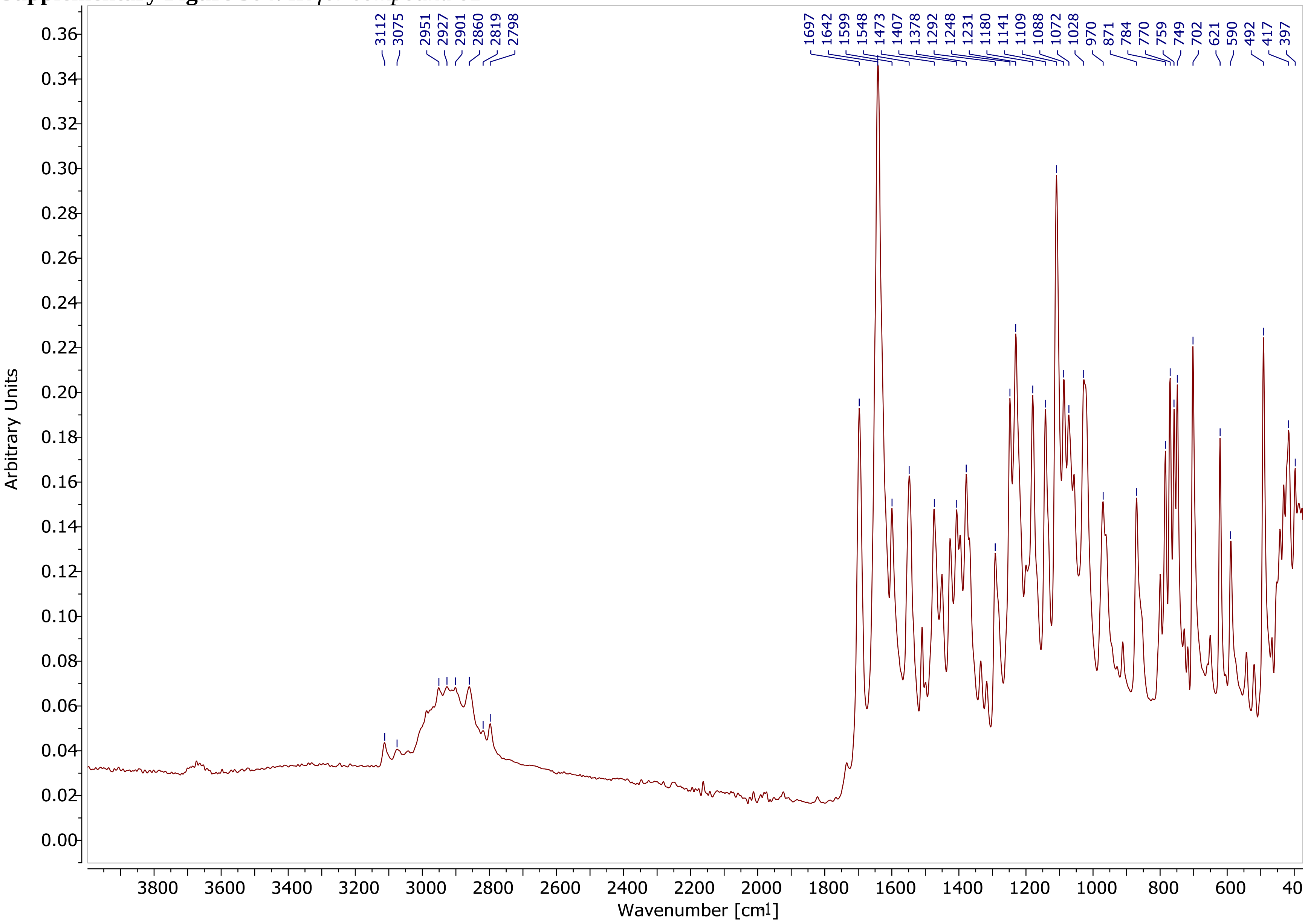

Supplementary Figure 305: *^{1}H-NMR for compound* ***62***

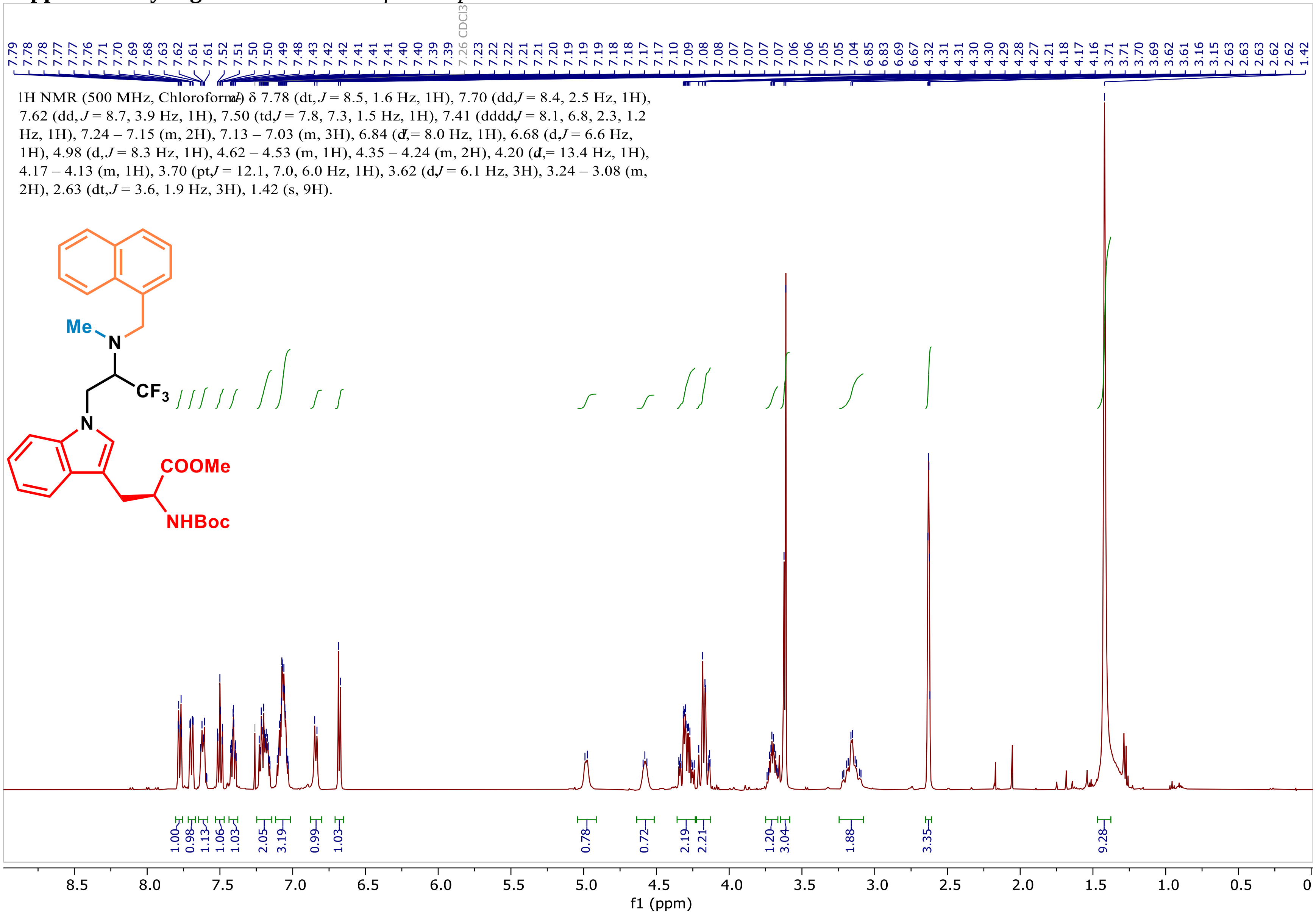

Supplementary Figure 306: *COSY NMR for compound* ***62***

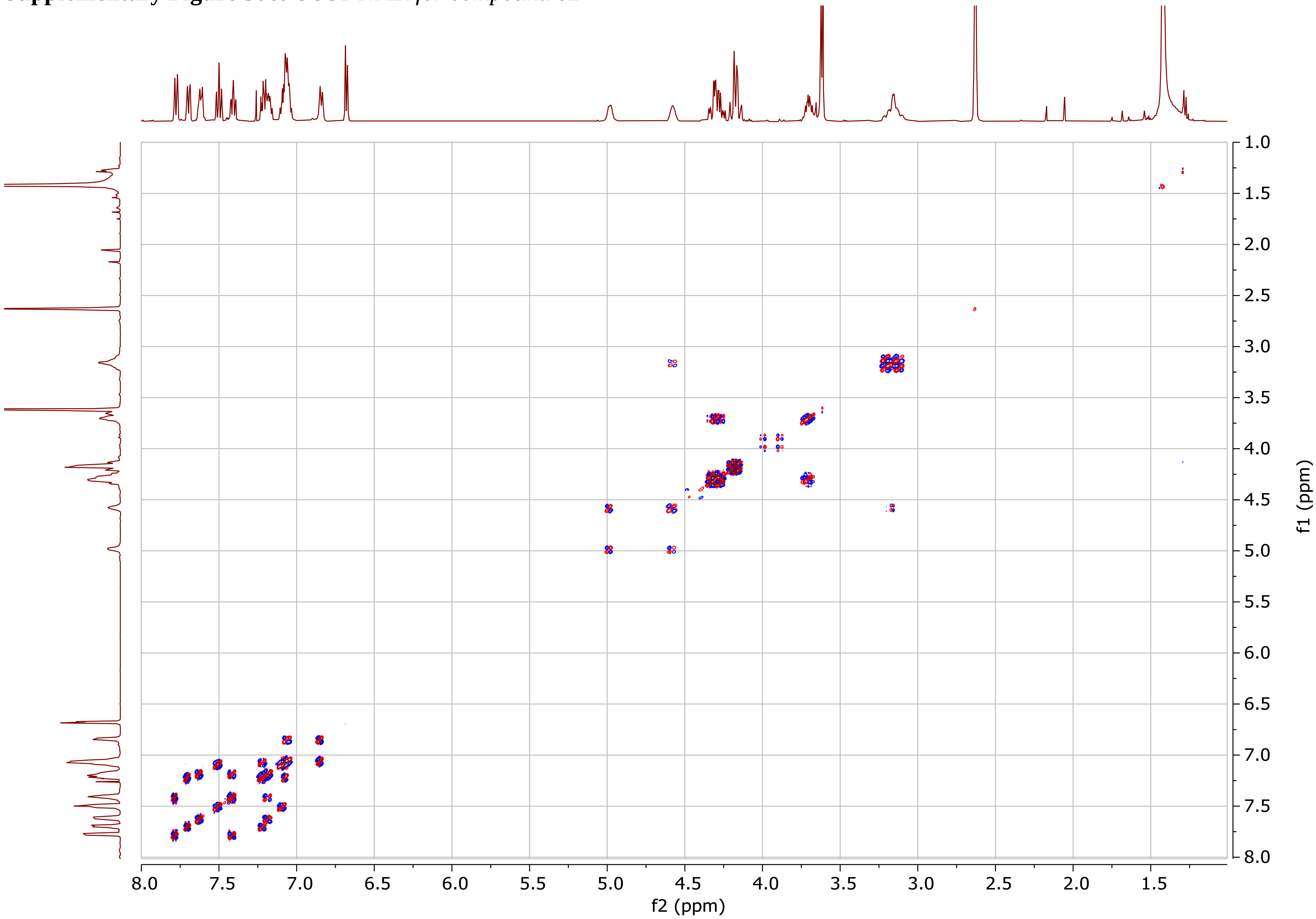

Supplementary Figure 307: *^{19}F NMR for compound* ***62***

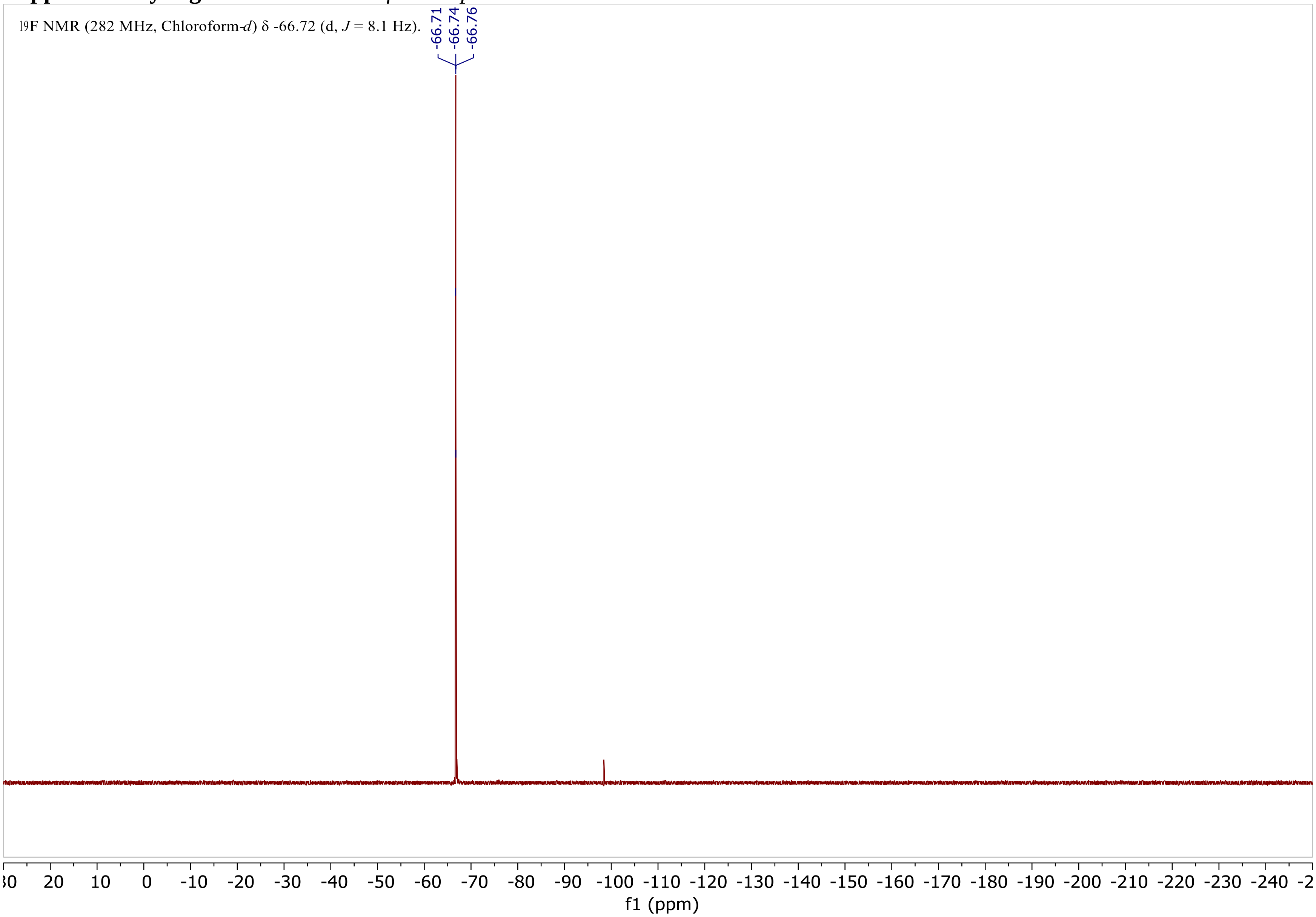

Supplementary Figure 308: *^{13}C NMR for compound **62***

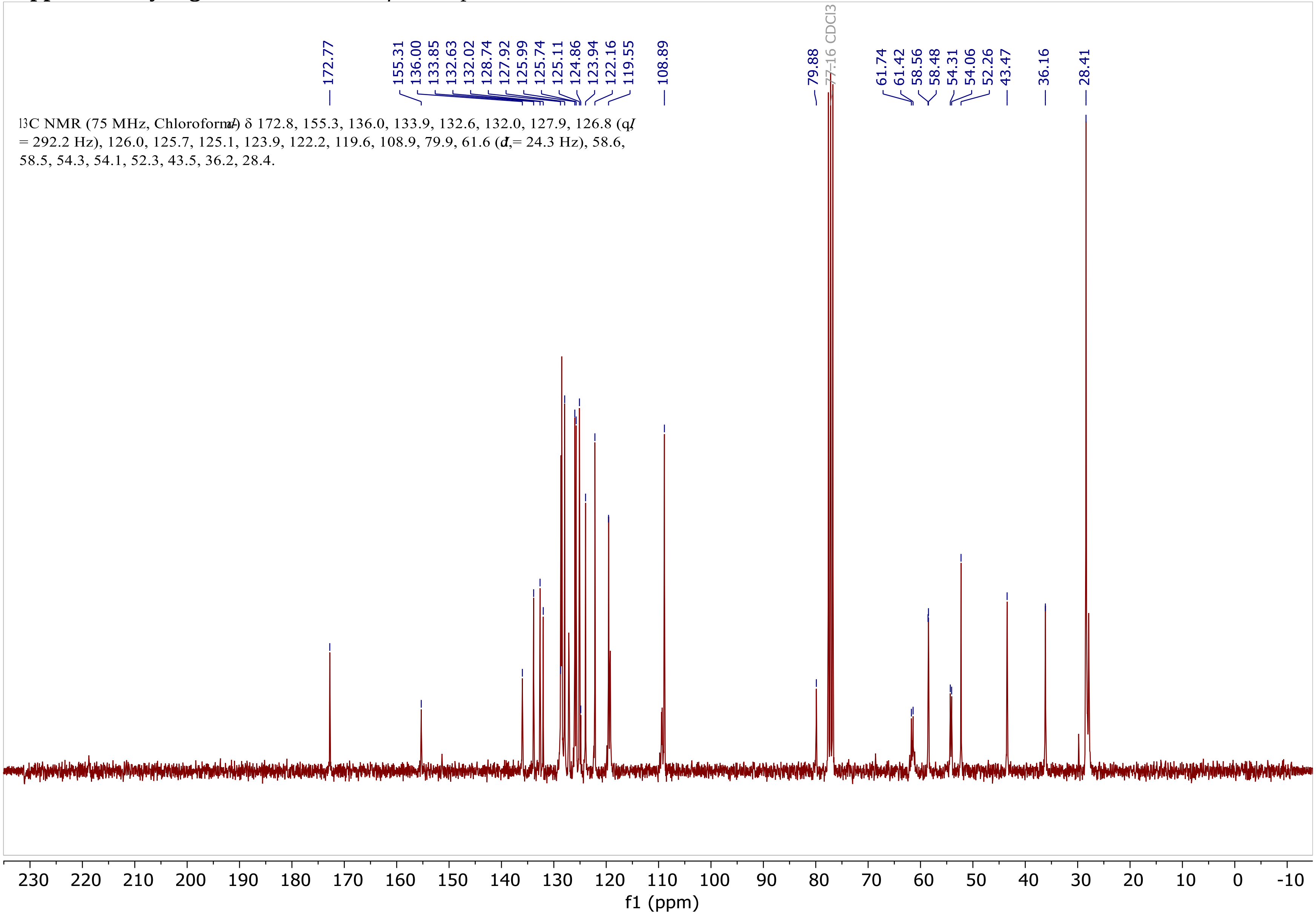

Supplementary Figure 309: *HSQC NMR for compound **62***

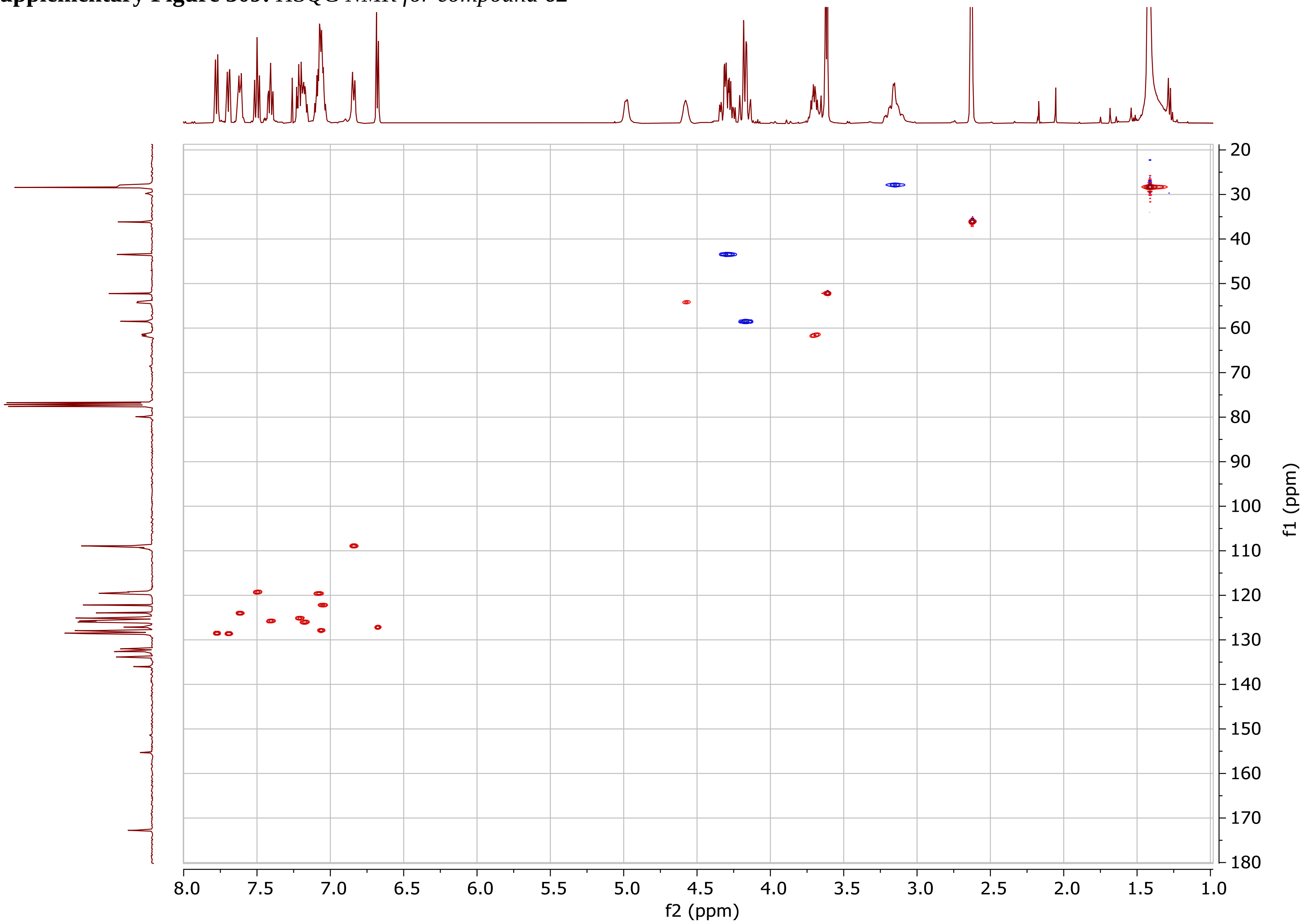

Supplementary Figure 310: *HMBC NMR for compound **62***

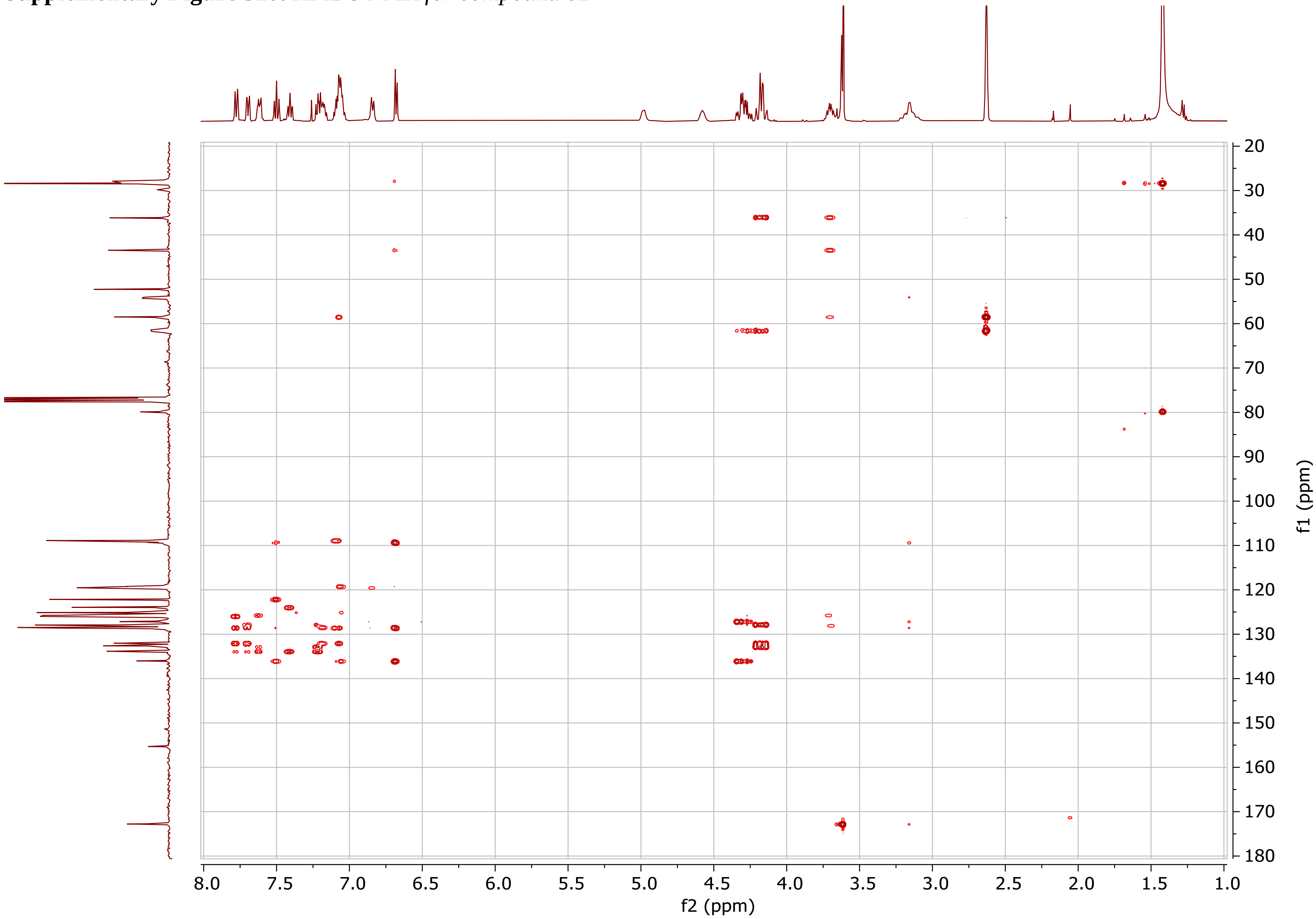

Supplementary Figure 311: *IR for compound **62***

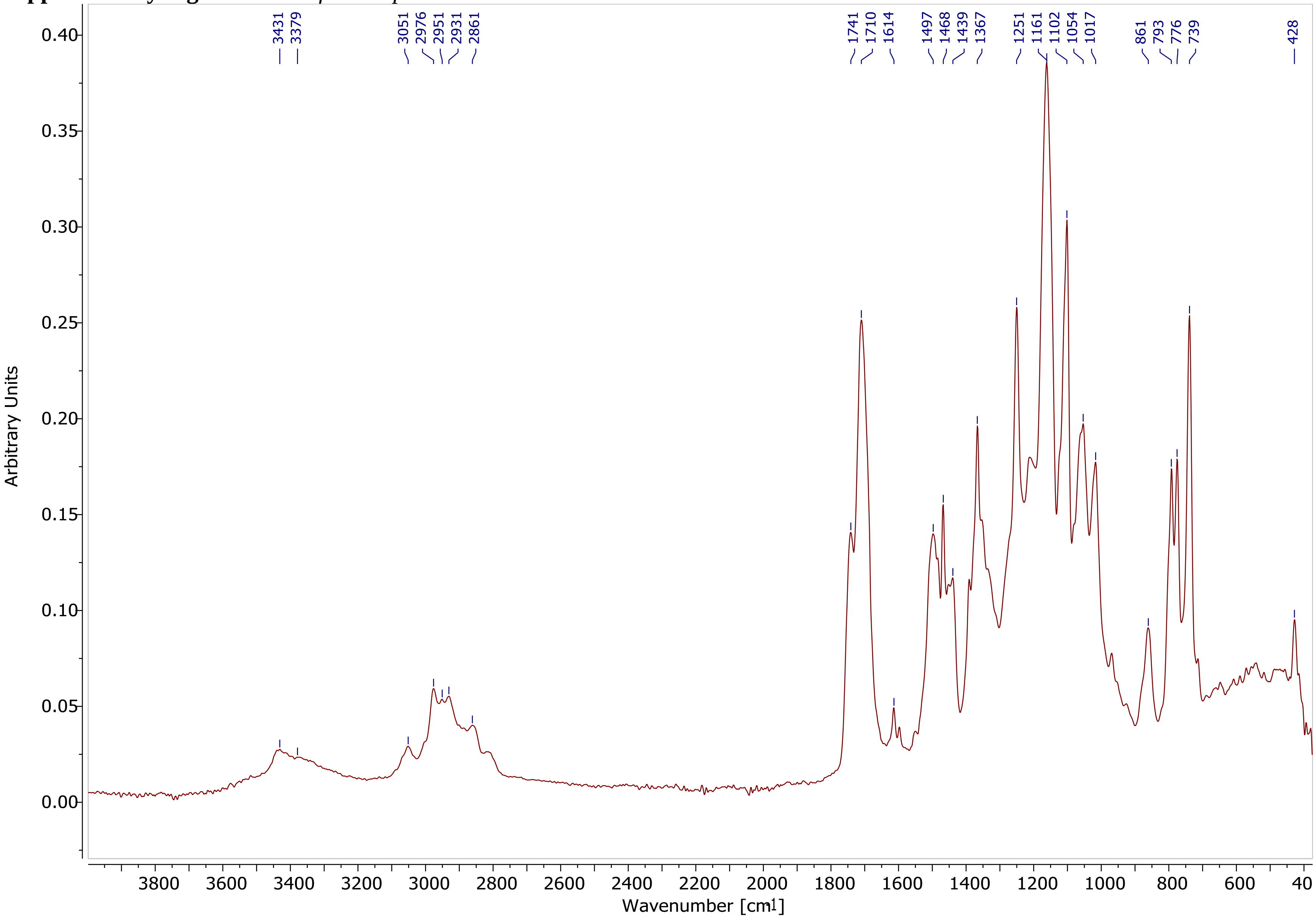

Supplementary Figure 312: *^{1}H-NMR for compound* ***63***

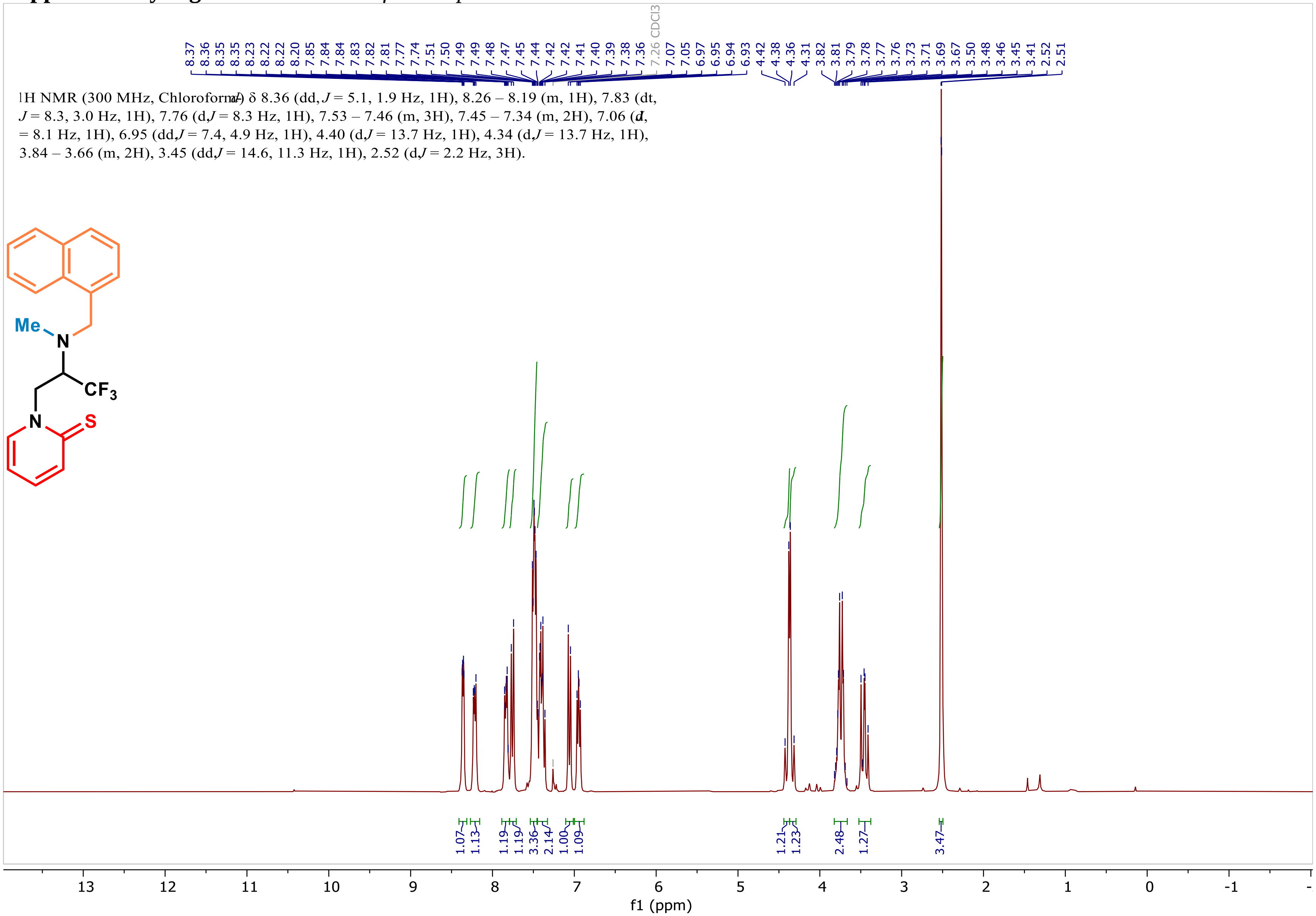

Supplementary Figure 313: *^{19}F NMR for compound* ***63***

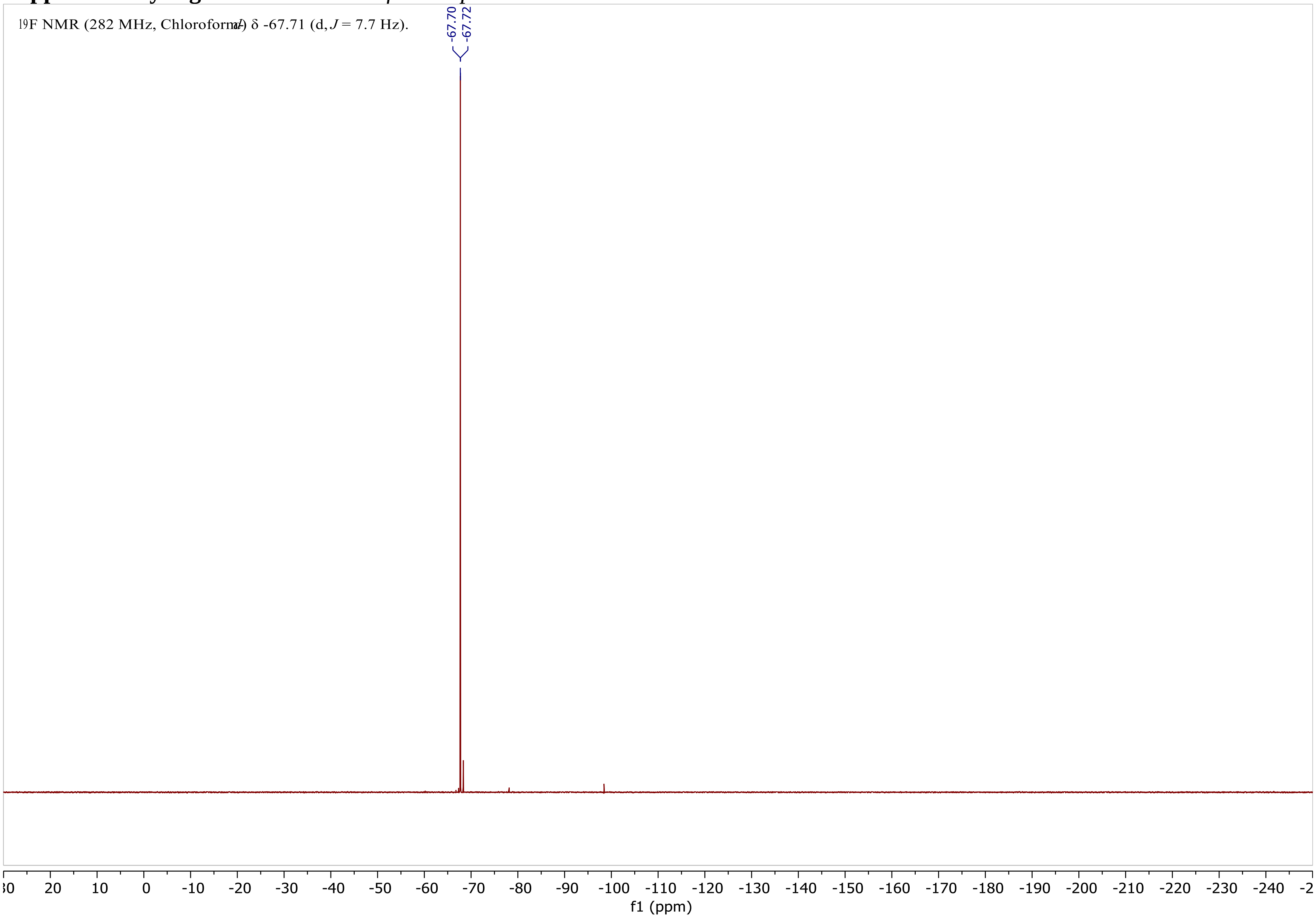

Supplementary Figure 314: *^{13}C NMR for compound* ***63***

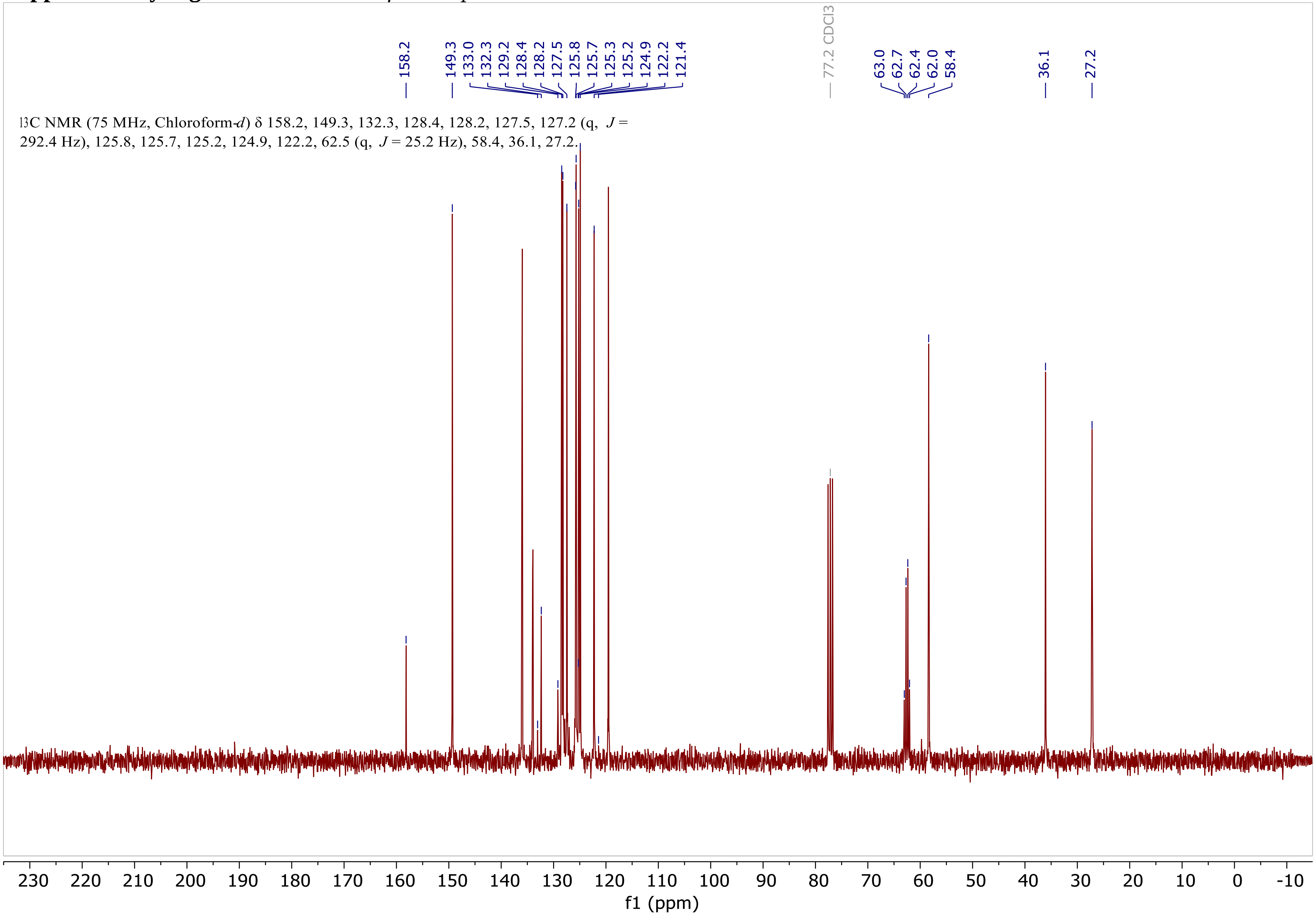

Supplementary Figure 315: *IR for compound* ***63***

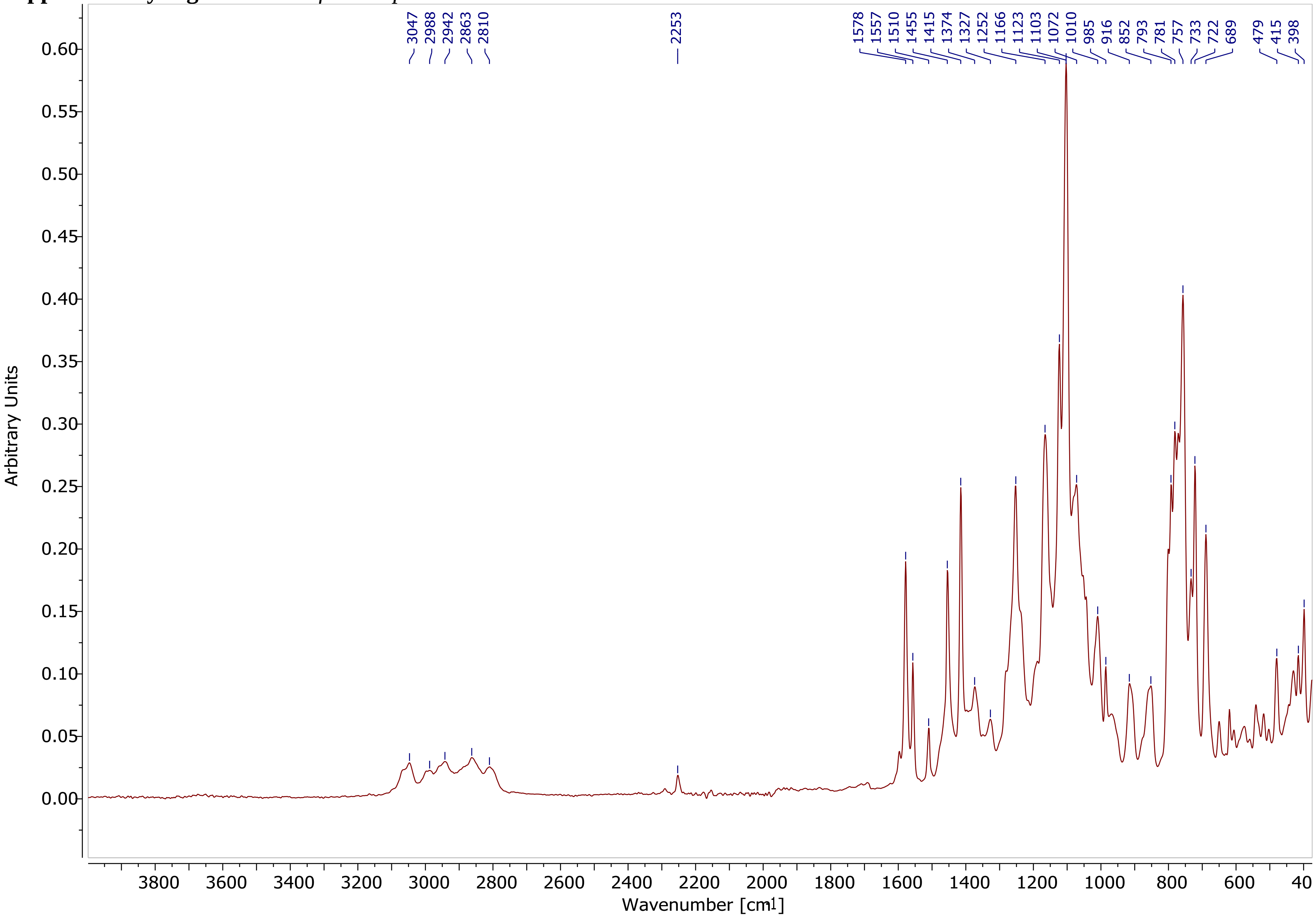

Supplementary Figure 316: *^{1}H-NMR for compound **64***

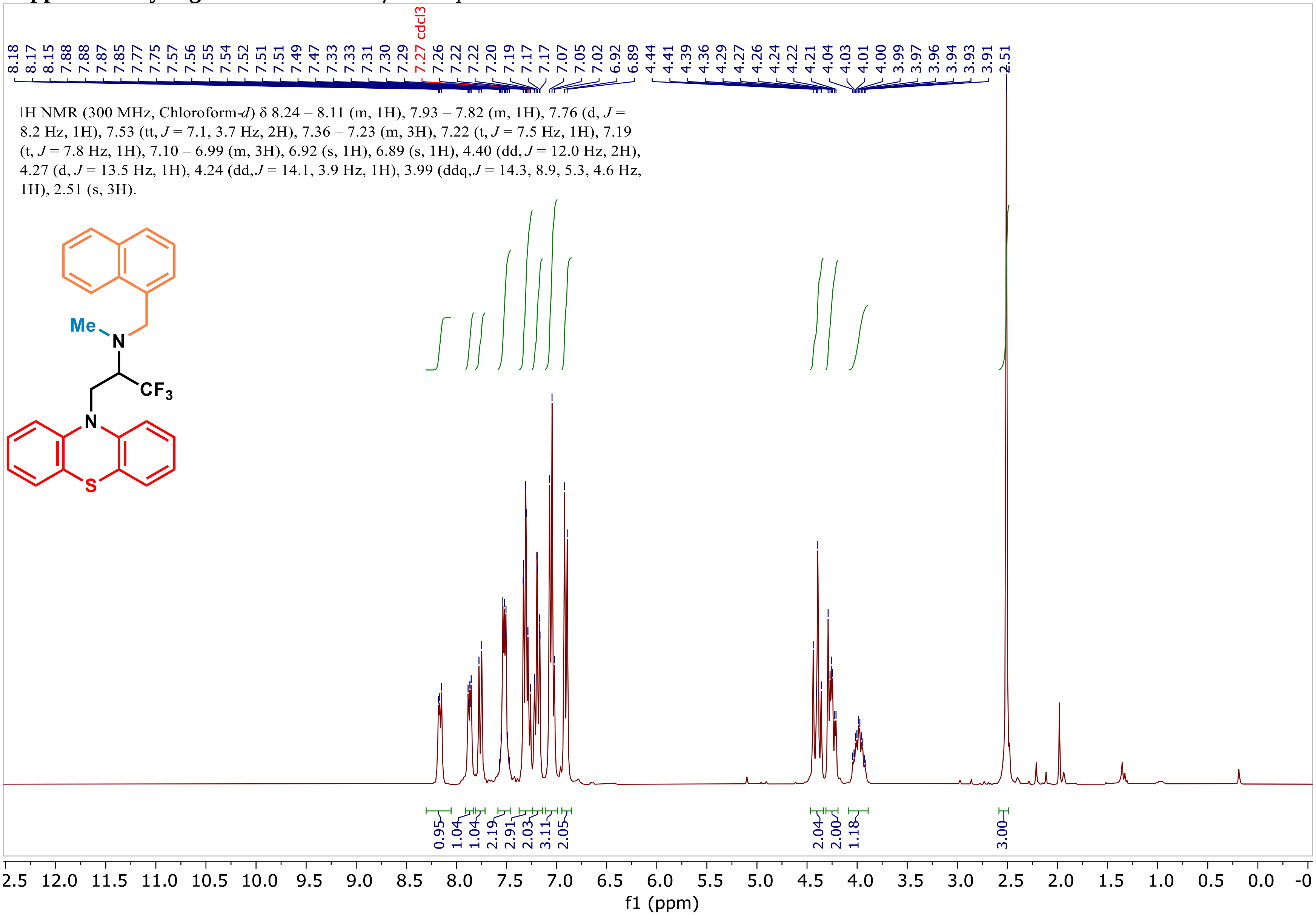

Supplementary Figure 317: *^{19}F NMR for compound* ***64***

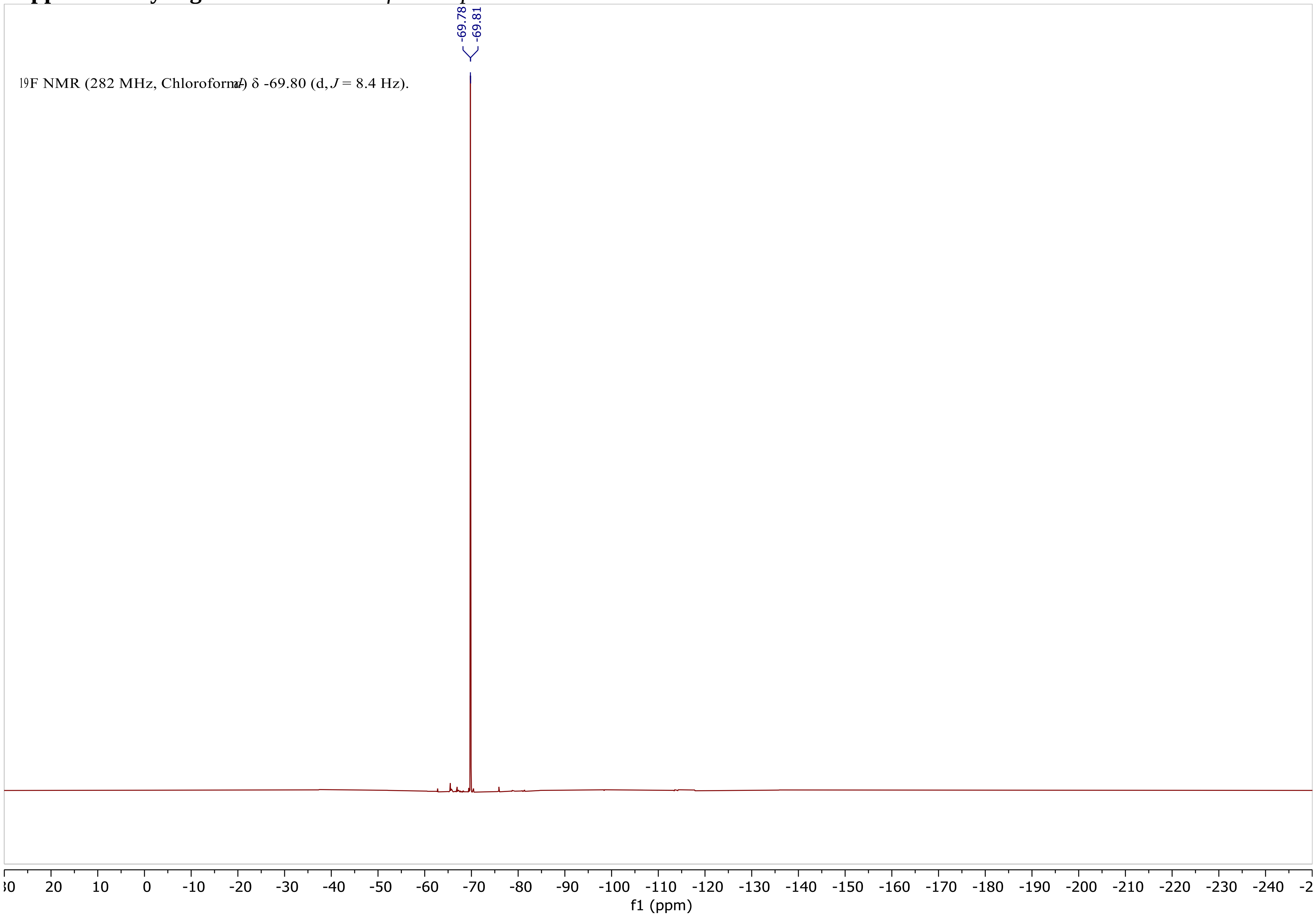

Supplementary Figure 318: *^{13}C NMR for compound* ***64***

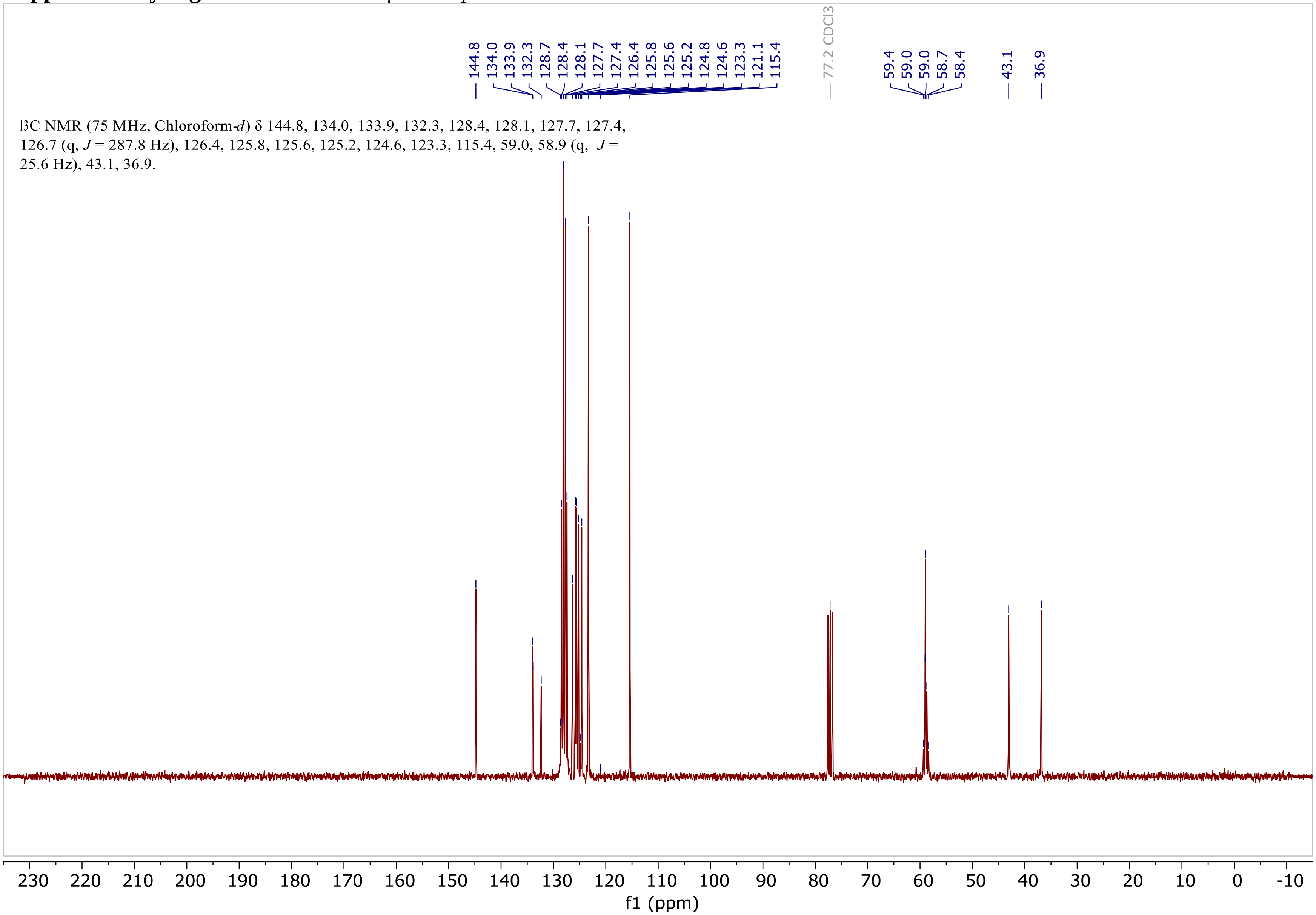

Supplementary Figure 319: *IR for compound* ***64***

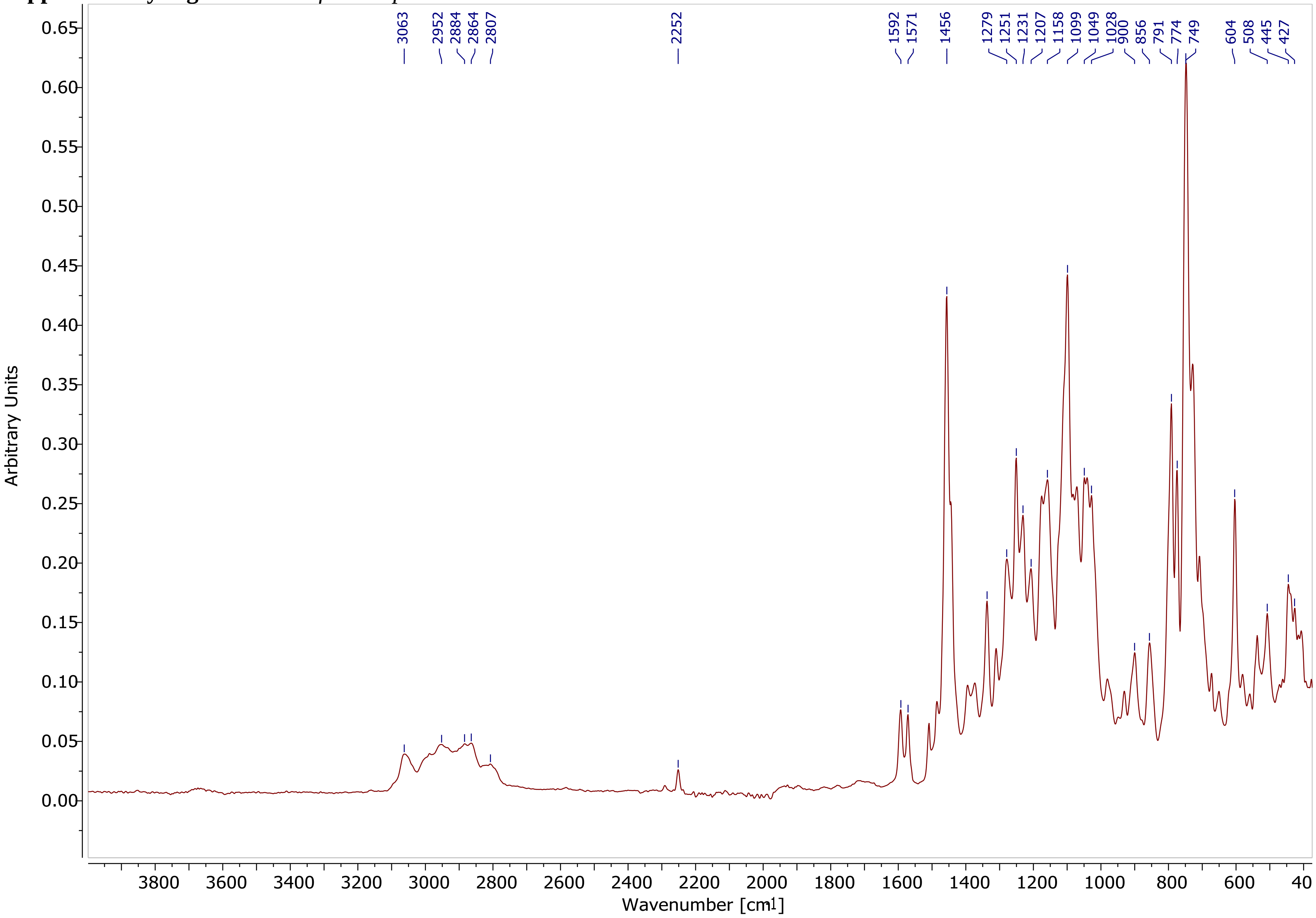

Supplementary Figure 320: *^{1}H-NMR for compound **65***

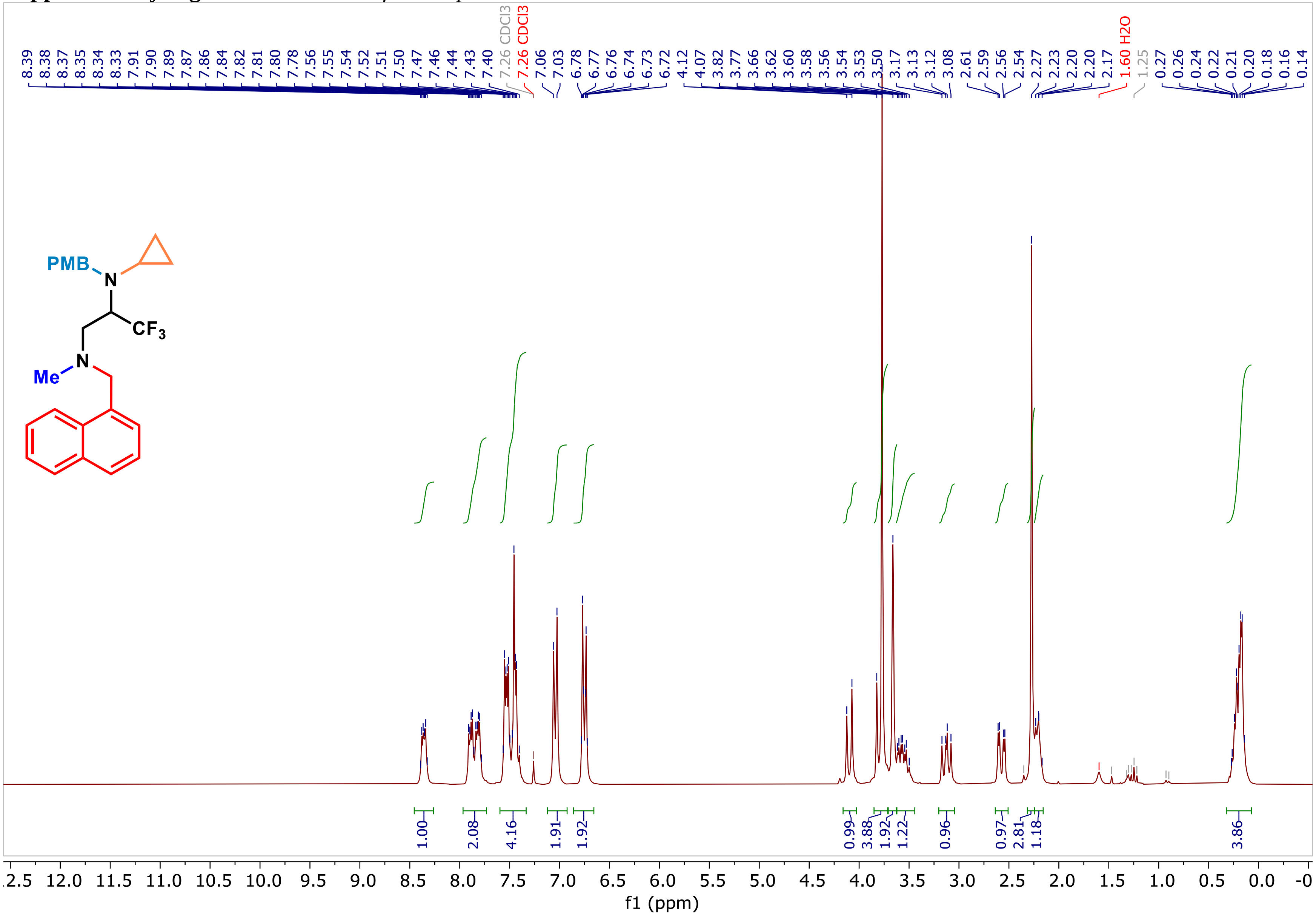

Supplementary Figure 321: *^{19}F NMR for compound* ***65***

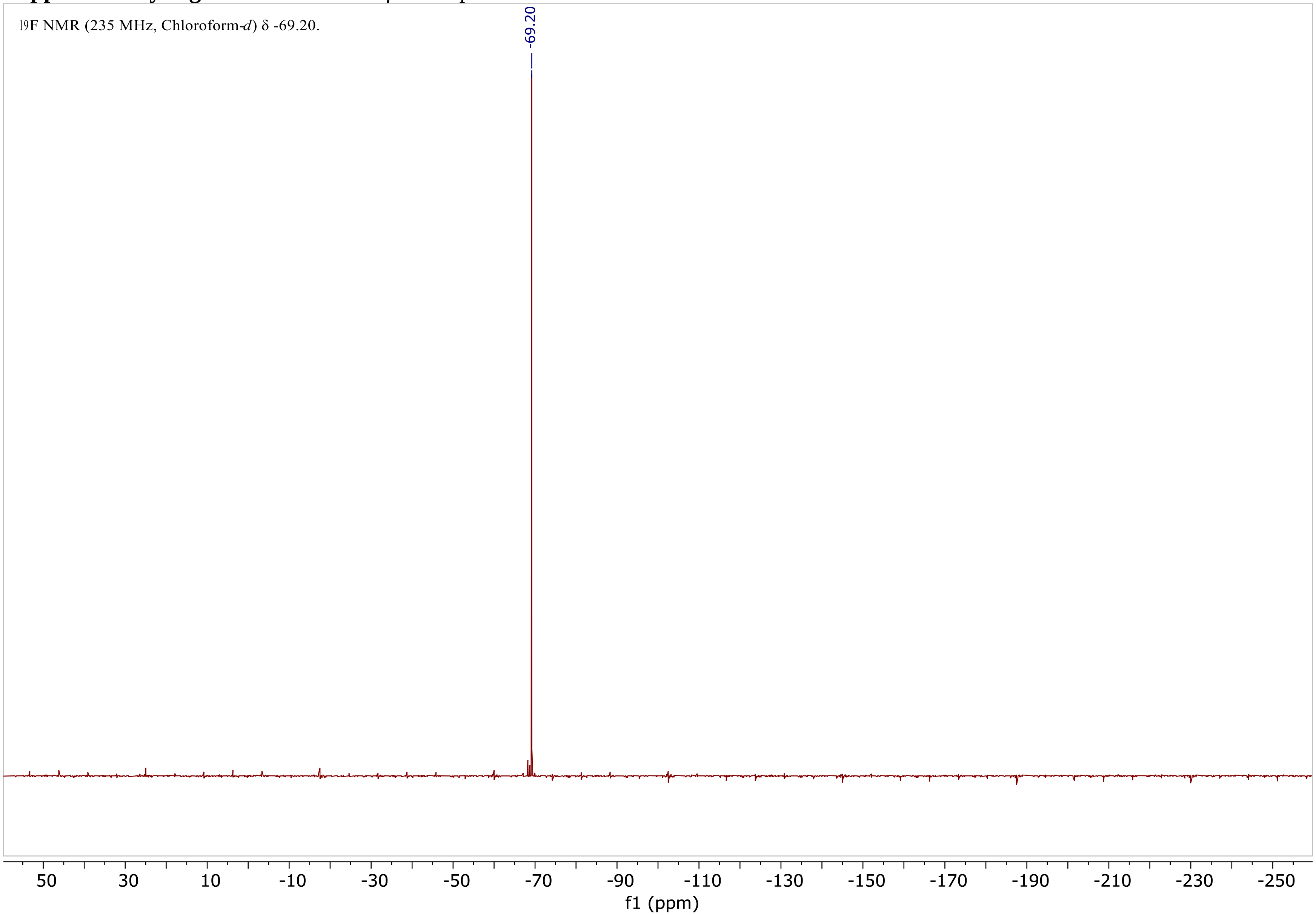

Supplementary Figure 322: ^{13}*C NMR for compound* ***65***

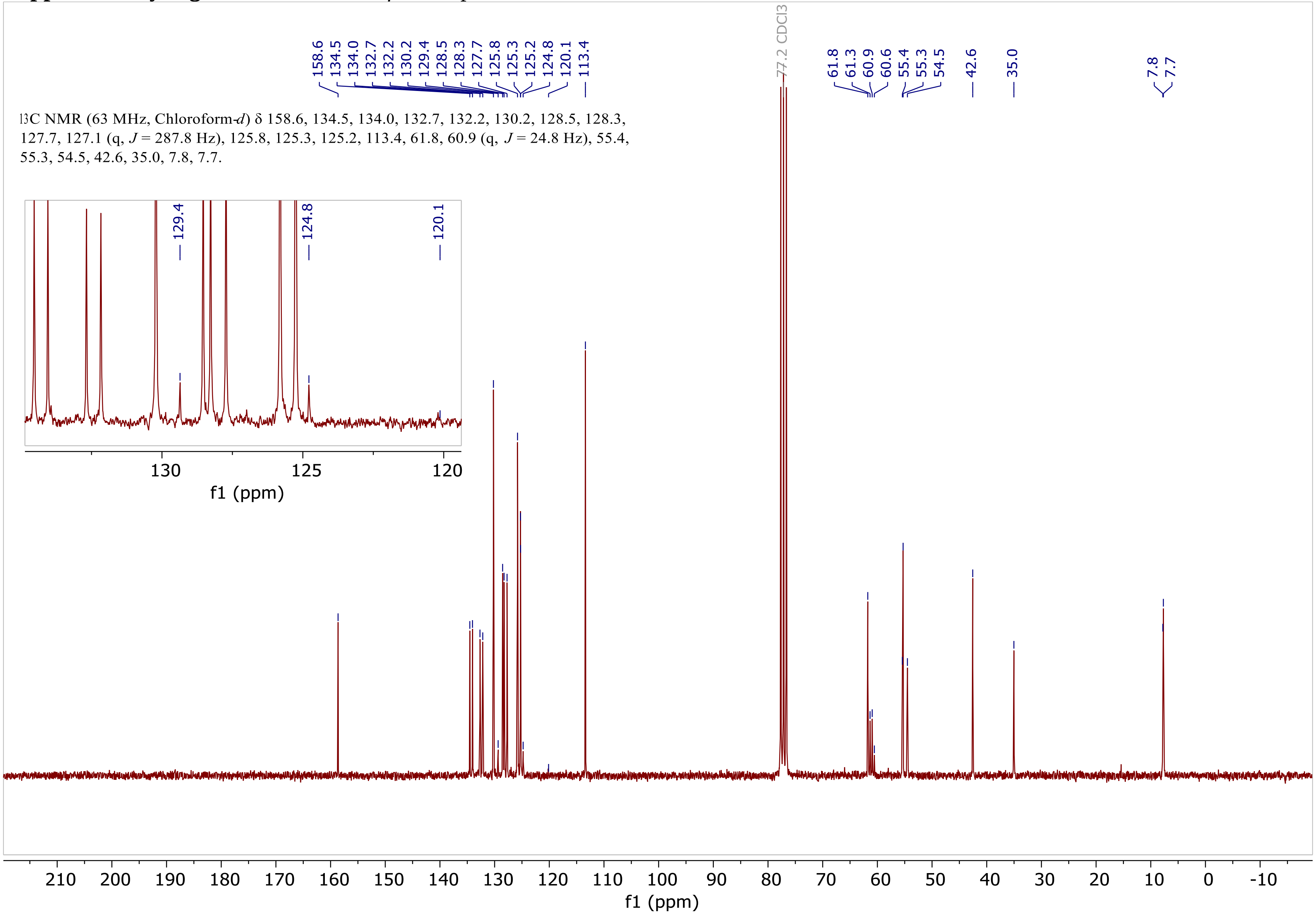

Supplementary Figure 323: *IR for compound **65***

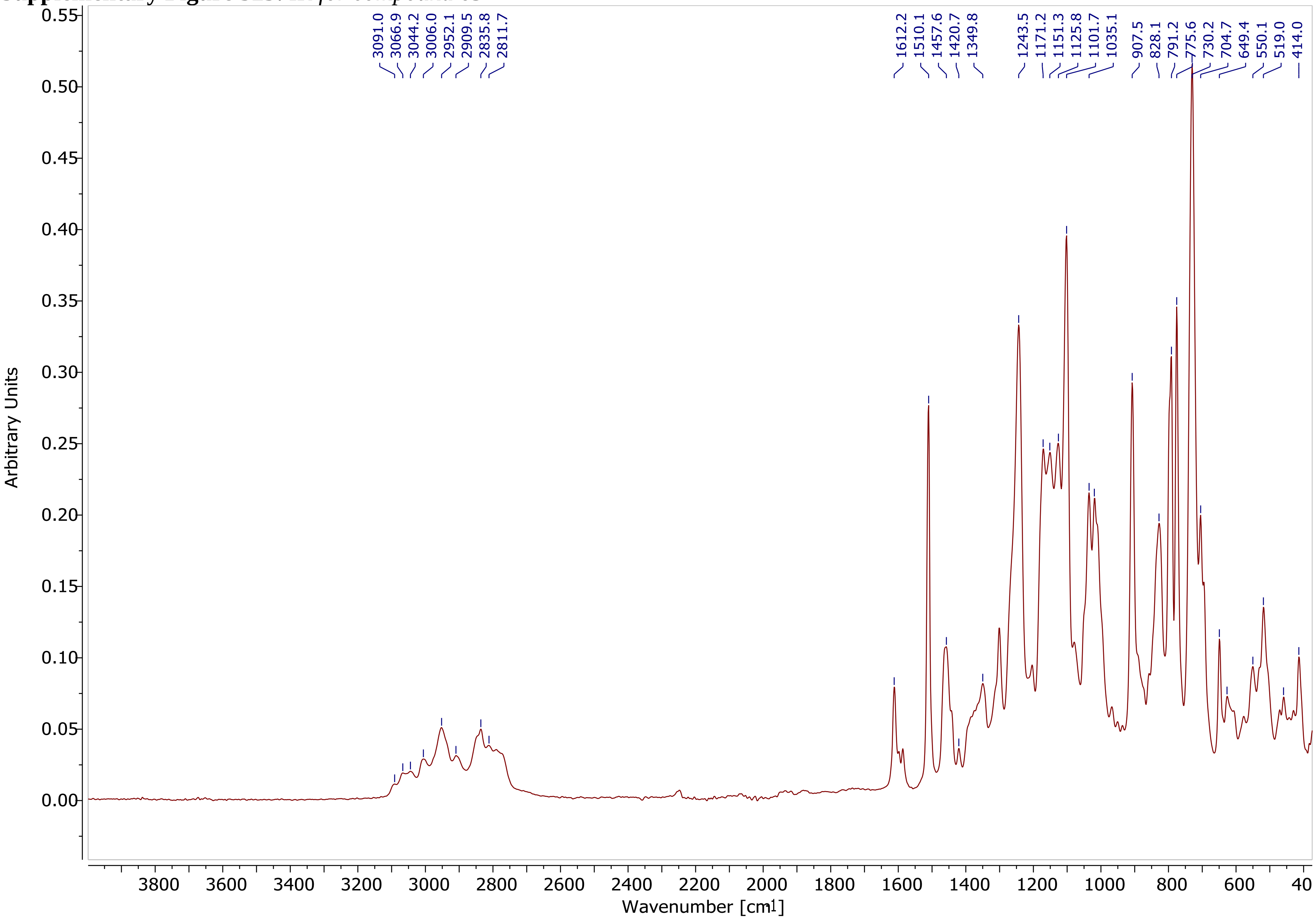

Supplementary Figure 324: *¹H-NMR for compound* ***66***

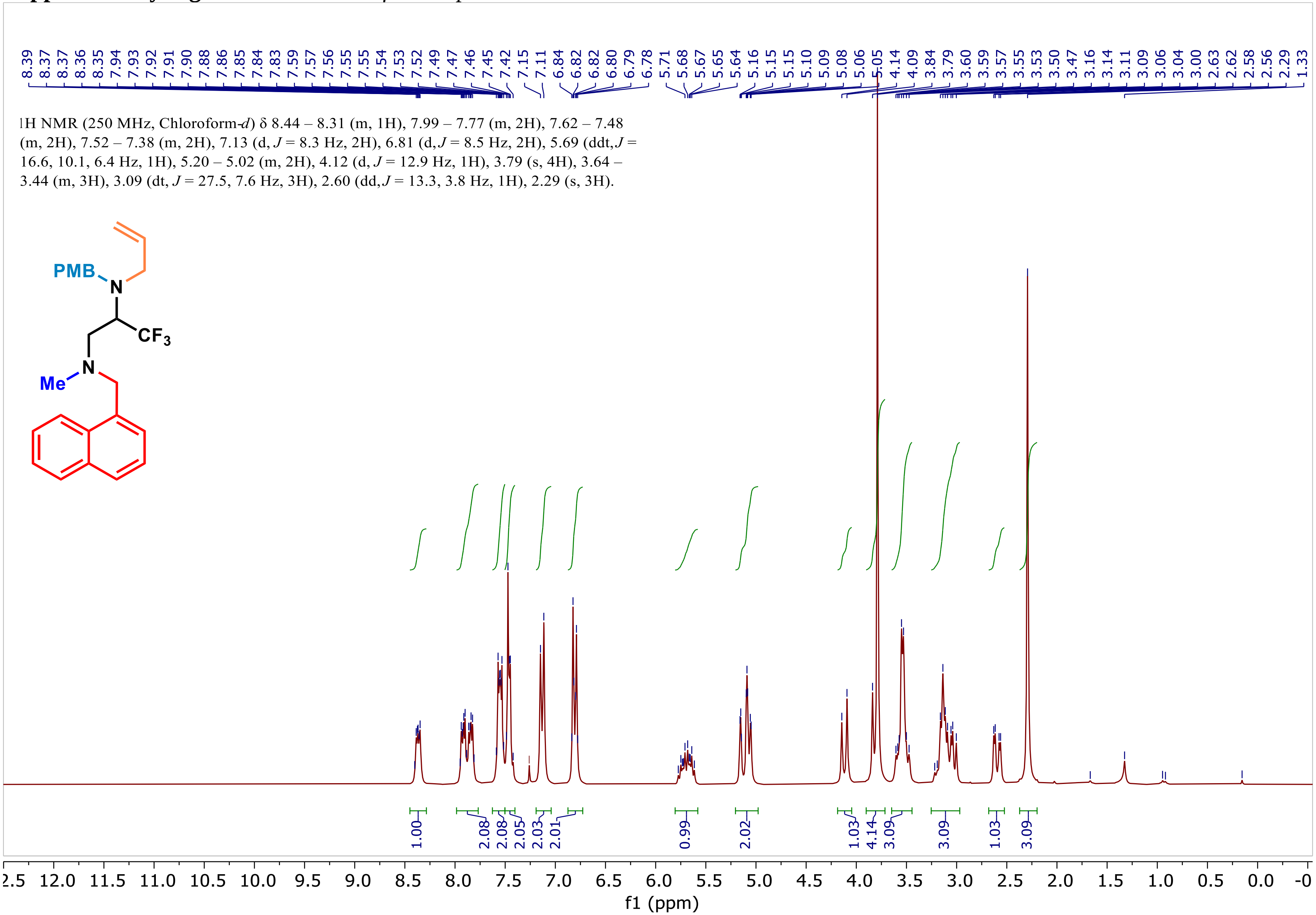

Supplementary Figure 325: ^{19}*F NMR for compound* ***66***

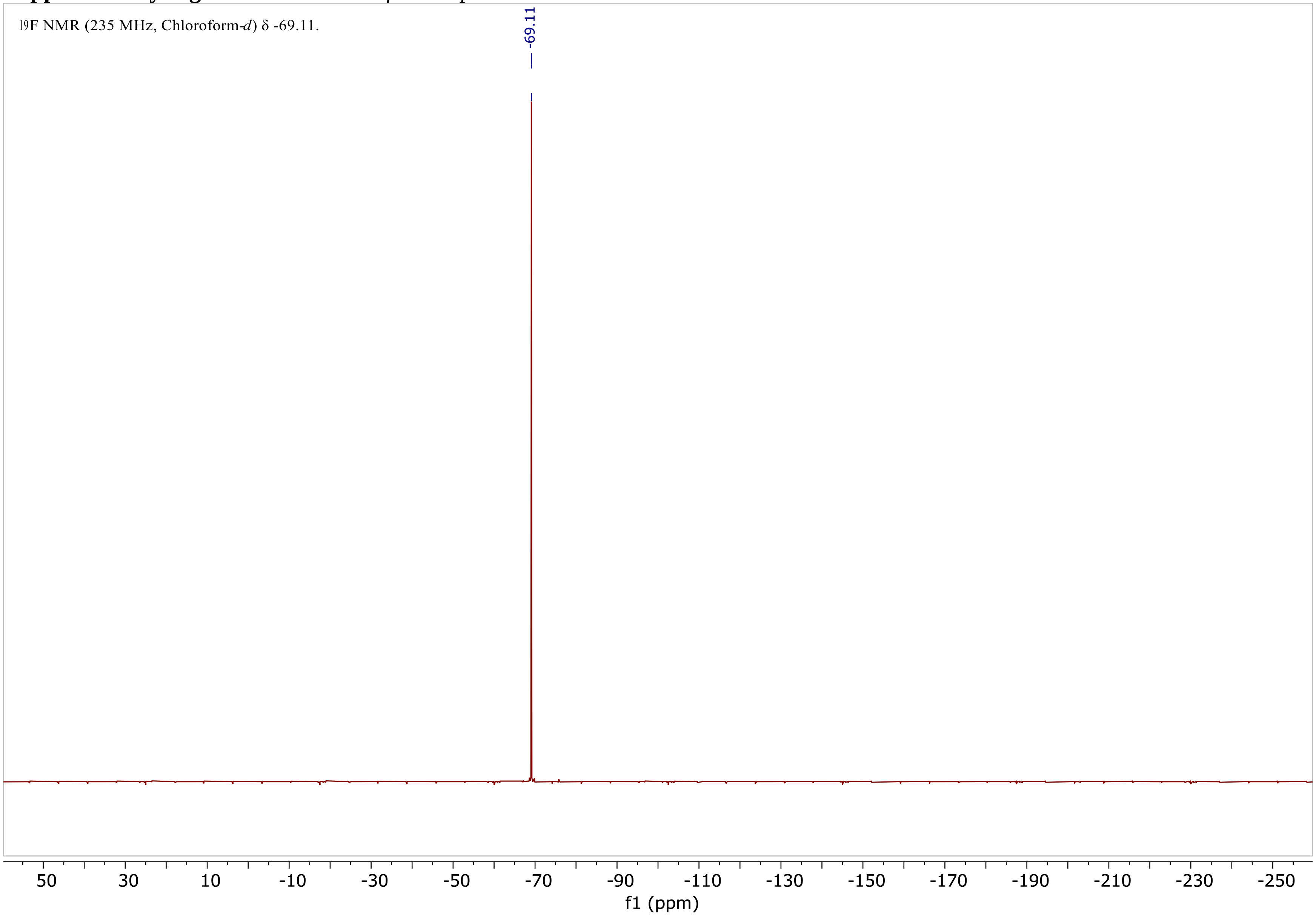

Supplementary Figure 326: *^{13}C NMR for compound* ***66***

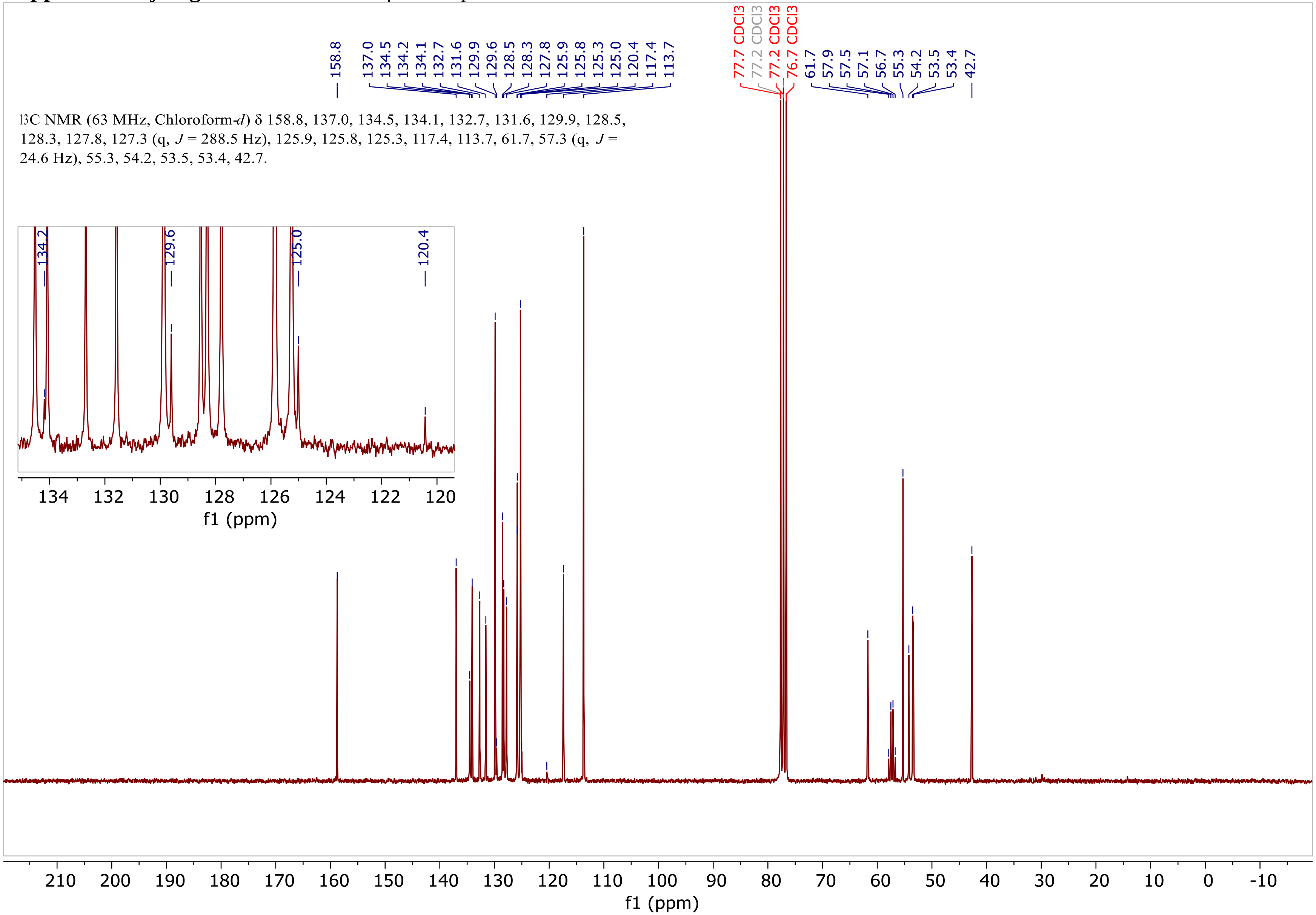

Supplementary Figure 327: *IR for compound **66***

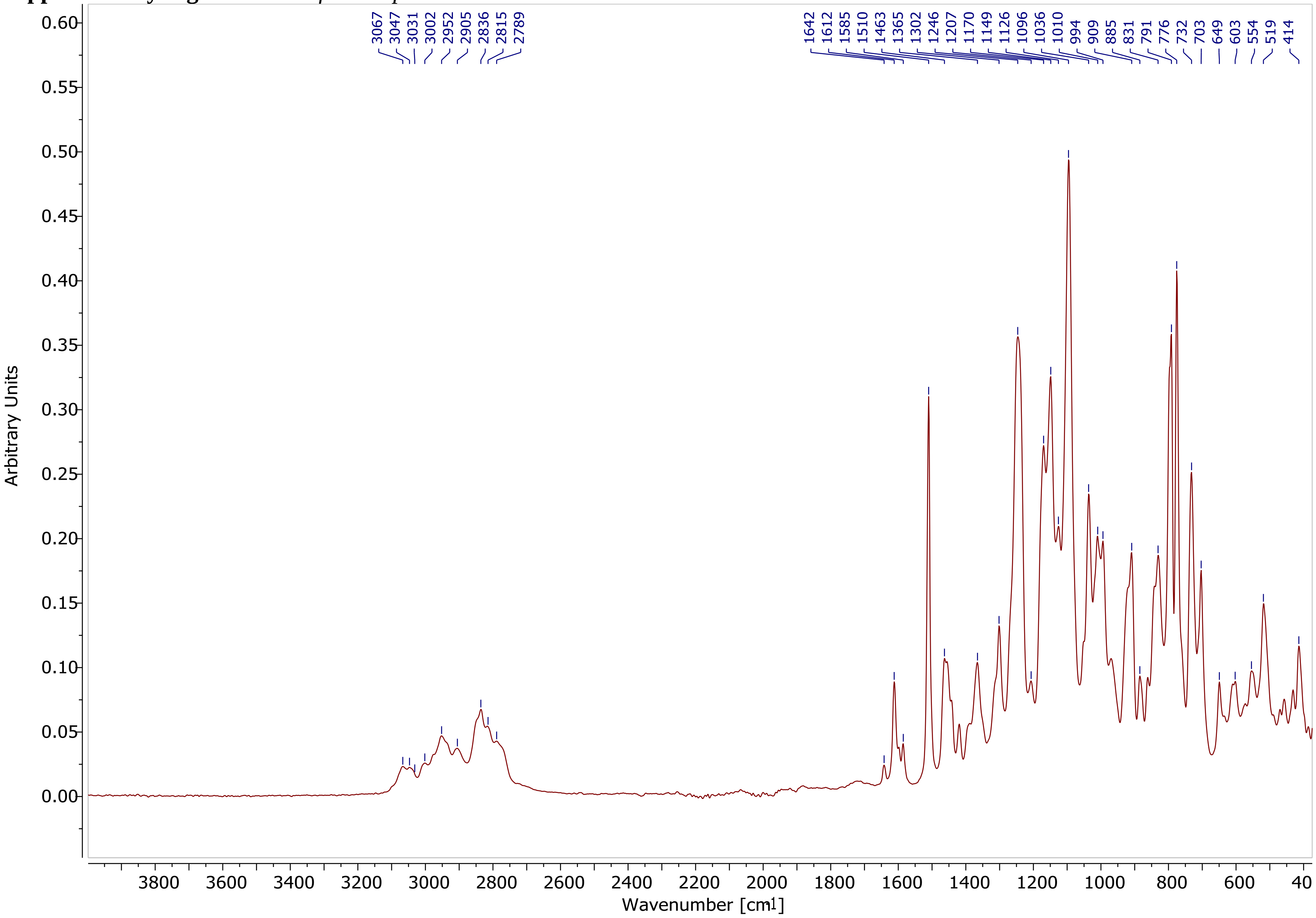

Supplementary Figure 328: *^{1}H-NMR for compound* ***67***

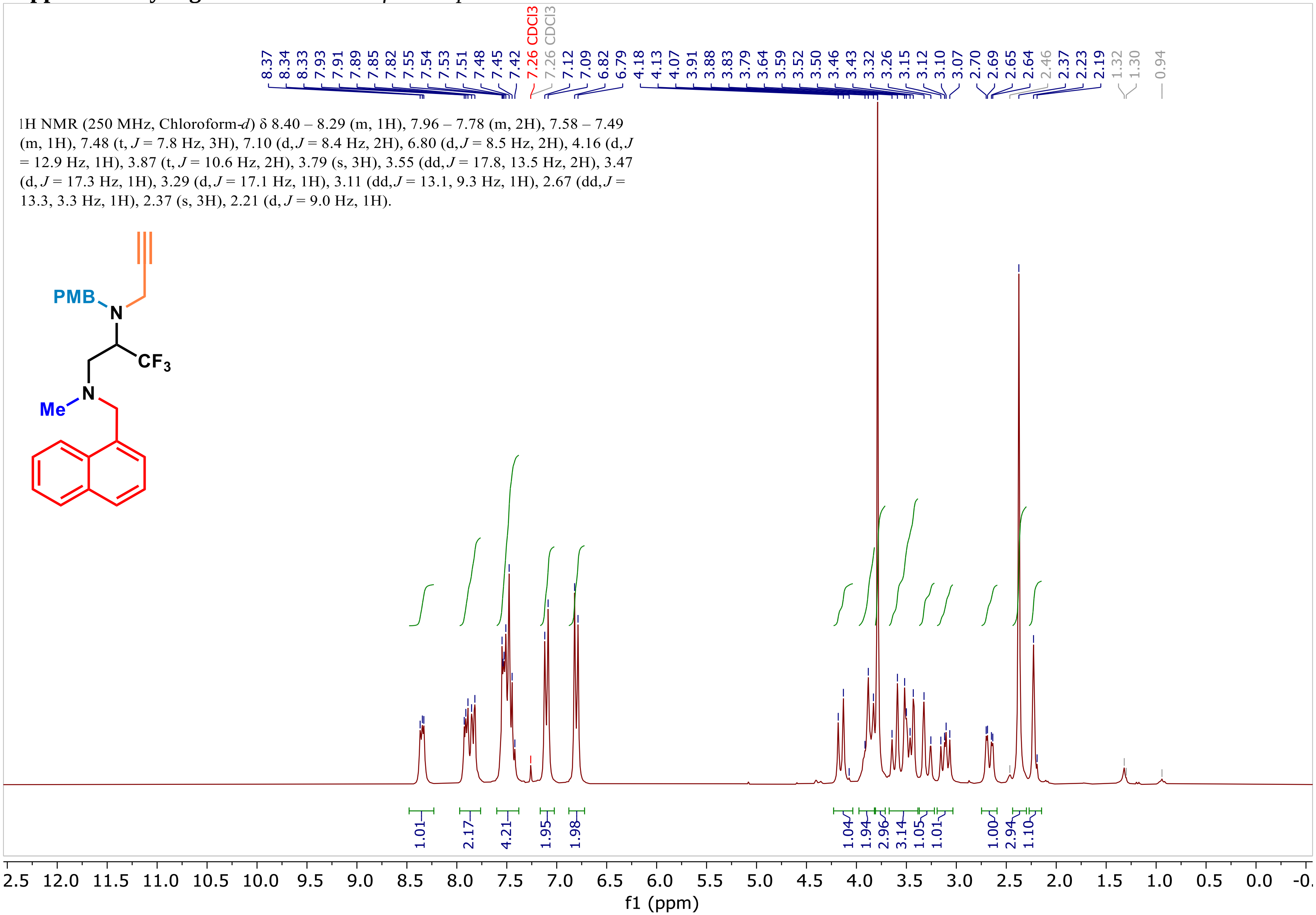

1H NMR (250 MHz, Chloroform-*d*) δ 8.40 – 8.29 (m, 1H), 7.96 – 7.78 (m, 2H), 7.58 – 7.49 (m, 1H), 7.48 (t, *J* = 7.8 Hz, 3H), 7.10 (d, *J* = 8.4 Hz, 2H), 6.80 (d, *J* = 8.5 Hz, 2H), 4.16 (d, *J* = 12.9 Hz, 1H), 3.87 (t, *J* = 10.6 Hz, 2H), 3.79 (s, 3H), 3.55 (dd, *J* = 17.8, 13.5 Hz, 2H), 3.47 (d, *J* = 17.3 Hz, 1H), 3.29 (d, *J* = 17.1 Hz, 1H), 3.11 (dd, *J* = 13.1, 9.3 Hz, 1H), 2.67 (dd, *J* = 13.3, 3.3 Hz, 1H), 2.37 (s, 3H), 2.21 (d, *J* = 9.0 Hz, 1H).

Supplementary Figure 329: *^{19}F NMR for compound* ***67***

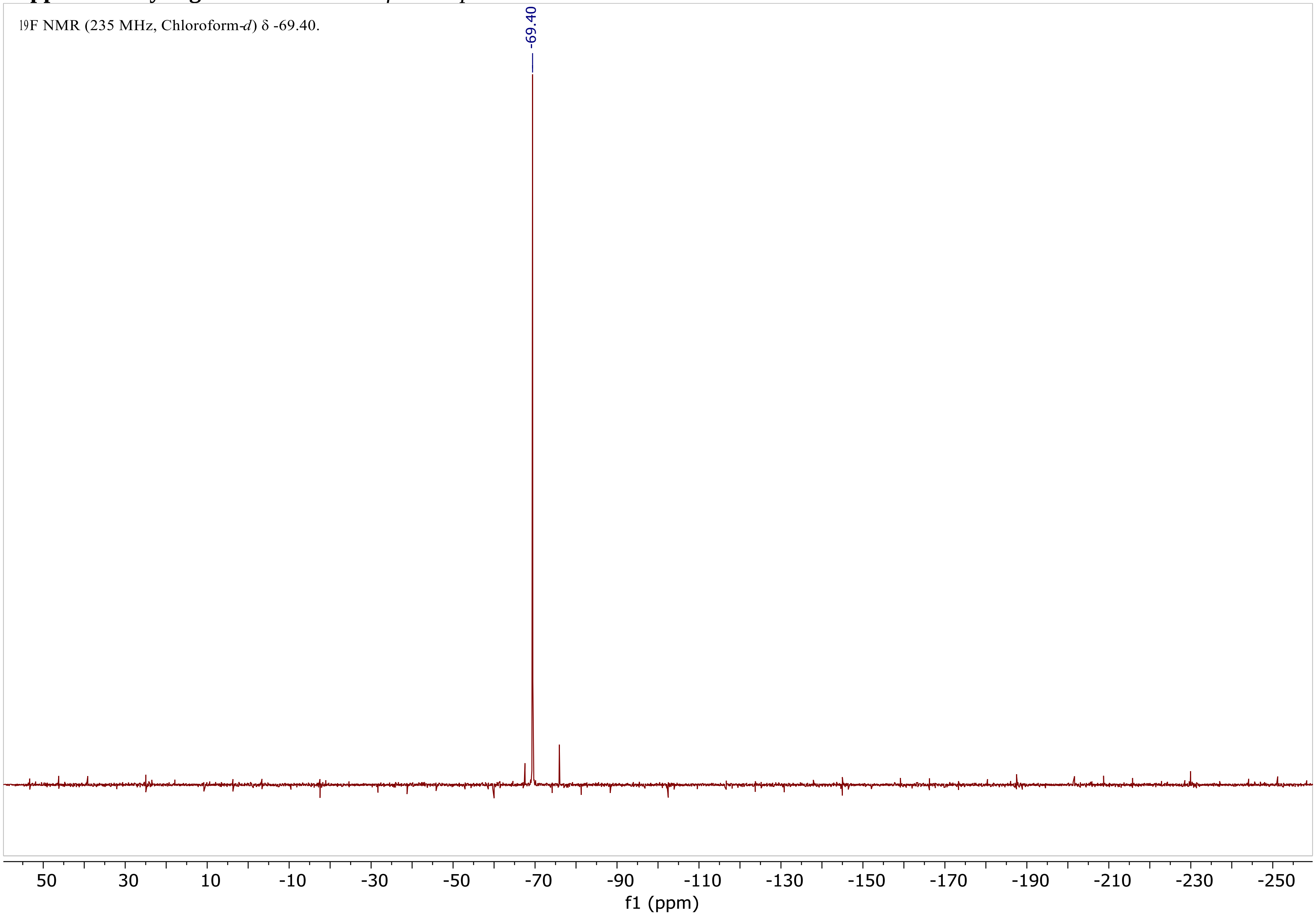

Supplementary Figure 330: ^{13}*C NMR for compound* ***67***

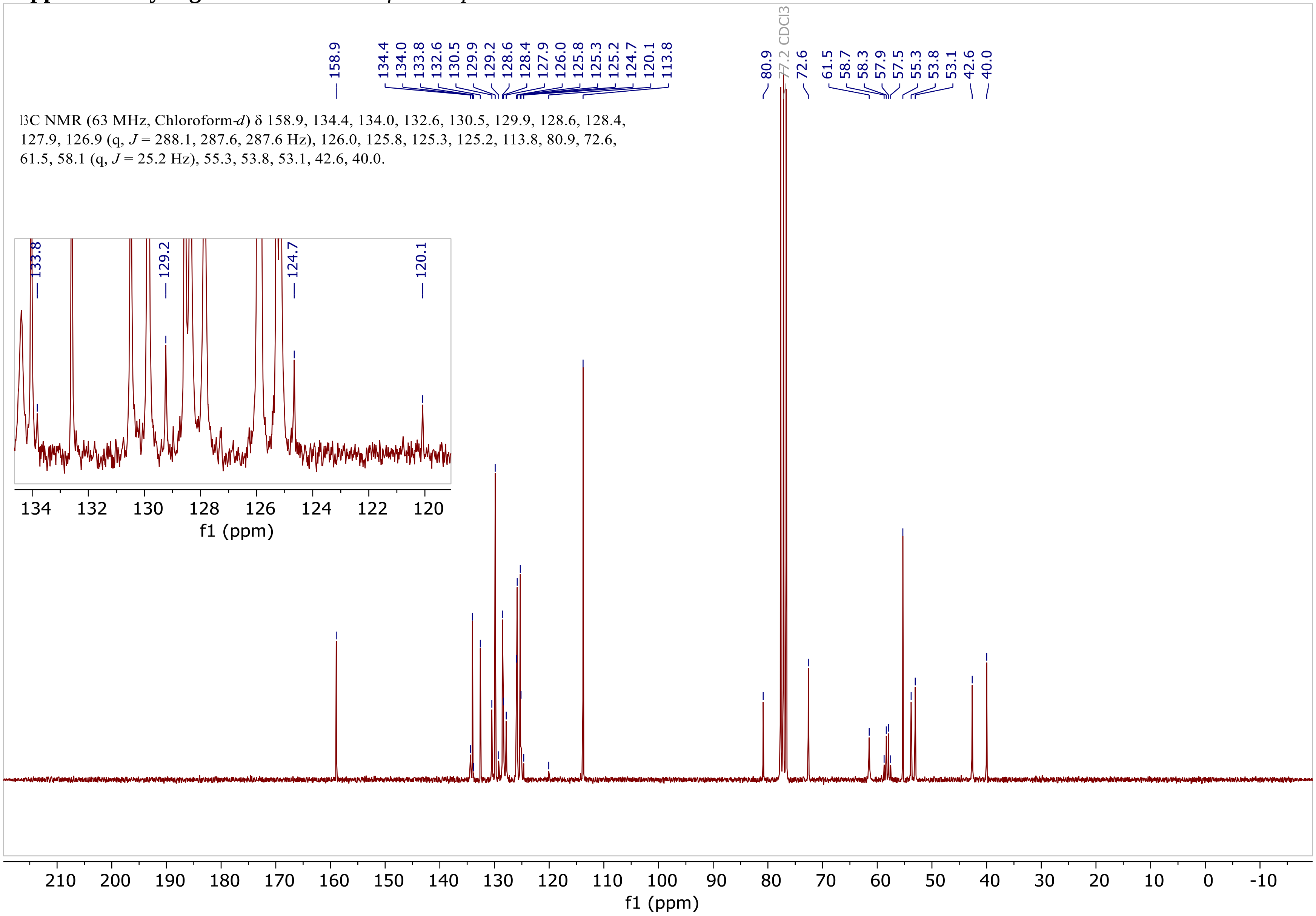

13C NMR (63 MHz, Chloroform-*d*) δ 158.9, 134.4, 134.0, 132.6, 130.5, 129.9, 128.6, 128.4, 127.9, 126.9 (q, *J* = 288.1, 287.6, 287.6 Hz), 126.0, 125.8, 125.3, 125.2, 113.8, 80.9, 72.6, 61.5, 58.1 (q, *J* = 25.2 Hz), 55.3, 53.8, 53.1, 42.6, 40.0.

Supplementary Figure 331: *IR for compound* ***67***

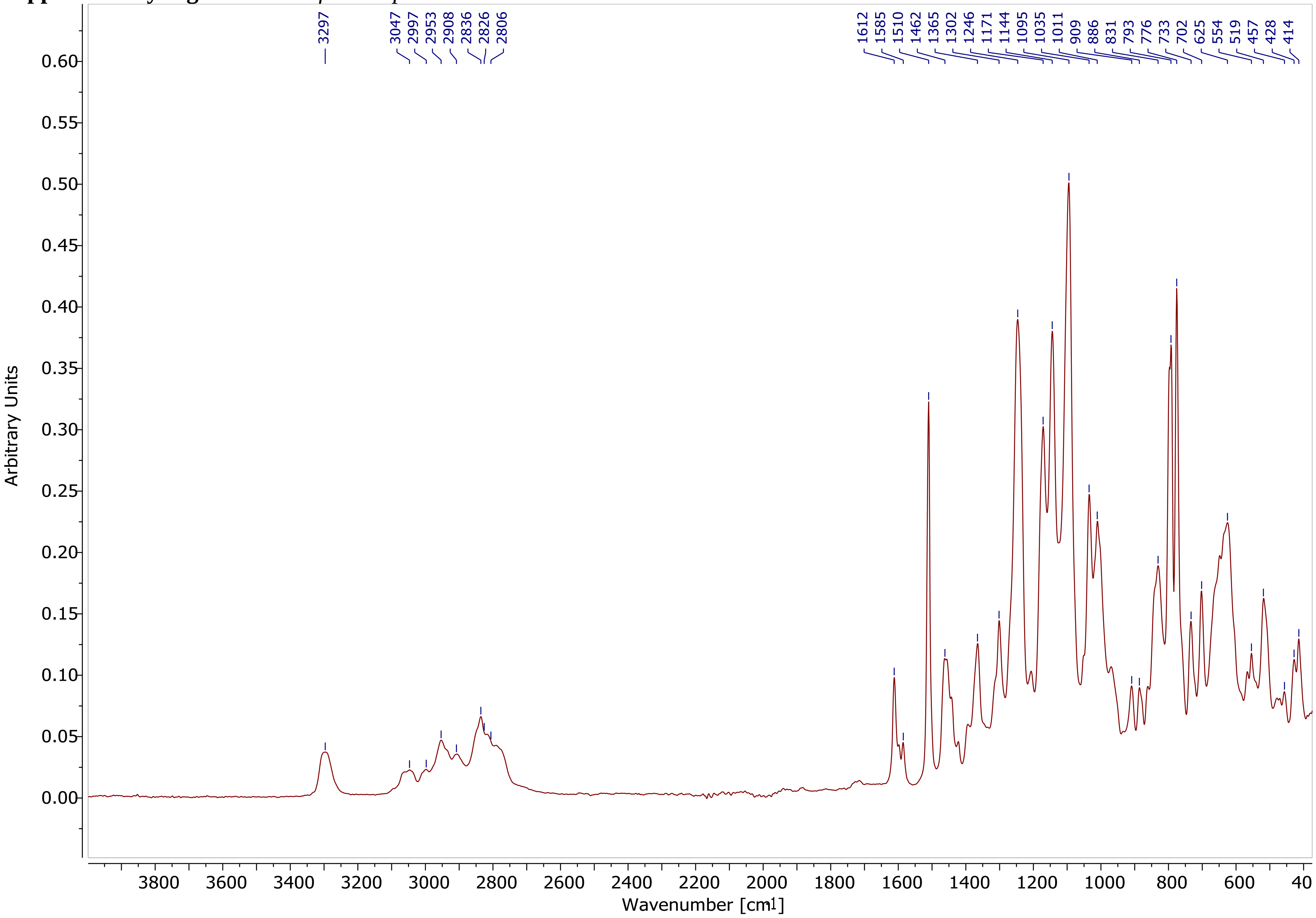

Supplementary Figure 332: *^{1}H-NMR for compound* ***68***

1H NMR (250 MHz, Chloroform-*d*) δ 8.25 – 8.12 (m, 1H), 7.82 – 7.59 (m, 2H), 7.30 (ddd, *J* = 21.7, 10.0, 5.6 Hz, 6H), 7.15 (s, 3H), 3.97 (d, *J* = 13.0 Hz, 1H), 3.78 (d, *J* = 13.0 Hz, 1H), 3.70 – 3.36 (m, 3H), 2.90 (dd, *J* = 13.2, 8.2 Hz, 1H), 2.58 (dd, *J* = 13.3, 3.8 Hz, 1H), 2.24 (s, 5H), 1.59 (dp, *J* = 13.6, 7.2 Hz, 1H), 0.76 (d, *J* = 6.4 Hz, 3H), 0.67 (d, *J* = 6.4 Hz, 3H).

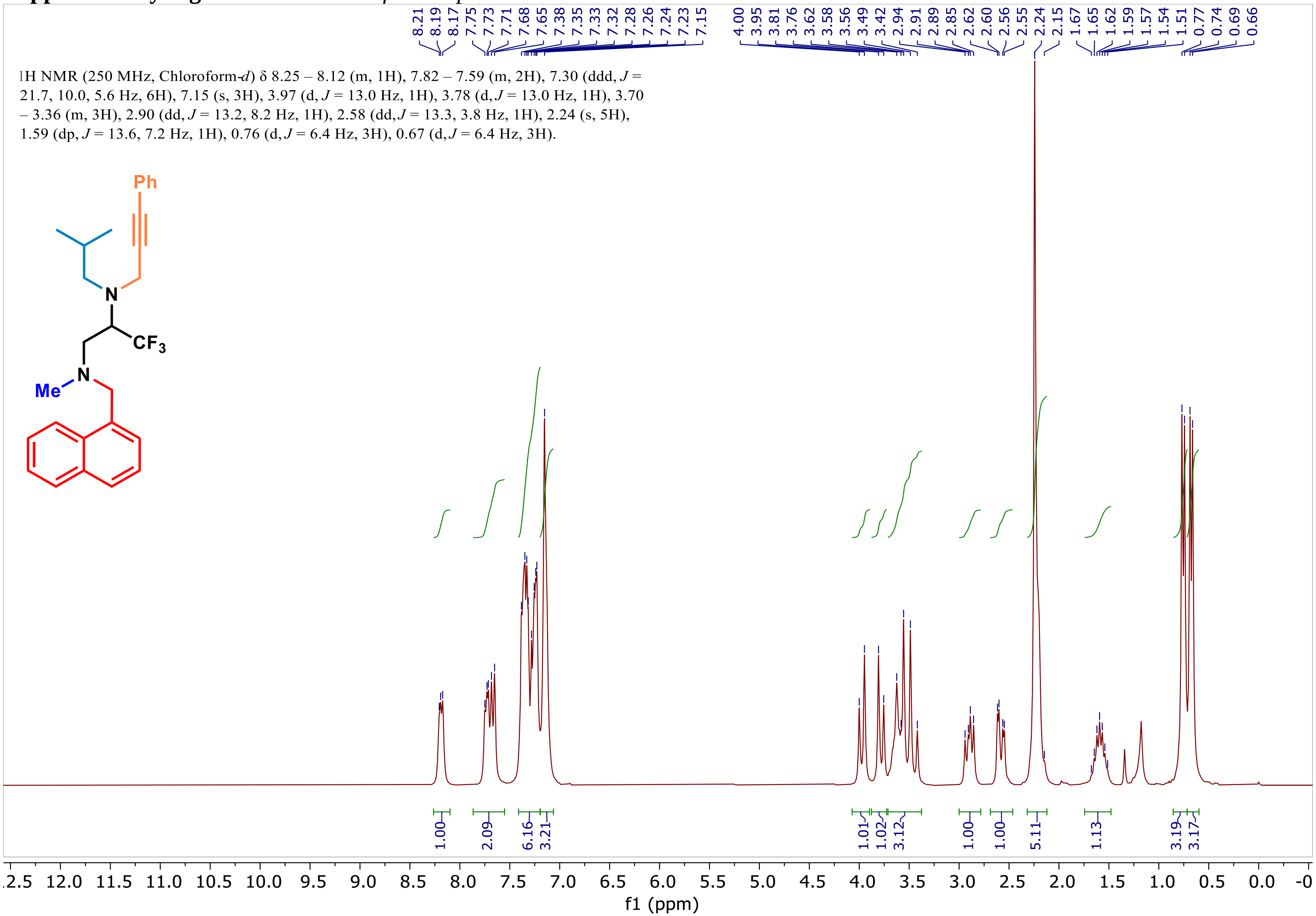

Supplementary Figure 333: *^{19}F NMR for compound* ***68***

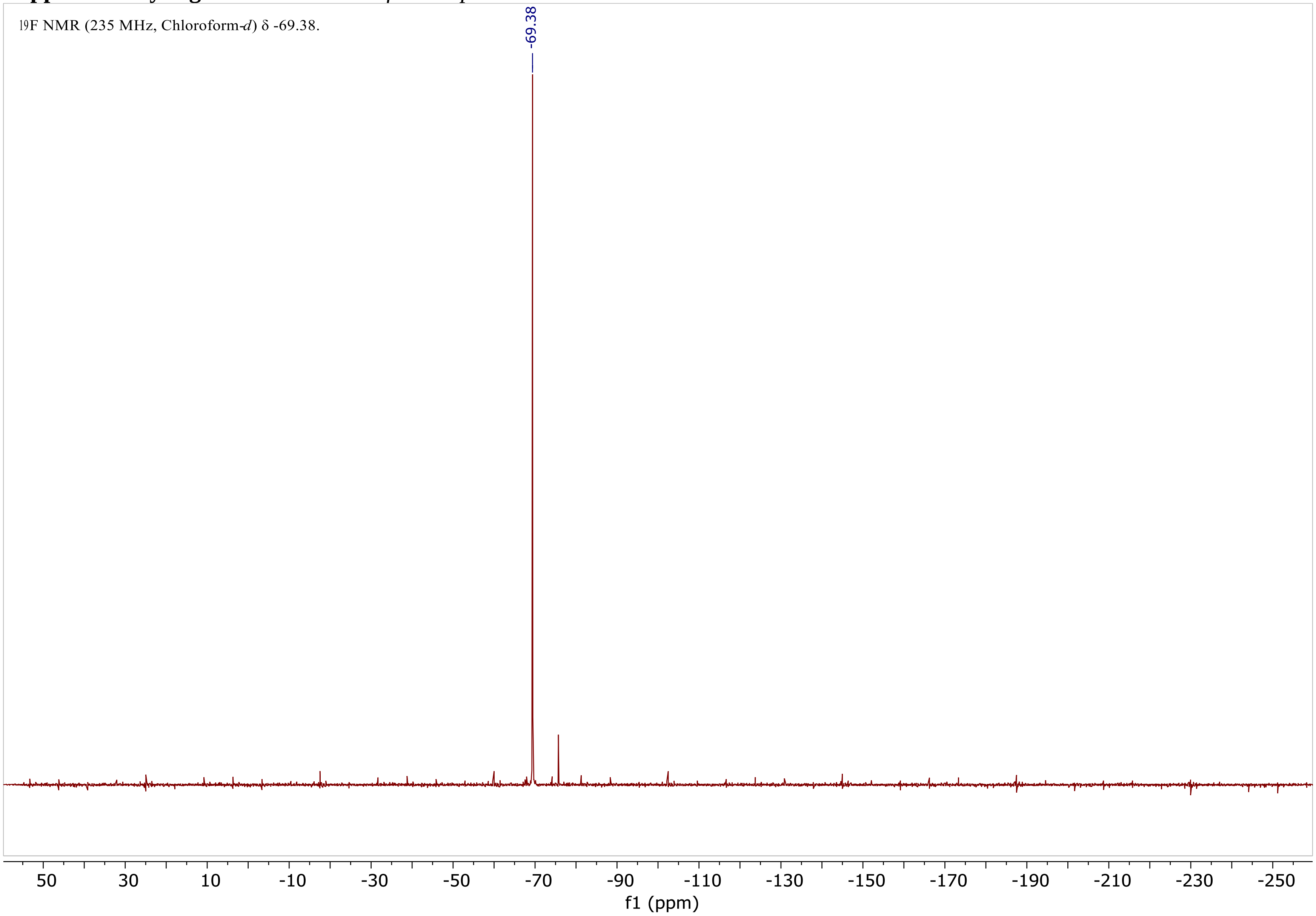

Supplementary Figure 334: *^{13}C NMR for compound* ***68***

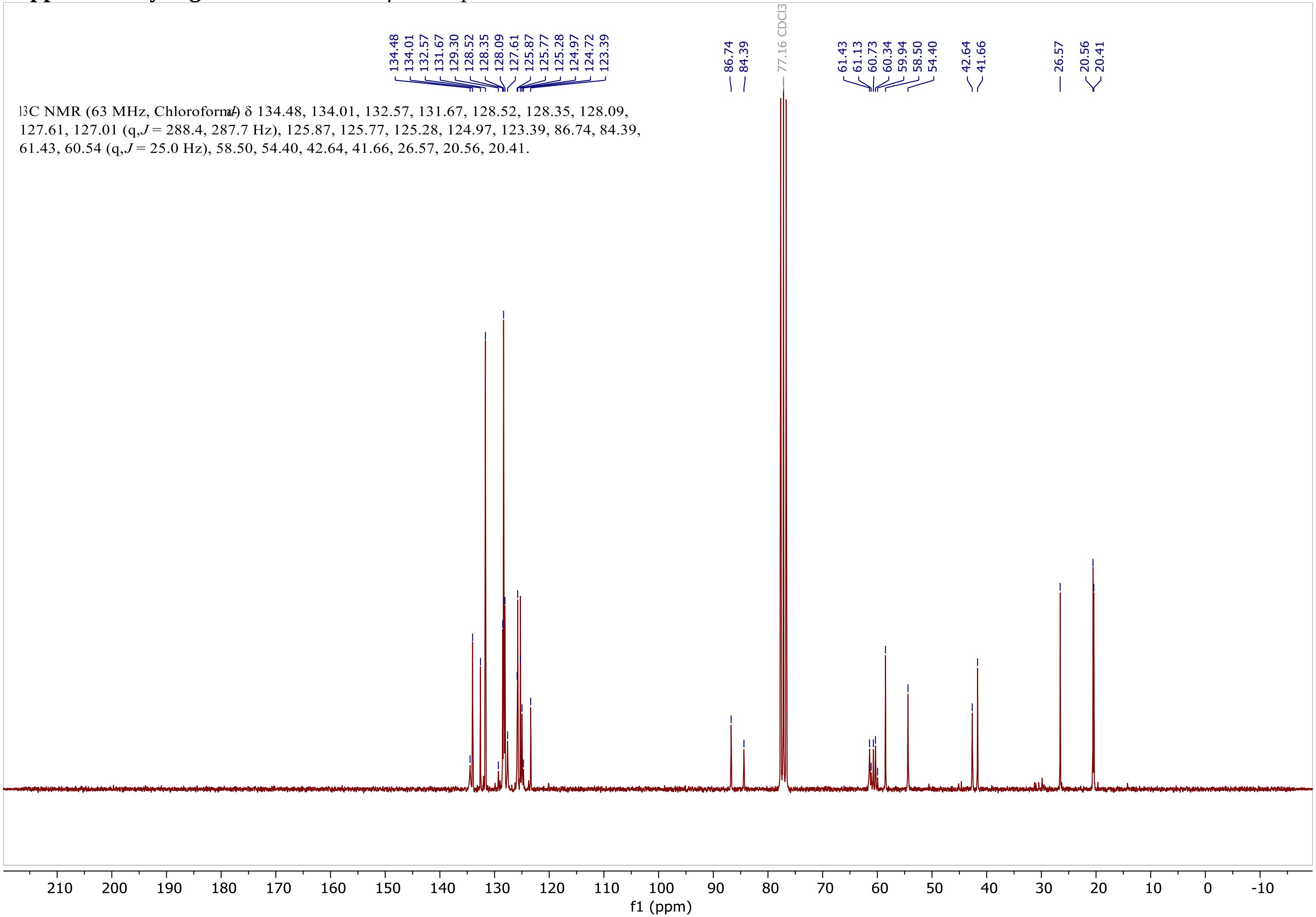

Supplementary Figure 335: *IR for compound* ***68***

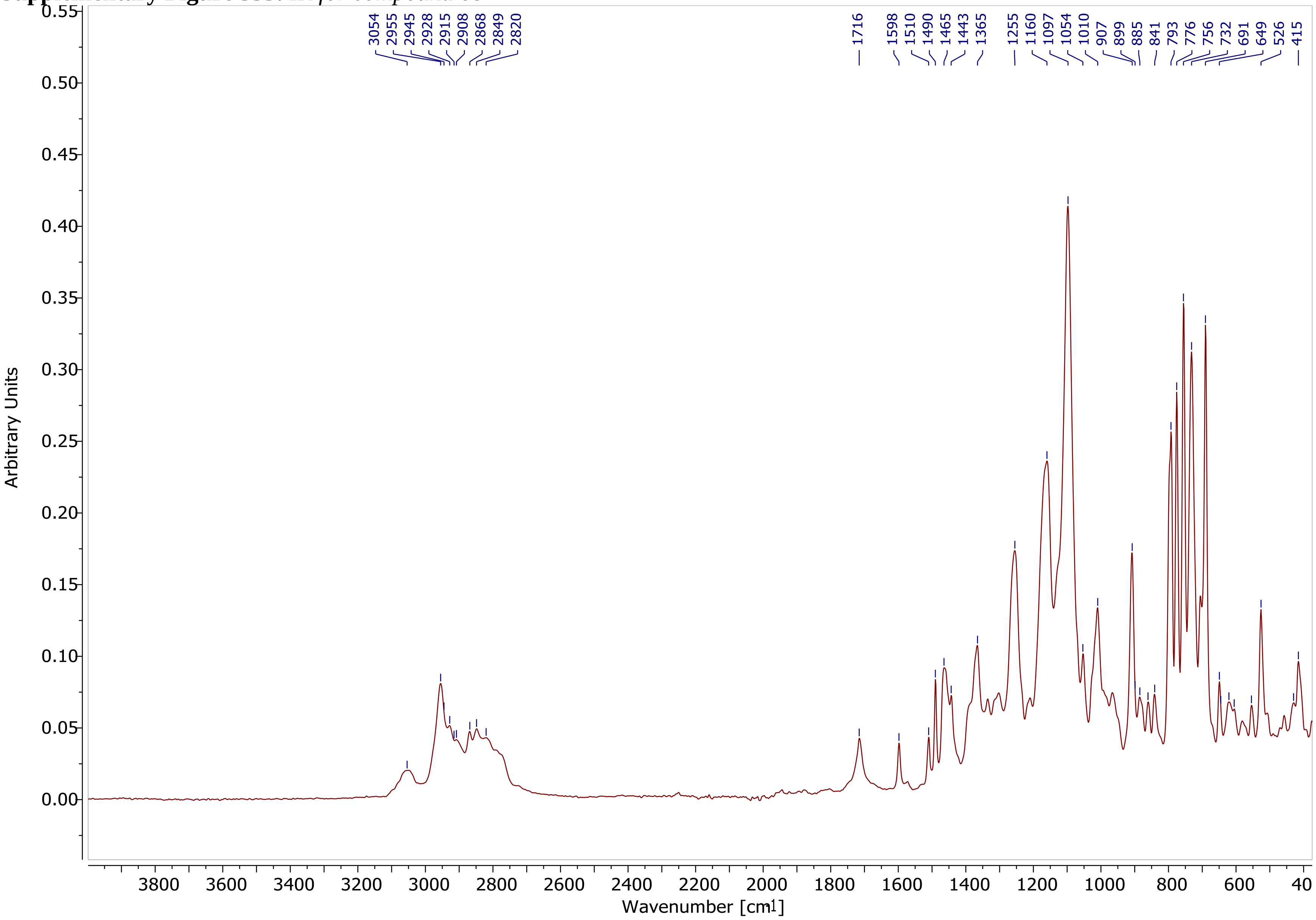

Supplementary Figure 336: *^{1}H-NMR for compound* ***69***

1H NMR (250 MHz, Chloroform-*d*) δ 8.26 (d, *J* = 7.7 Hz, 1H), 7.94 – 7.72 (m, 2H), 7.61 – 7.35 (m, 4H), 4.31 (p, *J* = 6.2 Hz, 1H), 4.09 (d, *J* = 12.9 Hz, 1H), 3.76 (d, *J* = 13.0 Hz, 1H), 3.71 – 3.60 (m, 1H), 3.30 (td, *J* = 6.5, 2.6 Hz, 1H), 3.15 (t, *J* = 6.7 Hz, 1H), 3.06 – 2.89 (m, 2H), 2.65 (qd, *J* = 13.6, 4.8 Hz, 2H), 2.32 (s, 3H), 0.88 (s, 9H), -0.00 (s, 6H).

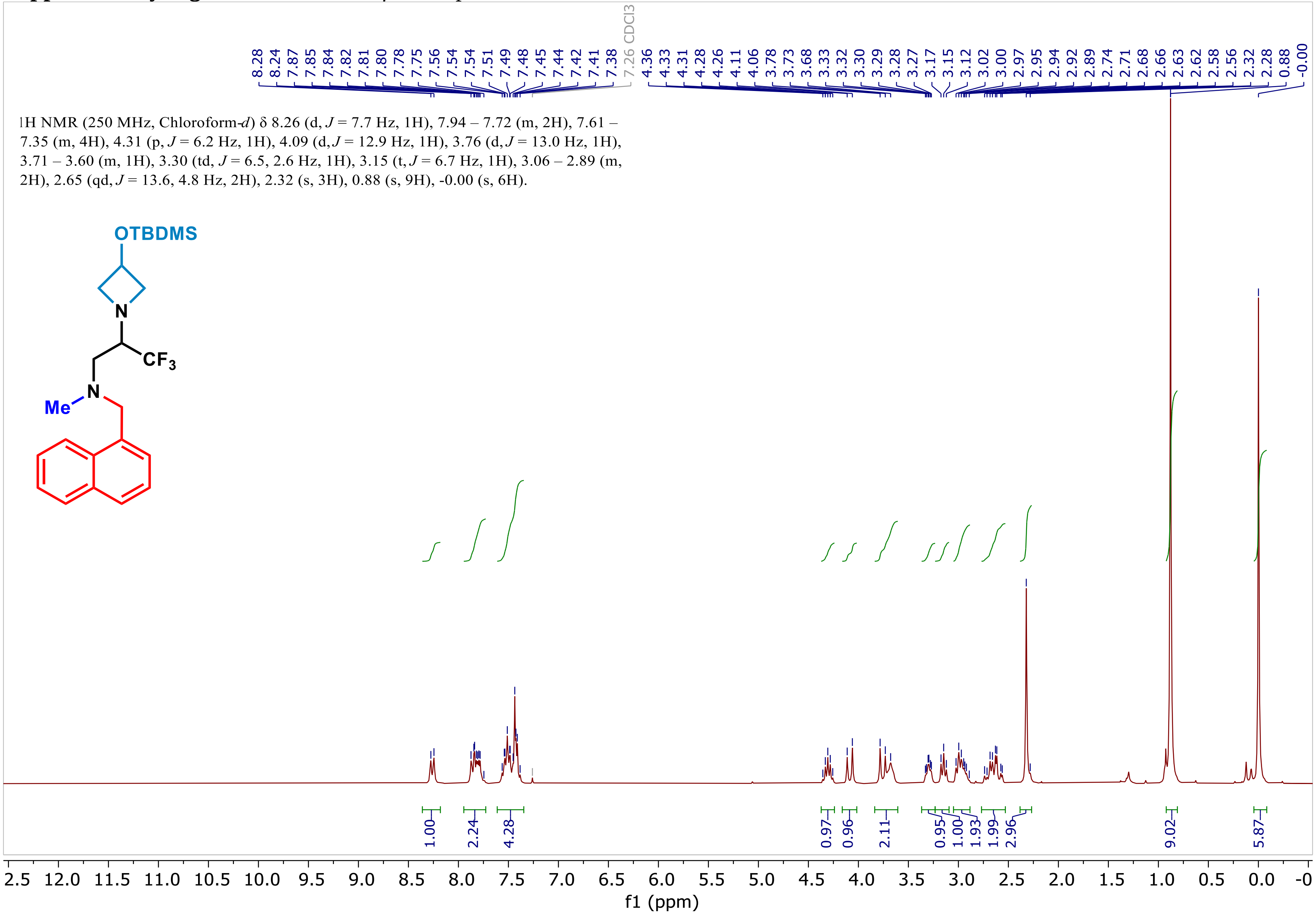

Supplementary Figure 337: *^{19}F NMR for compound* ***69***

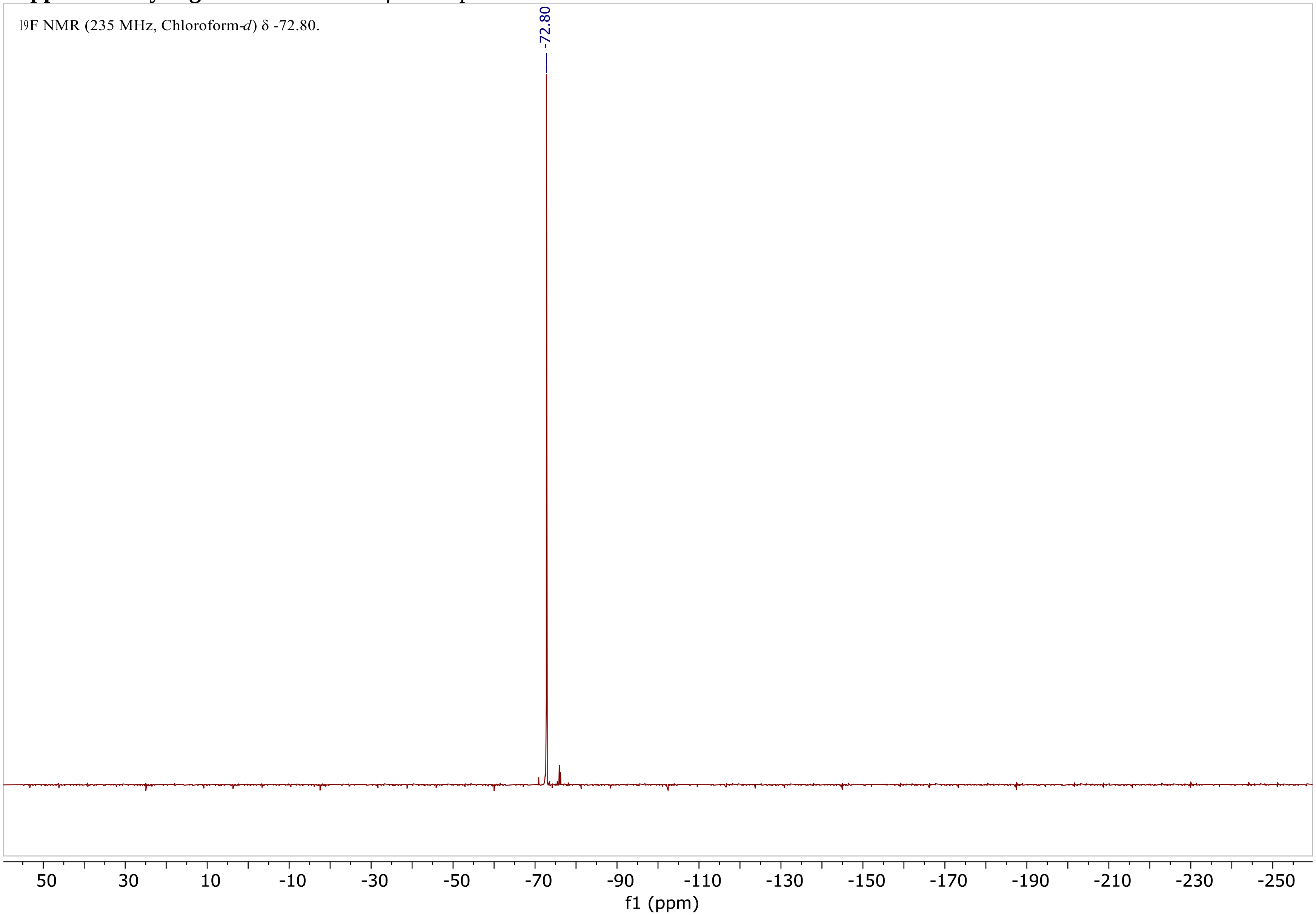

Supplementary Figure 338: *^{13}C NMR for compound **69***

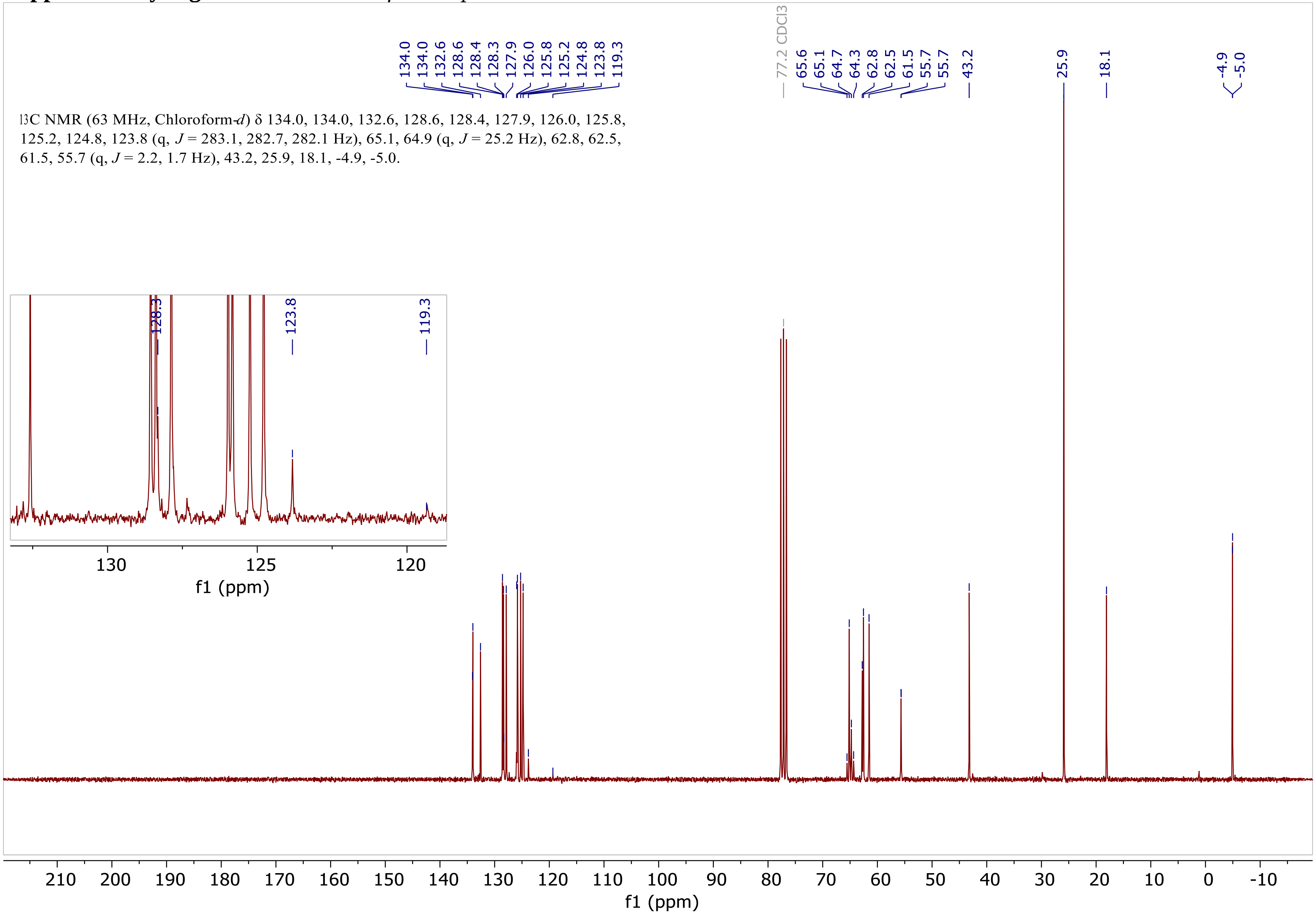

Supplementary Figure 339: *IR for compound* ***69***

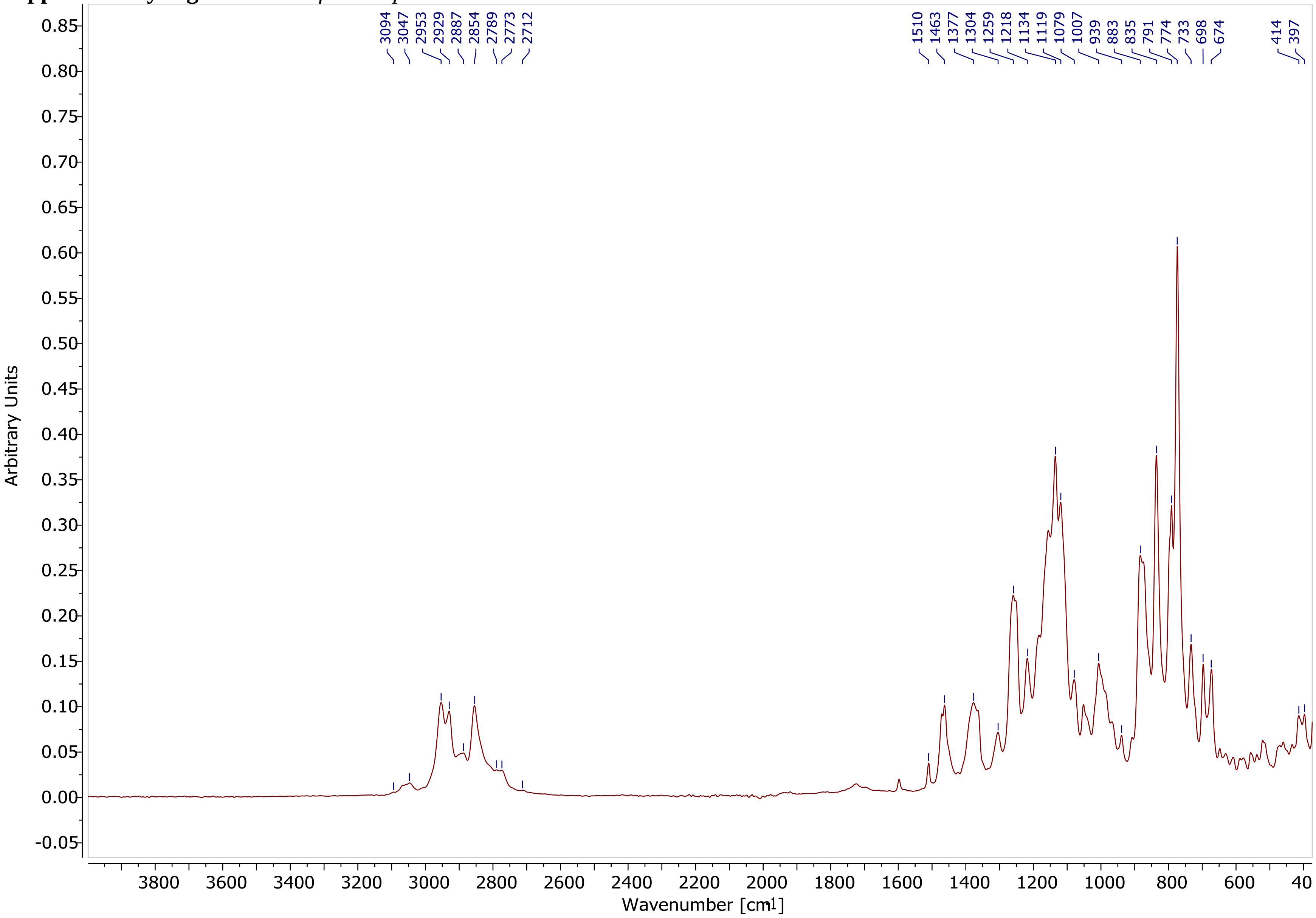

Supplementary Figure 340: *¹H-NMR for compound **70***

1H NMR (250 MHz, Chloroform-*d*) δ 8.41 – 8.27 (m, 1H), 7.99 – 7.73 (m, 2H), 7.61 – 7.37 (m, 4H), 4.09 (d, *J* = 12.8 Hz, 1H), 3.87 (d, *J* = 12.8 Hz, 1H), 3.58 (pd, *J* = 8.7, 3.6 Hz, 1H), 2.99 (dd, *J* = 13.3, 9.0 Hz, 1H), 2.88 – 2.71 (m, 4H), 2.63 (dd, *J* = 13.3, 3.5 Hz, 1H), 2.31 (s, 3H), 1.75 – 1.54 (m, 4H).

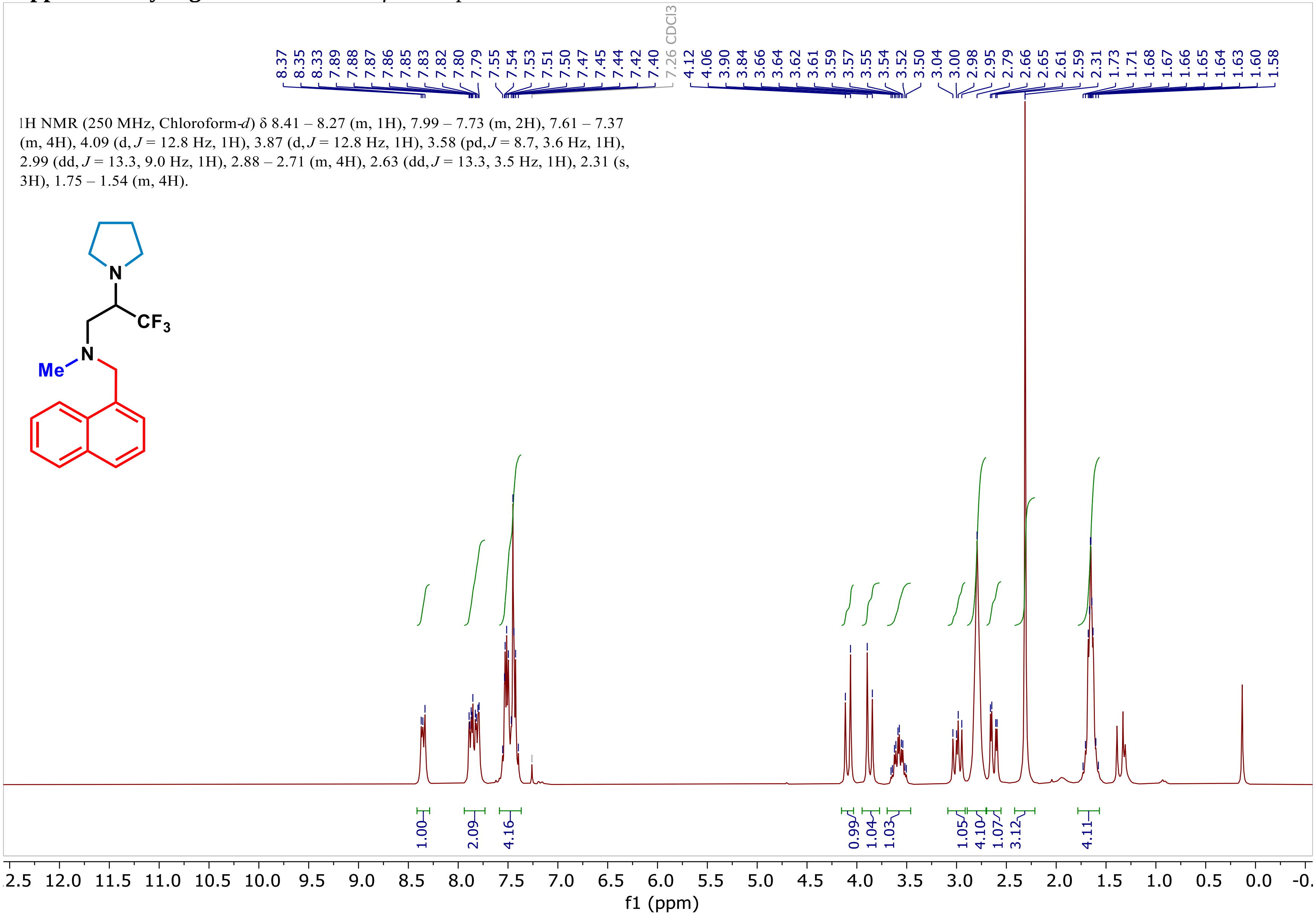

Supplementary Figure 341: Supplementary Figure 342: *COSY NMR for compound **70***

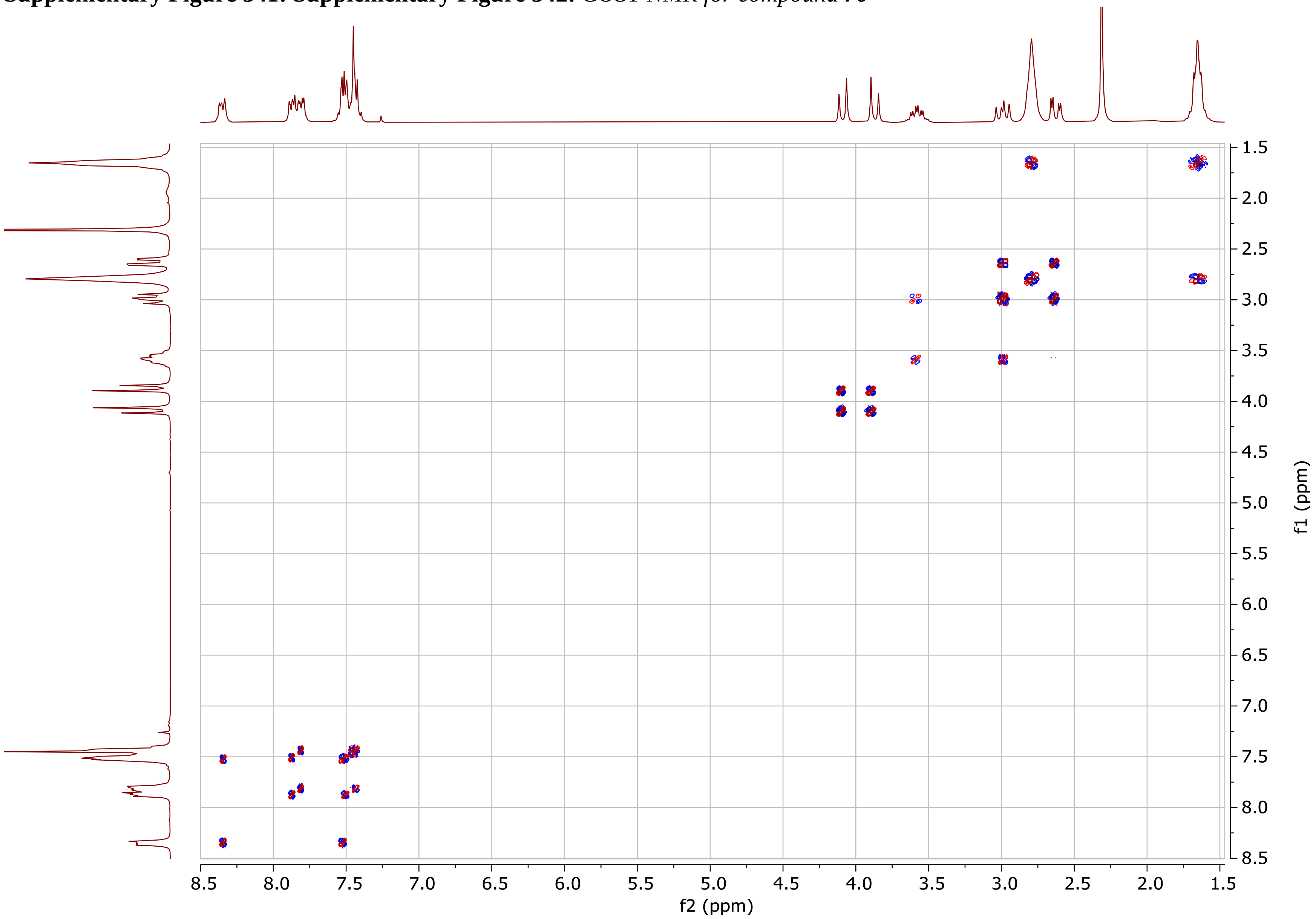

Supplementary Figure 343: *^{19}F NMR for compound* ***70***

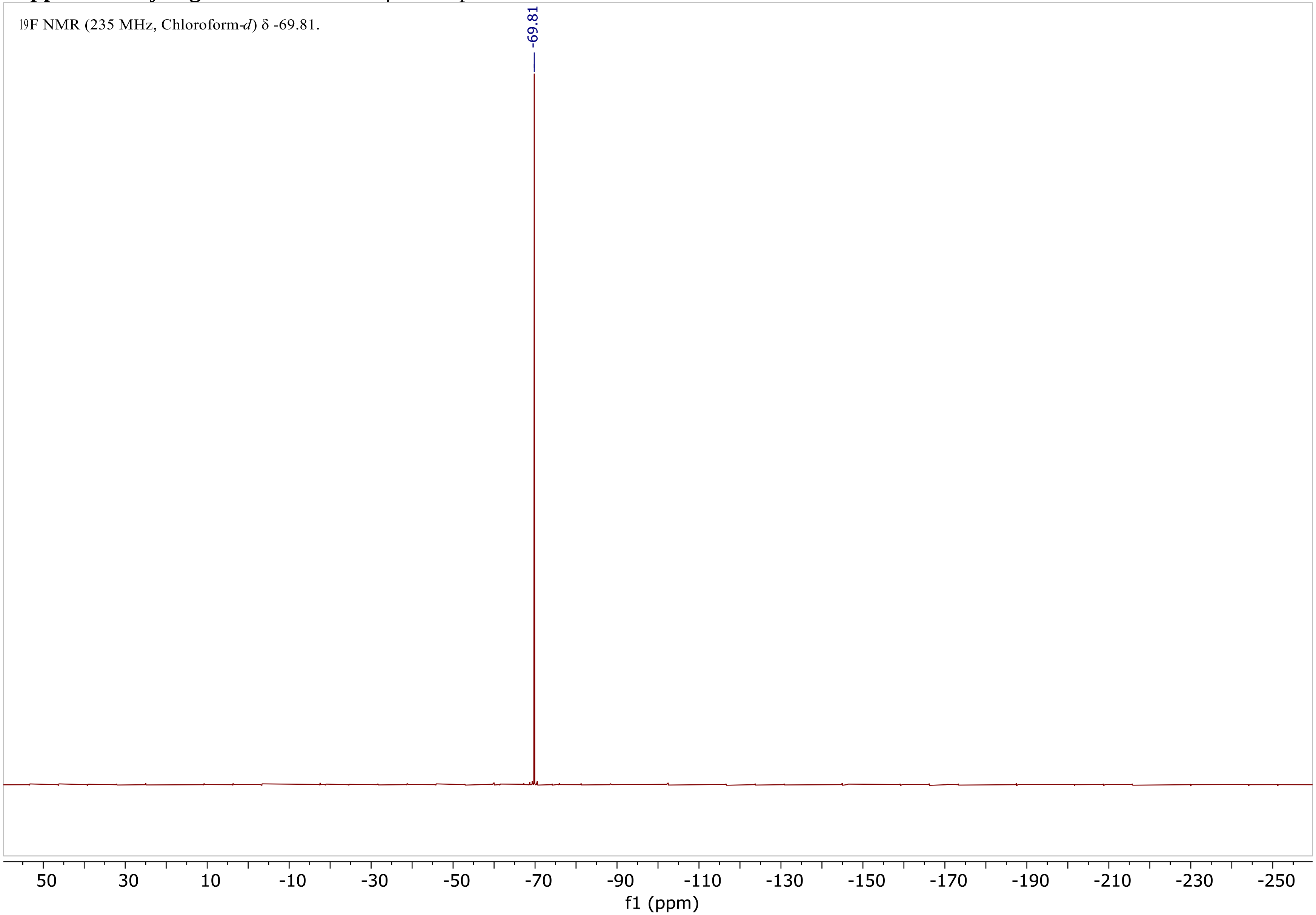

Supplementary Figure 344: *^{13}C NMR for compound **70***

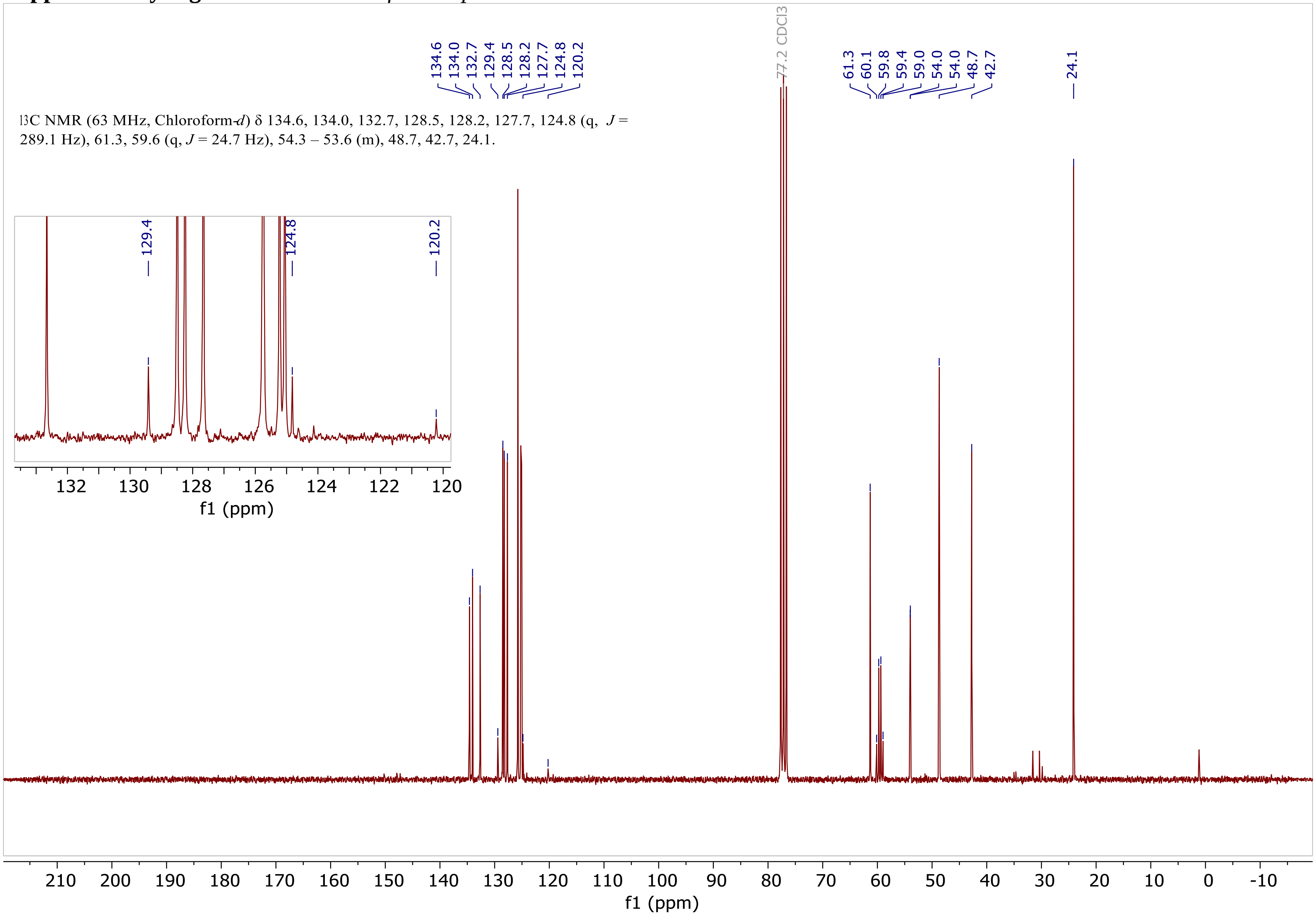

Supplementary Figure 345: *HSQC NMR for compound* ***70***

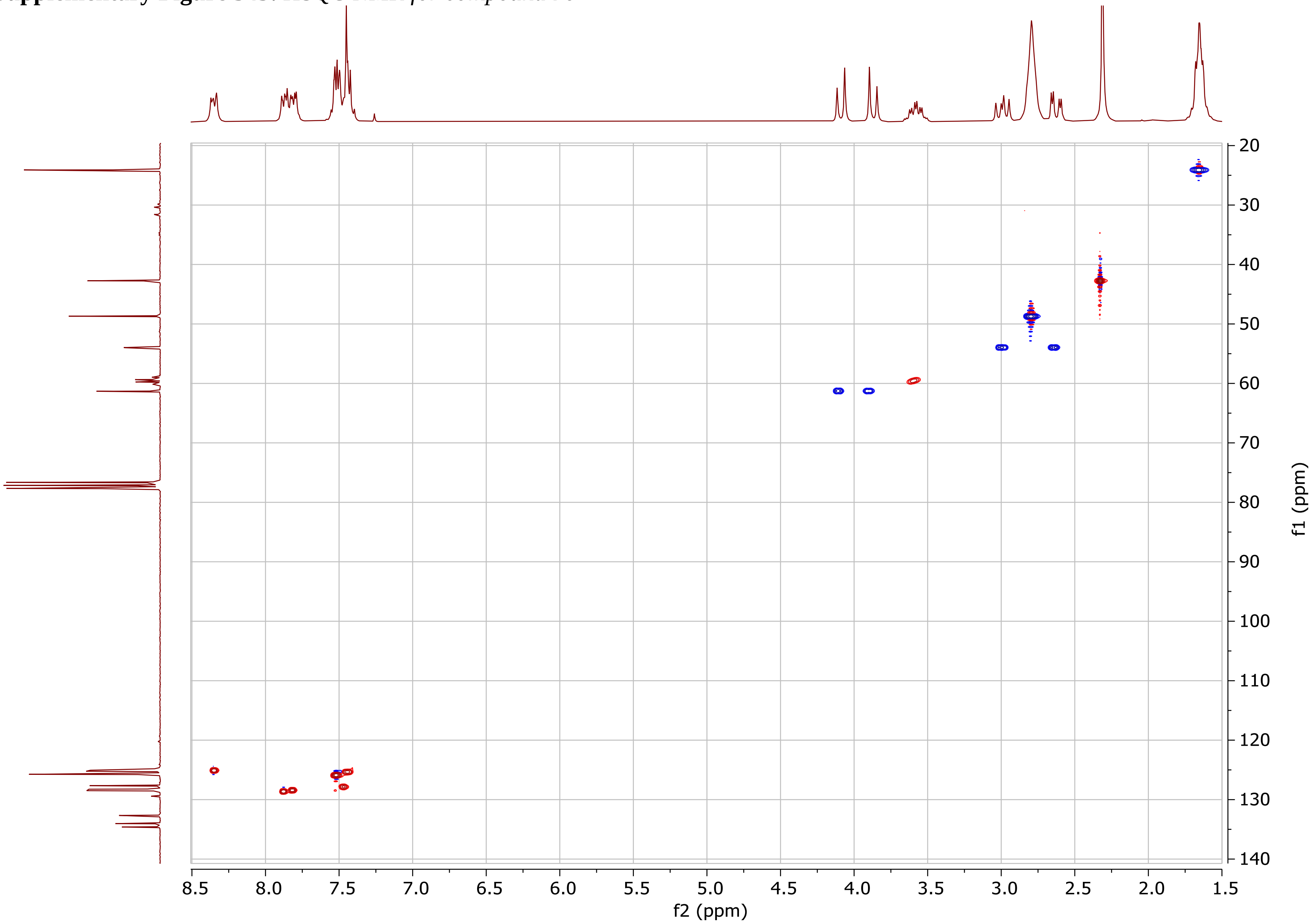

Supplementary Figure 346: *HMBC NMR for compound **70***

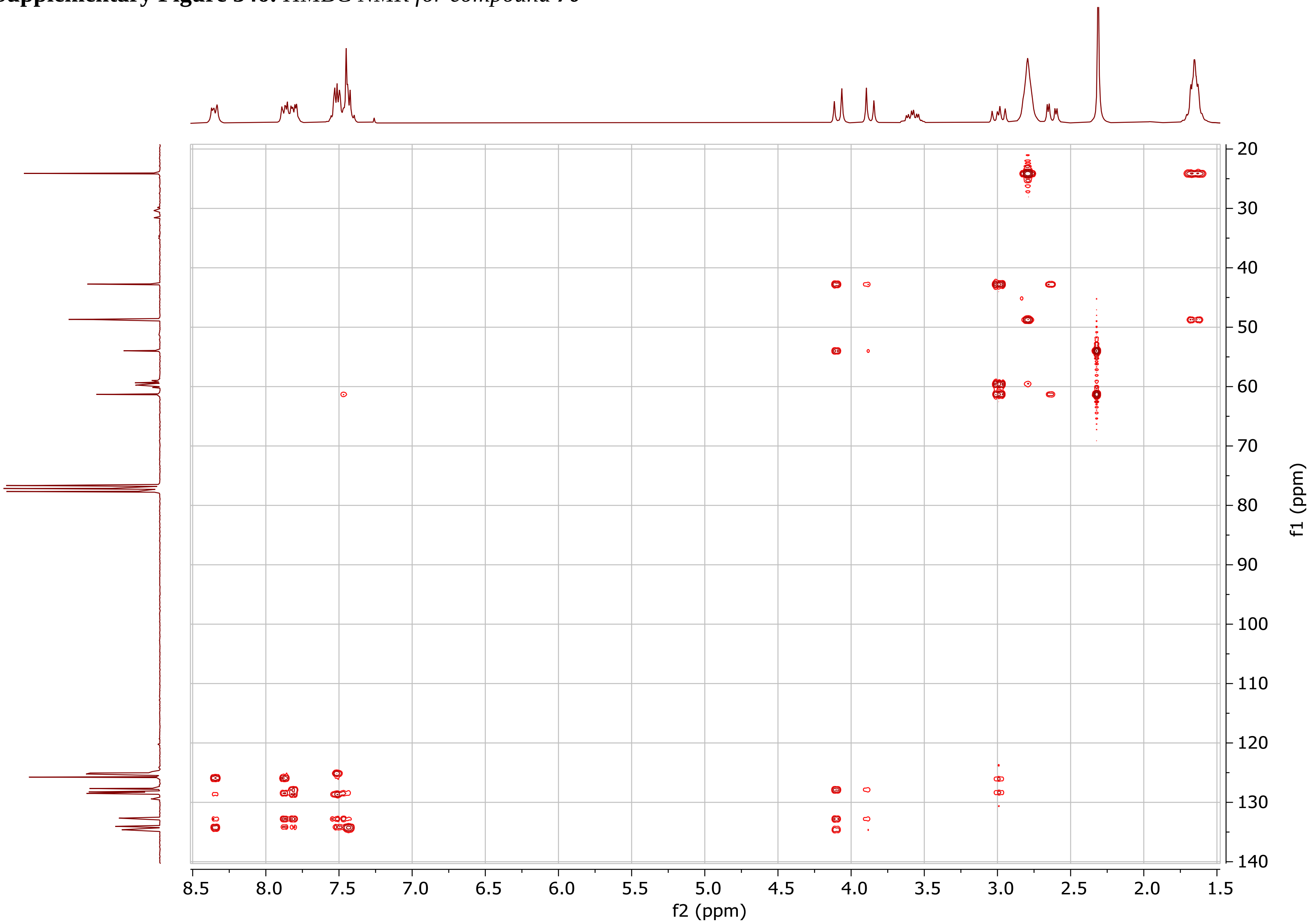

GC-MS TIC for compound ***70***

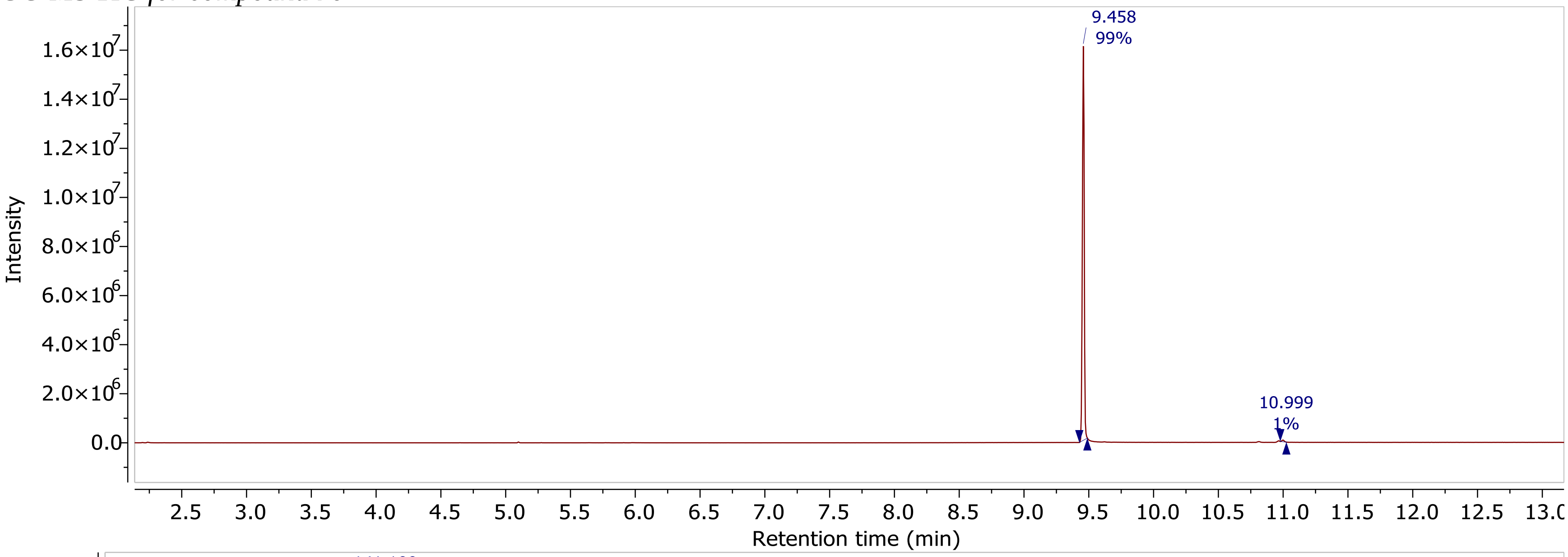

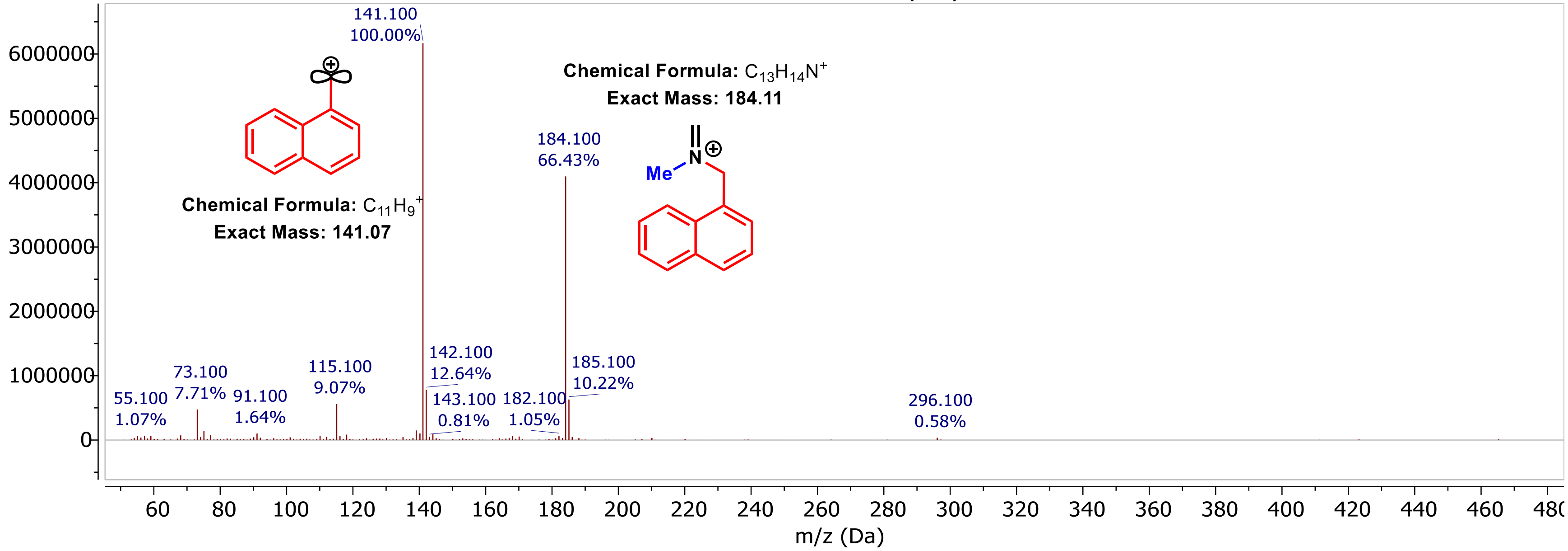

Supplementary Figure 347: *IR for compound* ***70***

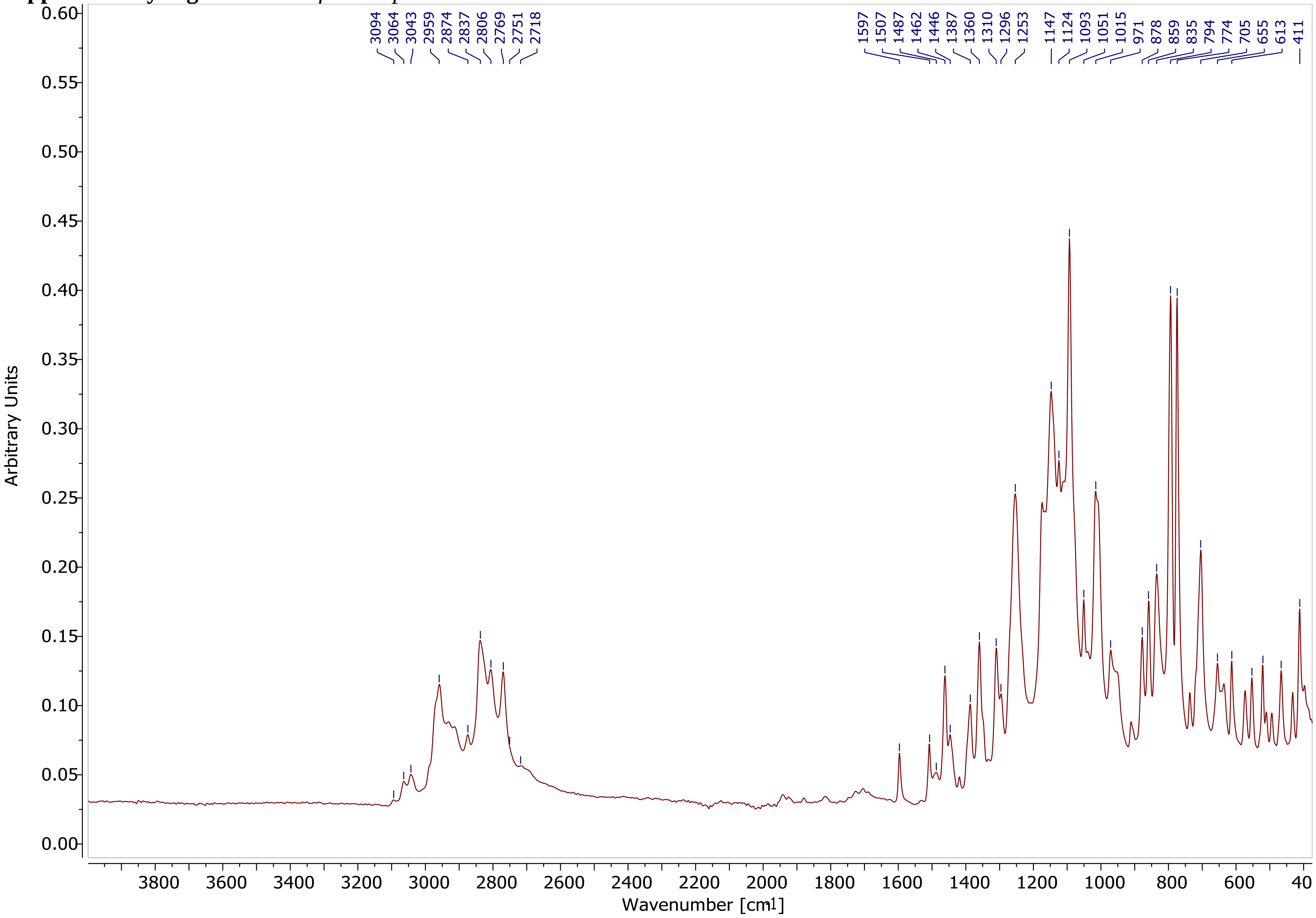

Supplementary Figure 348: *^{1}H-NMR for compound* ***71***

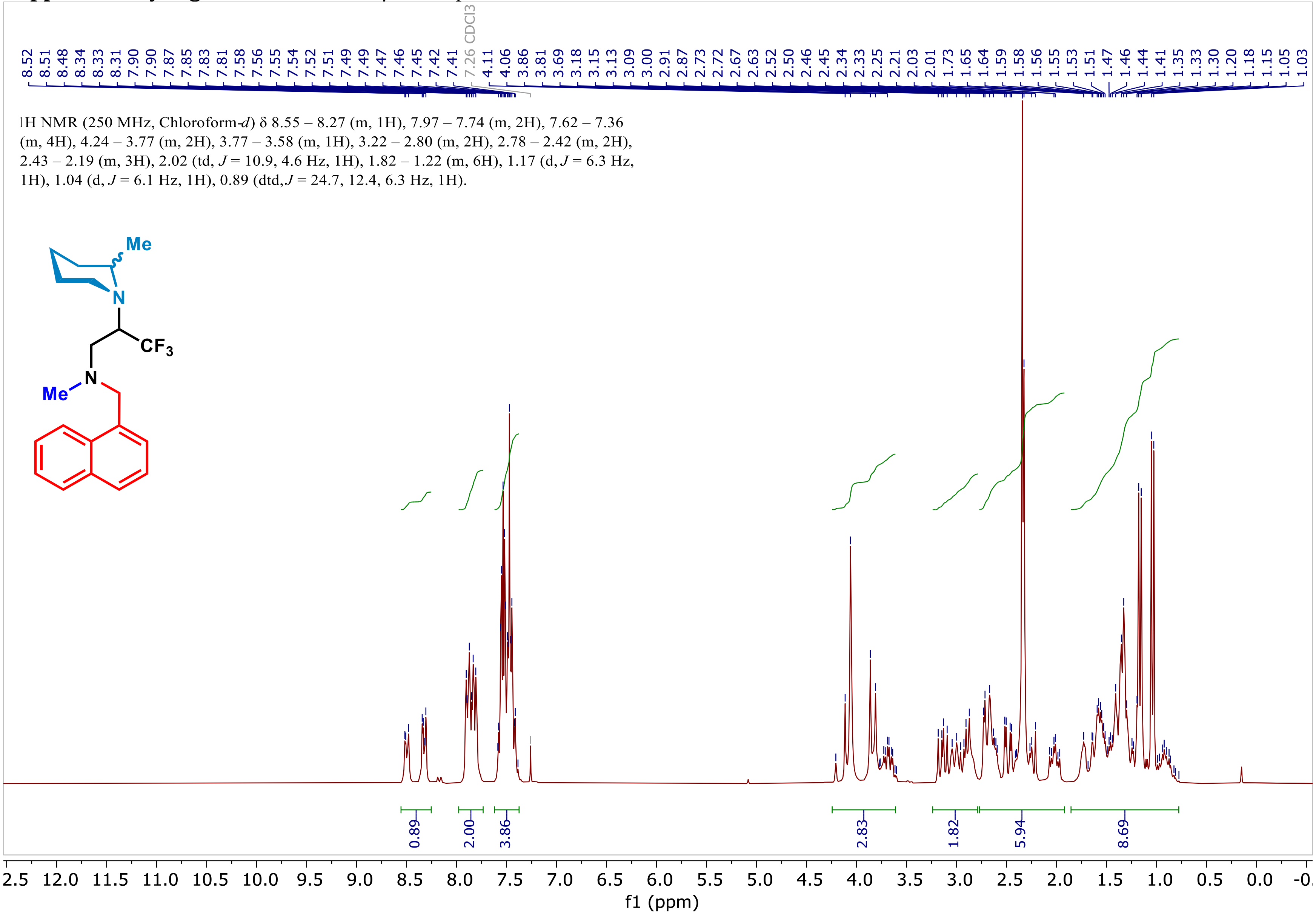

Supplementary Figure 349: *^{19}F NMR for compound* ***71***

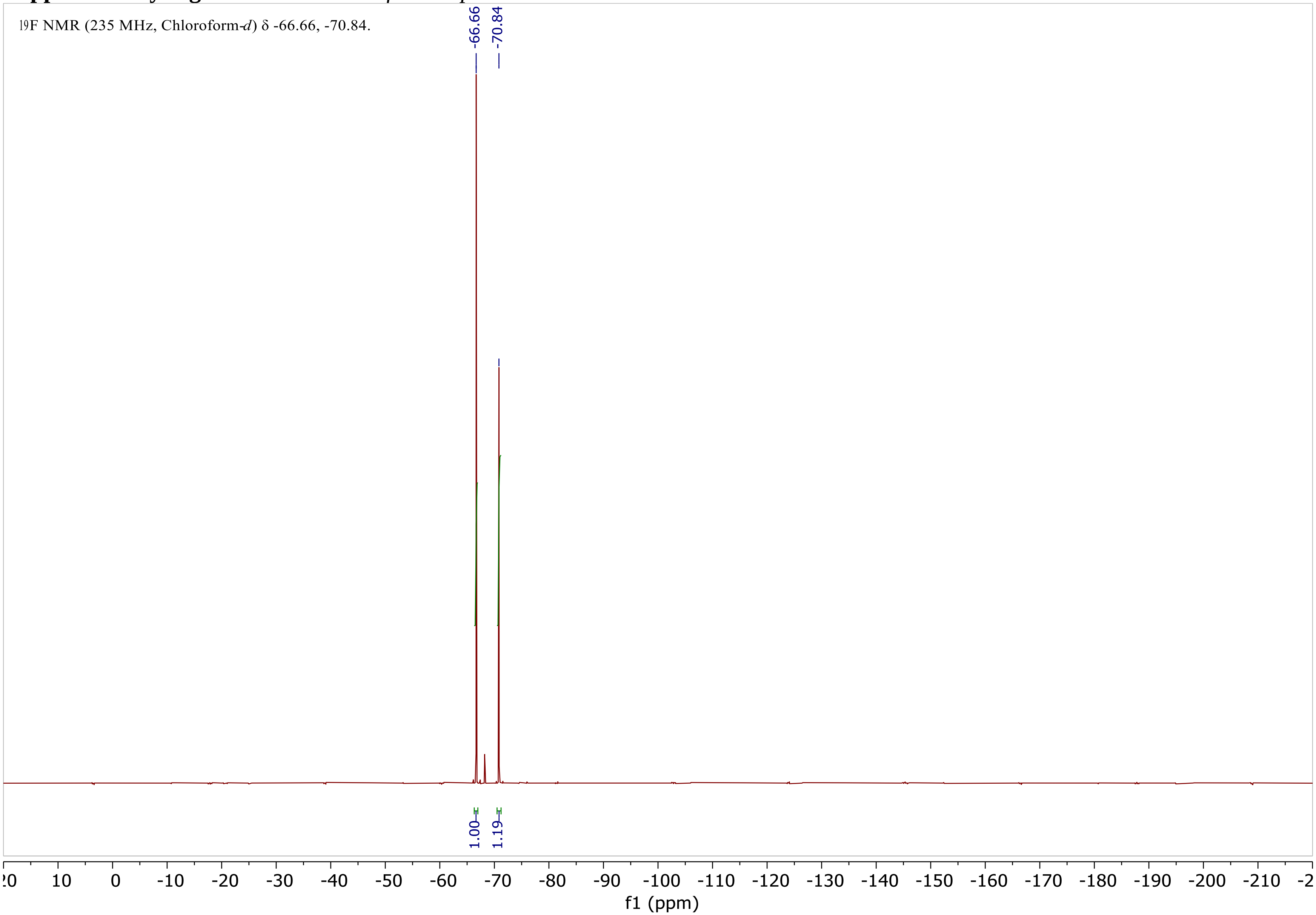

Supplementary Figure 350: *^{13}C NMR for compound **71***

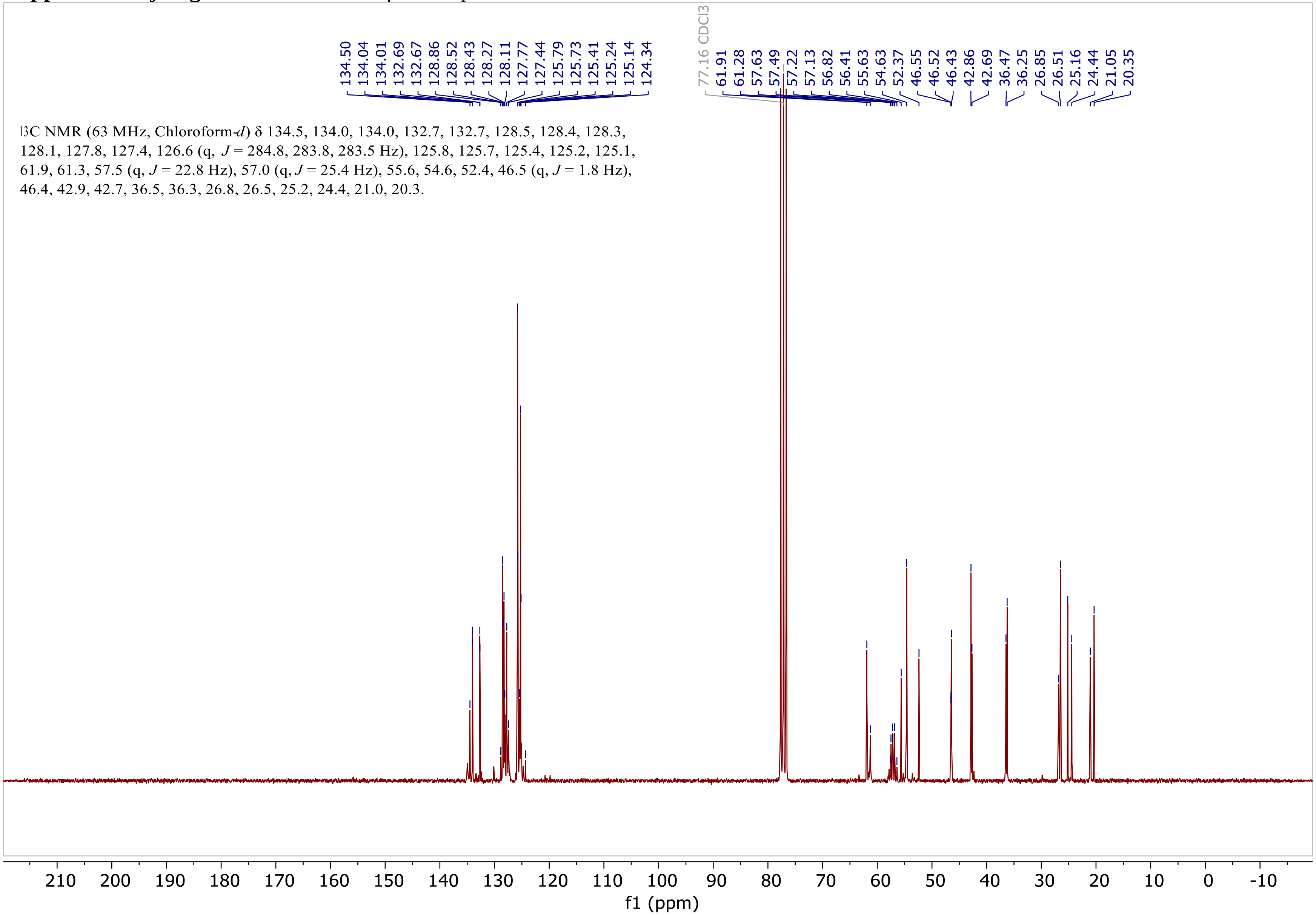

Supplementary Figure 351: *IR for compound **71***

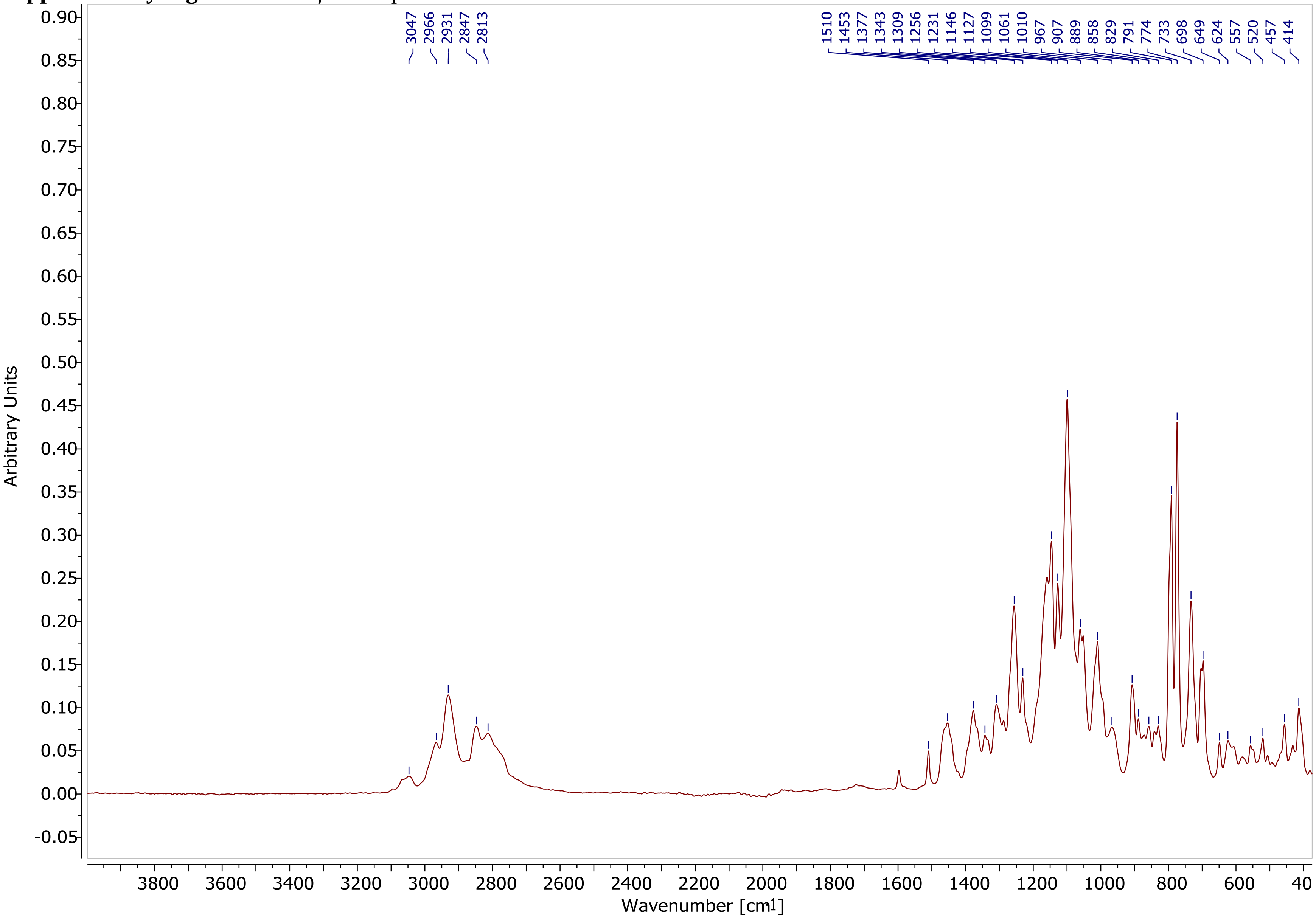

Supplementary Figure 352: *^{1}H-NMR for compound* ***72***

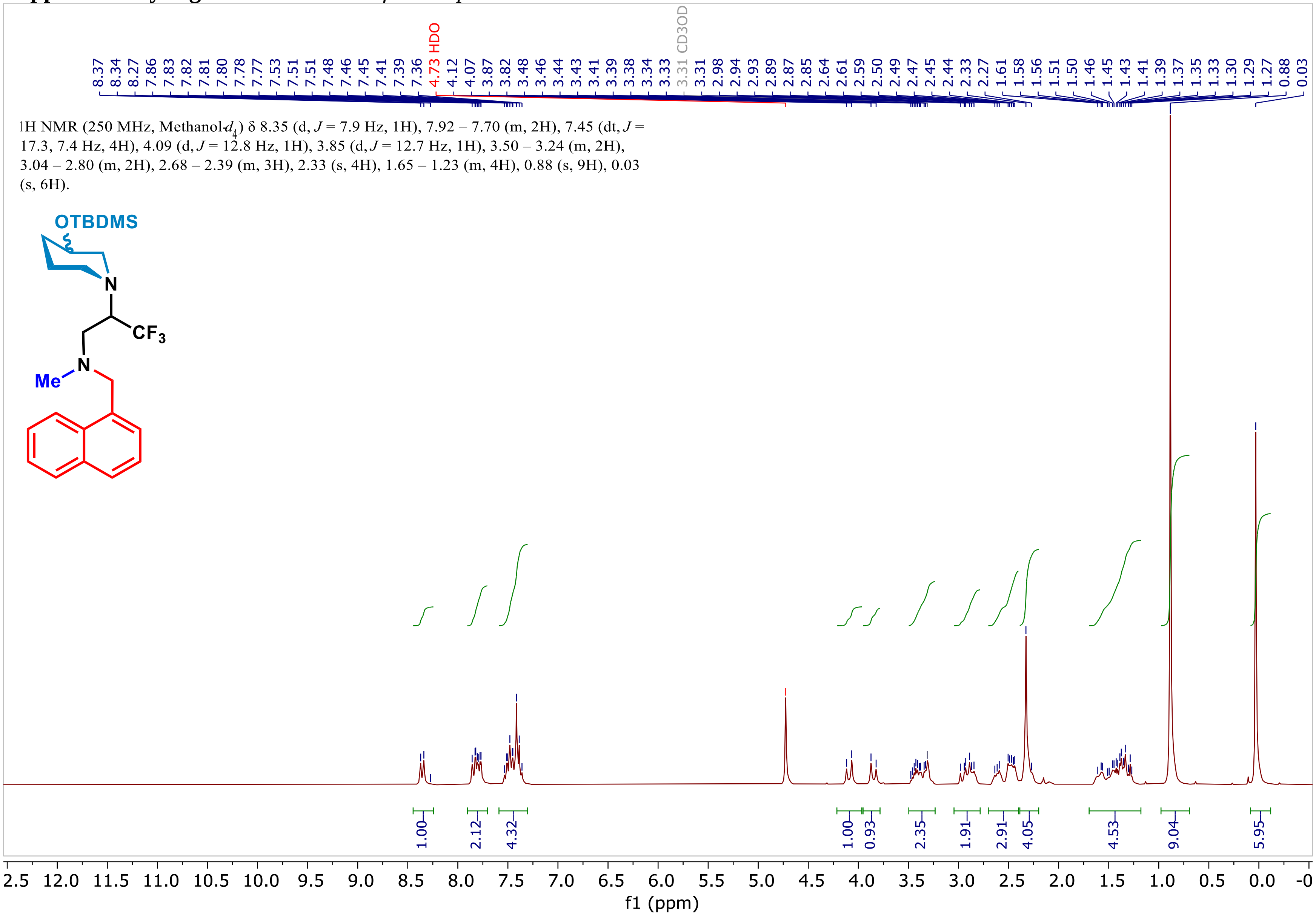

Supplementary Figure 353: *^{19}F NMR for compound* ***72***

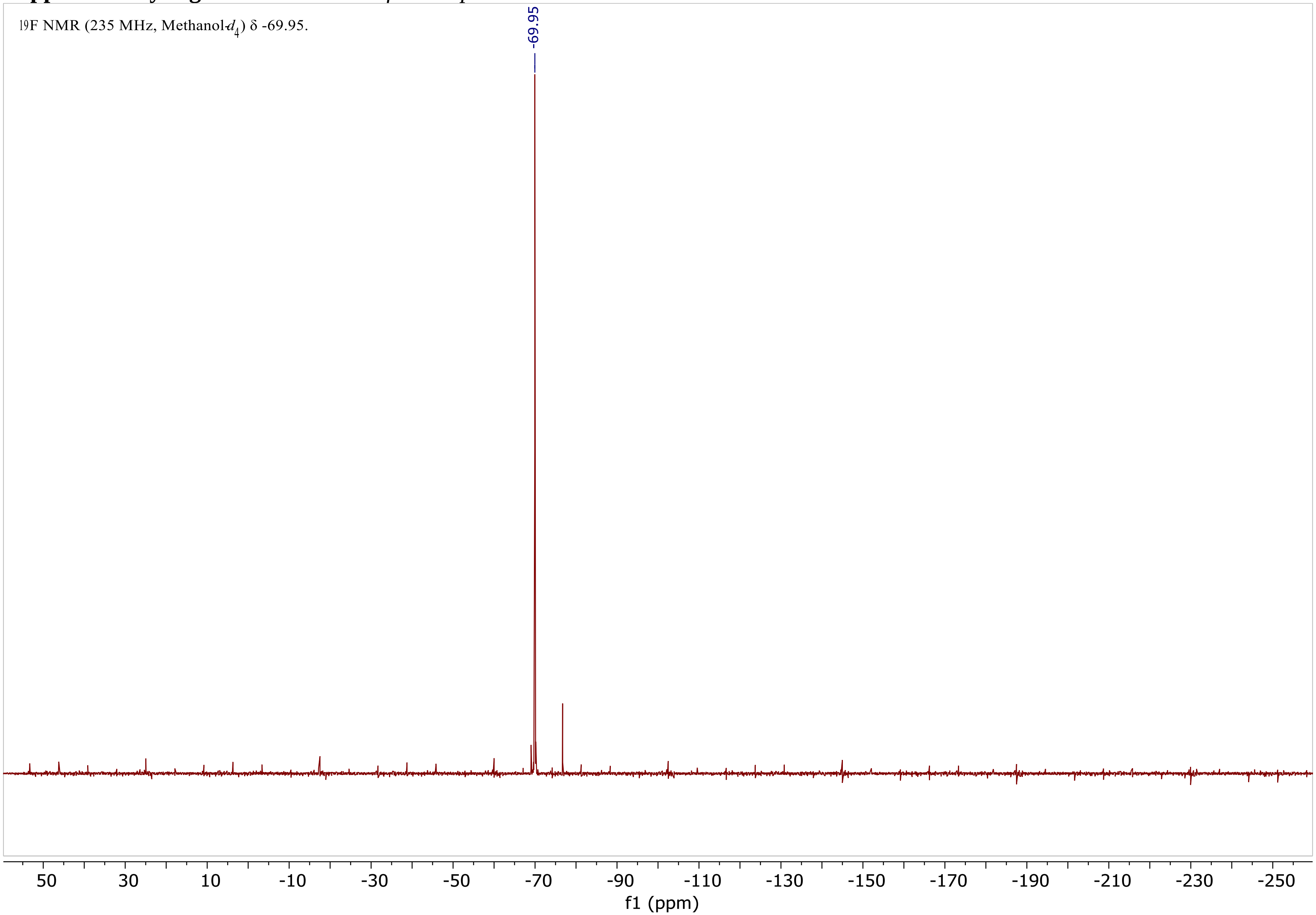

Supplementary Figure 354: *^{13}C NMR for compound* ***72***

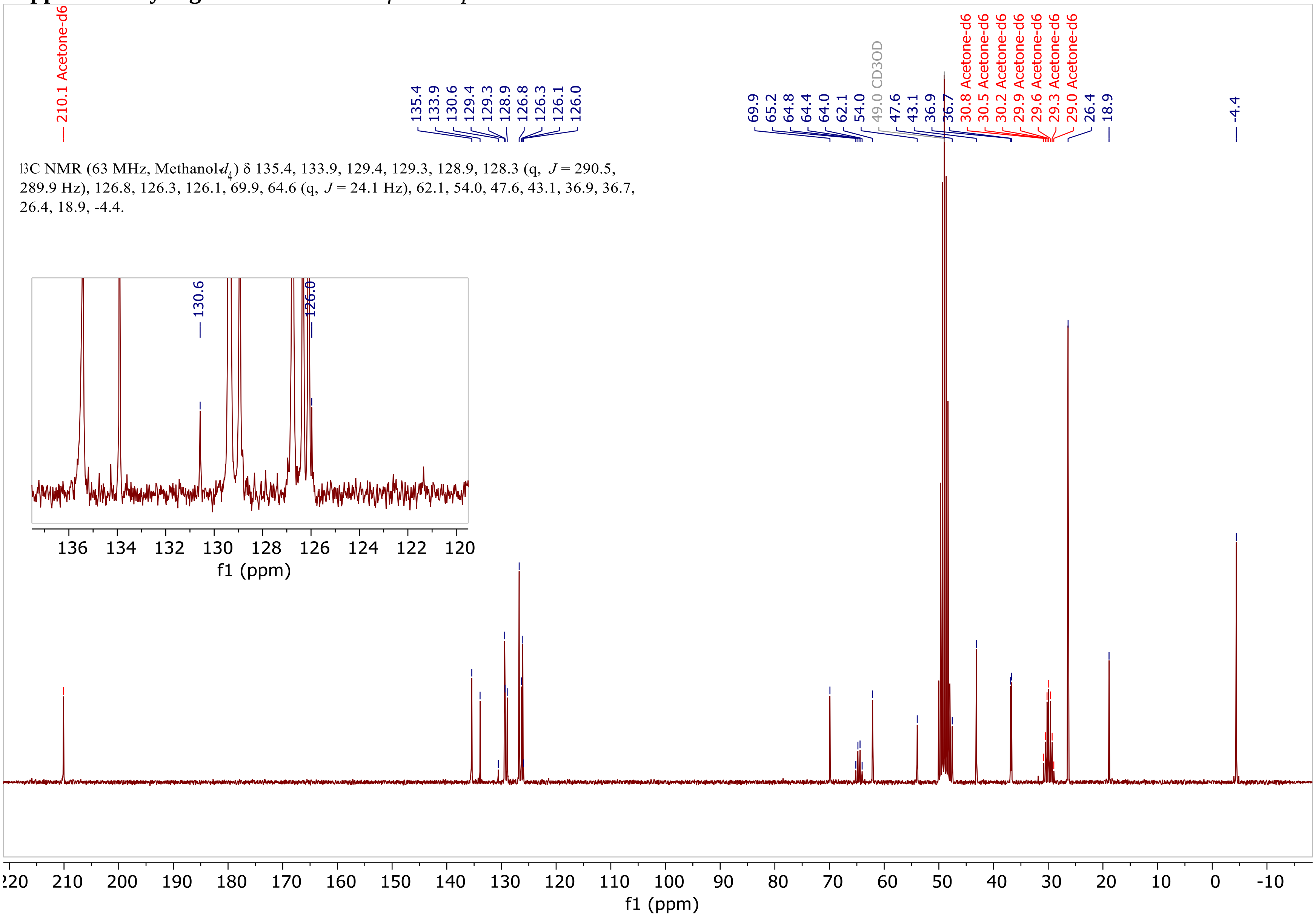

Supplementary Figure 355: *IR for compound* ***7****2*

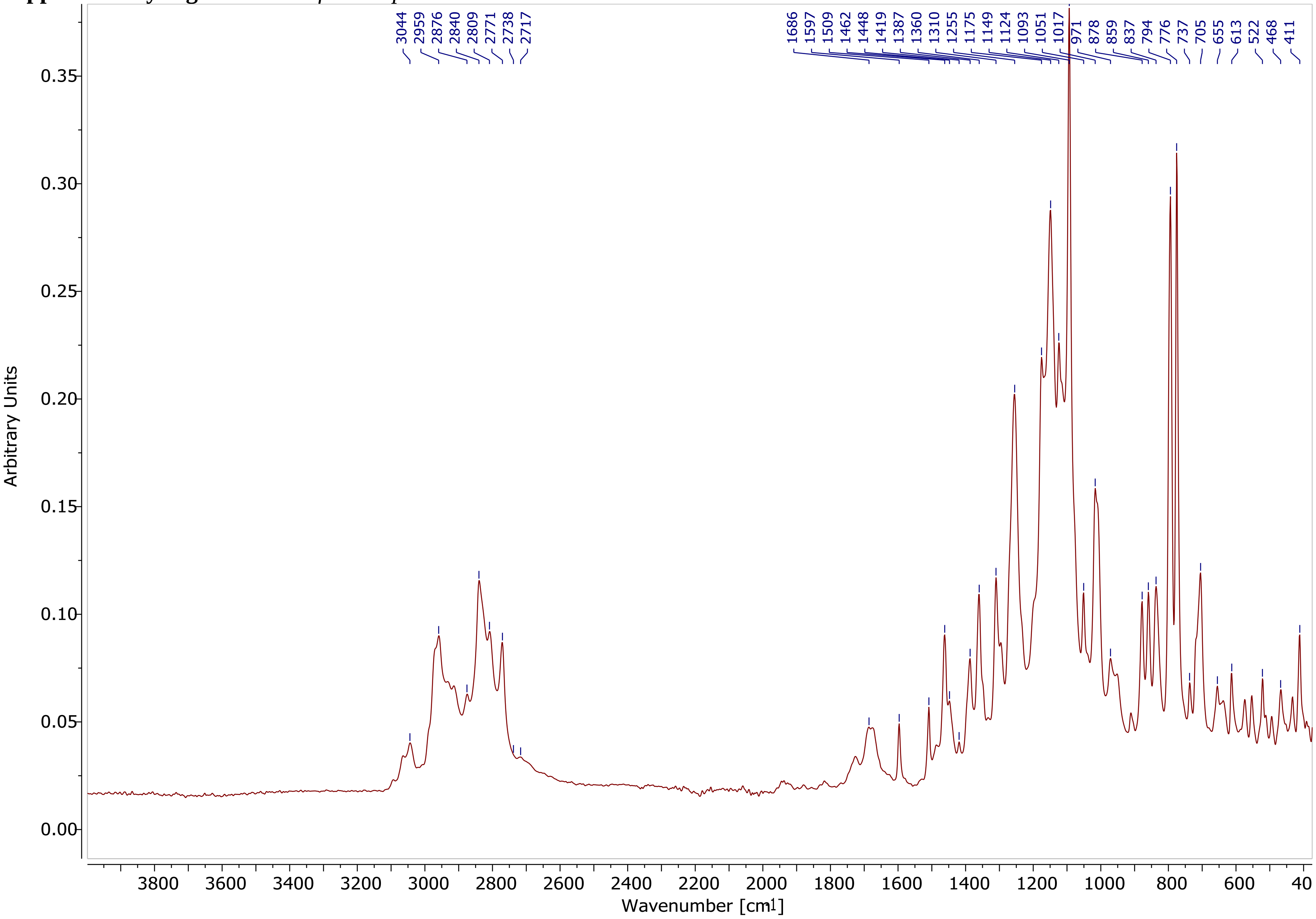

Supplementary Figure 356: *¹H-NMR for compound **73***

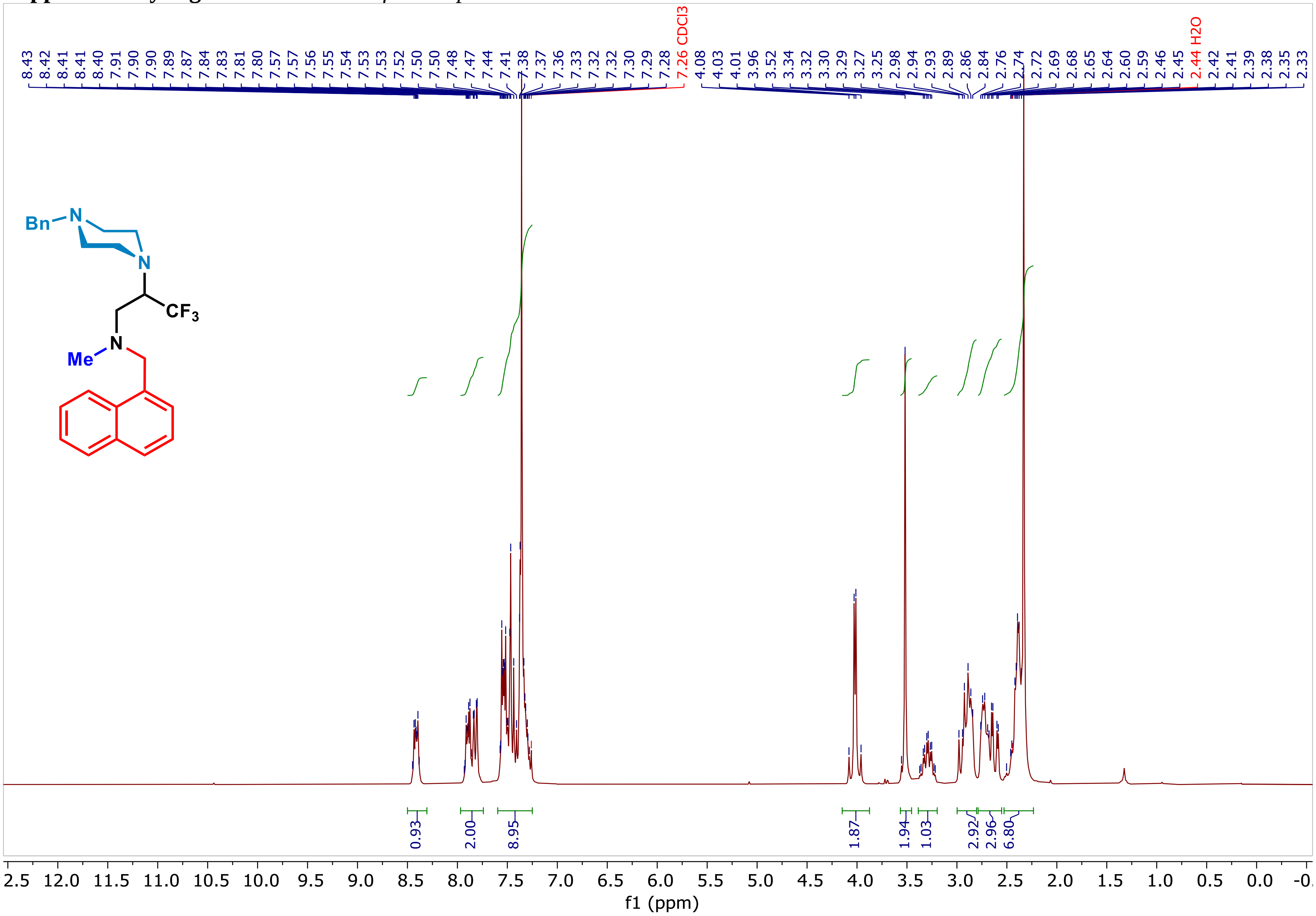

Supplementary Figure 357: *^{19}F NMR for compound* ***73***

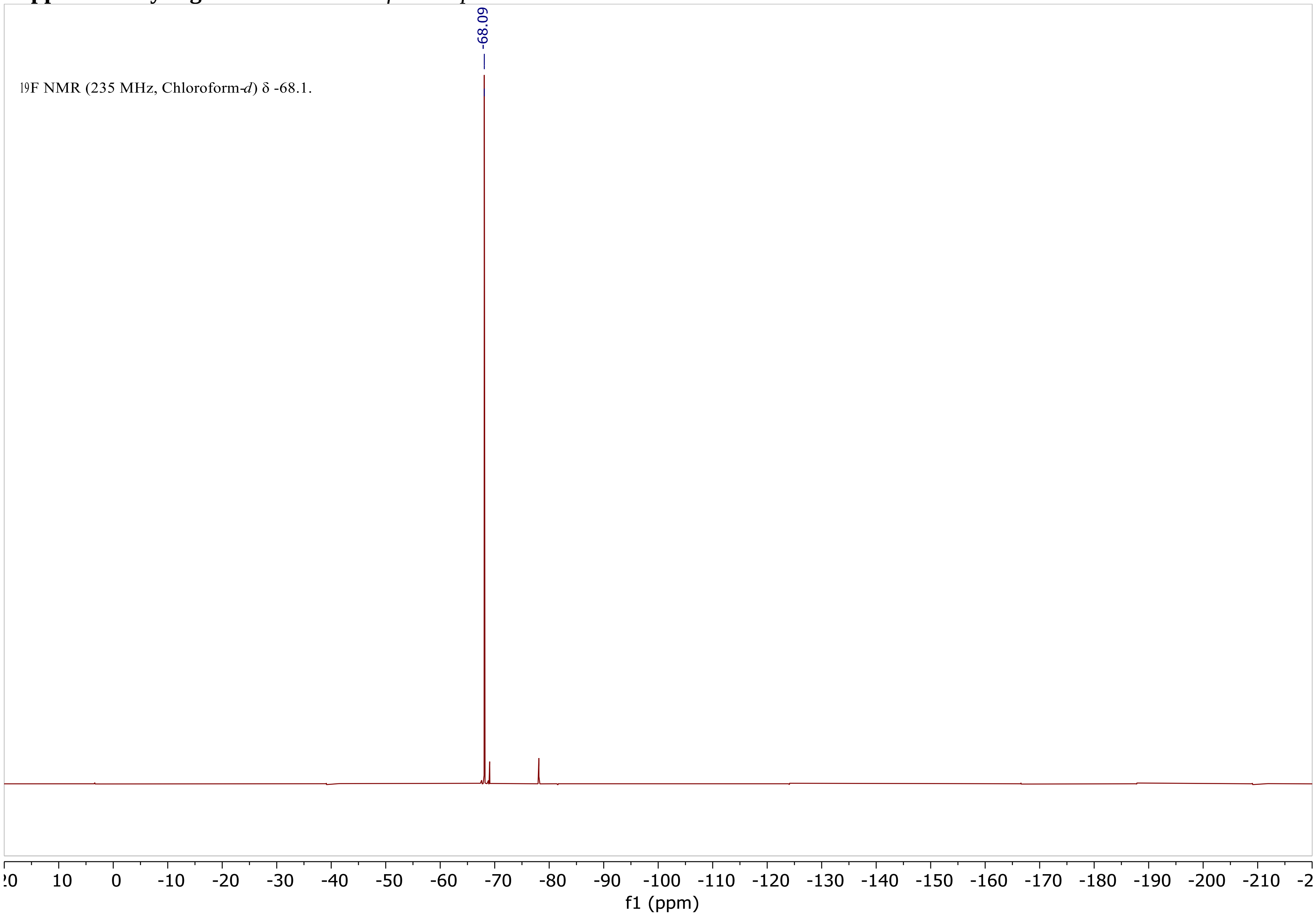

Supplementary Figure 358: *^{13}C NMR for compound* ***73***

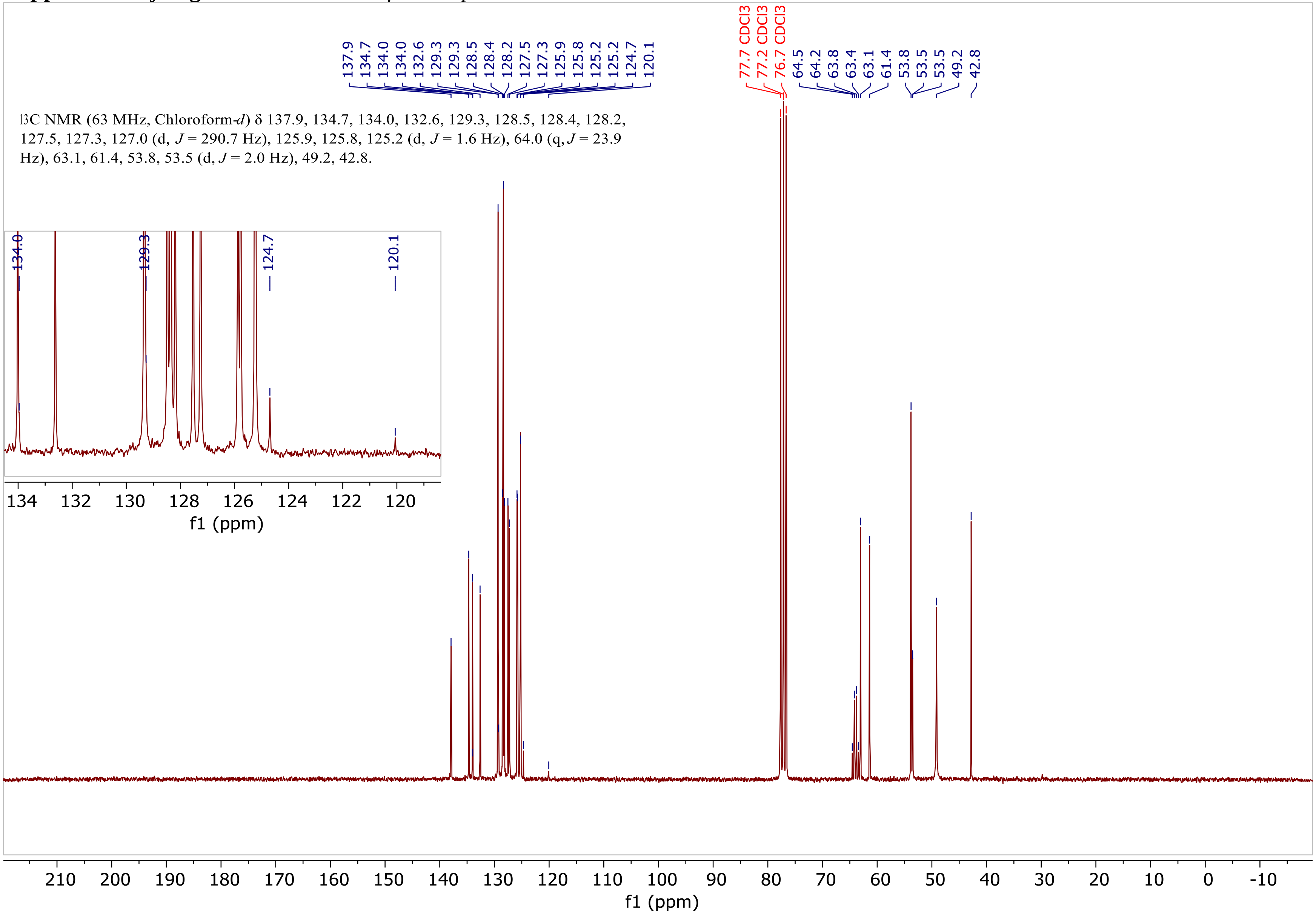

Supplementary Figure 359: *IR for compound* ***73***

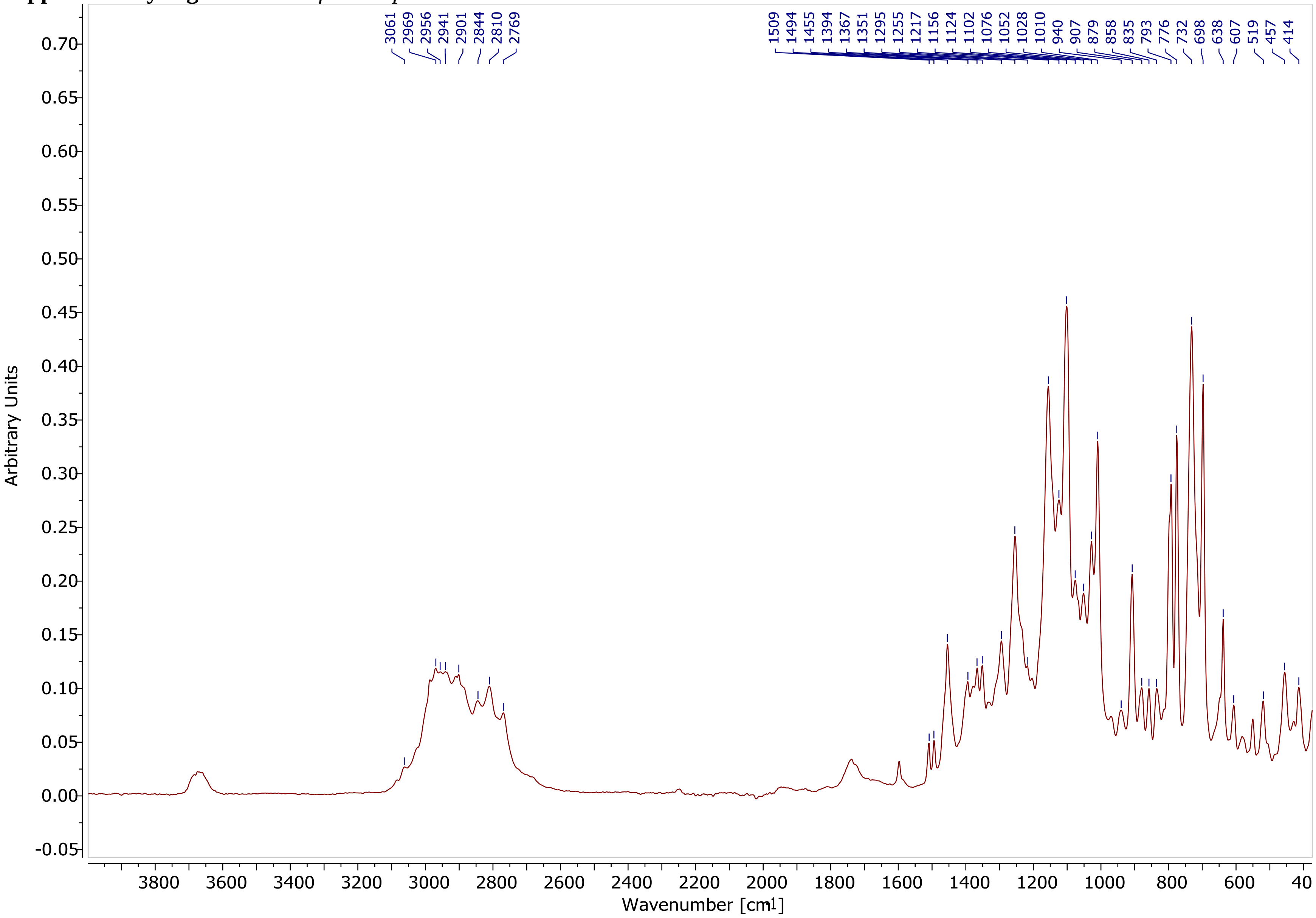

Supplementary Figure 360: *^{1}H-NMR for compound* ***74***

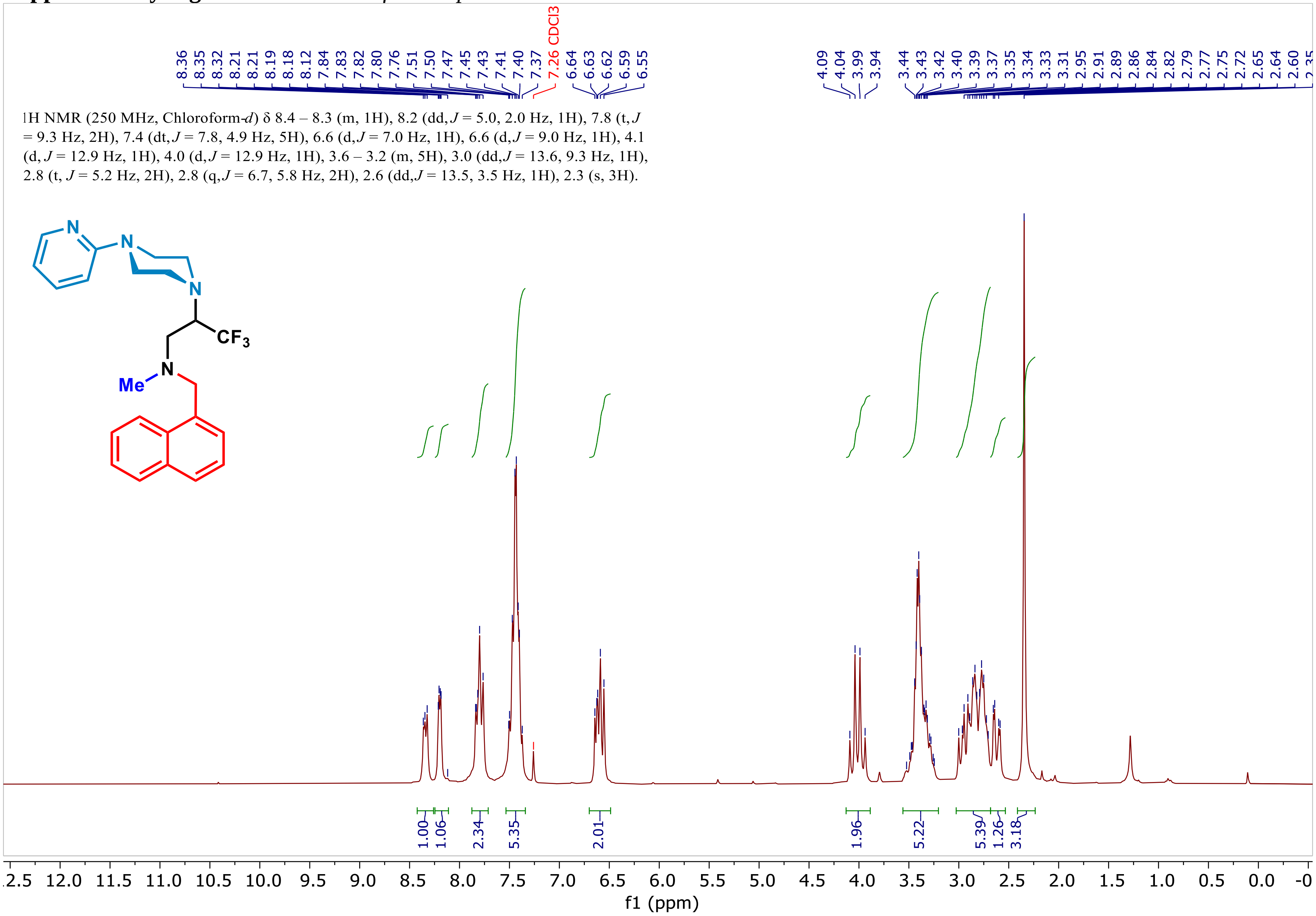

Supplementary Figure 361: *^{19}F NMR for compound* ***74***

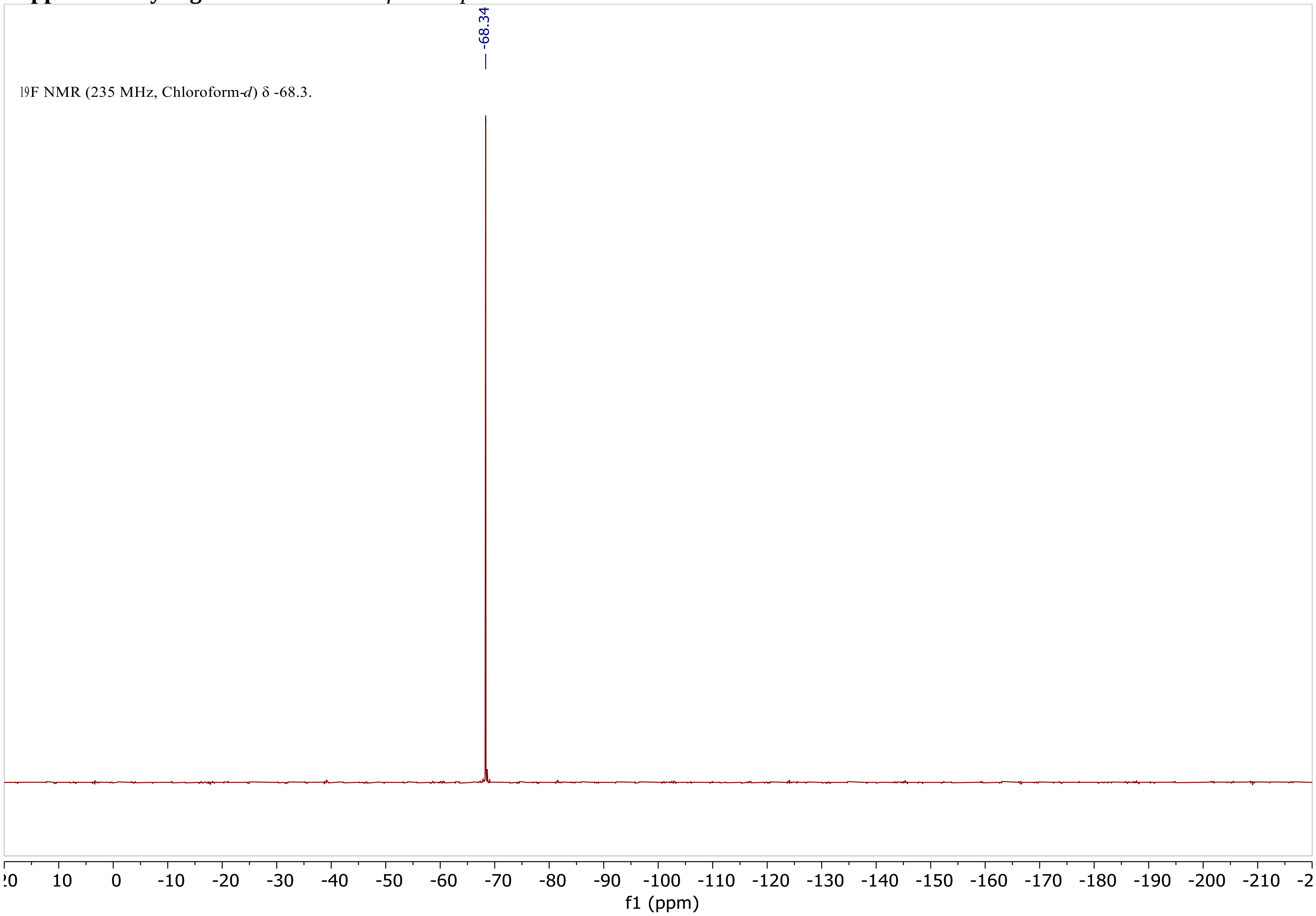

Supplementary Figure 362: *^{13}C NMR for compound* ***74***

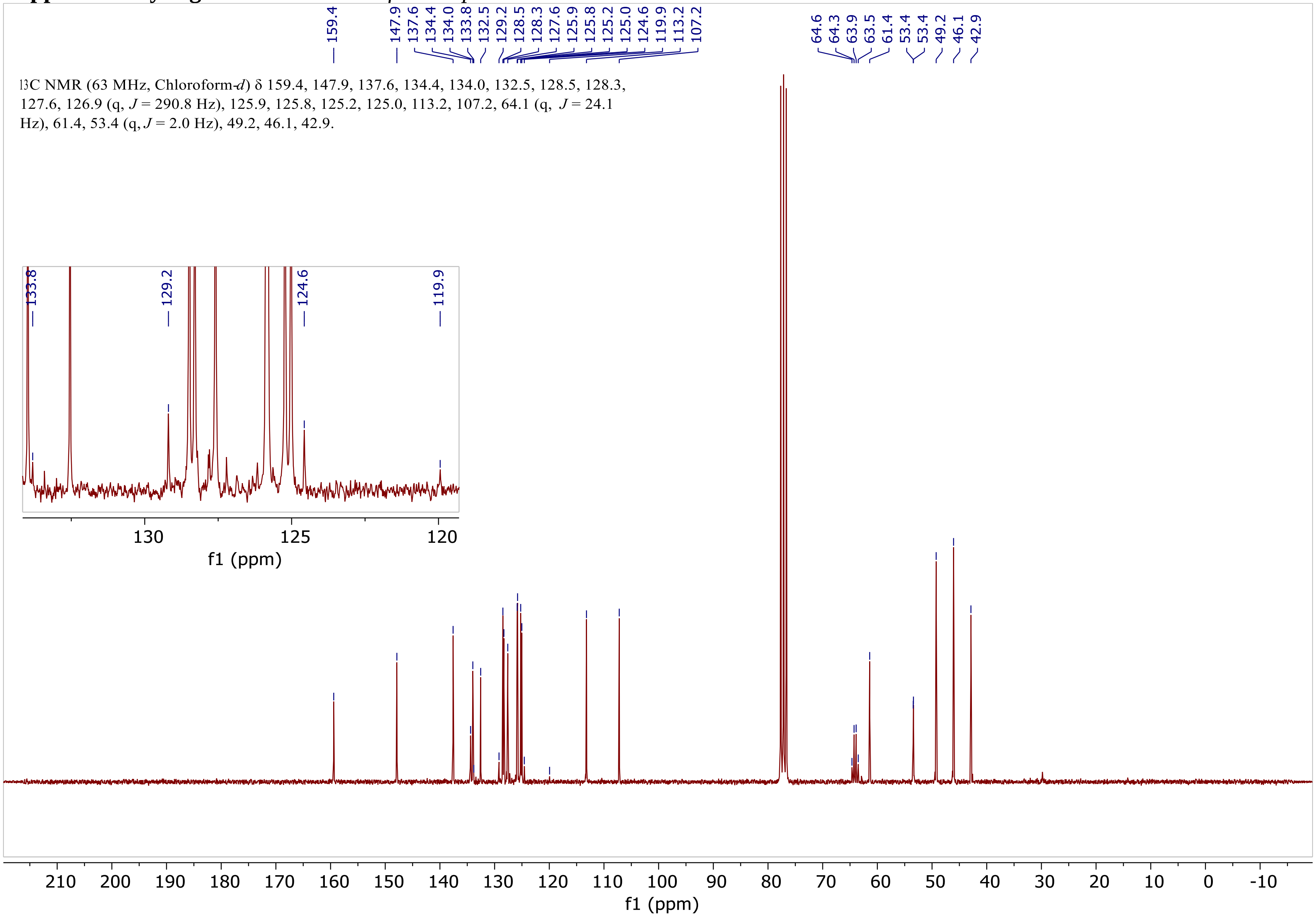

Supplementary Figure 363: *IR for compound* ***74***

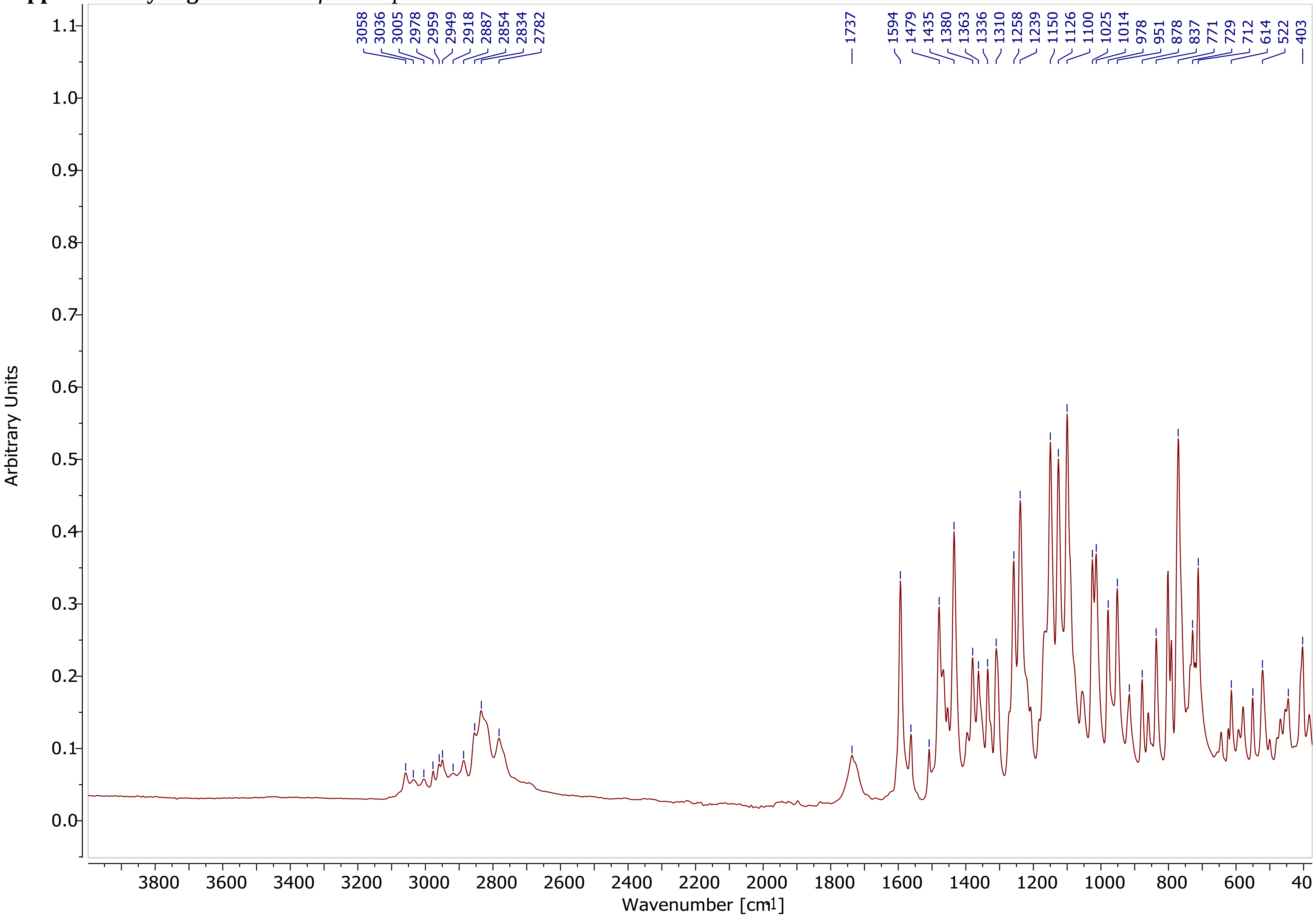

Supplementary Figure 364: *^{1}H-NMR for compound* ***75***

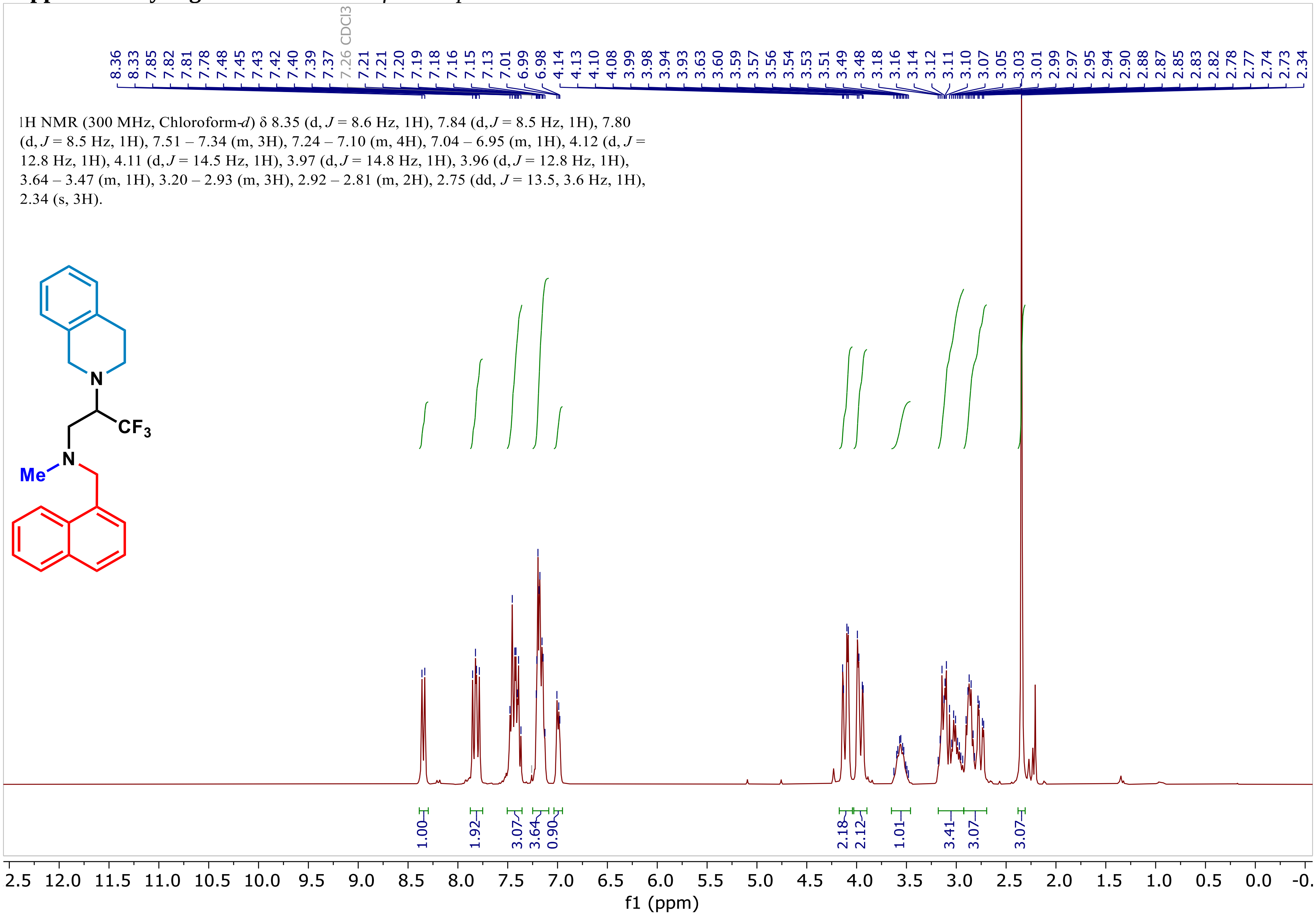

Supplementary Figure 365: *^{19}F NMR for compound **75***

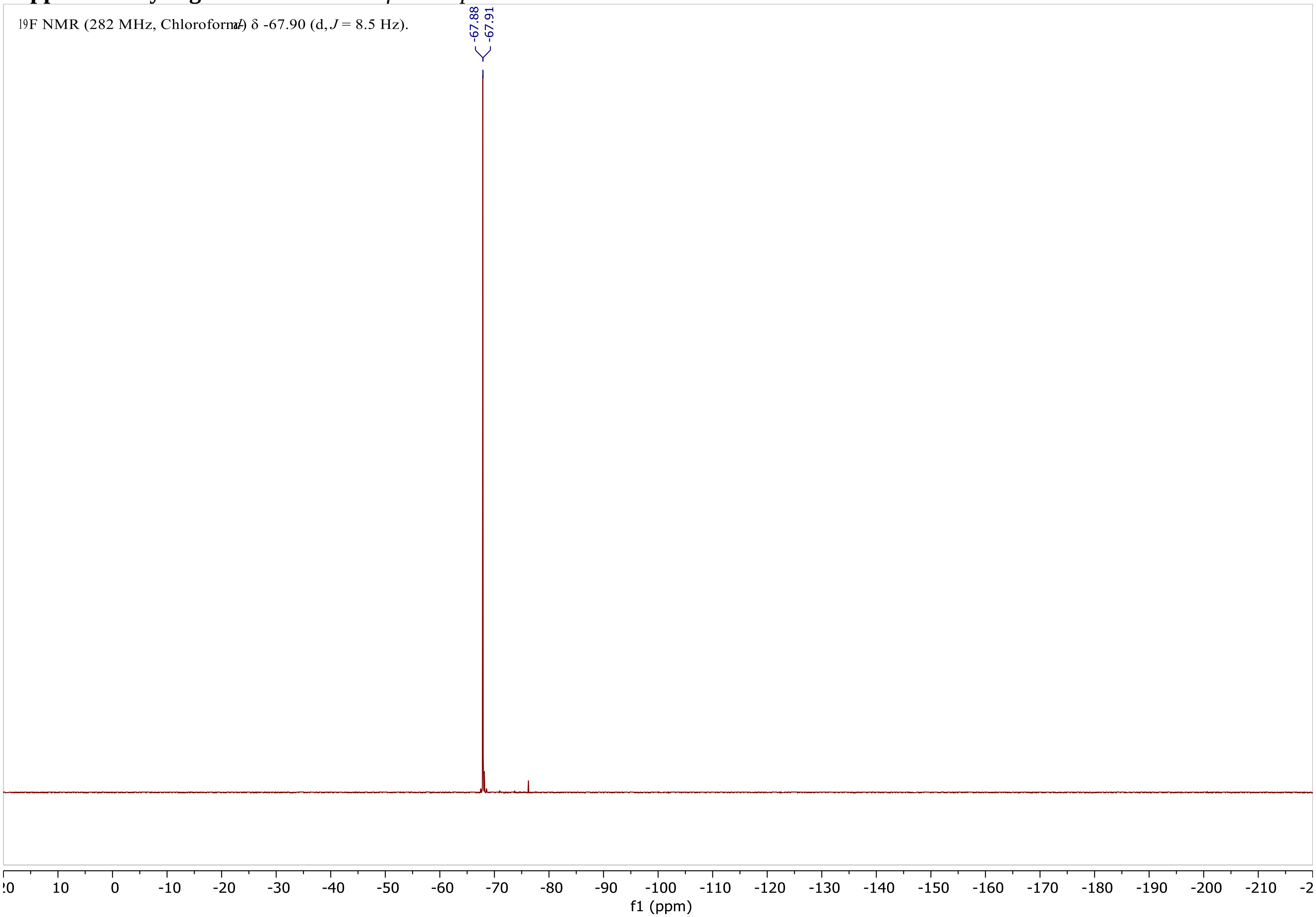

Supplementary Figure 366: *^{13}C NMR for compound **75***

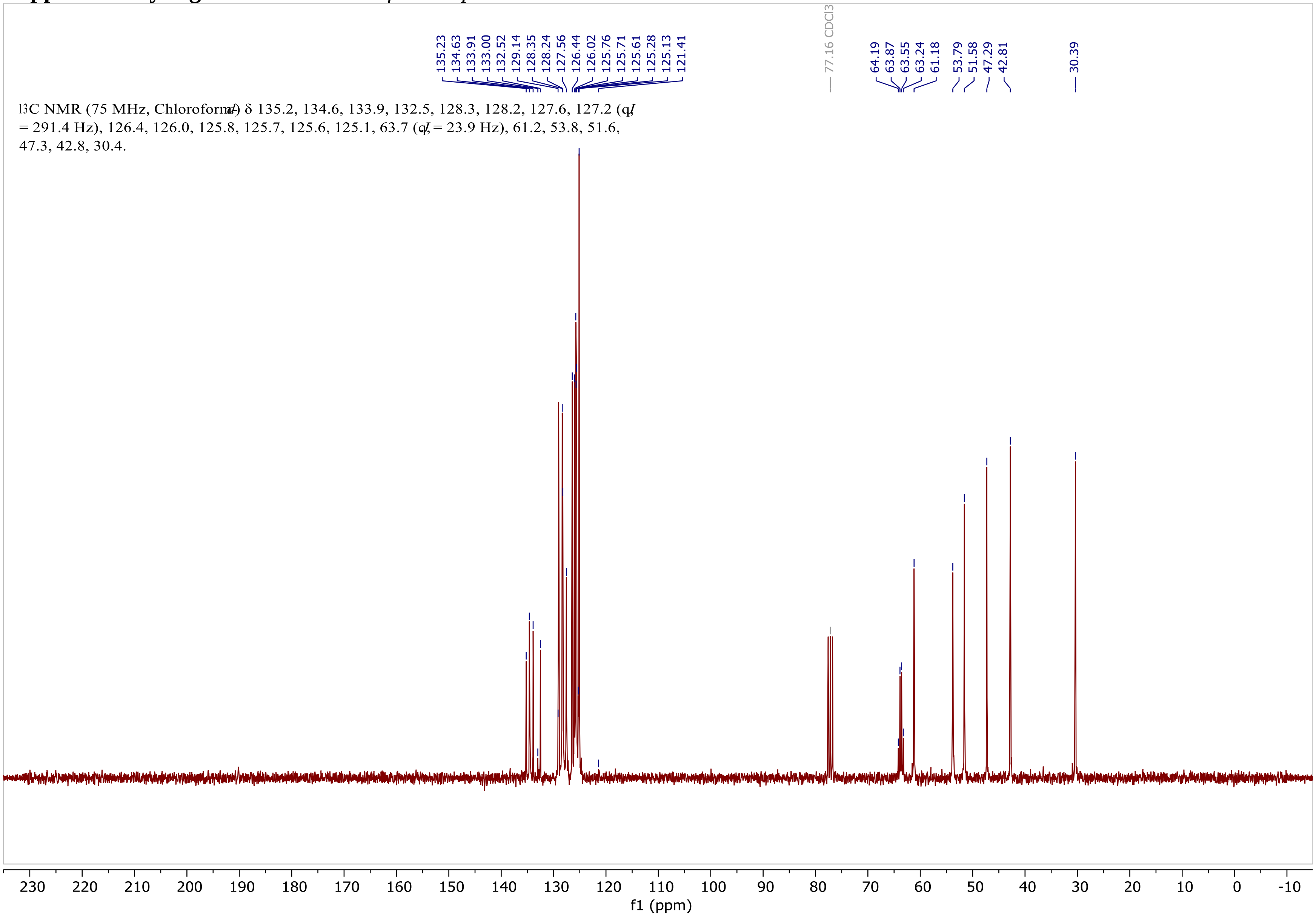

Supplementary Figure 367: *IR for compound **75***

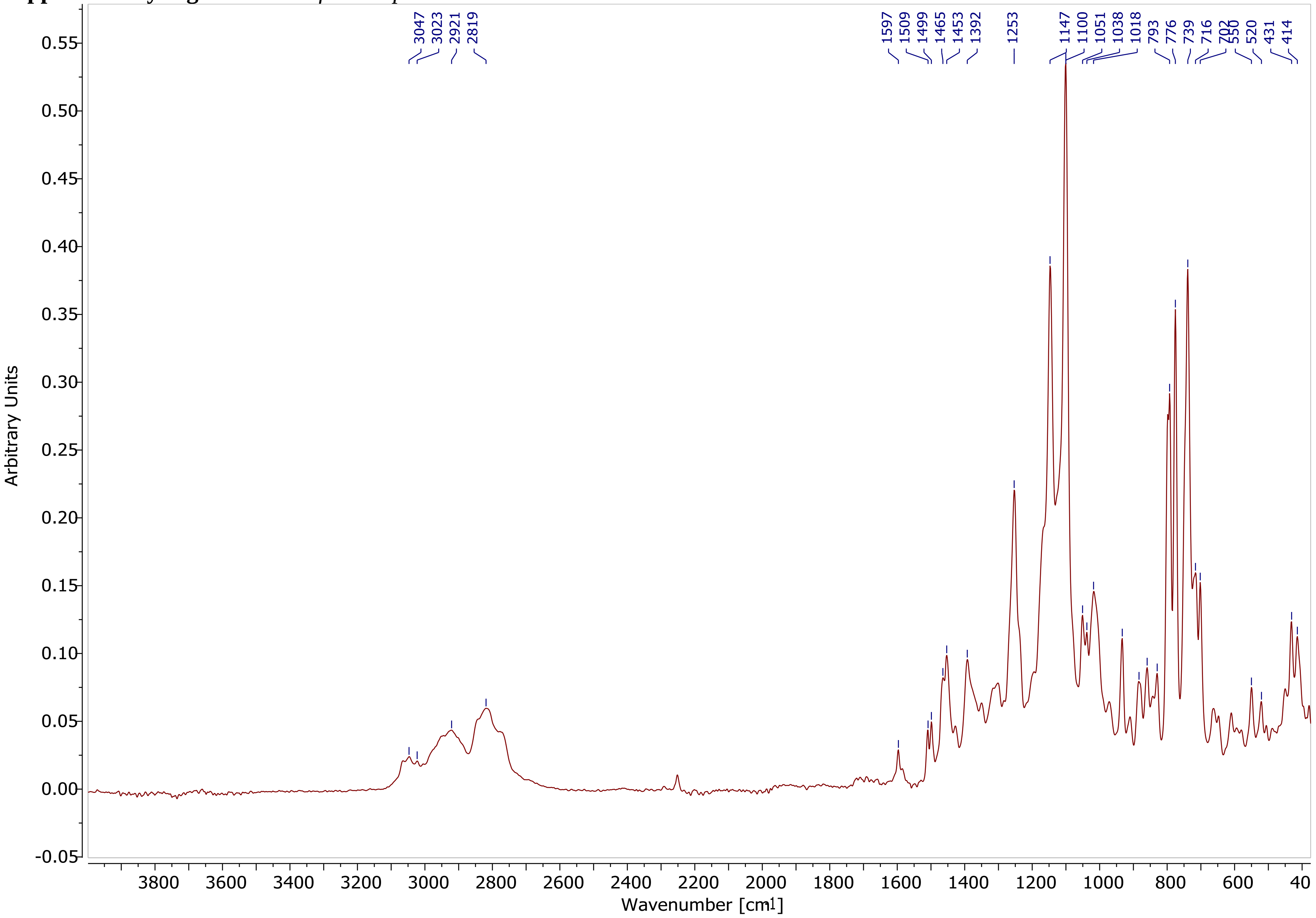

Supplementary Figure 368: *¹H-NMR for compound **76***

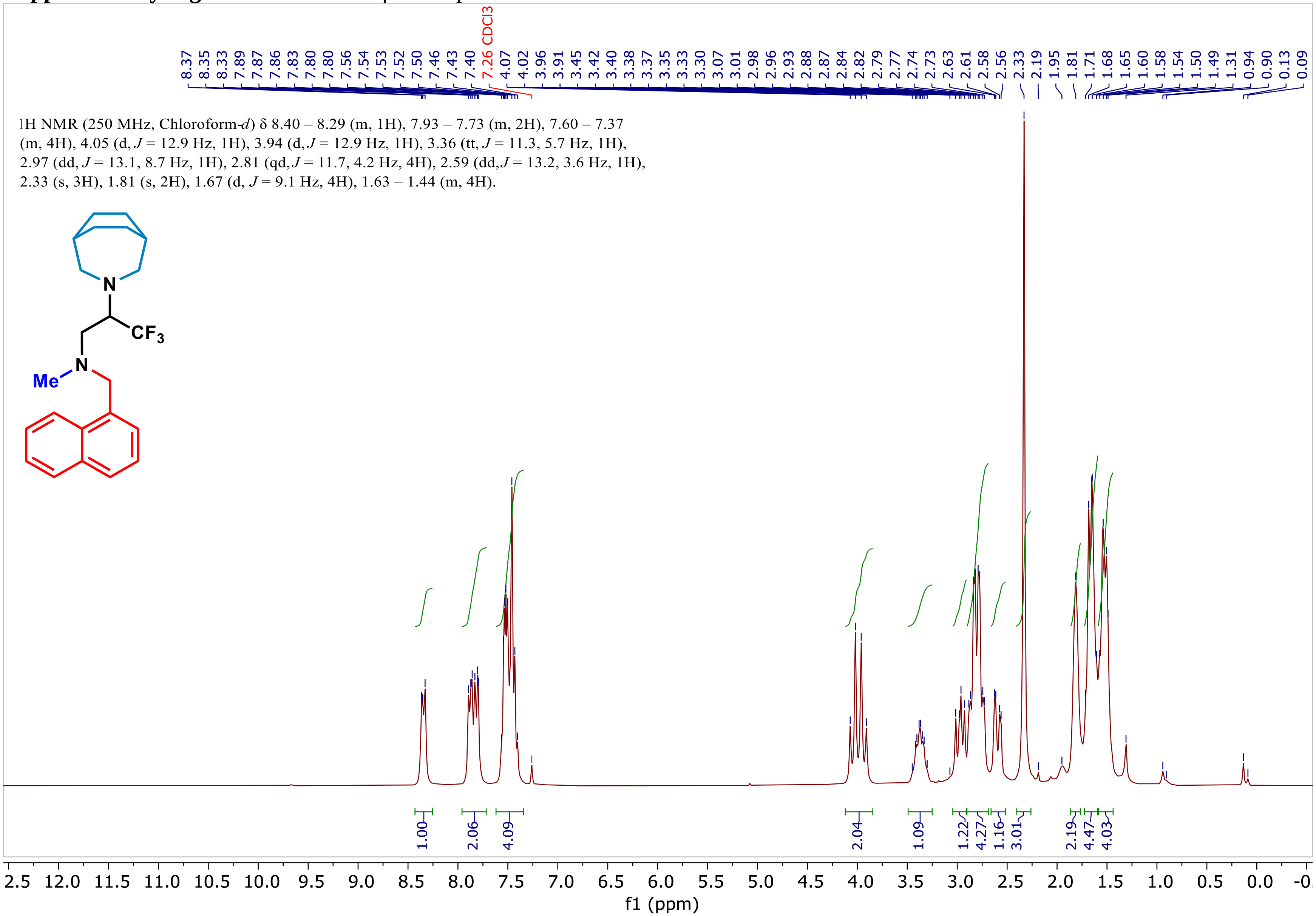

Supplementary Figure 369: *^{19}F NMR for compound* ***76***

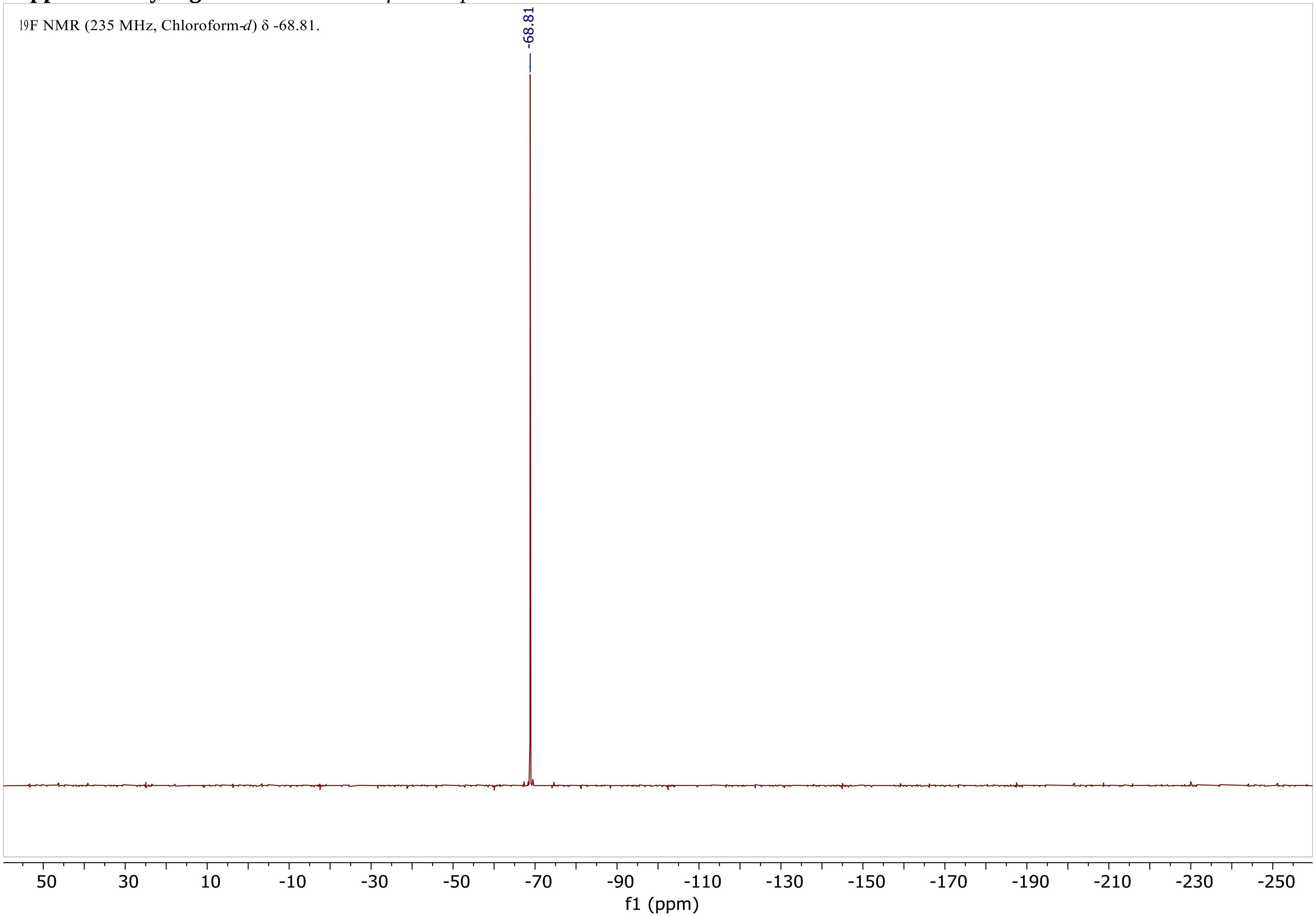

Supplementary Figure 370: *^{13}C NMR for compound* ***76***

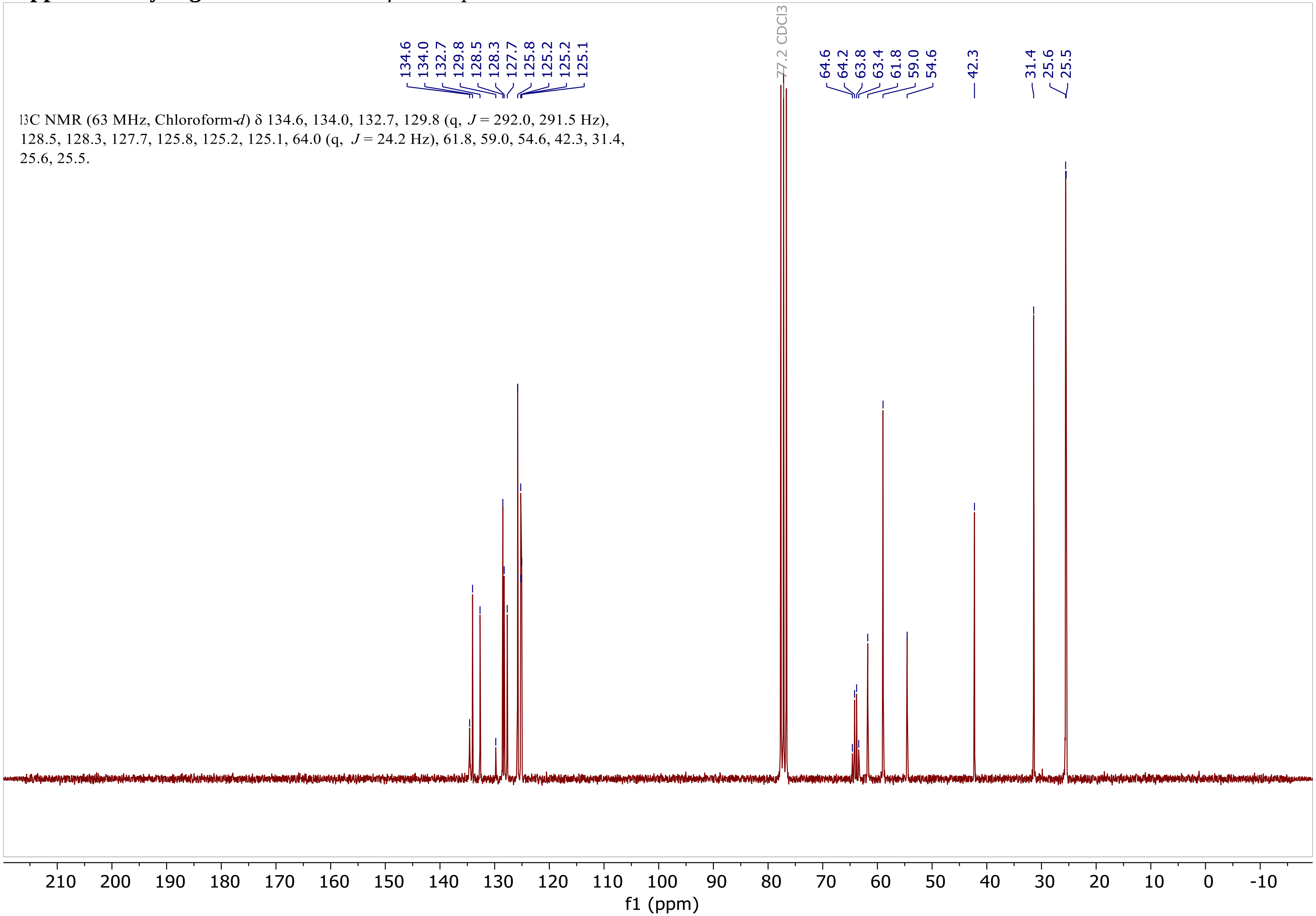

Supplementary Figure 371: *IR for compound **76***

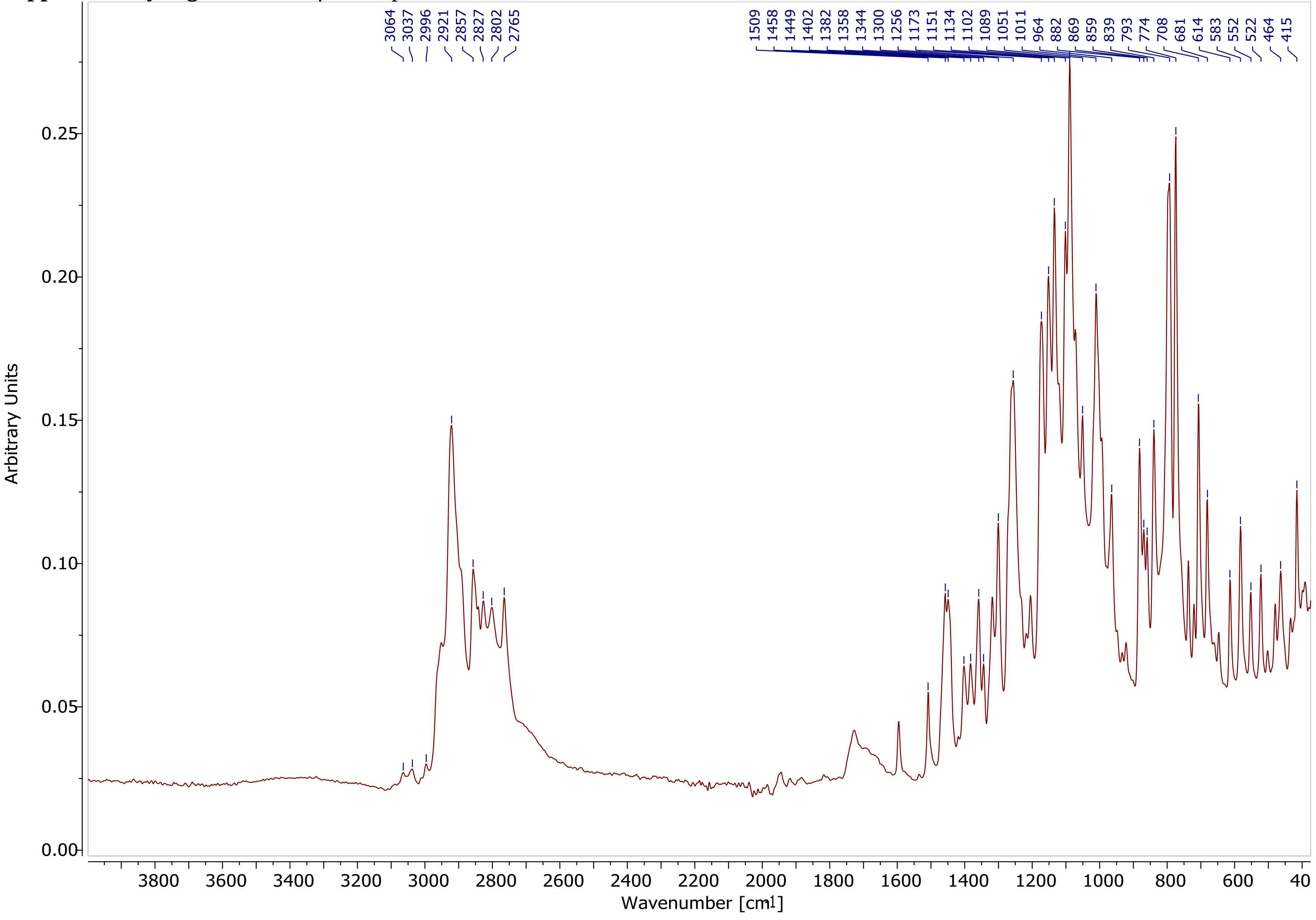

Supplementary Figure 372: *^{1}H-NMR for compound **77***

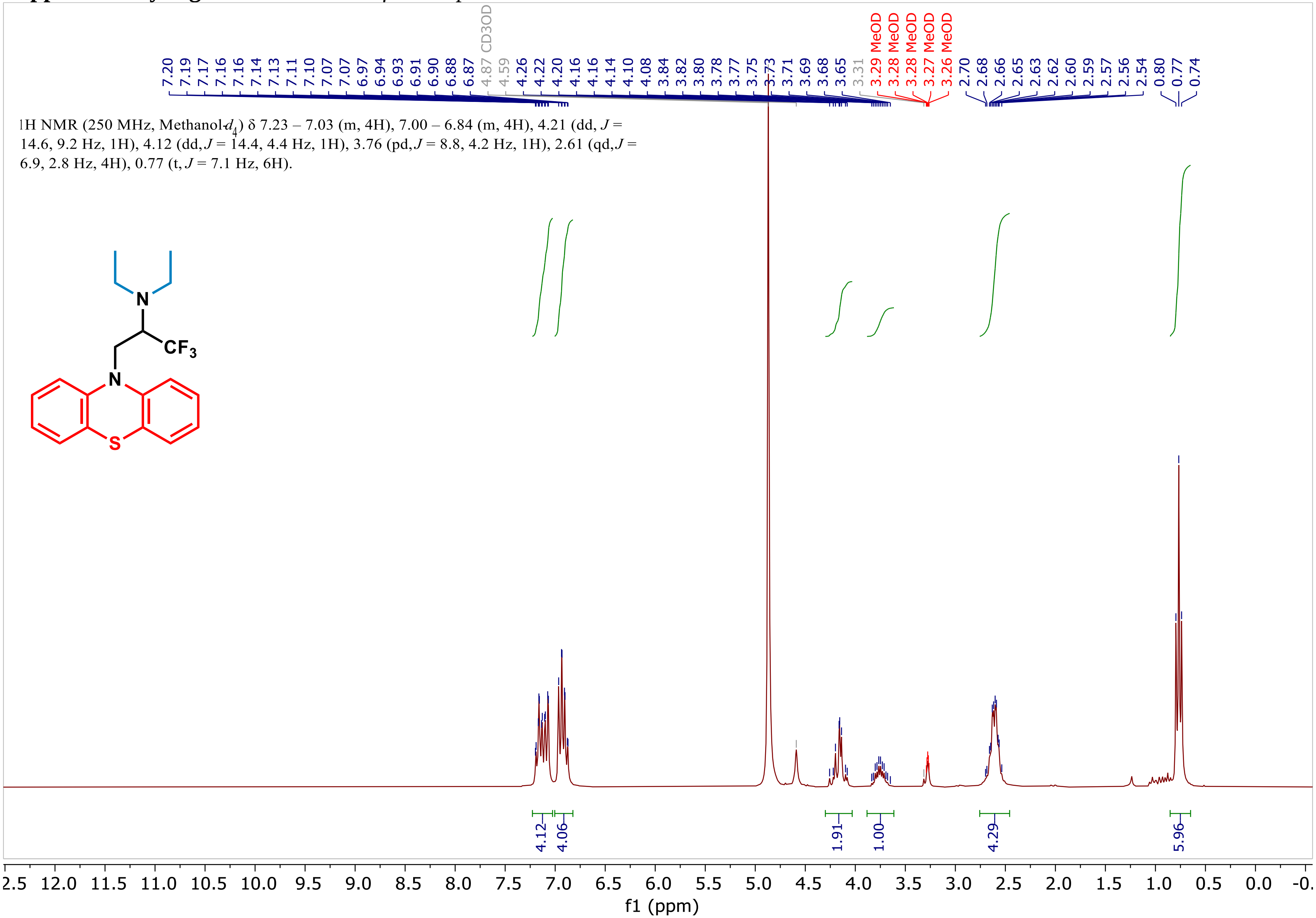

Supplementary Figure 373: *^{19}F NMR for compound* ***77***

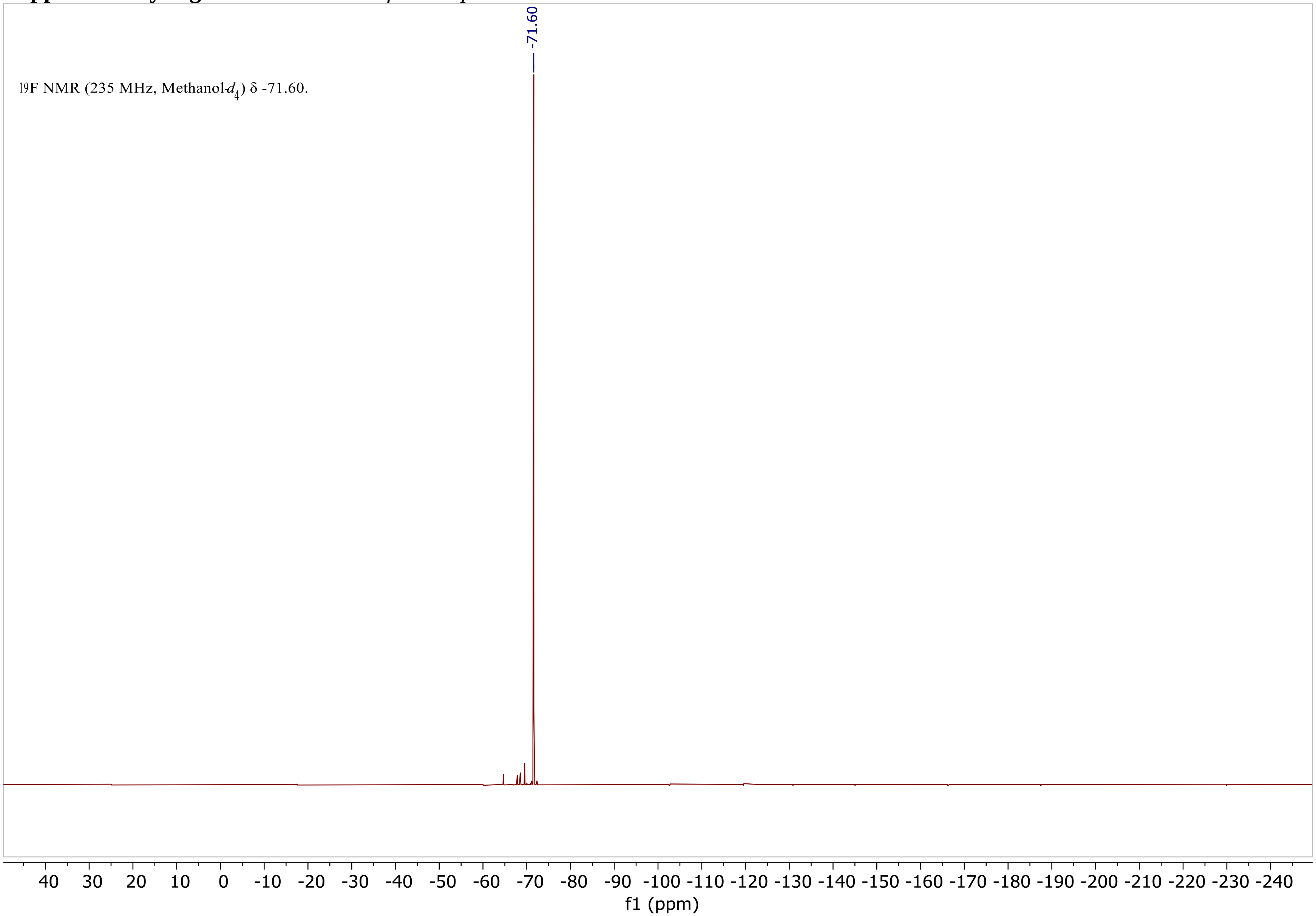

Supplementary Figure 374: *^{13}C NMR for compound* ***77***

13C NMR (63 MHz, Methanol-d_4) δ 145.1, 127.6, 127.5, 127.4 (q, J = 289.0 Hz), 126.4, 123.0, 58.0 (q, J = 24.9 Hz), 44.6, 43.5 (q, J = 1.8 Hz), 13.8.

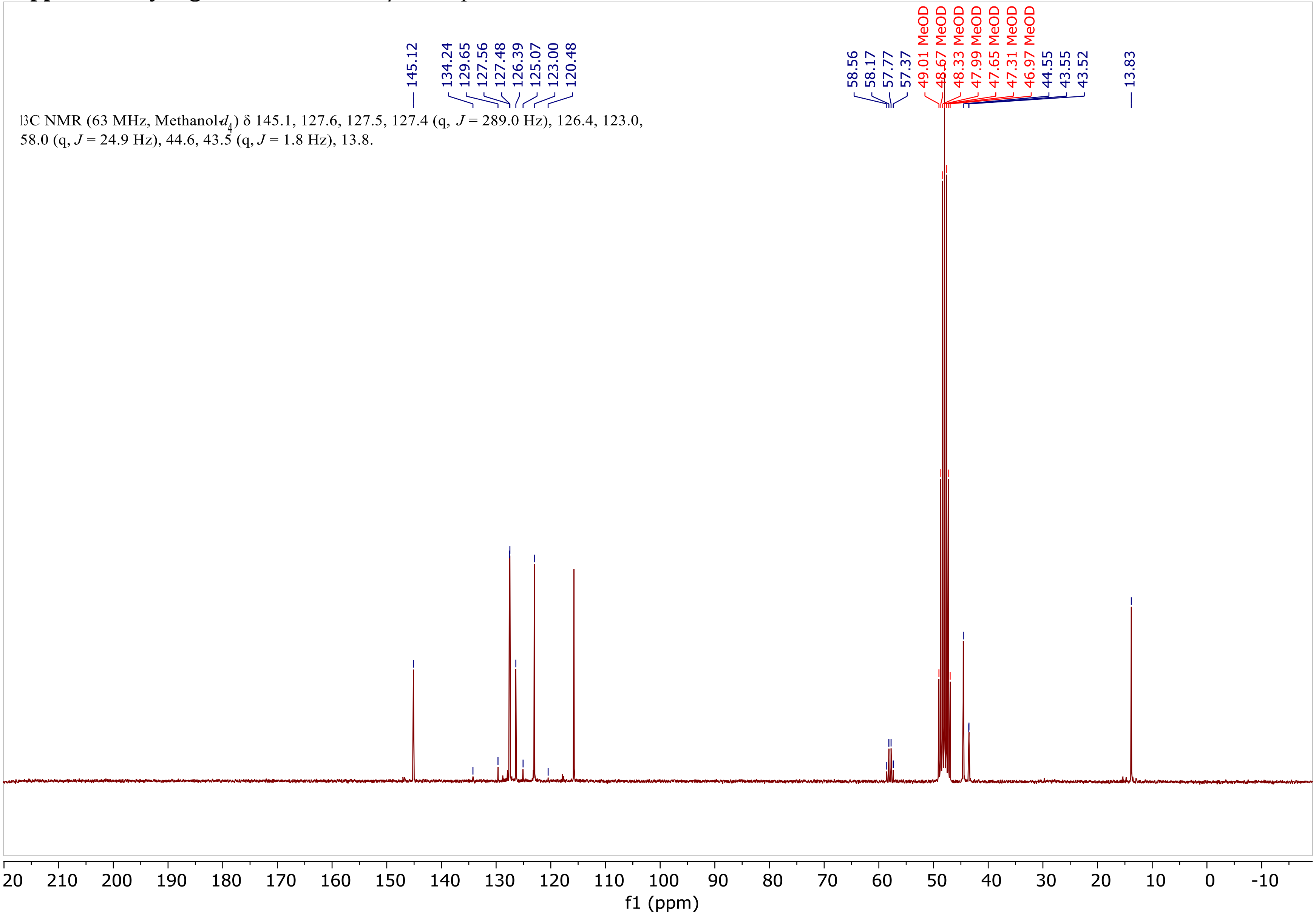

Supplementary Figure 375: *IR for compound **77***

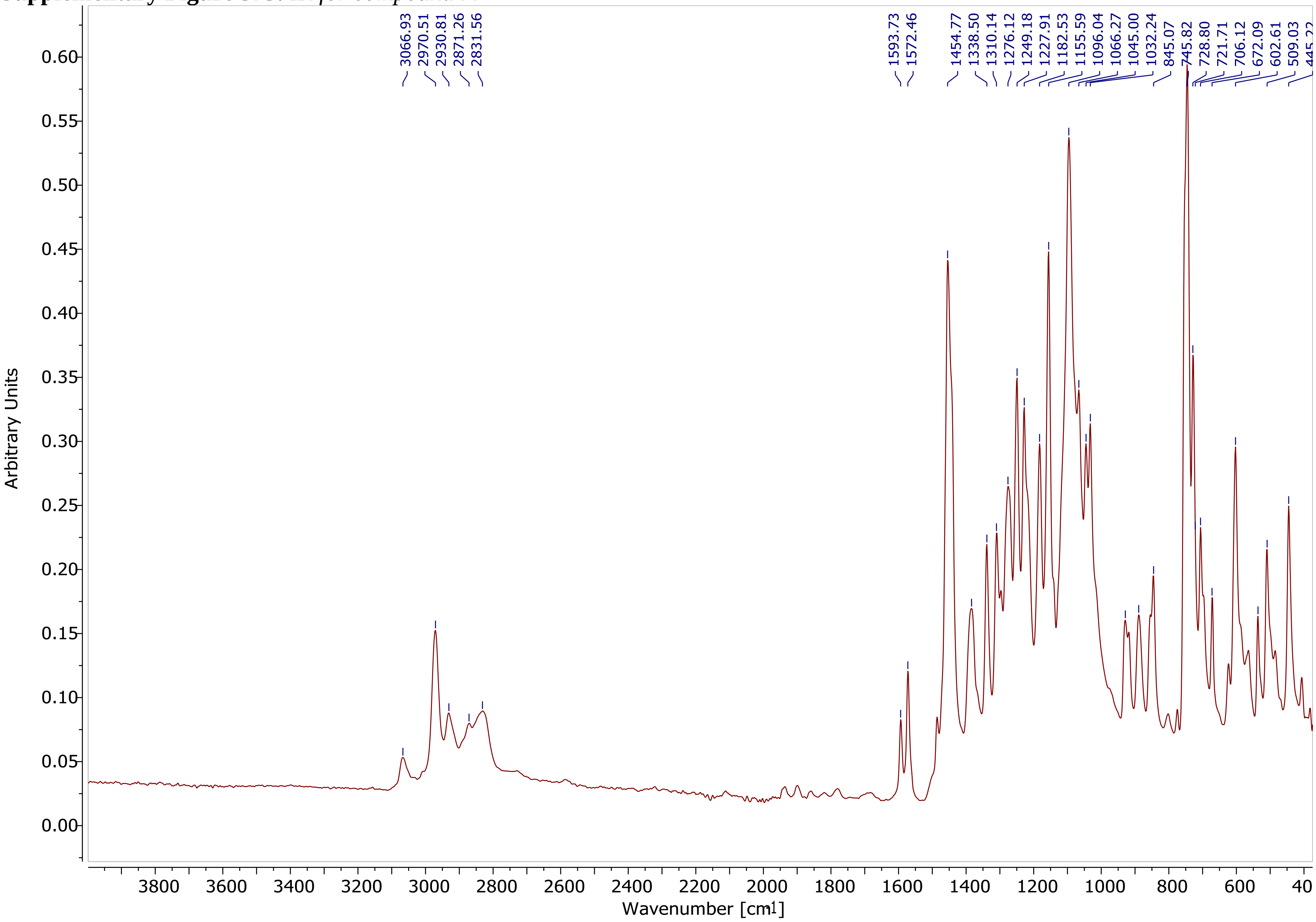

Supplementary Figure 376: *^{1}H-NMR for compound **78***

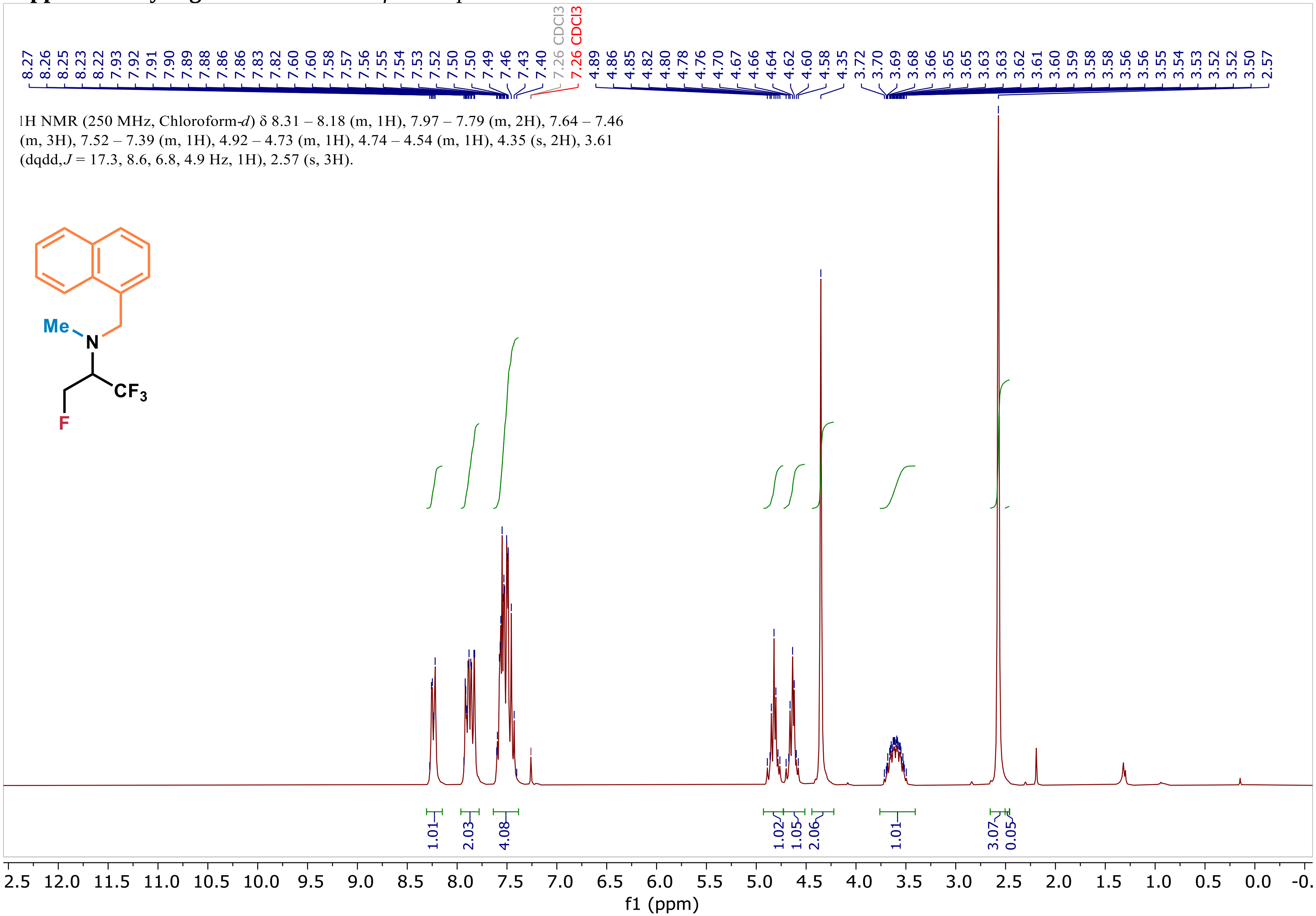

Supplementary Figure 377: *^{19}F NMR for compound* ***78***

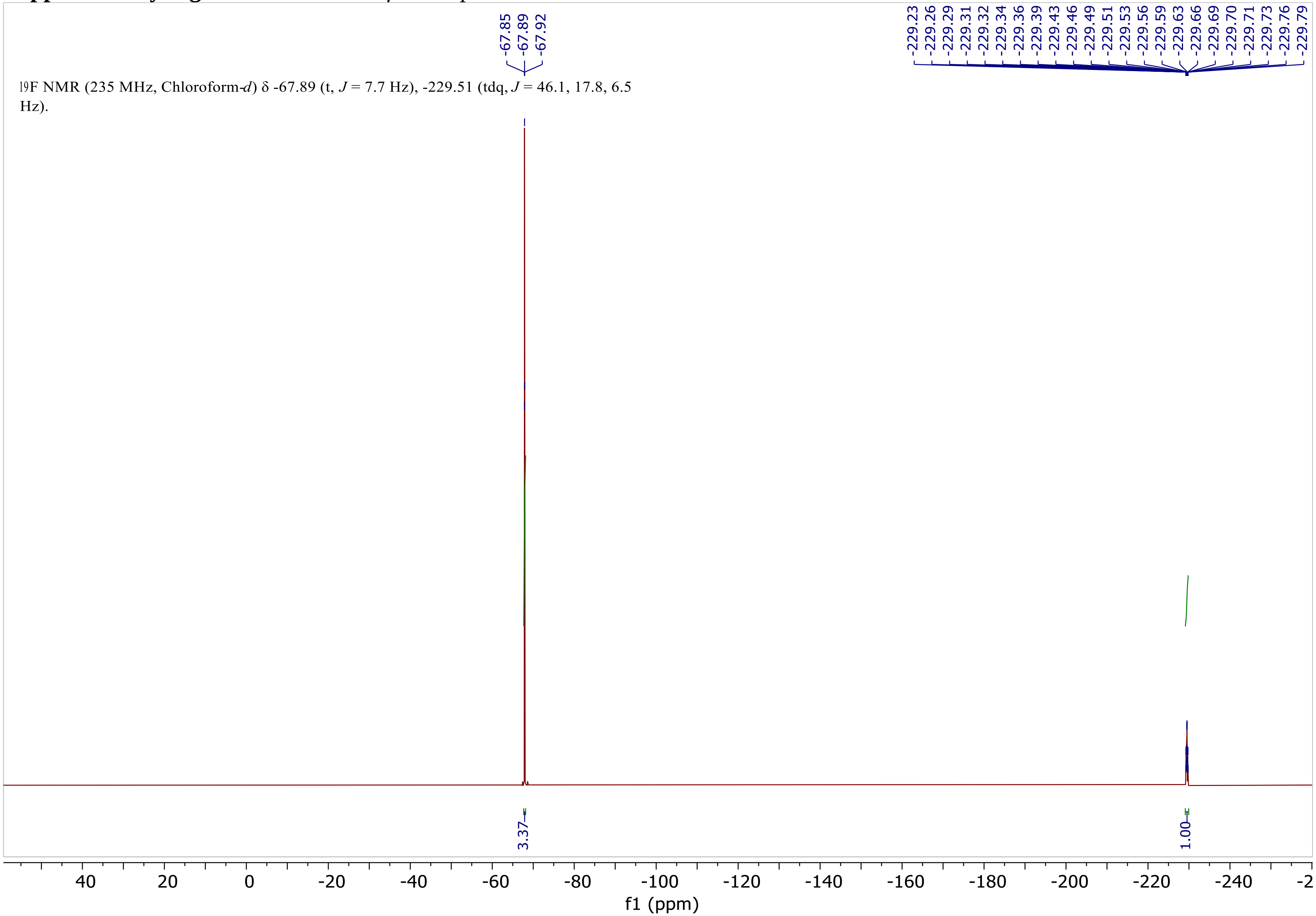

Supplementary Figure 378: *^{13}C NMR for compound* ***78***

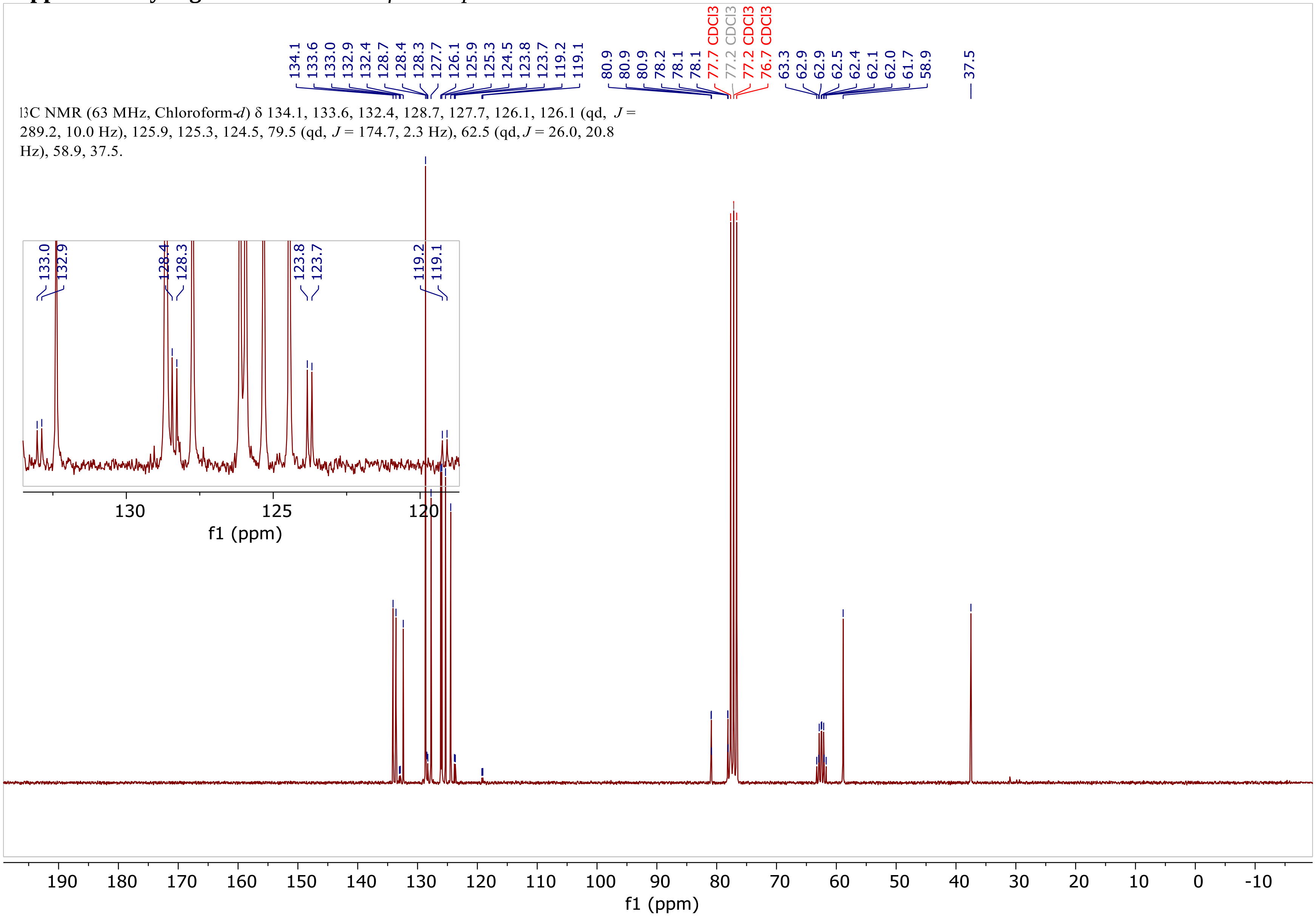

Supplementary Figure 379: *IR for compound* ***78***

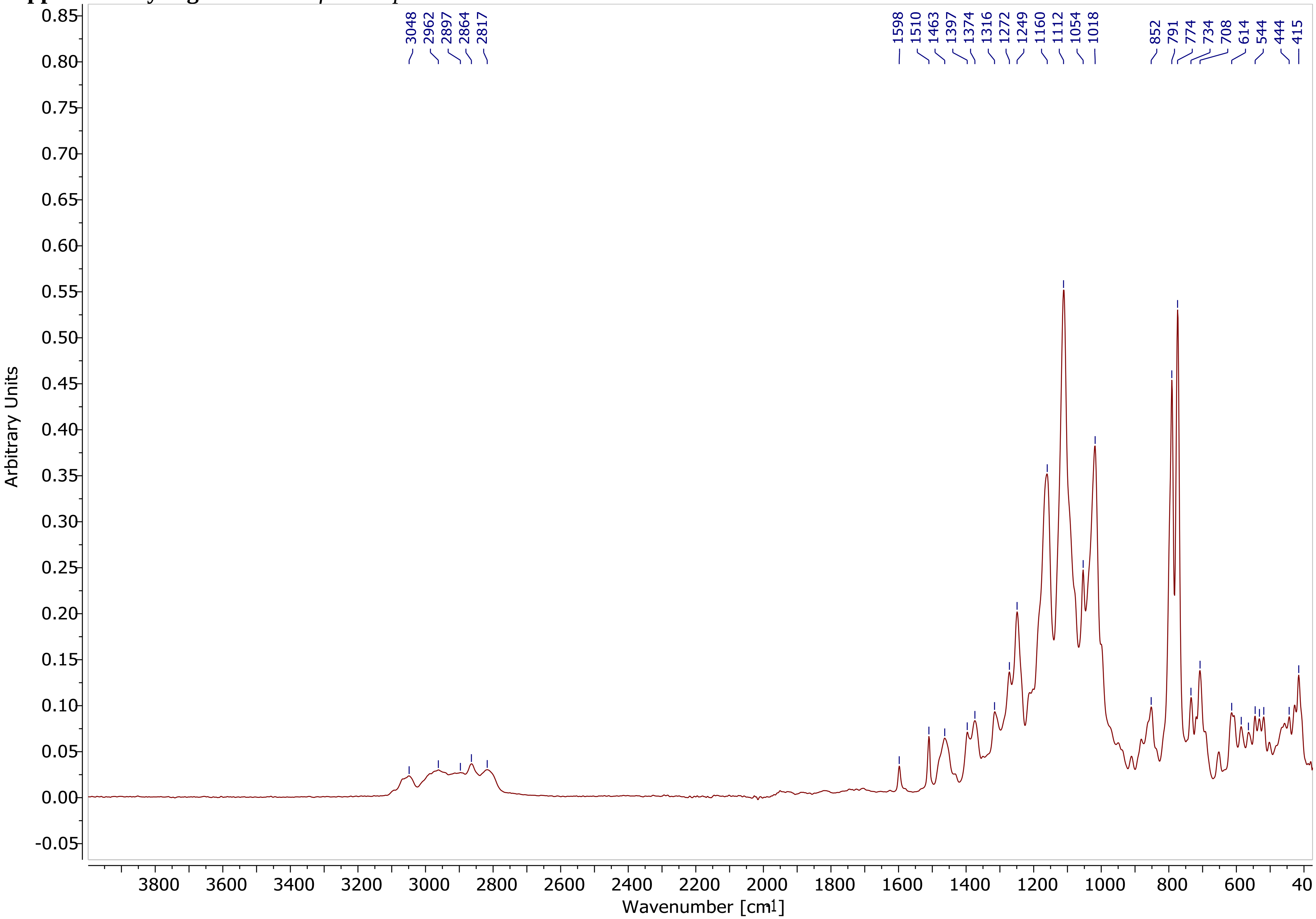

Supplementary Figure 380: *¹H-NMR for compound* ***79***

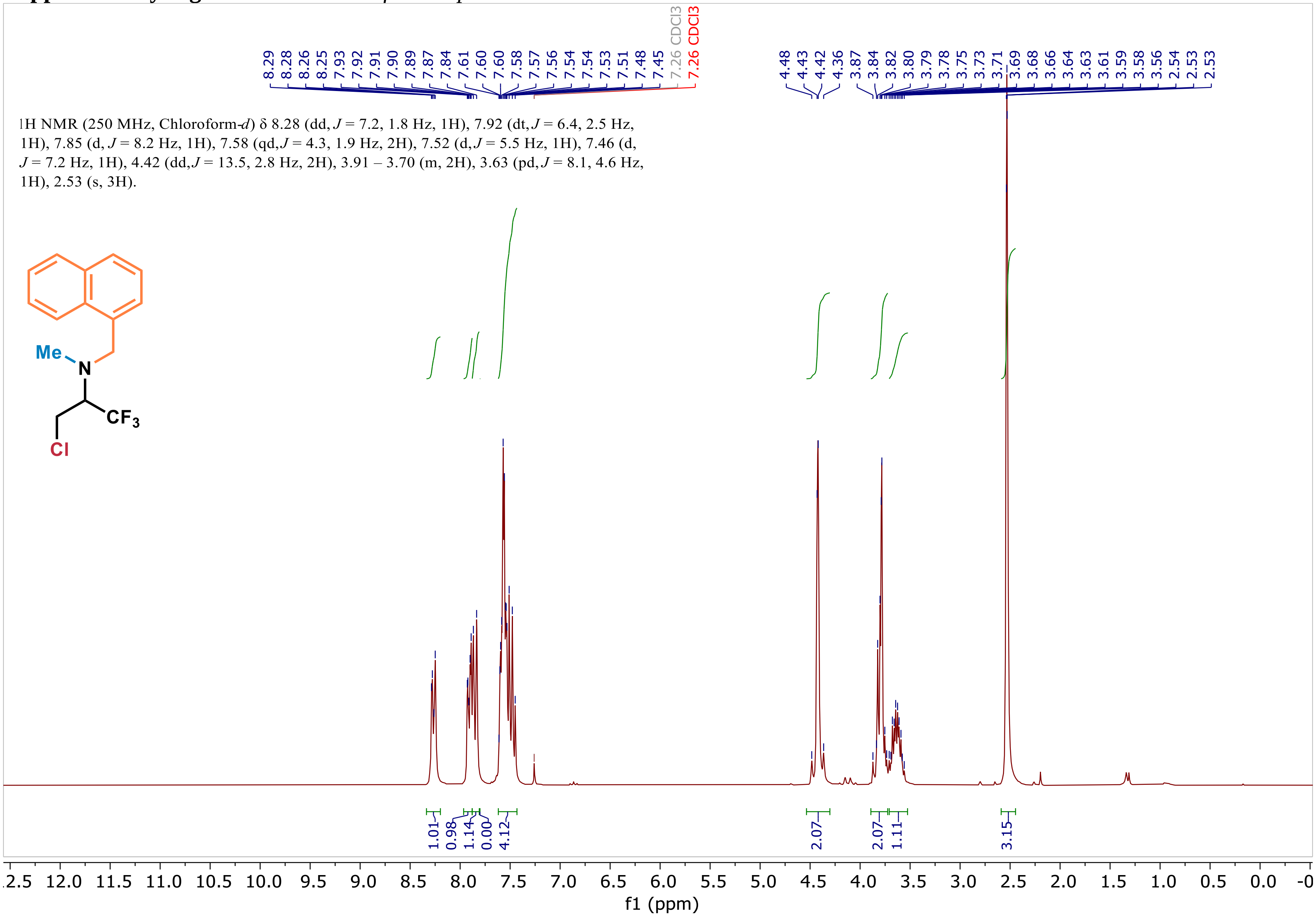

Supplementary Figure 381: ^{19}*F NMR for compound* ***79***

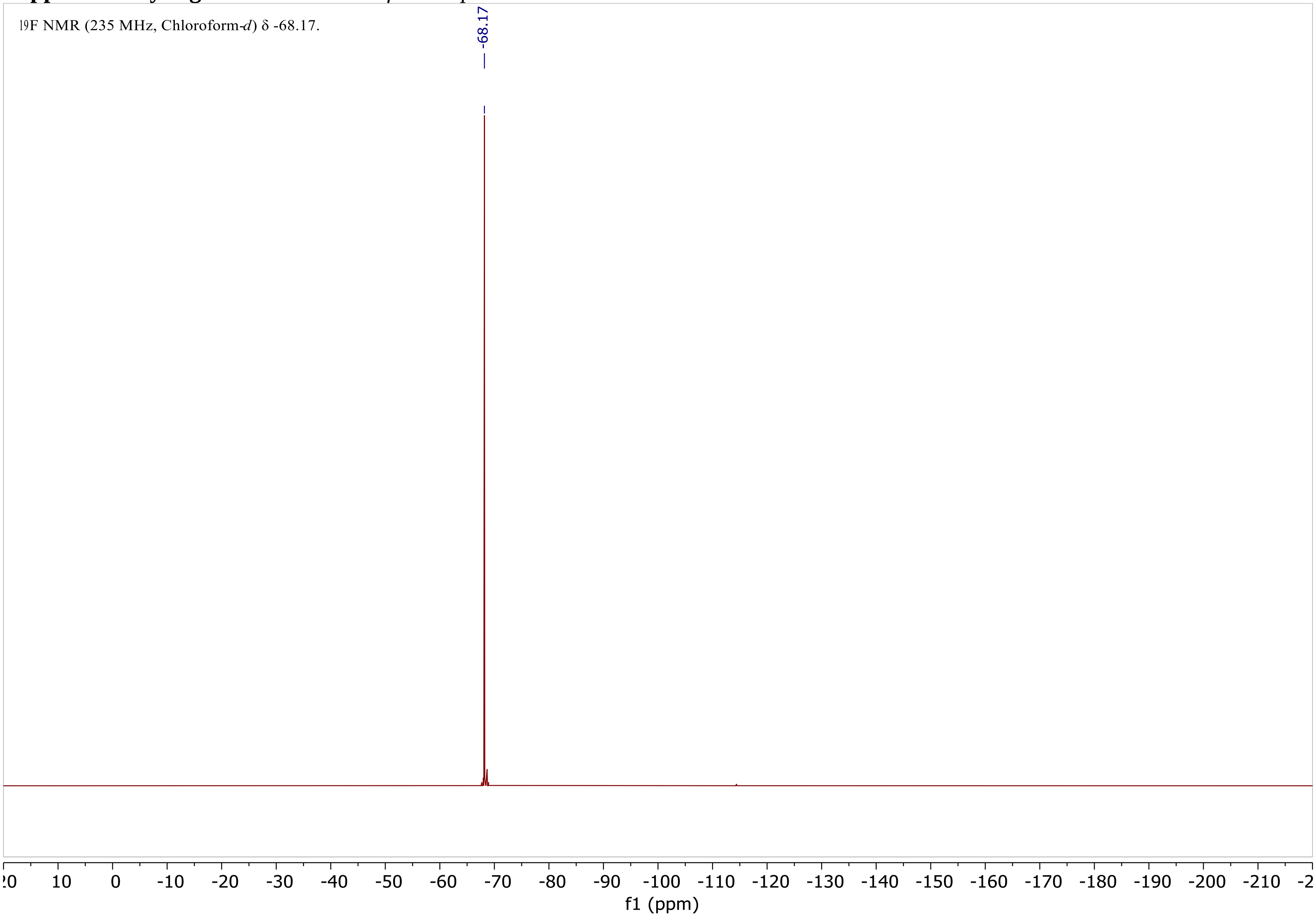

Supplementary Figure 382: *^{13}C NMR for compound* ***79***

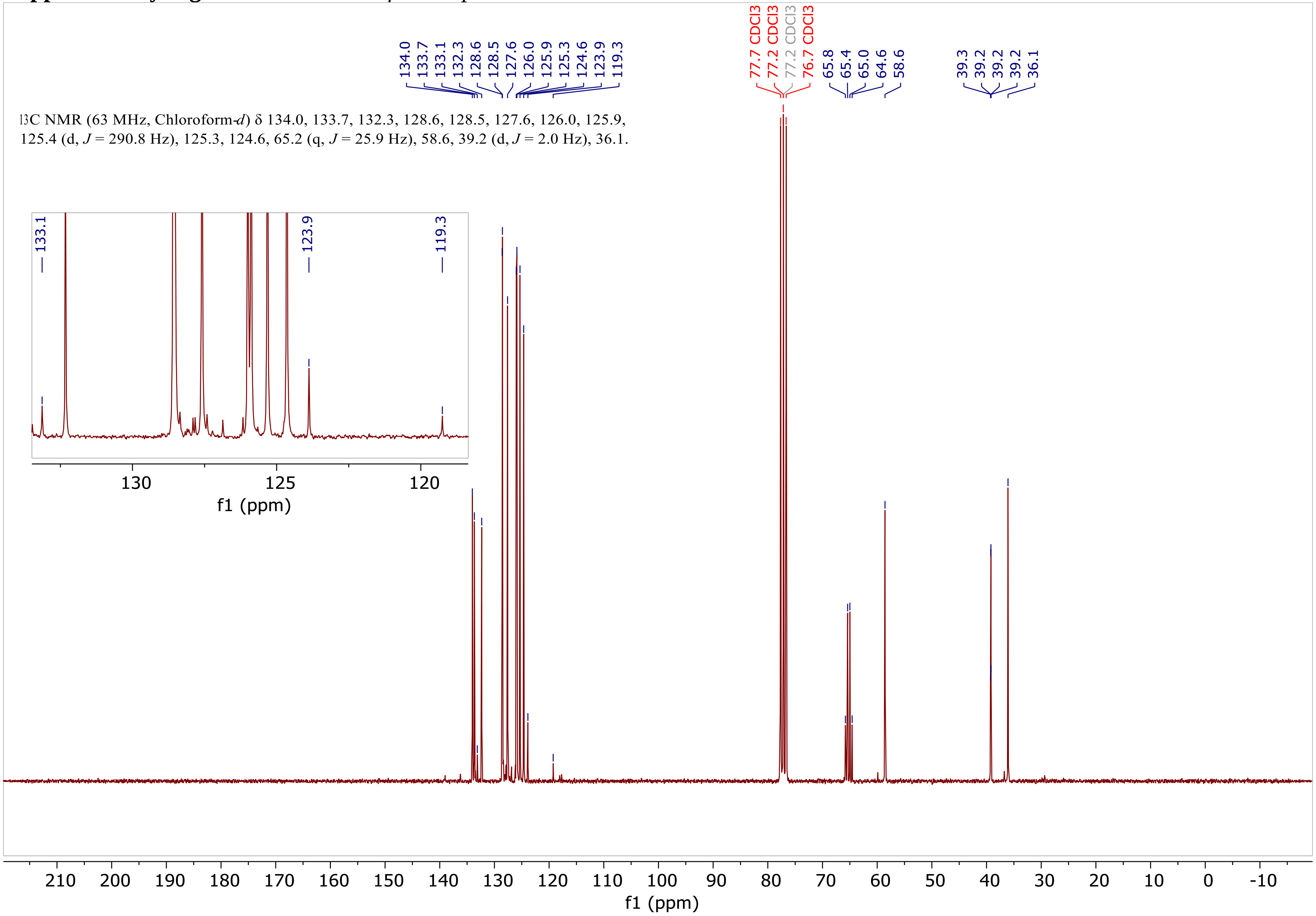

Supplementary Figure 383: *IR for compound **79***

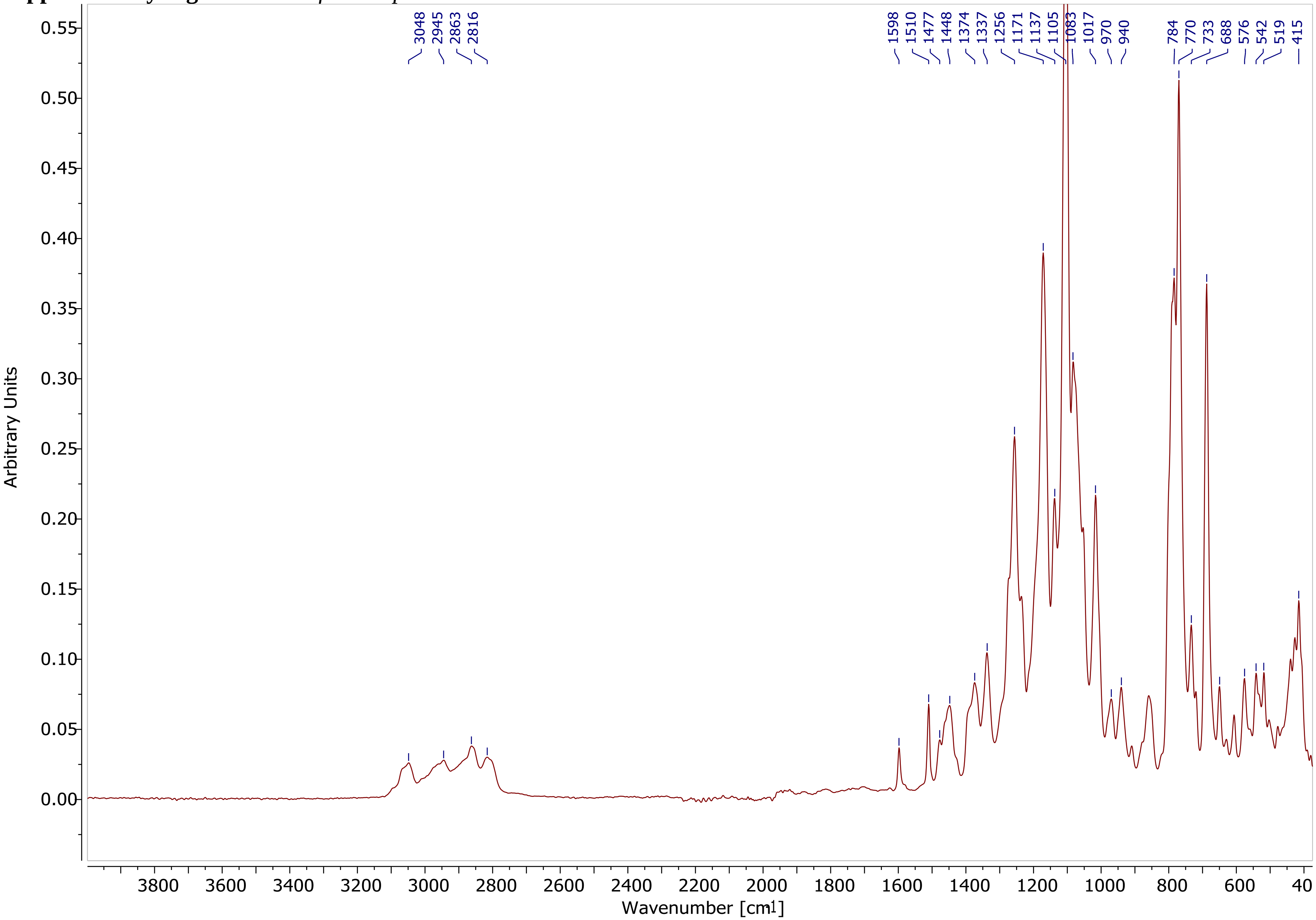

Supplementary Figure 384: *^{1}H-NMR for compound* ***80***

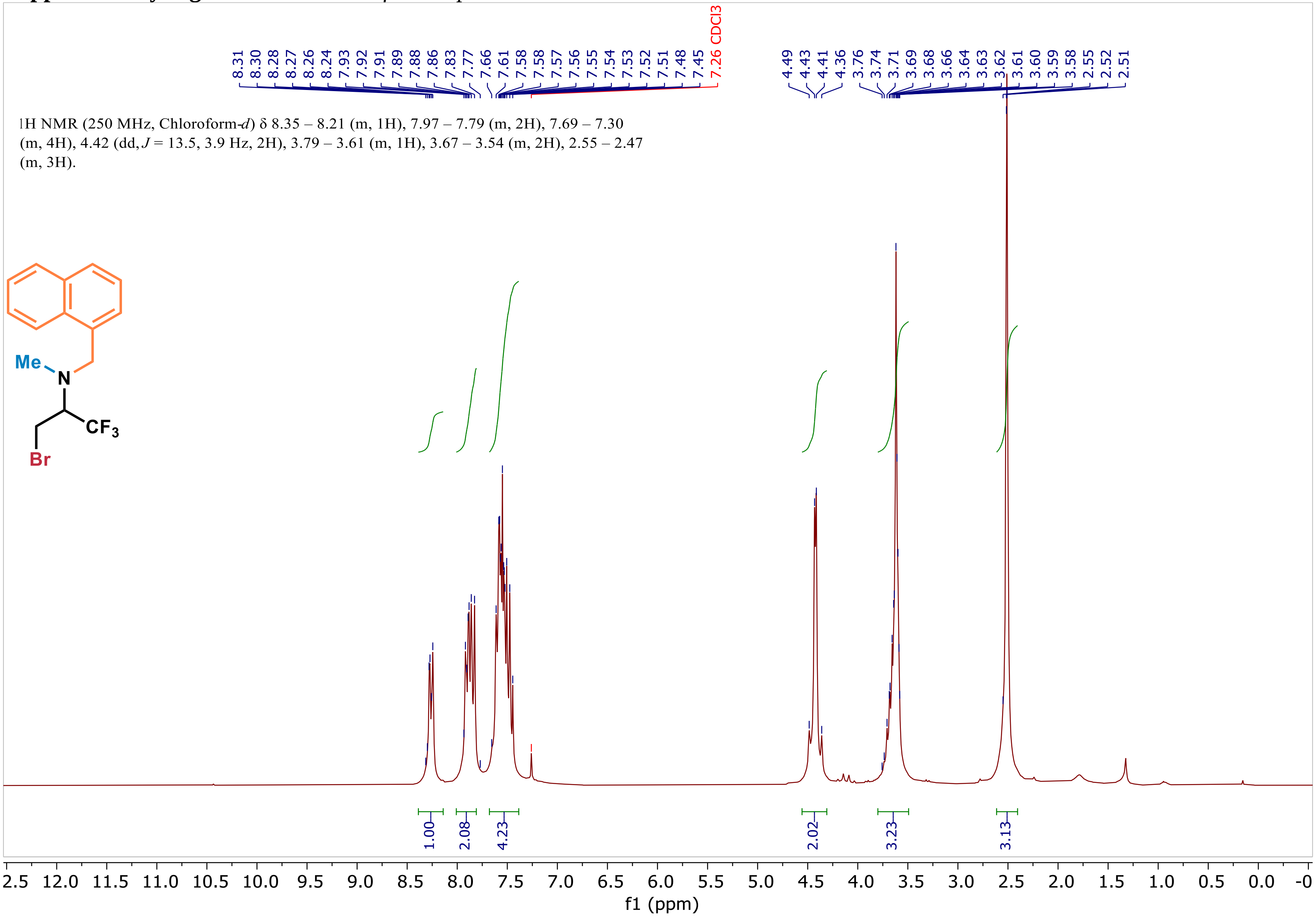

Supplementary Figure 385: *^{19}F NMR for compound* ***80***

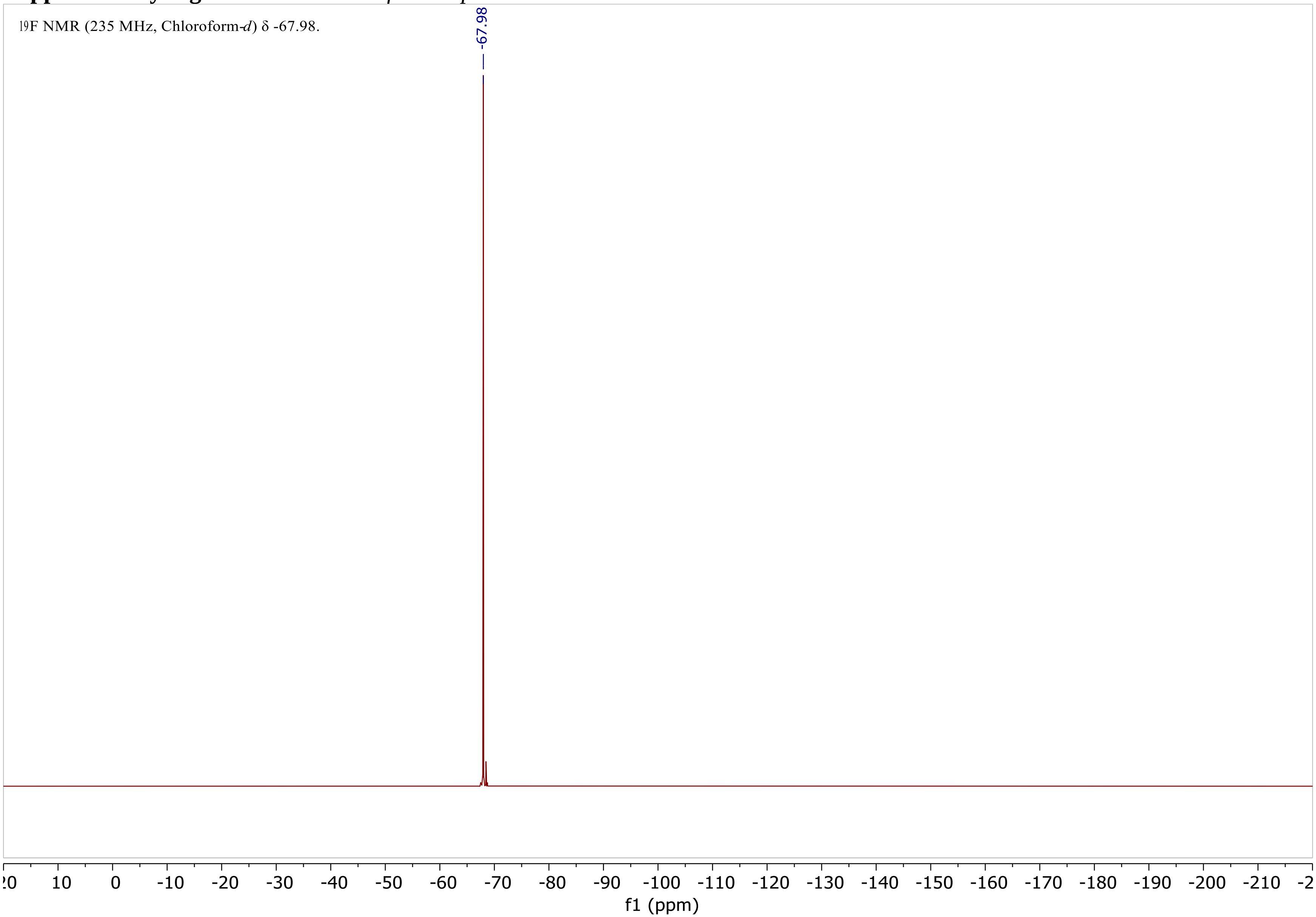

Supplementary Figure 386: *^{13}C NMR for compound* ***80***

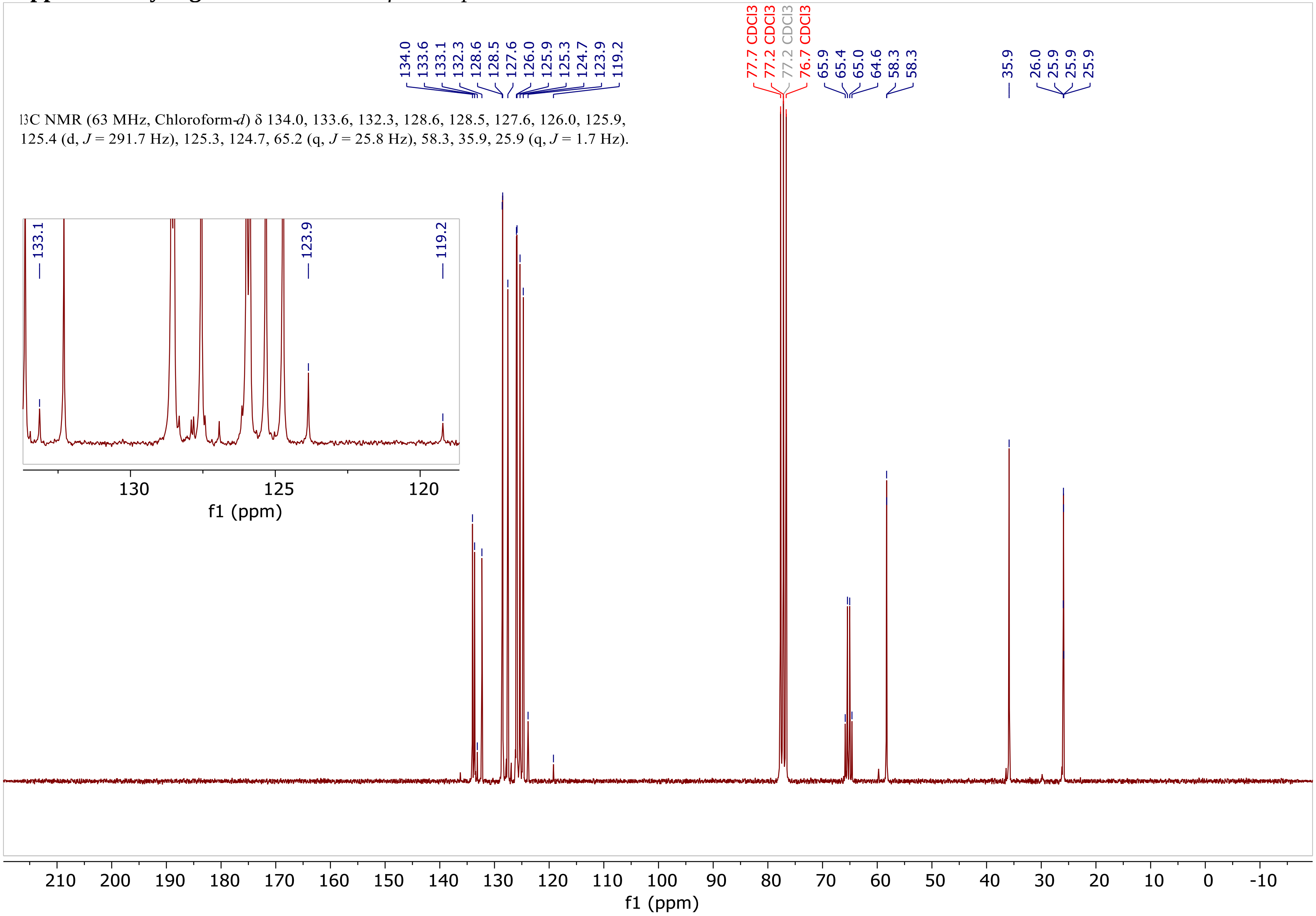

Supplementary Figure 387: *IR for compound **80***

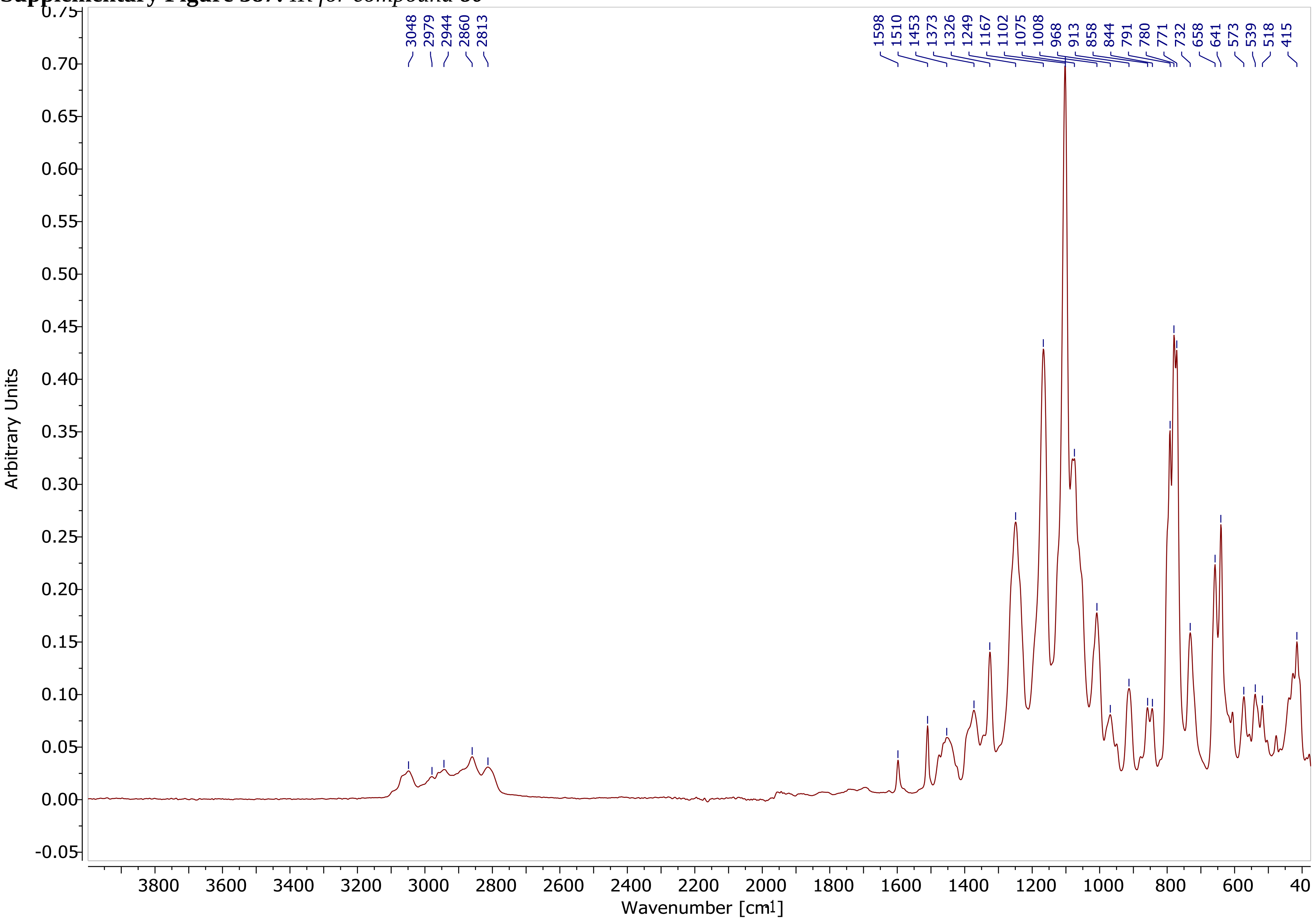

Supplementary Figure 388: *¹H-NMR for compound* ***81***

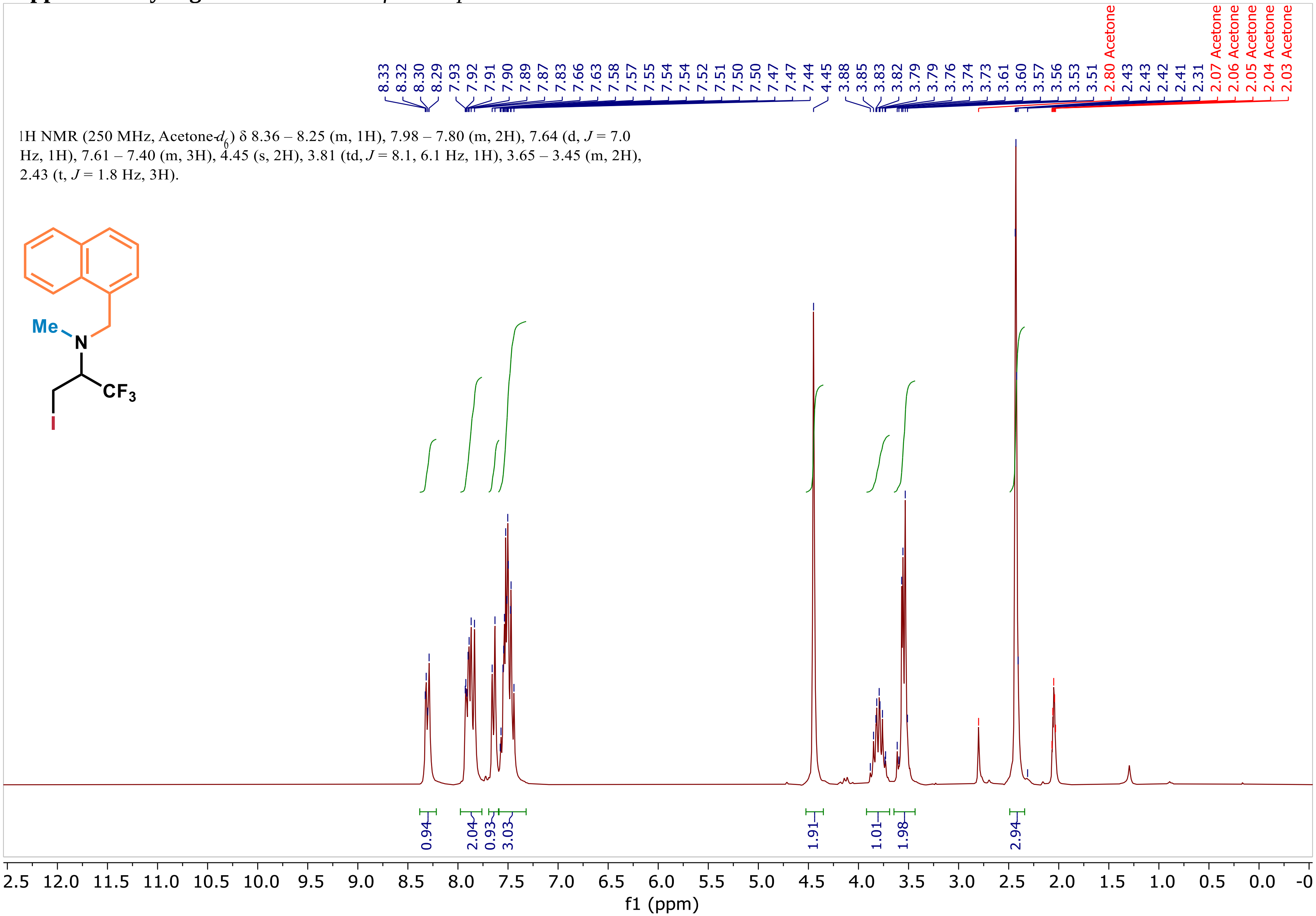

Supplementary Figure 389: *^{19}F NMR for compound* ***81***

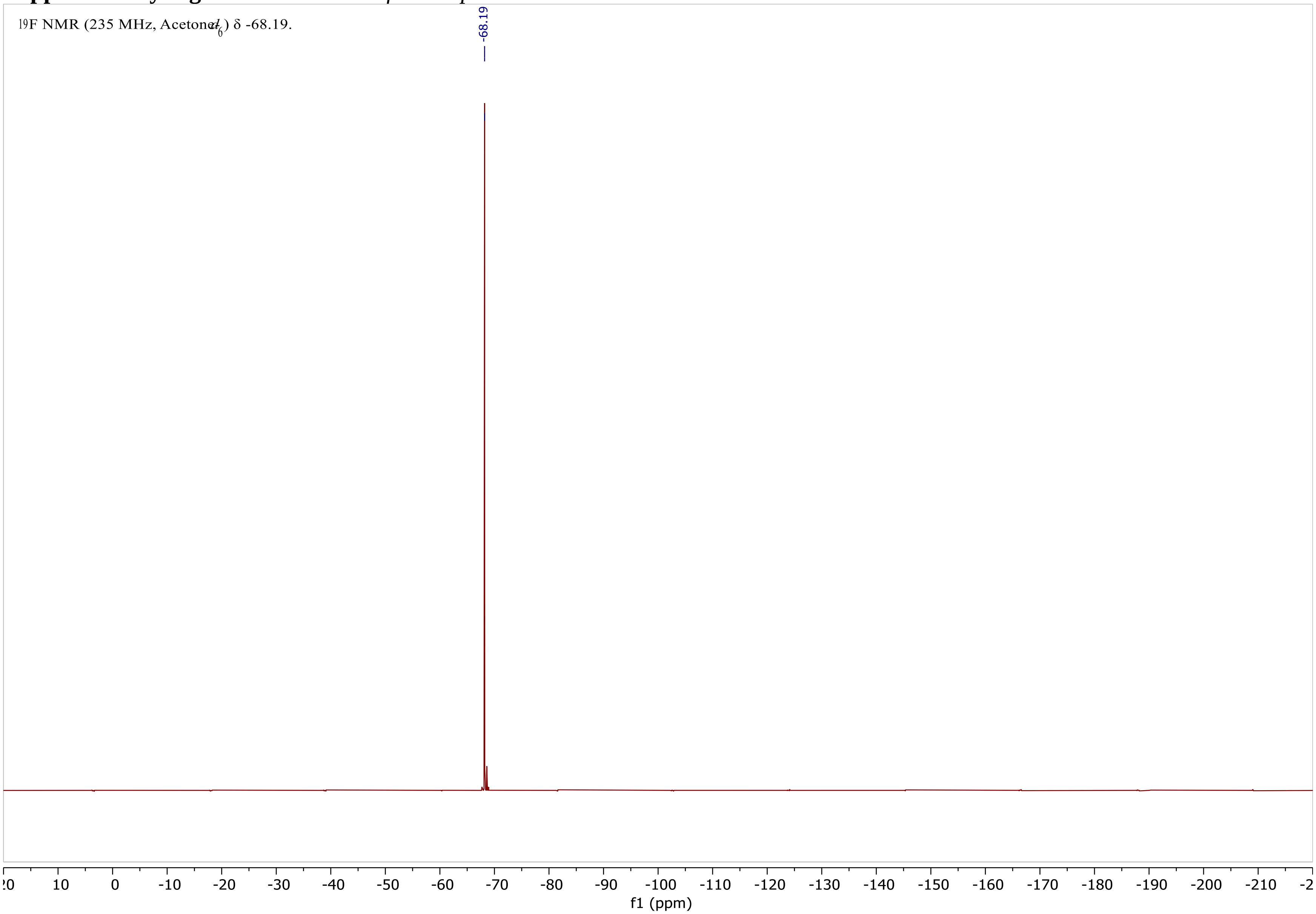

Supplementary Figure 390: *^{13}C NMR for compound **81***

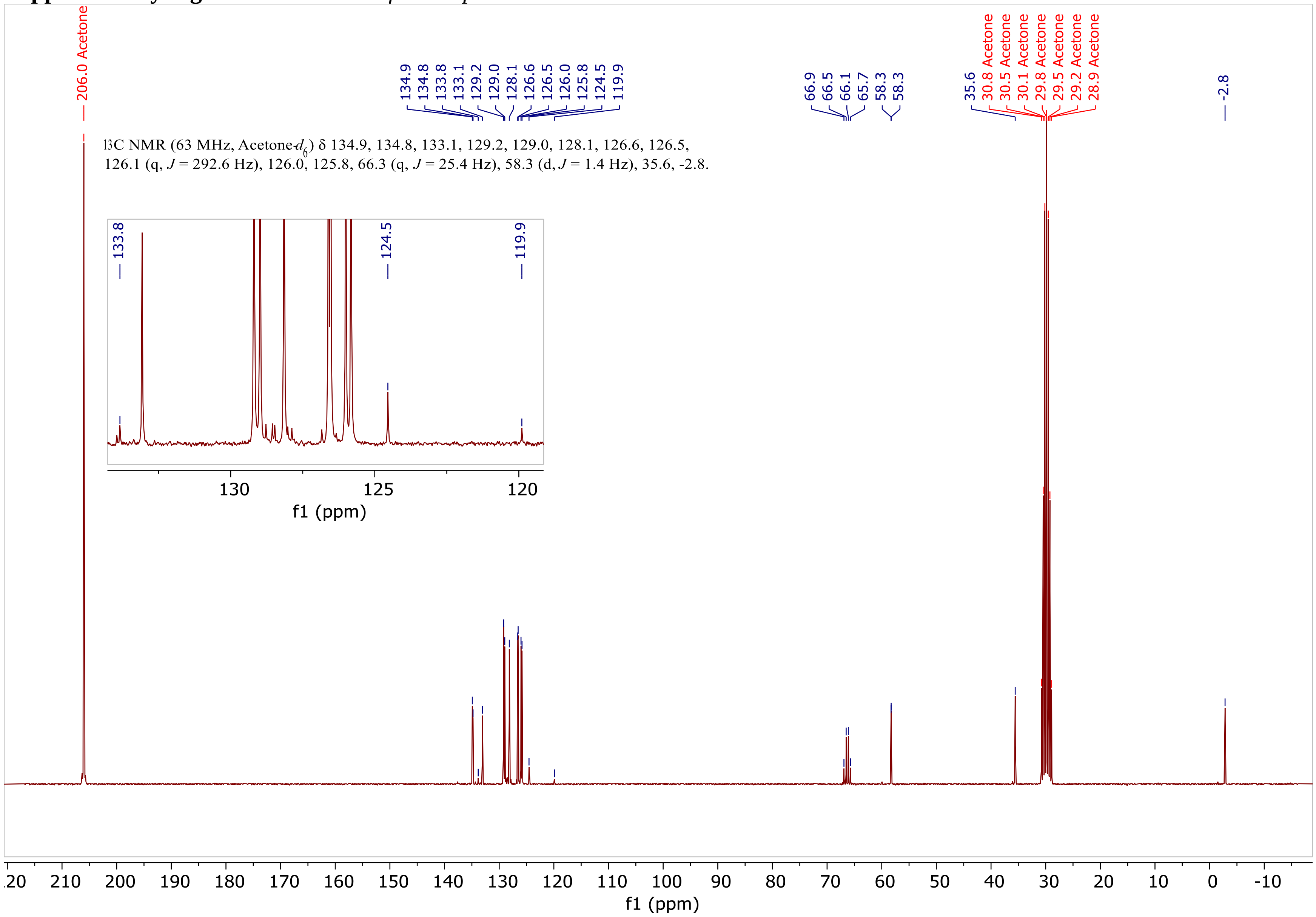

Supplementary Figure 391: *IR for compound **81***

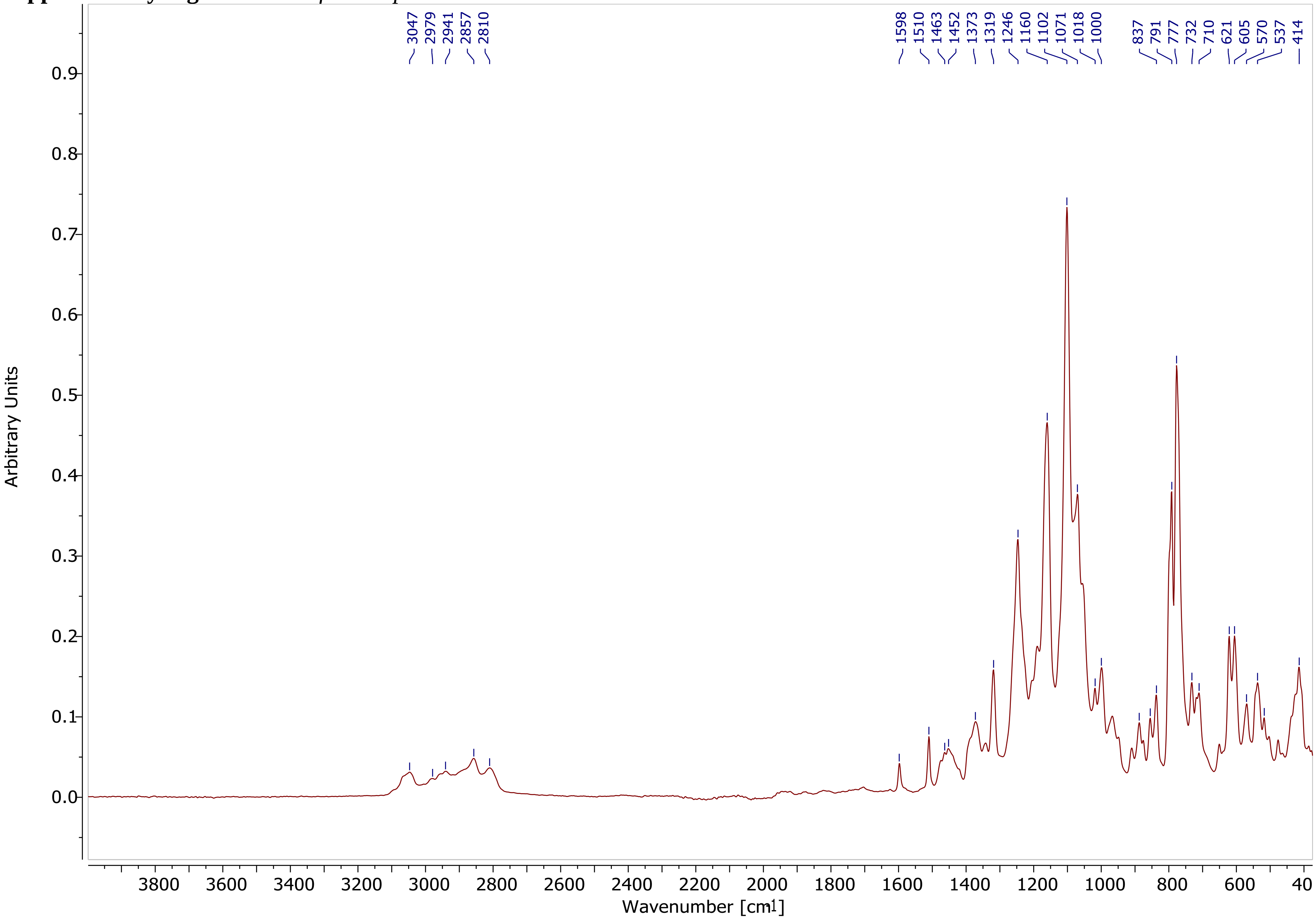

Supplementary Figure 392: *¹H-NMR for compound* ***82***

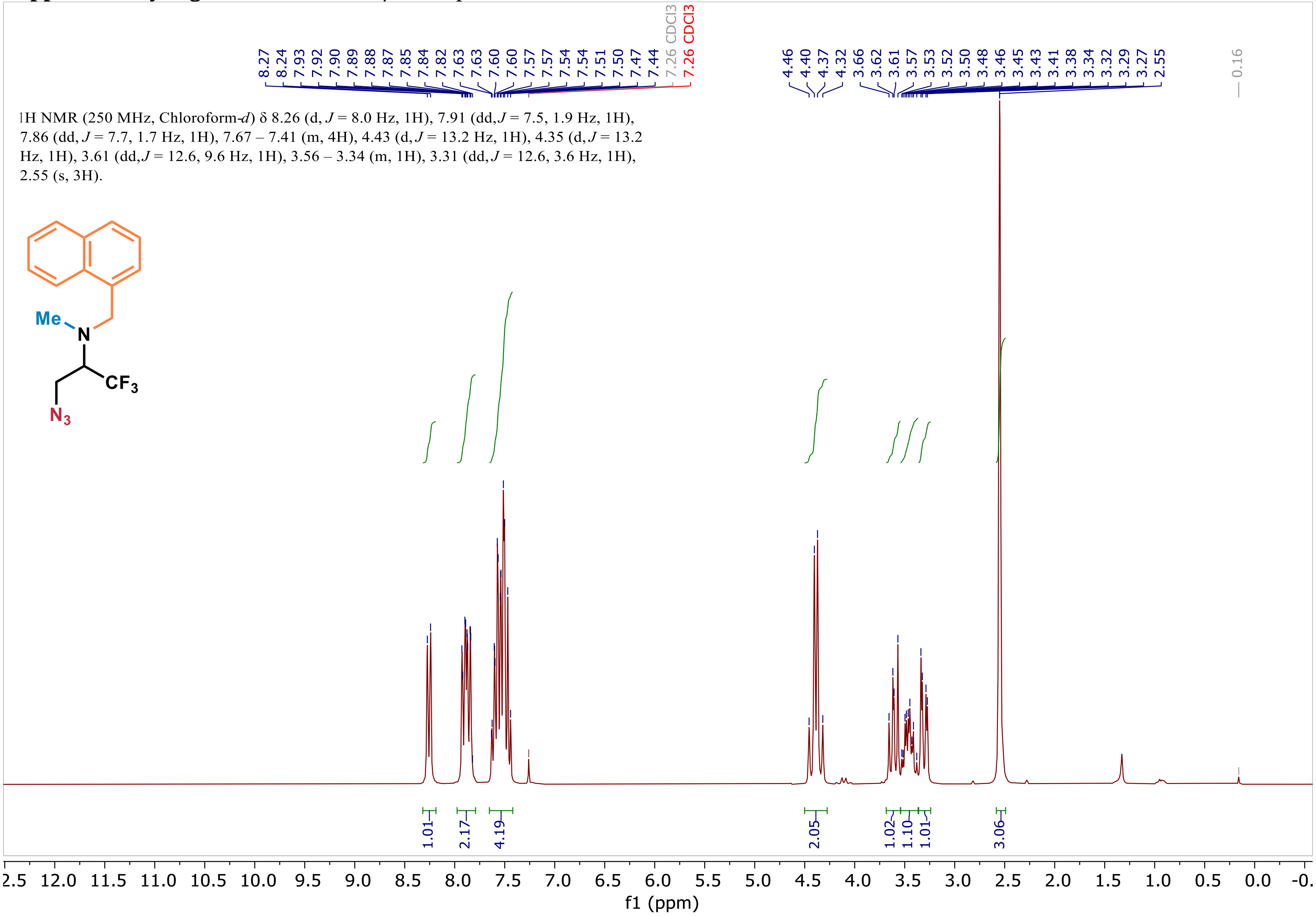

Supplementary Figure 393: *^{19}F NMR for compound* ***82***

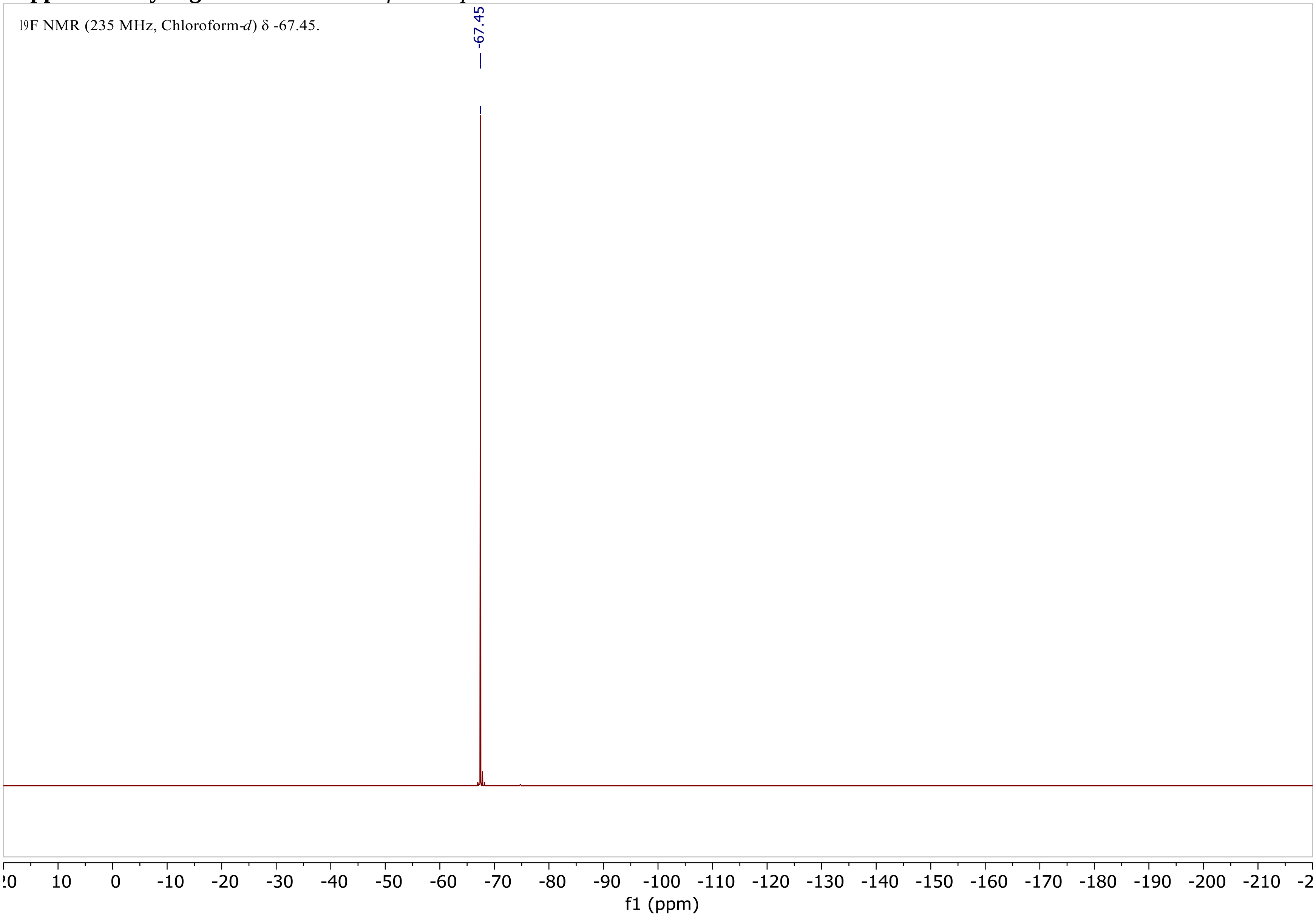

Supplementary Figure 394: *^{13}C NMR for compound* ***82***

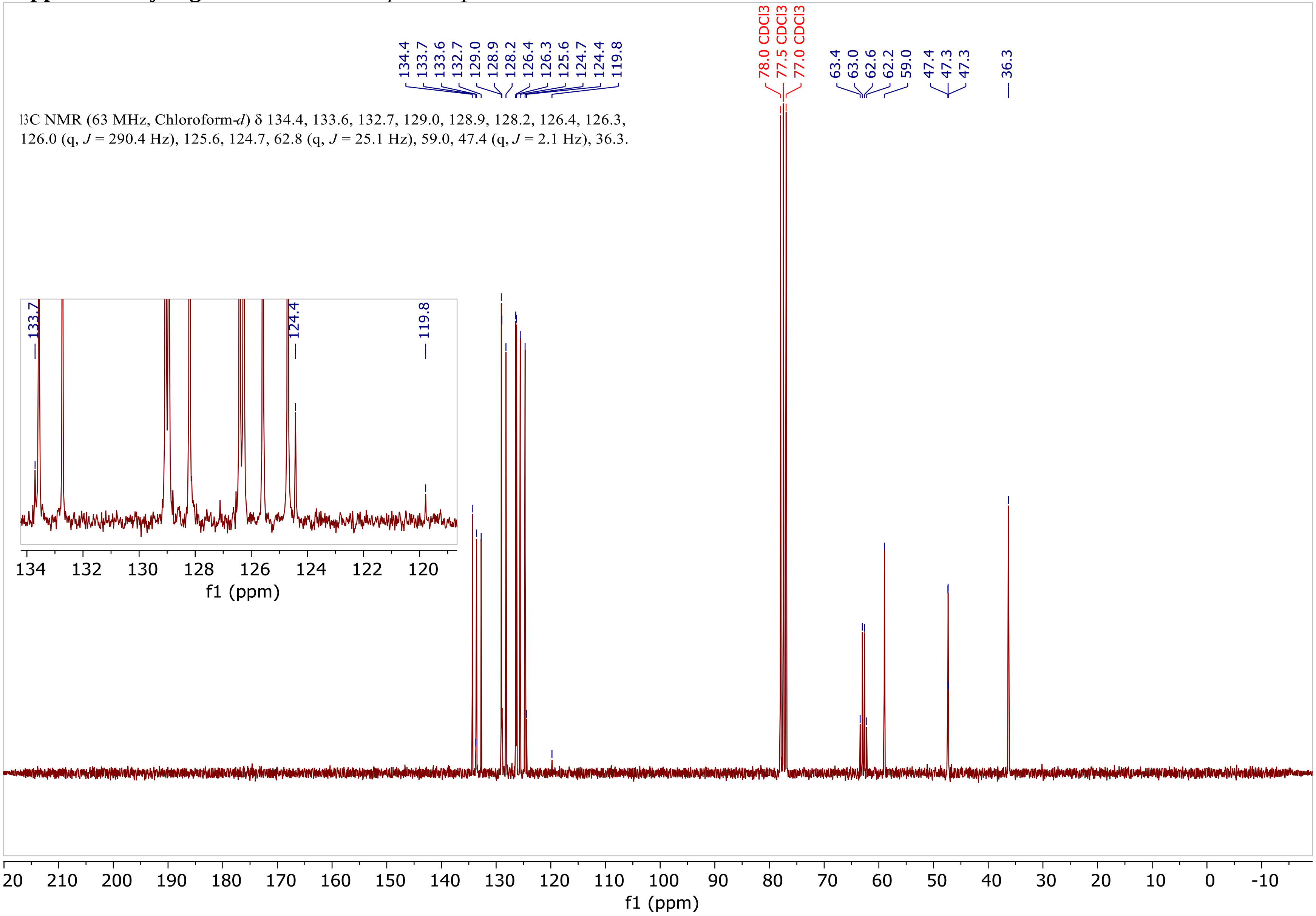

Supplementary Figure 395: *IR for compound* ***82***

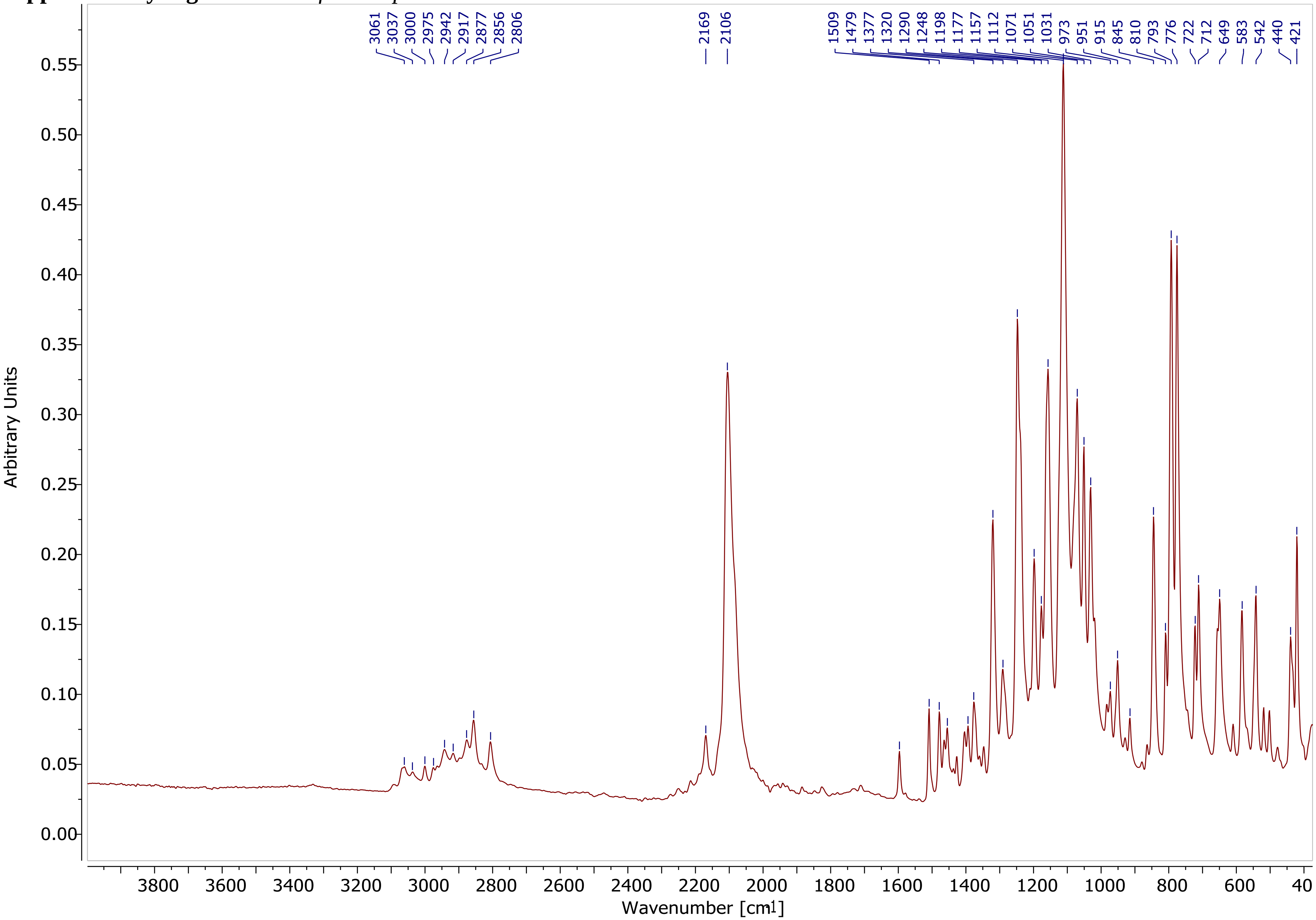

Supplementary Figure 396: *^{1}H-NMR for compound* ***83***

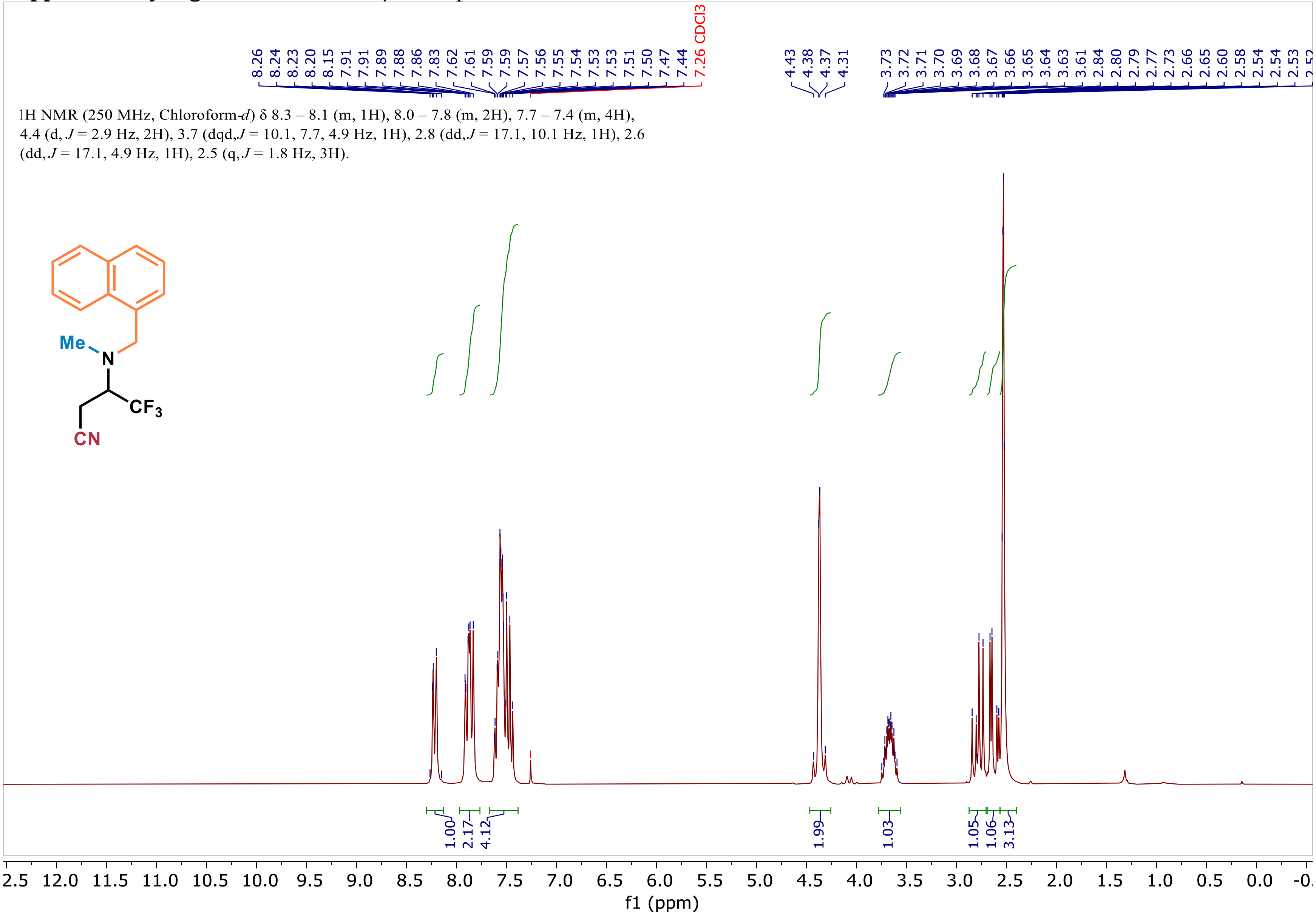

Supplementary Figure 397: *^{19}F NMR for compound* ***83***

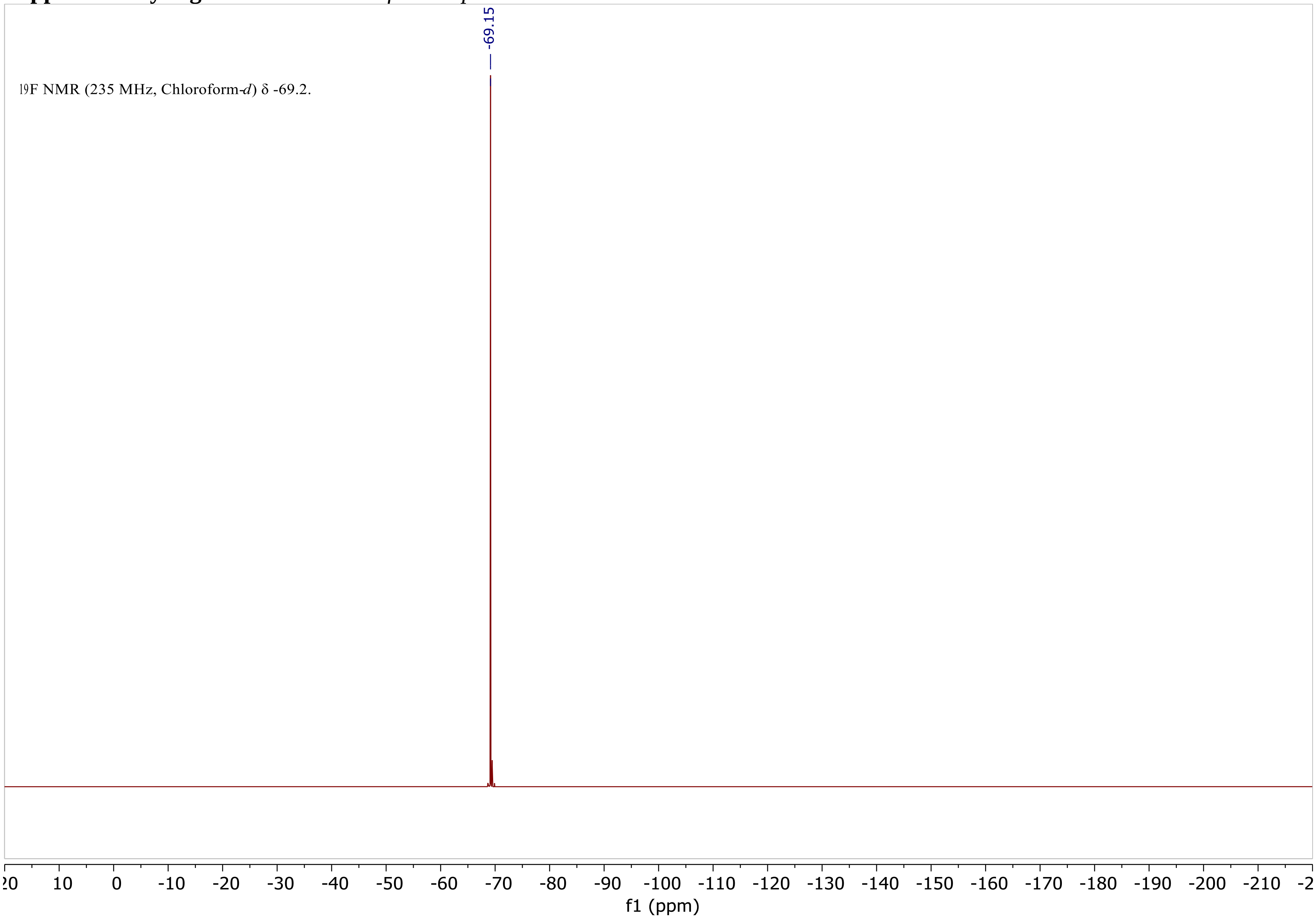

Supplementary Figure 398: *^{13}C NMR for compound* ***83***

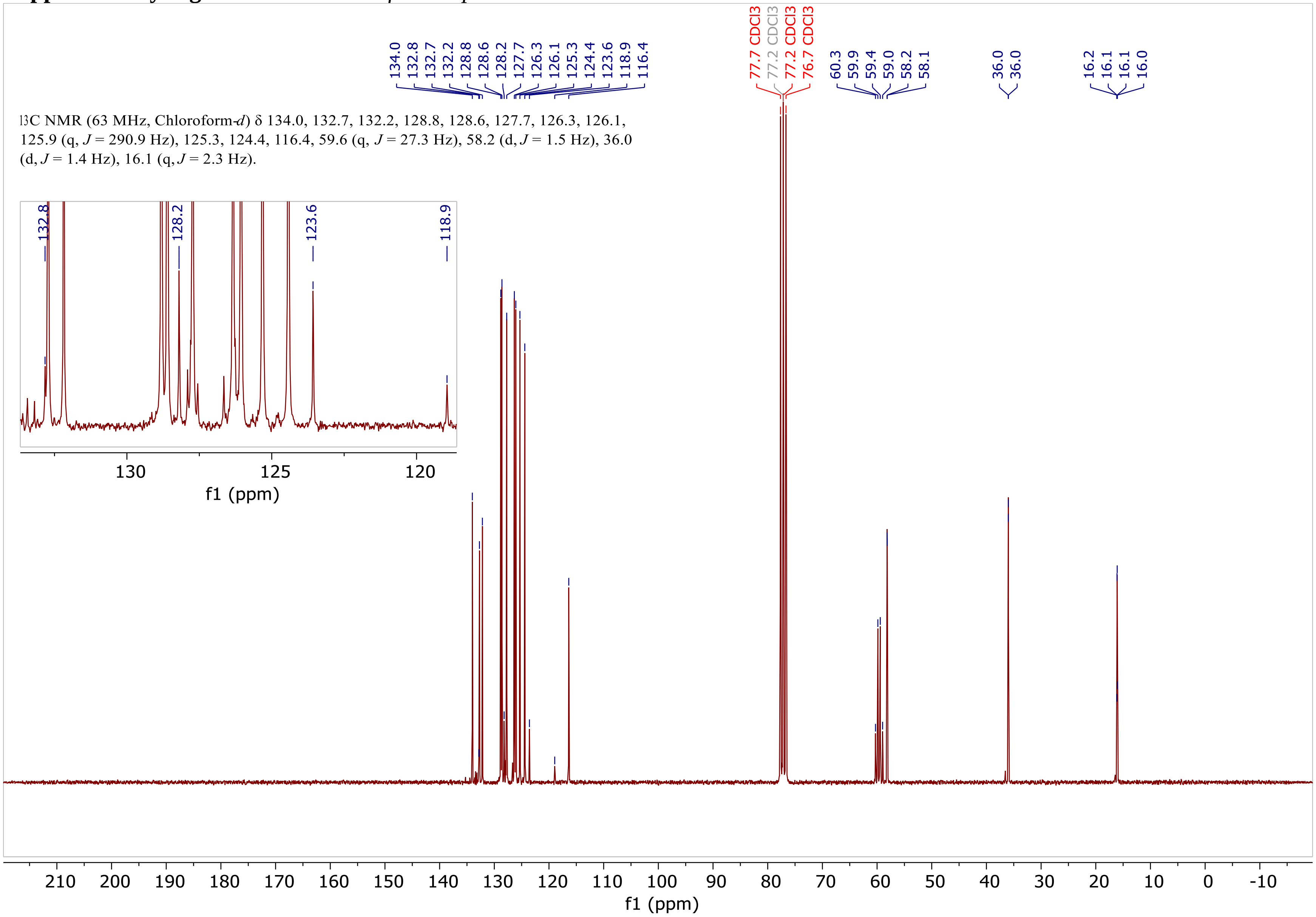

Supplementary Figure 399: *IR for compound **83***

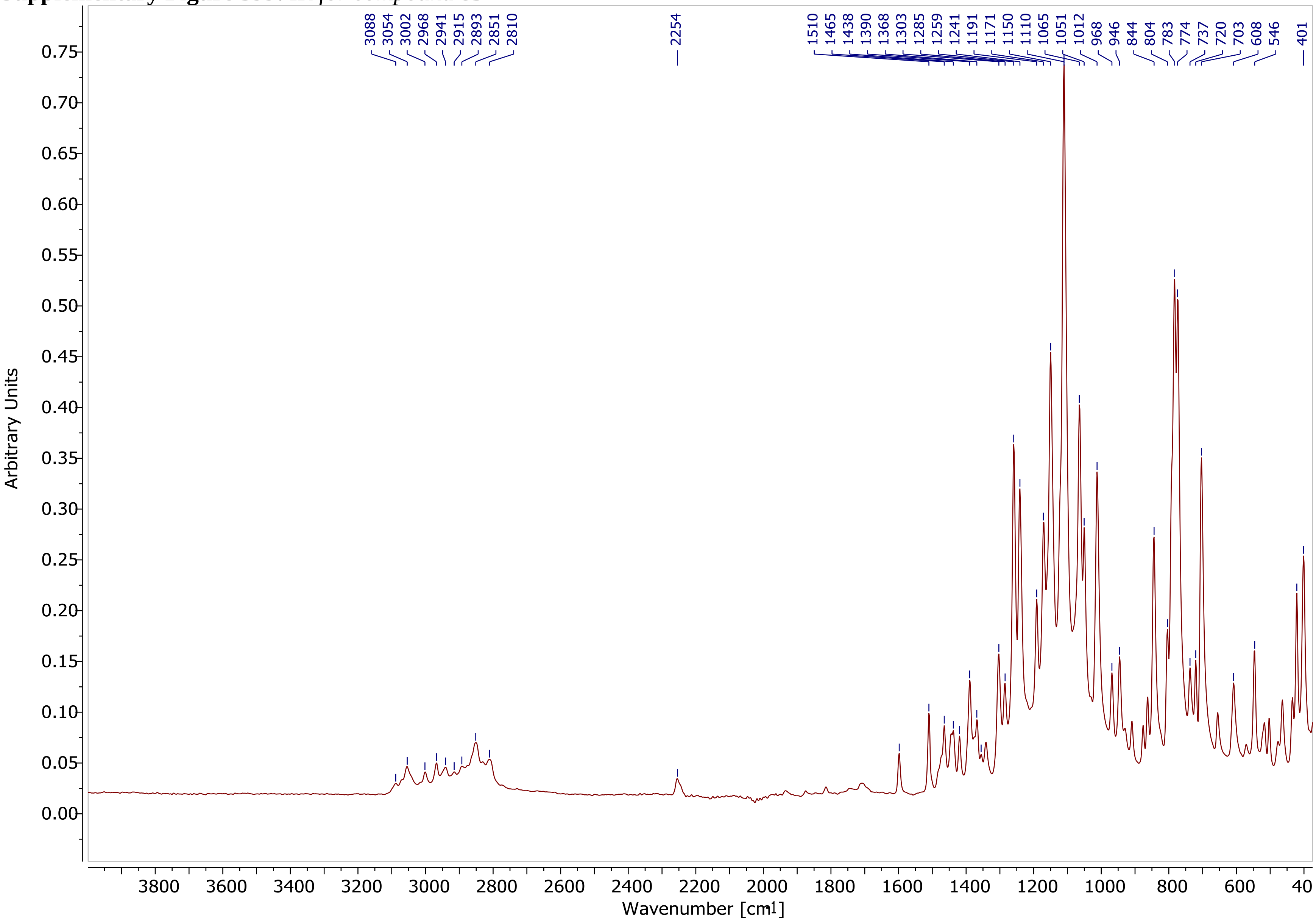

Supplementary Figure 400: *¹H-NMR for compound* ***84***

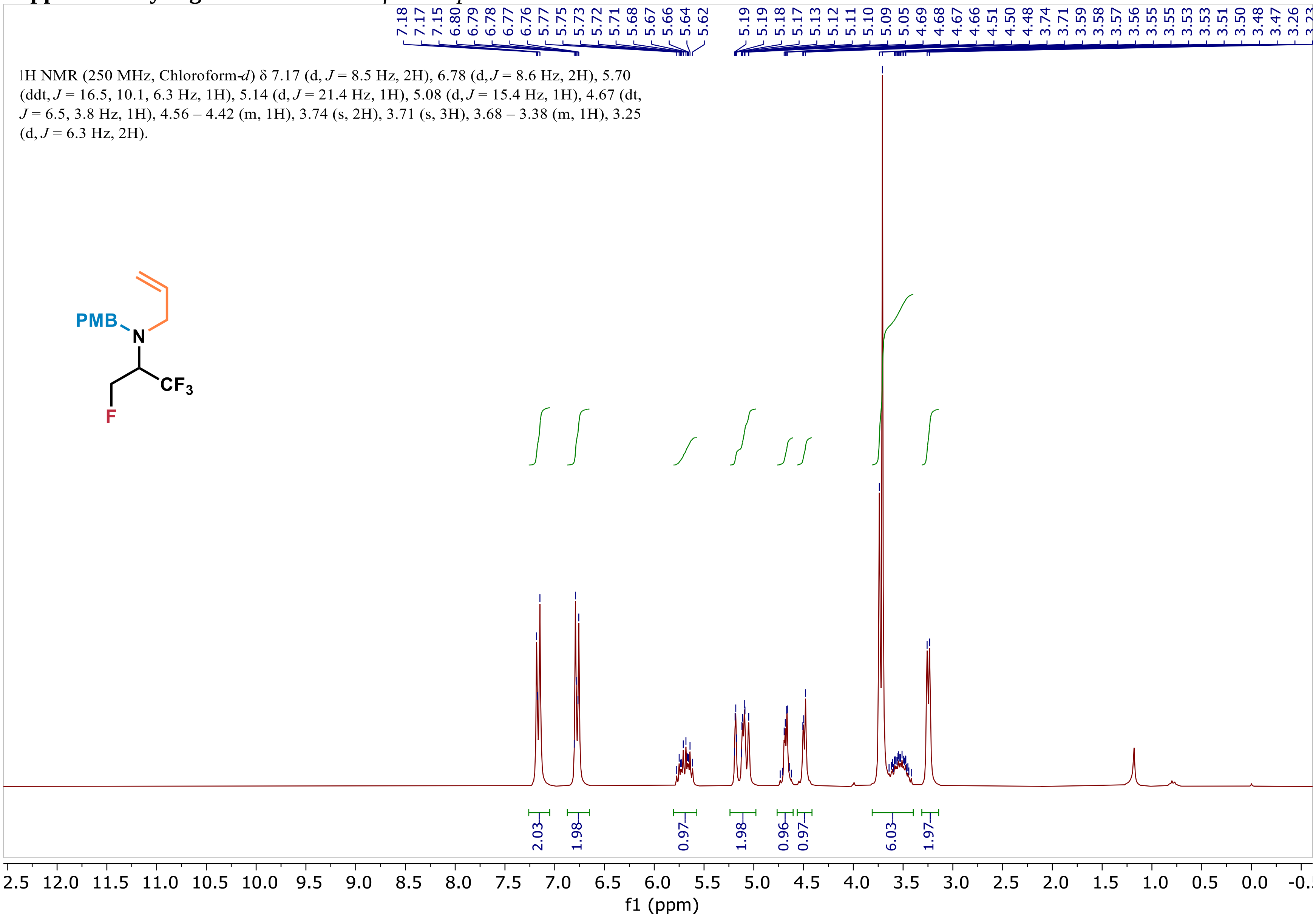

Supplementary Figure 401: ^{19}*F NMR for compound* ***84***

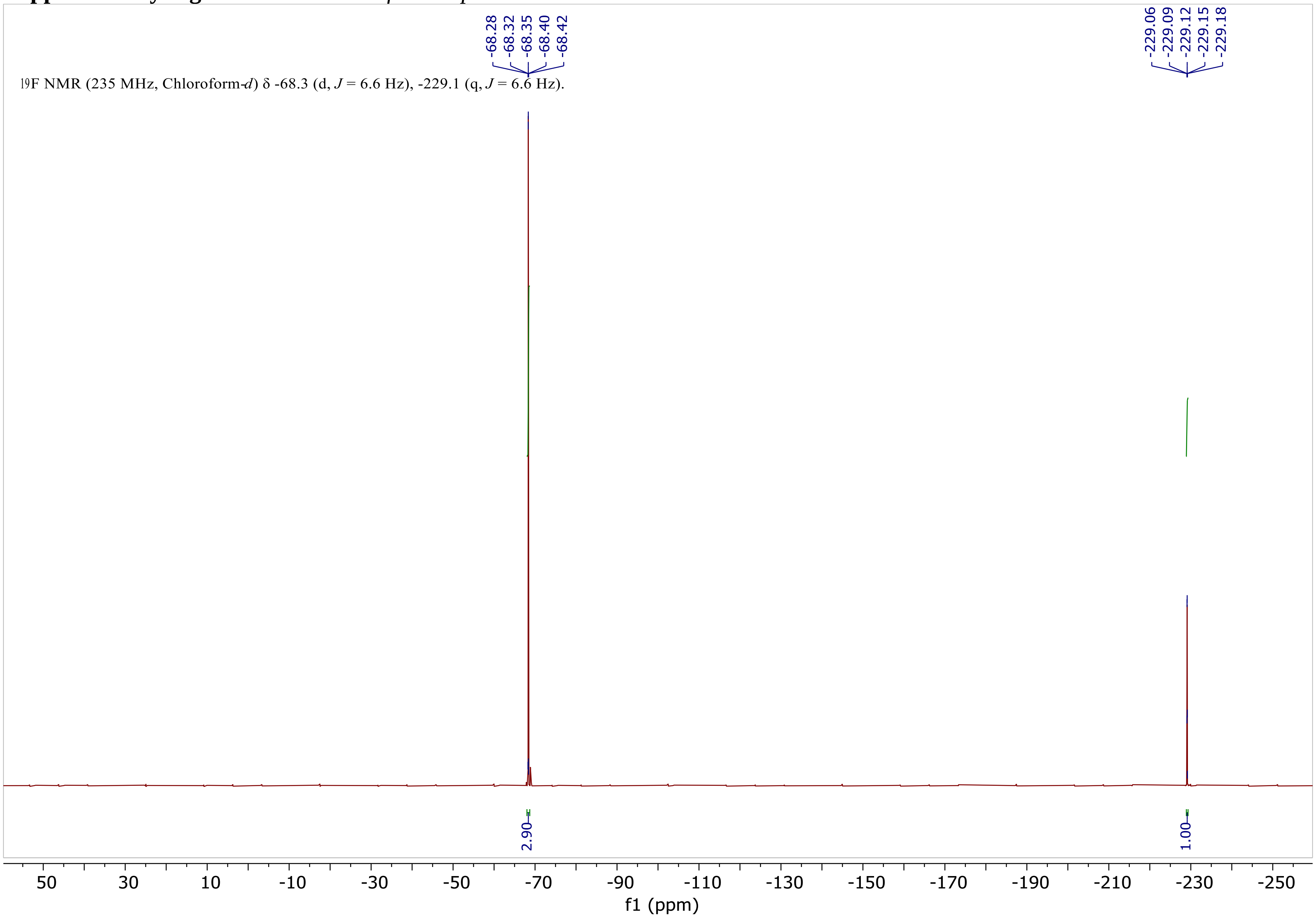

Supplementary Figure 402: *^{13}C NMR for compound* ***84***

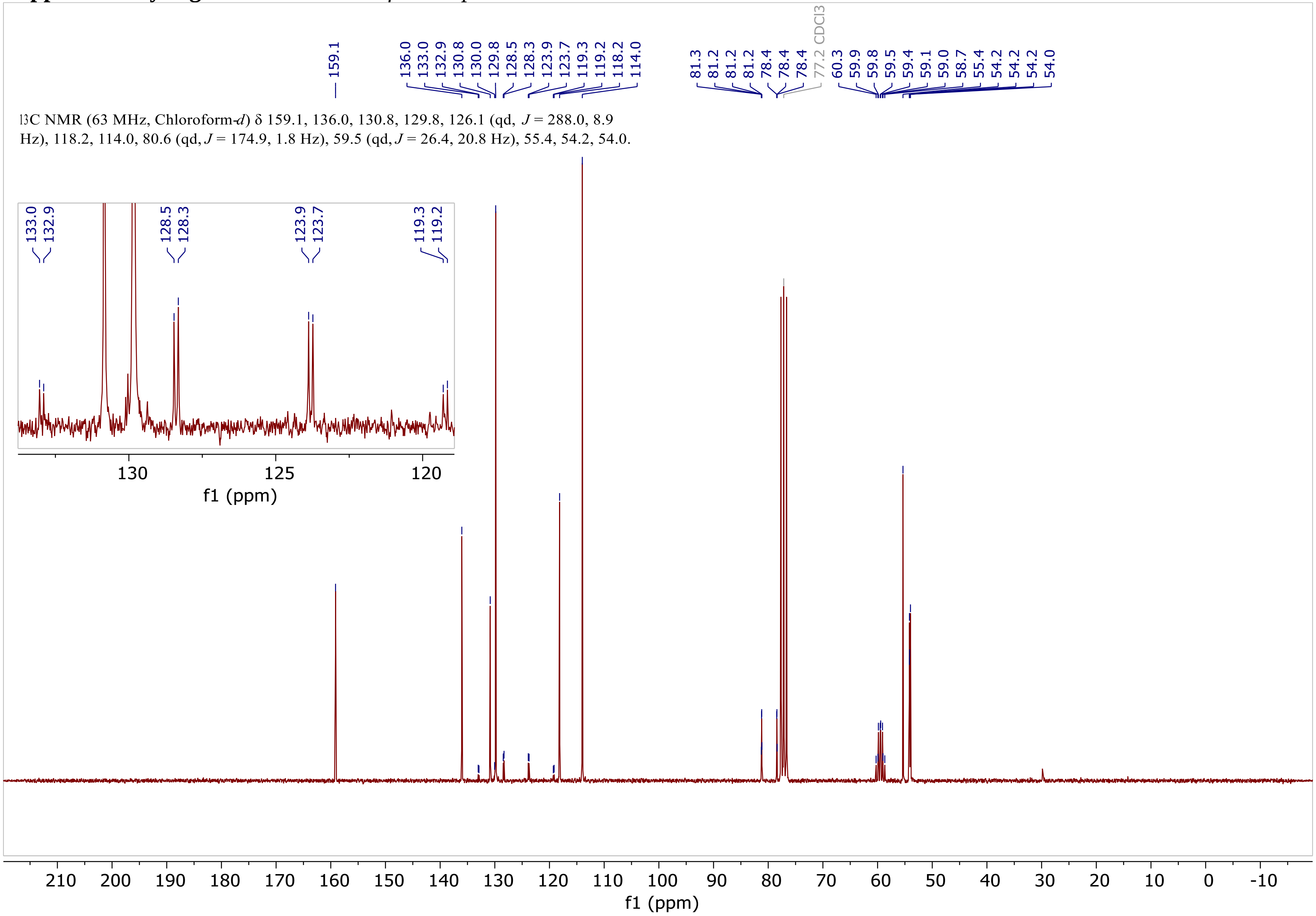

Supplementary Figure 403: *IR for compound **84***

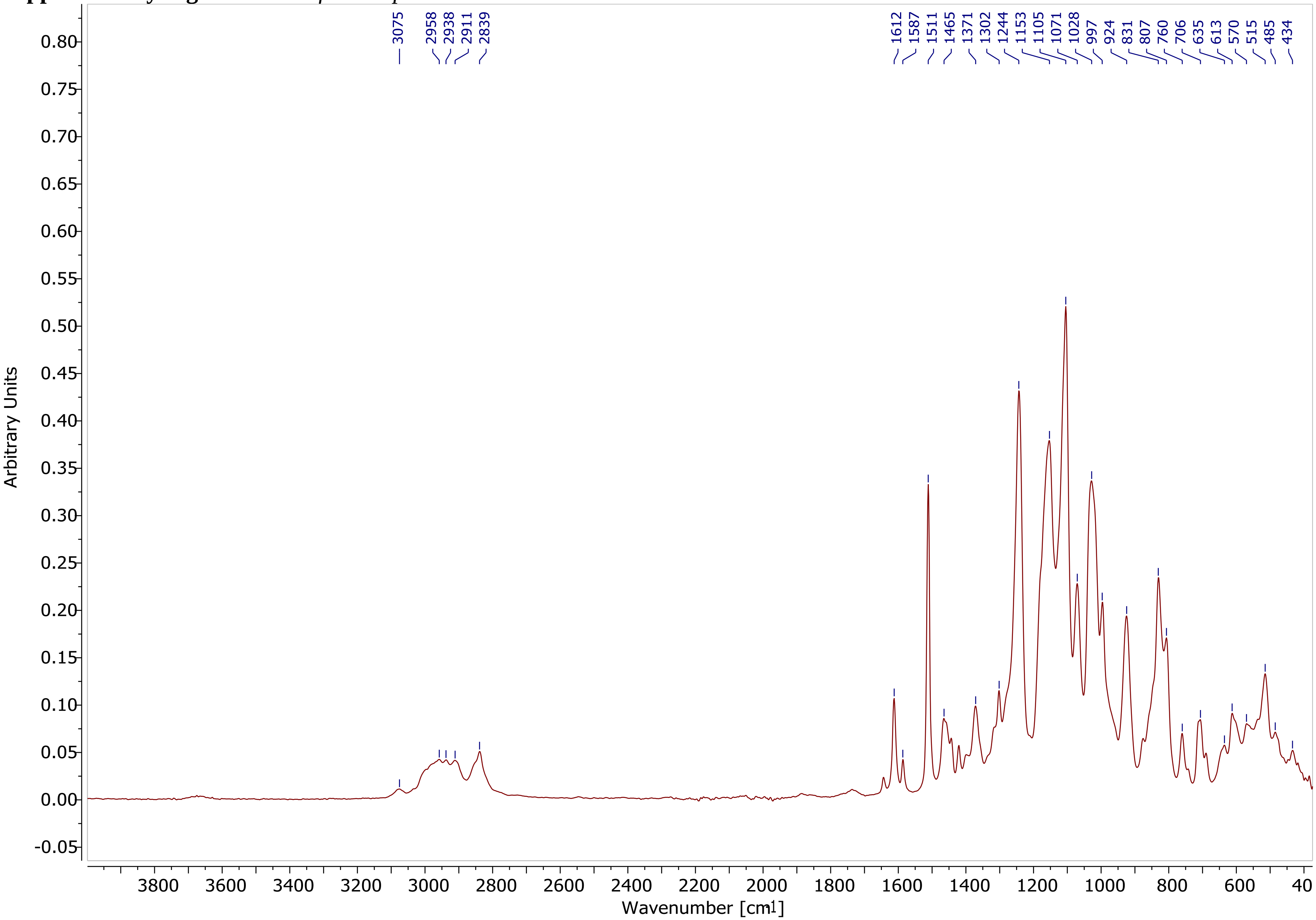

Supplementary Figure 404: *^{1}H-NMR for compound* ***85***

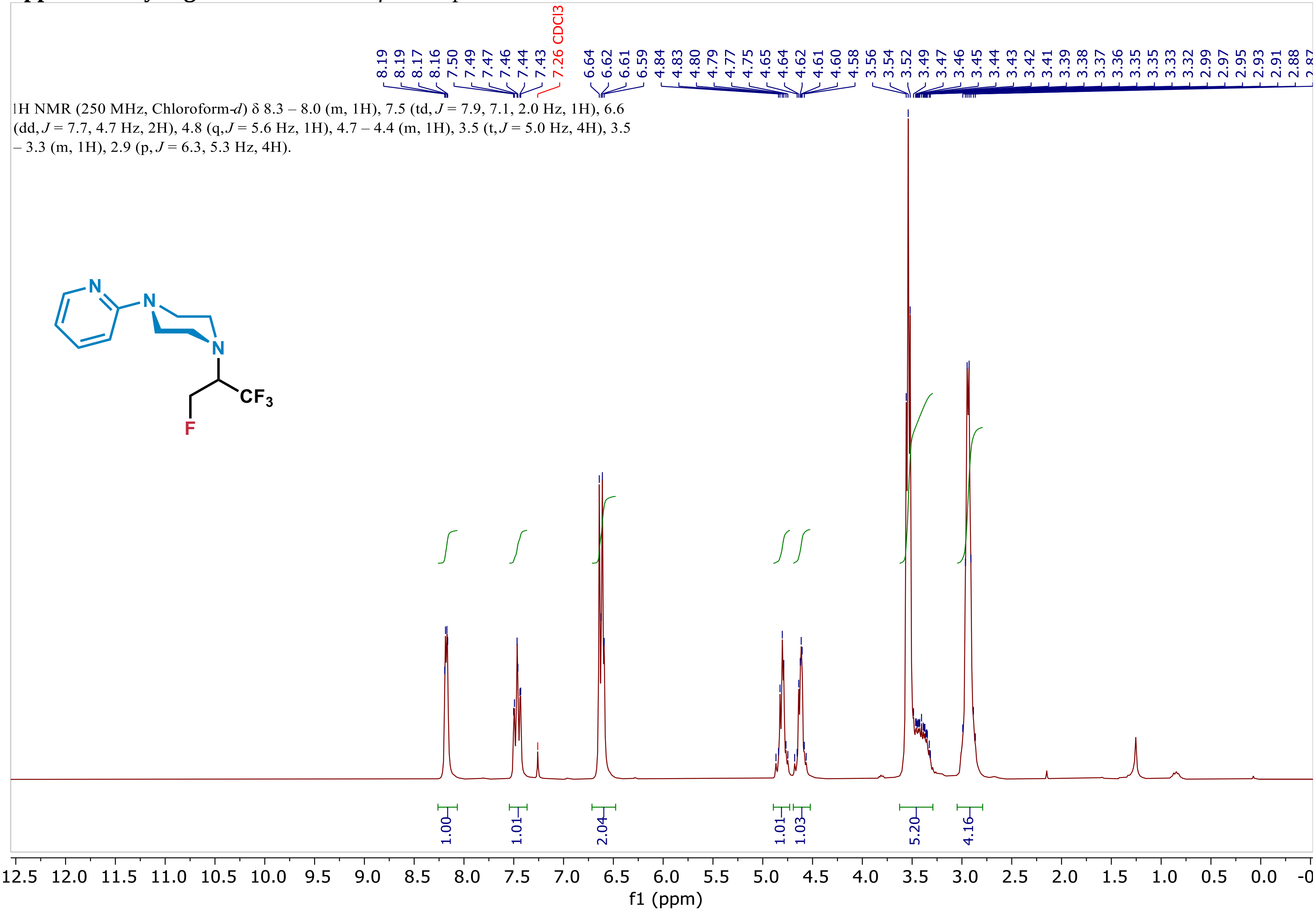

Supplementary Figure 405: *^{19}F NMR for compound* ***85***

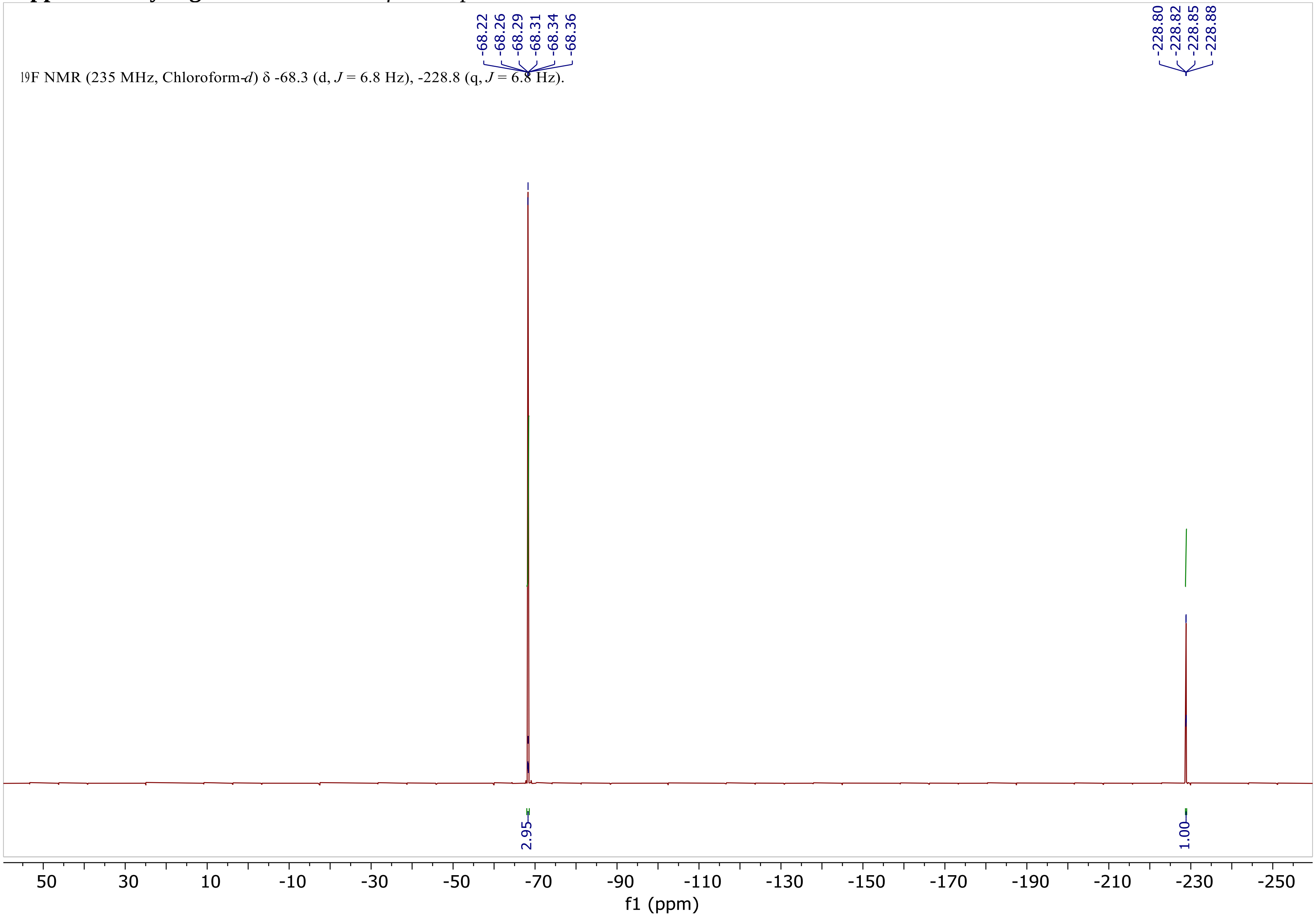

Supplementary Figure 406: *^{19}F NMR for compound* ***85***

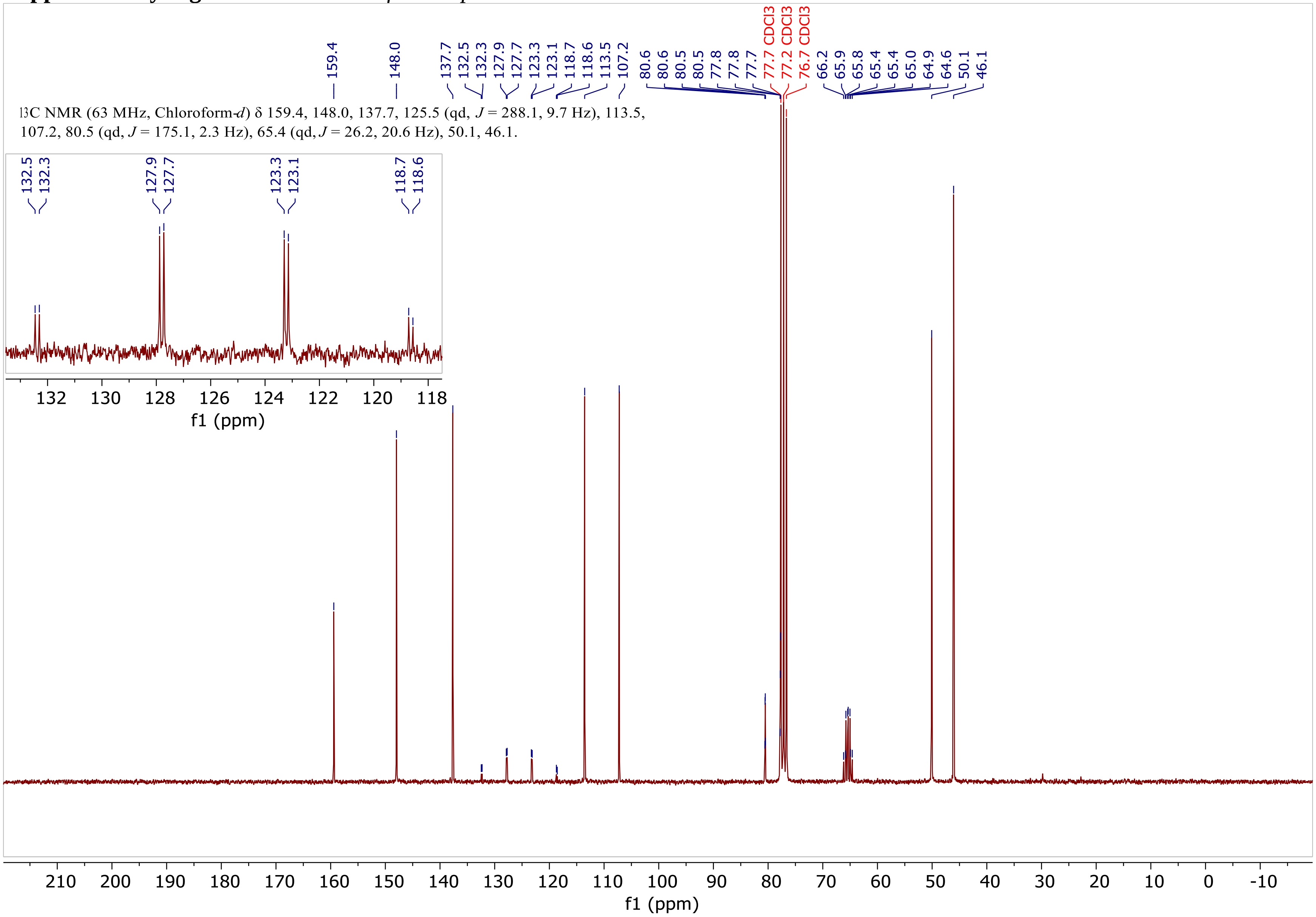

Supplementary Figure 407: *IR for compound* ***85***

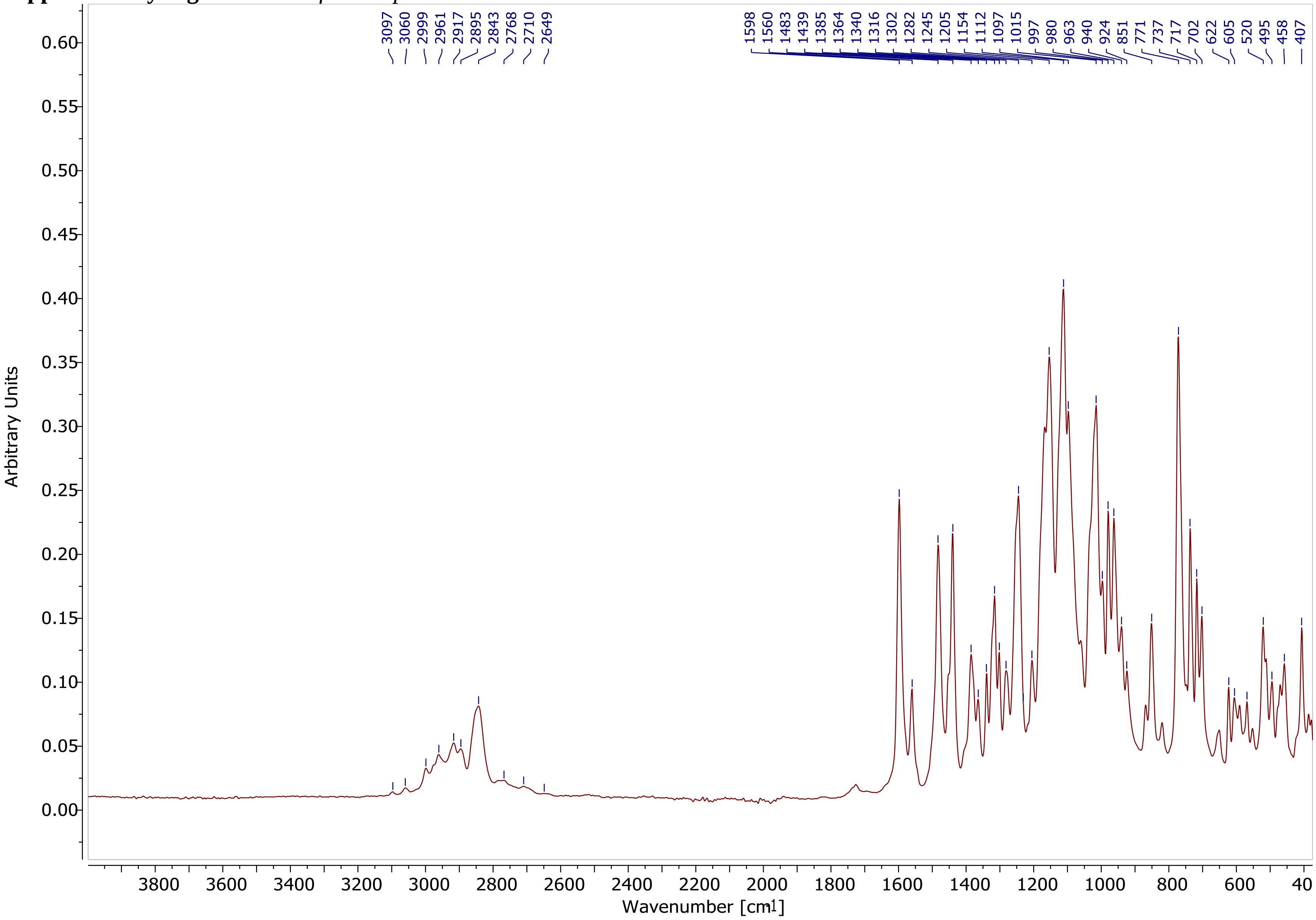

Supplementary Figure 408: *^{1}H-NMR for compound* ***86***

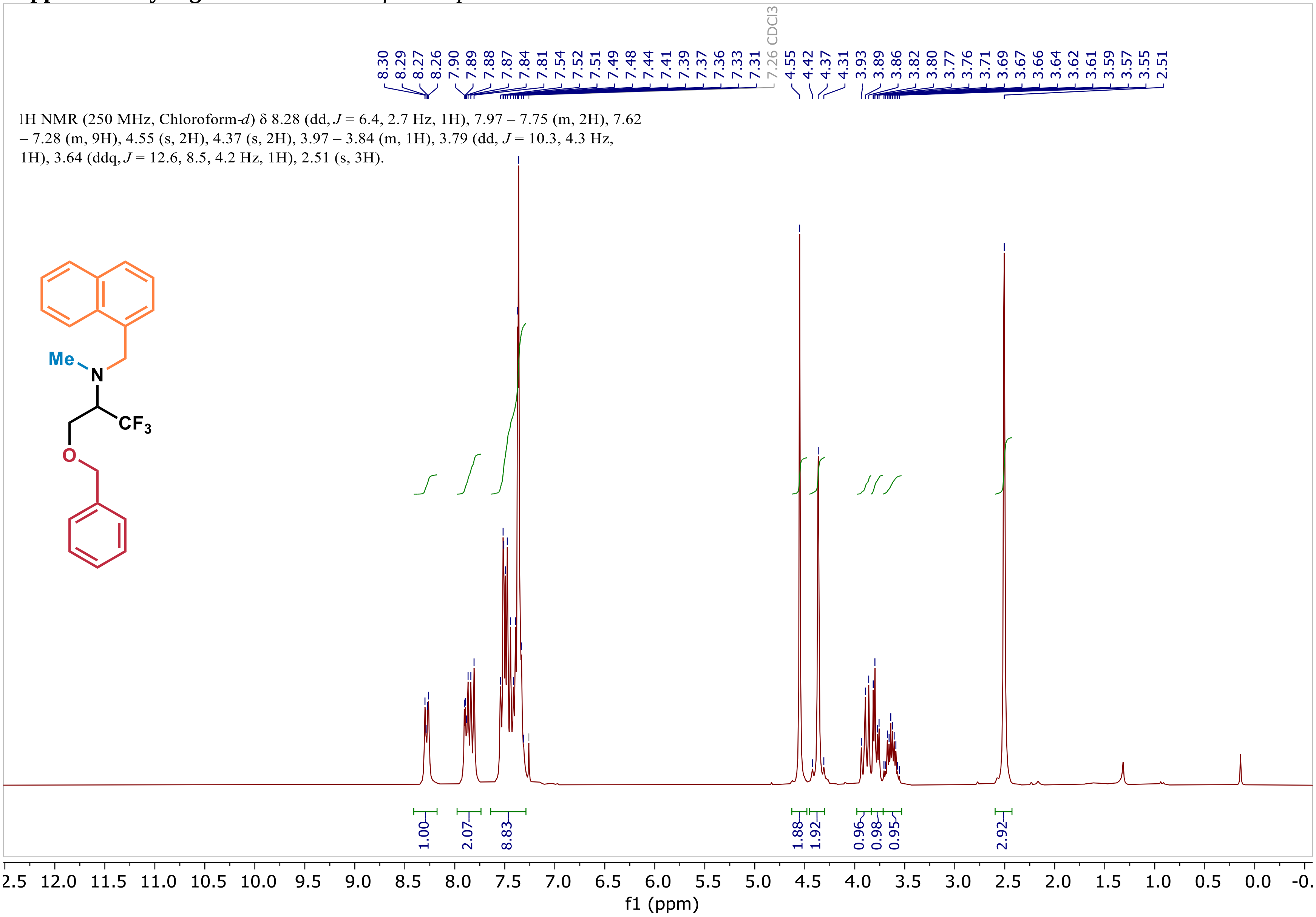

Supplementary Figure 409: *^{19}F NMR for compound* ***86***

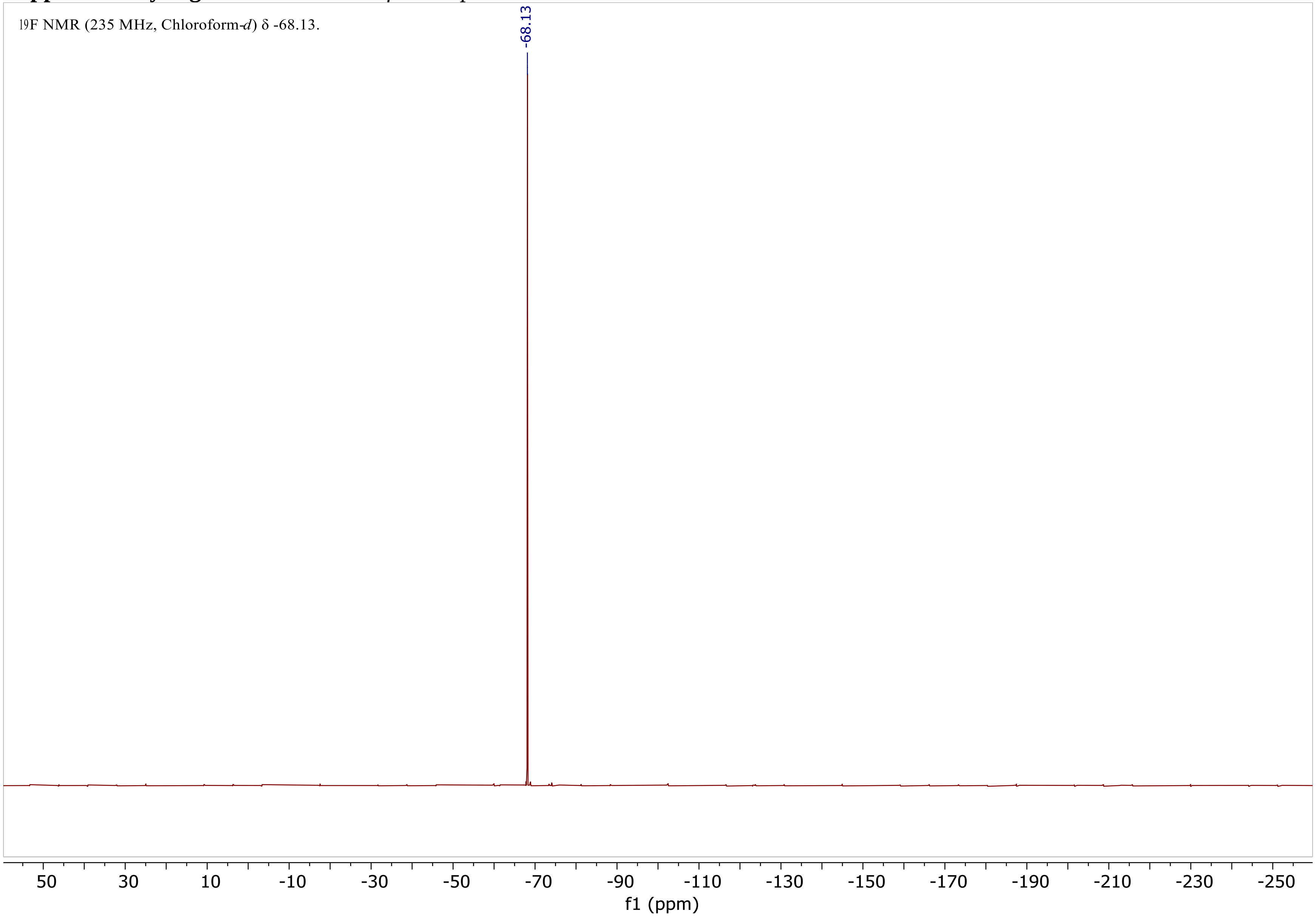

Supplementary Figure 410: *^{13}C NMR for compound* ***86***

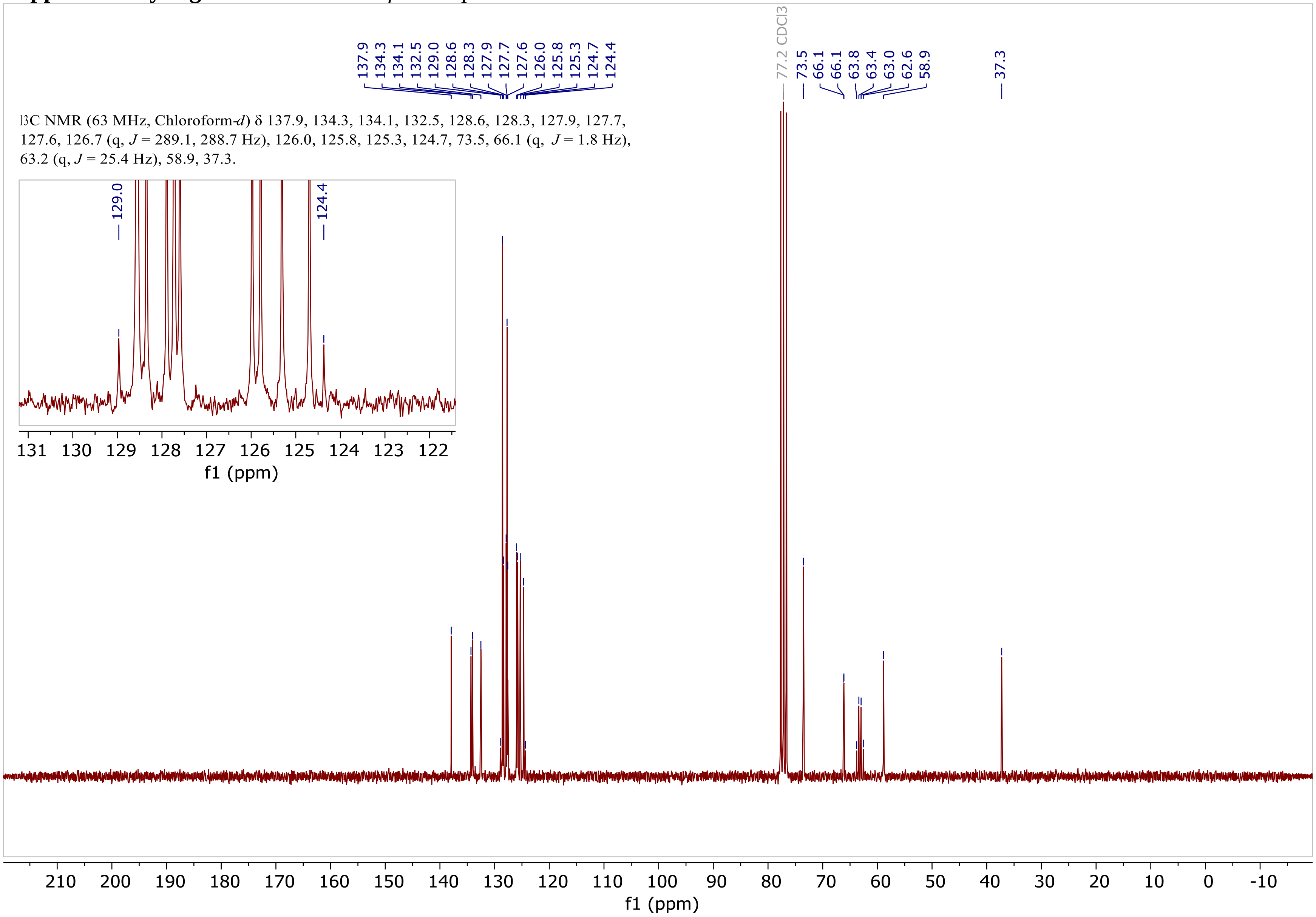

Supplementary Figure 411: *IR for compound* ***86***

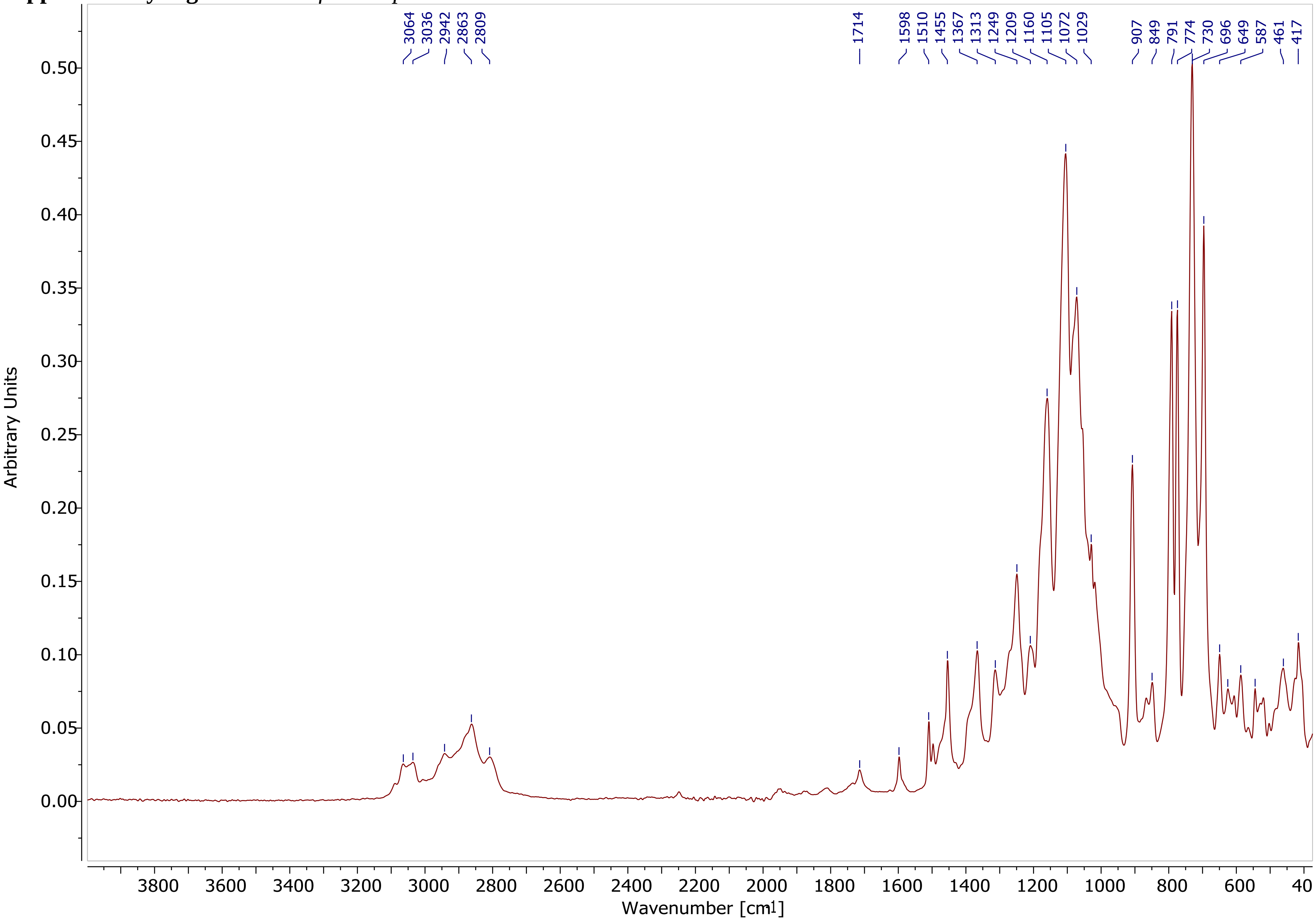

Supplementary Figure 412: *^{1}H-NMR for compound* ***87***

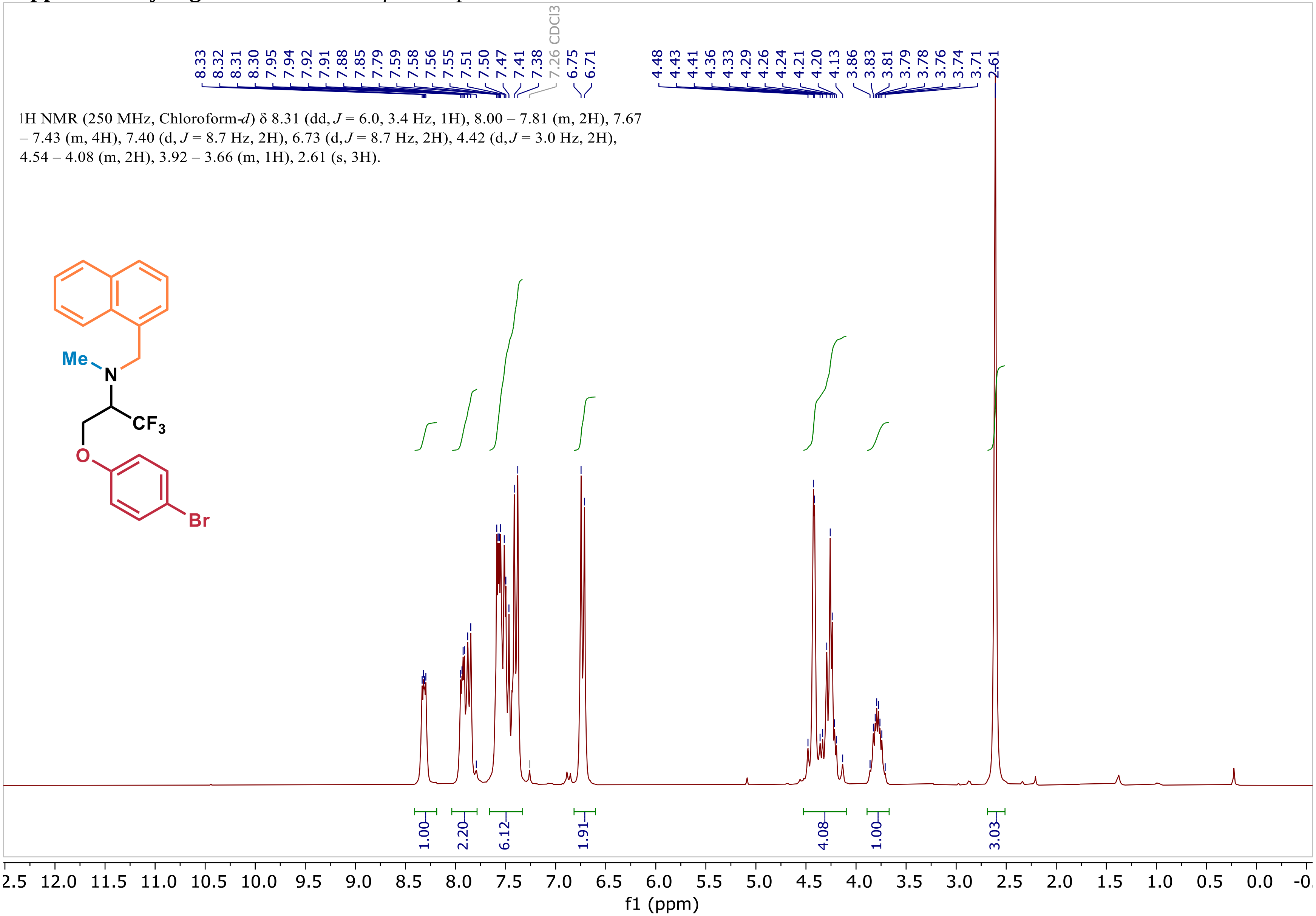

Supplementary Figure 413: *^{19}F NMR for compound* ***87***

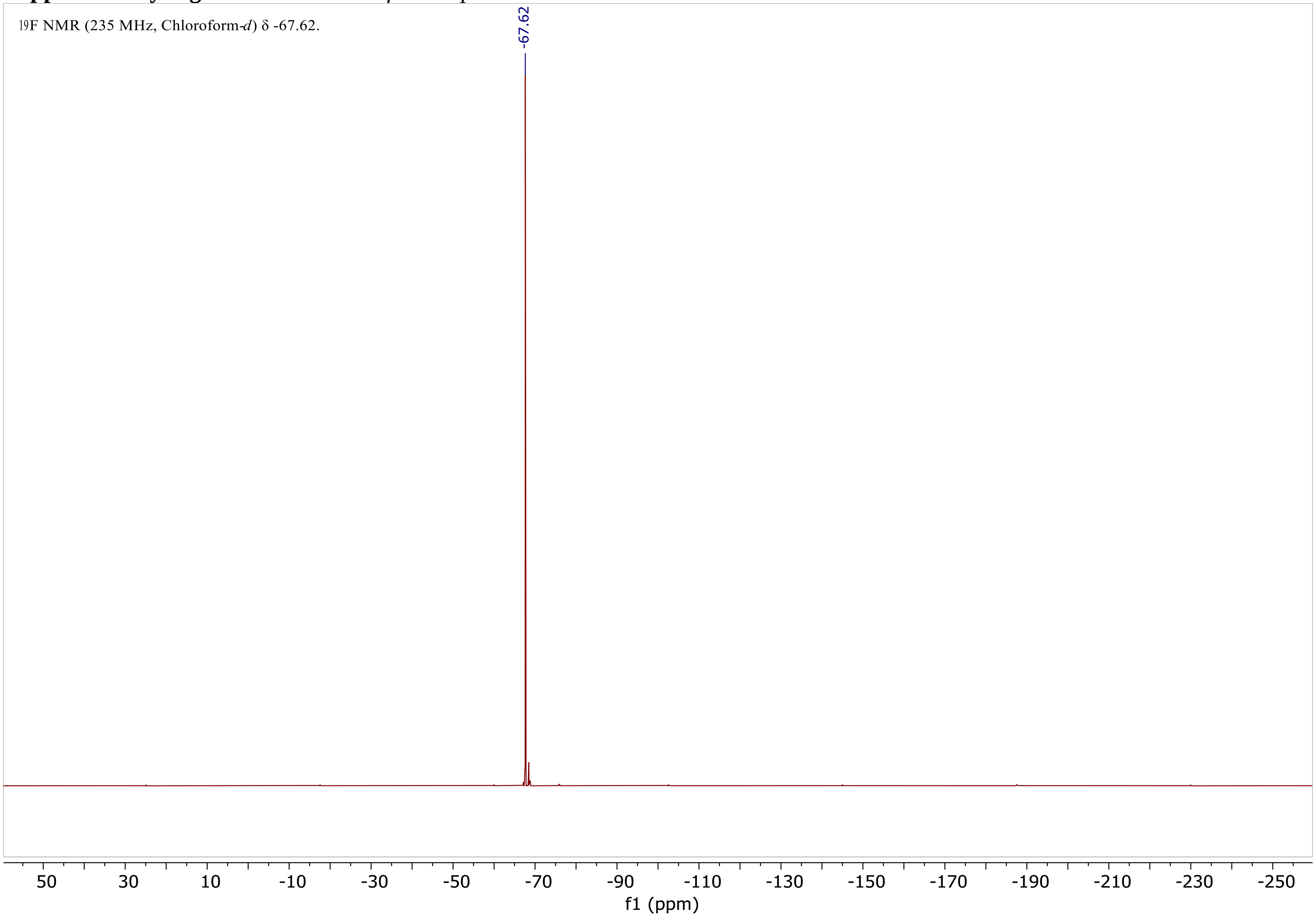

Supplementary Figure 414: *^{13}C NMR for compound* ***87***

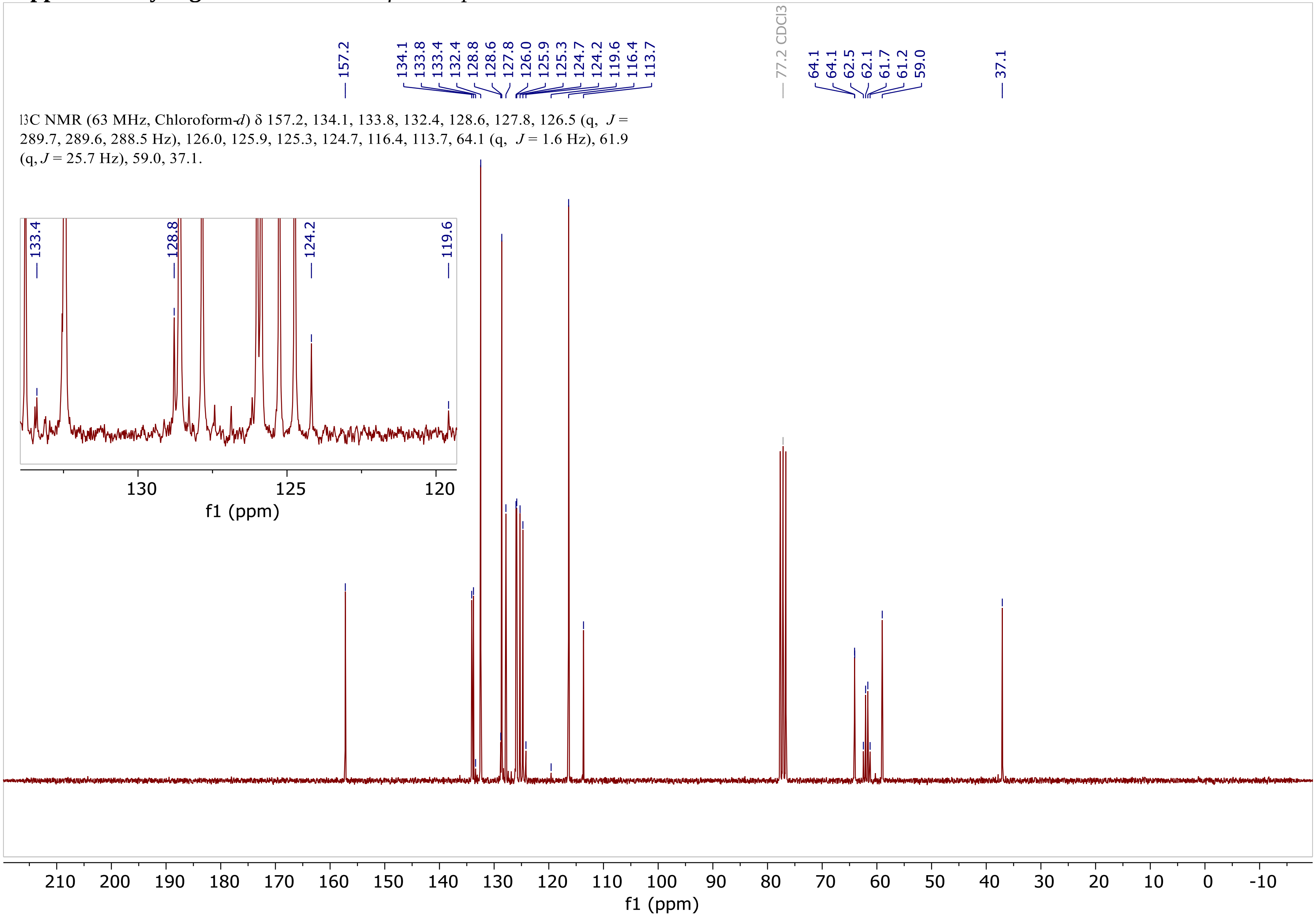

Supplementary Figure 415: *IR for compound* ***87***

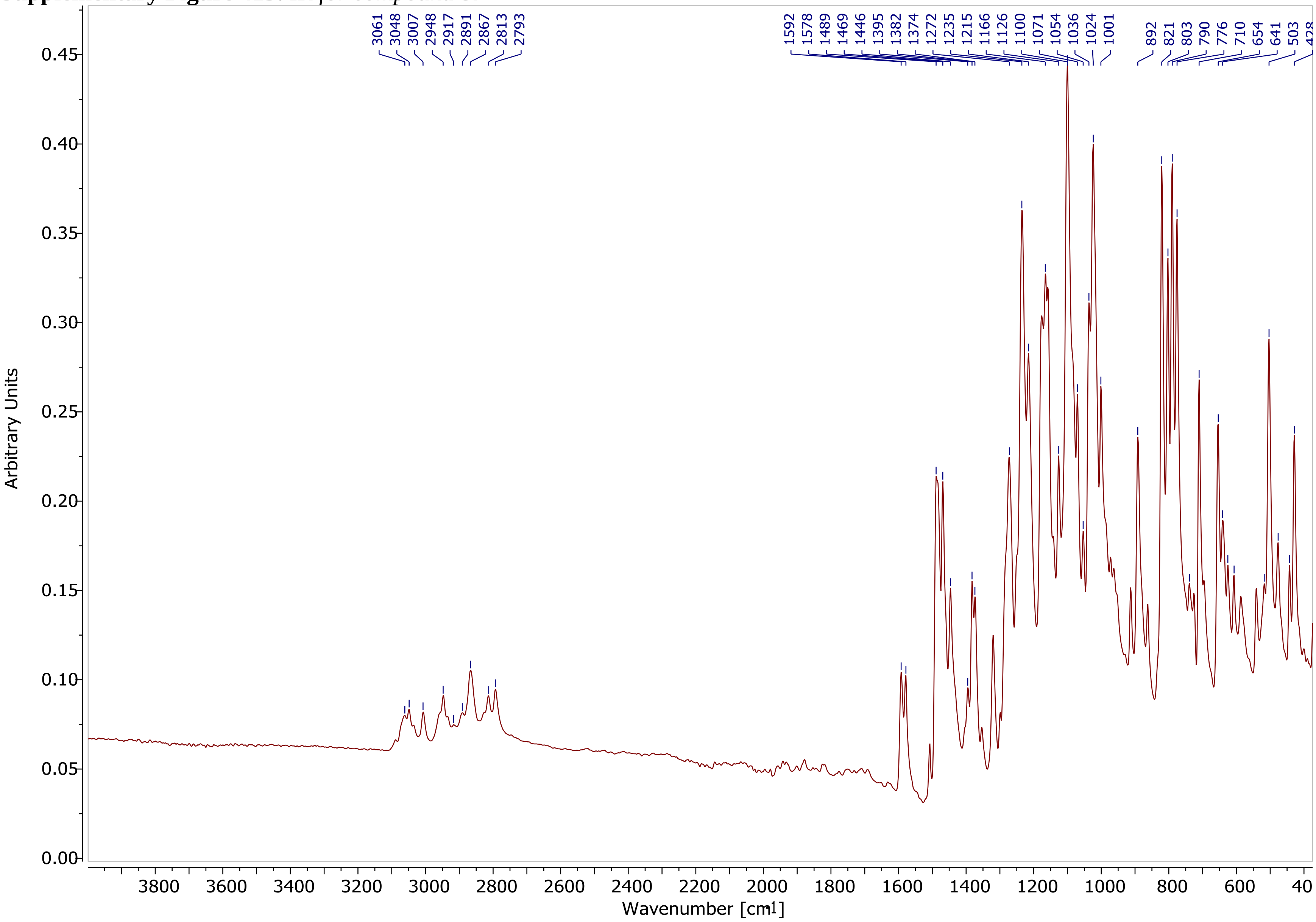

Supplementary Figure 416: *^{1}H-NMR for compound* ***88***

1H NMR (250 MHz, Chloroform-*d*) δ 8.16 (d, *J* = 8.4 Hz, 1H), 8.00 – 7.89 (m, 2H), 7.84 (t, *J* = 8.3 Hz, 2H), 7.61 (t, *J* = 7.3 Hz, 1H), 7.54 – 7.37 (m, 5H), 7.31 (t, *J* = 7.6 Hz, 1H), 4.74 (dd, *J* = 11.7, 8.6 Hz, 1H), 4.53 (dd, *J* = 11.8, 4.5 Hz, 1H), 4.38 (s, 2H), 3.80 (ddq, *J* = 13.1, 8.5, 4.8, 4.2 Hz, 1H), 2.62 (s, 3H).

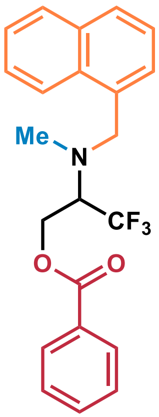

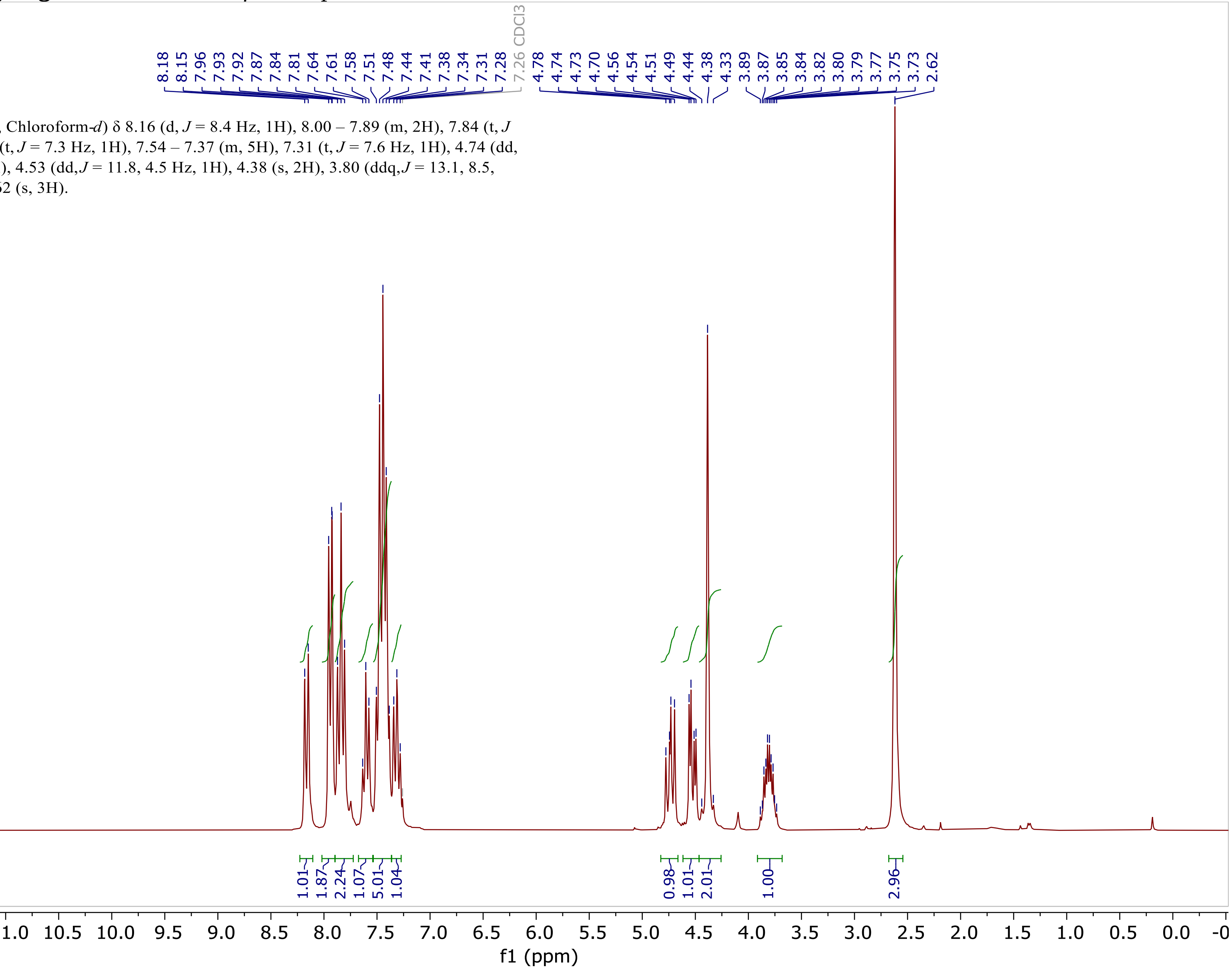

Supplementary Figure 417: *^{19}F NMR for compound **88***

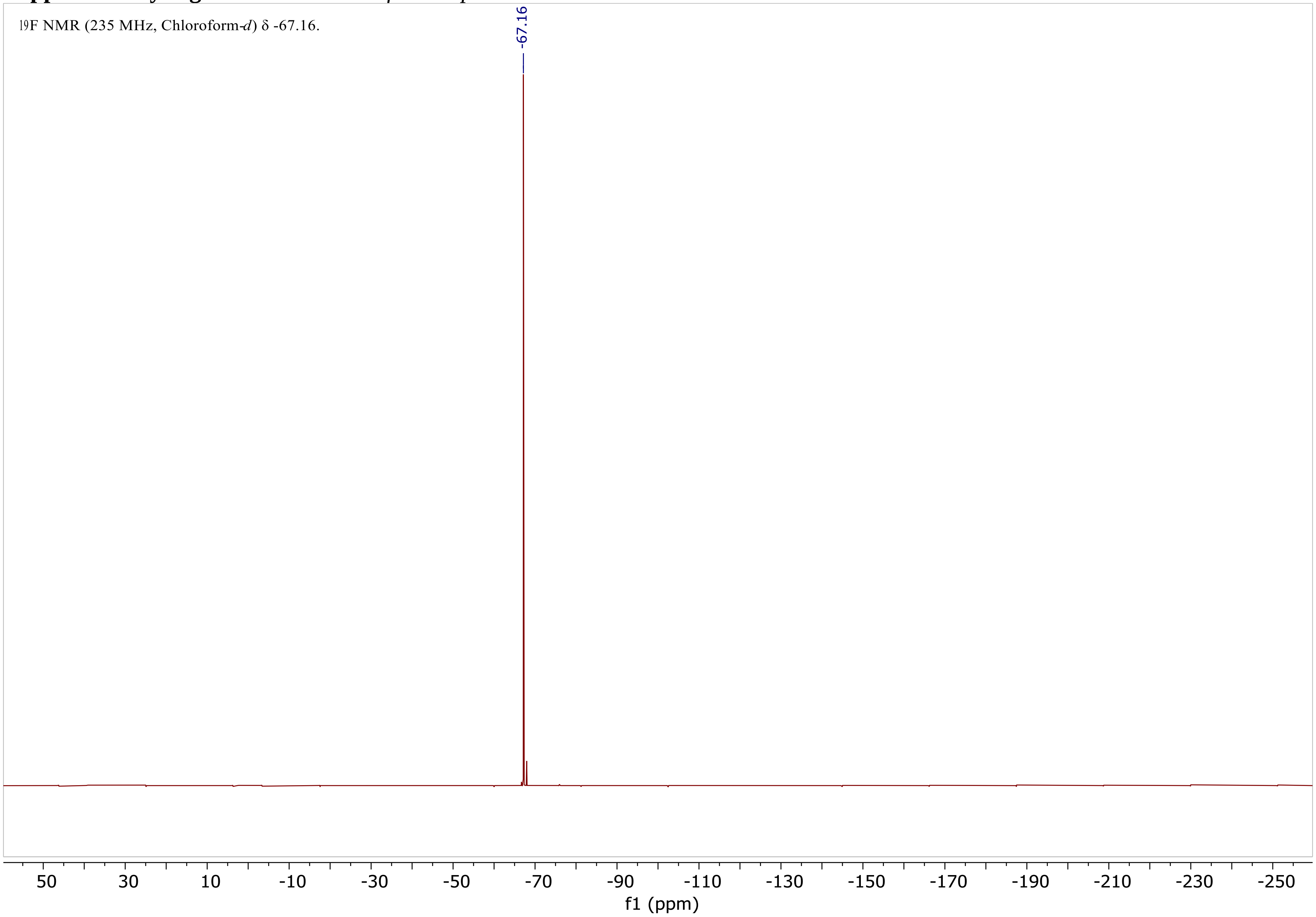

Supplementary Figure 418: *^{13}C NMR for compound* ***88***

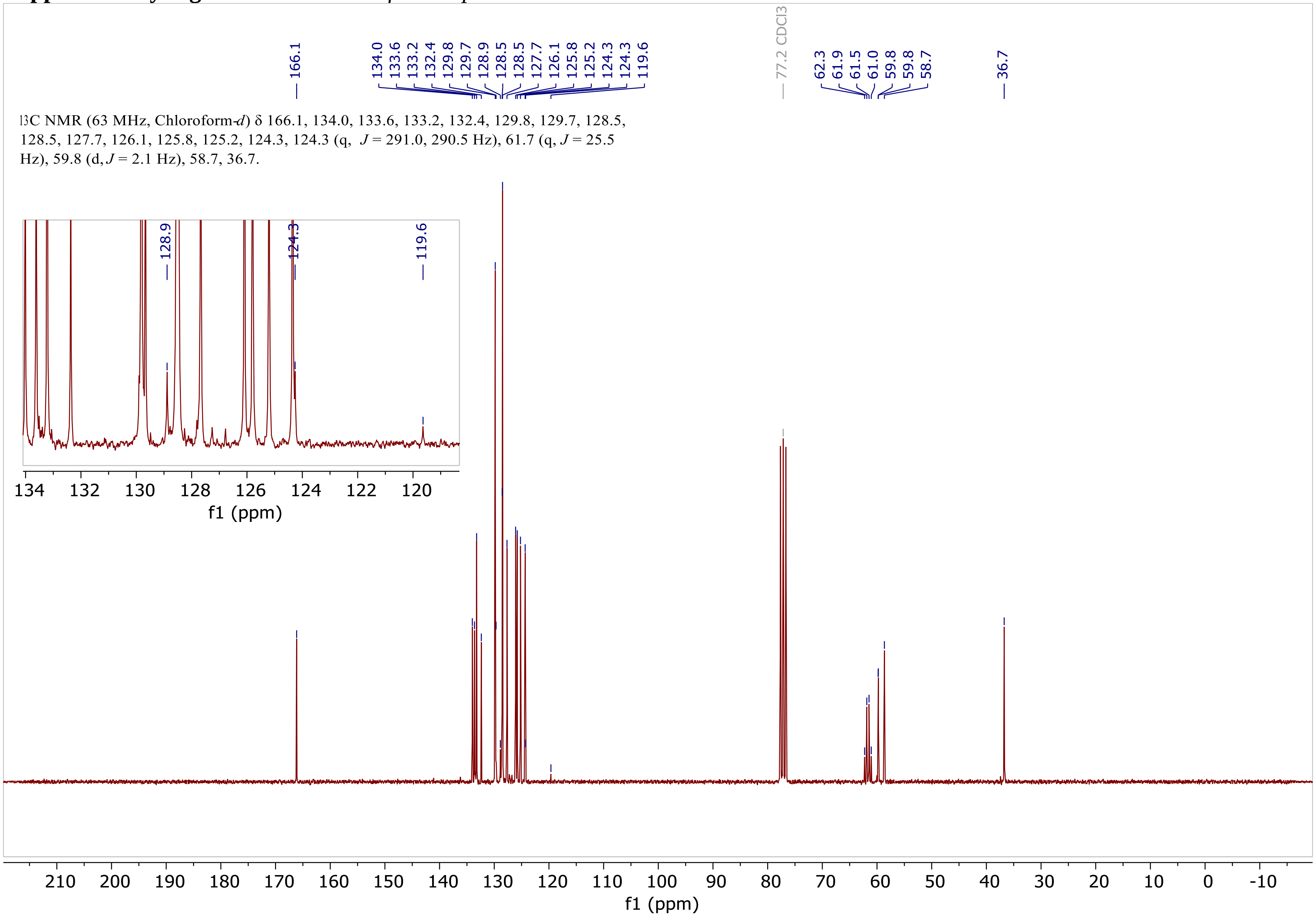

Supplementary Figure 419: *IR for compound* ***88***

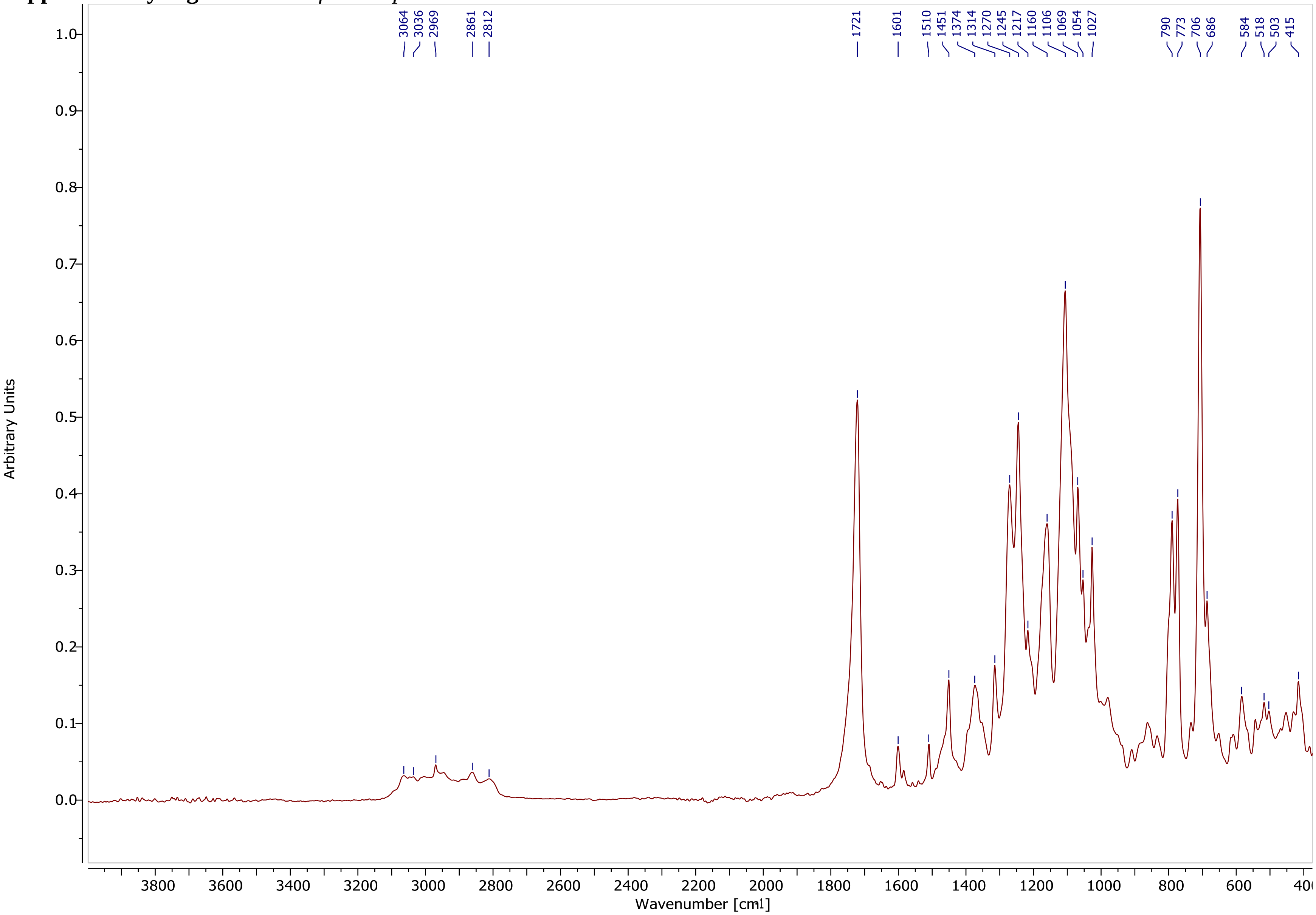

Supplementary Figure 420: *¹H-NMR for compound* ***89***

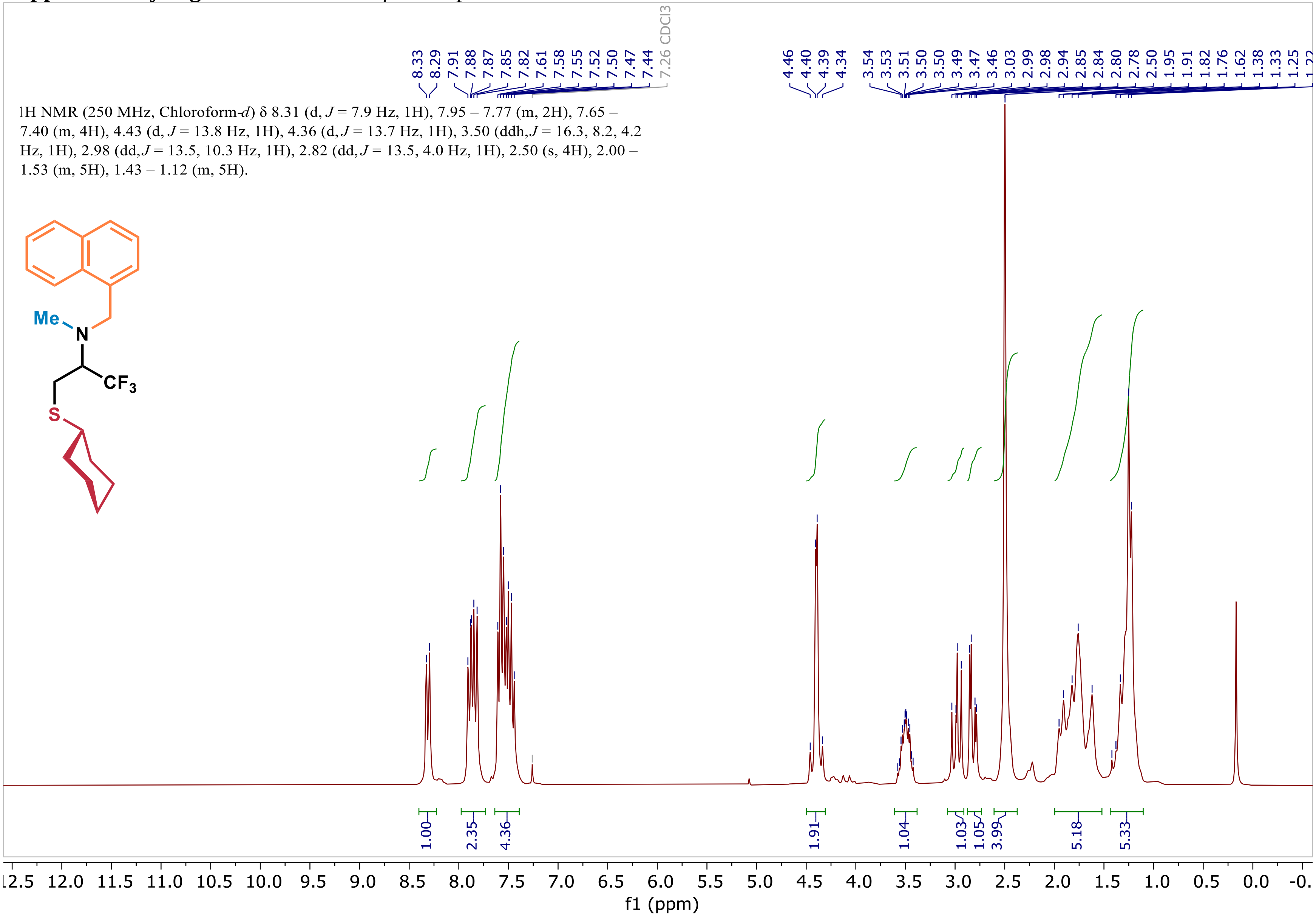

Supplementary Figure 421: ^{19}*F NMR for compound* ***89***

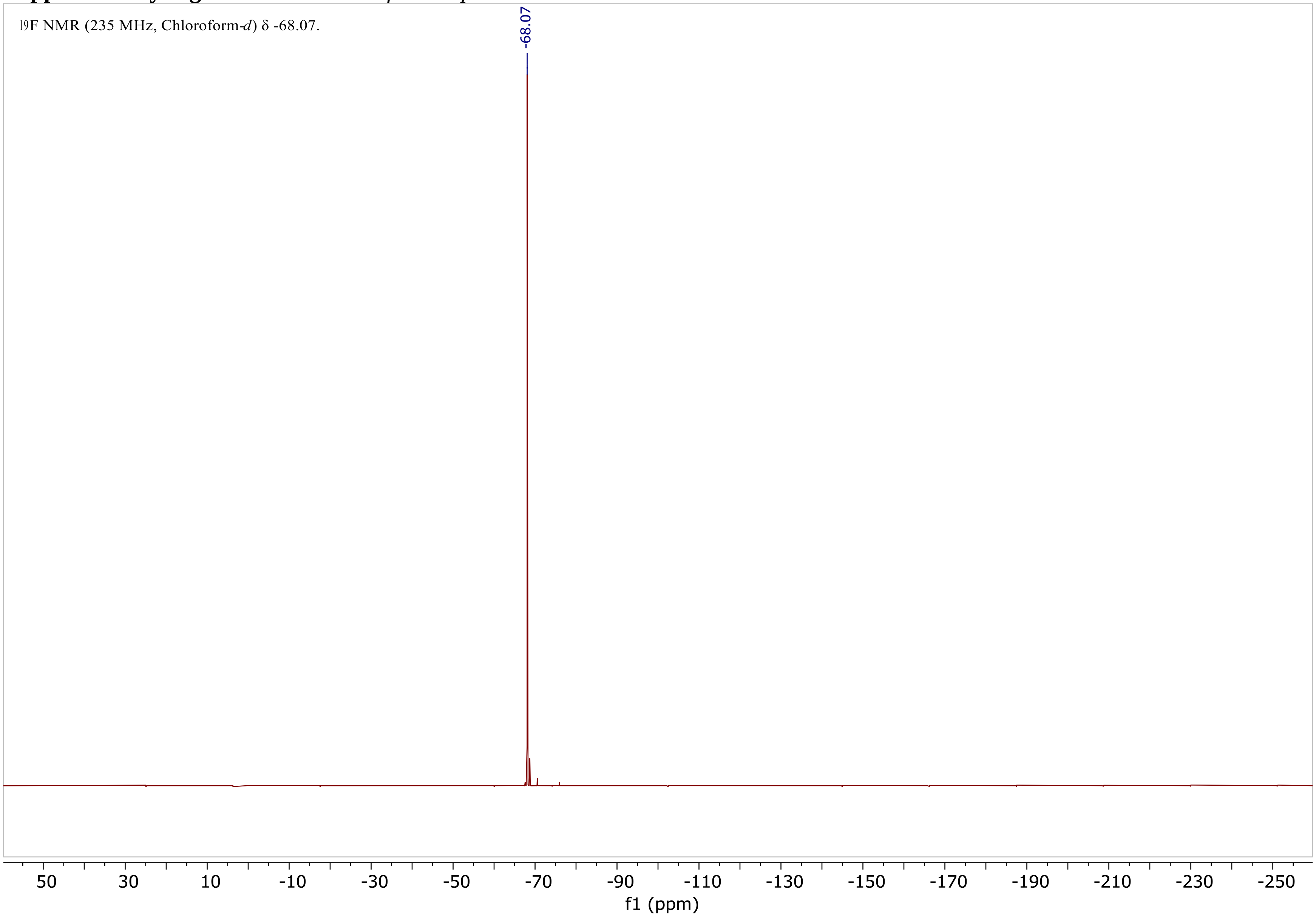

Supplementary Figure 422: *^{13}C NMR for compound* ***89***

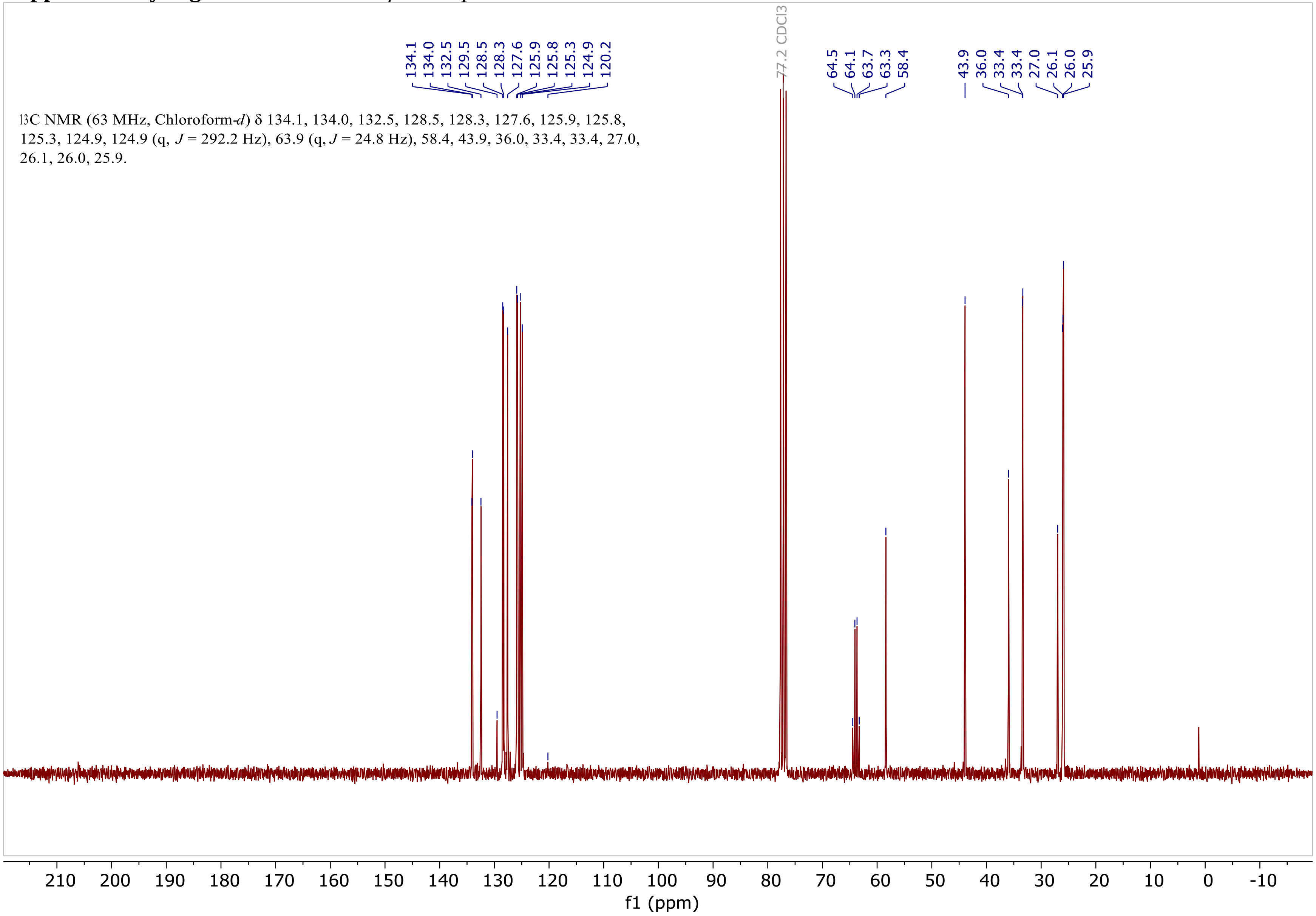

Supplementary Figure 423: *IR for compound* ***89***

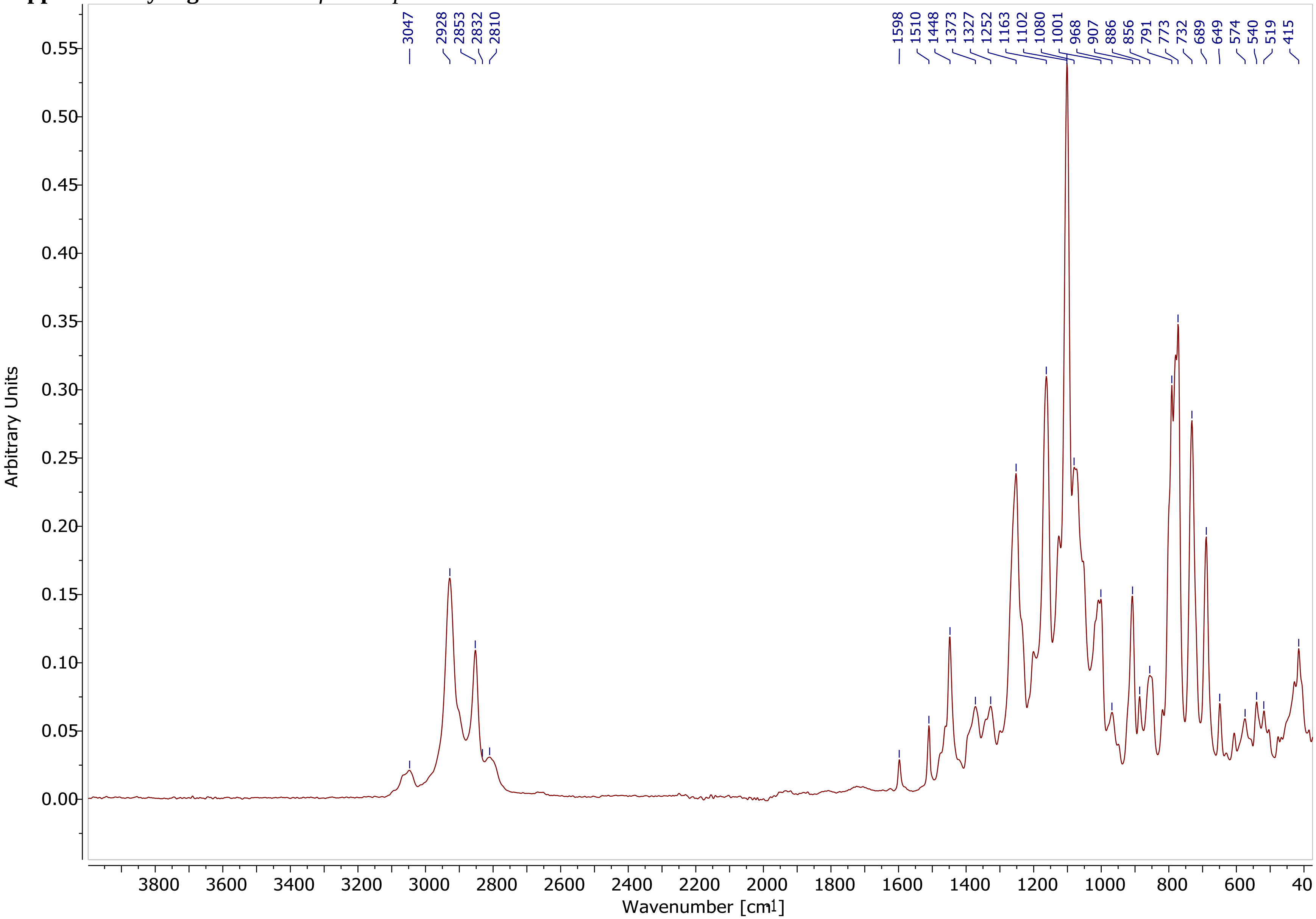

Supplementary Figure 424: *^{1}H-NMR for compound* ***90***

1H NMR (250 MHz, Chloroform-*d*) δ 8.28 (d, *J* = 7.5 Hz, 1H), 8.00 – 7.80 (m, 2H), 7.68 – 7.40 (m, 5H), 7.20 (d, *J* = 8.5 Hz, 2H), 7.10 (d, *J* = 8.6 Hz, 2H), 4.39 (td, *J* = 13.5, 6.5 Hz, 2H), 3.52 (tt, *J* = 12.9, 6.4 Hz, 1H), 3.27 (dd, *J* = 13.6, 9.6 Hz, 1H), 3.17 (dd, *J* = 13.5, 7.3 Hz, 1H), 2.56 (s, 3H).

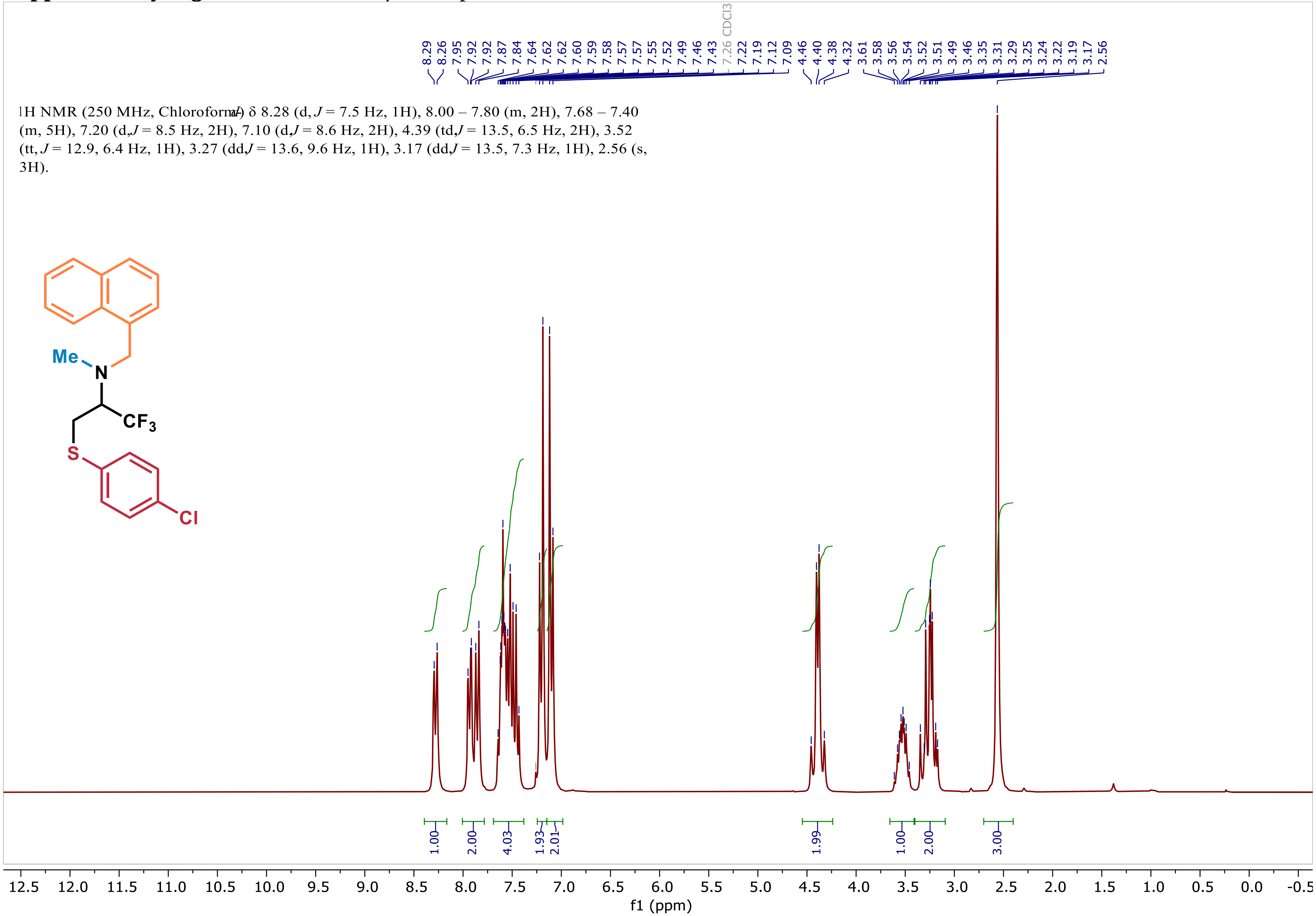

Supplementary Figure 425: *^{19}F NMR for compound* ***90***

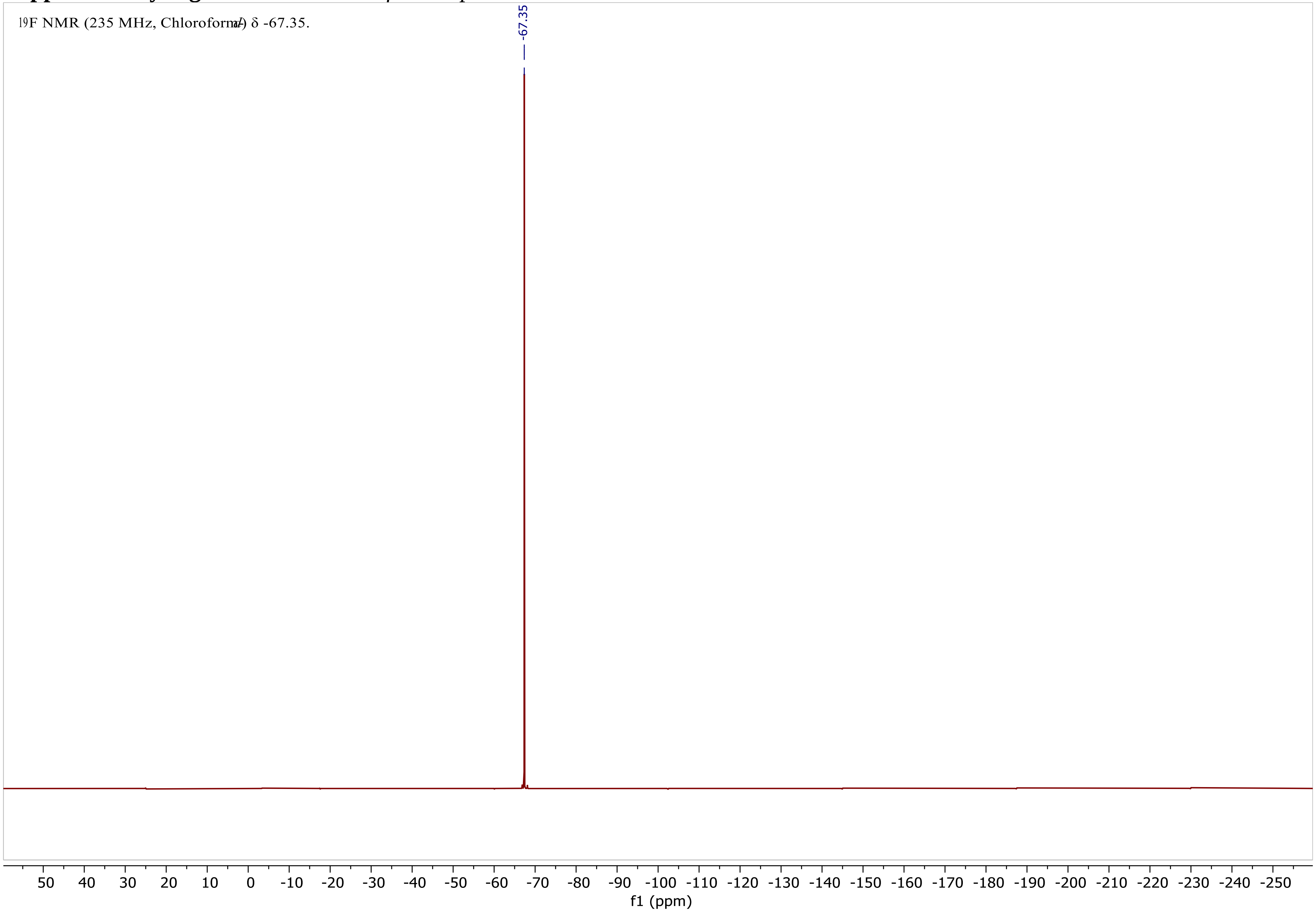

Supplementary Figure 426: *^{13}C NMR for compound **90***

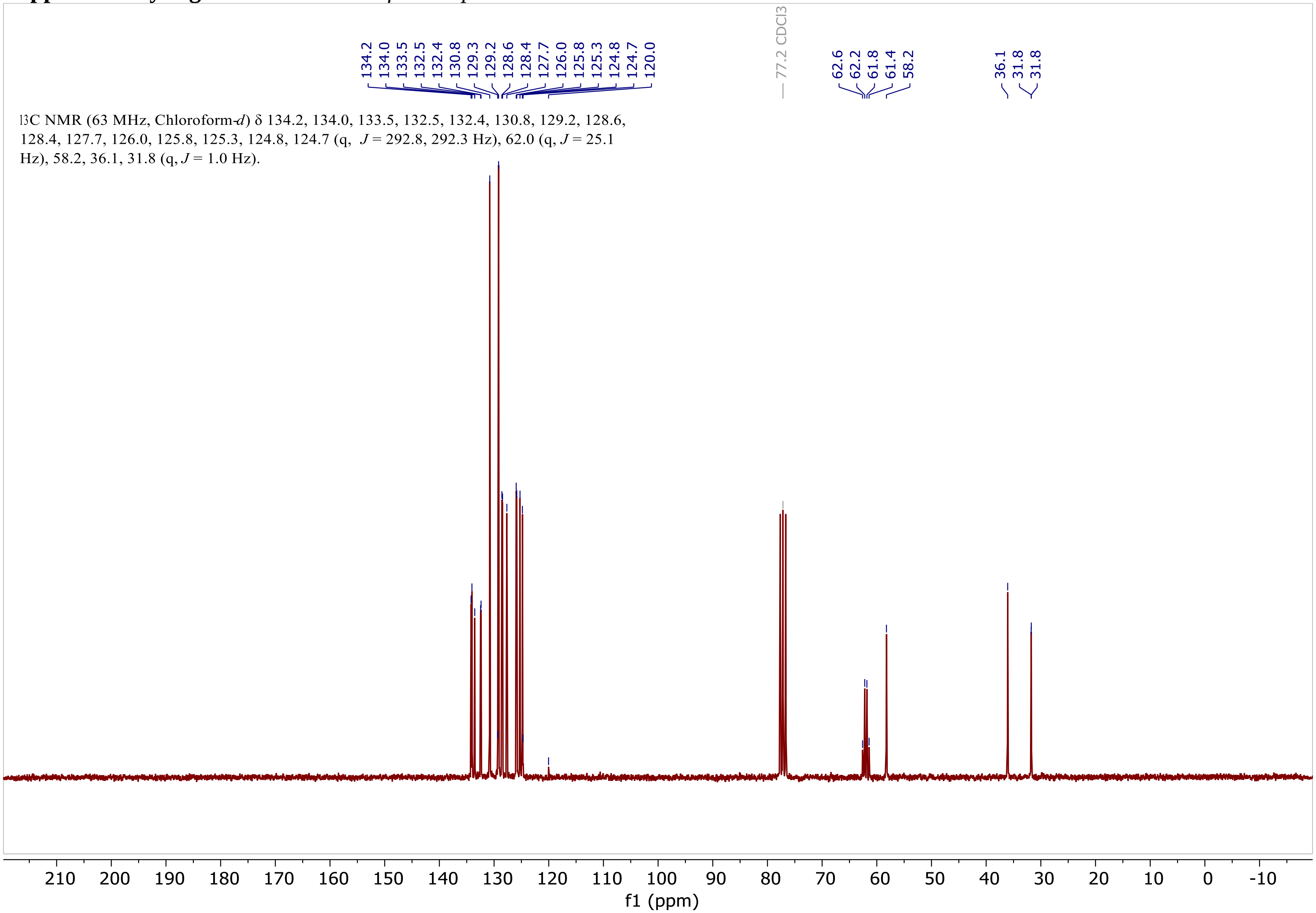

Supplementary Figure 427: *IR for compound **90***

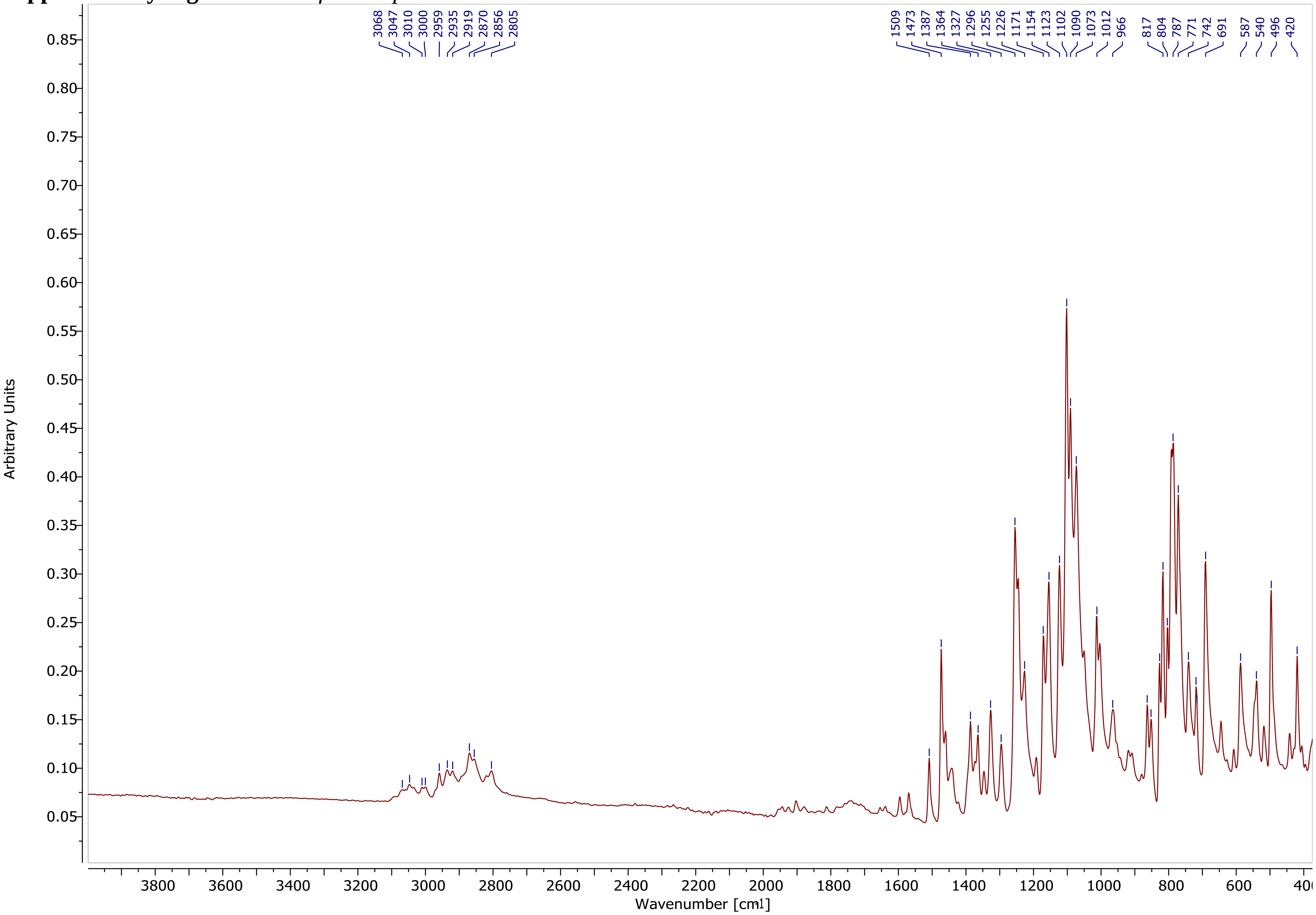

Supplementary Figure 428: *^{1}H-NMR for compound* ***91***

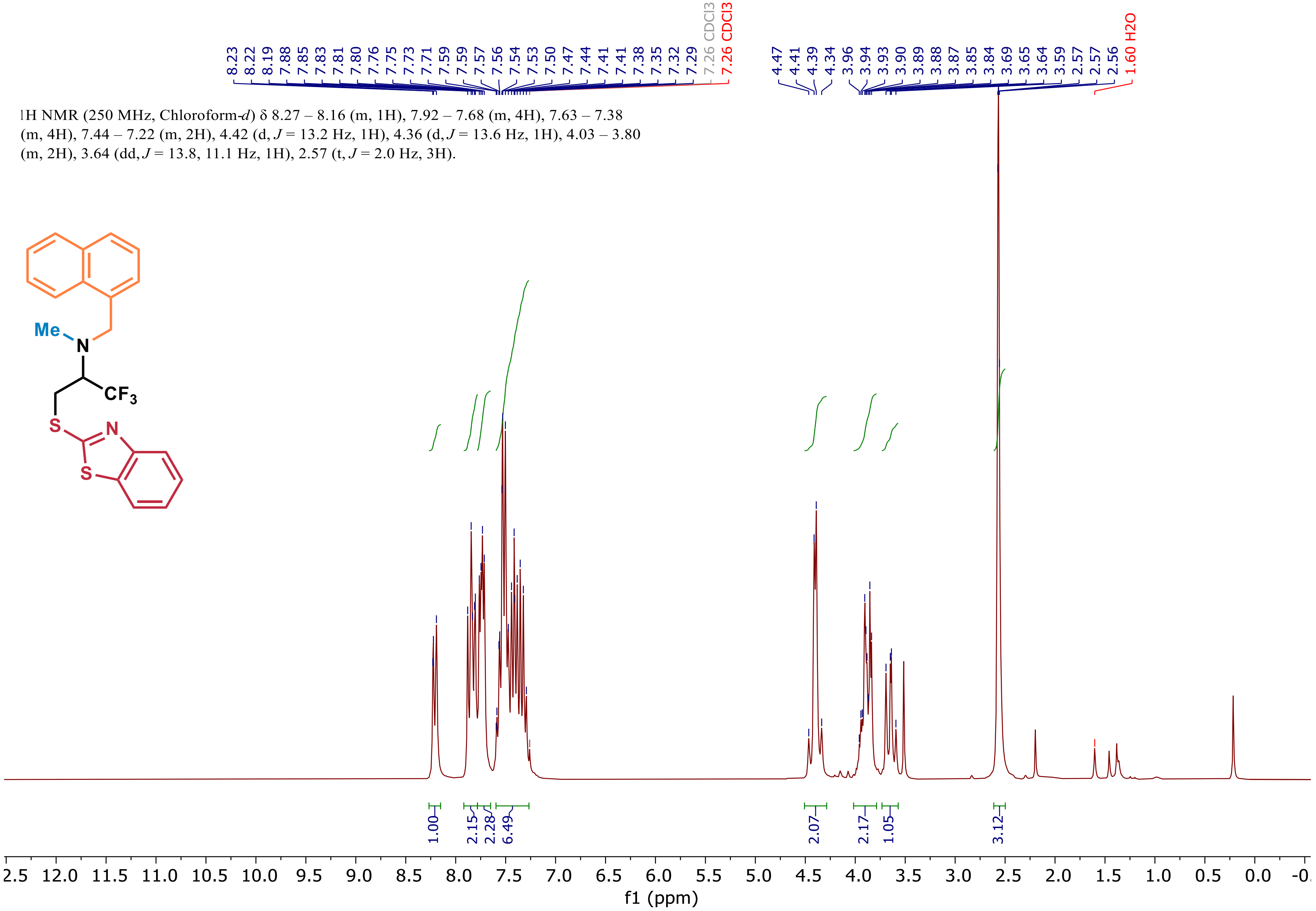

Supplementary Figure 429: *^{19}F NMR for compound* ***91***

19F NMR (235 MHz, Chloroform-*d*) δ -67.14.

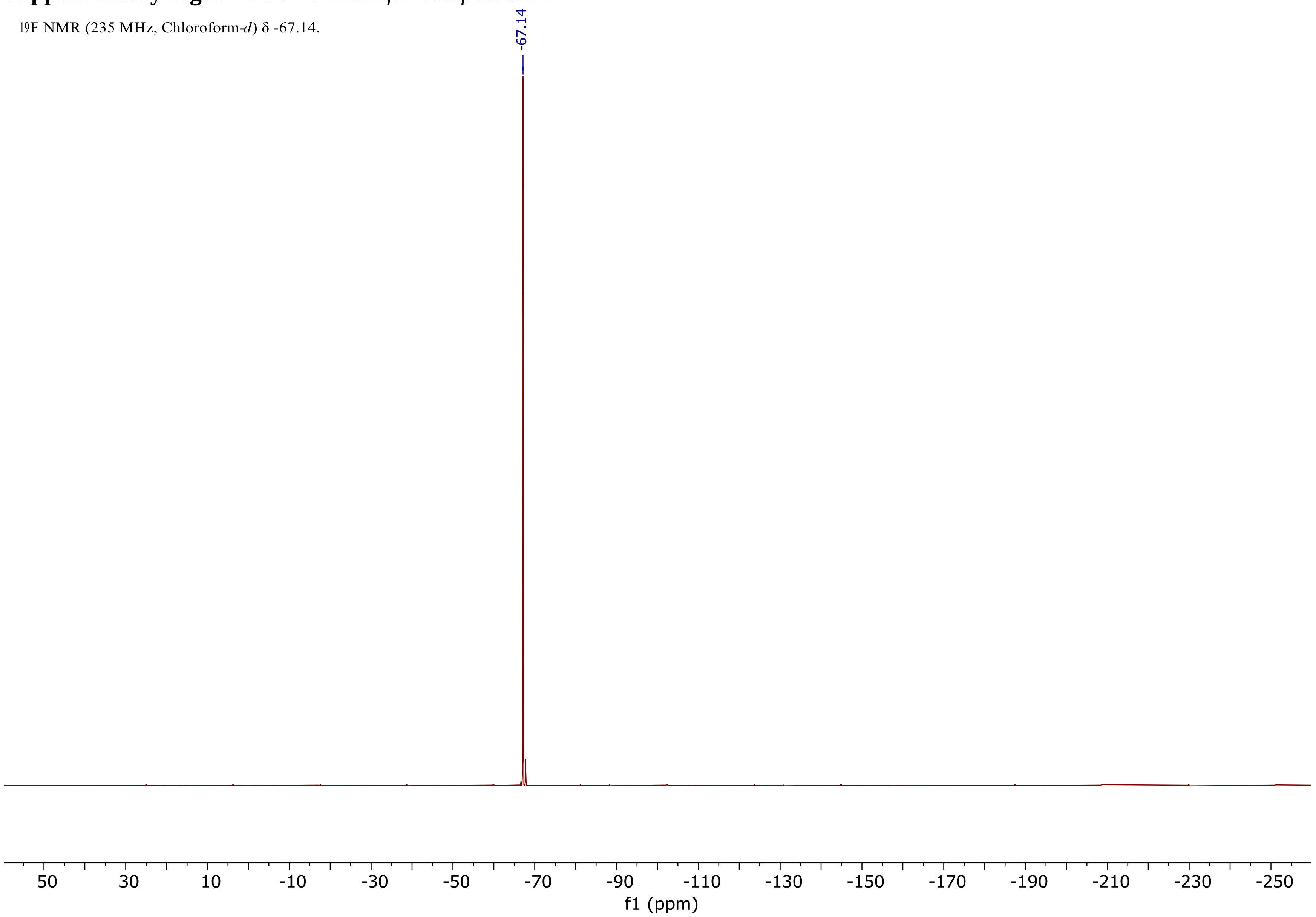

Supplementary Figure 430: ^{13}*C NMR for compound* ***91***

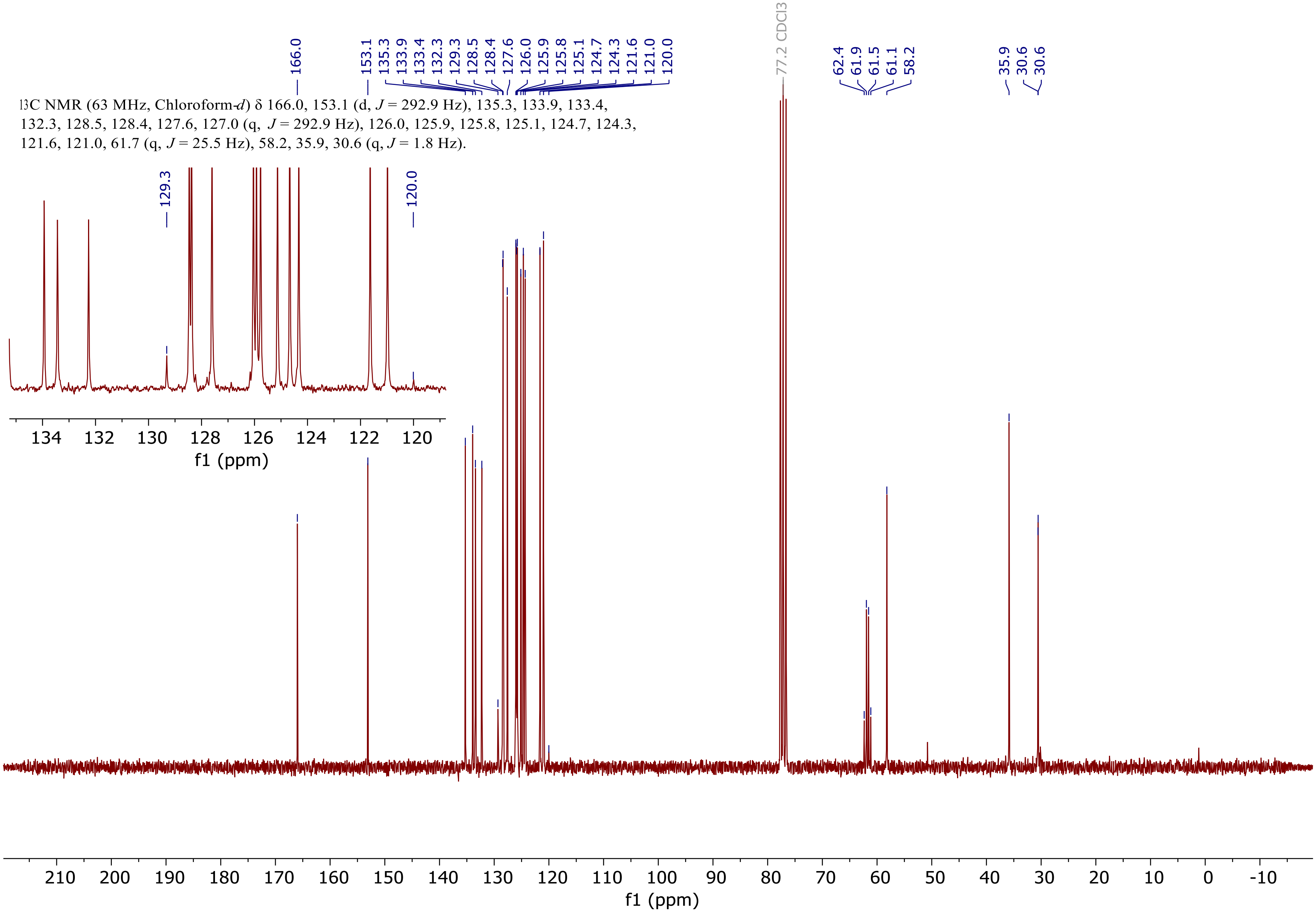

^{13}C NMR (63 MHz, Chloroform-*d*) δ 166.0, 153.1 (d, J = 292.9 Hz), 135.3, 133.9, 133.4, 132.3, 128.5, 128.4, 127.6, 127.0 (q, J = 292.9 Hz), 126.0, 125.9, 125.8, 125.1, 124.7, 124.3, 121.6, 121.0, 61.7 (q, J = 25.5 Hz), 58.2, 35.9, 30.6 (q, J = 1.8 Hz).

Supplementary Figure 431: *IR for compound* ***91***

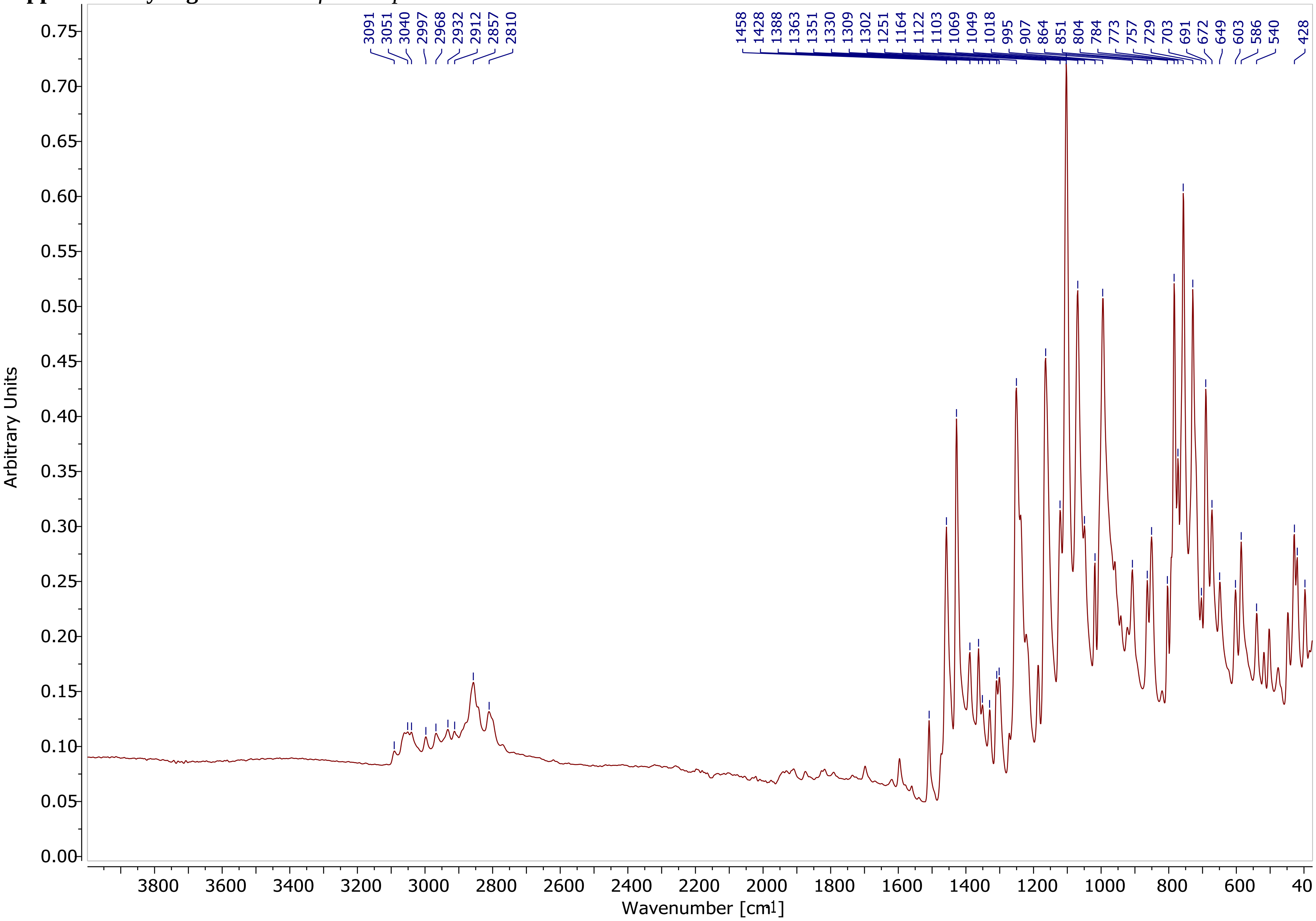

Supplementary Figure 432: *^{1}H-NMR for compound* ***92***

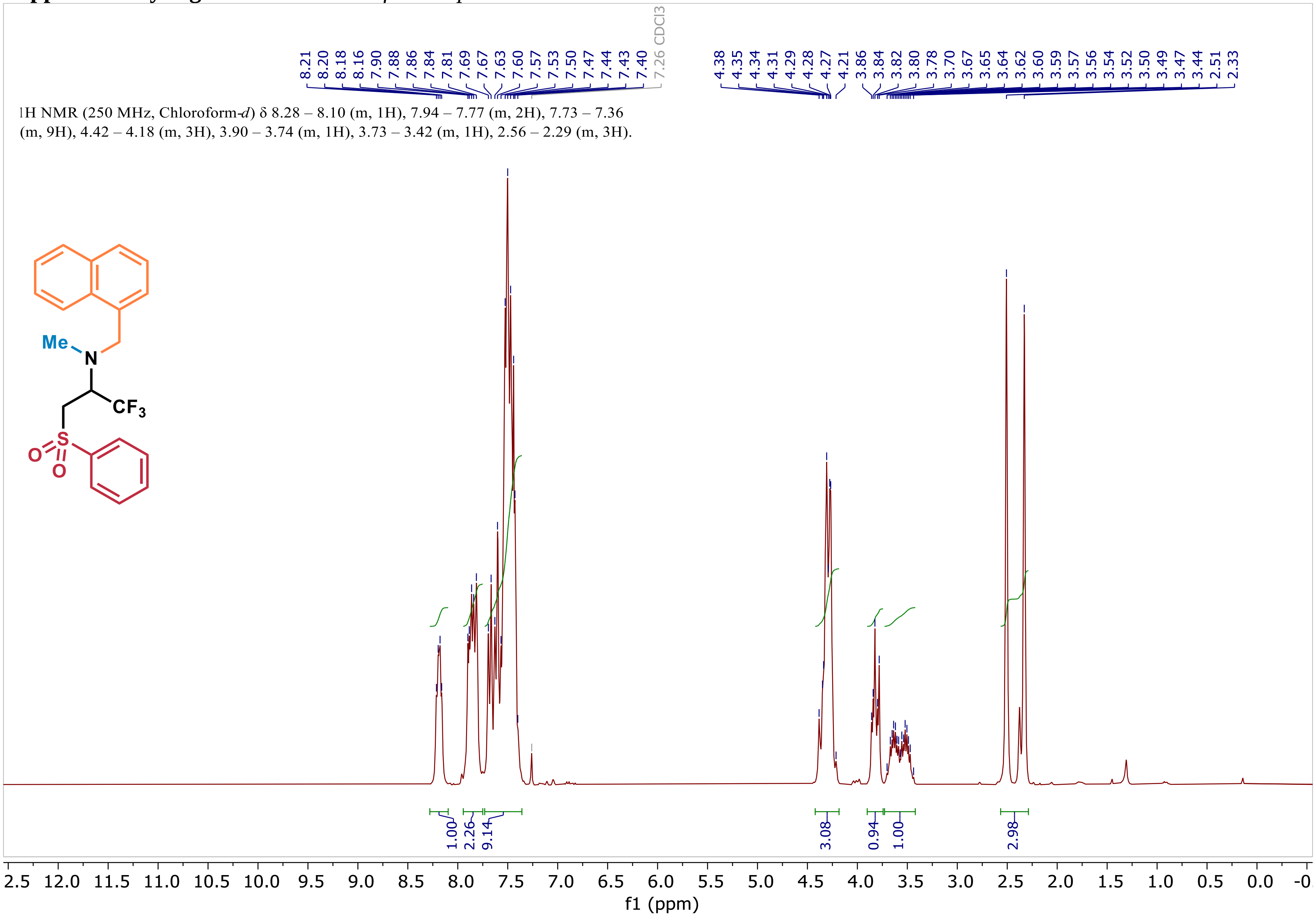

Supplementary Figure 433: *^{19}F NMR for compound* ***92***

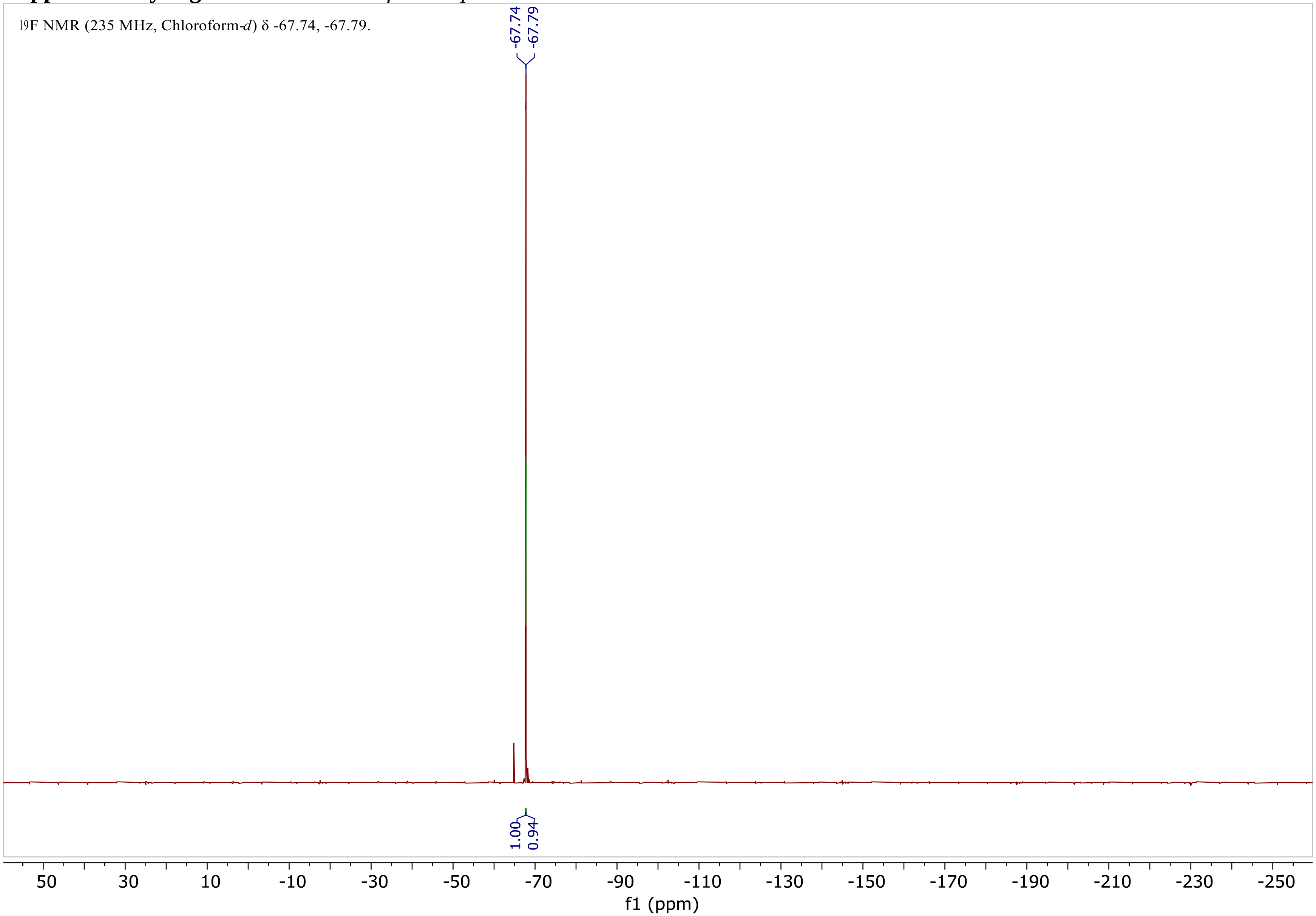

Supplementary Figure 434: *^{13}C NMR for compound **92***

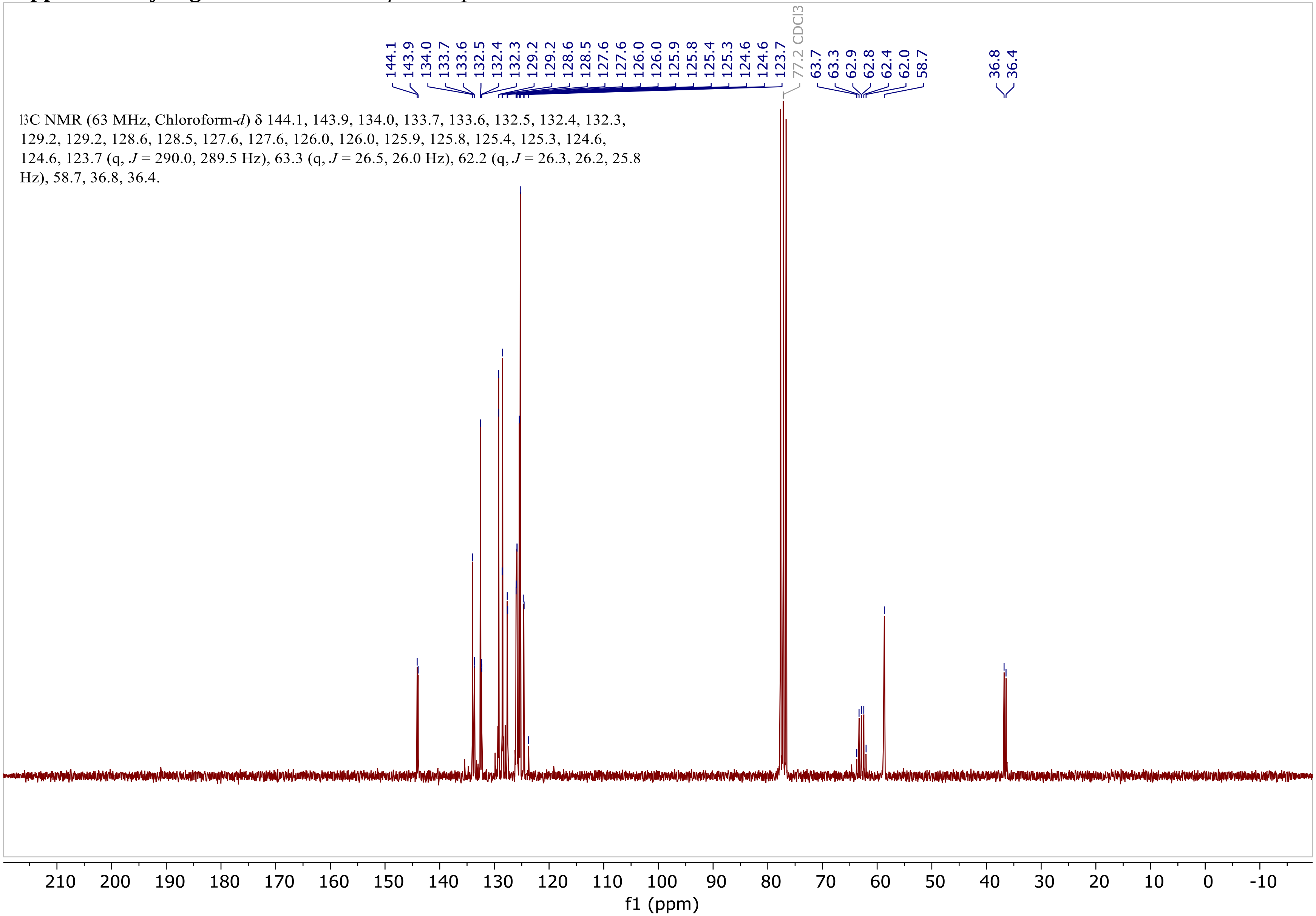

Supplementary Figure 435: *IR for compound **92***

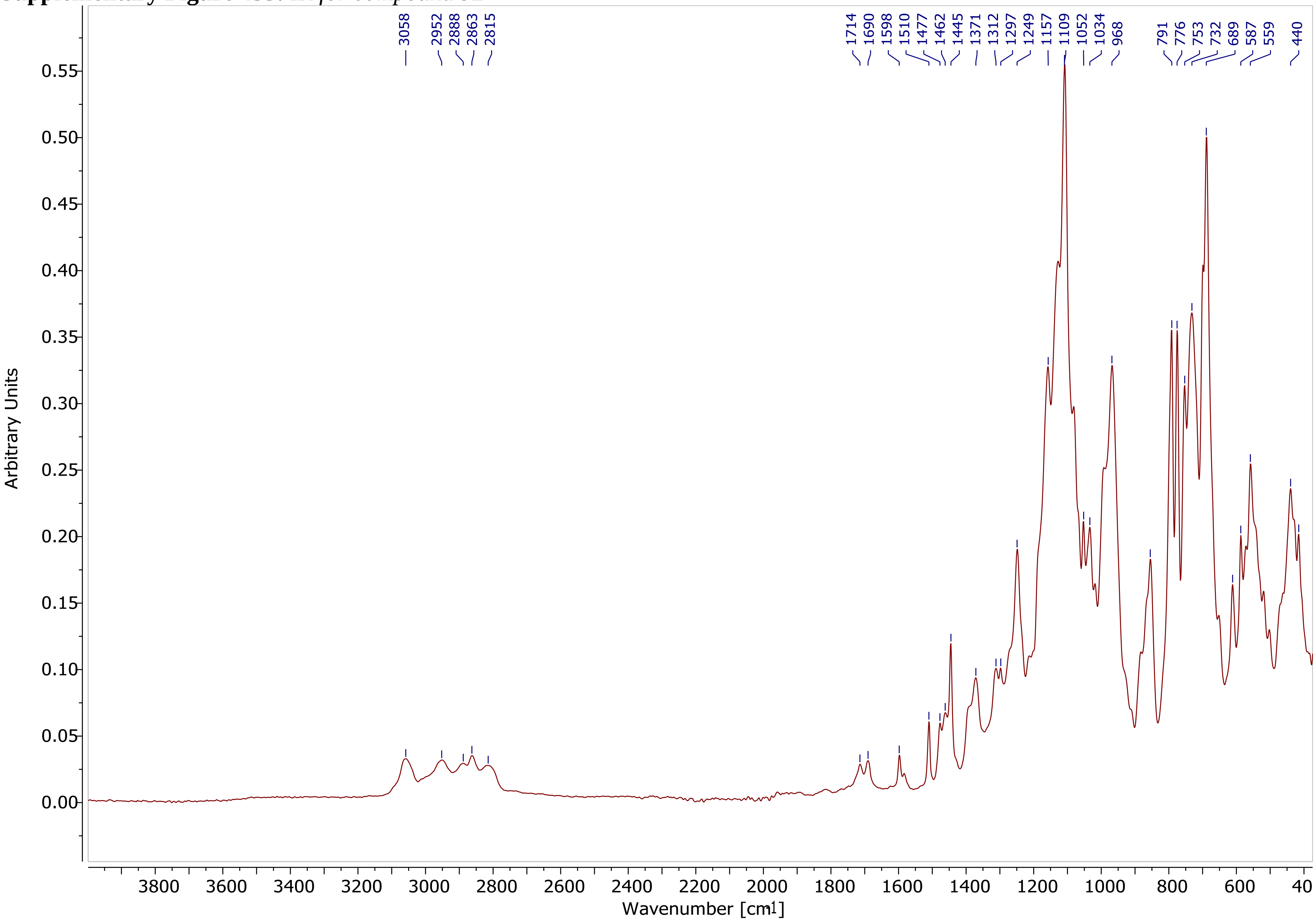

Supplementary Figure 436: *^{1}H-NMR for compound **93***

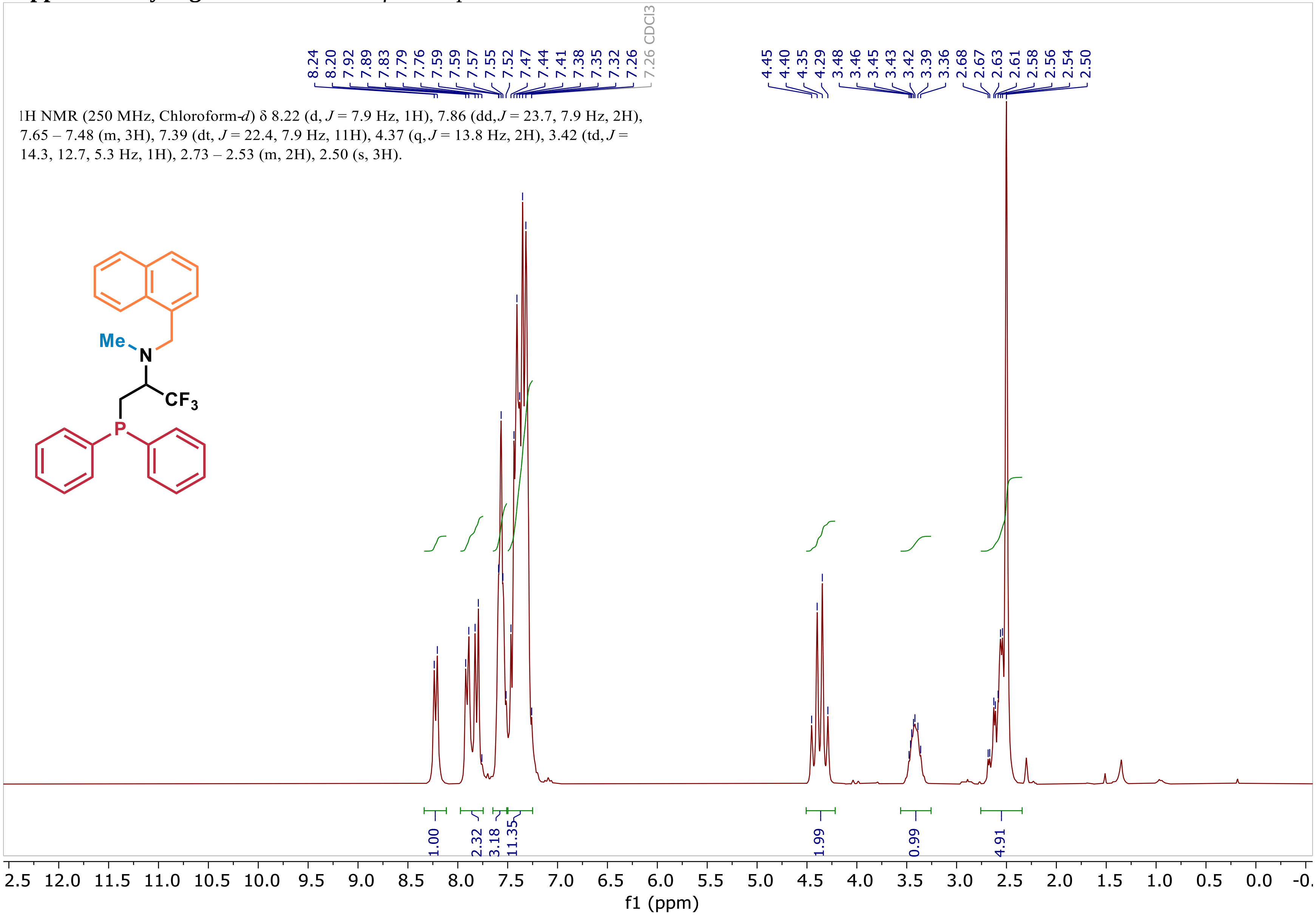

Supplementary Figure 437: *^{19}F NMR for compound* ***93***

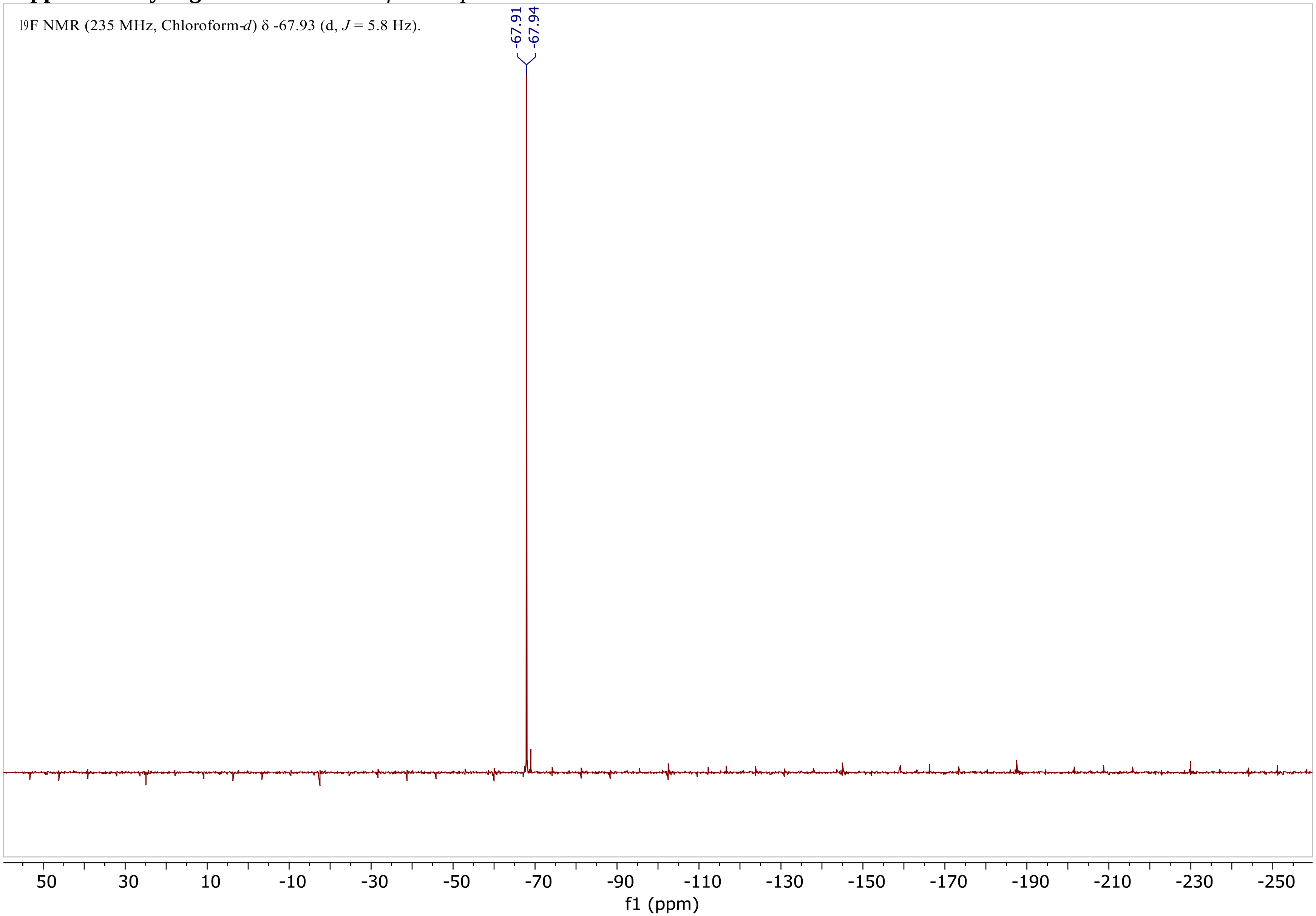

Supplementary Figure 438: *^{31}P NMR for compound* ***93***

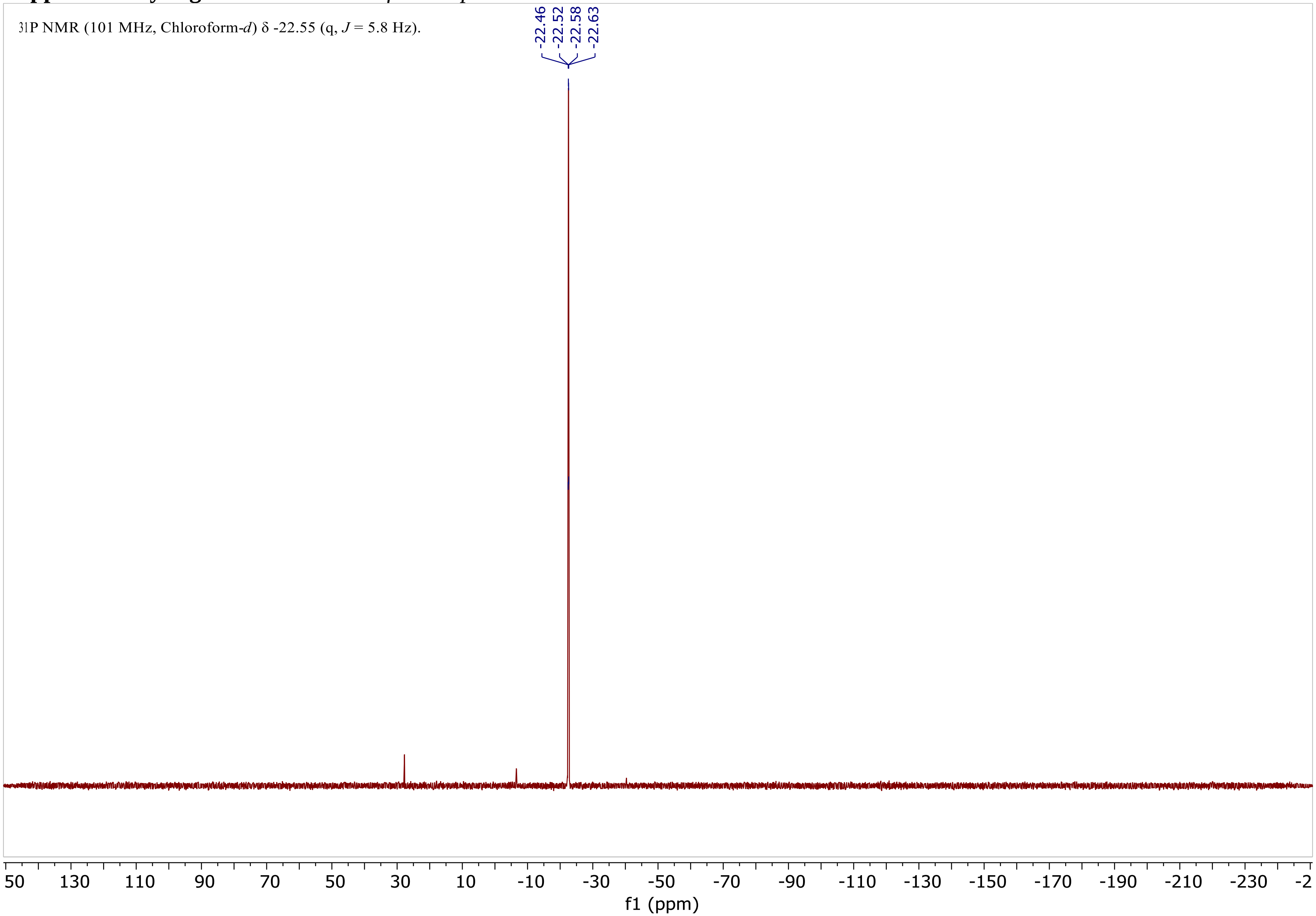

Supplementary Figure 439: *^{13}C NMR for compound **93***

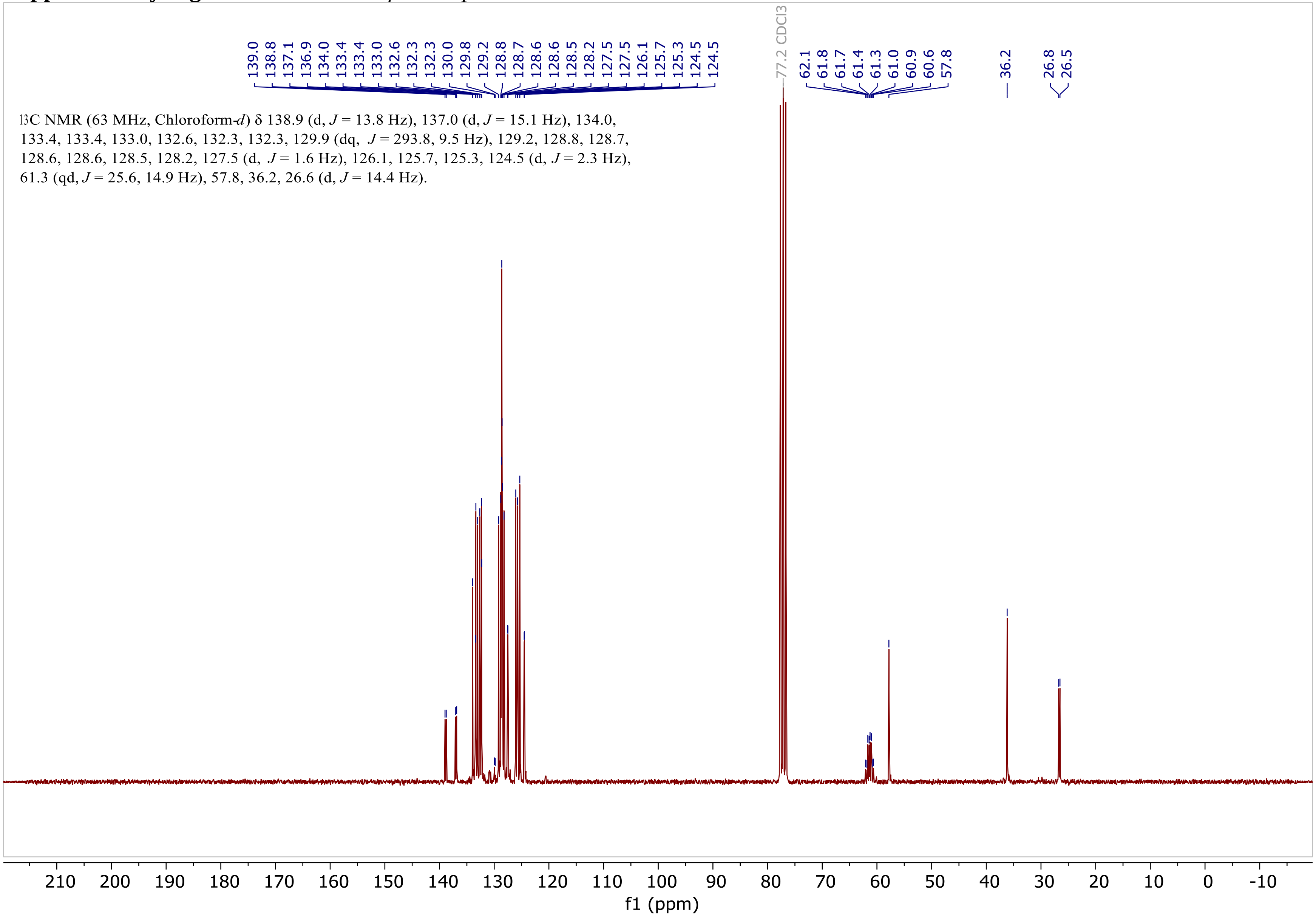

Supplementary Figure 440: *IR for compound* ***93***

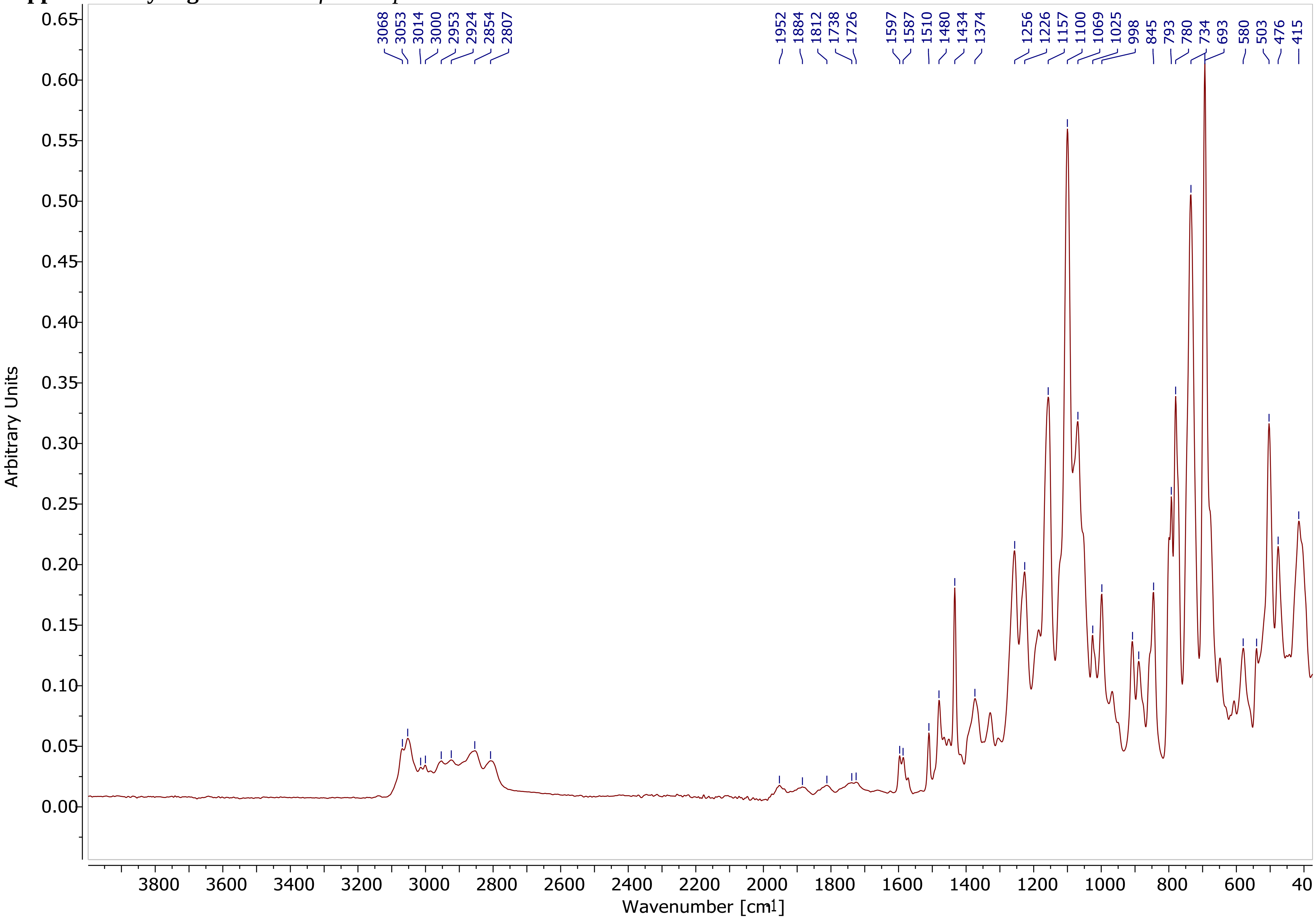

Supplementary Figure 441: *^{1}H-NMR for compound* ***94***

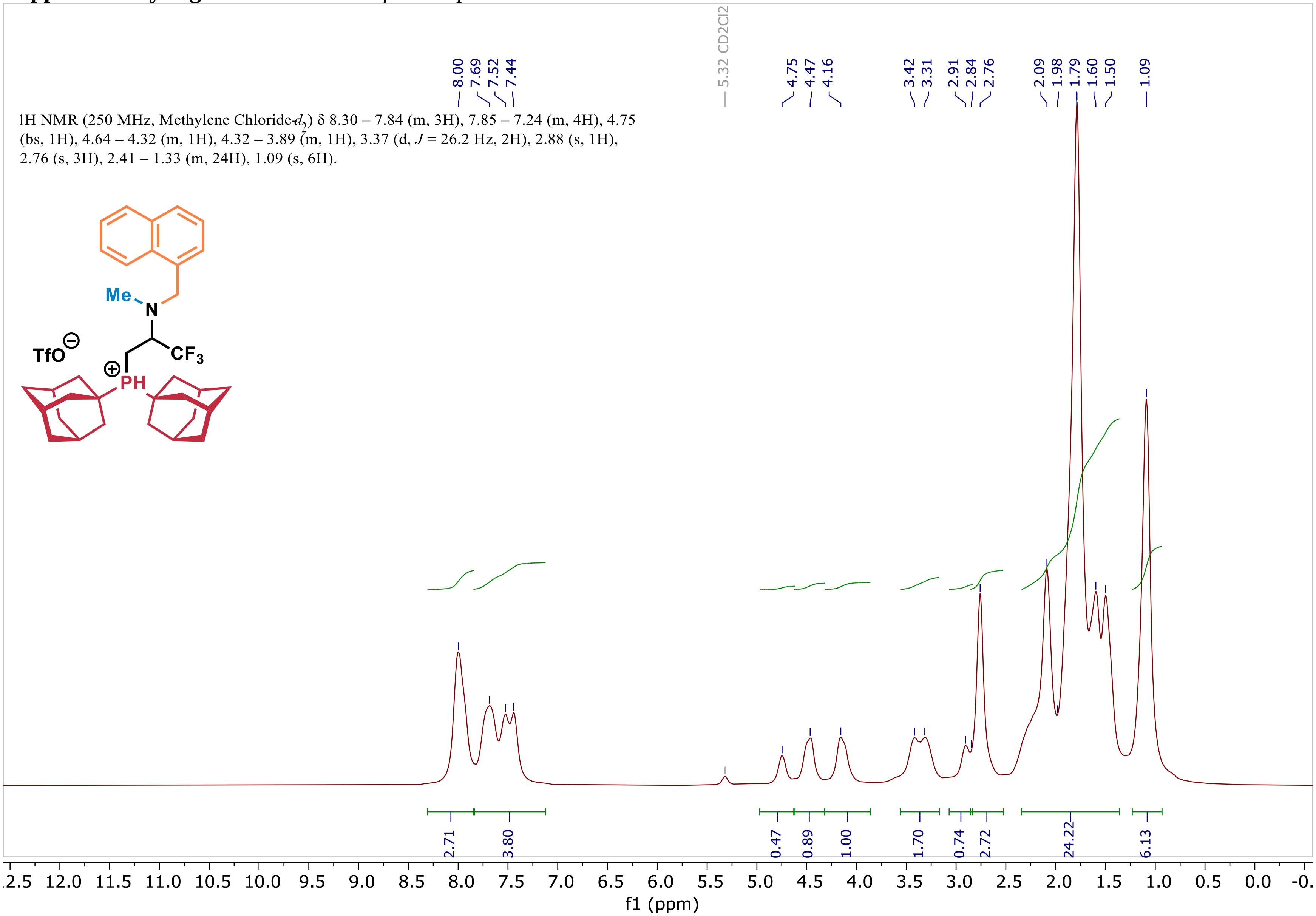

Supplementary Figure 442: *^{19}F NMR for compound* ***94***

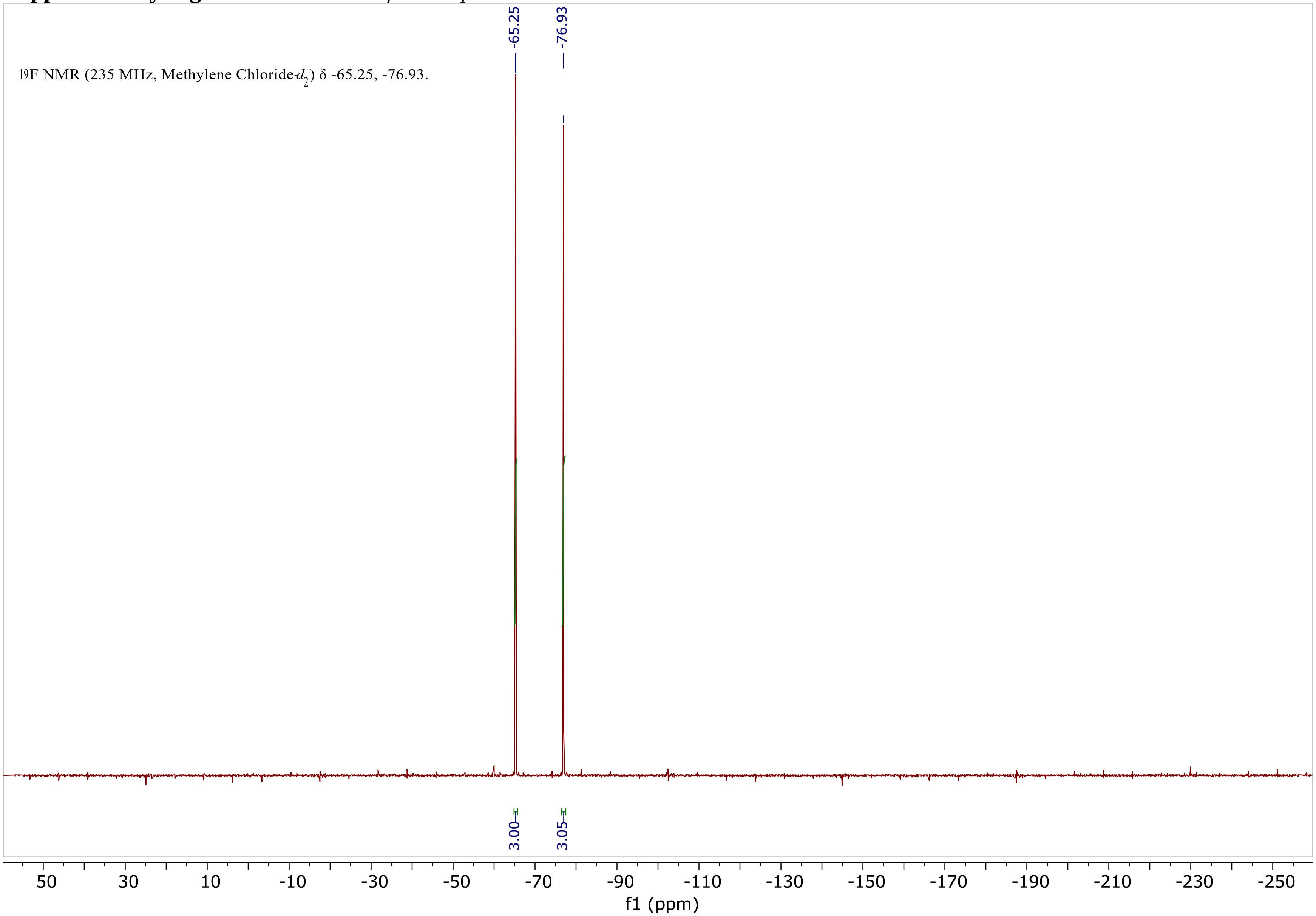

Supplementary Figure 443: *^{31}P NMR for compound **94***

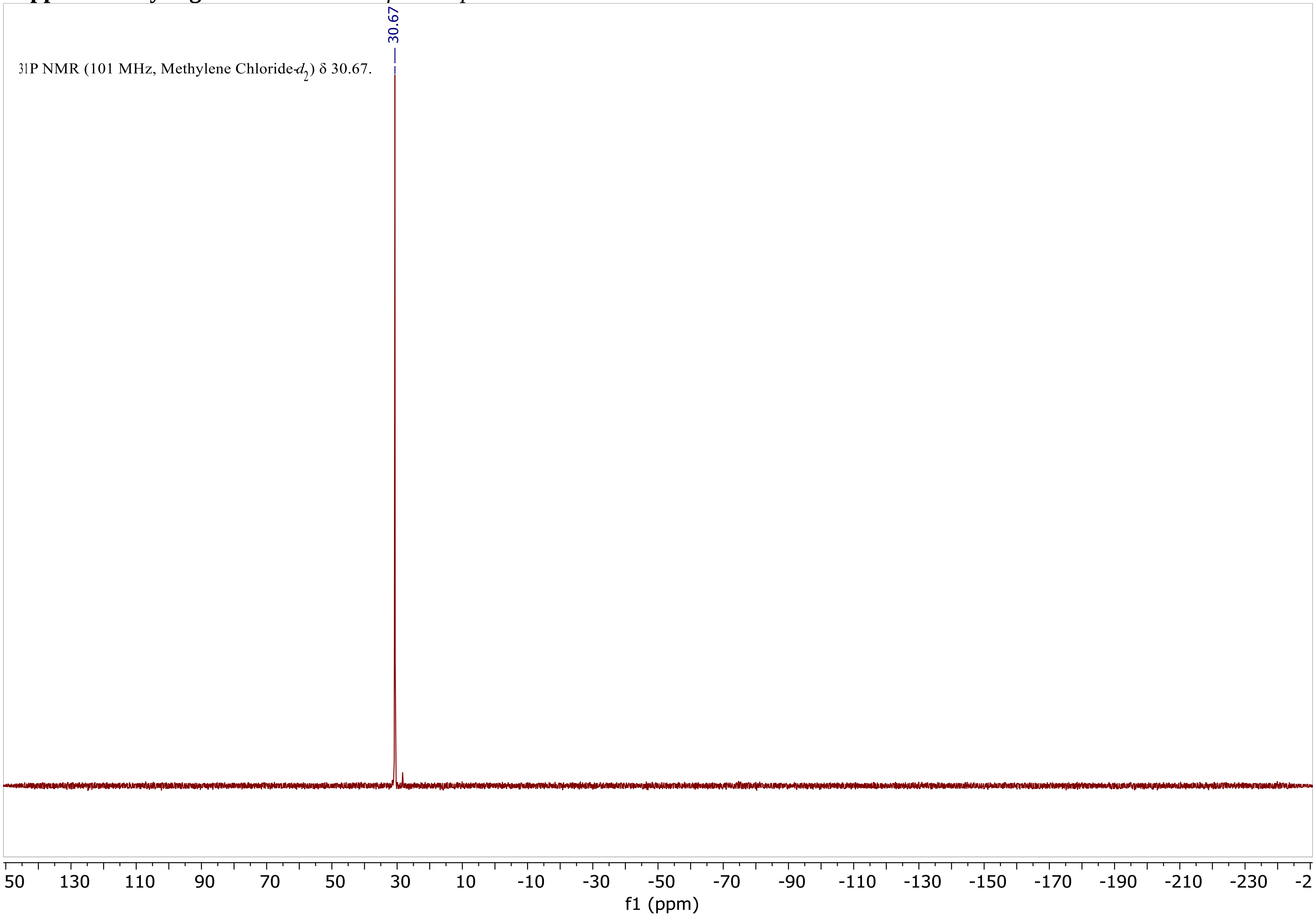

Supplementary Figure 444: *^{13}C NMR for compound* ***94***

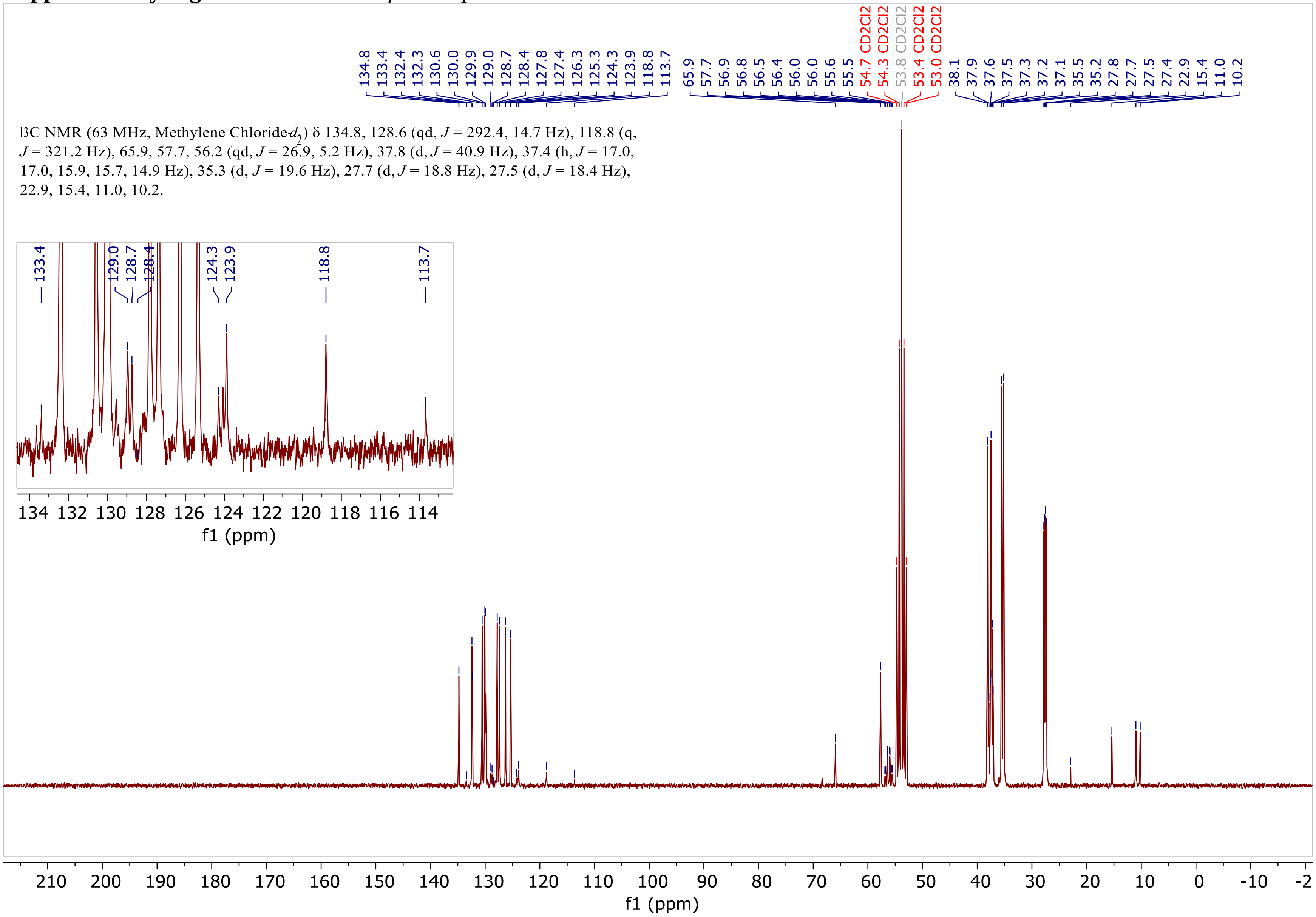

Supplementary Figure 445: *IR for compound* ***94***

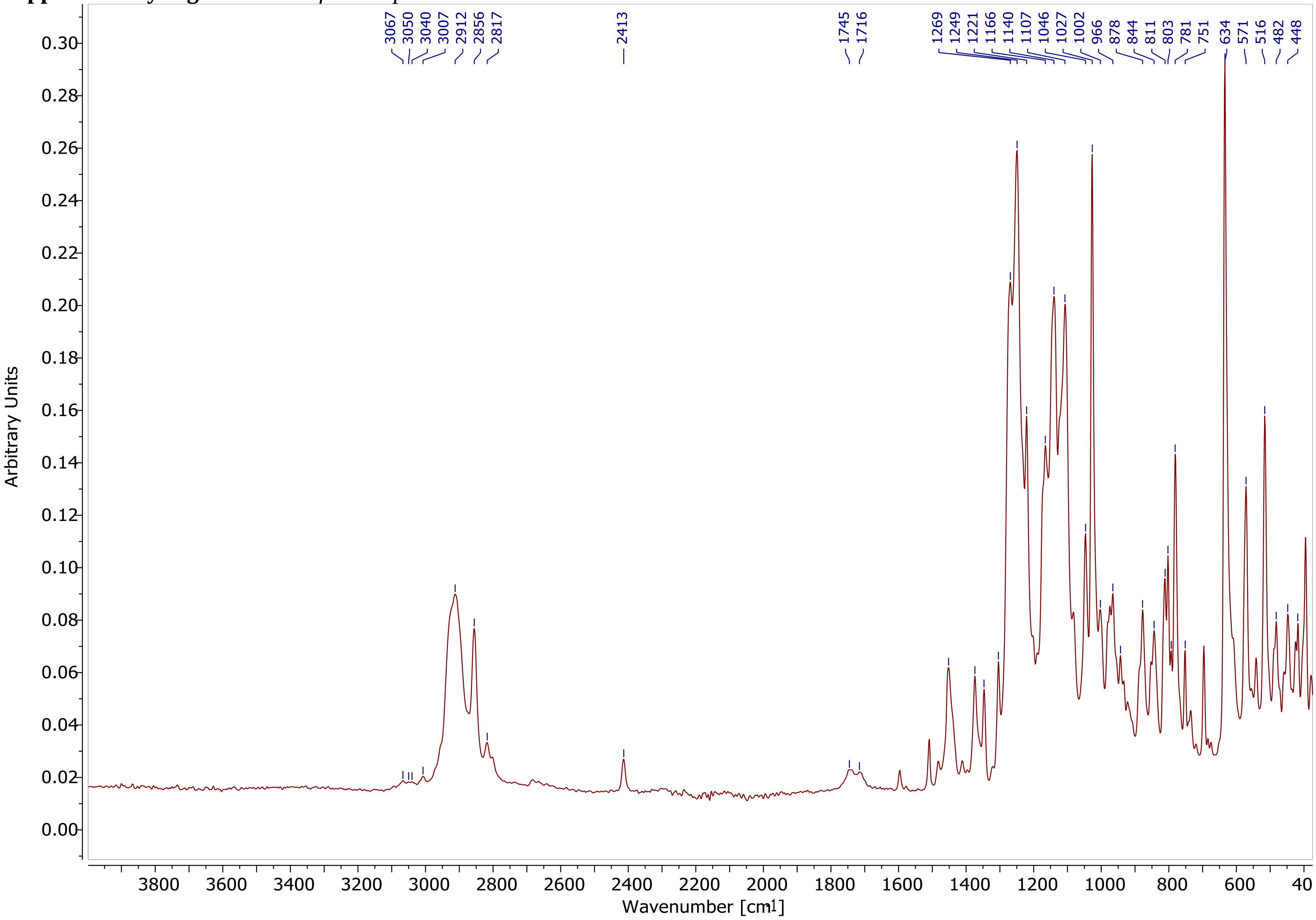

Supplementary Figure 446: *^{1}H-NMR for compound* ***95***

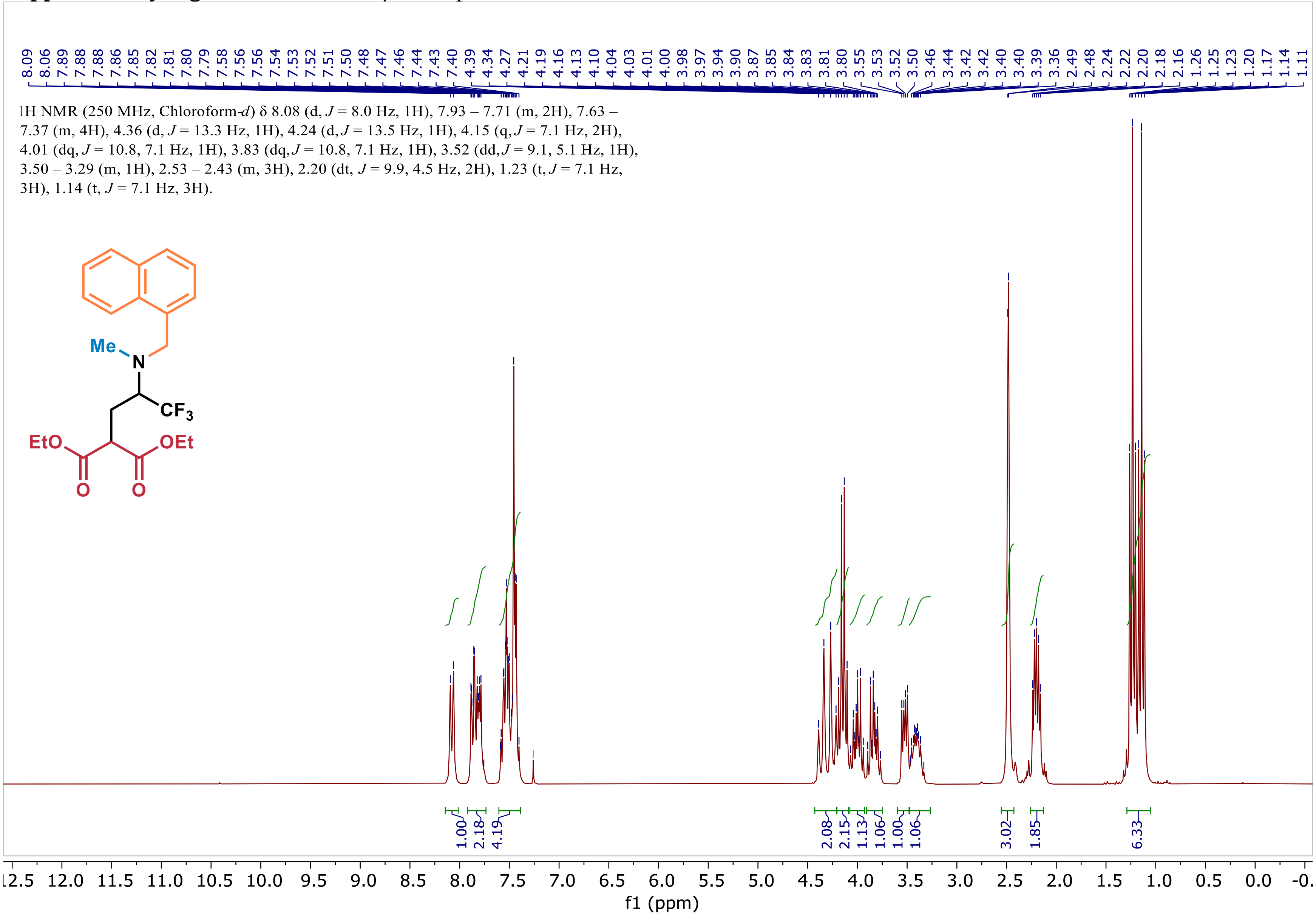

Supplementary Figure 447: *^{19}F NMR for compound* ***95***

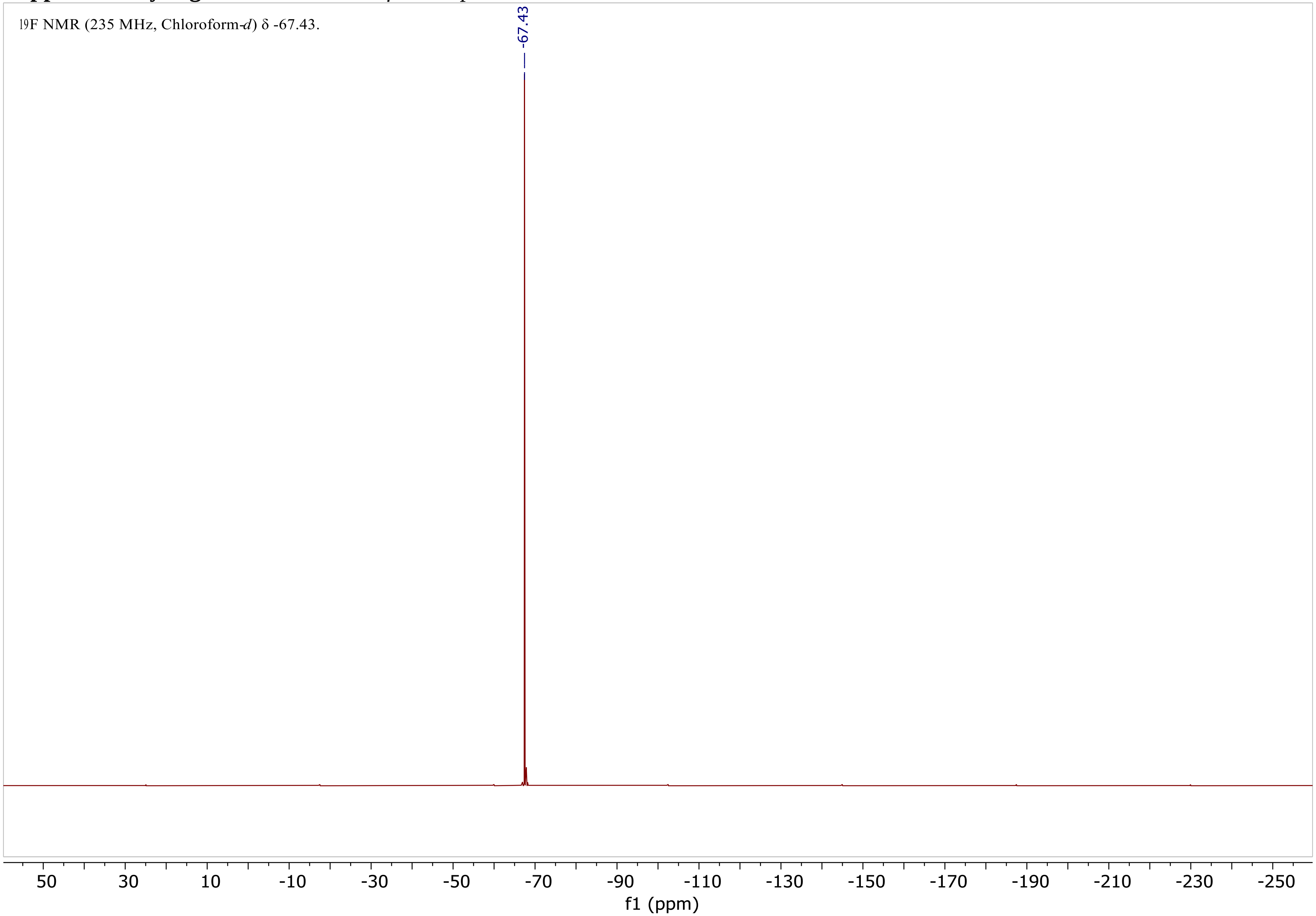

Supplementary Figure 448: ^{13}*C NMR for compound* ***95***

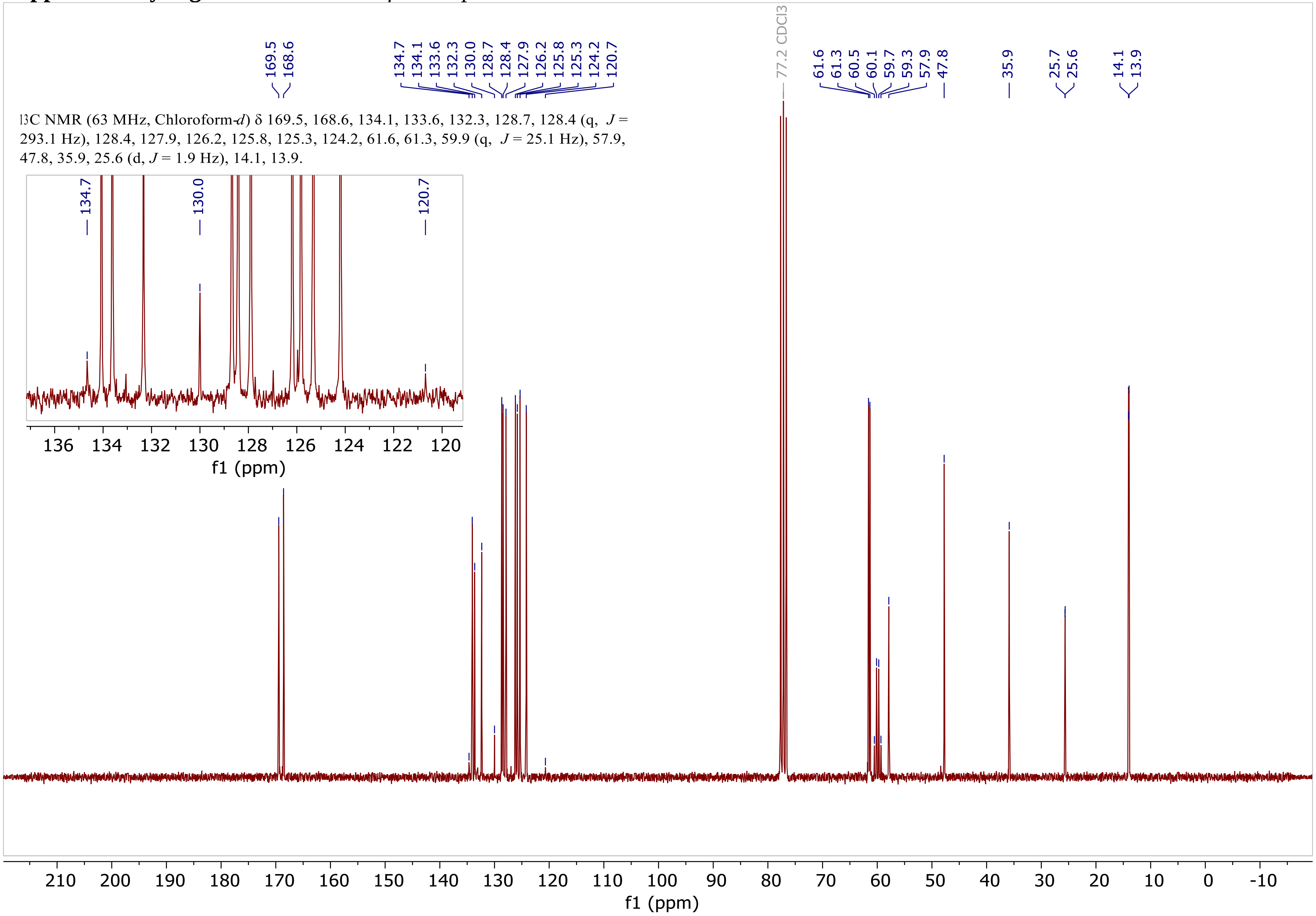

Supplementary Figure 449: *IR for compound* ***95***

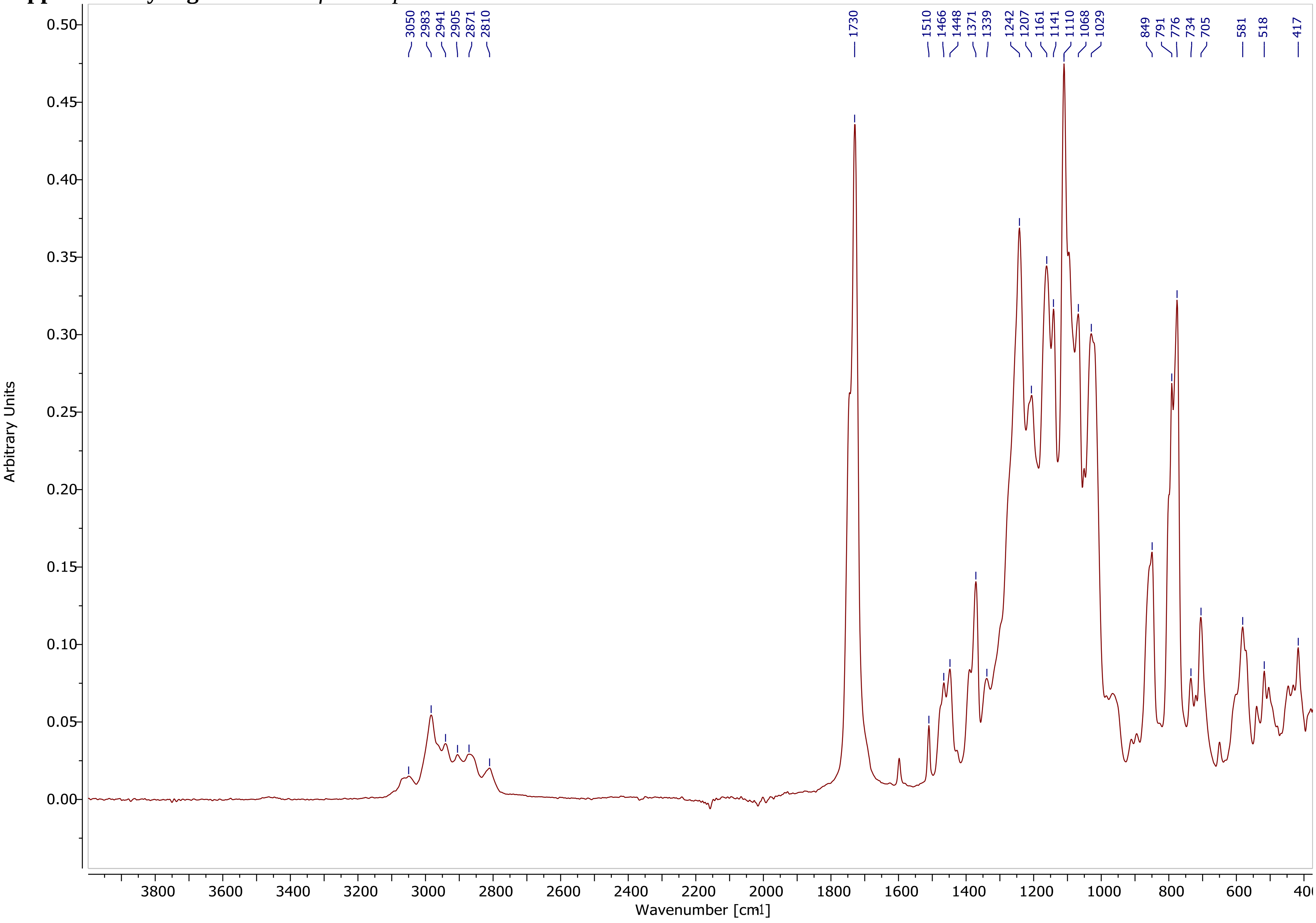

Supplementary Figure 450: *^{1}H-NMR for compound **96***

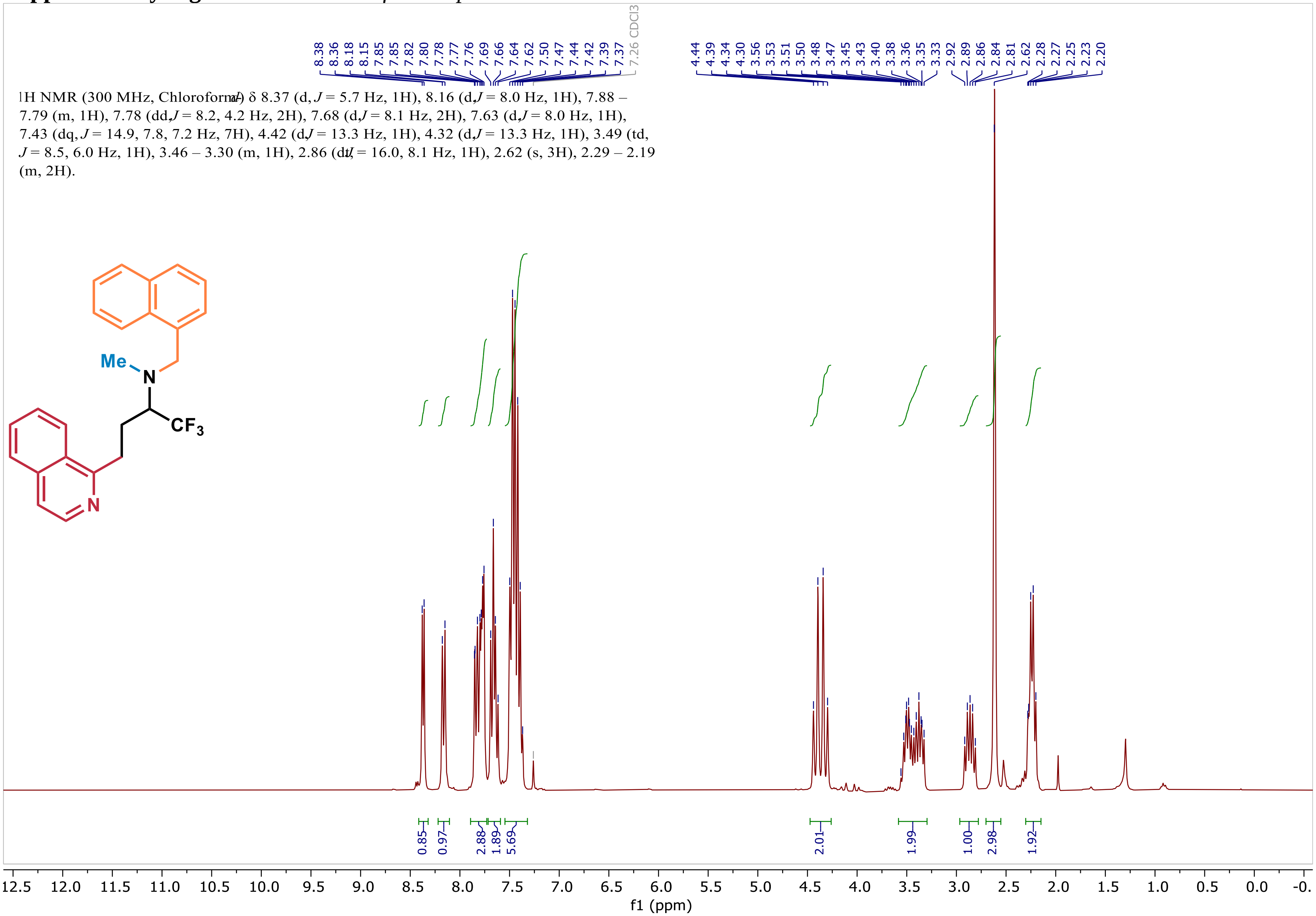

Supplementary Figure 451: *COSY NMR for compound* ***96***

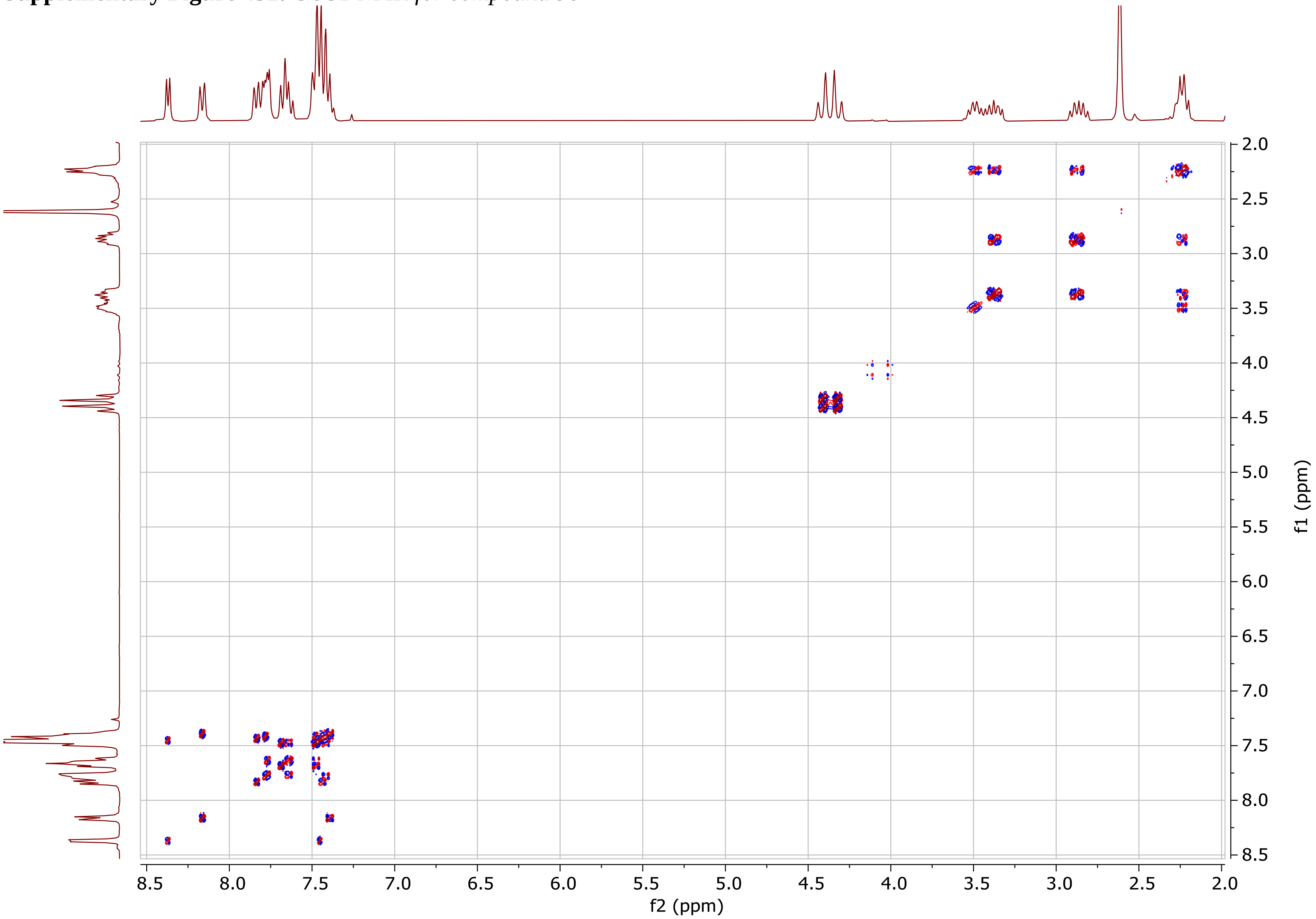

Supplementary Figure 452: *^{19}F NMR for compound* ***96***

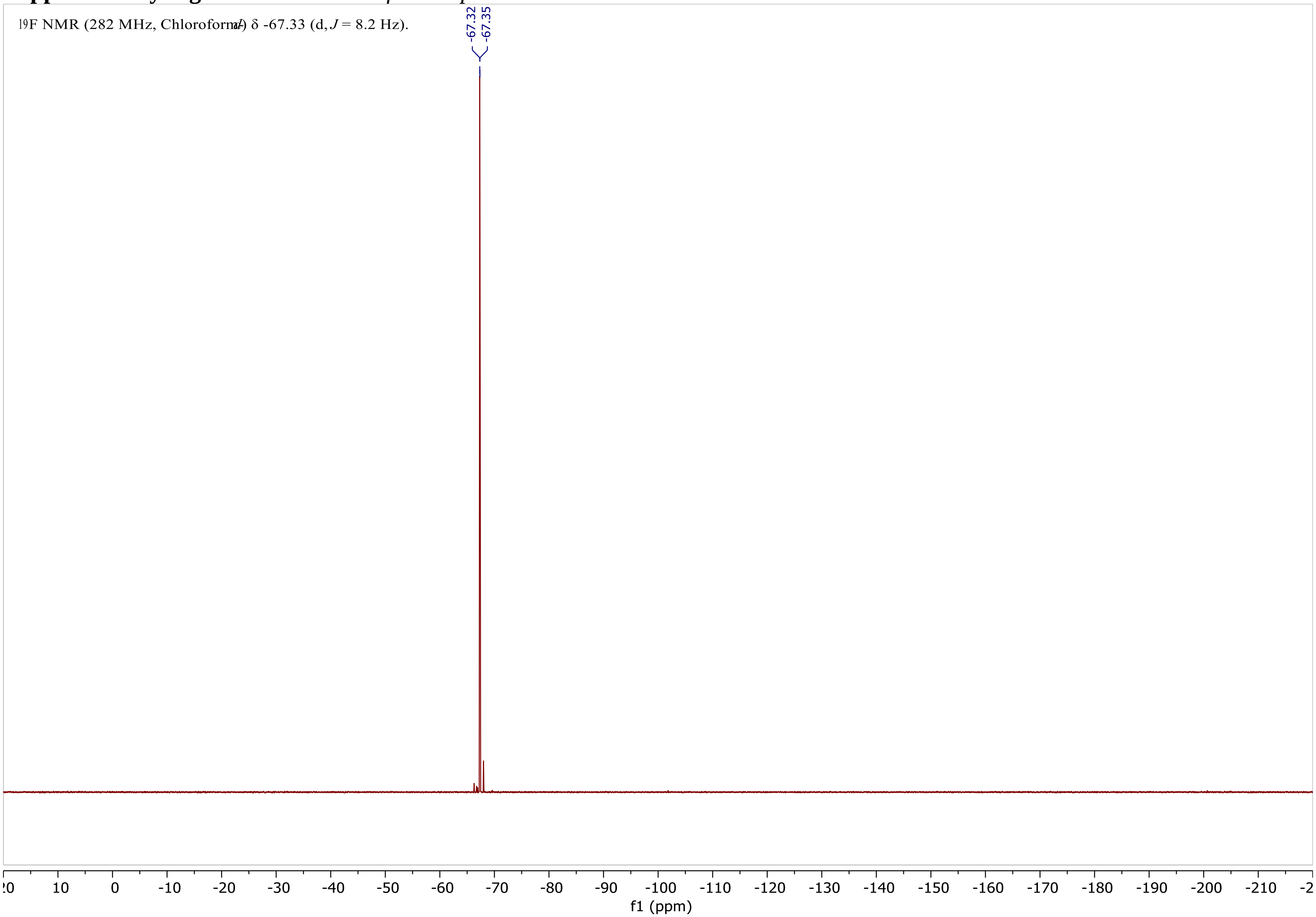

Supplementary Figure 453: *^{13}C NMR for compound* ***96***

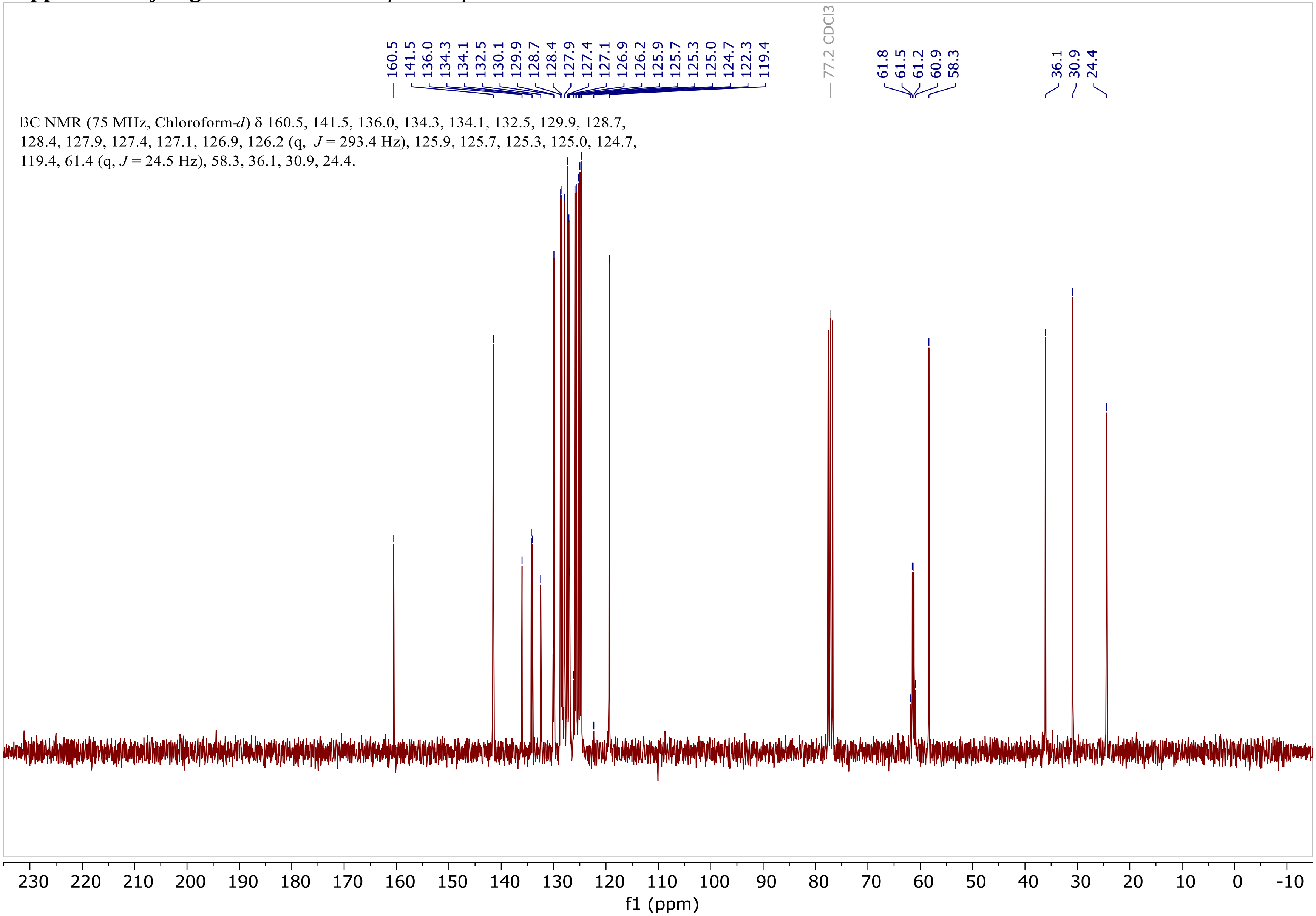

Supplementary Figure 454: *HSQC NMR for compound **96***

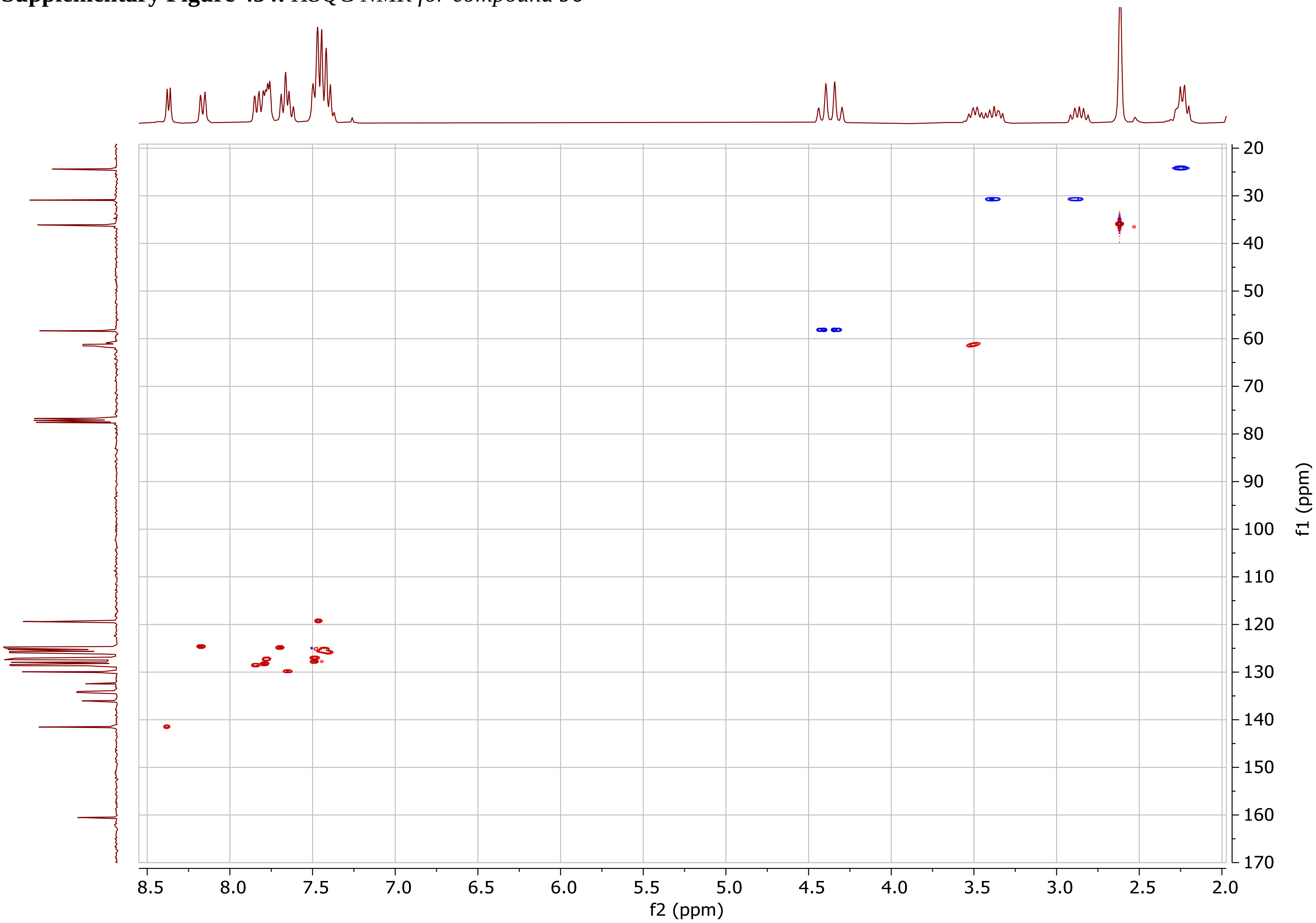

Supplementary Figure 455: *HMBC NMR for compound **96***

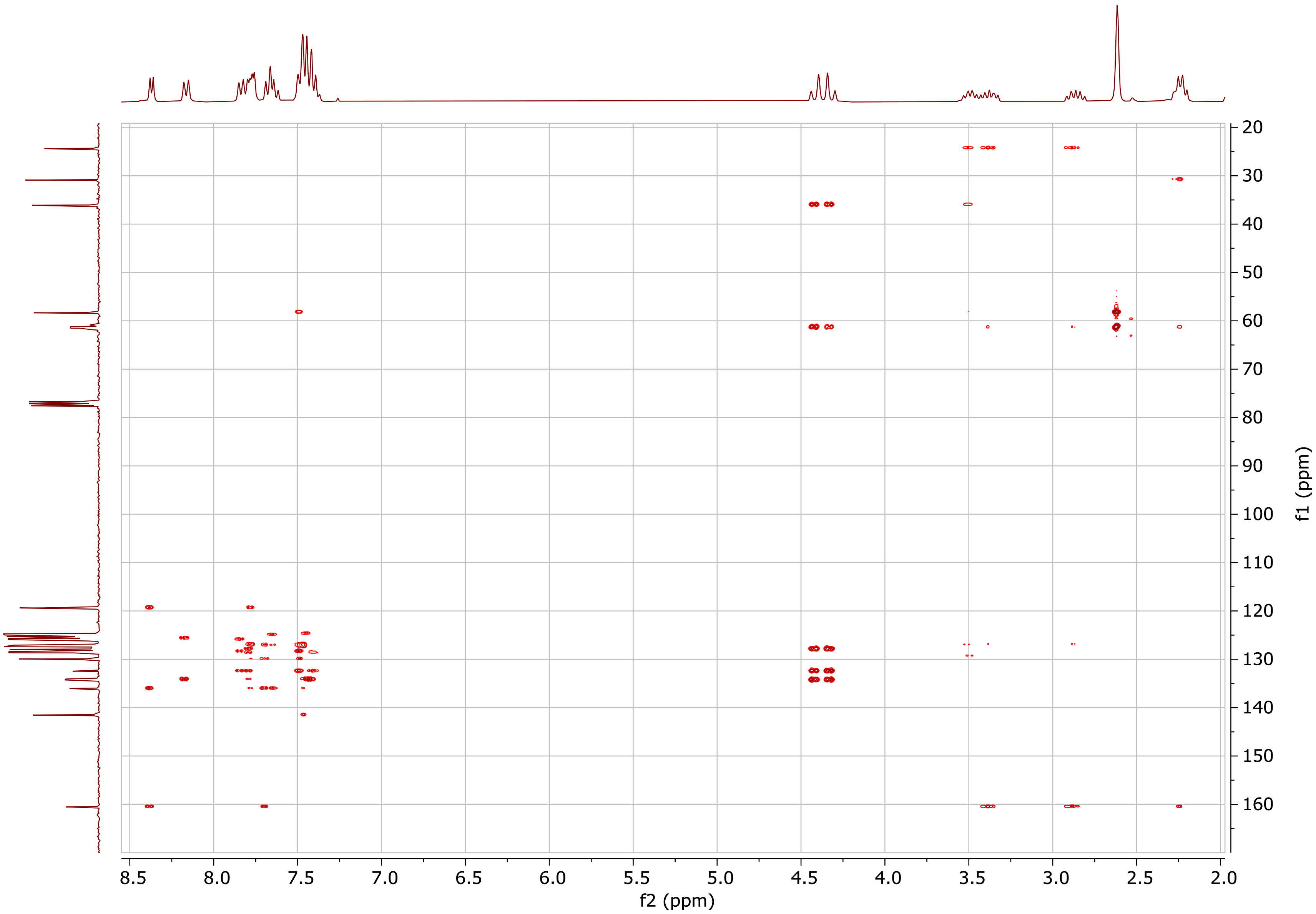

Supplementary Figure 456: *IR for compound* ***96***

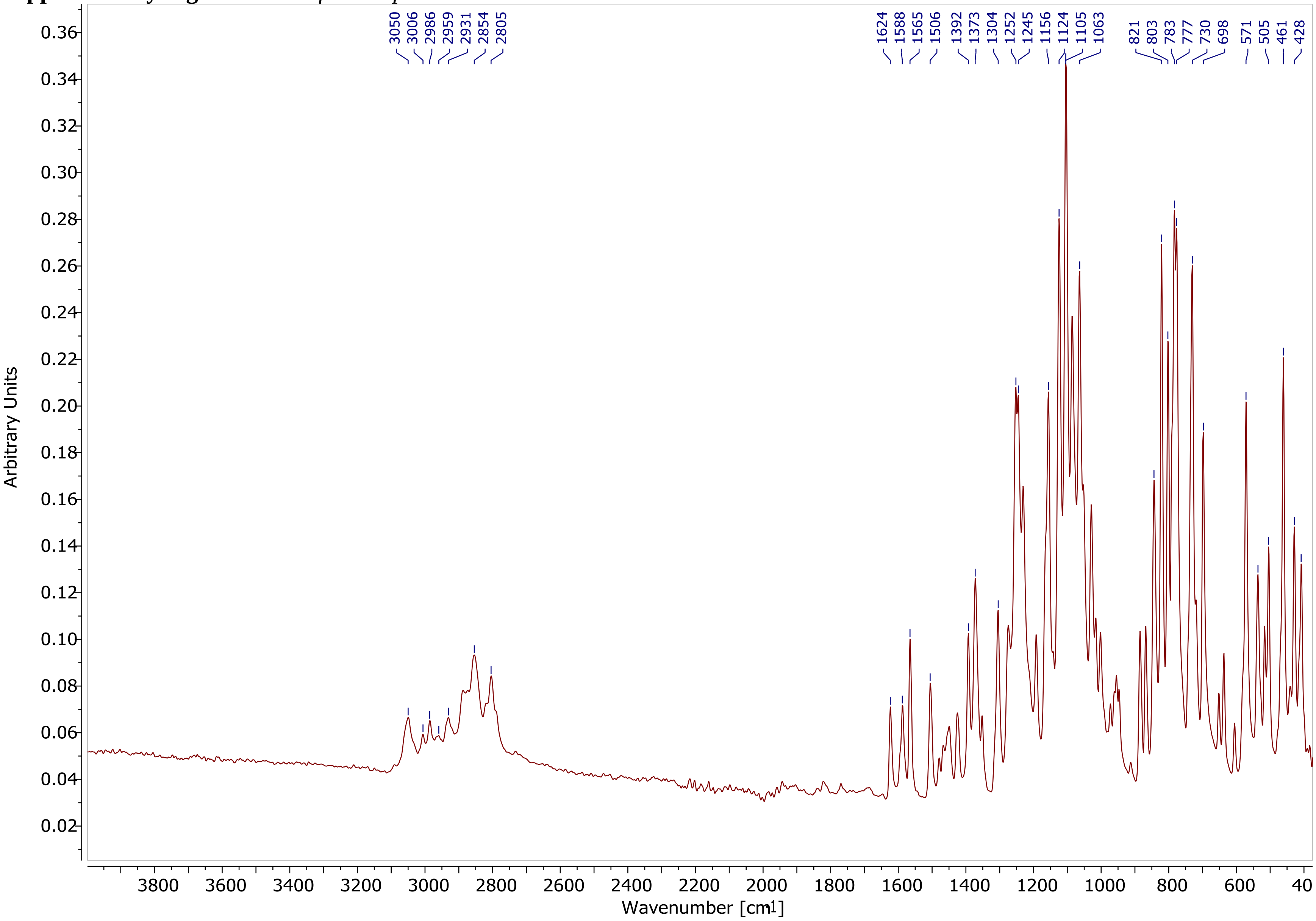

Supplementary Figure 457: *^{1}H-NMR for compound* ***97***

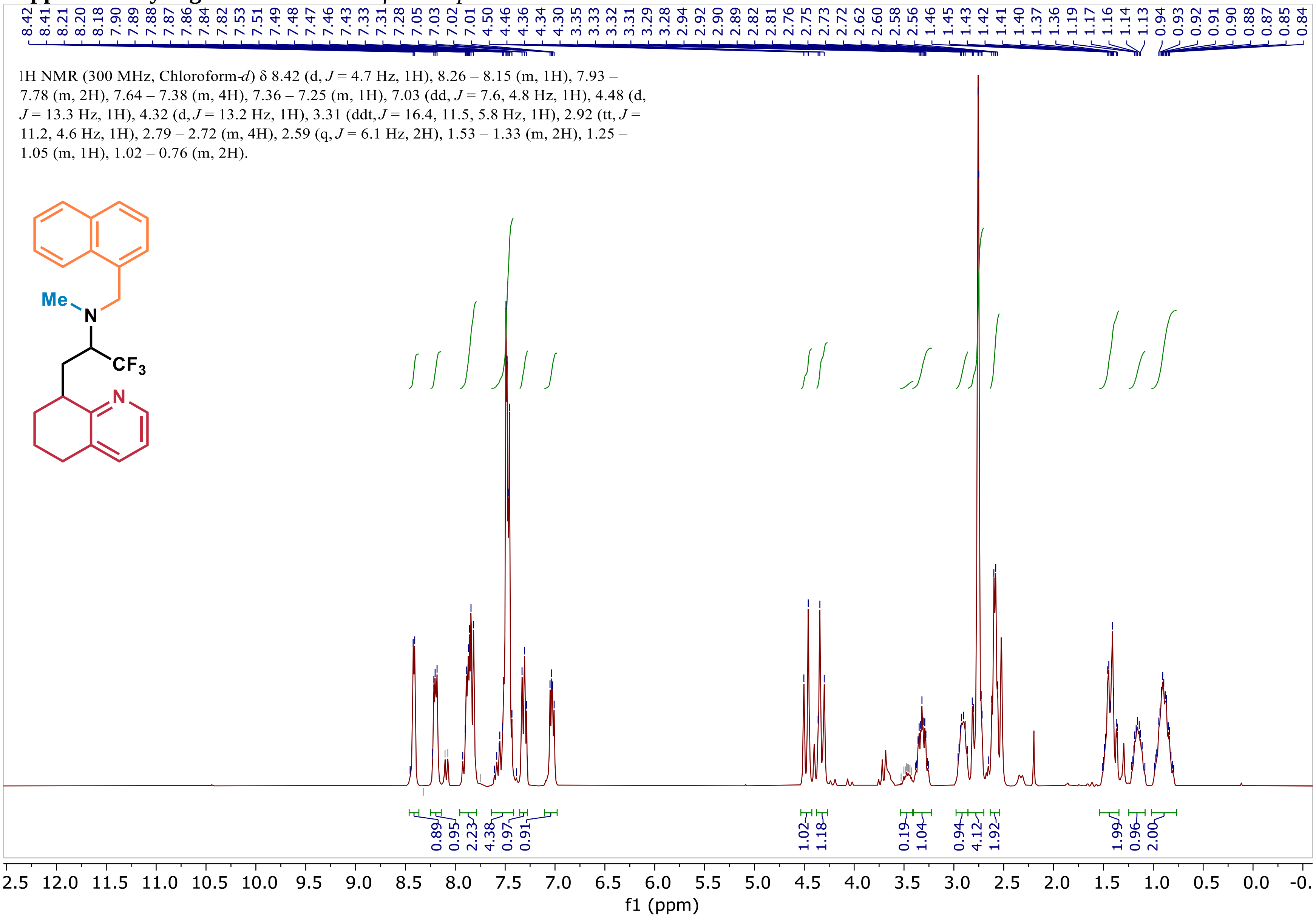

Supplementary Figure 458: *COSY NMR for compound **97***

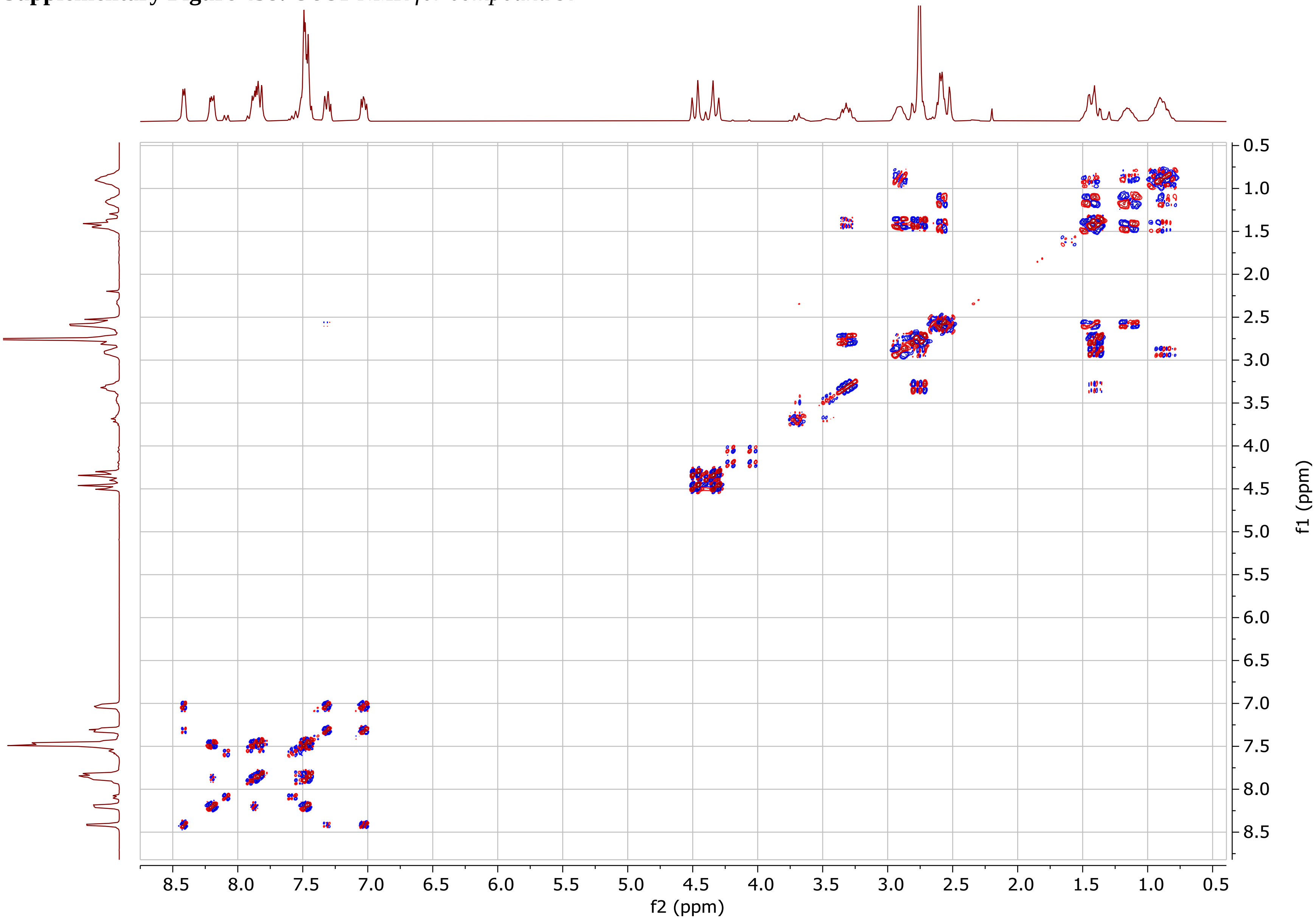

Supplementary Figure 459: *TOCSY NMR for compound* ***97***

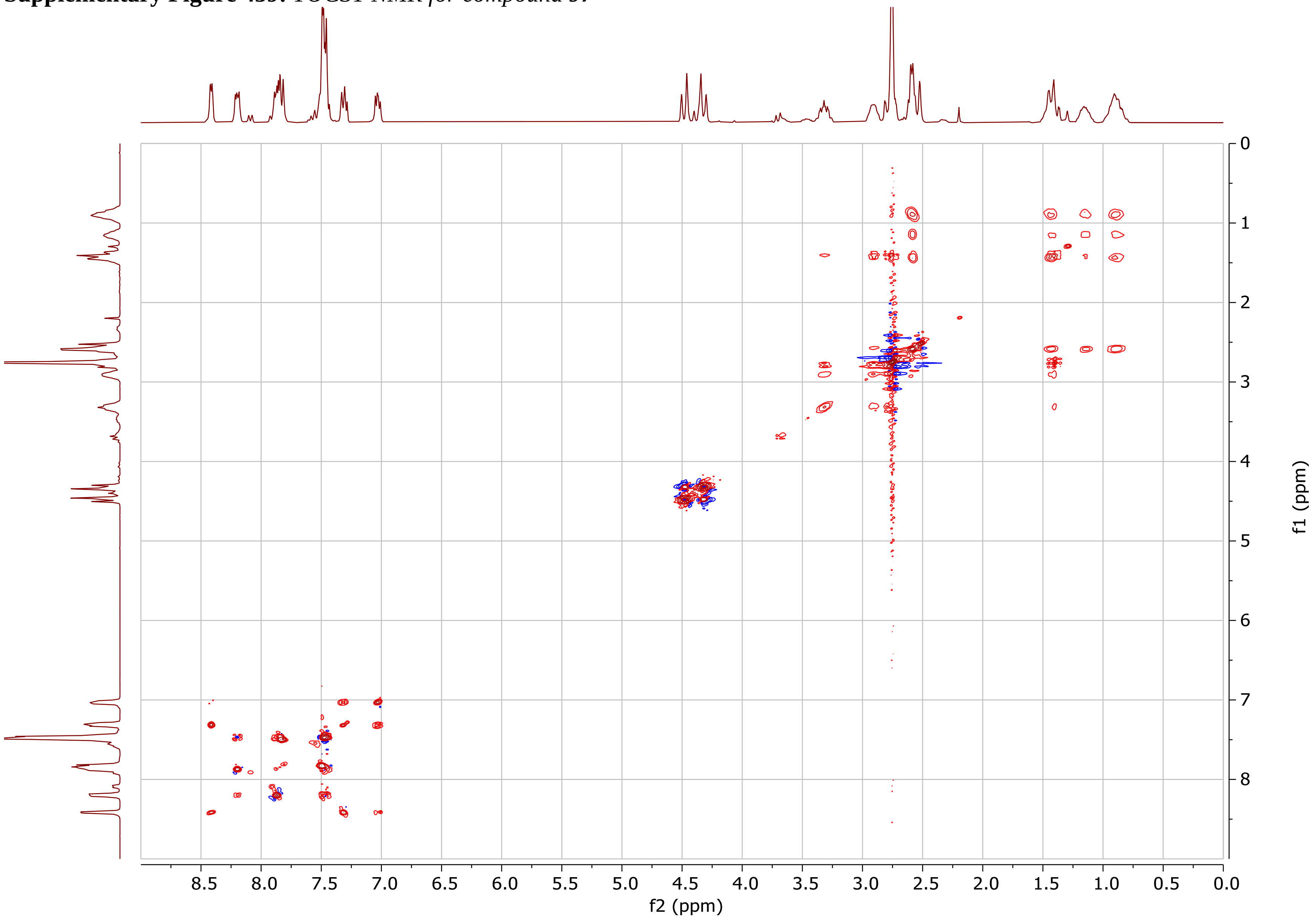

Supplementary Figure 460: *^{19}F NMR for compound* ***97***

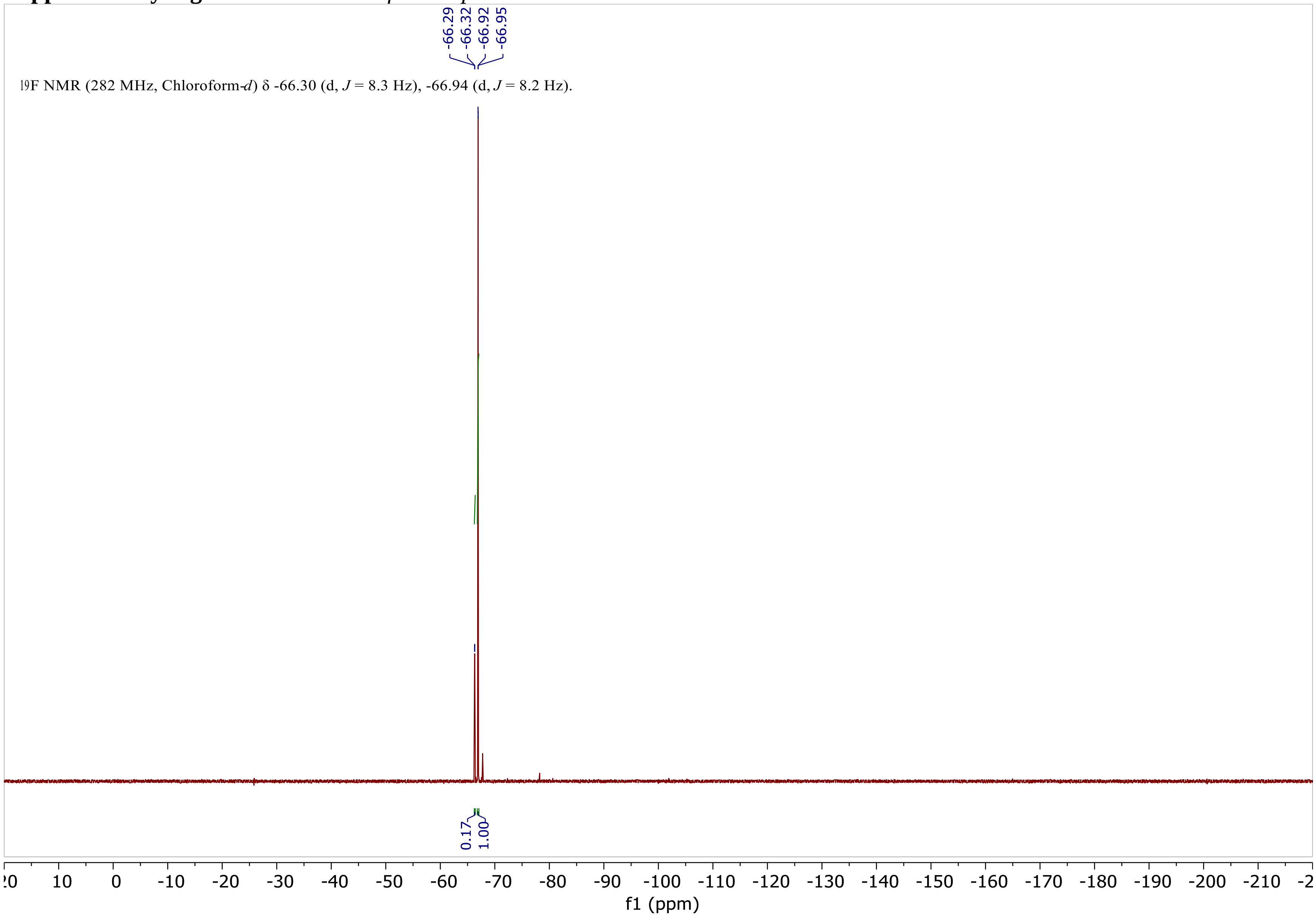

Supplementary Figure 461: *^{13}C NMR for compound **97***

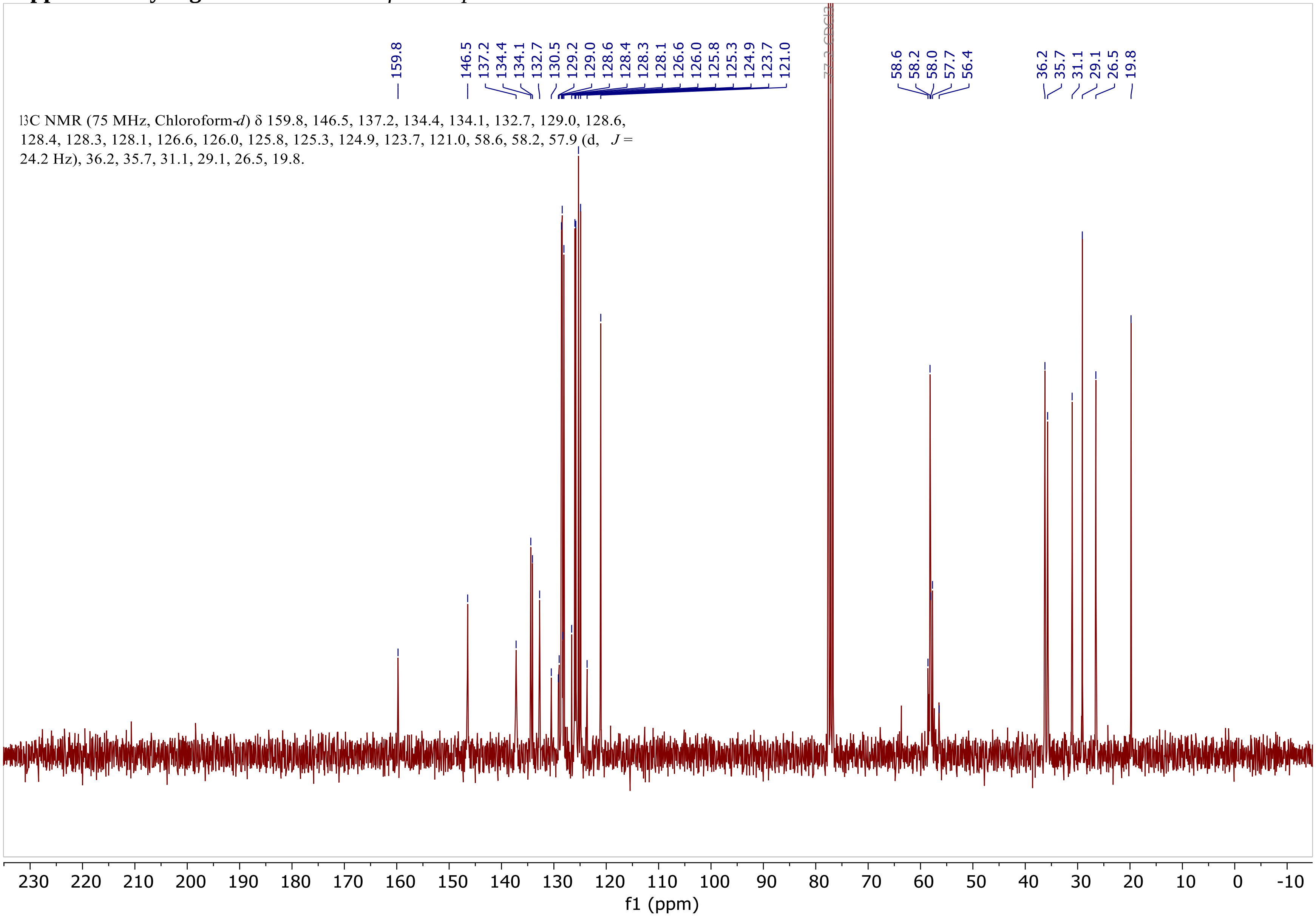

Supplementary Figure 462: *HSQC NMR for compound* ***97***

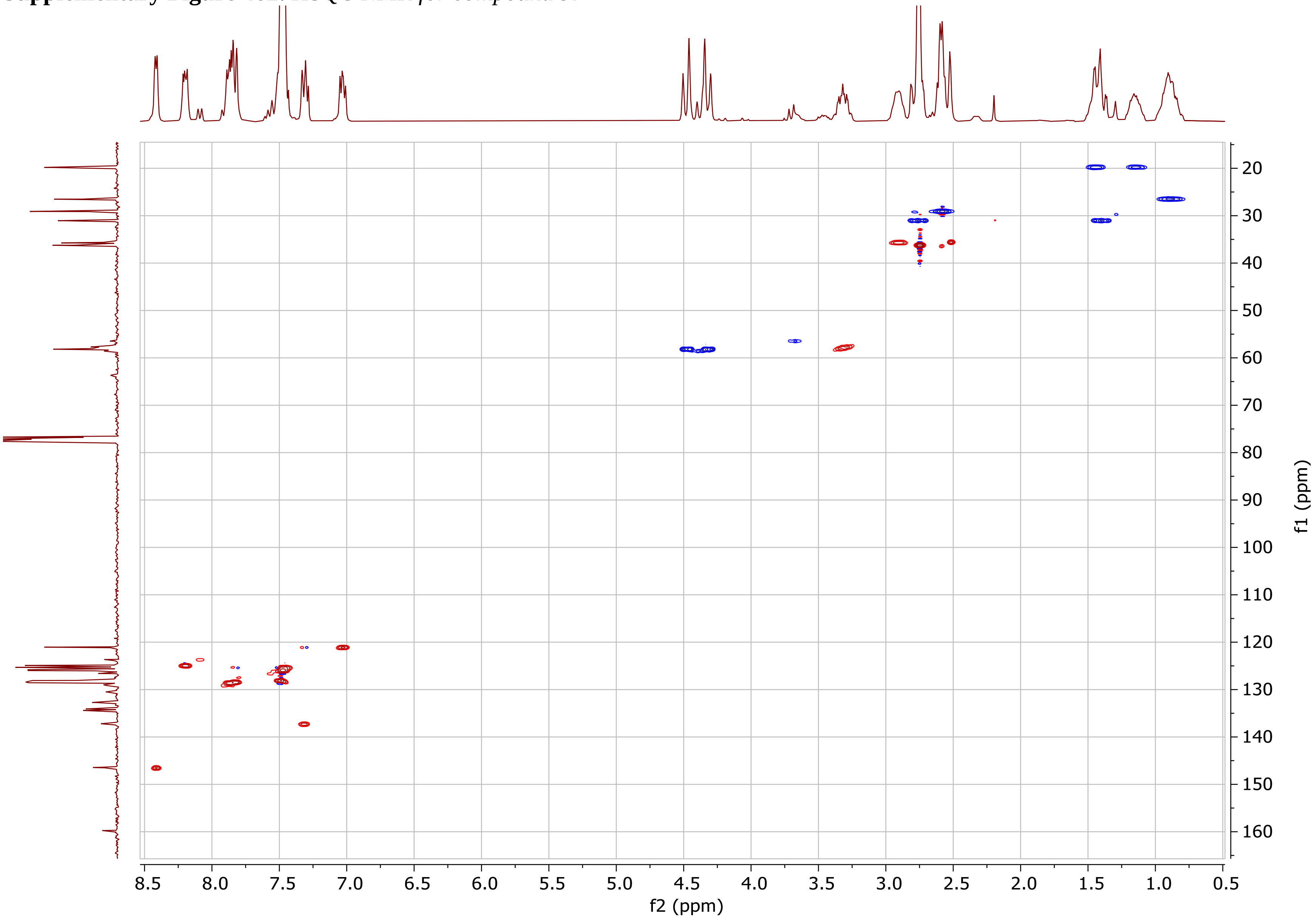

Supplementary Figure 463: *HMBC NMR for compound **97***

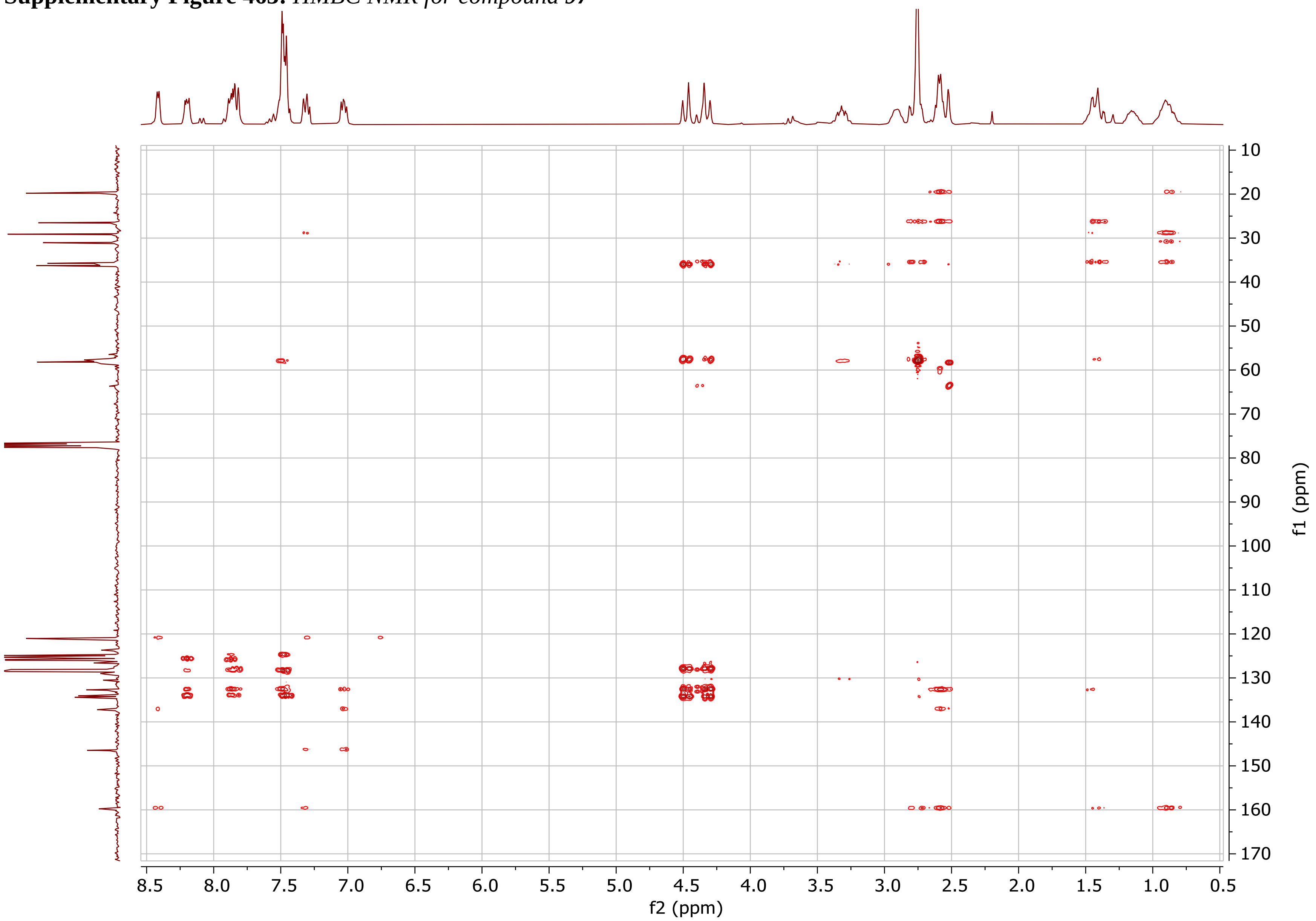

Supplementary Figure 464: *IR for compound **97***

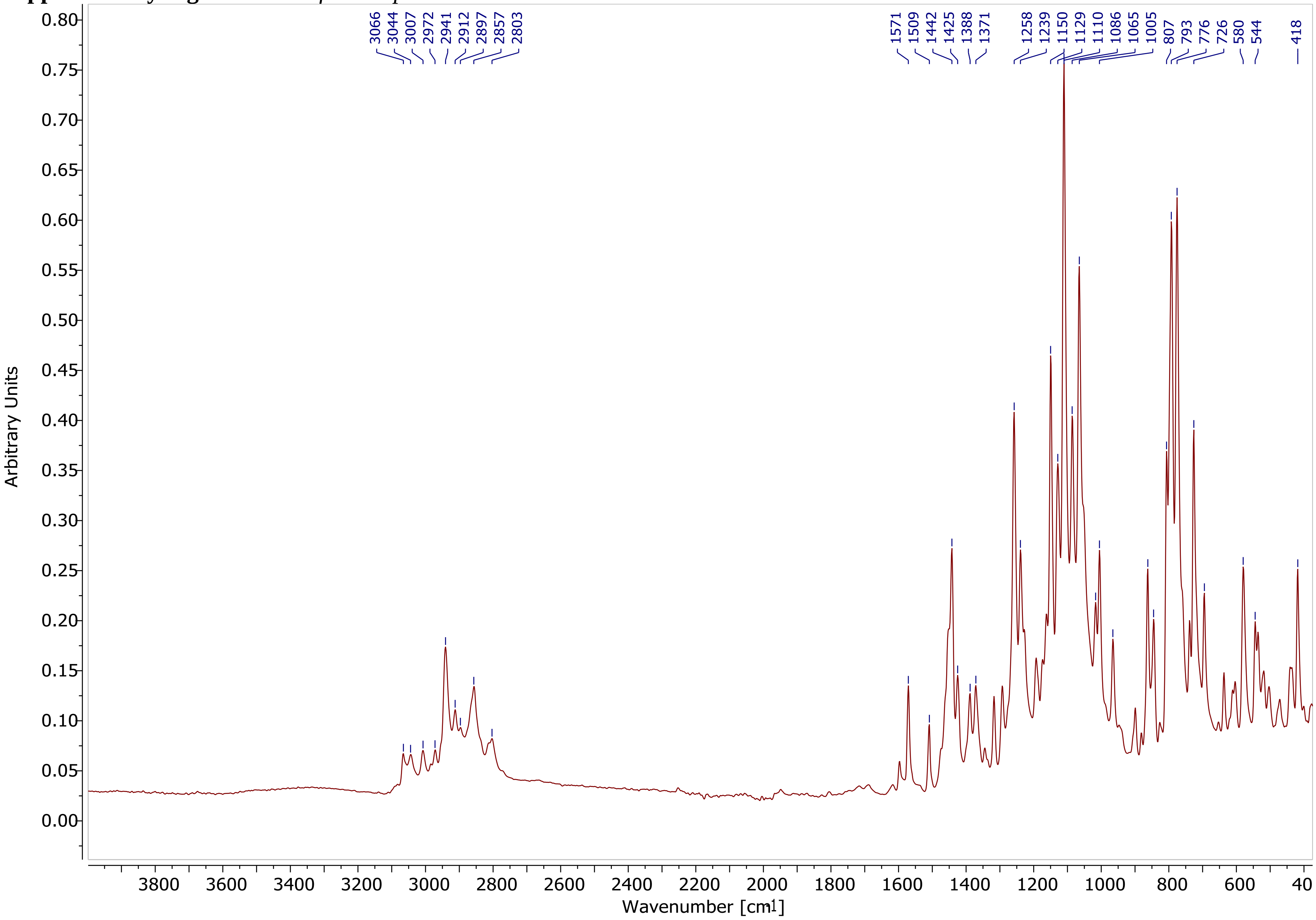

Supplementary Figure 465: *^{1}H-NMR for compound* ***98***

1H NMR (250 MHz, Chloroform-*d*) δ 7.78 (d, *J* = 8.2 Hz, 1H), 7.70 (d, *J* = 8.0 Hz, 1H), 7.67 – 7.58 (m, 1H), 7.48 – 7.17 (m, 6H), 7.11 (d, *J* = 7.0 Hz, 1H), 7.05 (d, *J* = 7.4 Hz, 1H), 6.74 (d, *J* = 2.4 Hz, 1H), 4.24 (d, *J* = 13.6 Hz, 1H), 4.16 (d, *J* = 13.4 Hz, 1H), 3.80 – 3.52 (m, 1H), 3.15 (s, 1H), 3.12 (s, 1H), 2.57 – 2.43 (m, 3H).

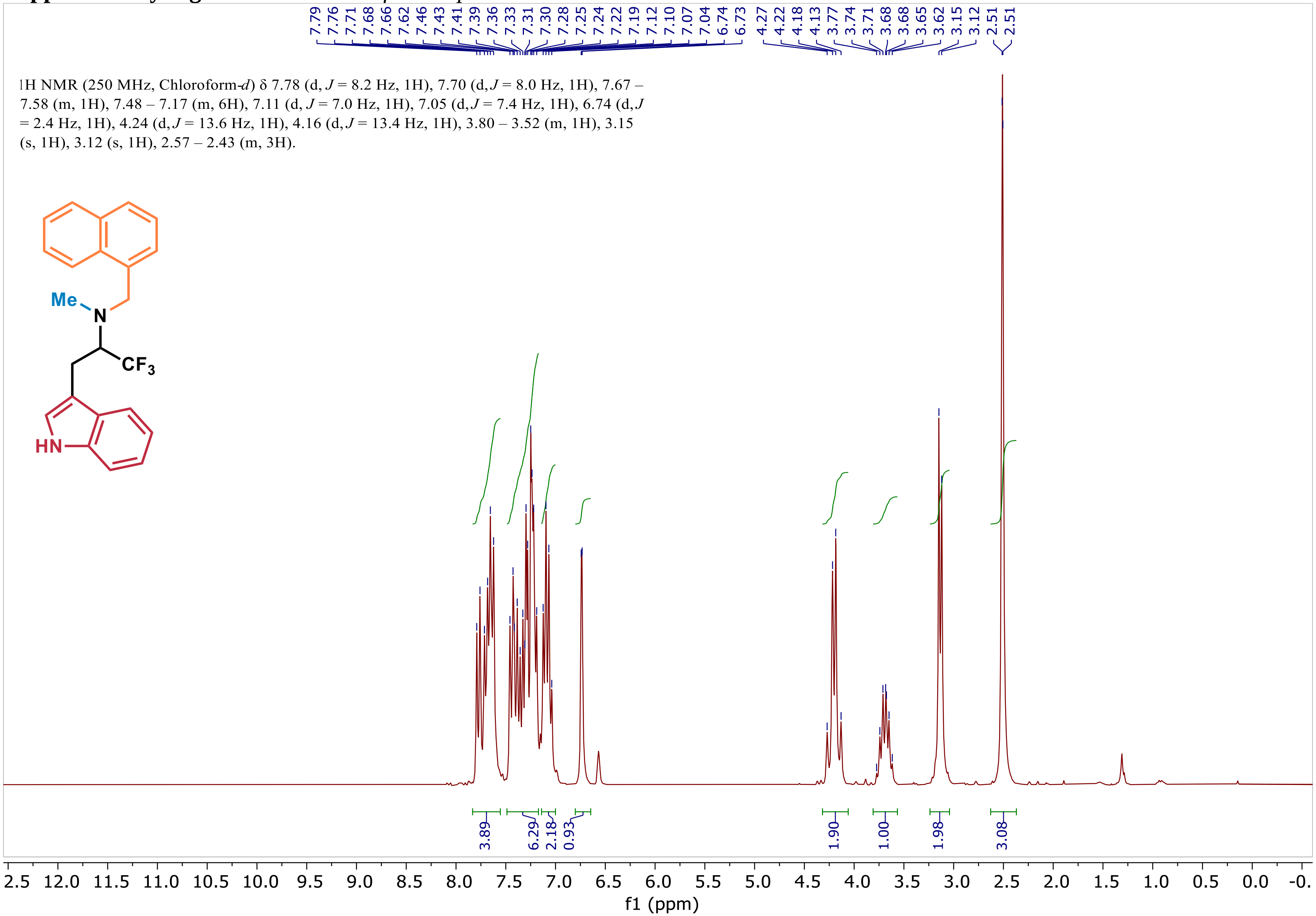

Supplementary Figure 466: *COSY NMR for compound **98***

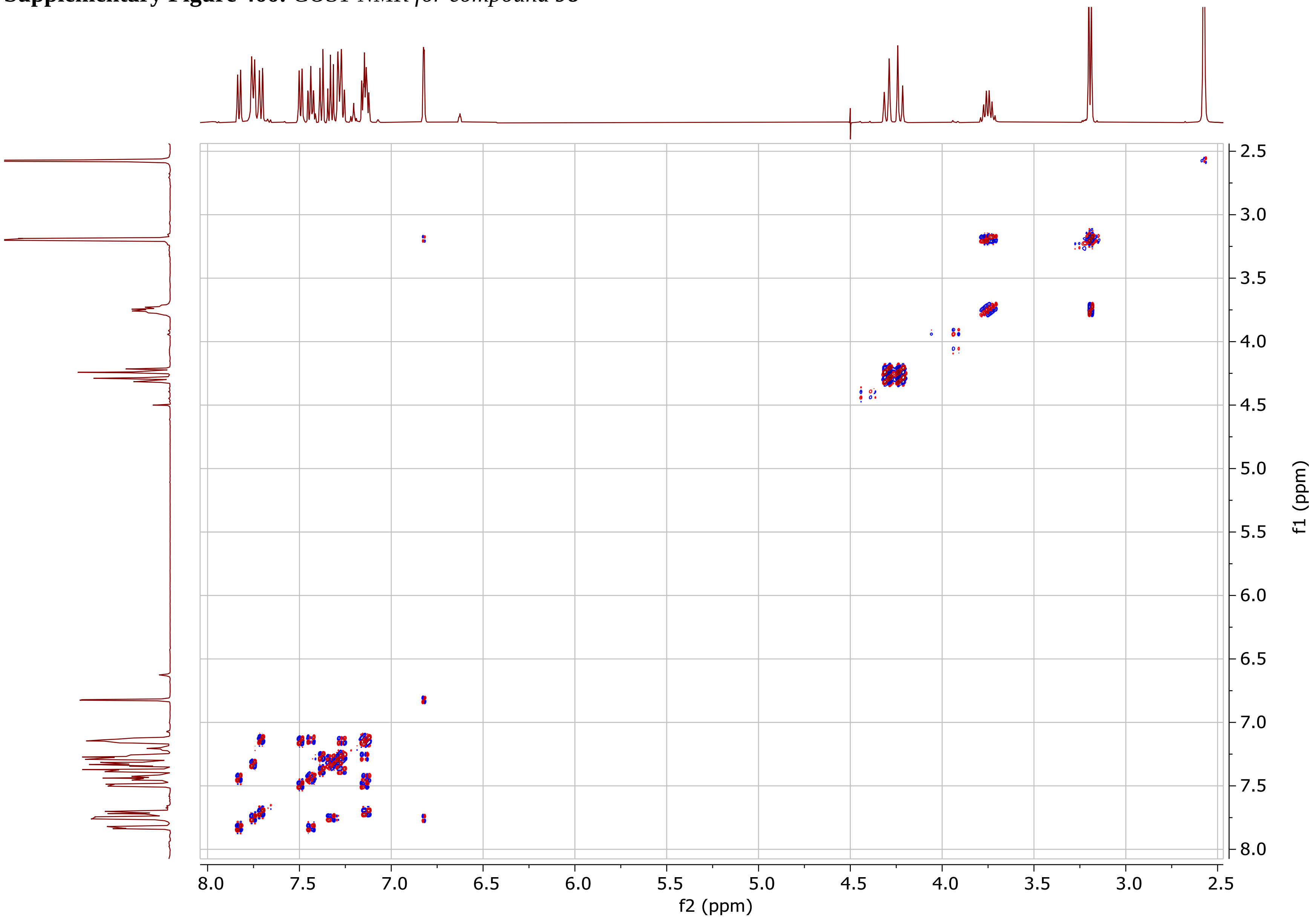

Supplementary Figure 467: *TOCSY NMR for compound* ***98***

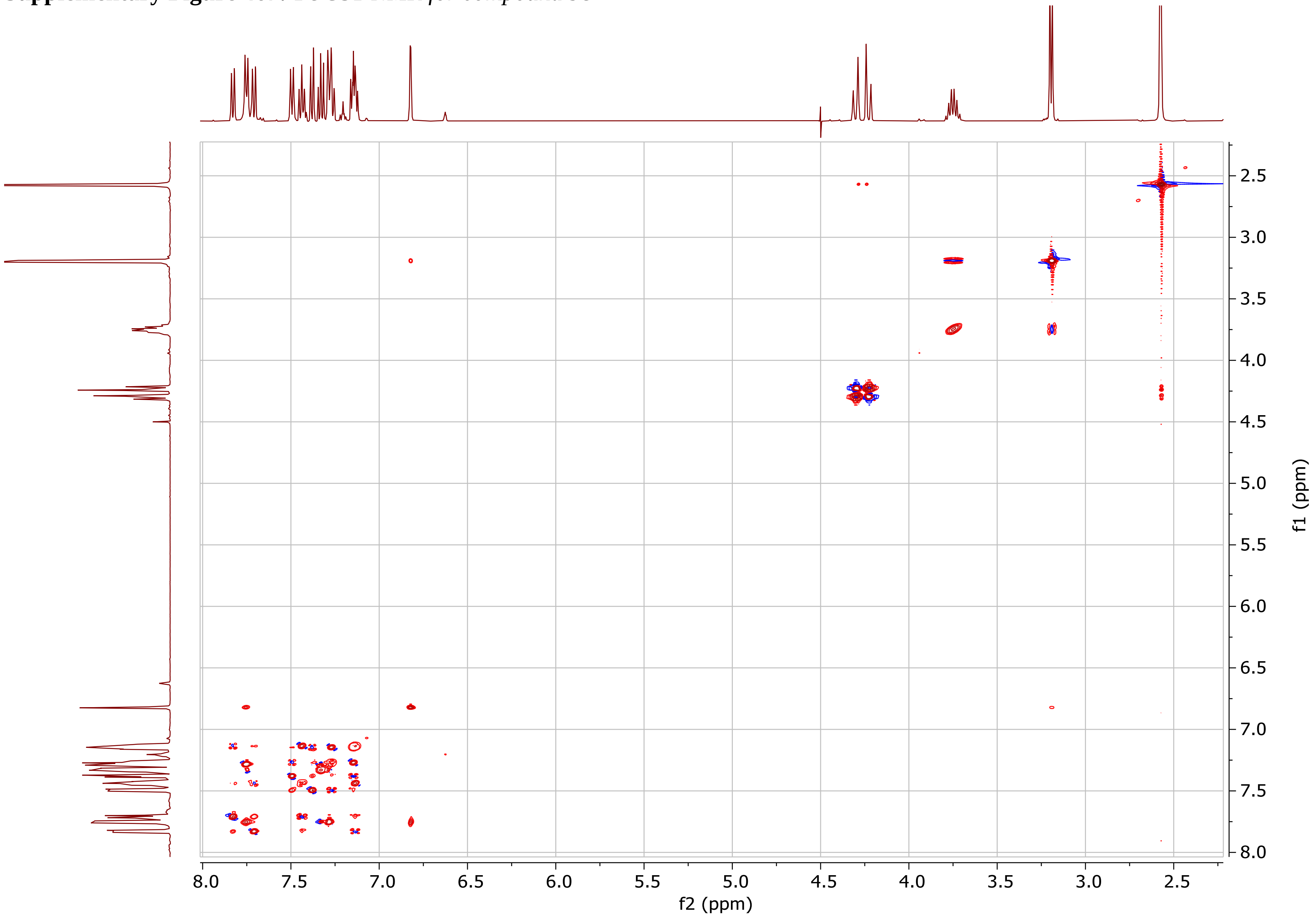

Supplementary Figure 468: *^{19}F NMR for compound* ***98***

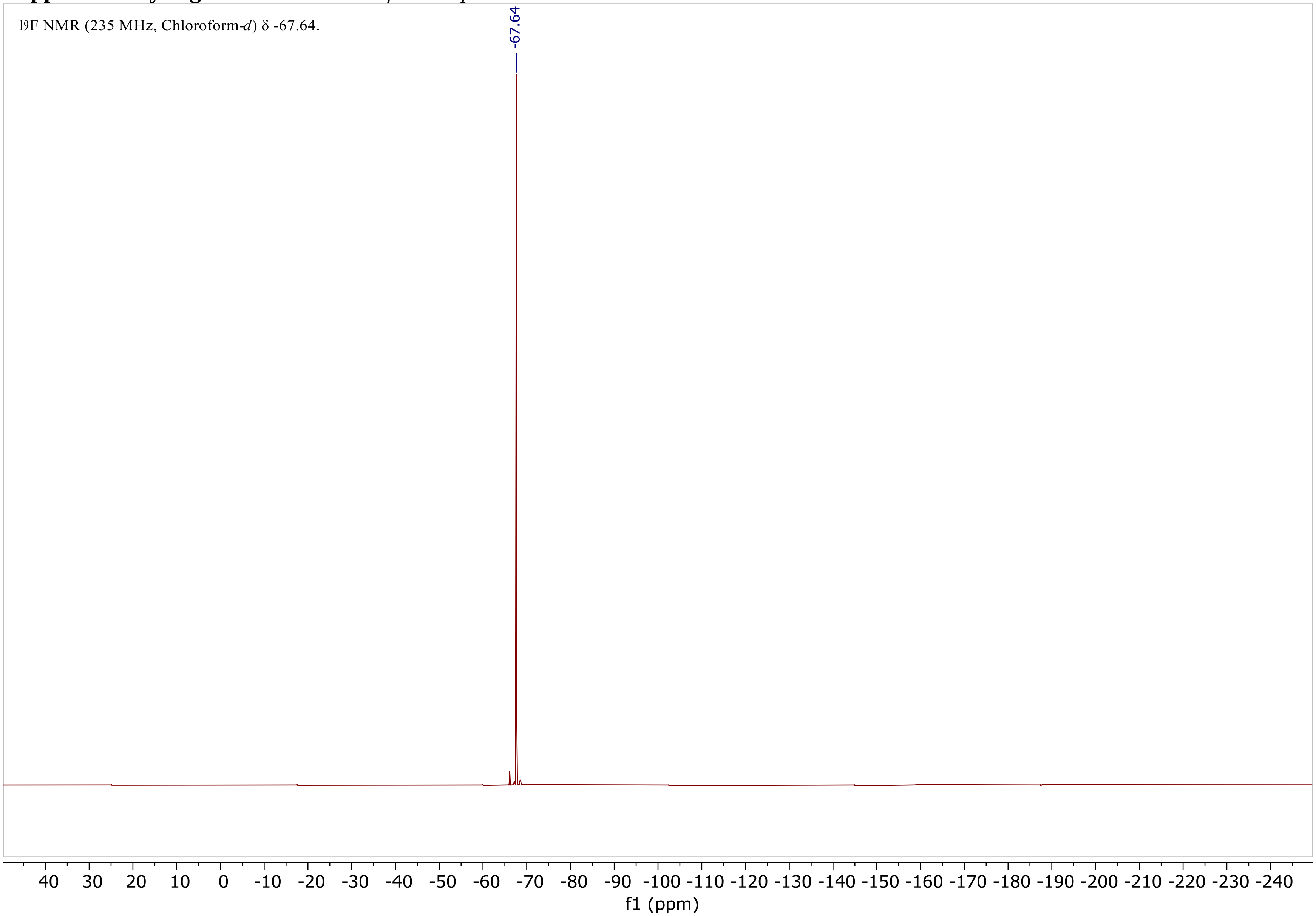

Supplementary Figure 469: *^{13}C NMR for compound* ***98***

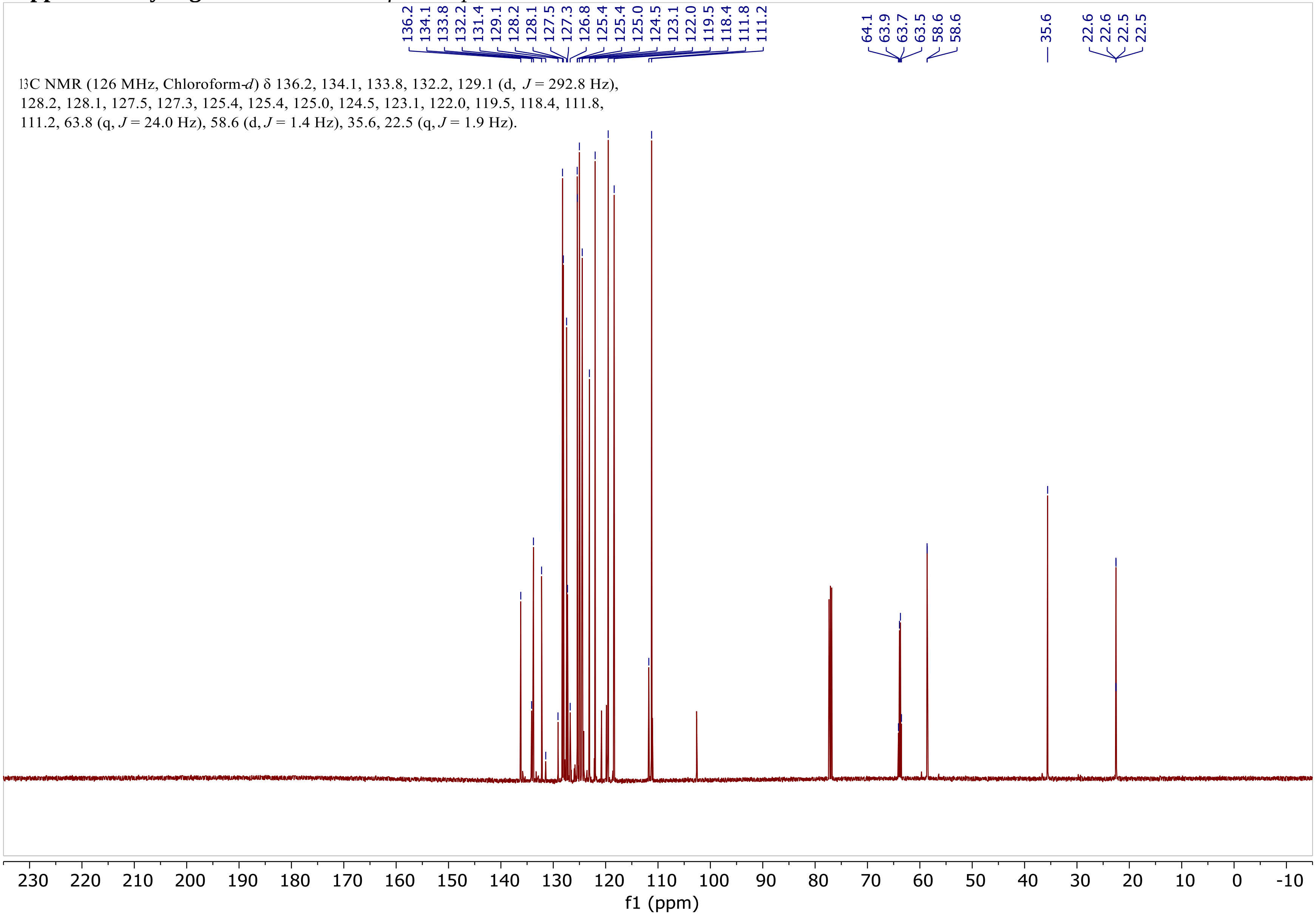

Supplementary Figure 470: *HSQC NMR for compound* ***98***

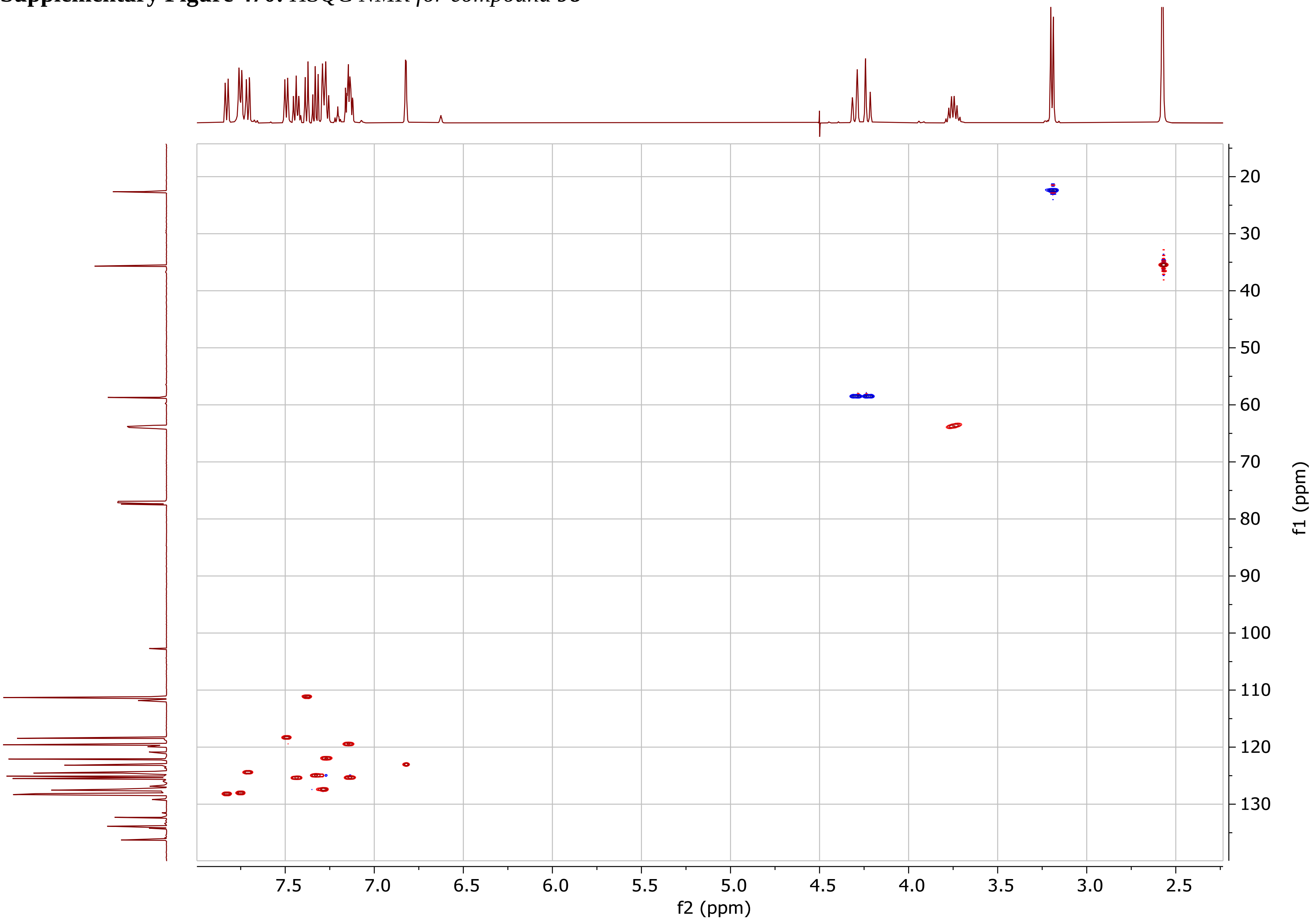

Supplementary Figure 471: *HMBC NMR for compound **98***

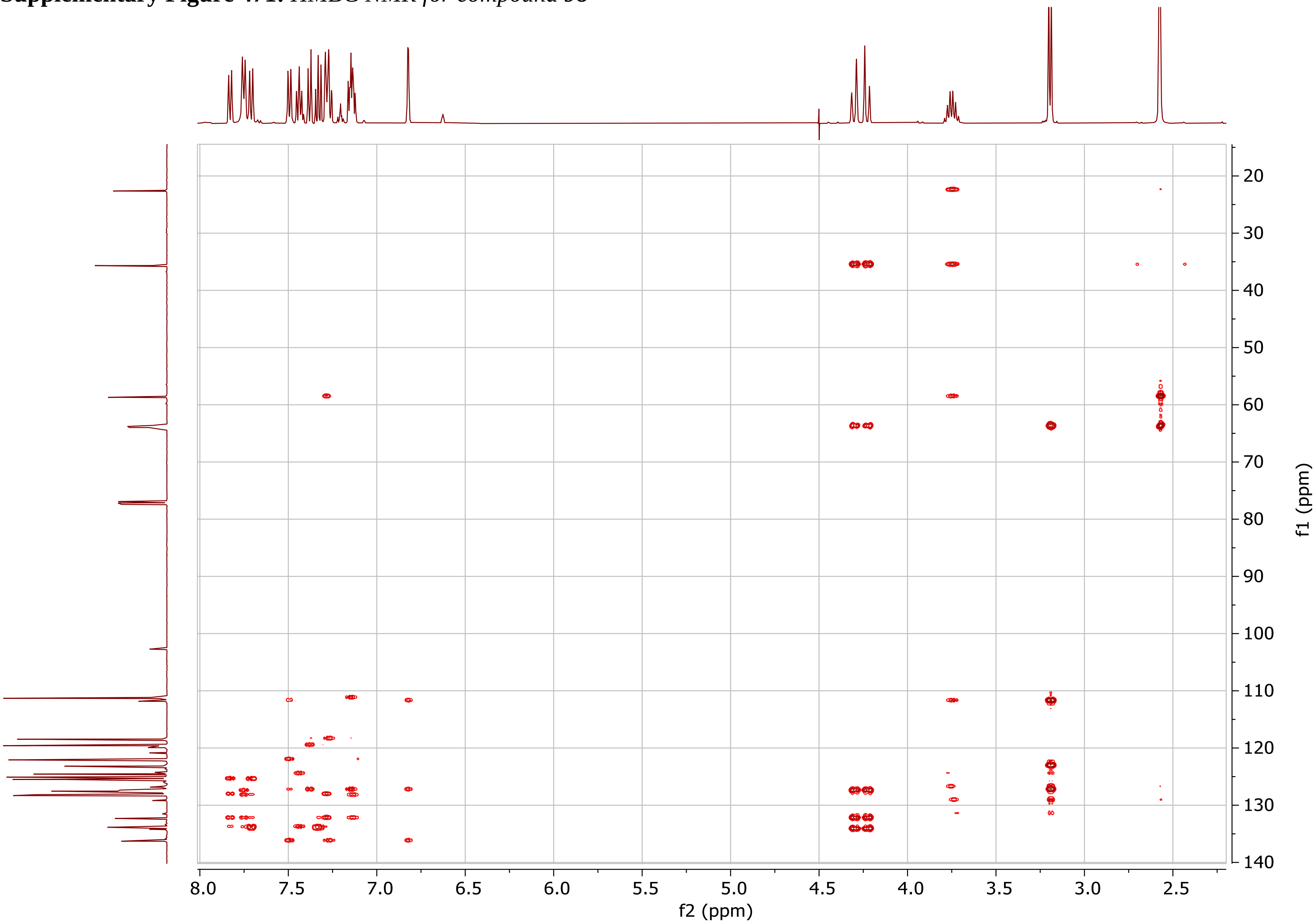

Supplementary Figure 472: *IR for compound* ***98***

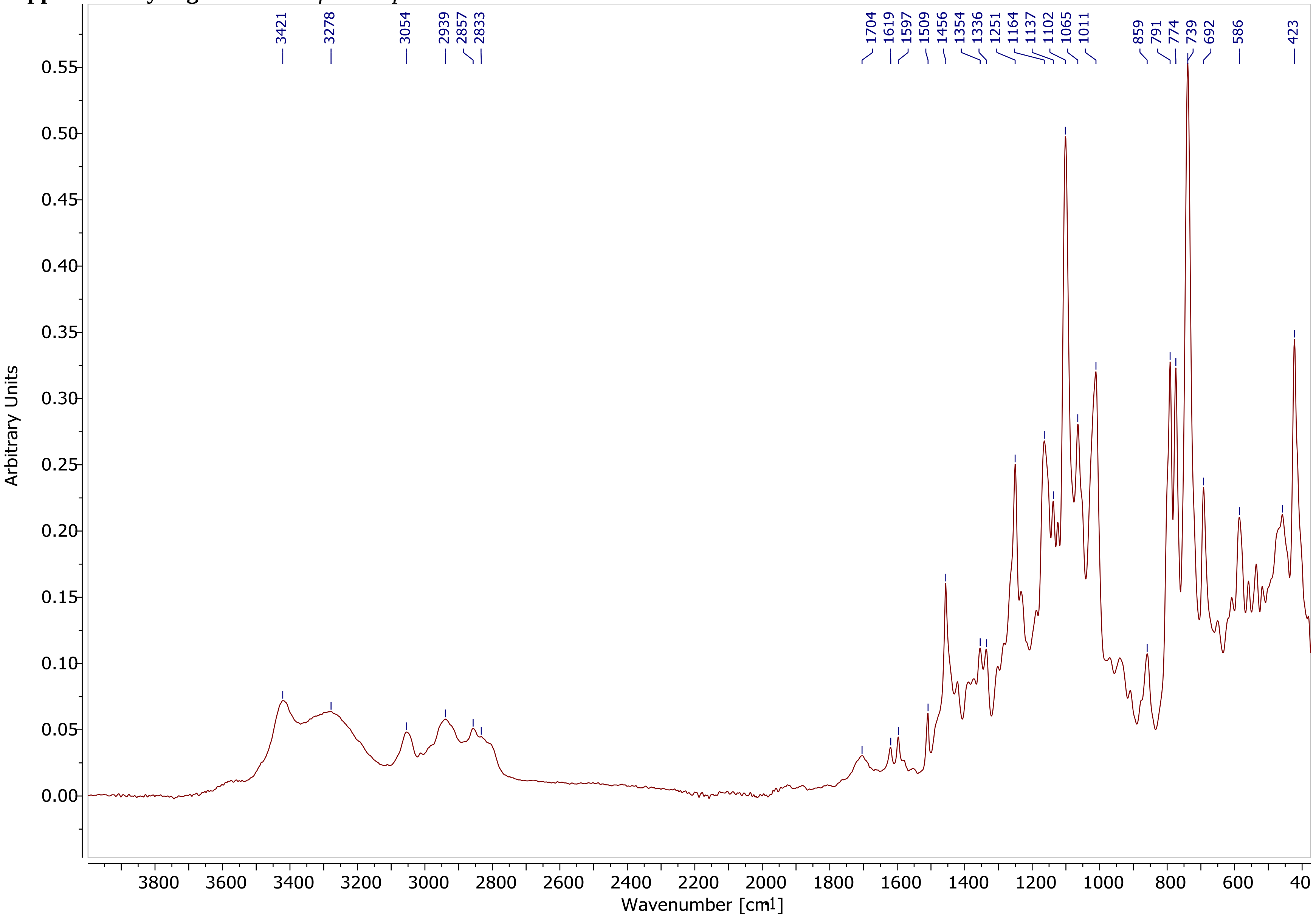

Supplementary References

1. Mészáros, Á., Székely, A., Stirling, A. & Novák, Z. Design of Trifluoroalkenyl Iodonium Salts for a Hypervalency-Aided Alkenylation-Cyclization Strategy: Metal-Free Construction of Aziridine Rings. *Angew. Chem. Int. Ed.* **57**, 6643–6647 (2018).
2. Kennemur, J. L., Kortman, G. D. & Hull, K. L. Rhodium-Catalyzed Regiodivergent Hydrothiolation of Allyl Amines and Imines. *J. Am. Chem. Soc.* **138**, 11914–11919 (2016).
3. Page, P. C. B. et al. Formal Total Synthesis of (mathplus)-C9-Deoxyomuralide from l-Leucine Using a Double Sacrificial Chirality Transfer Approach. *J. Org. Chem.* **82**, 12209–12223 (2017).
4. Fernando Albericio and Lois M. Bryman and Javier Garcia and Enrique L. Michelotti and Ernesto Nicolás and Colin M. Tice. Synthesis of a Sulfahydantoin Library. *J. Comb. Chem.* **3**, 290–300 (2001).
5. Barniol-Xicota, M., Turcu, A. L., Codony, S., Escolano, C. & Vázquez, S. Direct reductive alkylation of amine hydrochlorides with aldehyde bisulfite adducts. *Tetrahedron Lett.* **55**, 2548–2550 (2014).
6. Pedroni, J. & Cramer, N. Chiral γ-Lactams by Enantioselective Palladium(0)-Catalyzed Cyclopropane Functionalizations. *Angew. Chem. Int. Ed.* **54**, 11826–11829 (2015).
7. Hermant, F. et al. Reductive Alkylation of Thioamides with Grignard Reagents in the Presence of Ti(OiPr)4: Insight and Extension. *Organometallics* **33**, 5643–5653 (2014).
8. Kanemasa, S., Sakamoto, K. & Tsuge, O. Nonstabilized Azomethine Ylides Generated by Decarboxylative Condensation of upalpha-Amino Acids. Structural Variation, Reactivity, and Stereoselectivity. *Bull. Chem. Soc. Jpn.* **62**, 1960–1968 (1989).
9. Robichaud, A. & Ajjou, A. N. First example of direct reductive amination of aldehydes with primary and secondary amines catalyzed by water-soluble transition metal catalysts. *Tetrahedron Lett.* **47**, 3633–3636 (2006).
10. Reddy, P. S., Kanjilal, S., Sunitha, S. & Prasad, R. B. N. Reductive amination of carbonyl compounds using NaBH4 in a Brønsted acidic ionic liquid. *Tetrahedron Lett.* **48**, 8807–8810 (2007).
11. Lee, O.-Y., Law, K.-L. & Yang, D. Secondary Amine Formation from Reductive Amination of Carbonyl Compounds Promoted by Lewis Acid Using the $InCl_3/Et_3SiH$ System. *Org. Lett.* **11**, 3302–3305 (2009).

12. Yang, J.-M. et al. In$(OTf)_3$ catalyzed N-benzylation of amines utilizing benzyl alcohols in water. *Tetrahedron* **69**, 7988–7994 (2013).
13. Panteleev, J., Huang, R. Y., Lui, E. K. J. & Lautens, M. Addition of Arylboronic Acids to Arylpropargyl Alcohols en Route to Indenes and Quinolines. *Org. Lett.* **13**, 5314–5317 (2011).
14. Tomita, R., Koike, T. & Akita, M. Photoredox-catalyzed oxytrifluoromethylation of allenes: stereoselective synthesis of 2-trifluoromethylated allyl acetates. *Chem. Commun.* **53**, 4681–4684 (2017).
15. Blackburn, L. & Taylor, R. J. K. In Situ Oxidation-Imine Formation-Reduction Routes from Alcohols to Amines. *Org. Lett.* **3**, 1637–1639 (2001).
16. Yuan, B., Zhang, F., Li, Z., Yang, S. & Yan, R. $AgNO_2$ as the NO Source for the Synthesis of Substituted Pyrazole *N*-Oxides from *N*-Propargylamines. *Org. Lett.* **18**, 5928–5931 (2016).
17. Pereshivko, O. P., Peshkov, V. A. & der Eycken, E. V. V. Unprecedented Cu(I)-Catalyzed Microwave-Assisted Three-Component Coupling of a Ketone, an Alkyne, and a Primary Amine. *Org. Lett.* **12**, 2638–2641 (2010).
18. Chang, Z., Jing, X., He, C., Liu, X. & Duan, C. Silver Clusters as Robust Nodes and π-Activation Sites for the Construction of Heterogeneous Catalysts for the Cycloaddition of Propargylamines. *ACS Catalysis* **8**, 1384–1391 (2018).
